# EMBER multidimensional spectral microscopy enables quantitative determination of disease- and cell-specific amyloid strains

**DOI:** 10.1073/pnas.2300769120

**Published:** 2023-03-16

**Authors:** Hyunjun Yang, Peng Yuan, Yibing Wu, Marie Shi, Christoffer D. Caro, Atsushi Tengeiji, Shigeo Yamanoi, Masahiro Inoue, William F. DeGrado, Carlo Condello

**Affiliations:** ^a^Institute for Neurodegenerative Diseases, University of California, San Francisco, CA 94143; ^b^Department of Pharmaceutical Chemistry, Cardiovascular Research Institute, University of California, San Francisco, CA 94158; ^c^Daiichi Sankyo Co. Limited, Tokyo 103-8426, Japan; ^d^Department of Neurology, University of California, San Francisco, CA 94143

**Keywords:** neurodegenerative diseases, proteins fold, conformational strain, fluorescent excitation/emission, machine learning

## Abstract

In neurodegenerative diseases, proteins fold into amyloid structures with distinct conformations (strains) that are characteristic of different diseases. However, there is a need to rapidly identify amyloid conformations in situ. Here, we use machine learning on the full information available in fluorescent excitation/emission spectra of amyloid-binding dyes to identify six distinct different conformational strains in vitro, as well as amyloid-β (Aβ) deposits in different transgenic mouse models. Our EMBER (excitation multiplexed bright emission recording) imaging method rapidly identifies conformational differences in Aβ and tau deposits from Down syndrome, sporadic and familial Alzheimer’s disease human brain slices. EMBER has in situ identified distinct conformational strains of tau inclusions in astrocytes, oligodendrocytes, and neurons from Pick’s disease. In future studies, EMBER should enable high-throughput measurements of the fidelity of strain transmission in cellular and animal neurodegenerative diseases models, time course of amyloid strain propagation, and identification of pathogenic versus benign strains.

## Significance

In neurodegenerative diseases, proteins fold into amyloid structures with distinct conformations (strains). There is a need to rapidly identify these amyloid conformations in situ. Here, we use machine learning on the full information available in fluorescent excitation/emission spectra of amyloid-binding dyes to identify six distinct different conformational strains in vitro, as well as amyloid-β deposits in different transgenic mouse models. Our imaging method rapidly identifies conformational differences in Aβ and tau deposits from Down syndrome, sporadic and familial Alzheimer’s disease human brain slices. We also identified distinct conformational strains of tau inclusions in astrocytes, oligodendrocytes, and neurons from Pick’s disease. These findings will facilitate the identification of pathogenic protein aggregates to guide research and treatment of protein misfolding diseases.

Amyloid fibrils are insoluble protein aggregates with a diverse range of biophysical properties, biological functions, and association with human diseases. Their stability and resistance to degradation implicates them in: A) adhesion and biofilm formation in bacteria (1), B) spore development in fungi (2), C) rubber biosynthesis in plants (3), D) chemical catalysis (4), E) materials (5), and F) systemic organ amyloidosis and neurogenerative diseases in humans (6). Some of the most well-characterized amyloids are composed of amyloid-β (Aβ), tau, or α-synuclein (α-Syn) proteins and associate with cell death and brain dysfunction in Alzheimer’s (AD) and Parkinson’s disease, the most prevalent neurodegenerative diseases. These amyloidogenic proteins coalesce to form cross β-sheet fibrils, which are observed as deposits in the brain (7, 8). Each amyloidogenic protein is capable of adopting a number of different three-dimensional amyloid structures, each with distinct molecular repeating structures (9–11). Combined with biochemical and pathological processes such as posttranslational modifications (PTMs) or protease activity, these differences are known as conformational strains (12–16). Like viral strains, different amyloid strains can propagate over multiple passages in animals or cell culture as in the classical prion mechanism (17, 18). Also, different strains of disease-causing proteins such as Aβ and tau lead to different pathologies and localization in the brain. More generally, the biological, material, and chemical properties of amyloids depend critically on their conformations. Thus, there is a great need for a rapid method to differentiate distinct conformational strains of amyloids in brain tissues (derived from rodent models and human donors), cultured cells, and cell-free in vitro systems. The method developed here, which discriminates between amyloids with differing sequences and conformations, will benefit research on amyloids in any tissue type or biological system.

Structural methods to differentiate conformational strains are very labor intensive, generally involving biochemical or biophysical analysis [e.g., solid NMR (19–21), protease susceptibility (22), isotope-edited infrared spectroscopy (23), cryo-electron microscopy (24), etc.]. Moreover, these methods lose spatial biological context due to the stringent purification steps to extract amyloids. There are histological fluorescent dyes (25–28) and clinical PET imaging probes (29–31) retaining spatial information. Amyloid-staining dyes with spectral features that can be used as fingerprints to differentiate distinct conformational strains (32, 33), and cryo-EM structures (34–37), have been highly successful in identifying the conformations of a number of disease-associated amyloids. Thus, while strain-sensing dyes do not provide direct structural information, these dyes are sensitive to conformational strain behavior and enable rapid in situ assessment. For example, oligothiophene dyes discriminate PrP strains (38), Aβ strains in AD etiological subtypes, and α-Syn strains in Parkinson’s disease and multiple system atrophy (39, 40). However, they are difficult to synthesize, and their strain-sensing ability has not been quantified to find whether a single molecule can be used to distinguish a wide range of amyloids. To address some of these limitations, we previously developed a method, which utilizes multiple commercial dyes that individually have limited resolving power, but in aggregate were able to identify conformational strains when combined with principal component analysis (PCA) (41, 42). However, the collection of spectra from multiple dyes proved impractical, due to the need to either destain tissues between dye applications or to examine adjoining tissue slices, which limited spatial resolution.

Thus, we sought a technology that relies on only one single dye to sense a wide range of in vitro and biological amyloids. We show that the excitation/emission spectra from a single stain can provide a wealth of discriminating power when analyzed with advanced machine learning methods. The combination of excitation and emission spectra has only rarely been used to identify conformational strains, and there have been no attempts to automate the collection and analysis of data. Our EMBER (excitation multiplexed bright emission recordings) workflow enabled high-throughput measurements of in vitro generated amyloids, allowing identification of amyloid strains within a set of six different amyloid types. It also enabled the analysis of brain slices from diseased tissues, showing large in situ differences in the conformational strains of tau amyloids between multiple AD subtypes and cell-type specific and spatially resolved tau strains in Pick’s disease (PiD). This method should facilitate the measurement of the fidelity of transmission of conformational strains in cellular and neurodegenerative disease animal models used in fundamental research and drug discovery. Moreover, this technology will allow measurement of time courses of aggregation and fibril formation in aqueous solution. Thus, EMBER has the potential to significantly increase the resolution and information content of any application of fluorescence imaging or microscopy in normal and pathogenic amyloids.

## Results

**Fig. 1.**
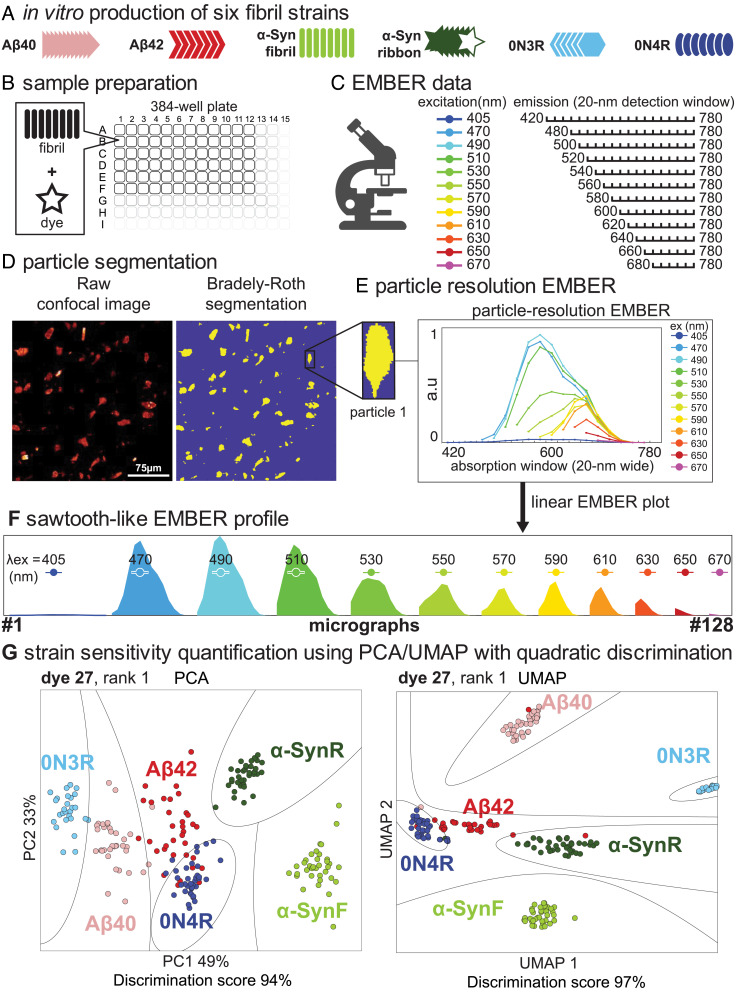
EMBER (excitation multiplexed bright emission recordings) workflow. (*A*) Six distinct conformational fibrils are prepared. (*B*) Each of the six in vitro prepared fibrils is mixed with each 145 dyes in a 384-well plate. (*C*) EMBERs of each fibril and dye mixture are collected. (*D*) Bradley–Roth segmentation is performed to provide a particle-resolution EMBER plot. (*E* and *F*) Overlay EMBER plot or linear sawtooth-like EMBER plot. (*G*) Individual EMBER plots are concatenated for PCA and UMAP analysis followed by quadratic discrimination to quantify conformational strain sensitivity of dye. Boundaries pertaining fit discriminants are presented in black lines.

EMBER uses fluorescence microscopy to evaluate the excitation/emission (XM) spectra of amyloid-dye complexes, followed by PCA (43) (principal component analysis), UMAP (44) (uniform manifold approximation and projection) analysis, or Resnet-based neural network (NN) (45) to identify and quantify spectral differences that are useful for differentiating amyloid strains (Fig. 1). The method can be used on either tissue slices, cultured cells, or amyloid fibrils prepared in vitro. We began with in vitro fibrils, as they are relatively homogeneous, reproducible, and devoid of complicating cellular factors. In particular, we sought to discover dyes that can cleanly identify 6 different amyloids, Aβ40, Aβ42, α-Syn fibril, α-Syn ribbon, 0N3R tau, and 0N4R tau. The fibrils were obtained by shaking the appropriate monomer from 3 to 7 d to assure complete fibril formation. The process begins by mixing a dye with a suspension of a given amyloid strain in a microtiter well, and the plate is centrifuged to settle the amyloids to the bottom of the well. The concentration of fibrils is adjusted to provide a sparse collection of individual clumps of amyloid fibrils, which are imaged by a fluorescent microscope capable of measuring excitation and emission spectra. The spectra can be viewed as conventional overlay plots of emission intensity versus wavelength, each spectrum representing a different excitation wavelength. Alternatively, the individual emission spectra can be laid next to one another to create a sawtooth-like profile (Fig. 1*F*), designated as the XM profile, which describes the full spectral details in a manner that allows easy visual comparison of dyes and subsequent analyses. XM profiles are measured for each particle in the well, and then this process is repeated for the six fibril types for a given dye. This results in approximately 300 XM profiles for a given fibril per micrograph, providing very rich structure-sensitive set of data for probing amyloid strain behavior.

**Fig. 2.**
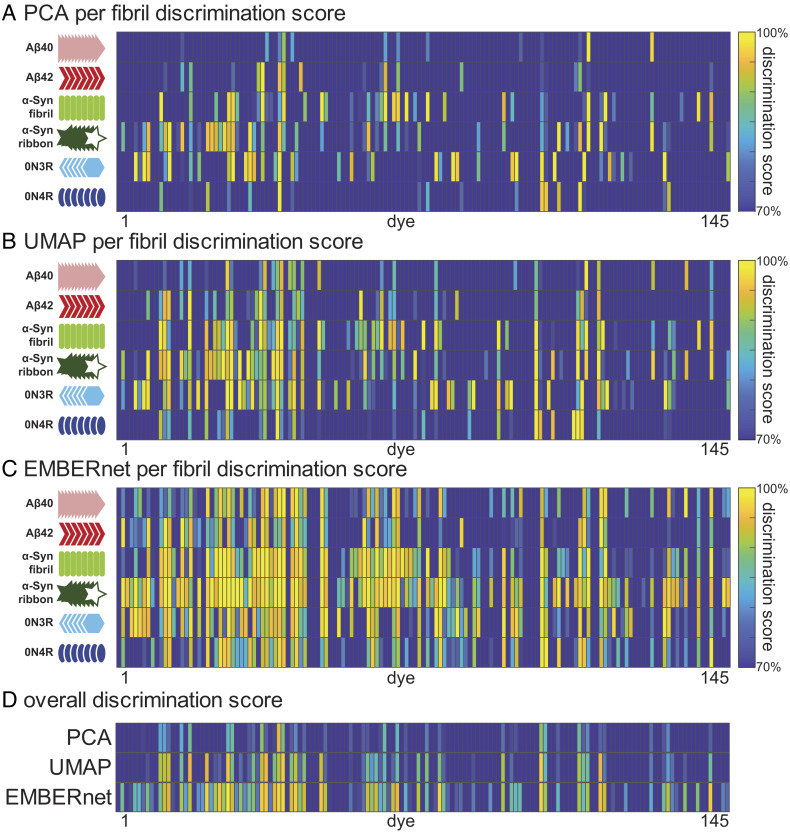
Per dye discrimination scores of each fibril type from (*A*) PCA, (*B*) UMAP, (*C*) EMBERnet, and (*D*) across all fibril types. The heatmap plots are showing all discrimination scores >70% in the gradient colormap and all discrimination scores <70% in dark blue.

We next use PCA, UMAP, and NN on the collection of XM profiles to determine how efficient the dye is at discriminating the six different fibril types in an unsupervised manner (Fig. 2). The intensities of the XM profile are listed as a column vector, and a X*Y matrix is created in which X is the total number of particles across all the six fibril types and Y is the number of intensities measured in a single XM profile. PCA and UMAP are then used to determine the variability of the XM profiles for each particle. The clustering of the points for a given type of fibril is useful in determining the degree of homogeneity of the sample. Points for additional fibril types that fall outside of a given fibril cluster reflect differences in the environment of the bound dye. If the spectral features for a given fibril (e.g., Aβ42) are distinct from those of the other fibril types, the Aβ42 points will form an isolated cluster. We determine both PCA and UMAP (discrimination) plots. PCA is not as discriminating as UMAP but the Eigenvectors of PCA readily provide important physically meaningful information about which spectral features contribute most to discrimination. Thus, once a highly discriminating dye has been identified by the UMAP process, the Eigenvectors can inform the choice of excitation and emission wavelengths for more conventional imaging. Fig. 1 illustrates PCA and UMAP profiles for one particularly discriminating dye from our collection and *SI Appendix*, Fig. S1 demonstrates strain sensitivity and reproducibility of EMBER data collection over a 3-d period by two operators.

This process was then repeated for 145 different dyes, to find which individual dyes are most able to discriminate this collection of fibrils. The dyes comprise 56 loosely associated homologs of PBBs, 24 laser dyes, 14 curcumin homologs, 4 fsb homologs, 2 oligothiophene derivatives, and 47 other published or patented amyloid-binding dyes (*SI Appendix*, Table S1). To aid the analysis, a MATLAB script was written to automate the segmentation and spectral measurement of individual particles. Using quadratic discrimination to determine the degree of overlap between clusters, we identified 18 different dyes with discriminating power beyond 90%—a metric that ranges from 0% for no discrimination to 100% for full discrimination (Fig. 2 and *SI Appendix*, Fig. S2 and Table S1).

We also examined supervised methods of analysis. A convolutional neural net (EMBERnet) was trained on 80% of the XM profiles, which had been preassigned to each fibril type, retaining 10% each for validation and test sets (Fig. 2 and *SI Appendix*, Figs. S3–S5). As we expected, EMBERnet performed favorably relative to PCA and UMAP. For the most discriminating dyes, we observed very high discrimination scores using both EMBERnet and UMAP. However, EMBERnet performed better over a much wider range of dyes, indicating that it is better able to discover differences in even closely related spectra. Thus, EMBERnet is the method of choice when there is a large body of spectral data that has been assigned to each fibril type. On the contrary, as an unsupervised method, UMAP shows its versatility in its ability to identify distinct clusters of conformers, even when the conformers have not been preassigned. This ability provides a powerful tool to discover systematic, unbiased differences between: 1) different amyloid preparations; 2) different cell types; or 3) distinct spatially resolved inclusions within a single tissue slice.

### Discrimination of In Vitro Fibrils with EMBER.

**Fig. 3.**
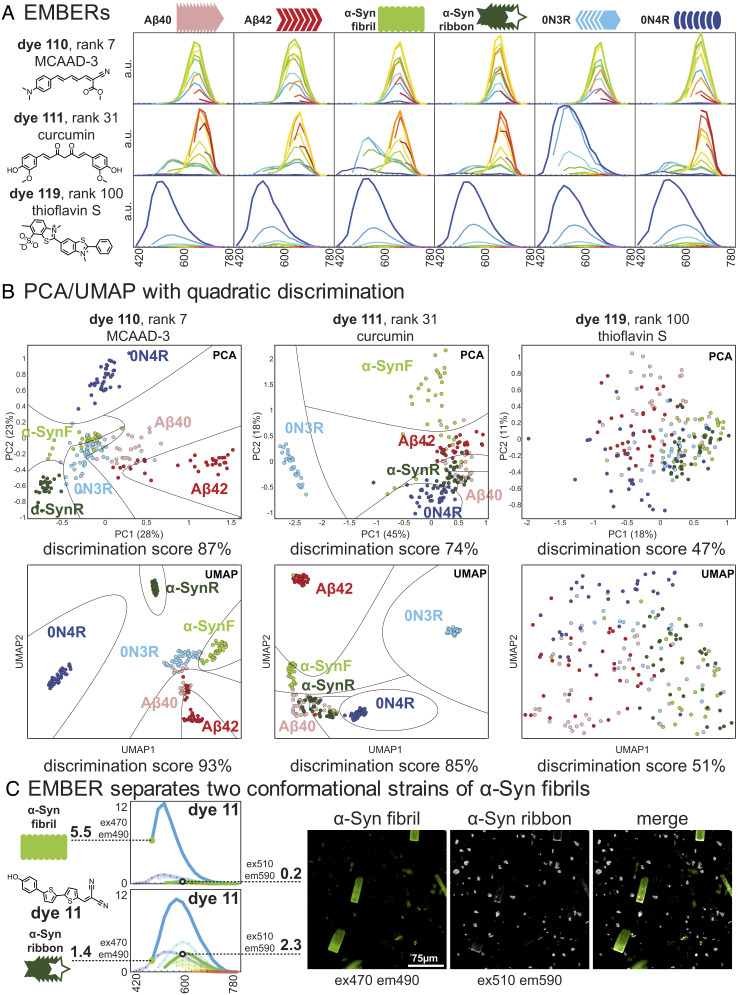
EMBERs of three dyes. (*A*) EMBERs of top, middle, low-tier dyes, and their chemical structures. (*B*) PCA and UMAP analysis of EMBERs. (*C*) Discrimination of α-SynF and α-SynR fibril with excitation and emission shift. α-SynF and α-SynR were mixed in a single well, and dye 11 was added to achieve two different stained fibrils in a single micrograph.

Fig. 3 illustrates typical spectra and discrimination plots for low, intermediate, and highly resolving dyes. MCAAD-3 (dye 110) is one in the most discriminating group (98%); although its emission spectra look similar for the different fibrils, their intensity profiles do not vary uniformly with respect to excitation wavelength. Thus, a full EMBER analysis is able to identify dyes that do not have large spectral shifts, but nevertheless have very well-defined differences that are highly reproducible between fibrils of a given type–but vary reproducibly between different fibril types. Curcumin-stained XM exhibits quite different behavior, showing large differences in XM intensities between fibril types. The emission spectrum has two peaks whose relative intensities vary markedly with respect to the excitation wavelength in a fibril type-specific manner. Additionally, λ_ex max_ for the two peaks shifts with the fibril type, providing an additional discriminating feature. Such a dye might be ideal when a single excitation and/or emission is monitored, and it is not feasible to record full spectra. In contrast, low-discrimination dyes can be quite useful for broad-spectrum staining. Indeed, the commonly used dye thioflavin S was found to have a low discrimination score (51%, rank = 100th). Interestingly, in some cases, dyes perform reasonably well when excited at single wavelength, while the performance of other dyes only manifests in upon examination of both excitation/emission spectrum, emphasizing the utility of the EMBER method (*SI Appendix*, Fig. S6).

Thus, we used single dyes in our collection that could readily discriminate fibril types in a suspension, without the need for full spectral analysis (Fig. 3*C*). For example, we found that dye 11 stained α-Syn fibrils—α-SynF emitted brightly with an excitation/emission pair of 470/490 nm XM, while α-SynR was dim at these wavelengths, but quite bright with 530/590 nm XM. Satisfyingly, the two conformational strains of a single protein were easily identified using this method. Other pairs of XM selectivity can be readily extracted from *SI Appendix*, Table S1 which outlines the XM_max_ pairs for given fibril type per dye.

### Quantification of Conformational Strain Sensitivity in Ex Vivo Mouse Brain Slices.

**Fig. 4.**
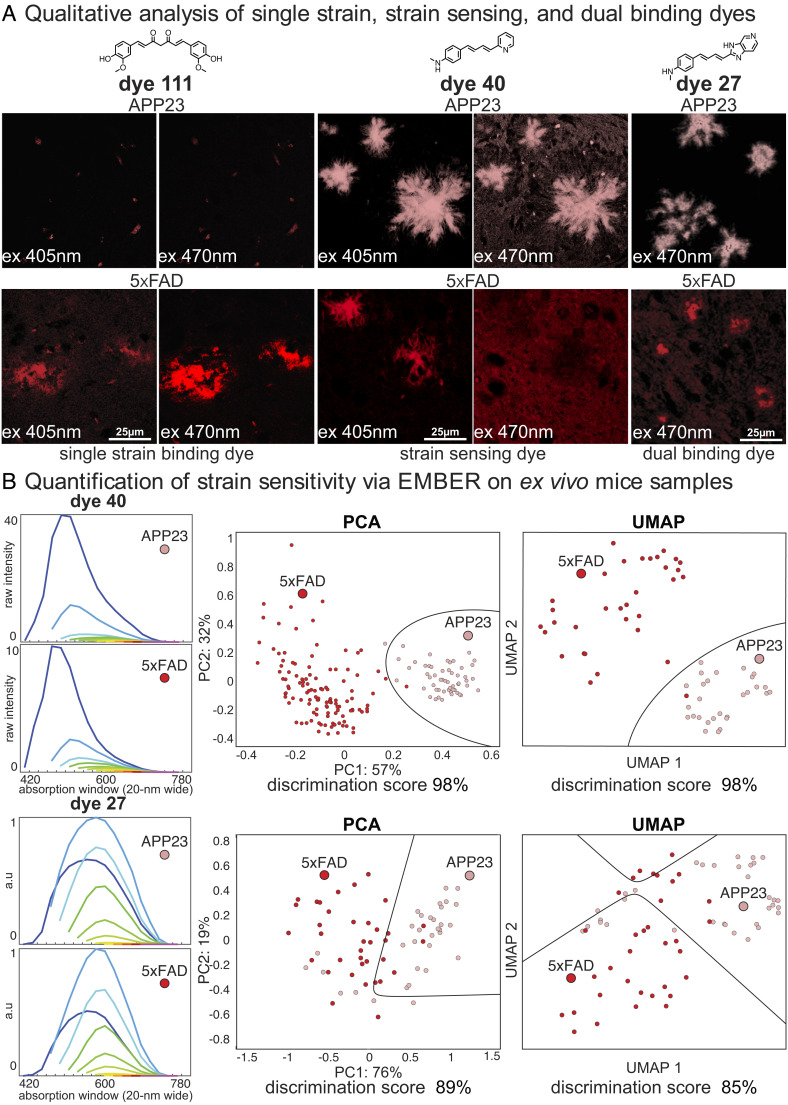
(*A*) Qualitative analysis of strain sensitivity against Aβ plaques formed in APP23 and 5xFAD tg AD mouse models. (*B*) Dye strain sensitivity quantified via EMBER on ex vivo mouse samples.

We next asked whether we could validate strain-sensing dyes discovered using our in vitro platform for recombinant fibrils in ex vivo applications. To evaluate whether novel strain-sensing dyes could readily identify differences in ex vivo brain slices, we selected two different, well-characterized transgenic AD mouse models of human Aβ deposition: 1) Tg(APP23) mice which produce plaques rich in Aβ40 isotype and deposit slowly, and 2) Tg (5xFAD) mice which produce plaques rich in Aβ42 isotype and deposit rapidly. Using formalin-fixed mouse brain sections, we evaluated approximately 50 top-scoring dyes (identified above) to assess strain sensitivity and tissue compatibility; 20 (of the 50) exhibited little nonspecific background tissue staining and were selected for further examination. Qualitatively, the dyes could be separated into three classes: The first class stained a single strain and were bright over a range of excitation and emission spectrum. Dye 111 is typical of this class, as it brightly stains 5xFAD plaques with λ_ex_ = 405 nm and 470 nm but failed to appreciably fluoresce when it is used to stain APP23 plaques (Fig. 4*A*). A second class of dyes stained plaques in both brain types, but the excitation wavelength required for detection was different between the two plaques. For example, dye 40 excites both plaque types with λ_ex_ = 405 nm but shows strong fluorescence for only APP23 when excited at 470 nm. Finally, some dyes, such as dye 27, stained plaques in a strain-independent manner over a range of wavelengths. Thus, our collection of dyes should be helpful, over a wide range of applications.

Further analysis of these data using the full EMBER pipeline showed additional resolving power. The plaques were identified, and spectra were measured using the same workflow as for the in vitro fibrils. In Fig. 4*B*, each particle on the PCA and UMAP plot represents one XM profile from a single plaque. The qualitative discrimination seen in Fig. 4*A* is consistent with the quantitative ability to discriminate (*SI Appendix*, Fig. S7). Dye 40, which showed a higher fluorescence intensity for APP23 plaques over 5xFAD plaques at 470 nm excitation, had a 98% strain-sensing score (based on UMAP) for the two brain types. Importantly, EMBER analysis was also able to provide some discrimination, even when the spectra appeared similar by visual examination as seen with dye 27 (Fig. 4*B*). This shows the power of EMBER and a library of dyes to solve the joint optimization problem of strain sensing and tissue compatibility.

### Discrimination of Aβ Plaques and Neurofibrillary tau Tangles in sAD Brain Samples.

**Fig. 5.**
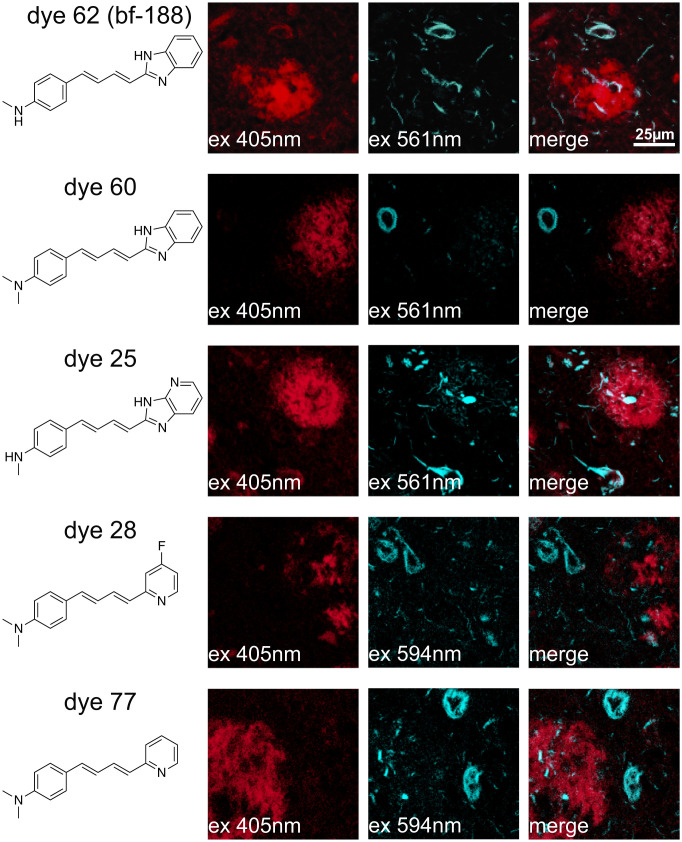
Structures of dyes that stain and strain sense Aβ plaque and tau tangle on sAD brain samples. Aβ plaques are excited at 405 nm excitation and tau tangles are excited at 561 nm excitation or above.

Using the top strain-sensing dyes that were compatible with mouse tissue, we next identified dyes that can stain and differentiate both Aβ plaques and the tau neurofibrillary tangles (NFTs) in AD brain samples from patient donors (Fig. 5). Since these two deposit types are both present in a single AD brain slice, strain discrimination analysis under the same micrograph was possible. The plaques and tangles were initially assigned by visual analysis of their morphologies and were confirmed by antibody staining (*SI Appendix*, Fig. S8). We focused on filamentous plaques as defined previously (46), which were highly abundant relative to compact dense-core plaques, neuritic plaques, and cerebral amyloid angiopathy (CAA), and found consistently in all AD and DS brain samples we examined. Contrary to diffuse plaques, filamentous plaques can be labeled by amyloid-binding dyes. Consistent with previous findings with bf-188^32^, excitation at 405 nm resulted in high fluorescence intensity for Aβ and very low intensity at wavelengths greater than 561 nm. Just the opposite was observed for tau tangles. Thus, these dyes are ideally suited for identifying tau versus Aβ plaques with a single reagent in a single field of view, obviating the need for additional antibody staining.

At the qualitative level, we looked for other dyes that can bind and discriminate between both deposit types by shifts in excitation. Of the top-scoring dyes, we identified five dyes that spectrally separate the Aβ plaques and tau tangles. Dye 60, for example, favored Aβ plaque emission at 405 nm excitation compared to tau tangle emission at a more red-shifted excitation at 561 nm. Interestingly, all the five dyes share chemical homology to PBBs and share the fluorescence properties of red-shifted XM for tau tangle and blue-shifted XM for Aβ plaque. We postulate that the shallow groove observed in the cryo-EM structures of tau fibril encourages dye interaction, causing the observed exciton coupling (47–49). Dye 60 was then selected for additional full EMBER analyses of Aβ plaques and tau tangles in a variety of different disease types.

### Aβ Strain Discrimination in Alzheimer’s Disease and Down Syndrome (DS).

**Fig. 6.**
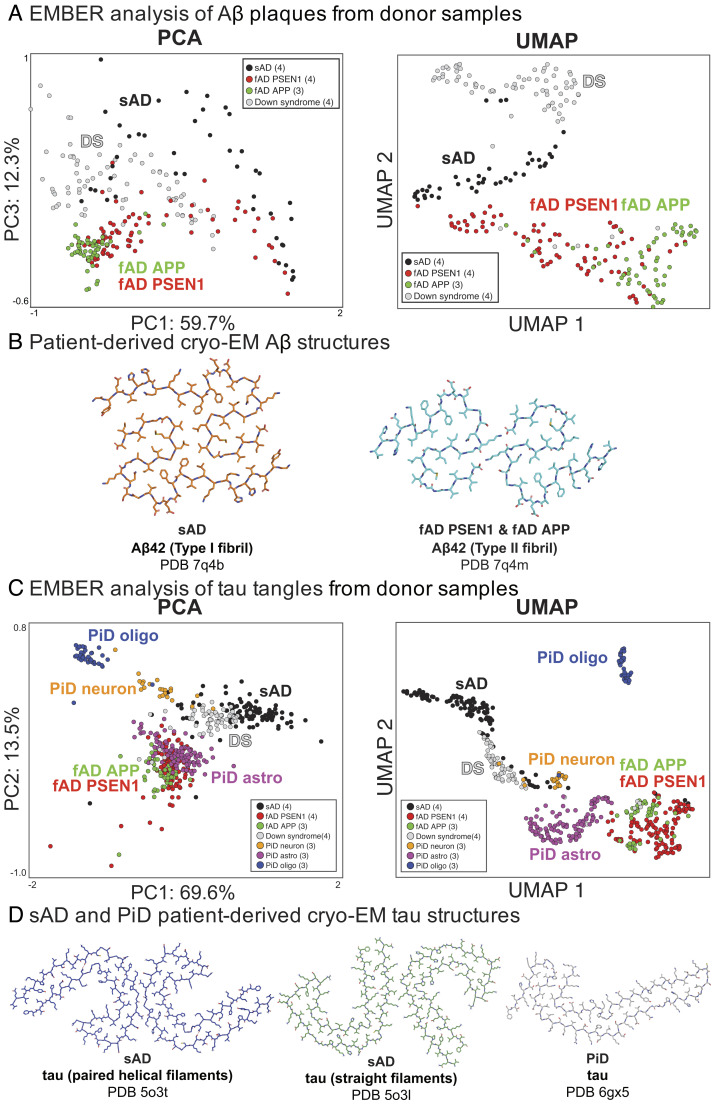
EMBER discriminates the conformational strains of Aβ plaques and tau tangles across neurogenerative diseases. (*A*) PCA and UMAP analysis of EMBERs of dye 60-stained Aβ plaques reveals interdisease type cluster separation. fAD PSEN1 (red) and APP (green) share cluster in both PCA and UMAP. sAD (black) and Down syndrome (gray) share a cluster in PCA but are separated in the UMAP. Each particle represents one segmented Aβ plaque from EMBER micrograph. The number of donor samples in each cohort is within the parenthesis. (*B*) Cryo-EM structures of Aβ42 fibrils (10). (*C*) PCA and UMAP analysis of EMBERs of dye 60-stained tau tangles reveals interdisease type cluster separation. fAD PSEN1 (red) and APP (green) share cluster in both PCA and UMAP. sAD (black), Down syndrome (gray), PiD neuron (orange), PiD astrocyte (purple), and PiD oligo (blue) form their own clusters depicting conformational strain polymorphism of tau tangles. Each particle represents one segmented tau tangle from EMBER micrograph. The number of patient samples in each cohort is within the parenthesis. (*D*) Patient-derived cryo-EM structures of tau fibrils (9, 34).

We found that dye 60 was particularly useful for discriminating conformational strains of Aβ plaques across four neurodegenerative diseases—sAD, fAD PSEN1, fAD APP, and Down syndrome (DS). Multiple brain samples from each of the neurodegenerative diseases (*SI Appendix*, Table S2) were stained with dye 60 and their EMBER data were acquired from Aβ plaques (*SI Appendix*, Fig. S9). Remarkably, plaques from each disease type form tight clusters in the plots (Fig. 6 and *SI Appendix*, Fig. S10). Generally, we observed little interpatient heterogeneity within a cohort even across fAD PSEN1 samples from three different brain banks for fAD PSEN1 (*SI Appendix*, Fig. S11). The use of multiple excitation wavelengths increased the resolution, relative to single-wavelength measurements (*SI Appendix*, Fig. S12).

fAD PSEN1 involves a mutation in the gene encoding one of the proteolytic processing enzymes (γ-secretase) of the Aβ precursor protein (APP), and it results in a higher Aβ42/Aβ40 ratio than wild type (WT). fAD APP is associated with a mutation to regions of APP that are outside of the Aβ peptide sequence, but within the γ-secretase cutting site. It too results in a higher Aβ42/Aβ40 ratio than WT. The structures of these two types of fAD had similar cryo-EM structures, which differed markedly from that of sAD (10). Thus, it is interesting that there is essentially no overlap (99% discrimination) between sAD (which has WT APP) and the two types of fAD. By contrast, there is extensive overlap between fAD PSEN1 and fAD APP in the UMAP plots. Nevertheless, the fAD cohorts do show some separation, which might arise from conformational differences in the bulk of the sample, differences in the Aβ42/40 ratio or other associated molecules that are not apparent in highly purified samples used for cryo-EM (50).

More impressively, the DS plaques were also fully separated from sAD and fAD plaques. Furthermore, we observed patient-specific (intra-DS group) clusters (*SI Appendix*, Fig. S11), which is consistent with our prior work (42). High-resolution structures of Aβ fibrils from DS donors have yet to be identified; we postulate that the strain is similar but distinct to sAD as DS and sAD clusters overlap in the PCA and DS forms its own cluster in the UMAP. The PCA cluster area was larger for sAD and DS, suggesting greater heterogeneity across the brain and amyloid deposits than that of the fAD samples.

### Quantitative Determination of Distinct Tau Strains within and across Neurodegenerative Diseases.

Since dye 60 stains both Aβ plaques and neurofibrillary tau tangles (NFT), EMBER data from the tau tangles were collected and analyzed in the same micrographs (*SI Appendix*, Fig. S13). In addition to the four disease types we analyzed for Aβ plaques (*SI Appendix*, Table S2), we also examined tau deposits from the “tau-only” Pick’s disease (PiD)—cryo-EM structures of PiD tau fibrils are distinct from sAD tau fibrils (34). Moreover, tau deposits are found in three types of cells including neurons, astrocytes, and oligodendrocytes in PiD (51, 52), providing an opportunity to evaluate differences in conformational strains associated with different cell types.

As was the case for Aβ plaques, the tau tangles in DS and sAD were partially overlapping (Fig. 6). On the UMAP plot, five clusters are formed capturing the polymorphic nature of tau tangle strains across neurodegenerative diseases. sAD, DS, and fAD all form their own clusters. Interestingly, the familial mutations in APP and PSEN1 both lead to overproduction of Aβ42 in fAD, and the consequent tau tangles localize to the same cluster.

A clear difference was seen between the fAD and sAD cases. This finding contrasts with a cryo-EM study that suggests similar conformations for tau from fAD and sAD. It is not yet clear whether the difference we observe is a result of subtle differences in structure, as the cryo-EM structure of tau from fAD APP was at low resolution. Alternatively, the difference we see might arise from differences in PTMs or proteolysis. Interestingly, while we observed clear interpatient variability of Aβ from fAD cases, the tau tangles showed no interpatient variability (*SI Appendix*, Fig. S14). Thus, different strains of Aβ plaques are capable of inducing the same strain of tau tangles. There was partial overlap between the UMAP clusters for DS and sAD, which is unsurprising given that aged DS patients often have comorbid AD-like symptoms and the accumulation of tau tangles (53). This finding suggests that while there are large differences in the EMBER-detected conformational strains for Aβ plaques in DS vs. sAD, they ultimately lead to similar conformational strains of the tau tangles. Thus, the major difference in tau tangles appears to be temporal rather than structural (54).

In addition to separating PiD tau deposits from all other neurodegenerative disease tau in our analysis, EMBER was strikingly useful for discriminating between tau deposits localized to neurons, astrocytes, and oligodendrocytes in PiD brain samples. We confirmed the localization of tau deposits to each cell type using histochemical markers (*SI Appendix*, Fig. S15) and their EMBER reproducibility (*SI Appendix*, Fig. S16). These findings emphasize the power of EMBER to discriminate distinct strains and their spatial distribution in situ in multiple cell types (*SI Appendix*, Fig. S17).

## Discussion

EMBER provides an attractive, well-automated, and objective approach for selection of strain-sensitive fluorescent dyes, and to maximize the strain information obtainable once a dye has been selected. The method is useful for examining very small samples with retention of spatial information. Although we have focused here on Aβ, tau, and α-Syn, the approach should be generalizable to address questions related to many other beneficial and pathologic biological amyloids (55), as well as synthetic materials (56). While we screened 145 dyes, the method can be easily extended to examine a larger library of commercial and custom dyes. It also provides a high degree of discrimination for selection of potential imaging agents that may differentiate only a single conformational form of the same protein. EMBER provides a very rapid method to probe the homogeneity of a sample, and when conformational information is available for a fibril type, it allows one to define a spectral signature for the structure. The method should also be highly useful for identifying dyes that are sensitive to conformation for in vitro or cell-free solution studies of intermediates and products for continuous monitoring of protein aggregation and amyloid formation on a fluorescent plate reader.

EMBER should also provide a highly discriminating probe for investigation of conformational fidelity during amplification or passage through cells and animal models. This microscopic approach provides a rapid conformational assessment, which can be correlated with cellular location of inclusions or their anatomical location. This will be particularly important for mechanistic studies and drug development, where it is important to confirm strain fidelity between multiple passages and time points.

While a fluorescent signature does not give direct structural information, it reports on the fine details of the binding site where the dye is located. As such, it not only reflects differences in conformation, but also might be influenced by PTMs, proteolysis, small molecule cofactors, and protein isoforms generated by differential gene splicing. EMBER is likely sensitive to each of these variables, and hence not entirely a measure of amyloid fibril structure, but also of these other features, which collectively define a conformational strain. For example, cryo-EM structures for sAD and fAD tau appear similar (57), but different PTMs or truncated tau species not recognized by cryo-EM may contribute to the differences we observed in our EMBER data.

Interestingly, the spatial resolution afforded by EMBER revealed different conformational strains associated with tau deposits in different neural cell types in PiD samples. This finding shows that neurons, astrocytes, and oligodendrocytes have distinct conformational strains in the same brain. At first, our data may seem difficult to reconcile with the single PiD tau structure (34) currently available from brain homogenates. However, this structure is derived from a single PiD donor and may only represent the predominate tau filament from the most abundant tau-laden cell type present in the sample used for fibril purification. Consistent with the observation, Falcon et al. have described a panel of western blots of purified tau extracts stained with different tau antibodies from nine PiD donors (including the one used for cryo-EM) that show varying band patterns and intensity differences (*SI Appendix*, Fig. S6 from ref. 34). Thus, it will be interesting to determine whether the variation we see with cell type reflects differences in the conformation of the ordered amyloid core, or differences in PTM and the proteolytic variability seen previously.

We observed a dramatic increase in resolving power when we consider three spectral features that include excitation/emission spectra and overall brightness. The structural and chemical environment of the binding site affects the fluorescence according to a number of effects, including rigidity, excited state/ground state pKa, dye–fibril interactions, and orbital overlap between dyes held in close proximity along the regularly repeating structure of the amyloid. For example, in some cases, we observe a doublet in the emission spectra, which likely reflects excitonic coupling between stacks of aromatic dyes (58).

Because EMBER relies on machine learning, it should be possible to expand EMBER to consider a number of different additional variables, each providing unique information. For example, dyes associate with differing amyloids at a range of affinities, so concentration should provide an additional discriminating variable. Moreover, the fluorescence lifetime and time-resolved Stokes shifts can be readily determined using instruments equipped with pulsed lasers and time-resolved spectral acquisition. Finally, the degree of immobilization of a dye within its binding site can be determined using steady-state and time-resolved fluorescence polarization.

In conclusion, EMBER is a highly resolving, automated method to discover and maximize the information of strain-sensing dyes with retention of spatial information. EMBER offers a facile quantitative approach that complements existing dye-based imaging methods to study conformational strains in cultured cells, animal models, and primary human tissues. The method should be applicable to a variety of amyloids. In principle, it may also be useful to examine dyes that are responsive to molten globule-like intermediates and oligomers (59). Moreover, EMBER may be useful to rapidly screen hundreds of neurodegenerative disease donor samples and prioritize new targets for cryo-EM structural characterization.

## Materials and Methods

### Plate-Based In Vitro Fibril Staining for High-Throughput EMBER Microscopy.

Each dye was dissolved in 1× PBS buffer and then centrifuged at 13,200 rpm to result either 25 µM or saturated dye solution. A 50-µL dye solution was mixed with 1-µL fibril solution which contained ~0.1 µg fibril in a 384-well plate (Corning™ BioCoat™ 384-Well, Collagen Type I-Treated, Flat-Bottom Microplate). Each well was mixed by pipetting up and down and then the plate was centrifuged at 50 × g to form fibril pellets.

### EMBER Microscopy Data Collection.

The 384-well plate containing the stained fibrils was imaged with Leica SP8 confocal microscope using a 40× water immersion lens (1.1 NA), a white light and 405 nm lasers, and a HyD detector at 512 × 512-pixel resolution at 0.75× zoom. For high-throughput data acquisition, sequential data collection was achieved using the *Live Data* mode, a module in the Leica LAS X software. For each field-of-view experiment, the optical plane was autofocused with the highest sensitivity setting. To reduce the background noise from the bottom of the plate well, LightGate was set to 0.5 to 18 ns. First, a total of 110 images were acquired using the Λ/λ-scan mode with excitations of 470, 490, 510, 530, 550, 570, 590, 610, 630, 650, and 670 nm wavelengths. The emission detection range started at 10 nm greater than the given excitation wavelength, and ended at 780 nM, with 20-nm window. For example of 470 nm excitation, the images were collected at 480 to 500, 500 to 520, 520 to 540, 540 to 560, 560 to 580, 580 to 600, 600 to 620, 620 to 640, 640 to 660, 660 to 680, 680 to 700, 700 to 720, 720 to 740, 740 to 760, 760 to 780 nm. Then in the λ-scan mode, 18 additional images were collected at 405 nm excitation with emission detection intervals of 20-nm for 420 nm to 780 nm. For ex vivo brain sample data collection, the zoom was increased to 2.0 and FOVs were manually focused.

### Postprocessing and Particle Segmentation.

We developed a set of custom scripts in MATLAB to process the raw fluorescent images and segment the aggregated protein deposits. In brief, we applied locally adaptive thresholding strategy. First, we generated a projection from the EMBER dataset. The maximum intensity projection was calculated across the λ stack: For the XY coordinate of a pixel, the highest fluorescence intensity within the corresponding coordinate through the λ stack was selected, resulting in a 2D image. The Bradley–Roth image thresholding method was applied on the resulting image: The image was divided into approximately eight smaller neighborhoods, each with an independent thresholding value calculated from the local mean fluorescence intensity; each pixel was then assigned a binary background or foreground value based on its neighborhood threshold value. Foreground noise was reduced by applying an image erosion calculation with a disk-shaped structuring element. Background noise was reduced by applying a flood-fill algorithm to fill holes in objects. Parameters for the segmentation processes were determined with a trial dataset and kept constant for each experiment. All deposits were then overlaid on the raw images for inspection and incorrect segmentations were removed from downstream analysis.

### PCA and UMAP with Quadratic Discriminant Classification.

The signal-processing algorithm for the analysis of particle resolution EMBER spectra was executed in MATLAB with *pca*. Each identified EMBER particle from the particle segmentation was normalized to [0, 1] and then concatenated in an array for PCA. The principal component scores PC1 and PC2 were plotted. UMAP was performed in MATLAB with *run_umap* with default settings. The cluster classification algorithm for the analysis of PCA and UMAP plot was executed in MATLAB with *fitcdisc*. From the PCA or UMAP plot, 40 random particles from each fibril were concatenated in an array and grouped for quadratic discrimination. This process was repeated ten times and the average of 10 accuracy scores was used as the discrimination score.

### Dye Staining in Brain Sections for EMBER Microscopy.

Formalin-fixed paraffin-embedded (FFPE) mouse brains were sectioned (8-μm thickness) and glass mounted. To reduce the autofluorescence signals by greater than 90% intensity (e.g., lipofuscin or hemosiderin), FFPE slides were photobleached up to 48 h using a multispectral LED array in a cold room overnight to reduce the autofluorescence in the brain tissue (60). The sections were deparaffinized, PBS washed, and stained with 25 µM for 30 min. The sections were washed with PBS buffer and coversliped with PermaFluor (Thermo) as the mounting media. For FFPE human brain sections, the same procedures were followed. For EMBER data collection, the same steps were taken without the autofocus function and with zoom of 1.5.

See *SI Appendix* for additional materials and methods.

## Supplementary Material

Appendix 01 (PDF)

Dataset S01 (PDF)

Dataset S02 (PDF)

## Data Availability

The data that supports the findings of this study including dye synthetic schemes are available from the corresponding author, upon reasonable request. The custom developed MATLAB code for micrograph particle segmentation and EMBER analysis as well as raw and normalized in vitro fibril EMBER data is available here: https://doi.org/10.7272/Q6G73BZN. All study data are included in the article and/or *SI Appendix*.
