# Supplementary material for: EMBER multidimensional spectral microscopy enables quantitative determination of disease- and cell-specific amyloid strains
Source: Proc Natl Acad Sci U S A. 2023 Mar 16;120(12):e2300769120. doi: 10.1073/pnas.2300769120 (PMC10041141; doi:10.1073/pnas.2300769120)
Supplement: Supplementary file 1 — Appendix 01 (PDF) [file pnas.2300769120.sapp.pdf]

SUPPORTING INFORMATION FOR:

EMBER multi-dimensional spectral microscopy enables quantitative determination of disease- and cell-specific amyloid strains

Hyunjun Yang[a,b], Peng Yuan[a], Yibing Wu[b], Marie Shi[a], Christoffer D. Caro[a], Atsushi Tengeiji[c], Shigeo Yamanoi[c], Masahiro Inoue[c], William F. DeGrado[a,b]*, Carlo Condello[a,d]*

[a]Institute for Neurodegenerative Diseases, University of California, San Francisco, CA 94143
[b]Department of Pharmaceutical Chemistry, Cardiovascular Research Institute, University of California, San Francisco, CA 94158
[c]Daiichi Sankyo Co. Ltd, Tokyo, Japan.
[d]Department of Neurology, University of California, San Francisco, CA 94143

Email: William.DeGrado@ucsf.edu and Carlo.Condello@ucsf.edu

Table of Contents

Additional Materials and Methods

Production of Aβ40 and Aβ42 fibrils S1
Production of α-Syn fibril and ribbon S2
Production of 0N3R tau and 0N4R tau fibrils. S3
EMBERnet data preparation. S4
EMBERnet model architecture. S5
EMBERnet model training and testing. S6
Mouse brain samples. S7

Supplementary Figures

Fig. S1. EMBER reproducibility study against *in vitro* fibrils. S8
Fig. S2. Randomization and quadratic discrimination of *in vitro* fibril data. S9
Fig. S3. Data preparation for EMBERnet. S10
Fig. S4. EMBERnet model architecture. S11
Fig. S5. EMBERnet model training and testing. S12
Fig. S6. EMBER vs single-wavelength excitation comparison against *in vitro* fibrils. S13
Fig. S7. Randomization and quadratic discrimination of plaque data in Tg mice. S14
Fig. S8. Validation of dye 60 dual XM properties in sAD sample using immunohistochemistry. S15
Fig. S9. EMBER plots of Aβ plaques across neurogenerative samples. S16
Fig. S10. PC1/PC2 and PC1/PC3 of Aβ plaque EMBERs across neurodegenerative diseases. S17
Fig. S11. Inter-patient heterogeneity plot for Aβ plaques. S18
Fig. S12. EMBER vs single-wavelength excitation comparison for Aβ plaques. S19
Fig. S13. EMBER plots of tau tangles across neurodegenerative samples. S20
Fig. S14. Inter-patient heterogeneity plot for tau tangles. S21
Fig. S15. Validation of dye 60 cell-type specific labeling in PiD sample using immunohistochemistry. S22
Fig. S16. EMBER reproducibility study against Pick astrocytes. S23
Fig. S17. EMBER vs single-wavelength excitation comparison for tau tangles. S24
Table S1. Dye structure, name, discrimination score, and λ_{max} em/ex against *in vitro* fibrils. S25
Table S2. Sources of postmortem human brain tissue samples. S48
Data S1. *In vitro* fibril PCA plots.
Data S2. *In vitro* fibril UMAP plots.

Production of Aβ40 and Aβ42 fibrils

Aβ fibrils were produced following published protocols to produce homogenous preparations for structural determination.[1] HPLC-purified and lyophilized Aβ40 and Aβ42 as TFA salts were purchased from rPeptide. 0.2 mg of lyophilized Aβ40 or Aβ42 was dissolved in 40 μL HFIP (5 mg/mL). The HFIP was evaporated overnight in the fume hood covered by Kimwipe and the resulting Aβ samples were speedvac for 30 min. The Aβ film was resuspended in 20 μL DMSO (10 mg/mL). The solution was briefly vortexed, water bath sonicated for 5 min, then diluted with 10 mM NaPhos buffer to 0.2 mg/mL final protein solution. The solution was shaken in Thermomixer at 900 RPM for 72 h at 37 °C.

[1] Wang, T., Jo, H., DeGrado, W. F., Hong, M. Water Distribution, Dynamics and Interactions with Alzheimer's β-Amyloid Fibrils Investigated by Solid-State NMR. *J. Am. Soc. Chem.* **139**, 6242–6252 (2017).

Production of α-Syn fibril and ribbon

α-Syn fibrils were produced following published protocols to produce homogenous preparations.[2,3,4] pET28a (kanamycin selected) vector encoding human α-synuclein was transformed into the E. coli strain BL21(DE3). Bacteria were grown to OD_{600}=0.8 then IPTG induced for 3 h. The cells were pelleted, resuspended in 100 mL osmotic shock buffer (20 mM Tris-HCl, 40% sucrose, 2 mM EDTA, pH 7.2), incubated at room temp for 10 min, then centrifuged at 12k rpm for 20 min. The supernatant was decanted then the pellet was resuspended in 100 mL cold water containing 40 μL saturated $MgCl_2$. The solution was left on ice for 3 min and centrifuged at 12k rpm for 20 min. The resulting supernatant was collected and lyophilized. The lyophilized powder was dissolved in 20 mL 5% acetonitrile (MeCN) containing 0.1% TFA, then filtered through 0.45-μm polypropylene syringe filter and purified on a semi-prep C4 HPLC column (H_2O/MeCN containing 0.1% TFA; 5% to 95% MeCN over 30 min). The pure α-synuclein fractions were collected and lyophilized. To prepare α-Syn fibrils*,* lyophilized α-synuclein was dissolved in 50 mM Tris-HCl, pH 7.5, 150 mM KCl to result in a final concentration of 100 μM α-Syn. To prepare α-Syn ribbons, lyophilized α-synuclein was dissolved in 5 mM Tris-HCl pH 7.5 to result in a final concentration of 100 μM α-Syn. Both fibrillization conditions were shaken in a Thermomixer at 1000 rpm for 7 d at 37 °C. The fibril quality was controlled with YFP-labelled cell aggregation assay, sedimentation assay, and TEM imaging.

---

[2] Bousset, L., et al. Structural and functional characterization of two alpha-synuclein strains. *Nat. Commun.* **4**, 2575 (2013).

[3] Huang, C. et al. A new method for purification of recombinant human α-synuclein in Escherichia coli. *Protein Expr. Purif.* **42**, 173–177 (2005).

[4] Kloepper, K. D. et al. Preparation of α-synuclein fibrils for solid-state NMR: expression, purification, and incubation of wild-type and mutant forms. *Protein Expr. Purif.* **48**, 112–117 (2006).

Production of 0N3R tau and 0N4R tau fibrils.

Tau fibrils were produced following published protocols to produce homogenous preparations for structural determination.[5,6] The E. coli strain Rosetta (DE3) was transformed with pET28a (kanamycin selected) vector encoding 0N3R tau and 0N4R tau. The bacteria were grown to OD_{600}=0.8 then IPTG induced for 6 h. Cells were pelleted and resuspended in 300 mL lysis buffer containing 20 mM MES (pH 6.8), 1 mM EGTA, 0.2 mM MgCl2, 5 mM DTT, and 1xcOmplete™ protease inhibitor cocktail (Roche). Cells were lysed with microfluidizer then boiled for 20 min, and the resulting solution was spun at 24,500 g. The supernatant was purified with a cation exchange column (self-packed with SP Sepharose Fast Flow resin, GE Healthcare) then further purified with a reverse-phase HPLC equipped with ZORBAX 300SB-C3 column (MeCN gradient from 5-50% over 45 min). HPLC fractions containing pure 0N3R tau and 0N4R tau were combined and lyophilized to result in a protein powder as TFA salts. The fibrillization of both 0N3R and 0N4R tau were performed in a 1.5-mL Eppendorf tube at 0.4 mg/mL tau concentration with 0.125 mg/mL heparin (8,000-25,000 Da). 0N4R tau was fibrillized in 1x PBS containing 1 mM DTT and 0N3R tau was fibrillized in 1x PBS containing 1 mM TCEP (pH 7). The solution was shaken at 37˚C and 1400 rpm for 3 d. The fibril quality was controlled with aggregation assay, sedimentation assay, trypsin-digested SDS-PAGE gel, and TEM imaging.

---

[5] Dregni, A. J., Mandala, V. S., Wu, H., Elkins, M. R., Wang, H. K., Hung, I., DeGrado, W. F., Hong, M. In vitro 0N4R tau fibrils contain a monomorphic β-sheet core enclosed by dynamically heterogeneous fuzzy coat segments. *Proc. Natl. Acad. Sci.* **116**, 16357–16366 (2019).

[6] Dregni, A. J., Wang, H. K., Wu, H., Duan, P., Jin, J., DeGrado, W. F., Hong, M. Inclusion of the C-Terminal Domain in the β-Sheet Core of Heparin-Fibrillized Three-Repeat Tau Protein Revealed by Solid-State Nuclear Magnetic Resonance Spectroscopy. *J. Am. Chem. Soc.* **143**, 7839–7851 (2021).

EMBERnet data preparation*.*

This neural network problem was formulated as an image classification problem that is solved using EMBERnet, a ResNet-based deep learning architecture.[7] An image was constructed for each particle from EMBER dataset creating a size 12 X 18 X 64 image representing 12 excitations and 18 emission windows. The image was resized to 12 X 128 X 128 with *OpenCV* with interpolation from the original set of pixels.[8] The final images were fed for deep learning model.

[7] He, K., Zhang, X., Ren, S., Sun, J. Deep Residual Learning for Image Recognition. *Proc. IEEE Comput. Soc. Conf. Comput. Vis. Pattern Recognit.* 770–778 (2016).

[8] https://pypi.org/project/opencv-python/

EMBERnet model architecture.

The basic unit of neural network architecture is a residual block, which consists of two convolutional layers[9], a batch-normalization layer[10], and a rectified linear unit (ReLU)[11]. Residual blocks were also applied to overcome the problem of exploding/vanishing gradients due to increasing the depth of neural networks.[7] Finalized EMBERnet model consisted of 22 layers — 20 convolutional layers and 2 multi-layer perceptron (MLP) layers. The skip connection was used to develop deep network with 20 layers, and also increased the convergence speed during training.[7]

---

[9] Alex Krizhevsky, A., Sutskever, I., Hinton, G. E. *Commun. ACM*, **60**, 84–90 (2017)

[10] Ioffe, S., Szegedy, C. Batch Normalization: Accelerating Deep Network Training by Reducing Internal Covariate Shift. Proceedings of the 32nd International Conference on Machine Learning, PMLR **37,** 448–456 (2015)

[11] Agara, A. F. Deep Learning using Rectified Linear Units (ReLU) (2019) https://doi.org/10.48550/arXiv.1803.08375

EMBERnet model training and testing. To find the optimum discrimination score of EMBER dataset, 10-fold Cross-Validation (CV) was utilized.[12] The EMBER dataset was randomly shuffled and then split in to 10 folds (0–9-folds) to ensure the distributions of the six fibril types are similar. For the training, validation, and test set, we utilized 8-folds (0–7-folds), 1-fold (fold-8), and 1-fold (fold-9) respectively. The models were trained for the maximum 120 epochs with a batch size of 32 on one NVIDIA GPU. During the training, cross-entropy loss between the target and predicted outputs was minimized using Adam optimizer.[13] The learning rate of the optimizer was set to 0.00001 at the beginning of the training, and then reduced by a factor of 10 after approximately 100 epochs if no improvement in the discrimination score was observed after 3 epochs. The model training is then terminated if no performance improvement was observed after 6 epochs. After the performance of the training, validation, and test sets were confirmed, we finalized the EMBERnet and moved onto EMBERnet validation by shifting the folds by 1. Here, we used the folds-1–8 for the training set, fold-9 as the validation set, and fold-0 as the test set, and the EMBERnet performance was similar validating our EMBERnet training and performance. The code for EMBERnet is available per request.

---

[12] Bates, S., Hastie, T., Tibshirani, R. Cross-validation: what does it estimate and how well does it do it? (2022) https://arxiv.org/abs/2104.00673

[13] Kingma, D. P., Ba, J. Adam: A Method for Stochastic Optimization. (2017) https://arxiv.org/abs/1412.6980

Mouse brain samples.

All comparisons were conducted on brains from age-matched animals. Animals were maintained in a facility accredited by the Association for Assessment and Accreditation of Laboratory Animal Care International in accordance with the Guide for the Care and Use of Laboratory Animals.[14] All procedures for animal use were approved by the University of California, San Francisco's Institutional Animal Care and Use Committee. Tg(APP23) mice, which express human APP (751-aa isoform) containing the Swedish mutation under the control of the Thy-1.2 promoter, were maintained on a C57BL/6 background.[15] Tg(5xFAD) mice, which express human APP containing the Swedish, Florida, and London mutation with PSEN1 harboring M146L and L286V mutation under the control of the Thy-1.2 promoter, were maintained on a C57BL/6 background.[16] In this study, Tg(APP23) mice were 24-months old and Tg(5xFAD) mice were 12-months-old. Mouse brain samples were harvested, immersion-fixed in 10% buffered formalin, and then embedded in paraffin following standard procedures.

[14] Committee for the Update of the Guide for the Care and Use of Laboratory Animals IfLAR; Division of Earth and Life Sciences; National Research Council of the National Academies (2011) Guide for the Care and Use of Laboratory Animals (The National Academies Press, Washington, DC), 8th Ed.

[15] Sturchler-Pierrat. C., et al. Two amyloid precursor protein transgenic mouse models with Alzheimer disease-like pathology. *Proc. Natl. Acad. Sci.* **94**, 13287–13292 (1997).

[16] Oakley, H., et al. Intraneuronal beta-amyloid aggregates, neurodegeneration, and neuron loss in transgenic mice with five familial Alzheimer's disease mutations: potential factors in amyloid plaque formation. *J. Neurosci.* **26**, 10129–10140 (2006).

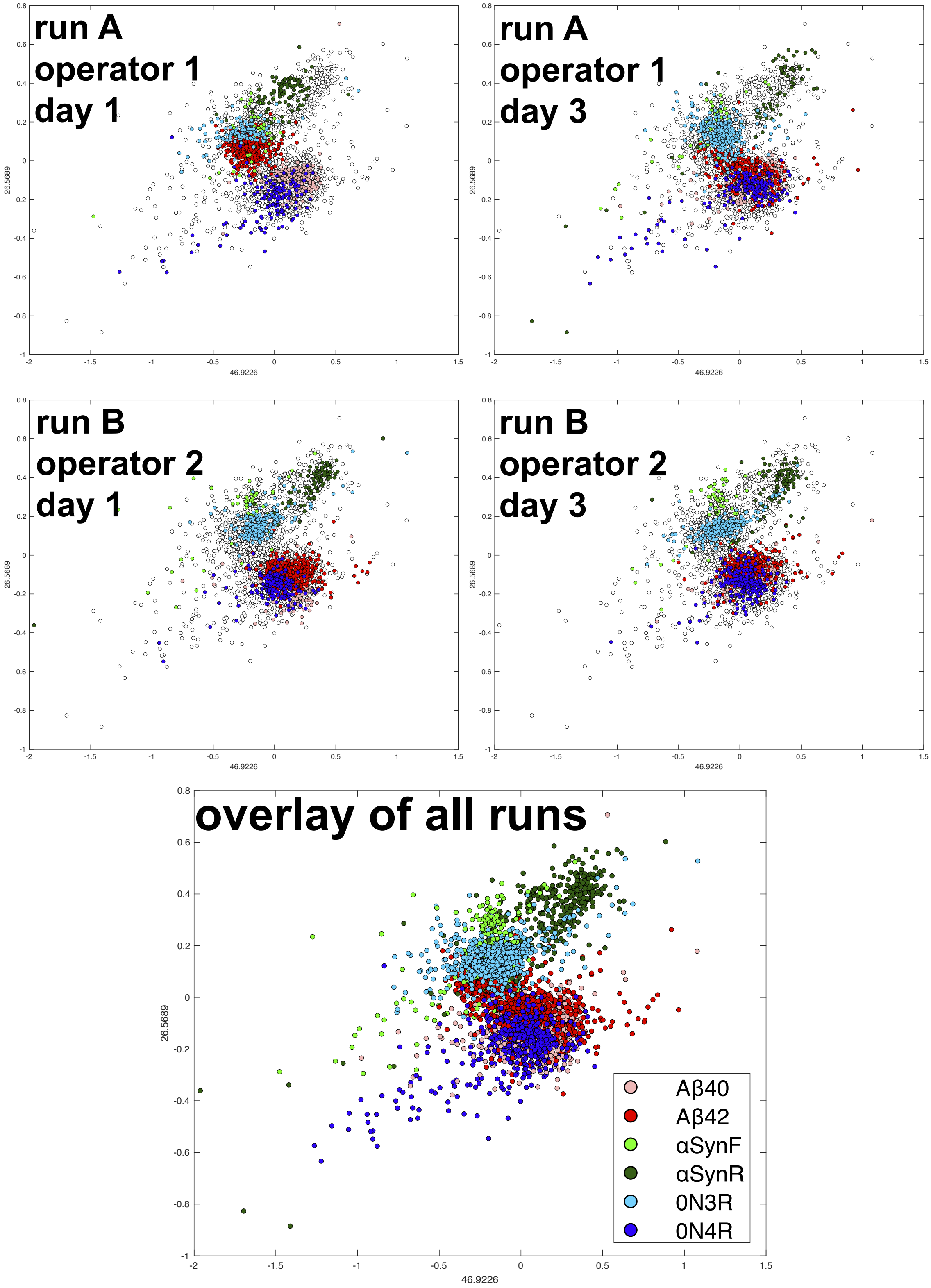

Fig. S1. EMBER reproducibility of six *in vitro* fibril types. Two operators (1 and 2) each prepared samples and collected EMBER dataset (run A and B) against six *in vitro* fibrils (Aβ40, Aβ42, αSynF, αSynR, 0N3R, 0N4R). EMBER data for each run was recollected at day 3. All four individual EMBER datasets were combined in an array and PCA was performed. PCA plots of overlay as well as the four individual runs are shown above.

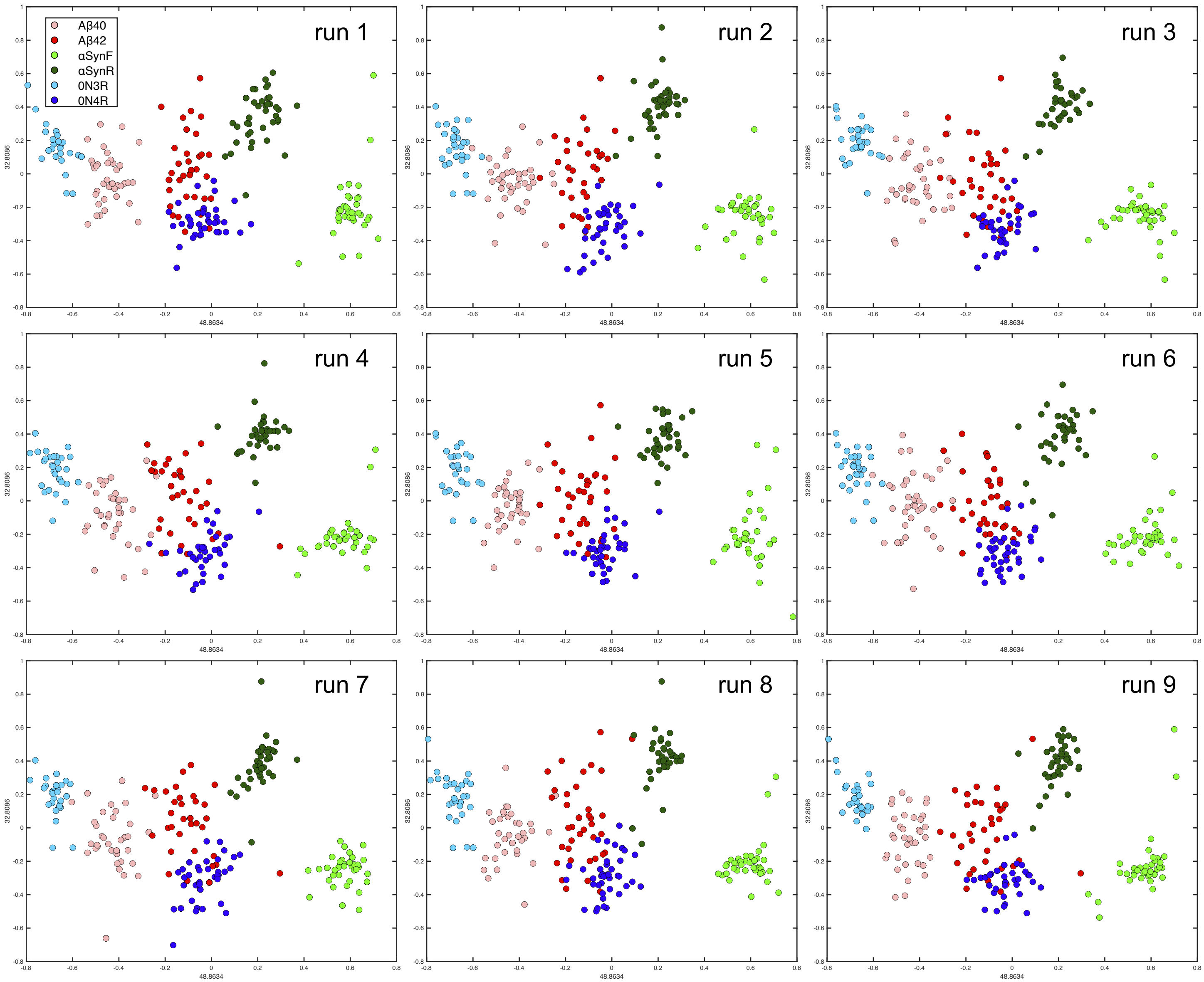

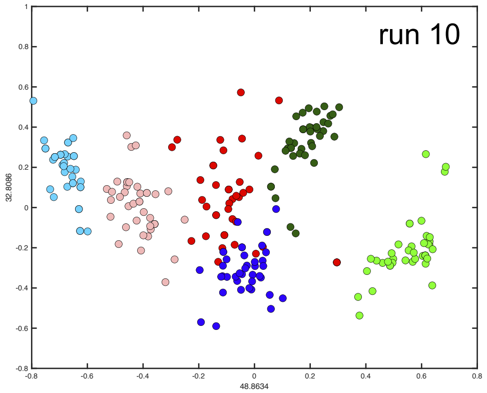

Run #	Discrimination scores	Average discrimination scores
run 1	93%	92%
run 2	91%	
run 3	94%	
run 4	93%	
run 5	94%	
run 6	92%	
run 7	93%	
run 8	92%	
run 9	91%	
run 10	90%	

Fig. S2. 10 randomized data point selections and quadratic discrimination for six *in vitro* fibril types. Ten repetitions of quadratic discrimination cluster classification algorithm were performed to quantify discrimination score against *in vitro* fibrils. For PCA or UMAP plot, 40 random particles from each fibril dataset were concatenated into an array and grouped for quadratic discrimination. Discrimination score is calculated per run as shown above, and the average of ten discrimination scores was used as the discrimination score as presented in the main Figure 1.

(a)

	excitation	405	470	490	510	530	550	570	590	610	630	650	670
emission		0	1	2	3	4	5	6	7	8	9	10	11
420	0	110											
440	1	111											
460	2	112											
480	3	113	0										
500	4	114	1	15									
520	5	115	2	16	29								
540	6	116	3	17	30	42							
560	7	117	4	18	31	41	54						
580	8	118	5	19	32	40	55	65					
600	9	119	6	20	33	39	56	64	75				
620	10	120	7	21	34	38	57	63	76	84			
640	11	121	8	22	35	37	58	62	77	85	92		
660	12	122	9	23	36	36	59	61	78	86	93	99	
680	13	123	10	24	37	35	60	60	79	87	94	100	105
700	14	124	11	25	38	34	61	59	80	88	95	101	106
720	15	125	12	26	39	33	62	58	81	89	96	102	107
740	16	126	13	27	40	32	63	57	82	90	97	103	108
760	17	127	14	28	41	31	64	56	83	91	98	104	109

(b)

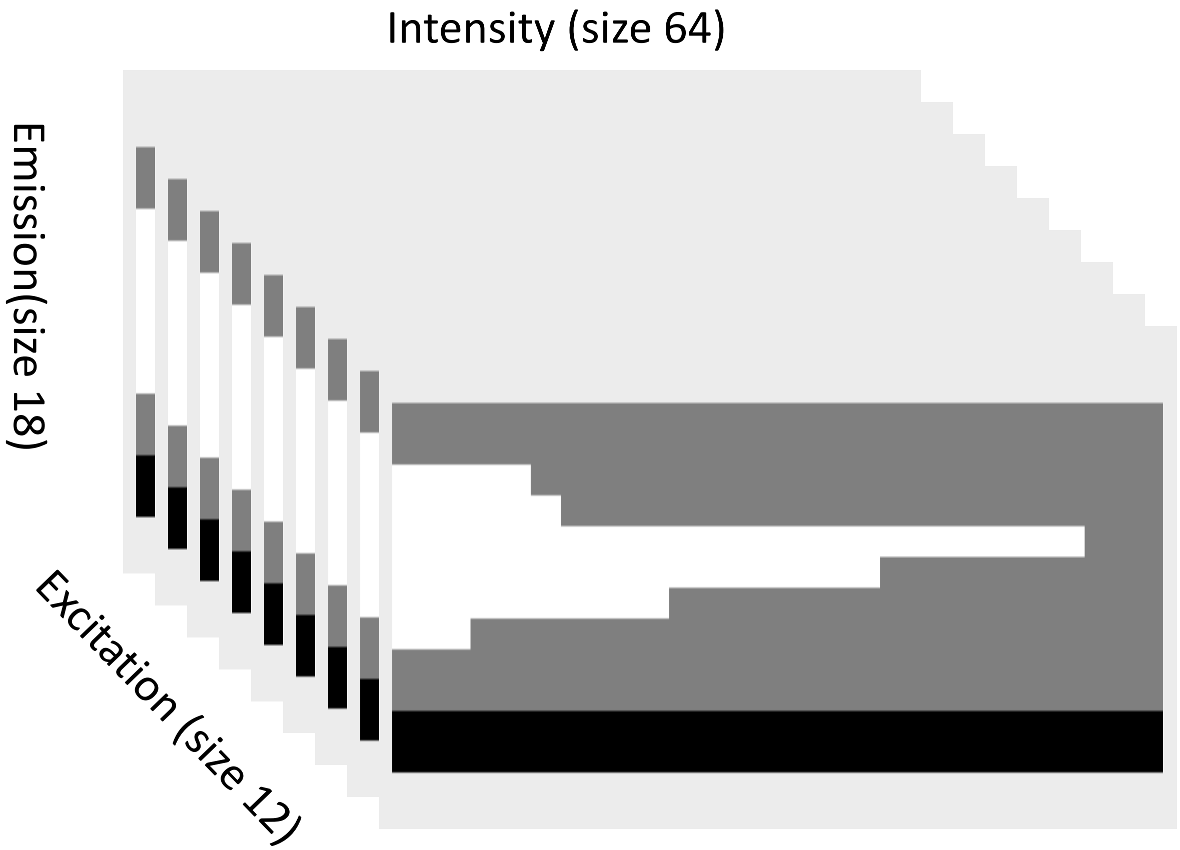

Fig. S3. The problem was formulated as an image classification problem that was investigated by an in-house ResNet-based deep learning architecture. (a) EMBER data format consisted of 12 excitations and 18 emission windows. (b) The constructed image for each experiment with size 12 x 18 x 64. The images were then resized to 12 X 128 X 128 by interpolating the pixel from the original set of pixels and were fed for the deep learning model.

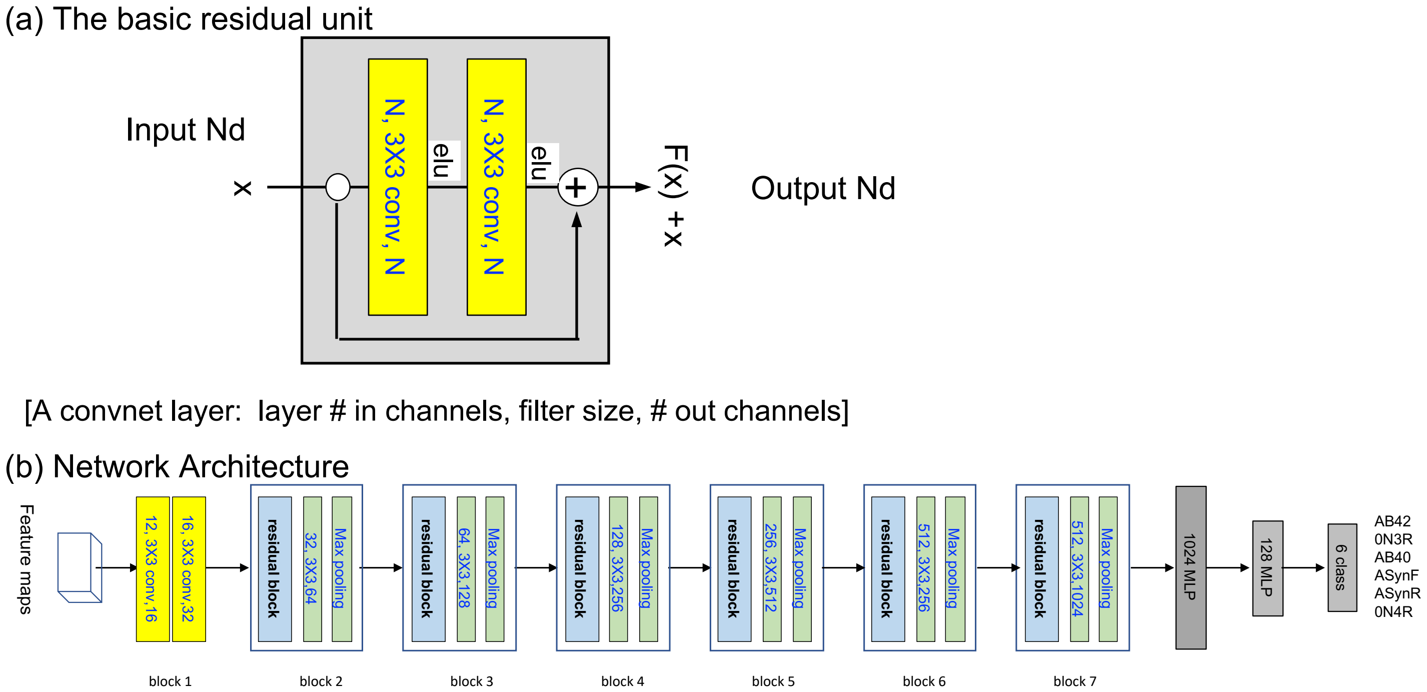

Fig. S4. Outline of ResNet-based 20-layer convolution neural network for multi-class image classification for each fibril types and all fibril types.

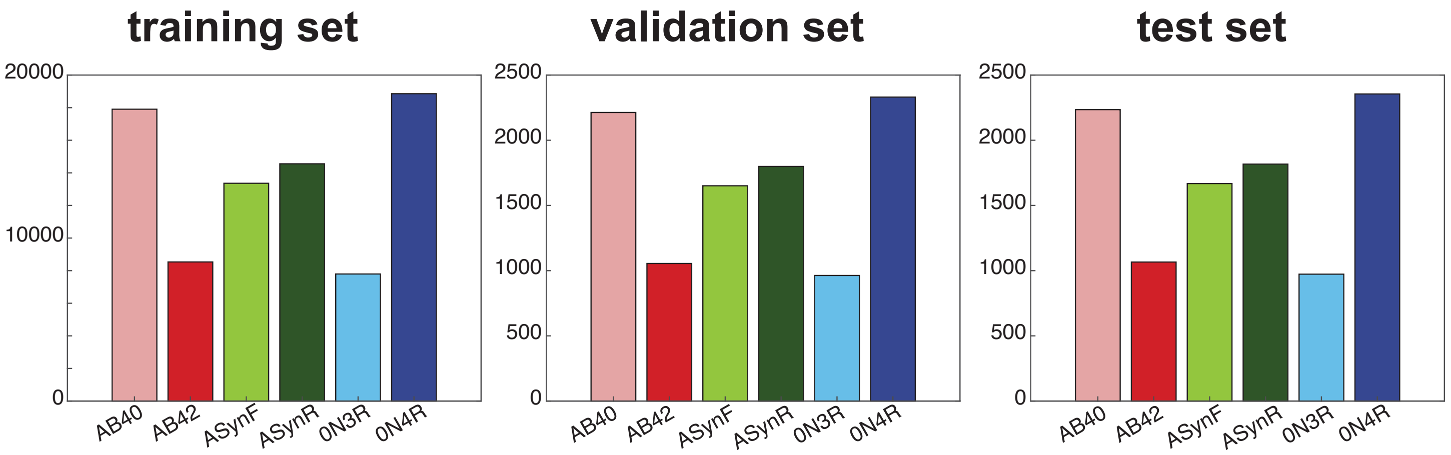

Fig. S5. The sample distribution of six fibril types in the first of 10 runs, where fold 0 to 7 are pooled as train set, fold 8 as validation set, and fold 9 as test set.

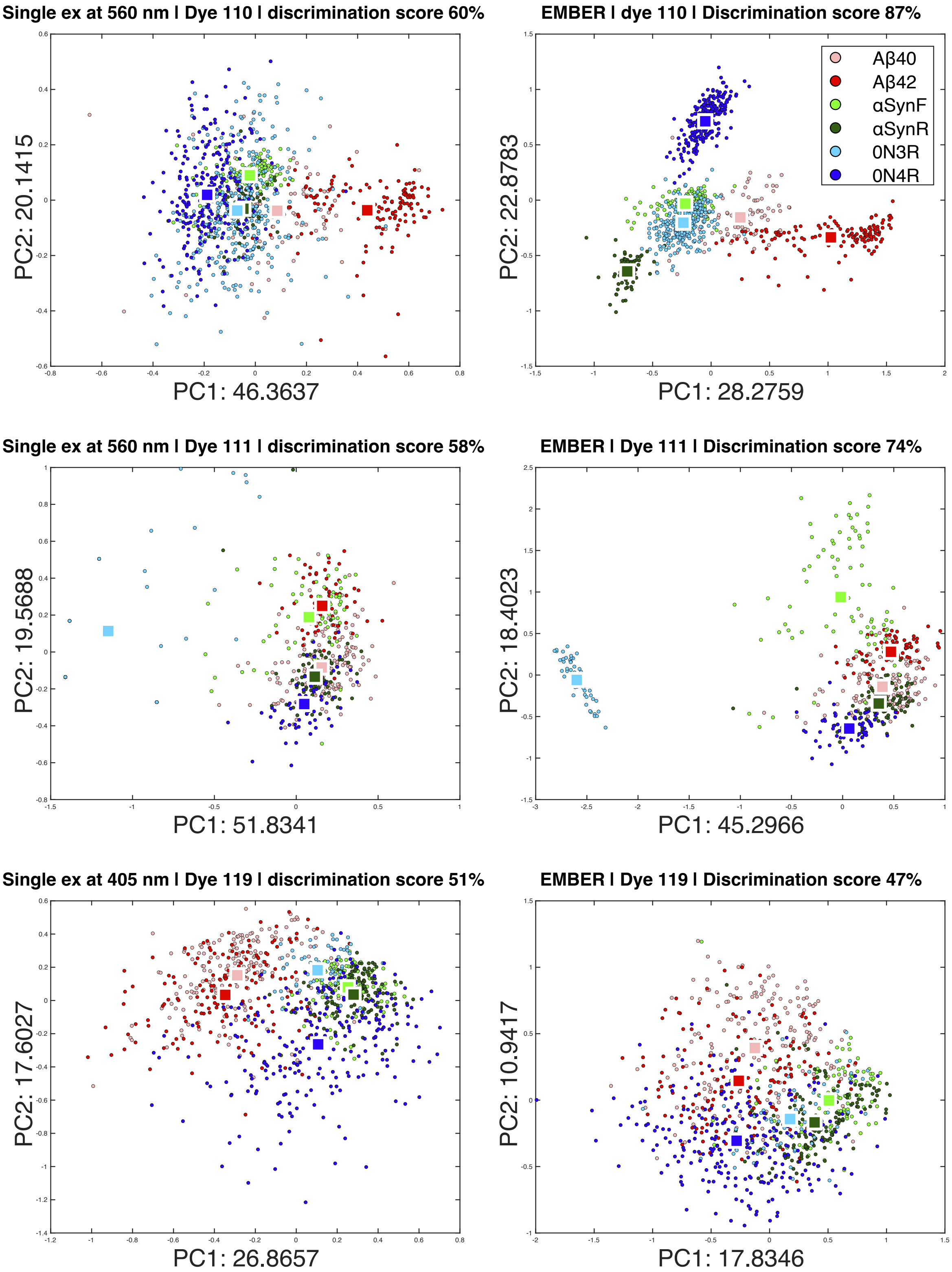

Fig. S6. EMBER vs single-wavelength excitation discrimination power comparison for six *in vitro* fibril types. λ_{max} single-wavelength excitation data of three dyes (110, 111, 119) from *in vitro* fibril dataset were pulled, and PCA and UMAP analysis were performed (left). The discrimination scores of single-wavelength excitations are lower than that of EMBER (right) representing the outperformance of EMBER in maximizing photophysical property of dyes bound to amyloid fibrils and to discriminate conformational strains of fibrils.

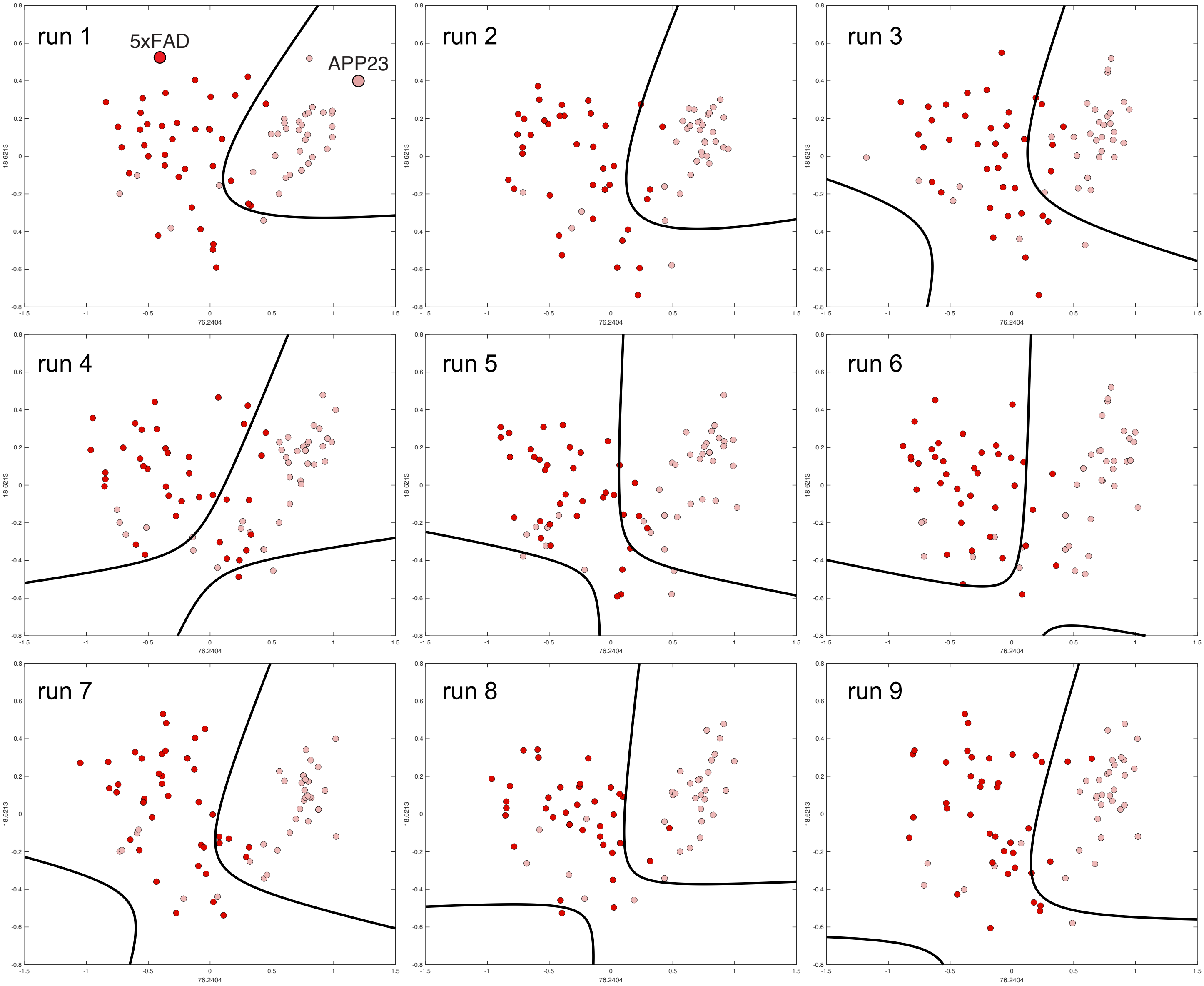

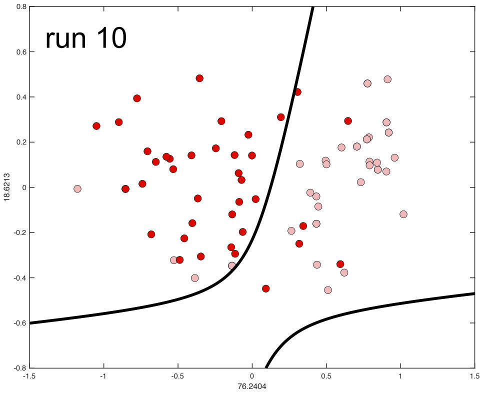

Run #	Discrimination scores	Average discrimination scores
run 1	89%	84.3%
run 2	88%	
run 3	84%	
run 4	80%	
run 5	81%	
run 6	83%	
run 7	86%	
run 8	84%	
run 9	85%	
run 10	83%	

Fig. S7. 10 repetitions of quadratic discrimination cluster classification algorithm were performed to quantify discrimination score of plaques in brains of two mouse models. For PCA or UMAP plot, 40 random particles from each fibril sets were concatenated into an array and grouped for quadratic discrimination. Discrimination score is calculated per run, and the average of ten discrimination scores was used as the discrimination score as presented in the main Figure 3. Boundaries pertaining fit discriminants are presented in black lines.

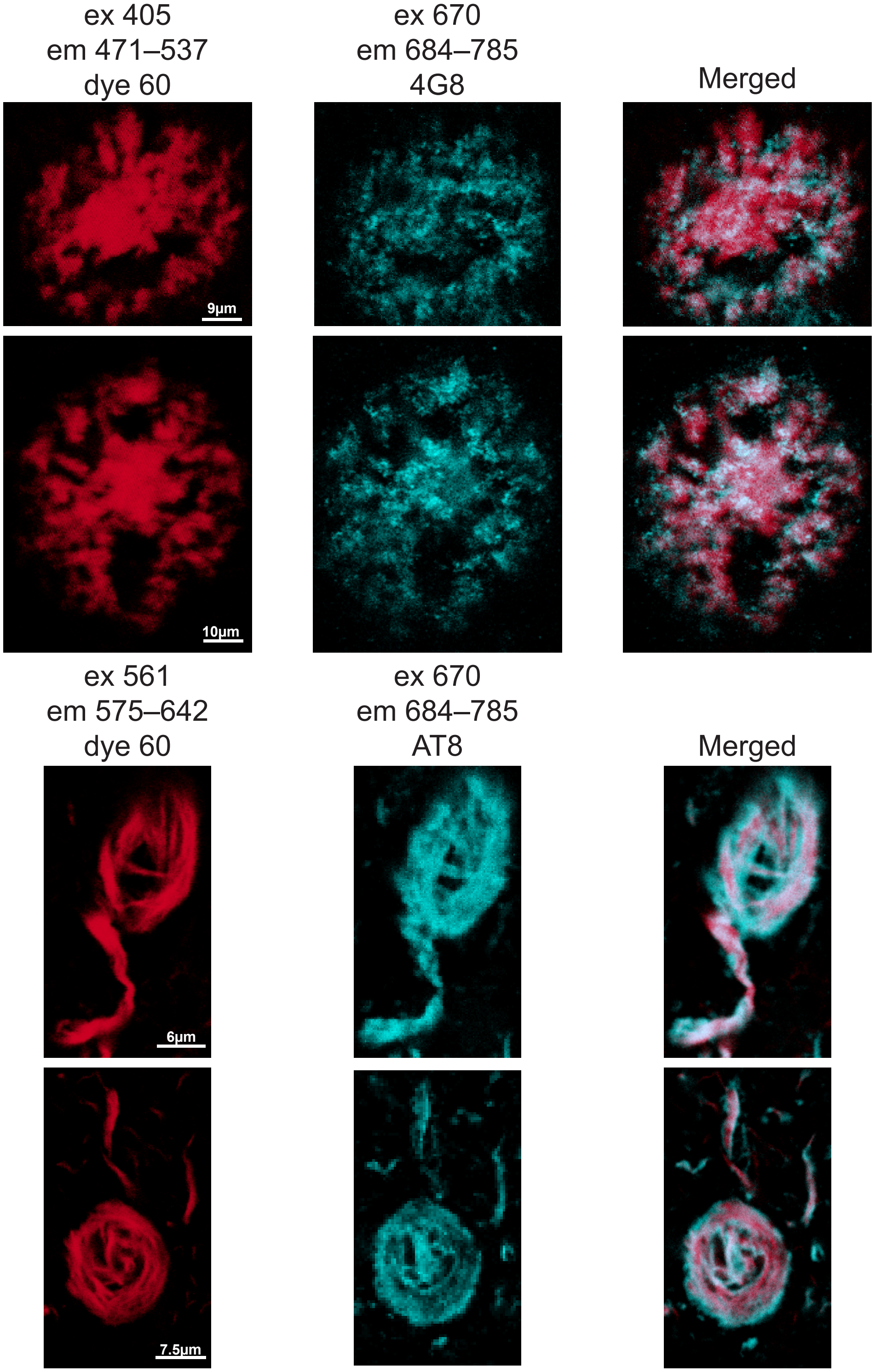

Fig. S8. sAD brain donor samples are co-stained with dye 60 and either 4G8 (Aβ) or AT8 (tau) antibody. Dye 60 excites Aβ plaques at 405 nm and tau tangles at 561 nm. The 4G8-stained Aβ plaques or and AT8-stained tau tangles are excited at 670 nm. The merged micrographs show good overlap between dye 60 labeling and corresponding immunostaining. For antibody staining, antigens were first retrieved by autoclaving FFPE sections in 0.01 M Citrate buffer followed by blocking with 10% goat serum. After incubating slides with the primary antibody in 10% goat serum, secondary antibody labelled with Alexa fluor 647 was applied.

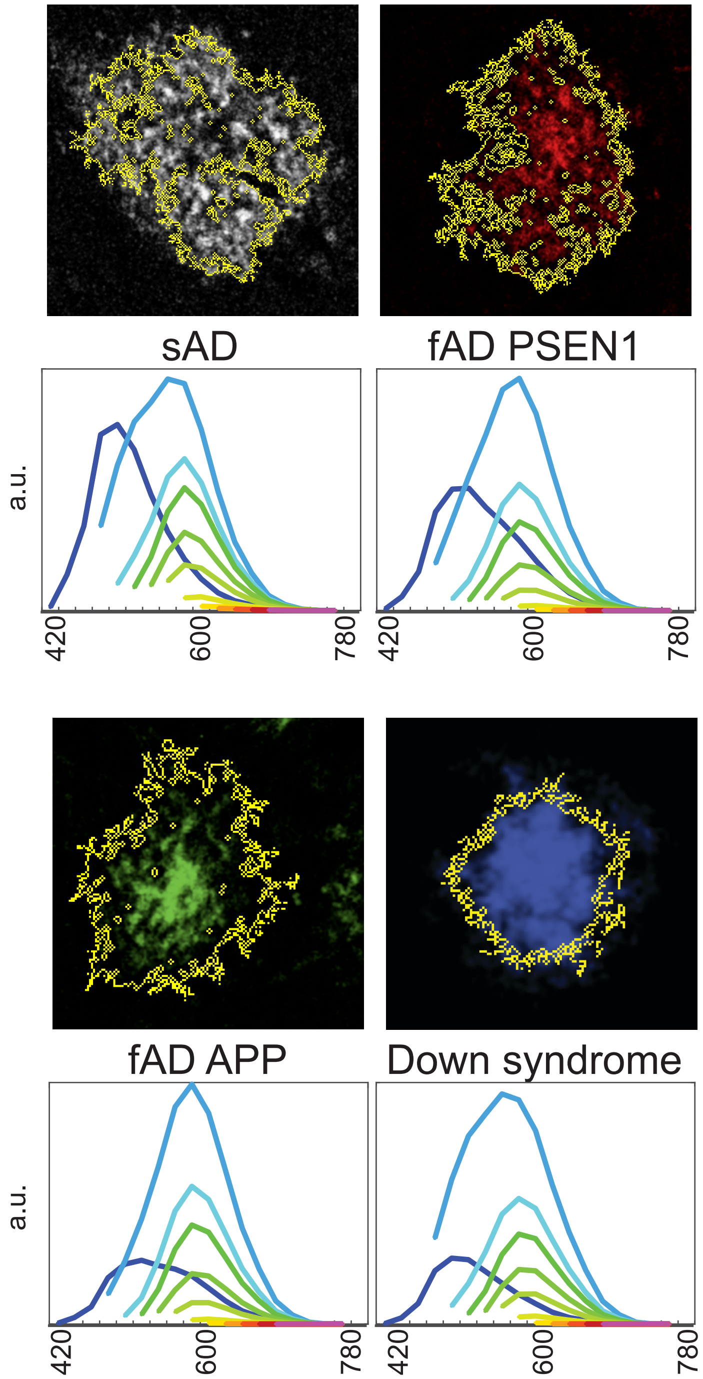

Fig. S9. Average EMBER plots for dye 60 stained Aβ plaques across neurogenerative disease brain donor samples. Yellow outlines indicate the particle segmentation performed by customized MATLAB algorithms. Each segmented particle becomes one particle on PCA and UMAP plots.

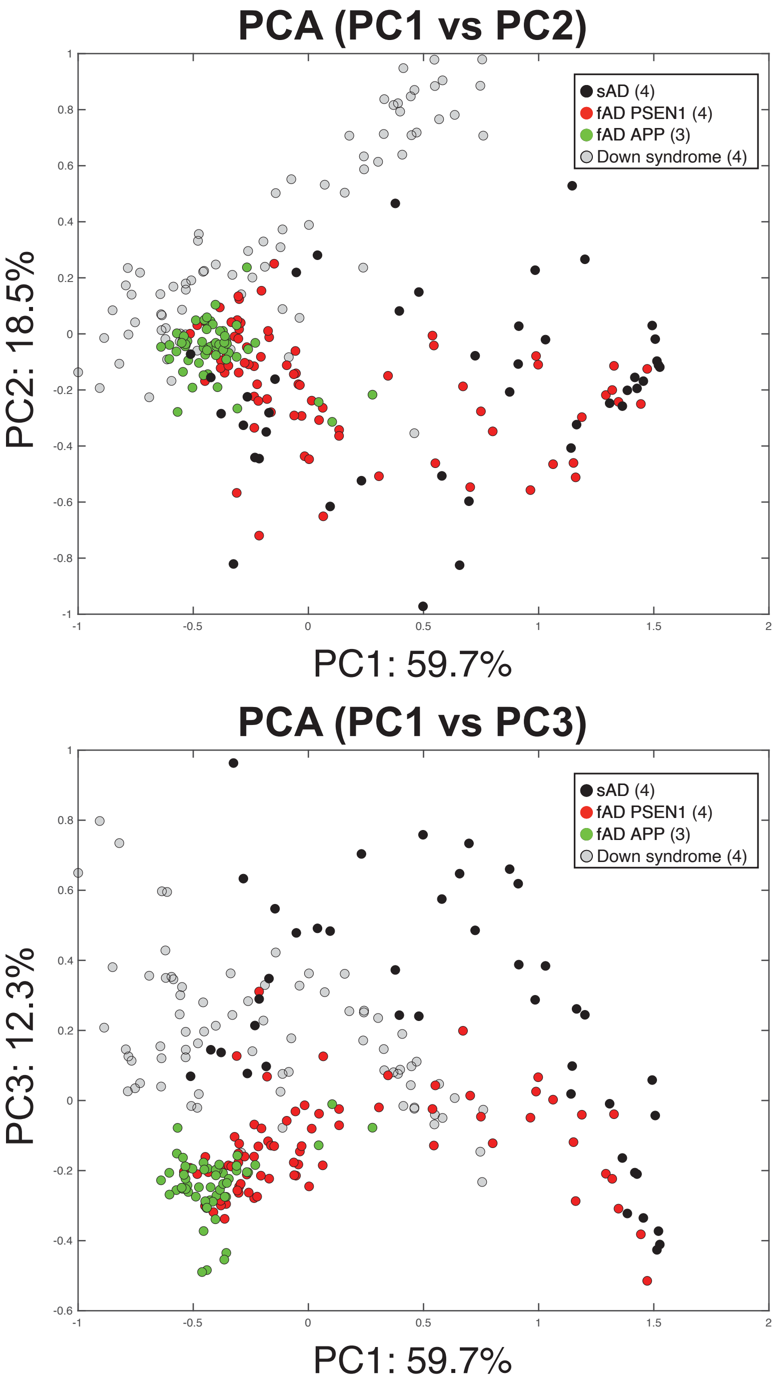

Fig. S10. PC1/PC2 and PC1/PC3 PCA plots for Aβ plaques data across neurodegenerative diseases. The number of brain donor samples in each cohort is within the parenthesis.

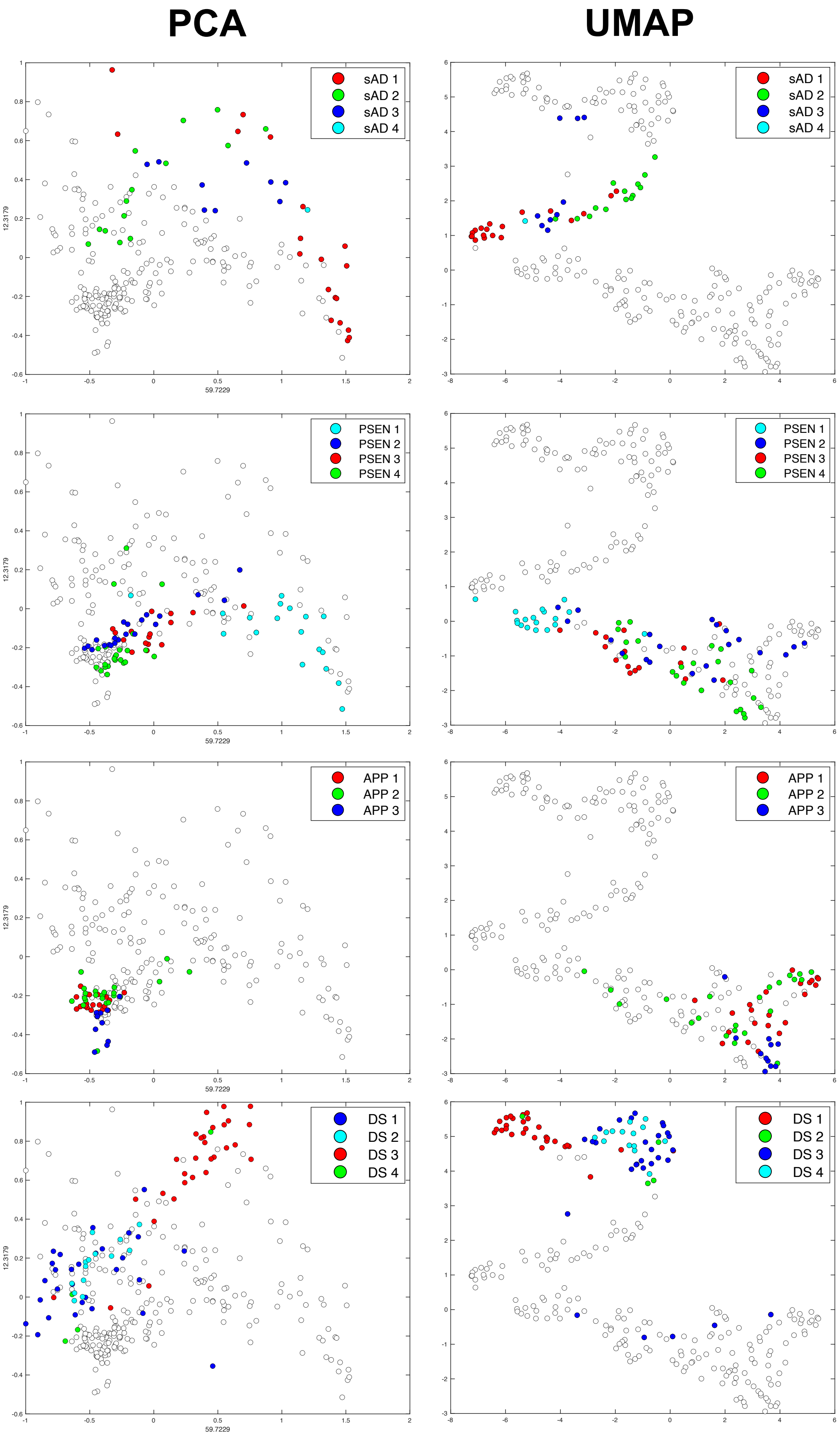

Fig. S11. Inter-patient heterogeneity plot for Aβ plaque EMBER data. As shown in main Fig. 6a, Aβ plaques from each patient in each disease cohort were re-plotted using varying colors. To aid visibility, particles pertaining to other EMBER datasets were colored white.

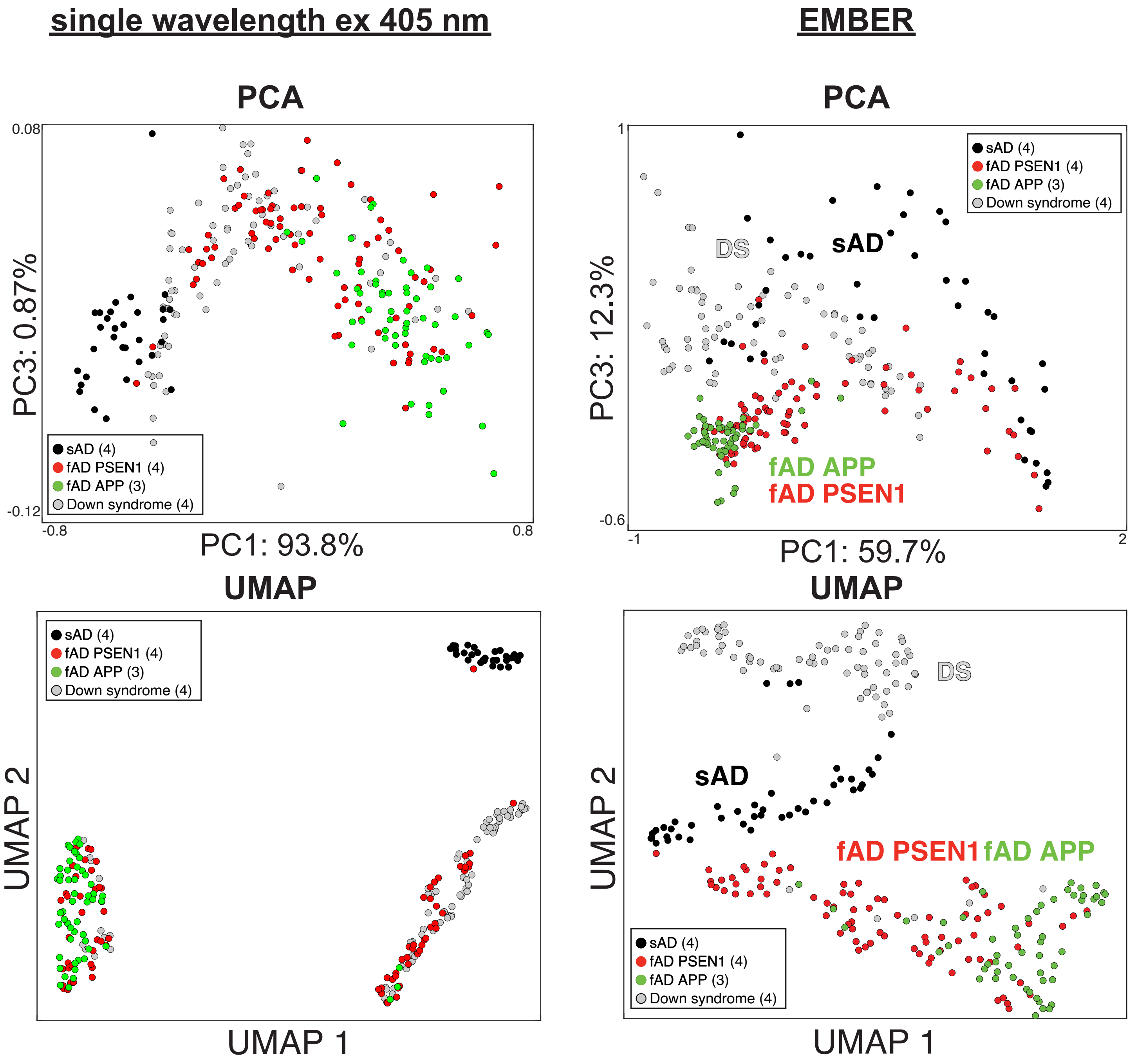

Fig. S12. EMBER vs single-wavelength excitation discrimination power comparison for Aβ plaques. 405 nm single-wavelength excitation data was pulled, and PCA and UMAP analysis were performed (left). For single-wavelength excitation, the separation between clusters is worse than that of EMBER (right) representing the outperformance of EMBER in maximizing photophysical property of dyes bound to Aβ plaques and to discriminate conformational strains of fibrils. The number of donor samples in each cohort is within the parenthesis.

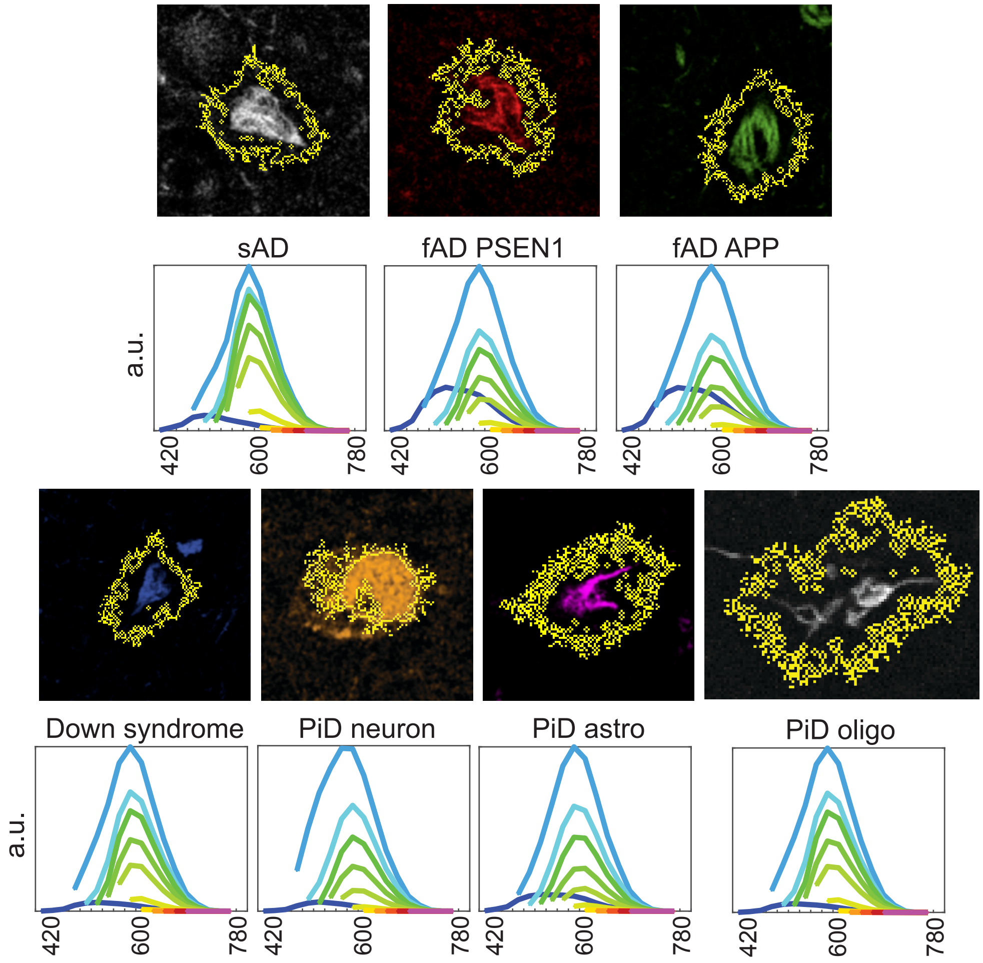

Fig. S13. Average EMBER plots for dye 60 stained tau deposits across neurogenerative disease brain donor samples. Yellow outlines indicate the particle segmentation performed by customized MATLAB algorithms. Each segmented particle becomes one particle on PCA and UMAP plots.

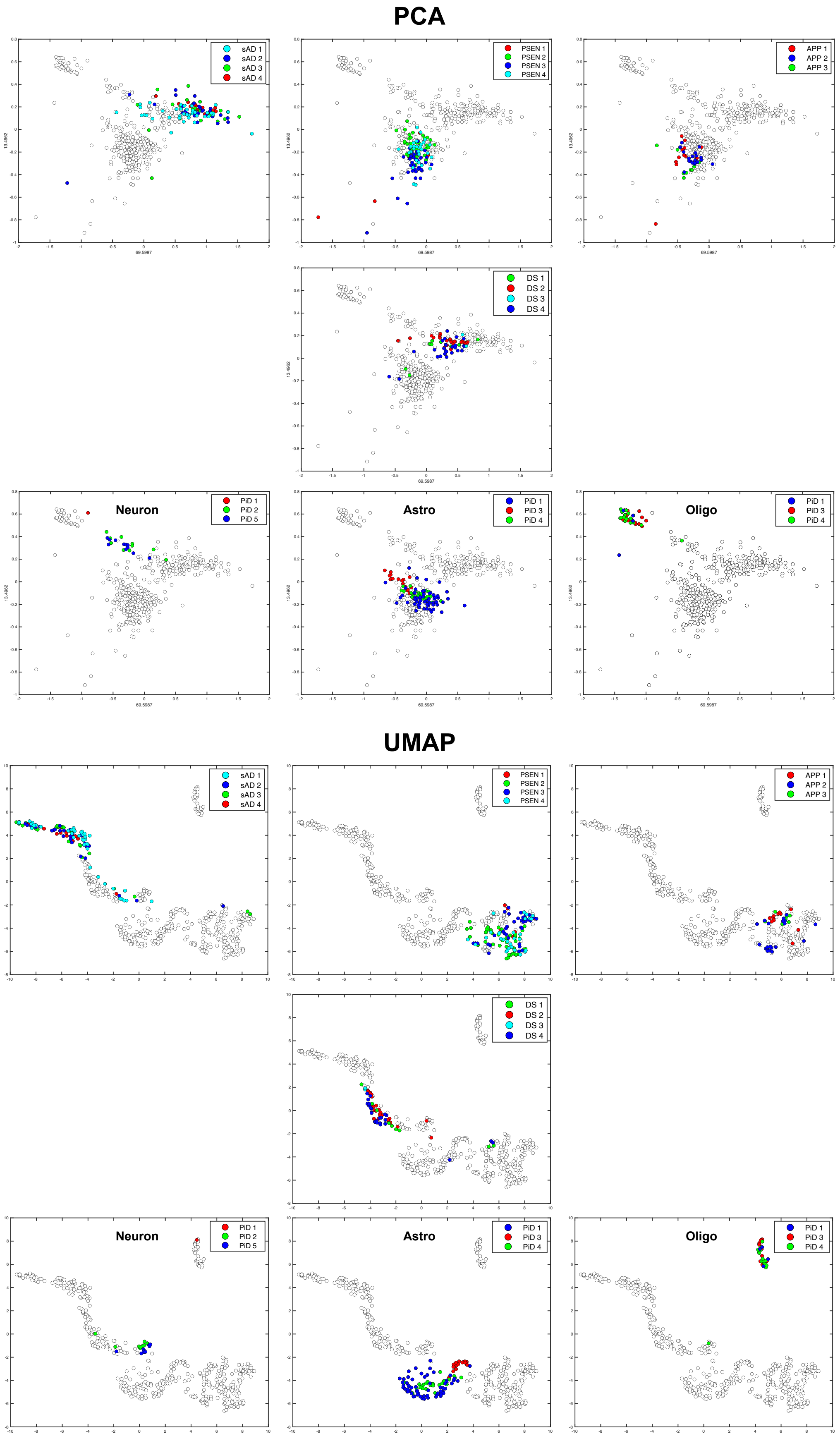

Fig. S14. Inter-patient heterogeneity plot for tau deposit EMBER data. As shown in main Fig. 6c, tau deposits of each patient in each disease cohort were re-plotted using varying colors. To aid visibility, particles pertaining to other EMBER datasets were colored white.

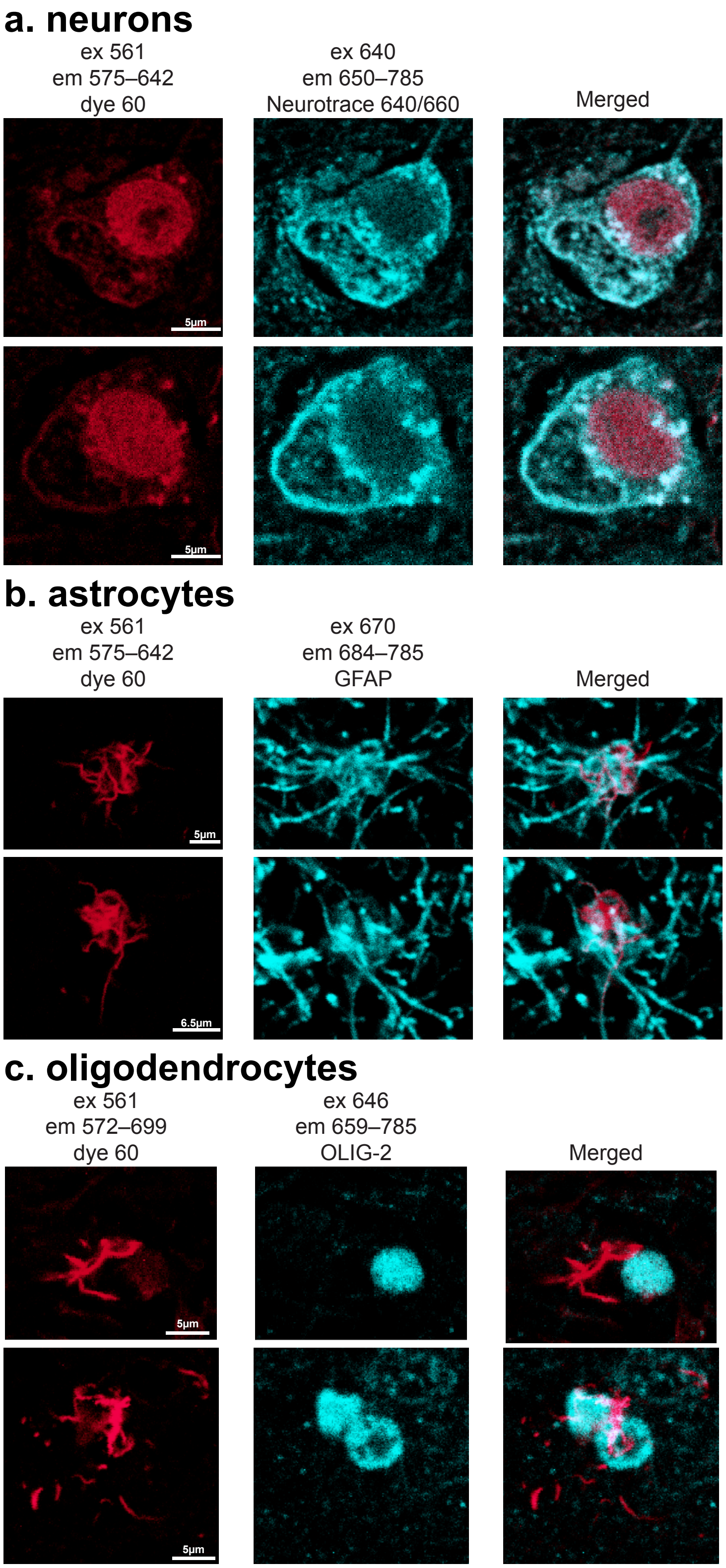

Fig. S15. Pick's disease brain donor samples are co-stained with dye 60 and either (a) NeuroTrace640/660 for neuron, (b) GFAP for astrocytes, or (c) OLIG-2 for oligodendrocytes. Dye 60 excites tau tangles of PiD neurons, astrocytes, and oligodendrocytes at 561 nm. Neurotrace640/660 stained neurons excited at 640 nm, GFAP antibody-stained astrocytes excited at 670 nm, and OLIG-2-stained oligodendrocytes at 646 nm. The merged micrographs show good overlap suggesting tau deposit morphology is linked to each cell type. For NeuroTrace640/650, dye 60 and NeuroTrace640/660 were mixed and applied to sample to stain tau tangles and neurons. For GFAP and OLIG-2 antibody staining, antigens were first retrieved by autoclaving slides with 0.01 M Citrate buffer and blocked with 10% goat serum. After incubating slides with primary antibody in 10% goat serum, secondary antibody labelled with Alexa fluor 647 was applied.

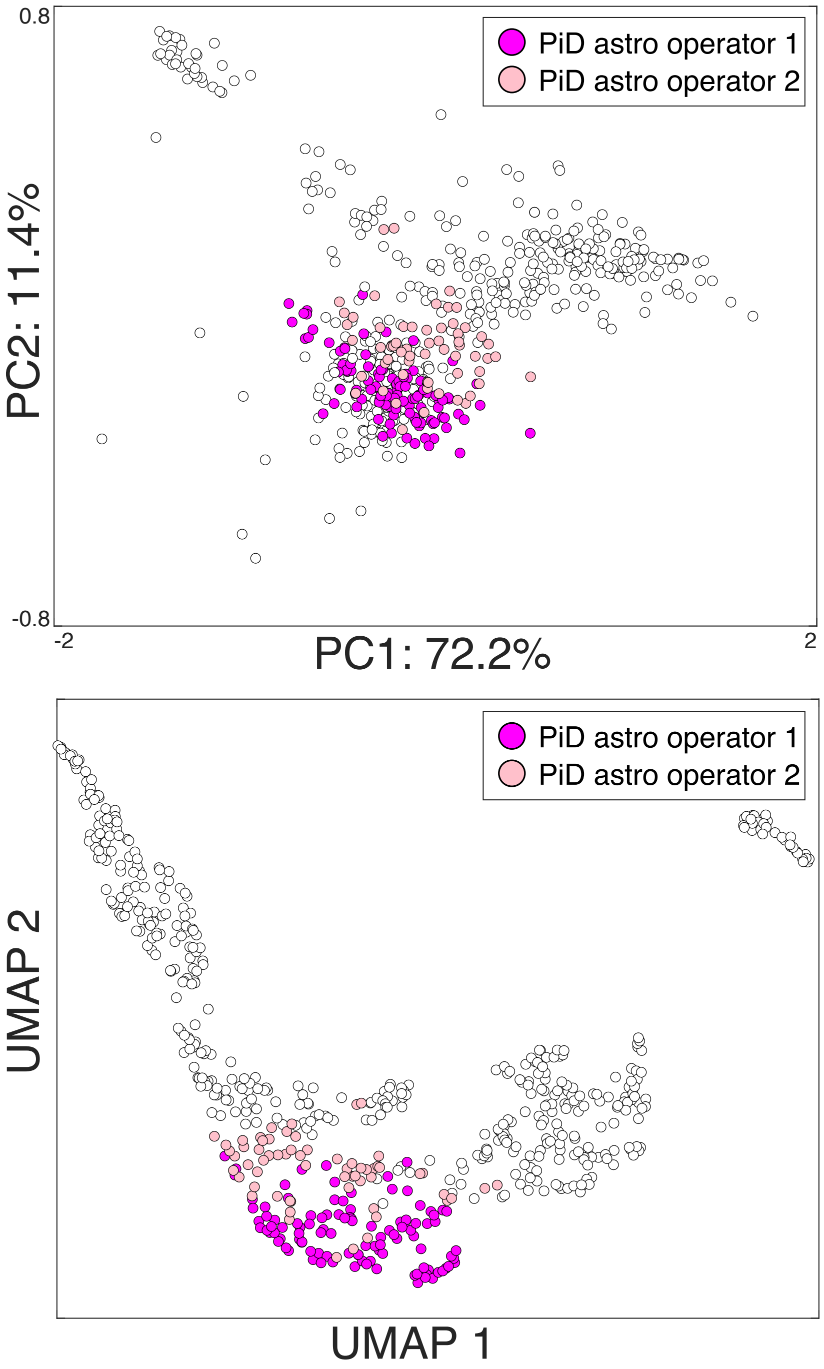

Fig. S16. EMBER reproducibility study of PiD tau in astrocytes. Two operators (pink and purple) performed two independent EMBER data collection on three PiD brain donor samples stained with dye 60. The collected data was appended onto the tau deposit dataset as shown in main Figure 6 and PCA and UMAP analysis were performed. The plot shows good overlap between two independent data collections suggesting good reproducibility. To aid visibility of the overlap, the particles pertaining to other EMBER datasets were colored white.

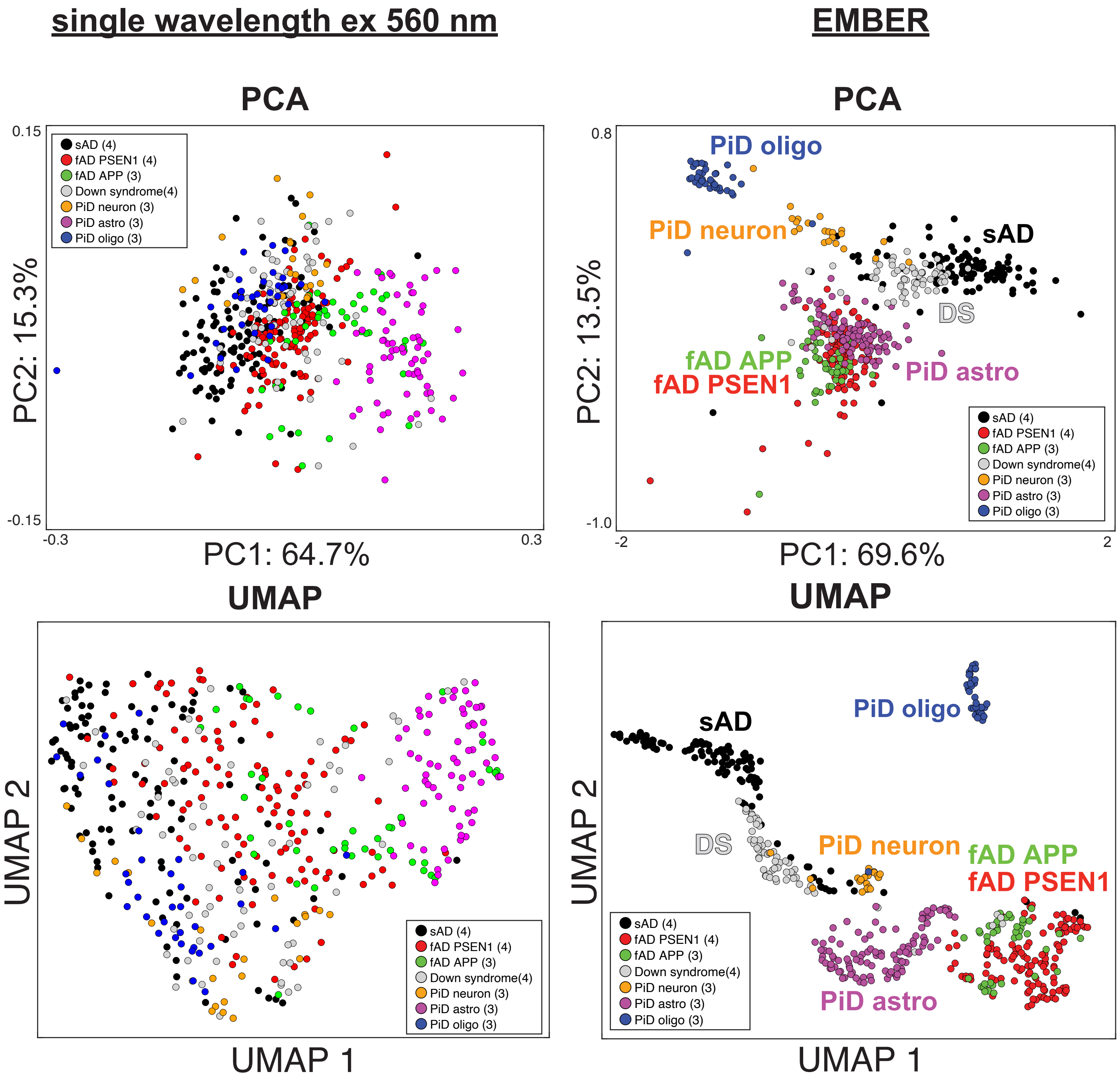

Fig. S17. EMBER vs single-wavelength excitation discrimination power comparison for tau deposits. λ_{max} single-wavelength excitation data of 560 nm excitation was pulled, and PCA and UMAP analysis were performed (left). For single-wavelength excitation, the separation between clusters is worse than that of EMBER (right) representing the outperformance of EMBER in maximizing photophysical property of dyes bound to tau deposits and to discriminate conformational strains of fibrils. The number of donor samples in each cohort is within the parenthesis.

Supplementary Table 1. Dye structures and EMBER data for *in vitro* fibrils.

#	Chemical structure	Name	Discrimination score (%)			Aβ40		Aβ42		ASynF		ASynR		0N3R		0N4R	
			PCA	UMAP	NN	ex	em	ex	em	ex	em	ex	em	ex	em	ex	em
1	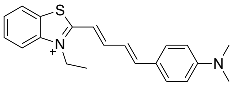 CC[N+]1=C(/C=C/C=C/C2=CC=C(N(C)C)C=C2)SC3=CC=CC=C31	PBB5	n.d.	n.d.	n.d.												
2	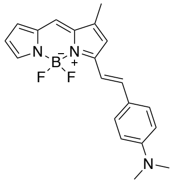 CC1=CC(/C=C/C2=CC=C(N(C)C)C=C2)=[N+](C1=C3)[B-](F)(F)N4C3=CC=C4	BAP-1	56	67	87	590	670	610	670	610	670	610	670	610	670	590	670
3	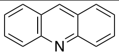 C1(N=C(C=CC=C2)C2=C3)=C3C=CC=C1	Acridine	44	39	60	470	530	470	530	470	530	470	510	470	510	470	530
4	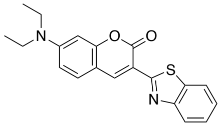 O=C1OC(C=C(N(CC)CC)C=C2)=C2C=C1C3=NC(C=CC=C4)=C4S3	Coumarin 6	40	41	71	470	510	470	510	470	510	470	510	470	510	470	510
5	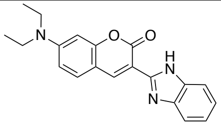 O=C1OC(C=C(N(CC)CC)C=C2)=C2C=C1C3=NC(C=CC=C4)=C4N3	Coumarin 7	67	69	86	470	510	470	510	470	490	470	490	470	510	470	490
6	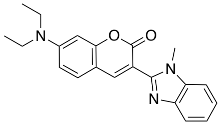 O=C1OC(C=C(N(CC)CC)C=C2)=C2C=C1C3=NC(C=CC=C4)=C4N3C	Coumarin 30	59	60	81	470	510	470	510	470	510	470	510	470	510	470	510
7	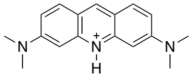 [H][N+]1=C(C=C(N(C)C)C=C2)C2=CC3=C1C=C(N(C)C)C=C3	Acridine Orange	59	50	76	470	510	470	530	470	510	470	530	470	510	470	530

8	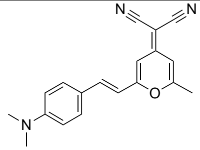 CC1=C/C(C=C(O1)/C=C/C2=CC=C(N(C)C)C=C2)=C(C#N)\C#N	DCM6490	65	74	86	490	610	490	590	490	610	510	610	490	590	490	610
9	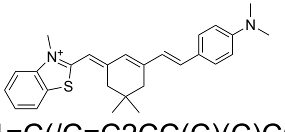 C[N+]1=C(/C=C2CC(C)(C)CC(/C=C/C3=CC=C(C=C3)N(C)C)=C\2)SC4=C1C=CC=C4	styryl9M	45	55	74	550	590	550	590	550	590	550	590	550	590	550	590
10	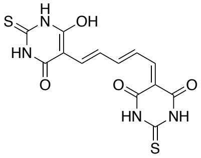 S=C1NC(C(/C=C/C=C/C=C2C(NC(NC\2=O)=S)=O)=C(O)N1)=O	thk265 (r277002)	n.d.	n.d.	n.d.												
11	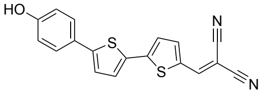 N#C/C(C#N)=C/C1=CC=C(S1)C2=CC=C(S2)C3=CC=C(O)C=C3	niad4	74	87	87	470	590	470	590	470	530	405	530	470	610	470	570
12	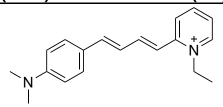 CN(C)C1=CC=C(C=C1)/C=C/C=C/C2=CC=[N+](CC)C=C2	lds698	82	93	92	550	670	510	650	510	670	550	670	550	650	550	670
13	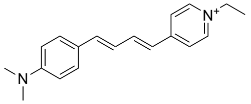 C[N+]1=CC=C(/C=C/C=C/C2=CC=C(N(C)C)C=C2)C=C1	lds722	76	92	95	550	670	550	670	550	670	550	670	550	670	550	670
14	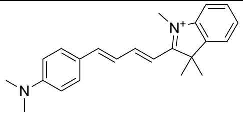 C[N+]1=C(/C=C/C=C/C2=CC=C(N(C)C)C=C2)C(C)(C)C3=C1C=CC=C3	lds730	53	51	71	650	690	650	690	650	690	650	690	650	690	650	690

15	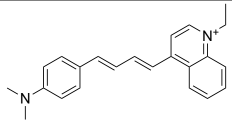 CC[N+]1=C2C=CC=CC2=C(/C=C/C=C/C3=CC=C(N(C)C)C=C3)C=C1	lds798	43	44	54	590	701	590	701	590	701	590	701	650	690	570	690
16	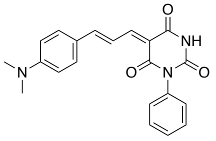 CN(C)C(C=C1)=CC=C1/C=C/C=C2C(N(C3=CC=CC=C3)C(NC\2=O)=O)=O		82	86	90	570	610	550	610	570	610	570	610	570	610	550	610
17	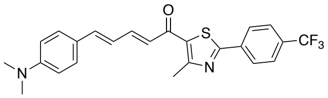 CN(C)C(C=C1)=CC=C1/C=C/C=C/C(C2=C(C)N=C(C3=CC=C(C(F)(F)F)C=C3)S2)=O		49	63	79	470	610	470	590	470	610	470	610	470	610	470	610
18	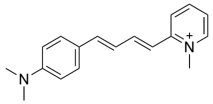 CN(C)C(C=C1)=CC=C1/C=C/C=C/C2=[N+](C)C=CC=C2		83	95	92	550	670	510	650	510	670	550	670	550	670	550	670
19	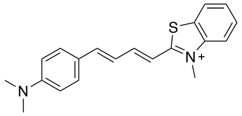 CN(C)C(C=C1)=CC=C1/C=C/C=C/C2=[N+](C)C(C=CC=C3)=C3S2		56	51	63	590	690	630	690	630	690	650	701	650	701	630	690
20	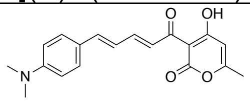 CN(C)C(C=C1)=CC=C1/C=C/C=C/C(C2=C(O)C=C(C)OC2=O)=O		67	68	79	470	610	470	590	470	610	470	610	470	610	470	610

21	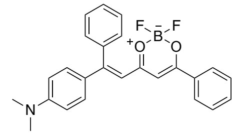 F[B-]1(F)OC(C2=CC=CC=C2)=CC(/C=C(C3=CC=CC=C3)/C4=CC=C(N(C)C)C=C4)=[O+]1		n.d.	n.d.	n.d.												
22	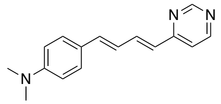 CN(C)C(C=C1)=CC=C1/C=C/C=C/C2=CC=NC=N2		71	93	96	470	590	470	590	470	610	470	590	470	590	470	590
23	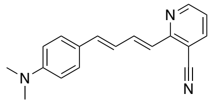 CN(C)C(C=C1)=CC=C1/C=C/C=C/C2=C(C#N)C=CC=N2		61	59	81	470	570	470	550	470	570	470	570	470	570	470	570
24	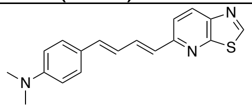 CN(C)C(C=C1)=CC=C1/C=C/C=C/C2=NC(SC=N3)=C3C=C2		69	77	90	470	570	470	570	470	570	470	550	470	570	470	570
25	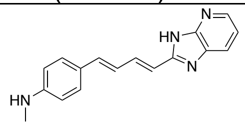 CNC(C=C1)=CC=C1/C=C/C=C/C2=NC(C=CC=N3)=C3N2		70	79	97	470	590	470	570	470	590	470	570	470	570	470	570
26	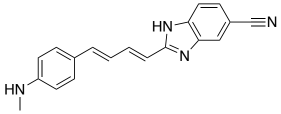 CNC(C=C1)=CC=C1/C=C/C=C/C2=NC(C=C(C#N)C=C3)=C3N2		65	69	91	470	570	470	570	470	570	470	570	470	570	470	570
27	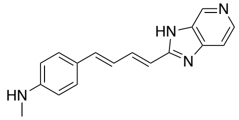 CNC(C=C1)=CC=C1/C=C/C=C/C2=NC(C=CN=C3)=C3N2		94	97	98	470	610	470	610	470	590	470	610	470	610	470	610

28	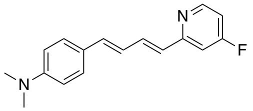 CN(C)C(C=C1)=CC=C1/C=C/C=C/C2=CC(F)=CC=N2		90	94	92	405	550	405	530	405	530	405	550	590	670	470	570
29	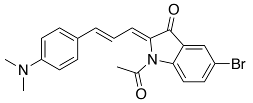 CC(N1C2=CC=C(Br)C=C2C(/C1=C/C=C/C3=CC=C(N(C)C)C=C3)=O)=O		56	66	79	470	570	470	570	470	550	470	570	470	570	470	570
30	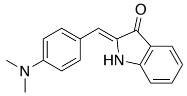 CN(C1=CC=C(/C=C2C(C3=C(N/2)C=CC=C3)=O)C=C1)C	Disperse Yellow 39	47	71	88	510	570	510	570	510	570	510	590	470	590	470	570
31	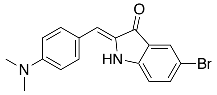 CN(C1=CC=C(/C=C2C(C3=CC(Br)=CC=C3N/2)=O)C=C1)C		48	55	79	530	590	510	590	530	590	510	590	550	610	510	570
32	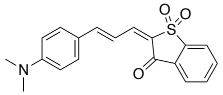 O=S(C1=CC=CC=C1C2=O)(/C2=C/C=C/C3=CC=C(N(C)C)C=C3)=O		72	77	87	550	630	550	610	550	630	550	630	550	610	550	610
33	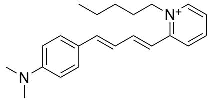 CCCCC[N+]1=C(/C=C/C=C/C2=CC=C(N(C)C)C=C2)C=CC=C1		75	86	90	550	670	510	650	510	670	550	670	550	670	550	670
34	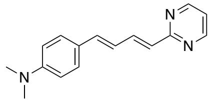 CN(C)C(C=C1)=CC=C1/C=C/C=C/C2=NC=CC=N2		65	75	77	405	550	405	530	405	550	405	550	405	550	405	550

35	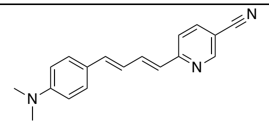 CN(C)C(C=C1)=CC=C1/C=C/C=C/ C2=NC=C(C#N)C=C2		88	93	95	470	610	470	590	470	570	470	610	470	610	470	610
36	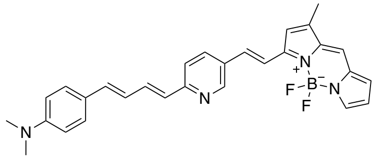 CC1=CC(/C=C/C(C=C2)=CN=C2/ C=C/C=C/C(C=C3)=CC=C3N(C)C) =[N+]4C1=CC5=CC=CN5[B-]4(F)F		75	94	96	470	610	470	590	470	590	570	590	470	610	550	570
37	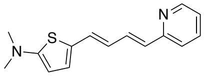 CN(C)C1=CC=C(S1)/C=C/C=C/C2 =CC=CC=N2		60	66	81	590	610	590	610	630	650	590	610	630	650	590	610
38	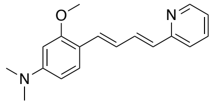 CN(C1=CC(OC)=C(/C=C/C=C/C2= CC=CC=N2)C=C1)C		74	84	94	405	530	405	530	405	530	405	530	405	650	405	630
39	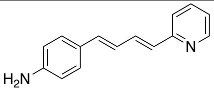 NC1=CC=C(C=C1)/C=C/C=C/C2= NC=CC=C2		92	97	97	405	510	405	490	405	490	405	490	405	610	470	610
40	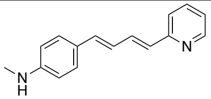 CNC(C=C1)=CC=C1/C=C/C=C/C2 =CC=CC=N2		80	95	98	405	530	405	510	405	510	405	530	405	630	405	630
41	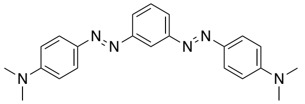 CN(C)C(C=C1)=CC=C1/N=N/C2= CC(/N=N/C3=CC=C(N(C)C)C=C3) =CC=C2		n.d.	n.d.	n.d.												

42	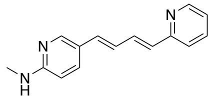 CNC1=NC=C(C=C1)/C=C/C=C/C2=NC=CC=C2		72	89	94	470	530	470	610	470	530	470	530	470	630	470	630
43	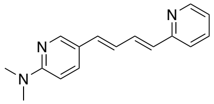 CN(C)C1=NC=C(C=C1)/C=C/C=C/C2=NC=CC=C2		78	94	97	405	510	405	490	405	610	405	510	405	610	405	610
44	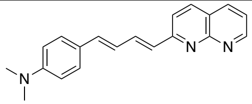 CN(C)C(C=C1)=CC=C1/C=C/C=C/C2=NC(N=CC=C3)=C3C=C2		68	70	88	470	630	470	610	470	630	470	630	470	630	470	610
45	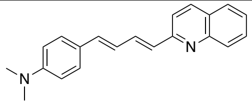 CN(C)C(C=C1)=CC=C1/C=C/C=C/C2=NC(C=CC=C3)=C3C=C2		88	92	91	470	570	405	530	405	510	405	550	470	670	405	530
46	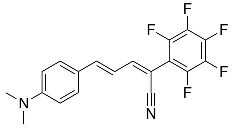 CN(C)C(C=C1)=CC=C1/C=C/C=C(C#N)/C2=C(F)C(F)=C(F)C(F)=C2F		n.d.	n.d.	n.d.												
47	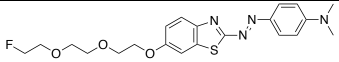 CN(C)C1=CC=C(C=C1)/N=N/C2=NC3=C(C=C(OCCOCCOCCF)C=C3)S2		n.d.	n.d.	n.d.												
48	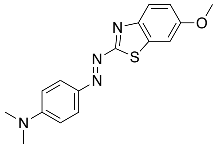 CN(C1=CC=C(/N=N/C2=NC3=C(S2)C=C(OC)C=C3)C=C1)C		n.d.	n.d.	n.d.												

49	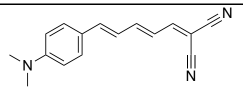 CN(C1=CC=C(/C=C/C=C/C=C(C#N)/C#N)C=C1)C	DCDAPH	58	91	95	550	670	550	650	470	630	550	670	590	670	550	650
50	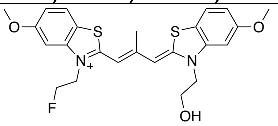 FCC[N+]1=C(SC2=C1C=C(OC)C=C2)/C=C(C)/C=C3N(CCO)C(C=C(OC)C=C4)=C4S/3		75	74	92	570	590	570	590	570	590	570	590	570	590	570	590
51	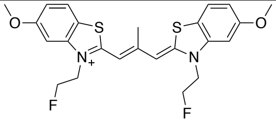 FCC[N+]1=C(SC2=C1C=C(OC)C=C2)/C=C(C)/C=C3N(CCF)C(C=C(OC)C=C4)=C4S/3		n.d.	n.d.	n.d.												
52	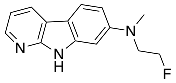 CN(CCF)C(C=C1)=CC2=C1C3=C(N2)N=CC=C3		n.d.	n.d.	n.d.												
53	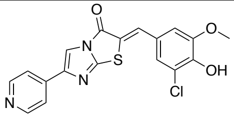 O=C(N1C(S/2)=NC(C3=CC=NC=C3)=C1)C2=C\C4=CC(Cl)=C(O)C(OC)=C4		53	55	61	510	550	510	570	510	570	510	570	510	570	490	550
54	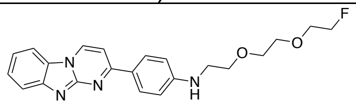 FCCOCCOCCNC(C=C1)=CC=C1C2=NC3=NC4=CC=CC=C4N3C=C2		42	39	70	405	530	405	530	405	530	405	530	405	530	405	530

55	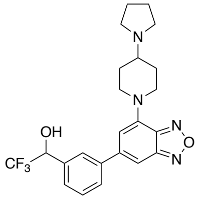 OC(C(F)(F)F)C1=CC=CC(C2=CC3=NON=C3C(N4CCC(N5CCCC5)CC4)=C2)=C1		34	32	47	470	590	470	570	470	590	470	570	470	590	470	590
56	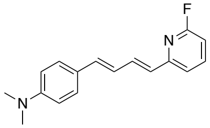 CN(C)C(C=C1)=CC=C1/C=C/C=C/C2=CC=CC(F)=N2		73	71	78	405	530	405	530	405	510	405	530	470	550	405	530
57	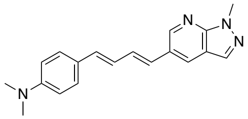 CN(C)C(C=C1)=CC=C1/C=C/C=C/C2=CC(C=NN3C)=C3N=C2		59	65	69	405	510	405	490	405	490	405	530	405	510	405	510
58	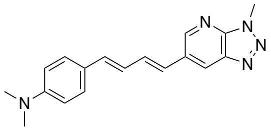 CN(C)C(C=C1)=CC=C1/C=C/C=C/C2=CC(N=NN3C)=C3N=C2		53	58	72	405	510	405	510	470	550	405	530	470	550	405	530
59	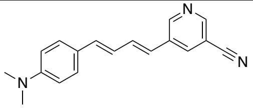 CN(C)C(C=C1)=CC=C1/C=C/C=C/C2=CC(C#N)=CN=C2		84	75	81	470	530	470	530	470	530	470	530	470	550	470	530
60	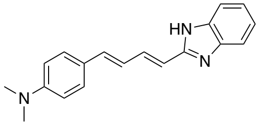 CN(C)C(C=C1)=CC=C1/C=C/C=C/C2=NC(C=CC=C3)=C3N2		80	86	93	405	590	405	590	405	550	405	570	470	610	405	590

61	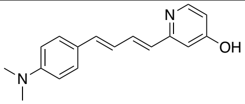 CN(C)C1=CC=C(C=C1)/C=C/C=C/C2=NC=CC(O)=C2		64	78	88	470	590	470	590	470	570	470	570	470	590	470	570
62	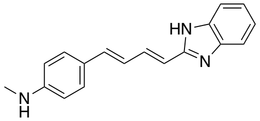 CNC1=CC=C(/C=C/C=C/C2=NC(C=CC=C3)=C3[N]2)C=C1	bf-188	79	82	93	405	590	405	590	405	530	405	570	470	590	405	590
63	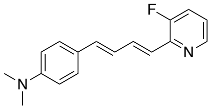 CN(C)C1=CC=C(C=C1)/C=C/C=C/C2=NC=CC=C2F		68	70	82	405	530	405	510	405	510	405	530	405	530	405	530
64	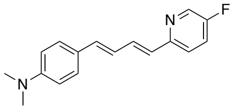 CN(C)C1=CC=C(C=C1)/C=C/C=C/C2=CC=C(F)C=N2		83	84	84	405	510	405	490	405	490	405	510	470	510	470	510
65	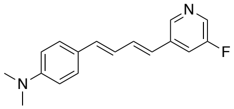 CN(C)C(C=C1)=CC=C1/C=C/C=C/C2=CC(F)=CN=C2		65	68	77	405	510	405	510	405	510	405	530	405	530	405	510
66	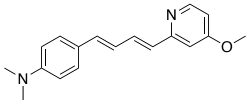 CN(C)C1=CC=C(C=C1)/C=C/C=C/C2=NC=CC(OC)=C2		54	80	94	405	610	405	610	405	610	405	610	405	610	405	610
67	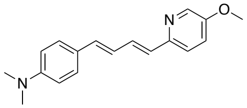 CN(C)C1=CC=C(C=C1)/C=C/C=C/C2=CC=C(OC)C=N2		82	91	95	405	510	405	490	405	490	405	510	405	490	405	490

68	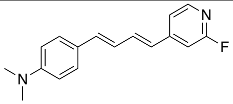 CN(C1=CC=C(/C=C/C=C/C2=CC(F)=NC=C2)C=C1)C		54	53	69	405	550	405	530	405	530	405	530	405	530	405	550
69	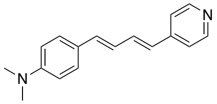 CN(C)C(C=C1)=CC=C1/C=C/C=C/C2=CC=NC=C2		73	75	80	405	530	405	530	405	510	405	530	405	570	405	530
70	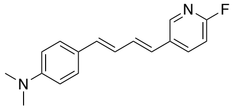 CN(C1=CC=C(/C=C/C=C/C2=CC=C(F)N=C2)C=C1)C		56	56	72	470	510	405	490	405	490	470	510	405	490	405	490
71	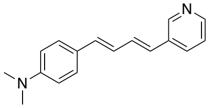 CN(C1=CC=C(/C=C/C=C/C2=CC=CN=C2)C=C1)C		61	58	70	470	510	470	490	470	490	405	510	405	510	470	510
72	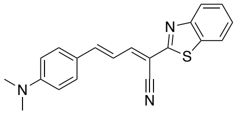 CN(C1=CC=C(/C=C/C=C(C#N)/C2=NC3=C(S2)C=CC=C3)C=C1)C		69	85	92	570	610	550	610	550	610	510	610	550	610	570	610
73	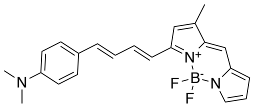 CC1=CC(/C=C/C=C/C2=CC=C(N(C)C)C=C2)=[N+]3C1=CC4=CC=CN4[B-]3(F)F		n.d.	n.d.	n.d.												
74	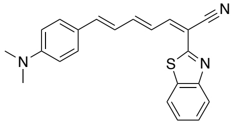 CN(C1=CC=C(/C=C/C=C/C=C(C2=NC3=C(S2)C=CC=C3)/C#N)C=C1)C		79	83	84	550	701	550	670	470	530	550	690	470	610	550	630

75	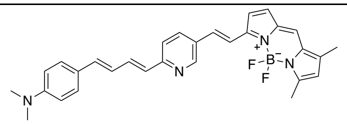 F[B-]1(F)[N+](C(C=C2)=CC3=C(C)C=C(C)N13)=C2/C=C/C(C=C4)=CN=C4/C=C/C=C/C(C=C5)=CC=C5N(C)C		50	50	60	470	590	470	590	470	570	470	590	470	590	470	590
76	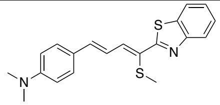 CN(C)C1=CC=C(C=C1)/C=C/C=C(SC)/C2=NC3=C(C=CC=C3)S2		64	60	70	490	610	490	610	490	610	490	610	510	630	490	610
77	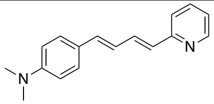 CN(C)C(C=C1)=CC=C1/C=C/C=C/C2=NC=CC=C2		74	92	96	405	530	405	510	405	510	405	530	405	650	405	510
78	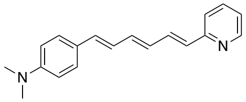 CN(C)C(C=C1)=CC=C1/C=C/C=C/C=C/C2=NC=CC=C2		63	77	85	405	570	405	550	405	530	405	570	405	550	405	550
79	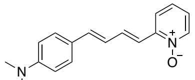 CN(C)C1=CC=C(C=C1)/C=C/C=C/C2=CC=CC=[N+]2[O-]		65	64	72	470	610	470	590	470	590	470	590	470	590	470	590
80	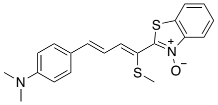 CN(C)C1=CC=C(C=C1)/C=C/C=C/C2=[N+]([O-])C3=C(C=CC=C3)S2		66	63	60	470	570	470	550	405	530	470	570	470	590	470	570
81	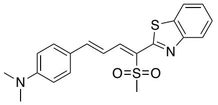 CN(C)C1=CC=C(C=C1)/C=C/C=C(S(=O)(C)=O)/C2=NC3=C(C=CC=C3)S2		62	59	70	510	610	510	610	510	610	510	610	550	630	550	610

82	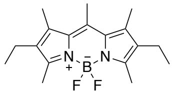 F[B-]1(F)[N+]2=C(C)C(CC)=C(C)C 2=C(C)C3=C(C)C(CC)=C(C)N13	Pyrrometh ene 605	53	54	75	510	550	510	570	510	550	510	550	510	550	510	530
83	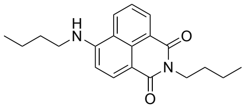 O=C(N(CCCC)C1=O)C2=CC=C(N CCCC)C3=CC=CC1=C32	Fluorol555	35	40	62	470	530	470	530	470	530	470	530	470	530	470	530
84	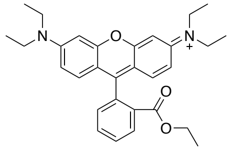 CC/[N+](CC)=C1C=CC2=C(C3=C C=CC=C3C(OCC)=O)C4=CC=C(N (CC)CC)C=C4OC2=C/1	Rhodamin e3B Perchlorat e	n.d.	n.d.	n.d.												
85	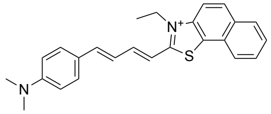 CC[N+]1=C(SC2=C1C=CC3=C2C =CC=C3)/C=C/C=C/C4=CC=C(N(C)C)C=C4	LDS750	54	71	77	650	701	590	690	590	690	650	701	650	701	590	690
86	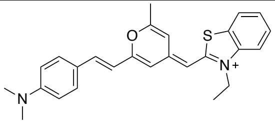 CN(C)C(C=C1)=CC=C1/C=C/C2= C/C(C=C(C)O2)=C/C3=[N+](CC)C(C=CC=C4)=C4S3	LDS759	64	63	65	590	670	590	670	550	670	590	670	570	670	570	670
87	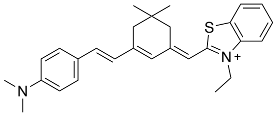 CN(C)C(C=C1)=CC=C1/C=C/C2= C/C(CC(C)(C)C2)=C/C3=[N+](CC) C(C=CC=C4)=C4S3	LDS821	n.d.	n.d.	n.d.												

88	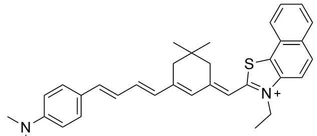 CN(C)C(C=C1)=CC=C1/C=C/C=C/C2=C/C(CC(C)(C)C2)=C/C3=[N+](CC)C(C=CC4=C5C=CC=C4)=C5S3	LDS925	n.d.	n.d.	n.d.												
89	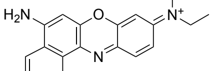 NC1=CC2=C(C3=C1C=CC=C3)N=C(C=C/4)C(O2)=CC4=[N+](CC)/CC	NileBlue690 Perchlorate	57	58	77	630	670	630	670	630	670	630	670	630	670	630	670
90	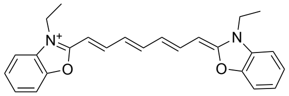 CCN1/C(OC2=C1C=CC=C2)=C/C=C/C=C/C=C/C(OC3=C4C=CC=C3)=[N+]4CC	DOTC Iodide	n.d.	n.d.	n.d.												
91	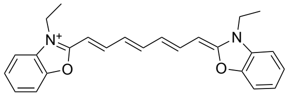 CCN1/C(OC2=C1C=CC=C2)=C/C=C/C=C/C=C/C(OC3=C4C=CC=C3)=[N+]4CC	DOTC Perchlorate	n.d.	n.d.	n.d.												
92	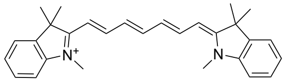 C[N+]1=C(/C=C/C=C/C=C/C=C2N(C)C(C=CC=C3)=C3C\2(C)C)C(C)(C)C4=C1C=CC=C4	HITC Perchlorate	77	89	91	650	670	650	670	550	570	650	670	650	670	650	670
93	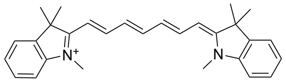 C[N+]1=C(/C=C/C=C/C=C/C=C2N(C)C(C=CC=C3)=C3C\2(C)C)C(C)(C)C4=C1C=CC=C4	HITC Iodide	n.d.	n.d.	n.d.												

94	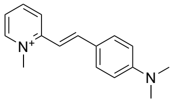 C[N+]1=C(C=CC=C1)/C=C/C2=CC=C(N(C)C)C=C2	DASPI	70	77	84	490	570	490	570	470	570	490	570	510	570	490	570
95	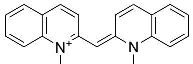 CC[N+]1=C(C=CC2=C1C=CC=C2)/C=C3N(CC)C(C=CC=C4)=C4C=C/3	PICI	70	70	81	490	630	530	590	530	570	530	570	510	530	490	610
96	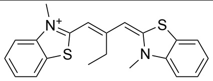 CCC(/C=C1SC2=CC=CC=C2N/1C)=C\C3=[N+](C)C4=CC=CC=C4S3	DMETCI	68	68	85	570	590	550	570	550	570	550	570	550	570	550	570
97	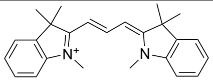 C[N+]1=C(/C=C/C=C2N(C)C(C=CC=C3)=C3C\2(C)C)C(C)(C)C4=C1C=CC=C4	HICI	n.d.	n.d.	n.d.												
98	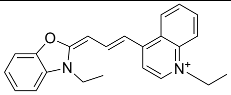 CCN1/C(OC2=CC=CC=C12)=C\C=C\C3=C4C=CC=CC4=[N+](CC)C=C3	DQOCI	n.d.	n.d.	n.d.												
99	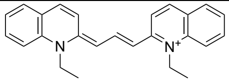 CCN1C2=C(C=C/C1=C\C=C\C(C=CC3=C4C=CC=C3)=[N+]4CC)C=CC=C2	Pinacyanol	n.d.	n.d.	n.d.												
100	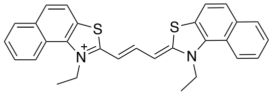 CC[N+]1=C(/C=C/C=C2N(CC)C(C(C=CC=C3)=C3C=C4)=C4S/2)SC5=C1C6=CC=CC=C6C=C5	DDBCI	n.d.	n.d.	n.d.												

101	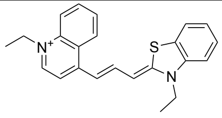 CC[N+]1=CC=C(/C=C/C=C2N(CC)C(C=CC=C3)=C3S/2)C4=C1C=CC=C4	DQTCI	90	97	97	570	650	570	650	570	590	570	590	570	590	570	650
102	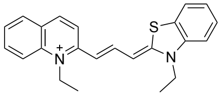 CCN(C(C=CC=C1)=C1S/2)C2=C\C=C\C3=[N+](CC)C4=C(C=CC=C4)C=C3	DQTCI(')	85	84	89	590	610	590	610	590	610	590	610	590	610	590	610
103	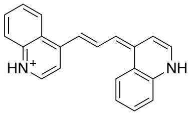 C1(/C=C/C=C2C=CNC3=C/2C=CC=C3)=C4C=CC=CC4=[NH+]C=C1	Cryptocyanine	n.d.	n.d.	n.d.												
104	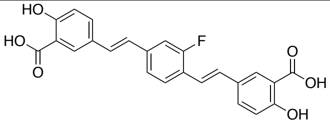 OC1=CC=C(C=C1C(O)=O)/C=C/C2=CC=C(C(F)=C2)/C=C/C3=CC(C(O)=O)=C(C=C3)O	HDITCP	61	55	63	590	610	590	610	590	610	590	610	490	550	590	610
105		T004	77	73	88	405	490	405	490	405	490	405	550	405	490	405	510
106	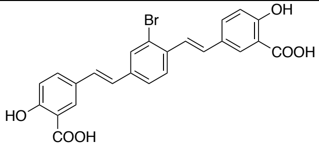 OC1=CC=C(/C=C/C2=CC=C(/C=C/C3=CC(C(O)=O)=C(O)C=C3)C(Br)=C2)C=C1C(O)=O	bsb	42	40	57	470	510	470	510	470	510	470	510	470	510	470	510
107	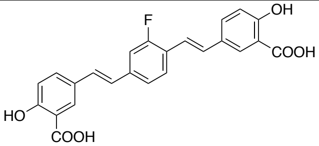 OC1=CC=C(/C=C/C2=CC=C(/C=C/C3=CC(C(O)=O)=C(O)C=C3)C(F)=C2)C=C1C(O)=O	fsb	47	45	67	470	510	470	510	470	510	470	510	405	510	470	510

108	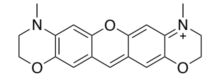 CN1C2=C(C=C(C=C3C(O4)=CC5=[N+](C)CCOC5=C3)C4=C2)OCC1	AOI987	n.d.	n.d.	n.d.												
109	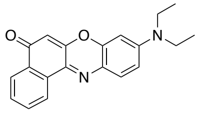 O=C1C=C2OC(C=C(N(CC)CC)C=C3)=C3N=C2C4=C1C=CC=C4	Nile red	54	52	67	570	630	550	610	570	630	570	630	590	630	570	630
110	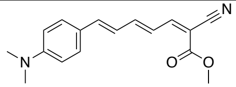 CN(C)C(C=C1)=CC=C1/C=C/C=C/C=C(C(OC)=O)/C#N	MCAAD-3	87	93	94	550	650	550	650	590	670	590	670	570	670	550	670
111	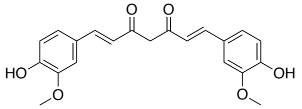 COC1=C(O)C=CC(/C=C/C(O)=C/C(/C=C/C2=CC=C(C)C(OC)=C2)=O)=C1	curcumin	74	86	84	610	670	590	670	610	670	630	670	405	530	650	670
112	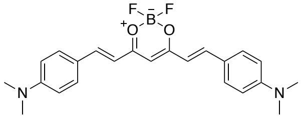 CN(C)C1=CC=C(/C=C/C2=[O+][B-](F)(F)OC(/C=C/C3=CC=C(N(C)C)C=C3)=C2)C=C1	CRANAD-2	80	83	88	610	670	550	670	550	670	550	670	550	650	510	670
113	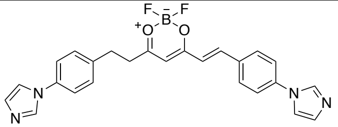 F[B-]1(F)OC(/C=C/C2=CC=C(N3C=CN=C3)C=C2)=CC(CCC4=CC=C(N5C=CN=C5)C=C4)=[O+]1	CRANAD-17	n.d.	n.d.	n.d.												
114	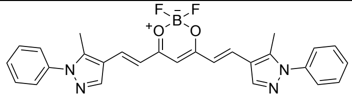 CC(N(N=C1)C2=CC=CC=C2)=C1/C=C/C3=CC(/C=C/C4=C(N(N=C4)C5=CC=CC=C5)C)=[O+][B-](F)(F)O3	CRANAD-28	n.d.	n.d.	n.d.												

115	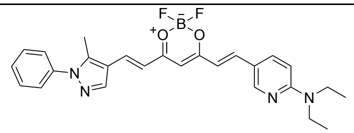 CC(N(N=C1)C2=CC=CC=C2)=C1/C=C/C3=[O+][B-](F)(F)OC(/C=C/C4=CN=C(N(CC)CC)C=C4)=C3	CRANAD-30	73	95	94	470	590	510	590	470	570	550	610	510	590	510	590
116	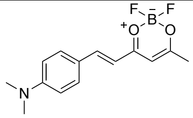 CC1=CC(/C=C/C2=CC=C(N(C)C)C=C2)=[O+][B-](F)(F)O1	CRANAD-54	74	80	93	470	530	470	530	470	510	470	530	470	530	470	530
117	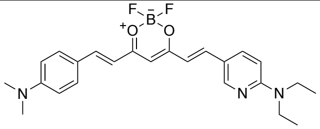 F[B-]1(F)OC(/C=C/C2=CN=C(N(CC)CC)C=C2)=CC(/C=C/C3=CC=C(N(C)C)C=C3)=[O+]1	CRANAD-58	66	68	64	550	610	550	610	550	610	470	530	510	530	470	550
118	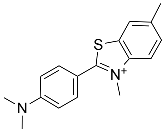 C[N+]1=C(C2=CC=C(N(C)C)C=C2)SC3=C1C=CC(C)=C3	Thioflavin T	56	58	70	470	490	470	490	405	490	470	490	470	490	470	490
119	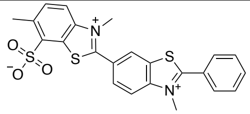 C[N+]1=C(C2=CC=CC=C2)SC3=C1C=CC(C4=[N+](C)C(C=CC(C)=C5S([O-])(=O)=O)=C5S4)=C3	Thioflavin S	47	49	70	405	530	405	530	405	550	405	550	405	530	405	550
120	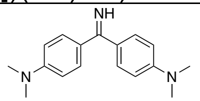 CN(C)C1=CC=C(C(C2=CC=C(N(C)C)C=C2)=N)C=C1	Auramine	42	44	57	470	530	470	530	470	530	470	530	470	510	470	530

121	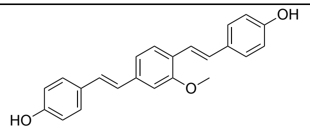 COC1=CC(/C=C/C2=CC=C(O)C=C2)=CC=C1/C=C/C3=CC=C(O)C=C3	Methoxy-X04	70	69	81	510	530	510	530	510	530	510	550	510	530	510	530
122	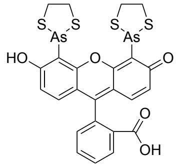 OC(C([As]1SCCS1)=C2O3)=CC=C2C(C4=CC=CC=C4C(O)=O)=C(C=C5)C3=C([As]6SCCS6)C5=O	FlAsH-EDT2	54	53	61	510	530	510	530	510	530	510	550	510	530	510	530
123	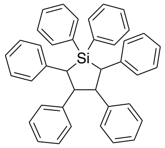 C1(C2=CC=CC=C2)C(C3=CC=CC=C3)[Si](C4=CC=CC=C4)(C5=CC=CC=C5)C(C6=CC=CC=C6)C1C7=CC=CC=C7	1,1,2,3,4,5-Hexaphenyl-1*H*-*silole*	n.d.	n.d.	n.d.												
124	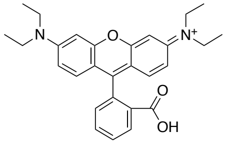 CC/[N+](CC)=C1C=CC2=C(C3=CC=CC=C3C(O)=O)C4=C(C=C(N(CC)CC)C=C4)OC2=C\1	Rhodamine B	n.d.	n.d.	n.d.												

125	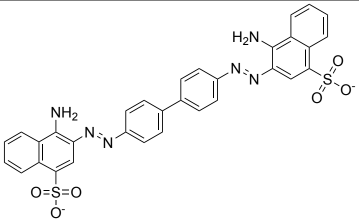 NC(C(C=CC=C1)=C1C(S([O-])(=O)=O)=C2)=C2/N=N/C3=CC=C(C4=CC=C(/N=N/C5=CC(S([O-])(=O)=O)=C(C=CC=C6)C6=C5N)C=C4)C=C3	Congo red	42	36	46	550	610	530	610	550	610	550	610	530	630	550	630
126	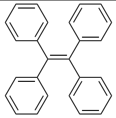 C1(/C(C2=CC=CC=C2)=C(C3=CC=CC=C3)/C4=CC=CC=C4)=CC=CC=C1	Tetraphenyl ethylene	n.d..	n.d.	n.d.												
127	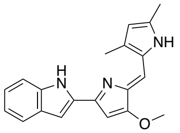 COC1=CC(C(N2)=CC3=C2C=CC=C3)=N/C1=C\C4=C(C)C=C(C)N4	Obatoclax (GX15-070)	76	79	82	550	590	550	590	550	570	490	570	550	590	550	590
128	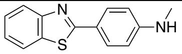 CNC(C=C1)=CC=C1C2=NC3=C(S2)C=CC=C3	BTA-1	n.d.	n.d.	n.d.												
129	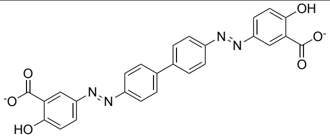 OC1=CC=C(/N=N/C2=CC=C(C3=CC=C(/N=N/C4=CC=C(O)C(C([O-])=O)=C4)C=C3)C=C2)C=C1C([O-])=O	Chrysamine G	n.d.	n.d.	n.d.												

130	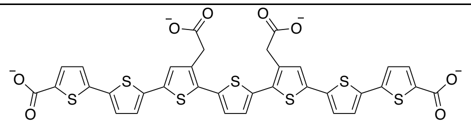 [O-]C(C(S1)=CC=C1C2=CC=C(C3=CC(CC([O-])=O)=C(C4=CC=C(C5=C(CC([O-])=O)C=C(C6=CC=C(C7=CC=C(C([O-])=O)S7)S6)S5)S4)S3)S2)=O	hFTAA	75	72	87	470	630	490	610	470	630	470	650	470	590	470	650
131	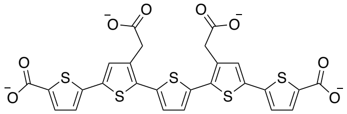 O=C([O-])CC1=C(C2=CC=C(C3=C(CC([O-])=O)C=C(C4=CC=C(C([O-])=O)S4)S3)S2)SC(C5=CC=C(C([O-])=O)S5)=C1	pFTAA	79	80	92	470	550	470	550	470	550	470	550	470	550	470	570
132	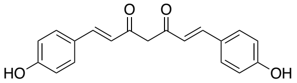 OC1=CC=C(/C=C/C(CC(/C=C/C2=CC=C(O)C=C2)=O)=O)C=C1	Bisdemethoxy curcumin	58	74	77	405	570	405	530	470	590	470	590	470	590	405	530
133	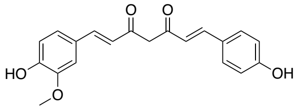 O=C(/C=C/C1=CC=C(O)C=C1)CC(/C=C/C2=CC=C(O)C(OC)=C2)=O	Dimethoxy curcumin	56	61	78	470	570	470	550	470	590	470	590	470	530	470	550
134	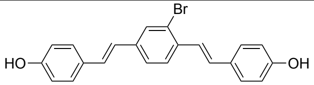 OC1=CC=C(/C=C/C2=CC=C(/C=C/C3=CC=C(O)C=C3)C(Br)=C2)C=C1	K114	62	55	52	470	610	470	530	470	510	470	550	470	590	470	530
135	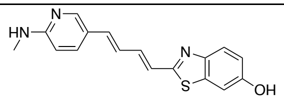 OC1=CC2=C(C=C1)N=C(/C=C/C=C/C3=CN=C(NC)C=C3)S2	PBB3	68	72	89	470	550	405	530	405	510	470	530	405	530	405	530

136	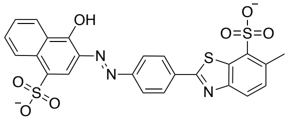 CC1=C(S([O-])(=O)=O)C2=C(C=C1)N=C(C3=CC=C(/N=N/C4=CC(S([O-])(=O)=O)=C5C(C=CC=C5)=C4O)C=C3)S2	Thiazin red	n.d.	n.d.	n.d.												
137	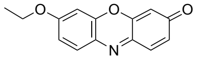 O=C1C=CC2=NC3=C(C=C(OCC)C=C3)OC2=C1	resorufin ethyl ether	n.d.	n.d.	n.d.												
138	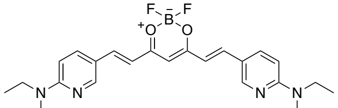 F[B-]1(F)OC(/C=C/C2=CN=C(N(CC)CC)C=C2)=CC(/C=C/C3=CC=C(N(CC)CC)N=C3)=[O+]1	CRANAD-3	62	71	88	470	550	470	550	470	530	470	550	470	550	470	550
139	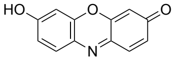 OC(C=C1)=CC2=C1N=C(C=C3)C(O2)=CC3=O	resorufin	n.d.	n.d.	n.d.												
140	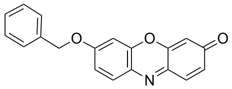 O=C1C=CC2=NC3=C(C=C(OCC4=CC=CC=C4)C=C3)OC2=C1	resorufin benzyl ether	n.d.	n.d.	n.d.												
141	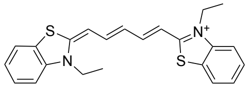 CCN1C(C=CC=C2)=C2S/C1=C/C=C/C=C/C(SC3=CC=CC=C43)=[N+]4CC	DTDCI	75	88	97	670	690	670	690	670	690	670	690	470	550	670	690
142	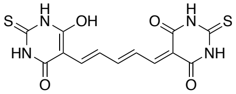 O=C(C(/C=C/C=C/C=C1C(NC(NC/1=O)=S)=O)=C(O)N2)NC2=S	Sigma R277002	n.d.	n.d.	n.d.												

143	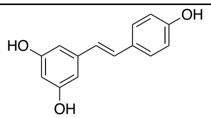 OC1=CC(O)=CC(/C=C/C2=CC=C(O)C=C2)=C1	Resveratrol	n.d.	n.d.	n.d.												
144	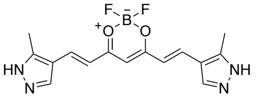 F[B-]1(F)OC(/C=C/C2=C(C)NN=C2)=CC(/C=C/C3=C(C)NN=C3)=[O+]1	CRANAD-44	52	52	78	405	510	405	510	405	510	405	510	670	690	405	510
145	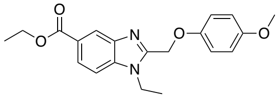 CCN1C2=CC=C(C(OCC)=O)C=C2N=C1COC3=CC=C(OC)C=C3	Amb14836	59	67	76	490	630	490	630	490	630	490	630	510	630	490	630

n.d. = not determined due to low fluorescence excitation from all six *in vitro* fibrils

Supplementary Table 2. Source of postmortem human brain tissue samples.

Cohort	Patient ID#	Sex	Age	NPDx (Clinical Dx)	Mutation	APOE genotype	CERAD score	Braak stage	Brain region	Source
sAD 1	2217 T3	M	63	AD		3/4	C3	VI	Temporal cortex	UCSF NDBB
sAD 2	2312.10 T2	M	59	AD		4/4	C3	VI	Temporal cortex	UCSF NDBB
sAD 3	P2376.11 R14	M	80	AD		3/3	C3	VI	Parietal cortex	UCSF NDBB
sAD 4	P2376.11 R15	M	80	AD		3/3	C3	VI	Parietal cortex	UCSF NDBB
fAD PSEN 1	MGH940	M	55	AD	L435F PSEN1	-	C3	V	Frontal cortex	MGH Harvard
fAD PSEN 2	BBN3246 DPM 99/06 T	F	57	AD	M139V PSEN1	-	C3	VI	Temporal cortex	University of Manchester (UK)
fAD PSEN 3	BBN13829 A029/98x30	M	42	AD	Δ4 PSEN1	3/3	C3	VI	Temporal cortex	King's College London (UK)
fAD PSEN 4	BBN13955 A141/93x34	M	54	AD	Δ4 PSEN1	3/3	C3	VI	Temporal cortex	King's College London (UK)
fAD APP 1	BBN9608 A051/97x30	F	62	AD	V717I APP (London)	3/4	C3	VI	Temporal cortex	King's College London (UK)
fAD APP 2	BBN13890 A211/94	M	61	AD	V717I APP (London)	3/3	C3	VI	Temporal cortex	King's College London (UK)
fAD APP 3	BBN13932 A0258/94	F	55	AD	V717I APP (London)	3/4	C3	VI	Temporal cortex	King's College London (UK)
DS 1	3-17	M	57	DS		3/3			Frontal cortex	UC Irvine
DS 2	7-05	M	54	DS		3/3			Frontal cortex	UC Irvine
DS 3	10-13	M	56	DS		4/4			Frontal cortex	UC Irvine
DS 4	21-14	M	50	DS		2/3			Frontal cortex	UC Irvine
PiD 1	2b2	M	62	PiD					Frontal cortex	Mayo
PiD 2	2b10	F	69	PiD					Frontal cortex	Mayo
PiD 3	2c4	F	82	PiD					Frontal cortex	Mayo
PiD 4	2c7	M	84	PiD					Frontal cortex	Mayo
PiD 5	2c9	F	63	PiD					Frontal cortex	Mayo
