# Supplementary material for: EMBER multidimensional spectral microscopy enables quantitative determination of disease- and cell-specific amyloid strains
Source: Proc Natl Acad Sci U S A. 2023 Mar 16;120(12):e2300769120. doi: 10.1073/pnas.2300769120 (PMC10041141; doi:10.1073/pnas.2300769120)

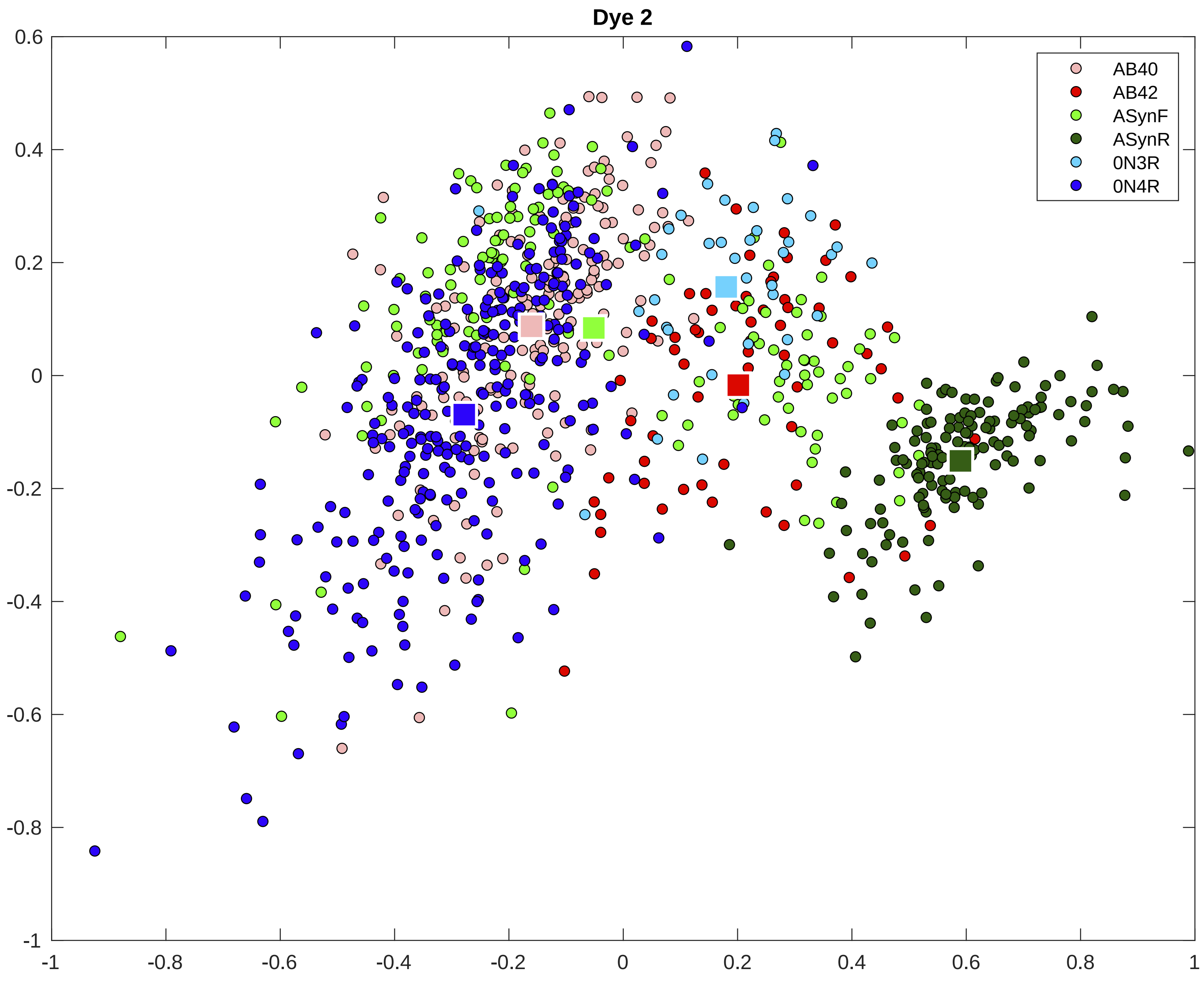
Dye 2
AB40
AB42
ASynF
ASynR
0N3R
0N4R
0.6
0.4
0.2
0
-0.2
-0.4
-0.6
-0.8
-1
-1
-0.8
-0.6
-0.4
-0.2
0
0.2
0.4
0.6
0.8
1

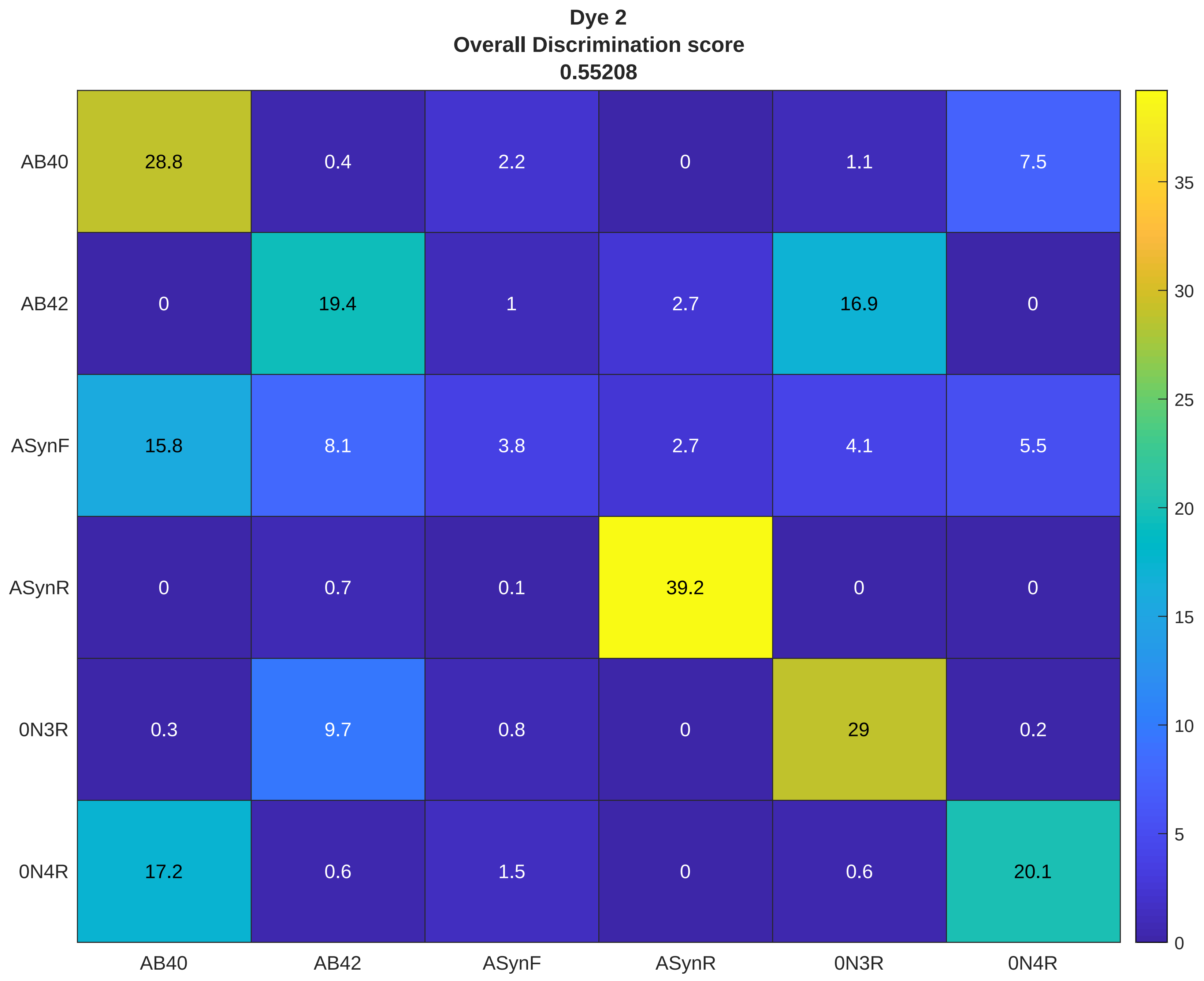
Dye 2
Overall Discrimination score
0.55208
AB40
AB42
ASynF
ASynR
0N3R
0N4R
28.8
0.4
2.2
0
1.1
7.5
0
19.4
1
2.7
16.9
0
15.8
8.1
3.8
2.7
4.1
5.5
0
0.7
0.1
39.2
0
0
0.3
9.7
0.8
0
29
0.2
17.2
0.6
1.5
0
0.6
20.1
AB40
AB42
ASynF
ASynR
0N3R
0N4R
35
30
25
20
15
10
5
0

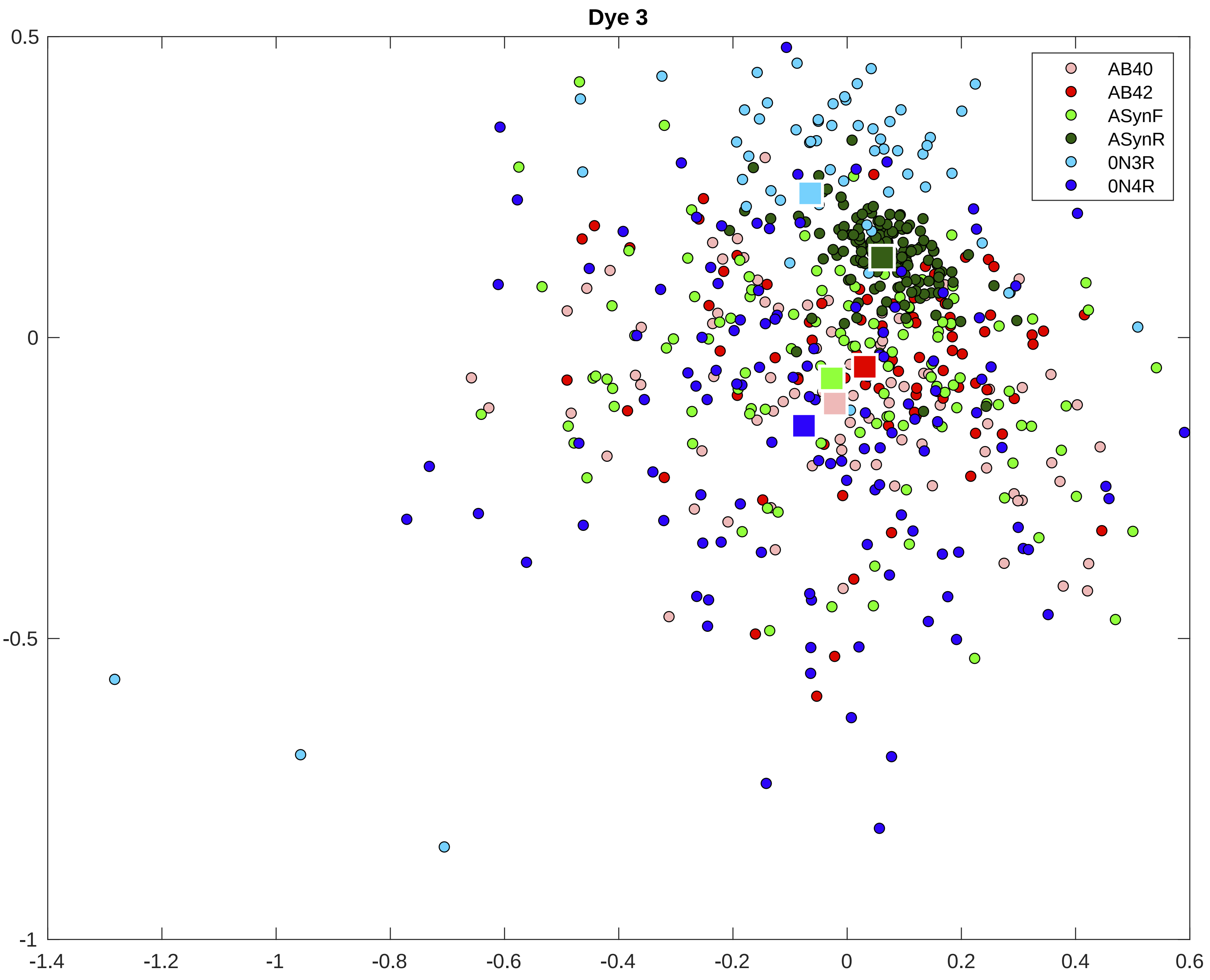

Dye 3
AB40
AB42
ASynF
ASynR
0N3R
0N4R
0.5
0
-0.5
-1
-1.4
-1.2
-1
-0.8
-0.6
-0.4
-0.2
0
0.2
0.4
0.6

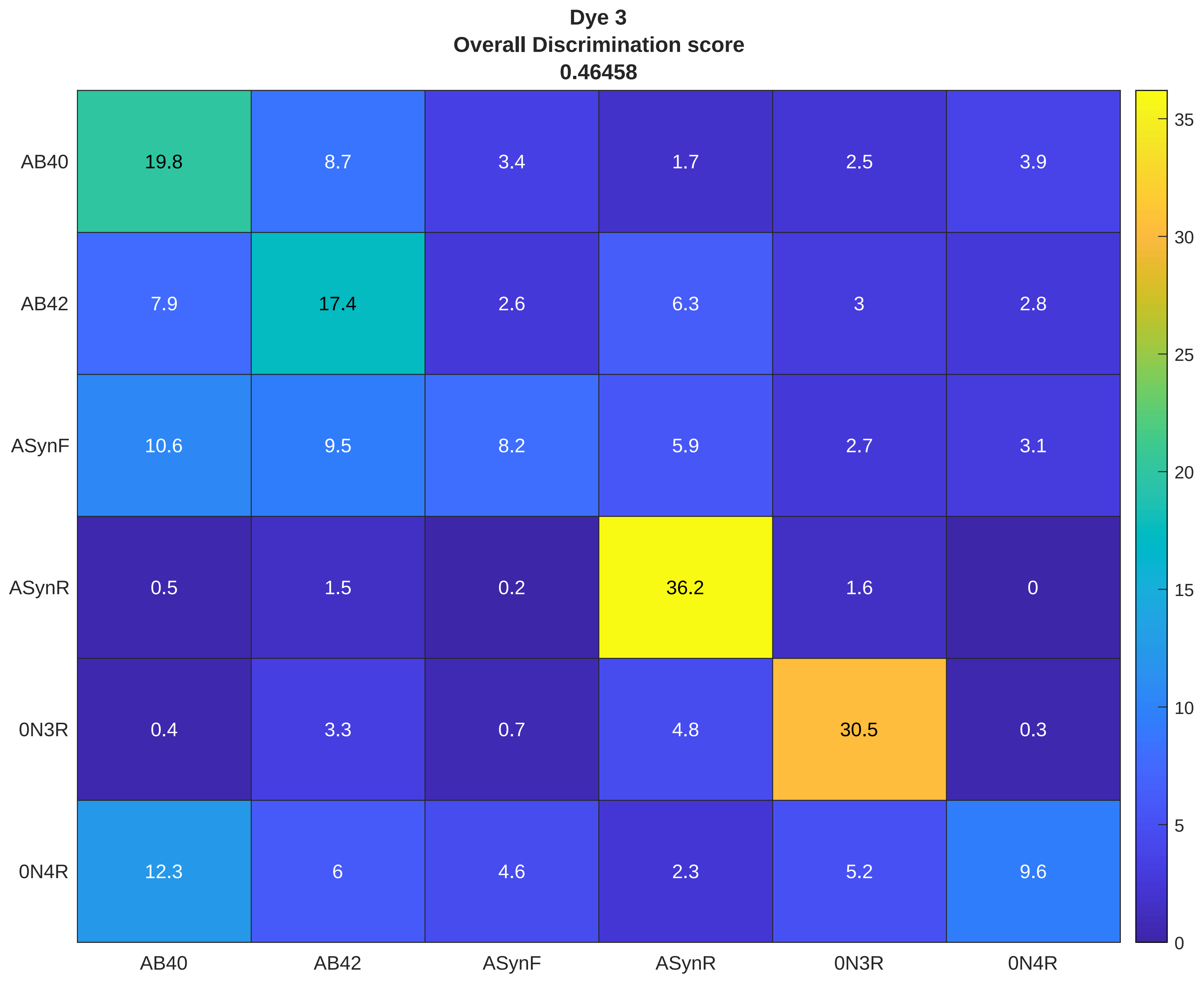
Dye 3
Overall Discrimination score
0.46458
AB40
AB42
ASynF
ASynR
0N3R
0N4R
19.8
8.7
3.4
1.7
2.5
3.9
7.9
17.4
2.6
6.3
3
2.8
10.6
9.5
8.2
5.9
2.7
3.1
0.5
1.5
0.2
36.2
1.6
0
0.4
3.3
0.7
4.8
30.5
0.3
12.3
6
4.6
2.3
5.2
9.6
AB40
AB42
ASynF
ASynR
0N3R
0N4R
35
30
25
20
15
10
5
0

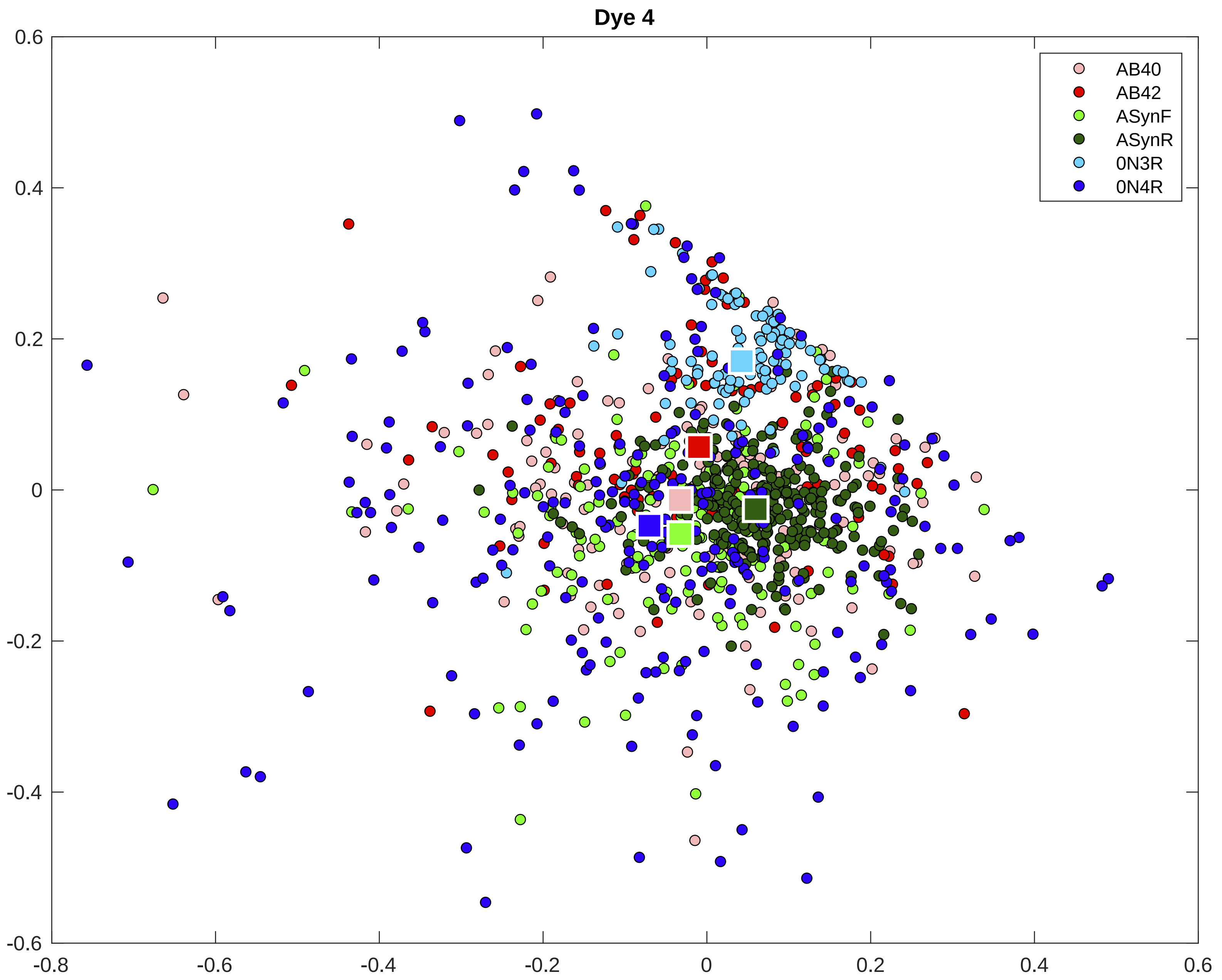

Dye 4
AB40
AB42
ASynF
ASynR
0N3R
0N4R
0.6
0.4
0.2
0
-0.2
-0.4
-0.6
-0.8
-0.6
-0.4
-0.2
0
0.2
0.4
0.6

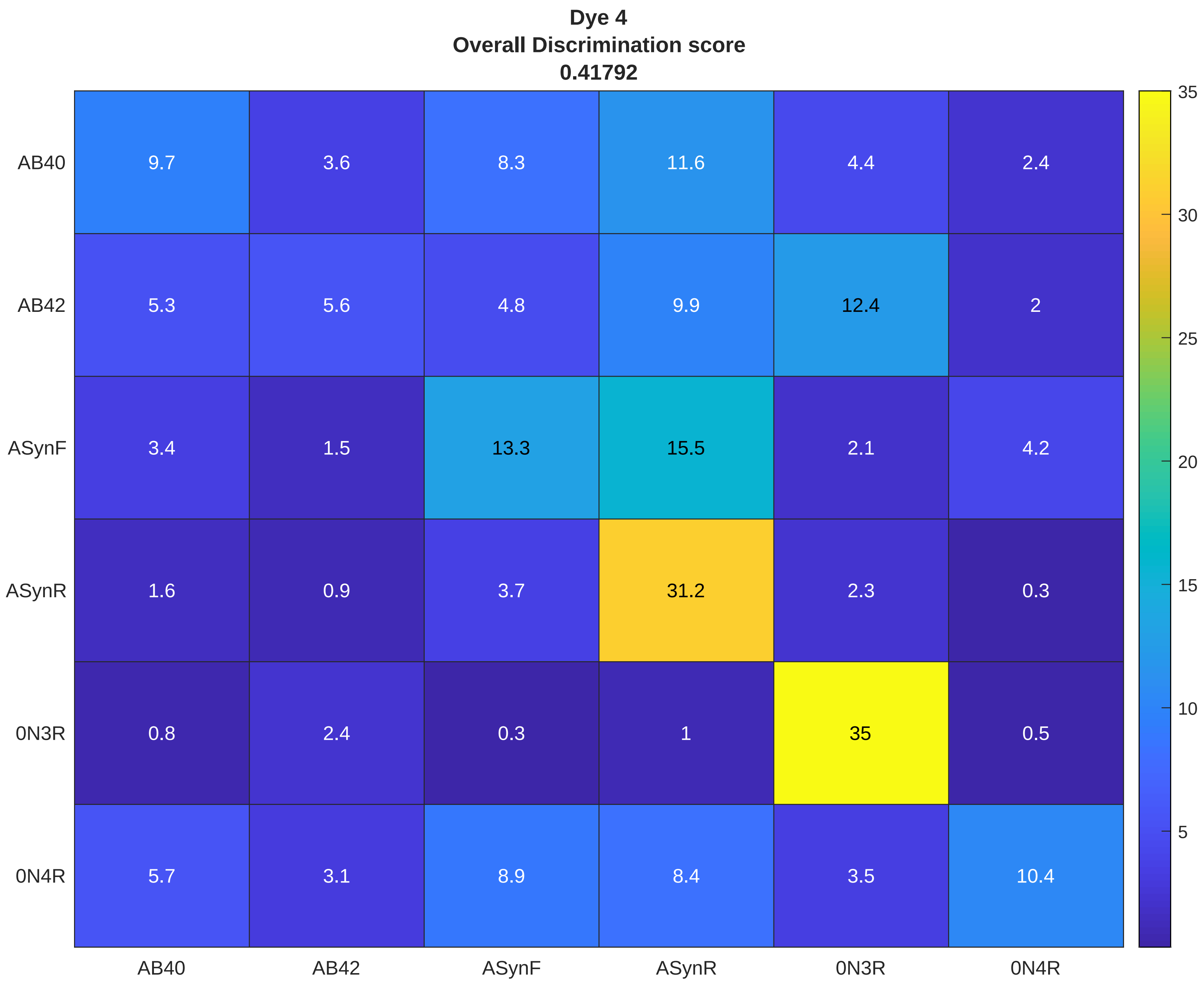

Dye 4
Overall Discrimination score
0.41792
AB40
AB42
ASynF
ASynR
0N3R
0N4R
AB40 9.7 3.6 8.3 11.6 4.4 2.4
AB42 5.3 5.6 4.8 9.9 12.4 2
ASynF 3.4 1.5 13.3 15.5 2.1 4.2
ASynR 1.6 0.9 3.7 31.2 2.3 0.3
0N3R 0.8 2.4 0.3 1 35 0.5
0N4R 5.7 3.1 8.9 8.4 3.5 10.4
AB40
AB42
ASynF
ASynR
0N3R
0N4R
35
30
25
20
15
10
5

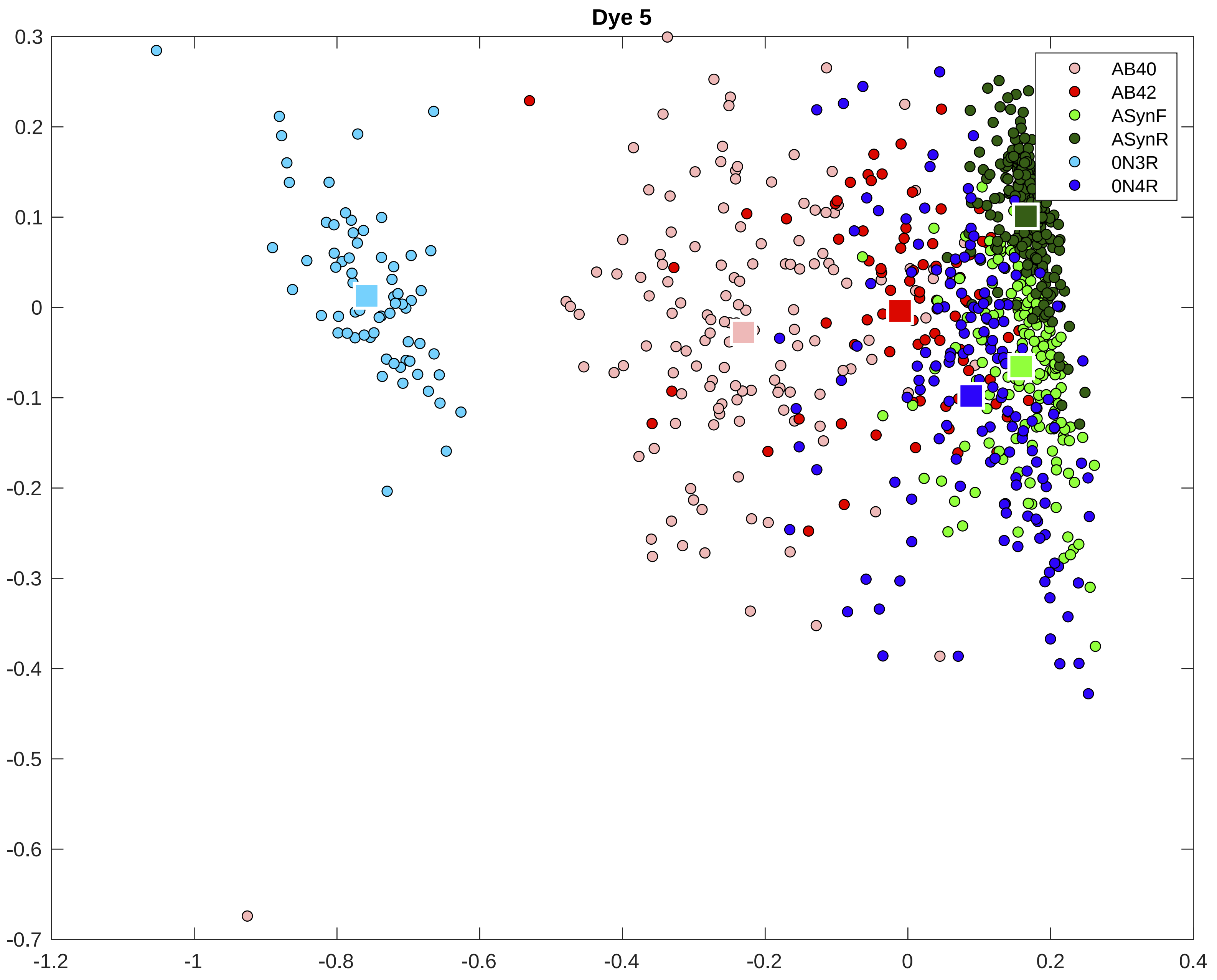
Dye 5
AB40
AB42
ASynF
ASynR
0N3R
0N4R
0.3
0.2
0.1
0
-0.1
-0.2
-0.3
-0.4
-0.5
-0.6
-0.7
-1.2
-1
-0.8
-0.6
-0.4
-0.2
0
0.2
0.4

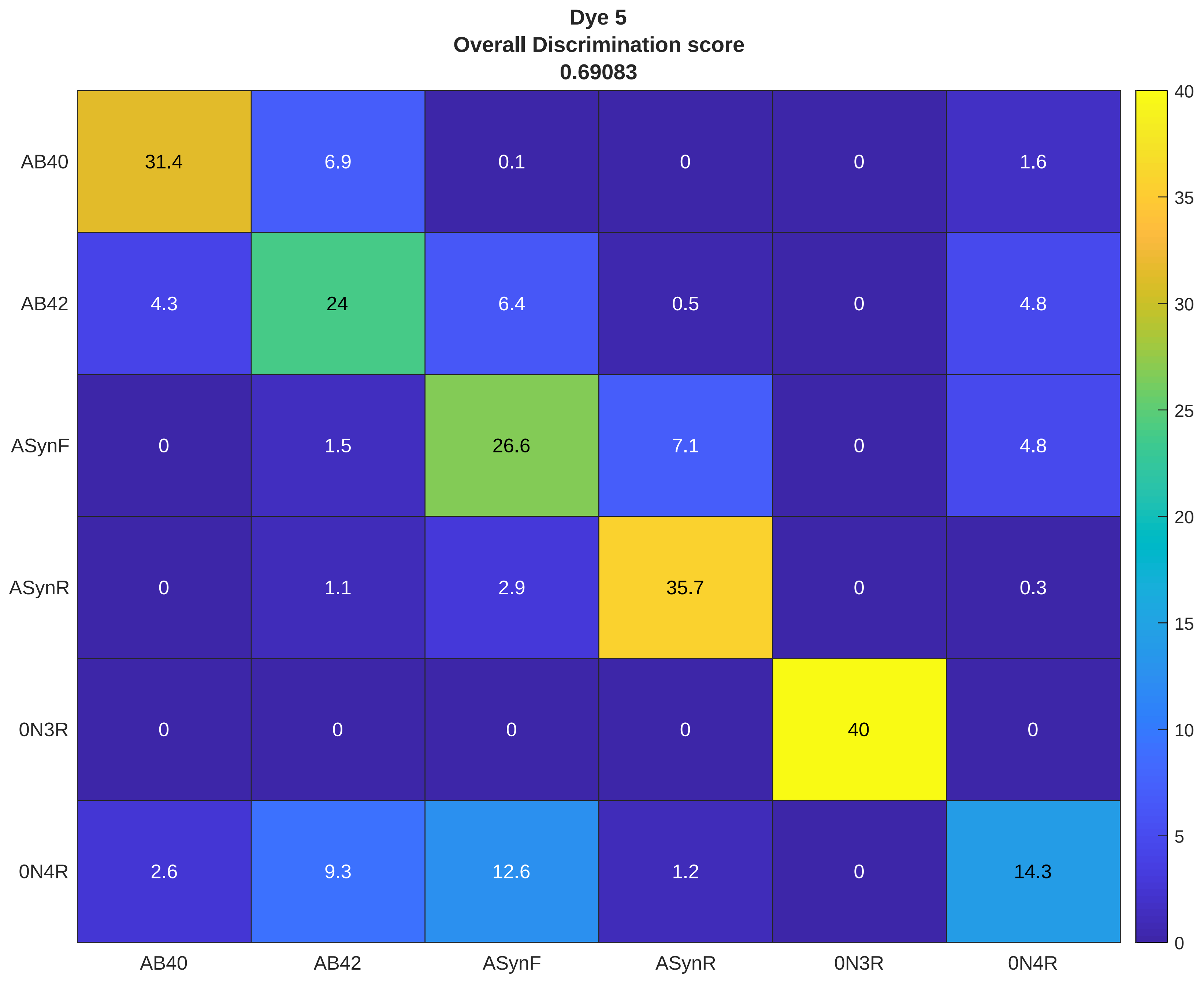
Dye 5
Overall Discrimination score
0.69083
AB40
AB42
ASynF
ASynR
0N3R
0N4R
31.4 6.9 0.1 0 0 1.6
4.3 24 6.4 0.5 0 4.8
0 1.5 26.6 7.1 0 4.8
0 1.1 2.9 35.7 0 0.3
0 0 0 0 40 0
2.6 9.3 12.6 1.2 0 14.3
AB40
AB42
ASynF
ASynR
0N3R
0N4R
40
35
30
25
20
15
10
5
0

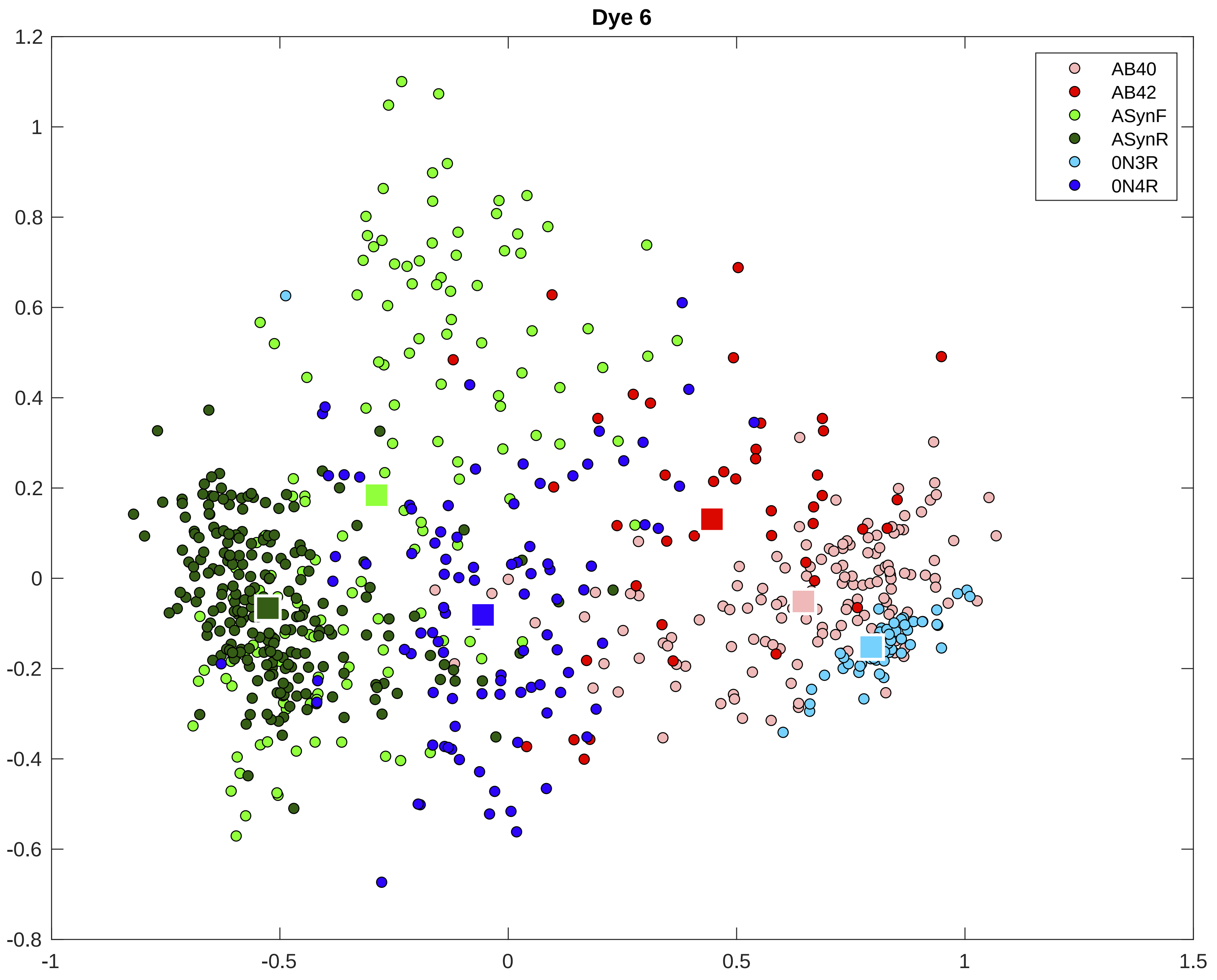

Dye 6
1.2
1
0.8
0.6
0.4
0.2
0
-0.2
-0.4
-0.6
-0.8
-1
-0.5
0
0.5
1
1.5
AB40
AB42
ASynF
ASynR
0N3R
0N4R

Dye 6
Overall Discrimination score
0.69167

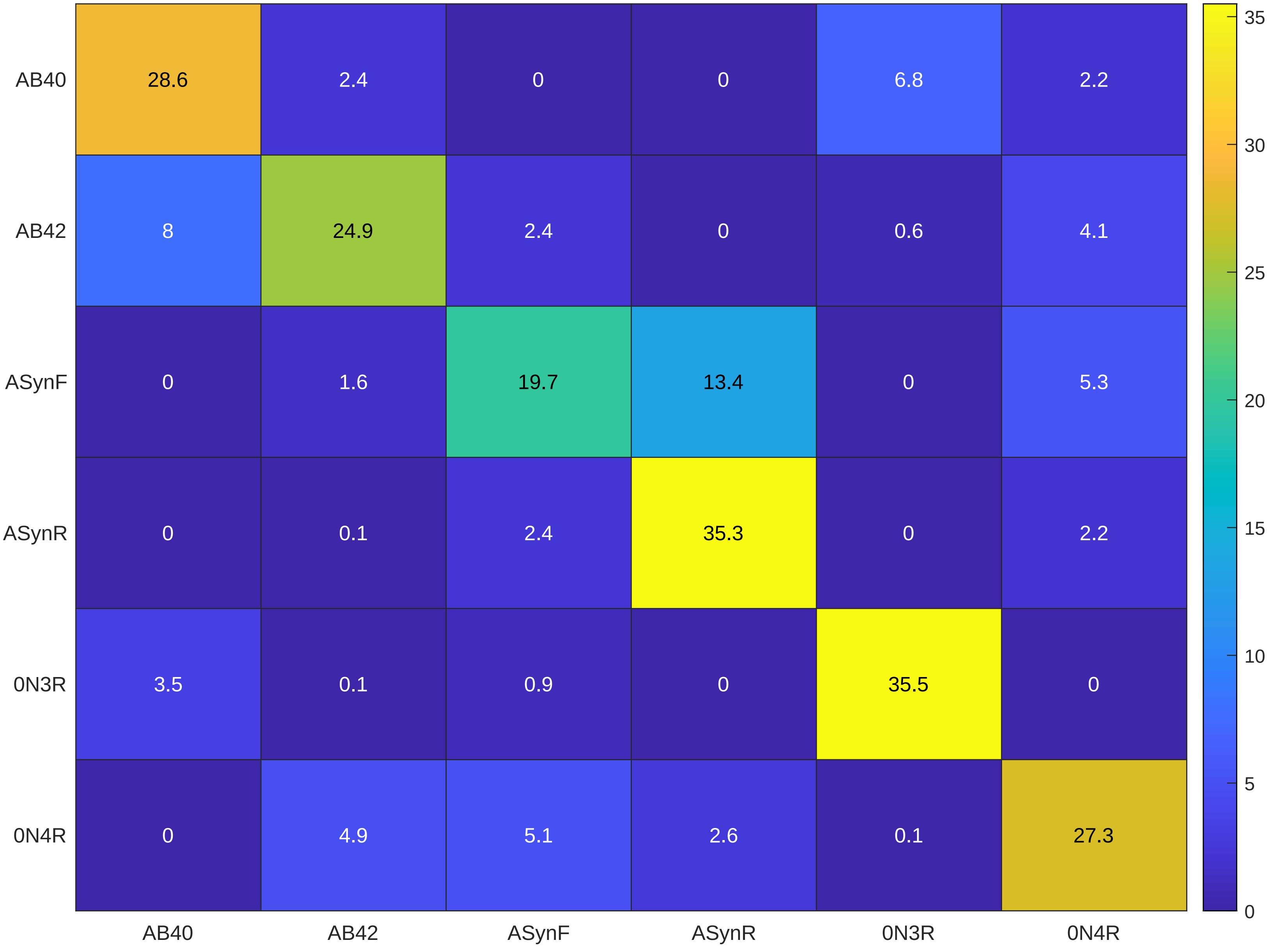

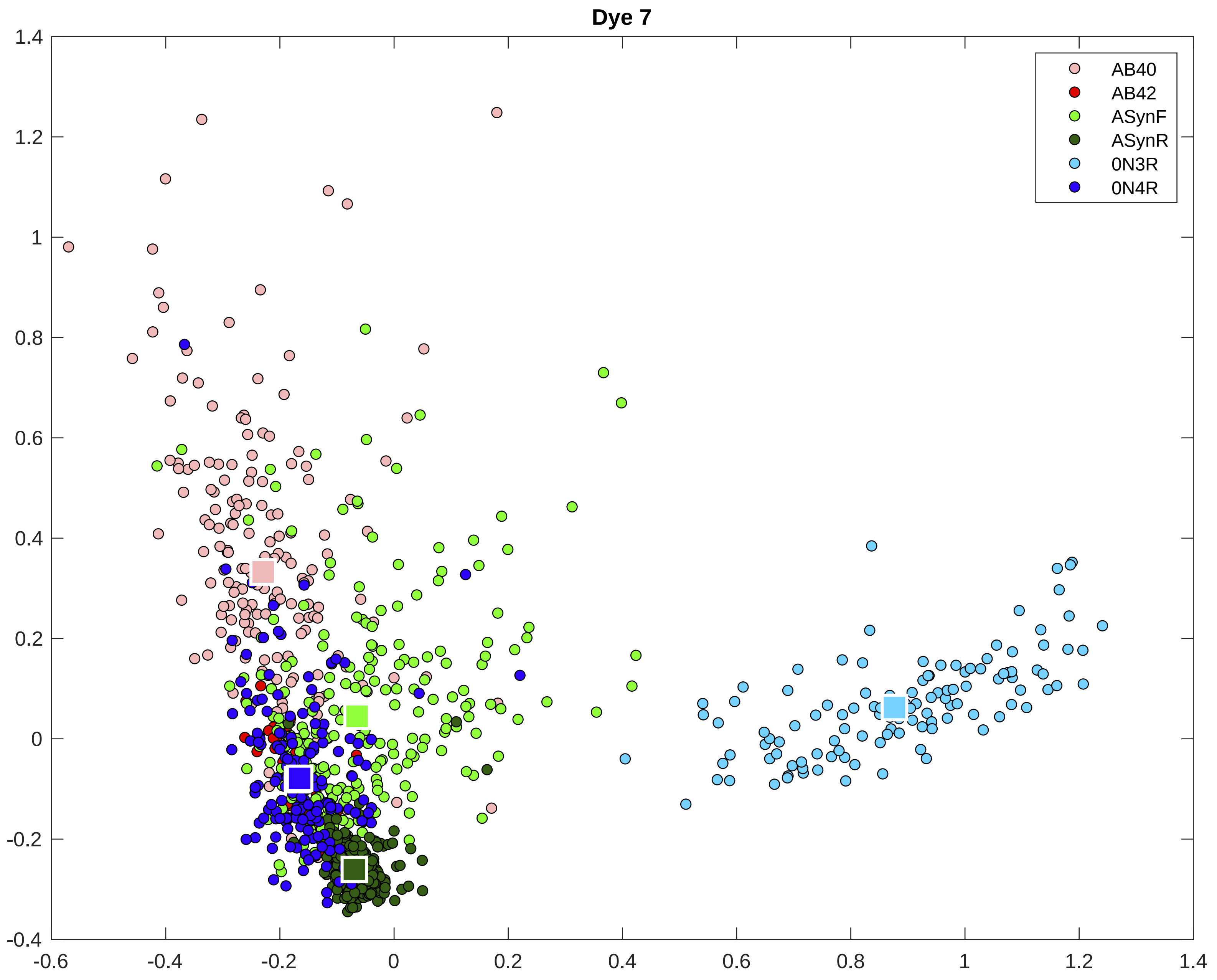
Dye 7
AB40
AB42
ASynF
ASynR
0N3R
0N4R
1.4
1.2
1
0.8
0.6
0.4
0.2
0
-0.2
-0.4
-0.6
-0.4
-0.2
0
0.2
0.4
0.6
0.8
1
1.2
1.4

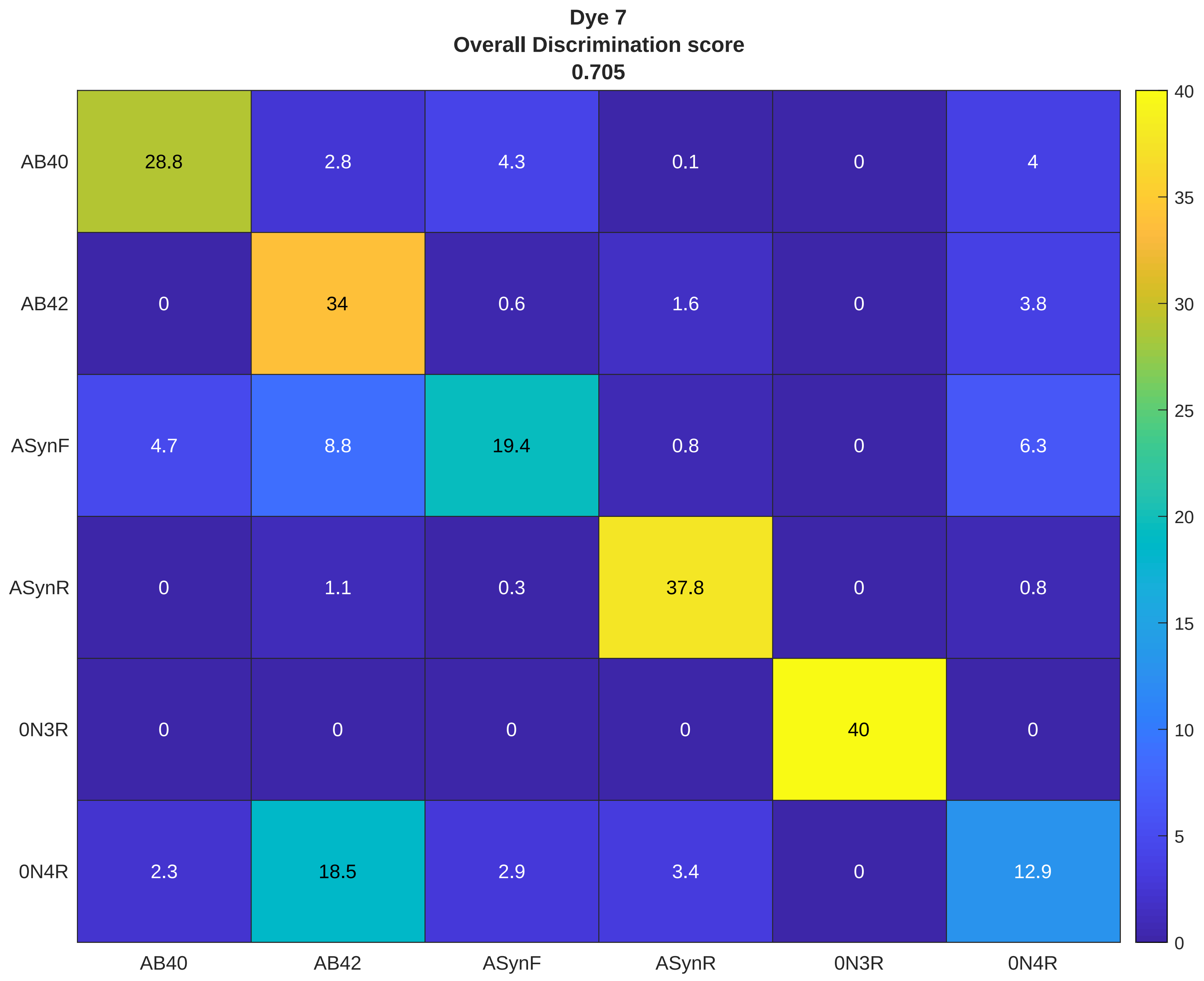

Dye 7
Overall Discrimination score
0.705
AB40
AB42
ASynF
ASynR
0N3R
0N4R
28.8
2.8
4.3
0.1
0
4
0
34
0.6
1.6
0
3.8
4.7
8.8
19.4
0.8
0
6.3
0
1.1
0.3
37.8
0
0.8
0
0
0
0
40
0
2.3
18.5
2.9
3.4
0
12.9
AB40
AB42
ASynF
ASynR
0N3R
0N4R
40
35
30
25
20
15
10
5
0

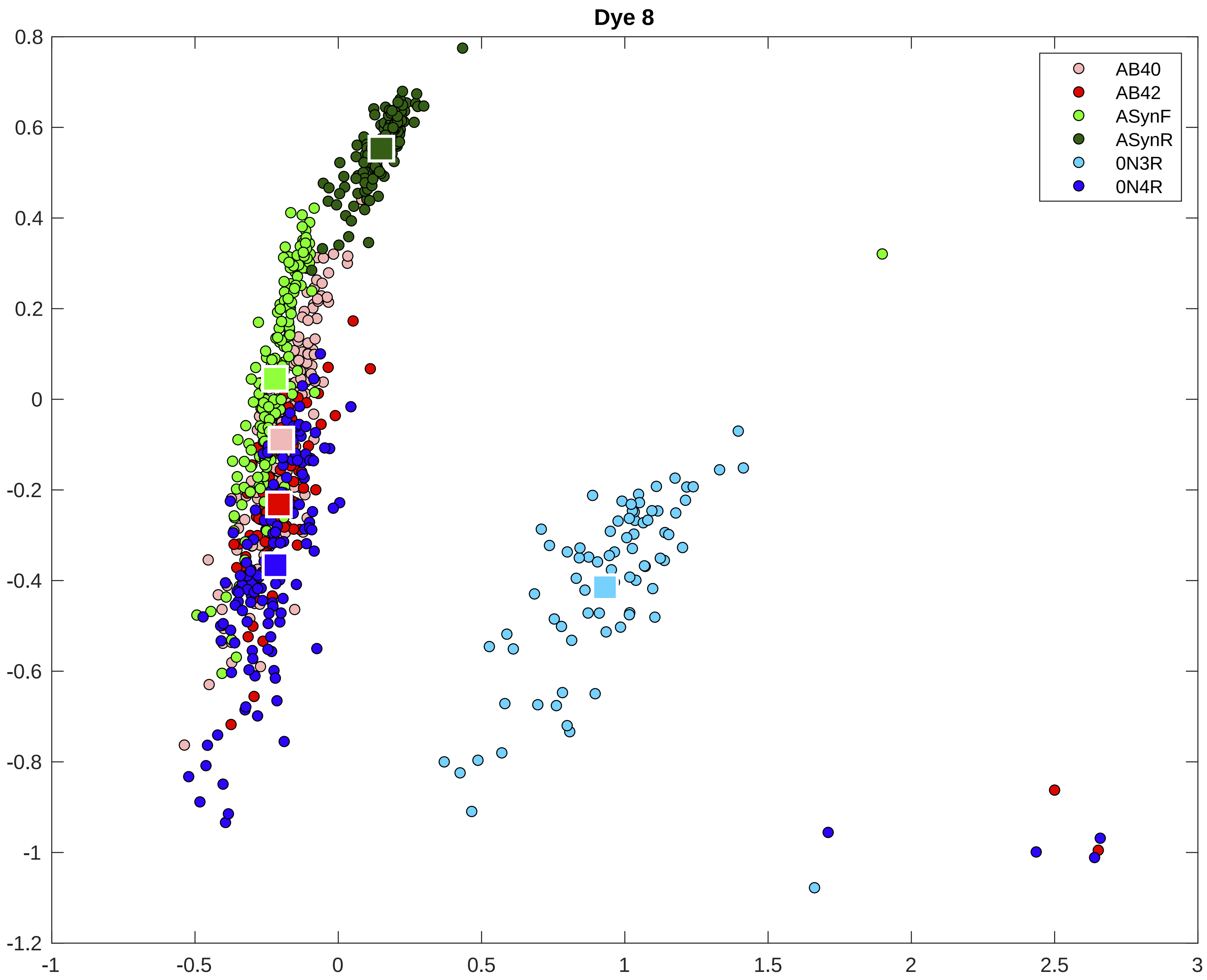

Dye 8
0.8
0.6
0.4
0.2
0
-0.2
-0.4
-0.6
-0.8
-1
-1.2
-1
-0.5
0
0.5
1
1.5
2
2.5
3
AB40
AB42
ASynF
ASynR
0N3R
0N4R

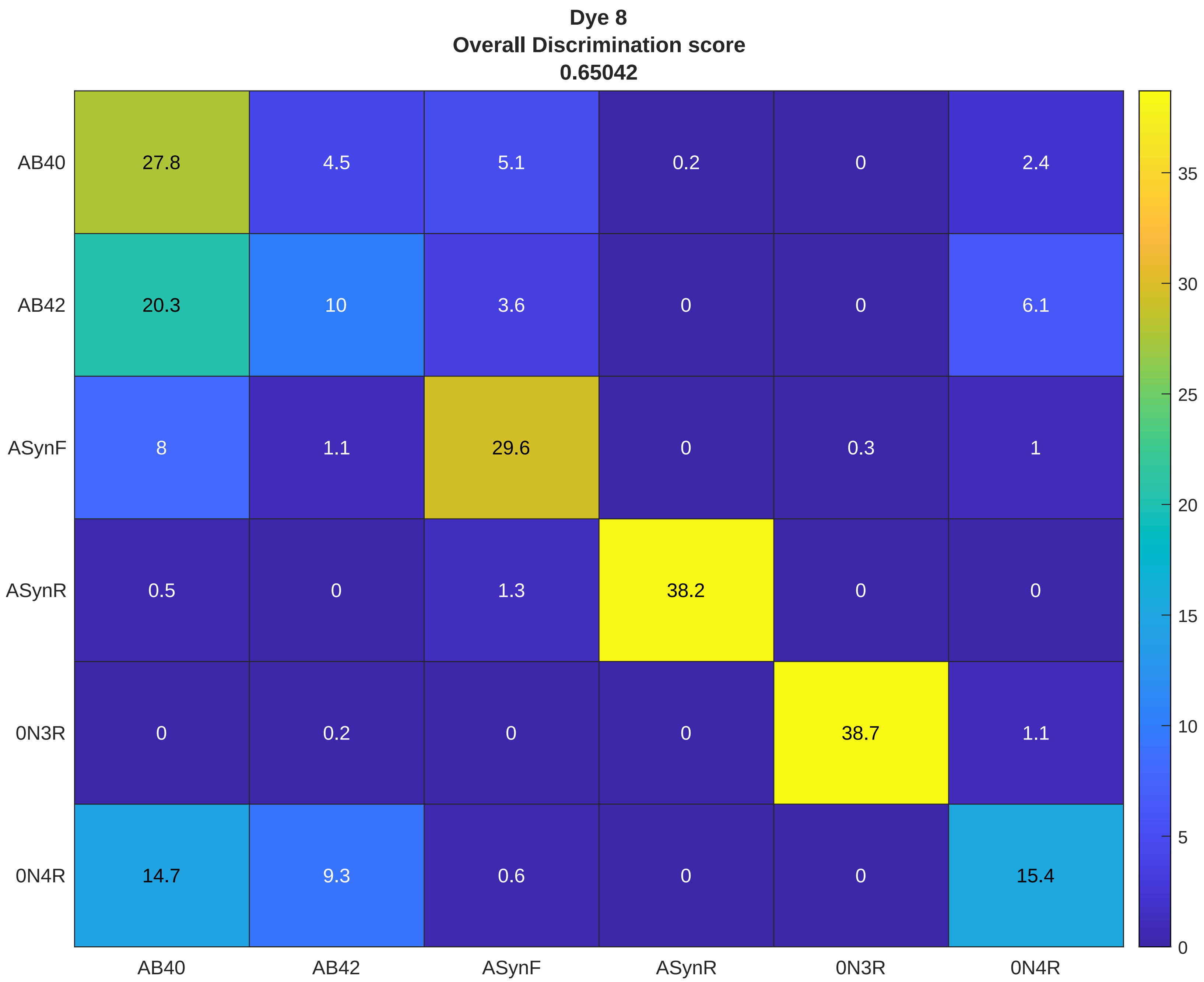

Dye 8
Overall Discrimination score
0.65042
AB40
AB42
ASynF
ASynR
0N3R
0N4R
27.8
4.5
5.1
0.2
0
2.4
20.3
10
3.6
0
0
6.1
8
1.1
29.6
0
0.3
1
0.5
0
1.3
38.2
0
0
0
0.2
0
0
38.7
1.1
14.7
9.3
0.6
0
0
15.4
AB40
AB42
ASynF
ASynR
0N3R
0N4R
35
30
25
20
15
10
5
0

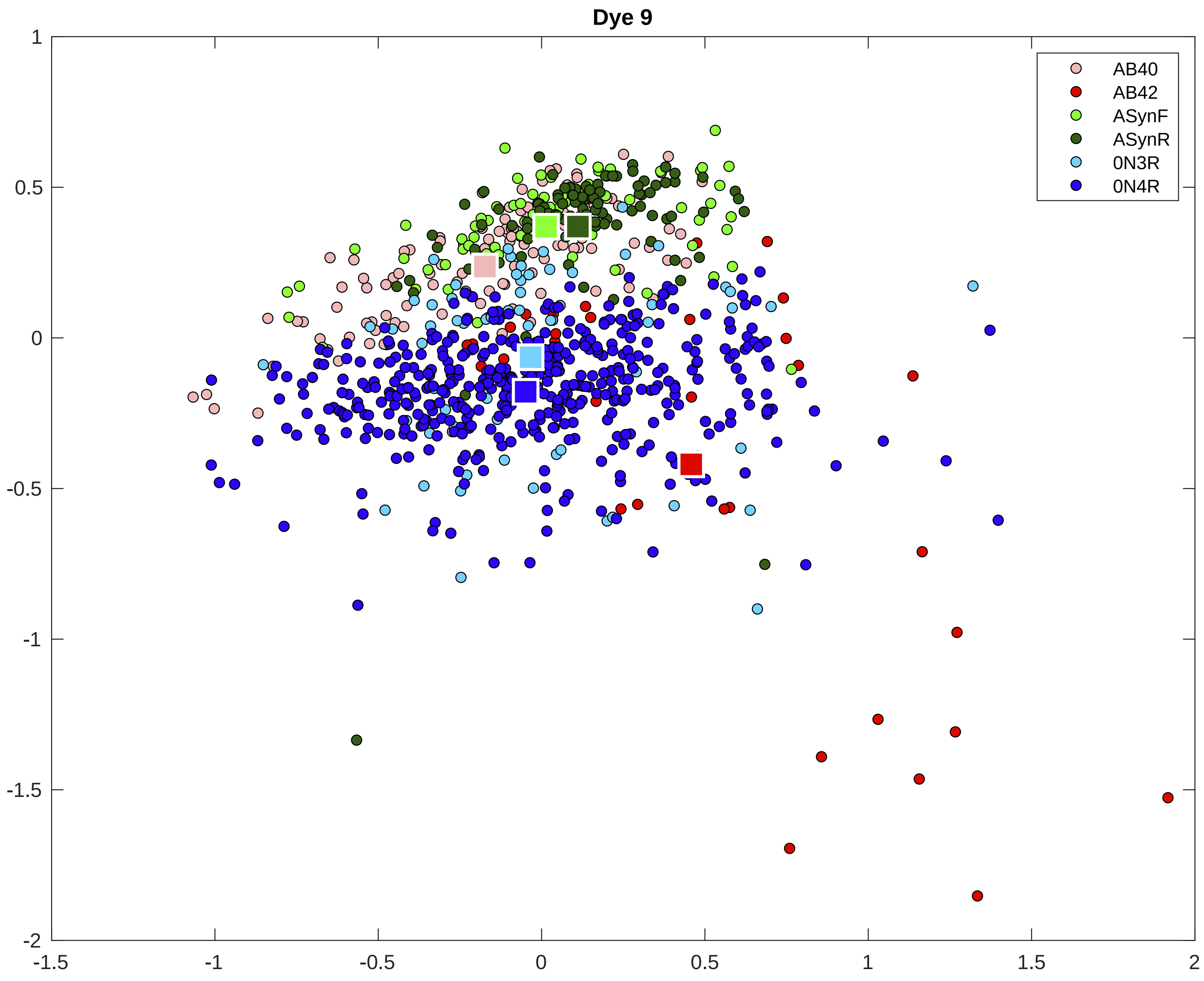

Dye 9
AB40
AB42
ASynF
ASynR
0N3R
0N4R
1
0.5
0
-0.5
-1
-1.5
-2
-1.5
-1
-0.5
0
0.5
1
1.5
2

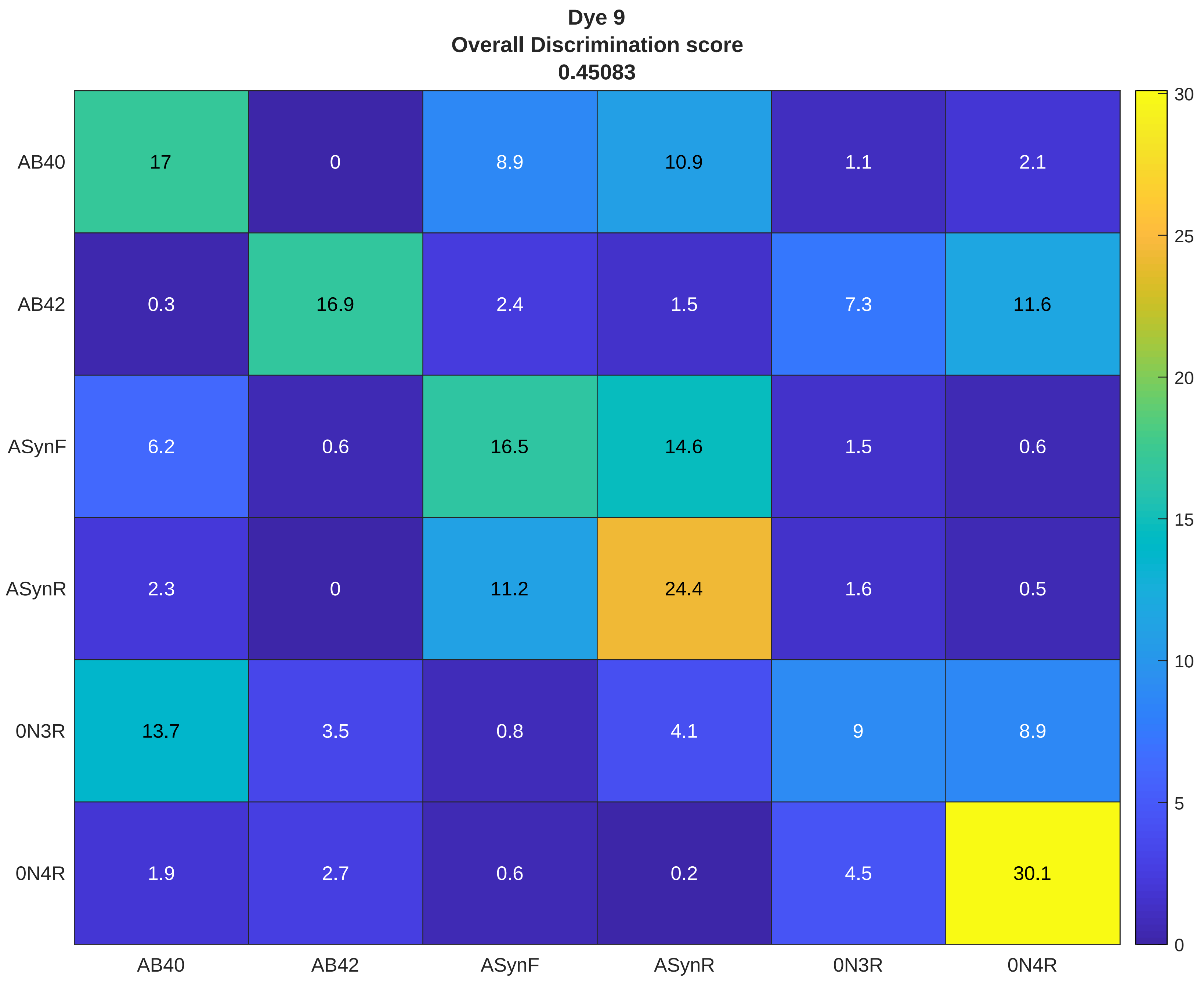

Dye 9
Overall Discrimination score
0.45083
AB40
AB42
ASynF
ASynR
0N3R
0N4R
17
0
8.9
10.9
1.1
2.1
0.3
16.9
2.4
1.5
7.3
11.6
6.2
0.6
16.5
14.6
1.5
0.6
2.3
0
11.2
24.4
1.6
0.5
13.7
3.5
0.8
4.1
9
8.9
1.9
2.7
0.6
0.2
4.5
30.1
AB40
AB42
ASynF
ASynR
0N3R
0N4R
30
25
20
15
10
5
0

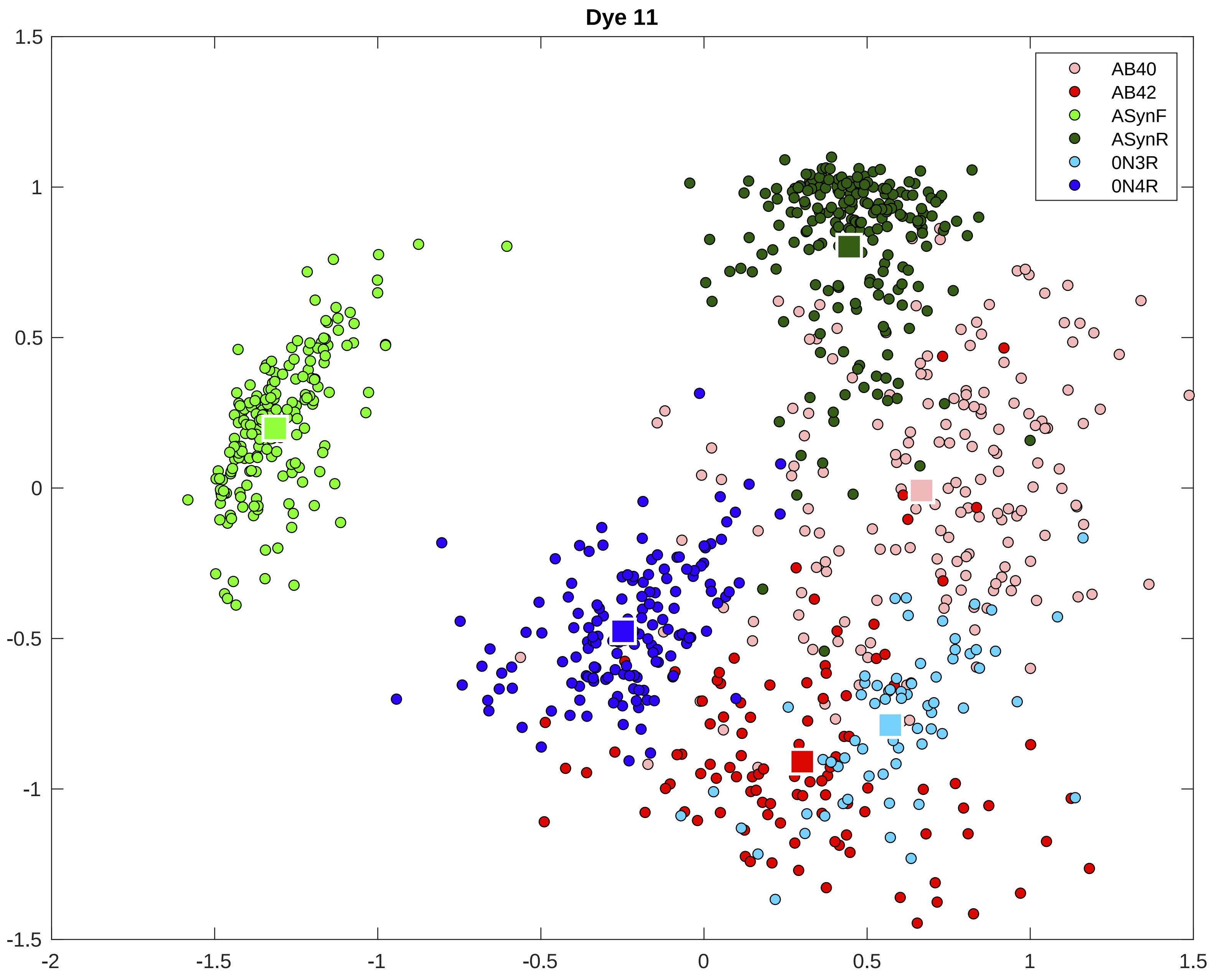
Dye 11
AB40
AB42
ASynF
ASynR
0N3R
0N4R
1.5
1
0.5
0
-0.5
-1
-1.5
-2
-1.5
-1
-0.5
0
0.5
1
1.5

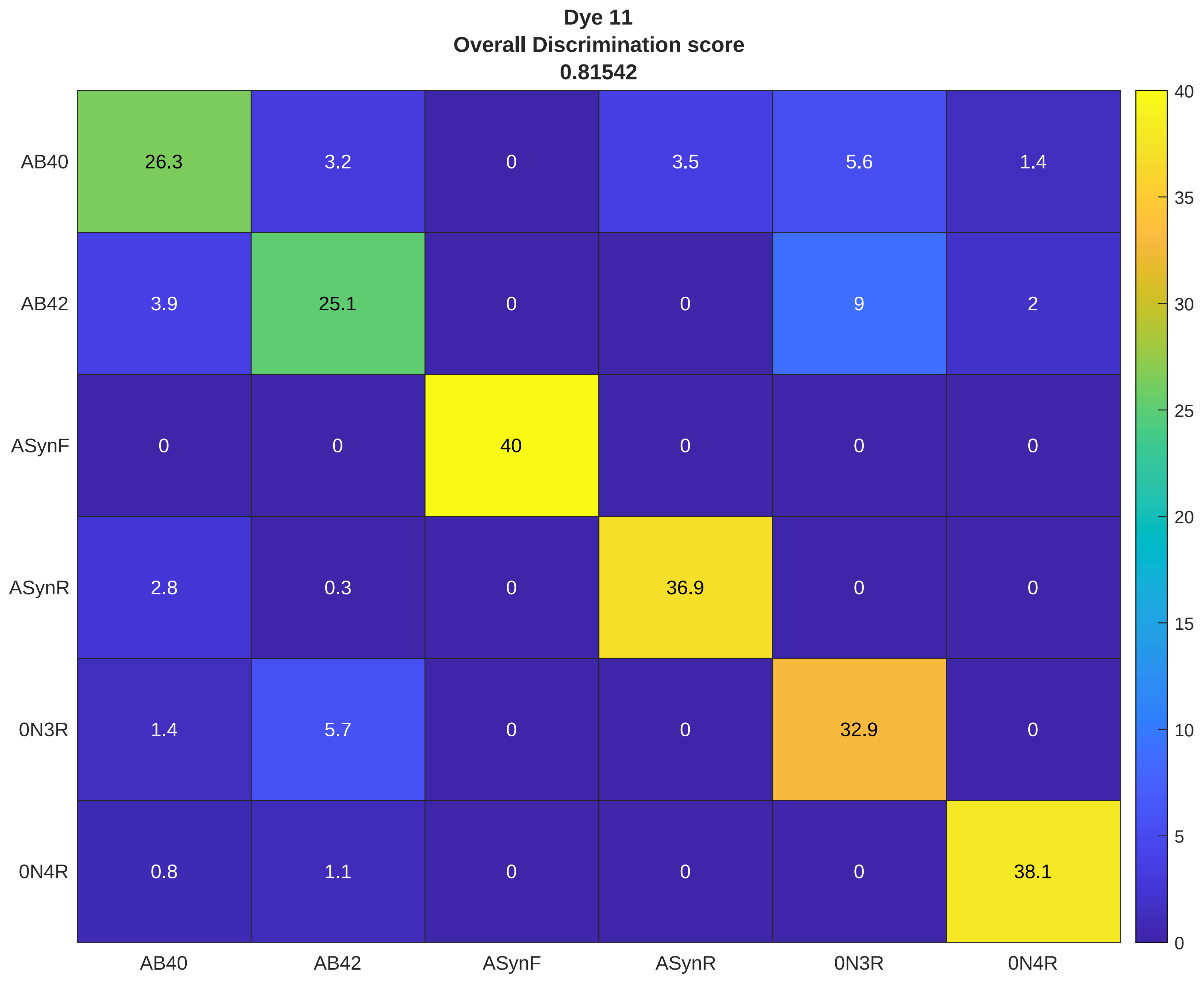
Dye 11
Overall Discrimination score
0.81542
AB40
AB42
ASynF
ASynR
0N3R
0N4R
26.3
3.2
0
3.5
5.6
1.4
3.9
25.1
0
0
9
2
0
0
40
0
0
0
2.8
0.3
0
36.9
0
0
1.4
5.7
0
0
32.9
0
0.8
1.1
0
0
0
38.1
AB40
AB42
ASynF
ASynR
0N3R
0N4R
40
35
30
25
20
15
10
5
0

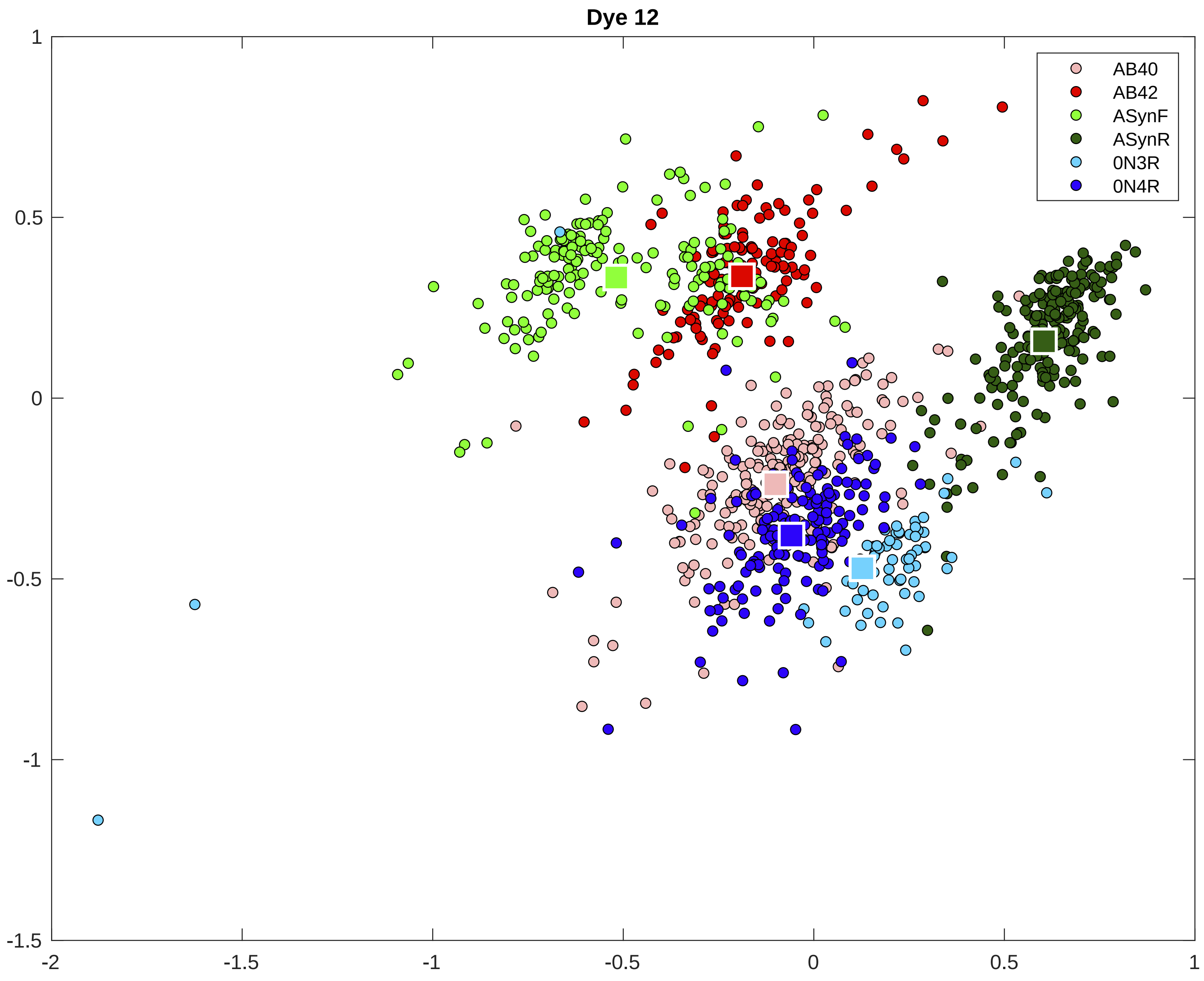

Dye 12
AB40
AB42
ASynF
ASynR
0N3R
0N4R
1
0.5
0
-0.5
-1
-1.5
-2
-1.5
-1
-0.5
0
0.5
1

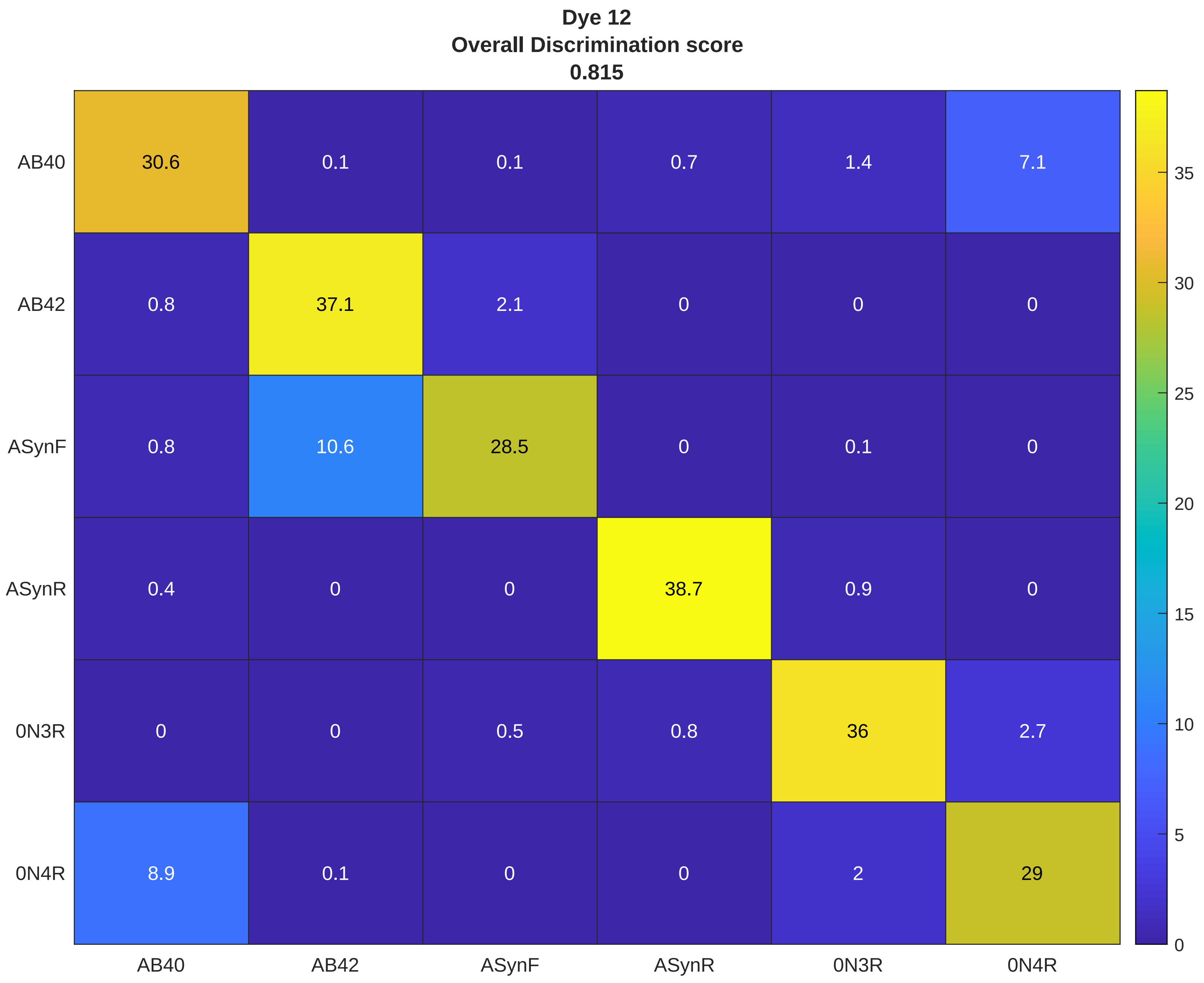
Dye 12
Overall Discrimination score
0.815
AB40
AB42
ASynF
ASynR
0N3R
0N4R
30.6
0.1
0.1
0.7
1.4
7.1
0.8
37.1
2.1
0
0
0
0.8
10.6
28.5
0
0.1
0
0.4
0
0
38.7
0.9
0
0
0
0.5
0.8
36
2.7
8.9
0.1
0
0
2
29
AB40
AB42
ASynF
ASynR
0N3R
0N4R
35
30
25
20
15
10
5
0

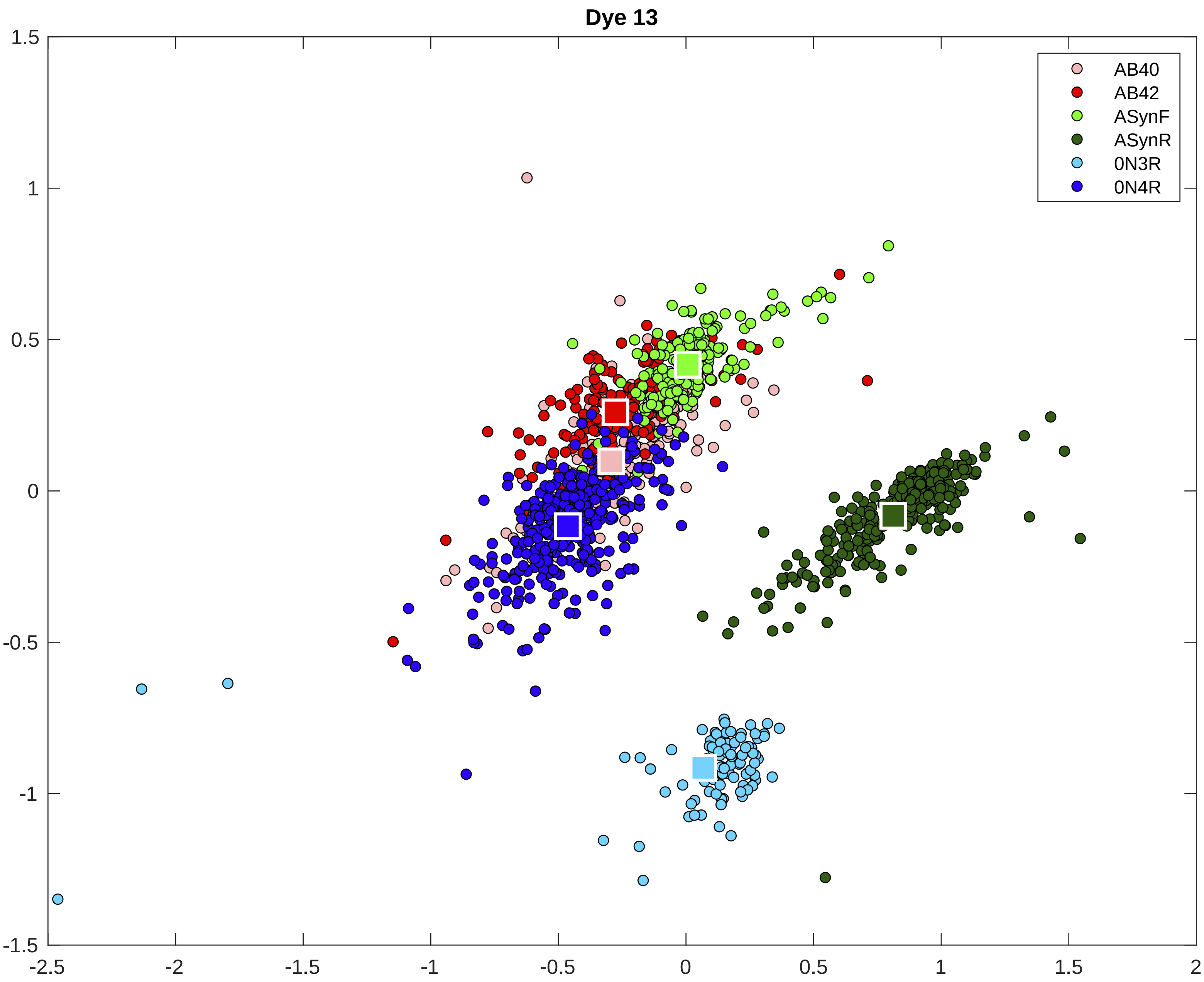
Dye 13
AB40
AB42
ASynF
ASynR
0N3R
0N4R
1.5
1
0.5
0
-0.5
-1
-1.5
-2.5
-2
-1.5
-1
-0.5
0
0.5
1
1.5
2

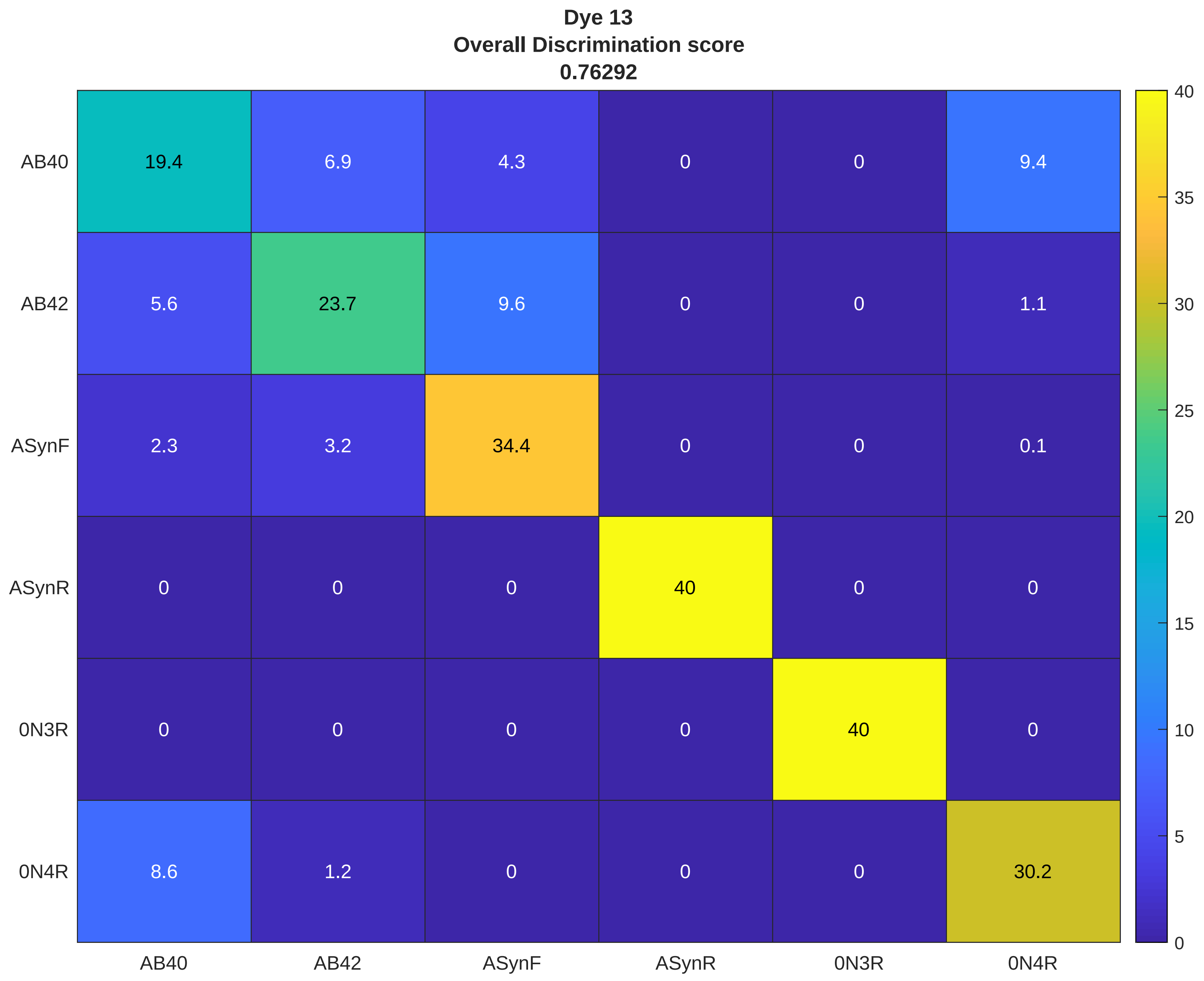
Dye 13
Overall Discrimination score
0.76292
AB40
AB42
ASynF
ASynR
0N3R
0N4R
19.4
6.9
4.3
0
0
9.4
5.6
23.7
9.6
0
0
1.1
2.3
3.2
34.4
0
0
0.1
0
0
0
40
0
0
0
0
0
0
40
0
8.6
1.2
0
0
0
30.2
AB40
AB42
ASynF
ASynR
0N3R
0N4R
40
35
30
25
20
15
10
5
0

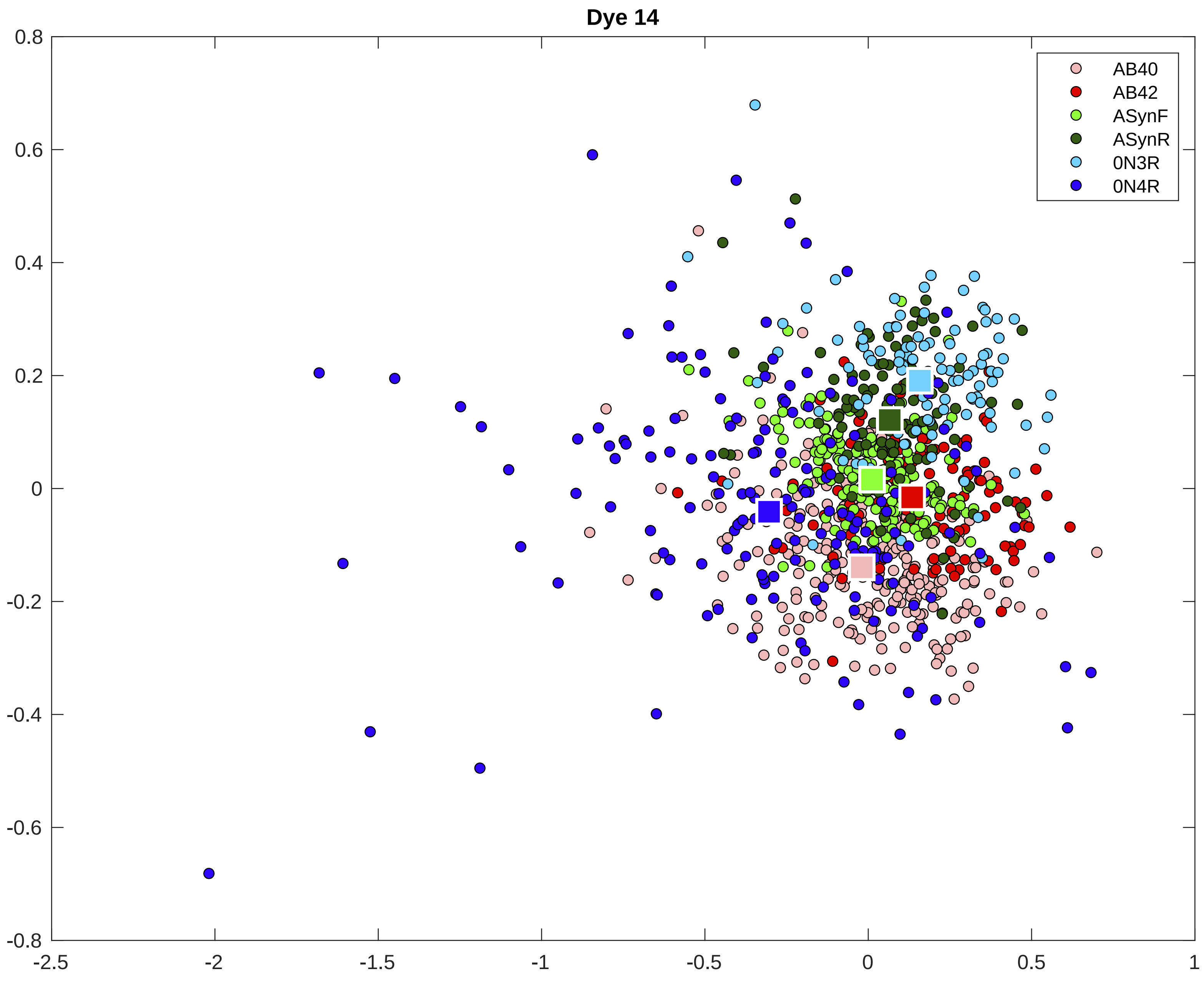
Dye 14
AB40
AB42
ASynF
ASynR
0N3R
0N4R
0.8
0.6
0.4
0.2
0
-0.2
-0.4
-0.6
-0.8
-2.5
-2
-1.5
-1
-0.5
0
0.5
1

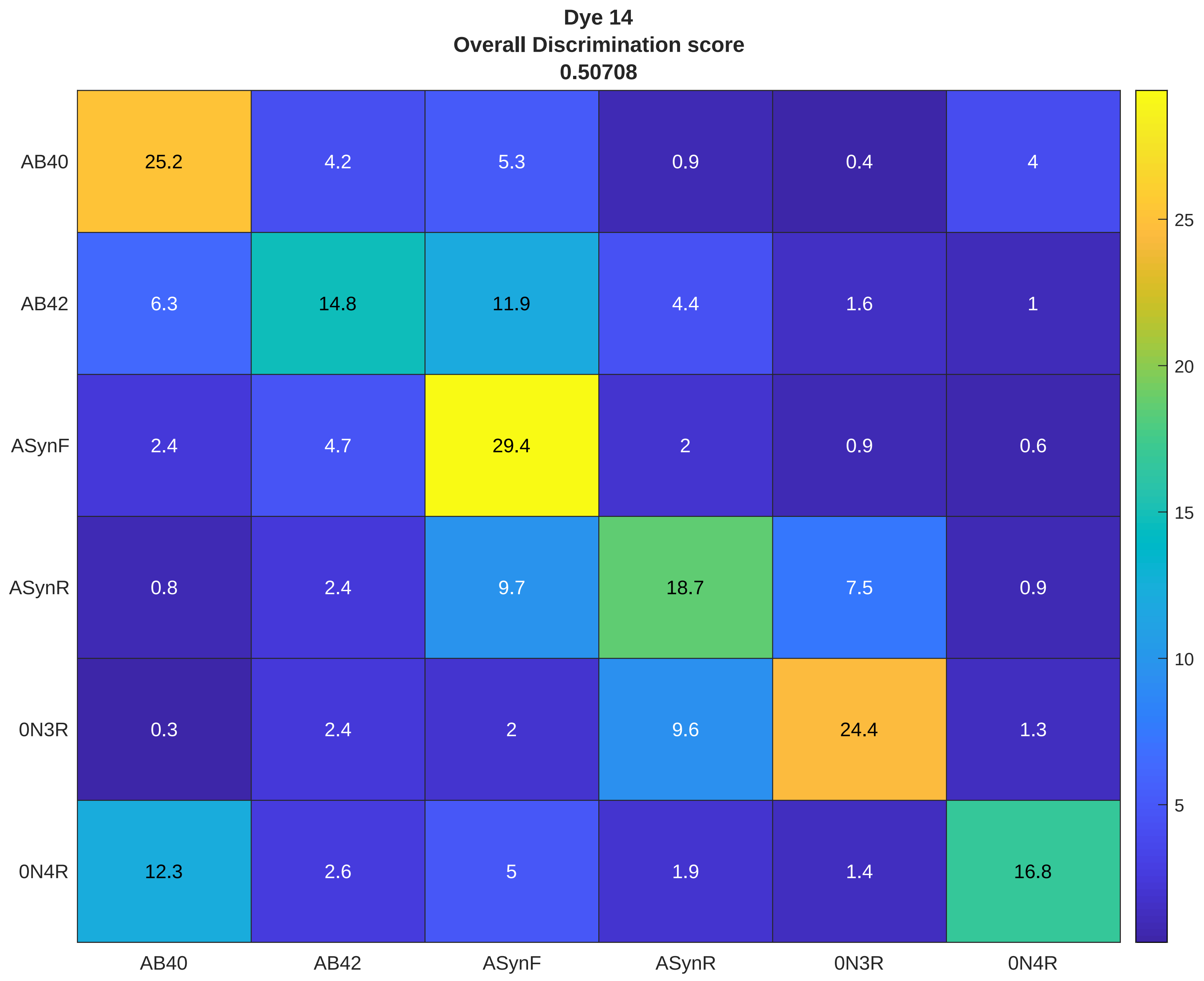

Dye 14
Overall Discrimination score
0.50708
AB40
AB42
ASynF
ASynR
0N3R
0N4R
25.2 4.2 5.3 0.9 0.4 4
6.3 14.8 11.9 4.4 1.6 1
2.4 4.7 29.4 2 0.9 0.6
0.8 2.4 9.7 18.7 7.5 0.9
0.3 2.4 2 9.6 24.4 1.3
12.3 2.6 5 1.9 1.4 16.8
AB40
AB42
ASynF
ASynR
0N3R
0N4R
25
20
15
10
5

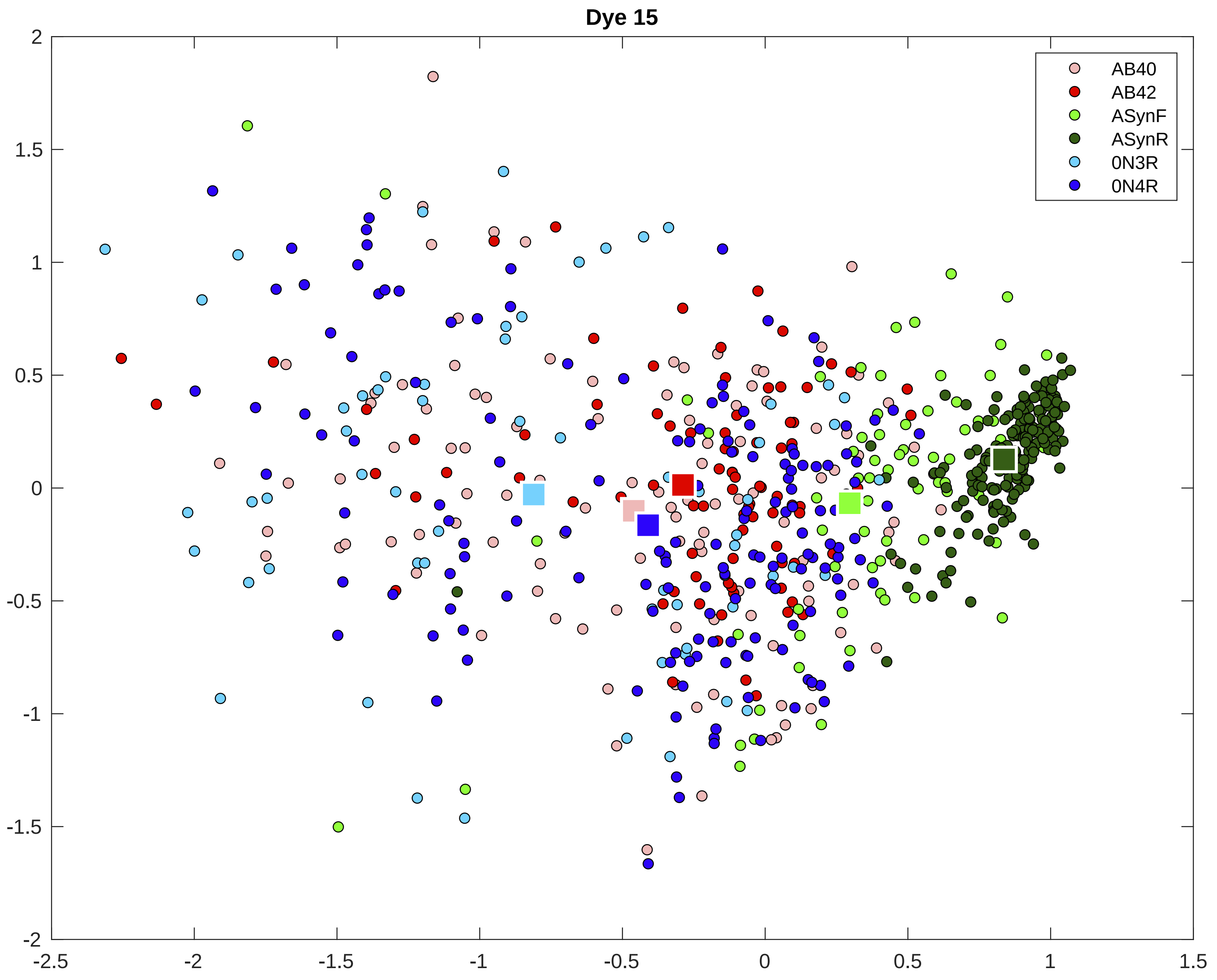
Dye 15
AB40
AB42
ASynF
ASynR
0N3R
0N4R
2
1.5
1
0.5
0
-0.5
-1
-1.5
-2
-2.5
-2
-1.5
-1
-0.5
0
0.5
1
1.5

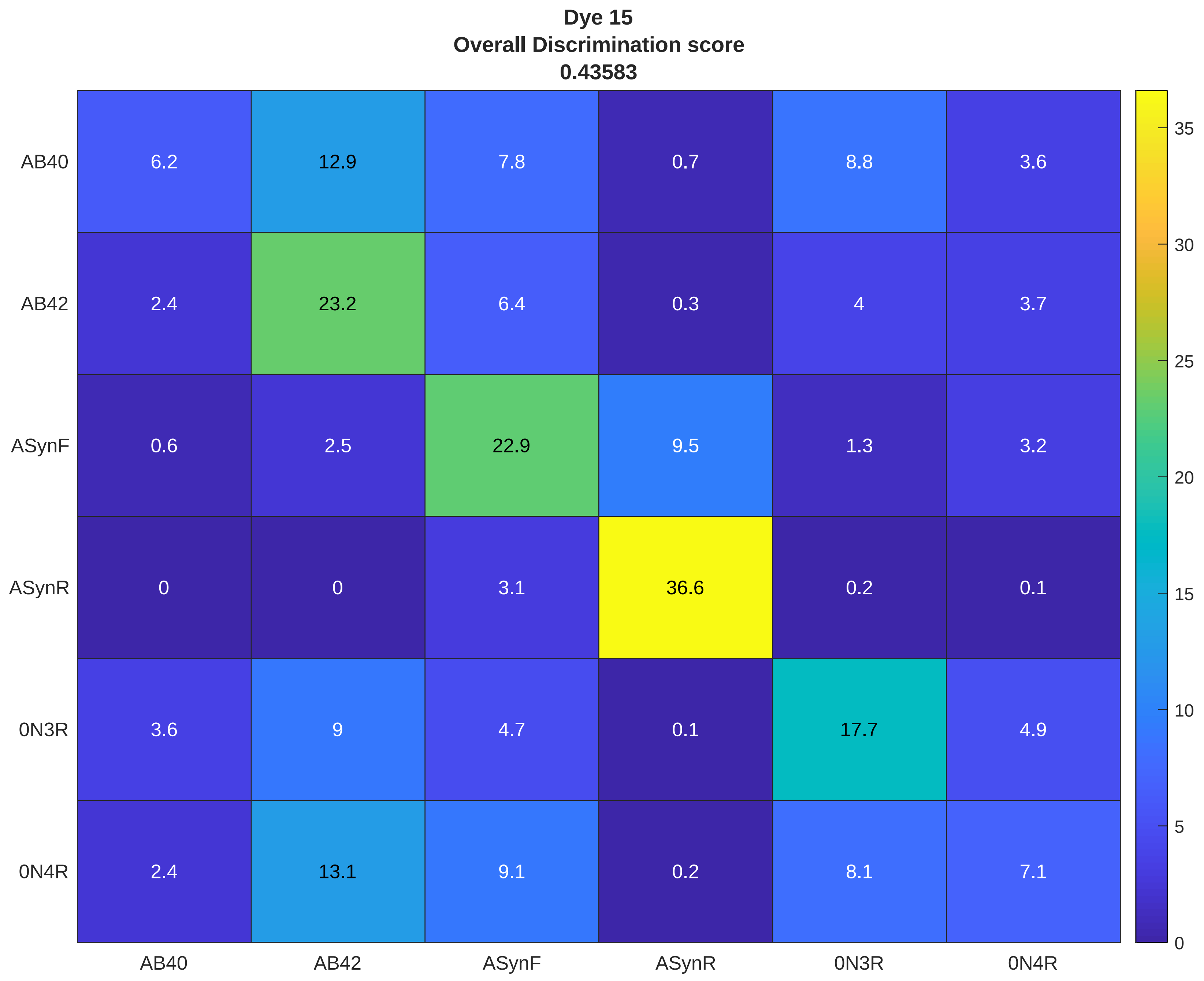
Dye 15
Overall Discrimination score
0.43583
AB40
AB42
ASynF
ASynR
0N3R
0N4R
6.2
12.9
7.8
0.7
8.8
3.6
2.4
23.2
6.4
0.3
4
3.7
0.6
2.5
22.9
9.5
1.3
3.2
0
0
3.1
36.6
0.2
0.1
3.6
9
4.7
0.1
17.7
4.9
2.4
13.1
9.1
0.2
8.1
7.1
AB40
AB42
ASynF
ASynR
0N3R
0N4R
35
30
25
20
15
10
5
0

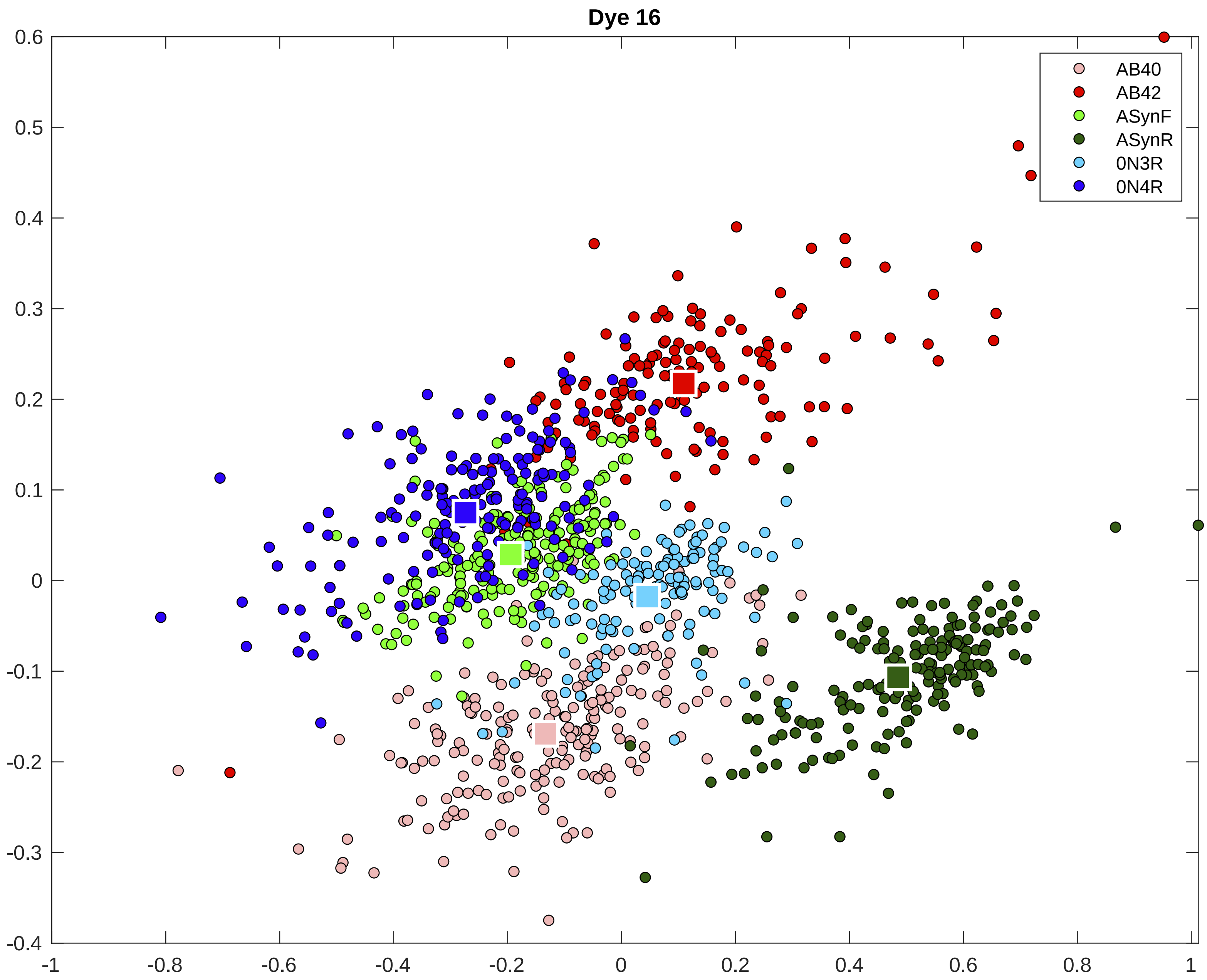

Dye 16
AB40
AB42
ASynF
ASynR
0N3R
0N4R
0.6
0.5
0.4
0.3
0.2
0.1
0
-0.1
-0.2
-0.3
-0.4
-1
-0.8
-0.6
-0.4
-0.2
0
0.2
0.4
0.6
0.8
1

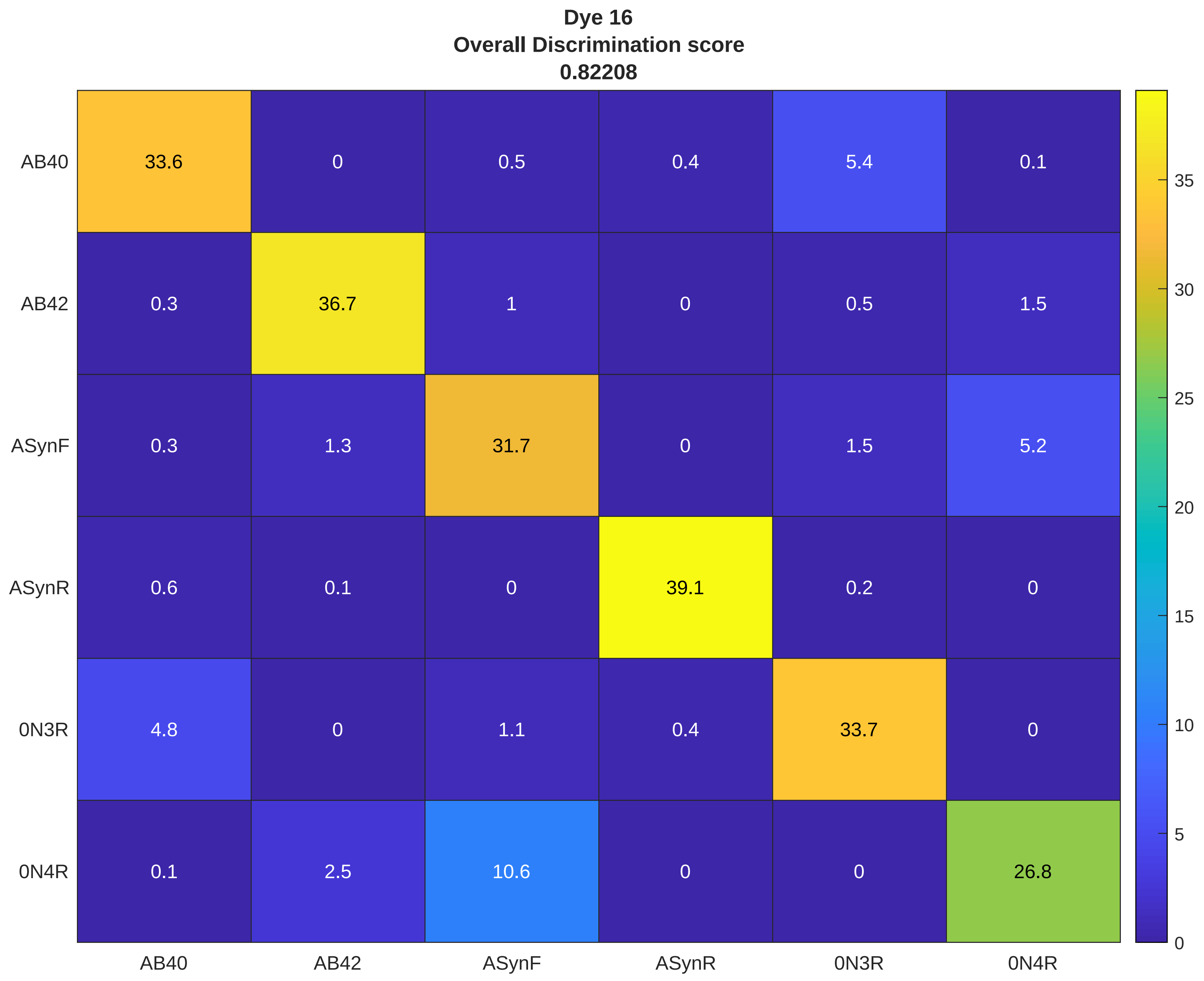
Dye 16
Overall Discrimination score
0.82208
AB40
AB42
ASynF
ASynR
0N3R
0N4R
33.6
0
0.5
0.4
5.4
0.1
0.3
36.7
1
0
0.5
1.5
0.3
1.3
31.7
0
1.5
5.2
0.6
0.1
0
39.1
0.2
0
4.8
0
1.1
0.4
33.7
0
0.1
2.5
10.6
0
0
26.8
AB40
AB42
ASynF
ASynR
0N3R
0N4R
35
30
25
20
15
10
5
0

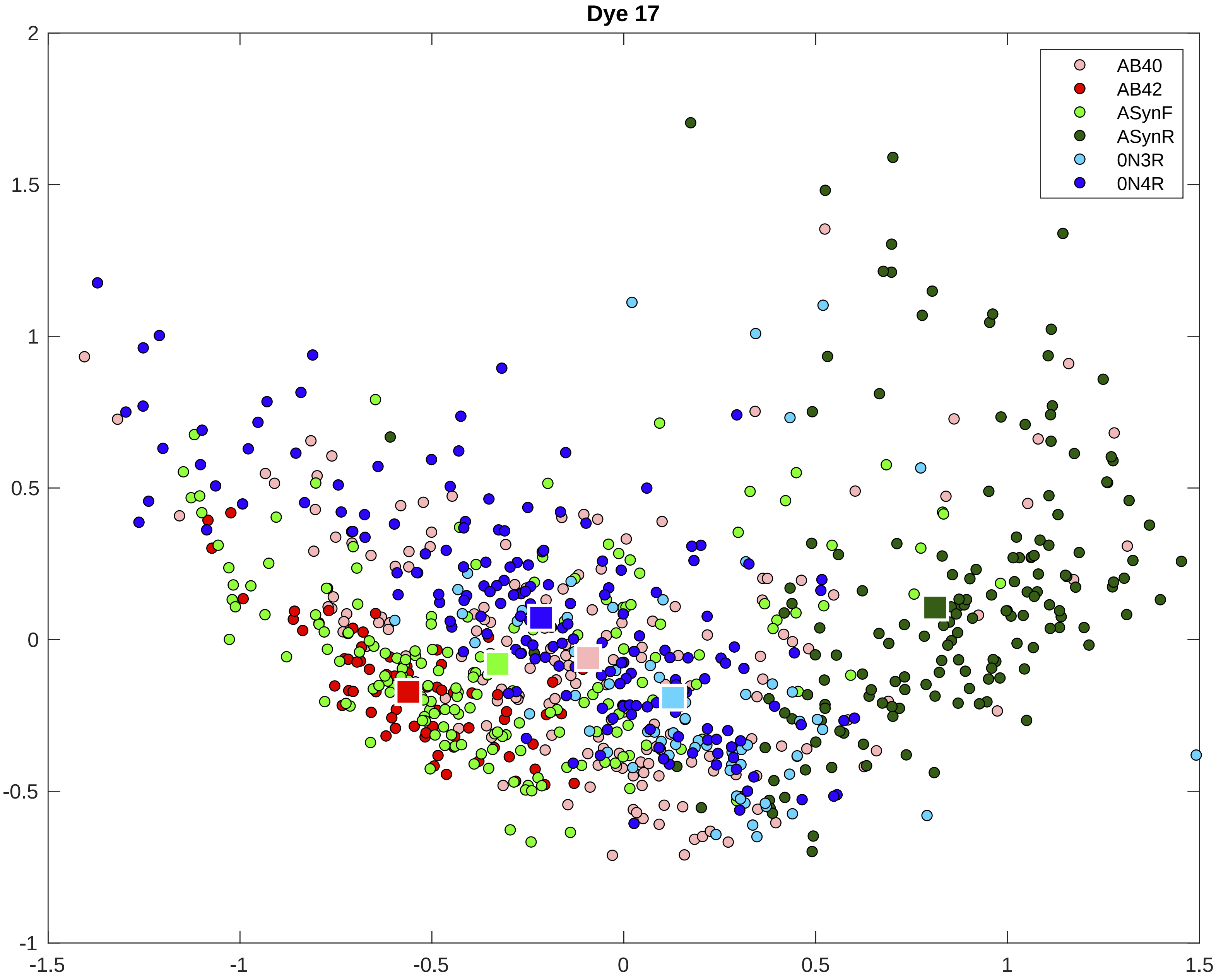
Dye 17
AB40
AB42
ASynF
ASynR
0N3R
0N4R
2
1.5
1
0.5
0
-0.5
-1
-1.5
-1
-0.5
0
0.5
1
1.5

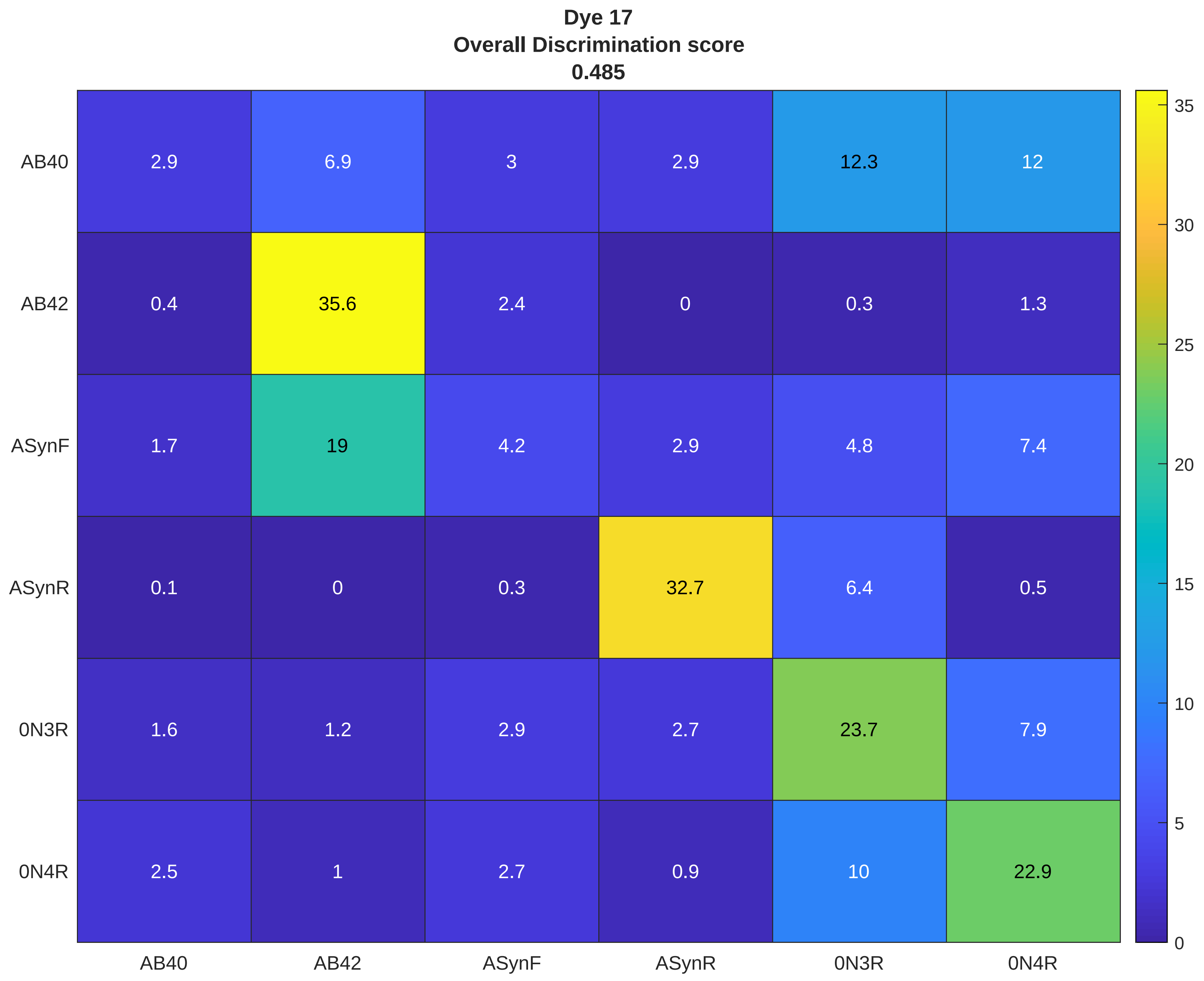
Dye 17
Overall Discrimination score
0.485
AB40
AB42
ASynF
ASynR
0N3R
0N4R
2.9
6.9
3
2.9
12.3
12
0.4
35.6
2.4
0
0.3
1.3
1.7
19
4.2
2.9
4.8
7.4
0.1
0
0.3
32.7
6.4
0.5
1.6
1.2
2.9
2.7
23.7
7.9
2.5
1
2.7
0.9
10
22.9
0
5
10
15
20
25
30
35

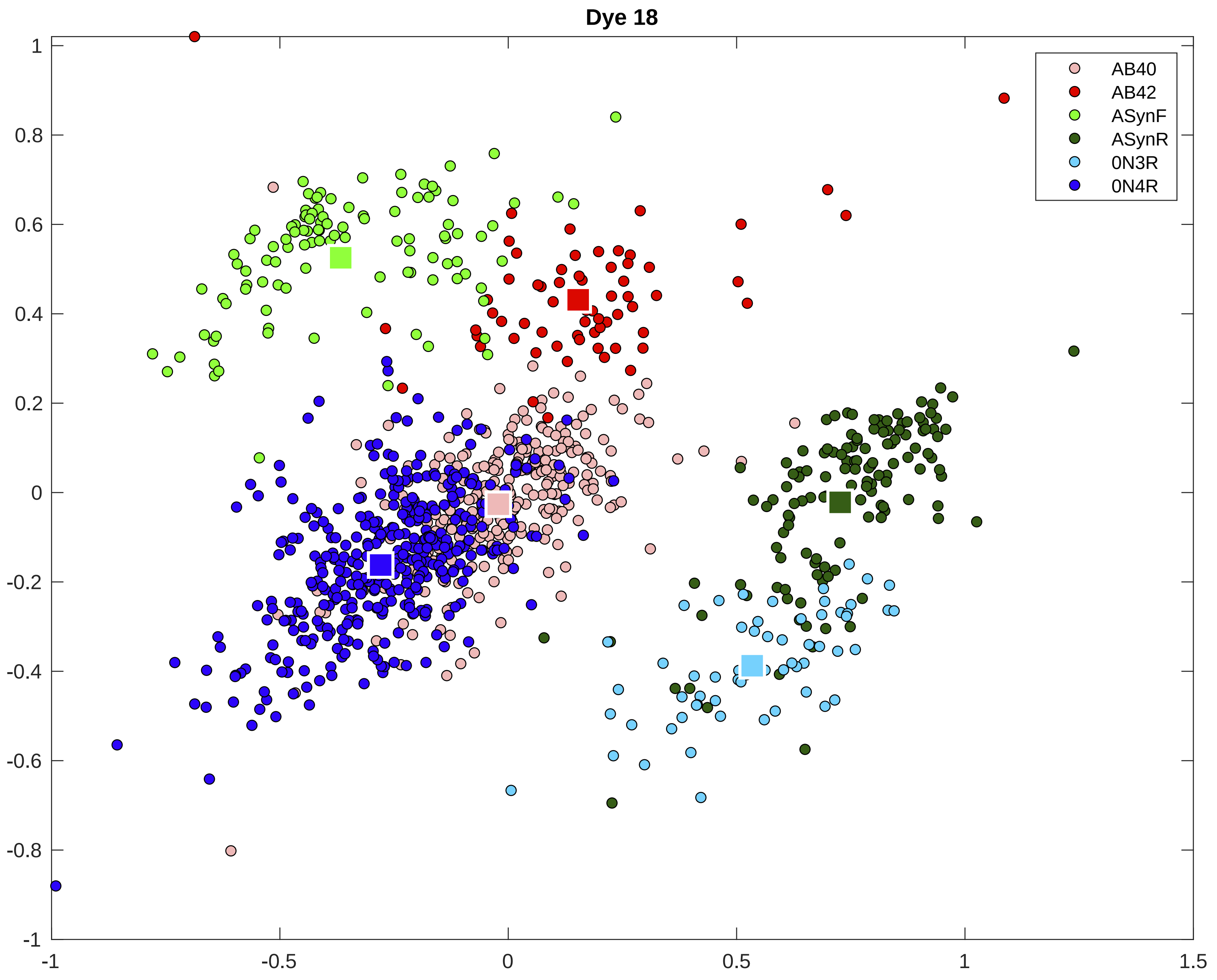
Dye 18
AB40
AB42
ASynF
ASynR
0N3R
0N4R
1
0.8
0.6
0.4
0.2
0
-0.2
-0.4
-0.6
-0.8
-1
-1
-0.5
0
0.5
1
1.5

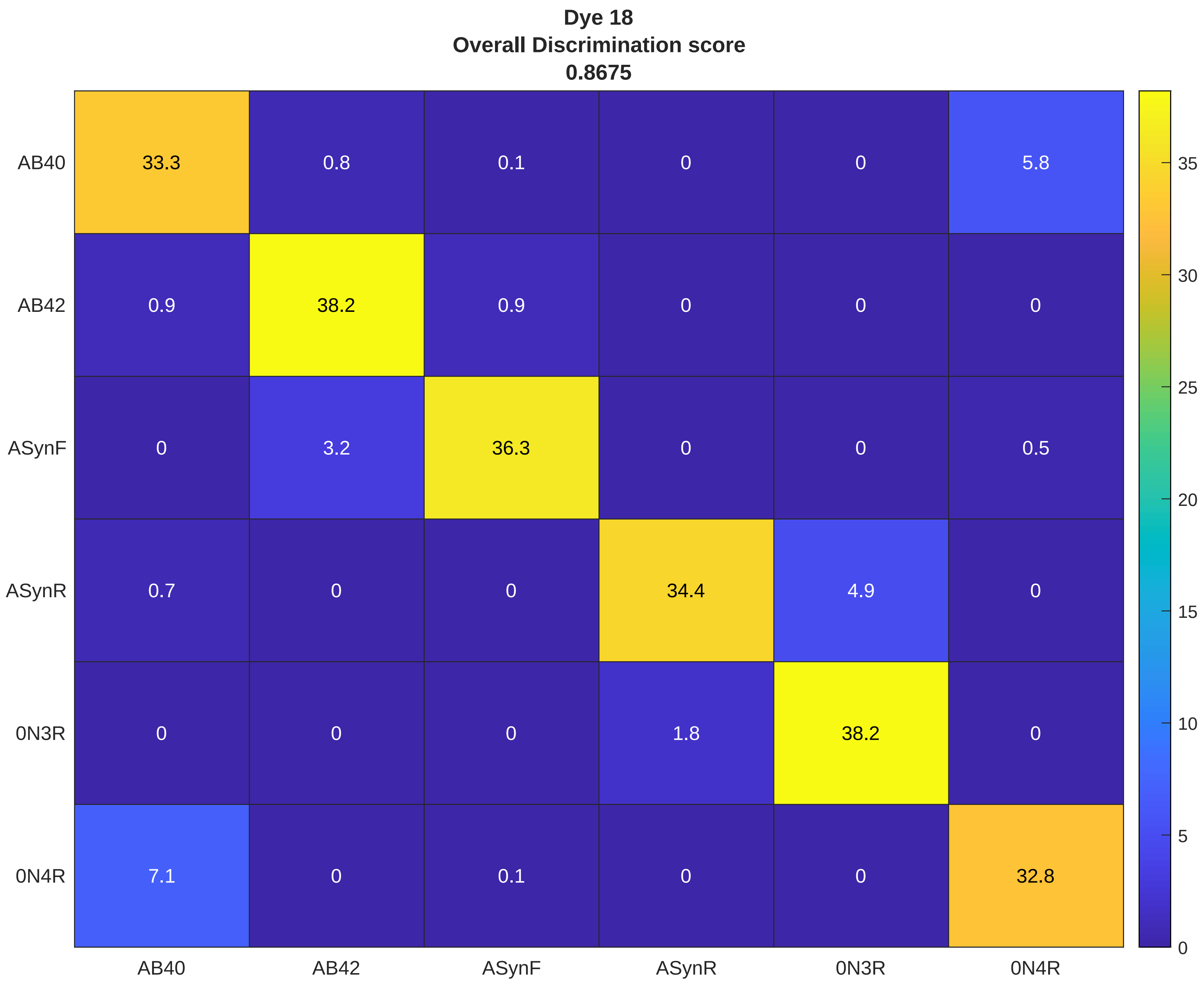
Dye 18
Overall Discrimination score
0.8675
AB40
AB42
ASynF
ASynR
0N3R
0N4R
33.3
0.8
0.1
0
0
5.8
0.9
38.2
0.9
0
0
0
0
3.2
36.3
0
0
0.5
0.7
0
0
34.4
4.9
0
0
0
0
1.8
38.2
0
7.1
0
0.1
0
0
32.8
AB40
AB42
ASynF
ASynR
0N3R
0N4R
35
30
25
20
15
10
5
0

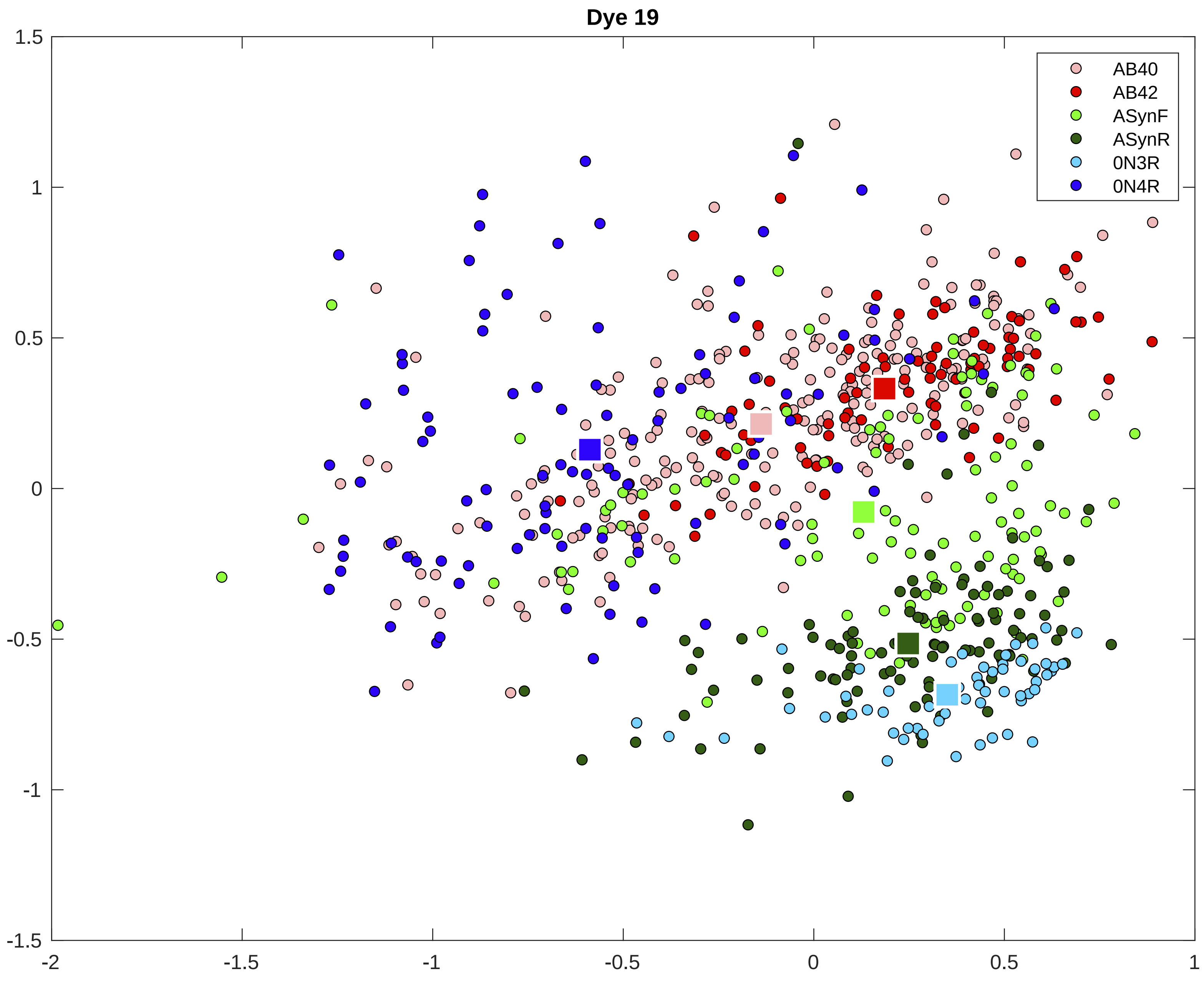
Dye 19
1.5
1
0.5
0
-0.5
-1
-1.5
-2
-1.5
-1
-0.5
0
0.5
1
AB40
AB42
ASynF
ASynR
0N3R
0N4R

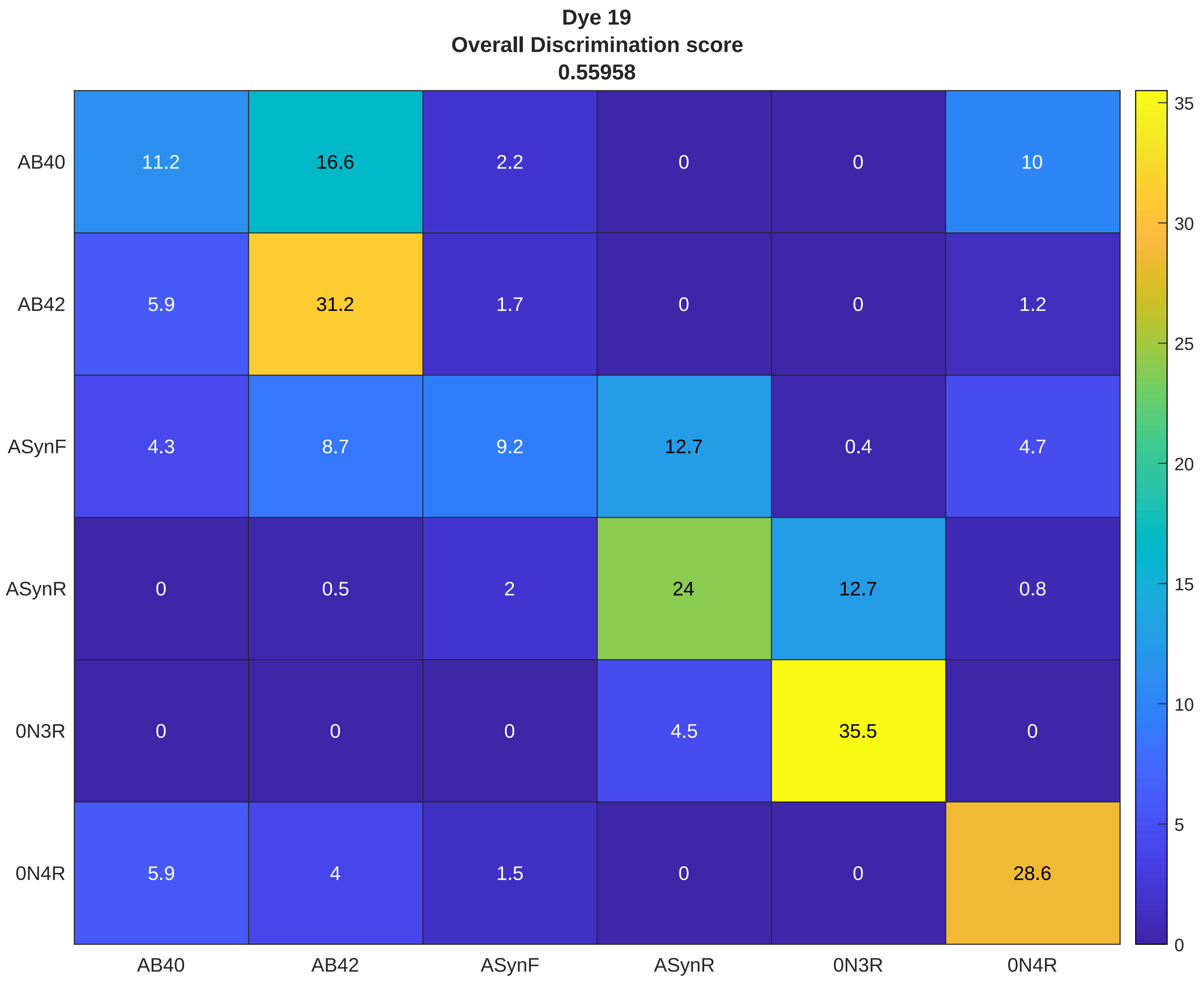

Dye 19
Overall Discrimination score
0.55958
AB40
AB42
ASynF
ASynR
0N3R
0N4R
11.2
16.6
2.2
0
0
10
5.9
31.2
1.7
0
0
1.2
4.3
8.7
9.2
12.7
0.4
4.7
0
0.5
2
24
12.7
0.8
0
0
0
4.5
35.5
0
5.9
4
1.5
0
0
28.6
AB40
AB42
ASynF
ASynR
0N3R
0N4R
35
30
25
20
15
10
5
0

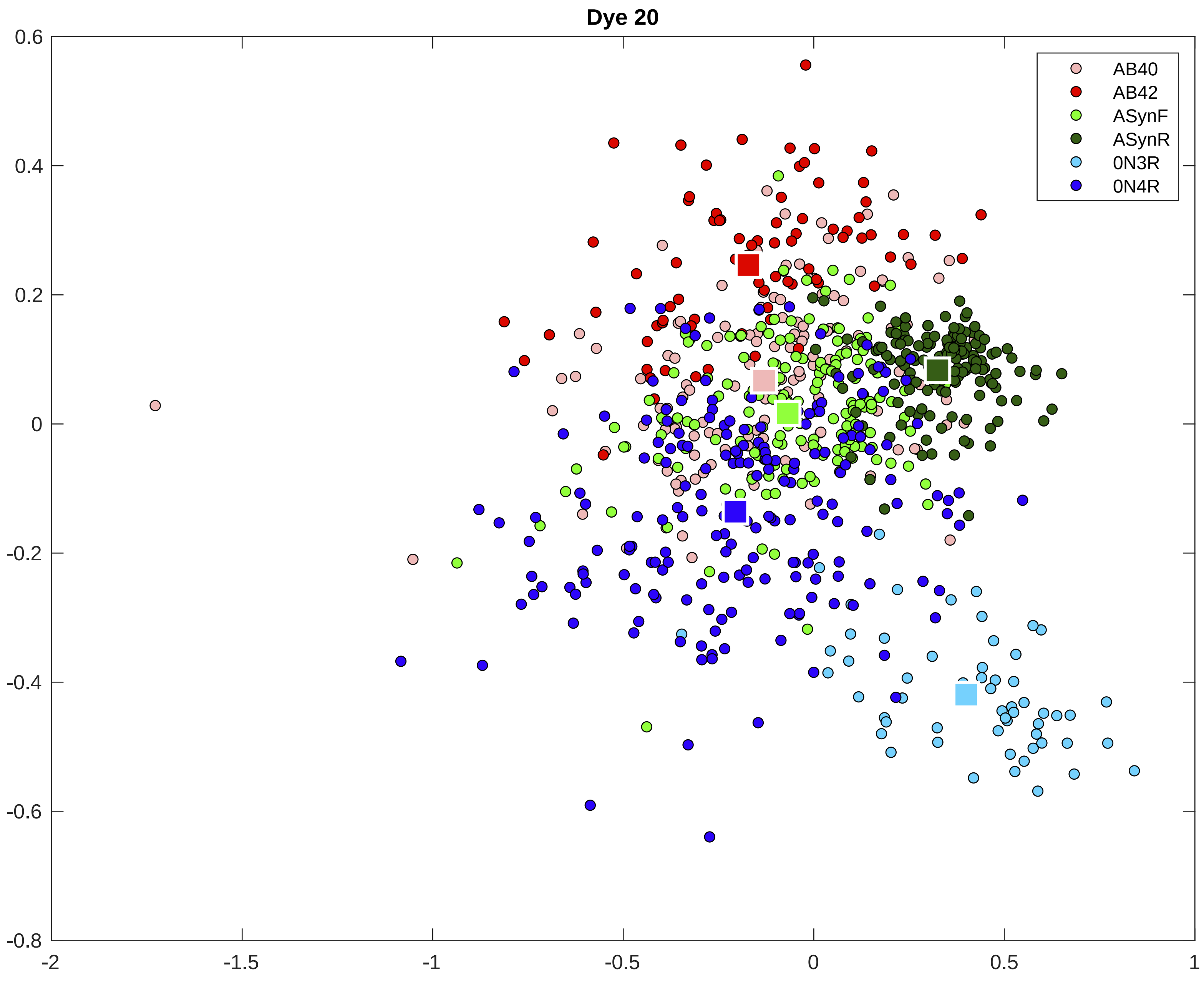

Dye 20
AB40
AB42
ASynF
ASynR
0N3R
0N4R
0.6
0.4
0.2
0
-0.2
-0.4
-0.6
-0.8
-2
-1.5
-1
-0.5
0
0.5
1

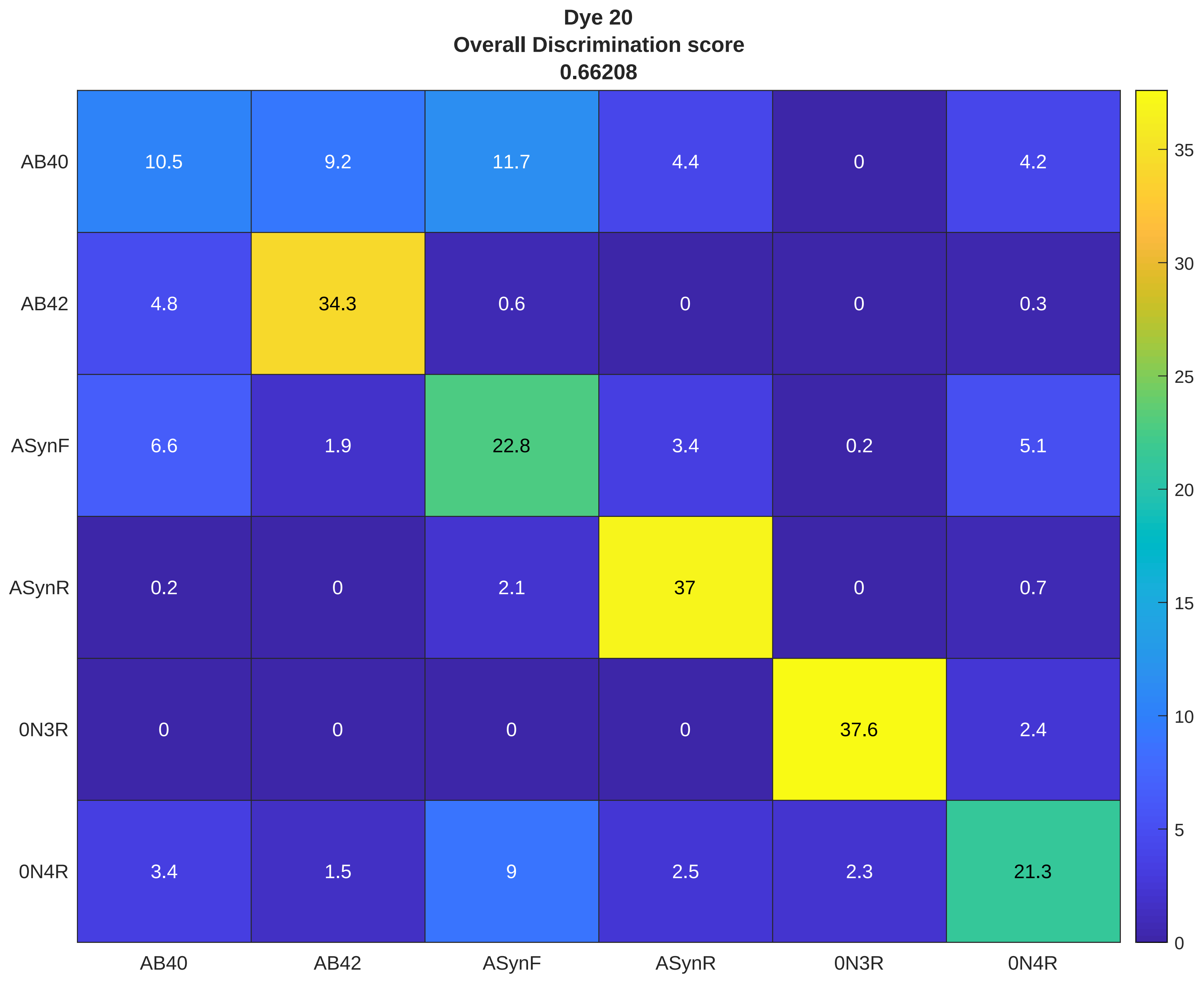
Dye 20
Overall Discrimination score
0.66208
AB40
AB42
ASynF
ASynR
0N3R
0N4R
10.5
9.2
11.7
4.4
0
4.2
4.8
34.3
0.6
0
0
0.3
6.6
1.9
22.8
3.4
0.2
5.1
0.2
0
2.1
37
0
0.7
0
0
0
0
37.6
2.4
3.4
1.5
9
2.5
2.3
21.3
0
5
10
15
20
25
30
35

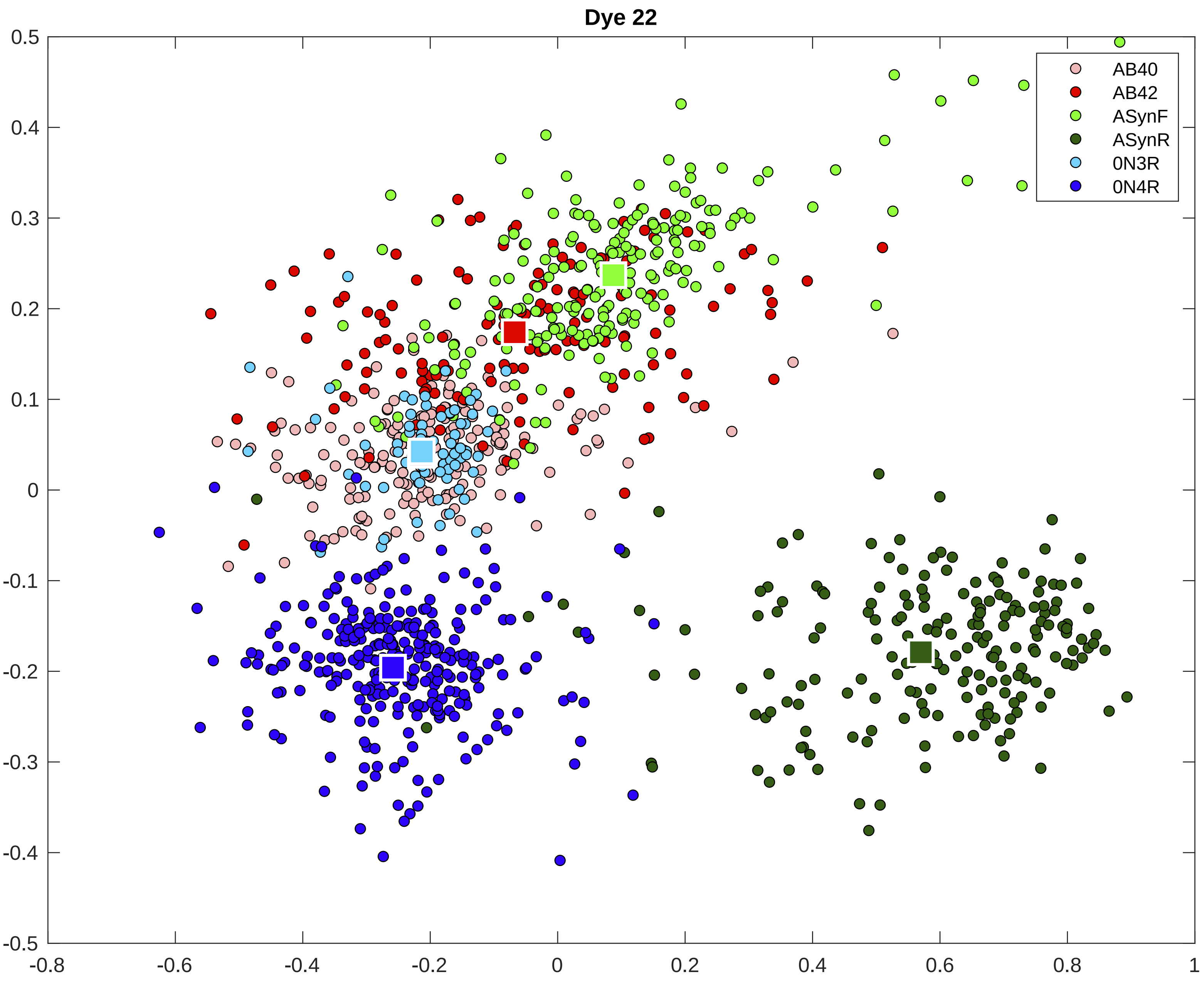

Dye 22
AB40
AB42
ASynF
ASynR
0N3R
0N4R
0.5
0.4
0.3
0.2
0.1
0
-0.1
-0.2
-0.3
-0.4
-0.5
-0.8
-0.6
-0.4
-0.2
0
0.2
0.4
0.6
0.8
1

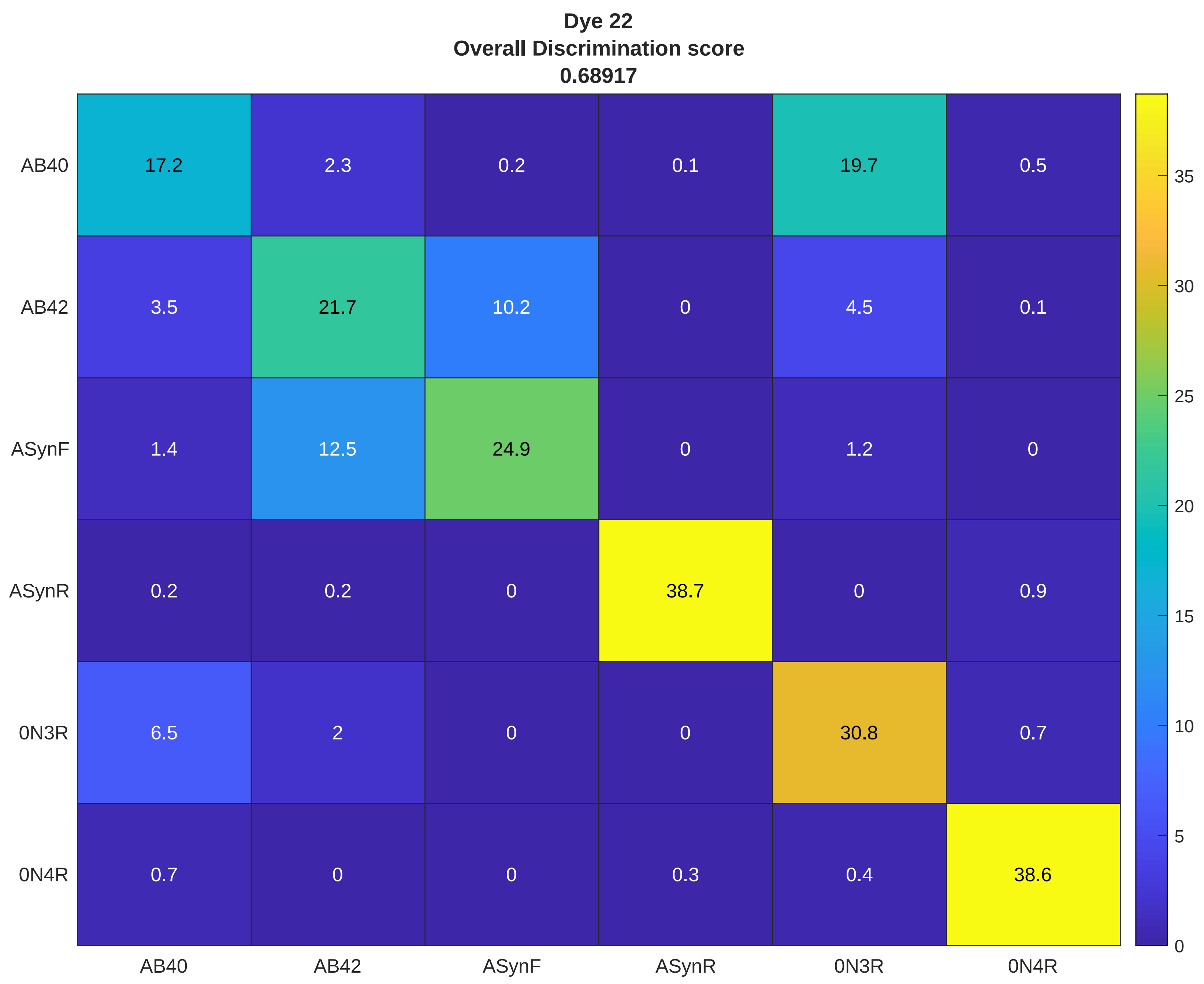
Dye 22
Overall Discrimination score
0.68917
AB40
AB42
ASynF
ASynR
0N3R
0N4R
17.2 2.3 0.2 0.1 19.7 0.5
3.5 21.7 10.2 0 4.5 0.1
1.4 12.5 24.9 0 1.2 0
0.2 0.2 0 38.7 0 0.9
6.5 2 0 0 30.8 0.7
0.7 0 0 0.3 0.4 38.6
AB40
AB42
ASynF
ASynR
0N3R
0N4R
35
30
25
20
15
10
5
0

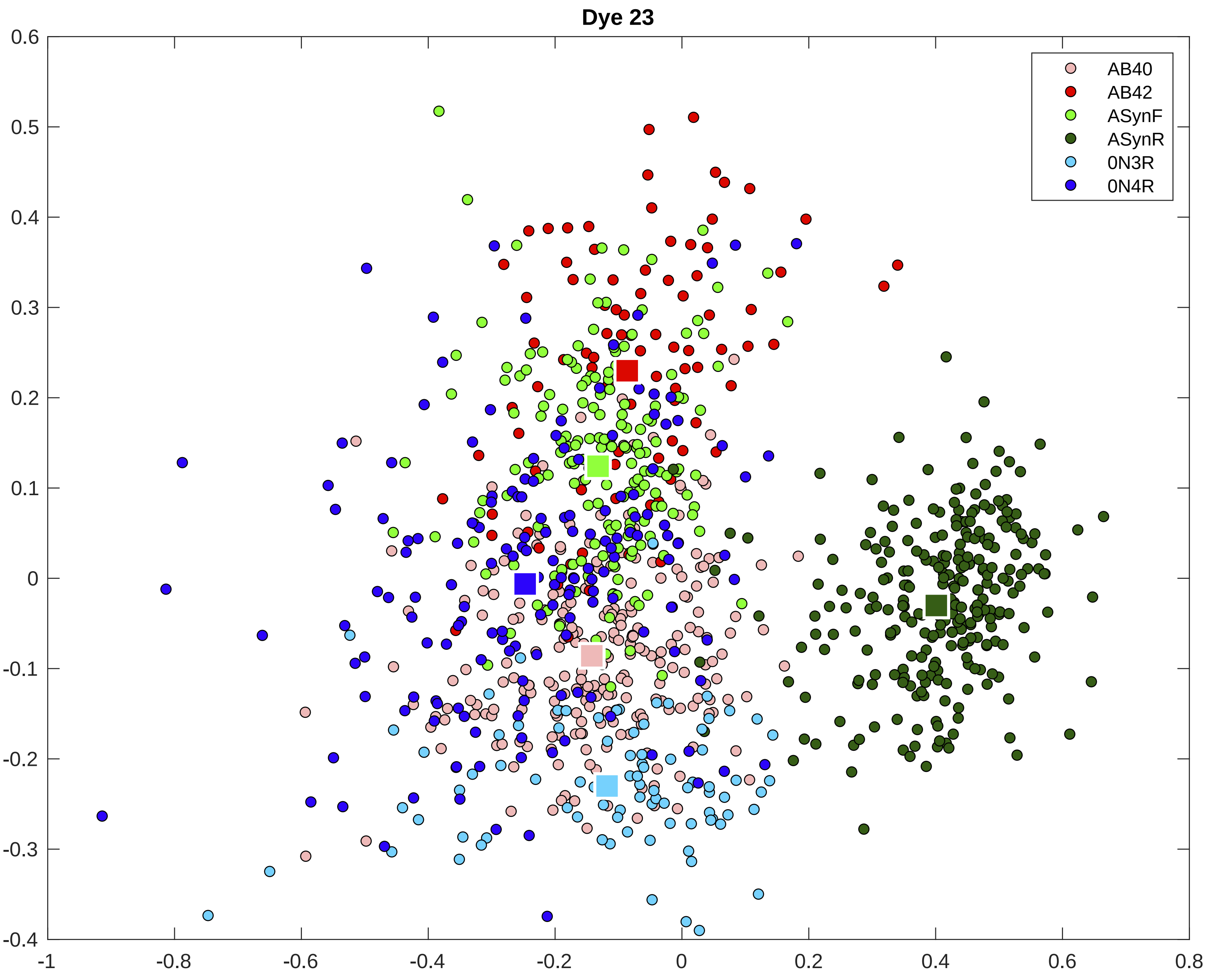

Dye 23
AB40
AB42
ASynF
ASynR
0N3R
0N4R
0.6
0.5
0.4
0.3
0.2
0.1
0
-0.1
-0.2
-0.3
-0.4
-1
-0.8
-0.6
-0.4
-0.2
0
0.2
0.4
0.6
0.8

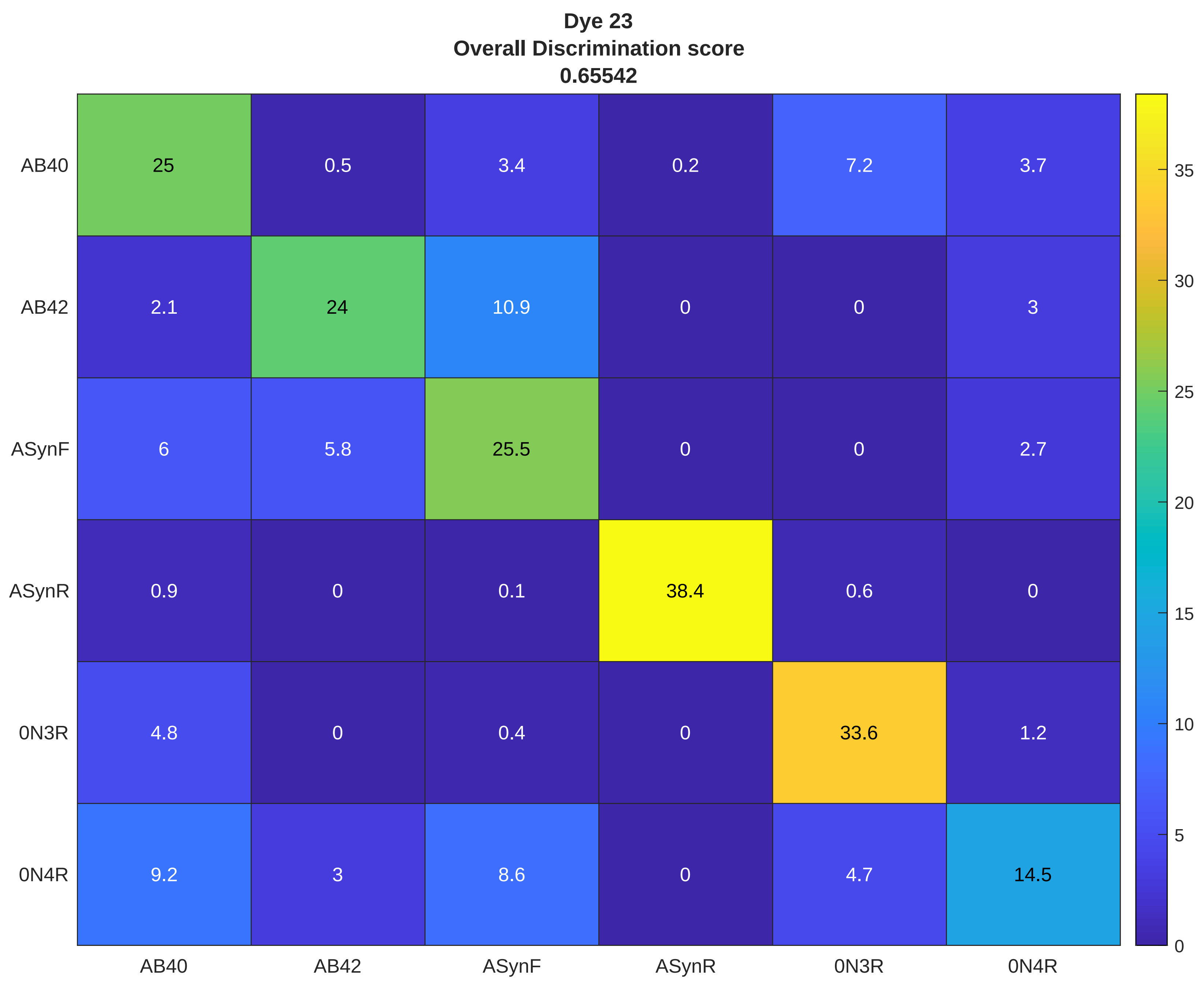

Dye 23
Overall Discrimination score
0.65542
AB40
AB42
ASynF
ASynR
0N3R
0N4R
25
0.5
3.4
0.2
7.2
3.7
2.1
24
10.9
0
0
3
6
5.8
25.5
0
0
2.7
0.9
0
0.1
38.4
0.6
0
4.8
0
0.4
0
33.6
1.2
9.2
3
8.6
0
4.7
14.5
AB40
AB42
ASynF
ASynR
0N3R
0N4R
35
30
25
20
15
10
5
0

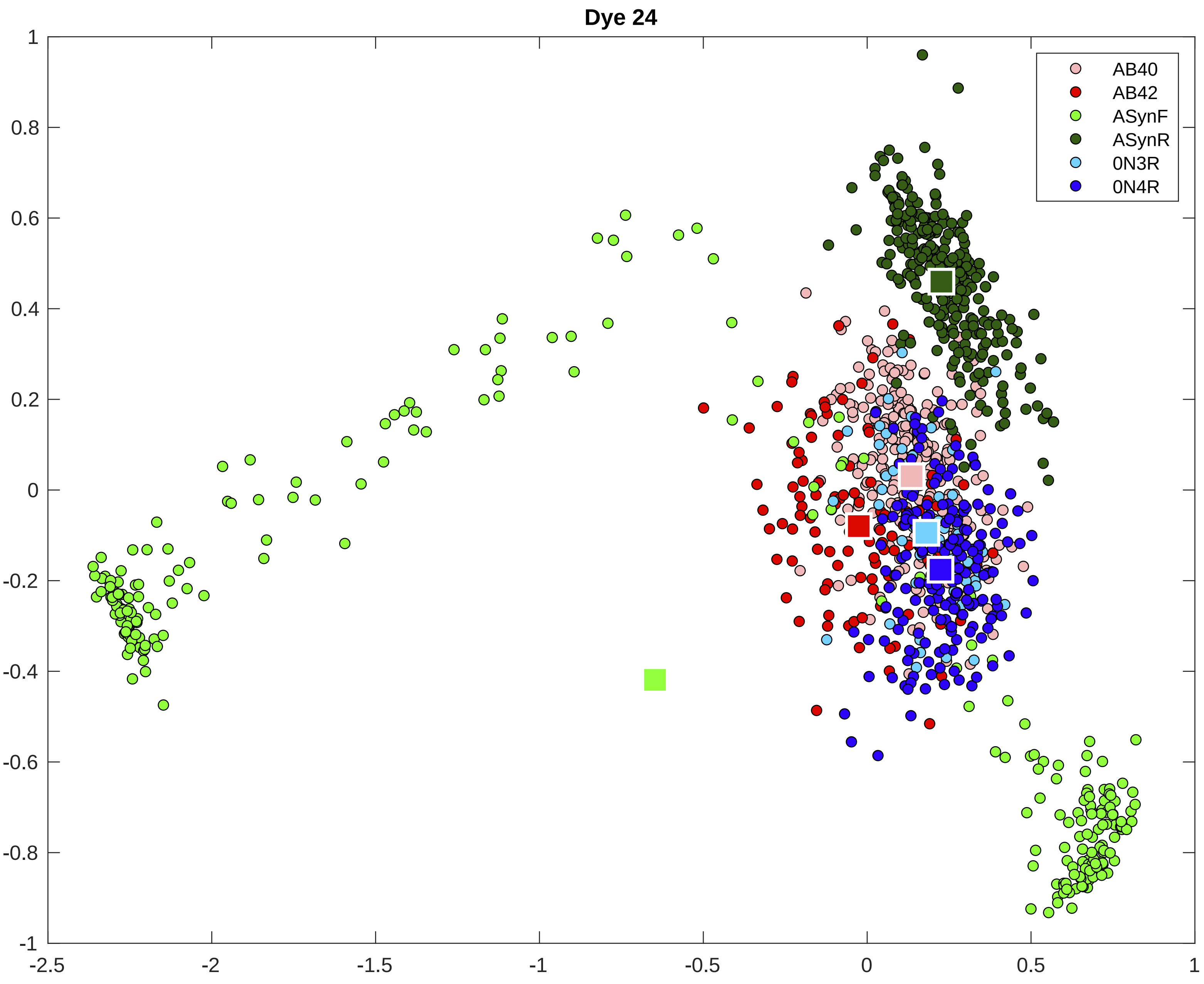

Dye 24
AB40
AB42
ASynF
ASynR
0N3R
0N4R
1
0.8
0.6
0.4
0.2
0
-0.2
-0.4
-0.6
-0.8
-1
-2.5
-2
-1.5
-1
-0.5
0
0.5
1

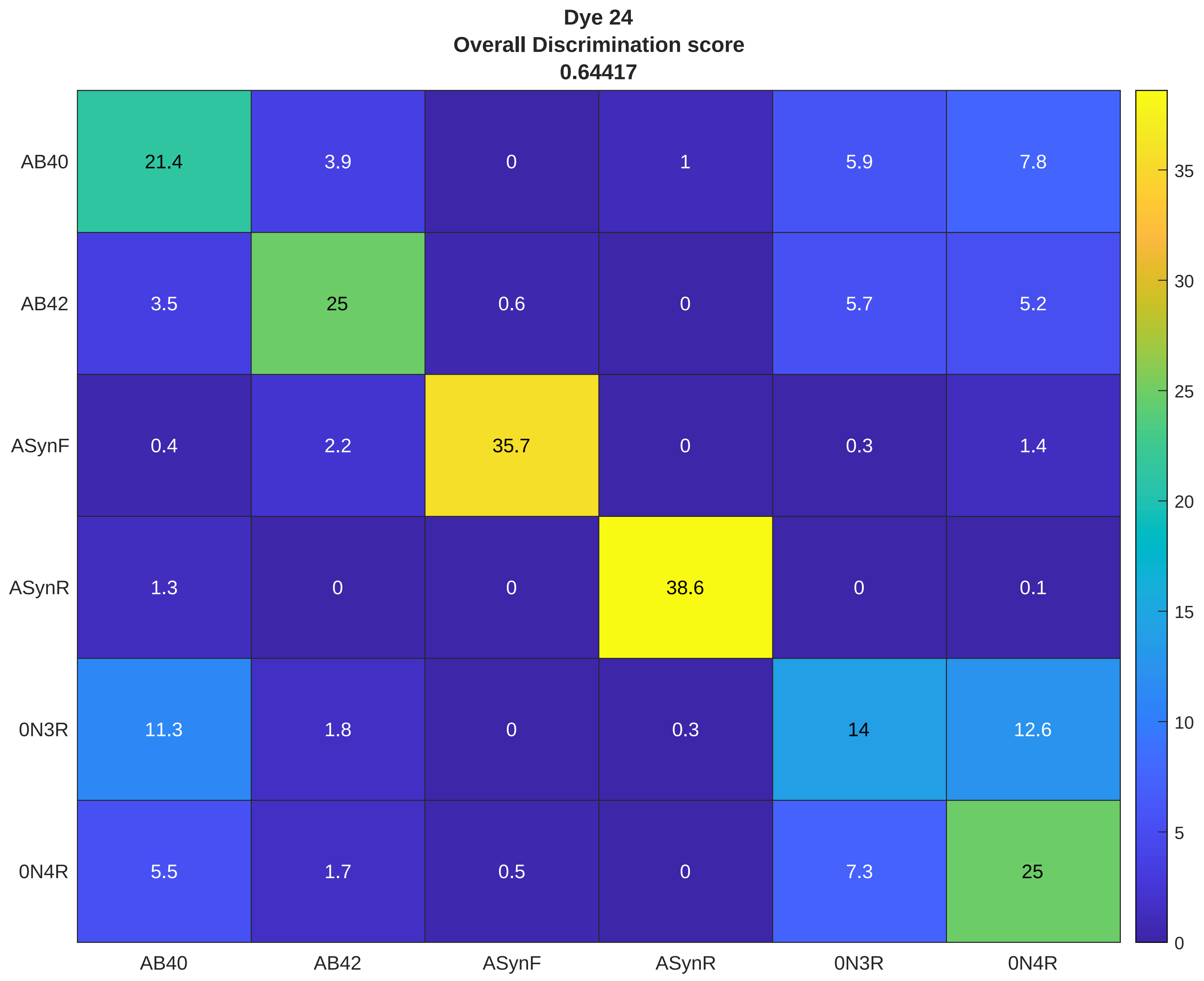
Dye 24
Overall Discrimination score
0.64417
AB40
AB42
ASynF
ASynR
0N3R
0N4R
21.4 3.9 0 1 5.9 7.8
3.5 25 0.6 0 5.7 5.2
0.4 2.2 35.7 0 0.3 1.4
1.3 0 0 38.6 0 0.1
11.3 1.8 0 0.3 14 12.6
5.5 1.7 0.5 0 7.3 25
0
5
10
15
20
25
30
35

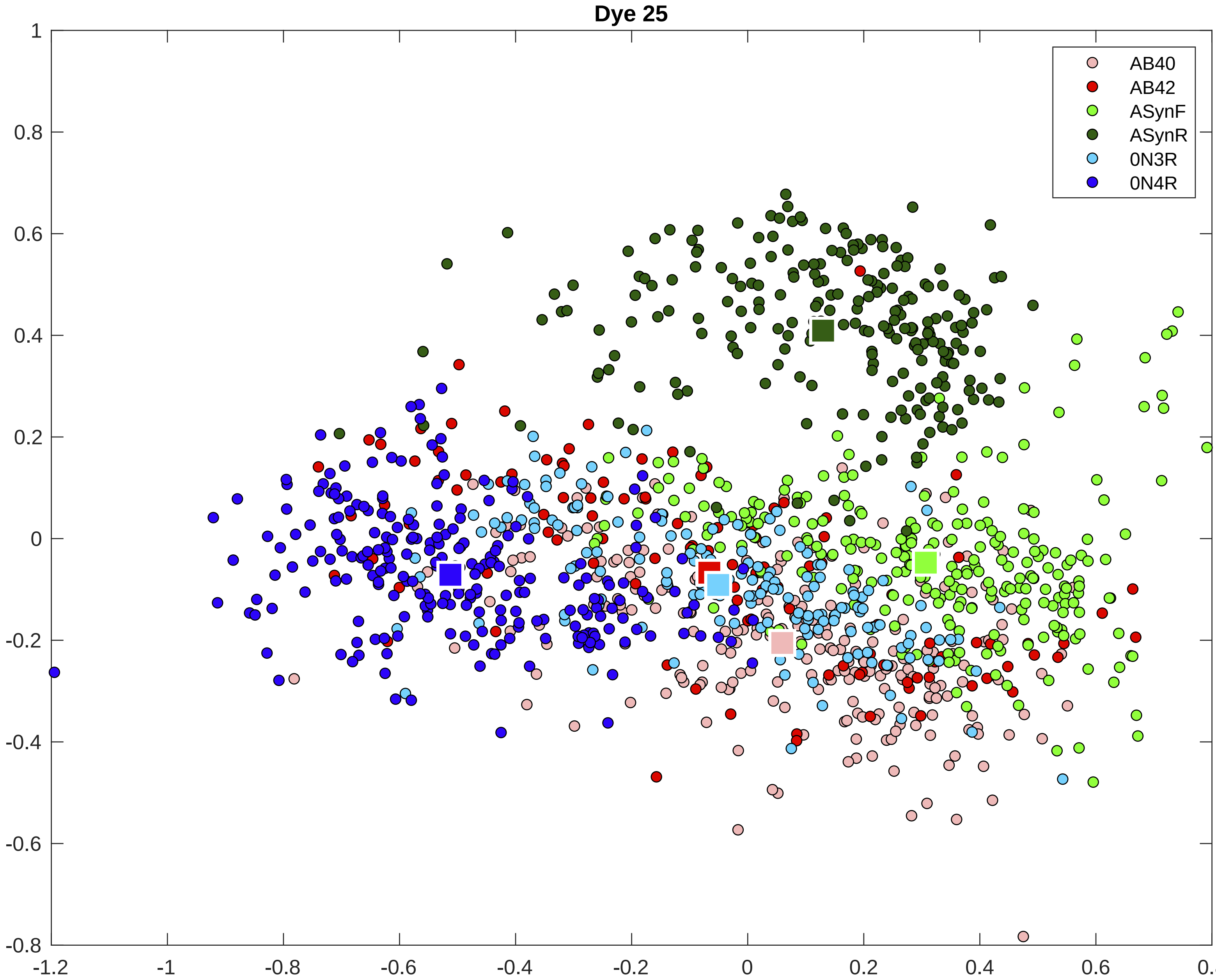

Dye 25
AB40
AB42
ASynF
ASynR
0N3R
0N4R
1
0.8
0.6
0.4
0.2
0
-0.2
-0.4
-0.6
-0.8
-1.2
-1
-0.8
-0.6
-0.4
-0.2
0
0.2
0.4
0.6
0.8

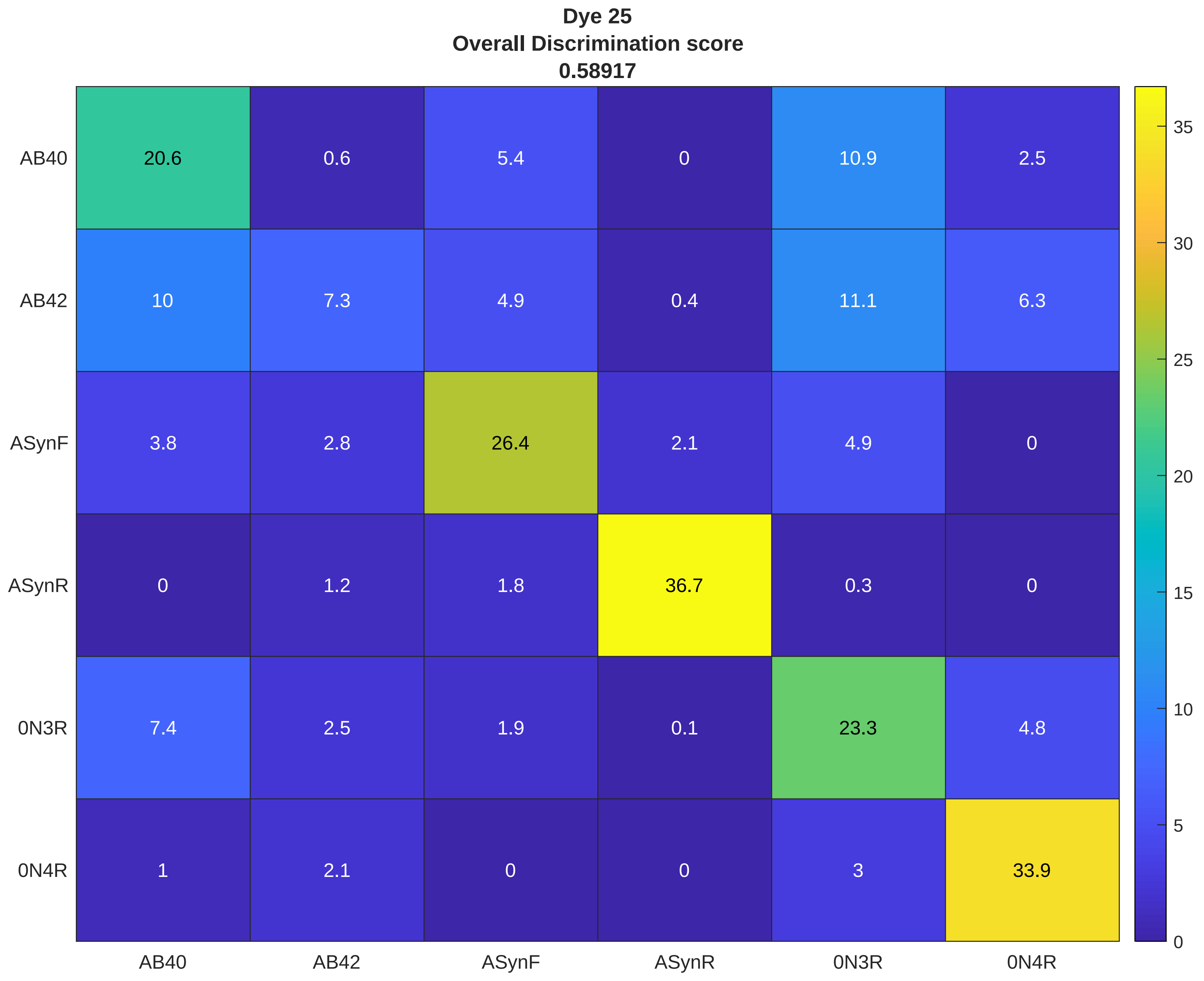

Dye 25
Overall Discrimination score
0.58917
AB40
AB42
ASynF
ASynR
0N3R
0N4R
20.6
0.6
5.4
0
10.9
2.5
10
7.3
4.9
0.4
11.1
6.3
3.8
2.8
26.4
2.1
4.9
0
0
1.2
1.8
36.7
0.3
0
7.4
2.5
1.9
0.1
23.3
4.8
1
2.1
0
0
3
33.9
AB40
AB42
ASynF
ASynR
0N3R
0N4R
35
30
25
20
15
10
5
0

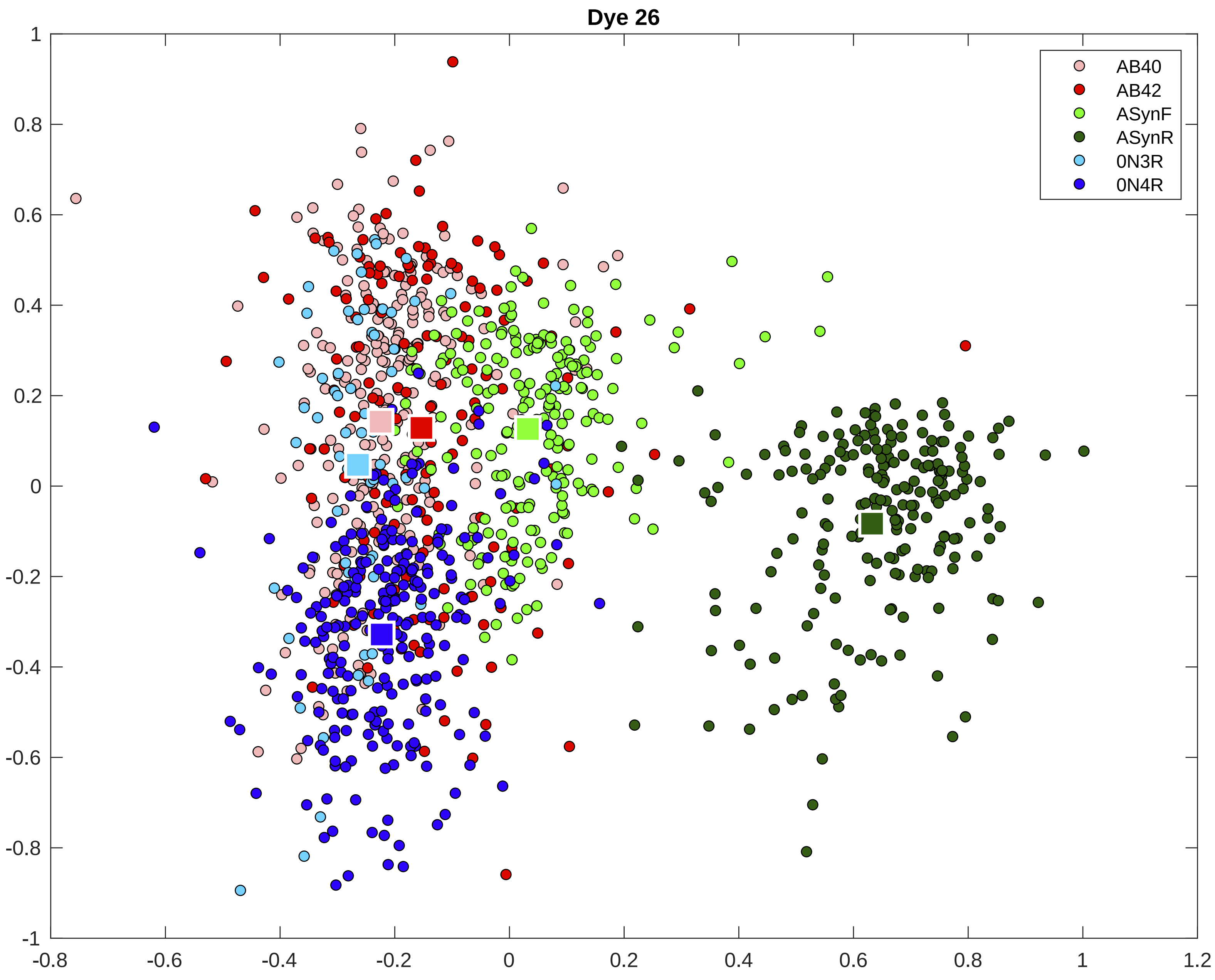

Dye 26
AB40
AB42
ASynF
ASynR
0N3R
0N4R
1
0.8
0.6
0.4
0.2
0
-0.2
-0.4
-0.6
-0.8
-1
-0.8
-0.6
-0.4
-0.2
0
0.2
0.4
0.6
0.8
1
1.2

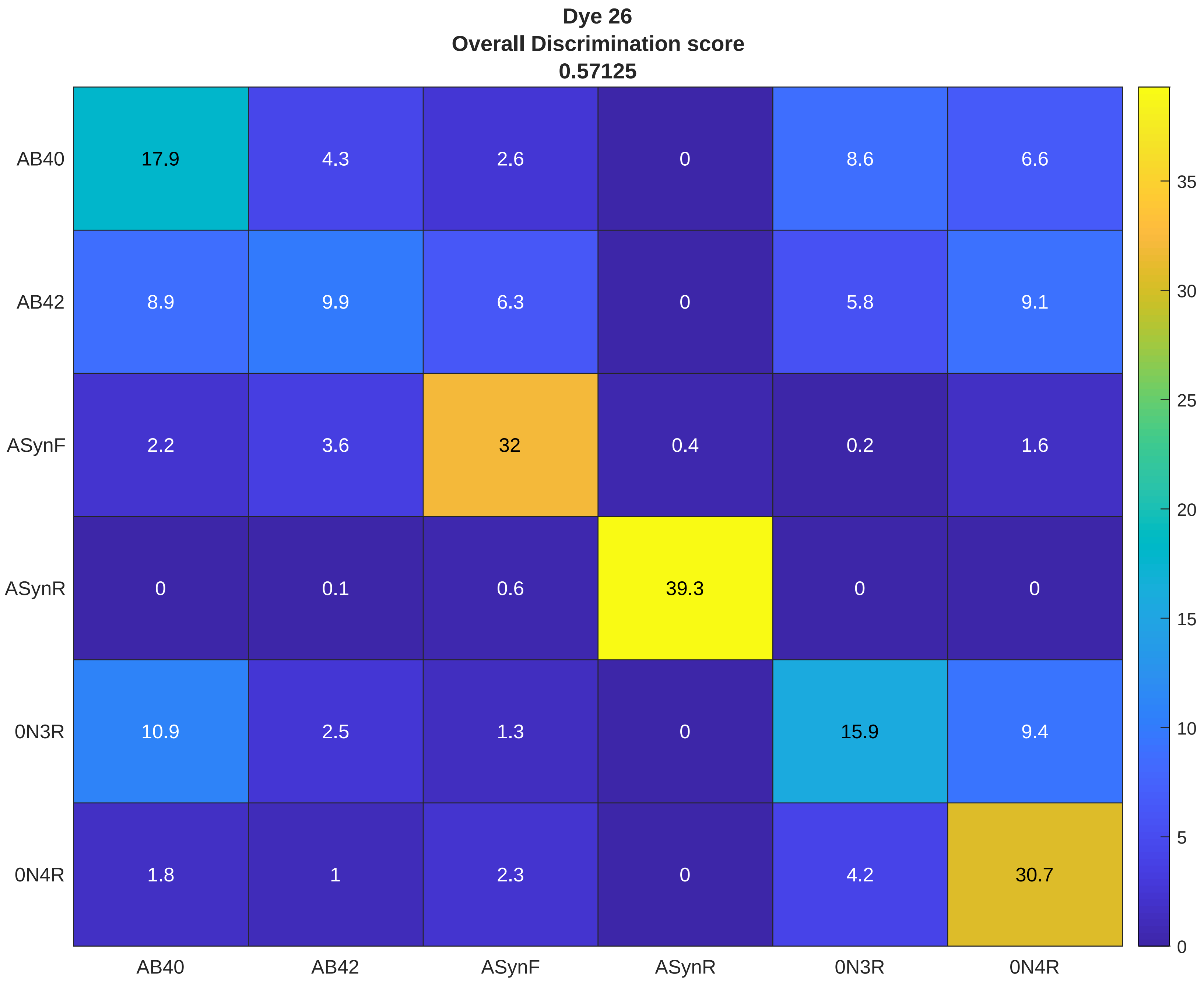
Dye 26
Overall Discrimination score
0.57125
AB40
AB42
ASynF
ASynR
0N3R
0N4R
17.9 4.3 2.6 0 8.6 6.6
8.9 9.9 6.3 0 5.8 9.1
2.2 3.6 32 0.4 0.2 1.6
0 0.1 0.6 39.3 0 0
10.9 2.5 1.3 0 15.9 9.4
1.8 1 2.3 0 4.2 30.7
AB40
AB42
ASynF
ASynR
0N3R
0N4R
35
30
25
20
15
10
5
0

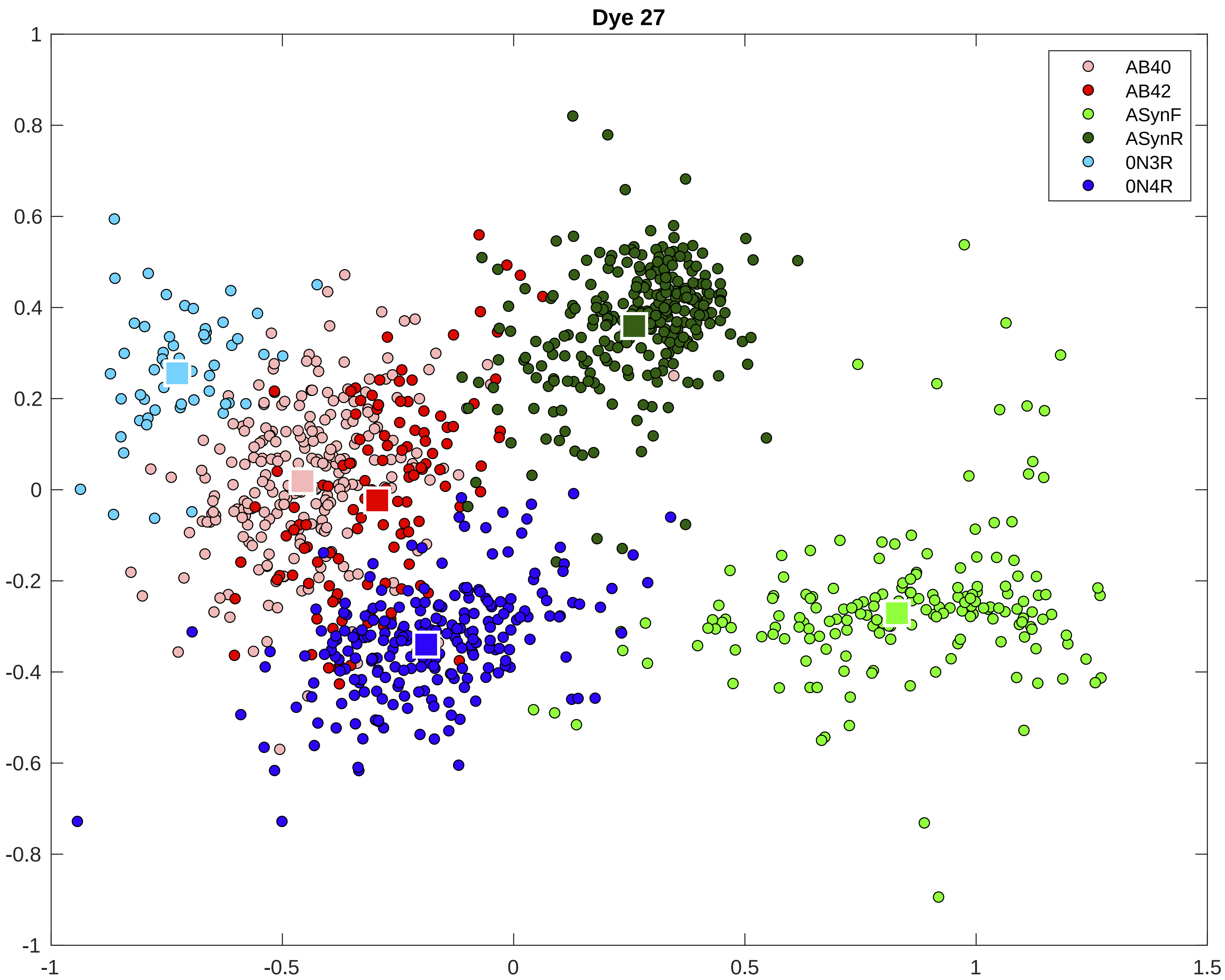
Dye 27
AB40
AB42
ASynF
ASynR
0N3R
0N4R
1
0.8
0.6
0.4
0.2
0
-0.2
-0.4
-0.6
-0.8
-1
-1
-0.5
0
0.5
1
1.5

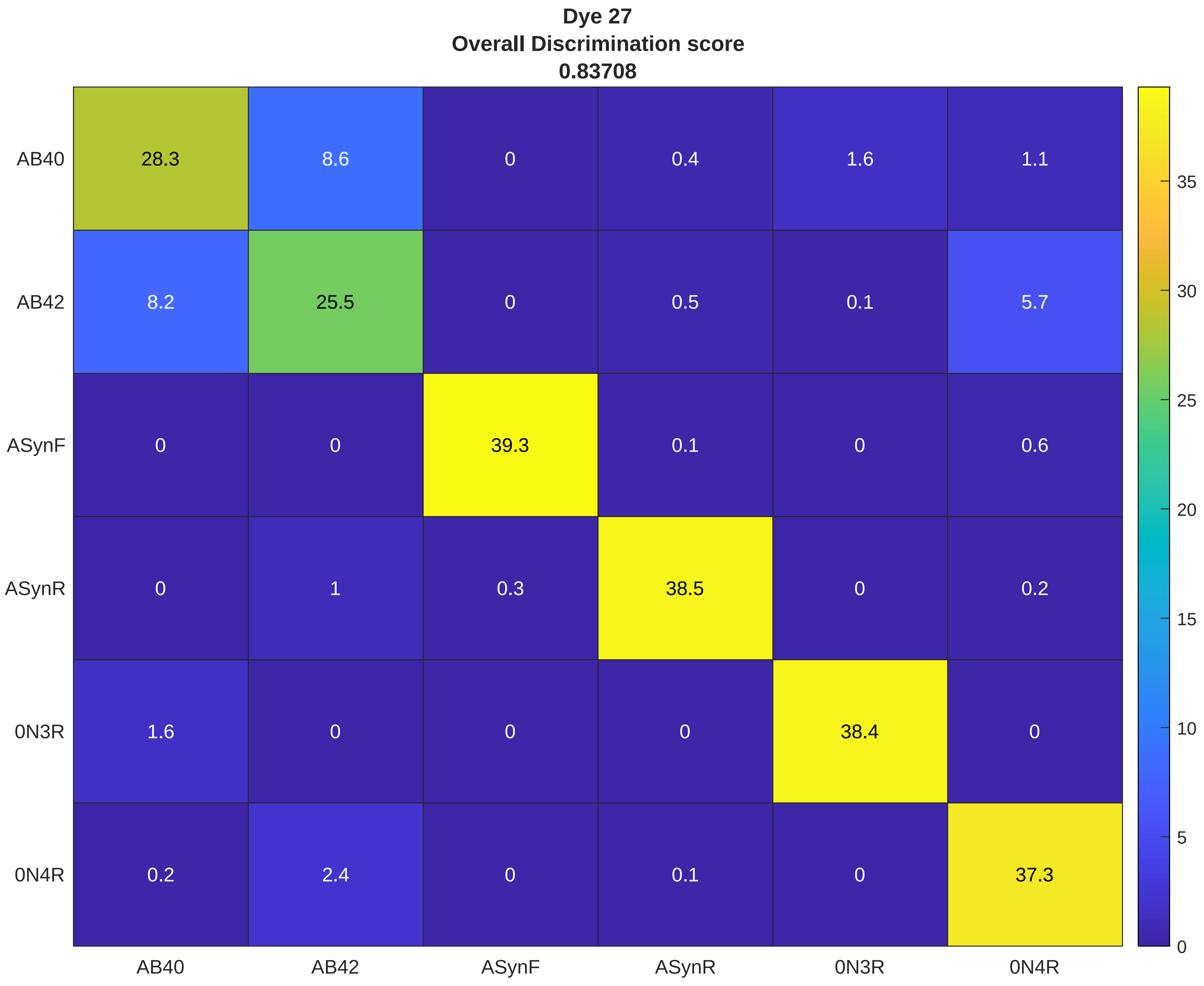
Dye 27
Overall Discrimination score
0.83708
AB40
AB42
ASynF
ASynR
0N3R
0N4R
28.3
8.6
0
0.4
1.6
1.1
8.2
25.5
0
0.5
0.1
5.7
0
0
39.3
0.1
0
0.6
0
1
0.3
38.5
0
0.2
1.6
0
0
0
38.4
0
0.2
2.4
0
0.1
0
37.3
AB40
AB42
ASynF
ASynR
0N3R
0N4R
35
30
25
20
15
10
5
0

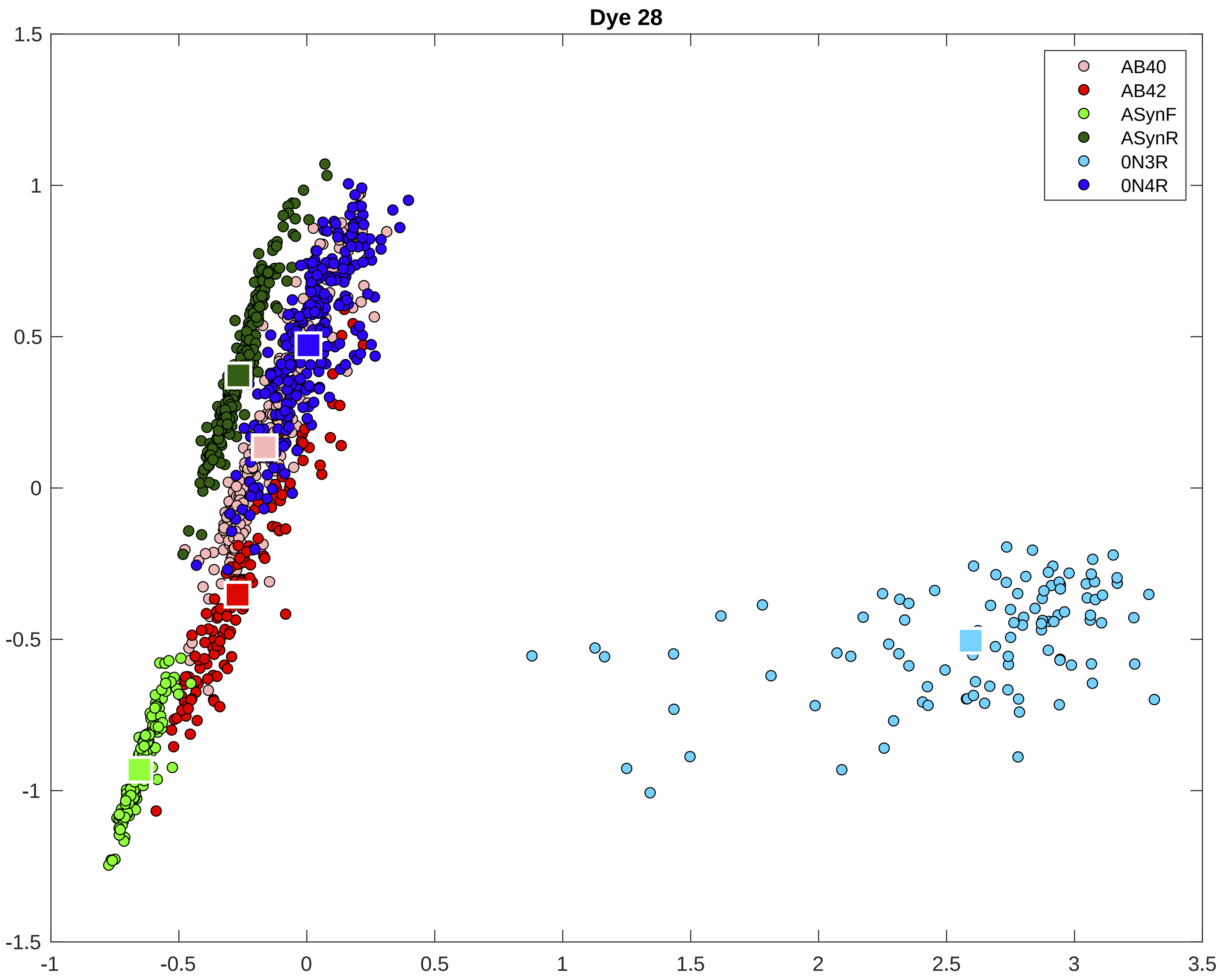
Dye 28
AB40
AB42
ASynF
ASynR
0N3R
0N4R
1.5
1
0.5
0
-0.5
-1
-1.5
-1
-0.5
0
0.5
1
1.5
2
2.5
3
3.5

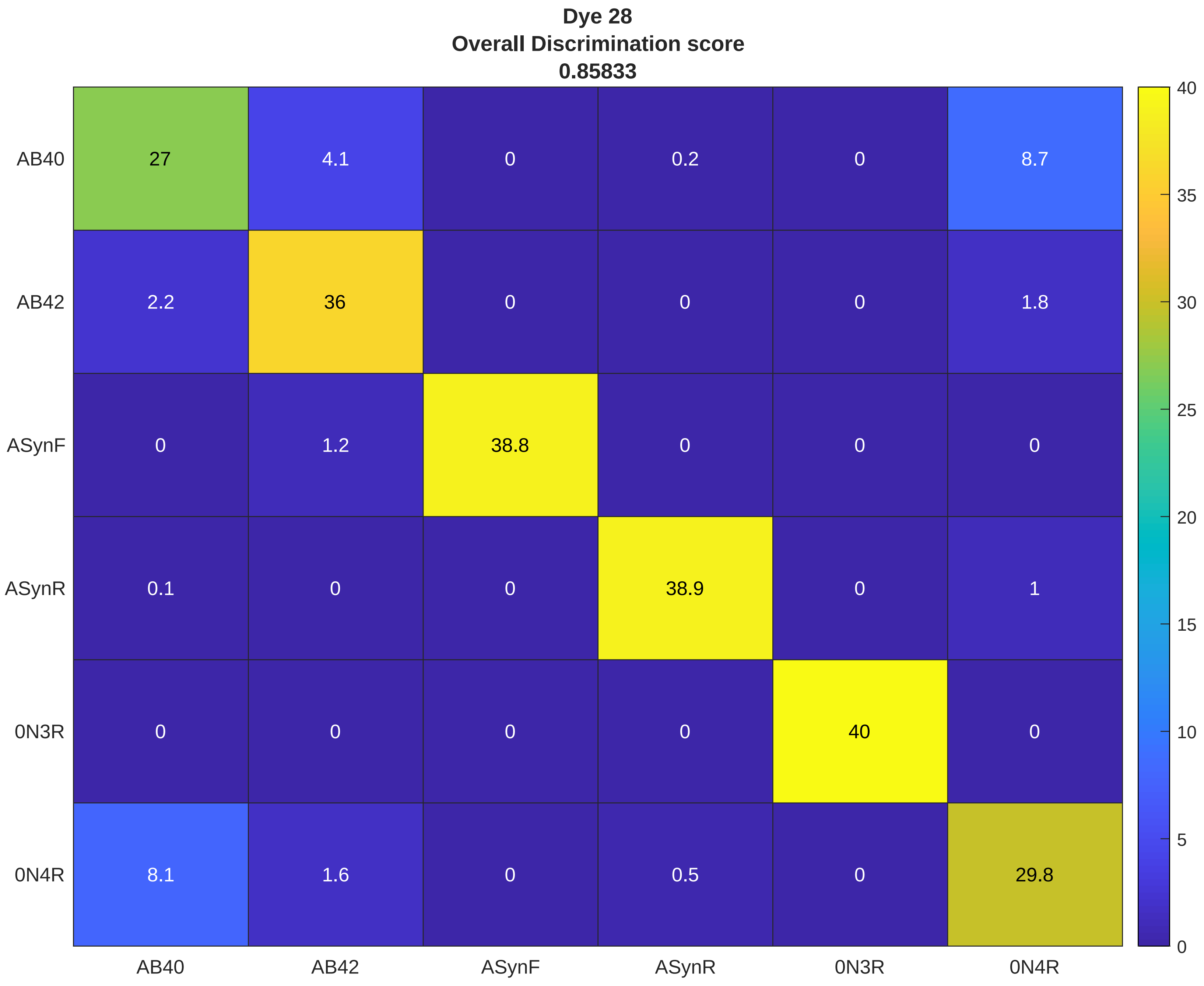
Dye 28
Overall Discrimination score
0.85833
AB40
AB42
ASynF
ASynR
0N3R
0N4R
27
4.1
0
0.2
0
8.7
2.2
36
0
0
0
1.8
0
1.2
38.8
0
0
0
0.1
0
0
38.9
0
1
0
0
0
0
40
0
8.1
1.6
0
0.5
0
29.8
AB40
AB42
ASynF
ASynR
0N3R
0N4R
40
35
30
25
20
15
10
5
0

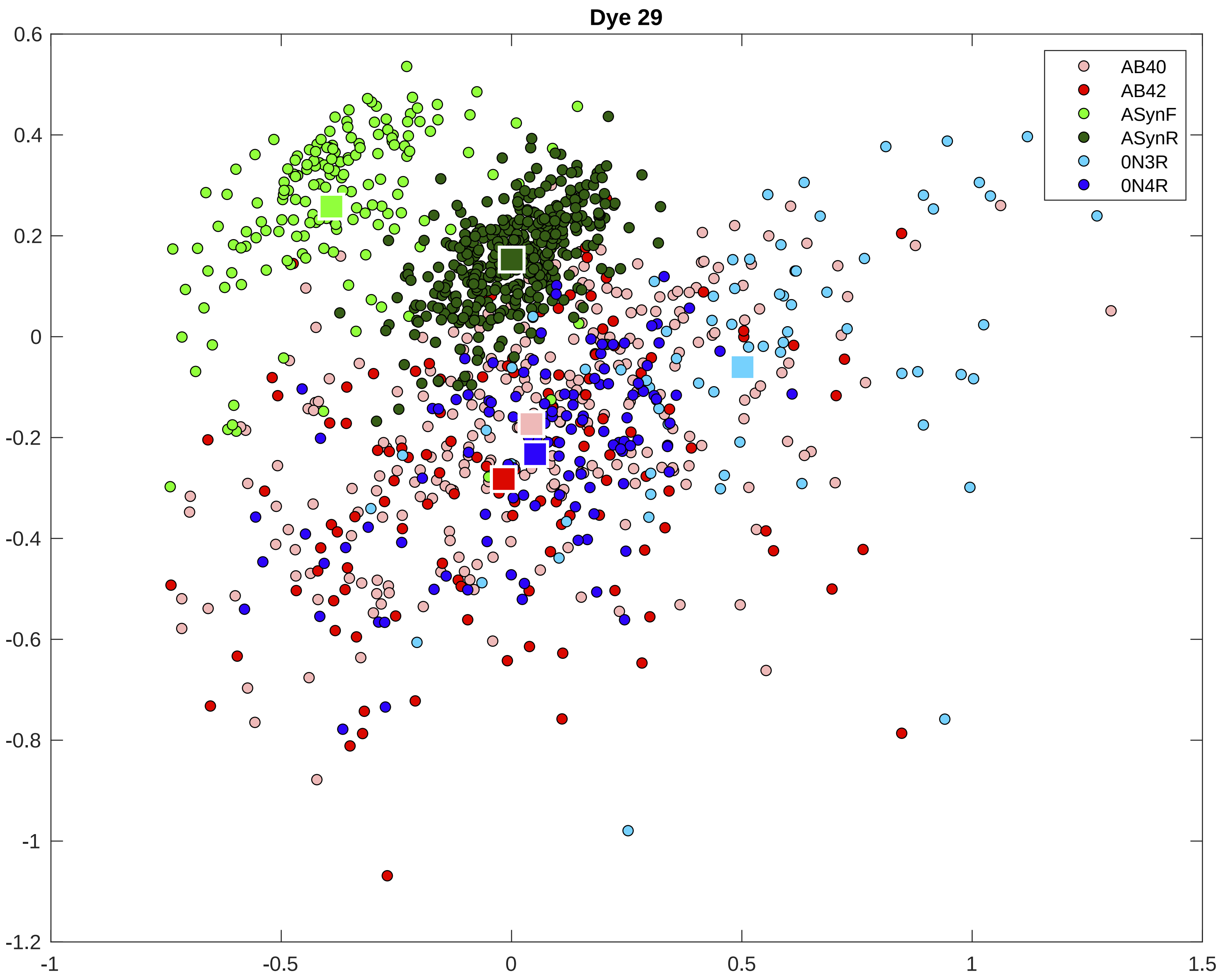
Dye 29
AB40
AB42
ASynF
ASynR
0N3R
0N4R
0.6
0.4
0.2
0
-0.2
-0.4
-0.6
-0.8
-1
-1.2
-1
-0.5
0
0.5
1
1.5

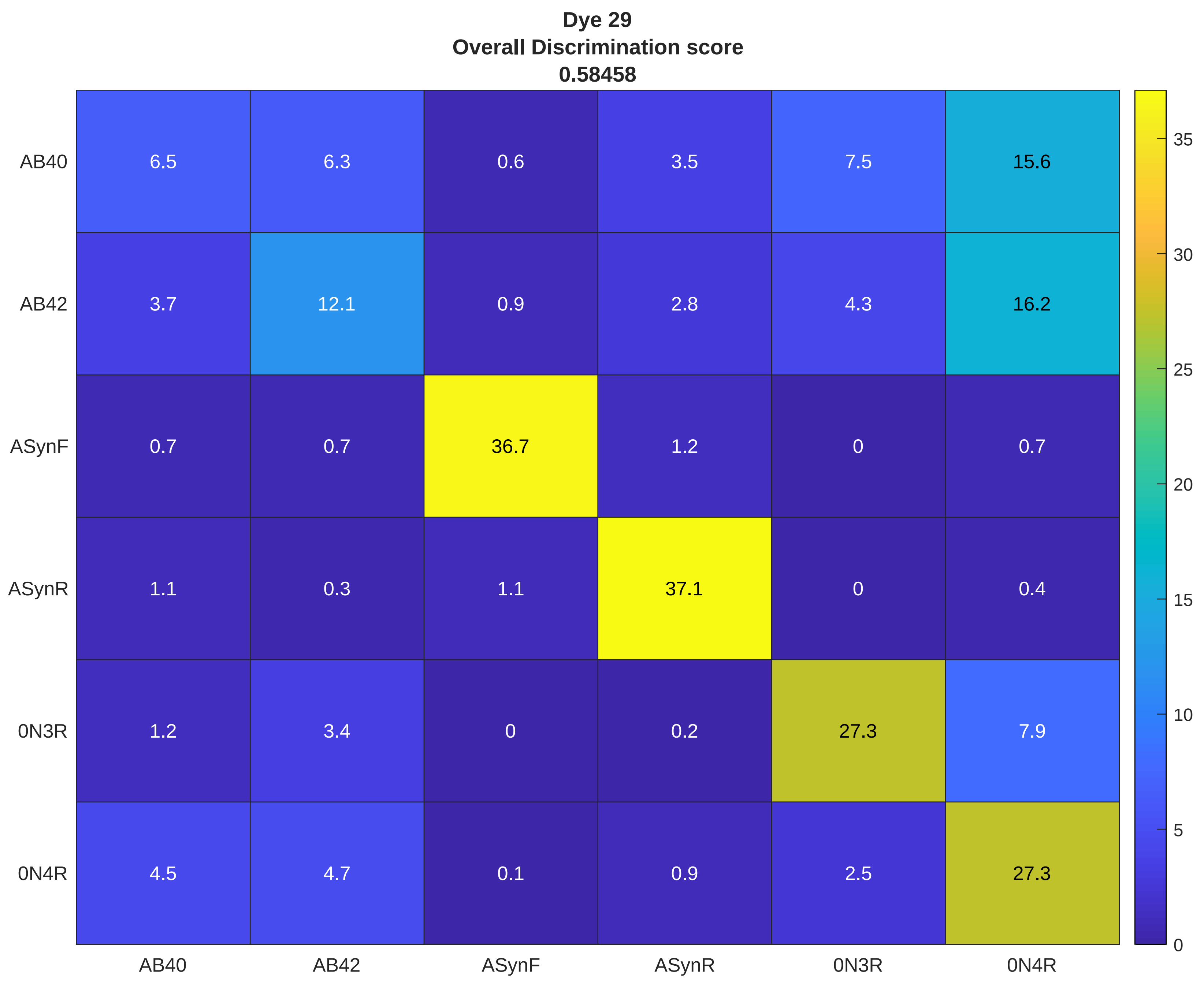
Dye 29
Overall Discrimination score
0.58458
AB40
AB42
ASynF
ASynR
0N3R
0N4R
6.5
6.3
0.6
3.5
7.5
15.6
3.7
12.1
0.9
2.8
4.3
16.2
0.7
0.7
36.7
1.2
0
0.7
1.1
0.3
1.1
37.1
0
0.4
1.2
3.4
0
0.2
27.3
7.9
4.5
4.7
0.1
0.9
2.5
27.3
AB40
AB42
ASynF
ASynR
0N3R
0N4R
35
30
25
20
15
10
5
0

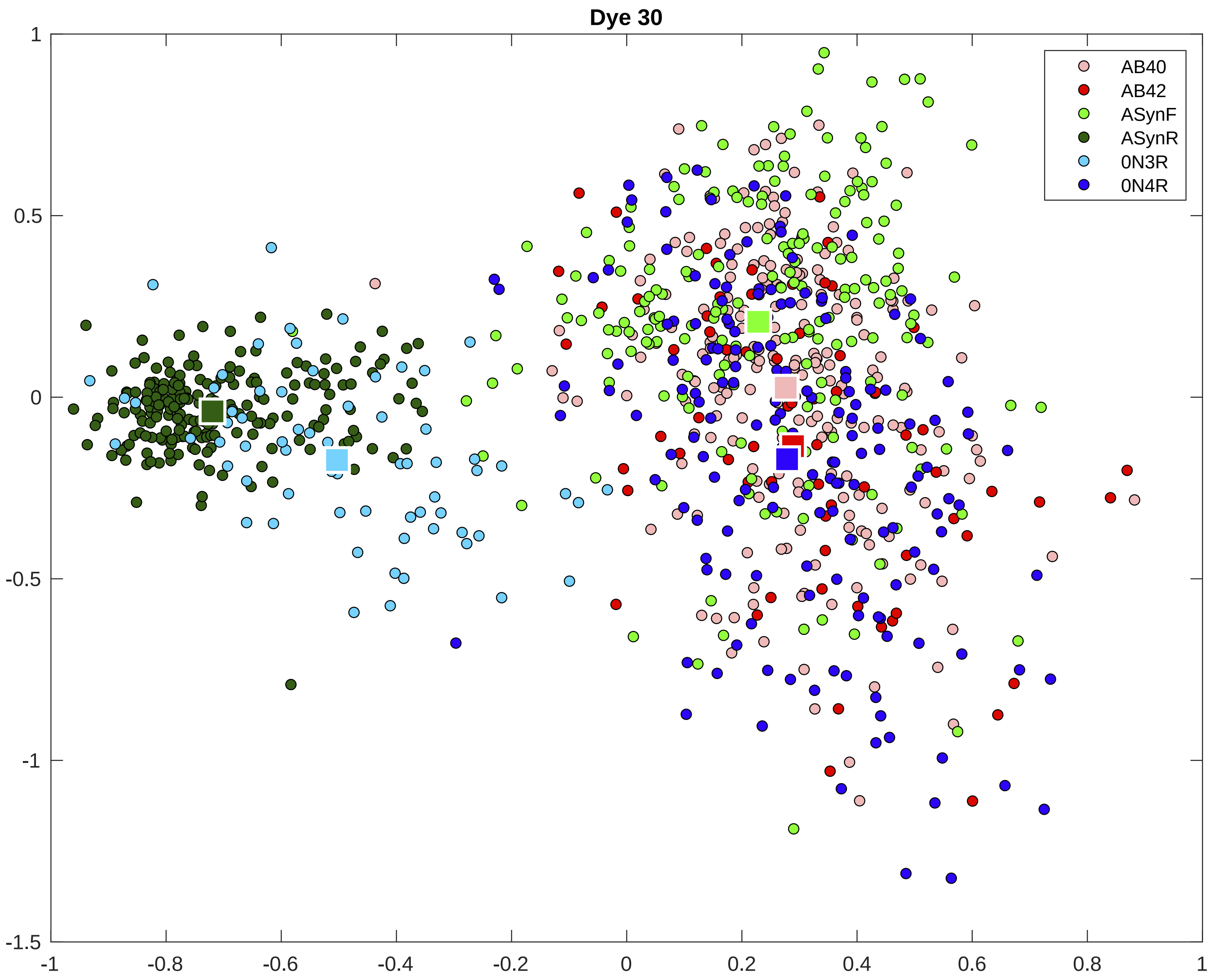
Dye 30
AB40
AB42
ASynF
ASynR
0N3R
0N4R
1
0.5
0
-0.5
-1
-1.5
-1
-0.8
-0.6
-0.4
-0.2
0
0.2
0.4
0.6
0.8
1

Dye 30
Overall Discrimination score
0.47583

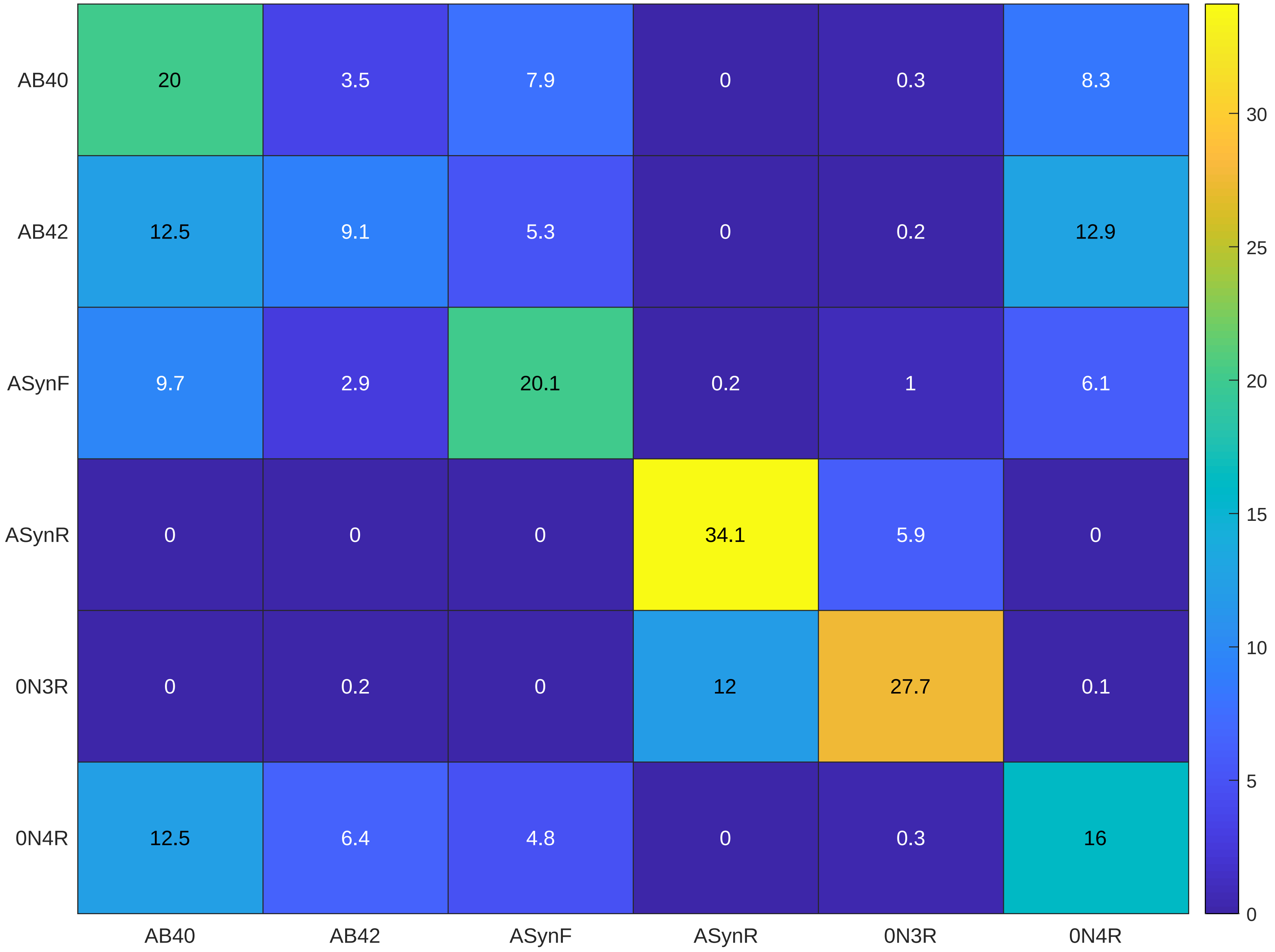

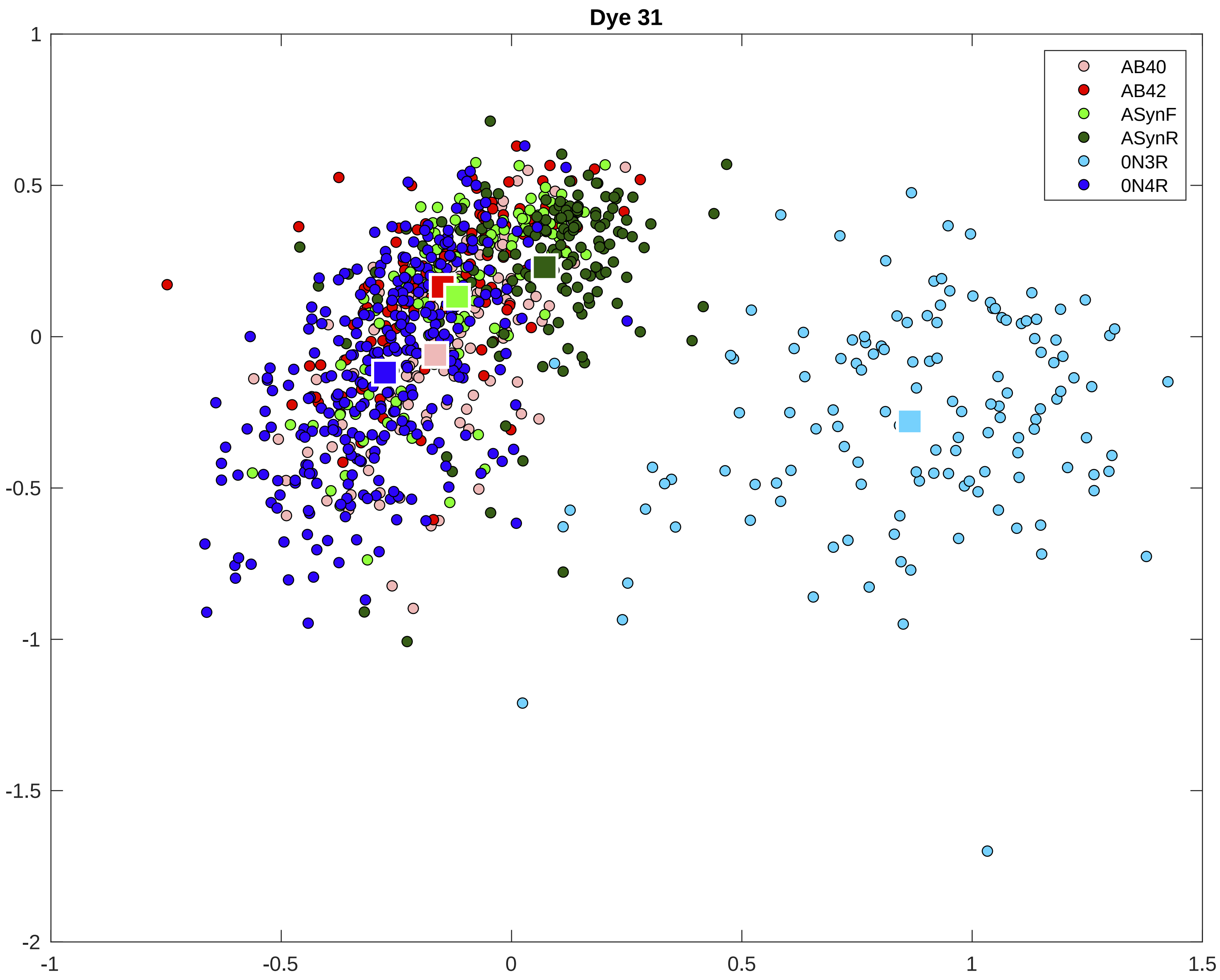

Dye 31
AB40
AB42
ASynF
ASynR
0N3R
0N4R
1
0.5
0
-0.5
-1
-1.5
-2
-1
-0.5
0
0.5
1
1.5

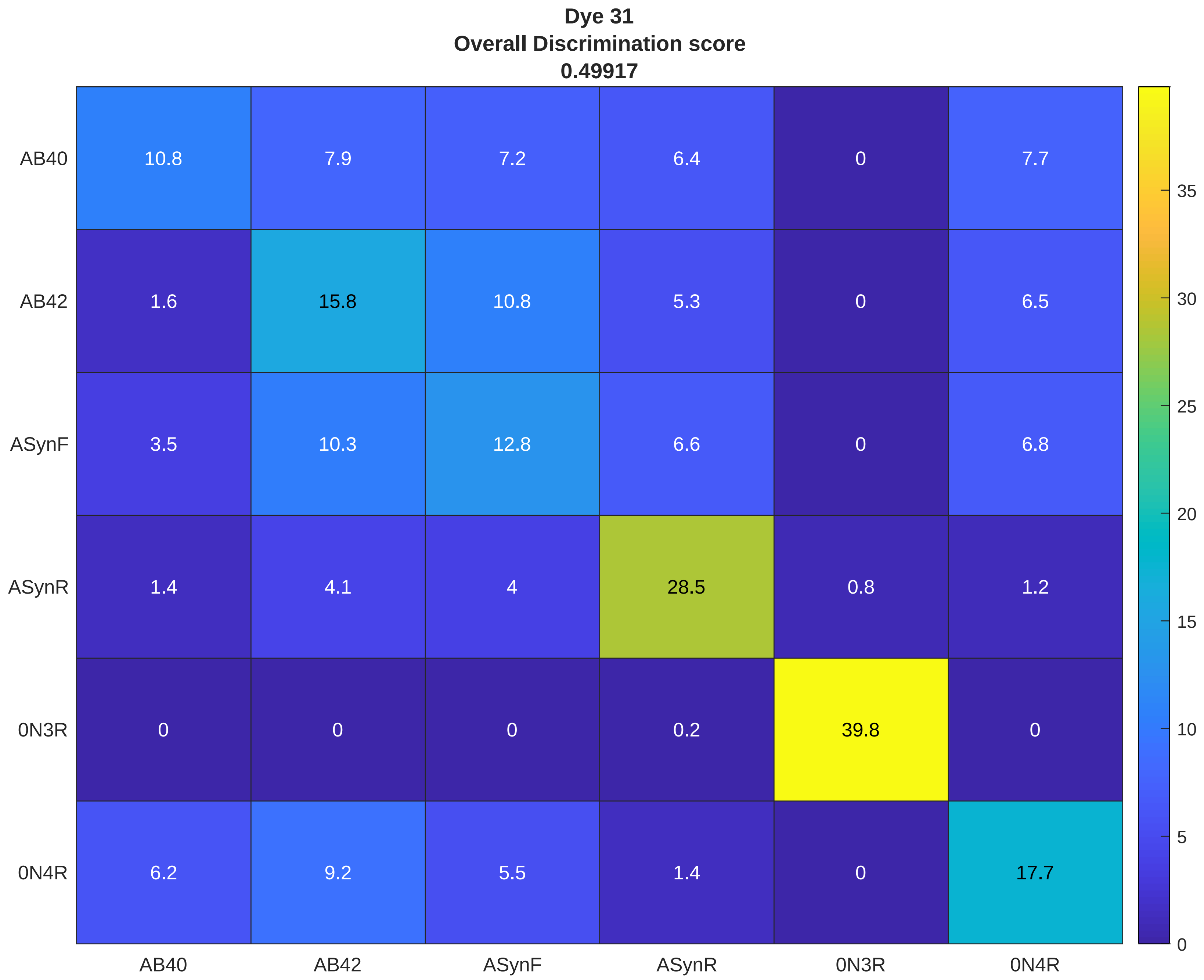
Dye 31
Overall Discrimination score
0.49917
AB40
AB42
ASynF
ASynR
0N3R
0N4R
10.8 7.9 7.2 6.4 0 7.7
1.6 15.8 10.8 5.3 0 6.5
3.5 10.3 12.8 6.6 0 6.8
1.4 4.1 4 28.5 0.8 1.2
0 0 0 0.2 39.8 0
6.2 9.2 5.5 1.4 0 17.7
AB40
AB42
ASynF
ASynR
0N3R
0N4R
35
30
25
20
15
10
5
0

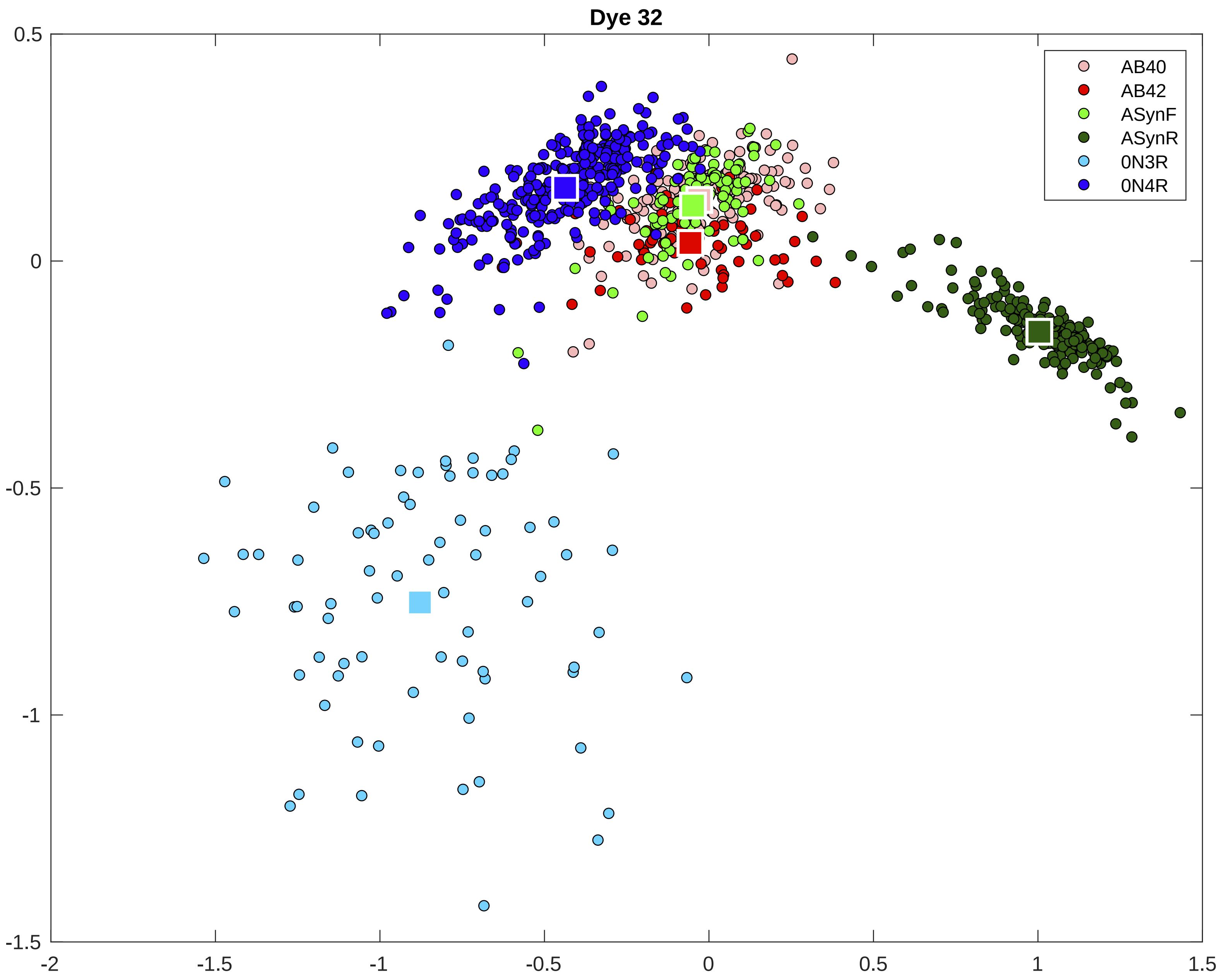
Dye 32
AB40
AB42
ASynF
ASynR
0N3R
0N4R
0.5
0
-0.5
-1
-1.5
-2
-1.5
-1
-0.5
0
0.5
1
1.5

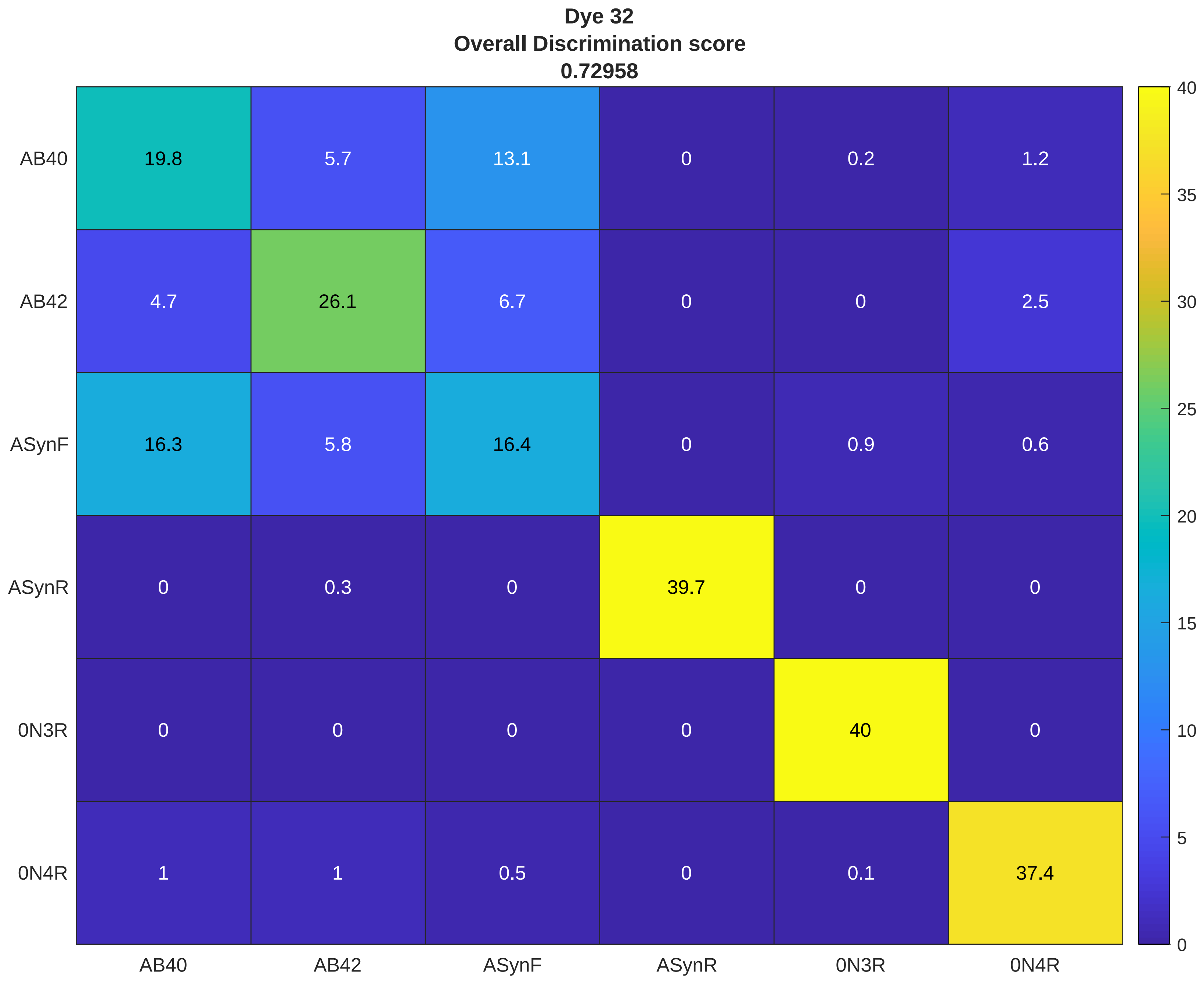
Dye 32
Overall Discrimination score
0.72958
AB40
AB42
ASynF
ASynR
0N3R
0N4R
19.8
5.7
13.1
0
0.2
1.2
4.7
26.1
6.7
0
0
2.5
16.3
5.8
16.4
0
0.9
0.6
0
0.3
0
39.7
0
0
0
0
0
0
40
0
1
1
0.5
0
0.1
37.4
AB40
AB42
ASynF
ASynR
0N3R
0N4R
40
35
30
25
20
15
10
5
0

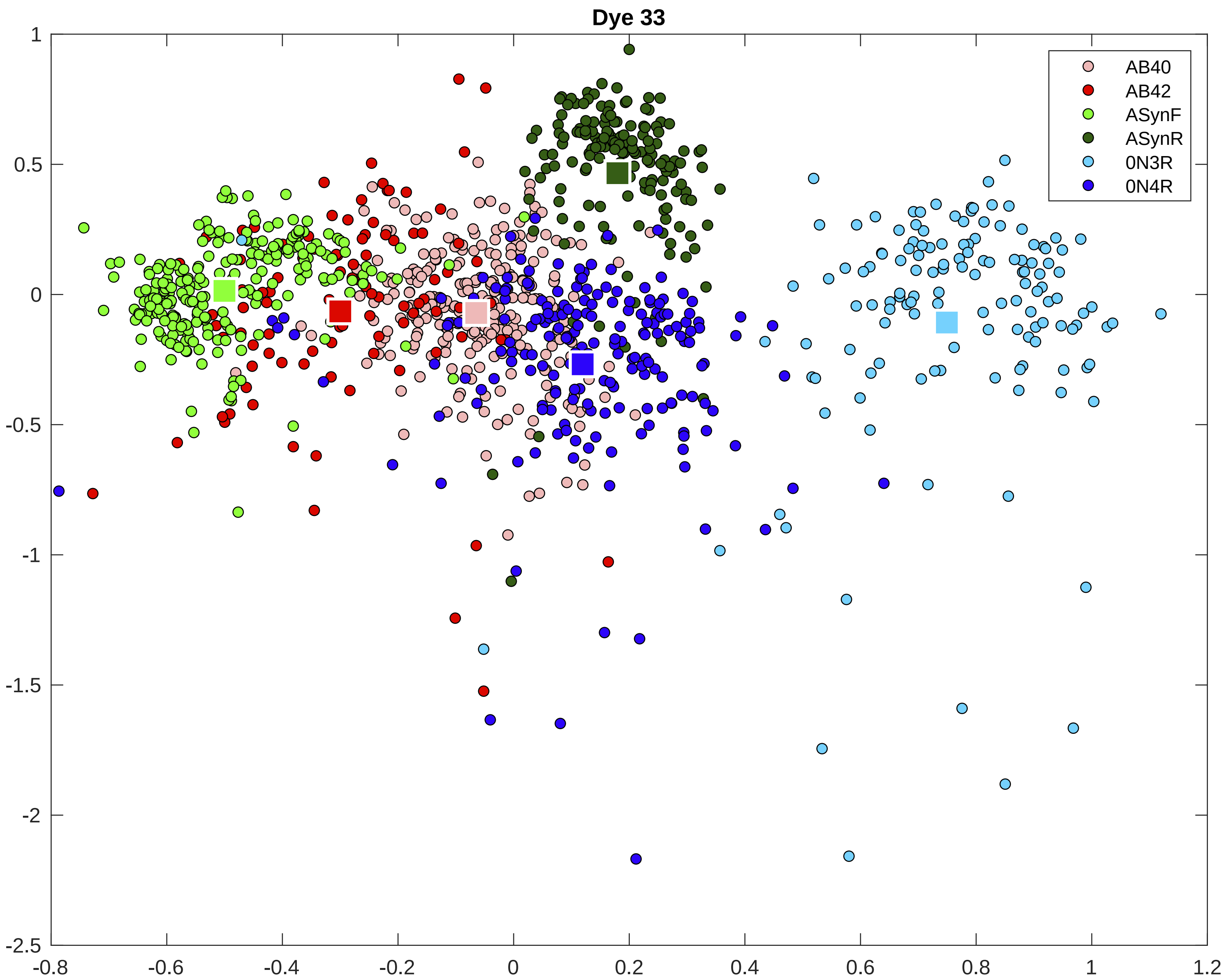

Dye 33
AB40
AB42
ASynF
ASynR
0N3R
0N4R
1
0.5
0
-0.5
-1
-1.5
-2
-2.5
-0.8
-0.6
-0.4
-0.2
0
0.2
0.4
0.6
0.8
1
1.2

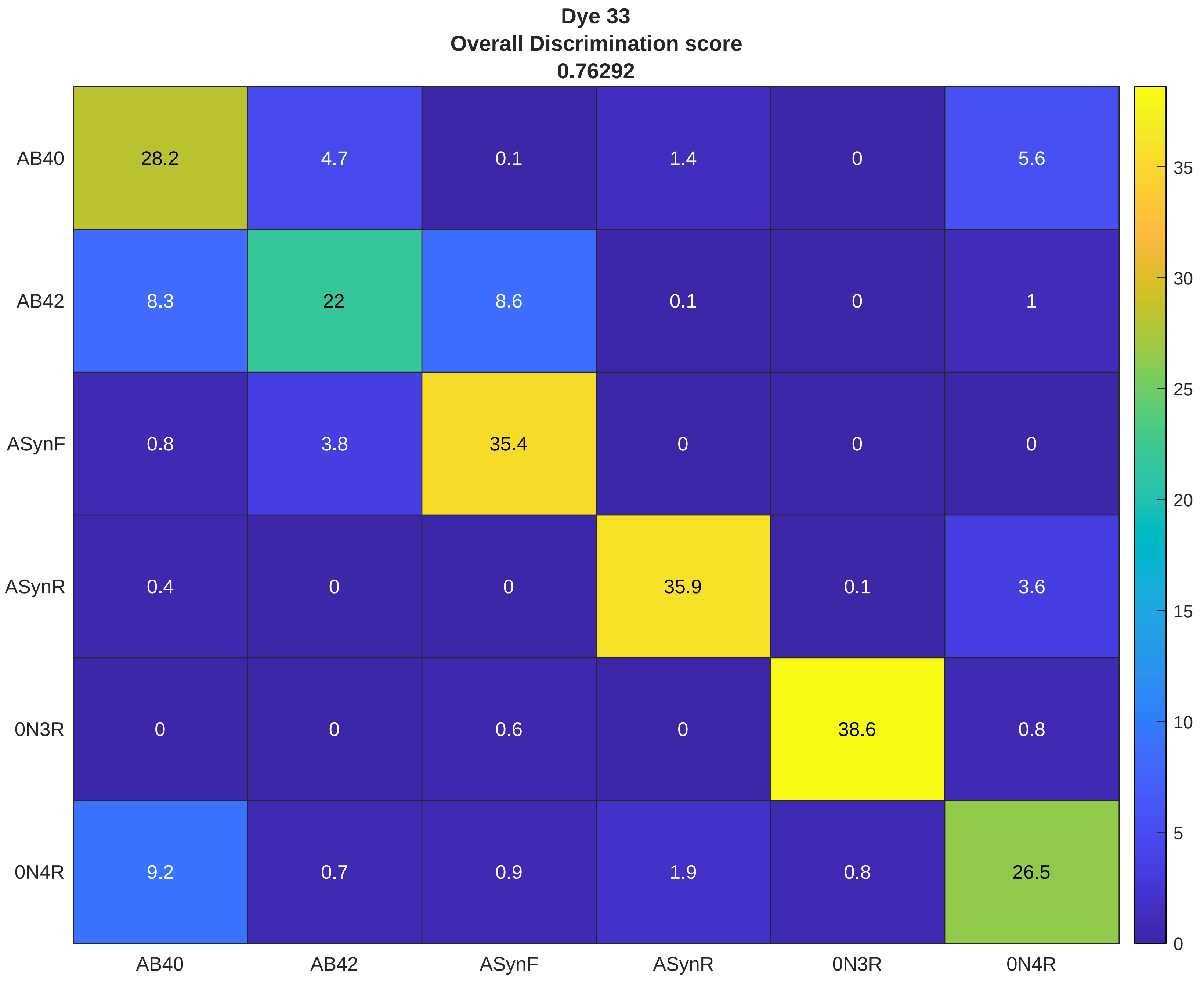

Dye 33
Overall Discrimination score
0.76292
AB40
AB42
ASynF
ASynR
0N3R
0N4R
28.2
4.7
0.1
1.4
0
5.6
8.3
22
8.6
0.1
0
1
0.8
3.8
35.4
0
0
0
0.4
0
0
35.9
0.1
3.6
0
0
0.6
0
38.6
0.8
9.2
0.7
0.9
1.9
0.8
26.5
AB40
AB42
ASynF
ASynR
0N3R
0N4R
35
30
25
20
15
10
5
0

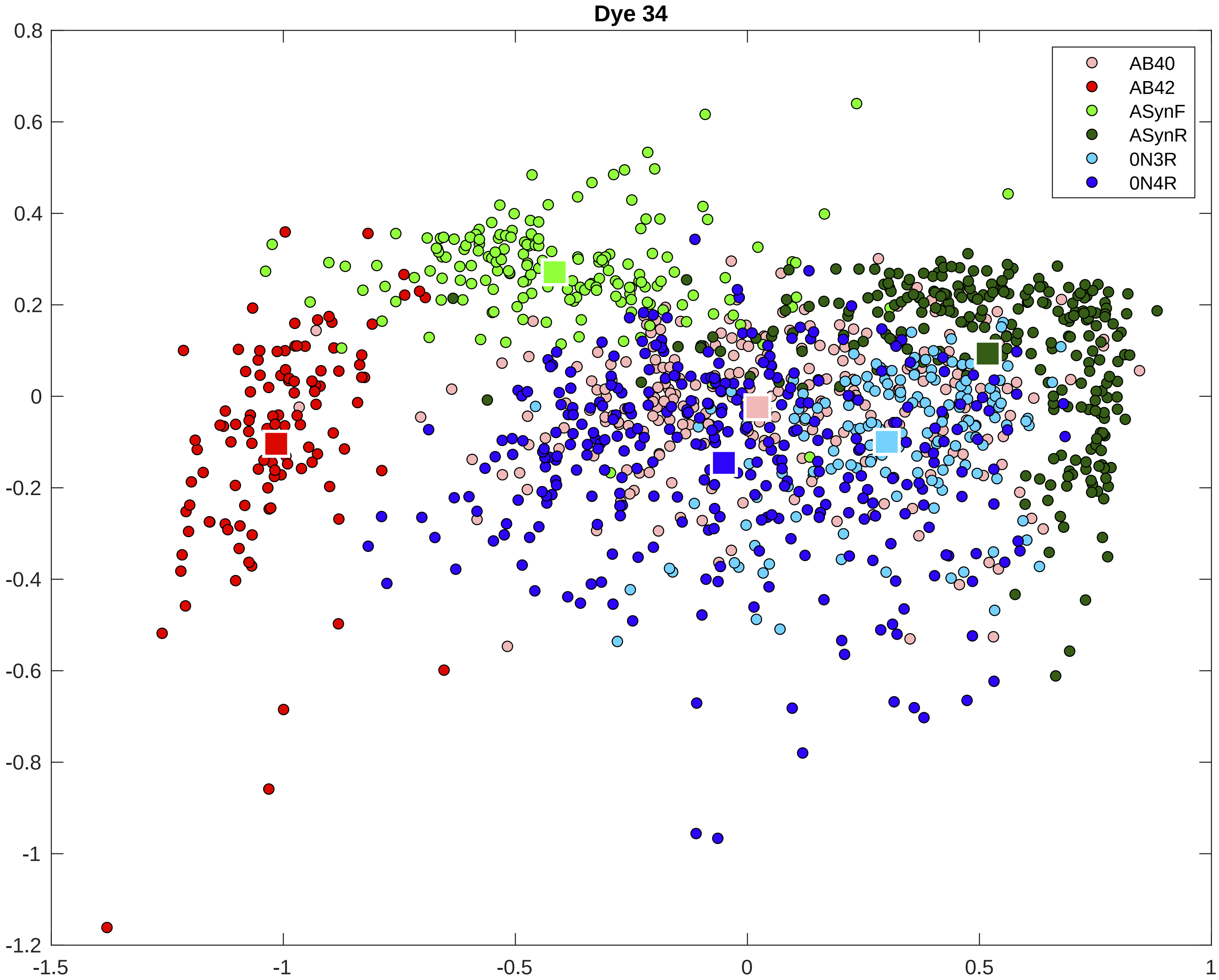

Dye 34
0.8
0.6
0.4
0.2
0
-0.2
-0.4
-0.6
-0.8
-1
-1.2
-1.5
-1
-0.5
0
0.5
1
AB40
AB42
ASynF
ASynR
0N3R
0N4R

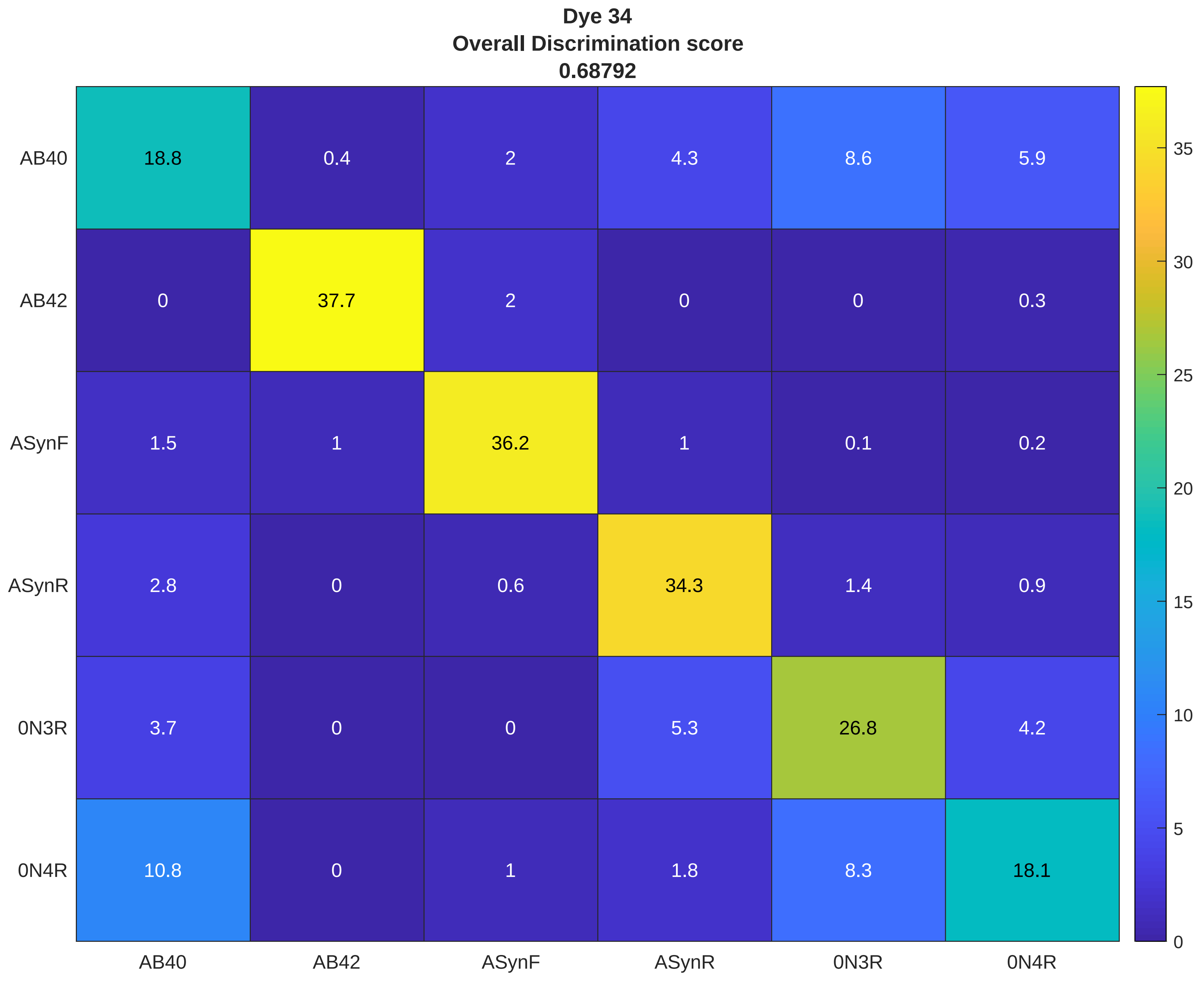
Dye 34
Overall Discrimination score
0.68792
AB40
AB42
ASynF
ASynR
0N3R
0N4R
18.8 0.4 2 4.3 8.6 5.9
0 37.7 2 0 0 0.3
1.5 1 36.2 1 0.1 0.2
2.8 0 0.6 34.3 1.4 0.9
3.7 0 0 5.3 26.8 4.2
10.8 0 1 1.8 8.3 18.1
AB40
AB42
ASynF
ASynR
0N3R
0N4R
35
30
25
20
15
10
5
0

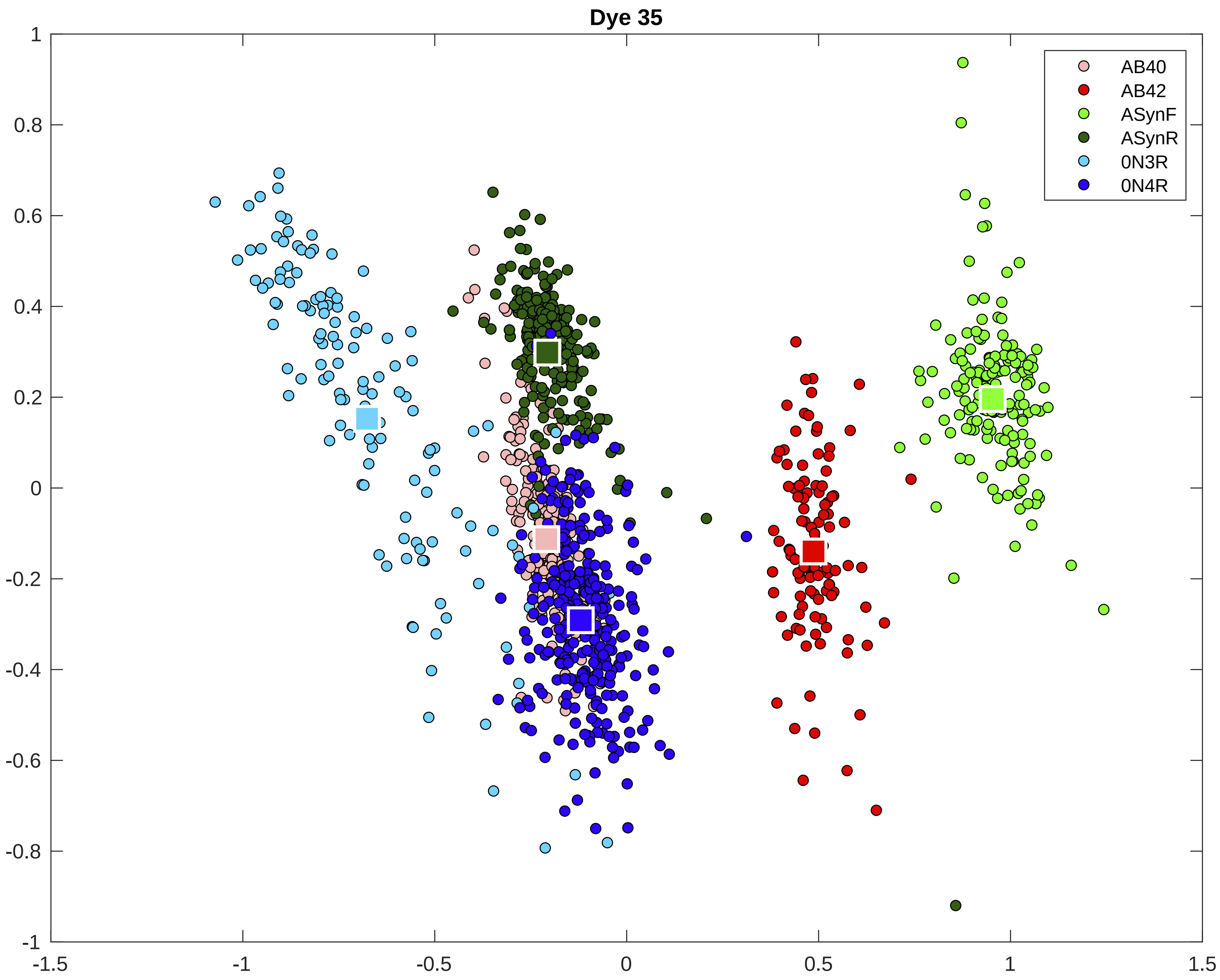

Dye 35
AB40
AB42
ASynF
ASynR
0N3R
0N4R
1
0.8
0.6
0.4
0.2
0
-0.2
-0.4
-0.6
-0.8
-1
-1.5
-1
-0.5
0
0.5
1
1.5

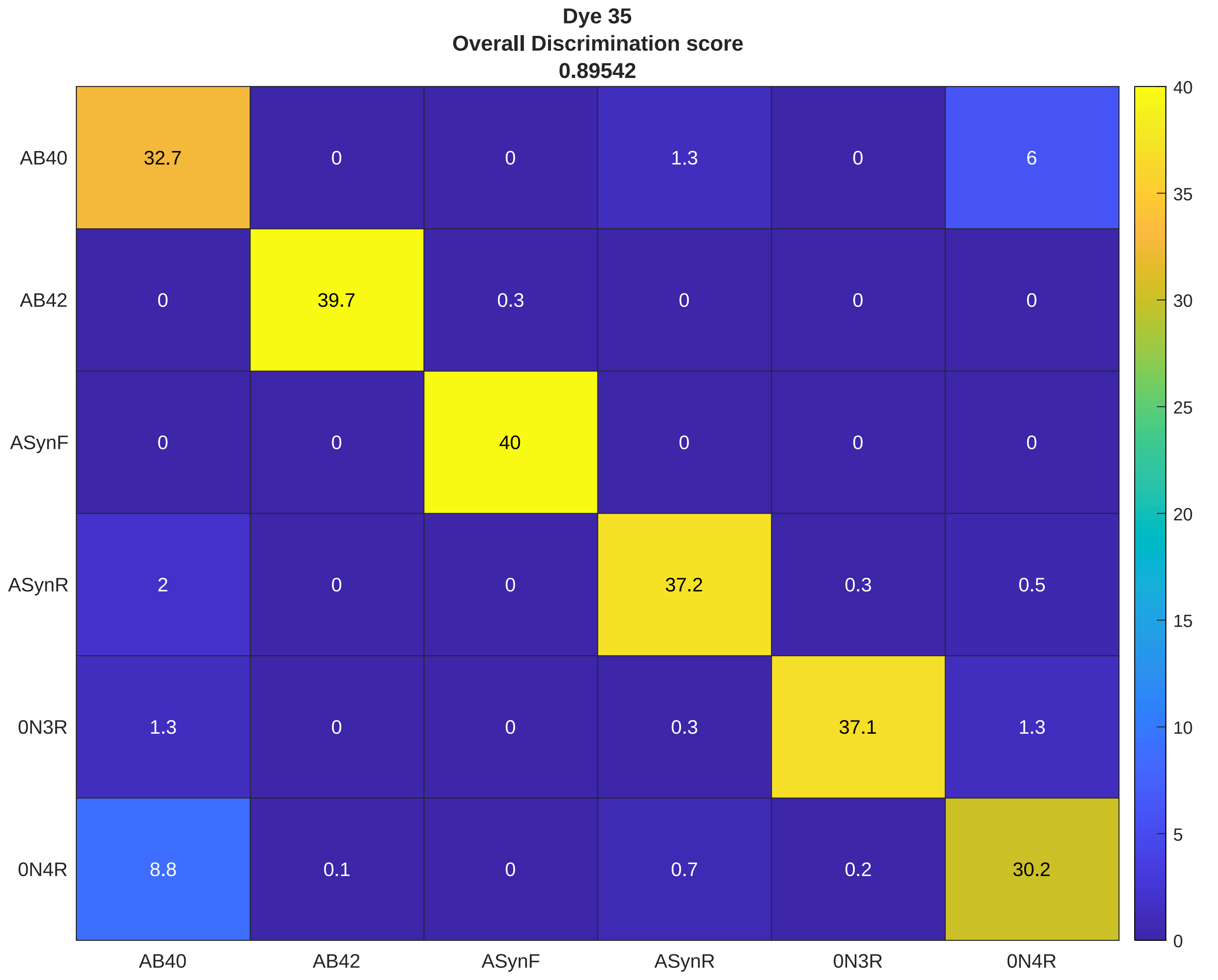
Dye 35
Overall Discrimination score
0.89542
AB40
AB42
ASynF
ASynR
0N3R
0N4R
32.7
0
0
1.3
0
6
0
39.7
0.3
0
0
0
0
0
40
0
0
0
2
0
0
37.2
0.3
0.5
1.3
0
0
0.3
37.1
1.3
8.8
0.1
0
0.7
0.2
30.2
AB40
AB42
ASynF
ASynR
0N3R
0N4R
40
35
30
25
20
15
10
5
0

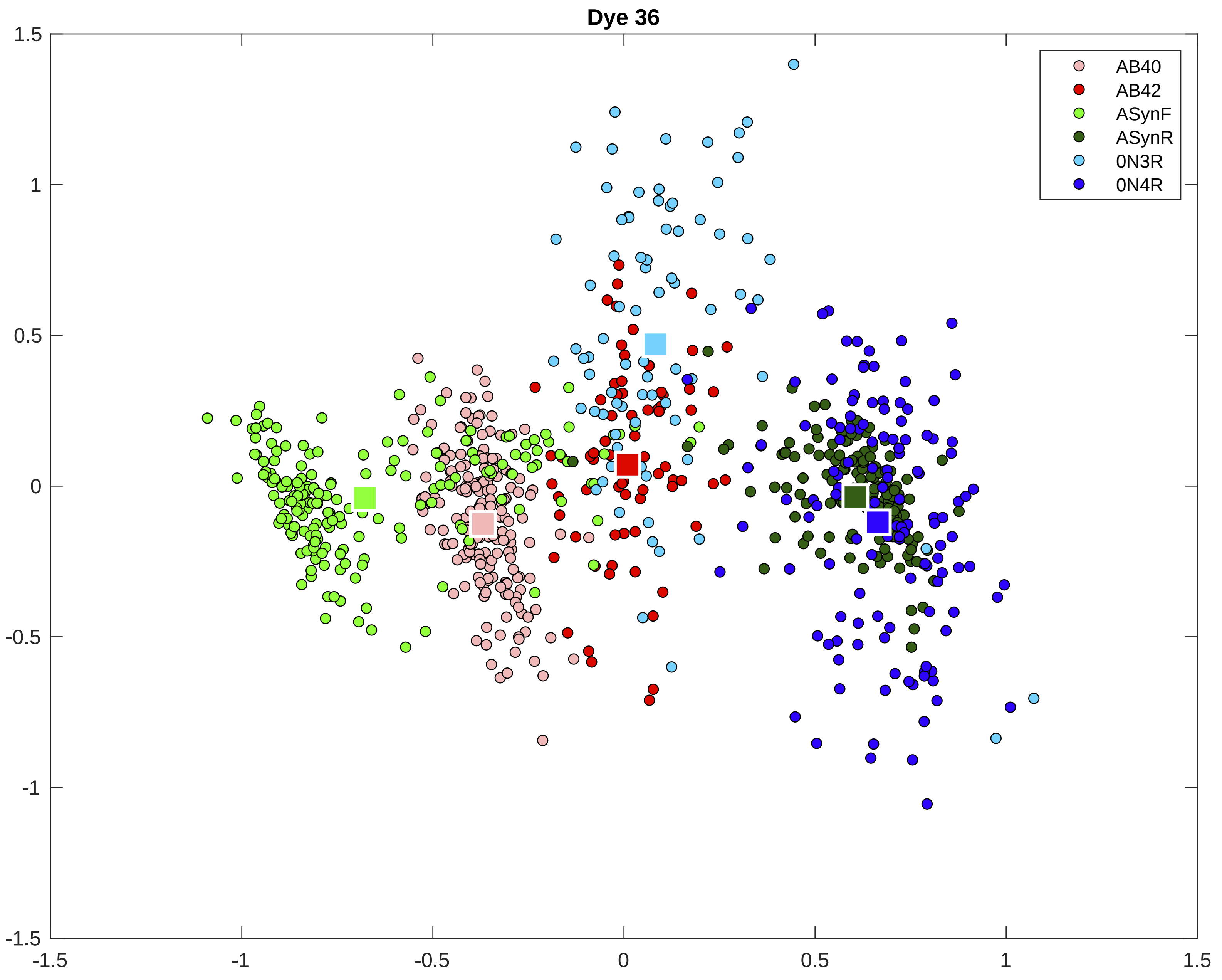
Dye 36
AB40
AB42
ASynF
ASynR
0N3R
0N4R
1.5
1
0.5
0
-0.5
-1
-1.5
-1.5
-1
-0.5
0
0.5
1
1.5

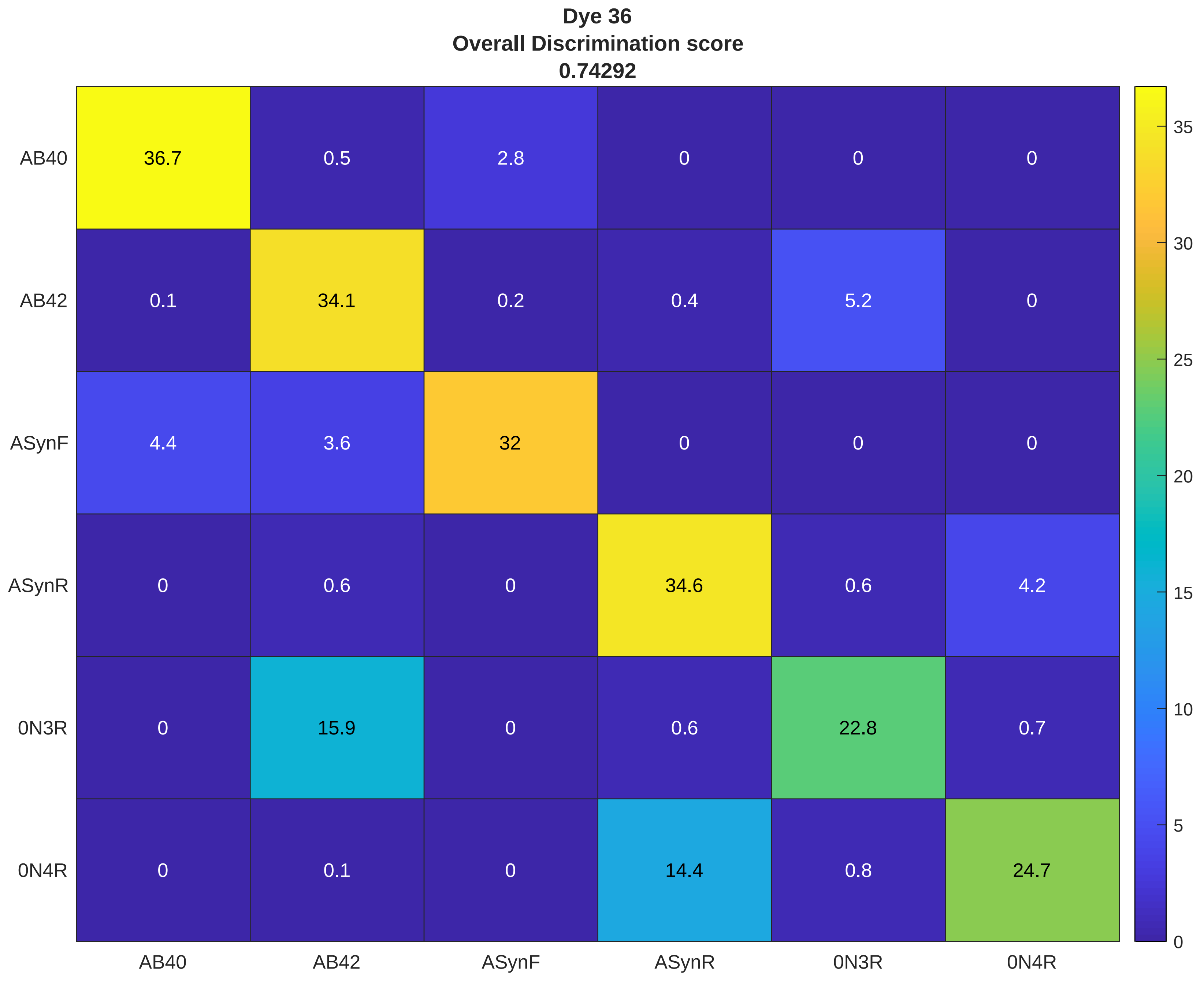
Dye 36
Overall Discrimination score
0.74292
AB40
AB42
ASynF
ASynR
0N3R
0N4R
36.7
0.5
2.8
0
0
0
0.1
34.1
0.2
0.4
5.2
0
4.4
3.6
32
0
0
0
0
0.6
0
34.6
0.6
4.2
0
15.9
0
0.6
22.8
0.7
0
0.1
0
14.4
0.8
24.7
AB40
AB42
ASynF
ASynR
0N3R
0N4R
35
30
25
20
15
10
5
0

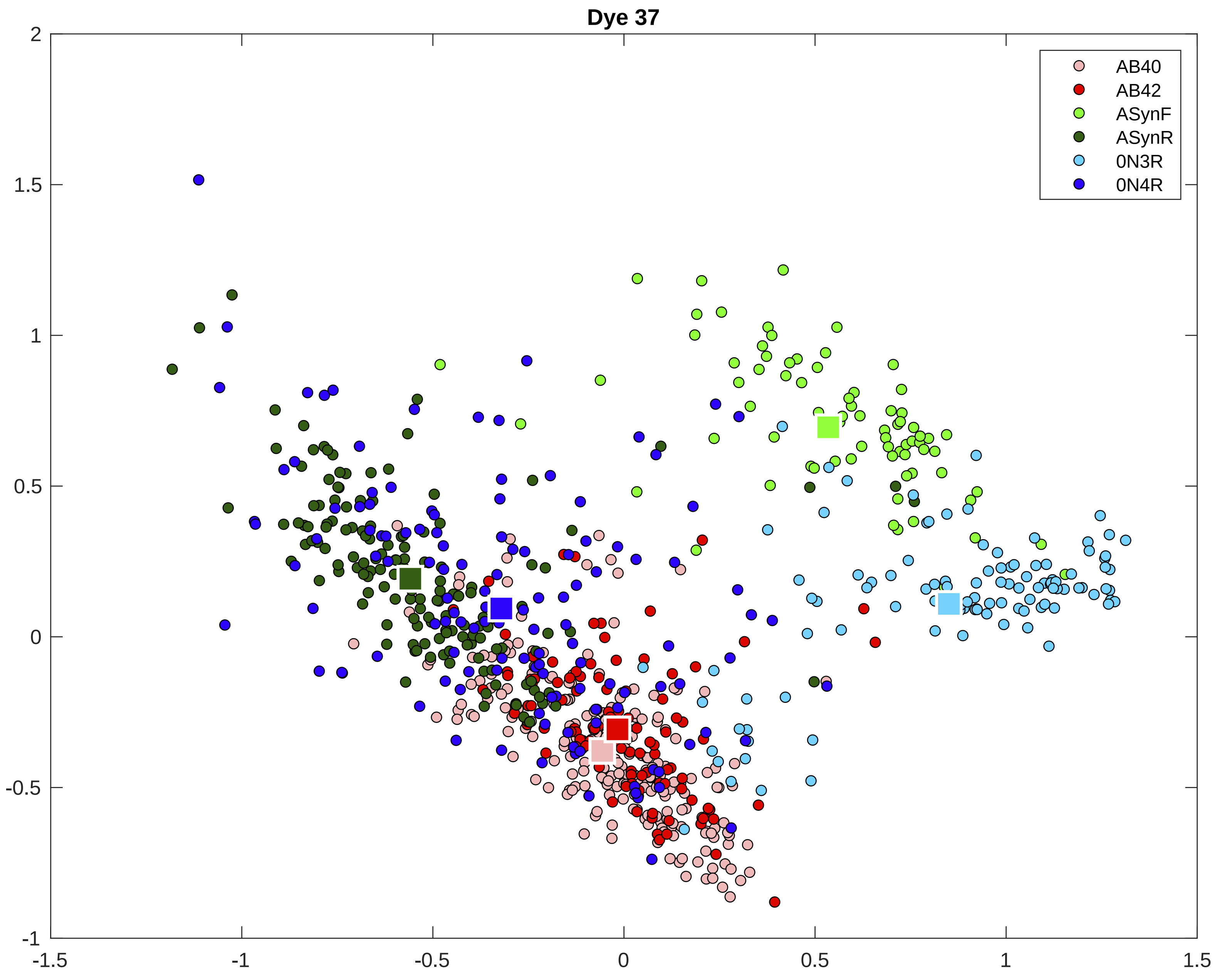

Dye 37
AB40
AB42
ASynF
ASynR
0N3R
0N4R
2
1.5
1
0.5
0
-0.5
-1
-1.5
-1
-0.5
0
0.5
1
1.5

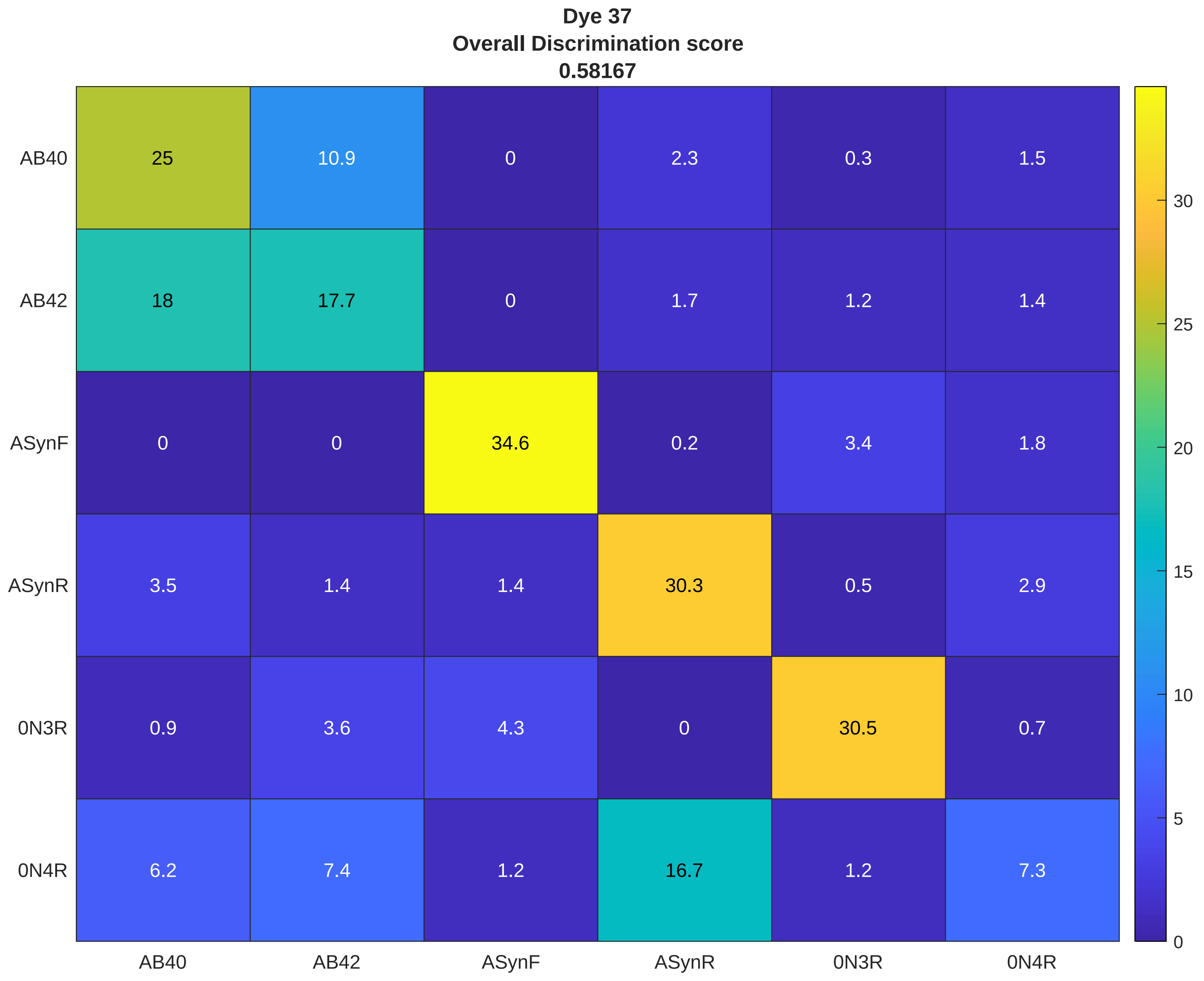

Dye 37
Overall Discrimination score
0.58167
AB40
AB42
ASynF
ASynR
0N3R
0N4R
25
10.9
0
2.3
0.3
1.5
18
17.7
0
1.7
1.2
1.4
0
0
34.6
0.2
3.4
1.8
3.5
1.4
1.4
30.3
0.5
2.9
0.9
3.6
4.3
0
30.5
0.7
6.2
7.4
1.2
16.7
1.2
7.3
AB40
AB42
ASynF
ASynR
0N3R
0N4R
30
25
20
15
10
5
0

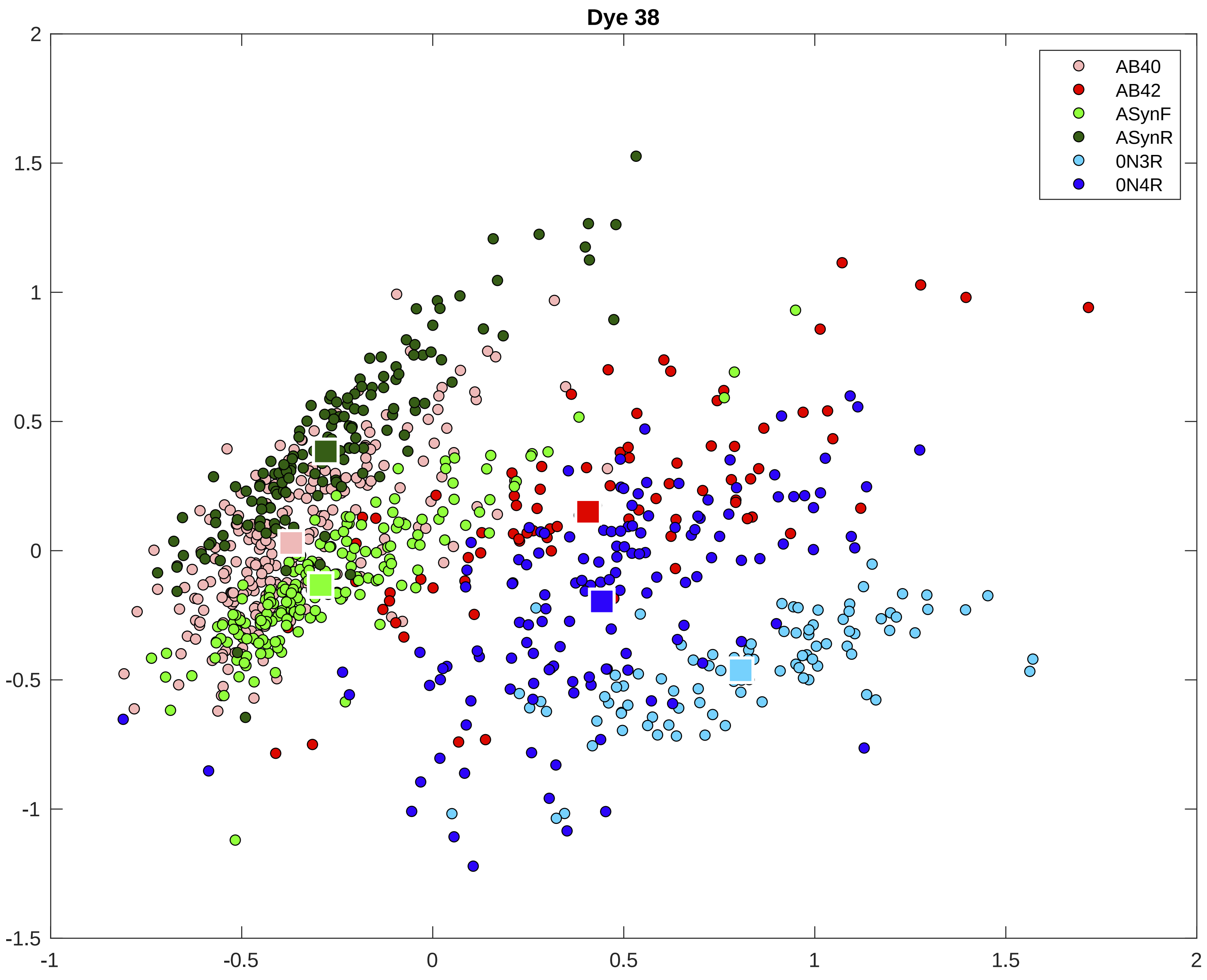
Dye 38
2
1.5
1
0.5
0
-0.5
-1
-1.5
-1
-0.5
0
0.5
1
1.5
2
AB40
AB42
ASynF
ASynR
0N3R
0N4R

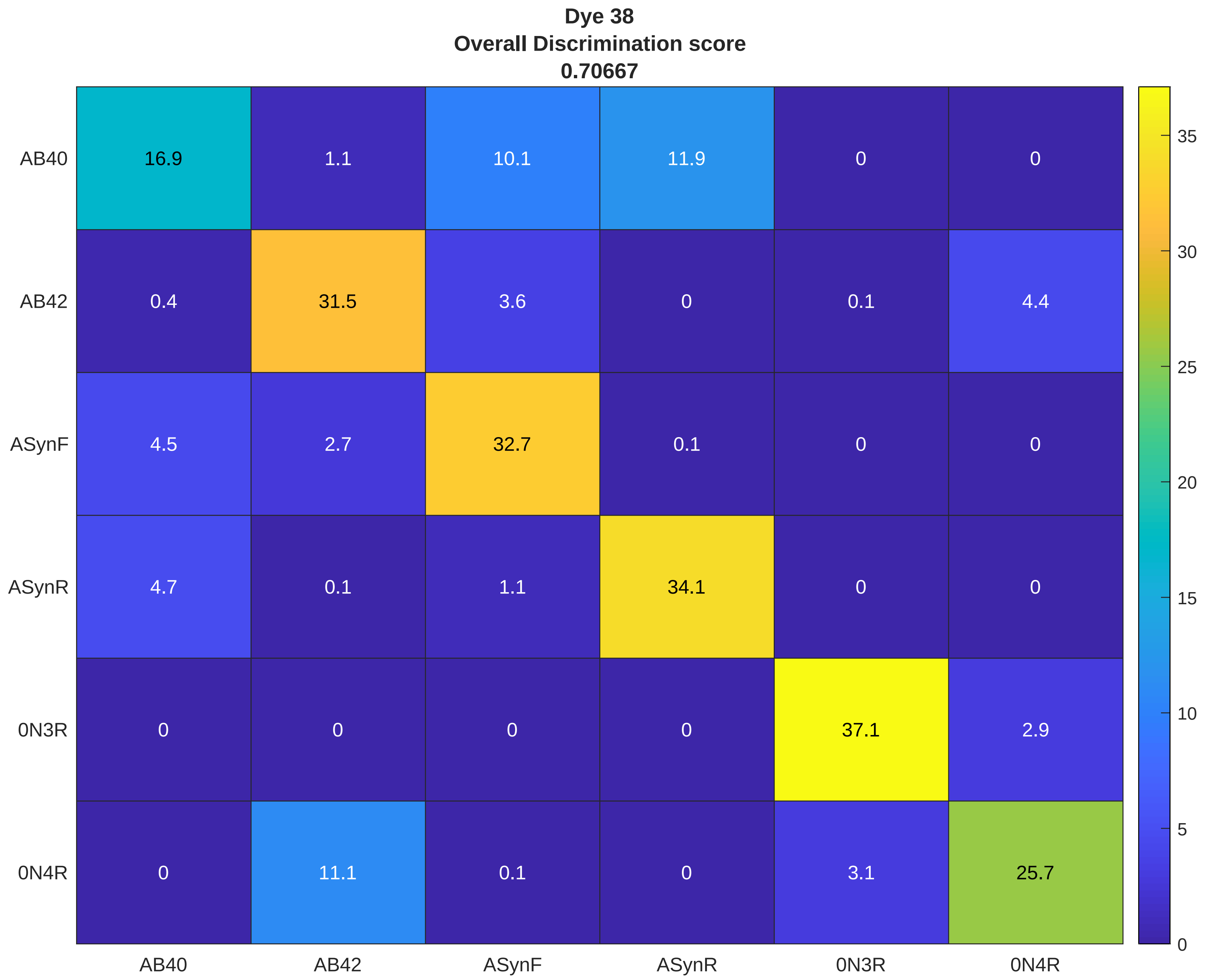
Dye 38
Overall Discrimination score
0.70667
AB40
AB42
ASynF
ASynR
0N3R
0N4R
16.9
1.1
10.1
11.9
0
0
0.4
31.5
3.6
0
0.1
4.4
4.5
2.7
32.7
0.1
0
0
4.7
0.1
1.1
34.1
0
0
0
0
0
0
37.1
2.9
0
11.1
0.1
0
3.1
25.7
AB40
AB42
ASynF
ASynR
0N3R
0N4R
35
30
25
20
15
10
5
0

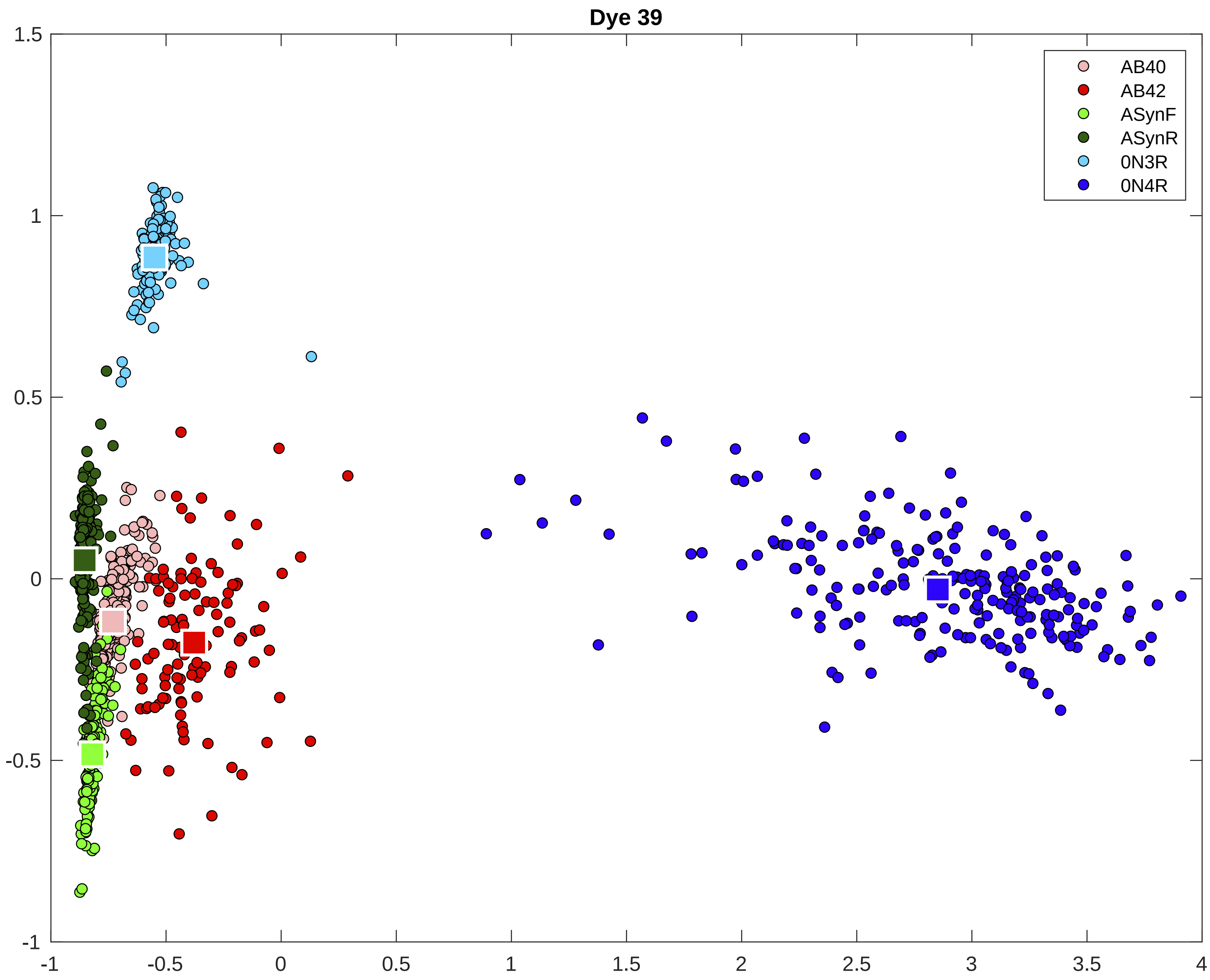

Dye 39
AB40
AB42
ASynF
ASynR
0N3R
0N4R
1.5
1
0.5
0
-0.5
-1
-1
-0.5
0
0.5
1
1.5
2
2.5
3
3.5
4

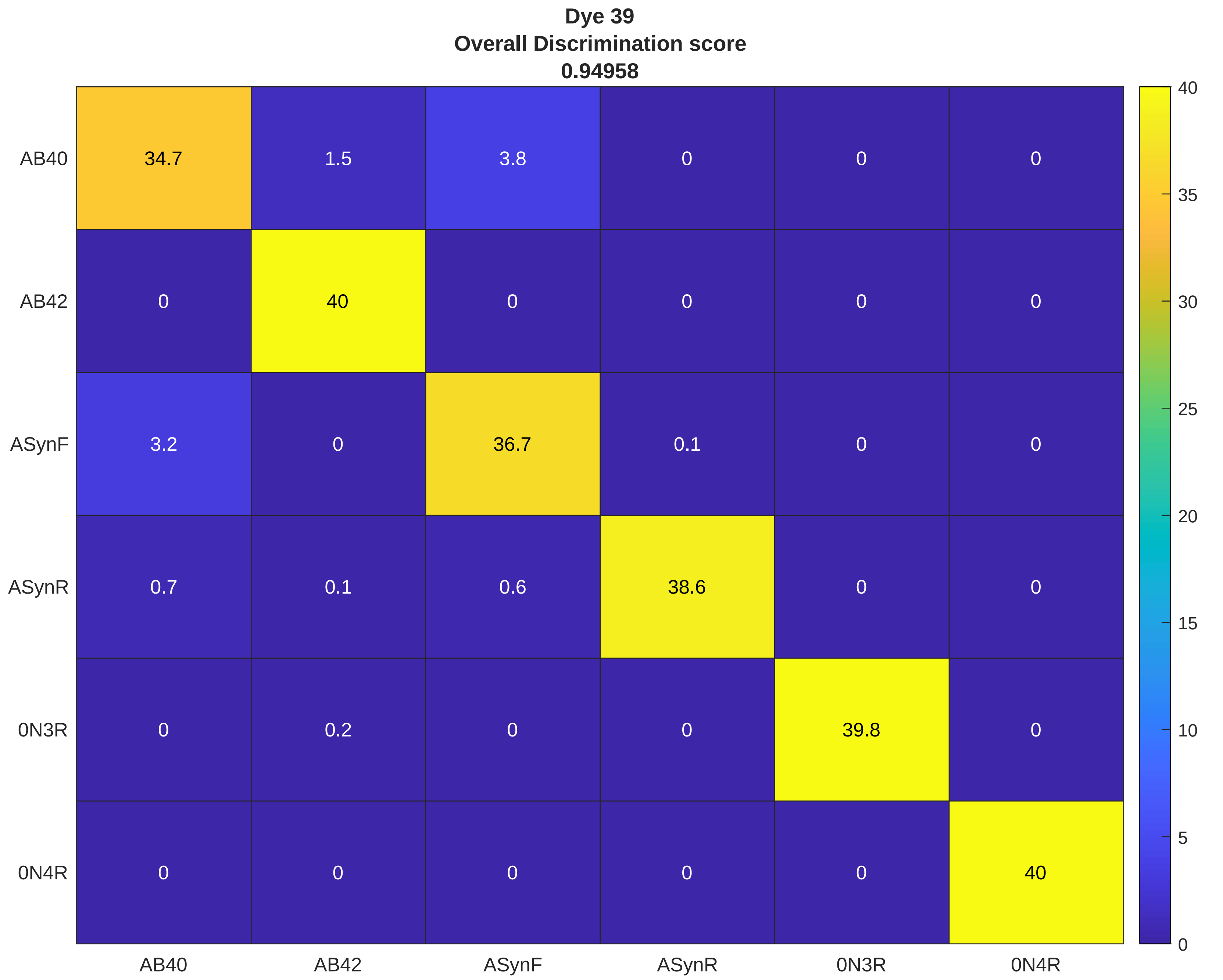
Dye 39
Overall Discrimination score
0.94958
AB40
AB42
ASynF
ASynR
0N3R
0N4R
34.7 1.5 3.8 0 0 0
0 40 0 0 0 0
3.2 0 36.7 0.1 0 0
0.7 0.1 0.6 38.6 0 0
0 0.2 0 0 39.8 0
0 0 0 0 0 40
AB40
AB42
ASynF
ASynR
0N3R
0N4R
40
35
30
25
20
15
10
5
0

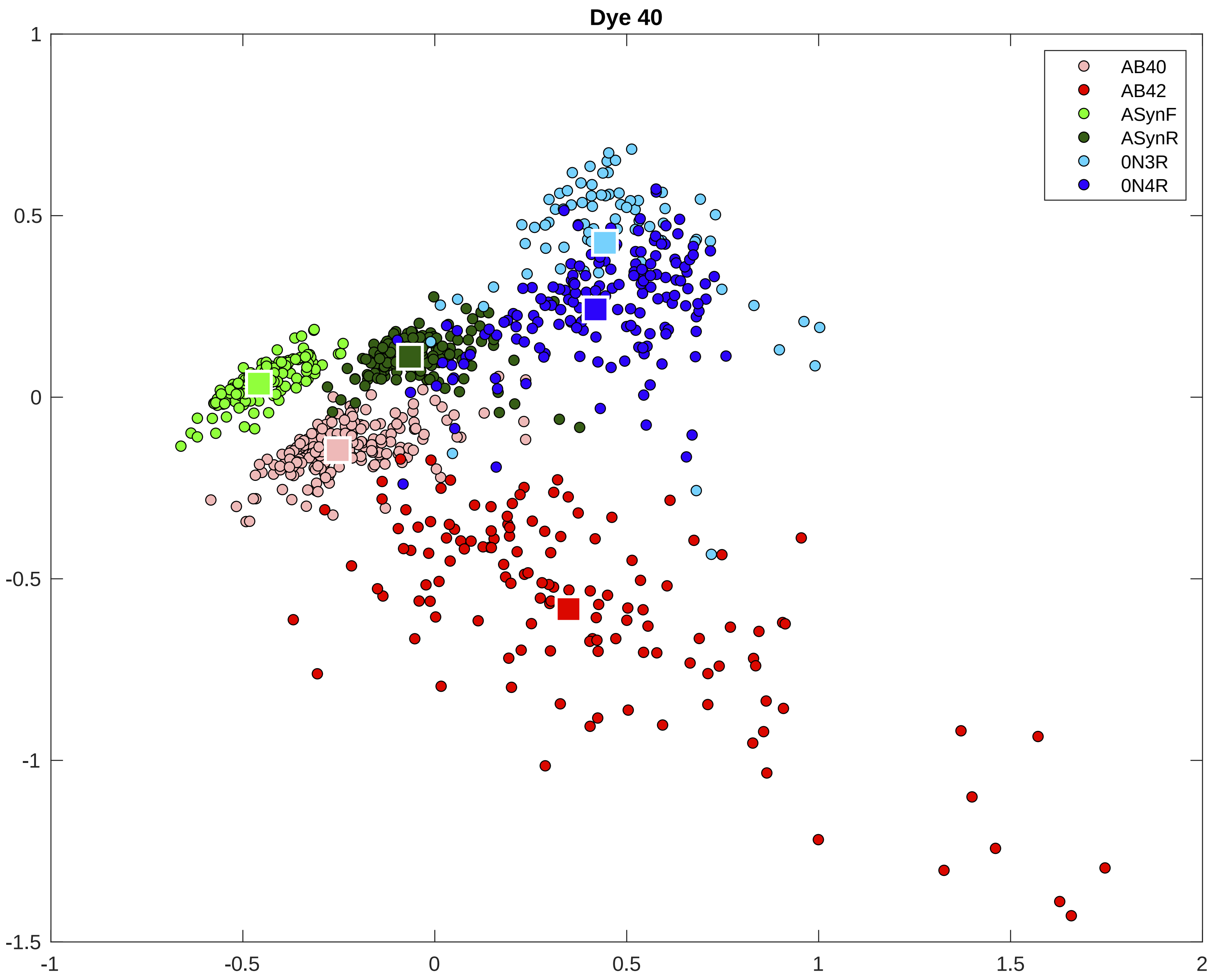
Dye 40
1
0.5
0
-0.5
-1
-1.5
-1
-0.5
0
0.5
1
1.5
2
AB40
AB42
ASynF
ASynR
0N3R
0N4R

Dye 40
Overall Discrimination score
0.87792

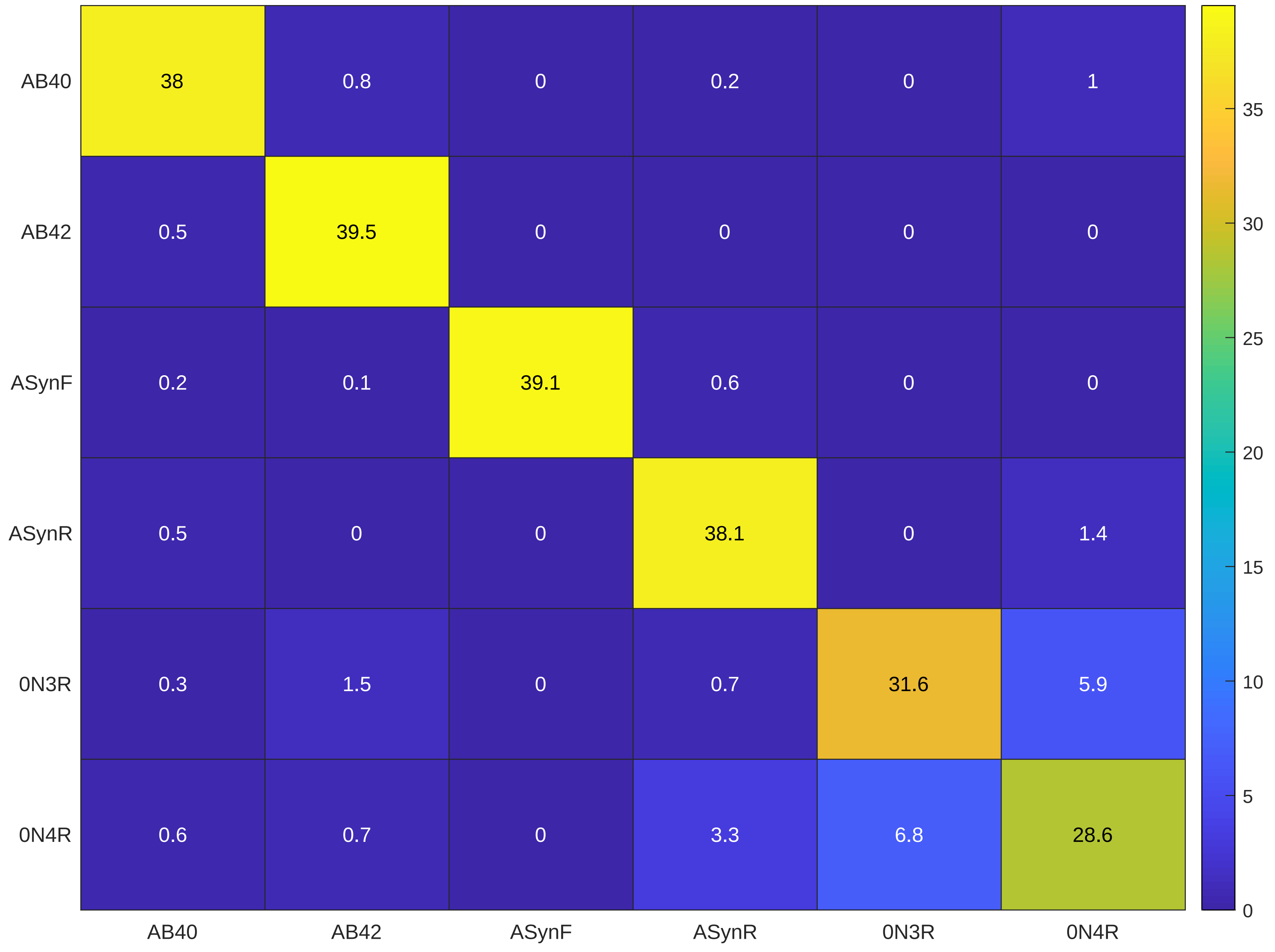

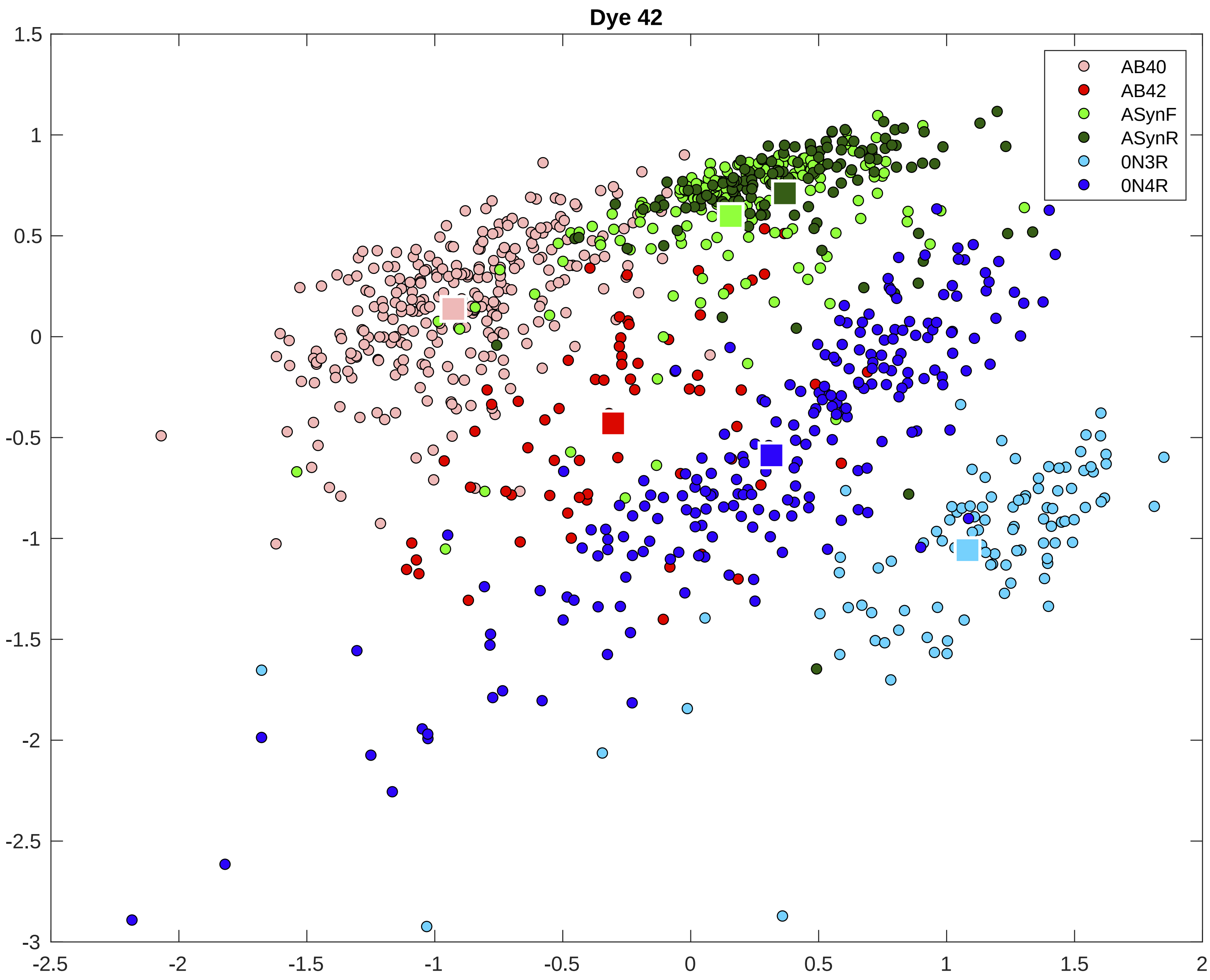

Dye 42
AB40
AB42
ASynF
ASynR
0N3R
0N4R
1.5
1
0.5
0
-0.5
-1
-1.5
-2
-2.5
-3
-2.5
-2
-1.5
-1
-0.5
0
0.5
1
1.5
2

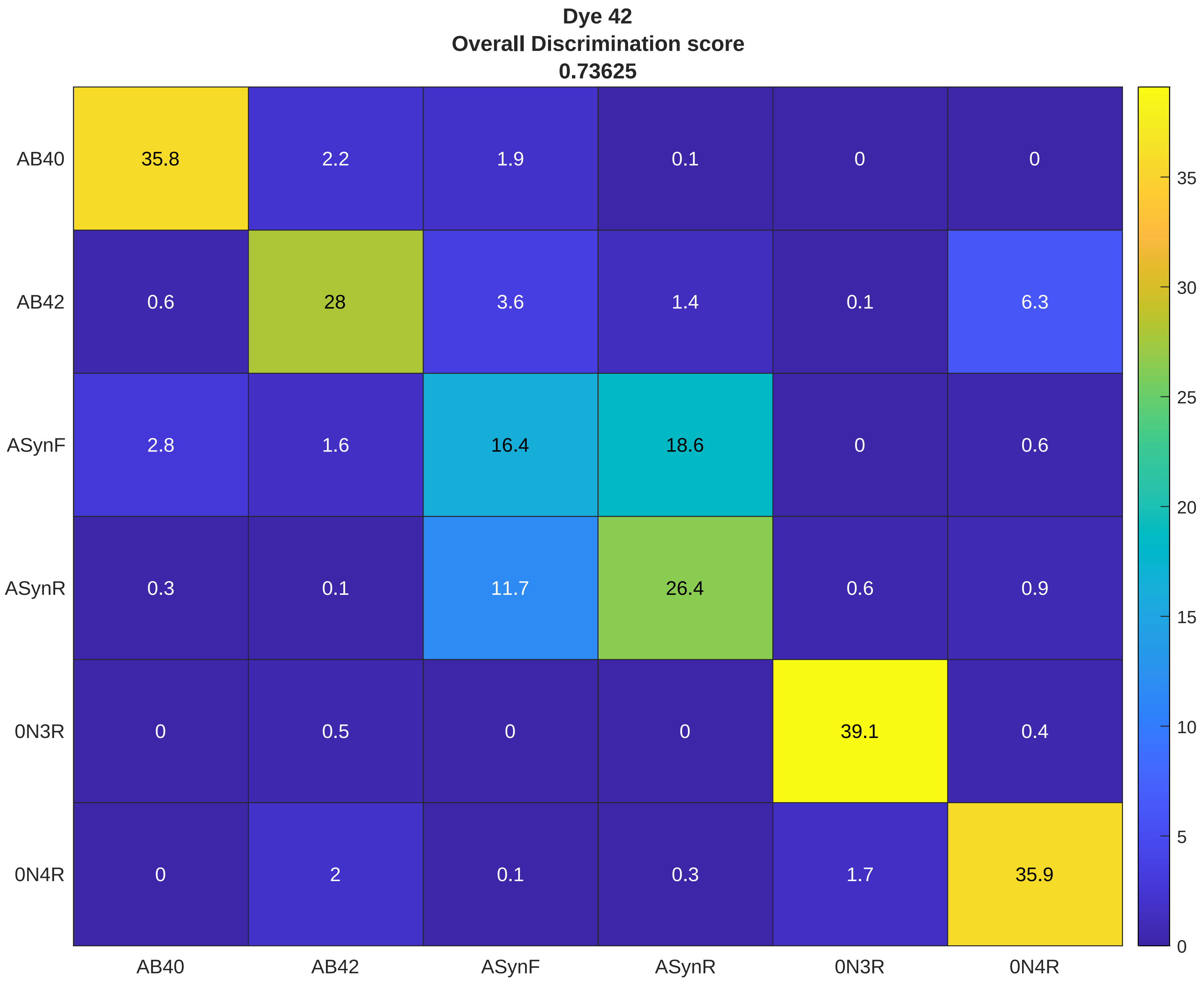
Dye 42
Overall Discrimination score
0.73625
AB40
AB42
ASynF
ASynR
0N3R
0N4R
35.8 2.2 1.9 0.1 0 0
0.6 28 3.6 1.4 0.1 6.3
2.8 1.6 16.4 18.6 0 0.6
0.3 0.1 11.7 26.4 0.6 0.9
0 0.5 0 0 39.1 0.4
0 2 0.1 0.3 1.7 35.9
AB40
AB42
ASynF
ASynR
0N3R
0N4R
35
30
25
20
15
10
5
0

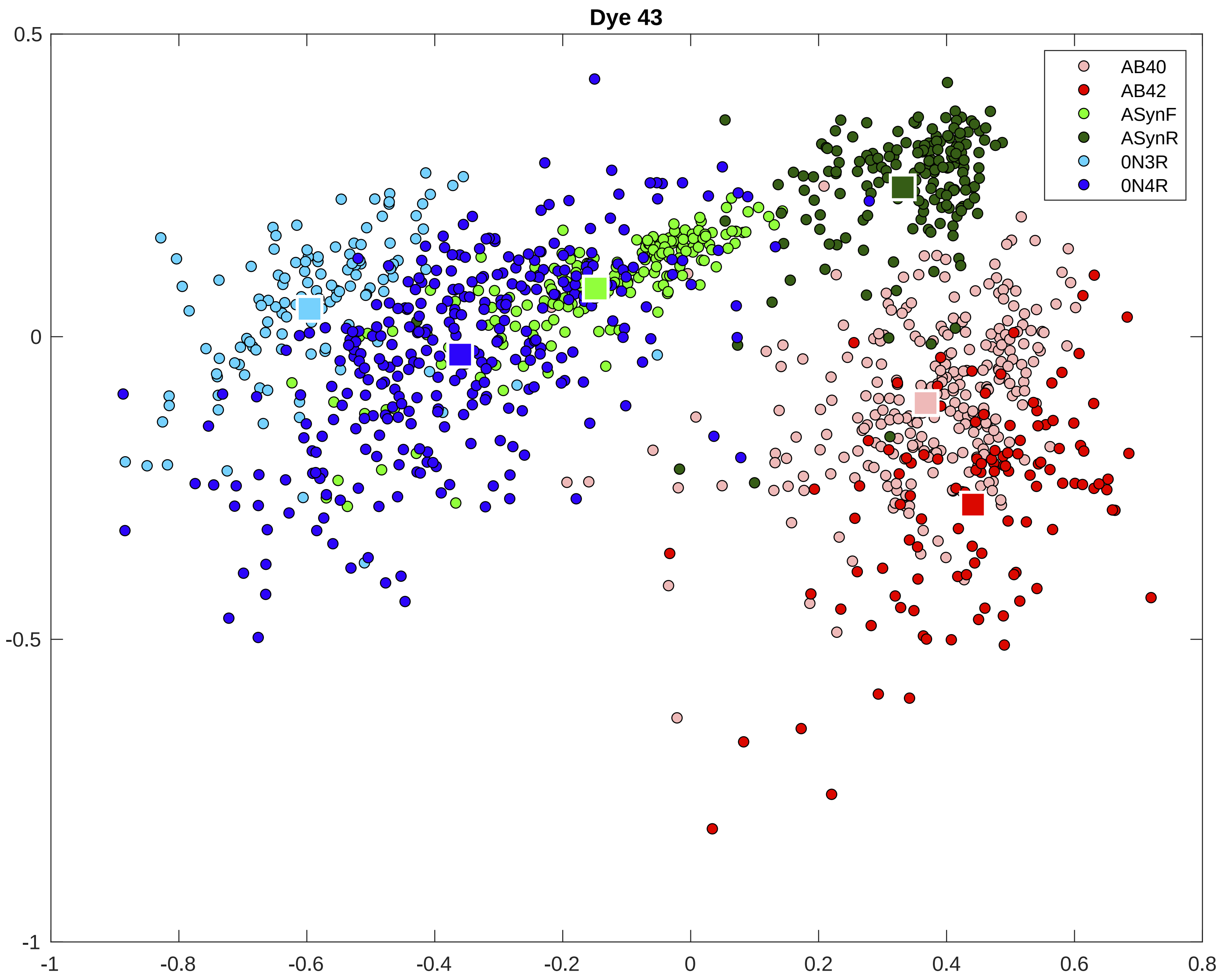

Dye 43
AB40
AB42
ASynF
ASynR
0N3R
0N4R
0.5
0
-0.5
-1
-0.8
-0.6
-0.4
-0.2
0.2
0.4
0.6
0.8

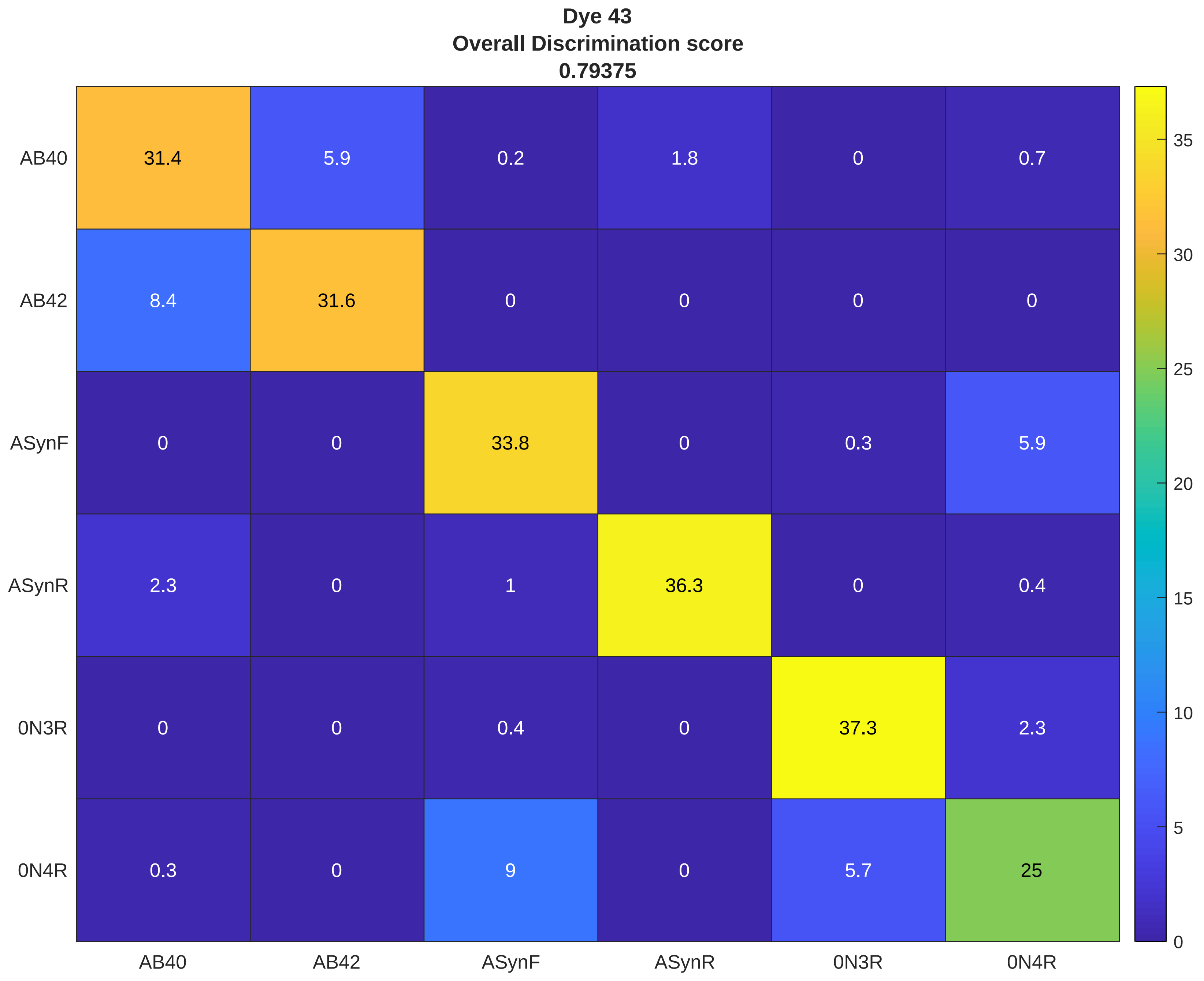
Dye 43
Overall Discrimination score
0.79375
AB40
AB42
ASynF
ASynR
0N3R
0N4R
31.4
5.9
0.2
1.8
0
0.7
8.4
31.6
0
0
0
0
0
0
33.8
0
0.3
5.9
2.3
0
1
36.3
0
0.4
0
0
0.4
0
37.3
2.3
0.3
0
9
0
5.7
25
AB40
AB42
ASynF
ASynR
0N3R
0N4R
35
30
25
20
15
10
5
0

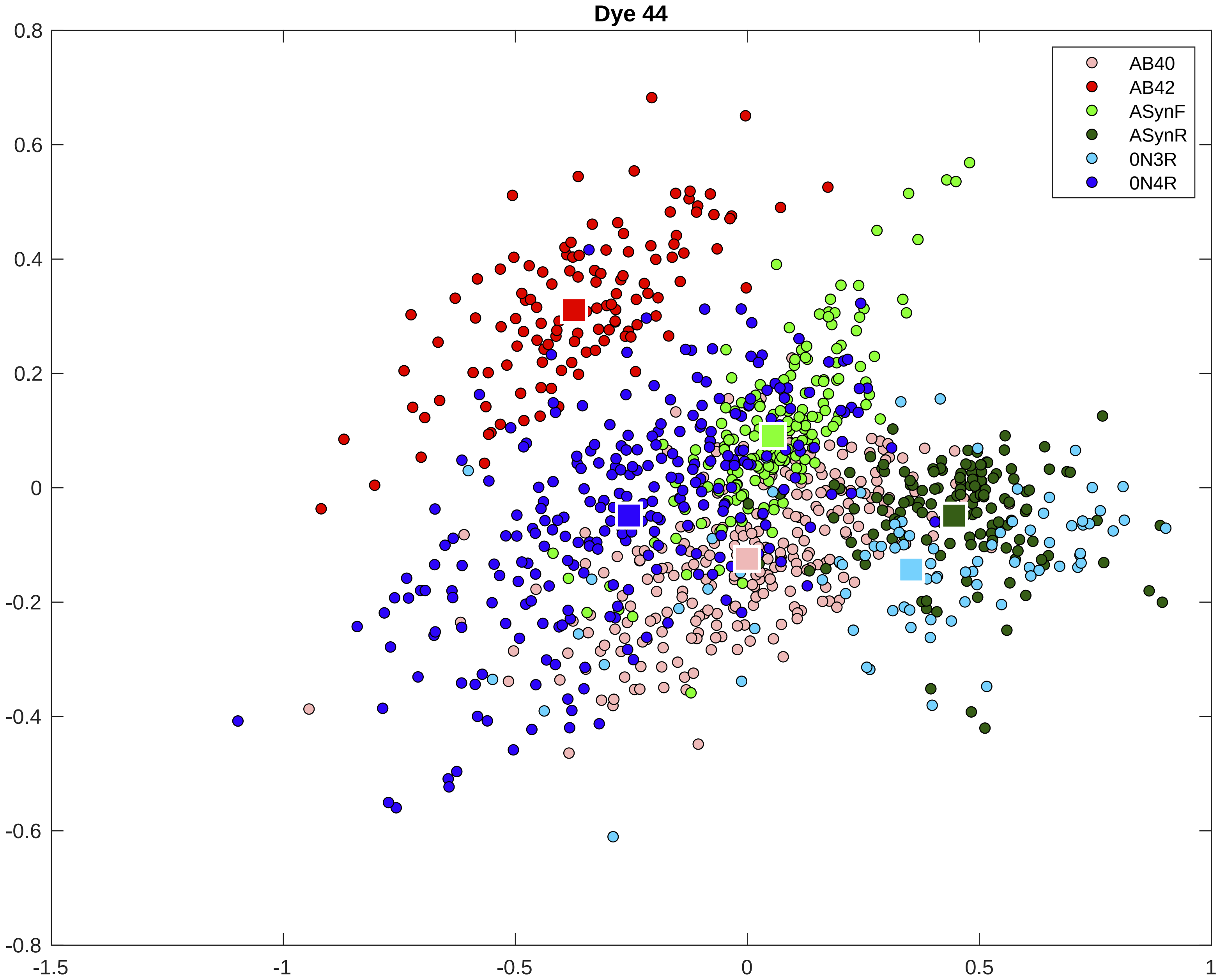

Dye 44
AB40
AB42
ASynF
ASynR
0N3R
0N4R
0.8
0.6
0.4
0.2
0
-0.2
-0.4
-0.6
-0.8
-1.5
-1
-0.5
0
0.5
1

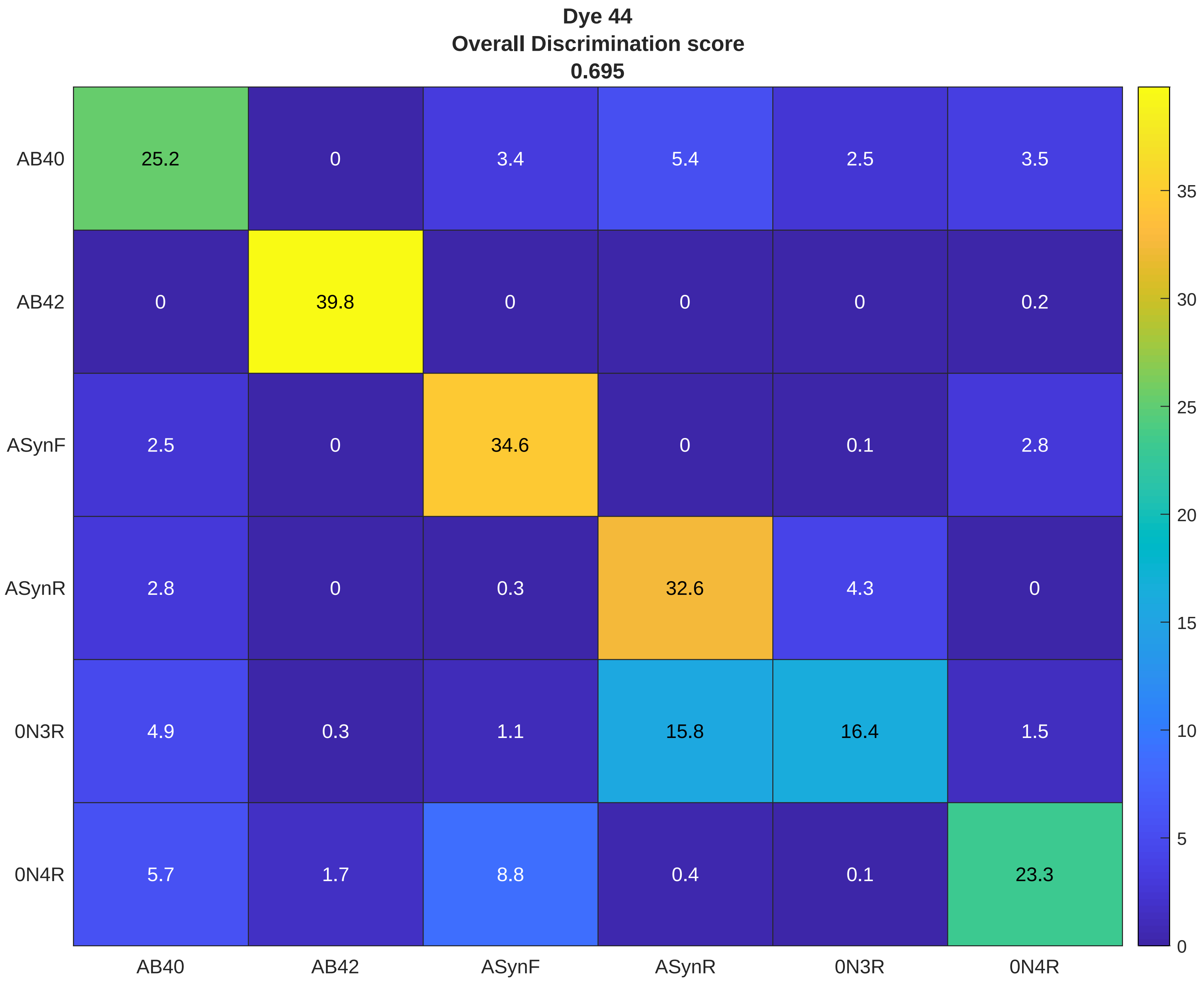
Dye 44
Overall Discrimination score
0.695
AB40 25.2 0 3.4 5.4 2.5 3.5
AB42 0 39.8 0 0 0 0.2
ASynF 2.5 0 34.6 0 0.1 2.8
ASynR 2.8 0 0.3 32.6 4.3 0
0N3R 4.9 0.3 1.1 15.8 16.4 1.5
0N4R 5.7 1.7 8.8 0.4 0.1 23.3
AB40
AB42
ASynF
ASynR
0N3R
0N4R
35
30
25
20
15
10
5
0

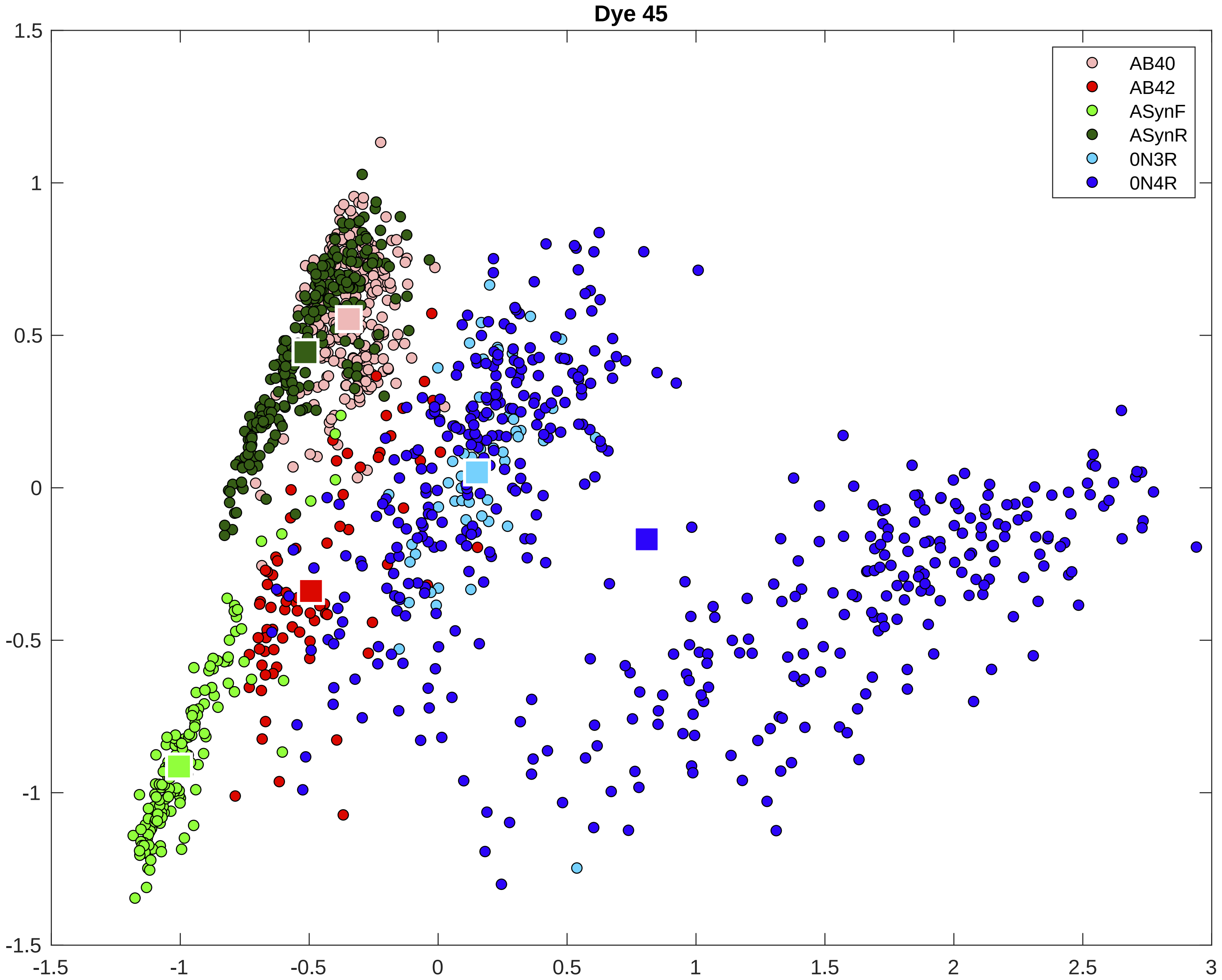

Dye 45
AB40
AB42
ASynF
ASynR
0N3R
0N4R
1.5
1
0.5
0
-0.5
-1
-1.5
-1.5
-1
-0.5
0
0.5
1
1.5
2
2.5
3

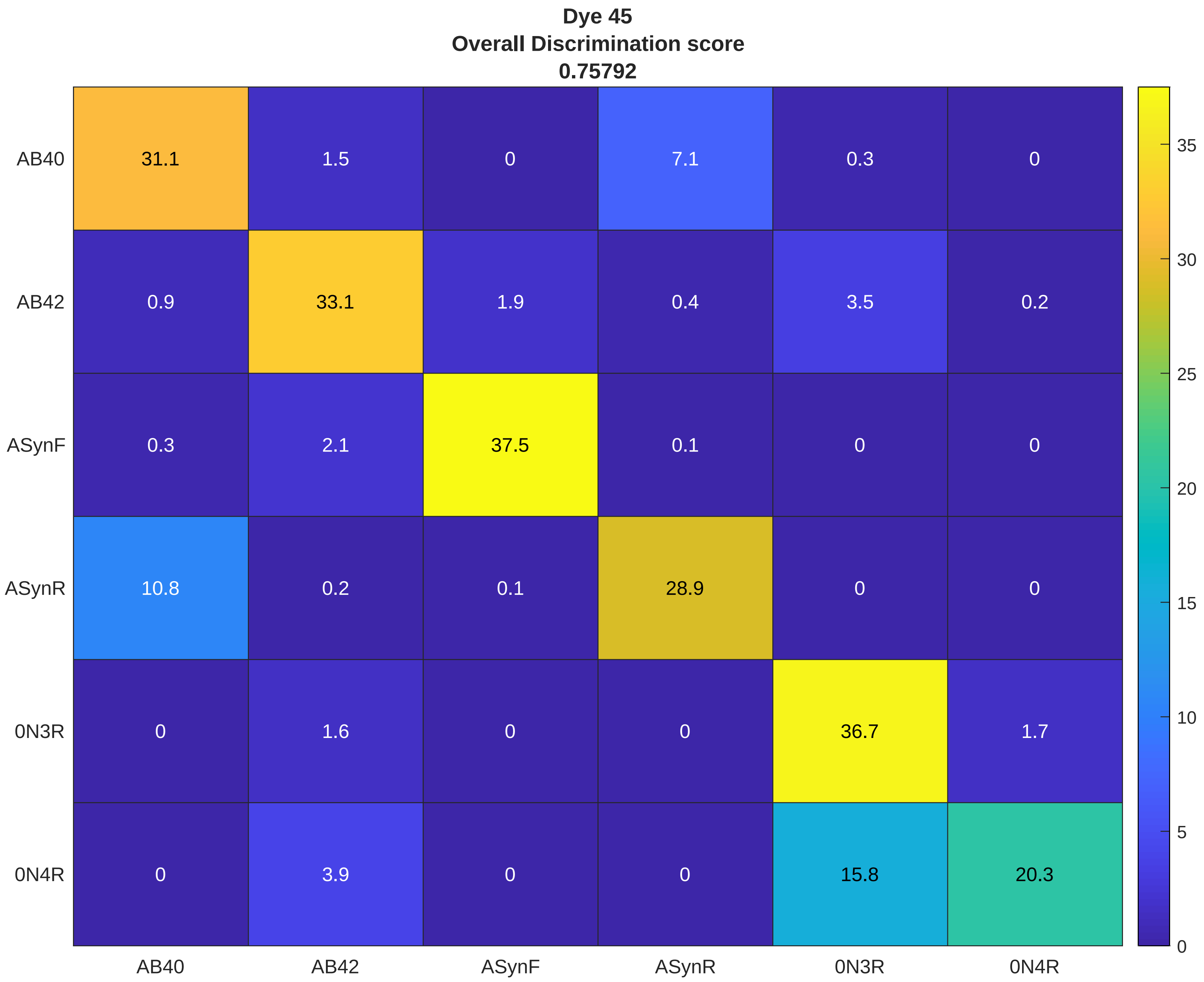
Dye 45
Overall Discrimination score
0.75792
AB40
AB42
ASynF
ASynR
0N3R
0N4R
31.1
1.5
0
7.1
0.3
0
0.9
33.1
1.9
0.4
3.5
0.2
0.3
2.1
37.5
0.1
0
0
10.8
0.2
0.1
28.9
0
0
0
1.6
0
0
36.7
1.7
0
3.9
0
0
15.8
20.3
AB40
AB42
ASynF
ASynR
0N3R
0N4R
35
30
25
20
15
10
5
0

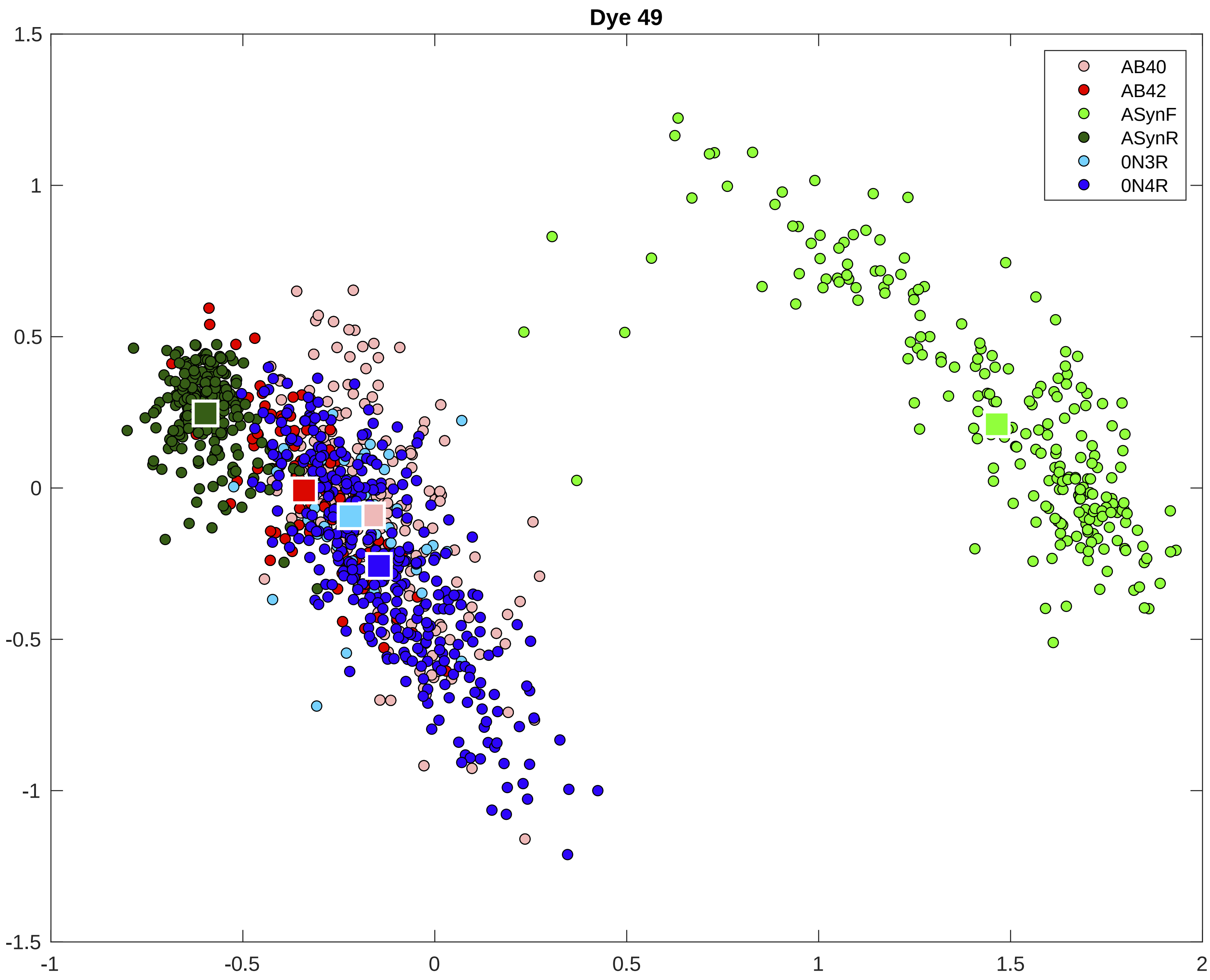

Dye 49
AB40
AB42
ASynF
ASynR
0N3R
0N4R
1.5
1
0.5
0
-0.5
-1
-1.5
-1
-0.5
0
0.5
1
1.5
2

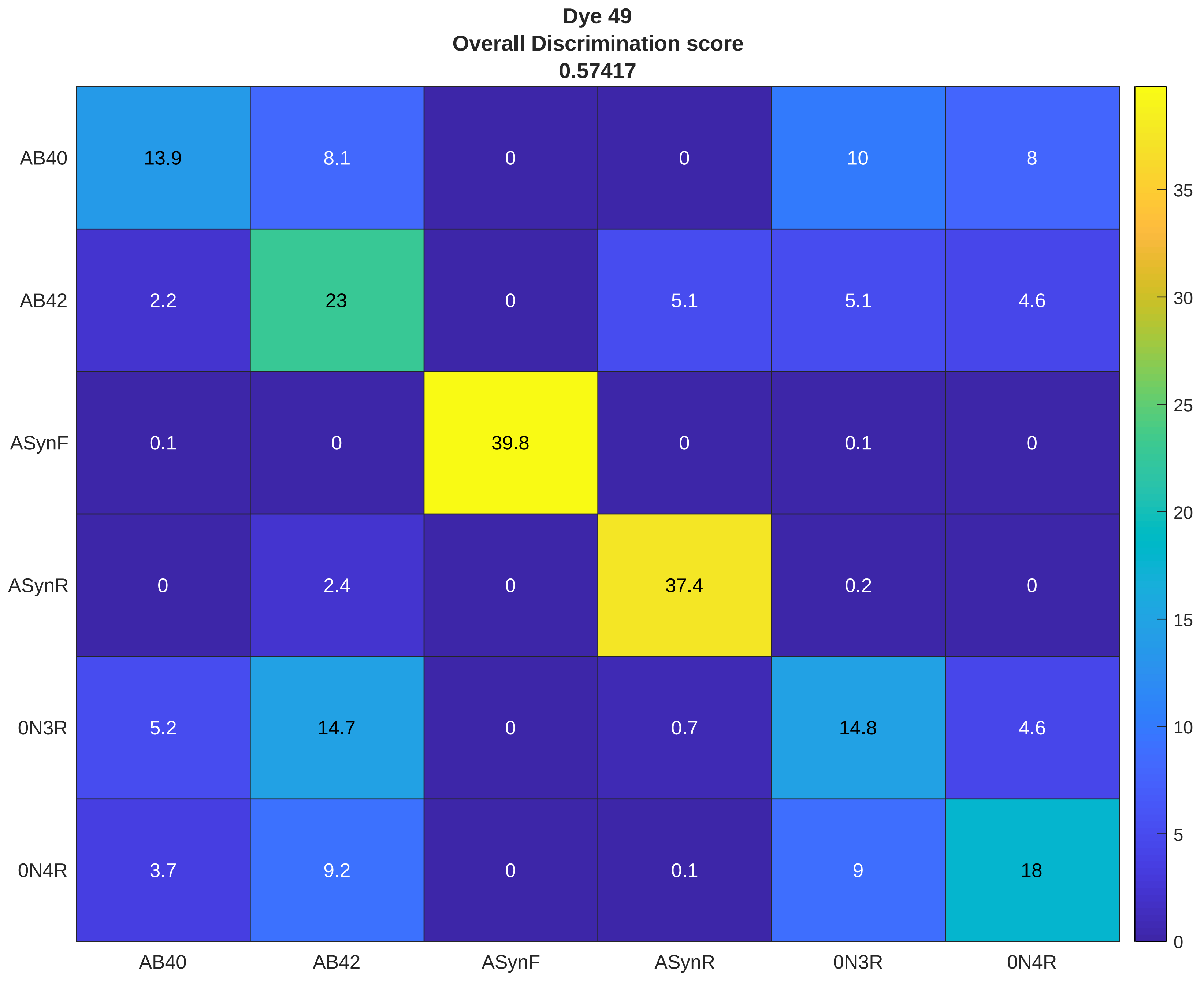
Dye 49
Overall Discrimination score
0.57417
AB40
AB42
ASynF
ASynR
0N3R
0N4R
13.9
8.1
0
0
10
8
2.2
23
0
5.1
5.1
4.6
0.1
0
39.8
0
0.1
0
0
2.4
0
37.4
0.2
0
5.2
14.7
0
0.7
14.8
4.6
3.7
9.2
0
0.1
9
18
AB40
AB42
ASynF
ASynR
0N3R
0N4R
35
30
25
20
15
10
5
0

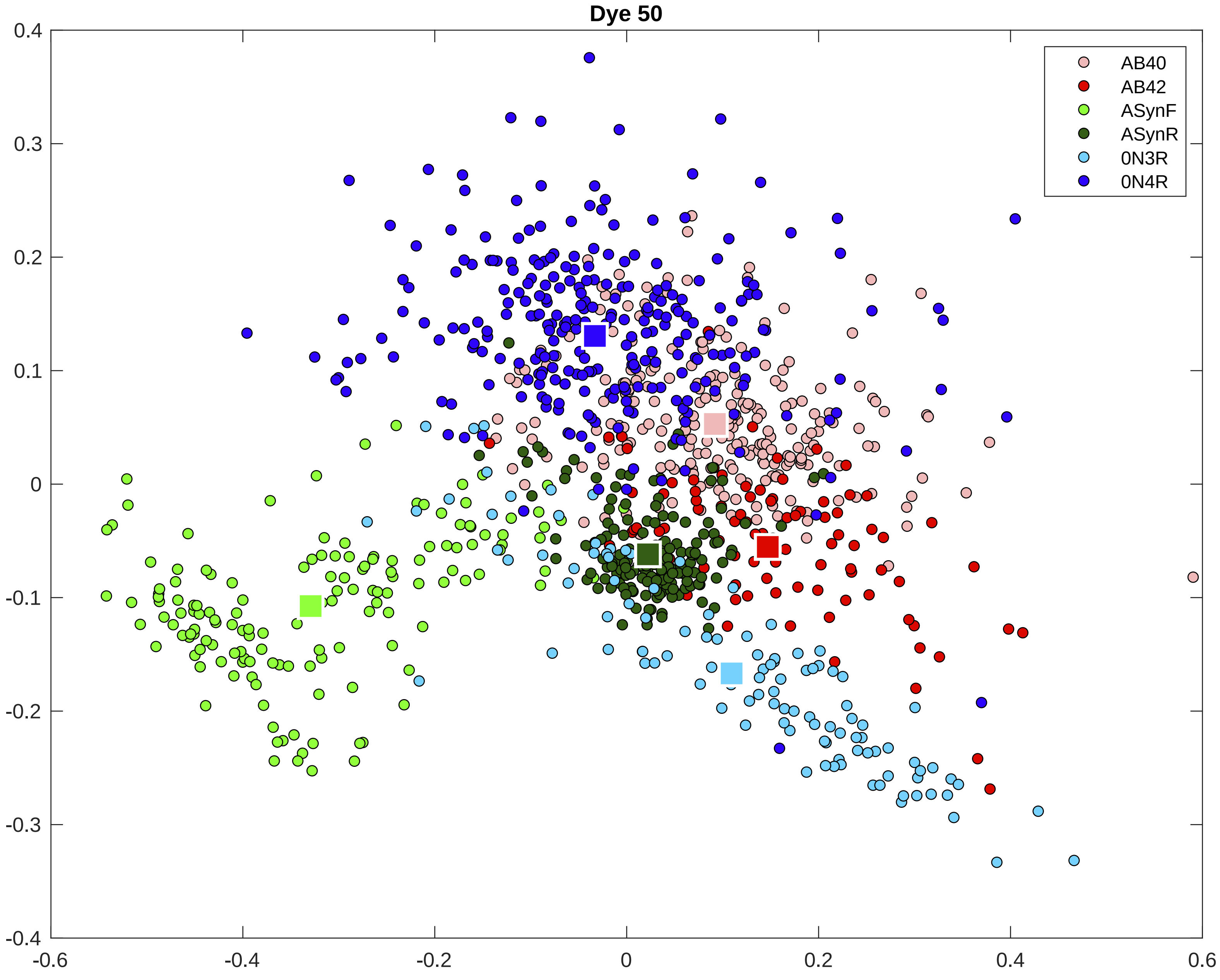

Dye 50
AB40
AB42
ASynF
ASynR
0N3R
0N4R
0.4
0.3
0.2
0.1
0
-0.1
-0.2
-0.3
-0.4
-0.6
-0.4
-0.2
0
0.2
0.4
0.6

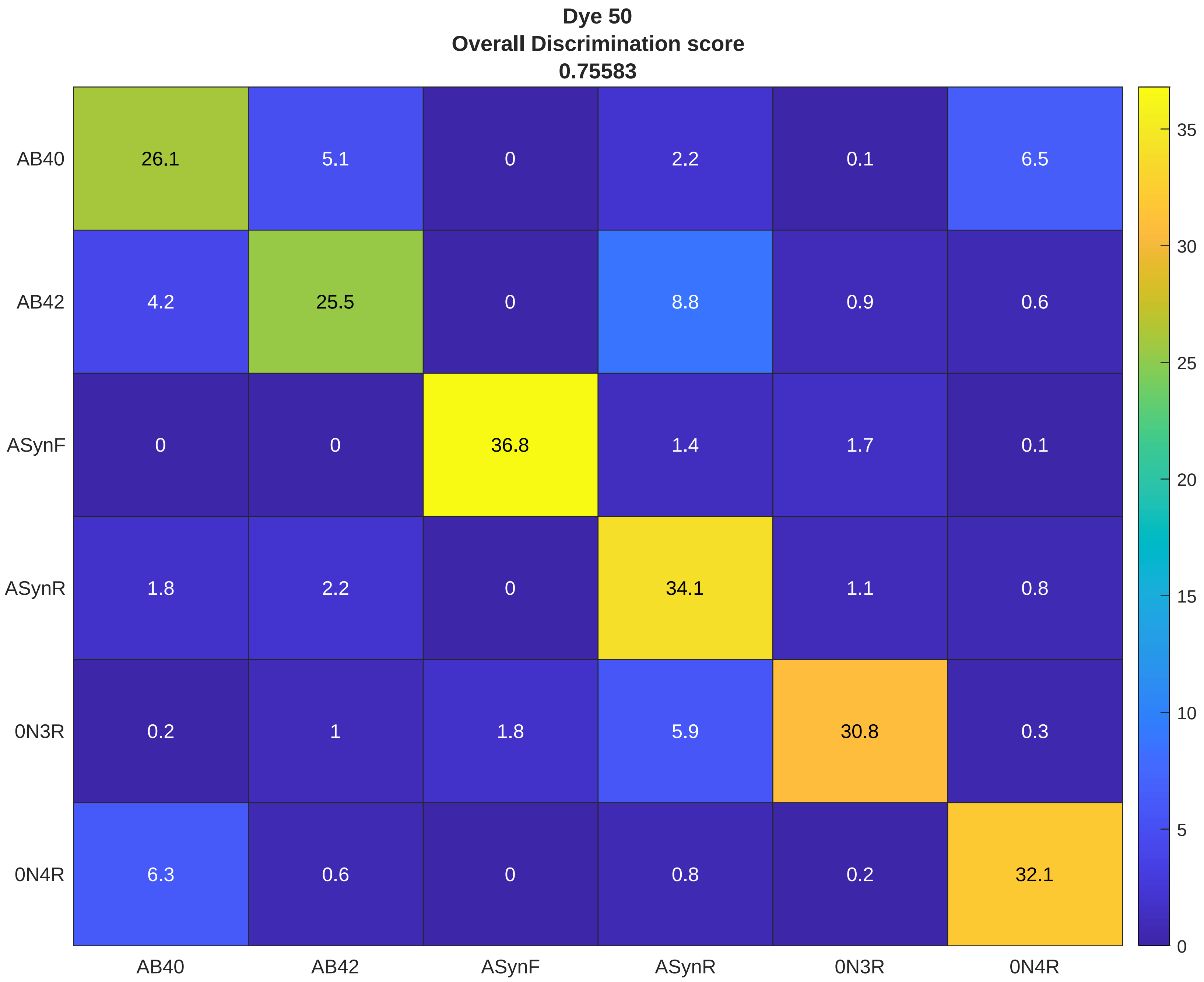

Dye 50
Overall Discrimination score
0.75583
AB40
AB42
ASynF
ASynR
0N3R
0N4R
26.1
5.1
0
2.2
0.1
6.5
4.2
25.5
0
8.8
0.9
0.6
0
0
36.8
1.4
1.7
0.1
1.8
2.2
0
34.1
1.1
0.8
0.2
1
1.8
5.9
30.8
0.3
6.3
0.6
0
0.8
0.2
32.1
AB40
AB42
ASynF
ASynR
0N3R
0N4R
35
30
25
20
15
10
5
0

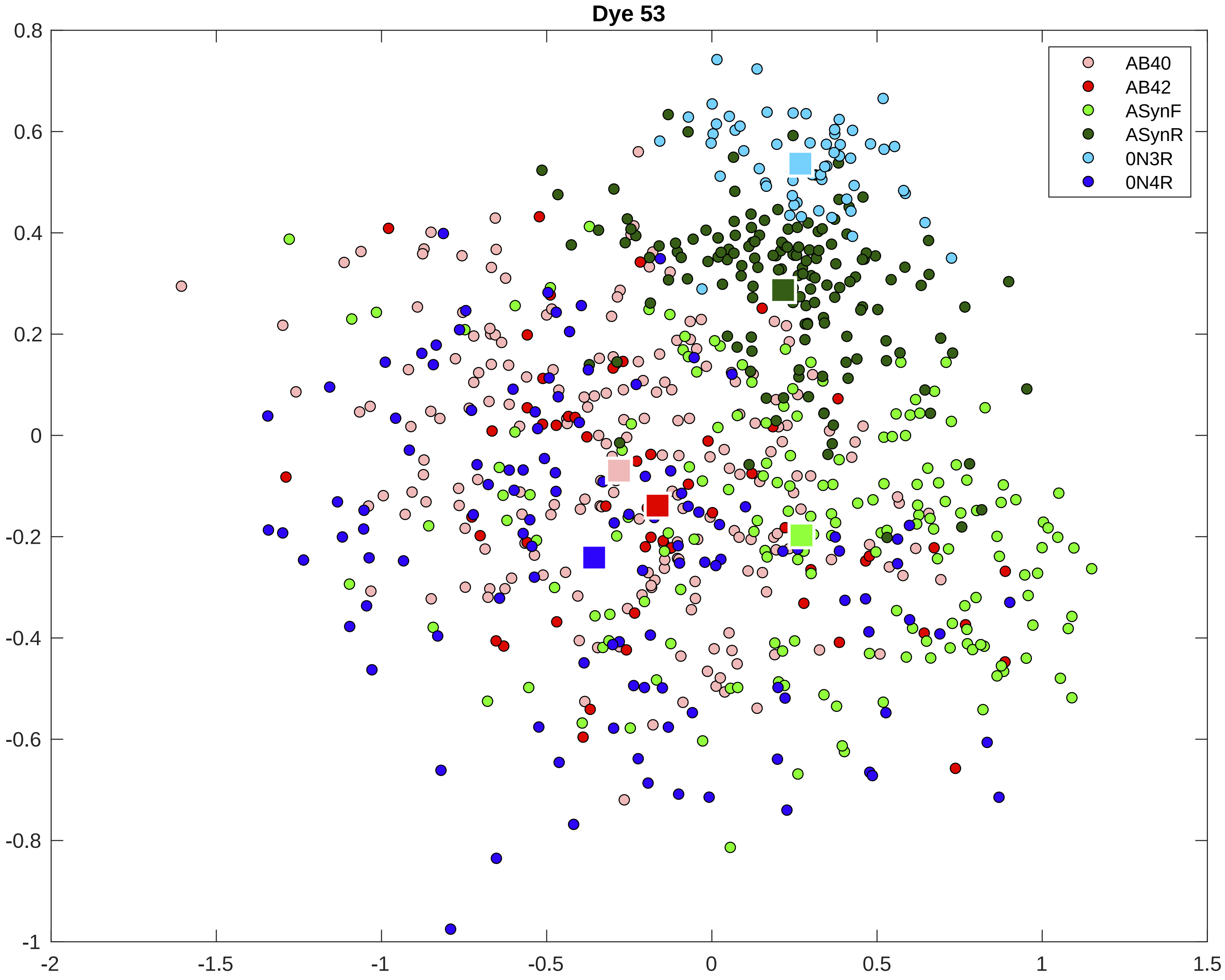

Dye 53
AB40
AB42
ASynF
ASynR
0N3R
0N4R
0.8
0.6
0.4
0.2
0
-0.2
-0.4
-0.6
-0.8
-1
-2
-1.5
-1
-0.5
0
0.5
1
1.5

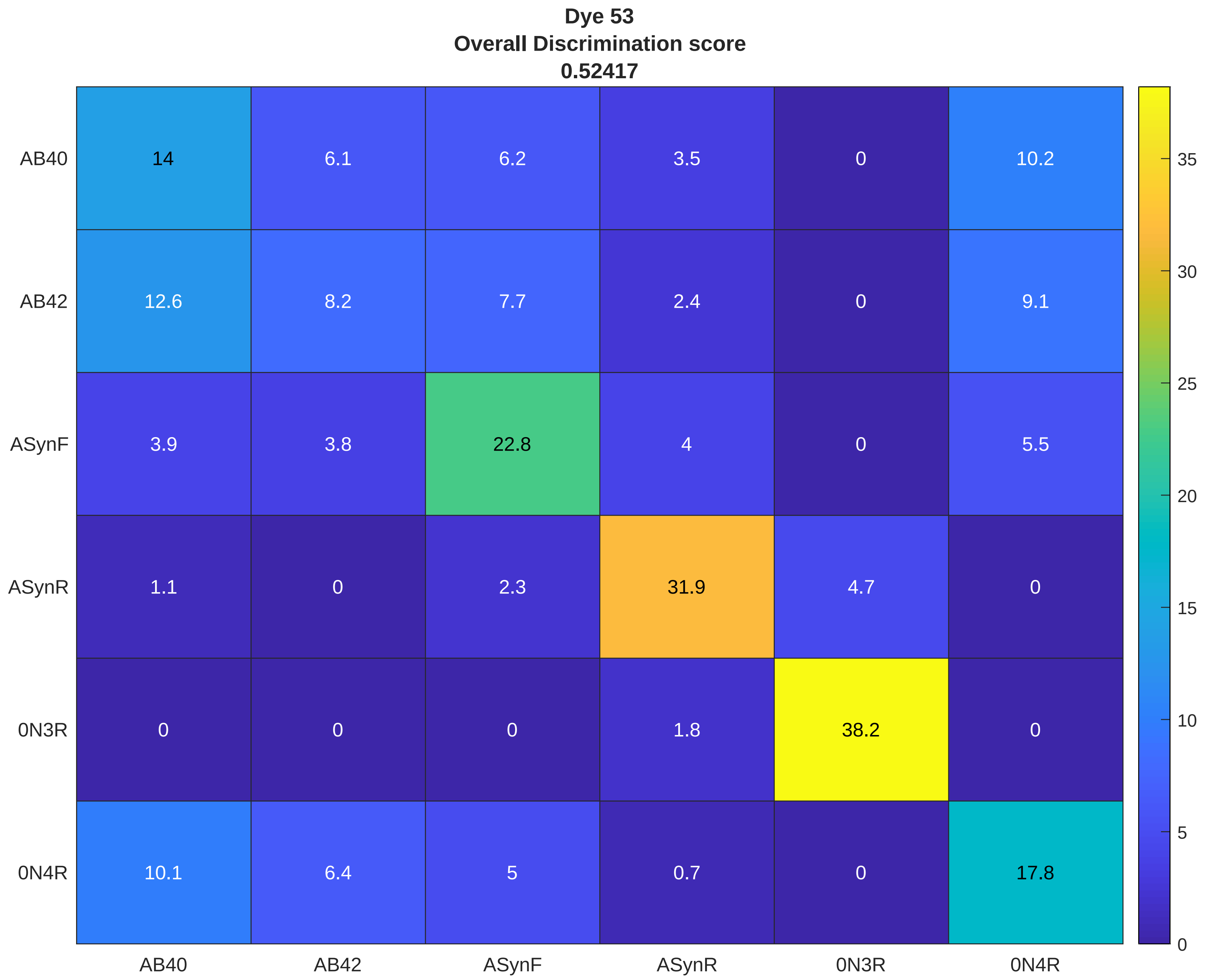
Dye 53
Overall Discrimination score
0.52417
AB40
AB42
ASynF
ASynR
0N3R
0N4R
14
6.1
6.2
3.5
0
10.2
12.6
8.2
7.7
2.4
0
9.1
3.9
3.8
22.8
4
0
5.5
1.1
0
2.3
31.9
4.7
0
0
0
0
1.8
38.2
0
10.1
6.4
5
0.7
0
17.8
AB40
AB42
ASynF
ASynR
0N3R
0N4R
35
30
25
20
15
10
5
0

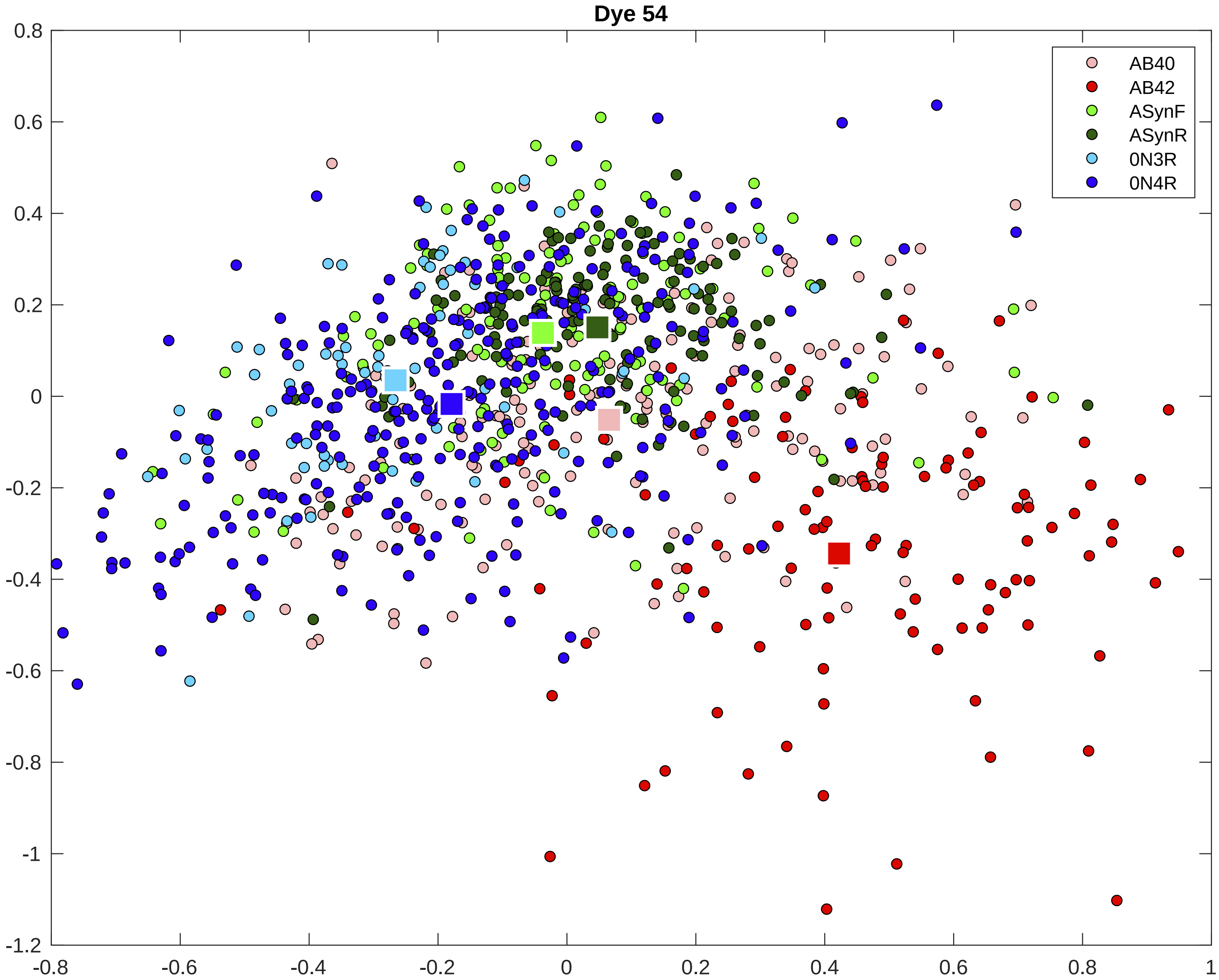
Dye 54
AB40
AB42
ASynF
ASynR
0N3R
0N4R
0.8
0.6
0.4
0.2
0
-0.2
-0.4
-0.6
-0.8
-1
-1.2
-0.8
-0.6
-0.4
-0.2
0
0.2
0.4
0.6
0.8
1

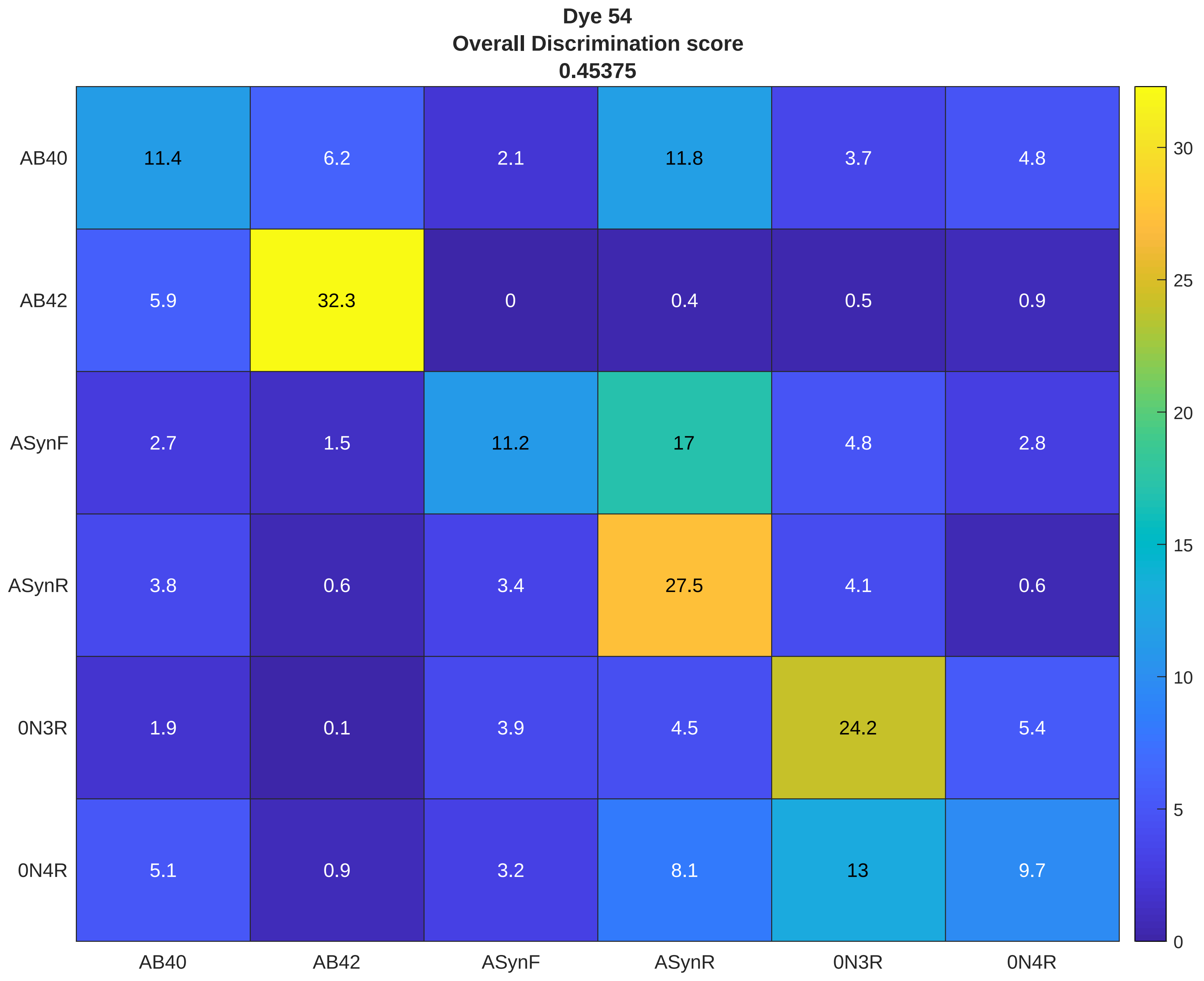

Dye 54
Overall Discrimination score
0.45375
AB40
AB42
ASynF
ASynR
0N3R
0N4R
AB40 11.4 6.2 2.1 11.8 3.7 4.8
AB42 5.9 32.3 0 0.4 0.5 0.9
ASynF 2.7 1.5 11.2 17 4.8 2.8
ASynR 3.8 0.6 3.4 27.5 4.1 0.6
0N3R 1.9 0.1 3.9 4.5 24.2 5.4
0N4R 5.1 0.9 3.2 8.1 13 9.7
30
25
20
15
10
5
0

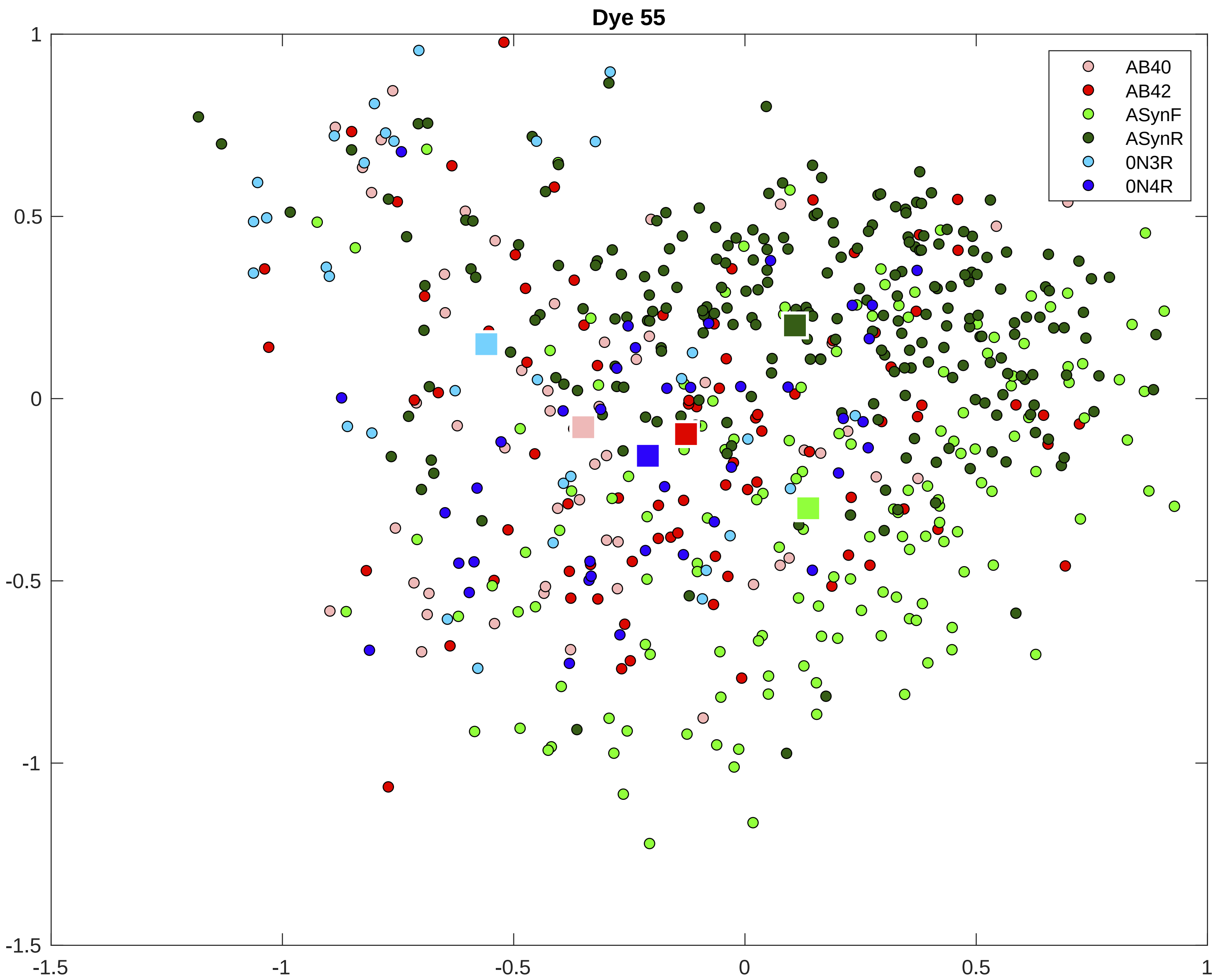

Dye 55
AB40
AB42
ASynF
ASynR
0N3R
0N4R
1
0.5
0
-0.5
-1
-1.5
-1.5
-1
-0.5
0
0.5
1

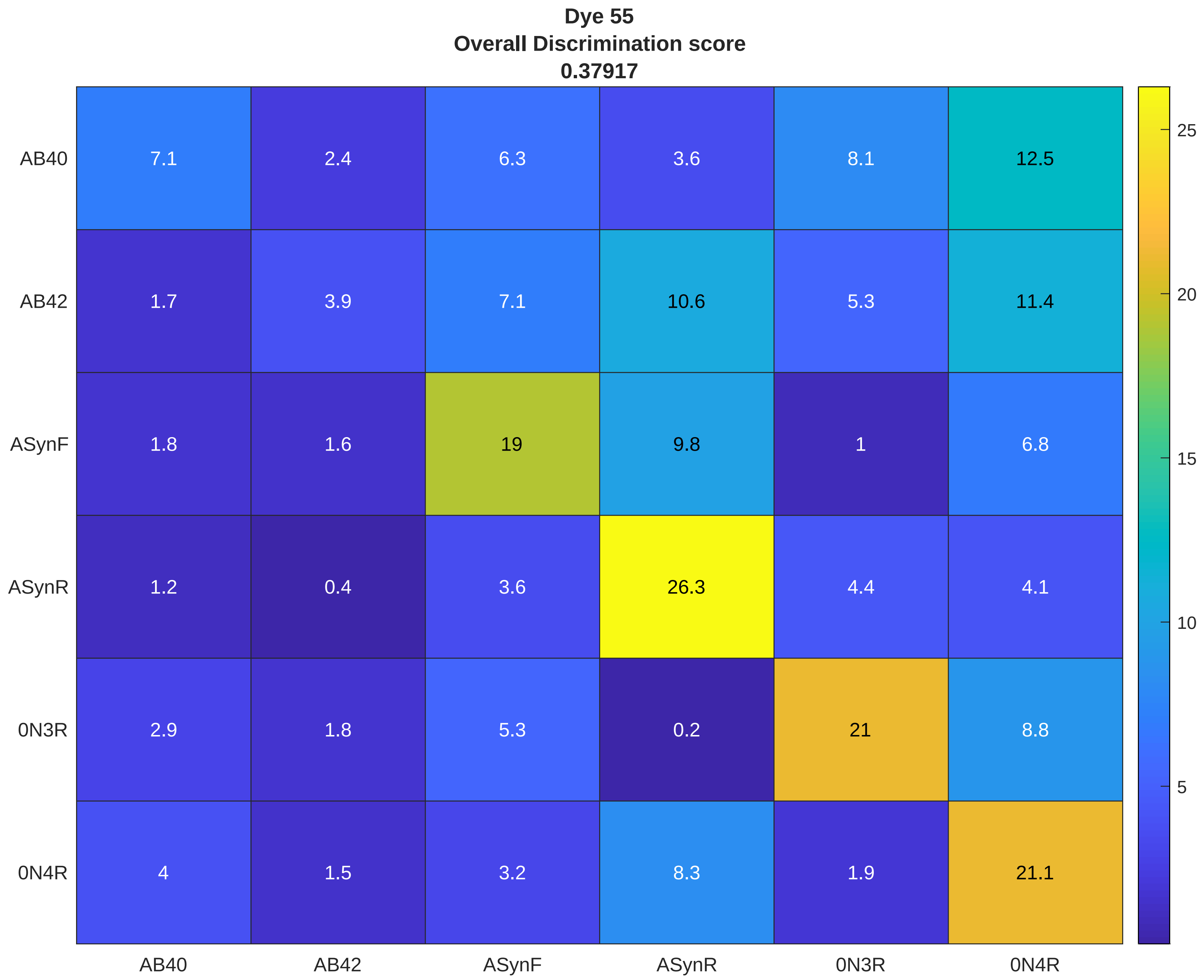
Dye 55
Overall Discrimination score
0.37917
AB40
AB42
ASynF
ASynR
0N3R
0N4R
7.1
2.4
6.3
3.6
8.1
12.5
1.7
3.9
7.1
10.6
5.3
11.4
1.8
1.6
19
9.8
1
6.8
1.2
0.4
3.6
26.3
4.4
4.1
2.9
1.8
5.3
0.2
21
8.8
4
1.5
3.2
8.3
1.9
21.1
AB40
AB42
ASynF
ASynR
0N3R
0N4R
25
20
15
10
5

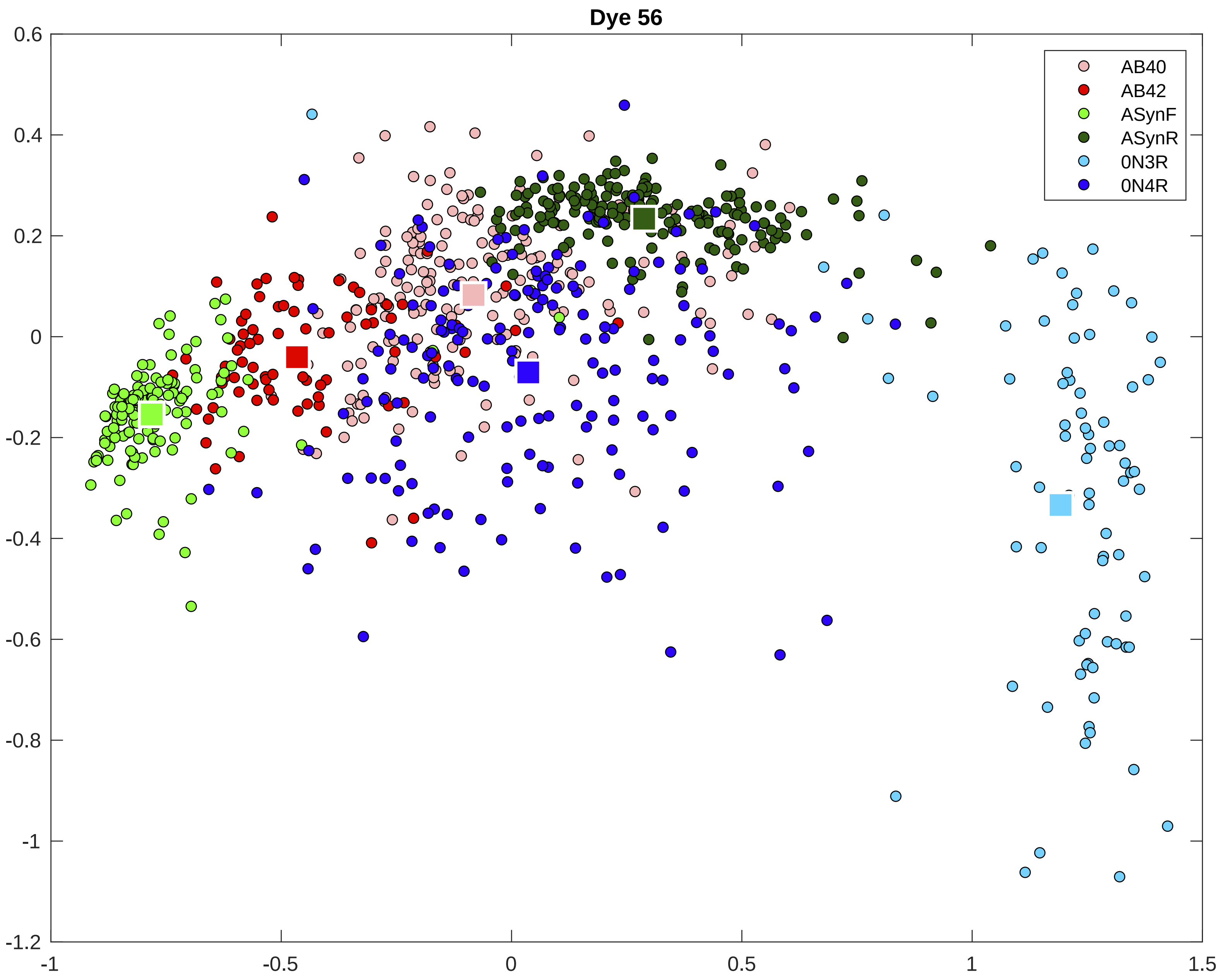
Dye 56
AB40
AB42
ASynF
ASynR
0N3R
0N4R
0.6
0.4
0.2
0
-0.2
-0.4
-0.6
-0.8
-1
-1.2
-1
-0.5
0
0.5
1
1.5

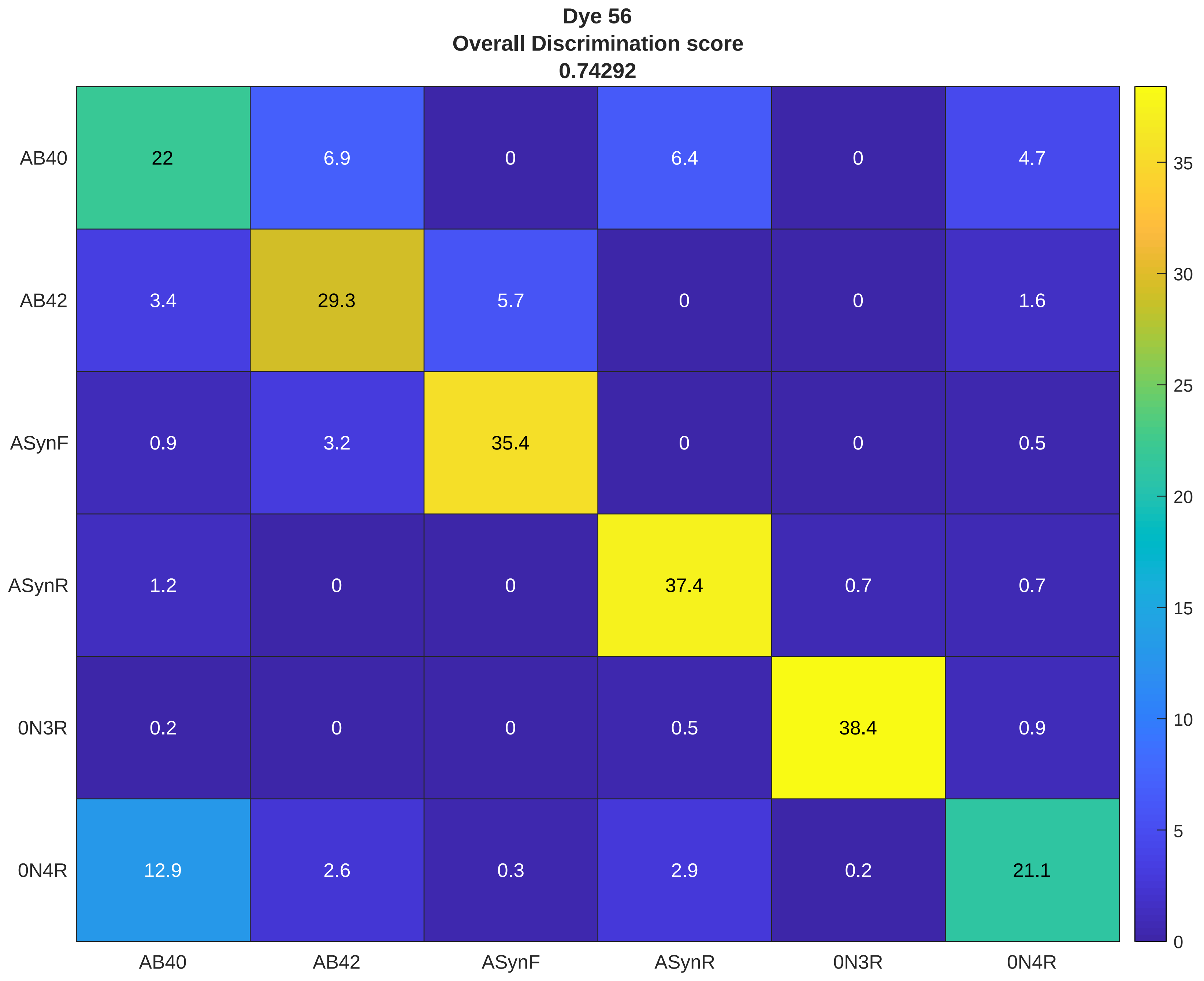
Dye 56
Overall Discrimination score
0.74292
AB40
AB42
ASynF
ASynR
0N3R
0N4R
22
6.9
0
6.4
0
4.7
3.4
29.3
5.7
0
0
1.6
0.9
3.2
35.4
0
0
0.5
1.2
0
0
37.4
0.7
0.7
0.2
0
0
0.5
38.4
0.9
12.9
2.6
0.3
2.9
0.2
21.1
AB40
AB42
ASynF
ASynR
0N3R
0N4R
35
30
25
20
15
10
5
0

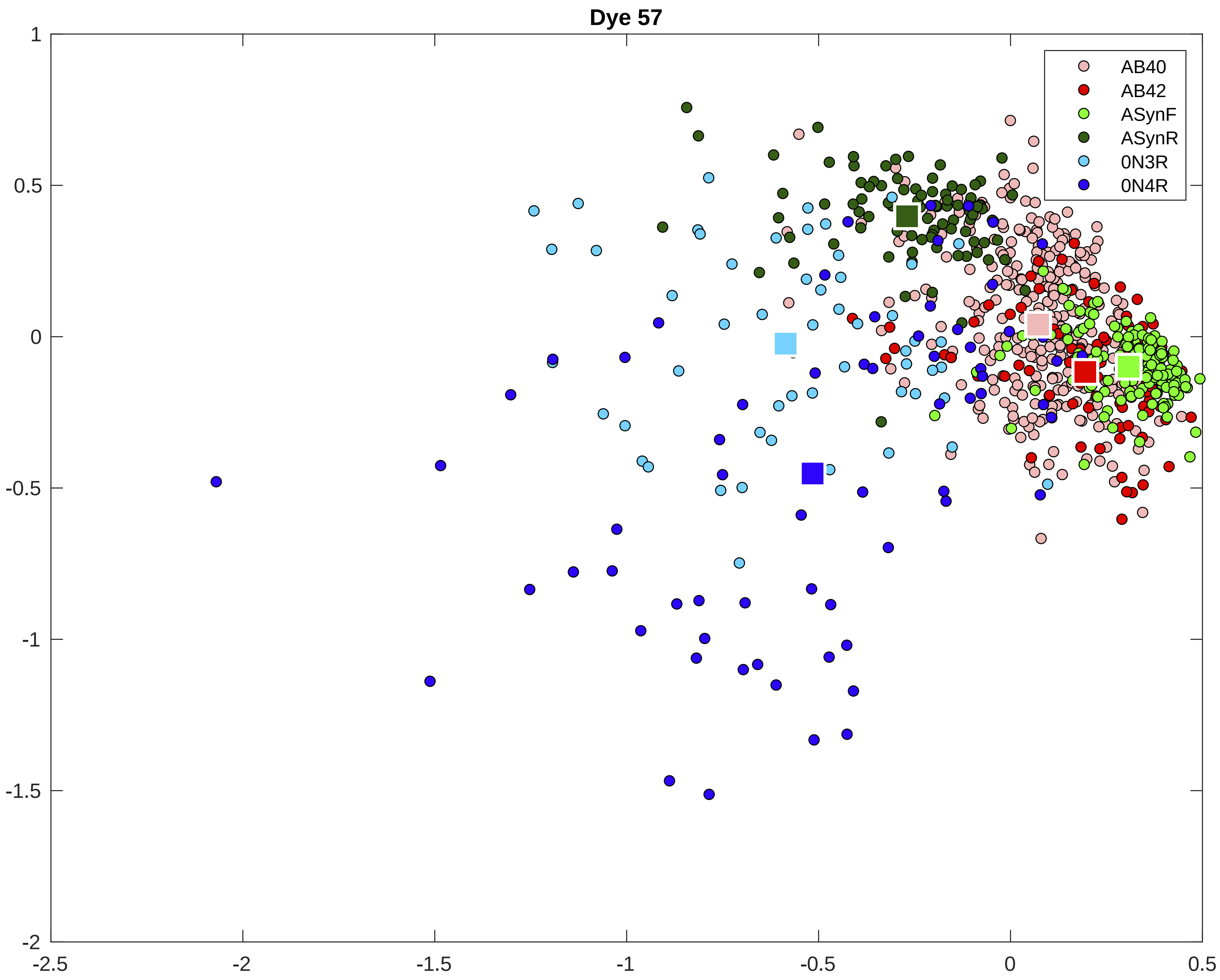

Dye 57
AB40
AB42
ASynF
ASynR
0N3R
0N4R
1
0.5
0
-0.5
-1
-1.5
-2
-2.5
-2
-1.5
-1
-0.5
0
0.5

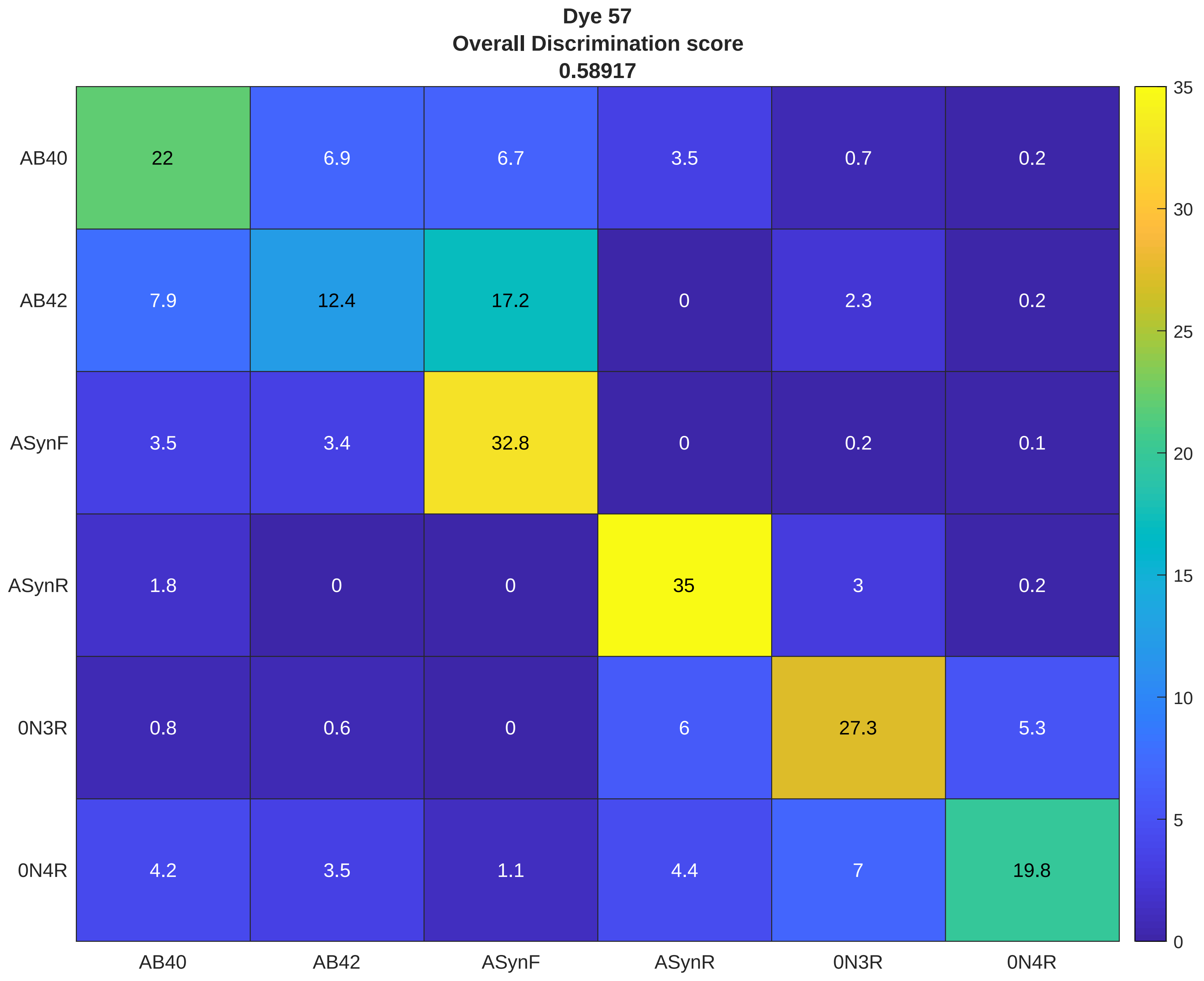
Dye 57
Overall Discrimination score
0.58917
AB40
AB42
ASynF
ASynR
0N3R
0N4R
22
6.9
6.7
3.5
0.7
0.2
7.9
12.4
17.2
0
2.3
0.2
3.5
3.4
32.8
0
0.2
0.1
1.8
0
0
35
3
0.2
0.8
0.6
0
6
27.3
5.3
4.2
3.5
1.1
4.4
7
19.8
AB40
AB42
ASynF
ASynR
0N3R
0N4R
35
30
25
20
15
10
5
0

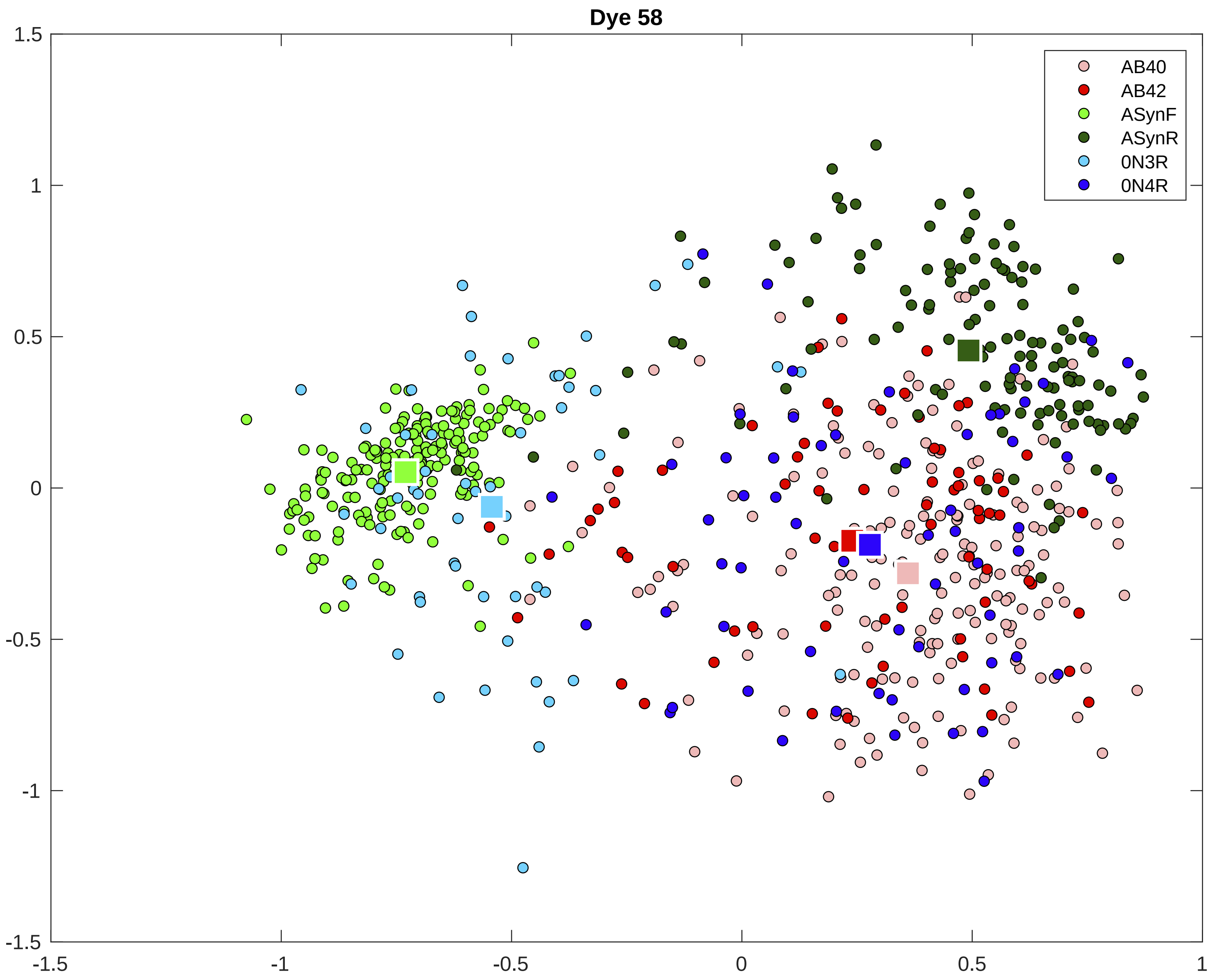

Dye 58
AB40
AB42
ASynF
ASynR
0N3R
0N4R
1.5
1
0.5
0
-0.5
-1
-1.5
-1.5
-1
-0.5
0
0.5
1

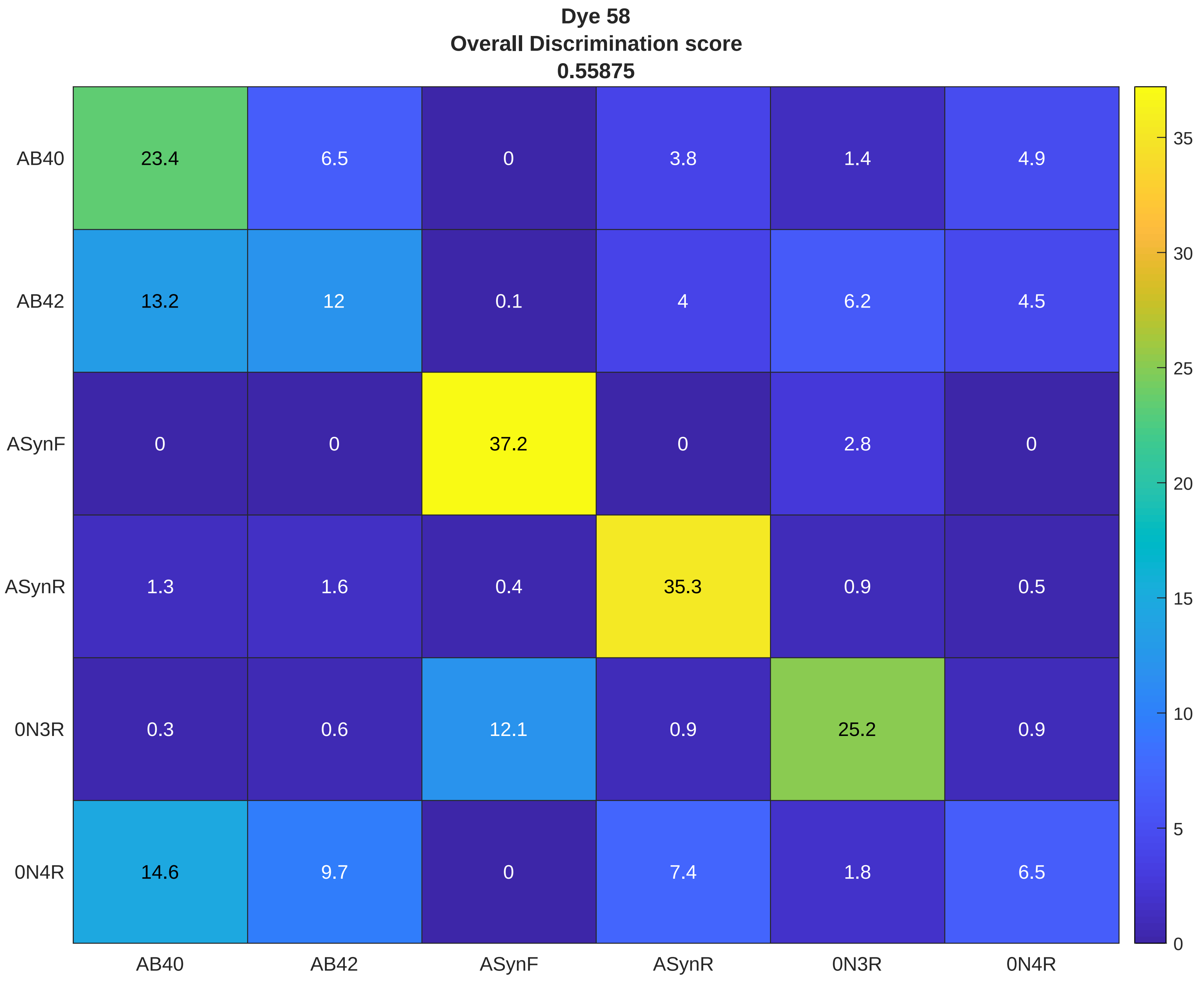
Dye 58
Overall Discrimination score
0.55875
AB40
AB42
ASynF
ASynR
0N3R
0N4R
23.4
6.5
0
3.8
1.4
4.9
13.2
12
0.1
4
6.2
4.5
0
0
37.2
0
2.8
0
1.3
1.6
0.4
35.3
0.9
0.5
0.3
0.6
12.1
0.9
25.2
0.9
14.6
9.7
0
7.4
1.8
6.5
AB40
AB42
ASynF
ASynR
0N3R
0N4R
35
30
25
20
15
10
5
0

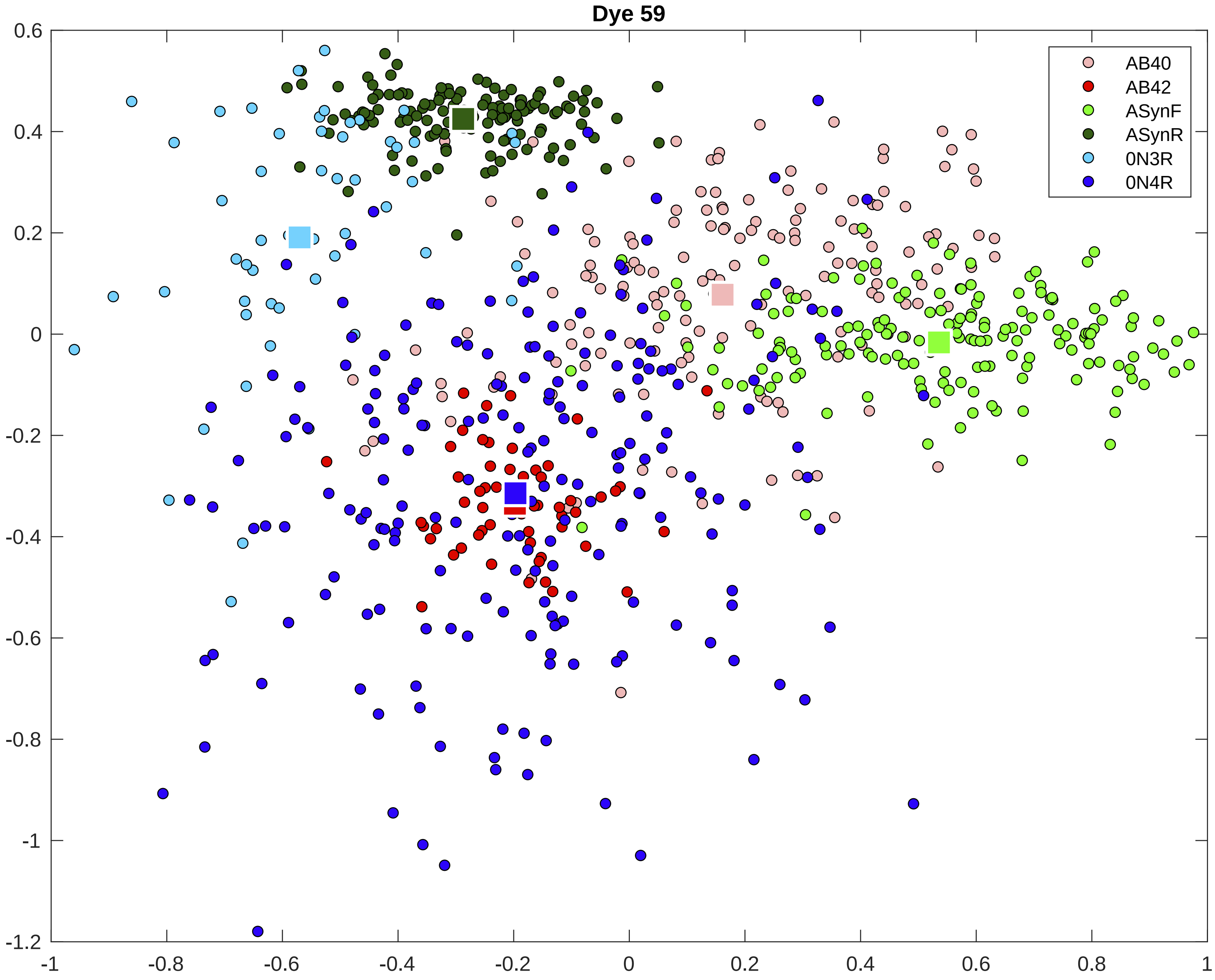

Dye 59
AB40
AB42
ASynF
ASynR
0N3R
0N4R
0.6
0.4
0.2
0
-0.2
-0.4
-0.6
-0.8
-1
-1.2
-1
-0.8
-0.6
-0.4
-0.2
0
0.2
0.4
0.6
0.8
1

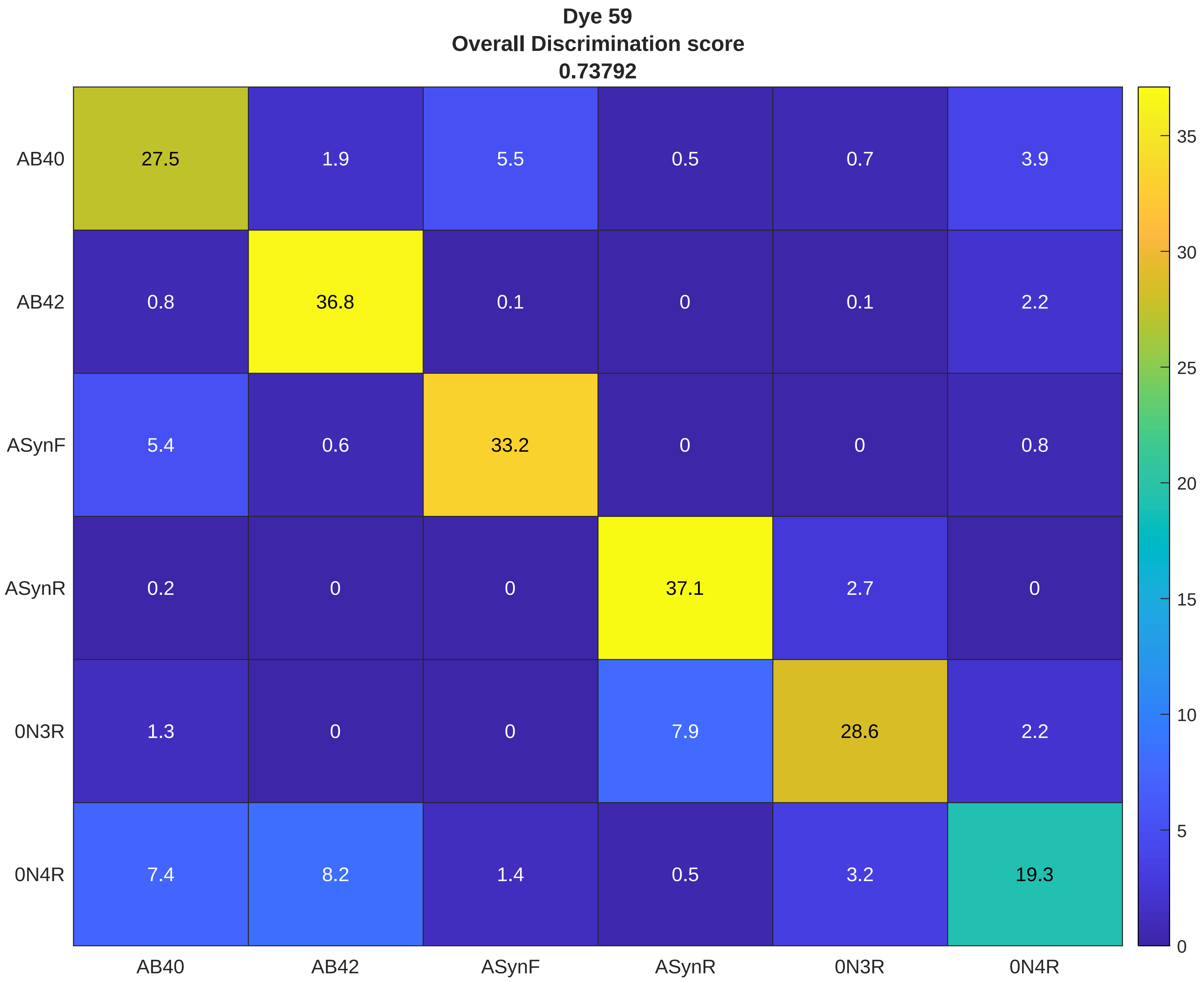
Dye 59
Overall Discrimination score
0.73792
AB40
AB42
ASynF
ASynR
0N3R
0N4R
27.5
1.9
5.5
0.5
0.7
3.9
0.8
36.8
0.1
0
0.1
2.2
5.4
0.6
33.2
0
0
0.8
0.2
0
0
37.1
2.7
0
1.3
0
0
7.9
28.6
2.2
7.4
8.2
1.4
0.5
3.2
19.3
AB40
AB42
ASynF
ASynR
0N3R
0N4R
35
30
25
20
15
10
5
0

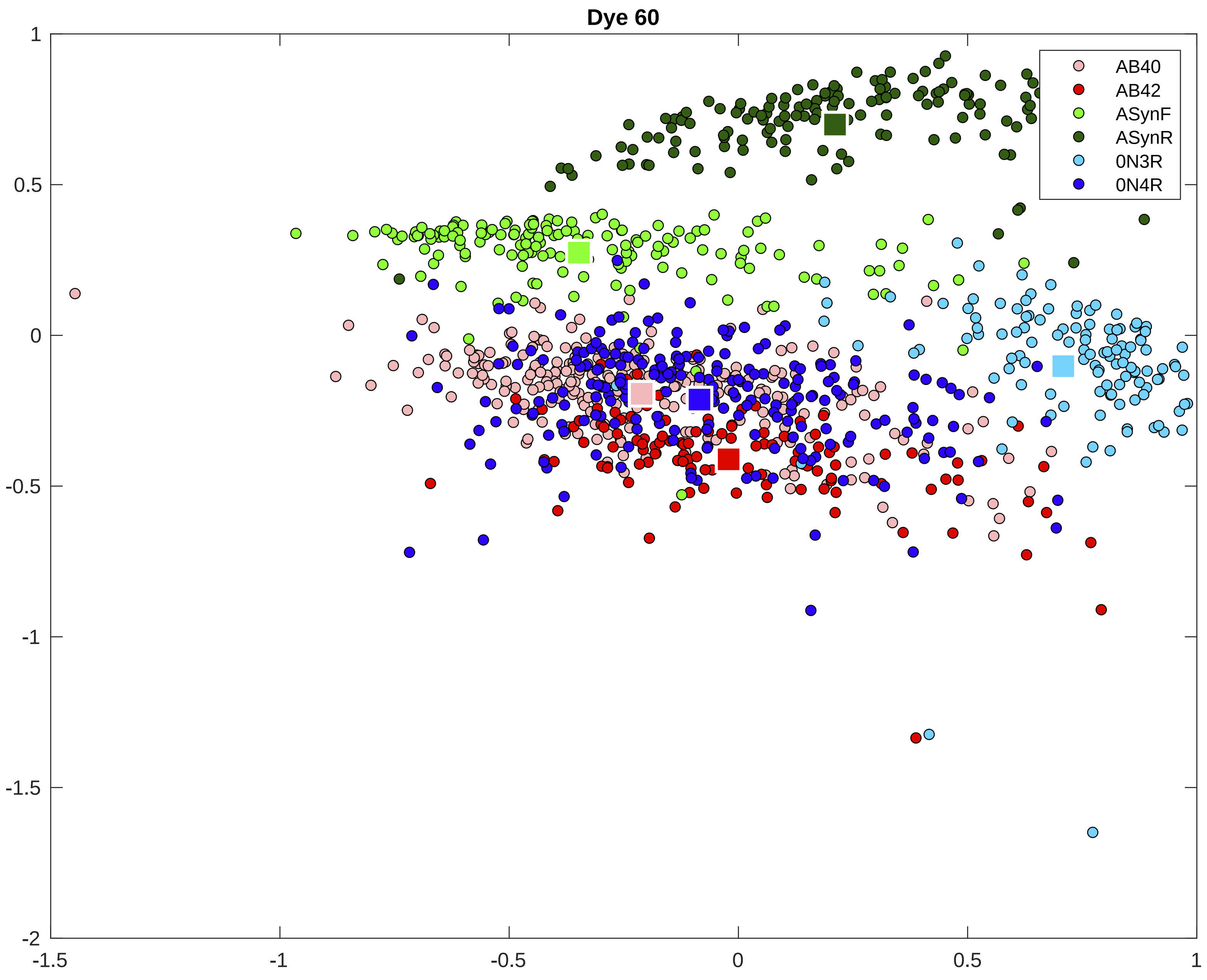

Dye 60
AB40
AB42
ASynF
ASynR
0N3R
0N4R
1
0.5
0
-0.5
-1
-1.5
-2
-1.5
-1
-0.5
0
0.5
1

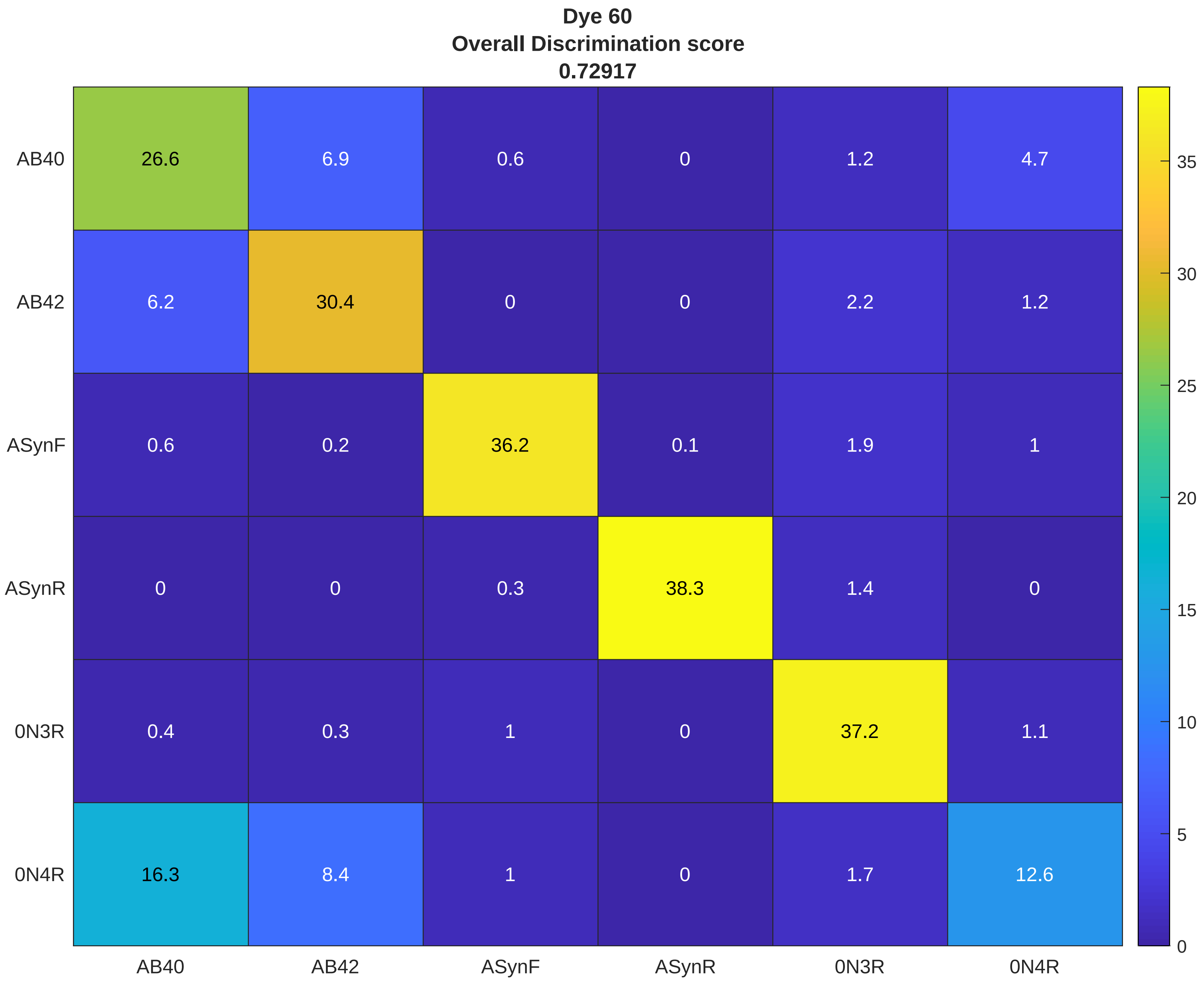
Dye 60
Overall Discrimination score
0.72917
AB40
AB42
ASynF
ASynR
0N3R
0N4R
26.6
6.9
0.6
0
1.2
4.7
6.2
30.4
0
0
2.2
1.2
0.6
0.2
36.2
0.1
1.9
1
0
0
0.3
38.3
1.4
0
0.4
0.3
1
0
37.2
1.1
16.3
8.4
1
0
1.7
12.6
AB40
AB42
ASynF
ASynR
0N3R
0N4R
35
30
25
20
15
10
5
0

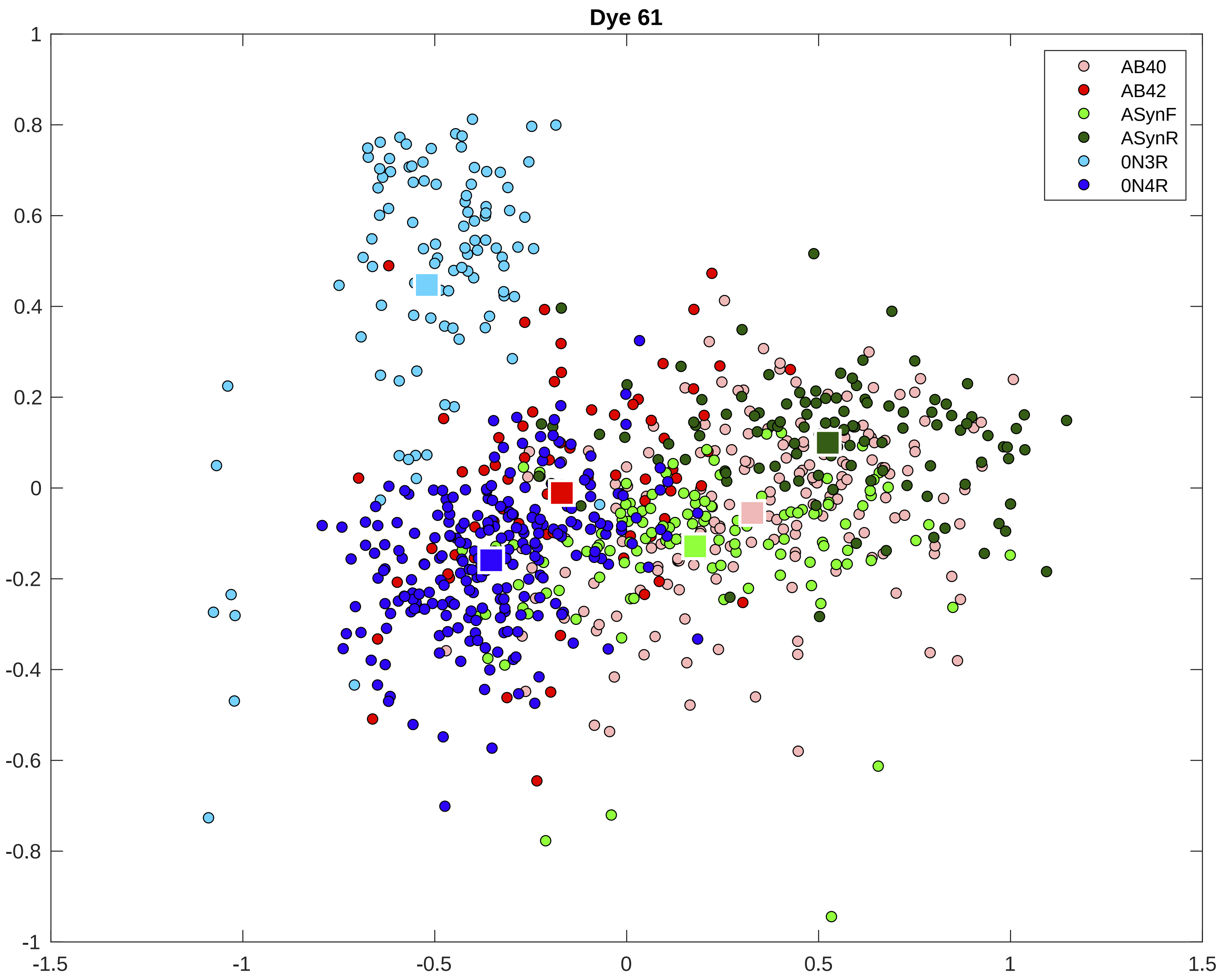
Dye 61
AB40
AB42
ASynF
ASynR
0N3R
0N4R
1
0.8
0.6
0.4
0.2
0
-0.2
-0.4
-0.6
-0.8
-1
-1.5
-1
-0.5
0
0.5
1
1.5

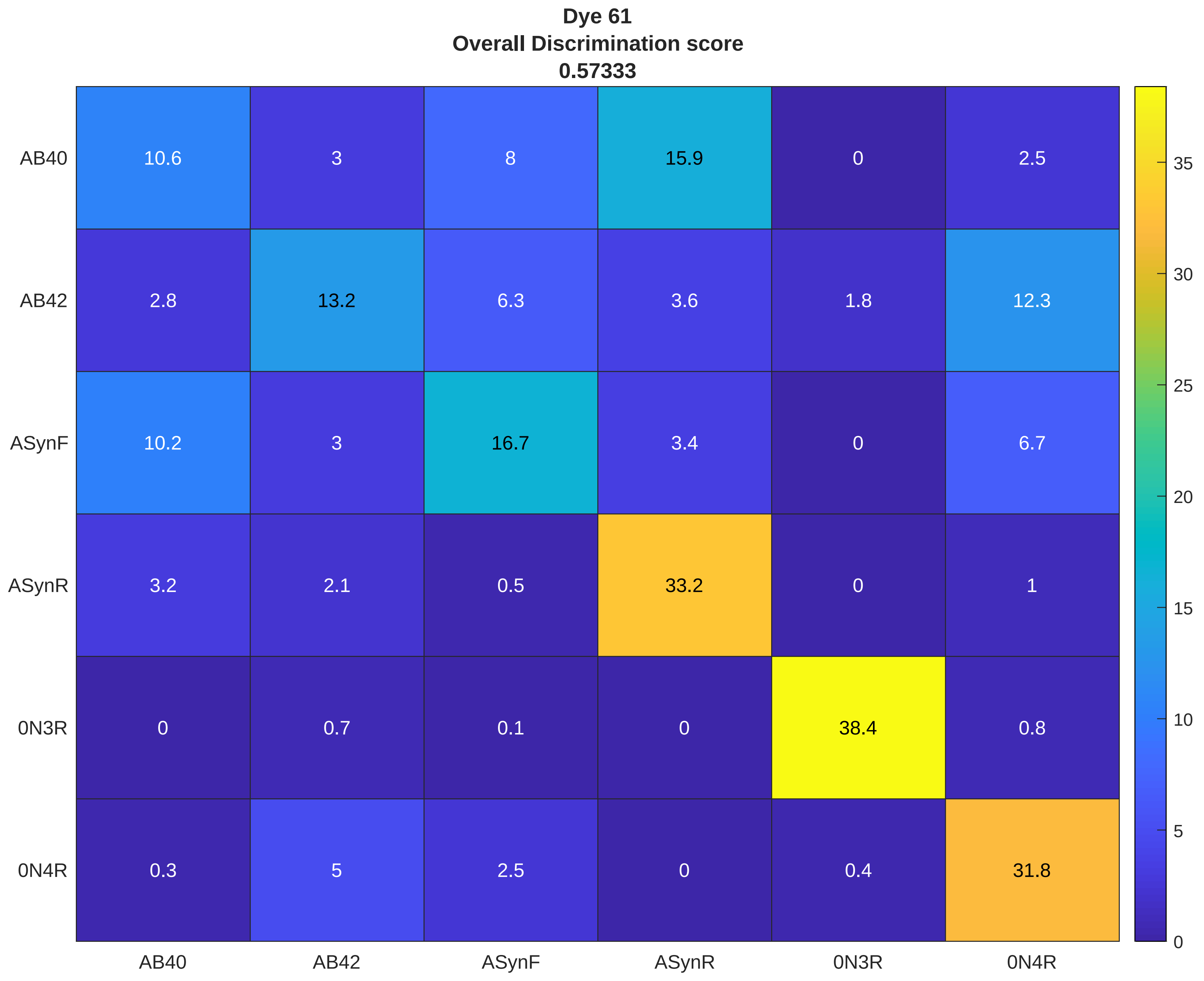
Dye 61
Overall Discrimination score
0.57333
AB40
AB42
ASynF
ASynR
0N3R
0N4R
10.6
3
8
15.9
0
2.5
2.8
13.2
6.3
3.6
1.8
12.3
10.2
3
16.7
3.4
0
6.7
3.2
2.1
0.5
33.2
0
1
0
0.7
0.1
0
38.4
0.8
0.3
5
2.5
0
0.4
31.8
AB40
AB42
ASynF
ASynR
0N3R
0N4R
35
30
25
20
15
10
5
0

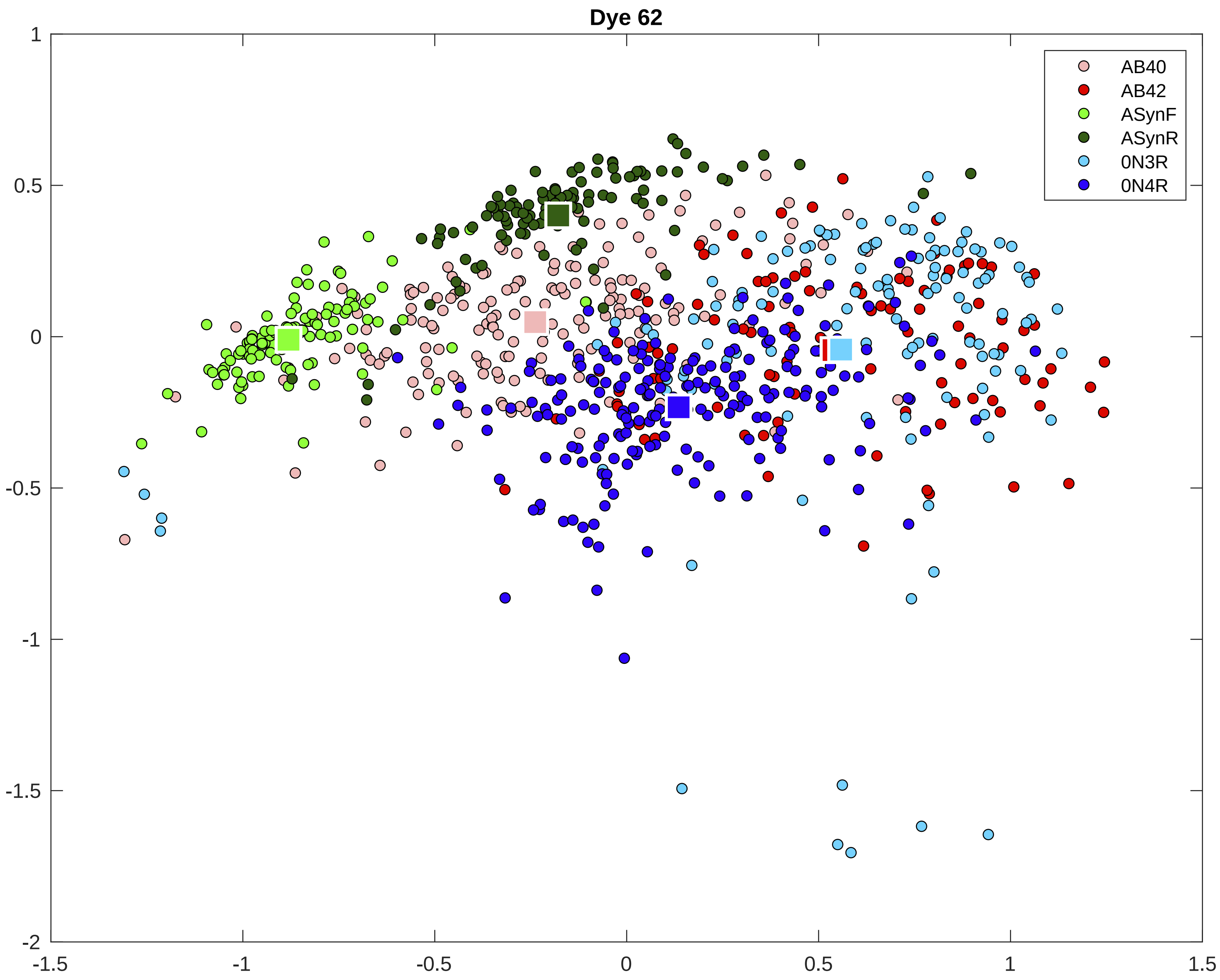

Dye 62
AB40
AB42
ASynF
ASynR
0N3R
0N4R
1
0.5
0
-0.5
-1
-1.5
-2
-1.5
-1
-0.5
0
0.5
1
1.5

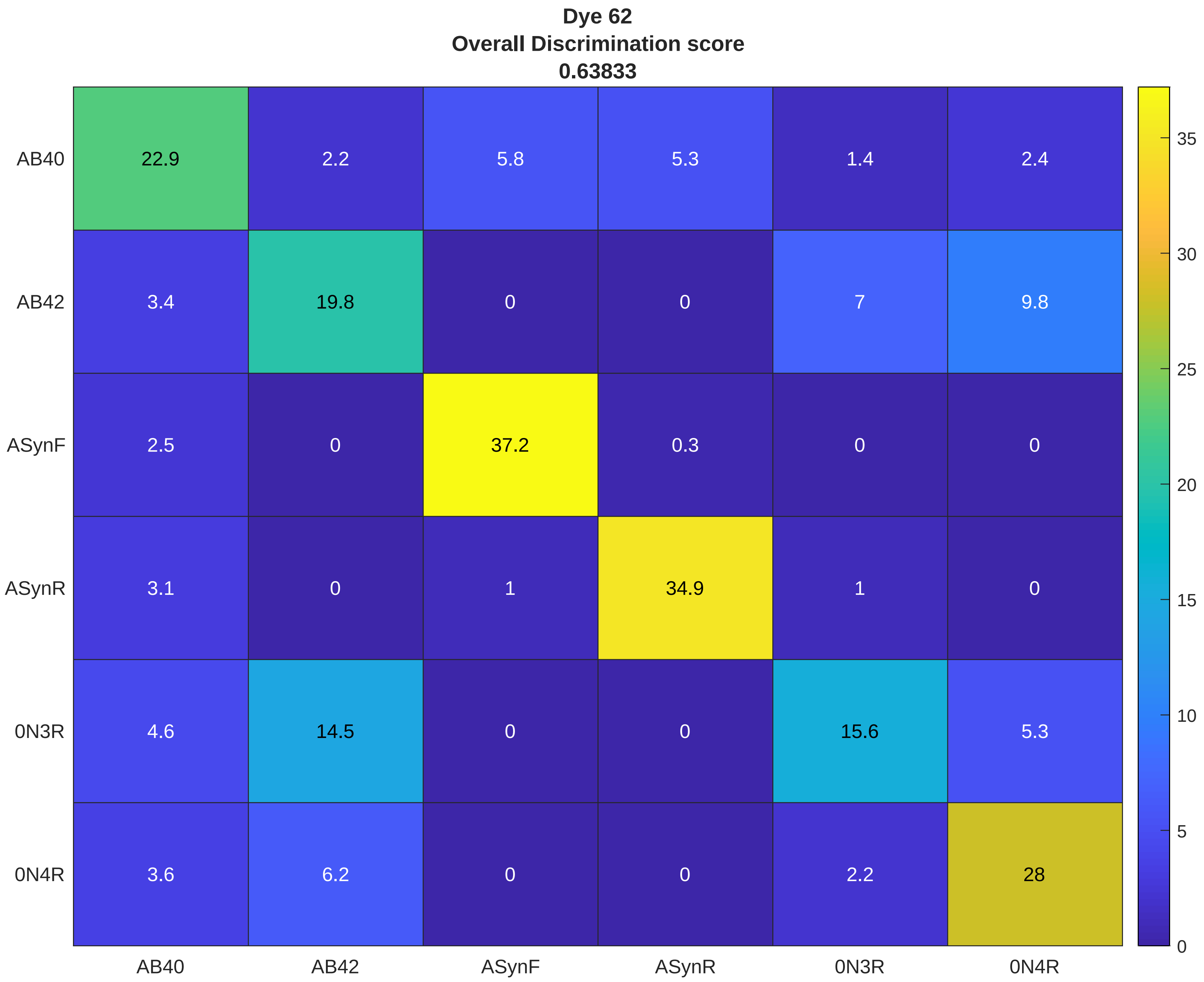
Dye 62
Overall Discrimination score
0.63833
AB40
AB42
ASynF
ASynR
0N3R
0N4R
22.9
2.2
5.8
5.3
1.4
2.4
3.4
19.8
0
0
7
9.8
2.5
0
37.2
0.3
0
0
3.1
0
1
34.9
1
0
4.6
14.5
0
0
15.6
5.3
3.6
6.2
0
0
2.2
28
AB40
AB42
ASynF
ASynR
0N3R
0N4R
35
30
25
20
15
10
5
0

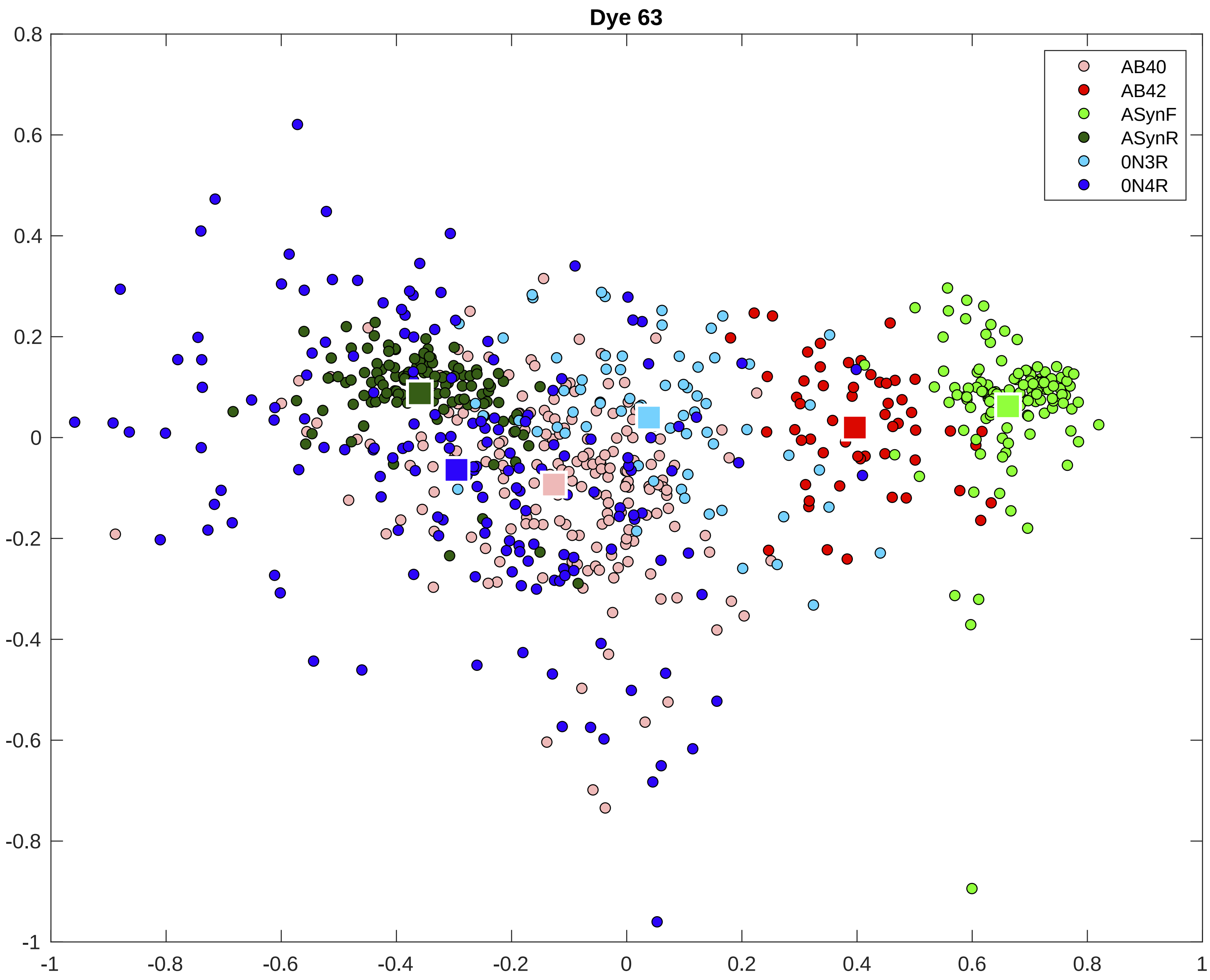

Dye 63
AB40
AB42
ASynF
ASynR
0N3R
0N4R
0.8
0.6
0.4
0.2
0
-0.2
-0.4
-0.6
-0.8
-1
-1
-0.8
-0.6
-0.4
-0.2
0
0.2
0.4
0.6
0.8
1

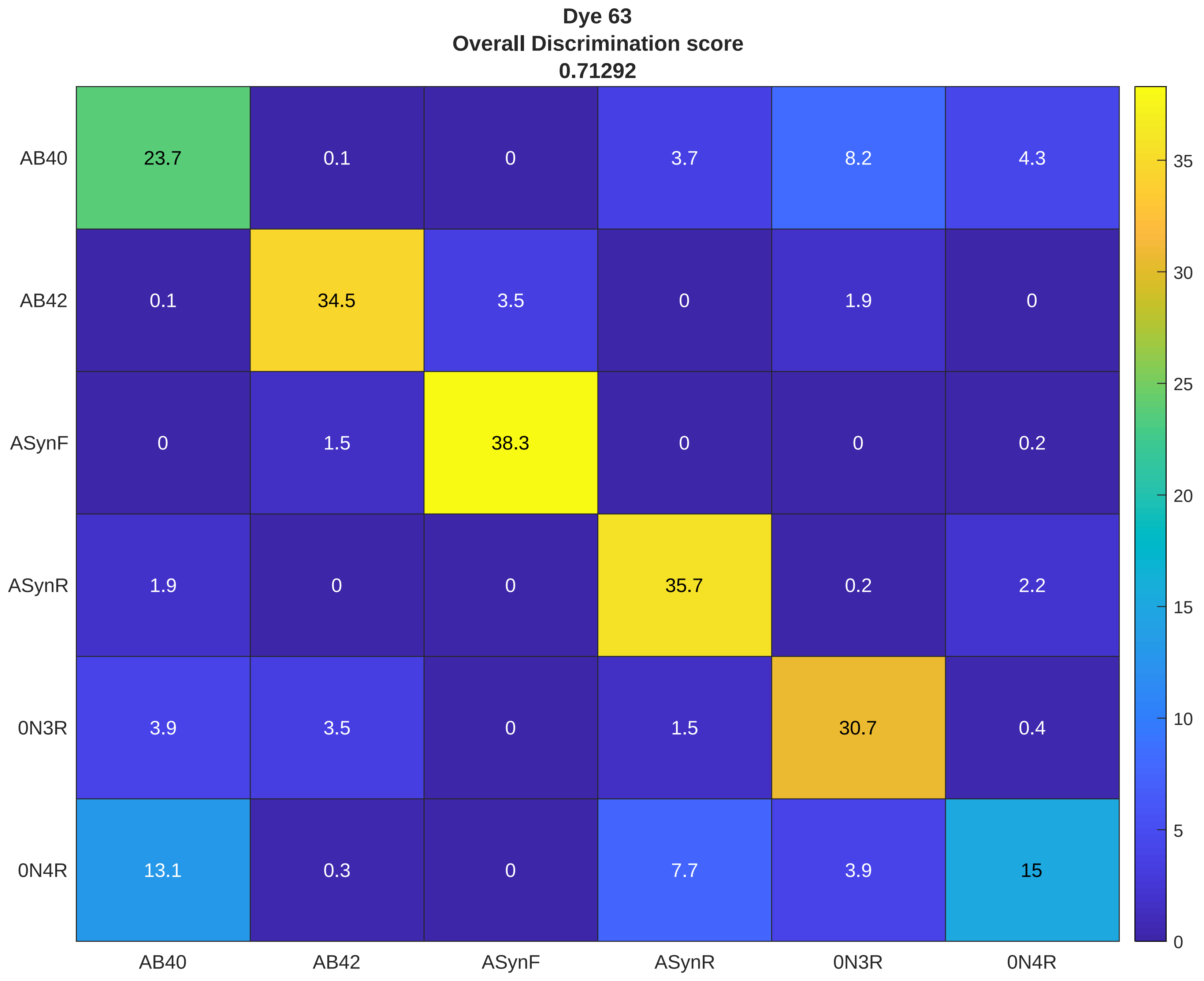
Dye 63
Overall Discrimination score
0.71292
AB40
23.7
0.1
0
3.7
8.2
4.3
AB42
0.1
34.5
3.5
0
1.9
0
ASynF
0
1.5
38.3
0
0
0.2
ASynR
1.9
0
0
35.7
0.2
2.2
0N3R
3.9
3.5
0
1.5
30.7
0.4
0N4R
13.1
0.3
0
7.7
3.9
15
AB40
AB42
ASynF
ASynR
0N3R
0N4R
35
30
25
20
15
10
5
0

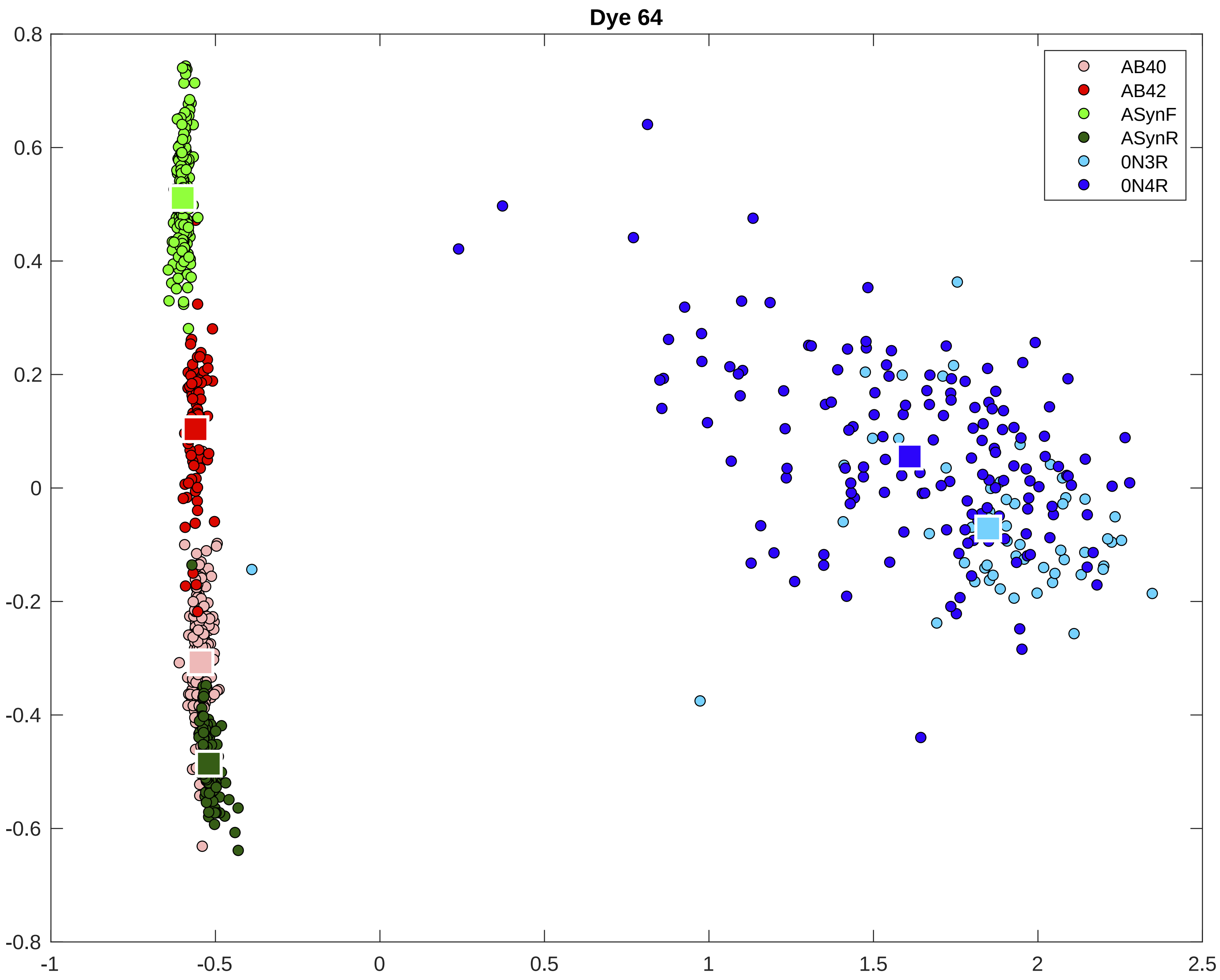

Dye 64
AB40
AB42
ASynF
ASynR
0N3R
0N4R
0.8
0.6
0.4
0.2
0
-0.2
-0.4
-0.6
-0.8
-1
-0.5
0
0.5
1
1.5
2
2.5

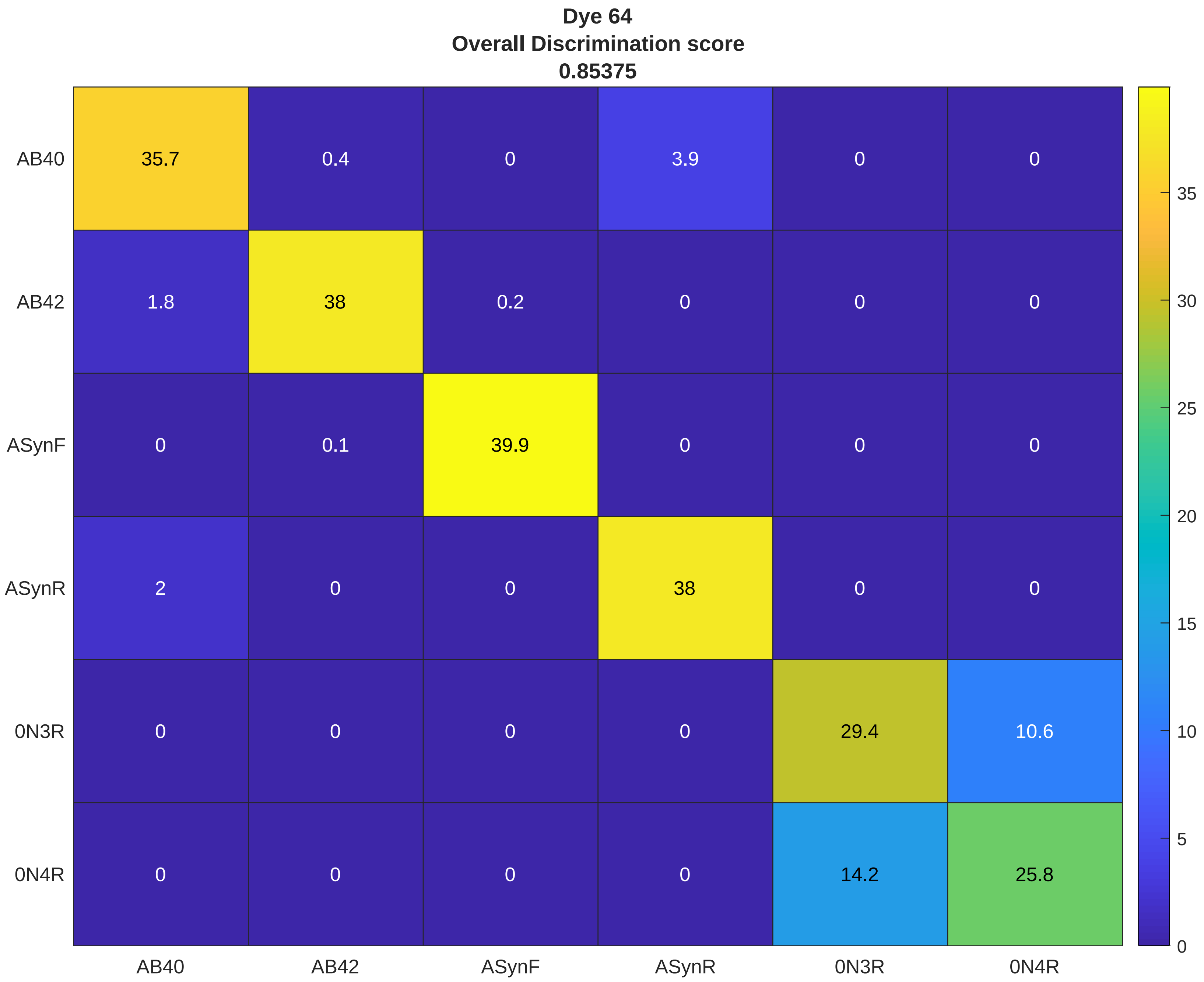
Dye 64
Overall Discrimination score
0.85375
AB40
AB42
ASynF
ASynR
0N3R
0N4R
35.7
0.4
0
3.9
0
0
1.8
38
0.2
0
0
0
0
0.1
39.9
0
0
0
2
0
0
38
0
0
0
0
0
0
29.4
10.6
0
0
0
0
14.2
25.8
AB40
AB42
ASynF
ASynR
0N3R
0N4R
35
30
25
20
15
10
5
0

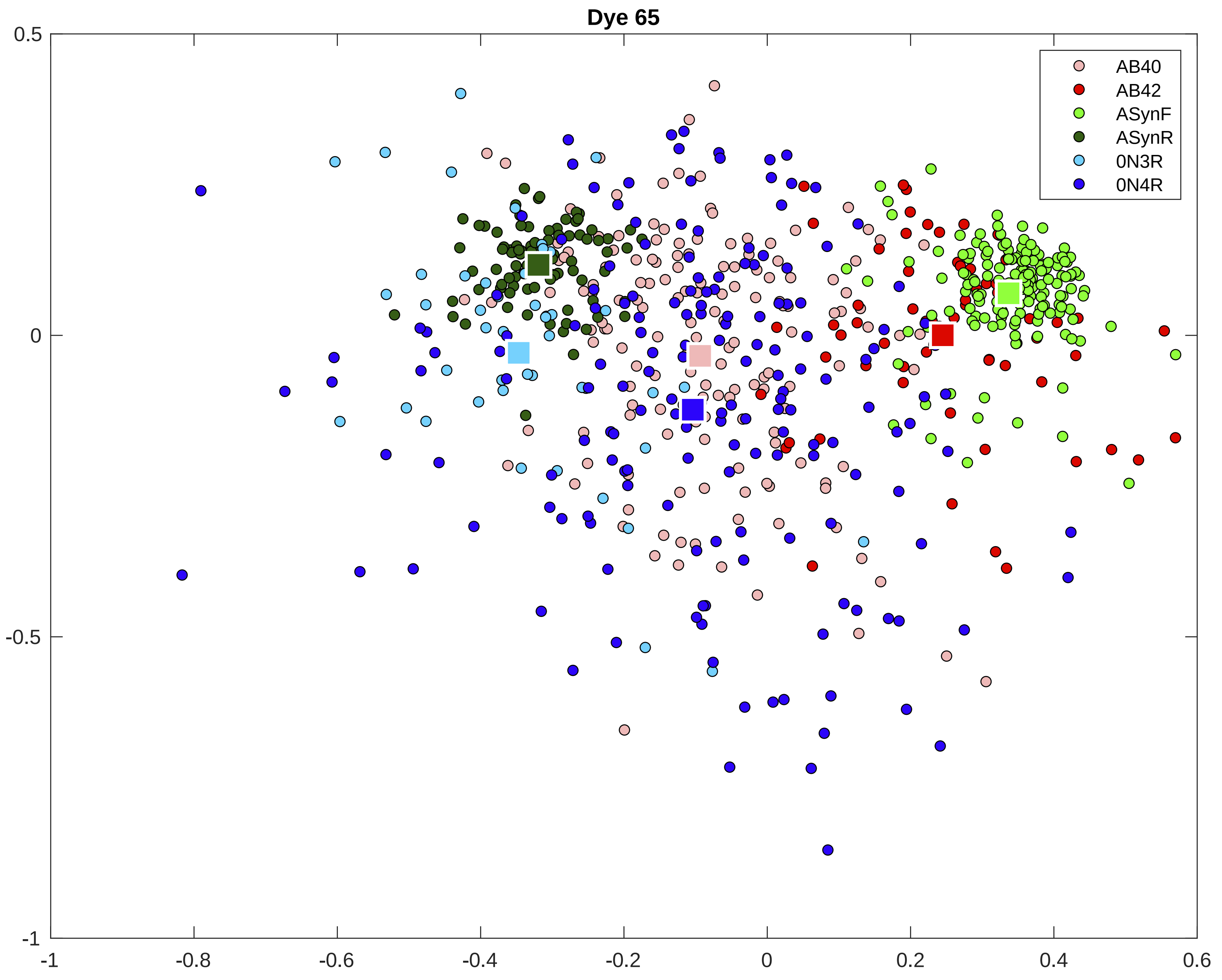

Dye 65
AB40
AB42
ASynF
ASynR
0N3R
0N4R
0.5
0
-0.5
-1
-1
-0.8
-0.6
-0.4
-0.2
0
0.2
0.4
0.6

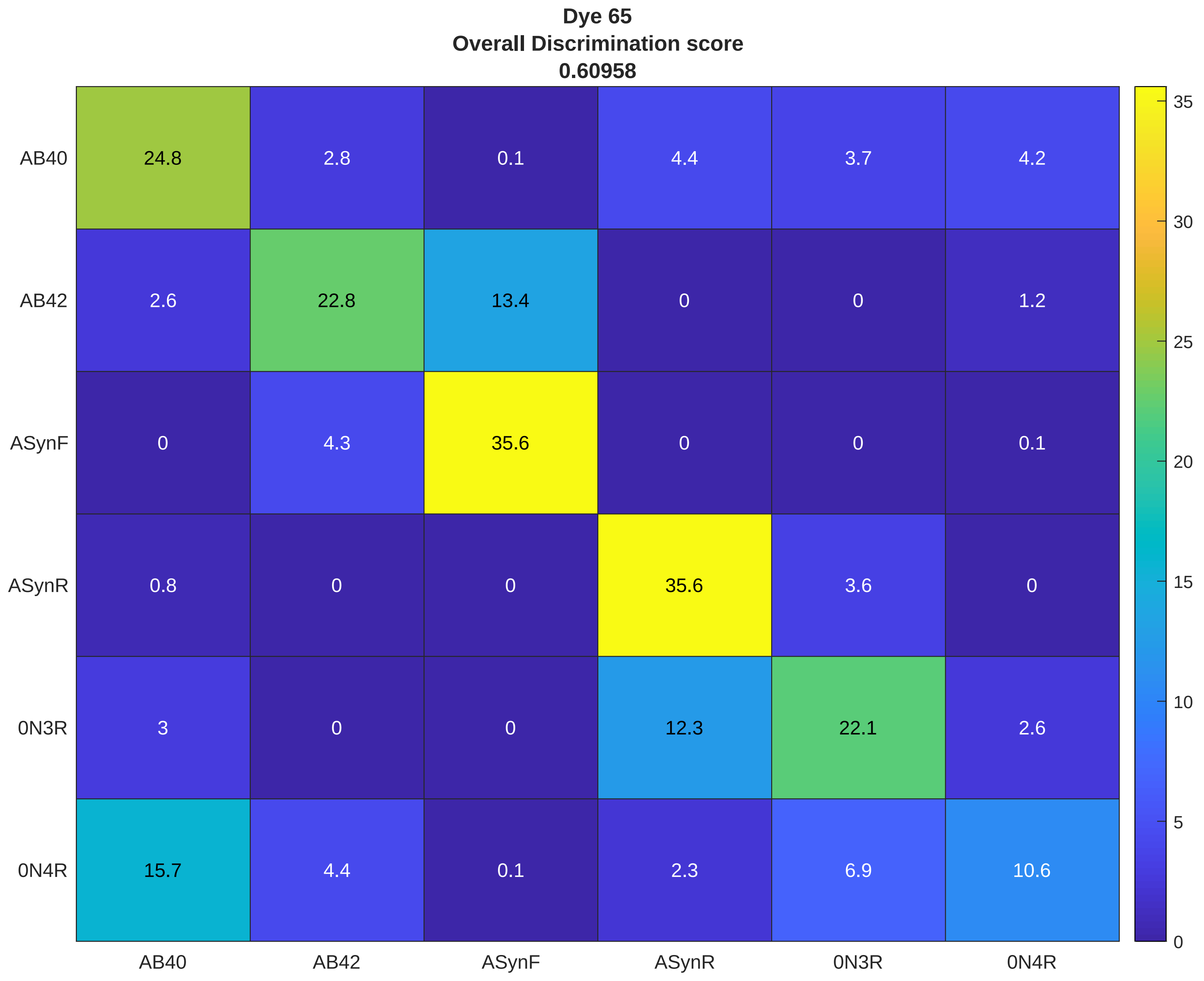
Dye 65
Overall Discrimination score
0.60958
AB40
AB42
ASynF
ASynR
0N3R
0N4R
24.8
2.8
0.1
4.4
3.7
4.2
2.6
22.8
13.4
0
0
1.2
0
4.3
35.6
0
0
0.1
0.8
0
0
35.6
3.6
0
3
0
0
12.3
22.1
2.6
15.7
4.4
0.1
2.3
6.9
10.6
AB40
AB42
ASynF
ASynR
0N3R
0N4R
35
30
25
20
15
10
5
0

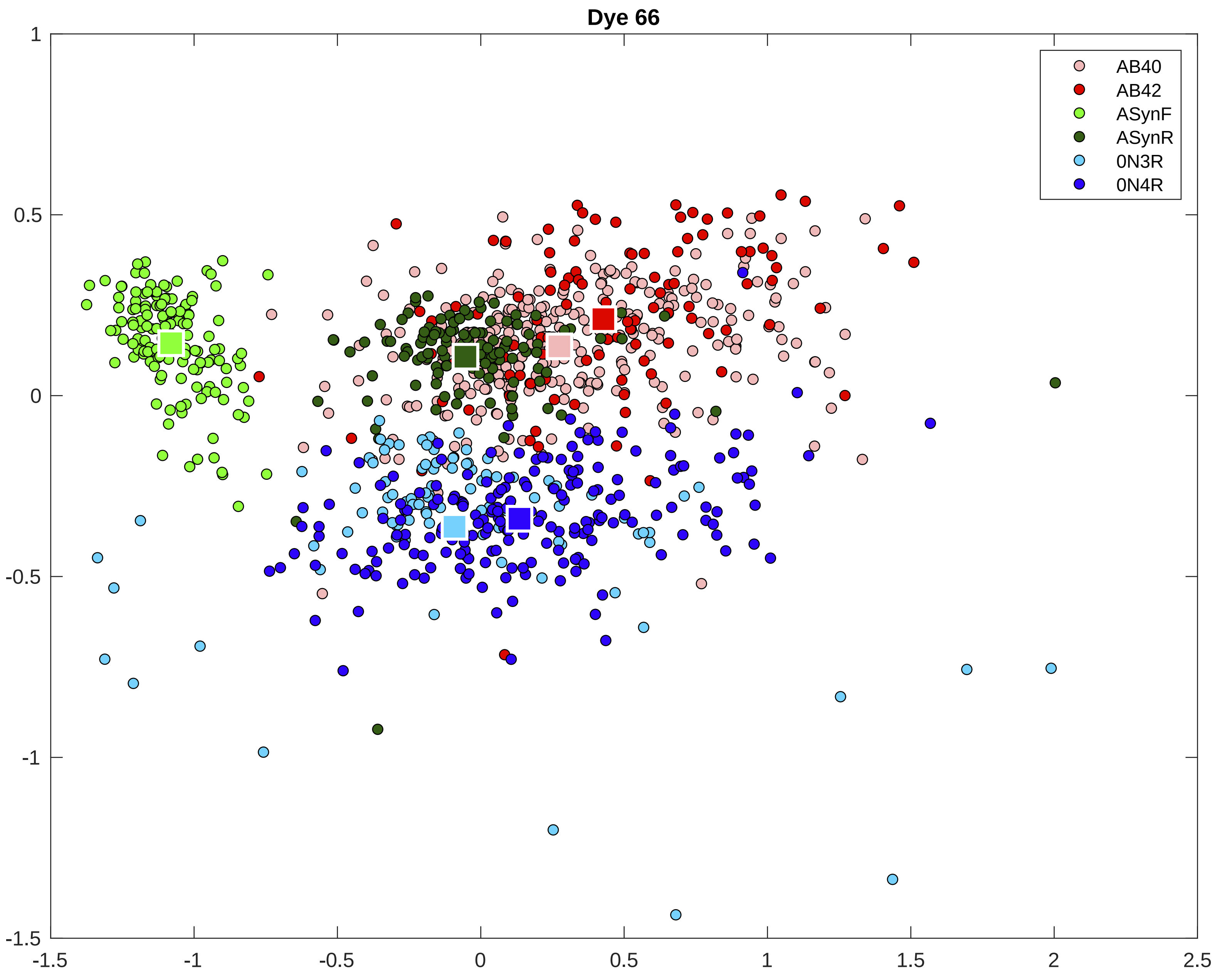
Dye 66
AB40
AB42
ASynF
ASynR
0N3R
0N4R
1
0.5
0
-0.5
-1
-1.5
-1.5
-1
-0.5
0
0.5
1
1.5
2
2.5

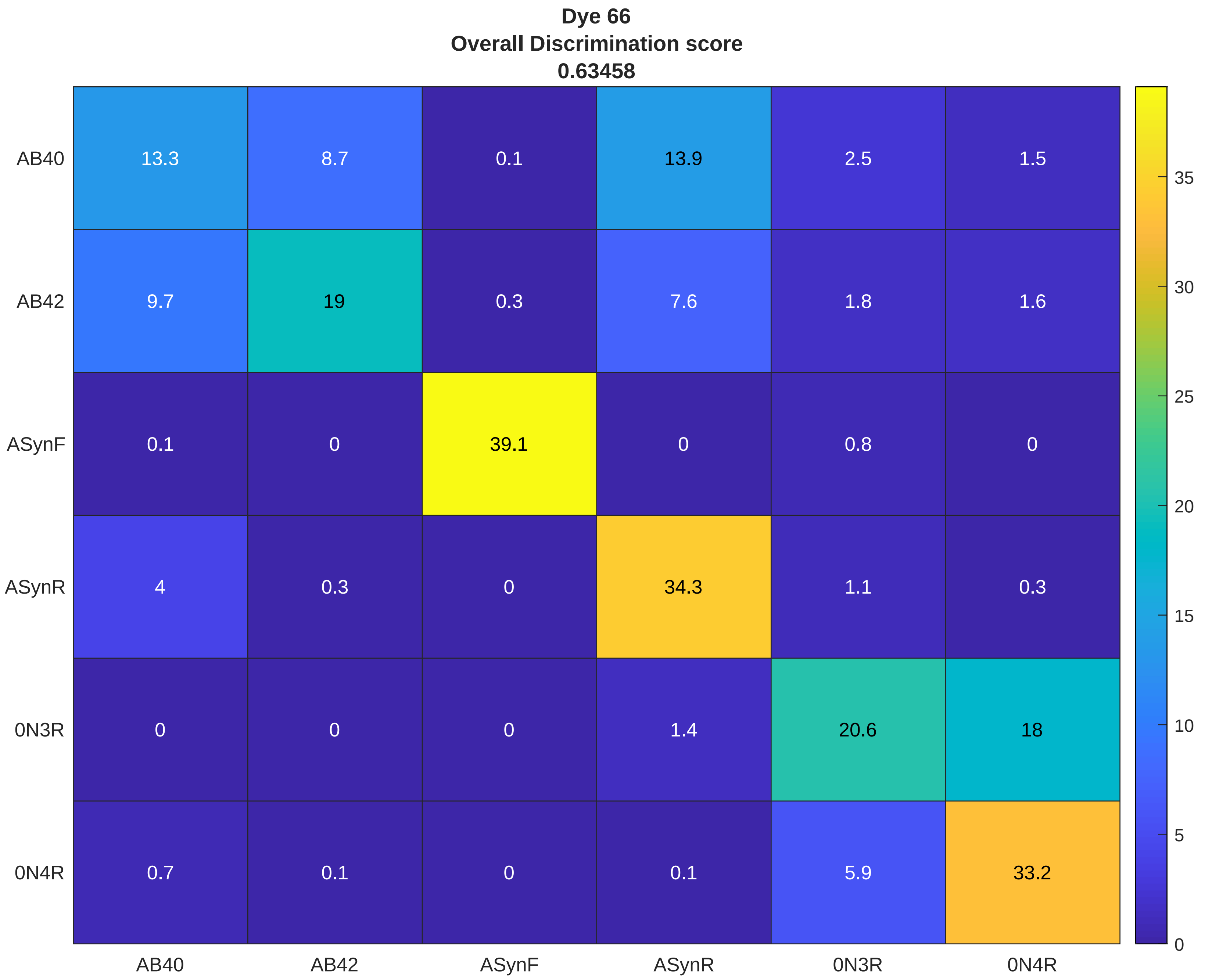
Dye 66
Overall Discrimination score
0.63458
AB40
AB42
ASynF
ASynR
0N3R
0N4R
13.3
8.7
0.1
13.9
2.5
1.5
9.7
19
0.3
7.6
1.8
1.6
0.1
0
39.1
0
0.8
0
4
0.3
0
34.3
1.1
0.3
0
0
0
1.4
20.6
18
0.7
0.1
0
0.1
5.9
33.2
35
30
25
20
15
10
5
0

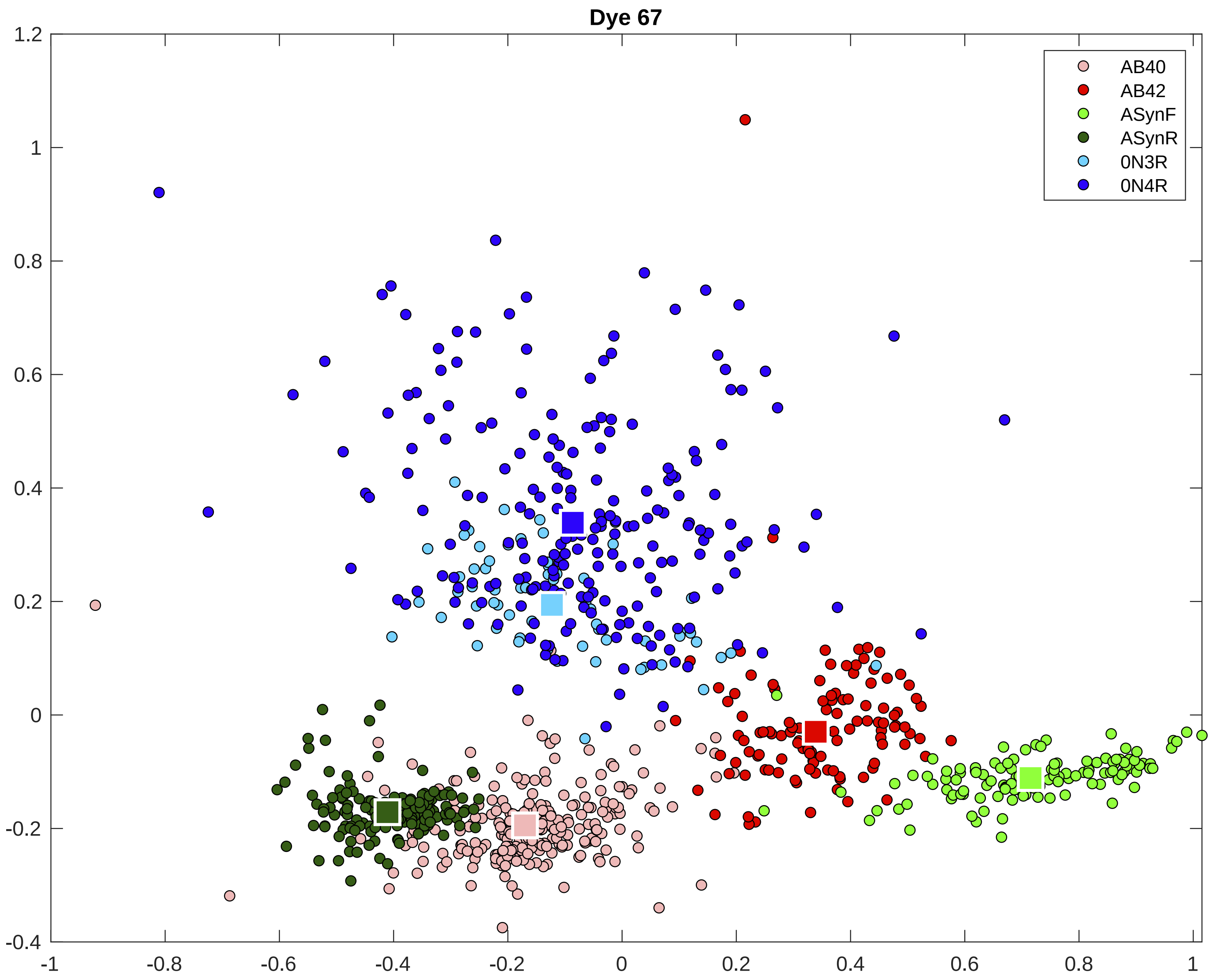

Dye 67
AB40
AB42
ASynF
ASynR
0N3R
0N4R
1.2
1
0.8
0.6
0.4
0.2
0
-0.2
-0.4
-1
-0.8
-0.6
-0.4
-0.2
0
0.2
0.4
0.6
0.8
1

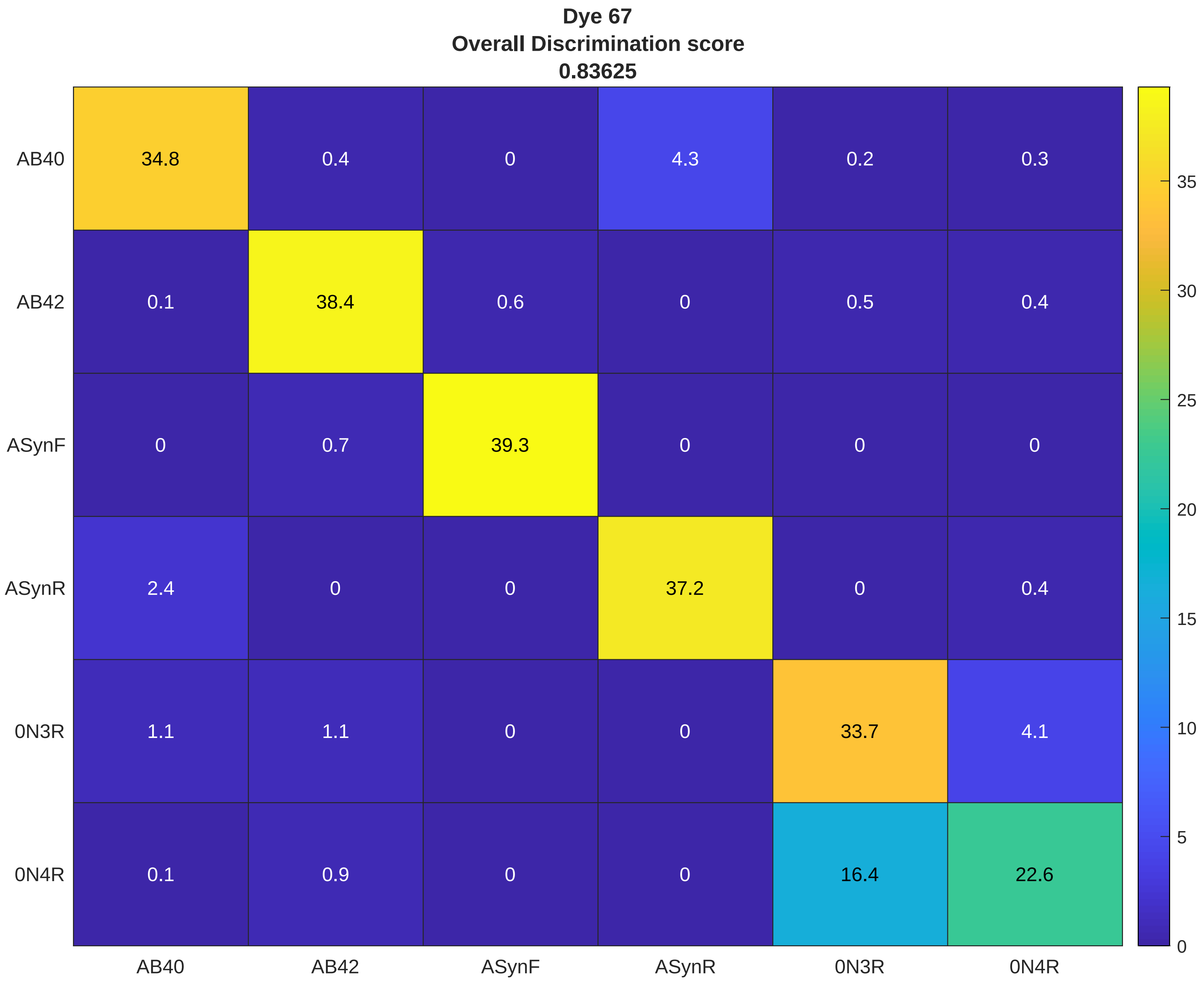
Dye 67
Overall Discrimination score
0.83625
AB40
AB42
ASynF
ASynR
0N3R
0N4R
34.8
0.4
0
4.3
0.2
0.3
0.1
38.4
0.6
0
0.5
0.4
0
0.7
39.3
0
0
0
2.4
0
0
37.2
0
0.4
1.1
1.1
0
0
33.7
4.1
0.1
0.9
0
0
16.4
22.6
AB40
AB42
ASynF
ASynR
0N3R
0N4R
35
30
25
20
15
10
5
0

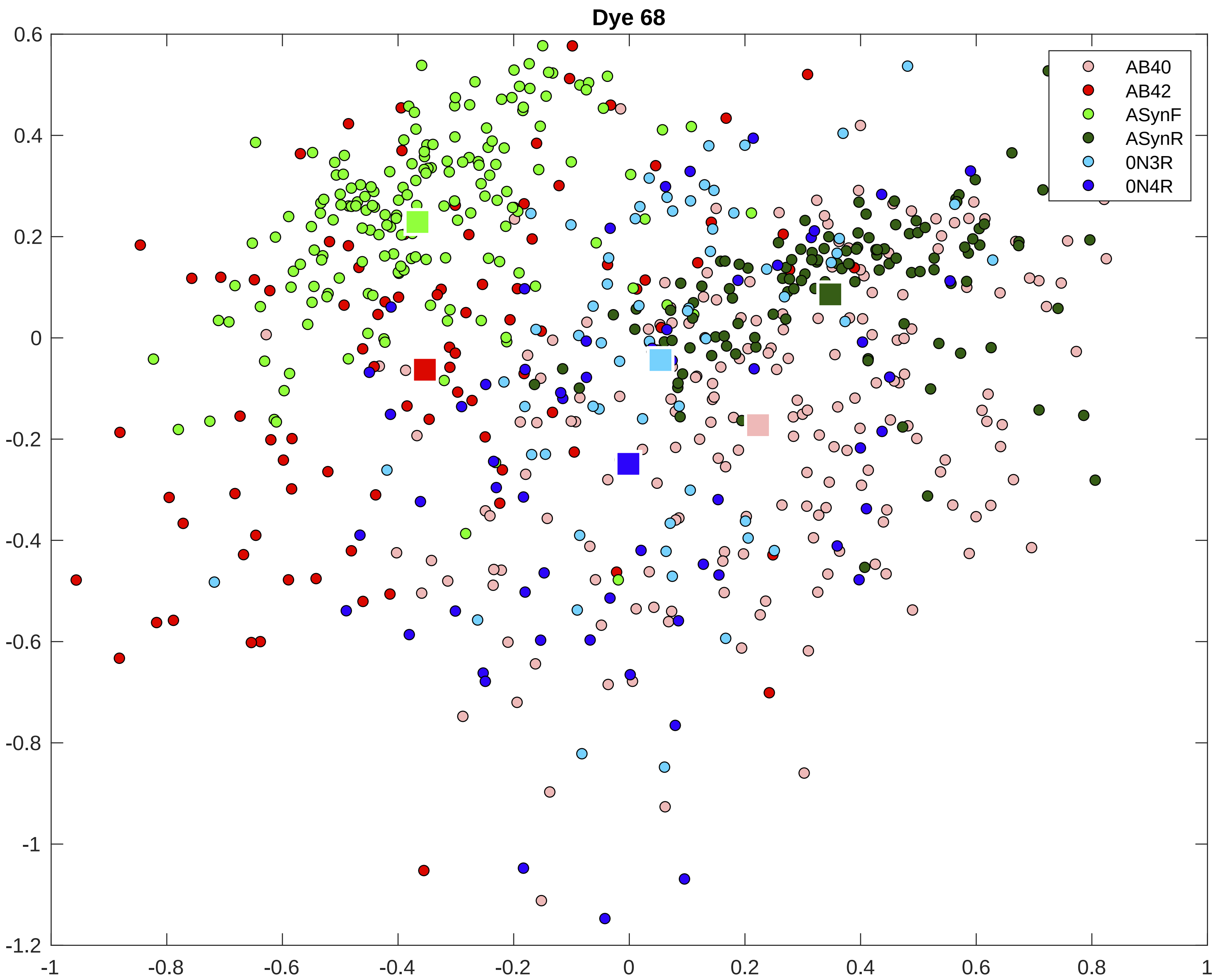

Dye 68
AB40
AB42
ASynF
ASynR
0N3R
0N4R
0.6
0.4
0.2
0
-0.2
-0.4
-0.6
-0.8
-1
-1.2
-1
-0.8
-0.6
-0.4
-0.2
0
0.2
0.4
0.6
0.8
1

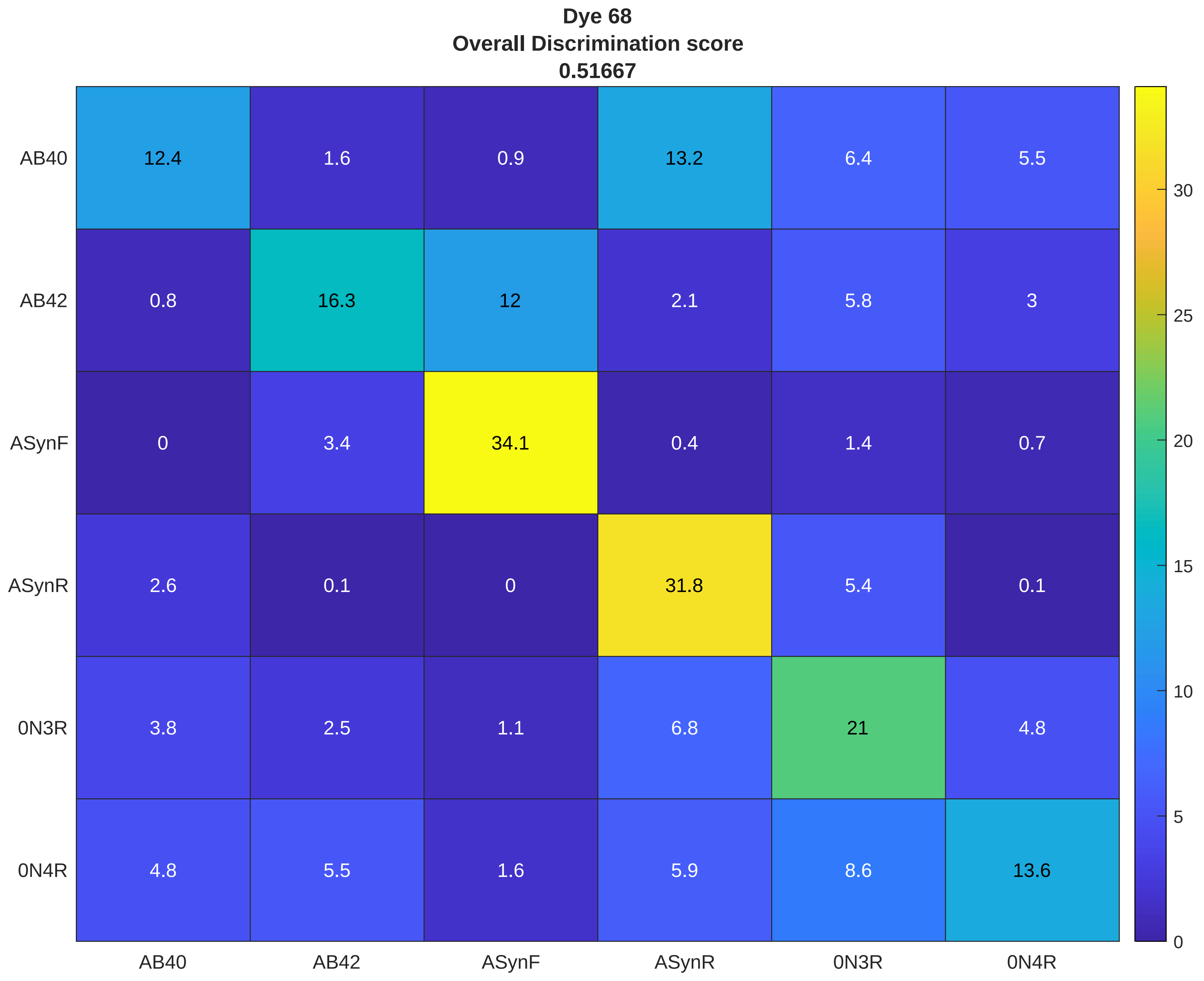
Dye 68
Overall Discrimination score
0.51667
AB40
AB42
ASynF
ASynR
0N3R
0N4R
12.4
1.6
0.9
13.2
6.4
5.5
0.8
16.3
12
2.1
5.8
3
0
3.4
34.1
0.4
1.4
0.7
2.6
0.1
0
31.8
5.4
0.1
3.8
2.5
1.1
6.8
21
4.8
4.8
5.5
1.6
5.9
8.6
13.6
AB40
AB42
ASynF
ASynR
0N3R
0N4R
30
25
20
15
10
5
0

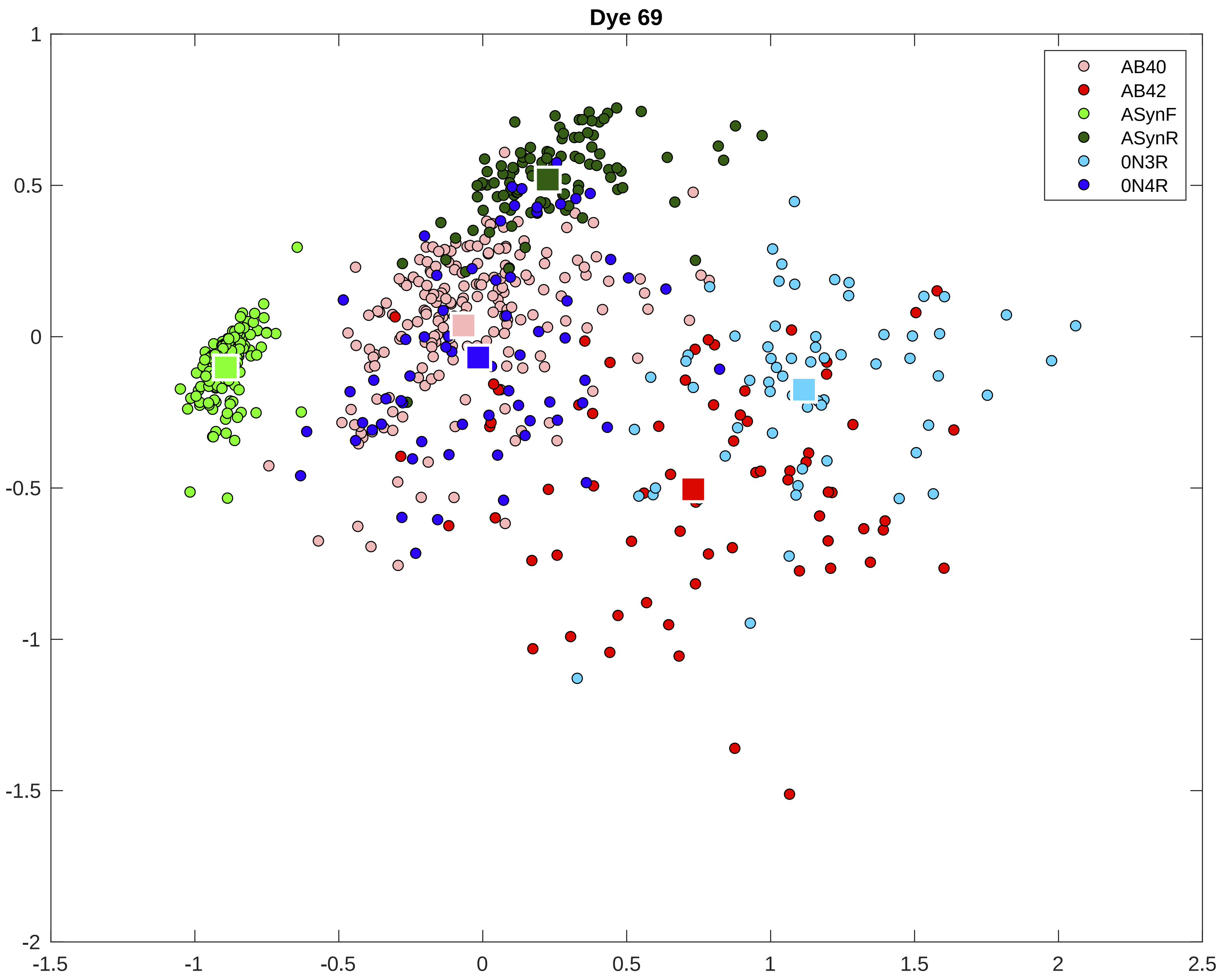

Dye 69
AB40
AB42
ASynF
ASynR
0N3R
0N4R
1
0.5
0
-0.5
-1
-1.5
-2
-1.5
-1
-0.5
0
0.5
1
1.5
2
2.5

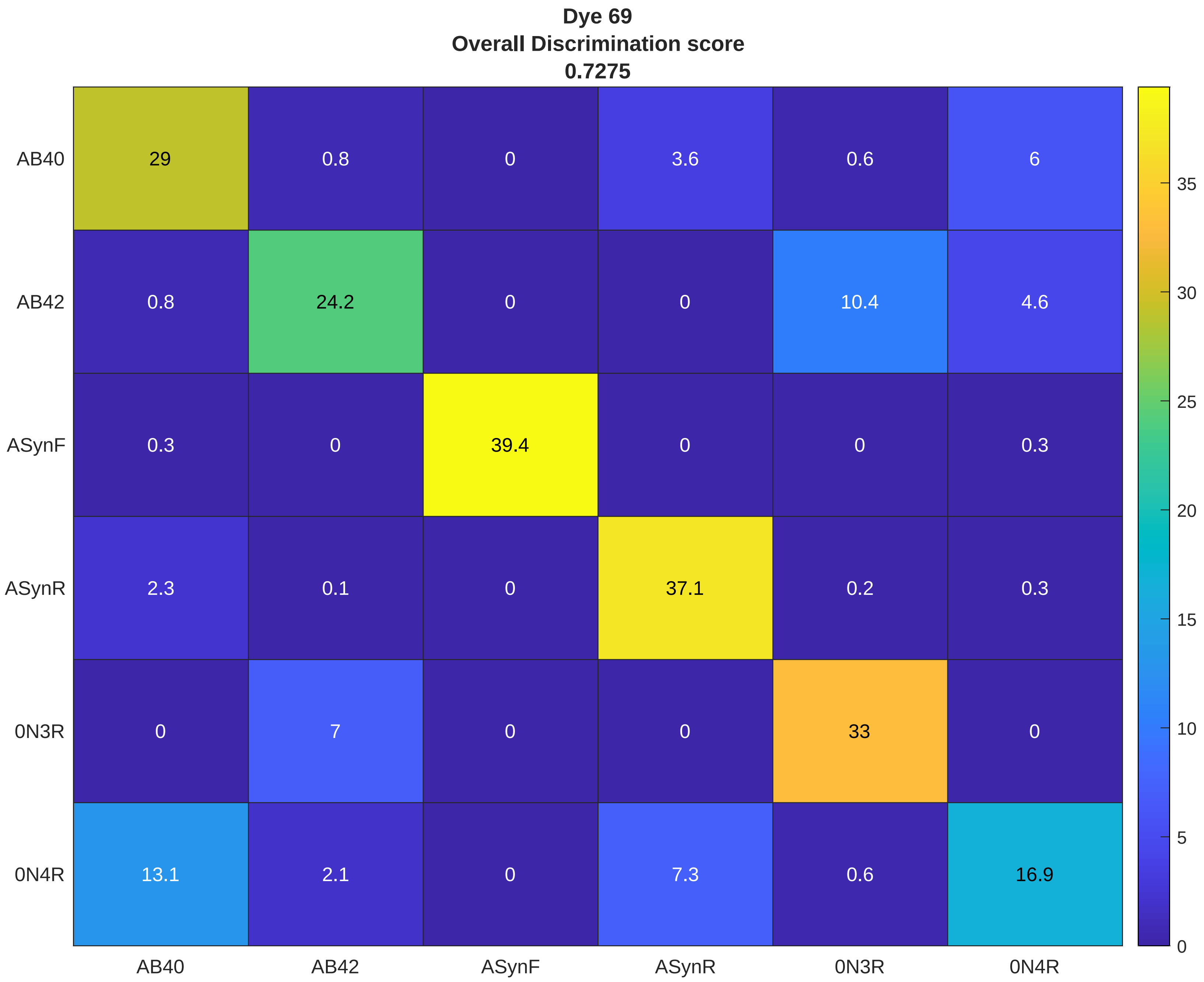
Dye 69
Overall Discrimination score
0.7275
AB40
AB42
ASynF
ASynR
0N3R
0N4R
29
0.8
0
3.6
0.6
6
0.8
24.2
0
0
10.4
4.6
0.3
0
39.4
0
0
0.3
2.3
0.1
0
37.1
0.2
0.3
0
7
0
0
33
0
13.1
2.1
0
7.3
0.6
16.9
AB40
AB42
ASynF
ASynR
0N3R
0N4R
35
30
25
20
15
10
5
0

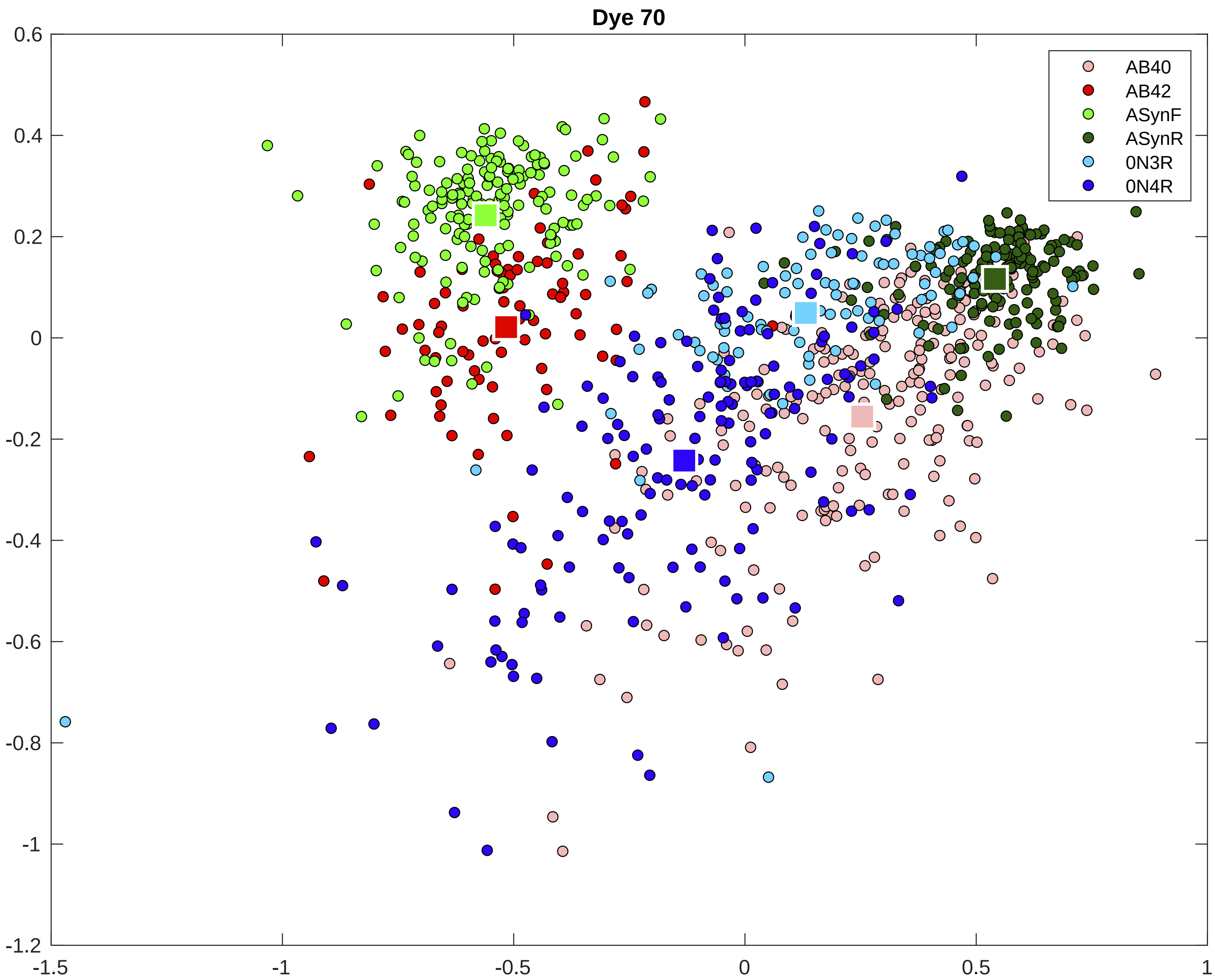
Dye 70
0.6
0.4
0.2
0
-0.2
-0.4
-0.6
-0.8
-1
-1.2
-1.5
-1
-0.5
0
0.5
1
AB40
AB42
ASynF
ASynR
0N3R
0N4R

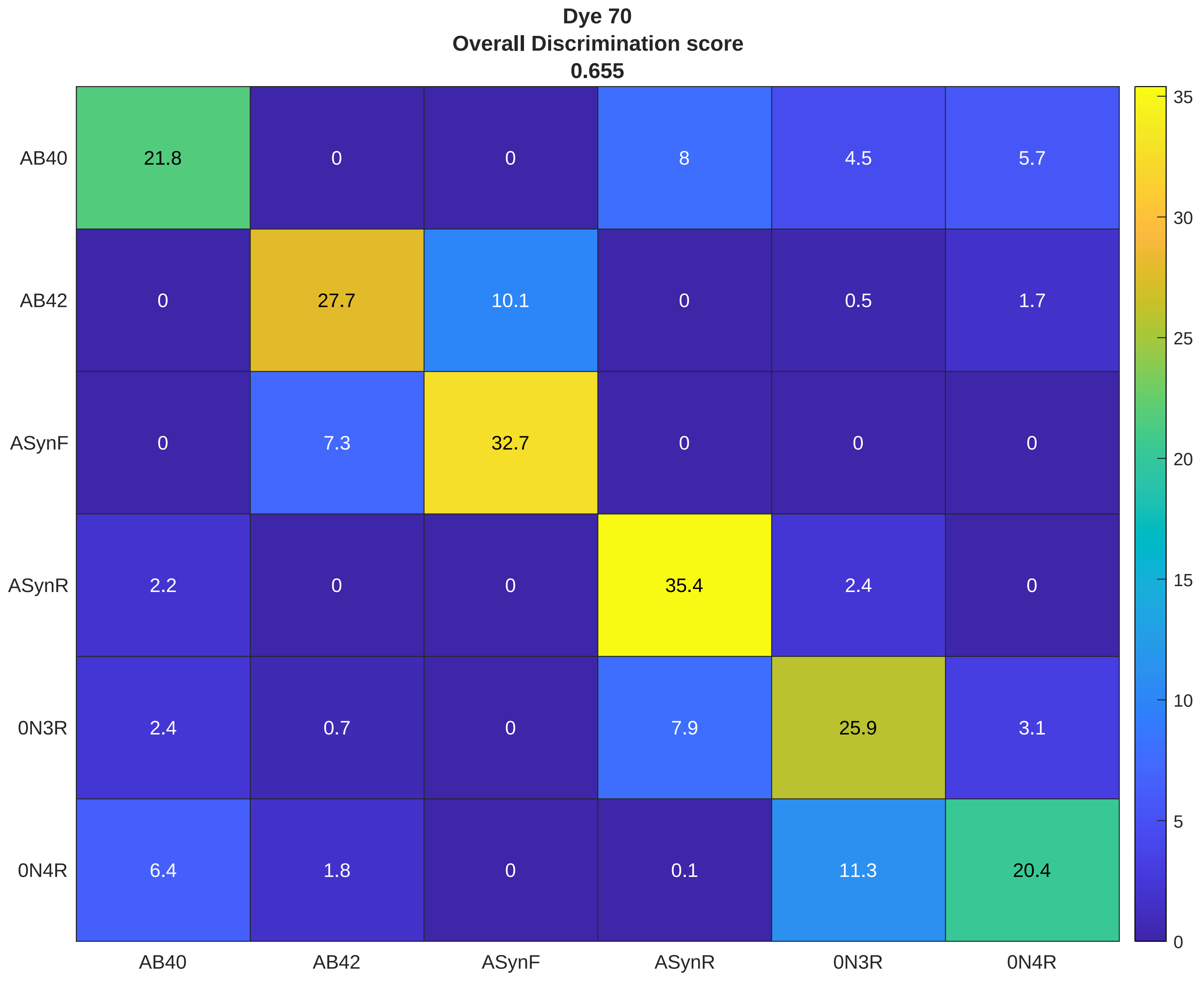

Dye 70
Overall Discrimination score
0.655
AB40
AB42
ASynF
ASynR
0N3R
0N4R
21.8
0
0
8
4.5
5.7
0
27.7
10.1
0
0.5
1.7
0
7.3
32.7
0
0
0
2.2
0
0
35.4
2.4
0
2.4
0.7
0
7.9
25.9
3.1
6.4
1.8
0
0.1
11.3
20.4
AB40
AB42
ASynF
ASynR
0N3R
0N4R
35
30
25
20
15
10
5
0

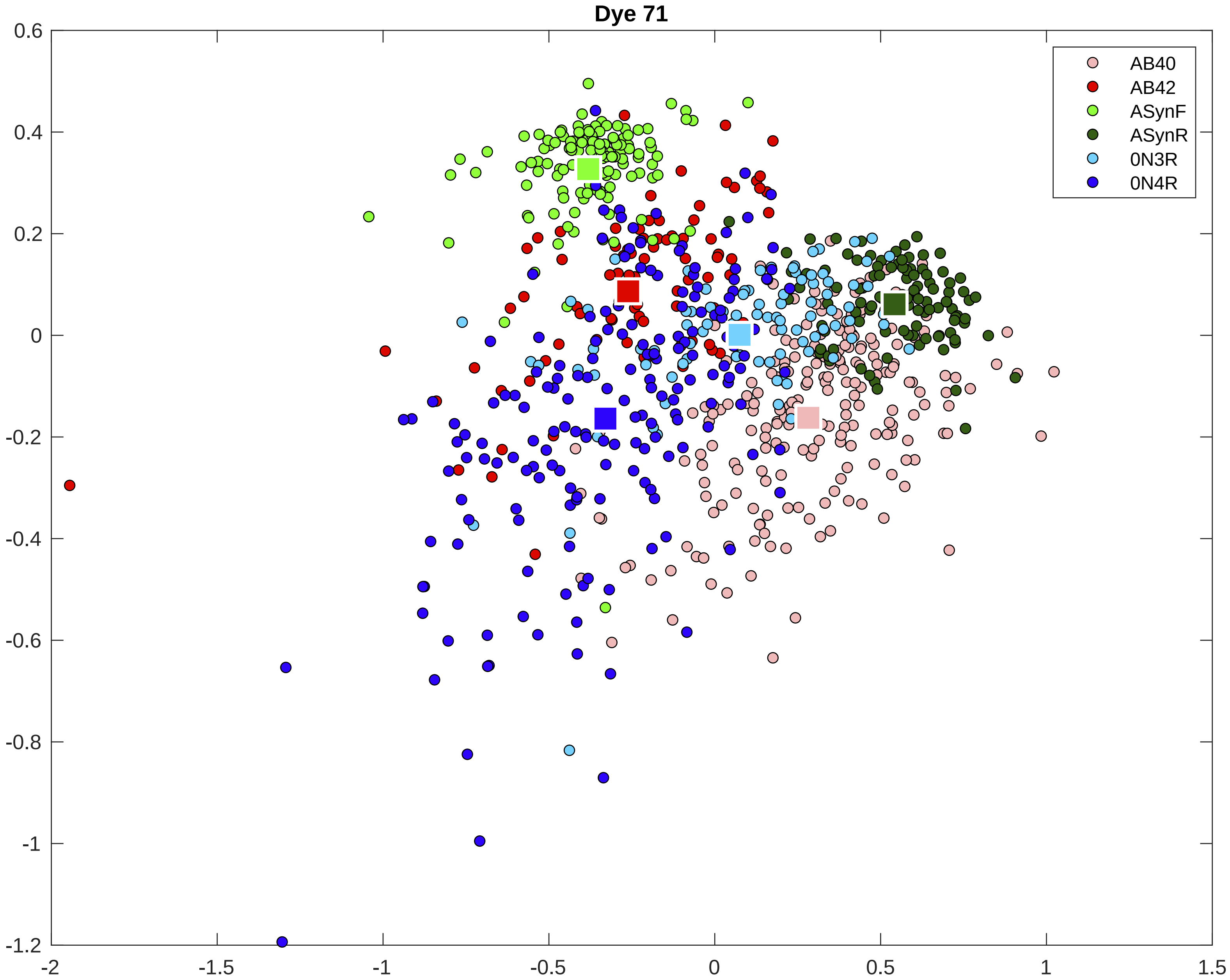

Dye 71
AB40
AB42
ASynF
ASynR
0N3R
0N4R
0.6
0.4
0.2
0
-0.2
-0.4
-0.6
-0.8
-1
-1.2
-2
-1.5
-1
-0.5
0
0.5
1
1.5

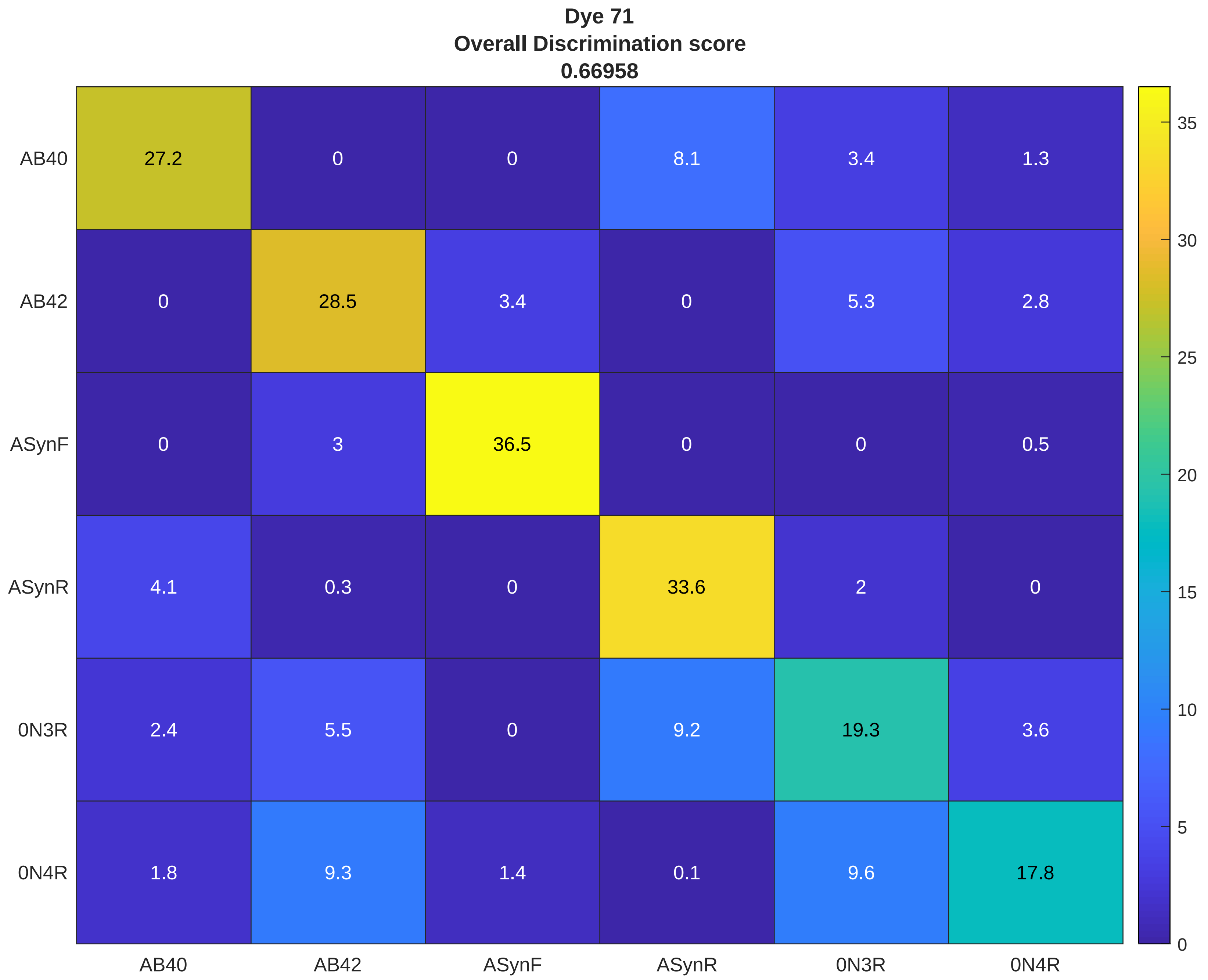
Dye 71
Overall Discrimination score
0.66958
AB40
AB42
ASynF
ASynR
0N3R
0N4R
27.2 0 0 8.1 3.4 1.3
0 28.5 3.4 0 5.3 2.8
0 3 36.5 0 0 0.5
4.1 0.3 0 33.6 2 0
2.4 5.5 0 9.2 19.3 3.6
1.8 9.3 1.4 0.1 9.6 17.8
AB40
AB42
ASynF
ASynR
0N3R
0N4R
35
30
25
20
15
10
5
0

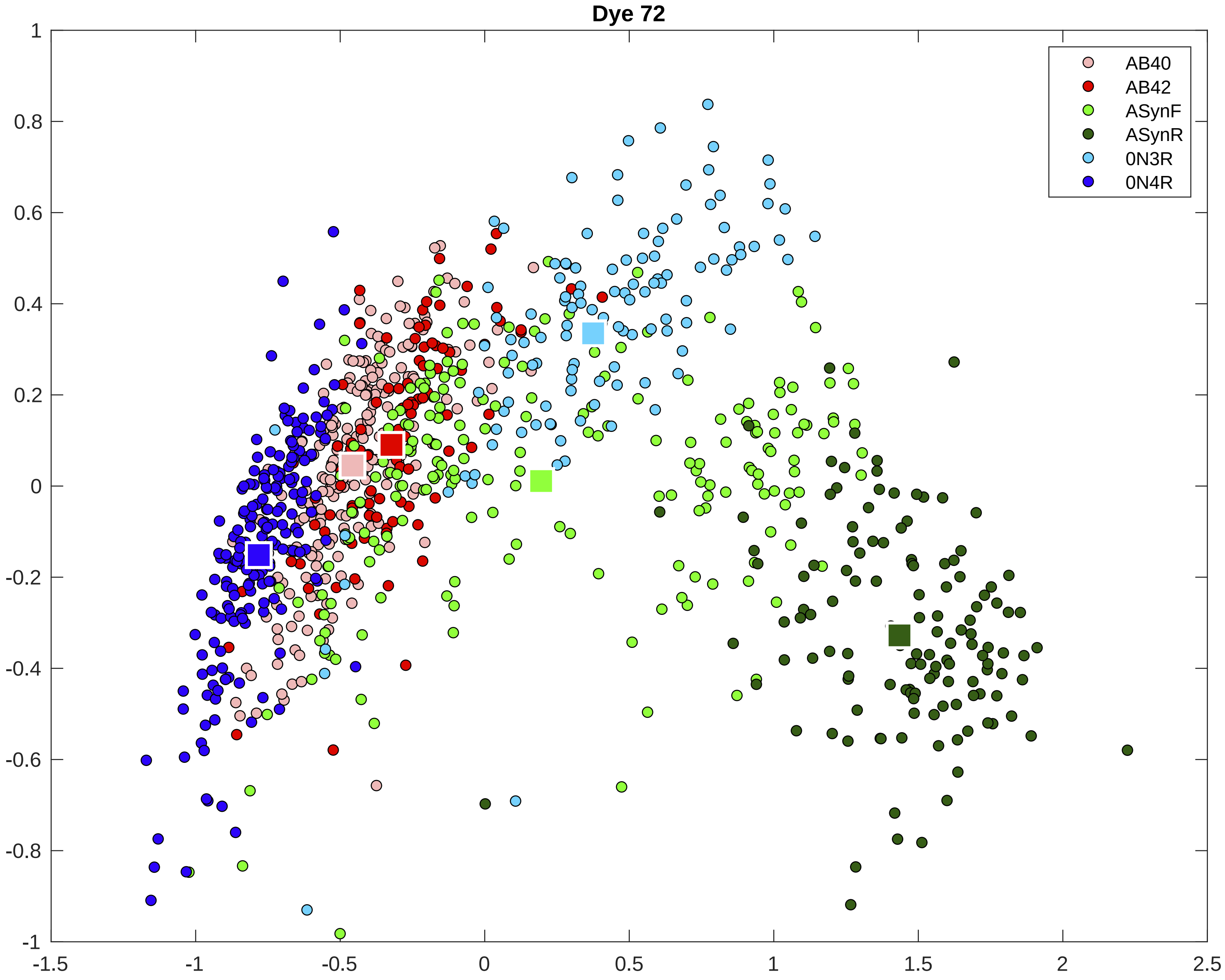

Dye 72
AB40
AB42
ASynF
ASynR
0N3R
0N4R
1
0.8
0.6
0.4
0.2
0
-0.2
-0.4
-0.6
-0.8
-1
-1.5
-1
-0.5
0
0.5
1
1.5
2
2.5

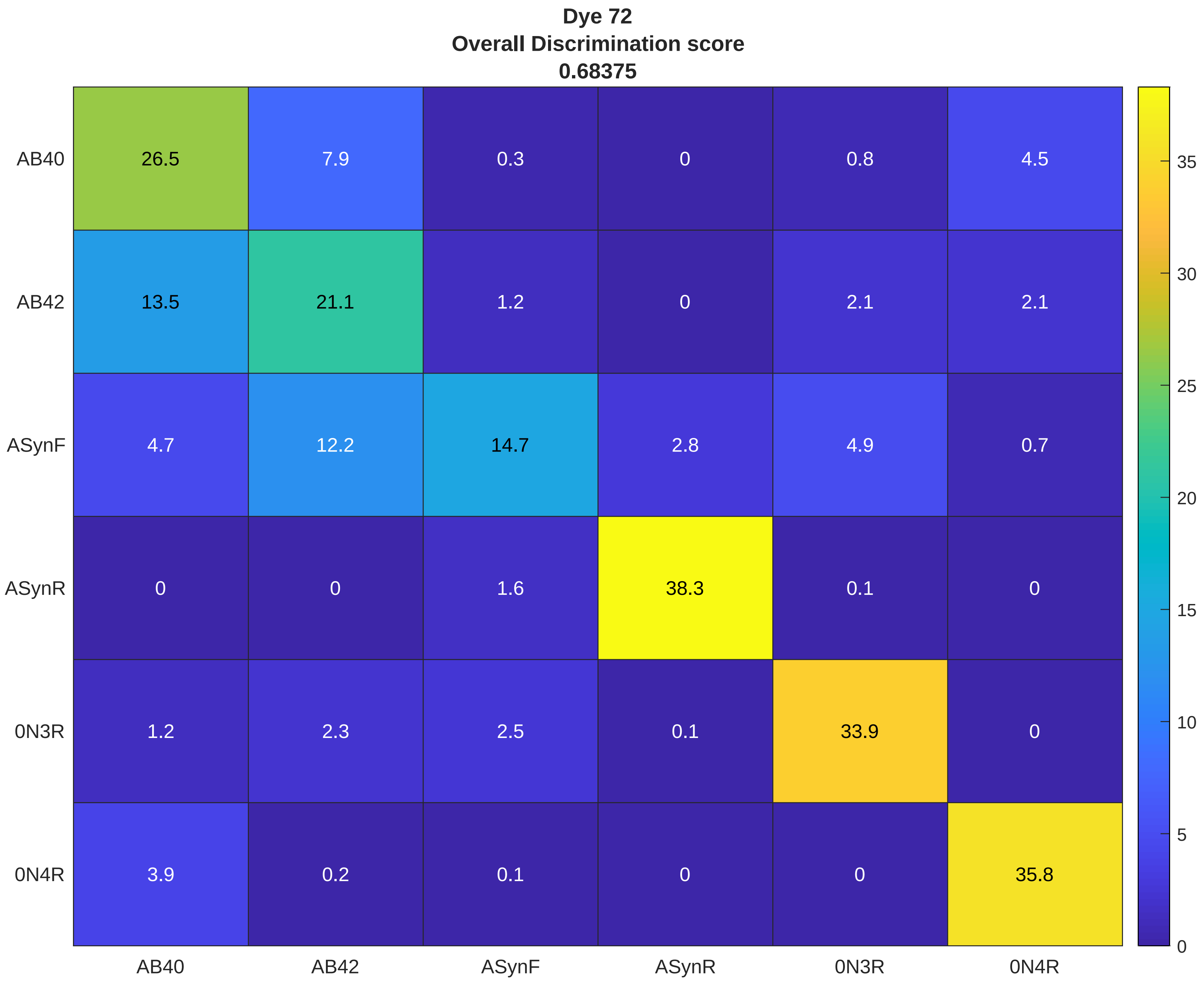
Dye 72
Overall Discrimination score
0.68375
AB40
AB42
ASynF
ASynR
0N3R
0N4R
26.5
7.9
0.3
0
0.8
4.5
13.5
21.1
1.2
0
2.1
2.1
4.7
12.2
14.7
2.8
4.9
0.7
0
0
1.6
38.3
0.1
0
1.2
2.3
2.5
0.1
33.9
0
3.9
0.2
0.1
0
0
35.8
AB40
AB42
ASynF
ASynR
0N3R
0N4R
35
30
25
20
15
10
5
0

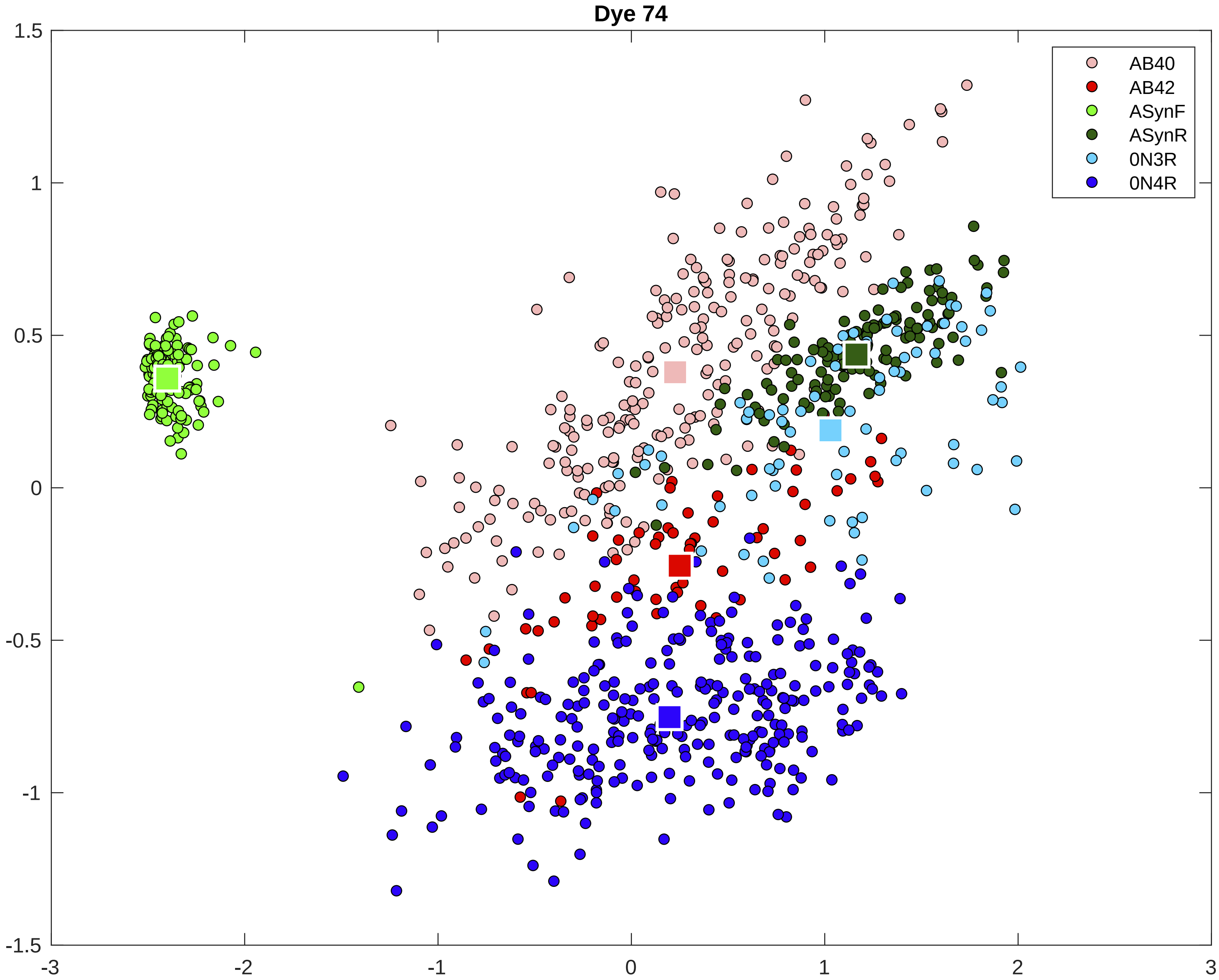
Dye 74
1.5
1
0.5
0
-0.5
-1
-1.5
-3
-2
-1
0
1
2
3
AB40
AB42
ASynF
ASynR
0N3R
0N4R

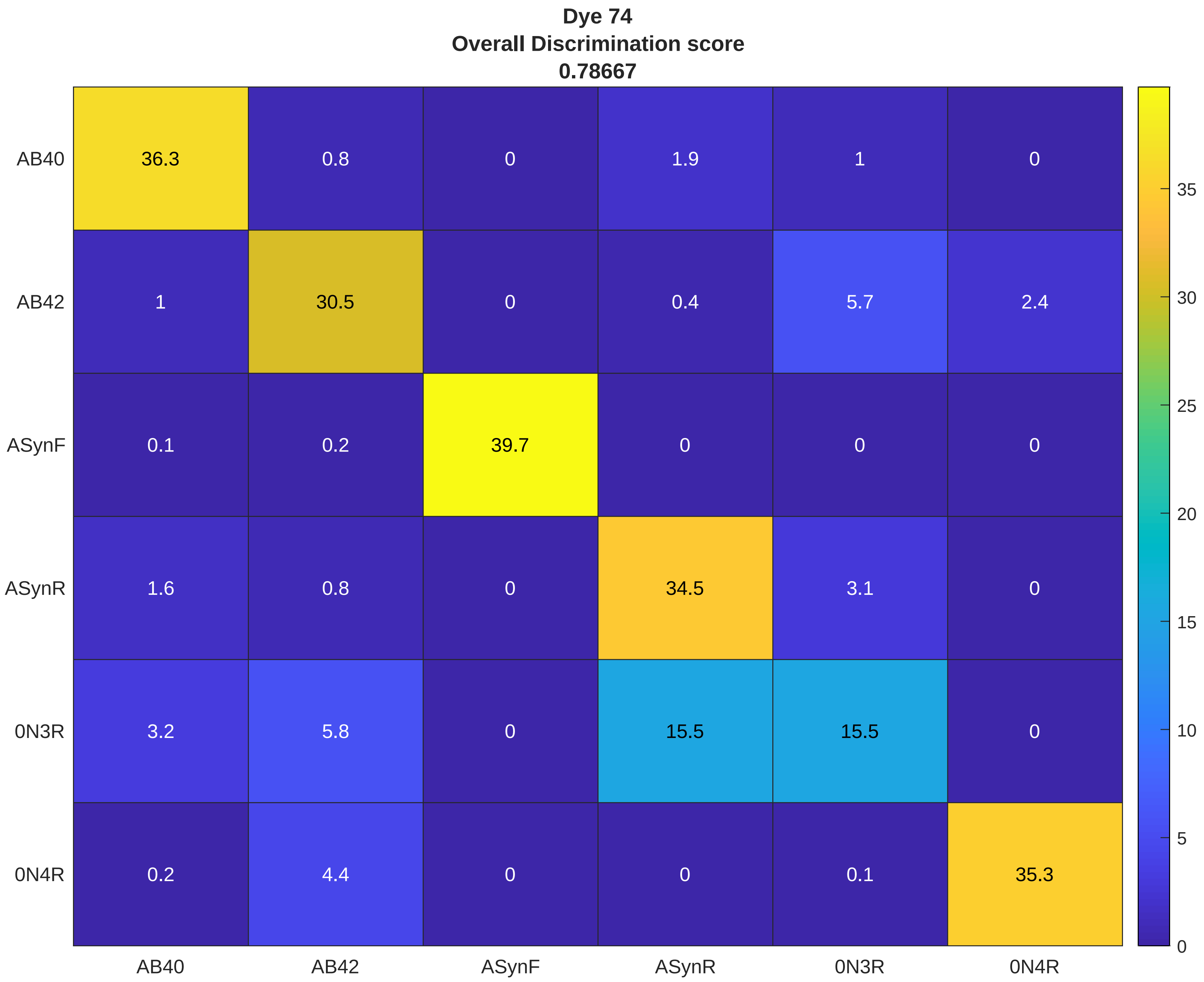

Dye 74
Overall Discrimination score
0.78667
AB40
AB42
ASynF
ASynR
0N3R
0N4R
36.3
0.8
0
1.9
1
0
1
30.5
0
0.4
5.7
2.4
0.1
0.2
39.7
0
0
0
1.6
0.8
0
34.5
3.1
0
3.2
5.8
0
15.5
15.5
0
0.2
4.4
0
0
0.1
35.3
AB40
AB42
ASynF
ASynR
0N3R
0N4R
0
5
10
15
20
25
30
35

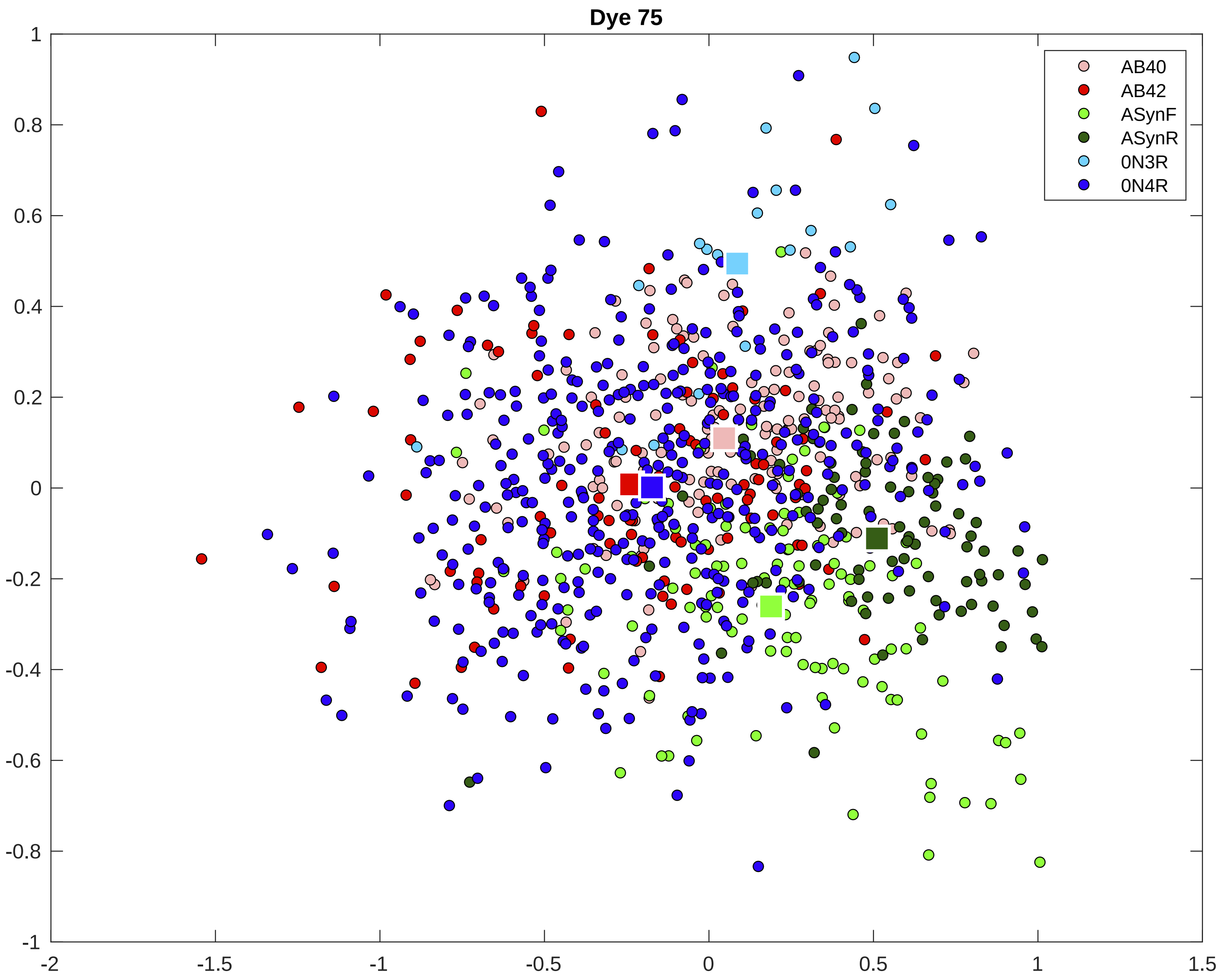

Dye 75
AB40
AB42
ASynF
ASynR
0N3R
0N4R
1
0.8
0.6
0.4
0.2
0
-0.2
-0.4
-0.6
-0.8
-1
-2
-1.5
-1
-0.5
0
0.5
1
1.5

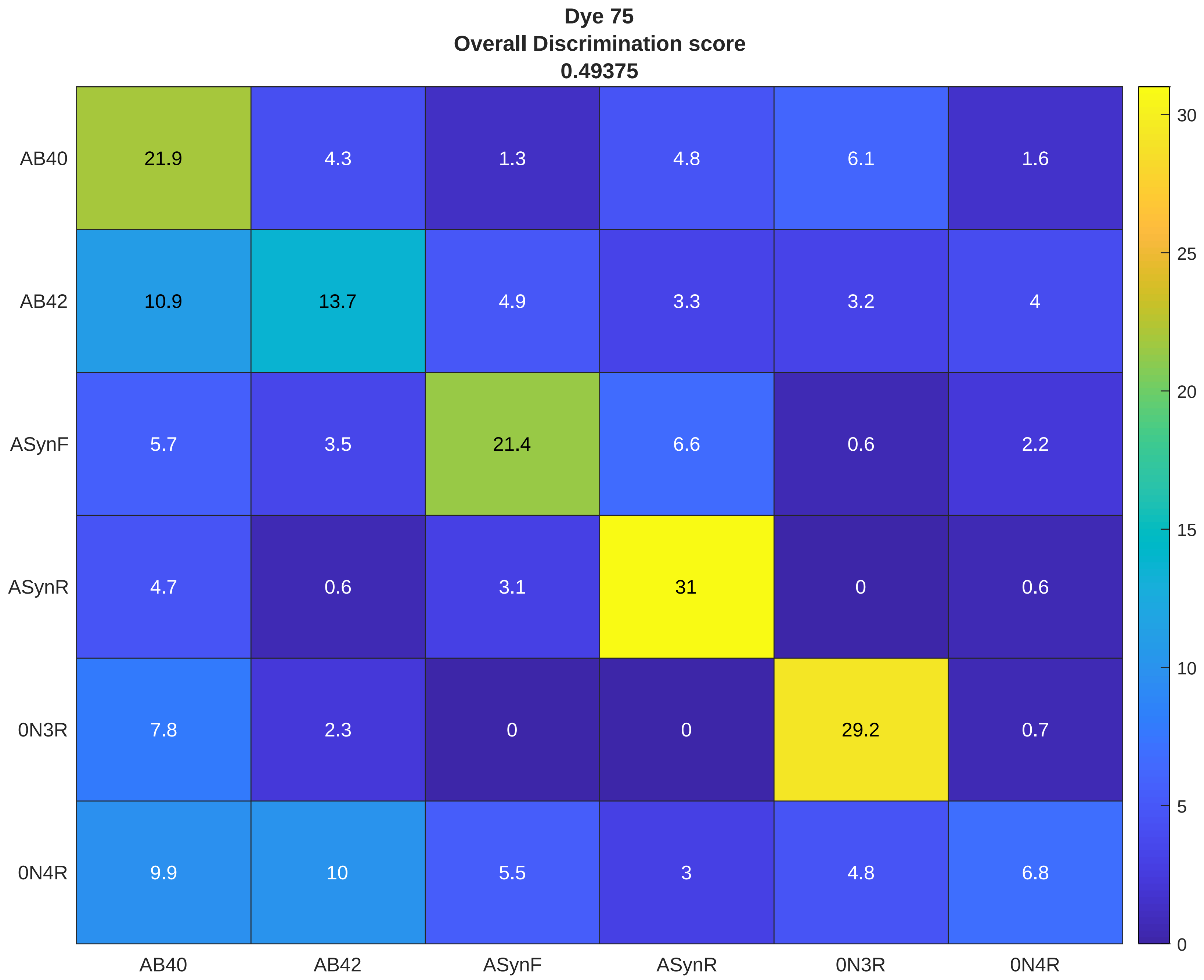
Dye 75
Overall Discrimination score
0.49375
AB40
AB42
ASynF
ASynR
0N3R
0N4R
21.9 4.3 1.3 4.8 6.1 1.6
10.9 13.7 4.9 3.3 3.2 4
5.7 3.5 21.4 6.6 0.6 2.2
4.7 0.6 3.1 31 0 0.6
7.8 2.3 0 0 29.2 0.7
9.9 10 5.5 3 4.8 6.8
AB40
AB42
ASynF
ASynR
0N3R
0N4R
30
25
20
15
10
5
0

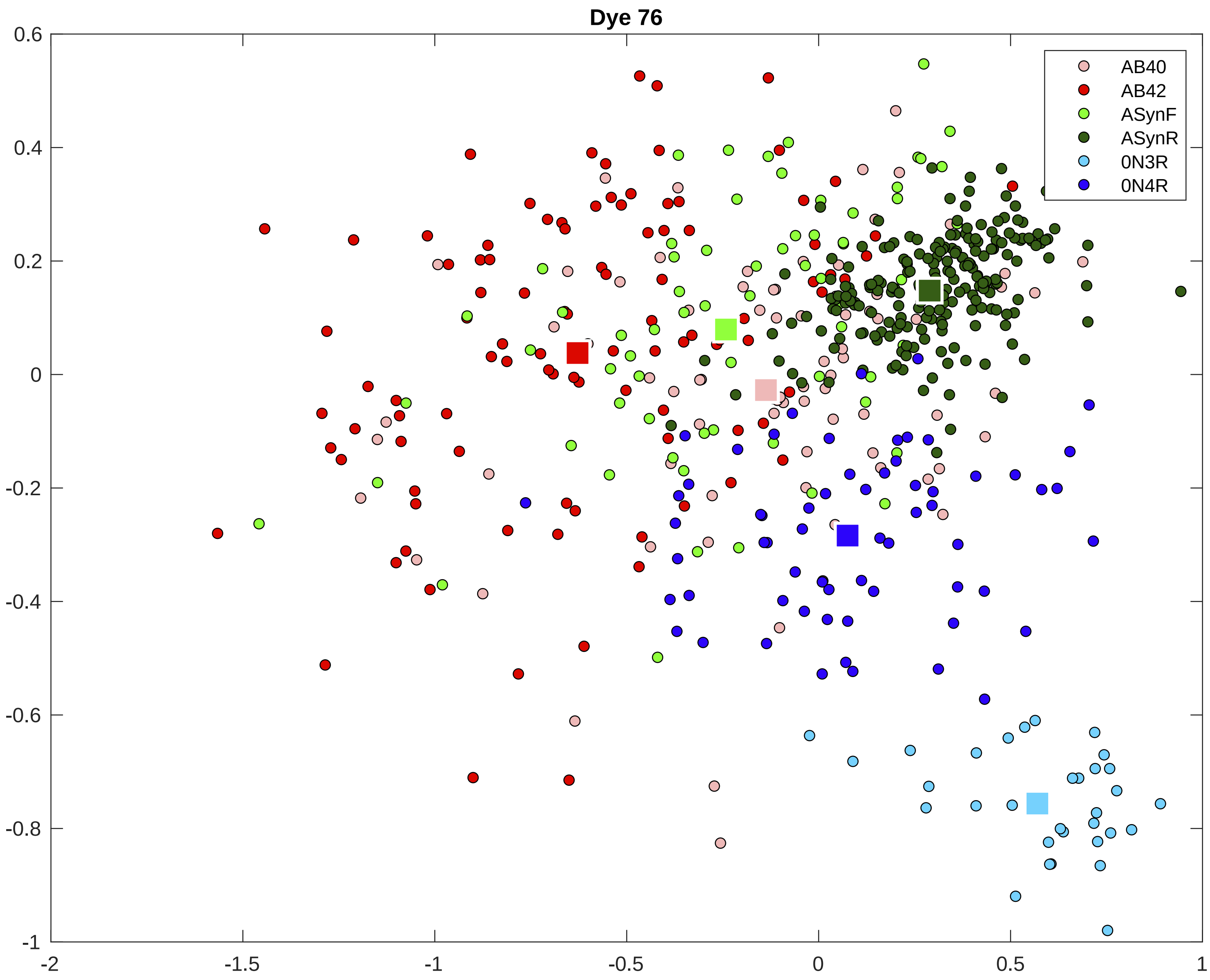

Dye 76
AB40
AB42
ASynF
ASynR
0N3R
0N4R
0.6
0.4
0.2
0
-0.2
-0.4
-0.6
-0.8
-1
-2
-1.5
-1
-0.5
0
0.5
1

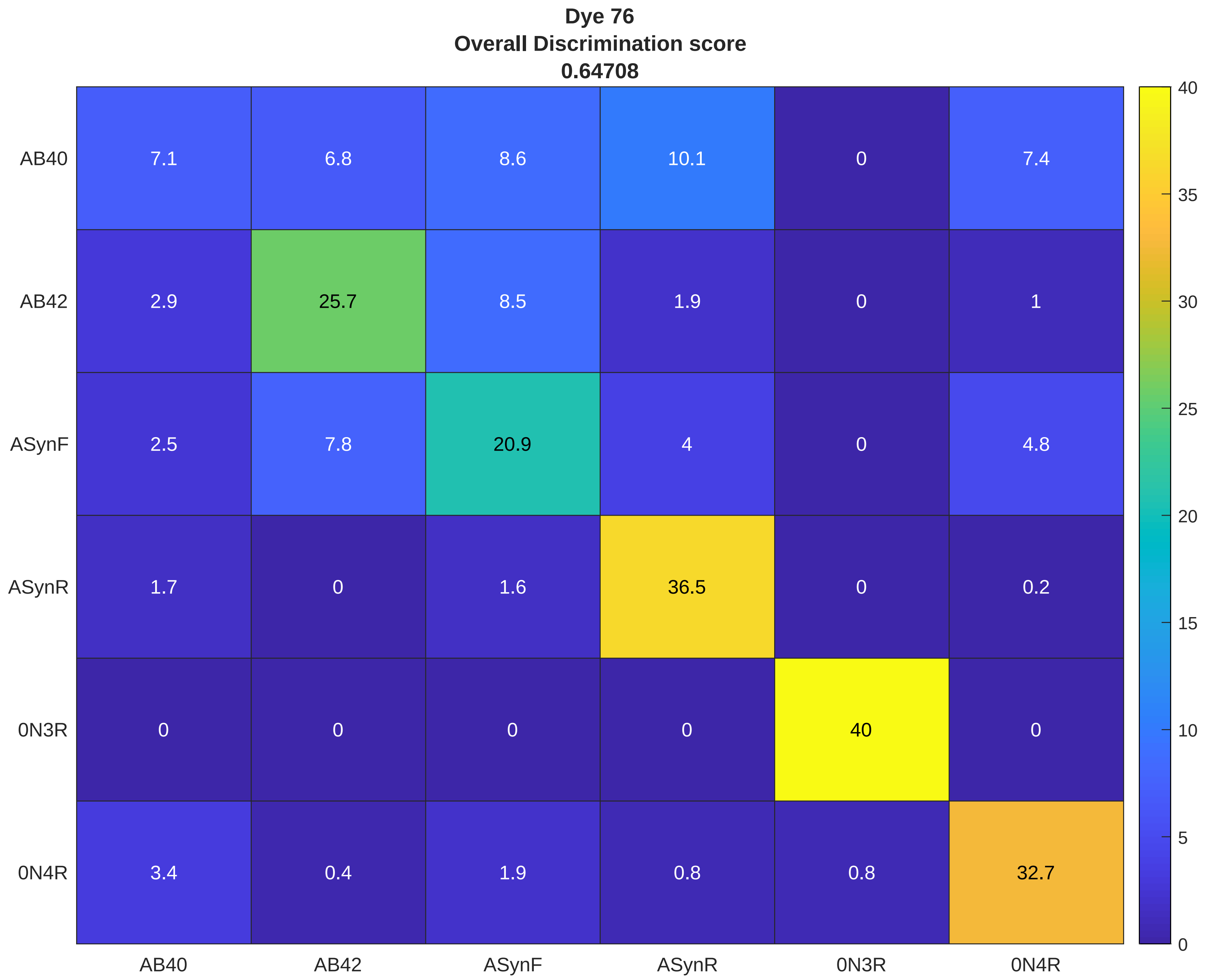
Dye 76
Overall Discrimination score
0.64708
AB40
AB42
ASynF
ASynR
0N3R
0N4R
7.1
6.8
8.6
10.1
0
7.4
2.9
25.7
8.5
1.9
0
1
2.5
7.8
20.9
4
0
4.8
1.7
0
1.6
36.5
0
0.2
0
0
0
0
40
0
3.4
0.4
1.9
0.8
0.8
32.7
AB40
AB42
ASynF
ASynR
0N3R
0N4R
40
35
30
25
20
15
10
5
0

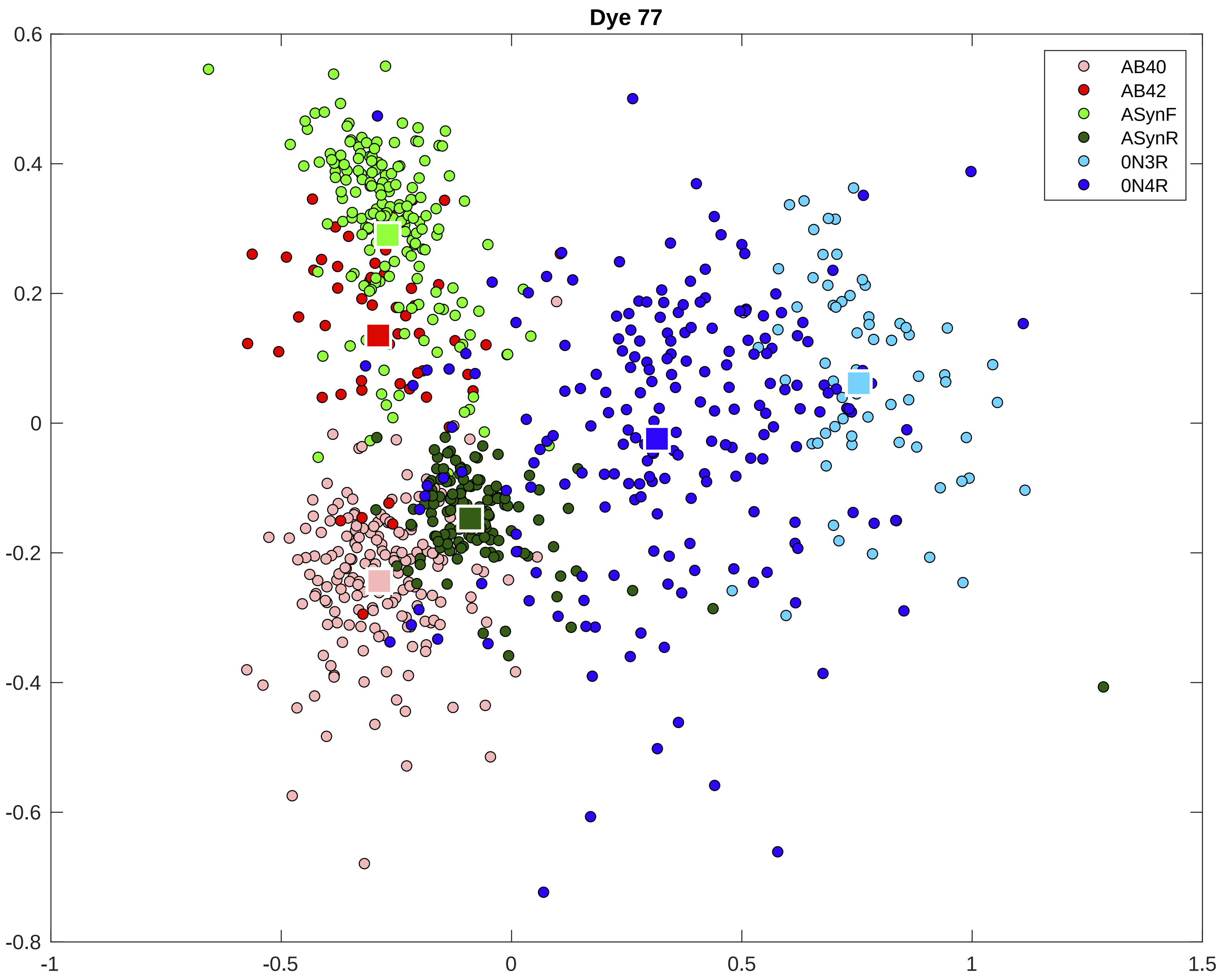
Dye 77
AB40
AB42
ASynF
ASynR
0N3R
0N4R
0.6
0.4
0.2
0
-0.2
-0.4
-0.6
-0.8
-1
-0.5
0
0.5
1
1.5

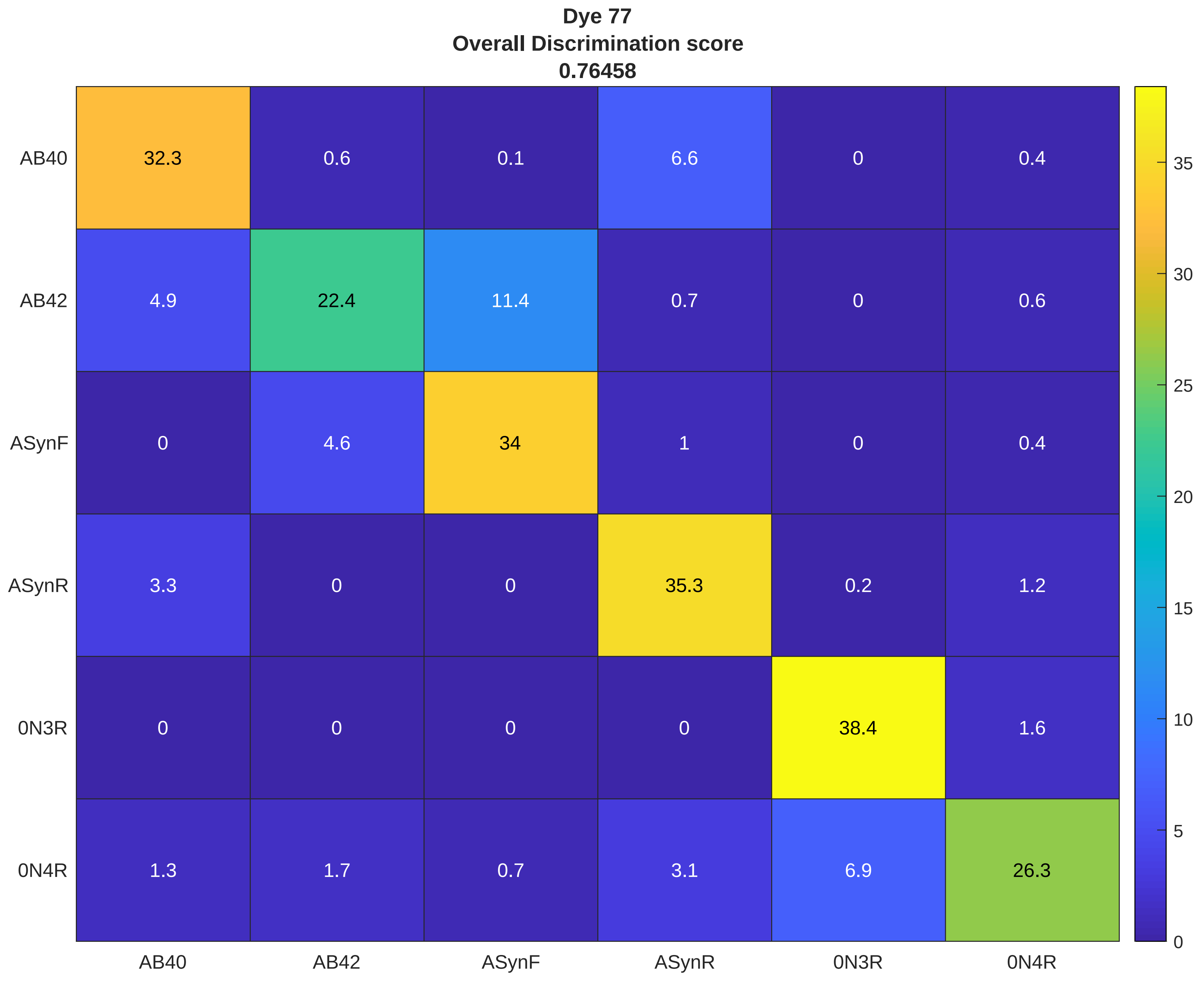
Dye 77
Overall Discrimination score
0.76458
AB40
AB42
ASynF
ASynR
0N3R
0N4R
32.3
0.6
0.1
6.6
0
0.4
4.9
22.4
11.4
0.7
0
0.6
0
4.6
34
1
0
0.4
3.3
0
0
35.3
0.2
1.2
0
0
0
0
38.4
1.6
1.3
1.7
0.7
3.1
6.9
26.3
AB40
AB42
ASynF
ASynR
0N3R
0N4R
35
30
25
20
15
10
5
0

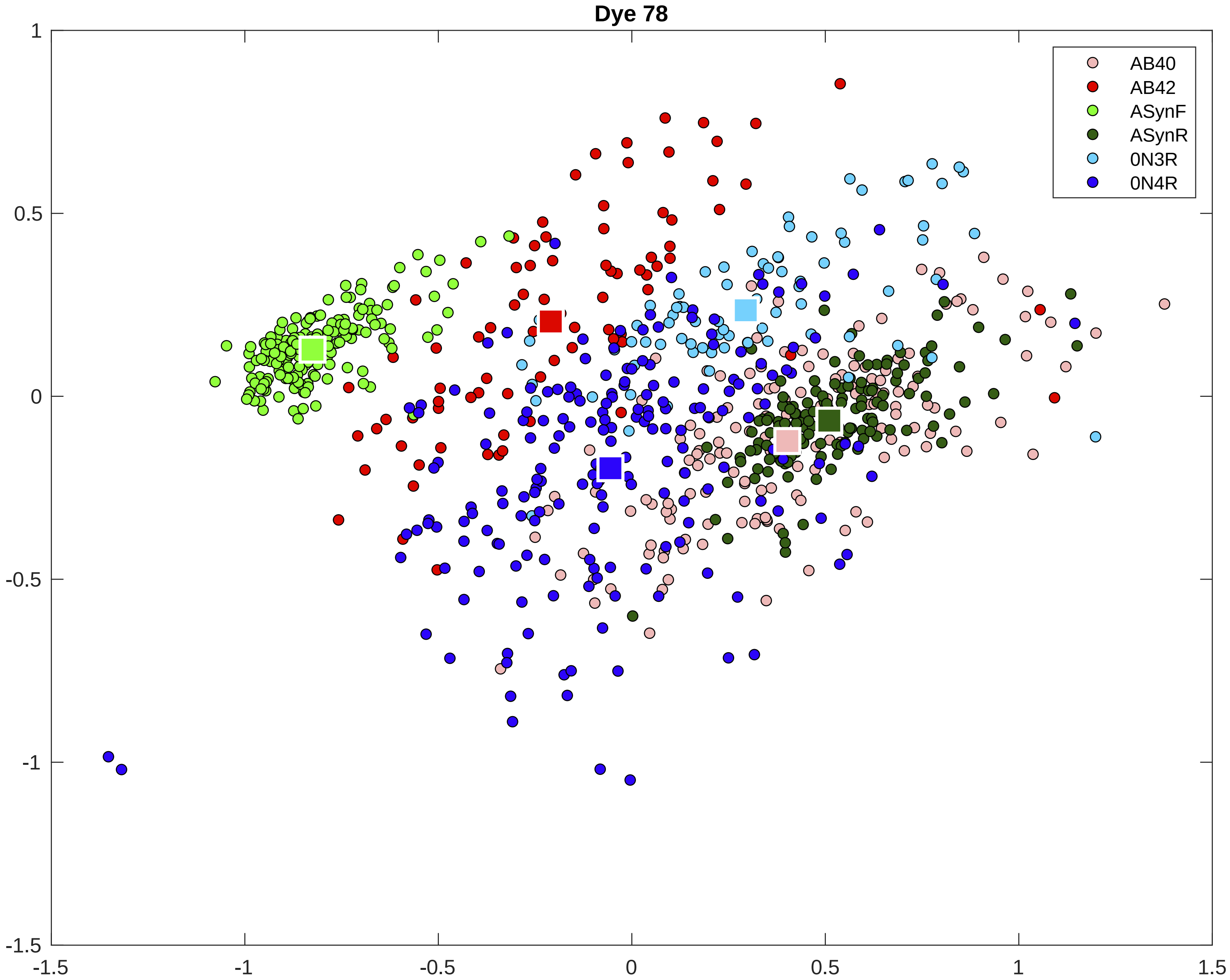

Dye 78
AB40
AB42
ASynF
ASynR
0N3R
0N4R
1
0.5
0
-0.5
-1
-1.5
-1.5
-1
-0.5
0
0.5
1
1.5

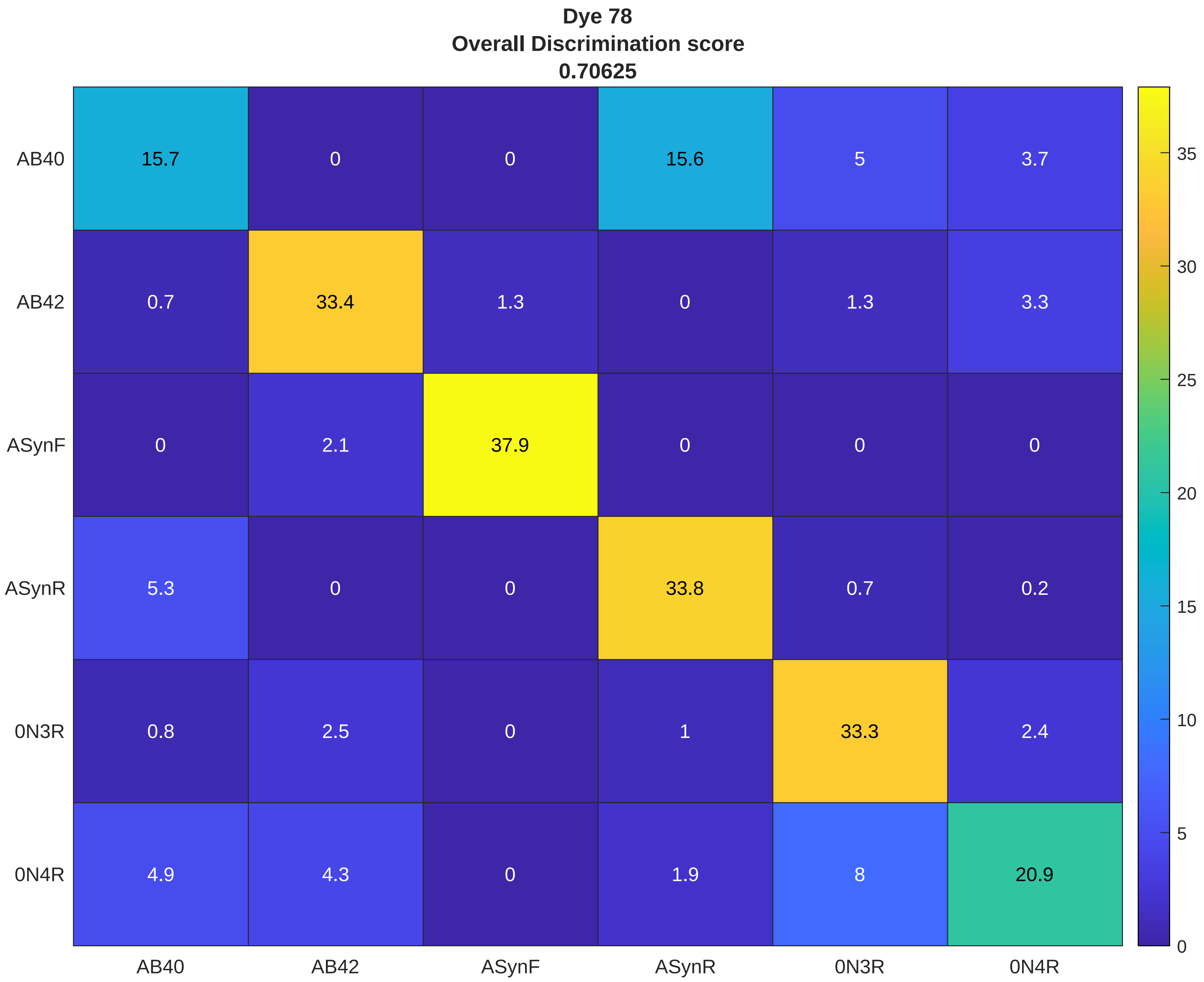

Dye 78
Overall Discrimination score
0.70625
AB40
AB42
ASynF
ASynR
0N3R
0N4R
15.7 0 0 15.6 5 3.7
0.7 33.4 1.3 0 1.3 3.3
0 2.1 37.9 0 0 0
5.3 0 0 33.8 0.7 0.2
0.8 2.5 0 1 33.3 2.4
4.9 4.3 0 1.9 8 20.9
AB40
AB42
ASynF
ASynR
0N3R
0N4R
35
30
25
20
15
10
5
0

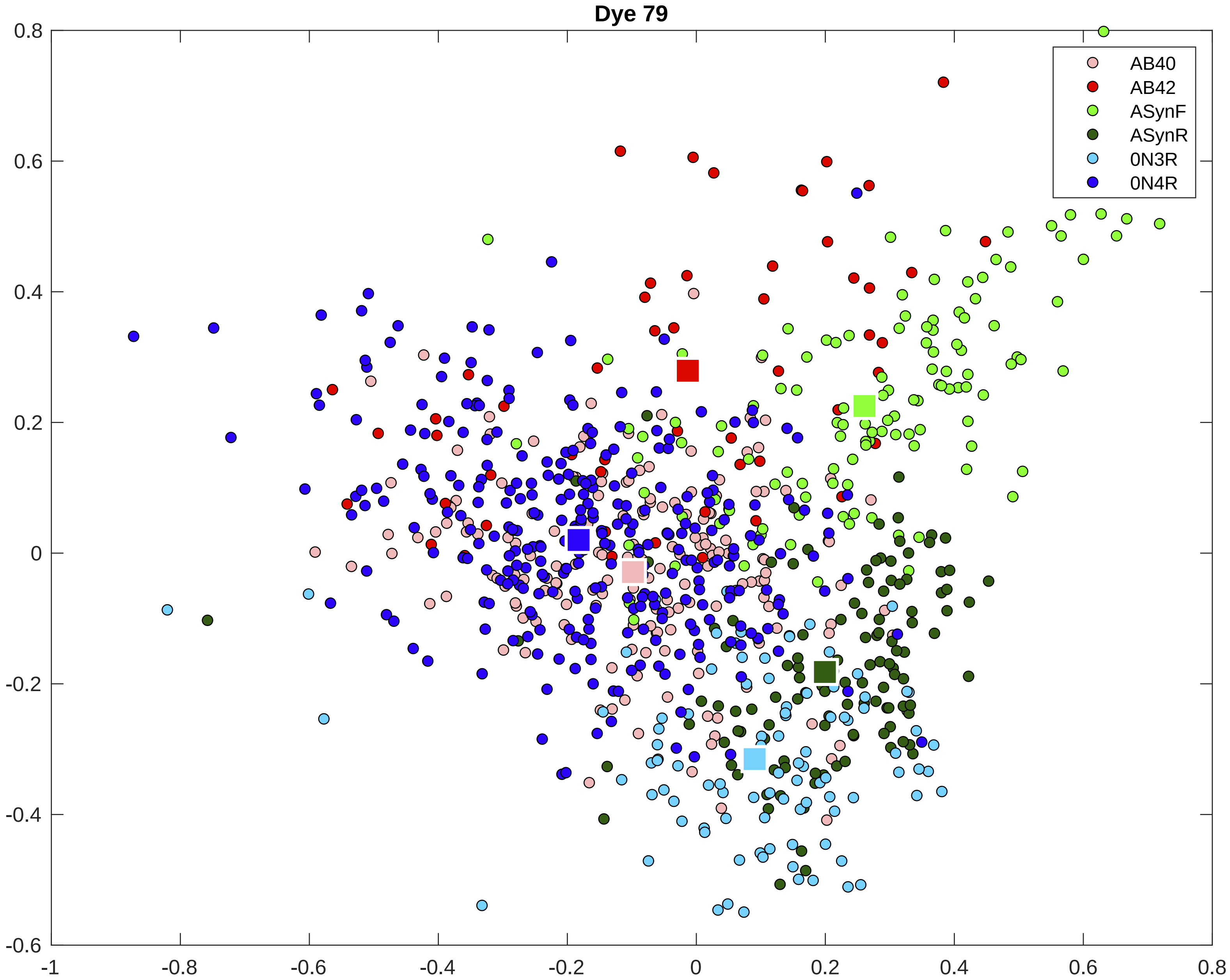

Dye 79
AB40
AB42
ASynF
ASynR
0N3R
0N4R
0.8
0.6
0.4
0.2
0
-0.2
-0.4
-0.6
-1
-0.8
-0.6
-0.4
-0.2
0
0.2
0.4
0.6
0.8

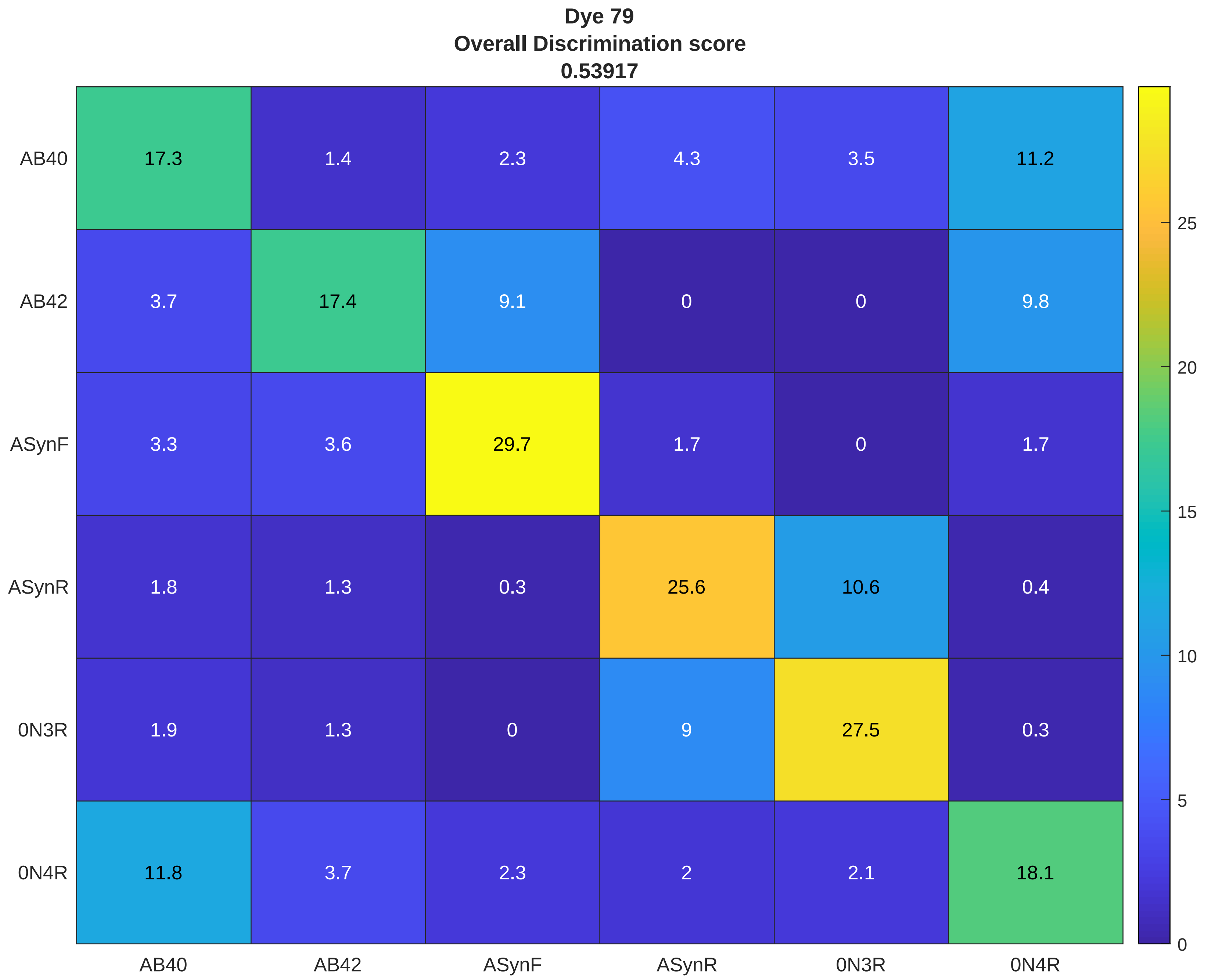
Dye 79
Overall Discrimination score
0.53917
AB40
AB42
ASynF
ASynR
0N3R
0N4R
17.3 1.4 2.3 4.3 3.5 11.2
3.7 17.4 9.1 0 0 9.8
3.3 3.6 29.7 1.7 0 1.7
1.8 1.3 0.3 25.6 10.6 0.4
1.9 1.3 0 9 27.5 0.3
11.8 3.7 2.3 2 2.1 18.1
AB40
AB42
ASynF
ASynR
0N3R
0N4R
0
5
10
15
20
25

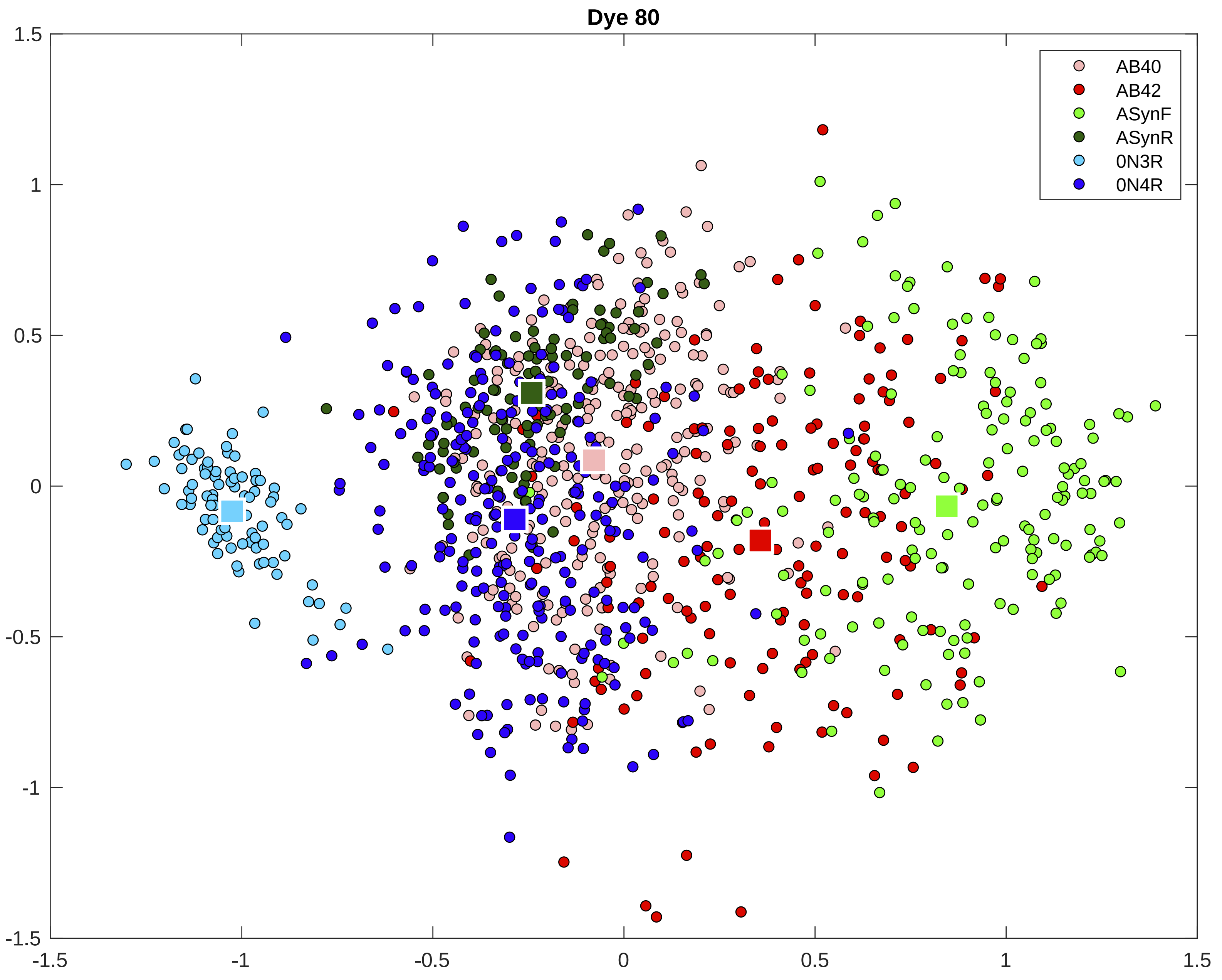

Dye 80
AB40
AB42
ASynF
ASynR
0N3R
0N4R
1.5
1
0.5
0
-0.5
-1
-1.5
-1.5
-1
-0.5
0
0.5
1
1.5

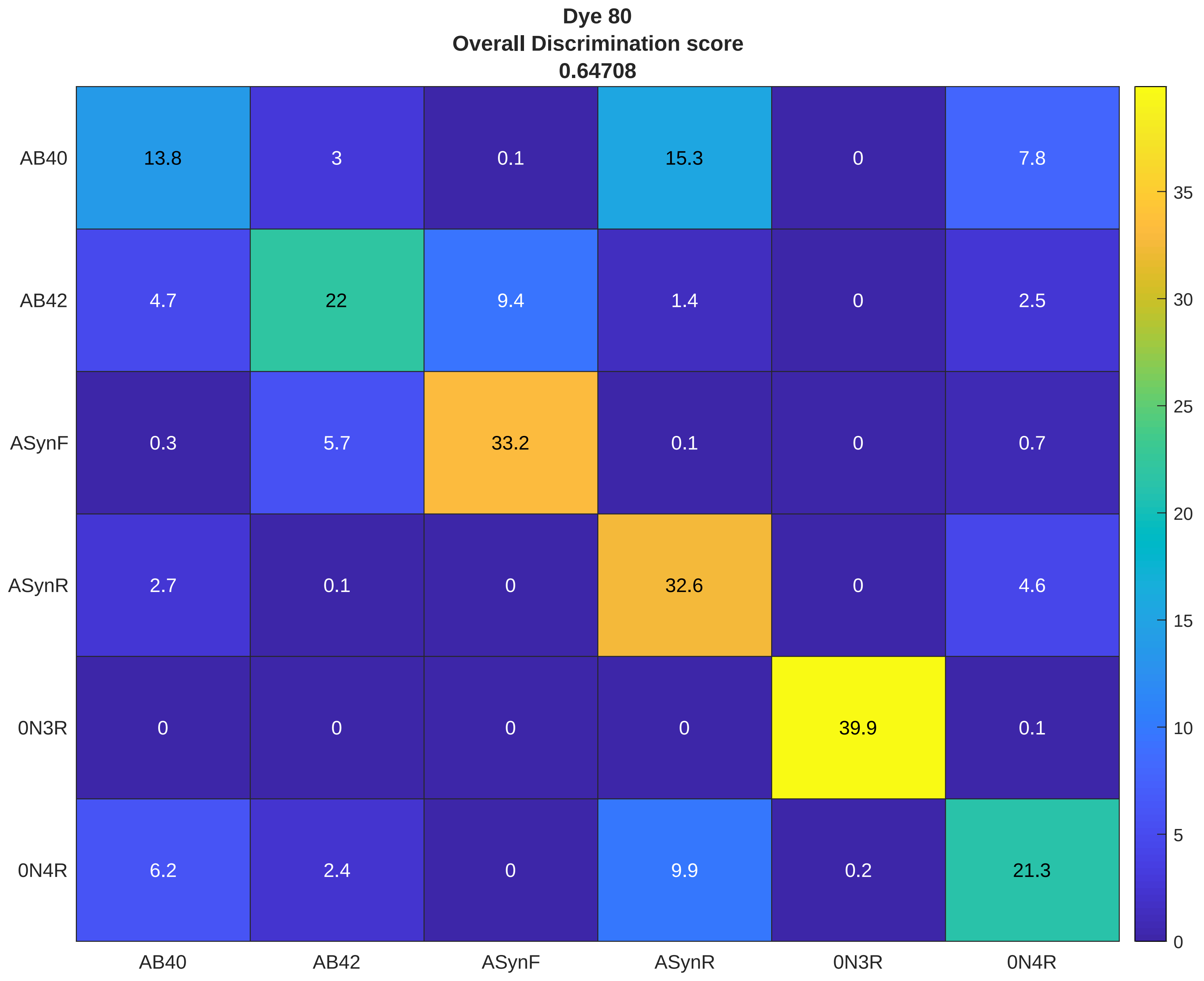
Dye 80
Overall Discrimination score
0.64708
AB40
AB42
ASynF
ASynR
0N3R
0N4R
13.8
3
0.1
15.3
0
7.8
4.7
22
9.4
1.4
0
2.5
0.3
5.7
33.2
0.1
0
0.7
2.7
0.1
0
32.6
0
4.6
0
0
0
0
39.9
0.1
6.2
2.4
0
9.9
0.2
21.3
AB40
AB42
ASynF
ASynR
0N3R
0N4R
35
30
25
20
15
10
5
0

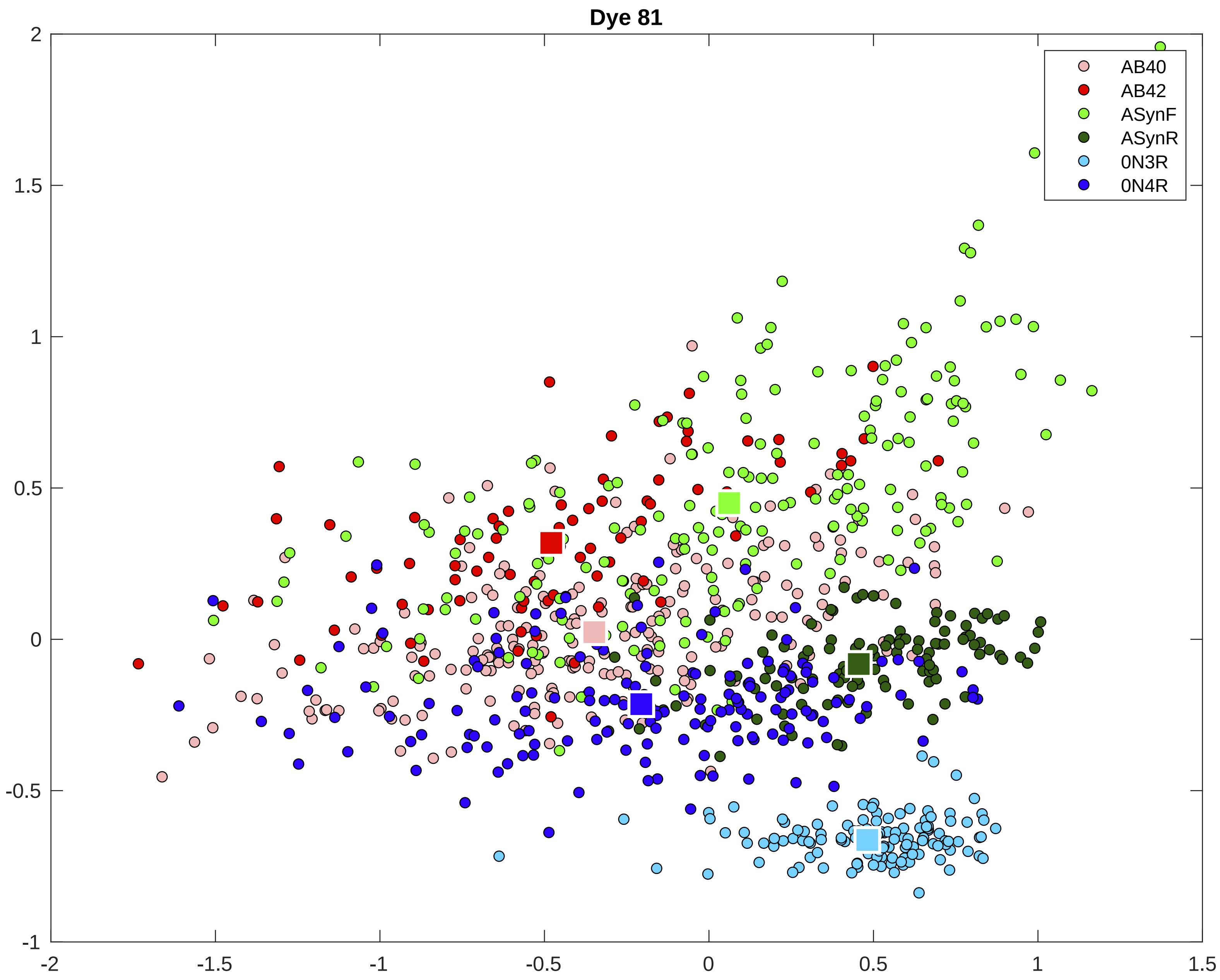

Dye 81
AB40
AB42
ASynF
ASynR
0N3R
0N4R
2
1.5
1
0.5
0
-0.5
-1
-2
-1.5
-1
-0.5
0
0.5
1
1.5

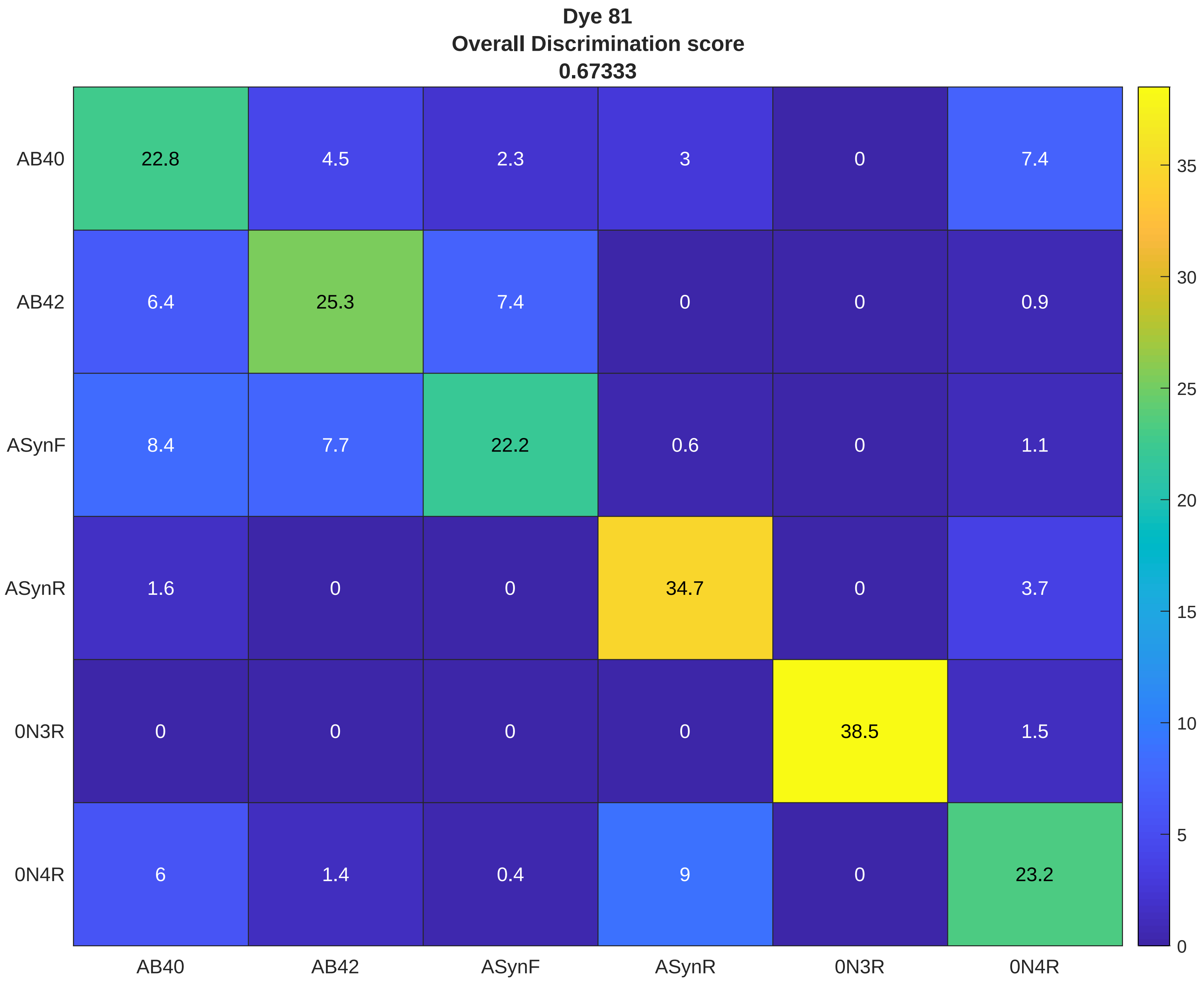
Dye 81
Overall Discrimination score
0.67333
AB40
AB42
ASynF
ASynR
0N3R
0N4R
22.8
4.5
2.3
3
0
7.4
6.4
25.3
7.4
0
0
0.9
8.4
7.7
22.2
0.6
0
1.1
1.6
0
0
34.7
0
3.7
0
0
0
0
38.5
1.5
6
1.4
0.4
9
0
23.2
AB40
AB42
ASynF
ASynR
0N3R
0N4R
35
30
25
20
15
10
5
0

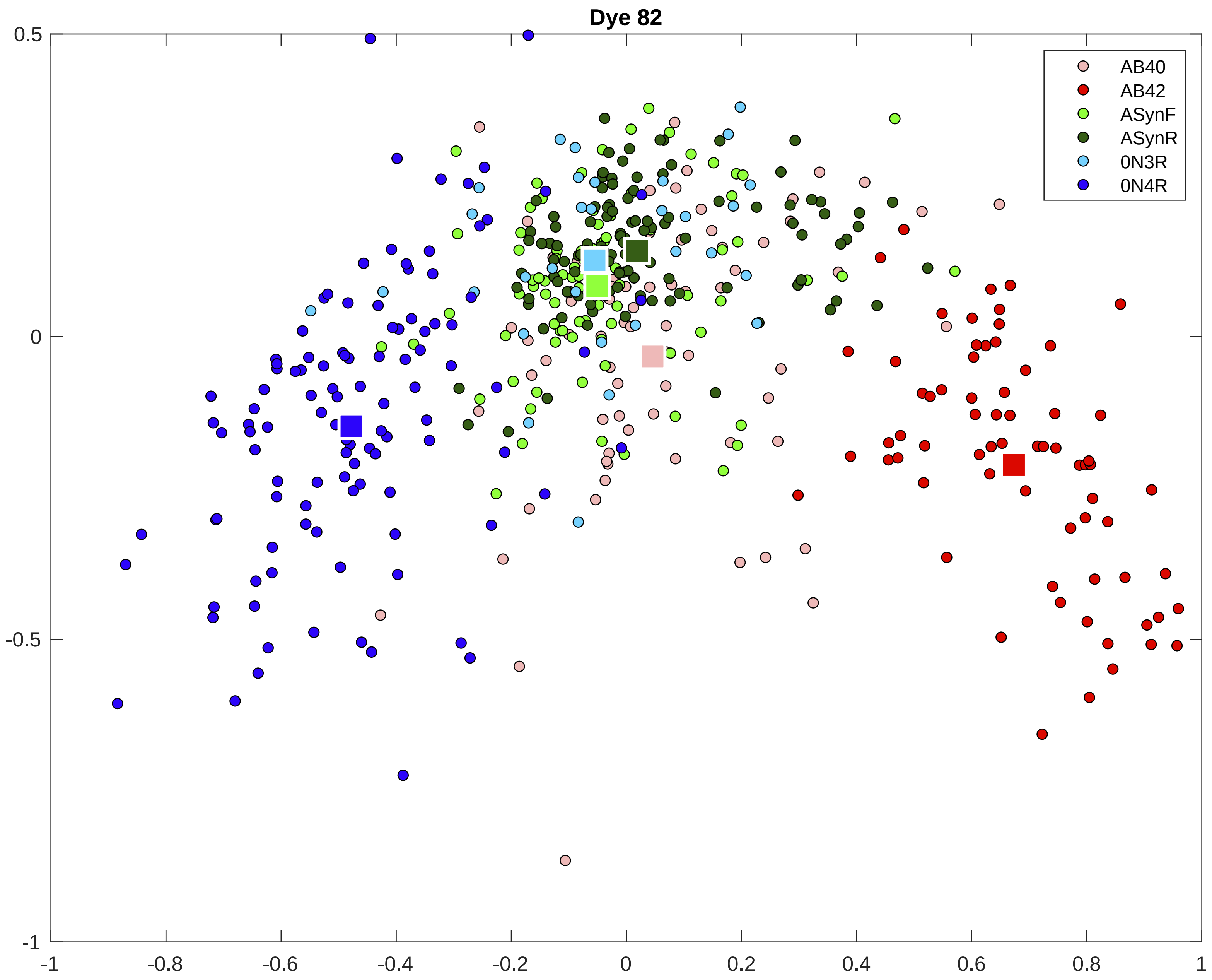
Dye 82
0.5
0
-0.5
-1
-1
-0.8
-0.6
-0.4
-0.2
0
0.2
0.4
0.6
0.8
1
AB40
AB42
ASynF
ASynR
0N3R
0N4R

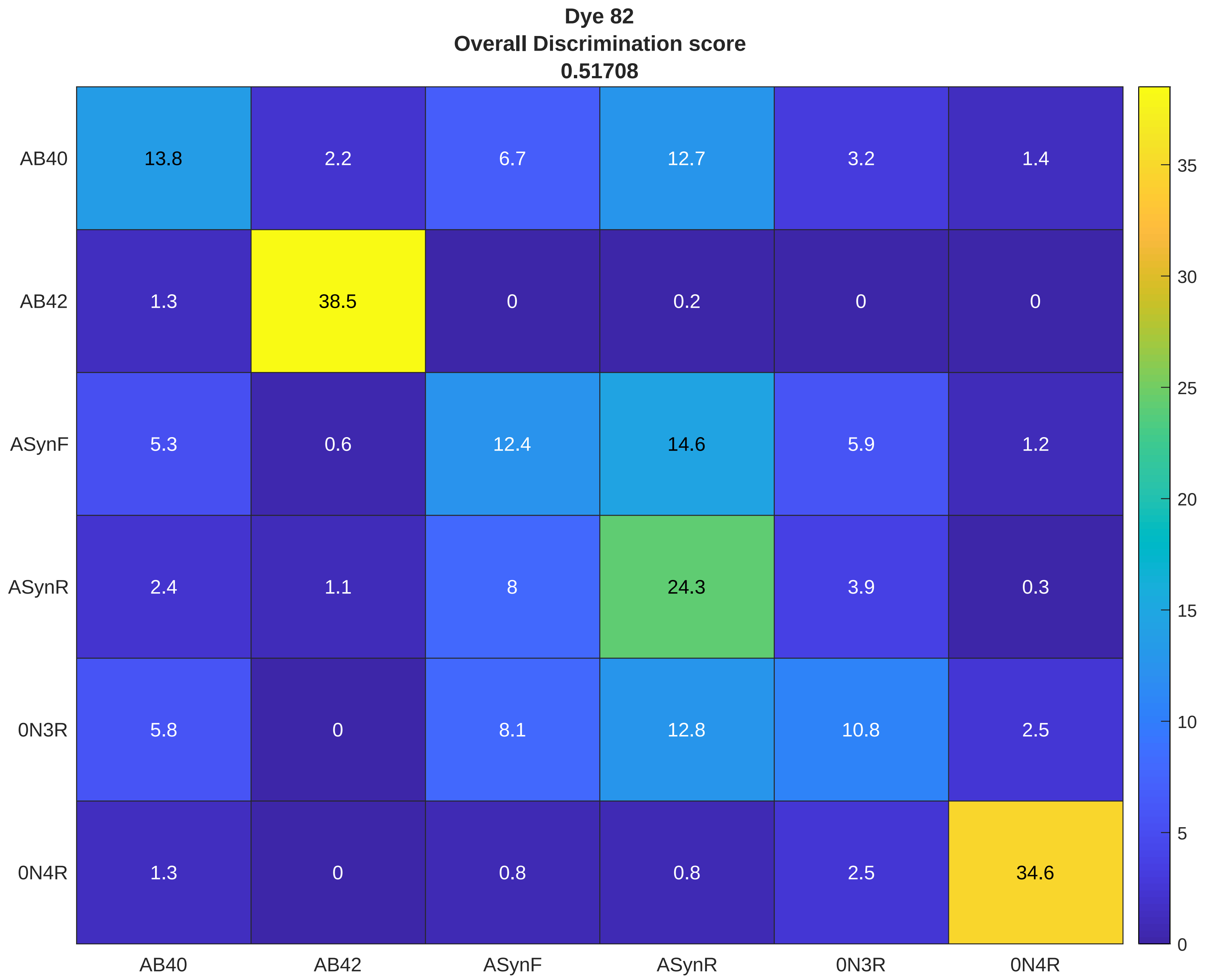
Dye 82
Overall Discrimination score
0.51708
AB40
AB42
ASynF
ASynR
0N3R
0N4R
13.8
2.2
6.7
12.7
3.2
1.4
1.3
38.5
0
0.2
0
0
5.3
0.6
12.4
14.6
5.9
1.2
2.4
1.1
8
24.3
3.9
0.3
5.8
0
8.1
12.8
10.8
2.5
1.3
0
0.8
0.8
2.5
34.6
AB40
AB42
ASynF
ASynR
0N3R
0N4R
35
30
25
20
15
10
5
0

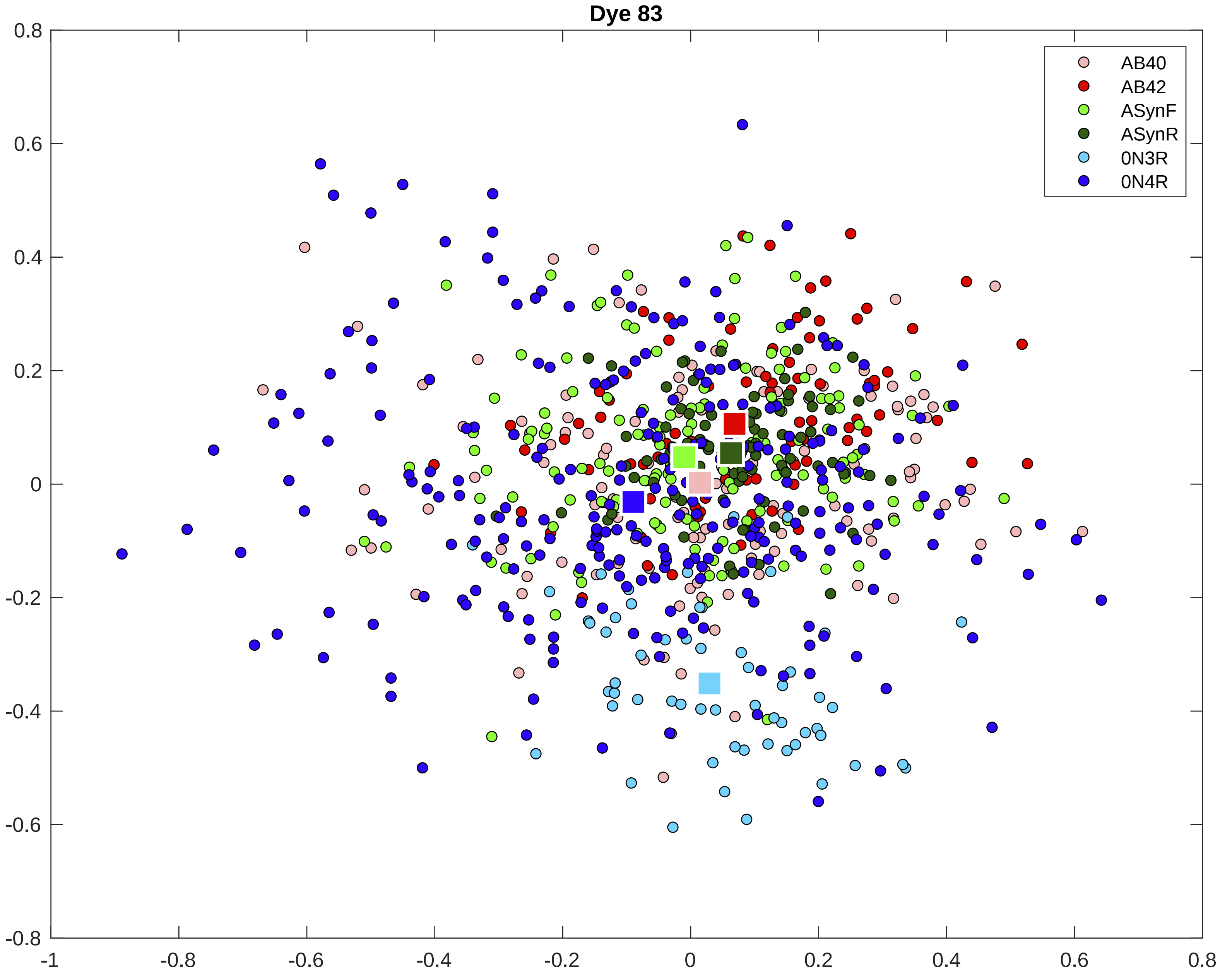
Dye 83
AB40
AB42
ASynF
ASynR
0N3R
0N4R
0.8
0.6
0.4
0.2
0
-0.2
-0.4
-0.6
-0.8
-1
-0.8
-0.6
-0.4
-0.2
0
0.2
0.4
0.6
0.8

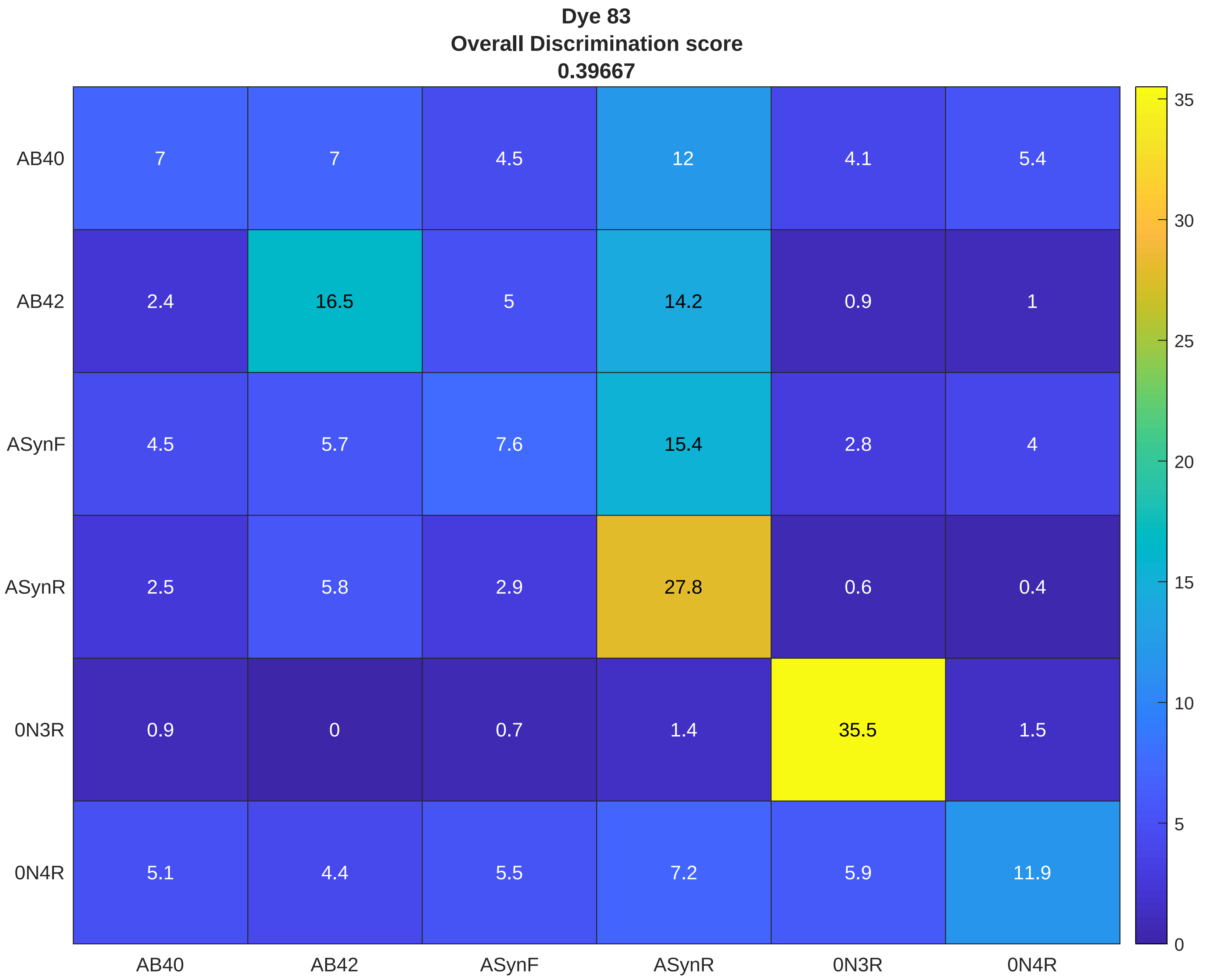

Dye 83
Overall Discrimination score
0.39667
AB40
AB42
ASynF
ASynR
0N3R
0N4R
7 7 4.5 12 4.1 5.4
2.4 16.5 5 14.2 0.9 1
4.5 5.7 7.6 15.4 2.8 4
2.5 5.8 2.9 27.8 0.6 0.4
0.9 0 0.7 1.4 35.5 1.5
5.1 4.4 5.5 7.2 5.9 11.9
AB40
AB42
ASynF
ASynR
0N3R
0N4R
35
30
25
20
15
10
5
0

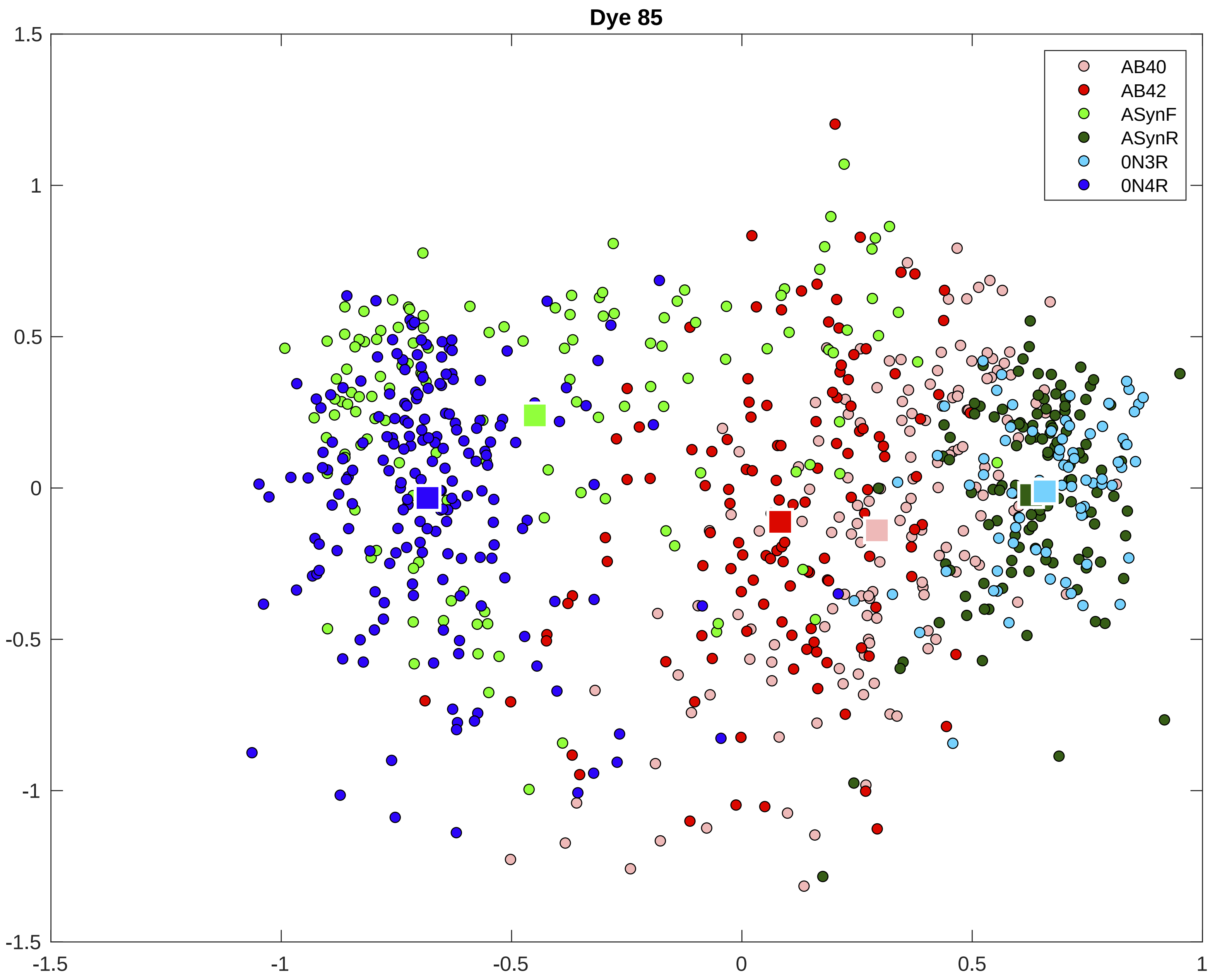

Dye 85
AB40
AB42
ASynF
ASynR
0N3R
0N4R
1.5
1
0.5
0
-0.5
-1
-1.5
-1.5
-1
-0.5
0
0.5
1

Dye 85
Overall Discrimination score
0.54583

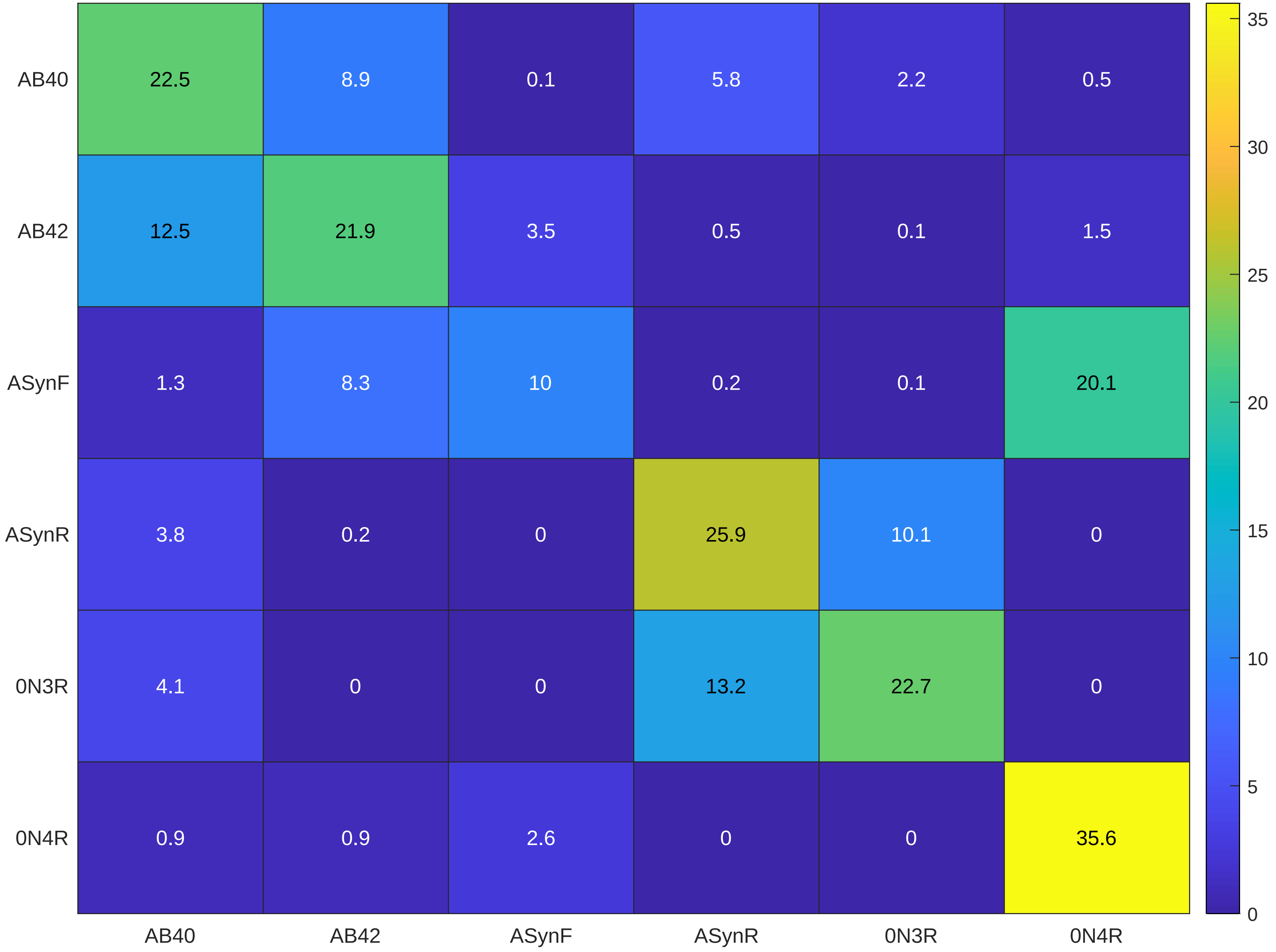

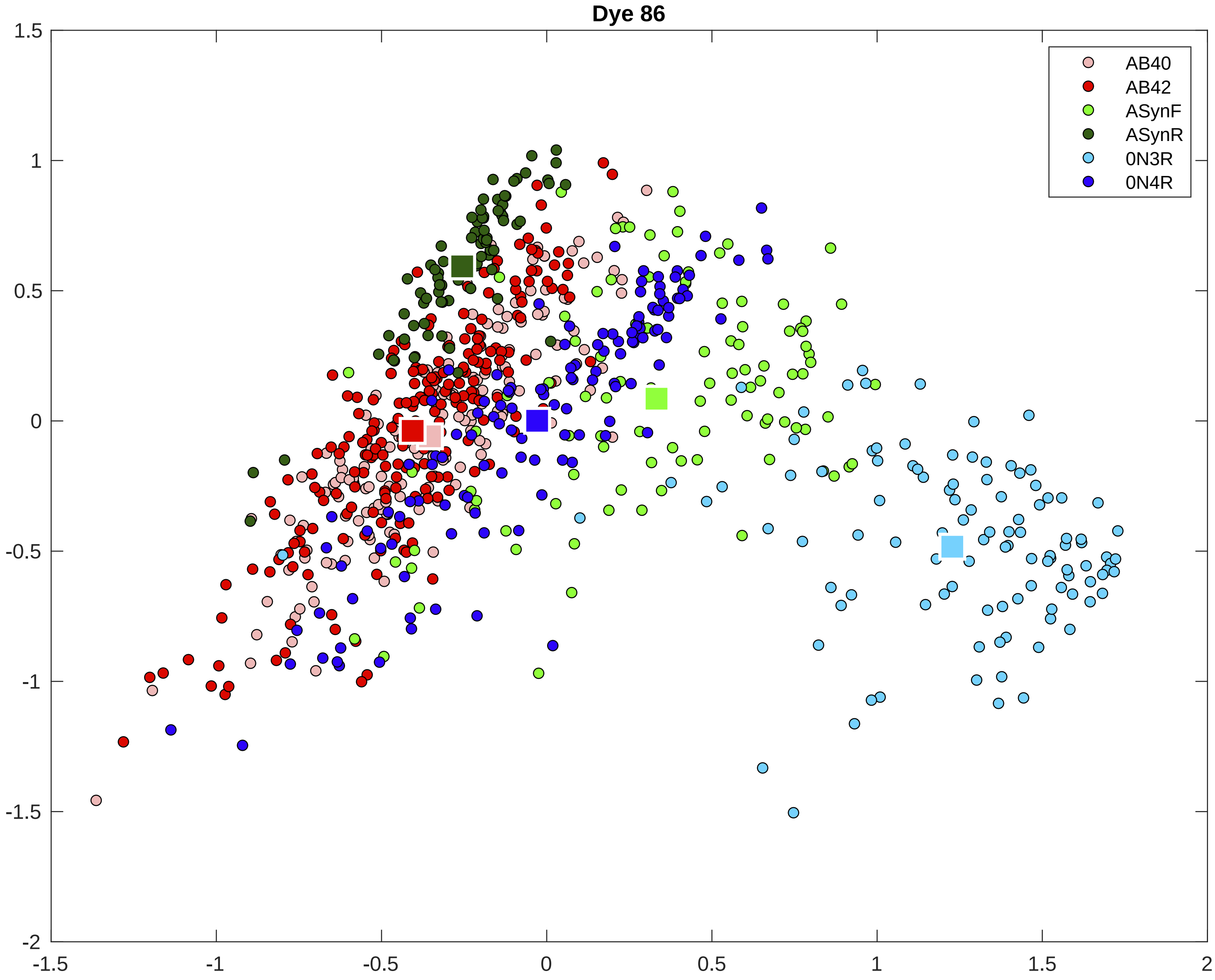

Dye 86
AB40
AB42
ASynF
ASynR
0N3R
0N4R
1.5
1
0.5
0
-0.5
-1
-1.5
-2
-1.5
-1
-0.5
0
0.5
1
1.5
2

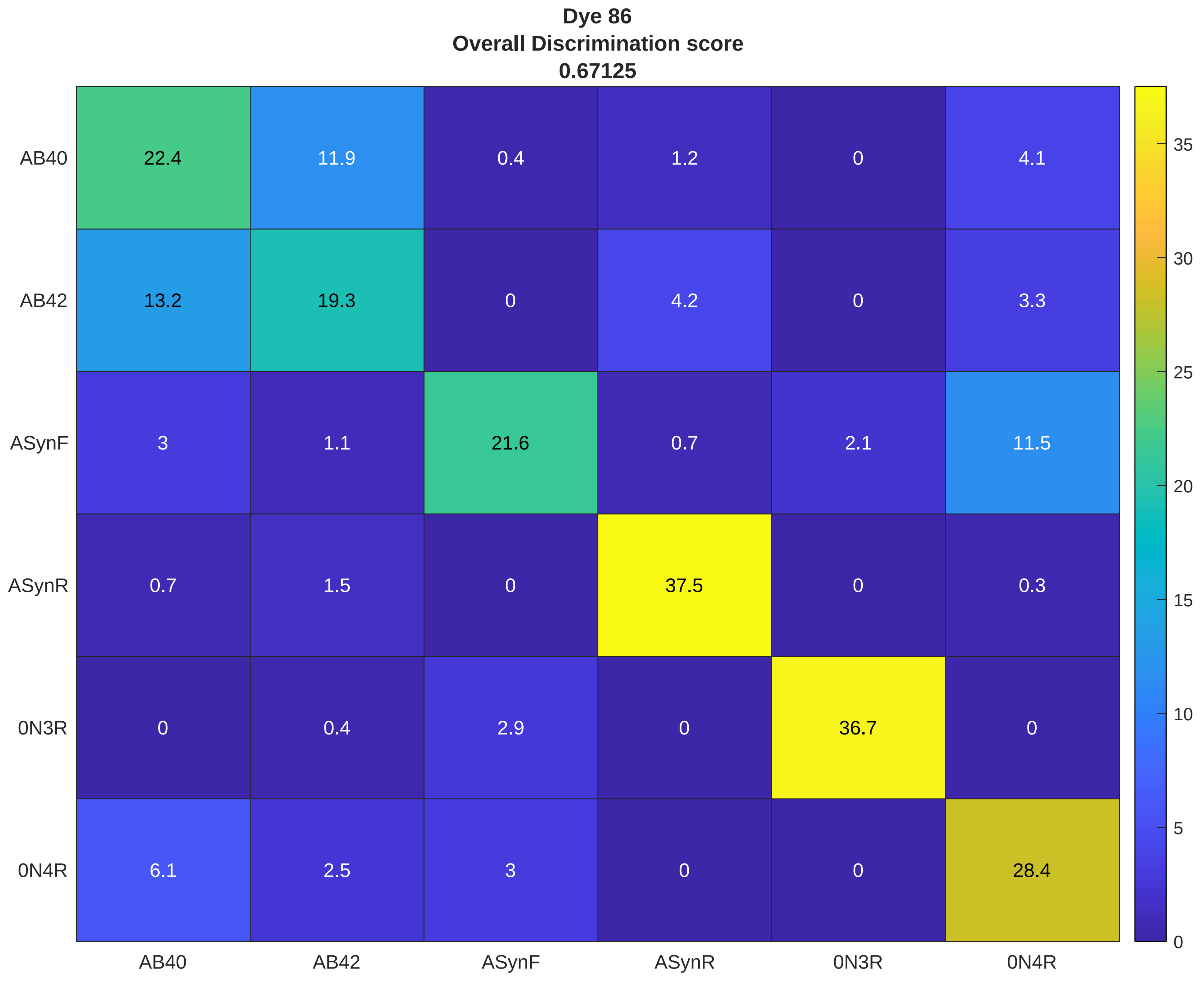
Dye 86
Overall Discrimination score
0.67125
AB40
AB42
ASynF
ASynR
0N3R
0N4R
22.4
11.9
0.4
1.2
0
4.1
13.2
19.3
0
4.2
0
3.3
3
1.1
21.6
0.7
2.1
11.5
0.7
1.5
0
37.5
0
0.3
0
0.4
2.9
0
36.7
0
6.1
2.5
3
0
0
28.4
AB40
AB42
ASynF
ASynR
0N3R
0N4R
35
30
25
20
15
10
5
0

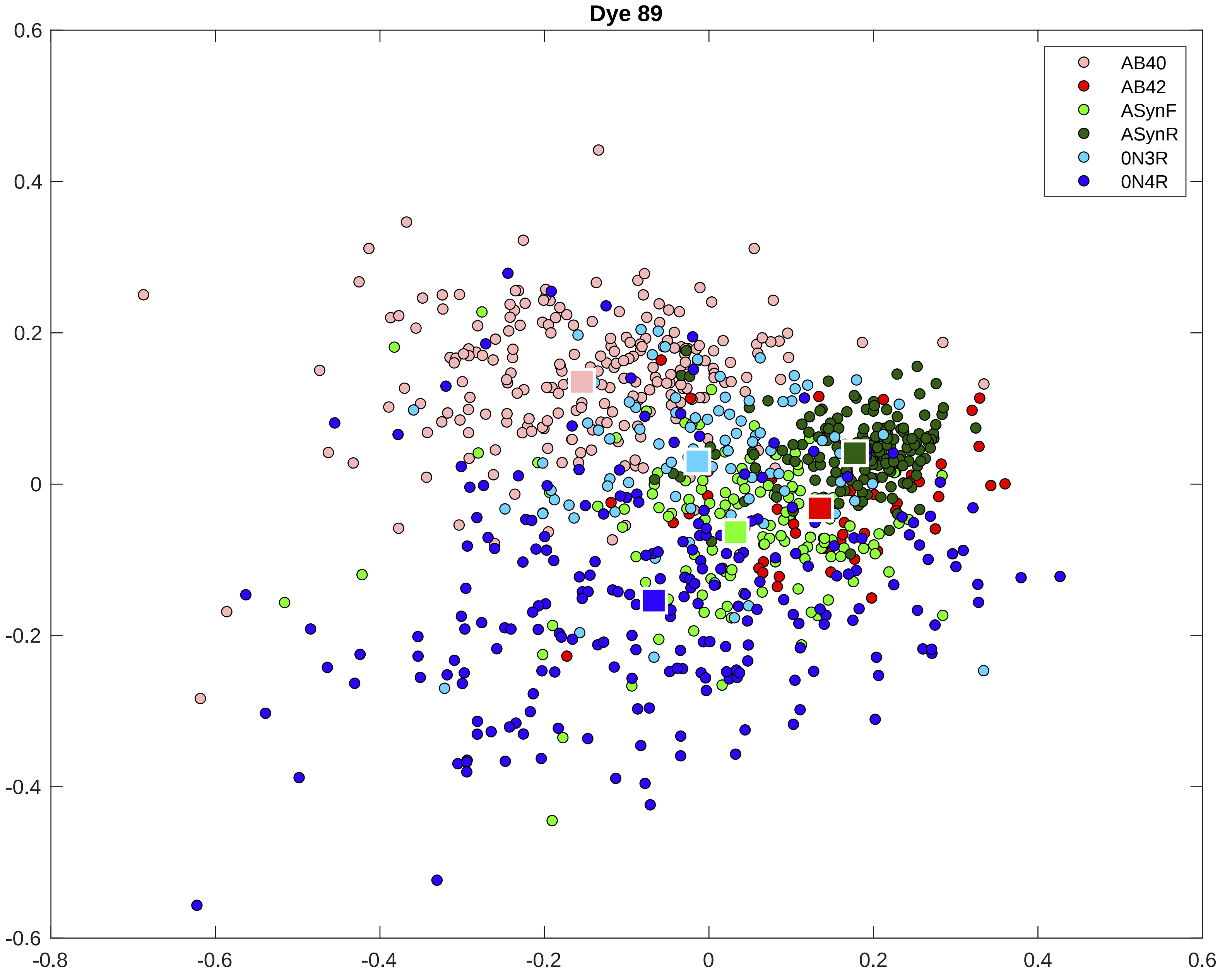
Dye 89
0.6
0.4
0.2
0
-0.2
-0.4
-0.6
-0.8
-0.6
-0.4
-0.2
0
0.2
0.4
0.6
AB40
AB42
ASynF
ASynR
0N3R
0N4R

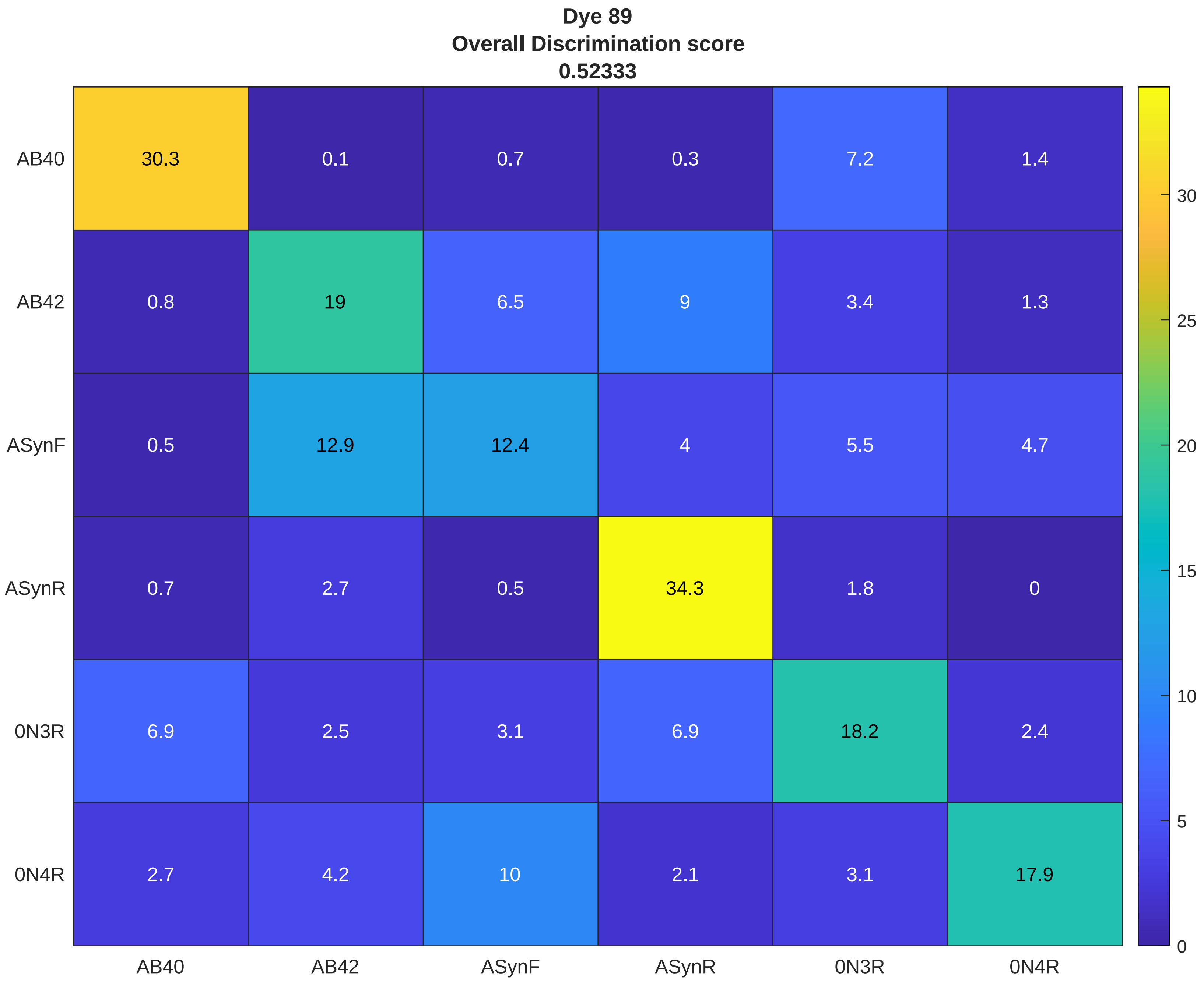

Dye 89
Overall Discrimination score
0.52333
AB40
AB42
ASynF
ASynR
0N3R
0N4R
30.3 0.1 0.7 0.3 7.2 1.4
0.8 19 6.5 9 3.4 1.3
0.5 12.9 12.4 4 5.5 4.7
0.7 2.7 0.5 34.3 1.8 0
6.9 2.5 3.1 6.9 18.2 2.4
2.7 4.2 10 2.1 3.1 17.9
AB40
AB42
ASynF
ASynR
0N3R
0N4R
30
25
20
15
10
5
0

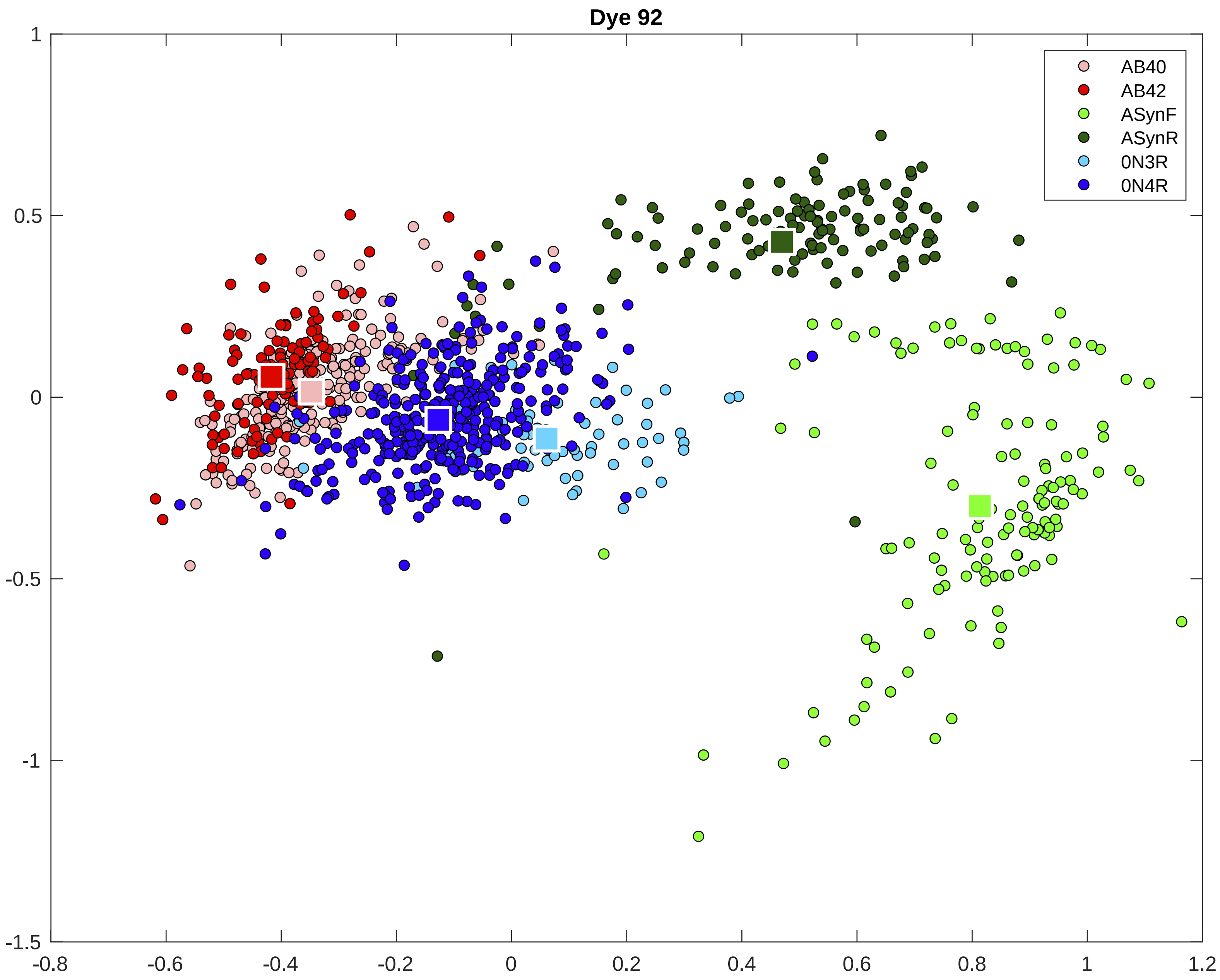

Dye 92
AB40
AB42
ASynF
ASynR
0N3R
0N4R
1
0.5
0
-0.5
-1
-1.5
-0.8
-0.6
-0.4
-0.2
0
0.2
0.4
0.6
0.8
1
1.2

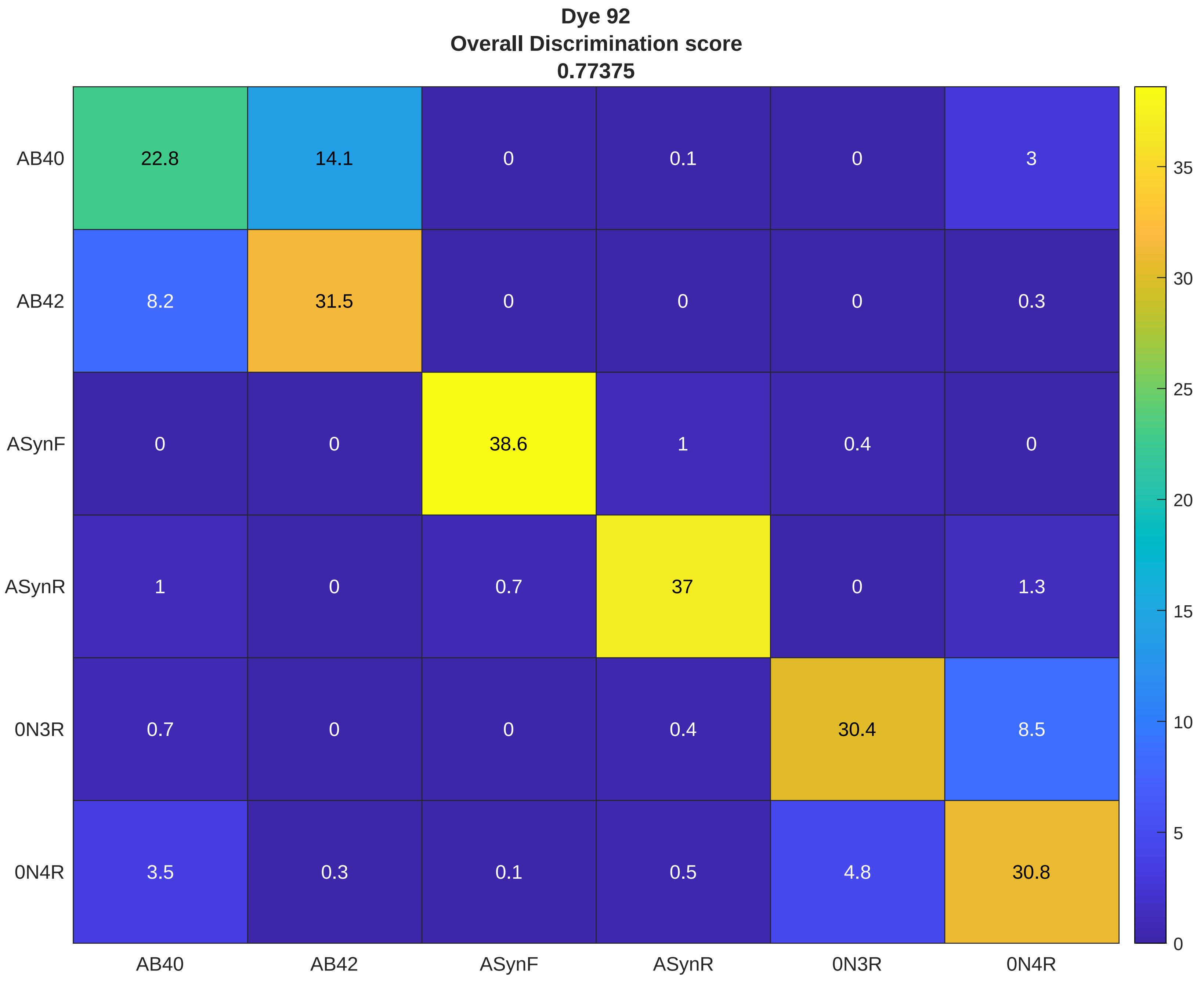
Dye 92
Overall Discrimination score
0.77375
AB40
AB42
ASynF
ASynR
0N3R
0N4R
22.8
14.1
0
0.1
0
3
8.2
31.5
0
0
0
0.3
0
0
38.6
1
0.4
0
1
0
0.7
37
0
1.3
0.7
0
0
0.4
30.4
8.5
3.5
0.3
0.1
0.5
4.8
30.8
AB40
AB42
ASynF
ASynR
0N3R
0N4R
35
30
25
20
15
10
5
0

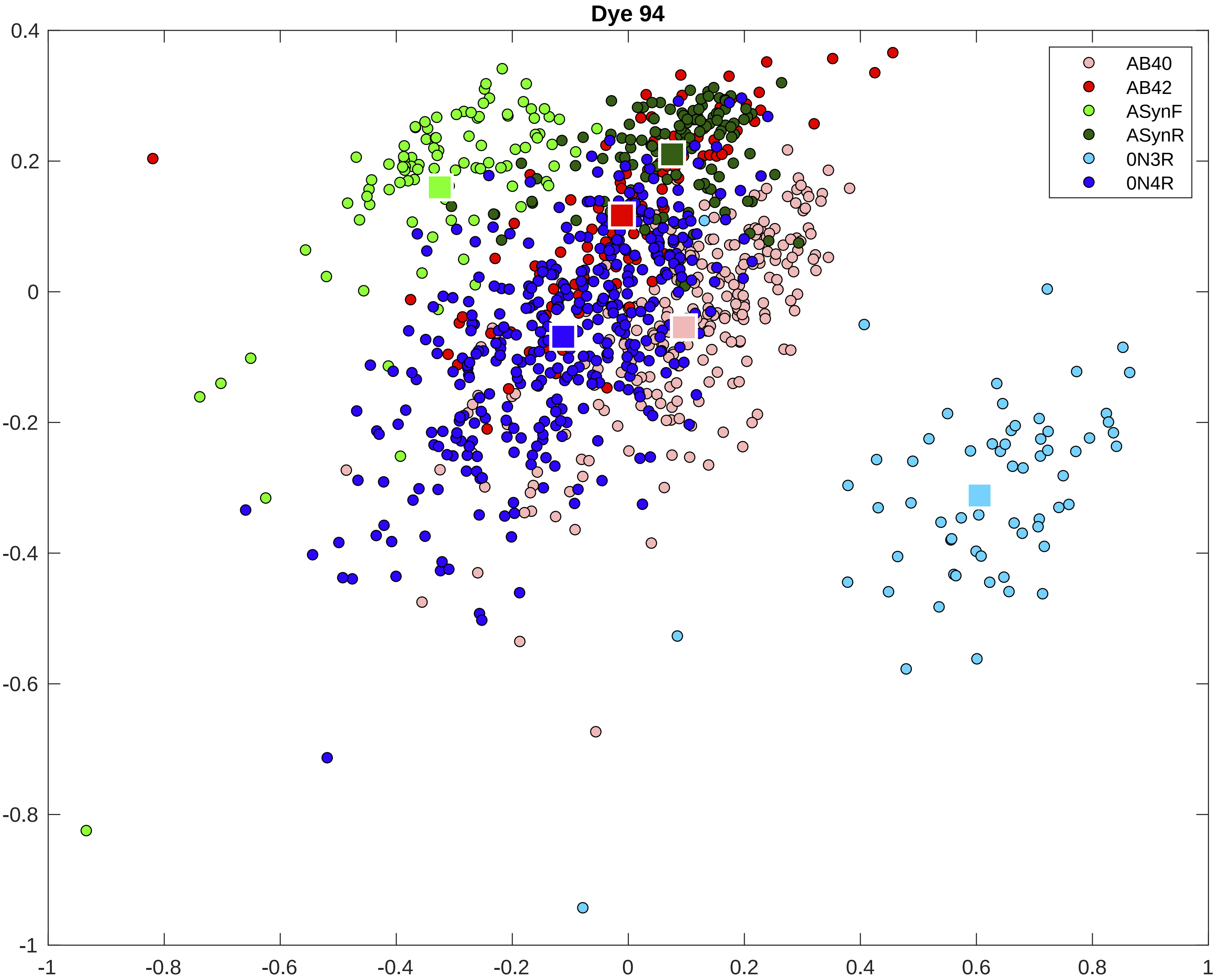

Dye 94
AB40
AB42
ASynF
ASynR
0N3R
0N4R
0.4
0.2
0
-0.2
-0.4
-0.6
-0.8
-1
-0.8
-0.6
-0.4
-0.2
0
0.2
0.4
0.6
0.8
1

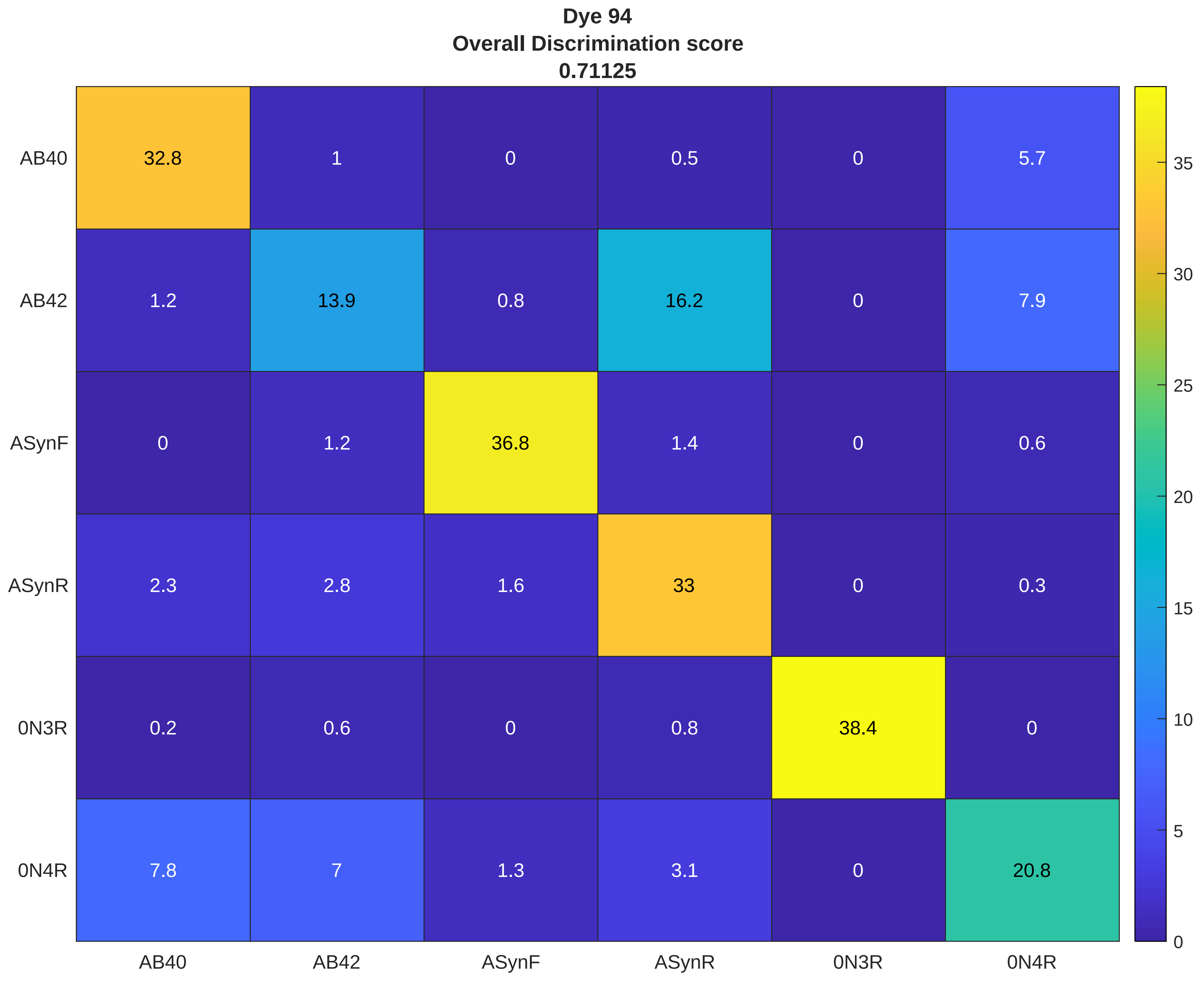
Dye 94
Overall Discrimination score
0.71125
AB40
AB42
ASynF
ASynR
0N3R
0N4R
32.8
1
0
0.5
0
5.7
1.2
13.9
0.8
16.2
0
7.9
0
1.2
36.8
1.4
0
0.6
2.3
2.8
1.6
33
0
0.3
0.2
0.6
0
0.8
38.4
0
7.8
7
1.3
3.1
0
20.8
AB40
AB42
ASynF
ASynR
0N3R
0N4R
35
30
25
20
15
10
5
0

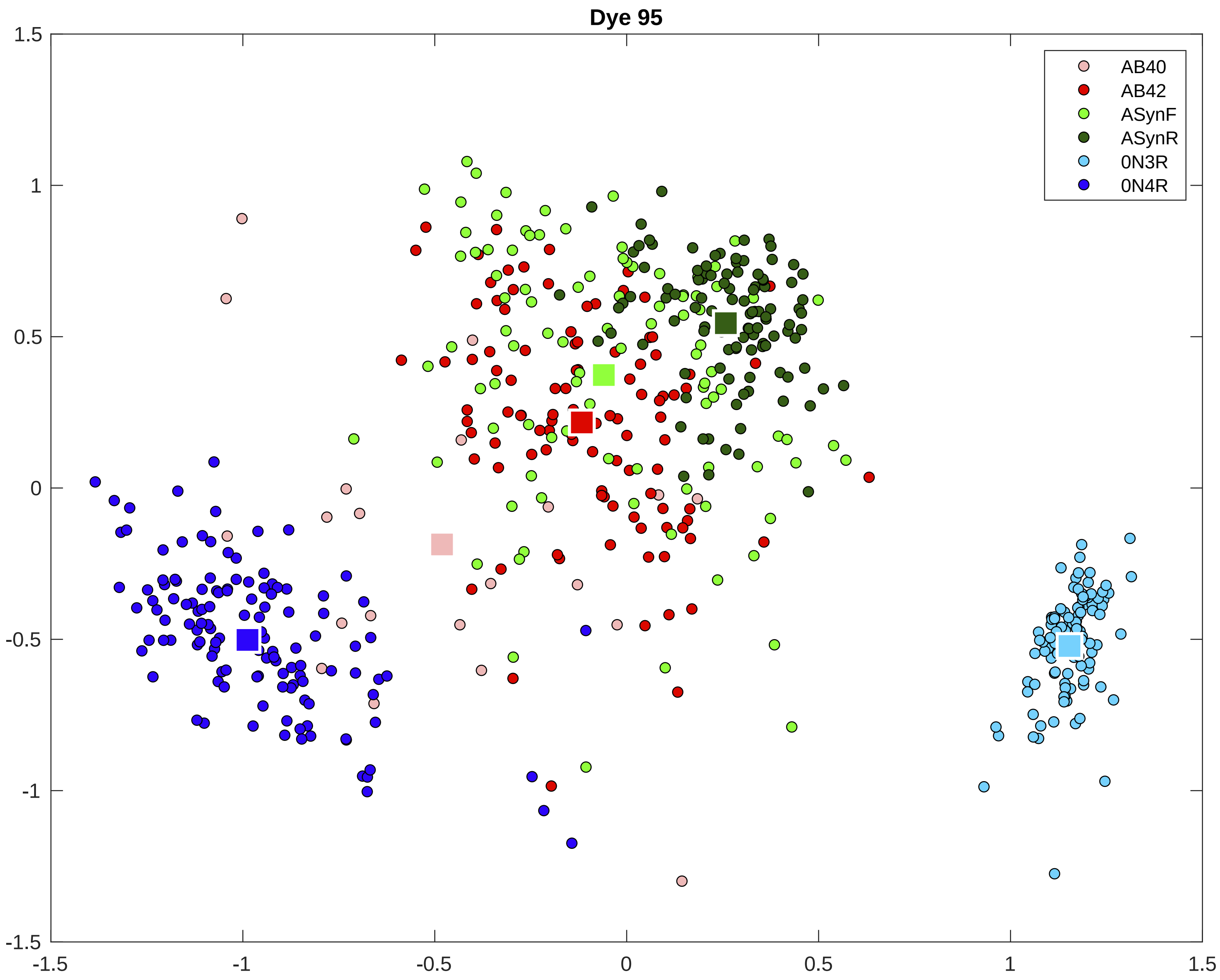
Dye 95
AB40
AB42
ASynF
ASynR
0N3R
0N4R
1.5
1
0.5
0
-0.5
-1
-1.5
-1.5
-1
-0.5
0
0.5
1
1.5

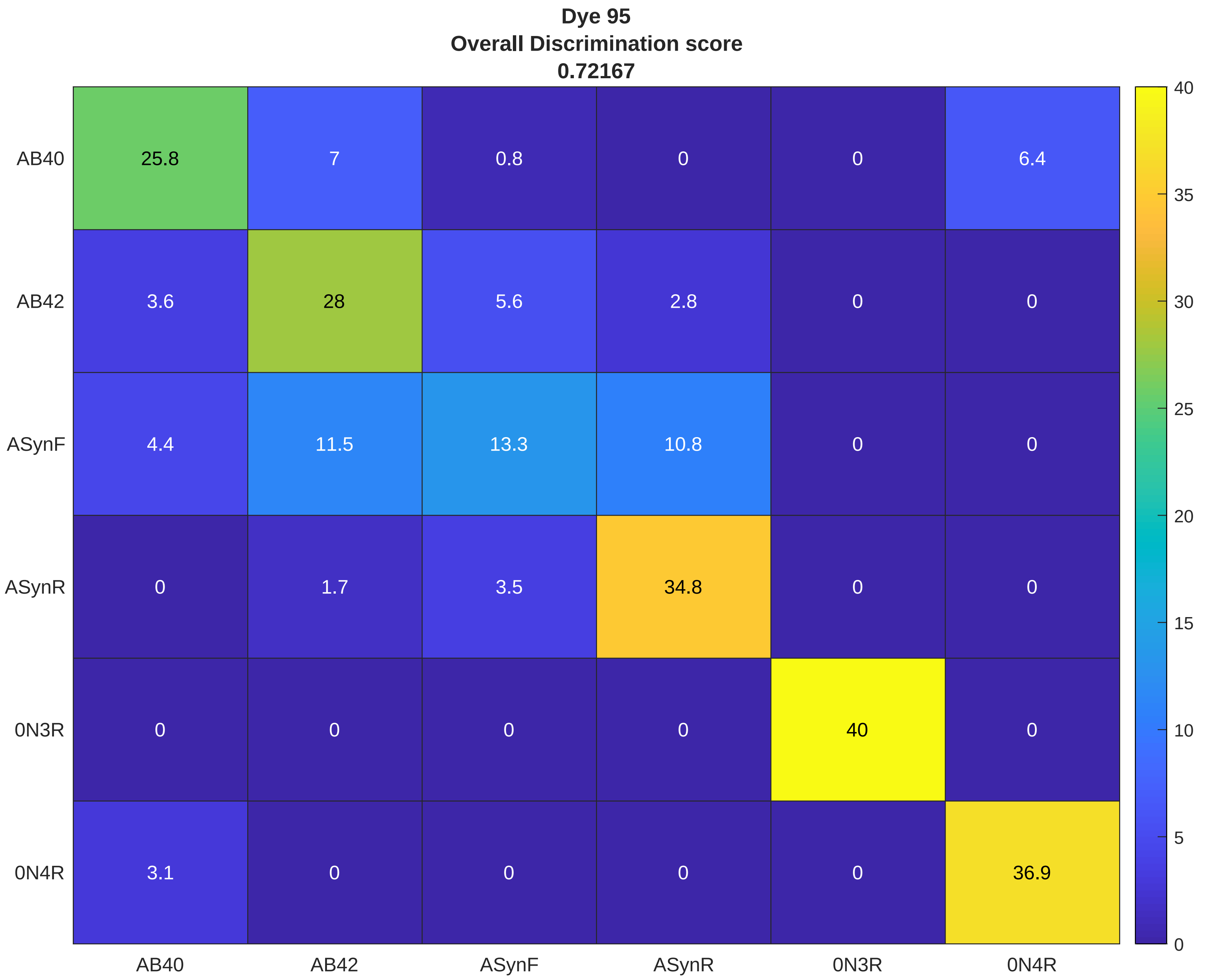
Dye 95
Overall Discrimination score
0.72167
AB40
AB42
ASynF
ASynR
0N3R
0N4R
25.8
7
0.8
0
0
6.4
3.6
28
5.6
2.8
0
0
4.4
11.5
13.3
10.8
0
0
0
1.7
3.5
34.8
0
0
0
0
0
0
40
0
3.1
0
0
0
0
36.9
AB40
AB42
ASynF
ASynR
0N3R
0N4R
40
35
30
25
20
15
10
5
0

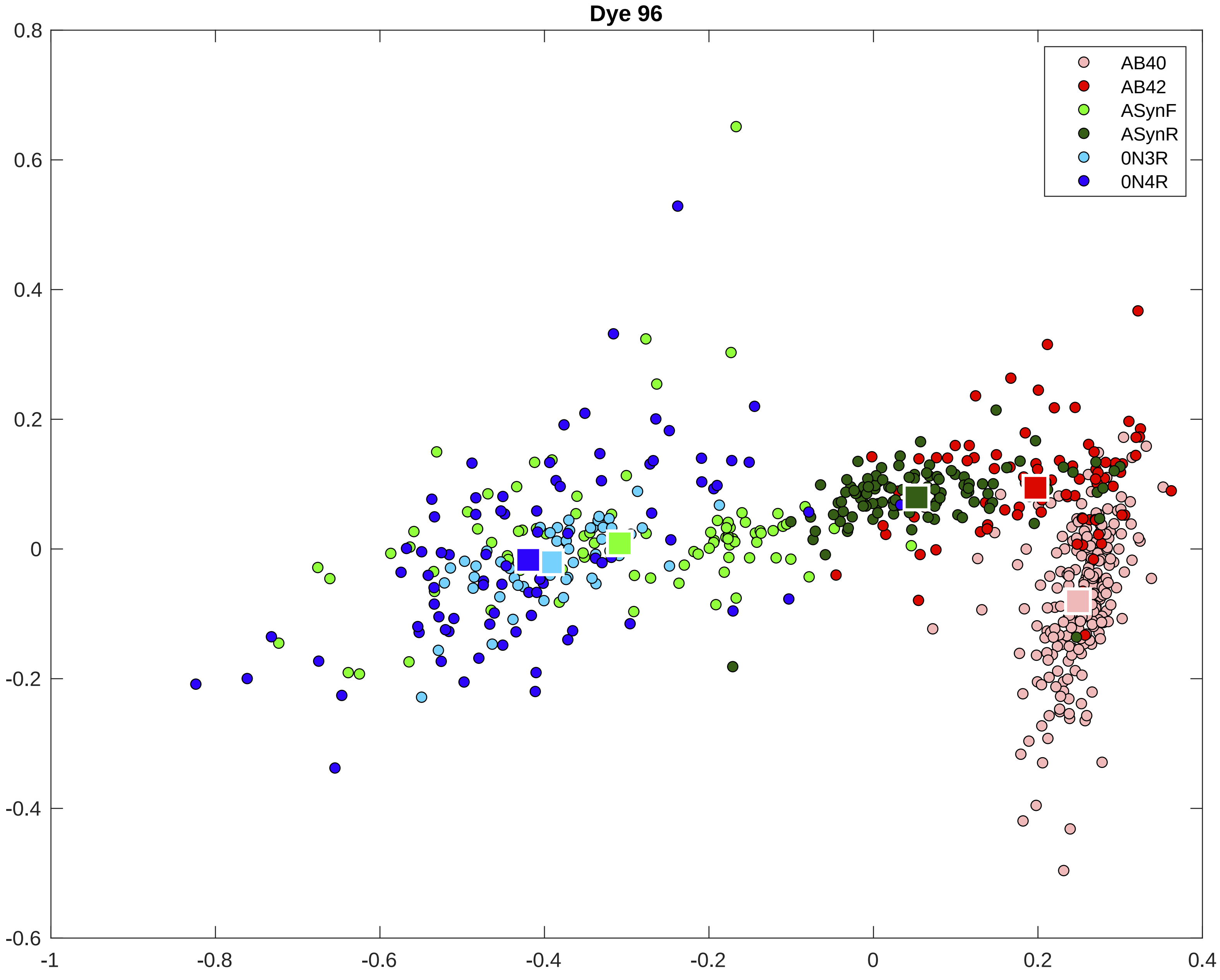

Dye 96
AB40
AB42
ASynF
ASynR
0N3R
0N4R
0.8
0.6
0.4
0.2
0
-0.2
-0.4
-0.6
-1
-0.8
-0.6
-0.4
-0.2
0
0.2
0.4

Dye 96
Overall Discrimination score
0.685

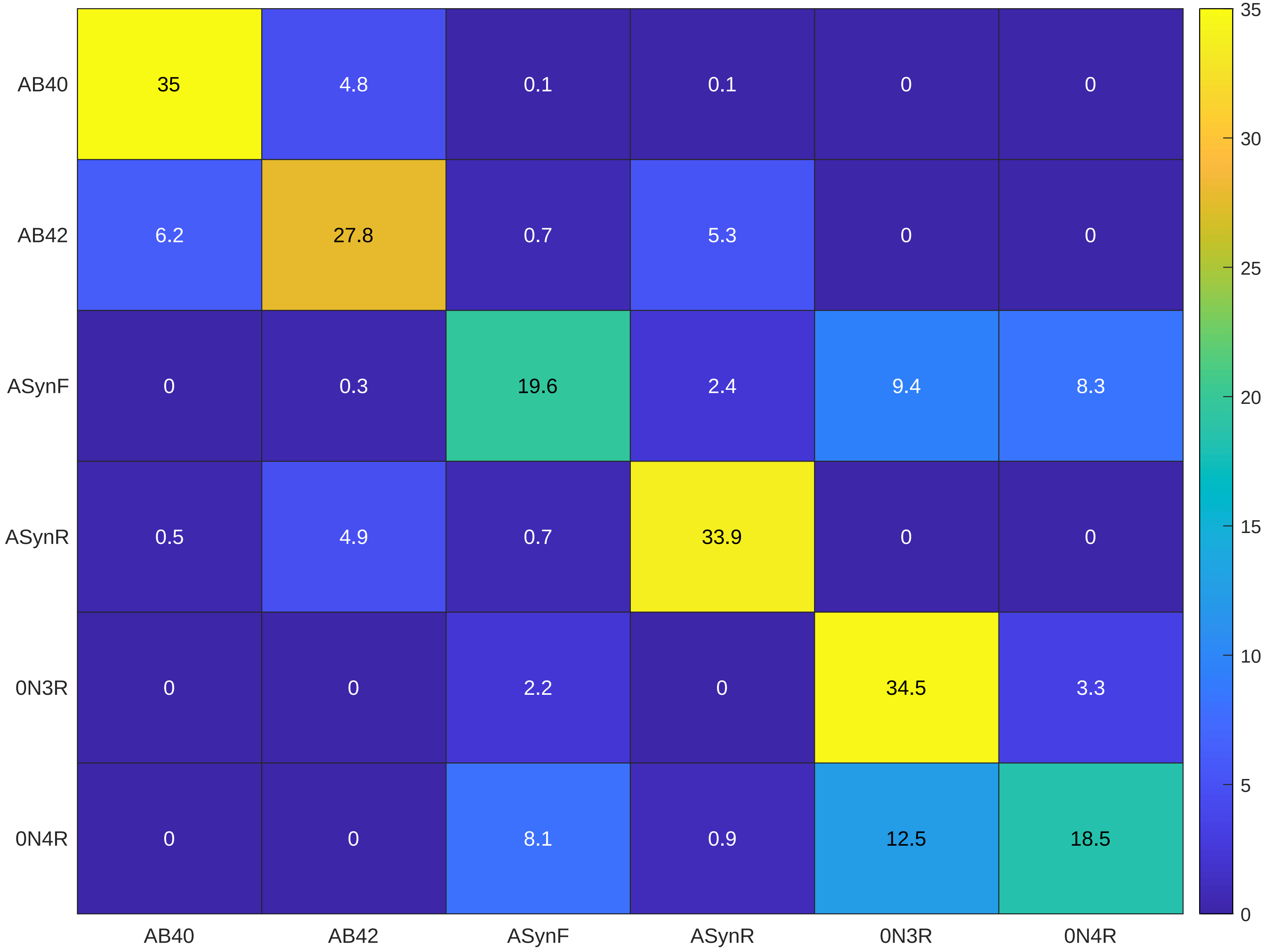

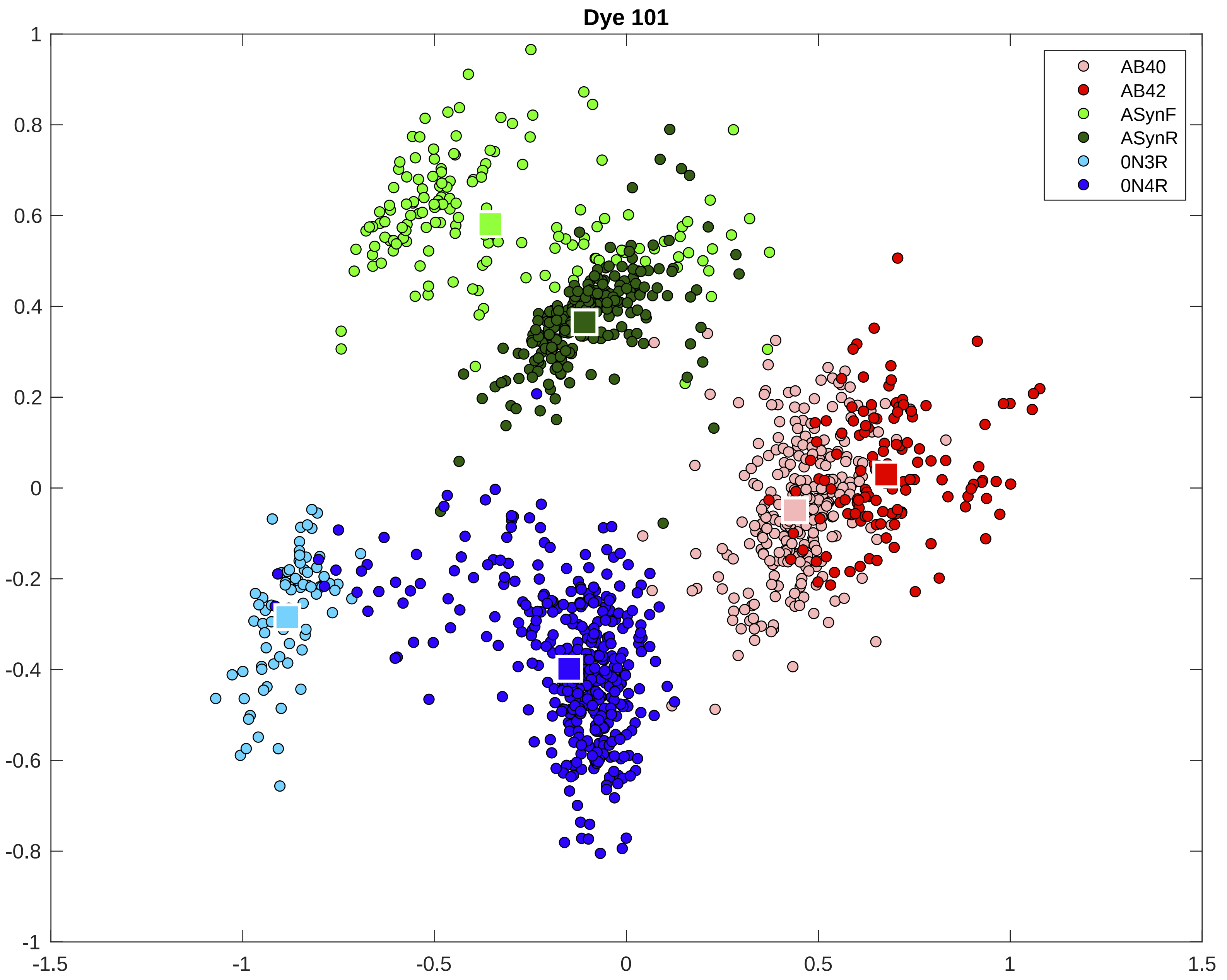

Dye 101
AB40
AB42
ASynF
ASynR
0N3R
0N4R
1
0.8
0.6
0.4
0.2
0
-0.2
-0.4
-0.6
-0.8
-1
-1.5
-1
-0.5
0
0.5
1
1.5

Dye 101
Overall Discrimination score
0.89625

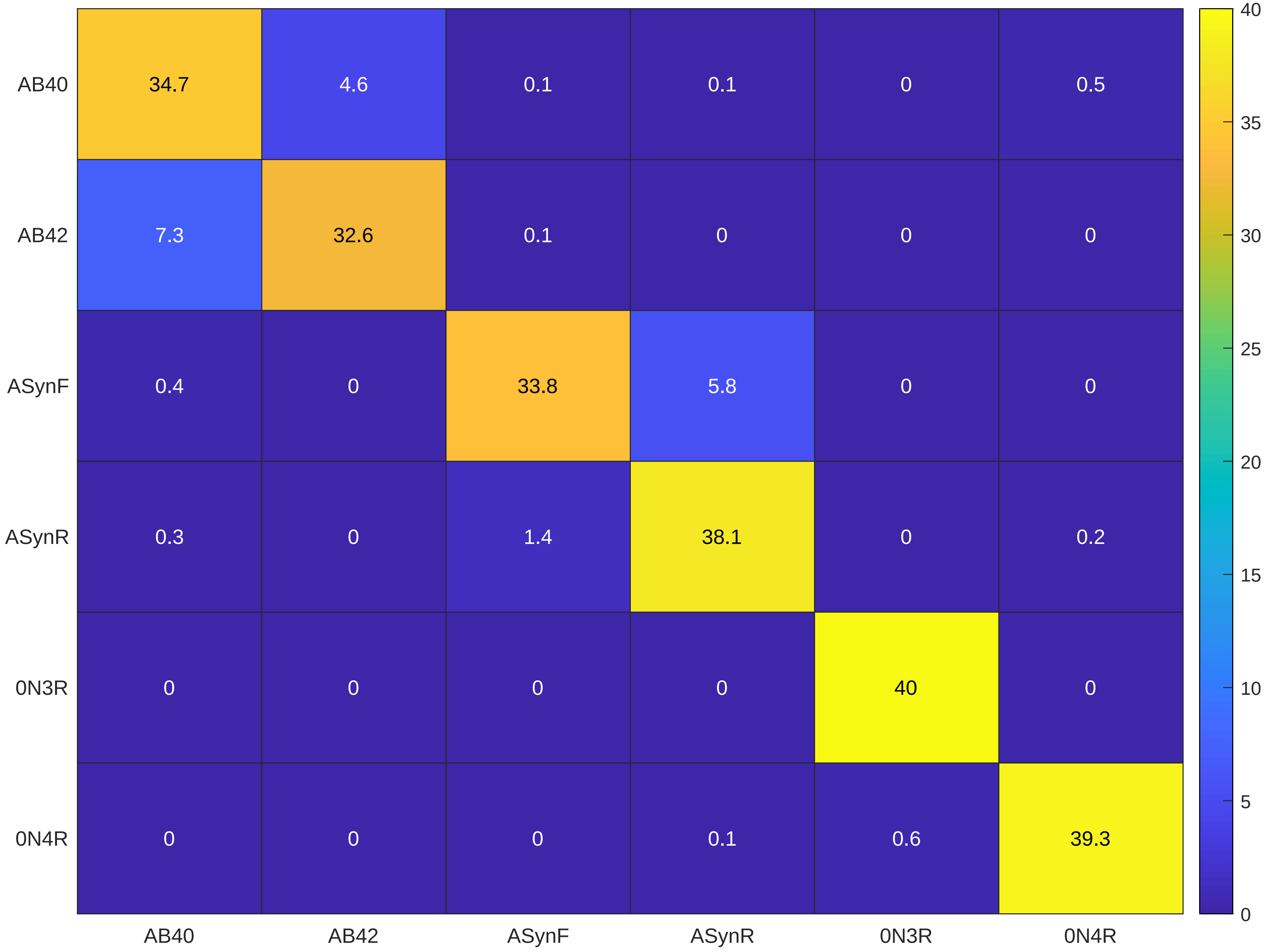

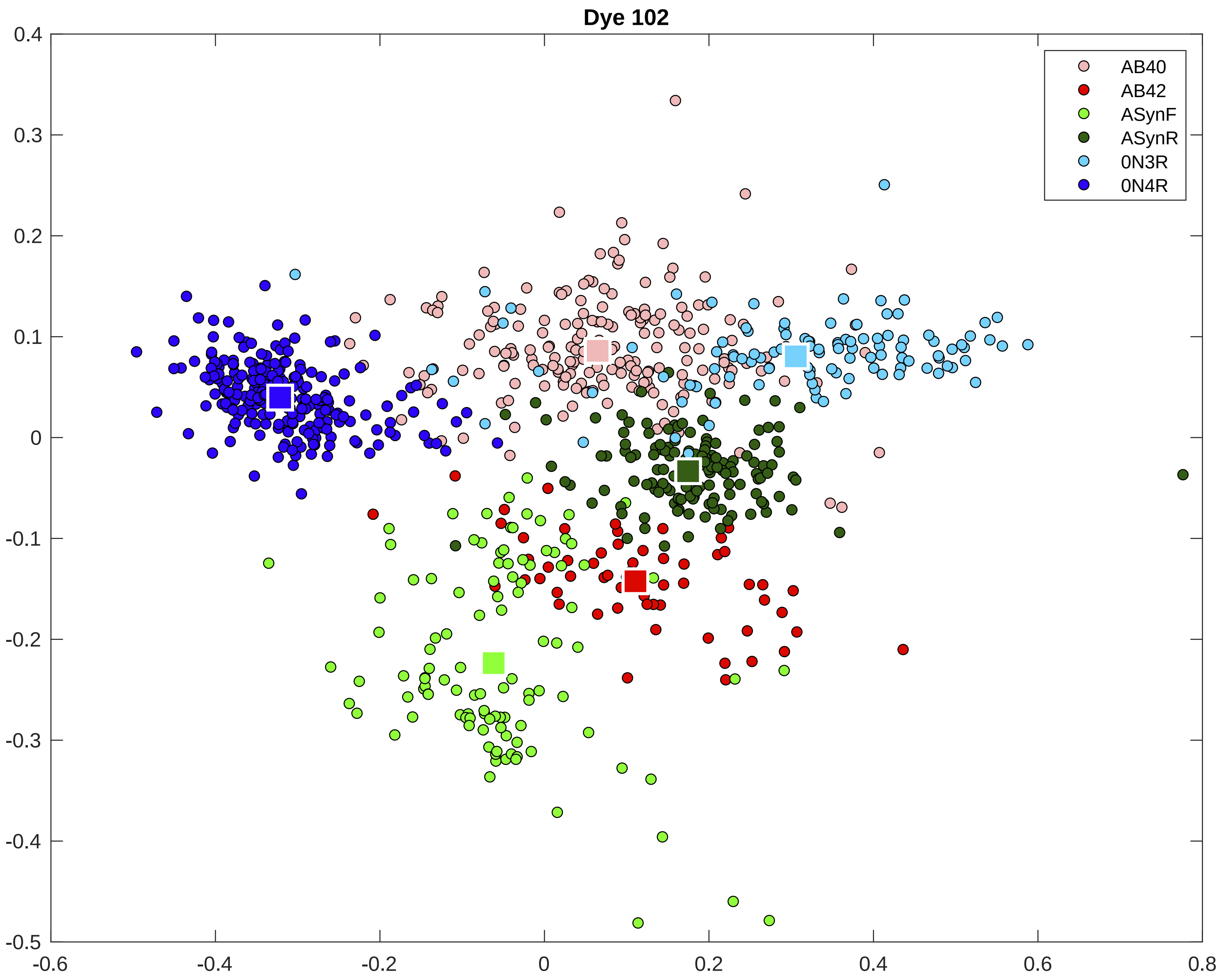
Dye 102
AB40
AB42
ASynF
ASynR
0N3R
0N4R
0.4
0.3
0.2
0.1
0
-0.1
-0.2
-0.3
-0.4
-0.5
-0.6
-0.4
-0.2
0
0.2
0.4
0.6
0.8

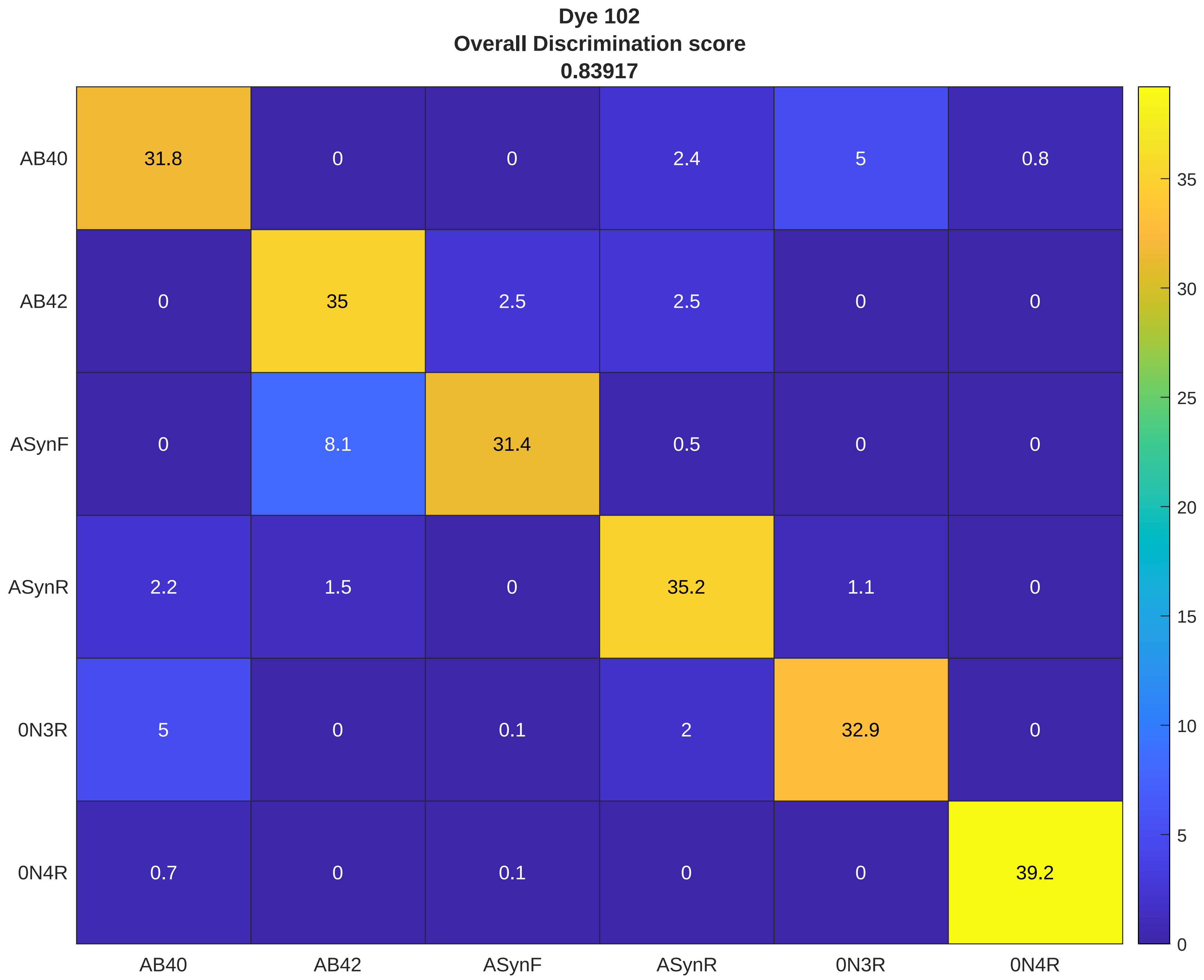
Dye 102
Overall Discrimination score
0.83917
AB40
AB42
ASynF
ASynR
0N3R
0N4R
31.8 0 0 2.4 5 0.8
0 35 2.5 2.5 0 0
0 8.1 31.4 0.5 0 0
2.2 1.5 0 35.2 1.1 0
5 0 0.1 2 32.9 0
0.7 0 0.1 0 0 39.2
AB40
AB42
ASynF
ASynR
0N3R
0N4R
35
30
25
20
15
10
5
0

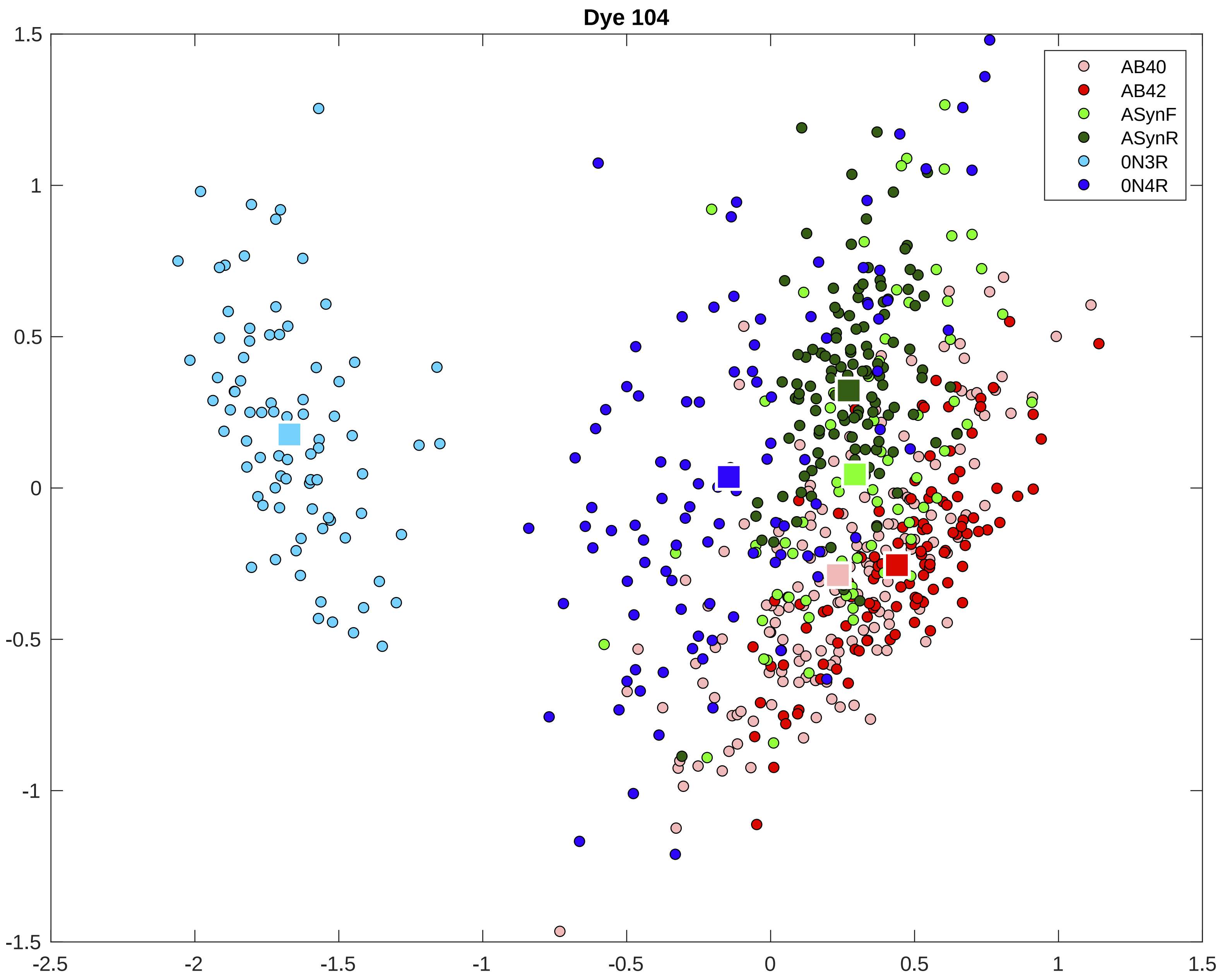

Dye 104
AB40
AB42
ASynF
ASynR
0N3R
0N4R
1.5
1
0.5
0
-0.5
-1
-1.5
-2.5
-2
-1.5
-1
-0.5
0
0.5
1
1.5

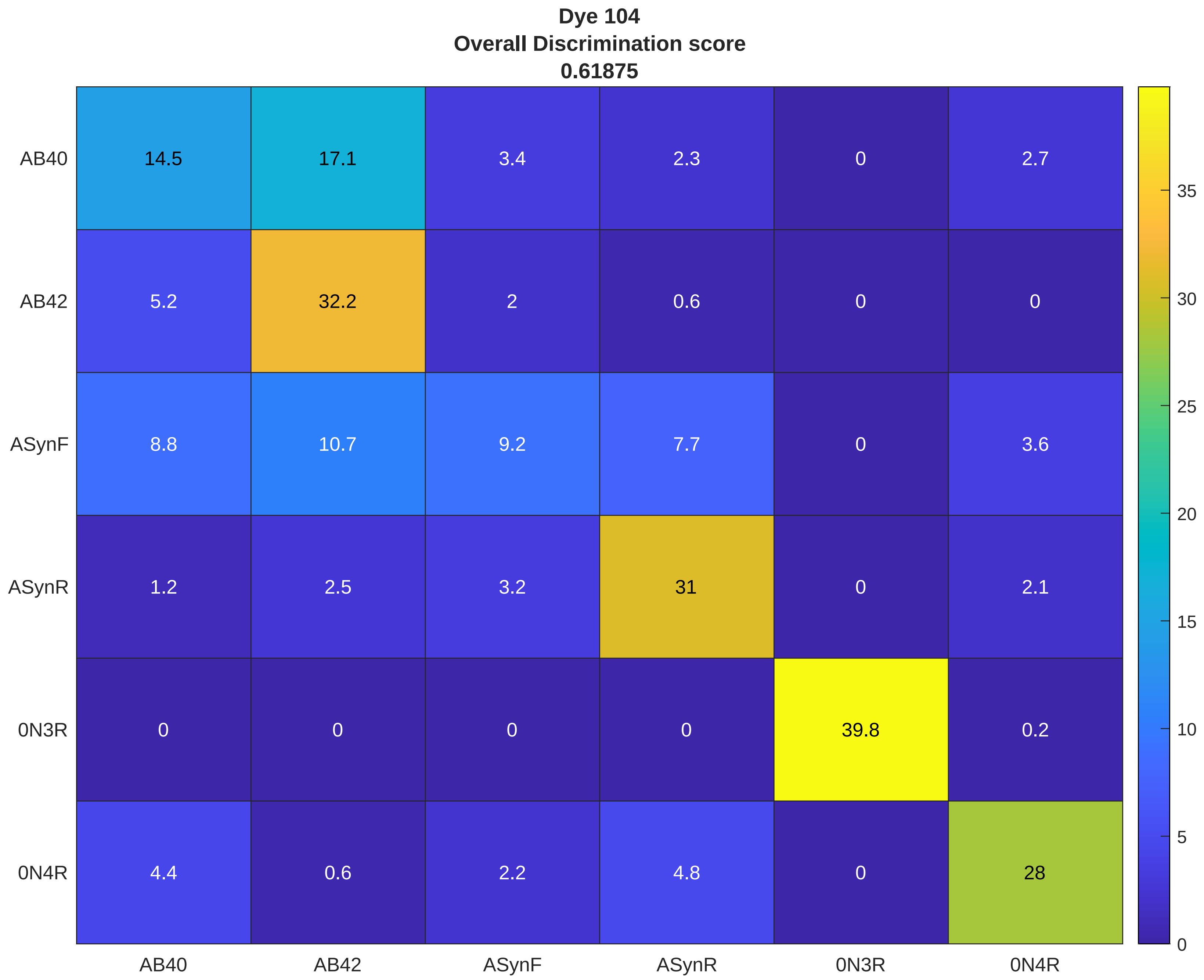
Dye 104
Overall Discrimination score
0.61875
AB40
AB42
ASynF
ASynR
0N3R
0N4R
14.5
17.1
3.4
2.3
0
2.7
5.2
32.2
2
0.6
0
0
8.8
10.7
9.2
7.7
0
3.6
1.2
2.5
3.2
31
0
2.1
0
0
0
0
39.8
0.2
4.4
0.6
2.2
4.8
0
28
AB40
AB42
ASynF
ASynR
0N3R
0N4R
35
30
25
20
15
10
5
0

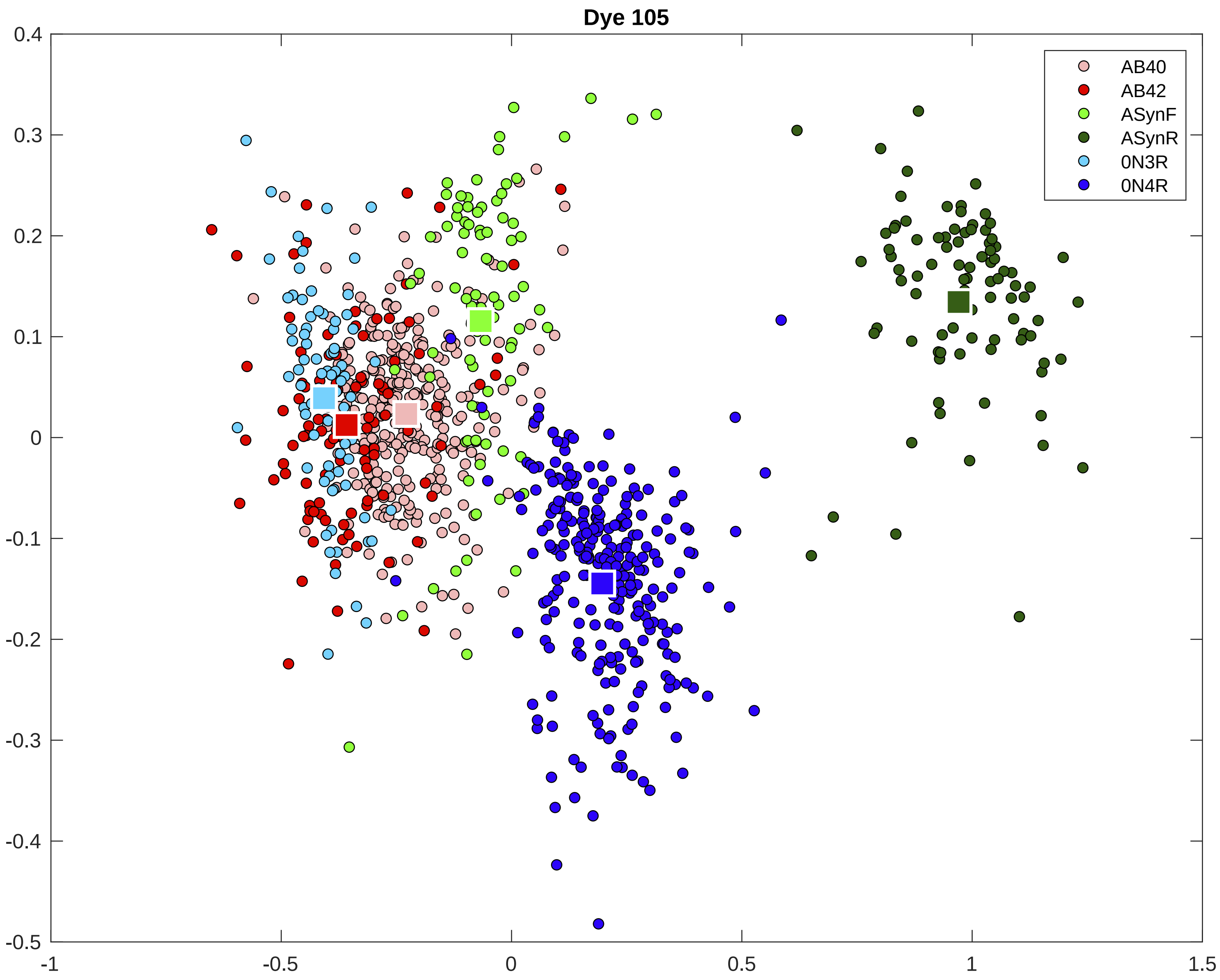

Dye 105
0.4
0.3
0.2
0.1
0
-0.1
-0.2
-0.3
-0.4
-0.5
-1
-0.5
0
0.5
1
1.5
AB40
AB42
ASynF
ASynR
0N3R
0N4R

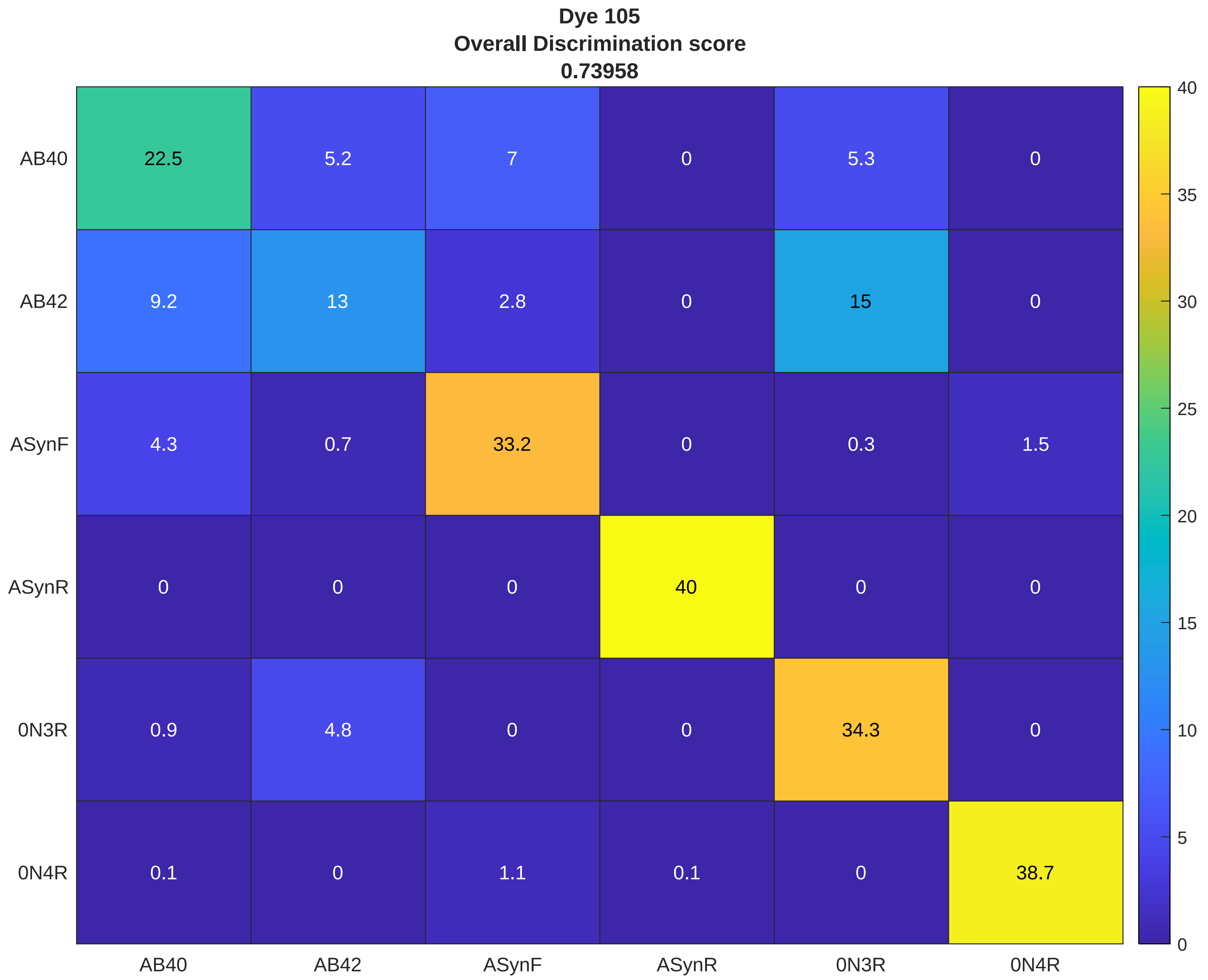

Dye 105
Overall Discrimination score
0.73958
AB40
AB42
ASynF
ASynR
0N3R
0N4R
22.5
5.2
7
0
5.3
0
9.2
13
2.8
0
15
0
4.3
0.7
33.2
0
0.3
1.5
0
0
0
40
0
0
0.9
4.8
0
0
34.3
0
0.1
0
1.1
0.1
0
38.7
AB40
AB42
ASynF
ASynR
0N3R
0N4R
40
35
30
25
20
15
10
5
0

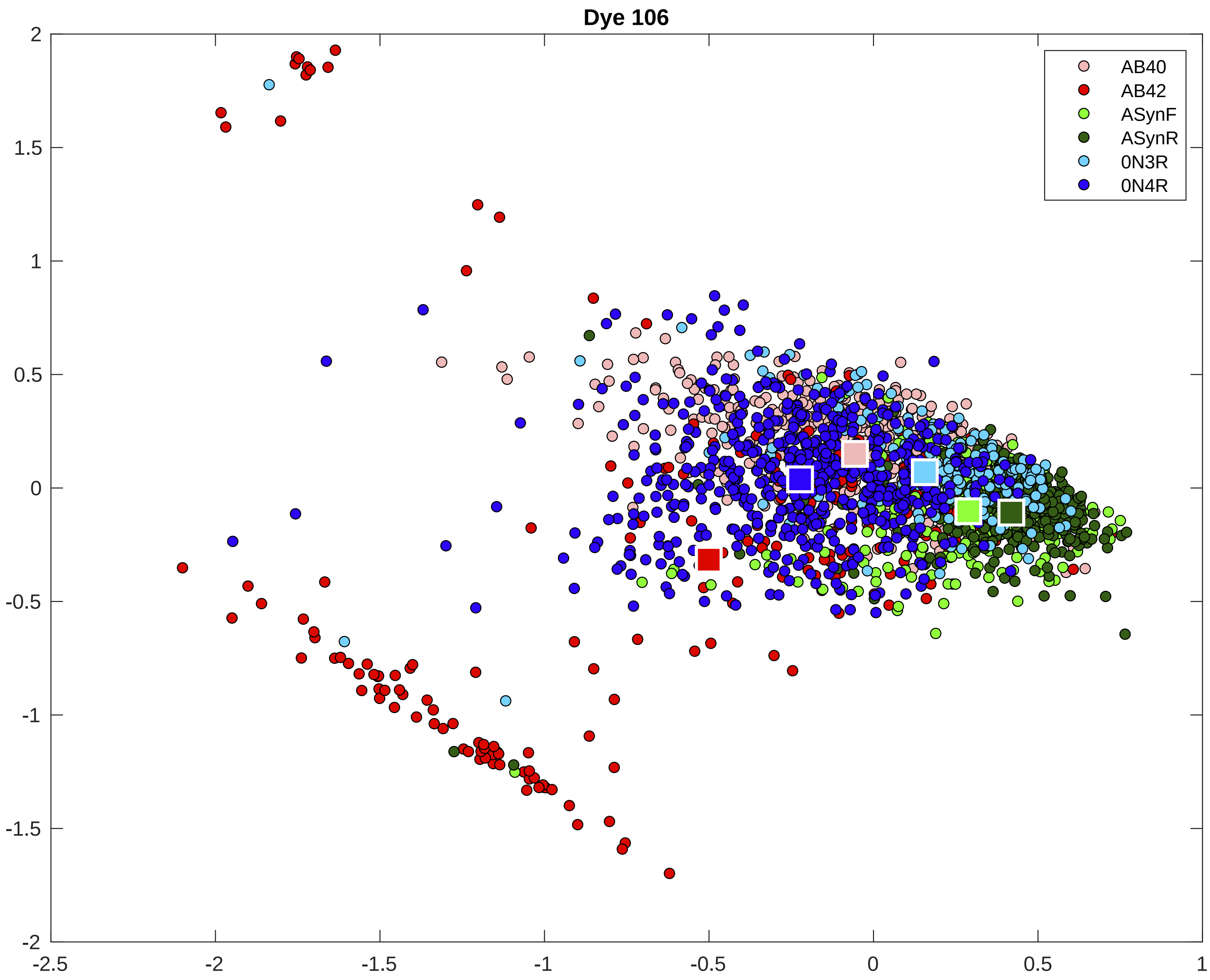

Dye 106
AB40
AB42
ASynF
ASynR
0N3R
0N4R
2
1.5
1
0.5
0
-0.5
-1
-1.5
-2
-2.5
-2
-1.5
-1
-0.5
0
0.5
1

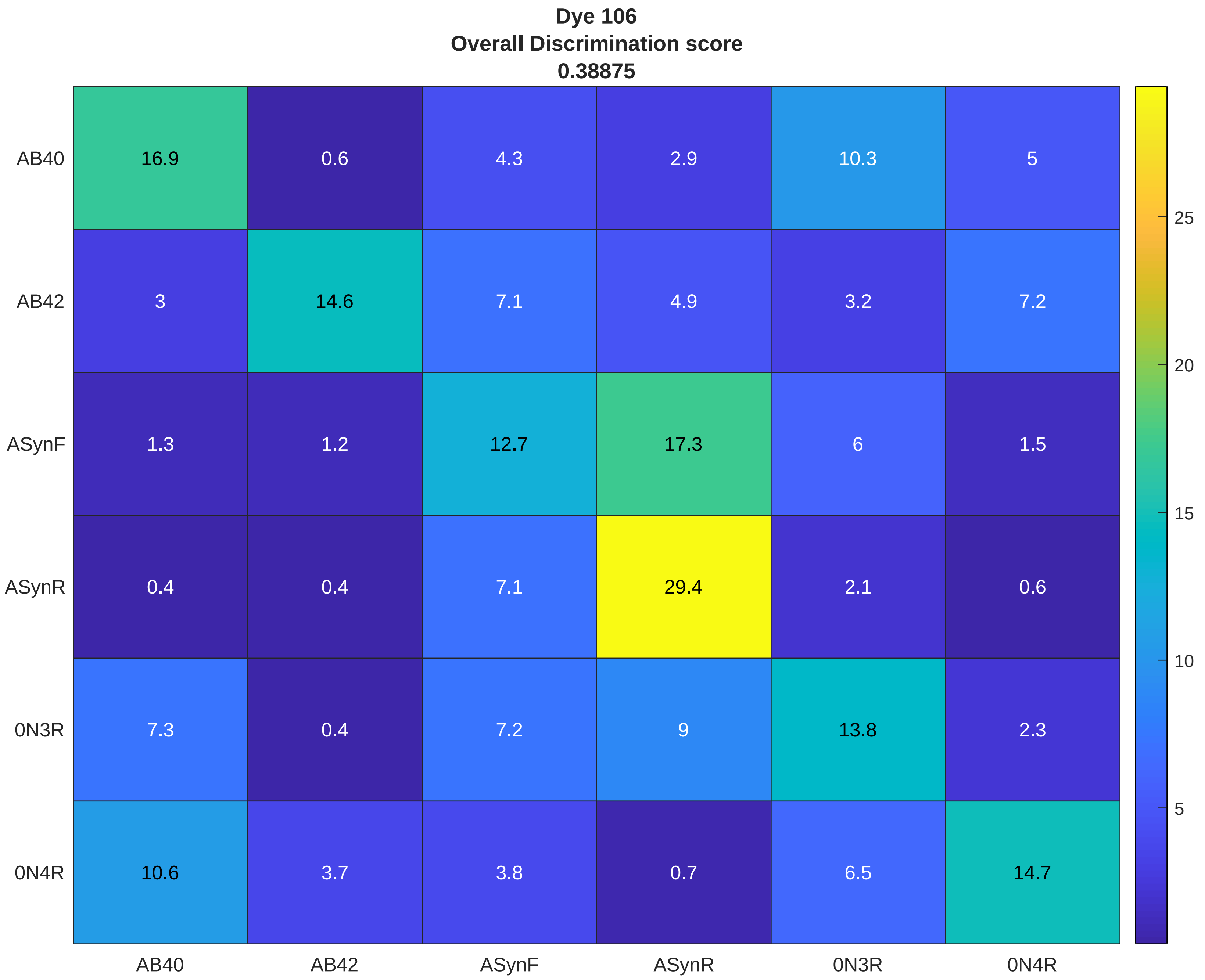

Dye 106
Overall Discrimination score
0.38875
AB40
AB42
ASynF
ASynR
0N3R
0N4R
16.9
0.6
4.3
2.9
10.3
5
3
14.6
7.1
4.9
3.2
7.2
1.3
1.2
12.7
17.3
6
1.5
0.4
0.4
7.1
29.4
2.1
0.6
7.3
0.4
7.2
9
13.8
2.3
10.6
3.7
3.8
0.7
6.5
14.7
AB40
AB42
ASynF
ASynR
0N3R
0N4R
25
20
15
10
5

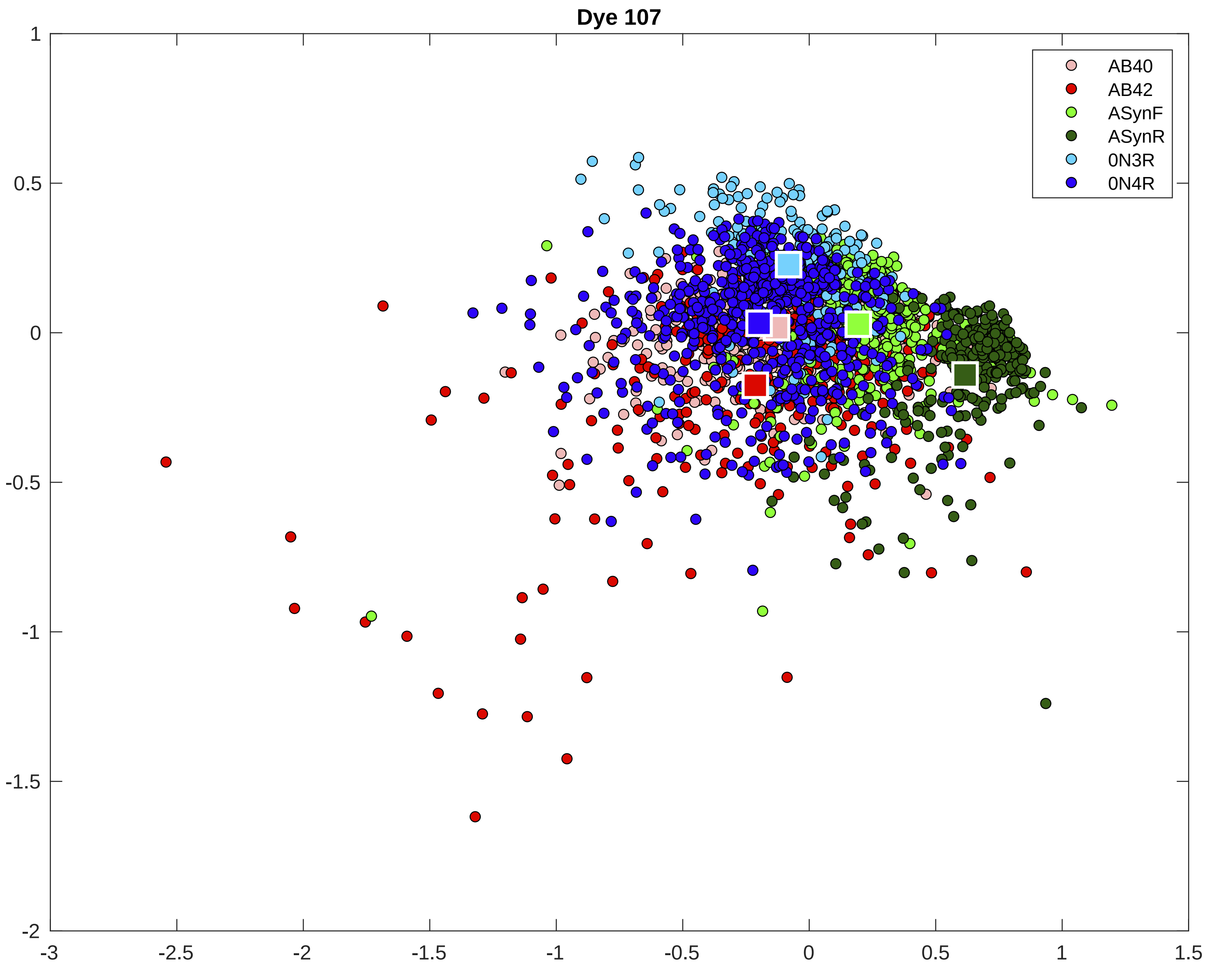

Dye 107
AB40
AB42
ASynF
ASynR
0N3R
0N4R
1
0.5
0
-0.5
-1
-1.5
-2
-3
-2.5
-2
-1.5
-1
-0.5
0
0.5
1
1.5

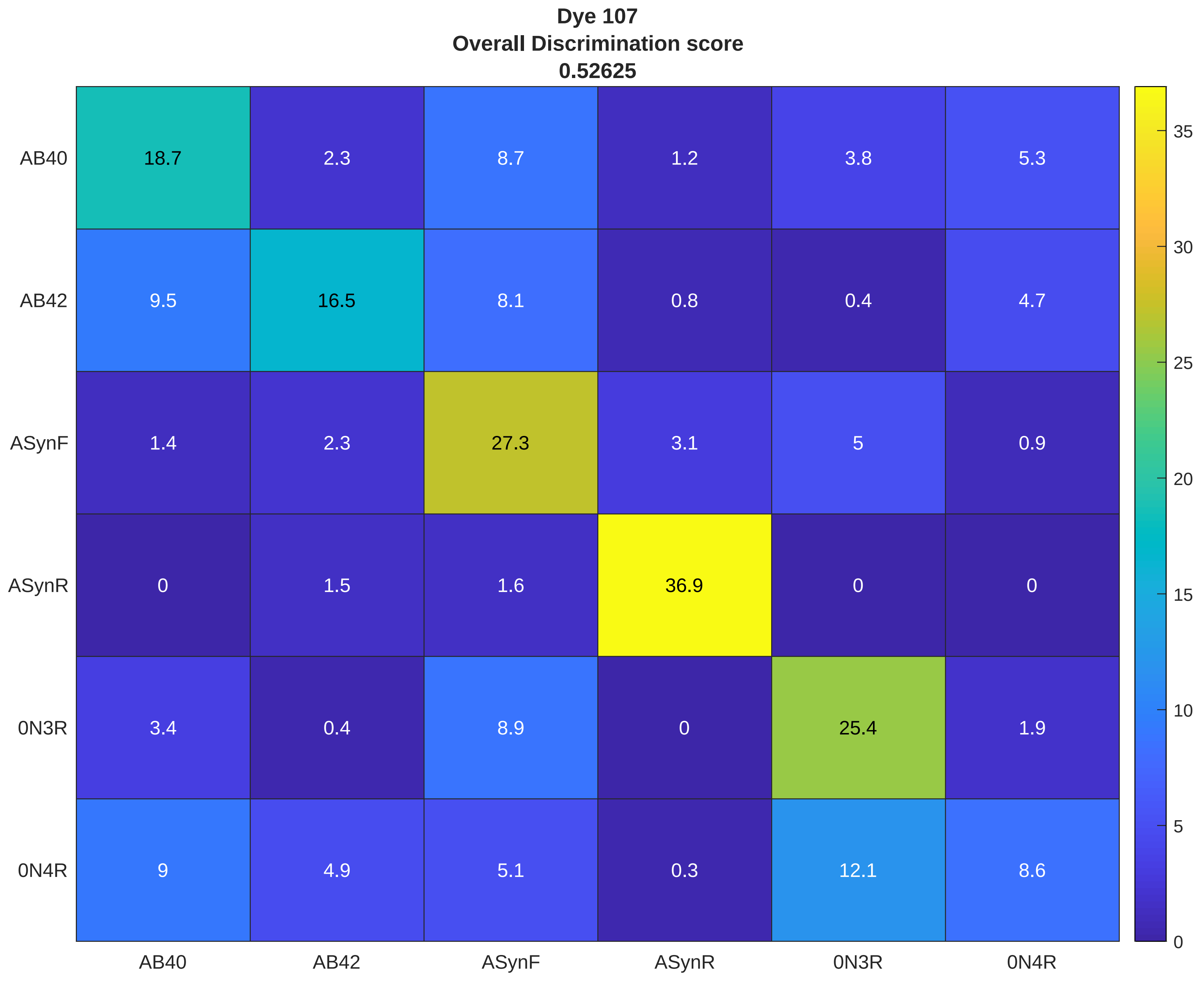
Dye 107
Overall Discrimination score
0.52625
AB40 18.7 2.3 8.7 1.2 3.8 5.3
AB42 9.5 16.5 8.1 0.8 0.4 4.7
ASynF 1.4 2.3 27.3 3.1 5 0.9
ASynR 0 1.5 1.6 36.9 0 0
0N3R 3.4 0.4 8.9 0 25.4 1.9
0N4R 9 4.9 5.1 0.3 12.1 8.6
AB40
AB42
ASynF
ASynR
0N3R
0N4R
35
30
25
20
15
10
5
0

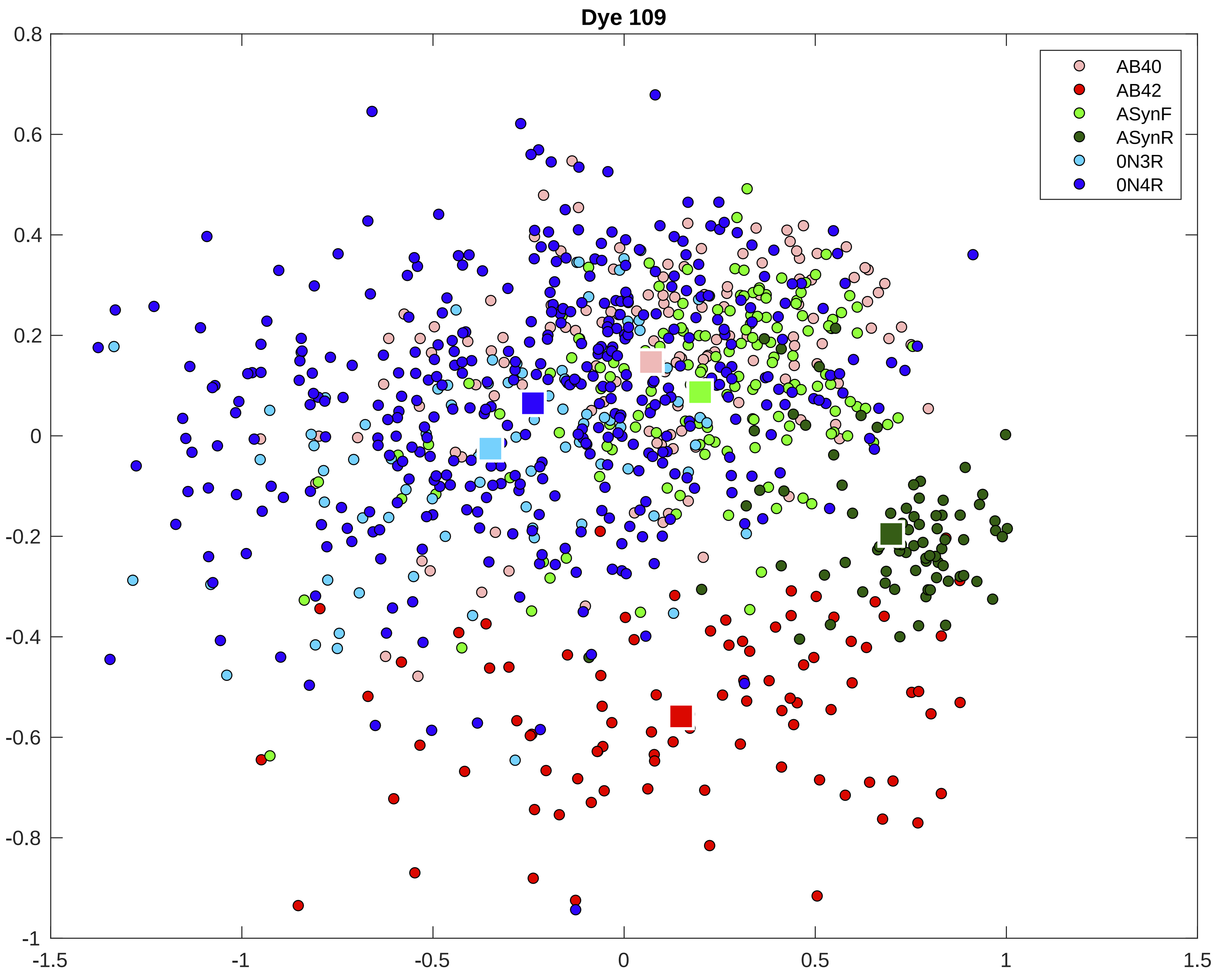
Dye 109
AB40
AB42
ASynF
ASynR
0N3R
0N4R
0.8
0.6
0.4
0.2
0
-0.2
-0.4
-0.6
-0.8
-1
-1.5
-1
-0.5
0
0.5
1
1.5

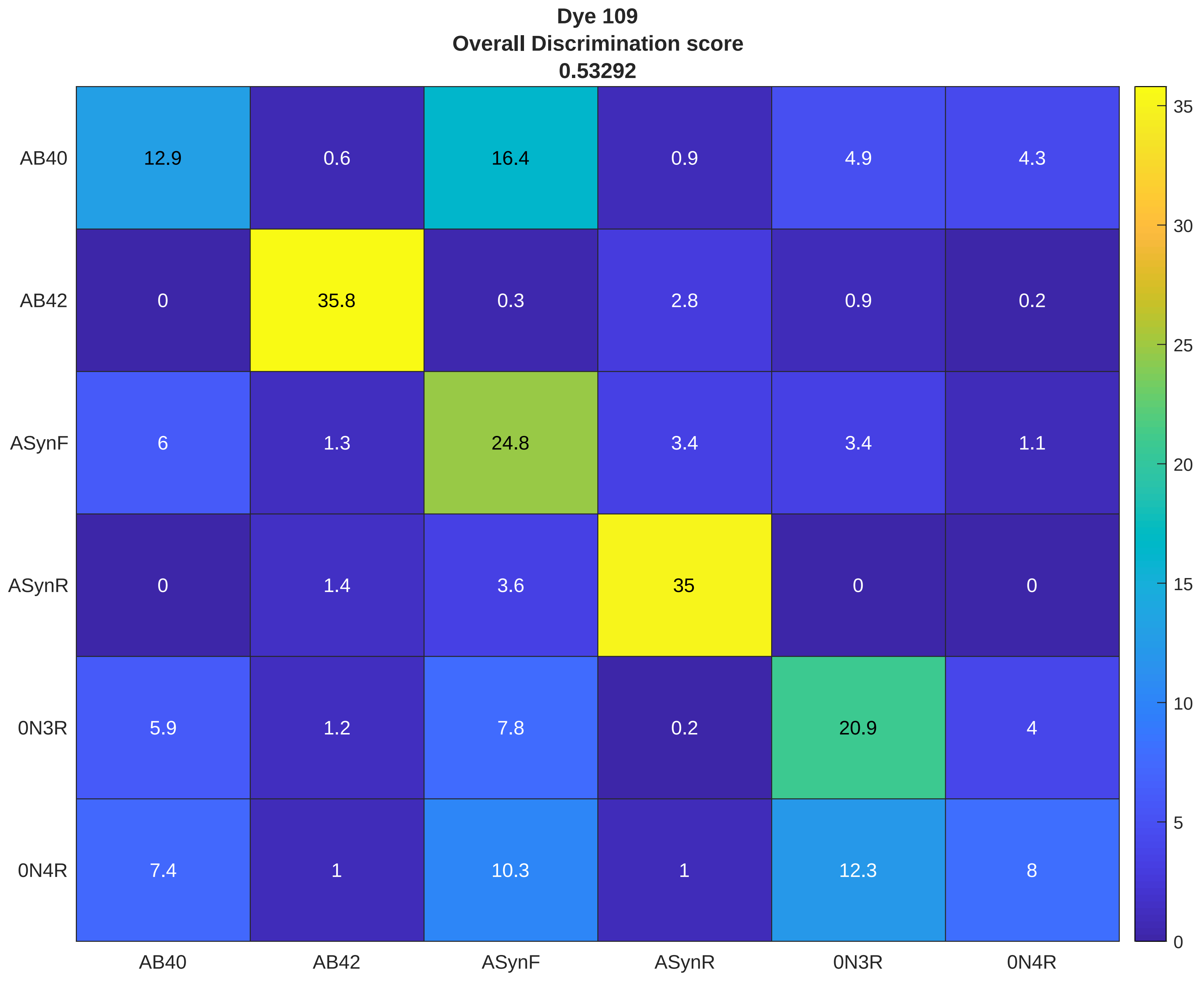
Dye 109
Overall Discrimination score
0.53292
AB40
AB42
ASynF
ASynR
0N3R
0N4R
12.9 0.6 16.4 0.9 4.9 4.3
0 35.8 0.3 2.8 0.9 0.2
6 1.3 24.8 3.4 3.4 1.1
0 1.4 3.6 35 0 0
5.9 1.2 7.8 0.2 20.9 4
7.4 1 10.3 1 12.3 8
AB40
AB42
ASynF
ASynR
0N3R
0N4R
35
30
25
20
15
10
5
0

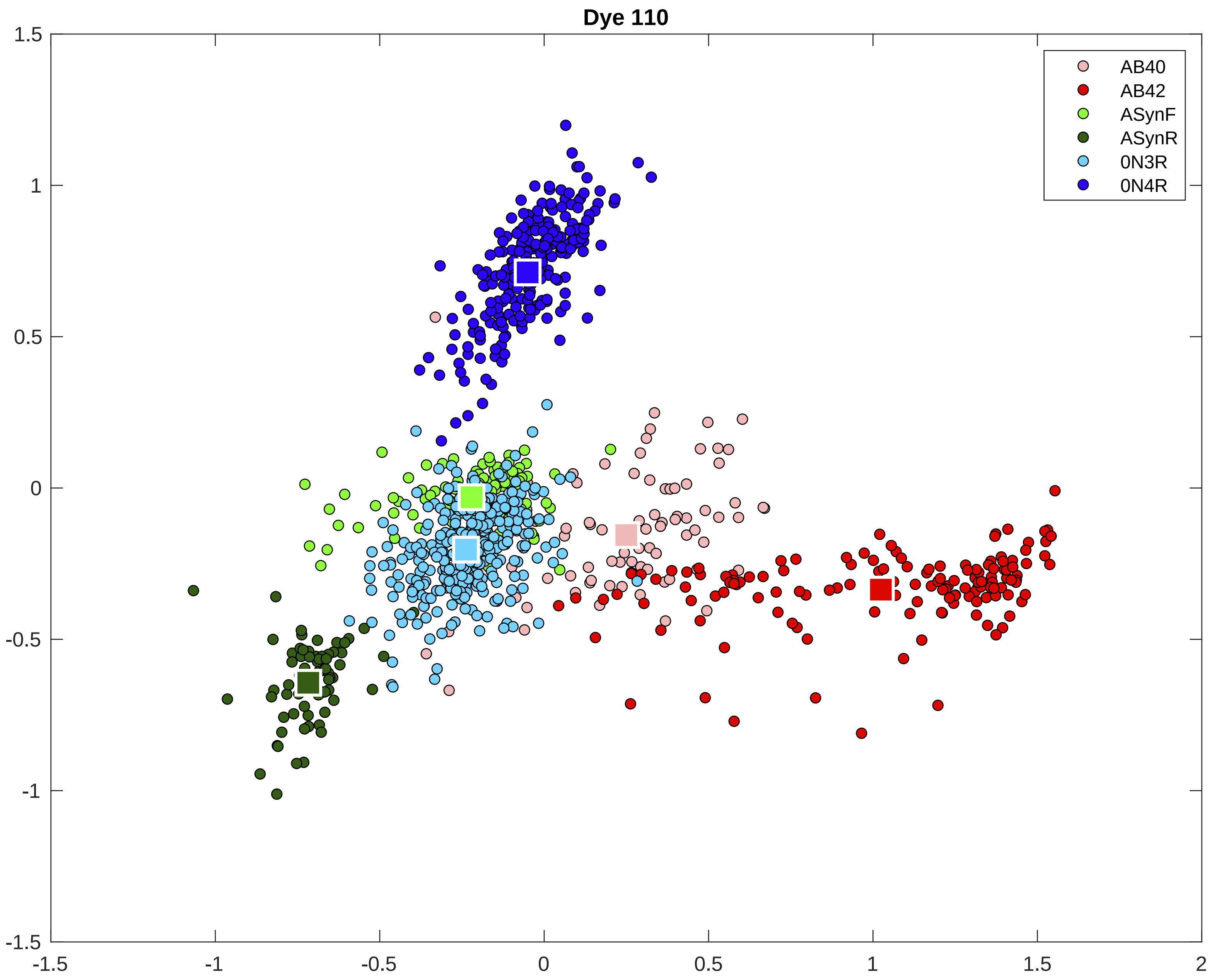
Dye 110
AB40
AB42
ASynF
ASynR
0N3R
0N4R
1.5
1
0.5
0
-0.5
-1
-1.5
-1.5
-1
-0.5
0
0.5
1
1.5
2

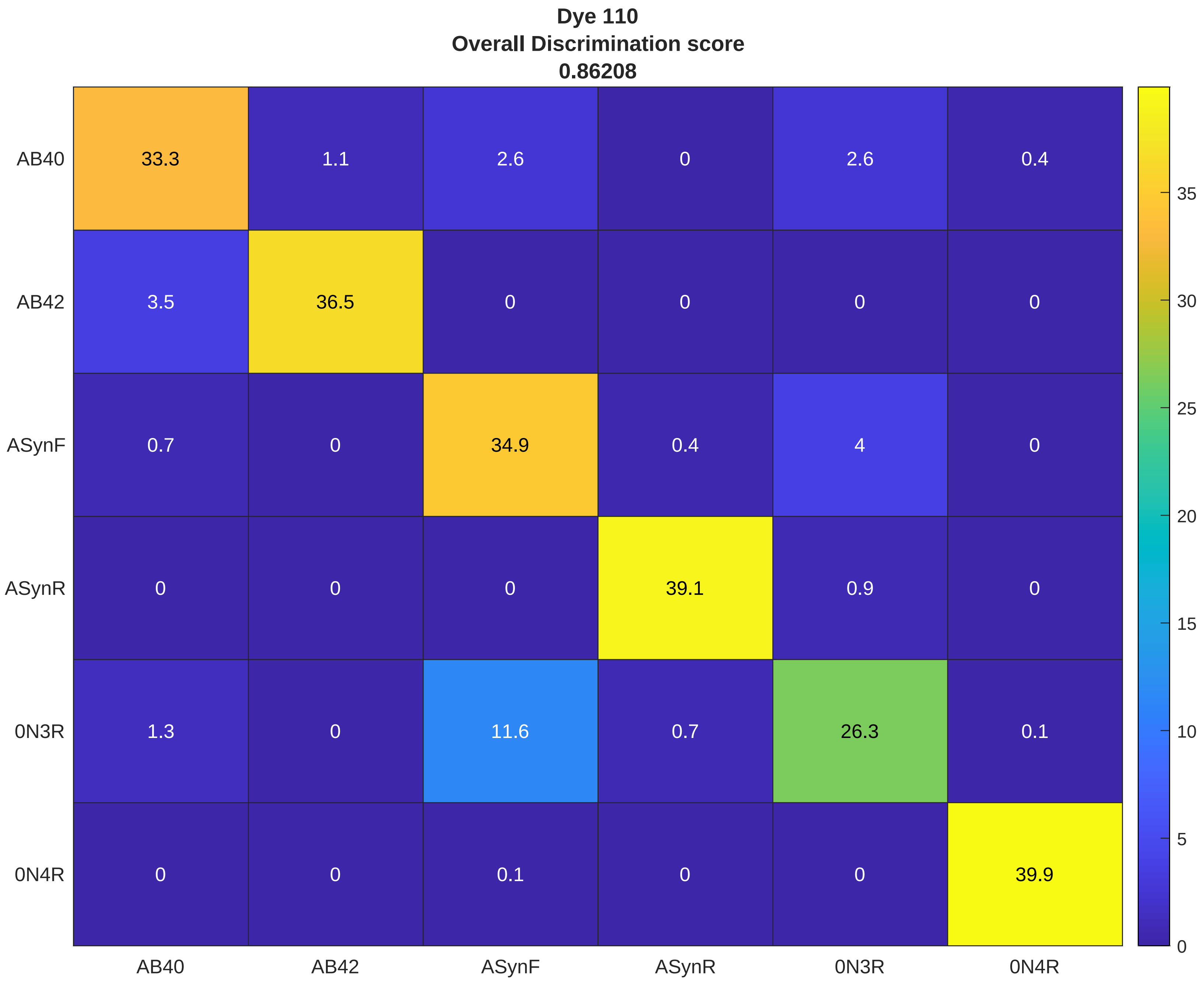
Dye 110
Overall Discrimination score
0.86208
AB40
AB42
ASynF
ASynR
0N3R
0N4R
33.3
1.1
2.6
0
2.6
0.4
3.5
36.5
0
0
0
0
0.7
0
34.9
0.4
4
0
0
0
0
39.1
0.9
0
1.3
0
11.6
0.7
26.3
0.1
0
0
0.1
0
0
39.9
AB40
AB42
ASynF
ASynR
0N3R
0N4R
35
30
25
20
15
10
5
0

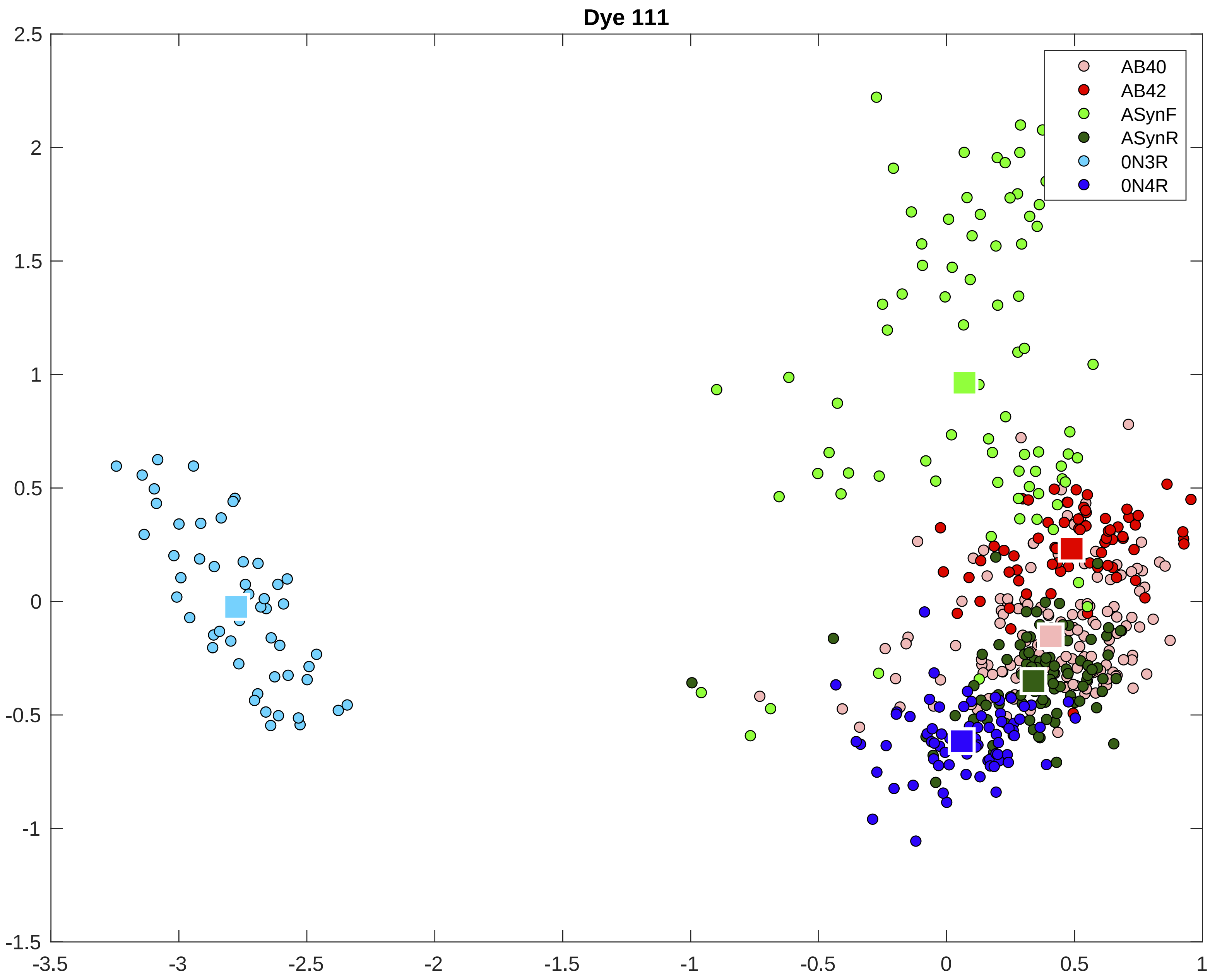

Dye 111
AB40
AB42
ASynF
ASynR
0N3R
0N4R
2.5
2
1.5
1
0.5
0
-0.5
-1
-1.5
-3.5
-3
-2.5
-2
-1.5
-1
-0.5
0
0.5
1

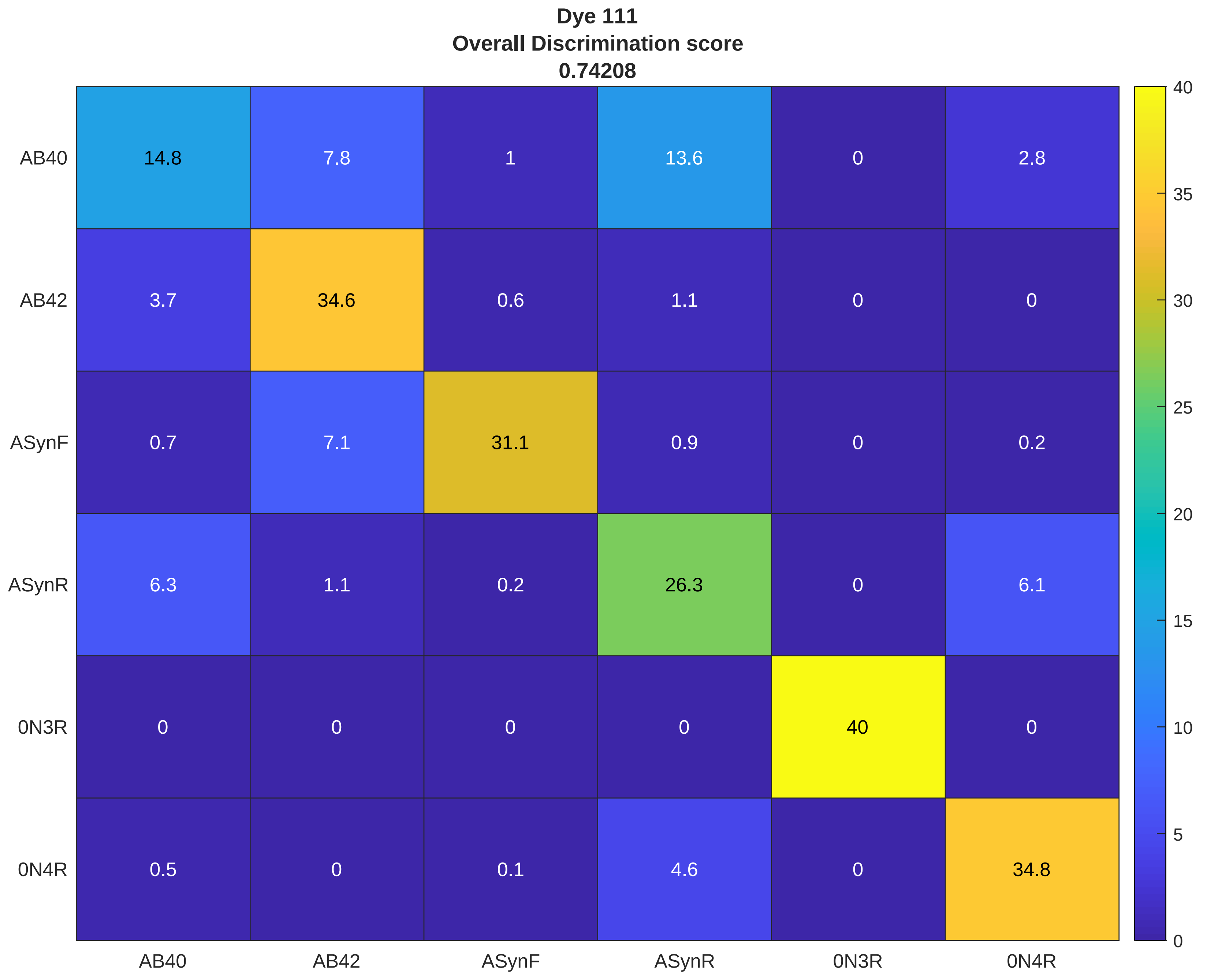
Dye 111
Overall Discrimination score
0.74208
AB40
AB42
ASynF
ASynR
0N3R
0N4R
14.8
7.8
1
13.6
0
2.8
3.7
34.6
0.6
1.1
0
0
0.7
7.1
31.1
0.9
0
0.2
6.3
1.1
0.2
26.3
0
6.1
0
0
0
0
40
0
0.5
0
0.1
4.6
0
34.8
AB40
AB42
ASynF
ASynR
0N3R
0N4R
40
35
30
25
20
15
10
5
0

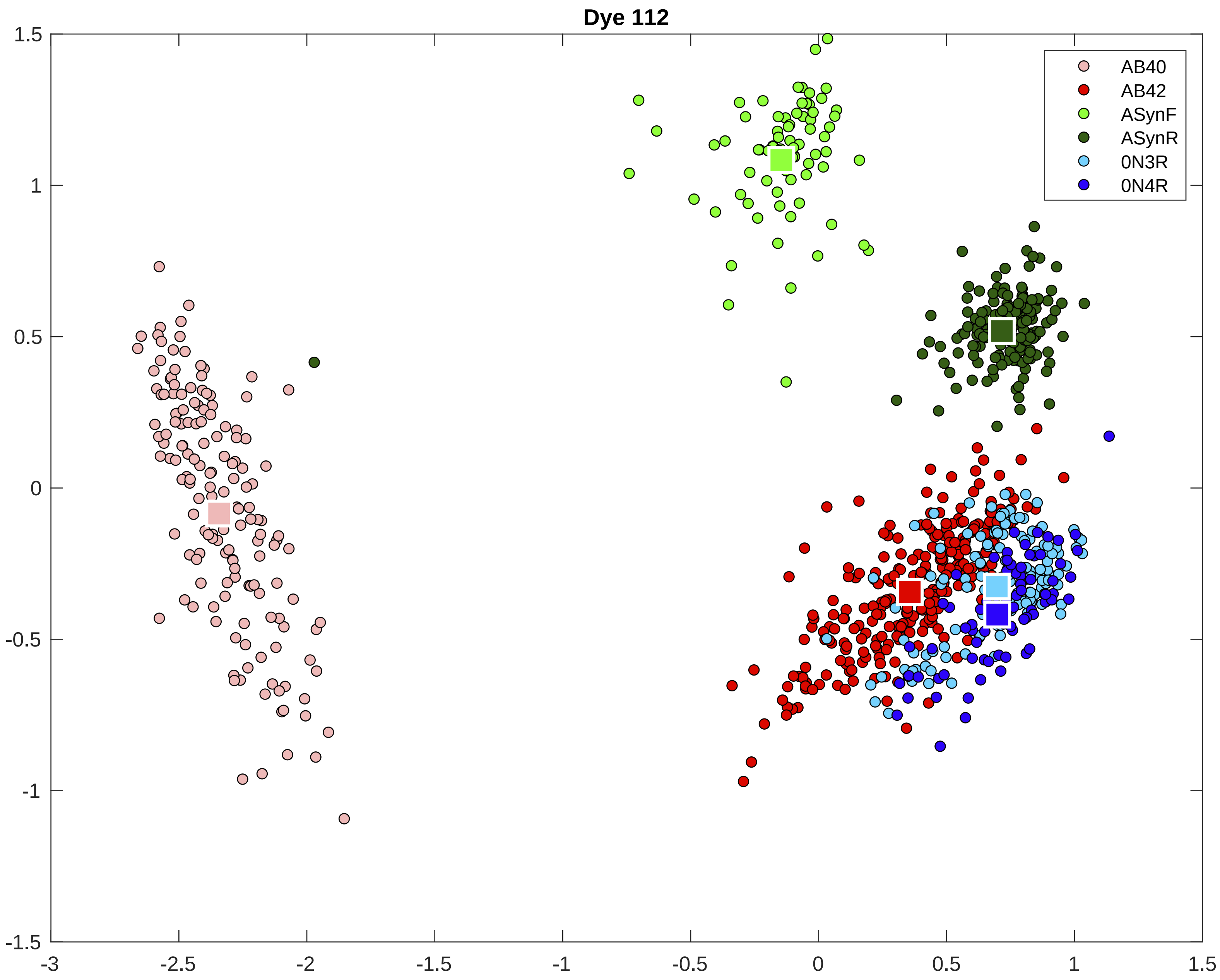
Dye 112
AB40
AB42
ASynF
ASynR
0N3R
0N4R
1.5
1
0.5
0
-0.5
-1
-1.5
-3
-2.5
-2
-1.5
-1
-0.5
0
0.5
1
1.5

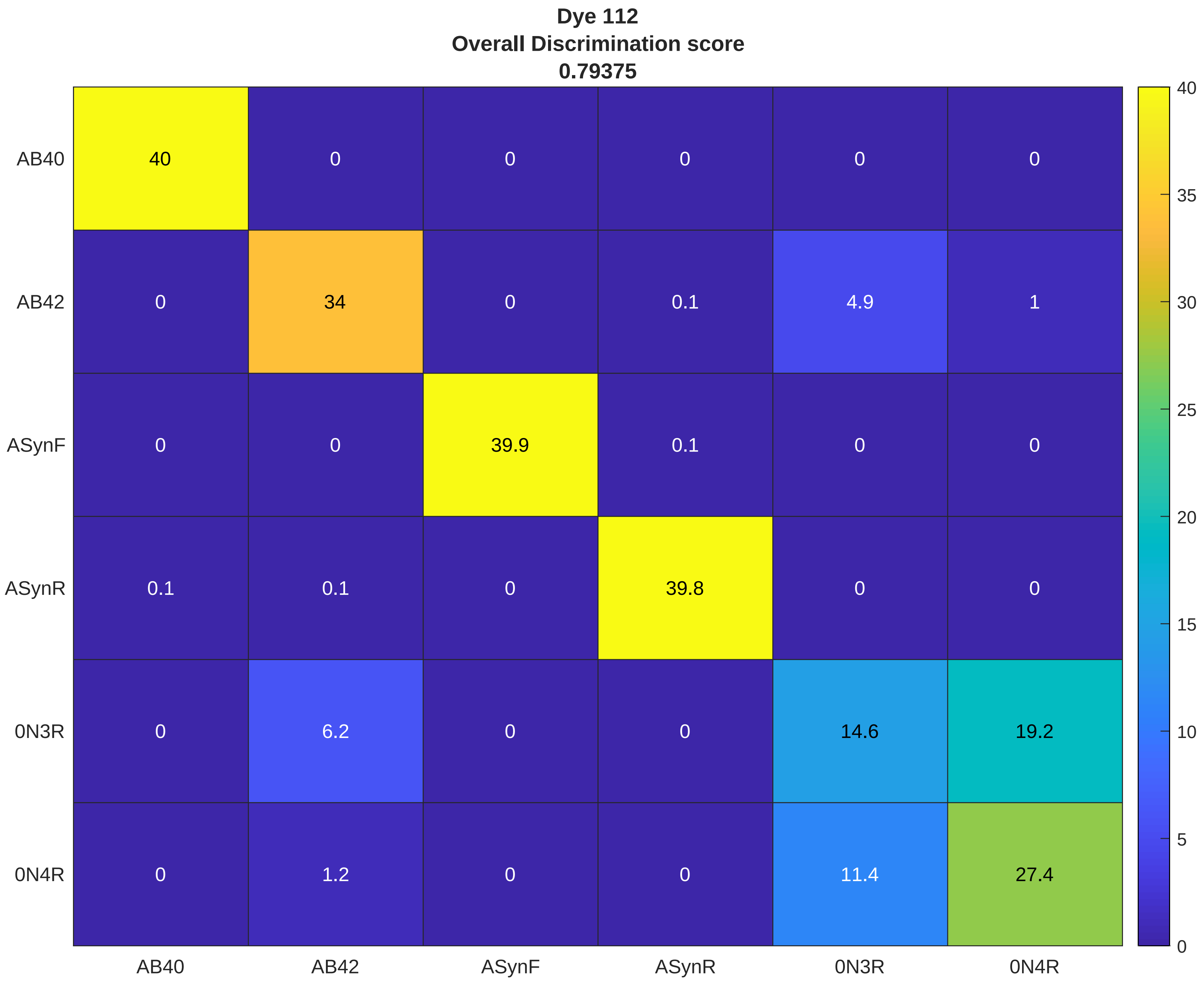
Dye 112
Overall Discrimination score
0.79375
AB40
AB42
ASynF
ASynR
0N3R
0N4R
40 0 0 0 0 0
0 34 0 0.1 4.9 1
0 0 39.9 0.1 0 0
0.1 0.1 0 39.8 0 0
0 6.2 0 0 14.6 19.2
0 1.2 0 0 11.4 27.4
AB40
AB42
ASynF
ASynR
0N3R
0N4R
40
35
30
25
20
15
10
5
0

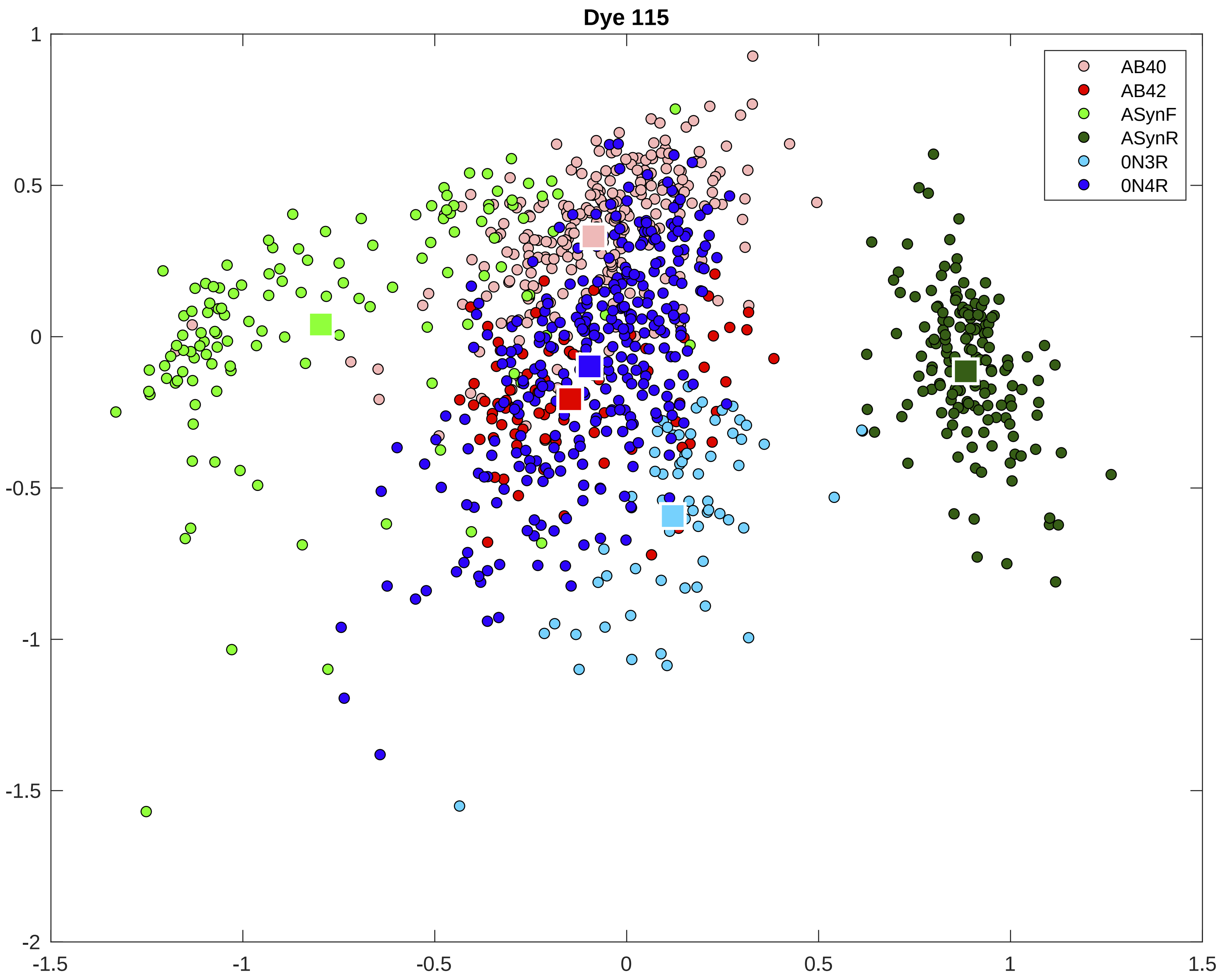

Dye 115
AB40
AB42
ASynF
ASynR
0N3R
0N4R
1
0.5
0
-0.5
-1
-1.5
-2
-1.5
-1
-0.5
0
0.5
1
1.5

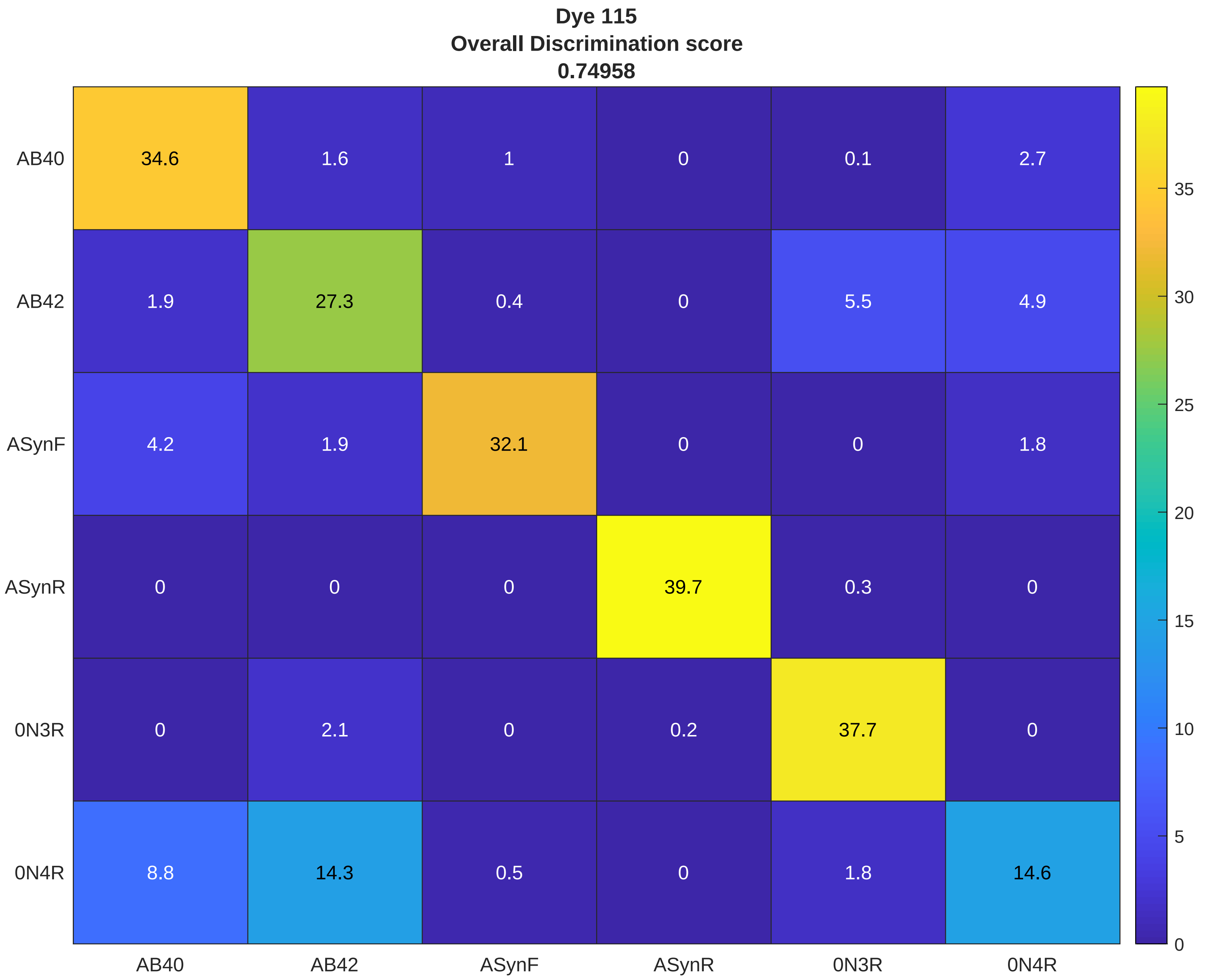
Dye 115
Overall Discrimination score
0.74958
AB40
AB42
ASynF
ASynR
0N3R
0N4R
34.6
1.6
1
0
0.1
2.7
1.9
27.3
0.4
0
5.5
4.9
4.2
1.9
32.1
0
0
1.8
0
0
0
39.7
0.3
0
0
2.1
0
0.2
37.7
0
8.8
14.3
0.5
0
1.8
14.6
AB40
AB42
ASynF
ASynR
0N3R
0N4R
35
30
25
20
15
10
5
0

Dye 116

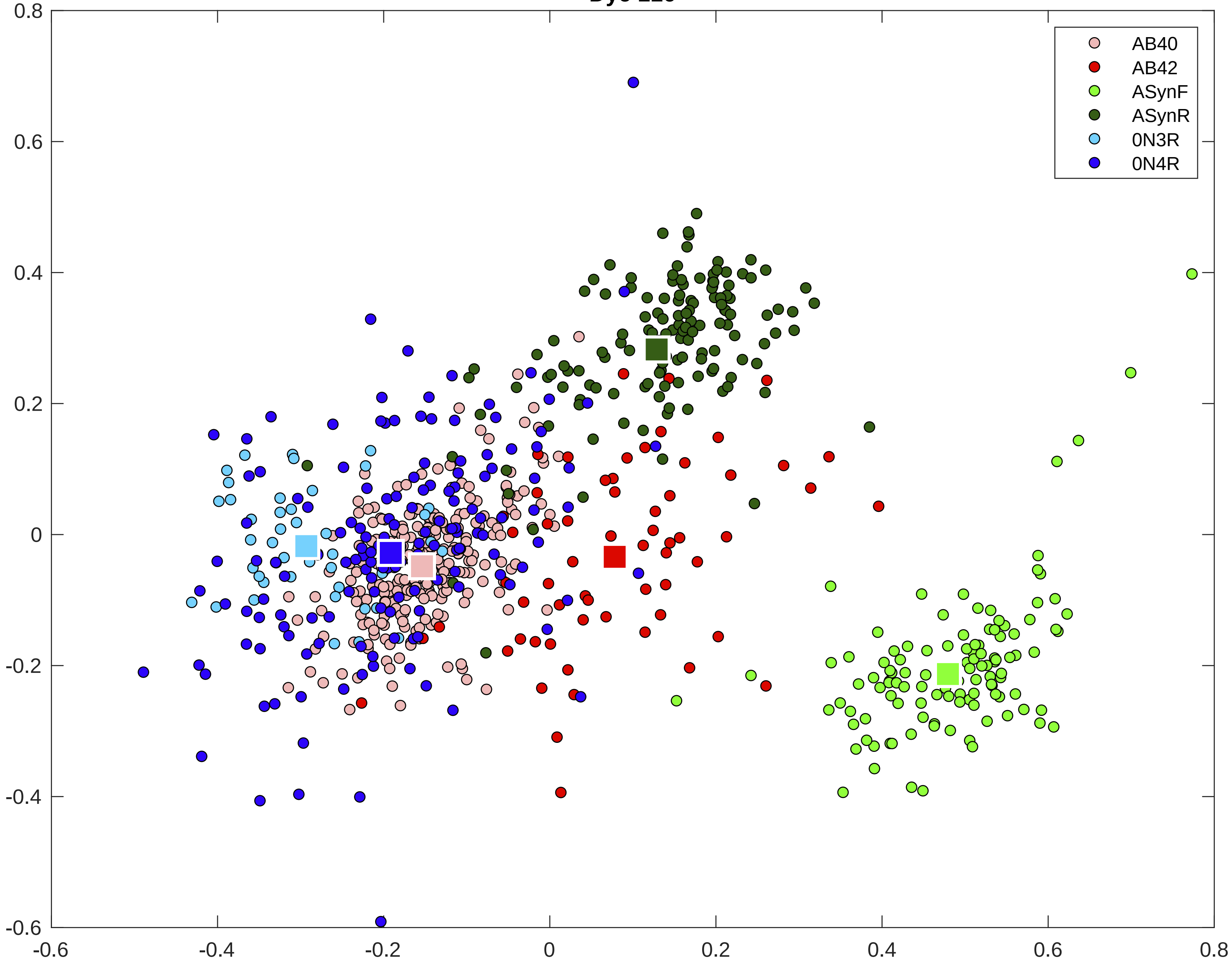

0.8
0.6
0.4
0.2
0
-0.2
-0.4
-0.6
-0.6
-0.4
-0.2
0
0.2
0.4
0.6
0.8
AB40
AB42
ASynF
ASynR
0N3R
0N4R

Dye 116
Overall Discrimination score
0.73958

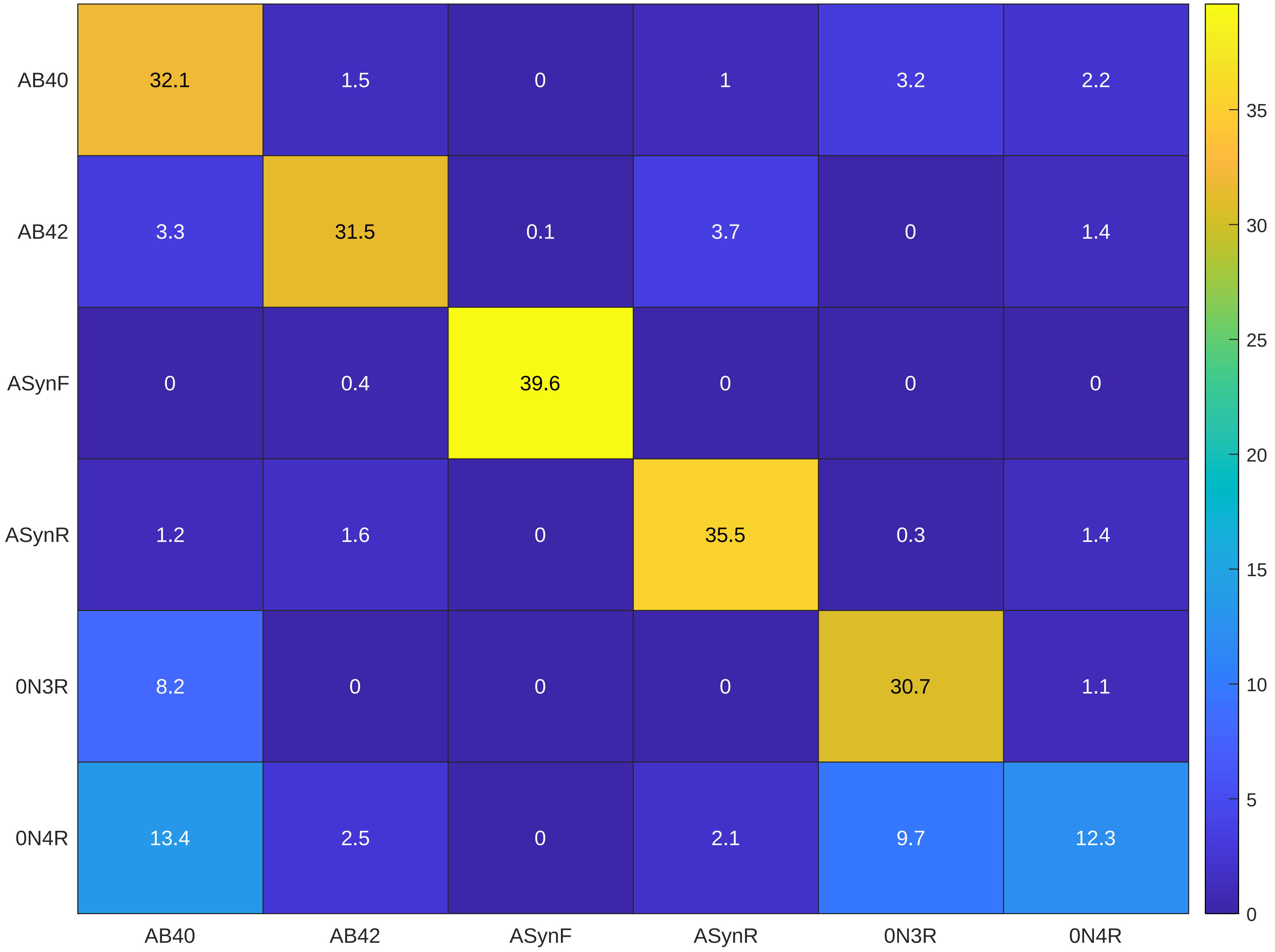

Dye 117

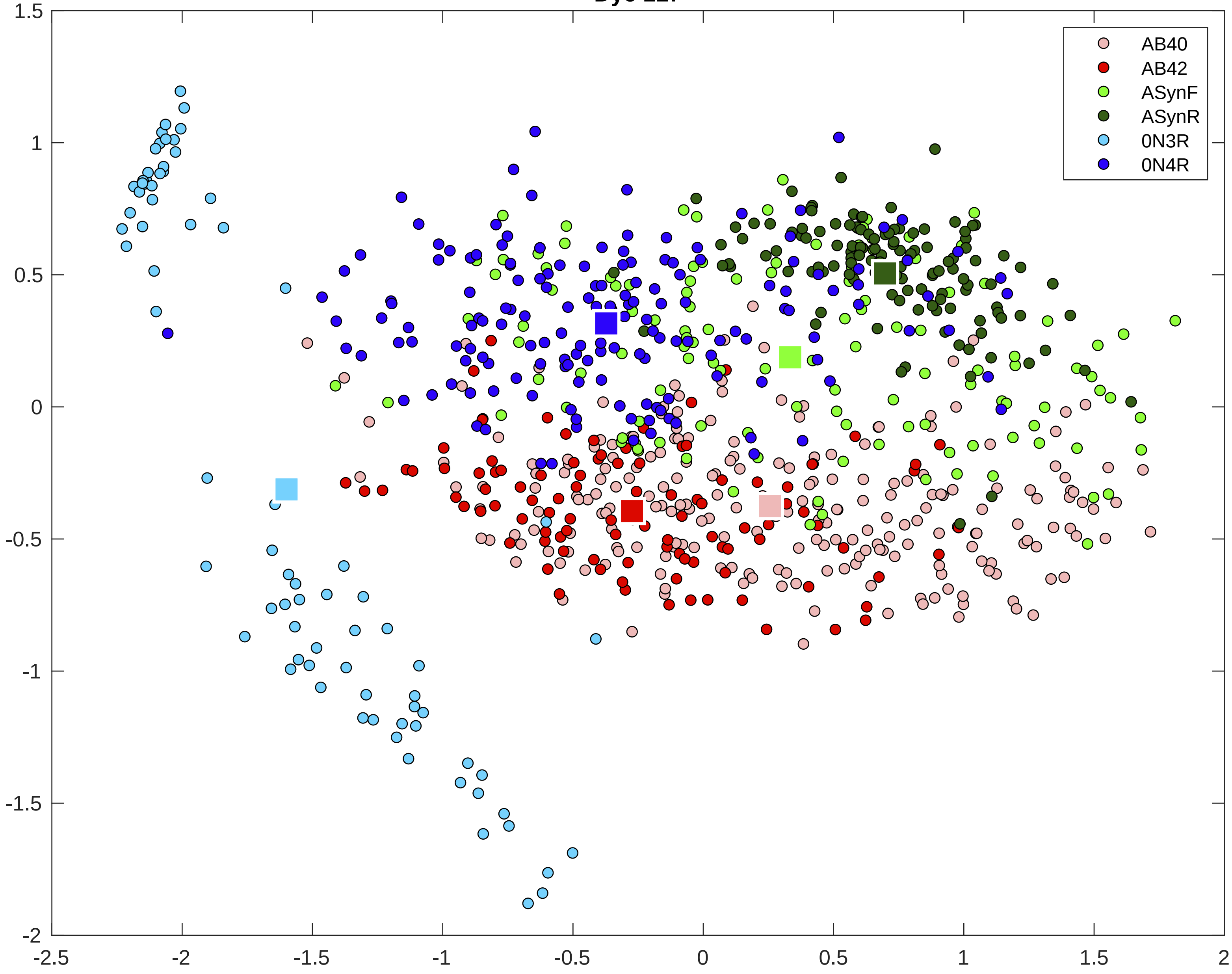

1.5
1
0.5
0
-0.5
-1
-1.5
-2
-2.5
-2
-1.5
-1
-0.5
0
0.5
1
1.5
2
AB40
AB42
ASynF
ASynR
0N3R
0N4R

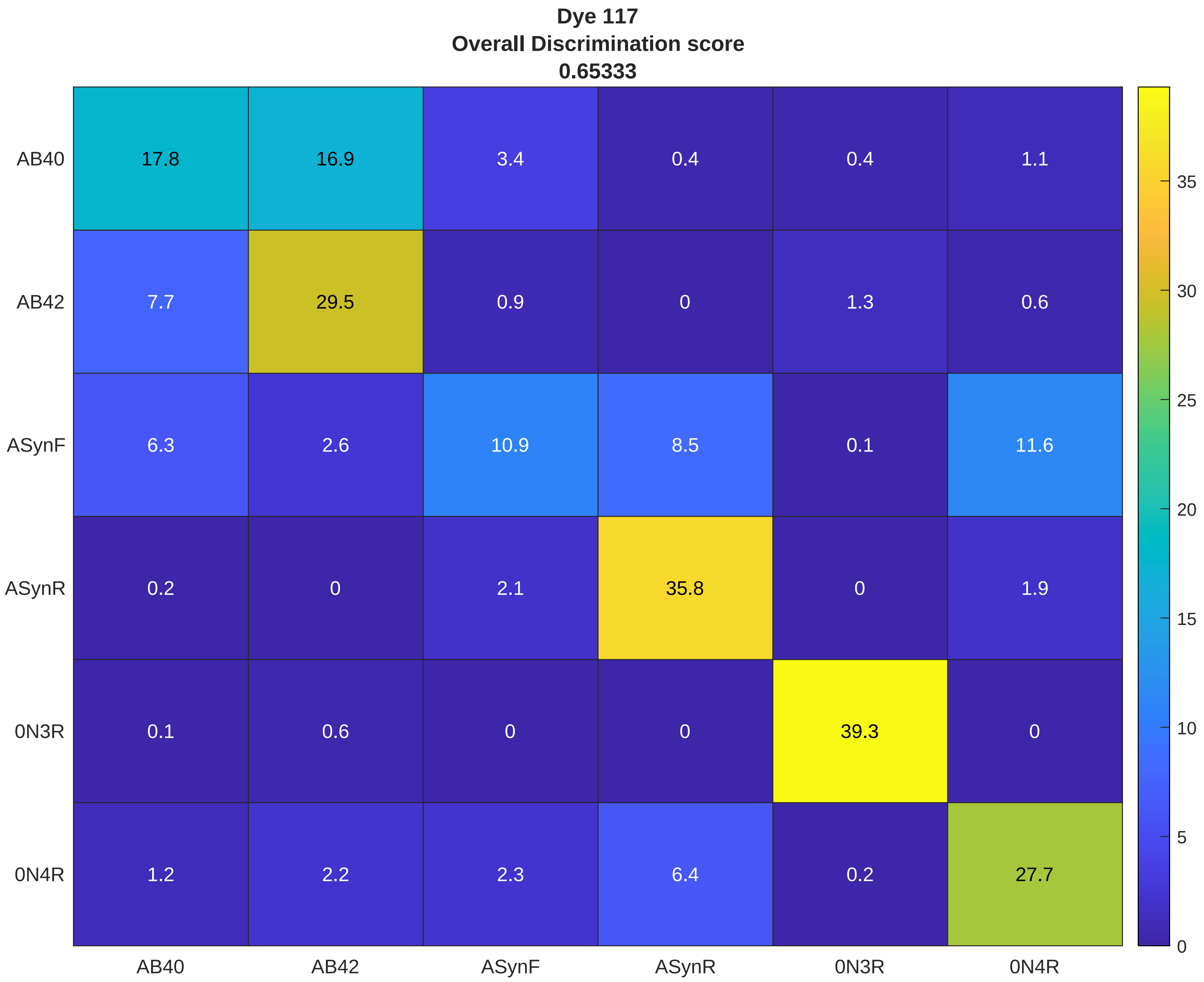
Dye 117
Overall Discrimination score
0.65333
AB40
AB42
ASynF
ASynR
0N3R
0N4R
17.8
16.9
3.4
0.4
0.4
1.1
7.7
29.5
0.9
0
1.3
0.6
6.3
2.6
10.9
8.5
0.1
11.6
0.2
0
2.1
35.8
0
1.9
0.1
0.6
0
0
39.3
0
1.2
2.2
2.3
6.4
0.2
27.7
AB40
AB42
ASynF
ASynR
0N3R
0N4R
35
30
25
20
15
10
5
0

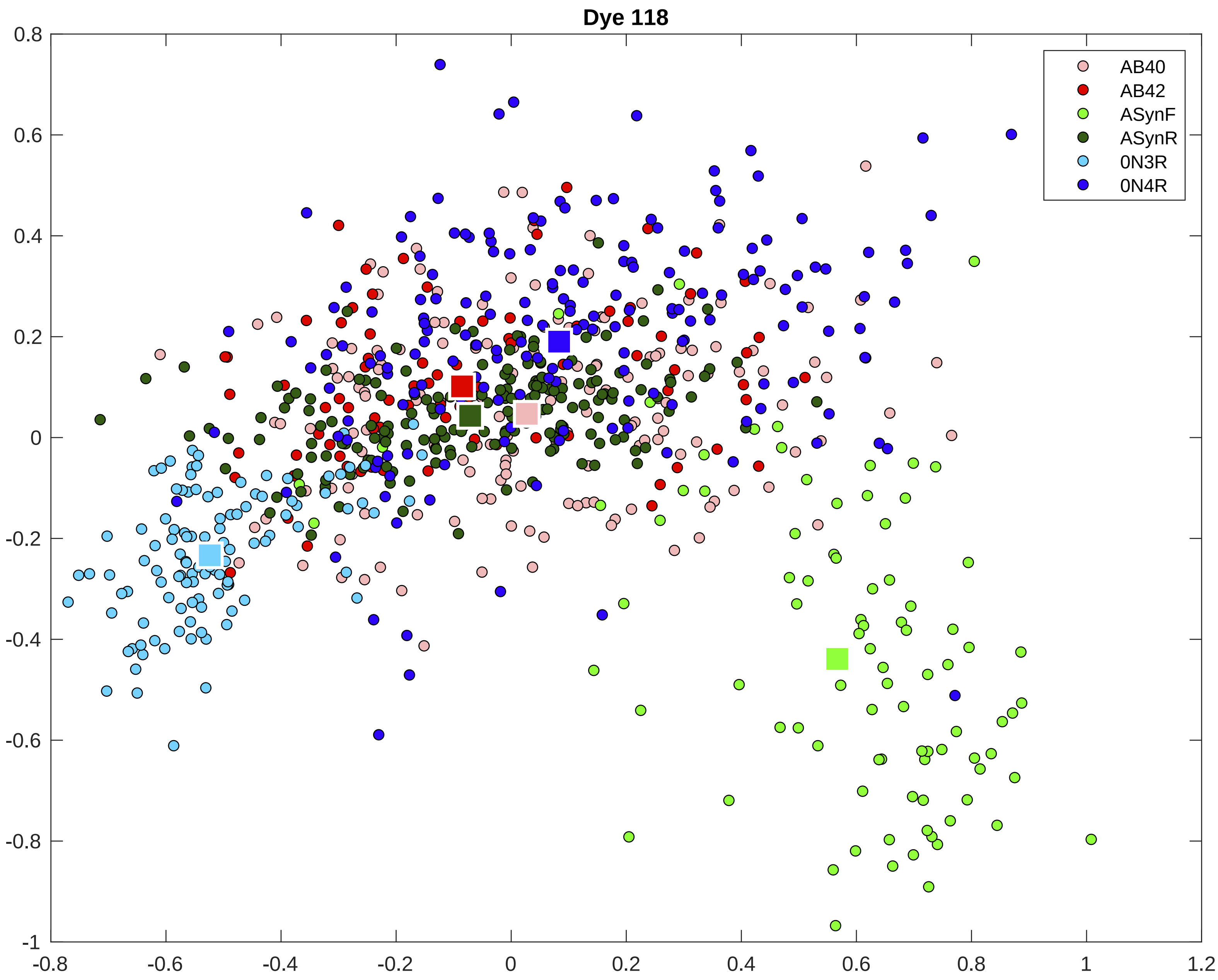

Dye 118
AB40
AB42
ASynF
ASynR
0N3R
0N4R
0.8
0.6
0.4
0.2
0
-0.2
-0.4
-0.6
-0.8
-1
-0.8
-0.6
-0.4
-0.2
0
0.2
0.4
0.6
0.8
1
1.2

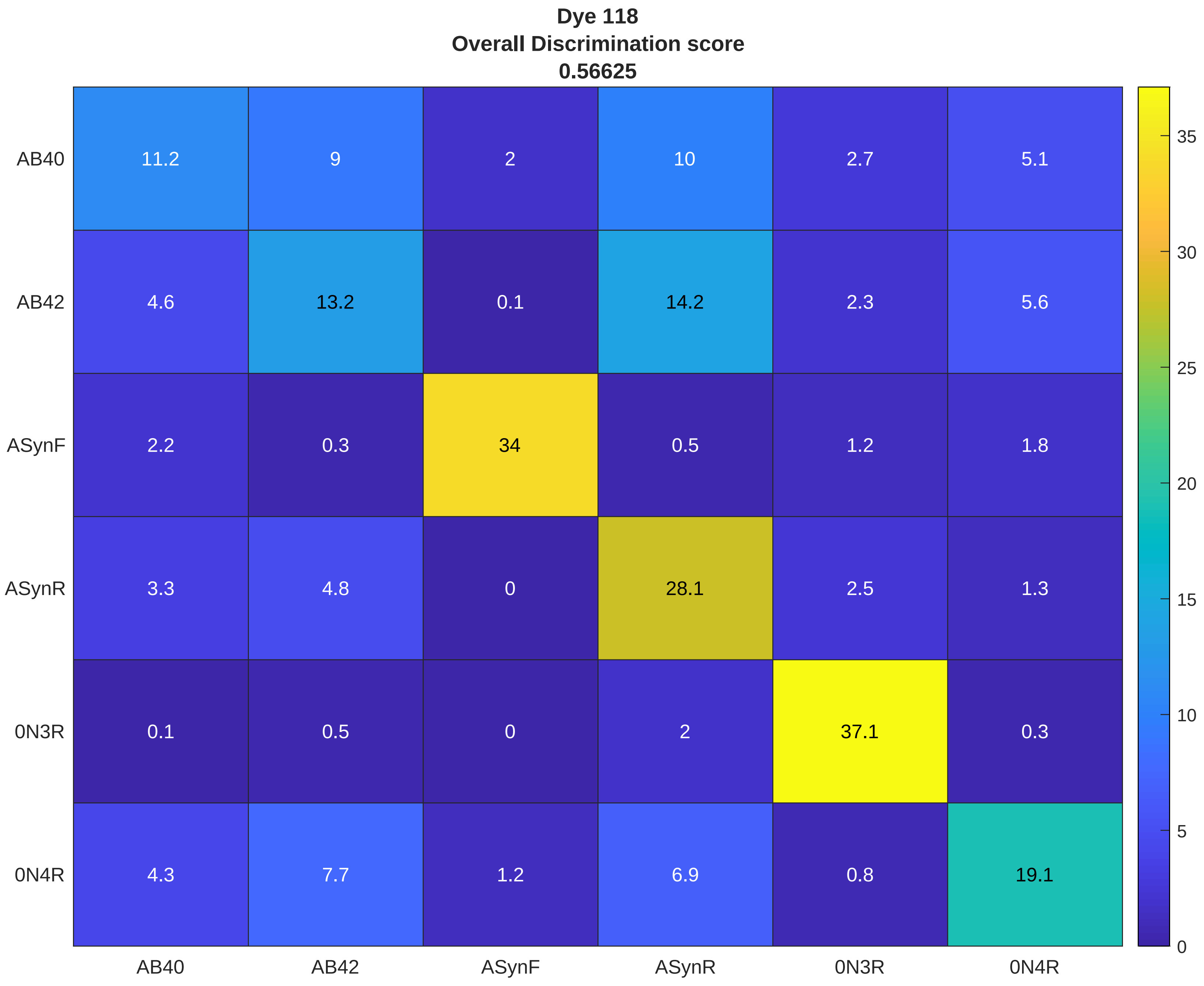
Dye 118
Overall Discrimination score
0.56625
AB40
AB42
ASynF
ASynR
0N3R
0N4R
11.2
9
2
10
2.7
5.1
4.6
13.2
0.1
14.2
2.3
5.6
2.2
0.3
34
0.5
1.2
1.8
3.3
4.8
0
28.1
2.5
1.3
0.1
0.5
0
2
37.1
0.3
4.3
7.7
1.2
6.9
0.8
19.1
AB40
AB42
ASynF
ASynR
0N3R
0N4R
35
30
25
20
15
10
5
0

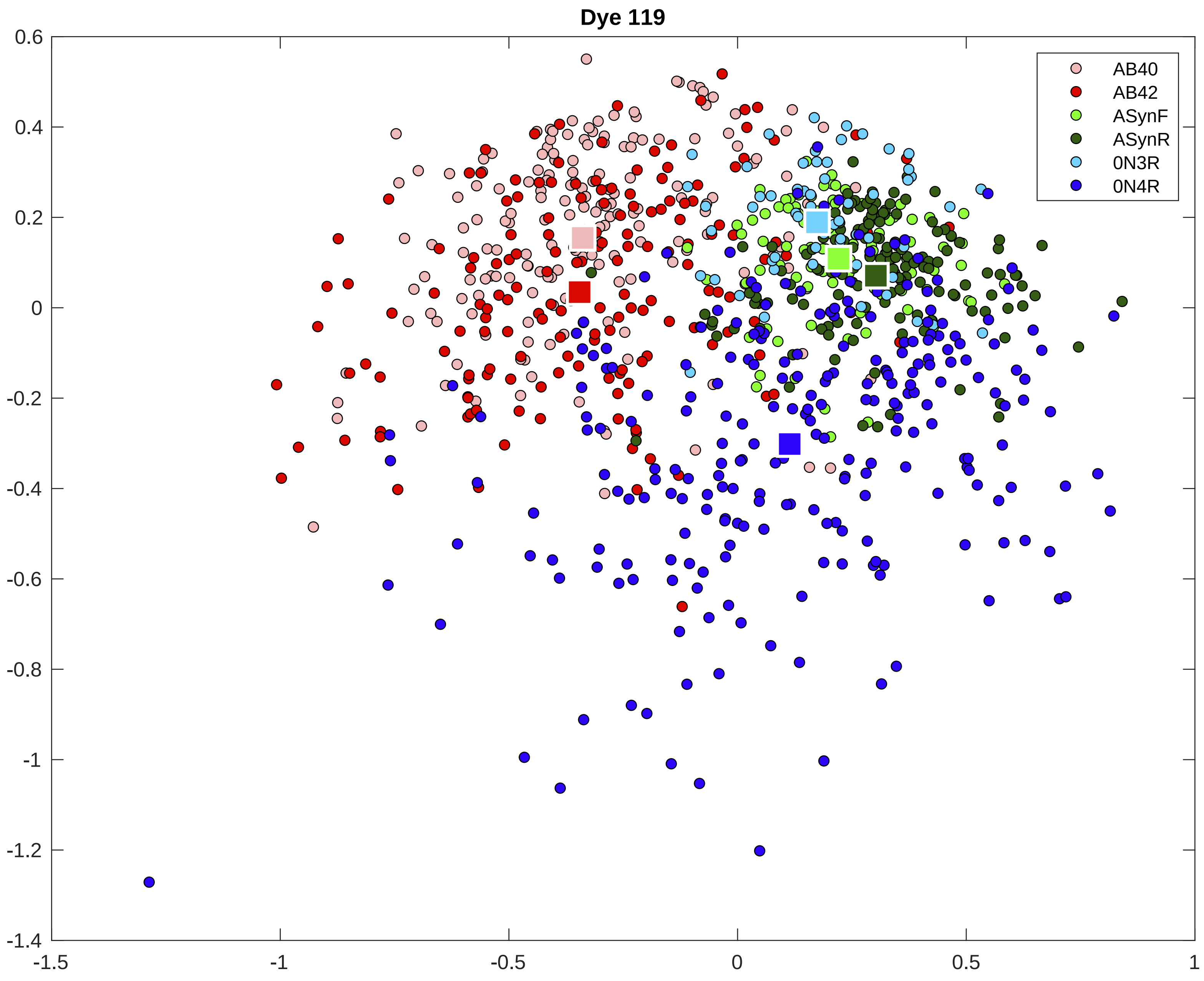

Dye 119
AB40
AB42
ASynF
ASynR
0N3R
0N4R
0.6
0.4
0.2
0
-0.2
-0.4
-0.6
-0.8
-1
-1.2
-1.4
-1.5
-1
-0.5
0
0.5
1

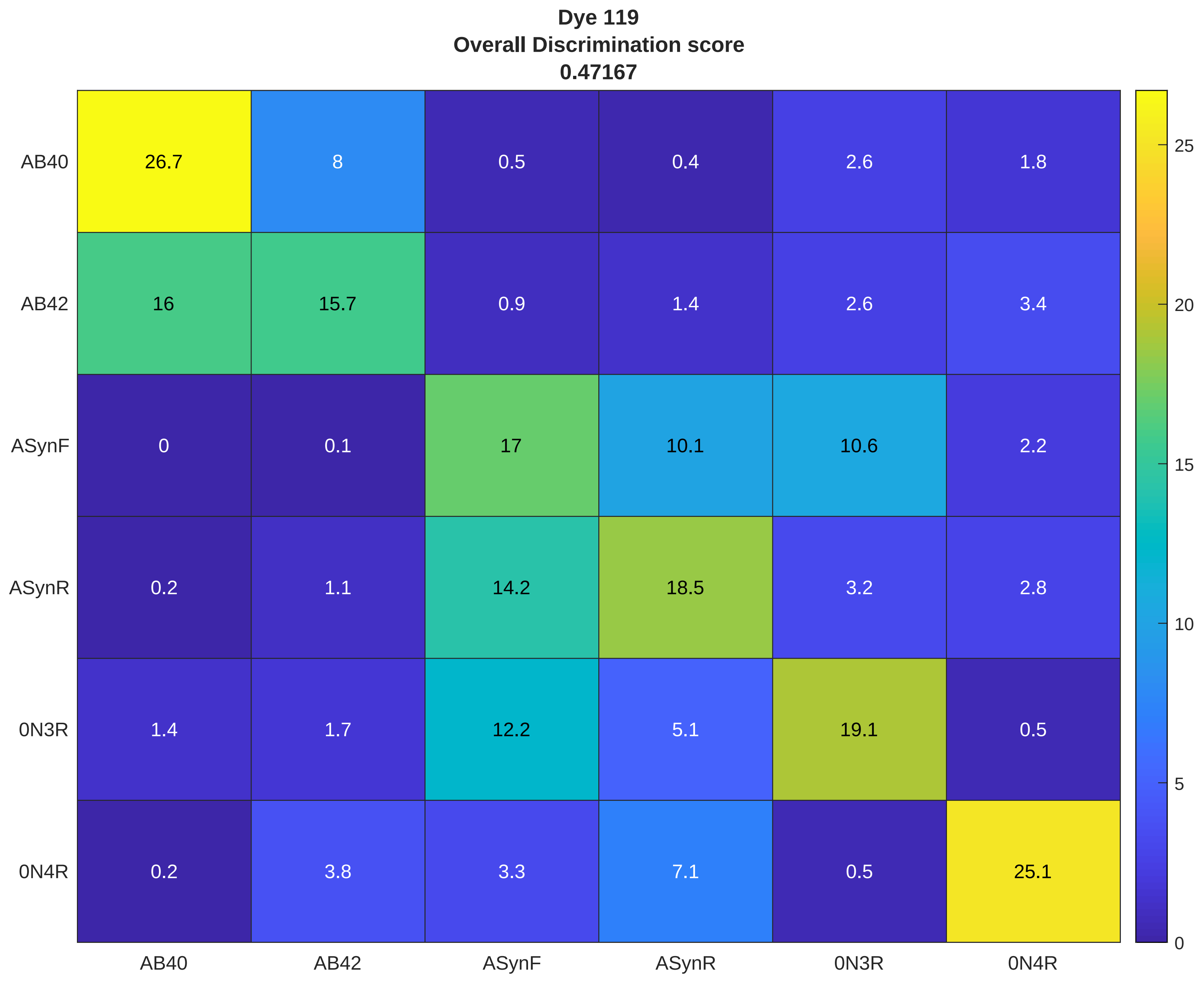
Dye 119
Overall Discrimination score
0.47167
AB40
AB42
ASynF
ASynR
0N3R
0N4R
26.7
8
0.5
0.4
2.6
1.8
16
15.7
0.9
1.4
2.6
3.4
0
0.1
17
10.1
10.6
2.2
0.2
1.1
14.2
18.5
3.2
2.8
1.4
1.7
12.2
5.1
19.1
0.5
0.2
3.8
3.3
7.1
0.5
25.1
AB40
AB42
ASynF
ASynR
0N3R
0N4R
25
20
15
10
5
0

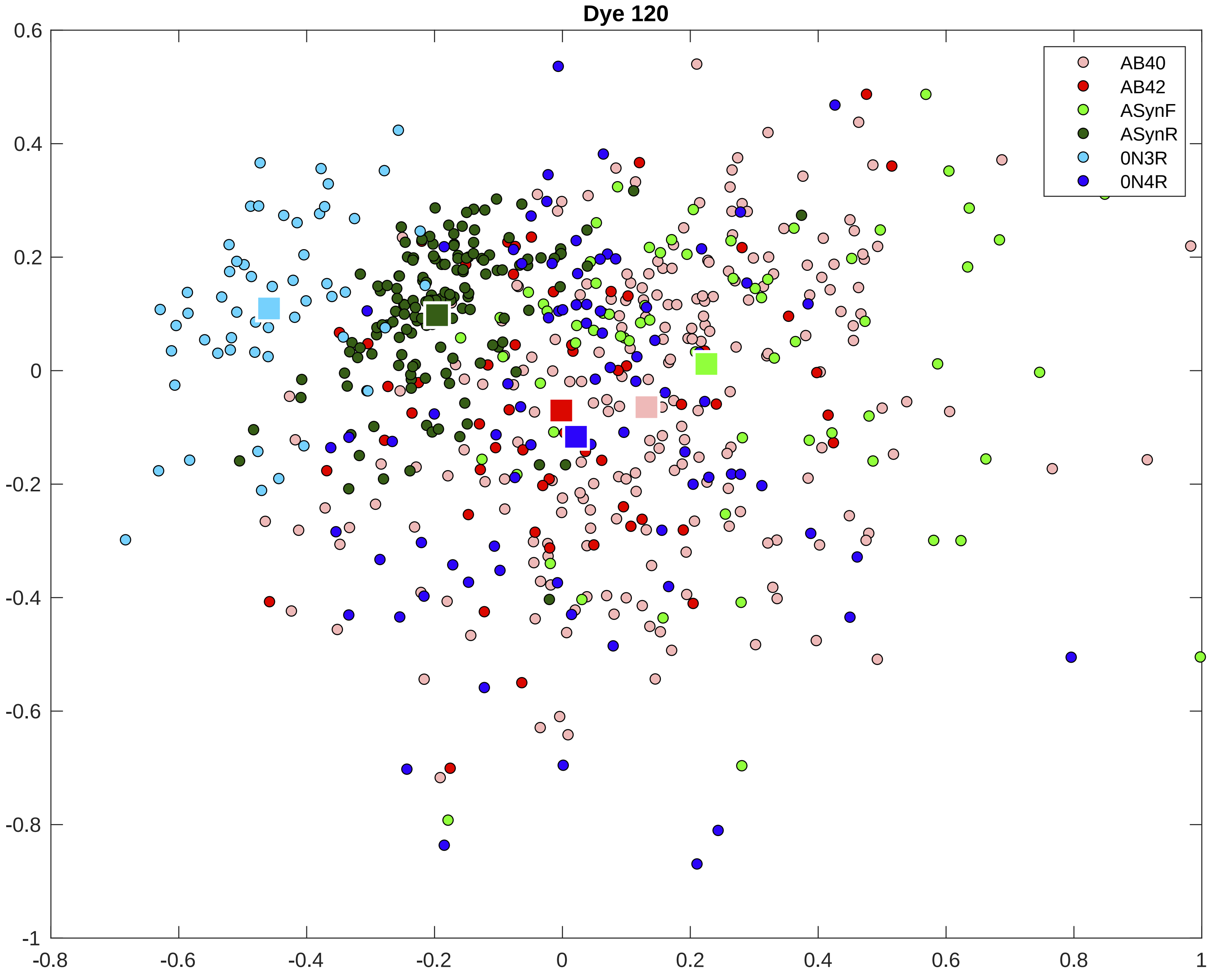
Dye 120
AB40
AB42
ASynF
ASynR
0N3R
0N4R
0.6
0.4
0.2
0
-0.2
-0.4
-0.6
-0.8
-1
-0.8
-0.6
-0.4
-0.2
0
0.2
0.4
0.6
0.8
1

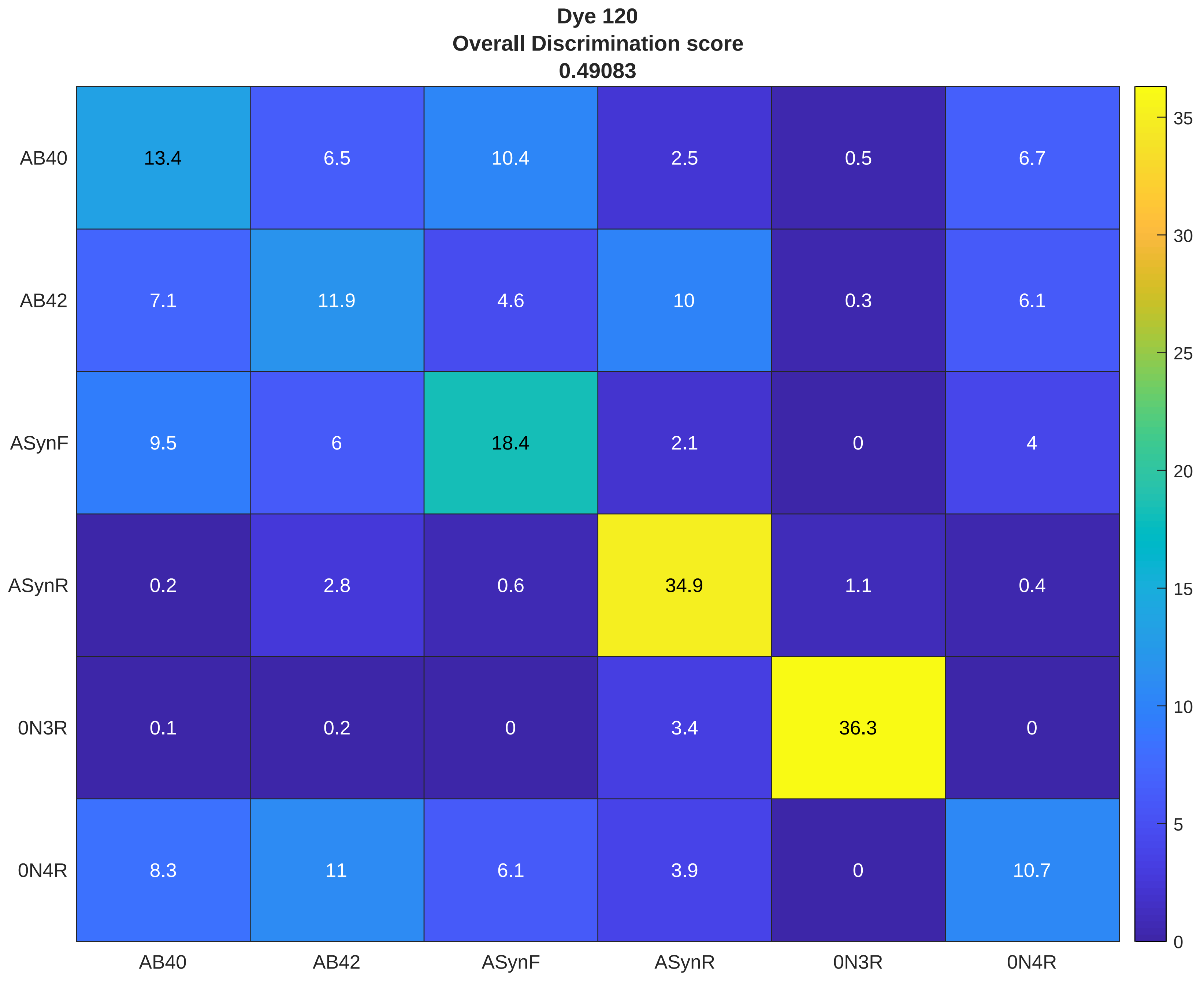
Dye 120
Overall Discrimination score
0.49083
AB40
AB42
ASynF
ASynR
0N3R
0N4R
13.4
6.5
10.4
2.5
0.5
6.7
7.1
11.9
4.6
10
0.3
6.1
9.5
6
18.4
2.1
0
4
0.2
2.8
0.6
34.9
1.1
0.4
0.1
0.2
0
3.4
36.3
0
8.3
11
6.1
3.9
0
10.7
AB40
AB42
ASynF
ASynR
0N3R
0N4R
35
30
25
20
15
10
5
0

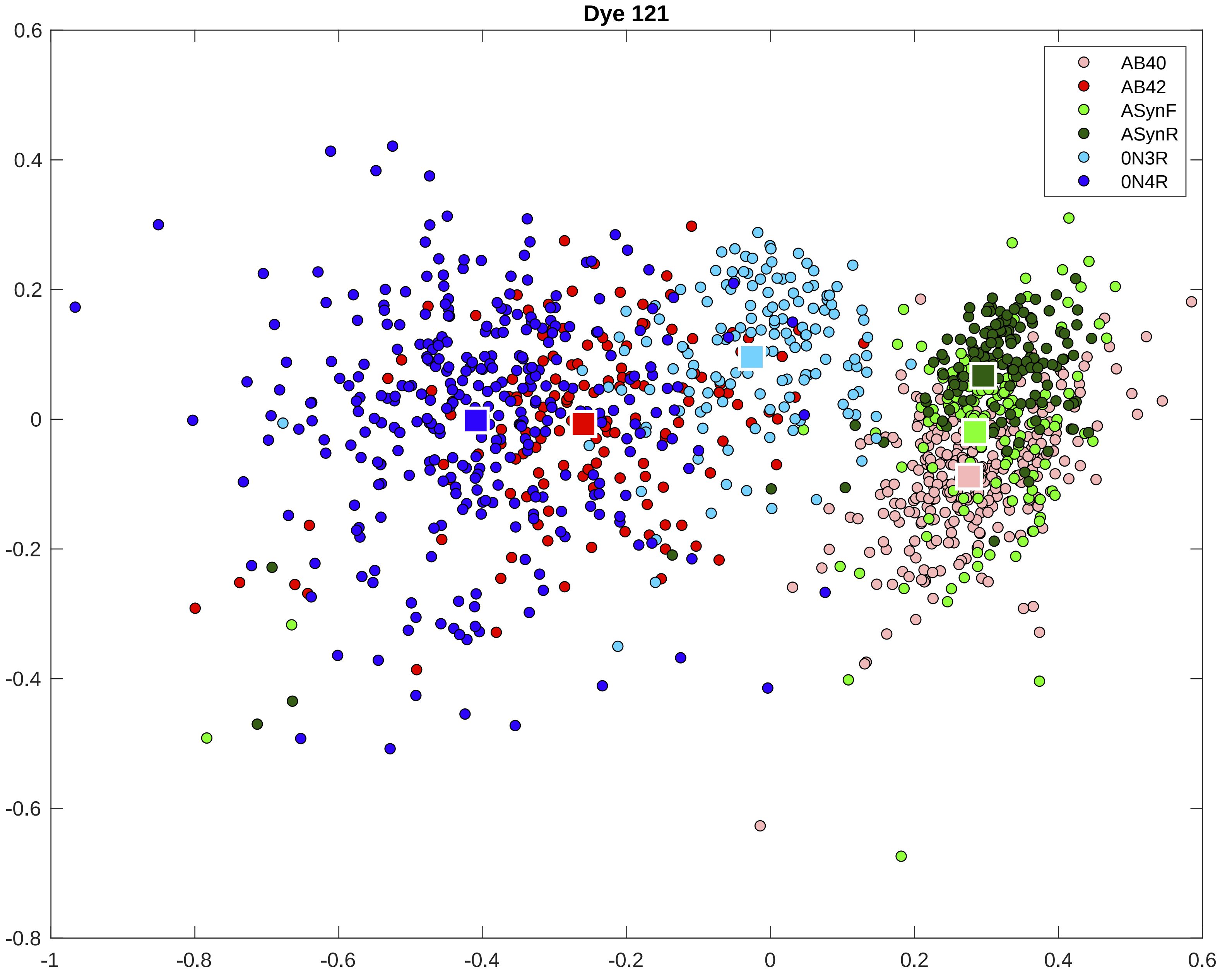

Dye 121
AB40
AB42
ASynF
ASynR
0N3R
0N4R
0.6
0.4
0.2
0
-0.2
-0.4
-0.6
-0.8
-1
-0.8
-0.6
-0.4
-0.2
0
0.2
0.4
0.6

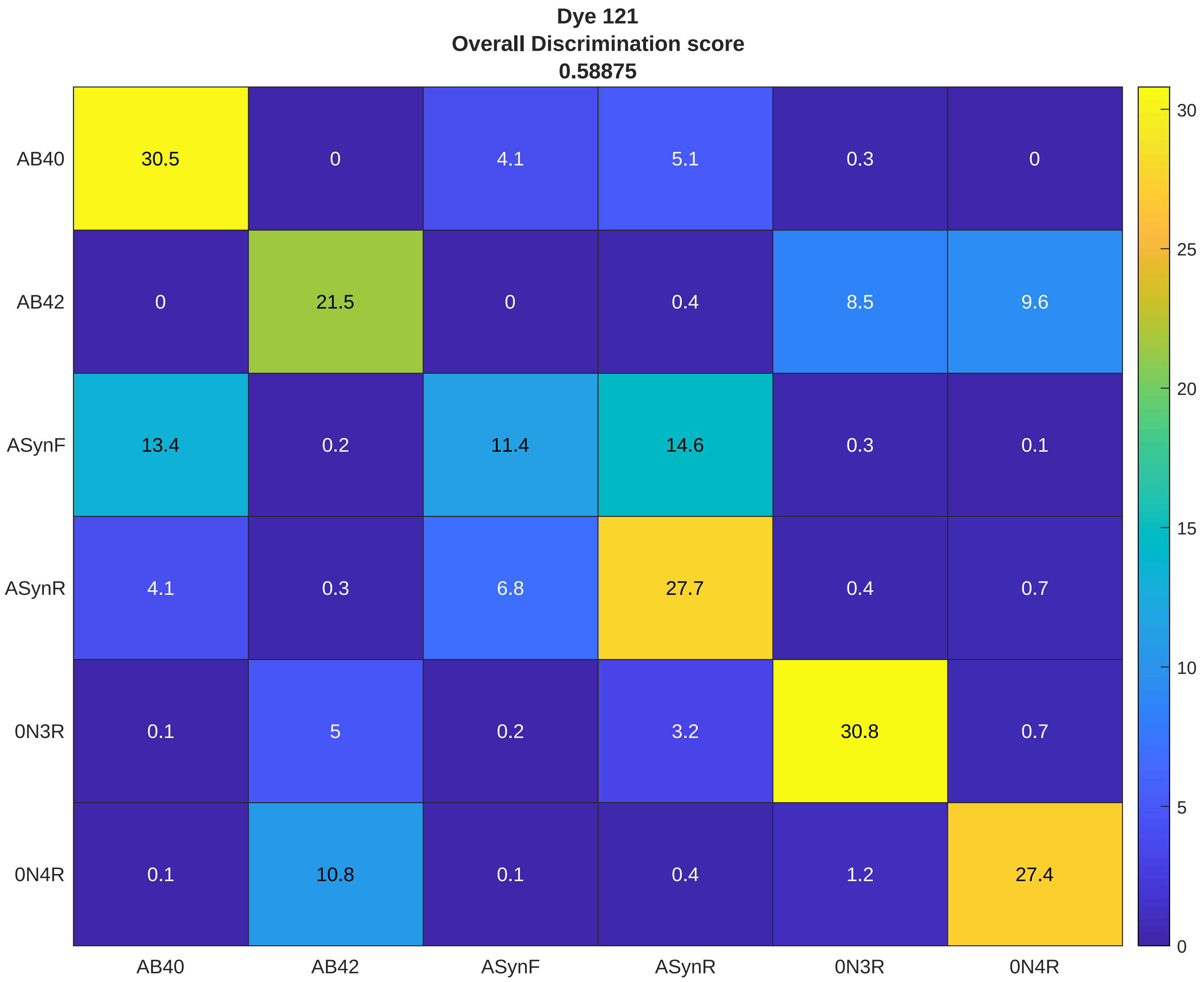

Dye 121
Overall Discrimination score
0.58875
AB40
AB42
ASynF
ASynR
0N3R
0N4R
30.5
0
4.1
5.1
0.3
0
0
21.5
0
0.4
8.5
9.6
13.4
0.2
11.4
14.6
0.3
0.1
4.1
0.3
6.8
27.7
0.4
0.7
0.1
5
0.2
3.2
30.8
0.7
0.1
10.8
0.1
0.4
1.2
27.4
AB40
AB42
ASynF
ASynR
0N3R
0N4R
30
25
20
15
10
5
0

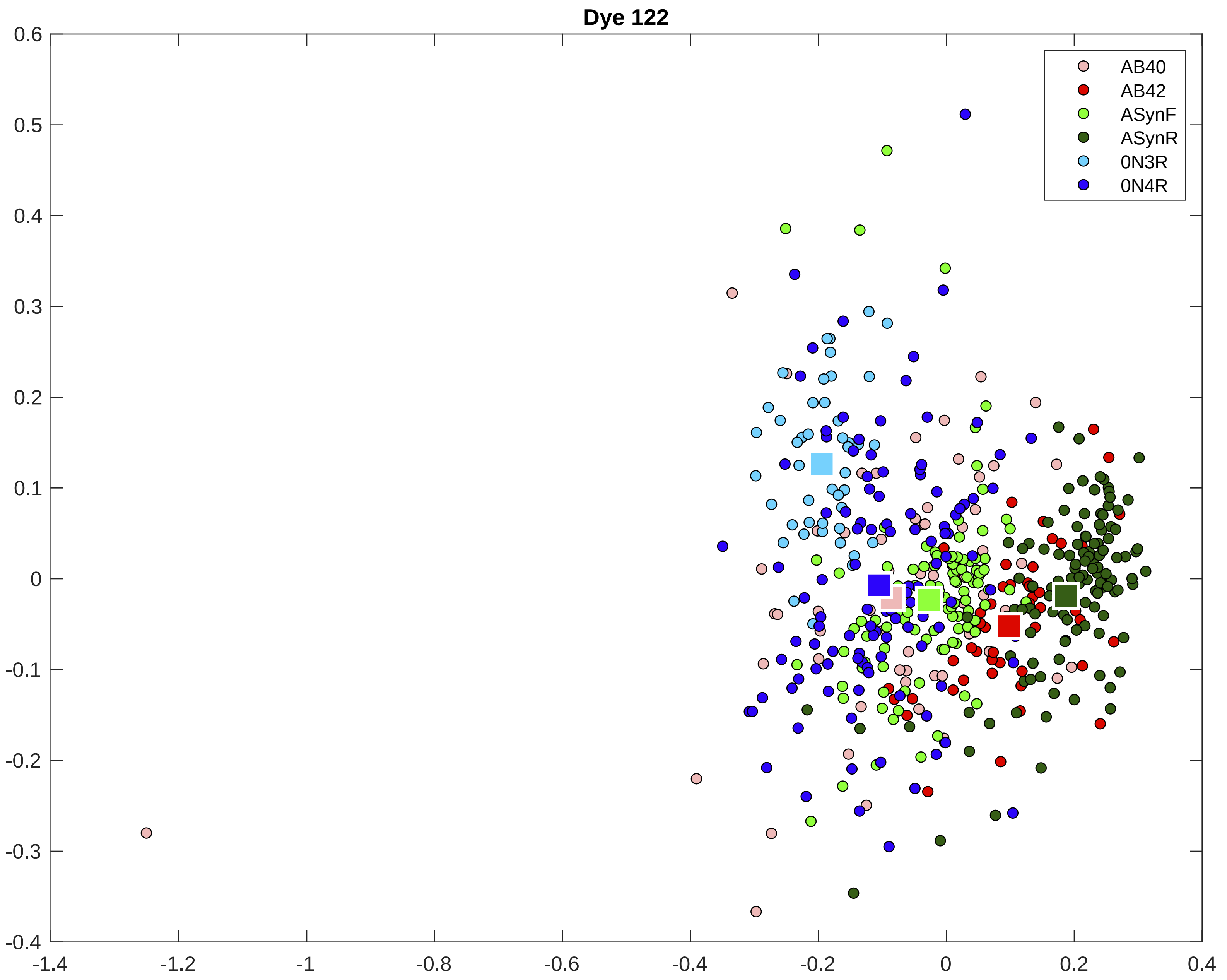

Dye 122
AB40
AB42
ASynF
ASynR
0N3R
0N4R
0.6
0.5
0.4
0.3
0.2
0.1
0
-0.1
-0.2
-0.3
-0.4
-1.4
-1.2
-1
-0.8
-0.6
-0.4
-0.2
0
0.2
0.4

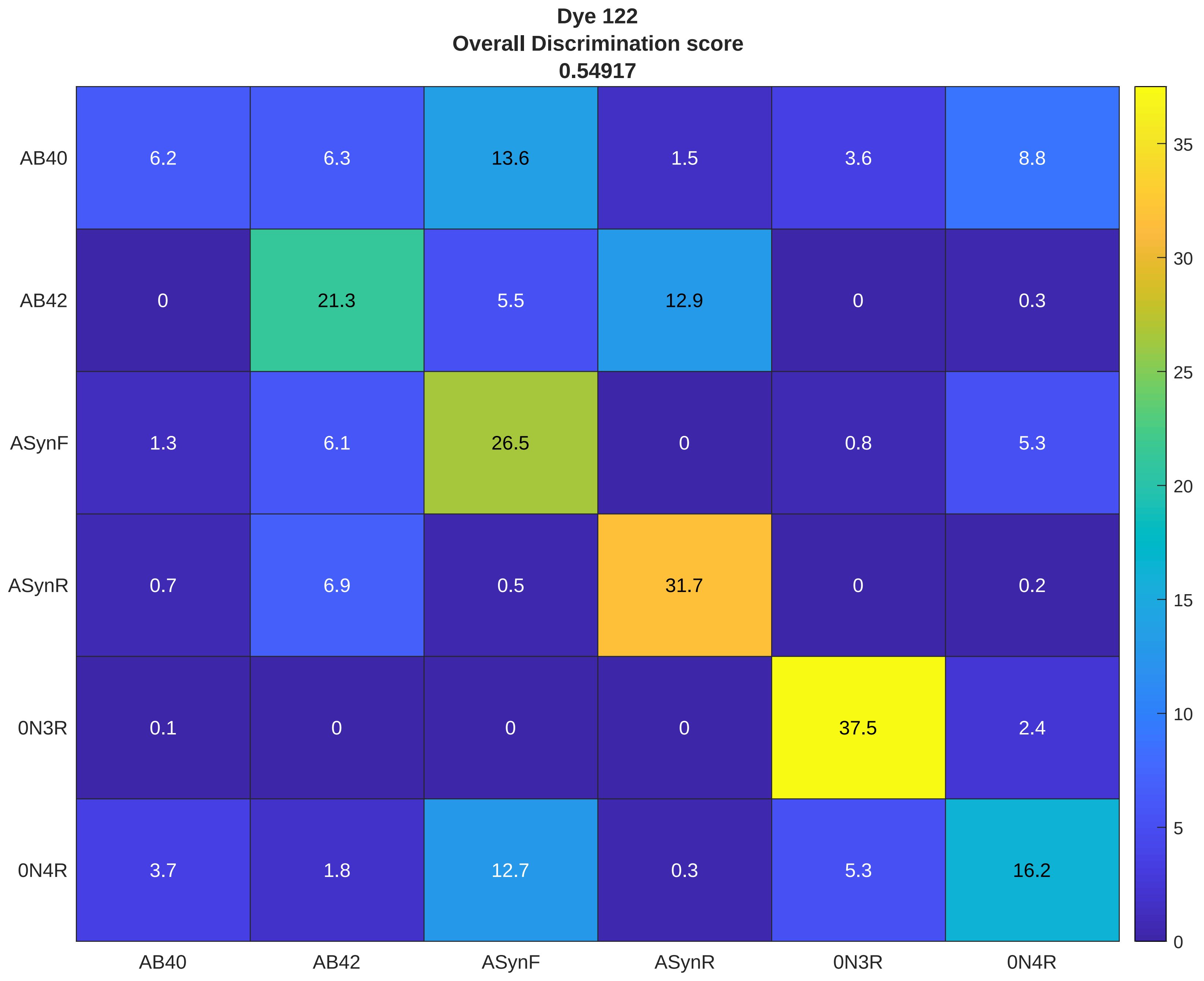
Dye 122
Overall Discrimination score
0.54917
AB40
AB42
ASynF
ASynR
0N3R
0N4R
6.2
6.3
13.6
1.5
3.6
8.8
0
21.3
5.5
12.9
0
0.3
1.3
6.1
26.5
0
0.8
5.3
0.7
6.9
0.5
31.7
0
0.2
0.1
0
0
0
37.5
2.4
3.7
1.8
12.7
0.3
5.3
16.2
0
5
10
15
20
25
30
35

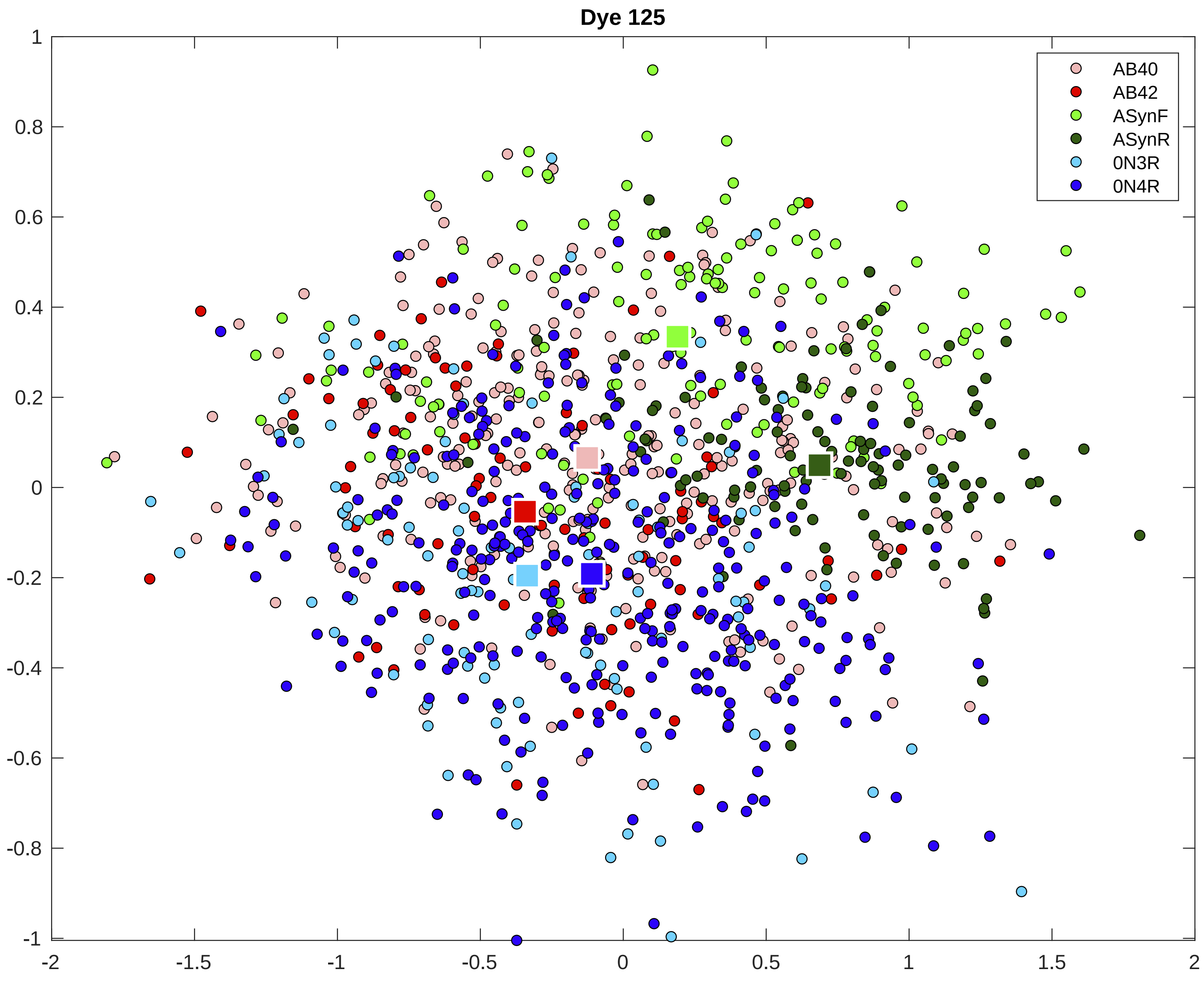

Dye 125
AB40
AB42
ASynF
ASynR
0N3R
0N4R
1
0.8
0.6
0.4
0.2
0
-0.2
-0.4
-0.6
-0.8
-1
-2
-1.5
-1
-0.5
0
0.5
1
1.5
2

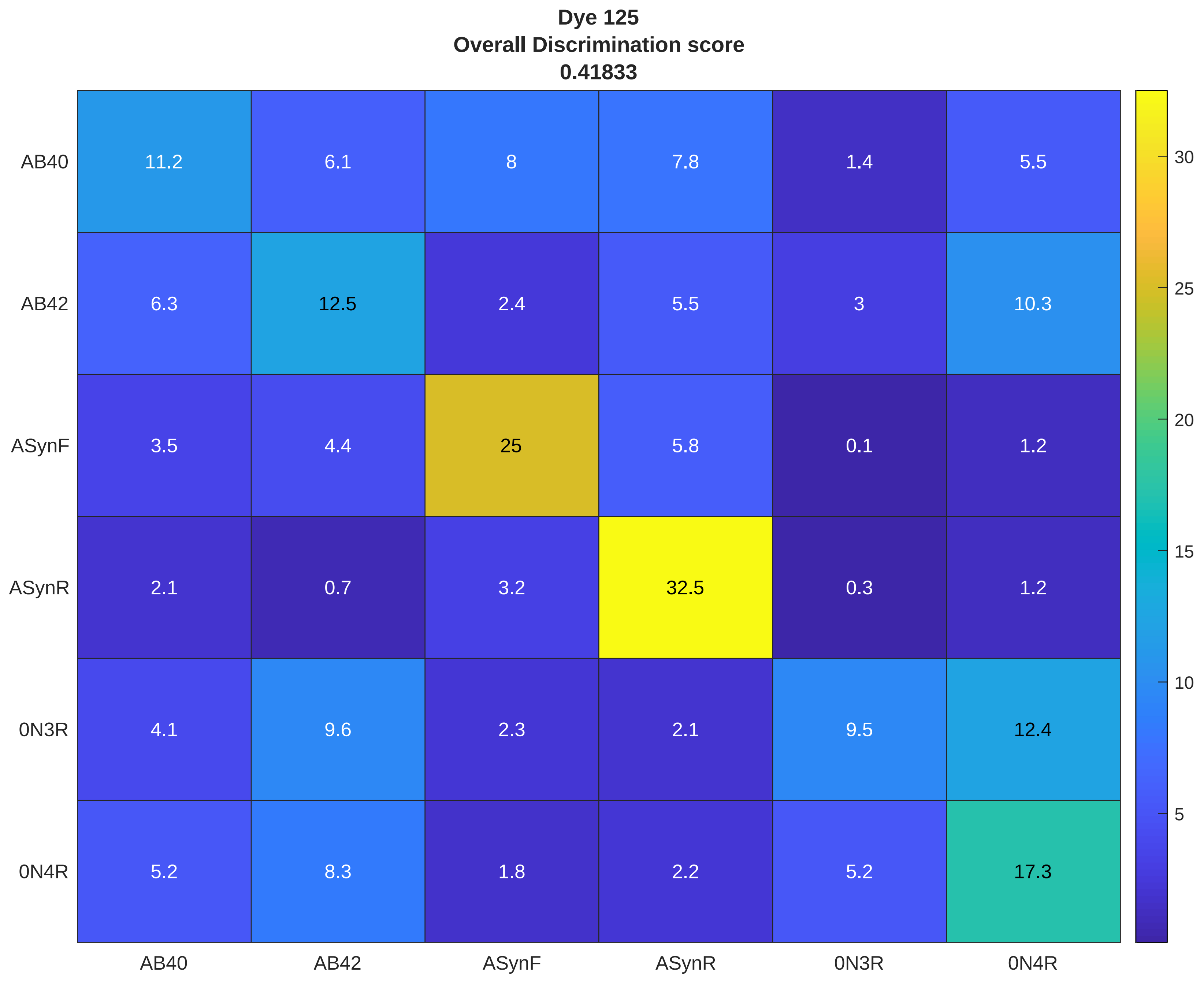
Dye 125
Overall Discrimination score
0.41833
AB40
AB42
ASynF
ASynR
0N3R
0N4R
11.2
6.1
8
7.8
1.4
5.5
6.3
12.5
2.4
5.5
3
10.3
3.5
4.4
25
5.8
0.1
1.2
2.1
0.7
3.2
32.5
0.3
1.2
4.1
9.6
2.3
2.1
9.5
12.4
5.2
8.3
1.8
2.2
5.2
17.3
30
25
20
15
10
5

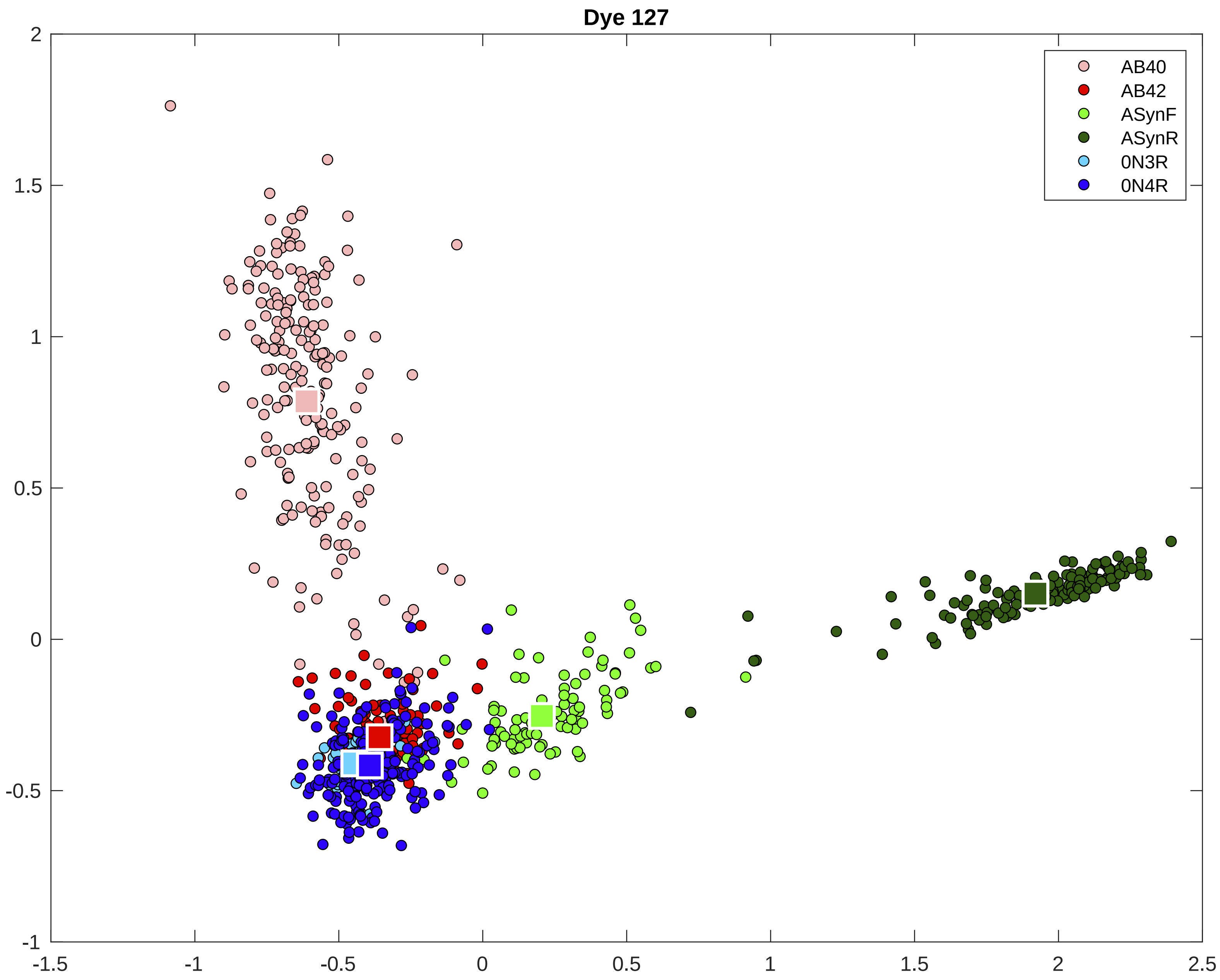
Dye 127
AB40
AB42
ASynF
ASynR
0N3R
0N4R
2
1.5
1
0.5
0
-0.5
-1
-1.5
-1
-0.5
0
0.5
1
1.5
2
2.5

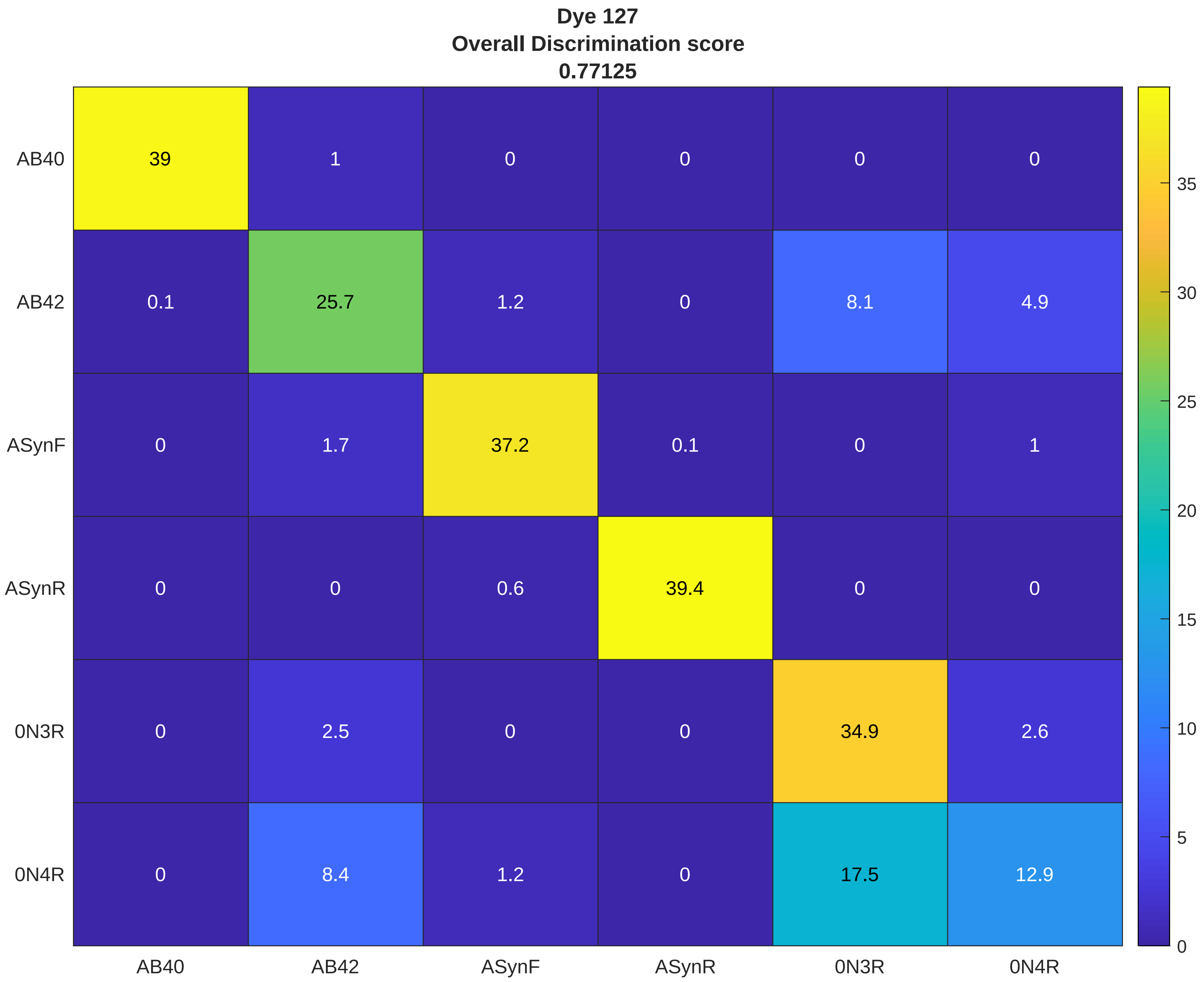

Dye 127
Overall Discrimination score
0.77125
AB40
AB42
ASynF
ASynR
0N3R
0N4R
39
1
0
0
0
0
0.1
25.7
1.2
0
8.1
4.9
0
1.7
37.2
0.1
0
1
0
0
0.6
39.4
0
0
0
2.5
0
0
34.9
2.6
0
8.4
1.2
0
17.5
12.9
AB40
AB42
ASynF
ASynR
0N3R
0N4R
35
30
25
20
15
10
5
0

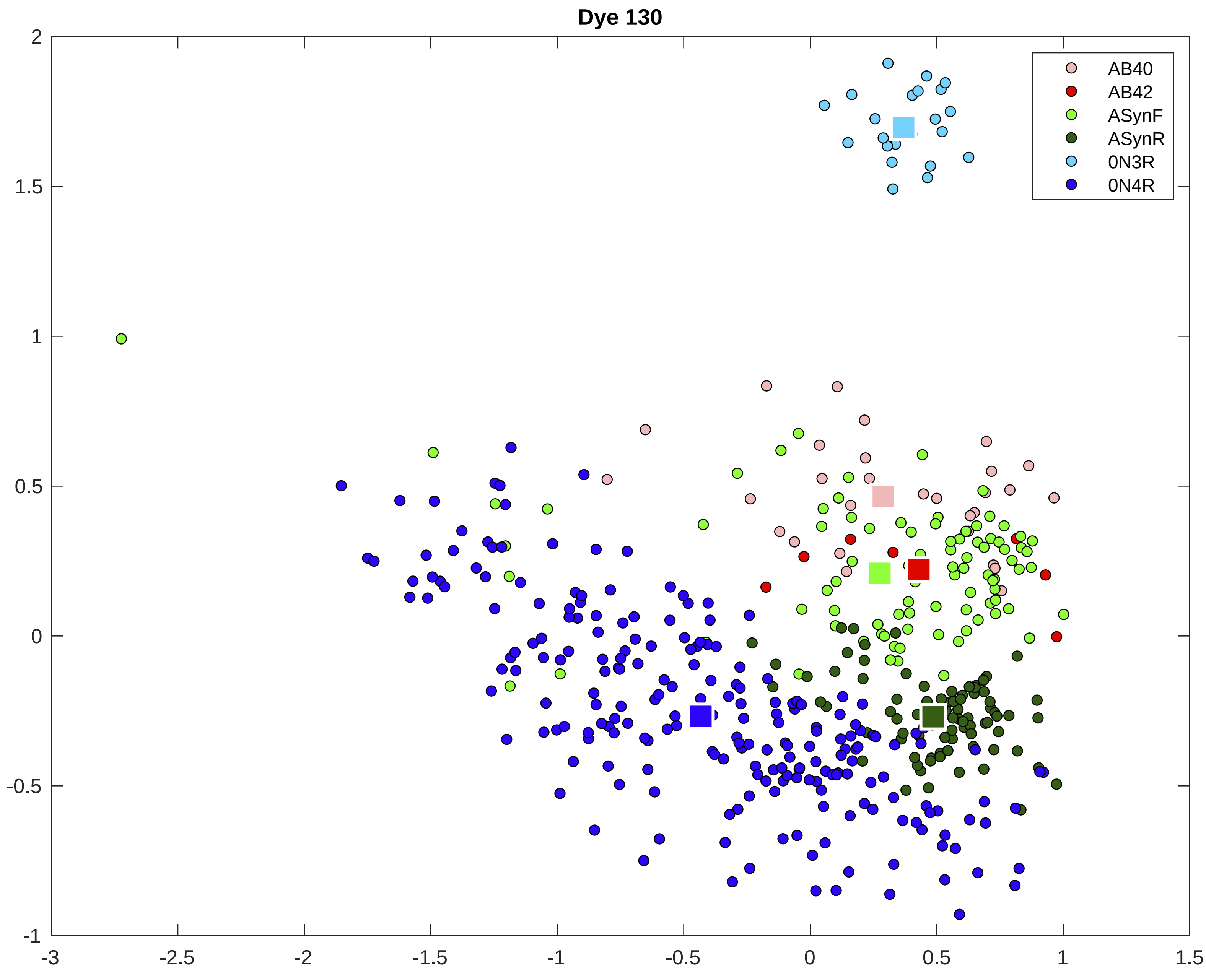

Dye 130
AB40
AB42
ASynF
ASynR
0N3R
0N4R
2
1.5
1
0.5
0
-0.5
-1
-3
-2.5
-2
-1.5
-1
-0.5
0
0.5
1
1.5

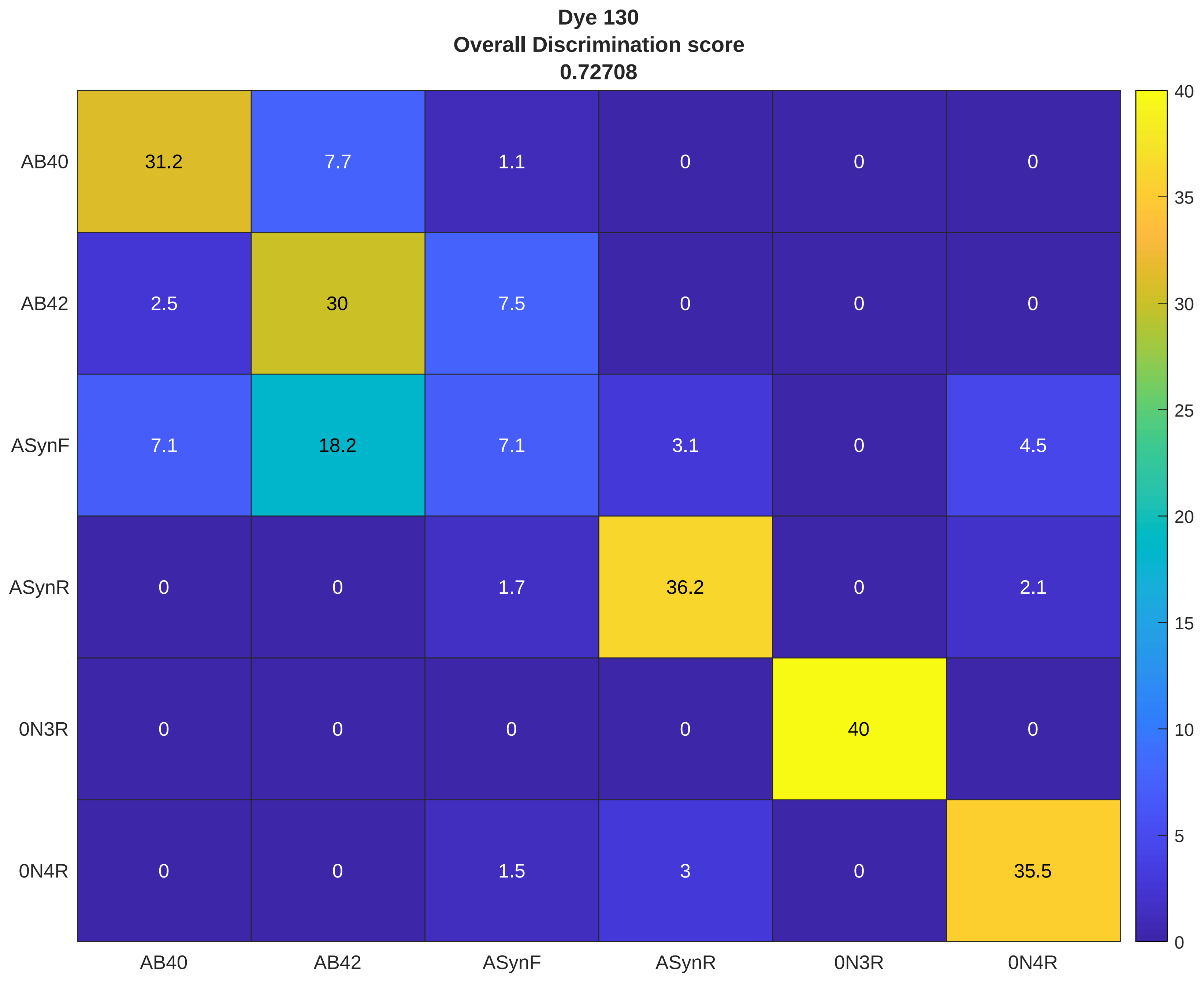

Dye 130
Overall Discrimination score
0.72708
AB40
AB42
ASynF
ASynR
0N3R
0N4R
31.2
7.7
1.1
0
0
0
2.5
30
7.5
0
0
0
7.1
18.2
7.1
3.1
0
4.5
0
0
1.7
36.2
0
2.1
0
0
0
0
40
0
0
0
1.5
3
0
35.5
AB40
AB42
ASynF
ASynR
0N3R
0N4R
40
35
30
25
20
15
10
5
0

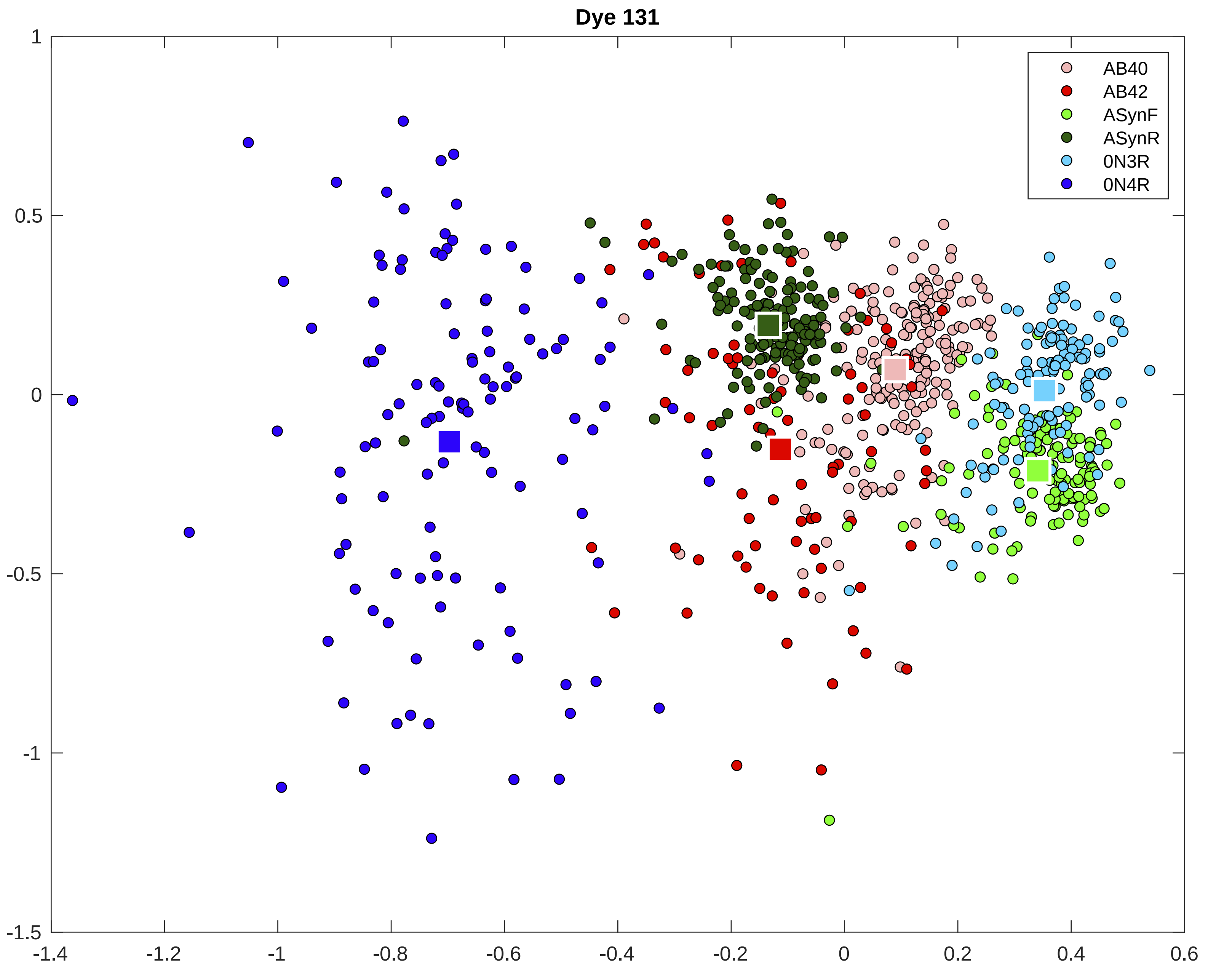

Dye 131
AB40
AB42
ASynF
ASynR
0N3R
0N4R
1
0.5
0
-0.5
-1
-1.5
-1.4
-1.2
-1
-0.8
-0.6
-0.4
-0.2
0
0.2
0.4
0.6

Dye 131
Overall Discrimination score
0.79458

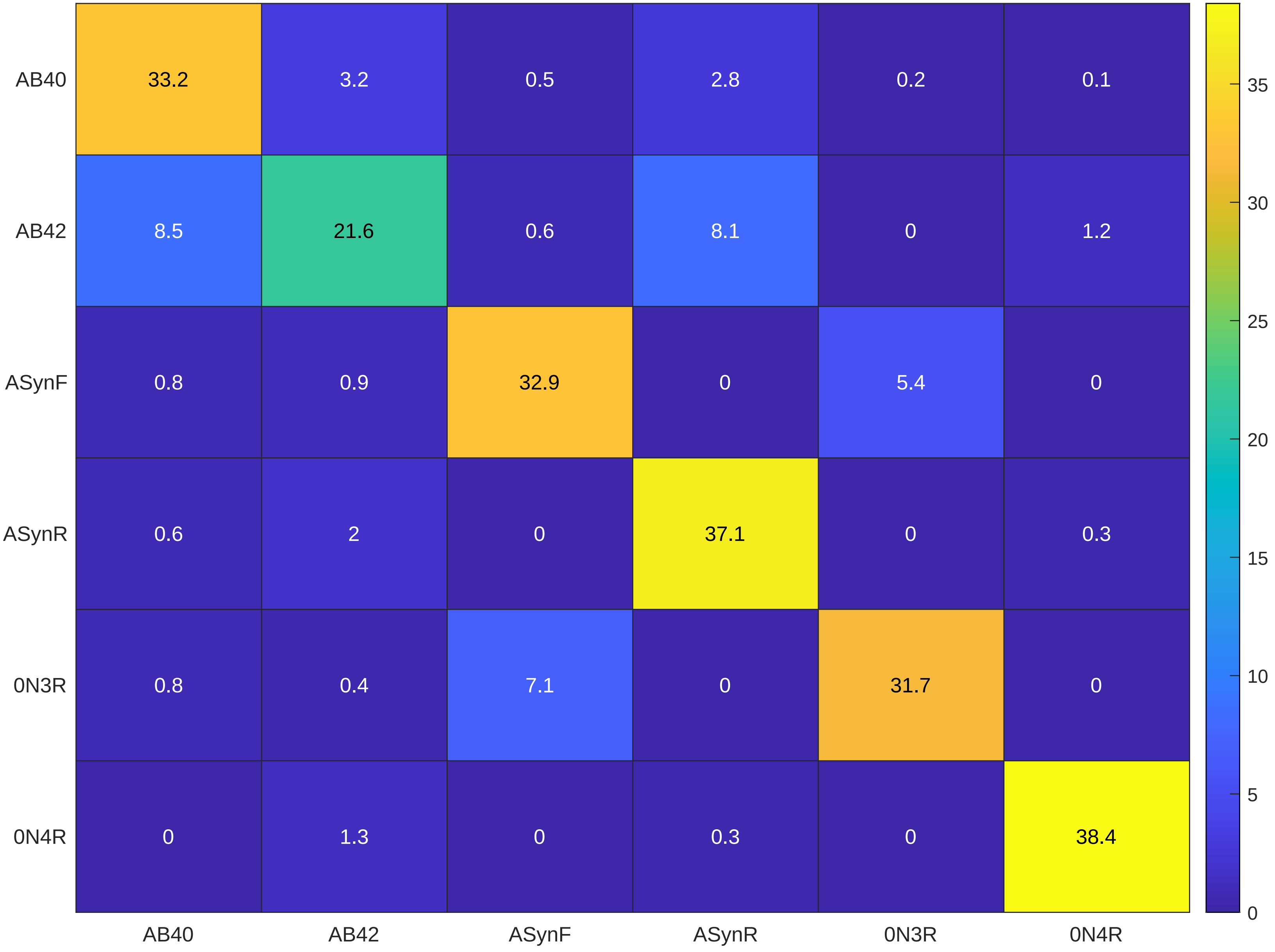

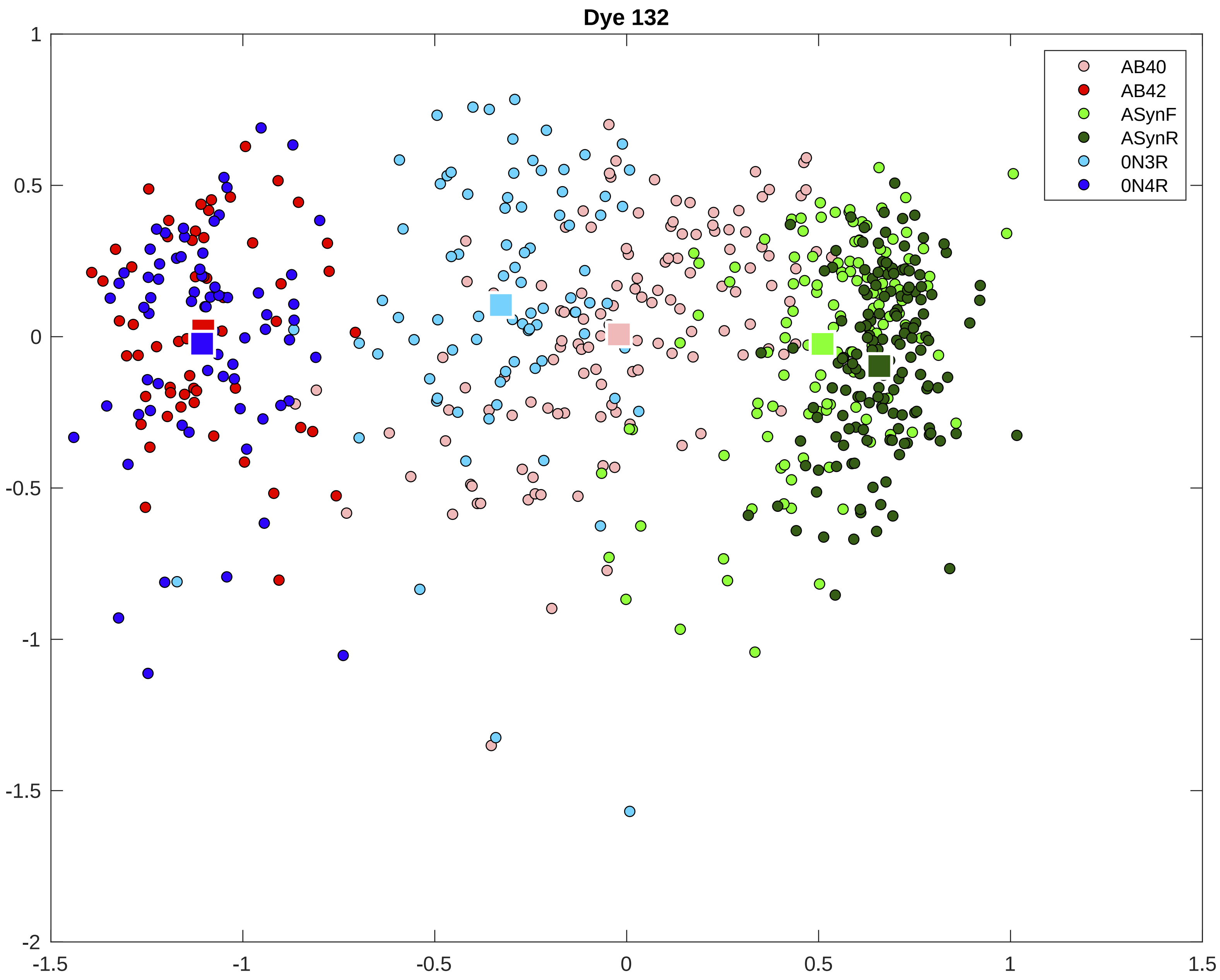

Dye 132
AB40
AB42
ASynF
ASynR
0N3R
0N4R
1
0.5
0
-0.5
-1
-1.5
-2
-1.5
-1
-0.5
0
0.5
1
1.5

Dye 132
Overall Discrimination score
0.60917

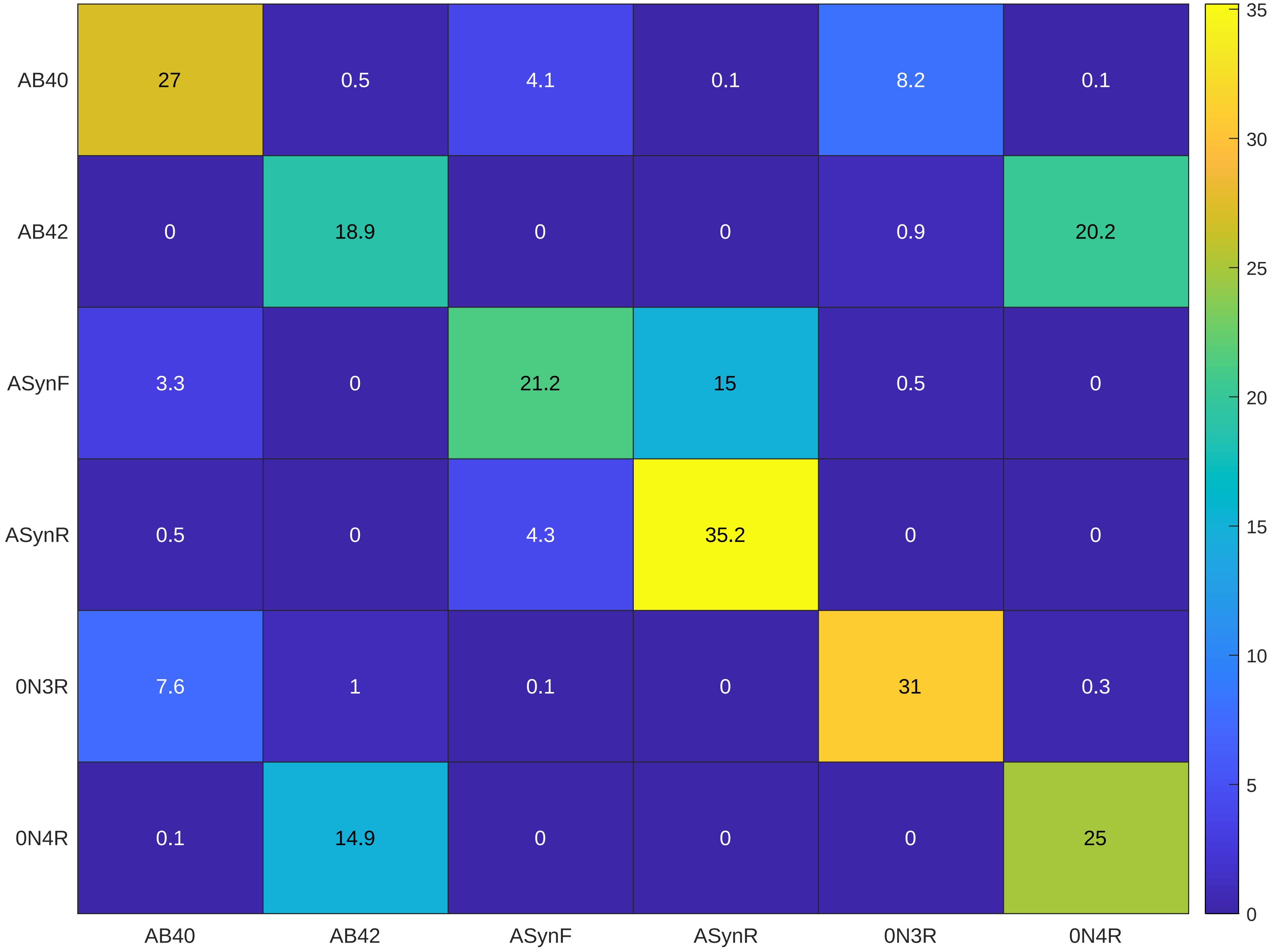

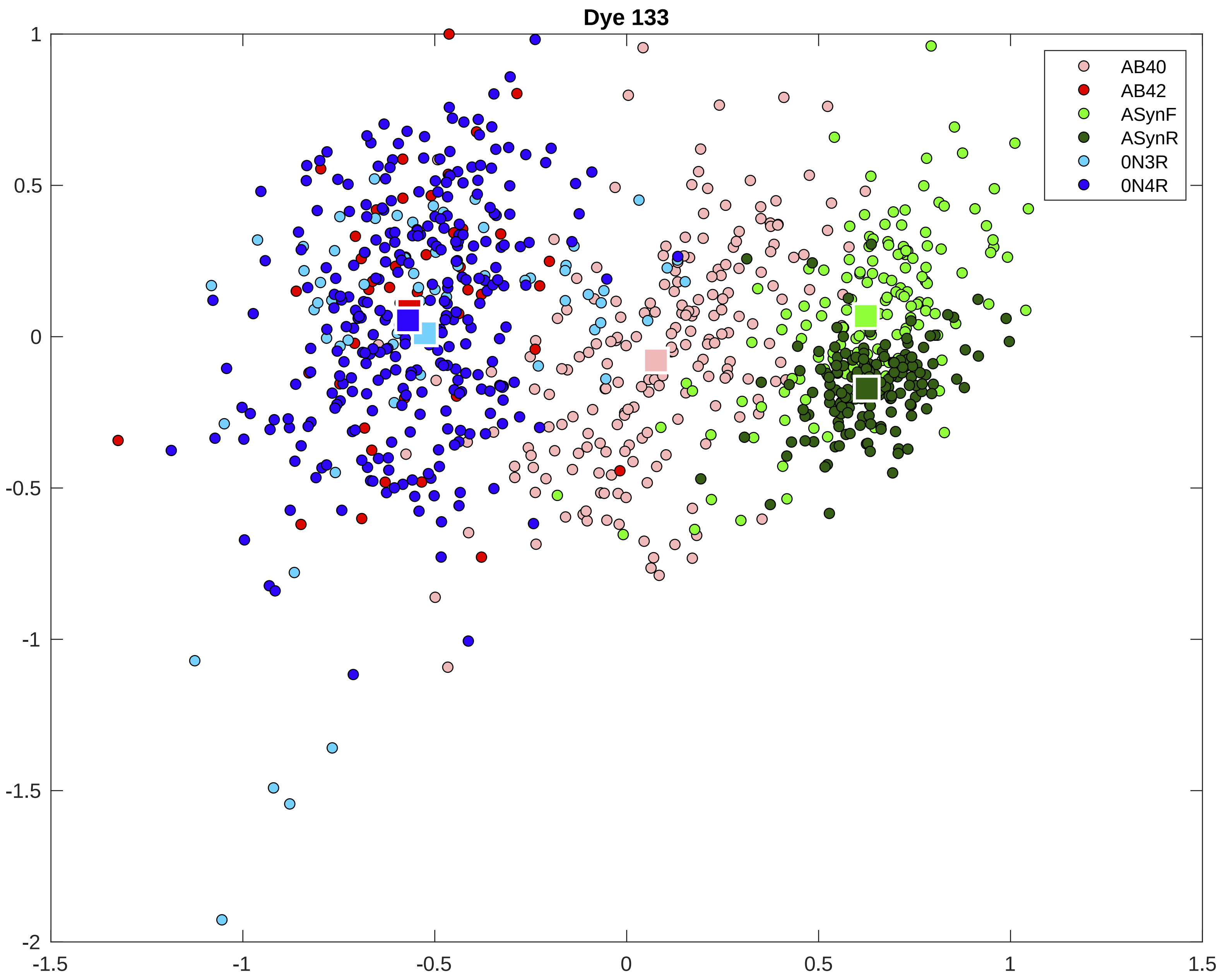

Dye 133
AB40
AB42
ASynF
ASynR
0N3R
0N4R
1
0.5
0
-0.5
-1
-1.5
-2
-1.5
-1
-0.5
0
0.5
1
1.5

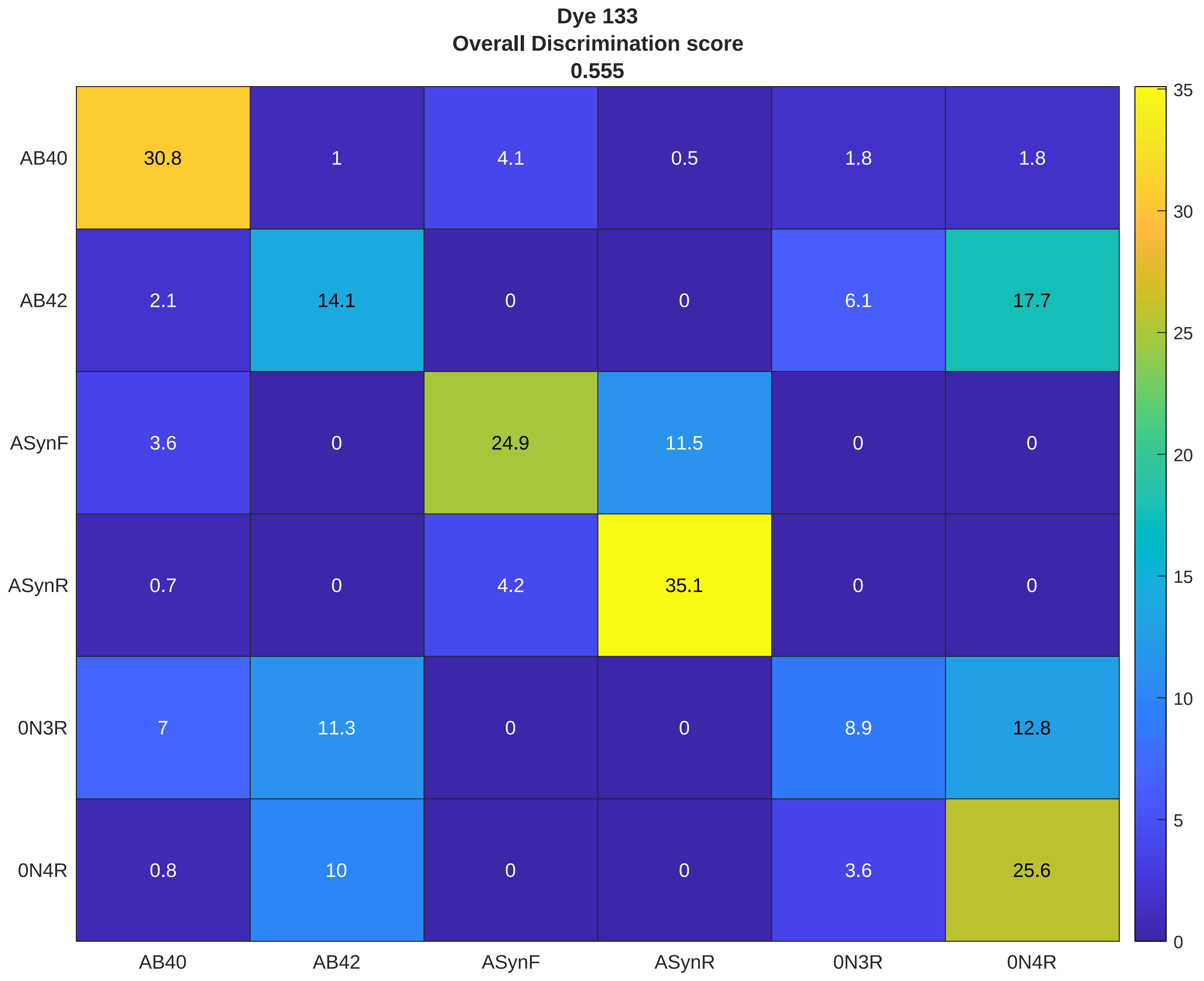
Dye 133
Overall Discrimination score
0.555
AB40
AB42
ASynF
ASynR
0N3R
0N4R
30.8
1
4.1
0.5
1.8
1.8
2.1
14.1
0
0
6.1
17.7
3.6
0
24.9
11.5
0
0
0.7
0
4.2
35.1
0
0
7
11.3
0
0
8.9
12.8
0.8
10
0
0
3.6
25.6
AB40
AB42
ASynF
ASynR
0N3R
0N4R
35
30
25
20
15
10
5
0

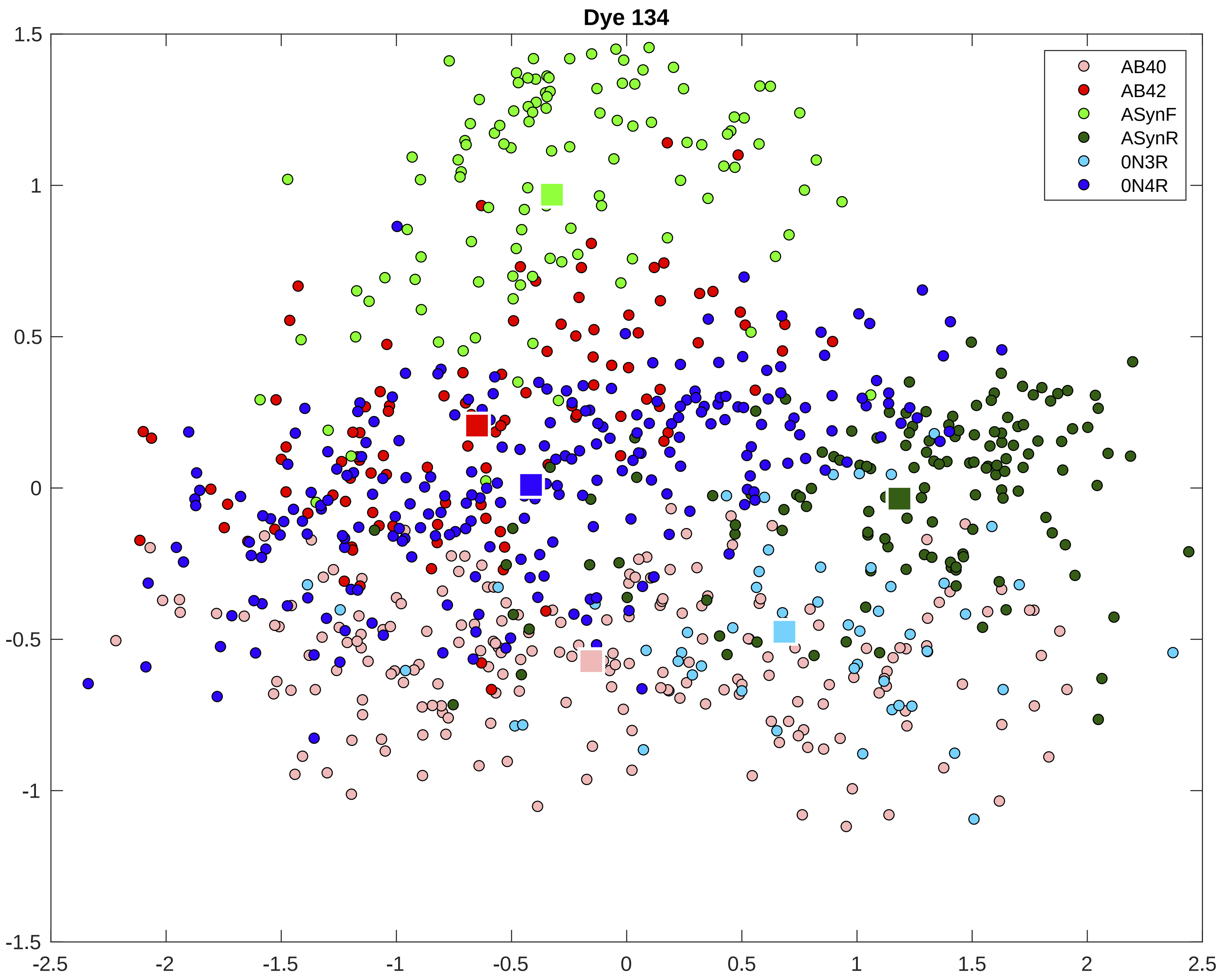

Dye 134
AB40
AB42
ASynF
ASynR
0N3R
0N4R
1.5
1
0.5
0
-0.5
-1
-1.5
-2.5
-2
-1.5
-1
-0.5
0
0.5
1
1.5
2
2.5

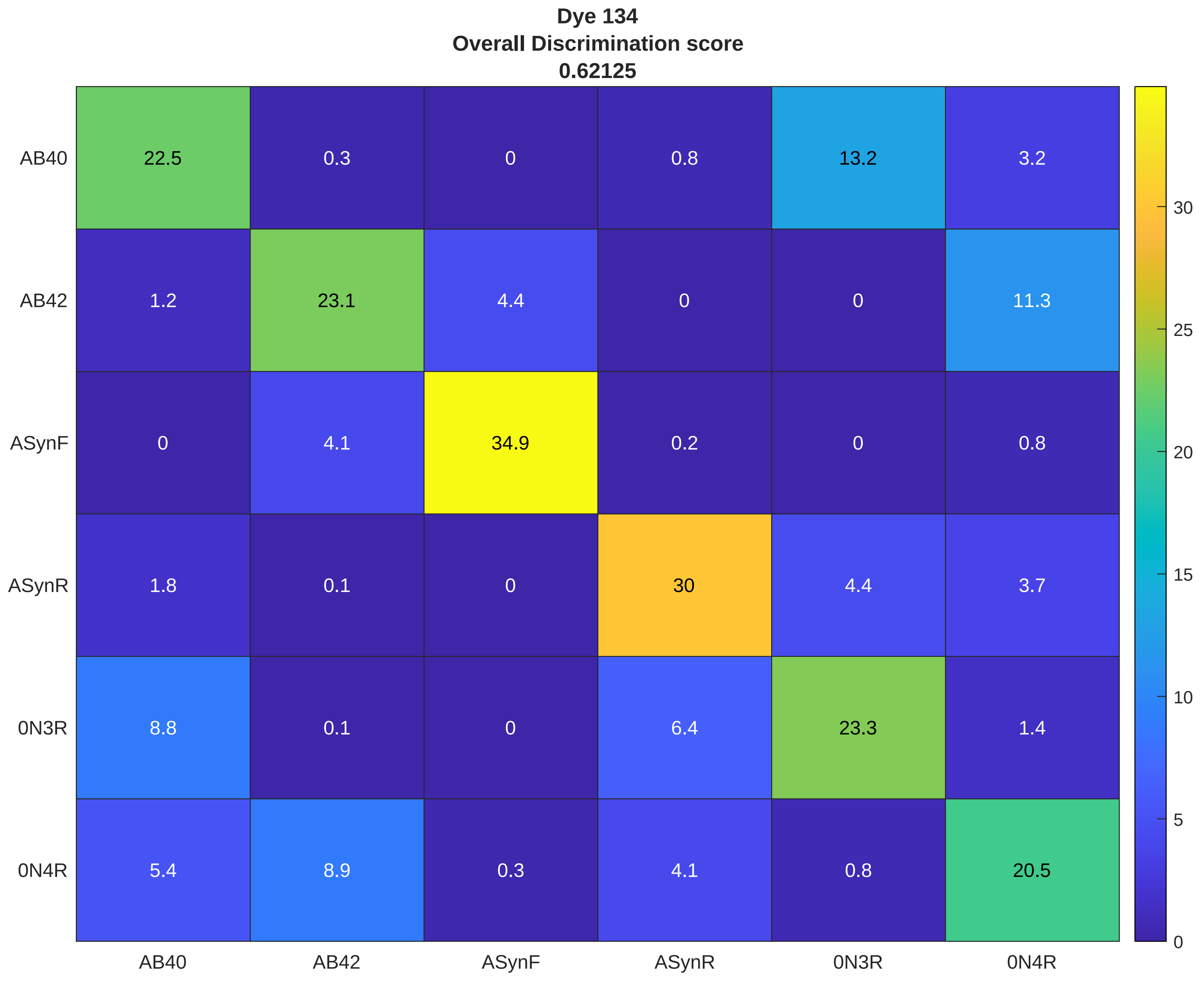

Dye 134
Overall Discrimination score
0.62125
AB40
AB42
ASynF
ASynR
0N3R
0N4R
22.5
0.3
0
0.8
13.2
3.2
1.2
23.1
4.4
0
0
11.3
0
4.1
34.9
0.2
0
0.8
1.8
0.1
0
30
4.4
3.7
8.8
0.1
0
6.4
23.3
1.4
5.4
8.9
0.3
4.1
0.8
20.5
30
25
20
15
10
5
0

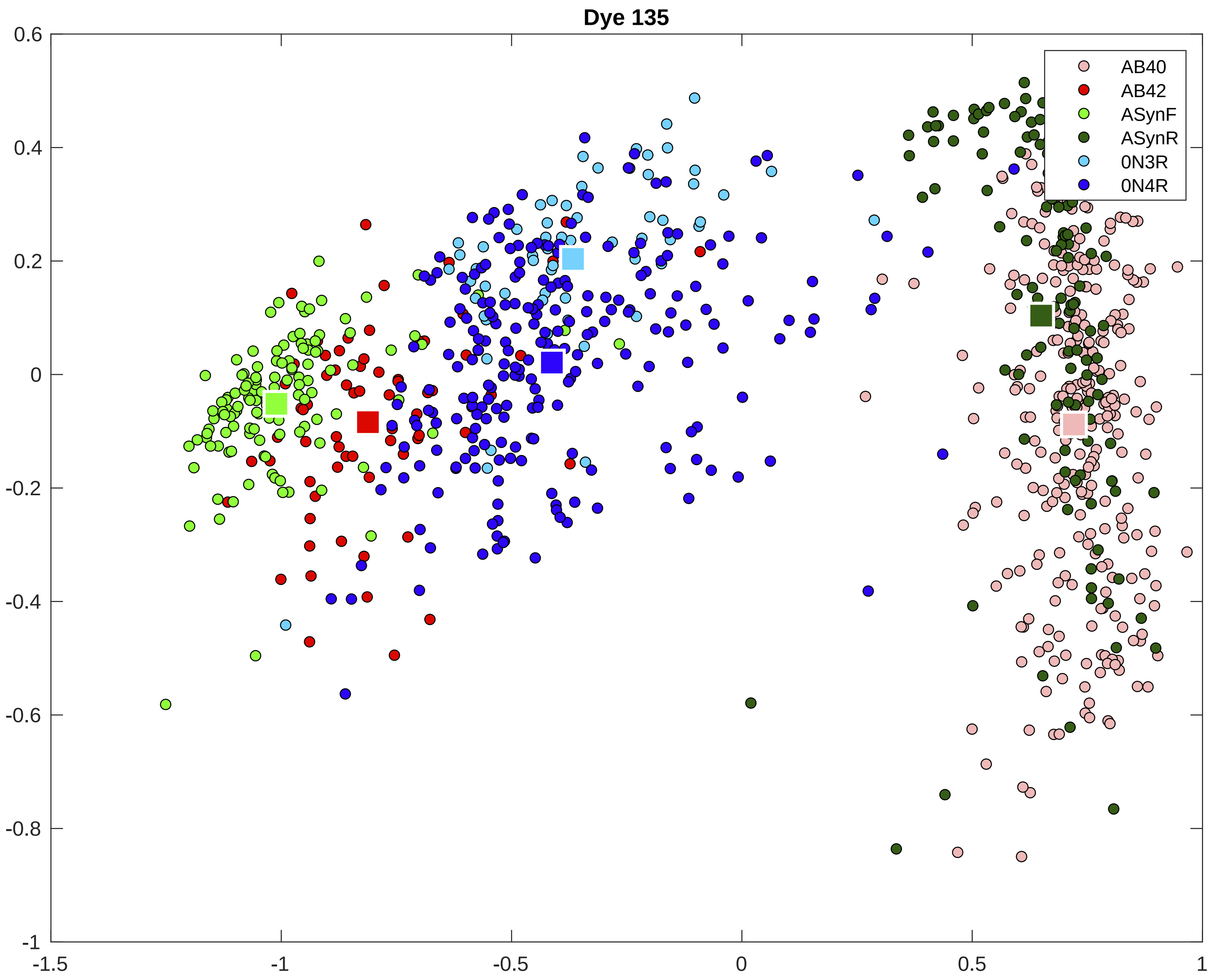

Dye 135
AB40
AB42
ASynF
ASynR
0N3R
0N4R
0.6
0.4
0.2
0
-0.2
-0.4
-0.6
-0.8
-1
-1.5
-1
-0.5
0
0.5
1

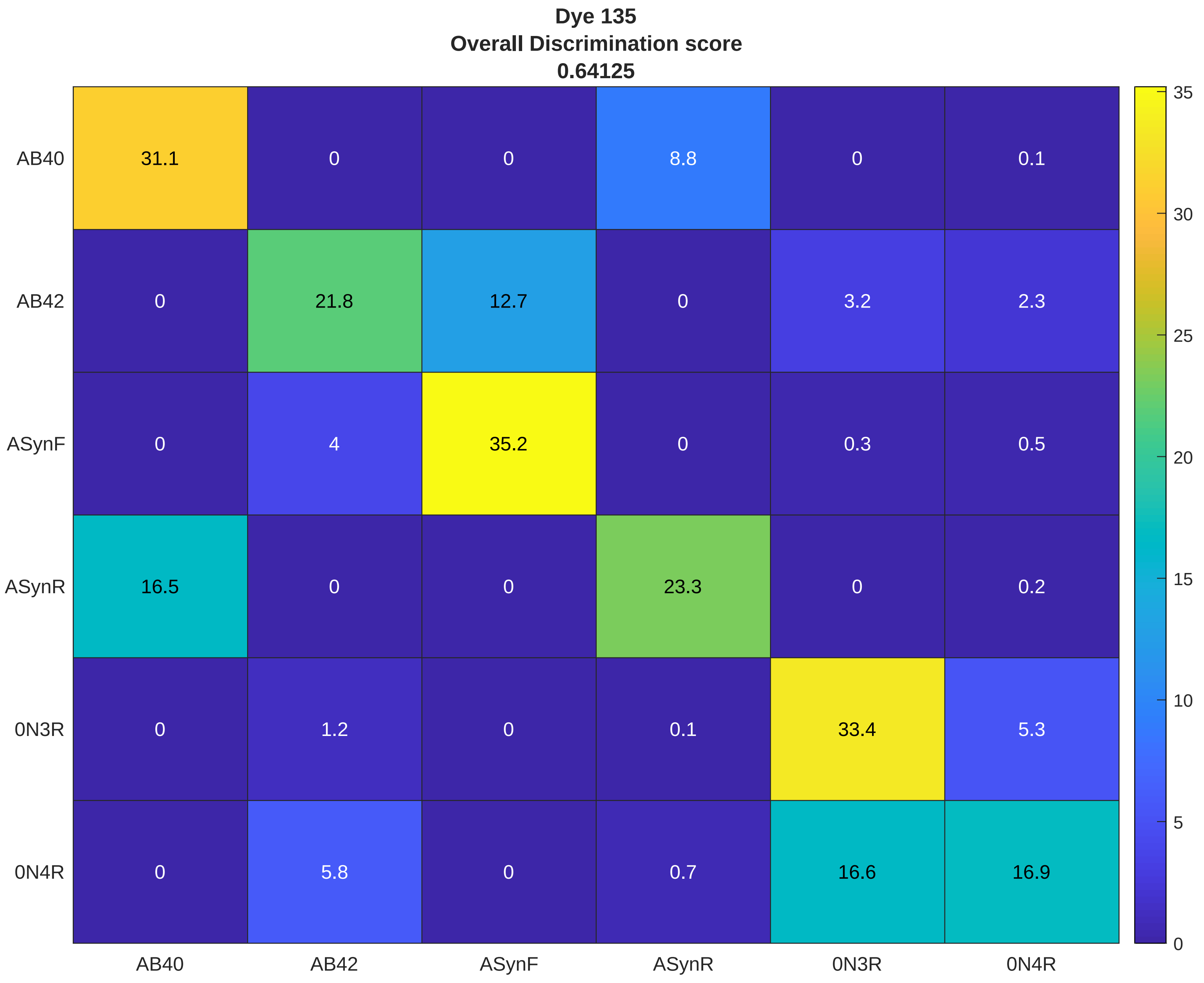
Dye 135
Overall Discrimination score
0.64125
AB40
AB42
ASynF
ASynR
0N3R
0N4R
31.1
0
0
8.8
0
0.1
0
21.8
12.7
0
3.2
2.3
0
4
35.2
0
0.3
0.5
16.5
0
0
23.3
0
0.2
0
1.2
0
0.1
33.4
5.3
0
5.8
0
0.7
16.6
16.9
35
30
25
20
15
10
5
0

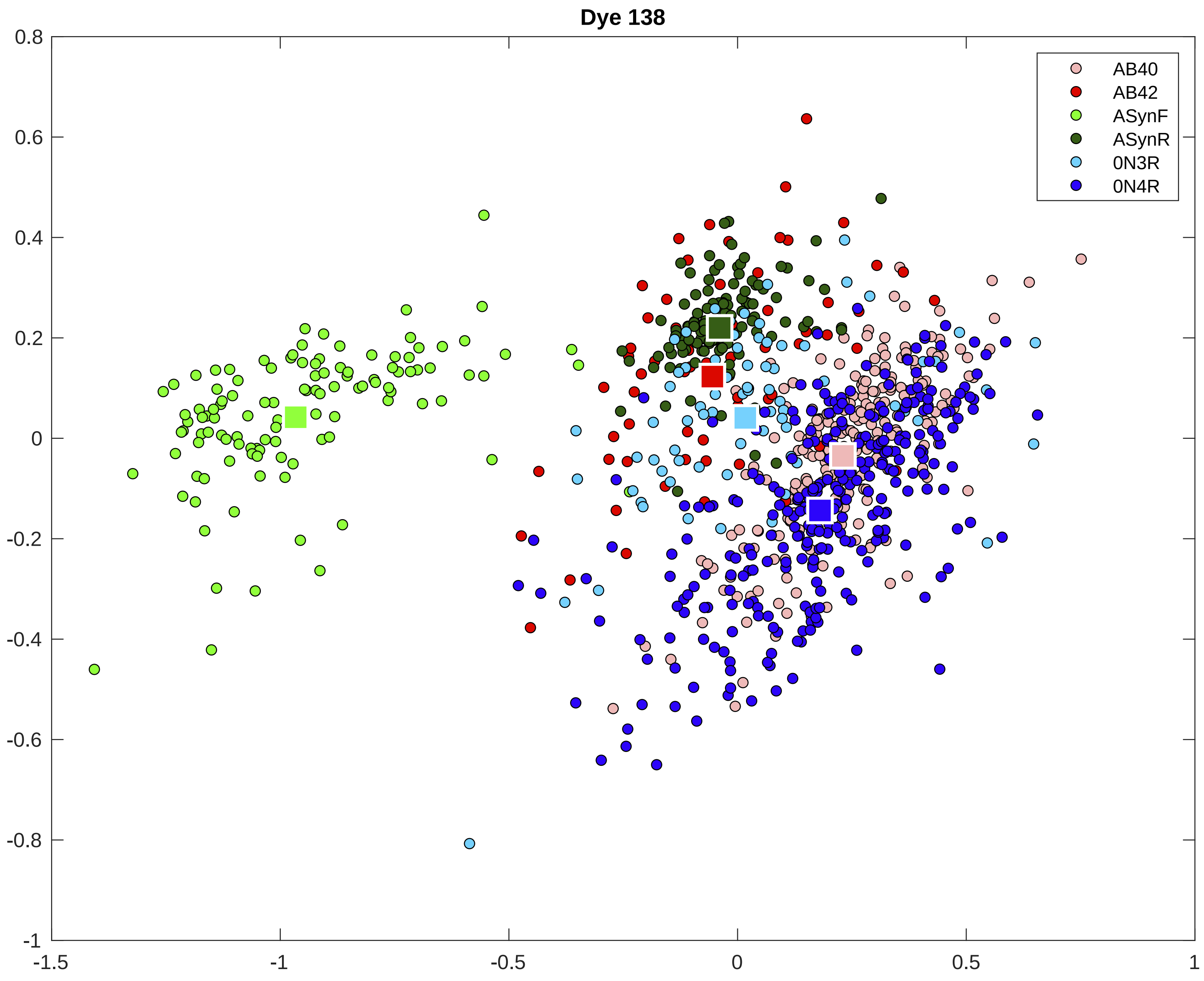

Dye 138
AB40
AB42
ASynF
ASynR
0N3R
0N4R
0.8
0.6
0.4
0.2
0
-0.2
-0.4
-0.6
-0.8
-1
-1.5
-1
-0.5
0
0.5
1

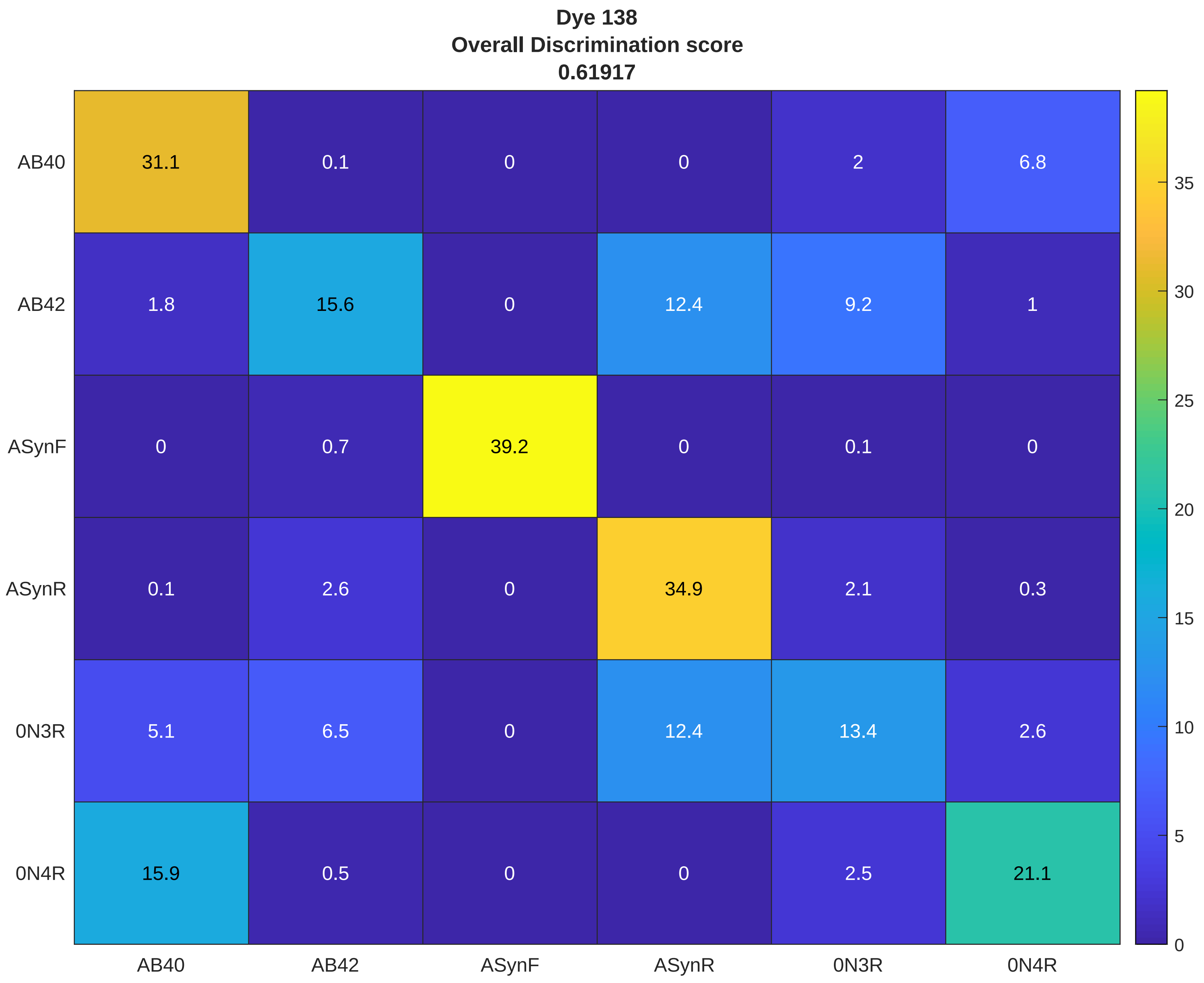
Dye 138
Overall Discrimination score
0.61917
AB40
AB42
ASynF
ASynR
0N3R
0N4R
31.1
0.1
0
0
2
6.8
1.8
15.6
0
12.4
9.2
1
0
0.7
39.2
0
0.1
0
0.1
2.6
0
34.9
2.1
0.3
5.1
6.5
0
12.4
13.4
2.6
15.9
0.5
0
0
2.5
21.1
AB40
AB42
ASynF
ASynR
0N3R
0N4R
35
30
25
20
15
10
5
0

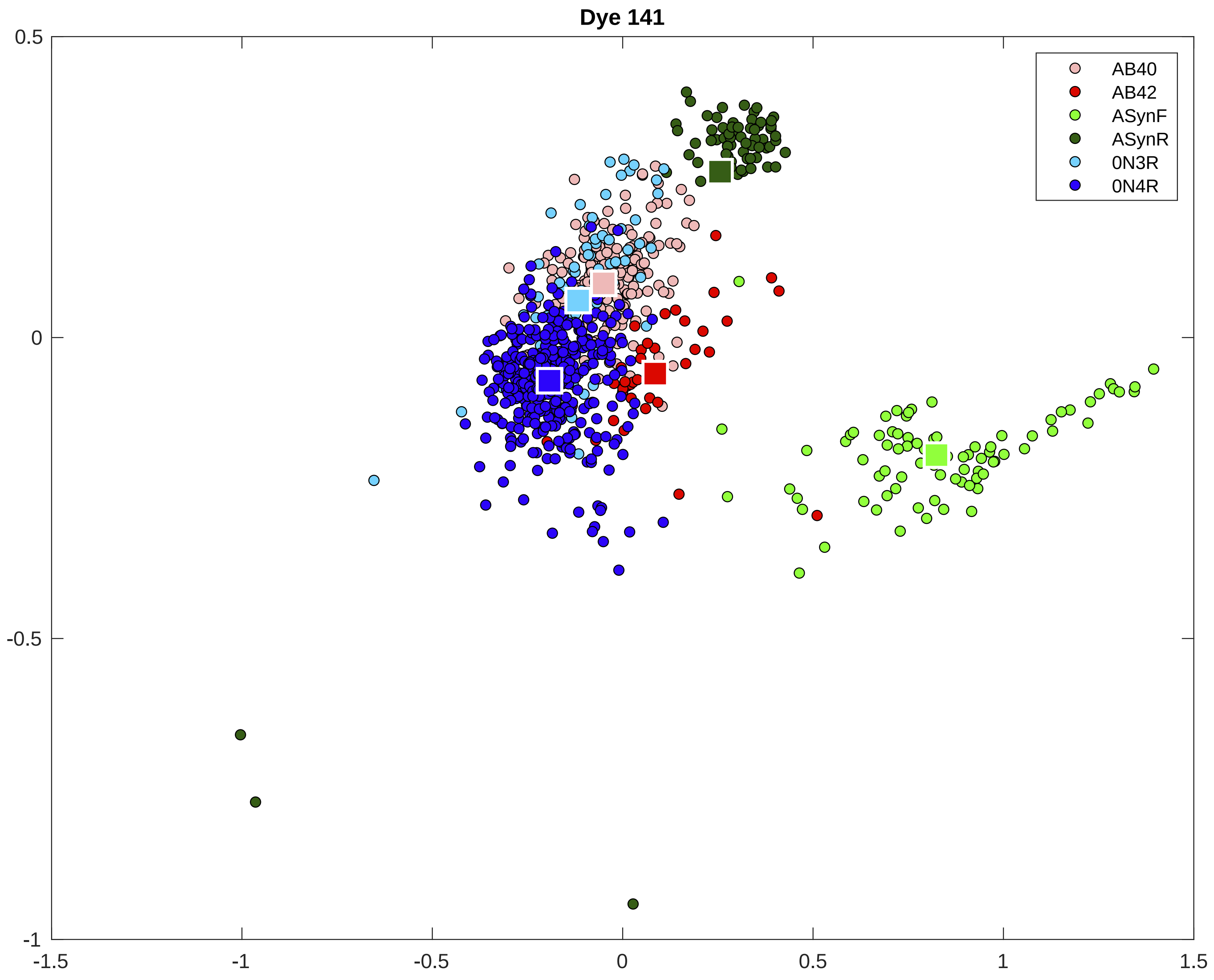
Dye 141
0.5
0
-0.5
-1
-1.5
-1
-0.5
0
0.5
1
1.5
AB40
AB42
ASynF
ASynR
0N3R
0N4R

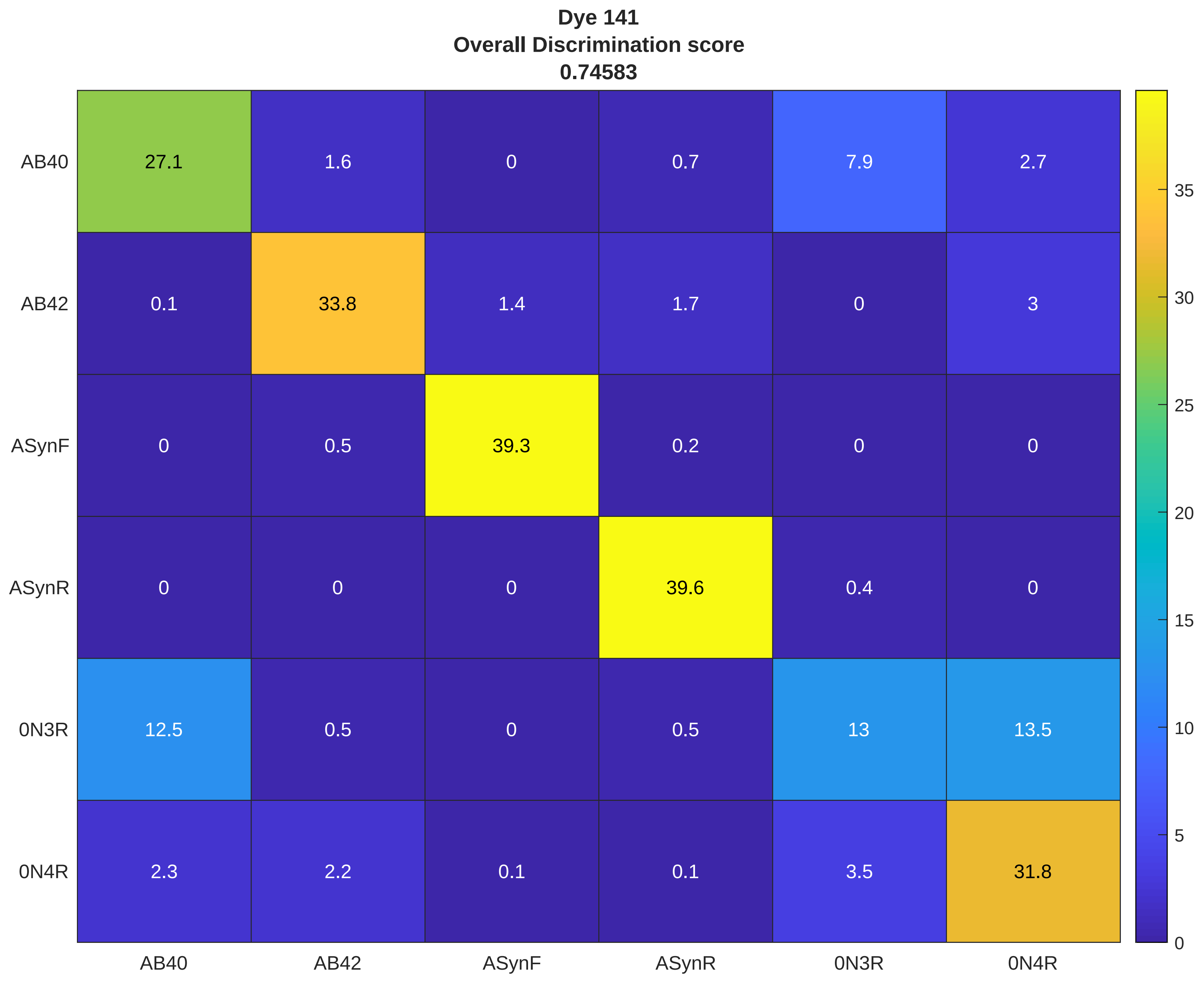

Dye 141
Overall Discrimination score
0.74583
AB40
AB42
ASynF
ASynR
0N3R
0N4R
27.1
1.6
0
0.7
7.9
2.7
0.1
33.8
1.4
1.7
0
3
0
0.5
39.3
0.2
0
0
0
0
0
39.6
0.4
0
12.5
0.5
0
0.5
13
13.5
2.3
2.2
0.1
0.1
3.5
31.8
AB40
AB42
ASynF
ASynR
0N3R
0N4R
35
30
25
20
15
10
5
0

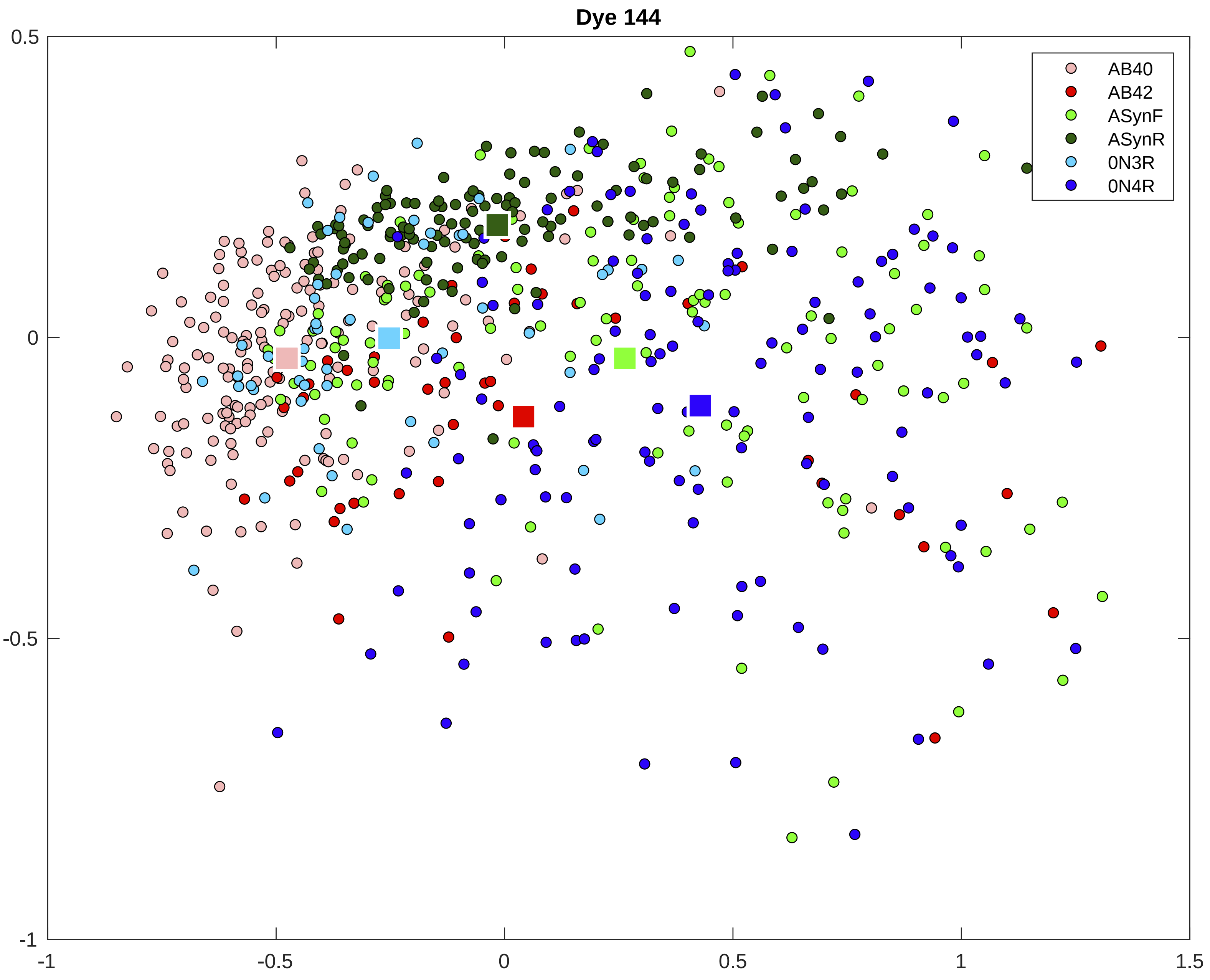

Dye 144
AB40
AB42
ASynF
ASynR
0N3R
0N4R
0.5
0
-0.5
-1
-1
-0.5
0
0.5
1
1.5

Dye 144
Overall Discrimination score
0.39625

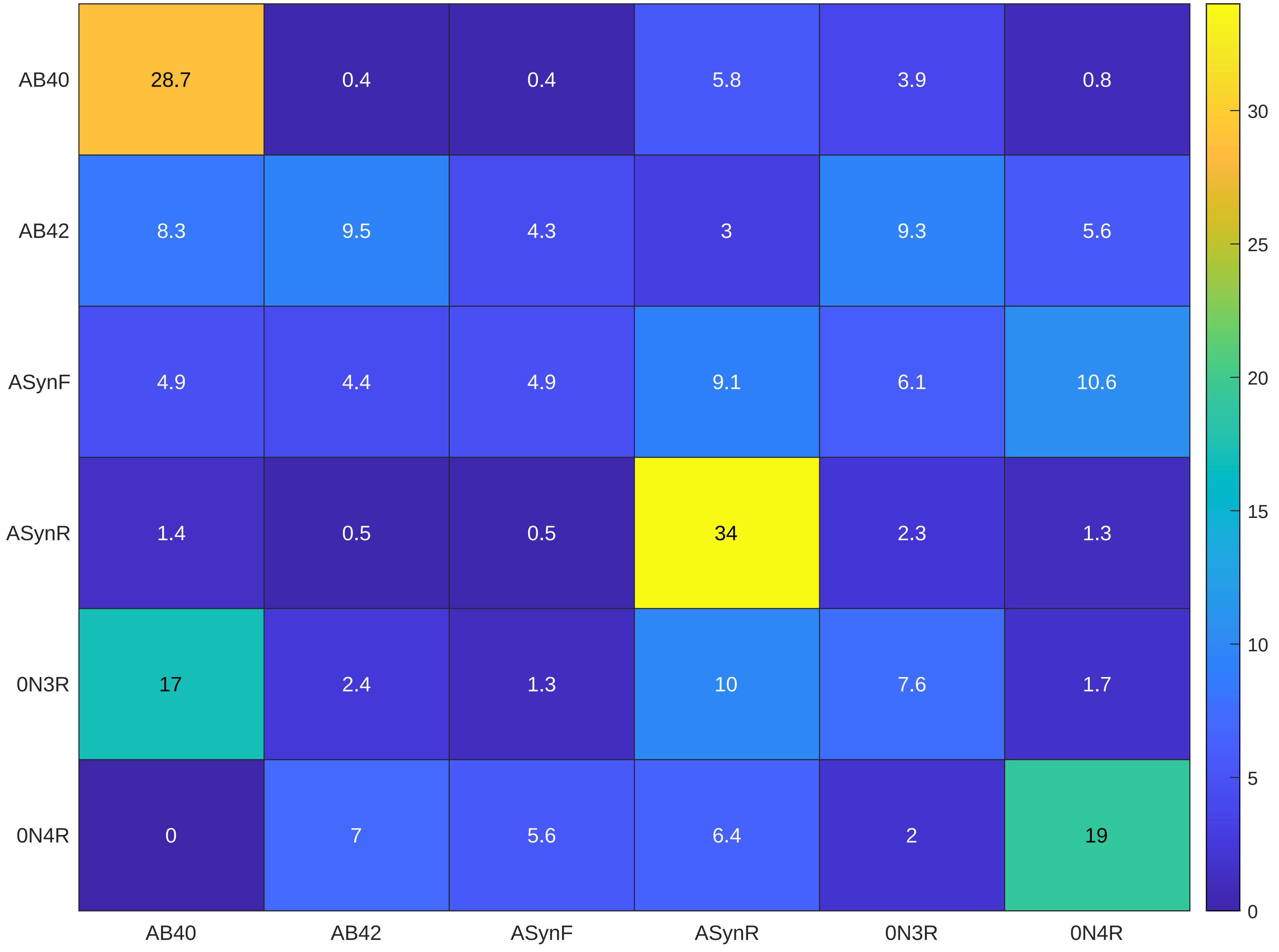

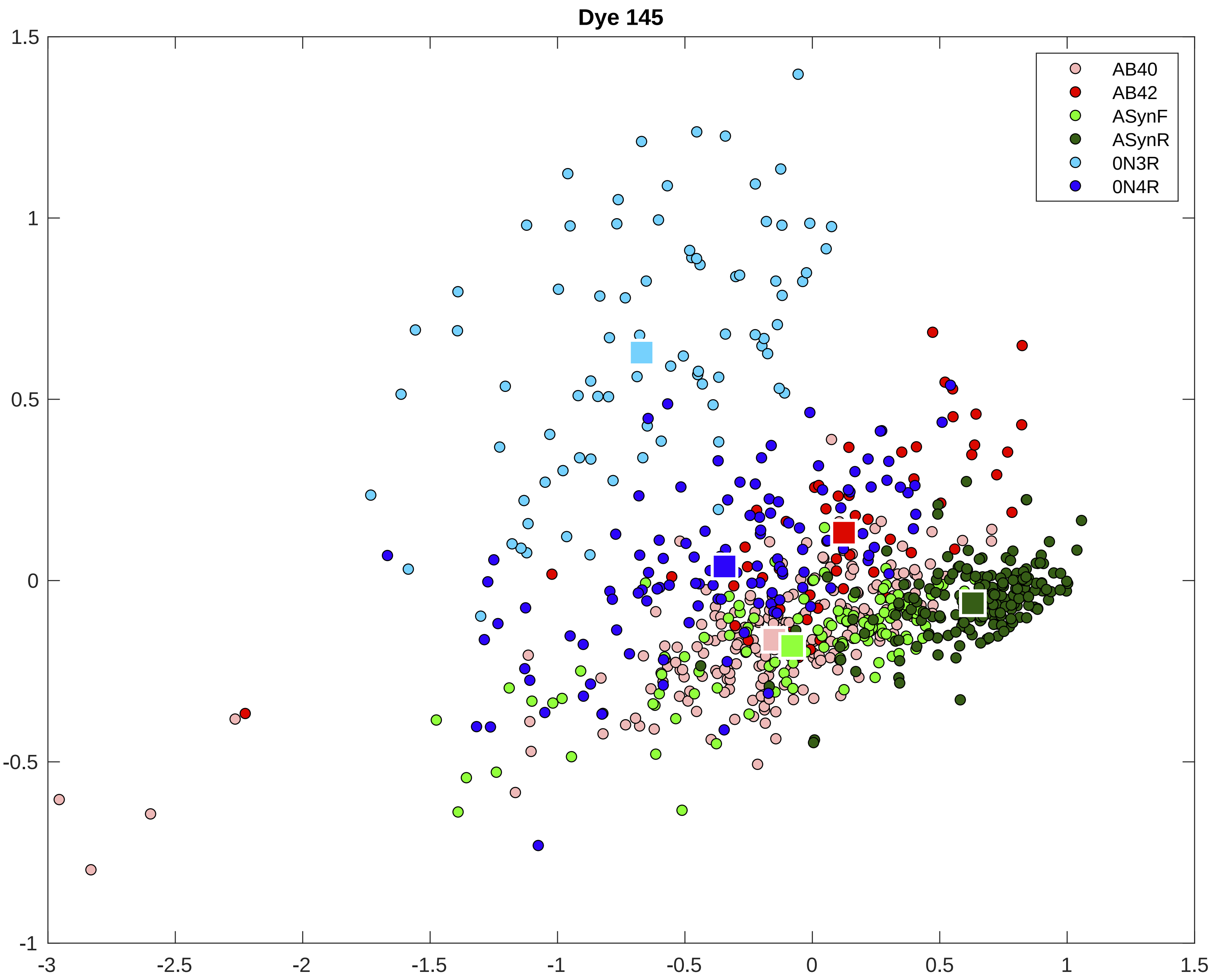

Dye 145
1.5
1
0.5
0
-0.5
-1
-3
-2.5
-2
-1.5
-1
-0.5
0
0.5
1
1.5
AB40
AB42
ASynF
ASynR
0N3R
0N4R

Dye 145
Overall Discrimination score
0.6025

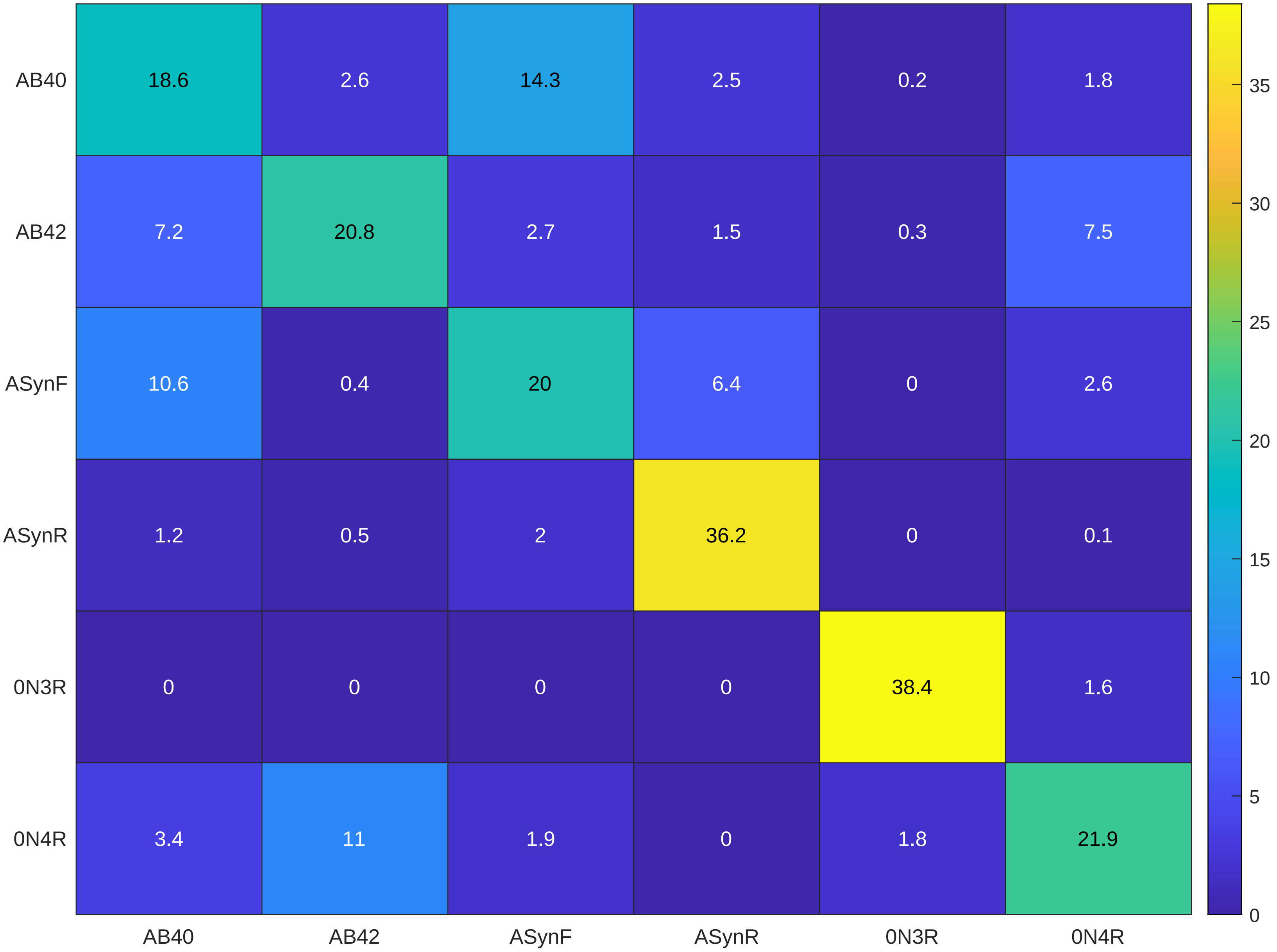

Supplement: Supplementary file 2 — Dataset S01 (PDF) [file pnas.2300769120.sd01.pdf]
