# Supplementary material for: EMBER multidimensional spectral microscopy enables quantitative determination of disease- and cell-specific amyloid strains
Source: Proc Natl Acad Sci U S A. 2023 Mar 16;120(12):e2300769120. doi: 10.1073/pnas.2300769120 (PMC10041141; doi:10.1073/pnas.2300769120)

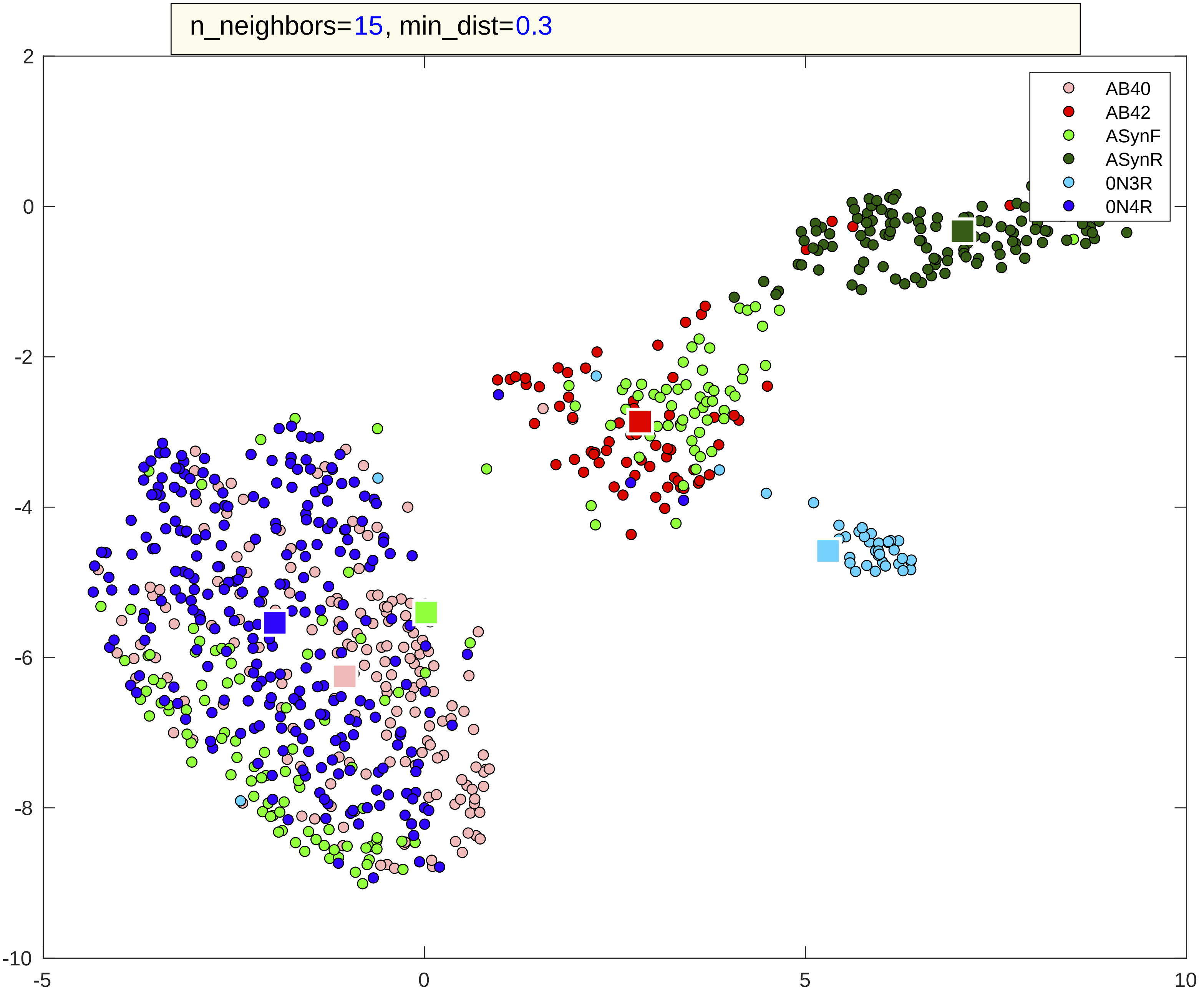

n_neighbors=15, min_dist=0.3
AB40
AB42
ASynF
ASynR
0N3R
0N4R
2
0
-2
-4
-6
-8
-10
-5
0
5
10

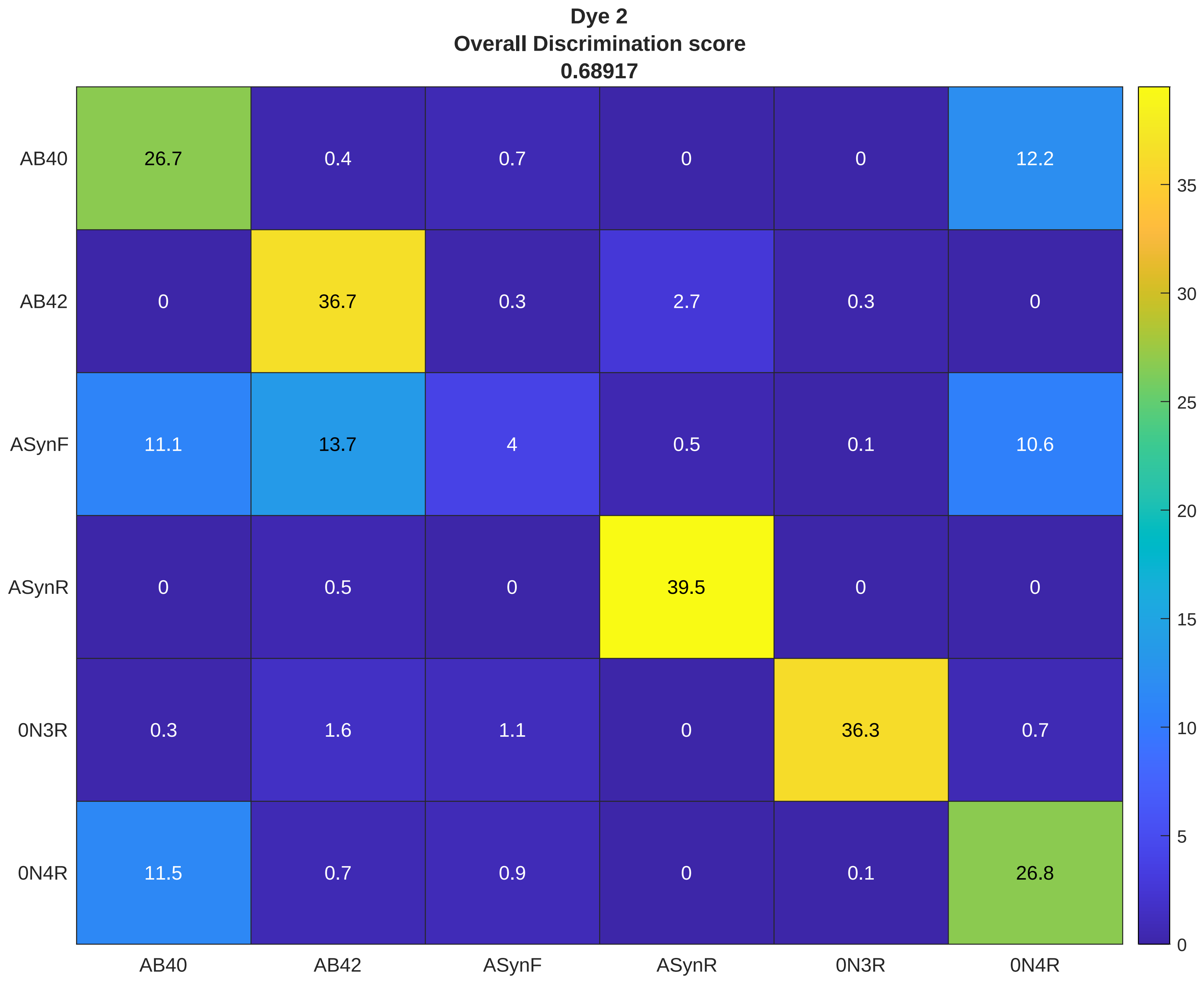
Dye 2
Overall Discrimination score
0.68917
AB40
AB42
ASynF
ASynR
0N3R
0N4R
26.7 0.4 0.7 0 0 12.2
0 36.7 0.3 2.7 0.3 0
11.1 13.7 4 0.5 0.1 10.6
0 0.5 0 39.5 0 0
0.3 1.6 1.1 0 36.3 0.7
11.5 0.7 0.9 0 0.1 26.8
AB40
AB42
ASynF
ASynR
0N3R
0N4R
35
30
25
20
15
10
5
0

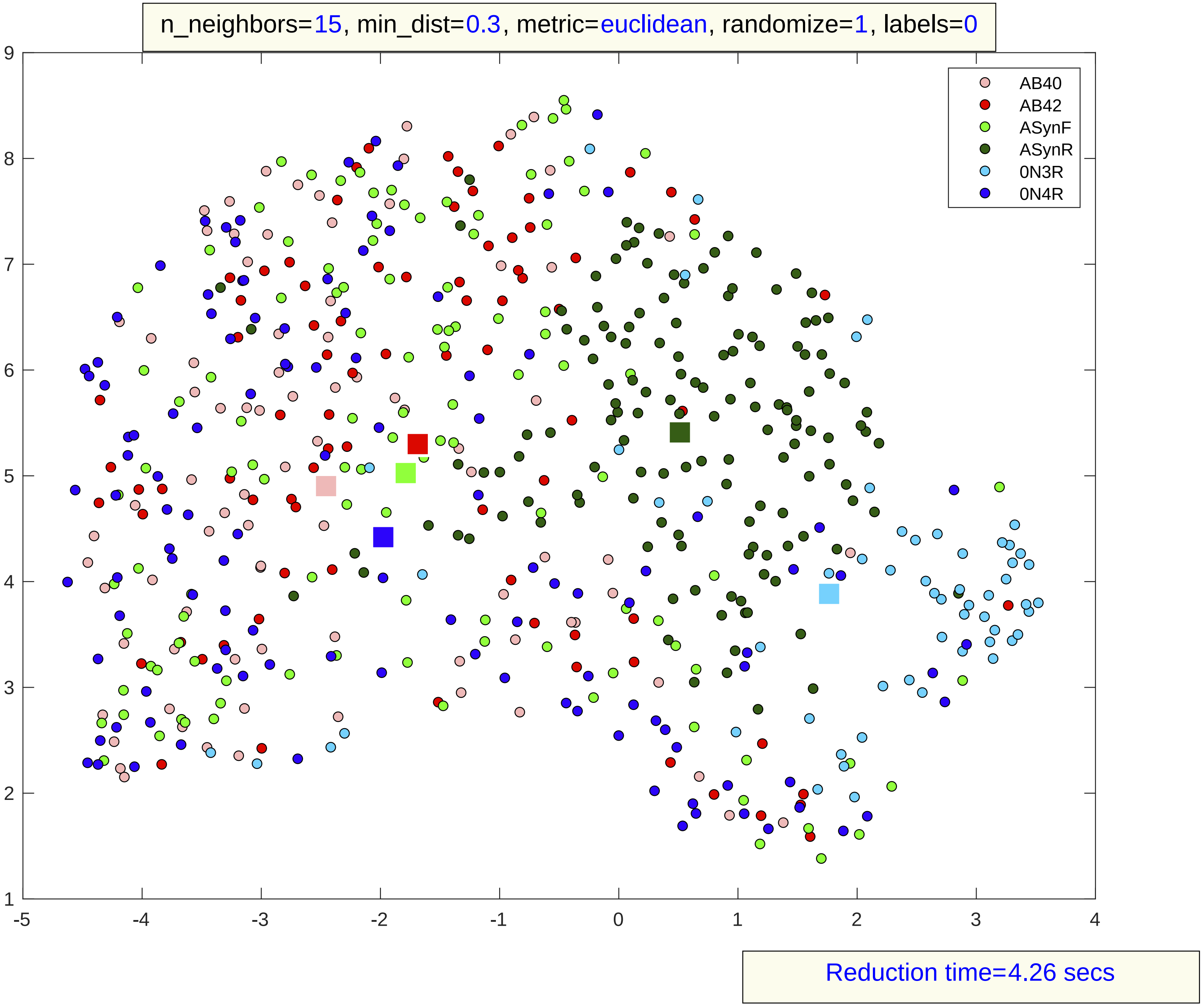
n_neighbors=15, min_dist=0.3, metric=euclidean, randomize=1, labels=0
AB40
AB42
ASynF
ASynR
0N3R
0N4R
9
8
7
6
5
4
3
2
1
-5
-4
-3
-2
-1
0
1
2
3
4
Reduction time=4.26 secs

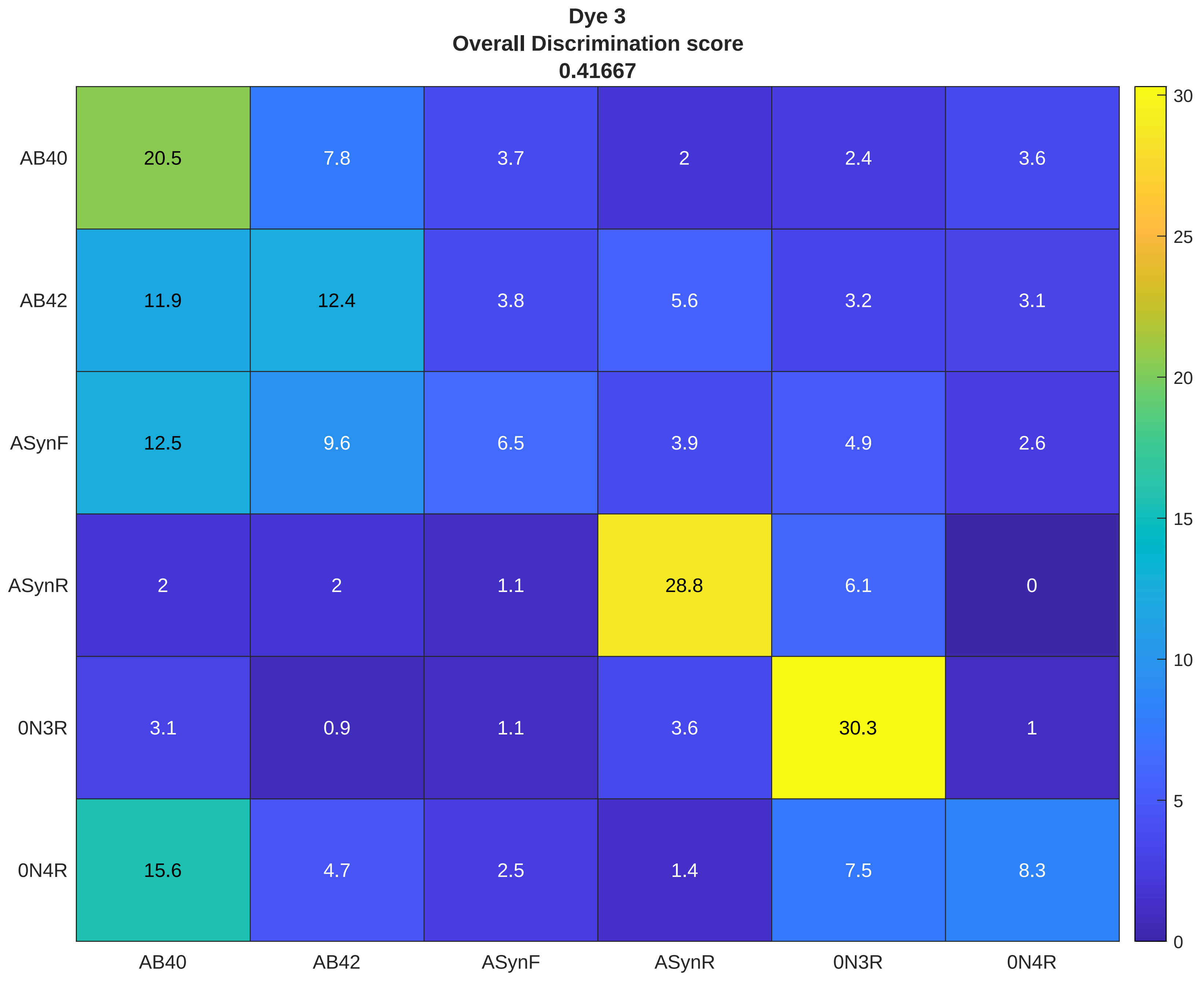
Dye 3
Overall Discrimination score
0.41667
AB40
AB42
ASynF
ASynR
0N3R
0N4R
20.5
7.8
3.7
2
2.4
3.6
11.9
12.4
3.8
5.6
3.2
3.1
12.5
9.6
6.5
3.9
4.9
2.6
2
2
1.1
28.8
6.1
0
3.1
0.9
1.1
3.6
30.3
1
15.6
4.7
2.5
1.4
7.5
8.3
AB40
AB42
ASynF
ASynR
0N3R
0N4R
30
25
20
15
10
5
0

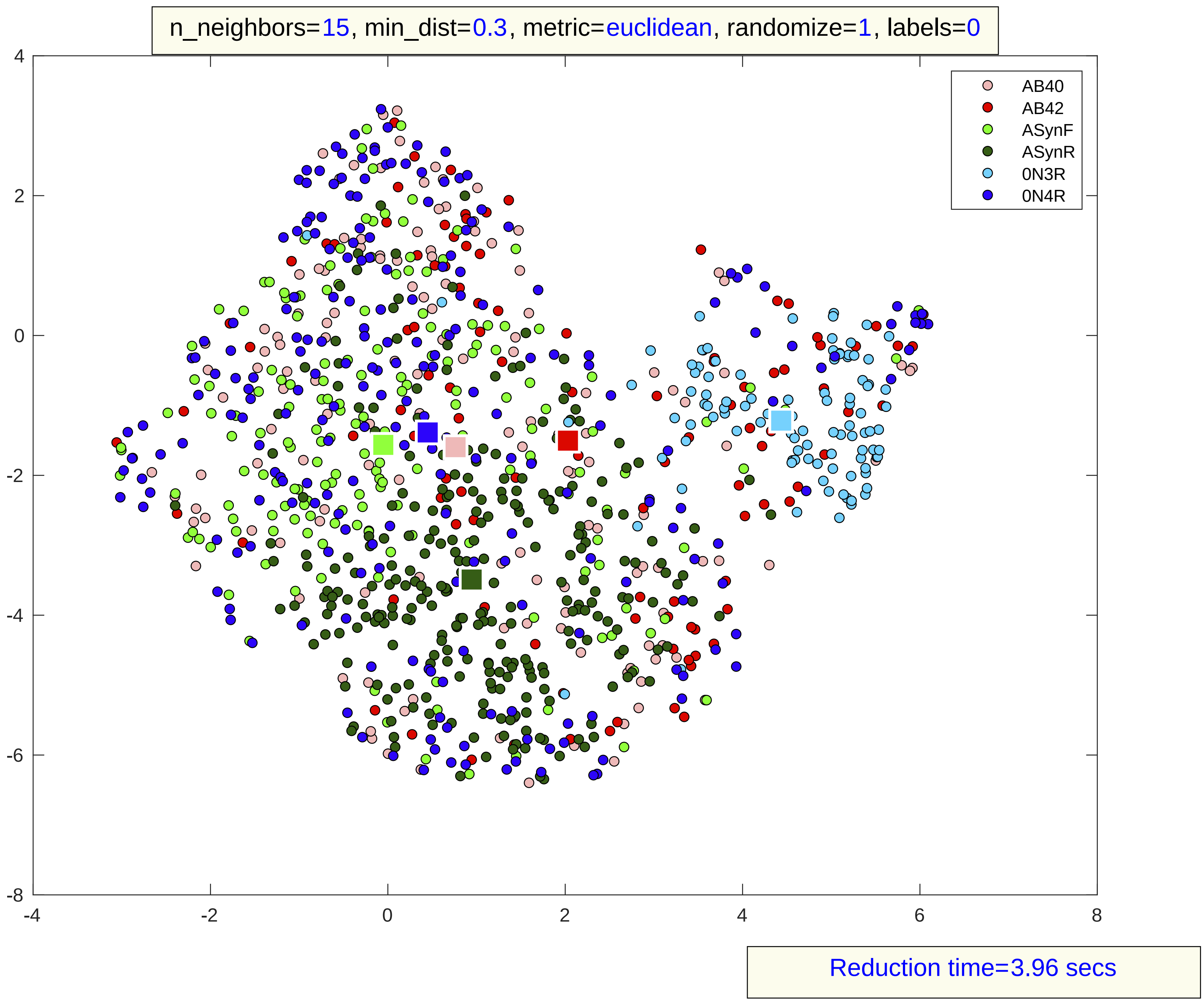

n_neighbors=15, min_dist=0.3, metric=euclidean, randomize=1, labels=0
AB40
AB42
ASynF
ASynR
0N3R
0N4R
4
2
0
-2
-4
-6
-8
-4
-2
0
2
4
6
8
Reduction time=3.96 secs

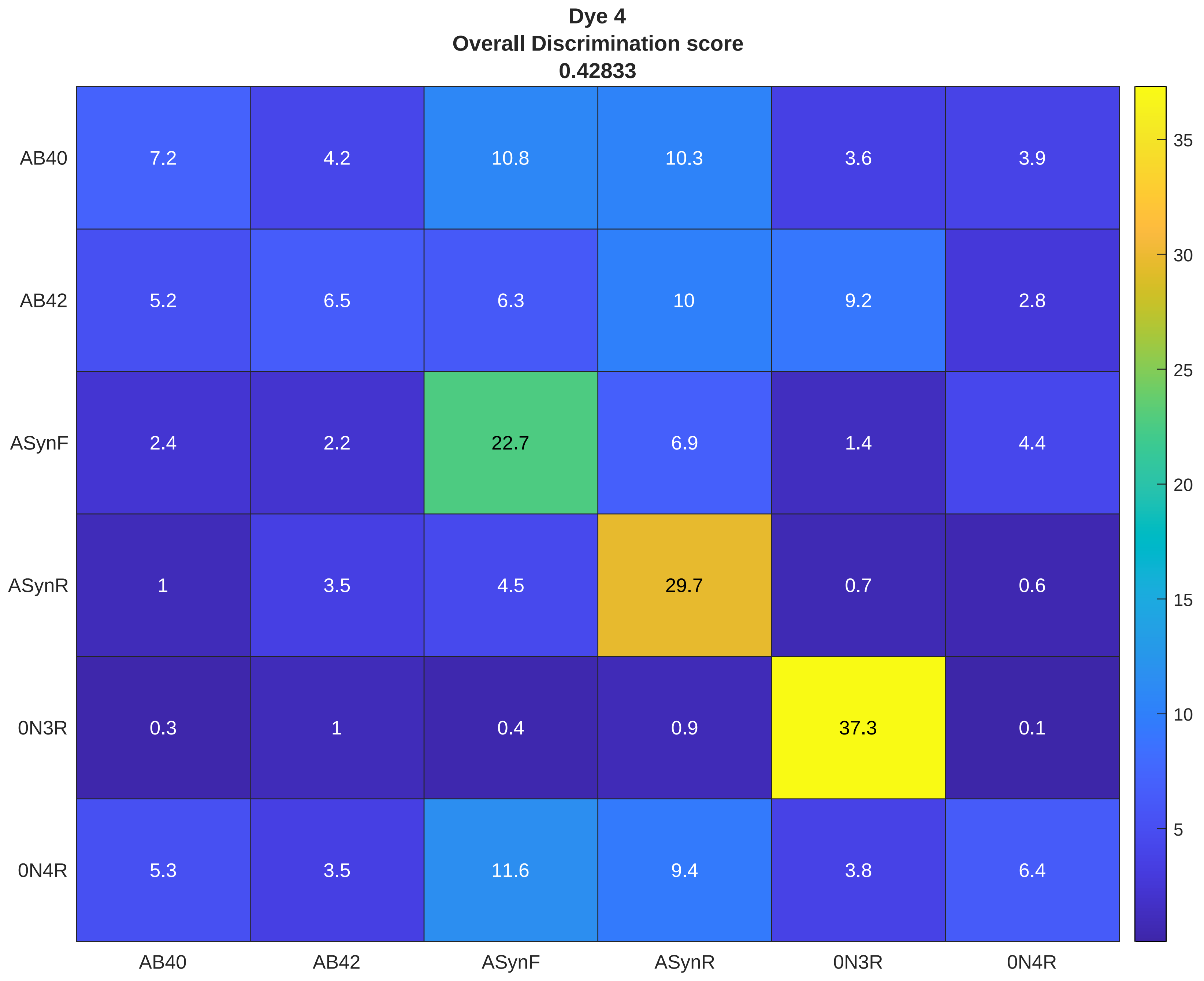

Dye 4
Overall Discrimination score
0.42833
AB40
AB42
ASynF
ASynR
0N3R
0N4R
7.2
4.2
10.8
10.3
3.6
3.9
5.2
6.5
6.3
10
9.2
2.8
2.4
2.2
22.7
6.9
1.4
4.4
1
3.5
4.5
29.7
0.7
0.6
0.3
1
0.4
0.9
37.3
0.1
5.3
3.5
11.6
9.4
3.8
6.4
AB40
AB42
ASynF
ASynR
0N3R
0N4R
35
30
25
20
15
10
5

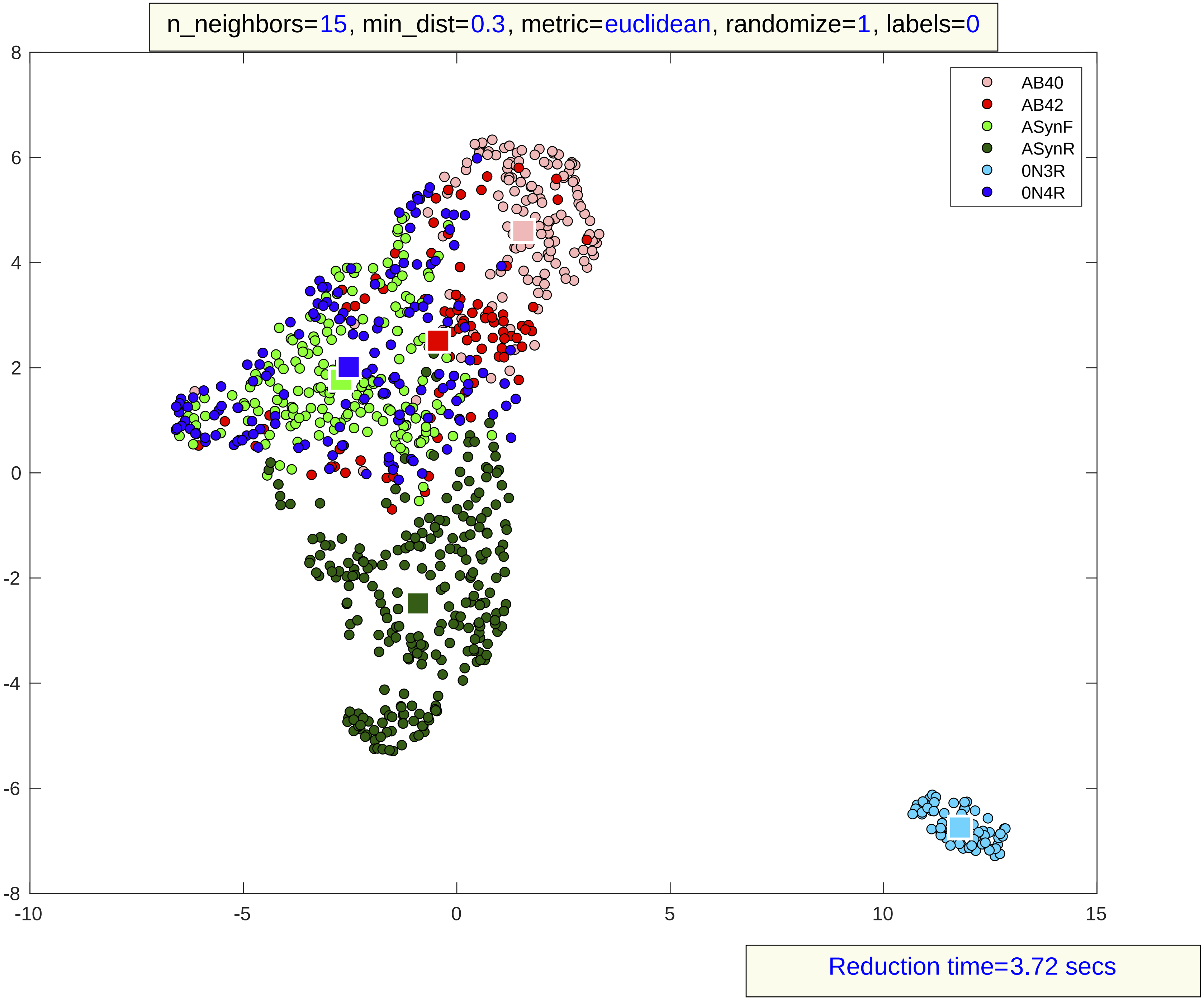
n_neighbors=15, min_dist=0.3, metric=euclidean, randomize=1, labels=0
AB40
AB42
ASynF
ASynR
0N3R
0N4R
8
6
4
2
0
-2
-4
-6
-8
-10
-5
0
5
10
15
Reduction time=3.72 secs

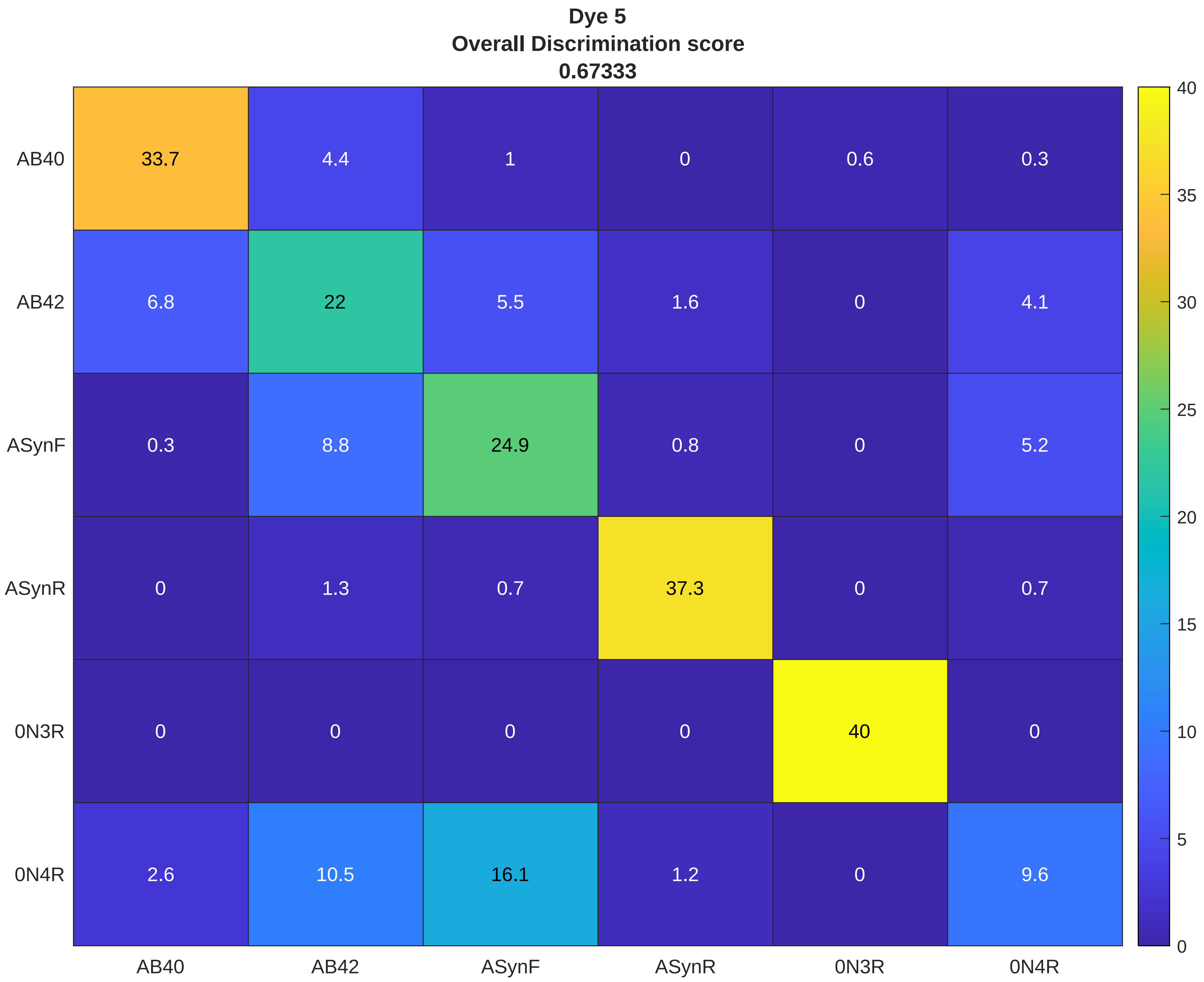

Dye 5
Overall Discrimination score
0.67333
AB40
AB42
ASynF
ASynR
0N3R
0N4R
33.7
4.4
1
0
0.6
0.3
6.8
22
5.5
1.6
0
4.1
0.3
8.8
24.9
0.8
0
5.2
0
1.3
0.7
37.3
0
0.7
0
0
0
0
40
0
2.6
10.5
16.1
1.2
0
9.6
AB40
AB42
ASynF
ASynR
0N3R
0N4R
40
35
30
25
20
15
10
5
0

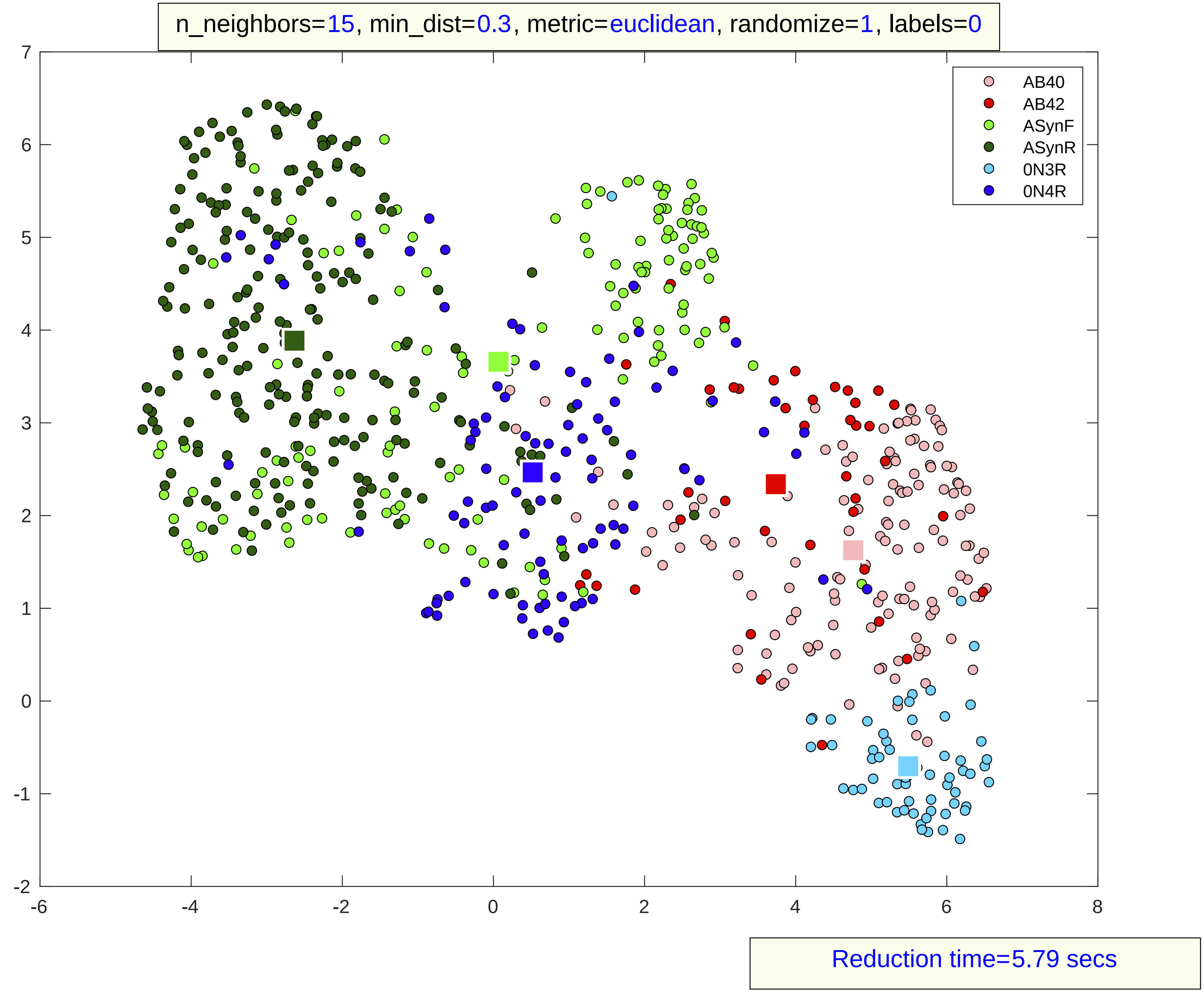

n_neighbors=15, min_dist=0.3, metric=euclidean, randomize=1, labels=0
AB40
AB42
ASynF
ASynR
0N3R
0N4R
7
6
5
4
3
2
1
0
-1
-2
-6
-4
-2
0
2
4
6
8
Reduction time=5.79 secs

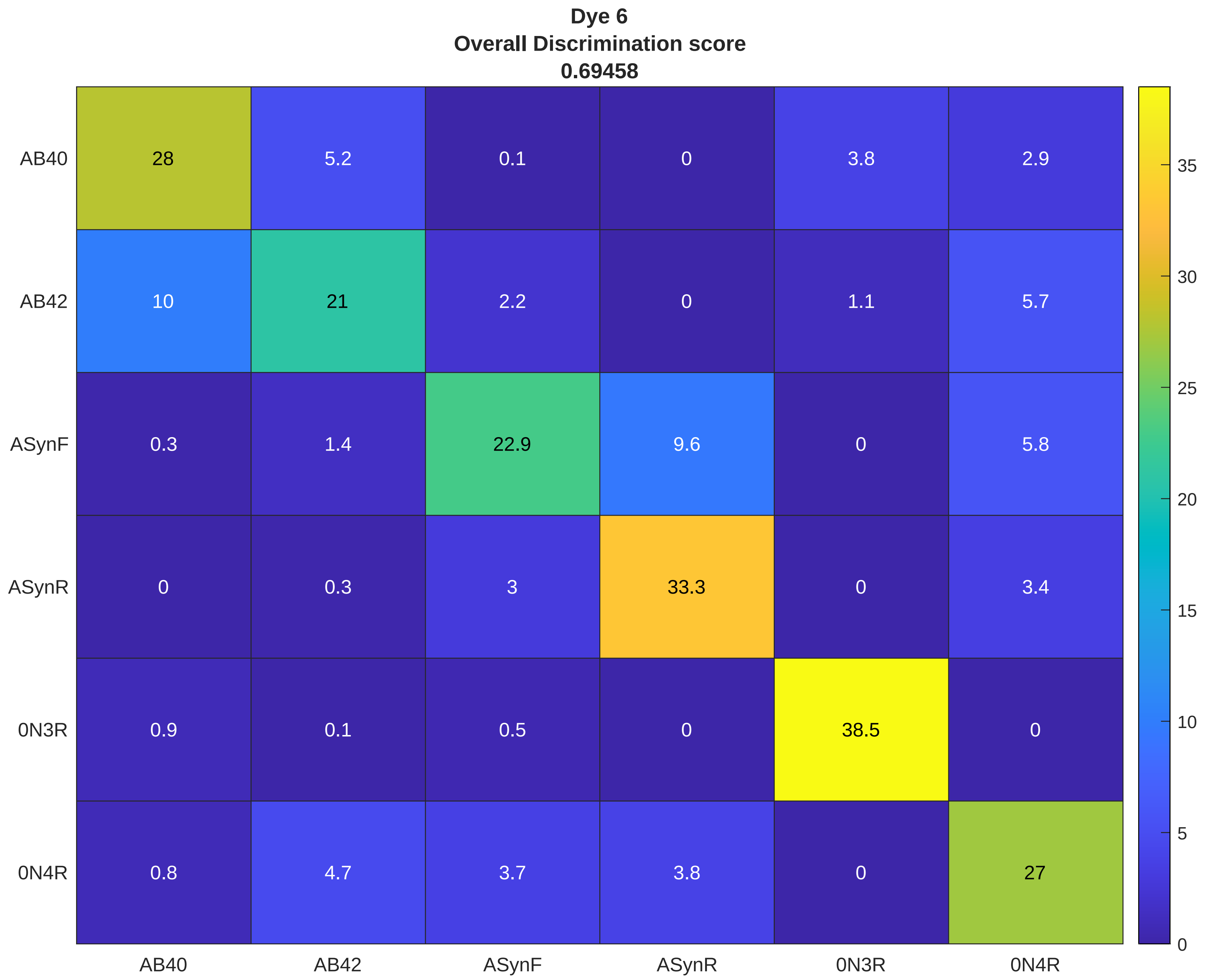
Dye 6
Overall Discrimination score
0.69458
AB40
AB42
ASynF
ASynR
0N3R
0N4R
28
5.2
0.1
0
3.8
2.9
10
21
2.2
0
1.1
5.7
0.3
1.4
22.9
9.6
0
5.8
0
0.3
3
33.3
0
3.4
0.9
0.1
0.5
0
38.5
0
0.8
4.7
3.7
3.8
0
27
AB40
AB42
ASynF
ASynR
0N3R
0N4R
35
30
25
20
15
10
5
0

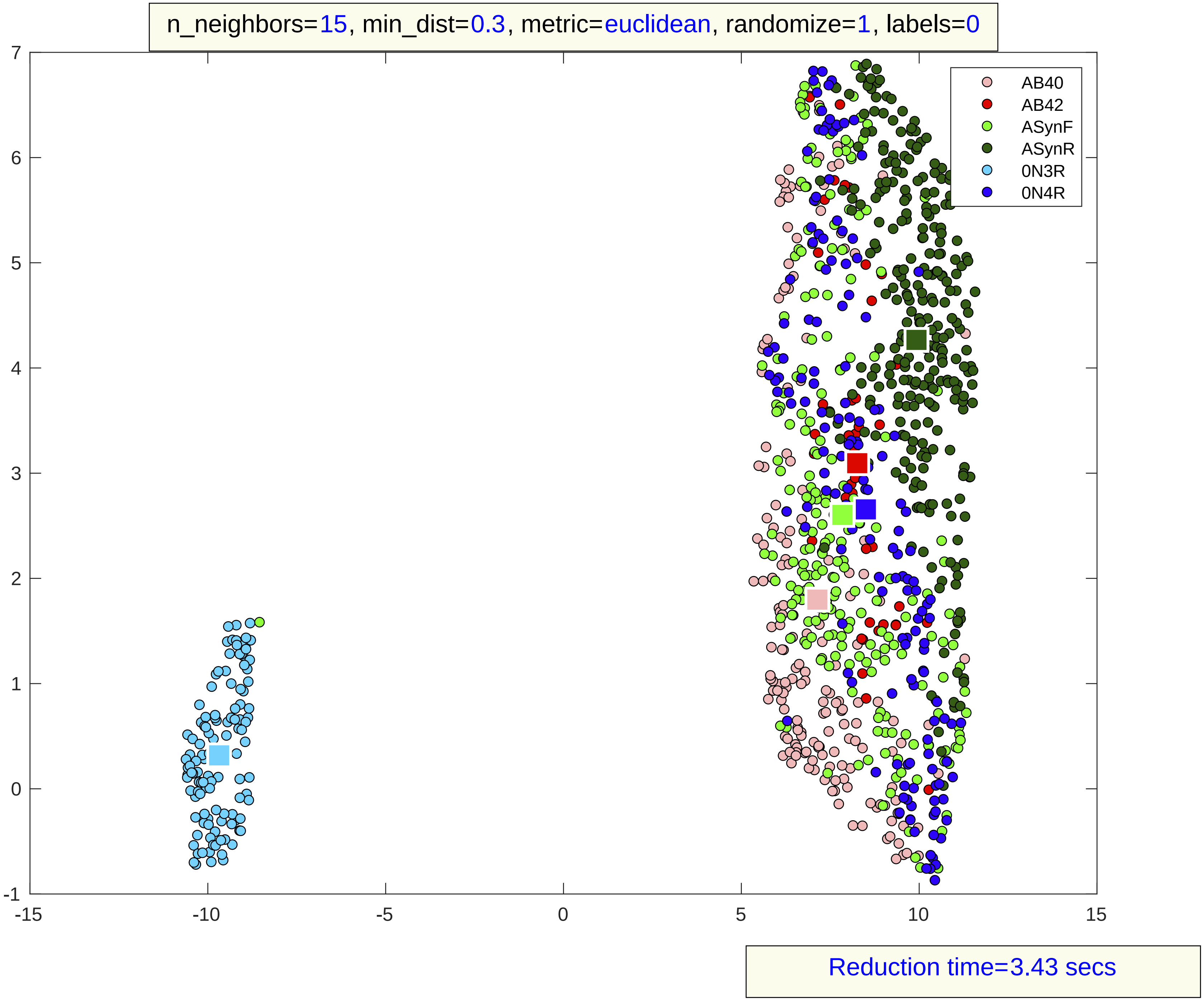
n_neighbors=15, min_dist=0.3, metric=euclidean, randomize=1, labels=0
AB40
AB42
ASynF
ASynR
0N3R
0N4R
7
6
5
4
3
2
1
0
-1
-15
-10
-5
0
5
10
15
Reduction time=3.43 secs

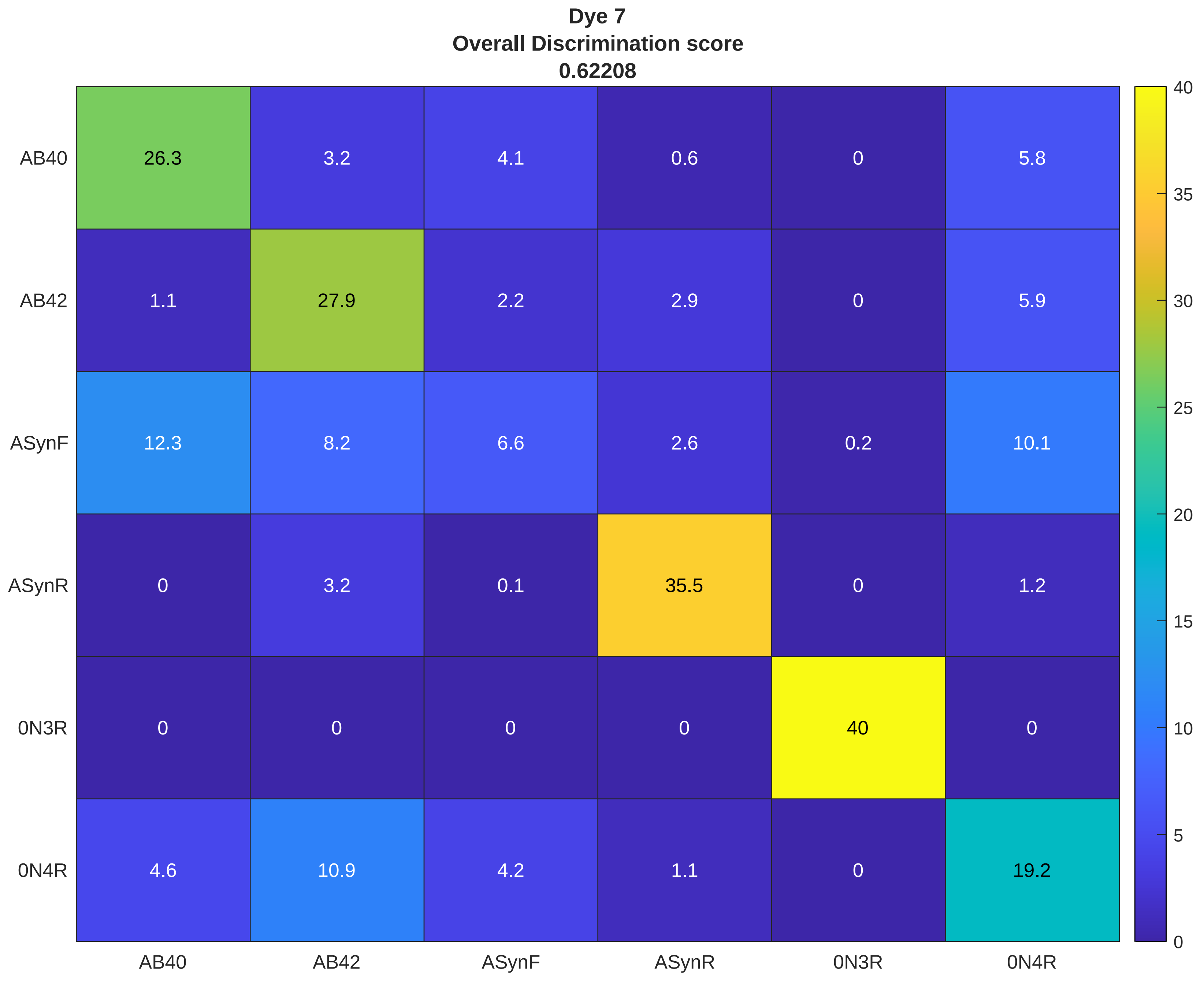

Dye 7
Overall Discrimination score
0.62208
AB40
26.3
3.2
4.1
0.6
0
5.8
AB42
1.1
27.9
2.2
2.9
0
5.9
ASynF
12.3
8.2
6.6
2.6
0.2
10.1
ASynR
0
3.2
0.1
35.5
0
1.2
0N3R
0
0
0
0
40
0
0N4R
4.6
10.9
4.2
1.1
0
19.2
AB40
AB42
ASynF
ASynR
0N3R
0N4R
40
35
30
25
20
15
10
5
0

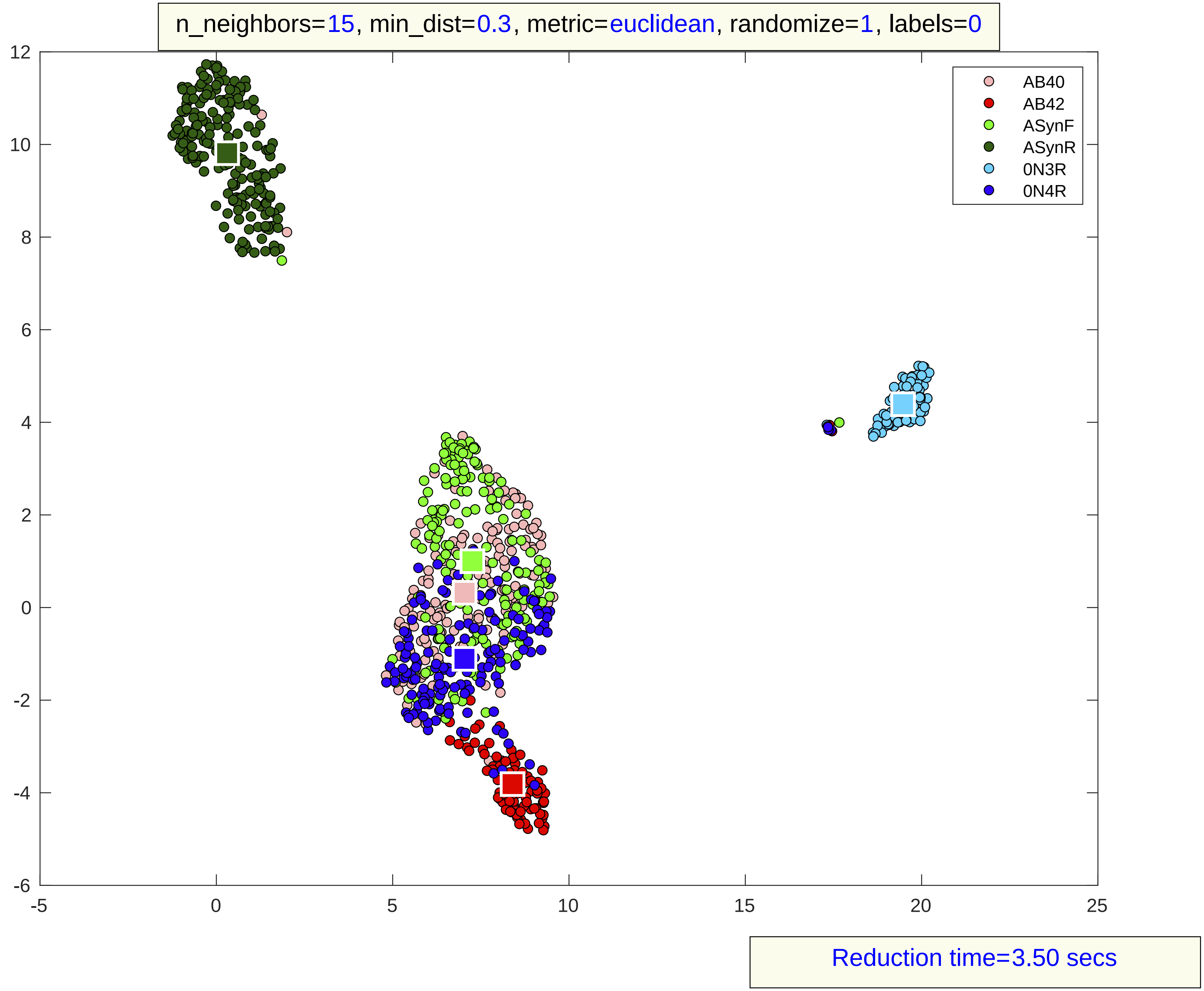

n_neighbors=15, min_dist=0.3, metric=euclidean, randomize=1, labels=0
AB40
AB42
ASynF
ASynR
0N3R
0N4R
12
10
8
6
4
2
0
-2
-4
-6
-5
0
5
10
15
20
25
Reduction time=3.50 secs

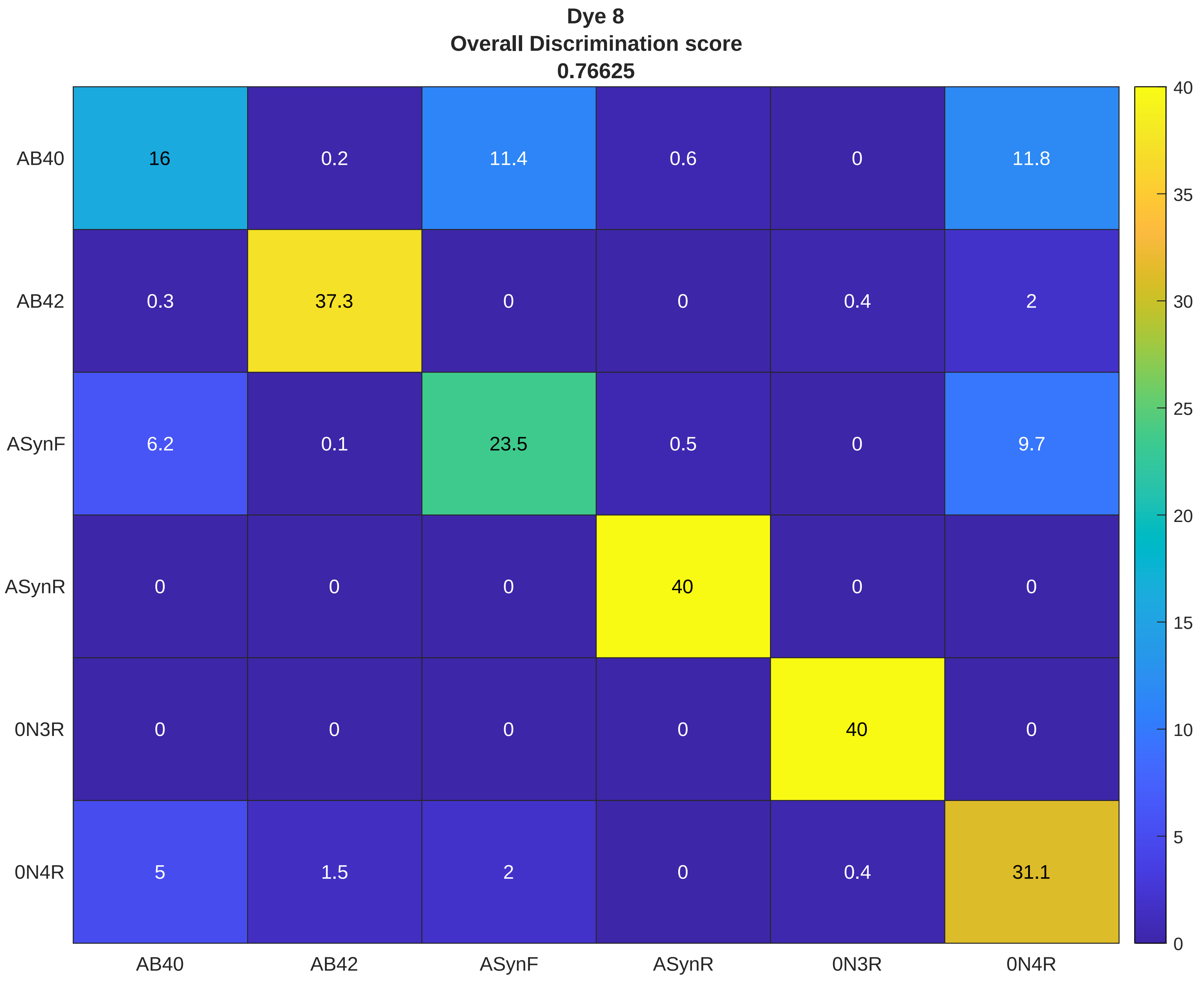
Dye 8
Overall Discrimination score
0.76625
AB40
AB42
ASynF
ASynR
0N3R
0N4R
16
0.2
11.4
0.6
0
11.8
0.3
37.3
0
0
0.4
2
6.2
0.1
23.5
0.5
0
9.7
0
0
0
40
0
0
0
0
0
0
40
0
5
1.5
2
0
0.4
31.1
AB40
AB42
ASynF
ASynR
0N3R
0N4R
40
35
30
25
20
15
10
5
0

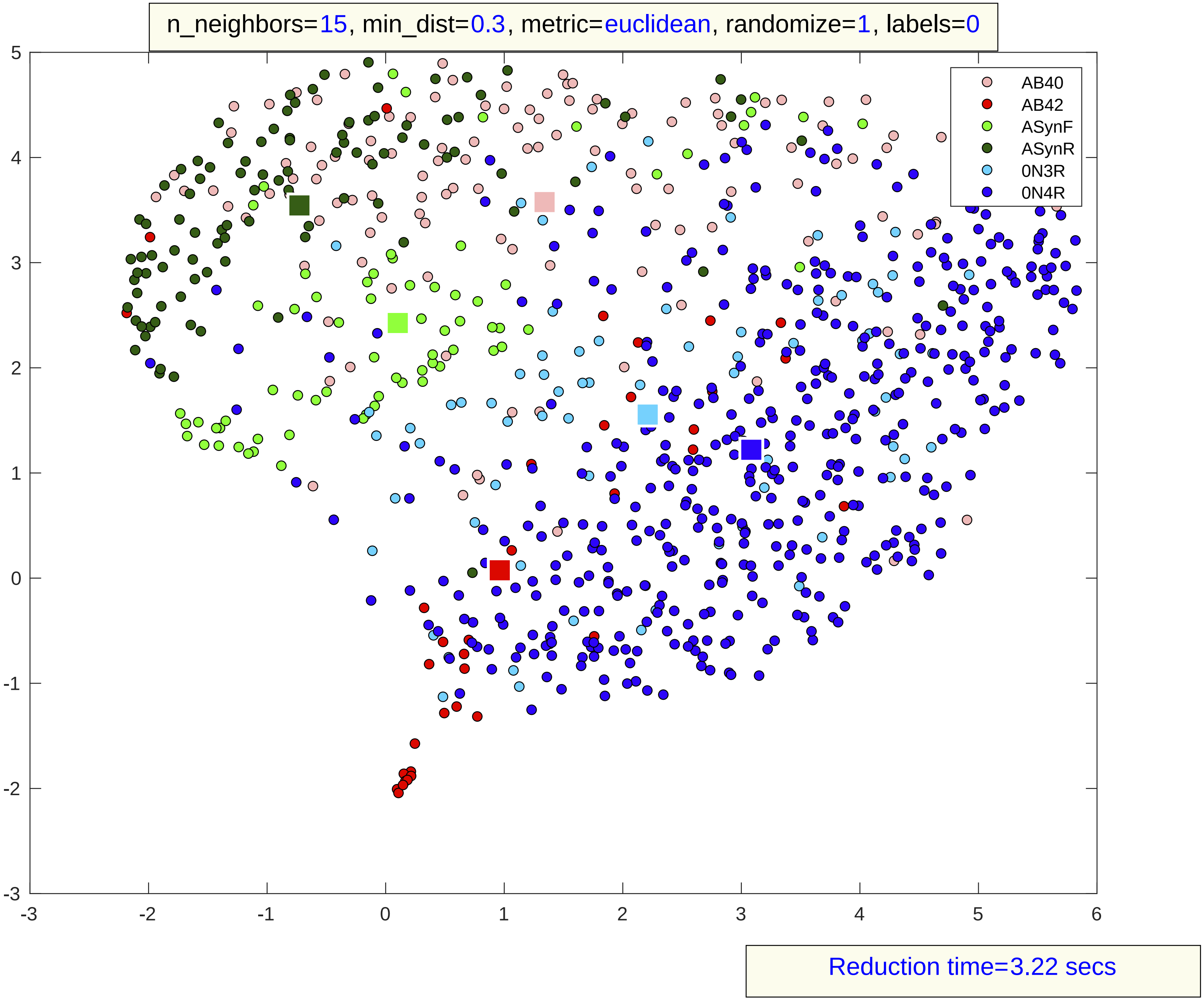
n_neighbors=15, min_dist=0.3, metric=euclidean, randomize=1, labels=0
AB40
AB42
ASynF
ASynR
0N3R
0N4R
5
4
3
2
1
0
-1
-2
-3
-3
-2
-1
0
1
2
3
4
5
6
Reduction time=3.22 secs

Dye 9
Overall Discrimination score
0.56125

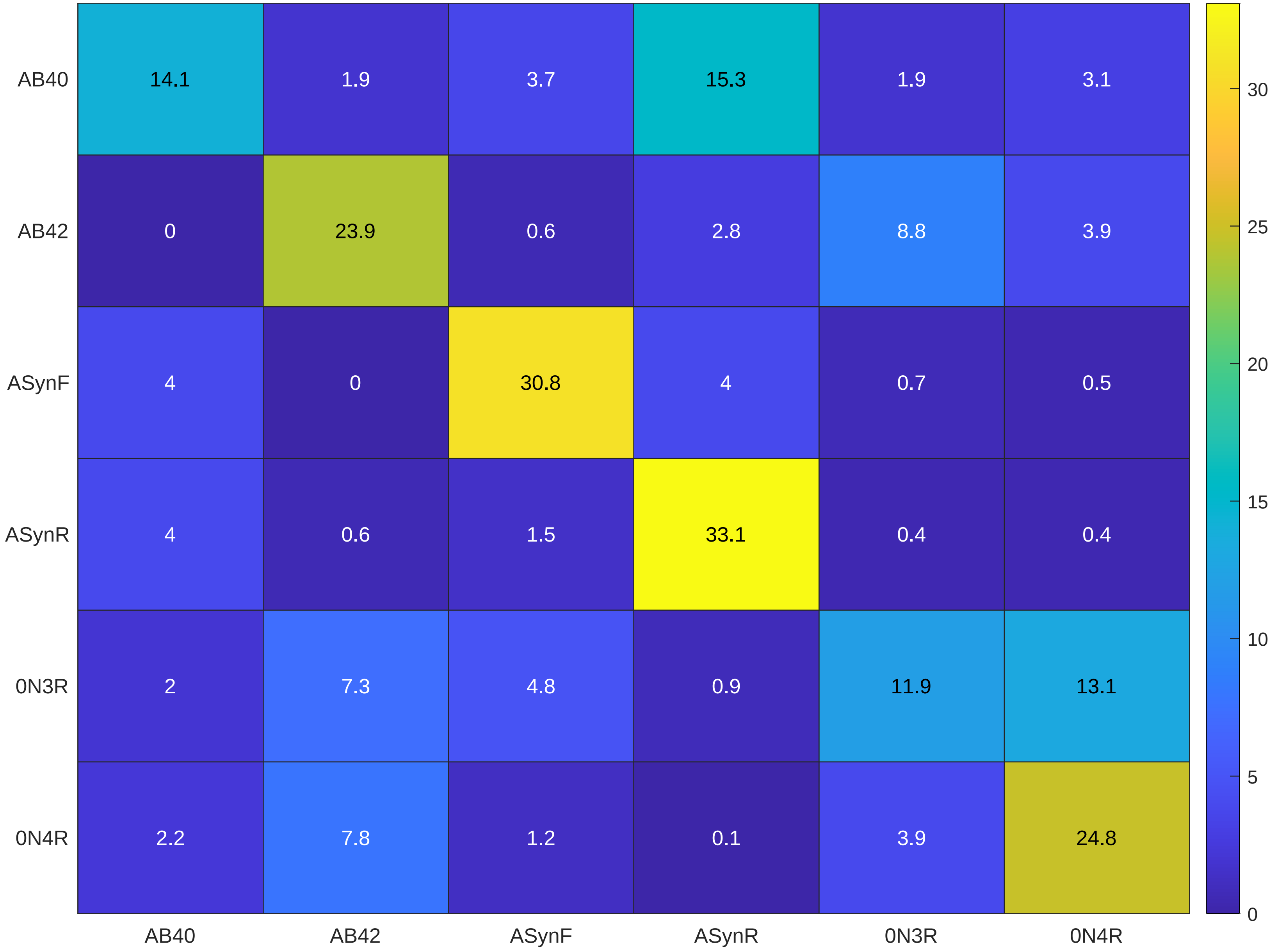

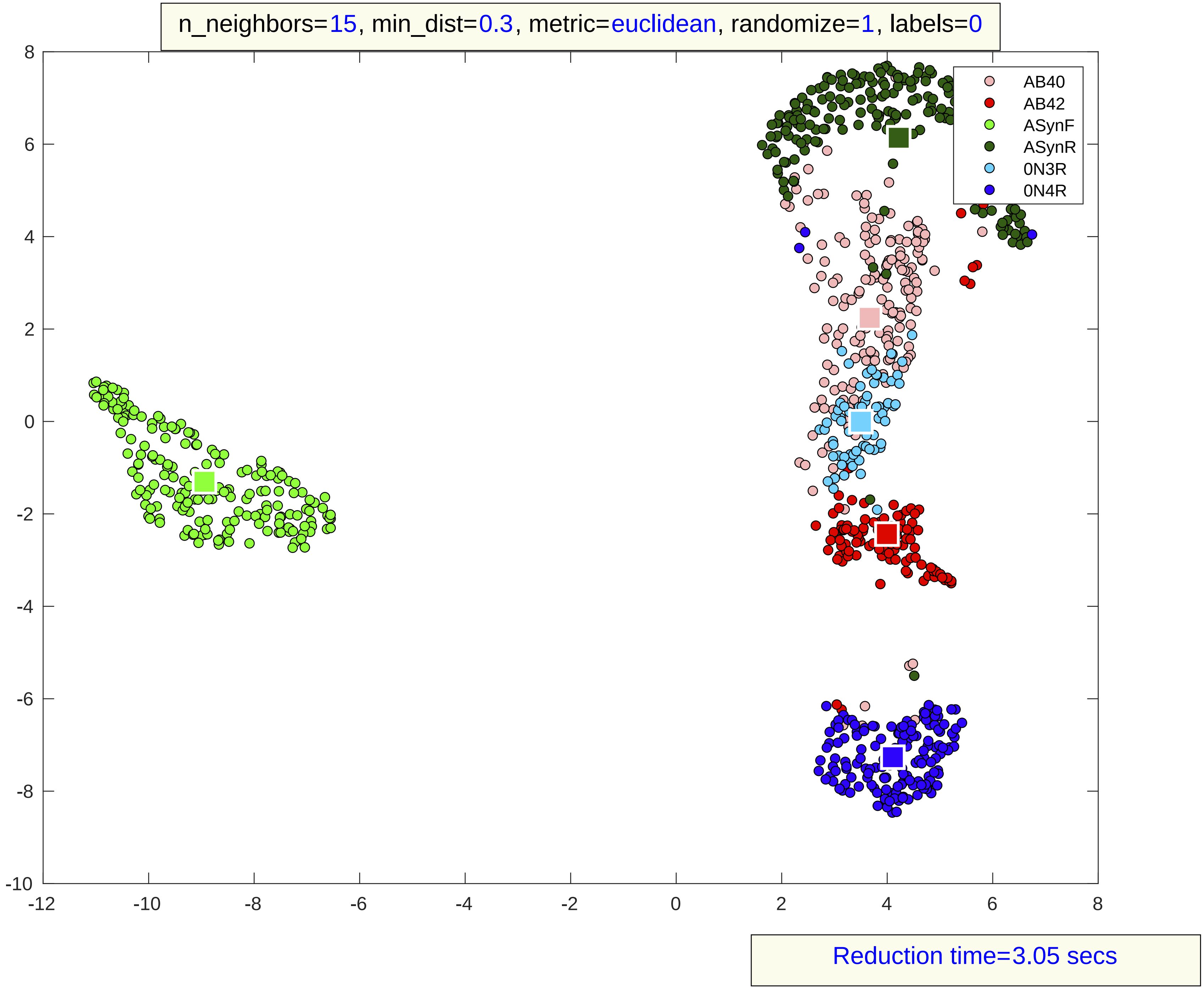
n_neighbors=15, min_dist=0.3, metric=euclidean, randomize=1, labels=0
AB40
AB42
ASynF
ASynR
0N3R
0N4R
8
6
4
2
0
-2
-4
-6
-8
-10
-12
-10
-8
-6
-4
-2
0
2
4
6
8
Reduction time=3.05 secs

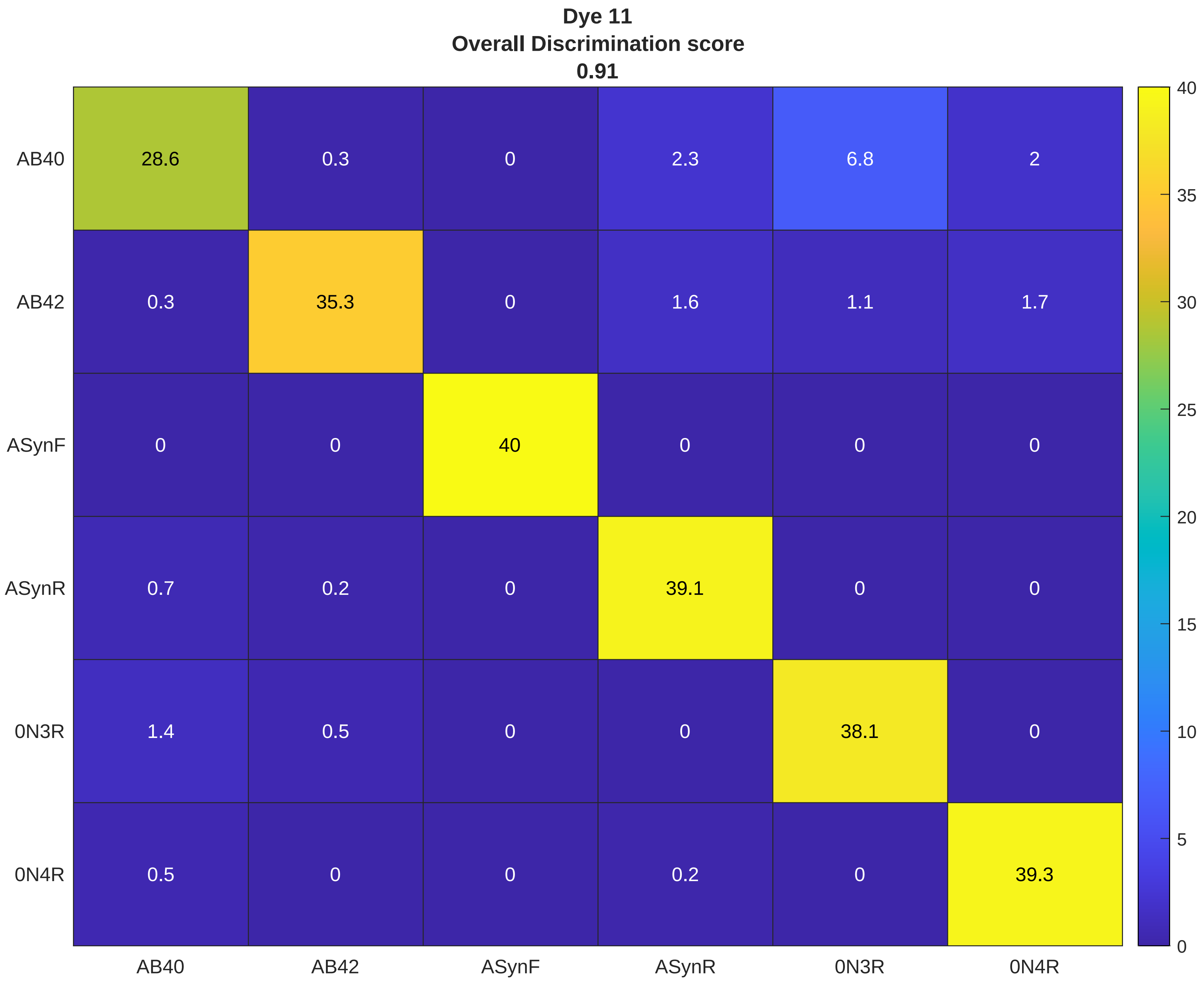
Dye 11
Overall Discrimination score
0.91
AB40
AB42
ASynF
ASynR
0N3R
0N4R
28.6
0.3
0
2.3
6.8
2
0.3
35.3
0
1.6
1.1
1.7
0
0
40
0
0
0
0.7
0.2
0
39.1
0
0
1.4
0.5
0
0
38.1
0
0.5
0
0
0.2
0
39.3
AB40
AB42
ASynF
ASynR
0N3R
0N4R
40
35
30
25
20
15
10
5
0

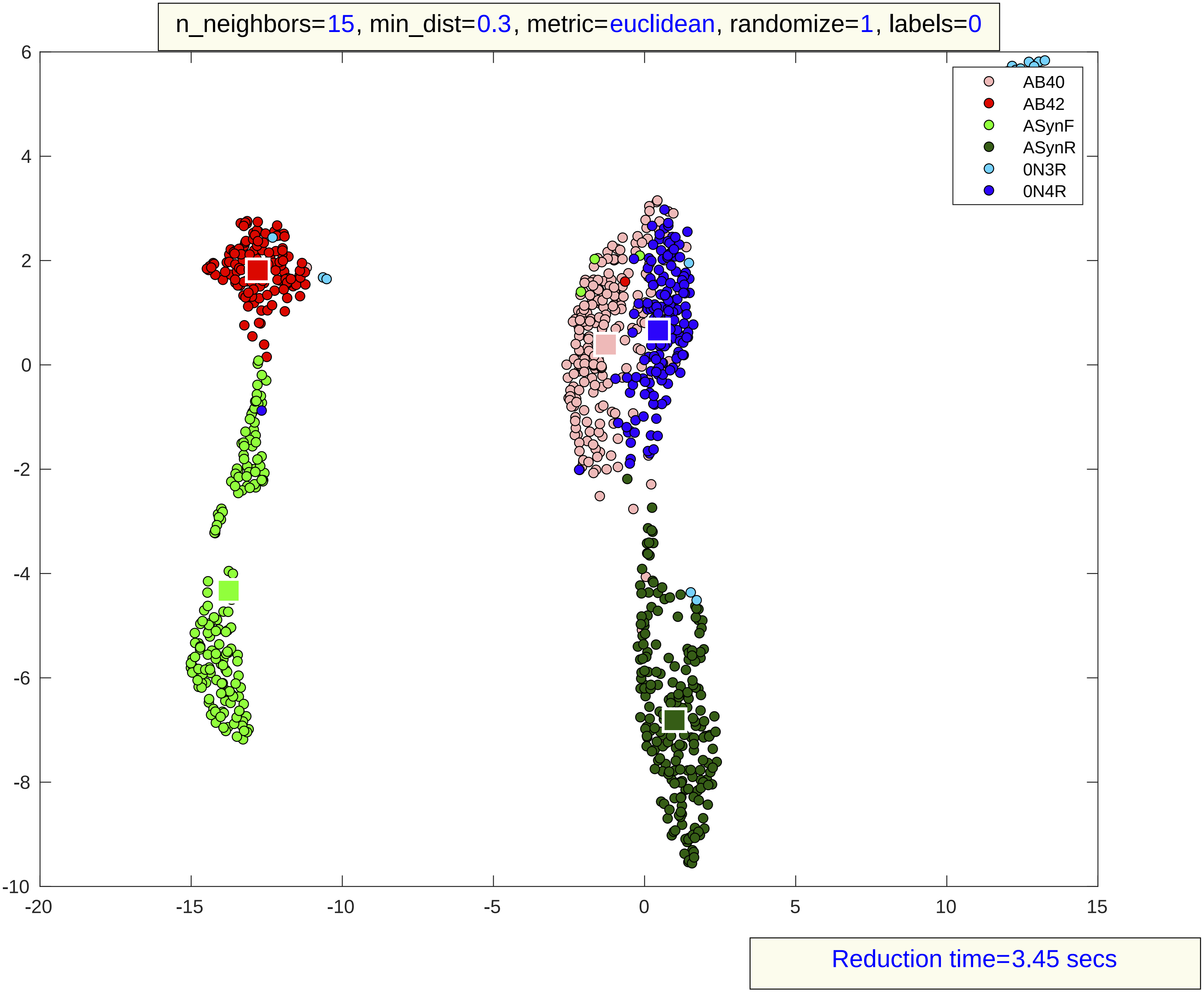
n_neighbors=15, min_dist=0.3, metric=euclidean, randomize=1, labels=0
AB40
AB42
ASynF
ASynR
0N3R
0N4R
6
4
2
0
-2
-4
-6
-8
-10
-20
-15
-10
-5
0
5
10
15
Reduction time=3.45 secs

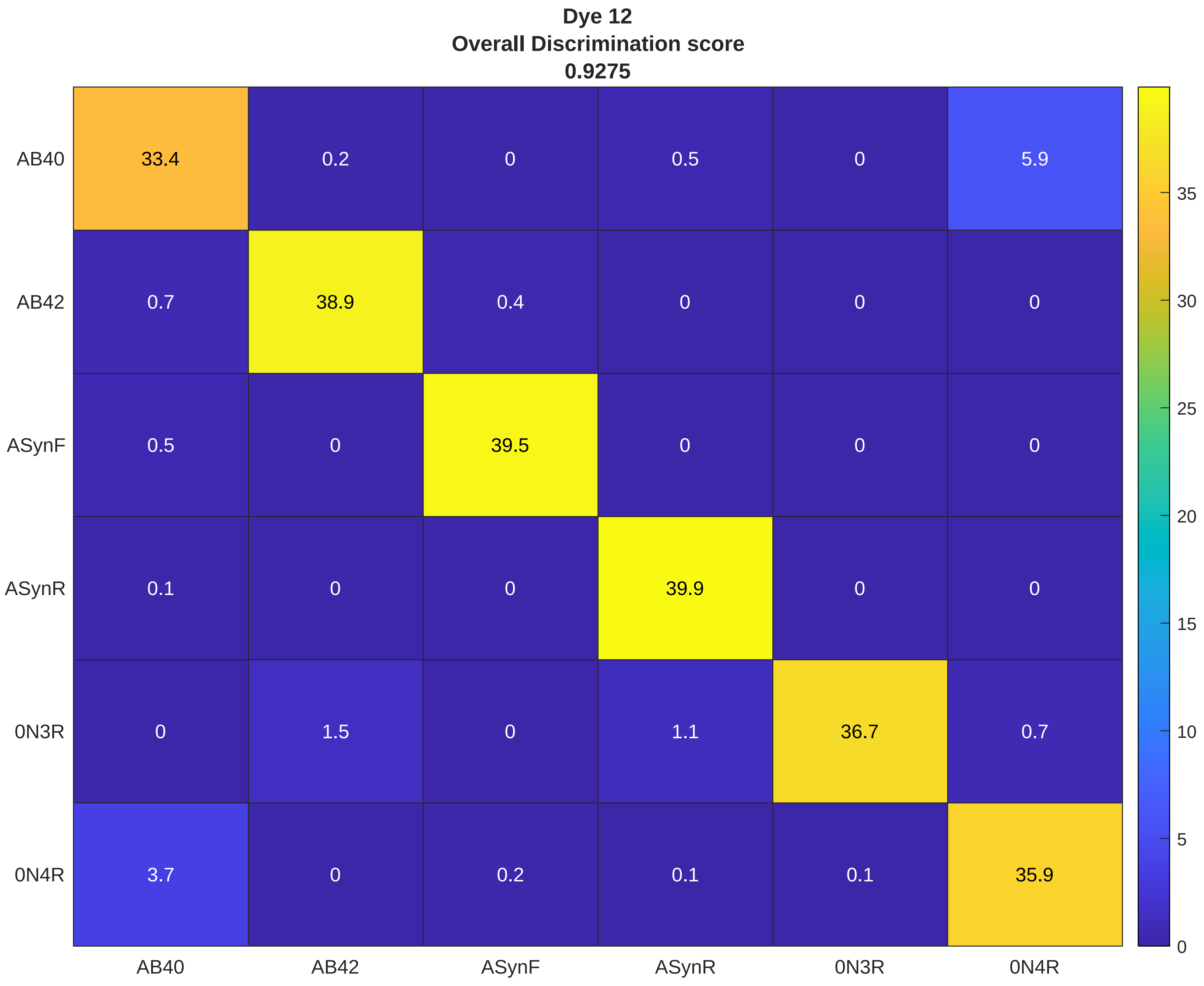

Dye 12
Overall Discrimination score
0.9275
AB40
AB42
ASynF
ASynR
0N3R
0N4R
33.4
0.2
0
0.5
0
5.9
0.7
38.9
0.4
0
0
0
0.5
0
39.5
0
0
0
0.1
0
0
39.9
0
0
0
1.5
0
1.1
36.7
0.7
3.7
0
0.2
0.1
0.1
35.9
AB40
AB42
ASynF
ASynR
0N3R
0N4R
35
30
25
20
15
10
5
0

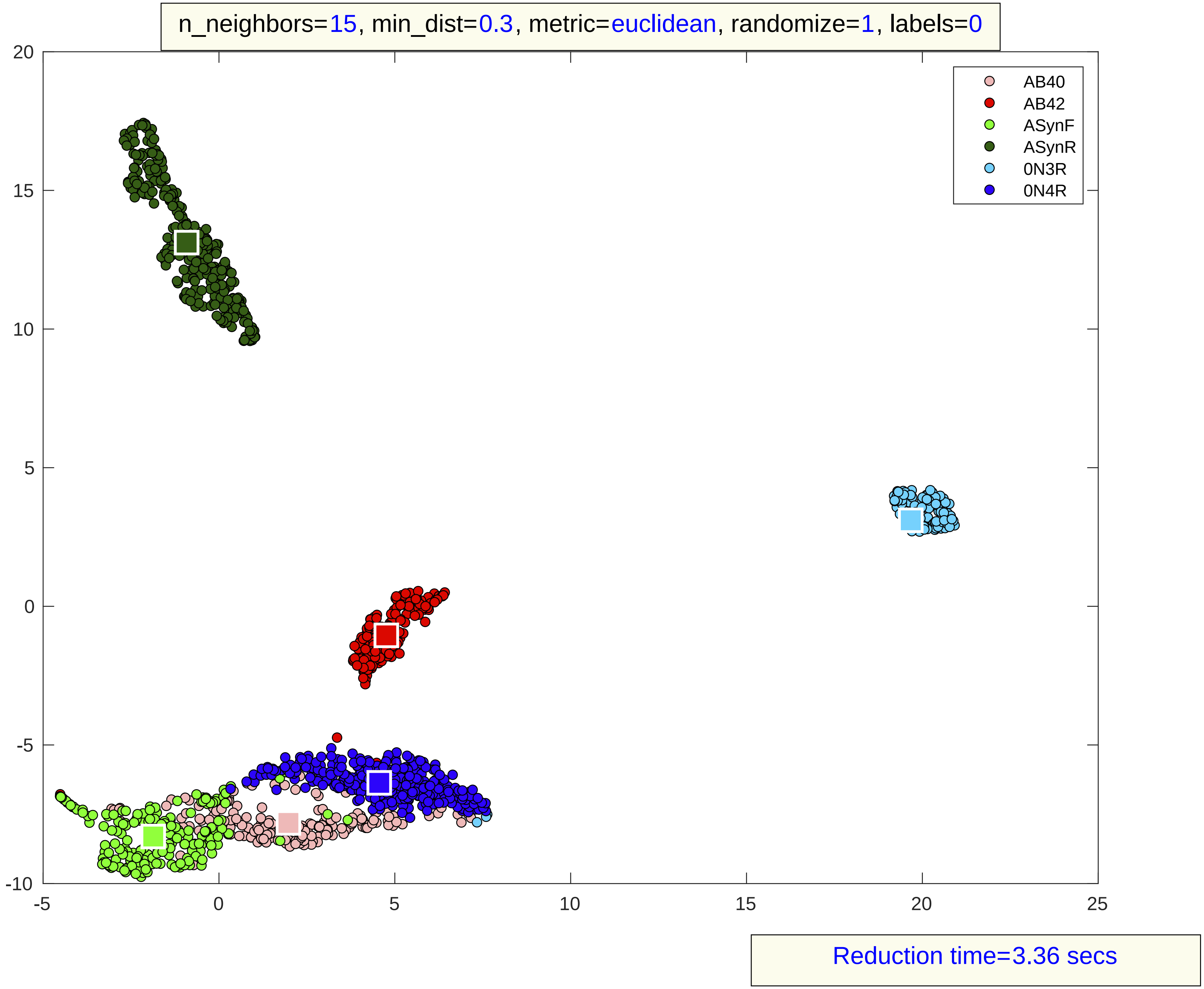
n_neighbors=15, min_dist=0.3, metric=euclidean, randomize=1, labels=0
AB40
AB42
ASynF
ASynR
0N3R
0N4R
20
15
10
5
0
-5
-10
-5
0
5
10
15
20
25
Reduction time=3.36 secs

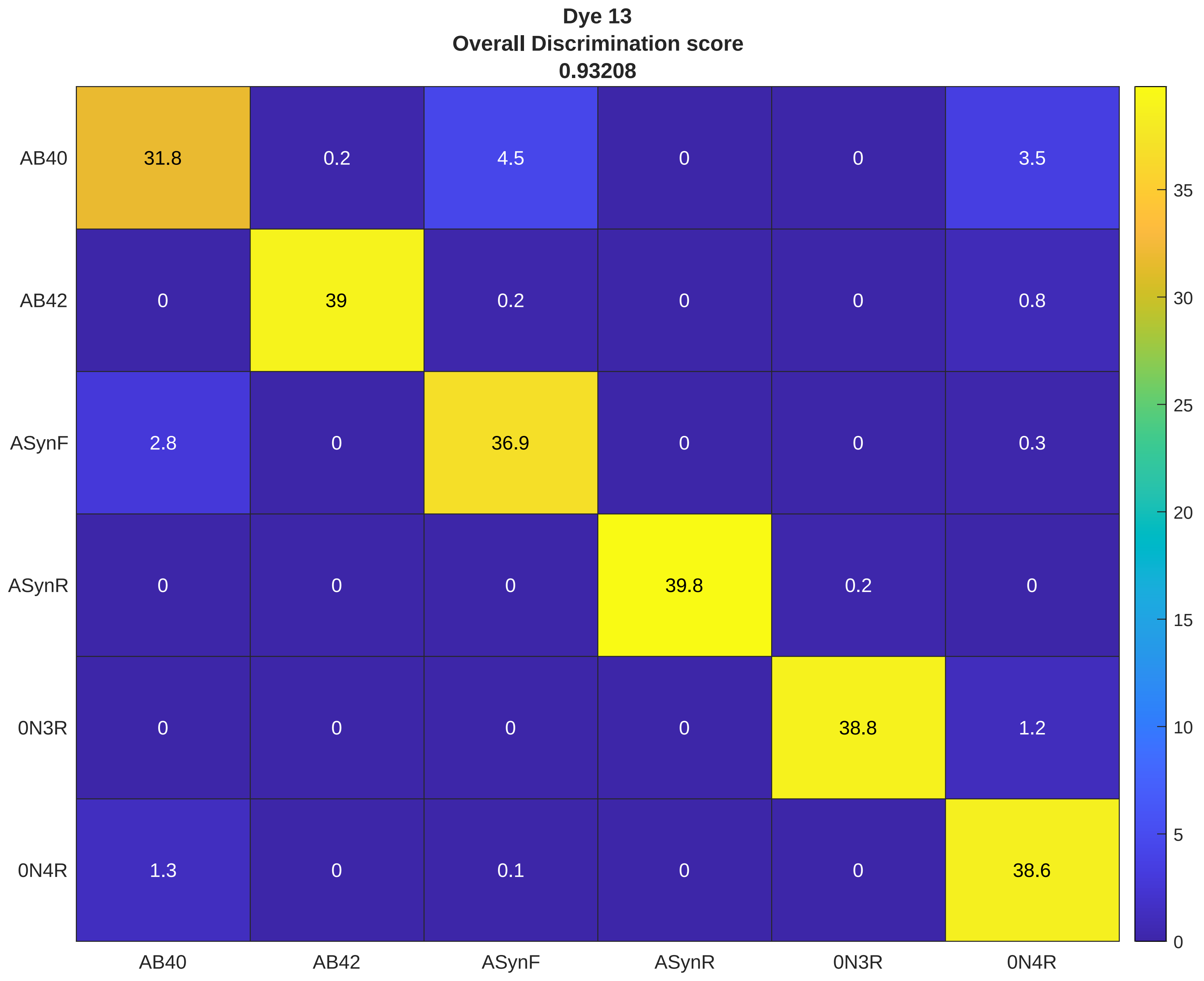
Dye 13
Overall Discrimination score
0.93208
AB40
AB42
ASynF
ASynR
0N3R
0N4R
31.8
0.2
4.5
0
0
3.5
0
39
0.2
0
0
0.8
2.8
0
36.9
0
0
0.3
0
0
0
39.8
0.2
0
0
0
0
0
38.8
1.2
1.3
0
0.1
0
0
38.6
AB40
AB42
ASynF
ASynR
0N3R
0N4R
35
30
25
20
15
10
5
0

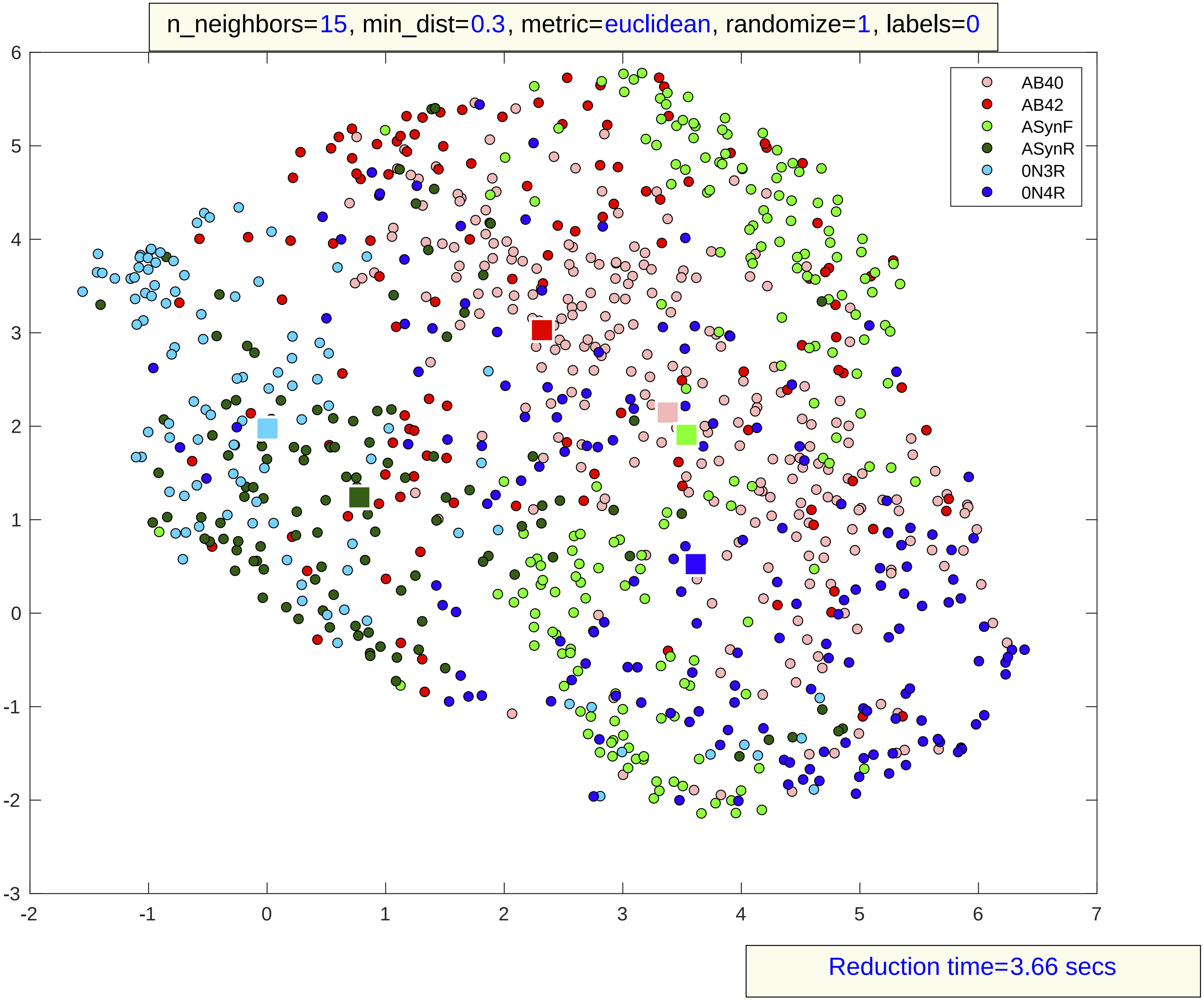

n_neighbors=15, min_dist=0.3, metric=euclidean, randomize=1, labels=0
AB40
AB42
ASynF
ASynR
0N3R
0N4R
6
5
4
3
2
1
0
-1
-2
-3
-2
-1
0
1
2
3
4
5
6
7
Reduction time=3.66 secs

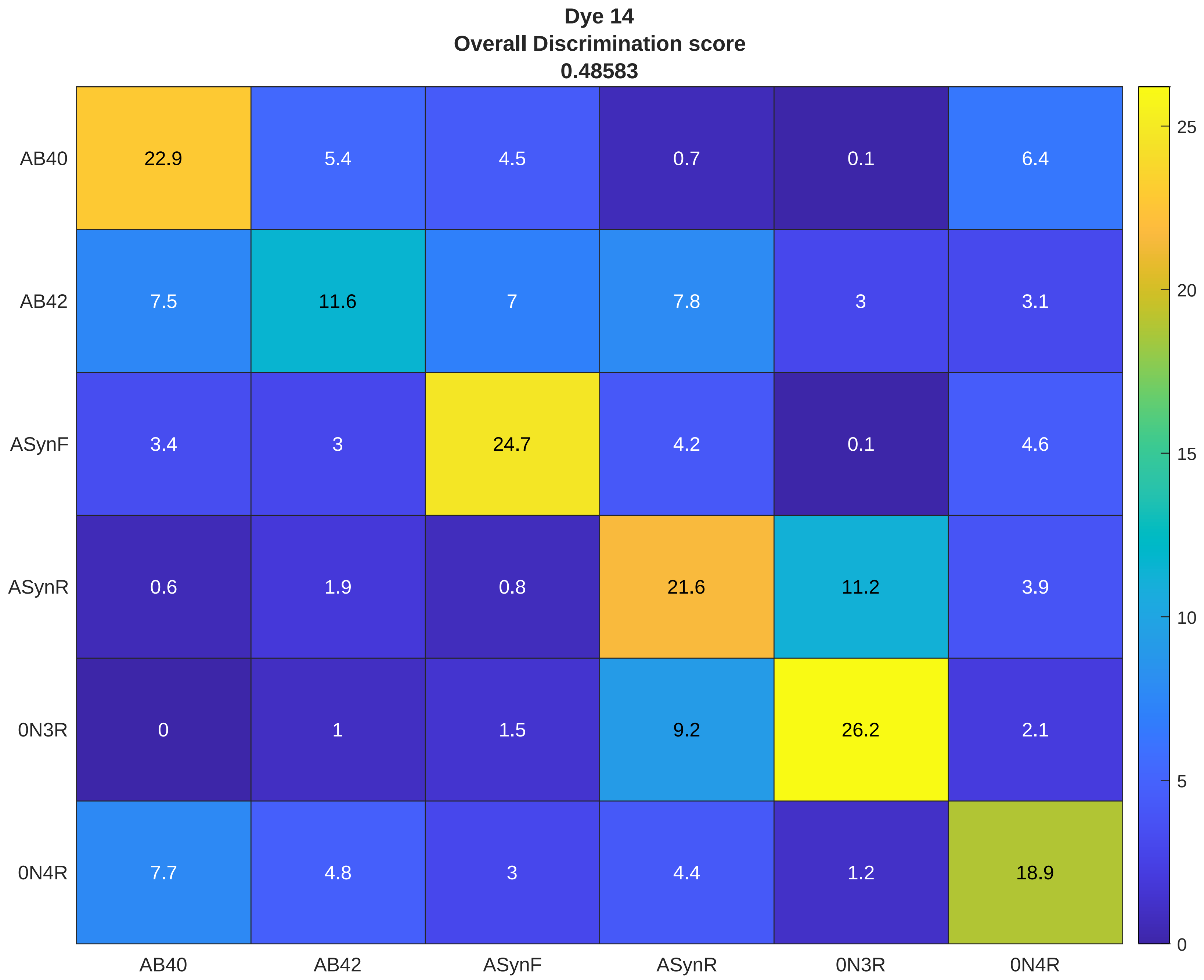
Dye 14
Overall Discrimination score
0.48583
AB40
AB42
ASynF
ASynR
0N3R
0N4R
22.9
5.4
4.5
0.7
0.1
6.4
7.5
11.6
7
7.8
3
3.1
3.4
3
24.7
4.2
0.1
4.6
0.6
1.9
0.8
21.6
11.2
3.9
0
1
1.5
9.2
26.2
2.1
7.7
4.8
3
4.4
1.2
18.9
AB40
AB42
ASynF
ASynR
0N3R
0N4R
25
20
15
10
5
0

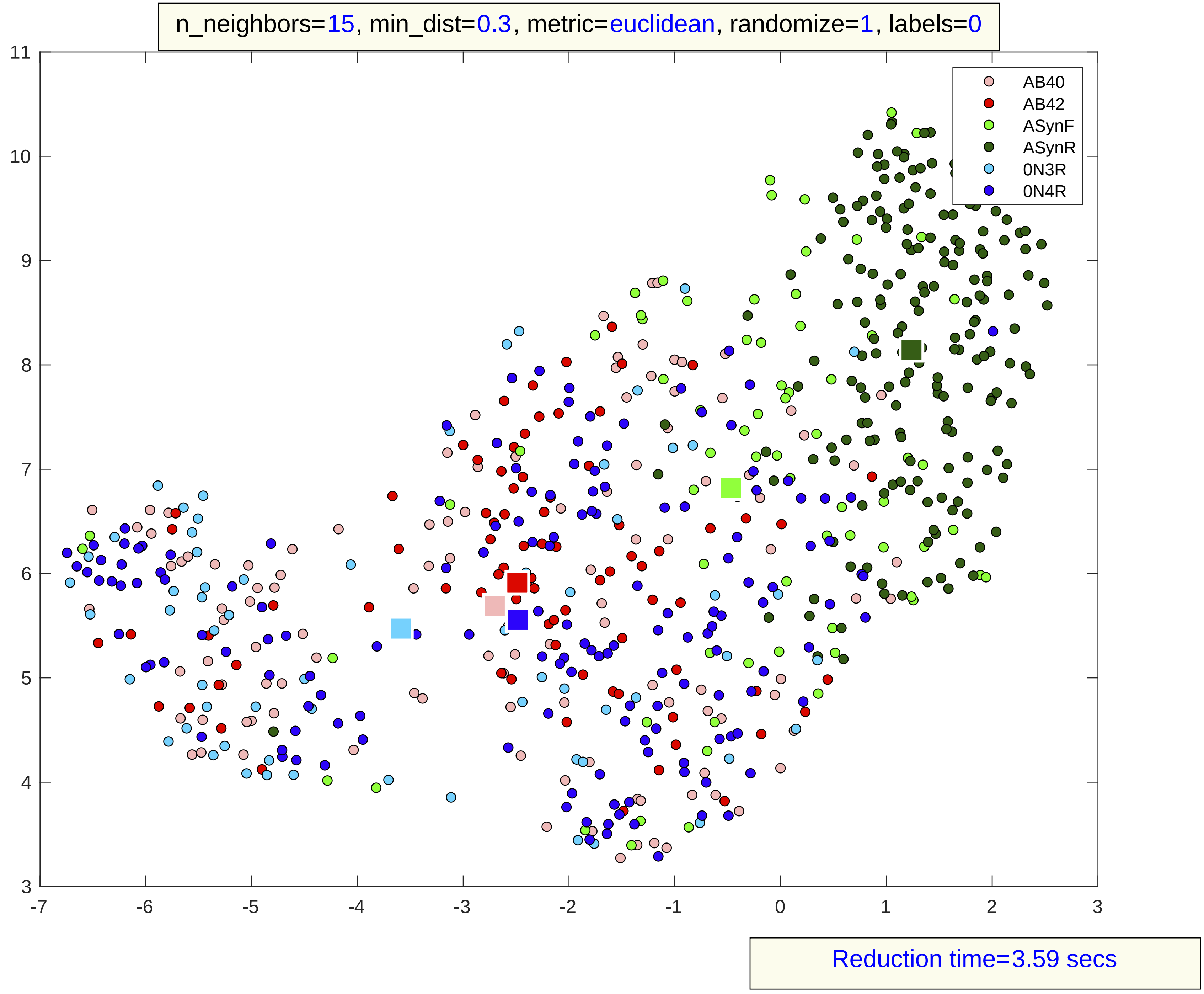

n_neighbors=15, min_dist=0.3, metric=euclidean, randomize=1, labels=0
AB40
AB42
ASynF
ASynR
0N3R
0N4R
11
10
9
8
7
6
5
4
3
-7
-6
-5
-4
-3
-2
-1
0
1
2
3
Reduction time=3.59 secs

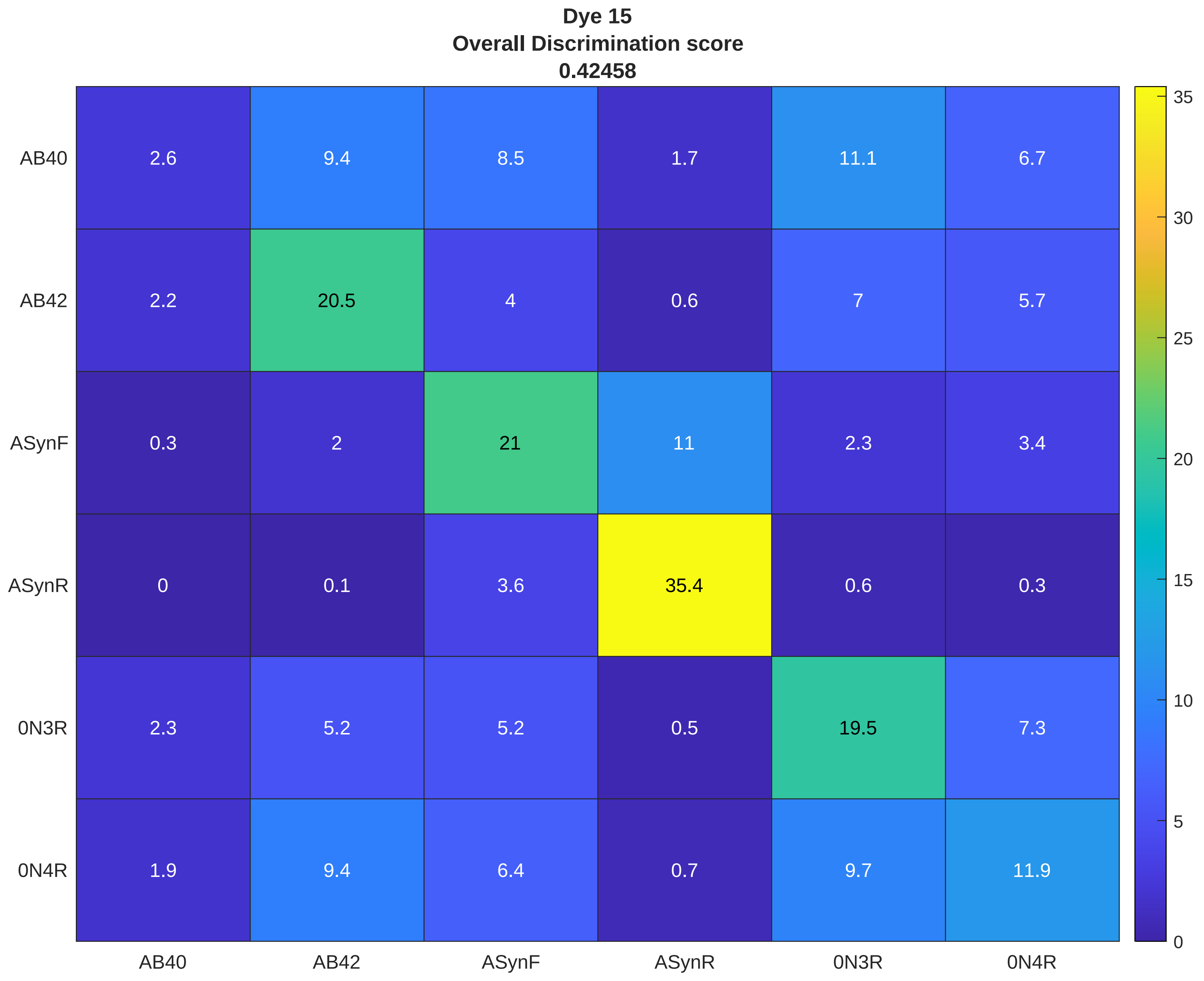

Dye 15
Overall Discrimination score
0.42458
AB40
AB42
ASynF
ASynR
0N3R
0N4R
2.6
9.4
8.5
1.7
11.1
6.7
2.2
20.5
4
0.6
7
5.7
0.3
2
21
11
2.3
3.4
0
0.1
3.6
35.4
0.6
0.3
2.3
5.2
5.2
0.5
19.5
7.3
1.9
9.4
6.4
0.7
9.7
11.9
AB40
AB42
ASynF
ASynR
0N3R
0N4R
35
30
25
20
15
10
5
0

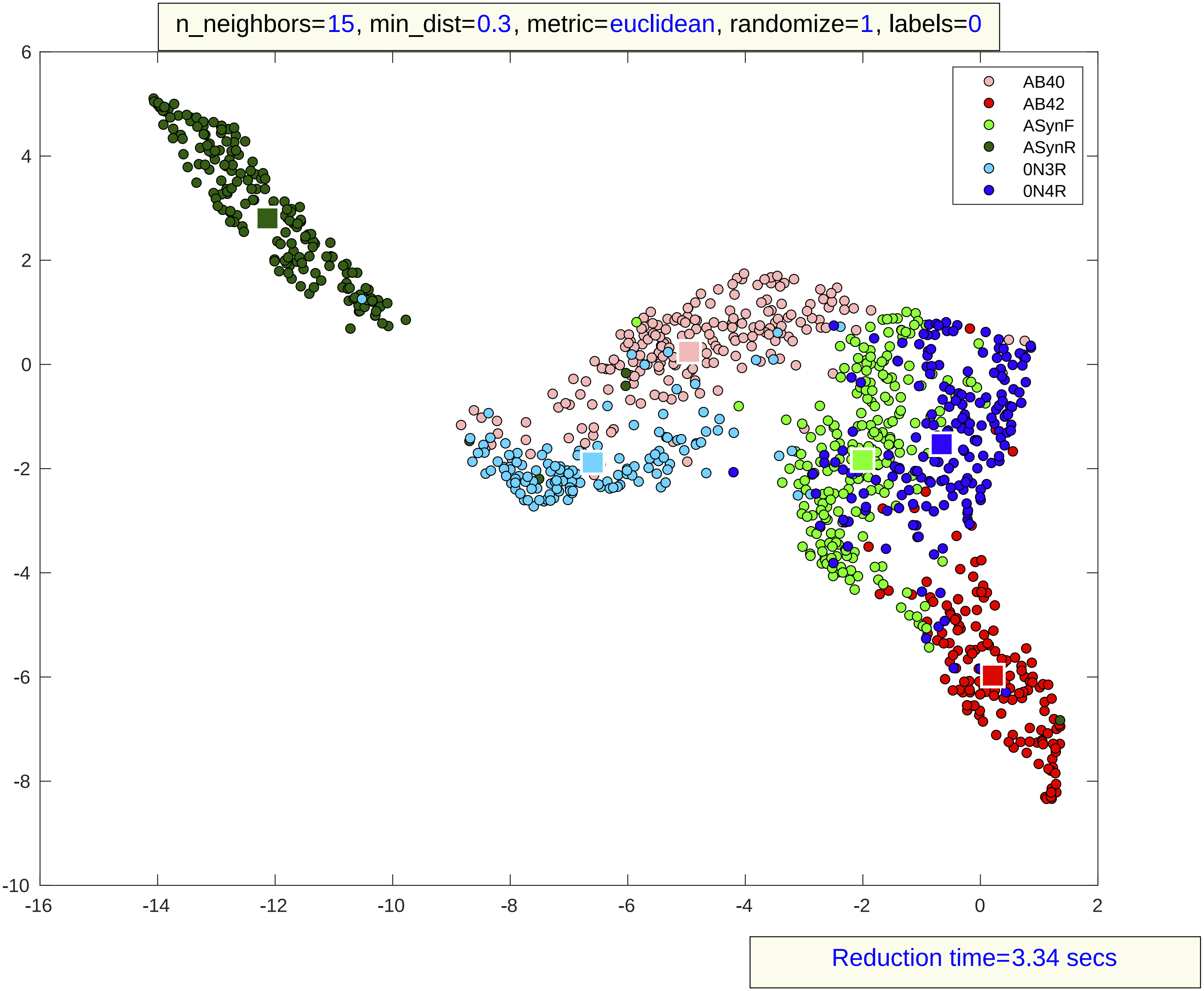

n_neighbors=15, min_dist=0.3, metric=euclidean, randomize=1, labels=0
AB40
AB42
ASynF
ASynR
0N3R
0N4R
6
4
2
0
-2
-4
-6
-8
-10
-16
-14
-12
-10
-8
-6
-4
-2
0
2
Reduction time=3.34 secs

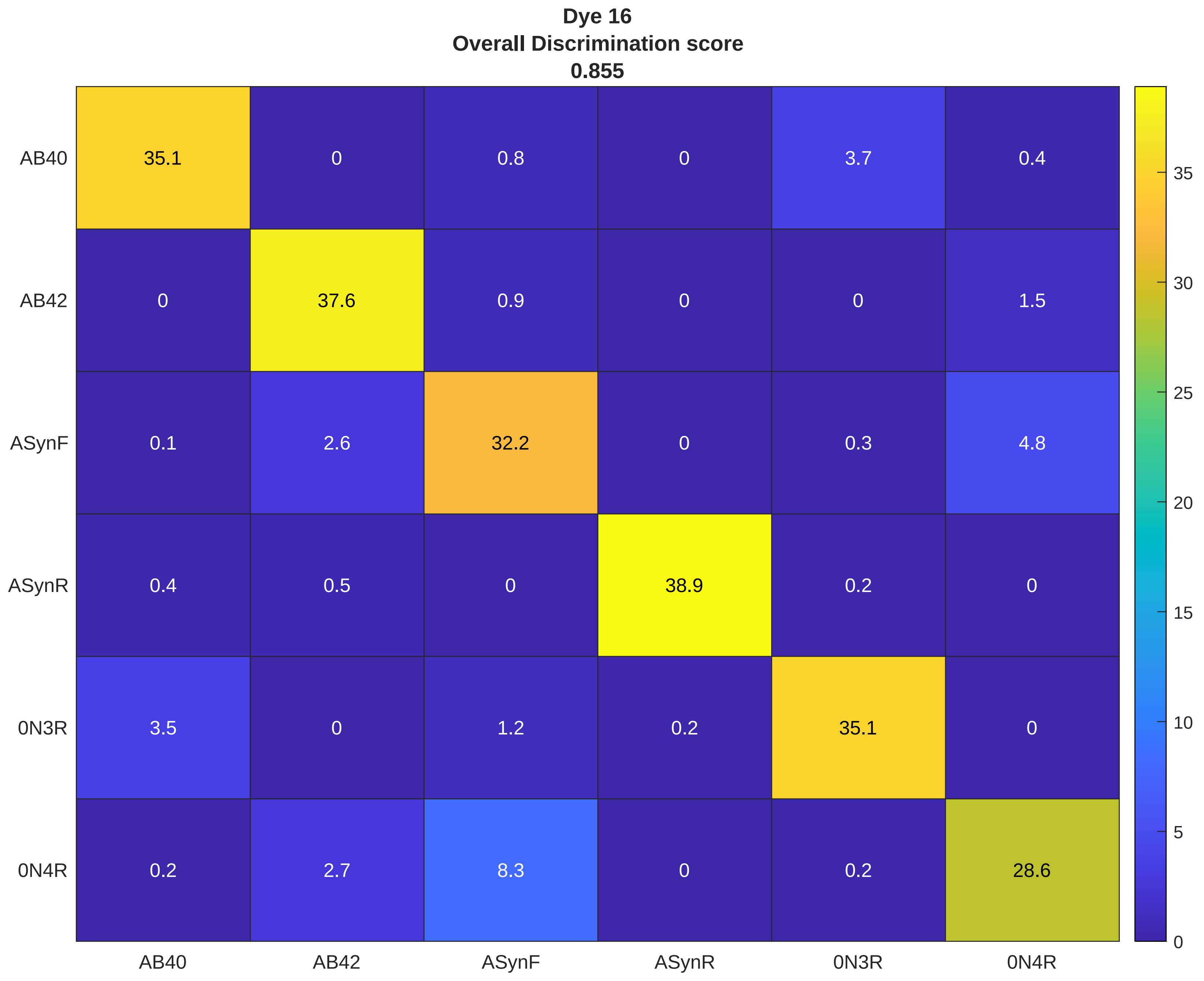
Dye 16
Overall Discrimination score
0.855
AB40
AB42
ASynF
ASynR
0N3R
0N4R
35.1
0
0.8
0
3.7
0.4
0
37.6
0.9
0
0
1.5
0.1
2.6
32.2
0
0.3
4.8
0.4
0.5
0
38.9
0.2
0
3.5
0
1.2
0.2
35.1
0
0.2
2.7
8.3
0
0.2
28.6
AB40
AB42
ASynF
ASynR
0N3R
0N4R
35
30
25
20
15
10
5
0

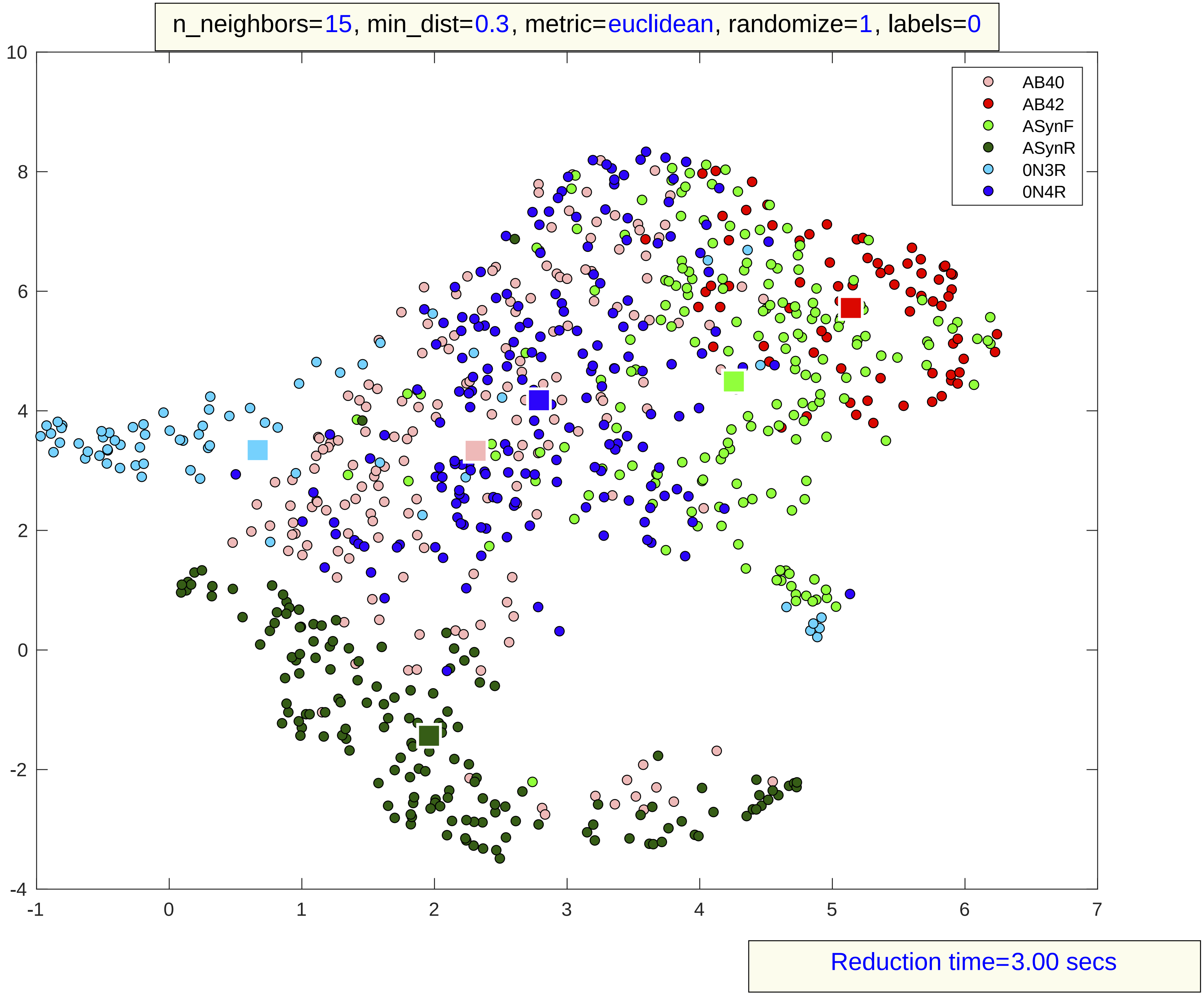

n_neighbors=15, min_dist=0.3, metric=euclidean, randomize=1, labels=0
AB40
AB42
ASynF
ASynR
0N3R
0N4R
10
8
6
4
2
0
-2
-4
-1
0
1
2
3
4
5
6
7
Reduction time=3.00 secs

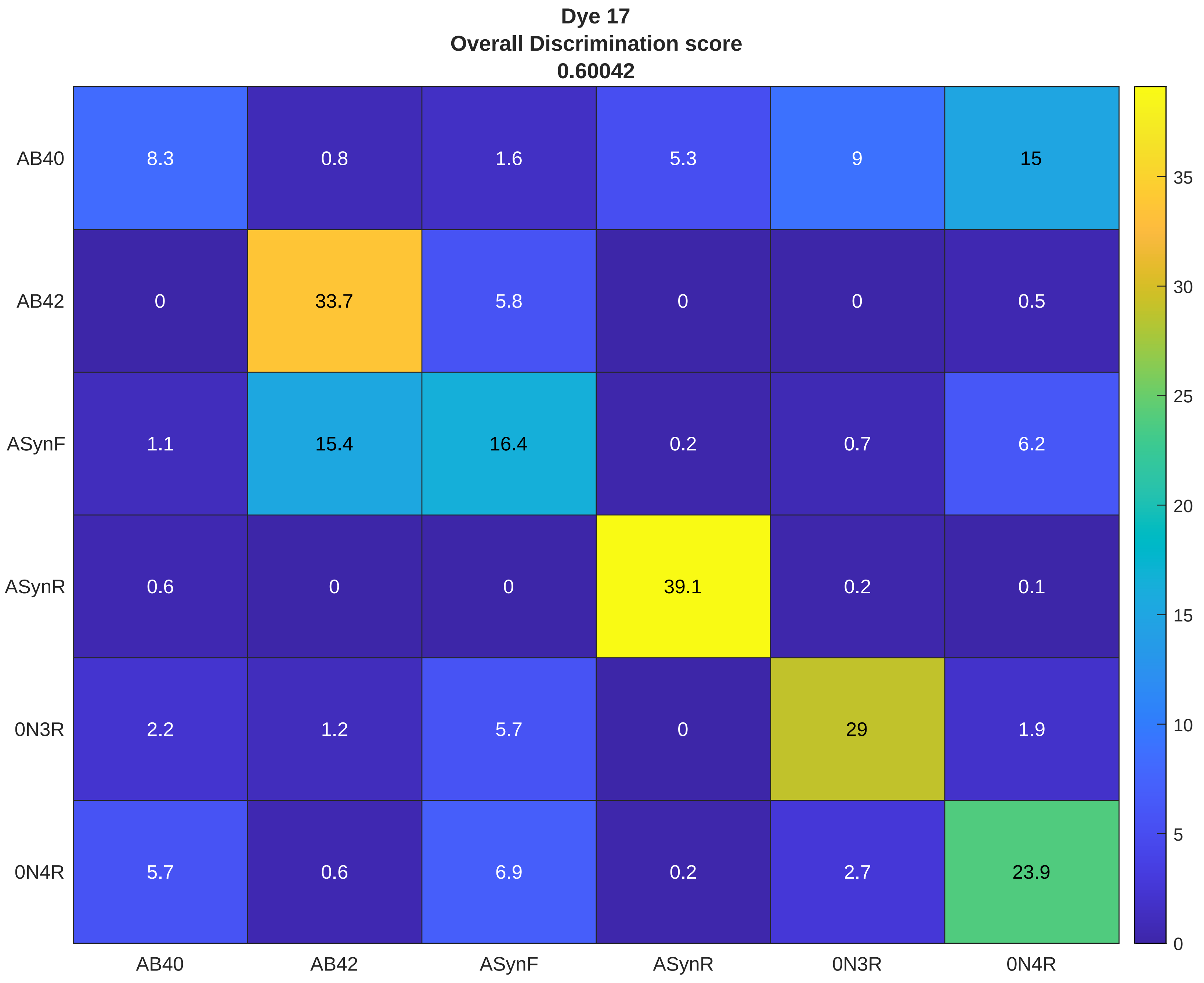
Dye 17
Overall Discrimination score
0.60042
AB40
AB42
ASynF
ASynR
0N3R
0N4R
8.3 0.8 1.6 5.3 9 15
0 33.7 5.8 0 0 0.5
1.1 15.4 16.4 0.2 0.7 6.2
0.6 0 0 39.1 0.2 0.1
2.2 1.2 5.7 0 29 1.9
5.7 0.6 6.9 0.2 2.7 23.9
AB40
AB42
ASynF
ASynR
0N3R
0N4R
35
30
25
20
15
10
5
0

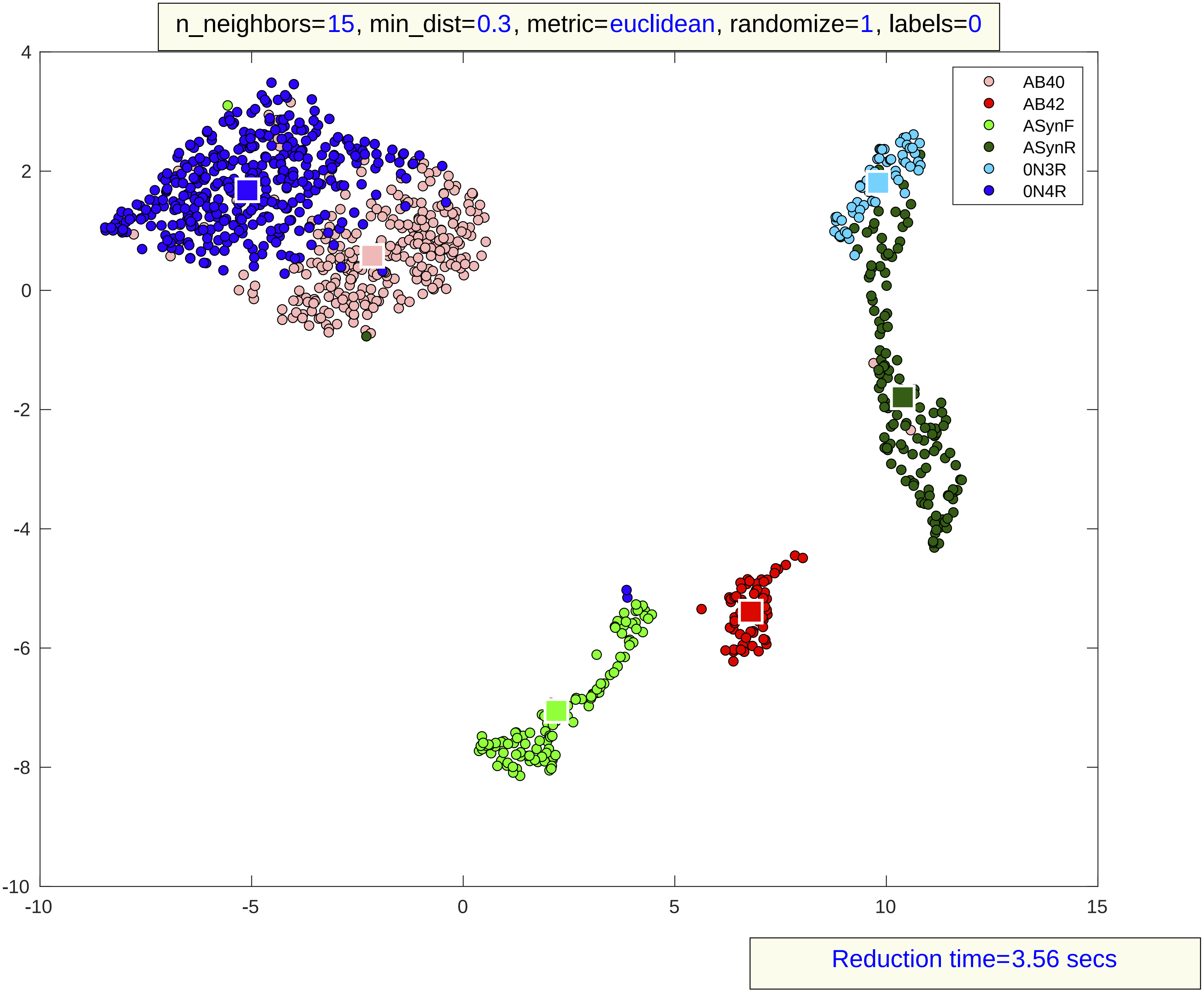

n_neighbors=15, min_dist=0.3, metric=euclidean, randomize=1, labels=0
AB40
AB42
ASynF
ASynR
0N3R
0N4R
4
2
0
-2
-4
-6
-8
-10
-10
-5
0
5
10
15
Reduction time=3.56 secs

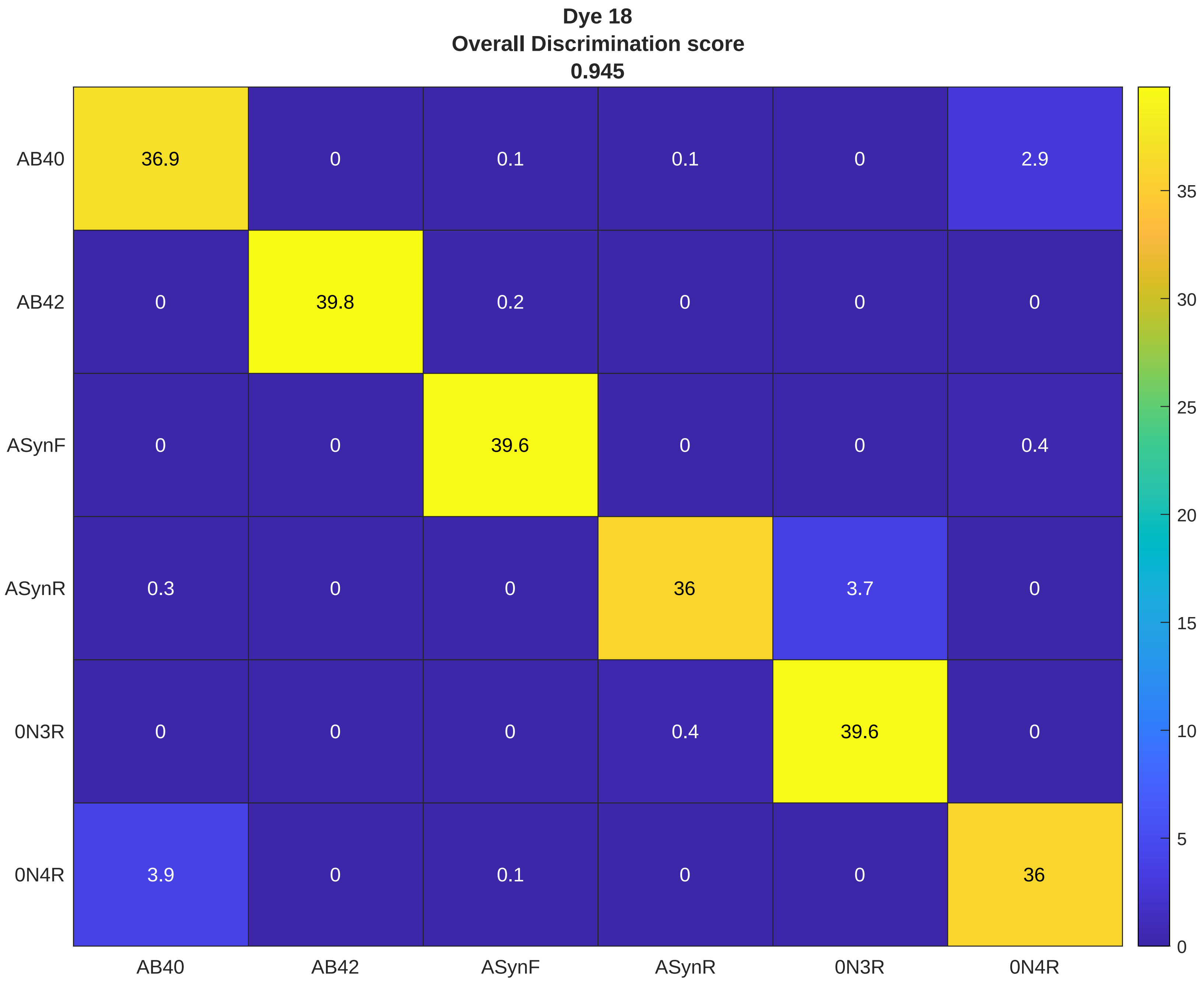
Dye 18
Overall Discrimination score
0.945
AB40
AB42
ASynF
ASynR
0N3R
0N4R
36.9
0
0.1
0.1
0
2.9
0
39.8
0.2
0
0
0
0
0
39.6
0
0
0.4
0.3
0
0
36
3.7
0
0
0
0
0.4
39.6
0
3.9
0
0.1
0
0
36
AB40
AB42
ASynF
ASynR
0N3R
0N4R
35
30
25
20
15
10
5
0

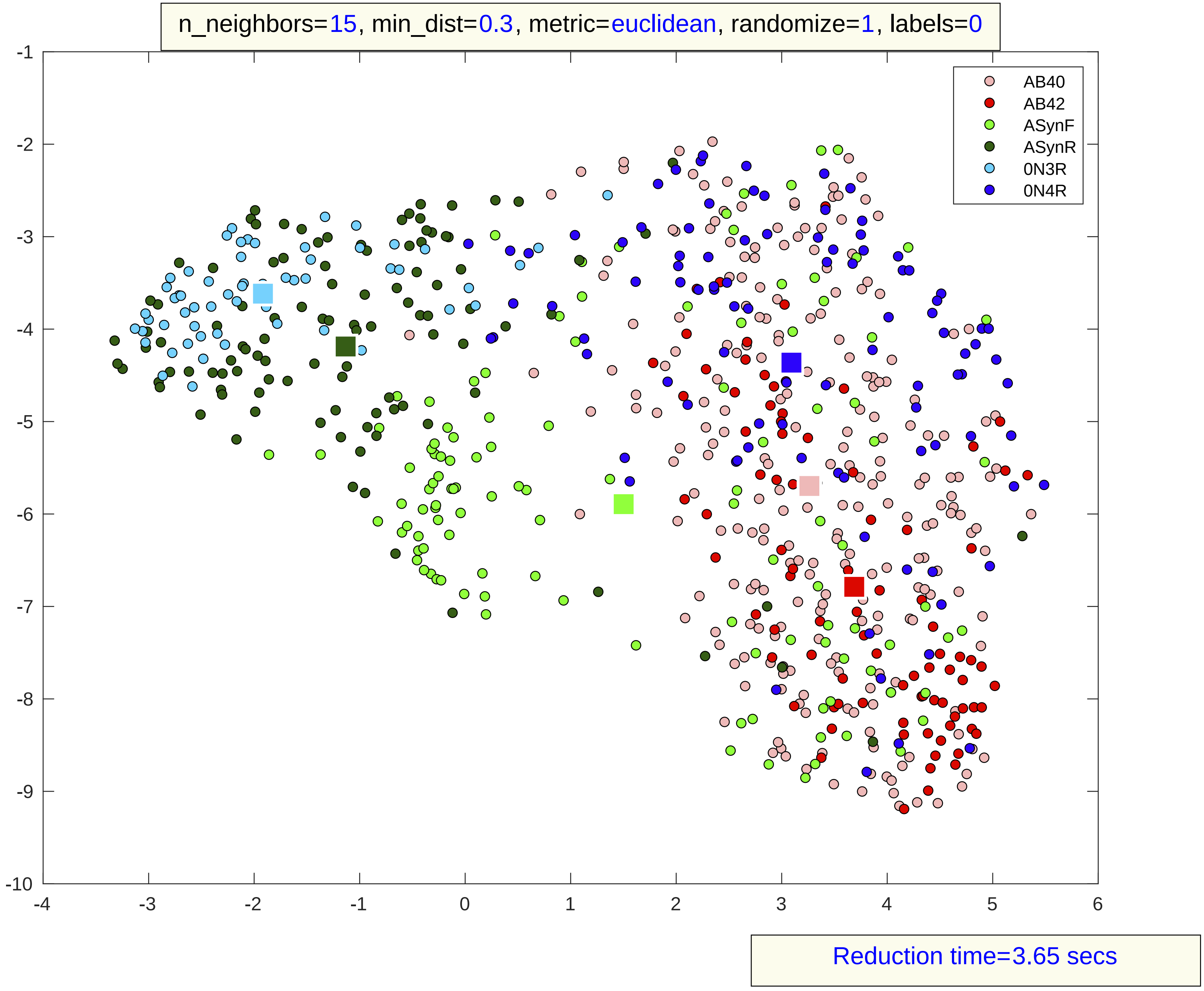

n_neighbors=15, min_dist=0.3, metric=euclidean, randomize=1, labels=0
AB40
AB42
ASynF
ASynR
0N3R
0N4R
-1
-2
-3
-4
-5
-6
-7
-8
-9
-10
-4
-3
-2
-1
0
1
2
3
4
5
6
Reduction time=3.65 secs

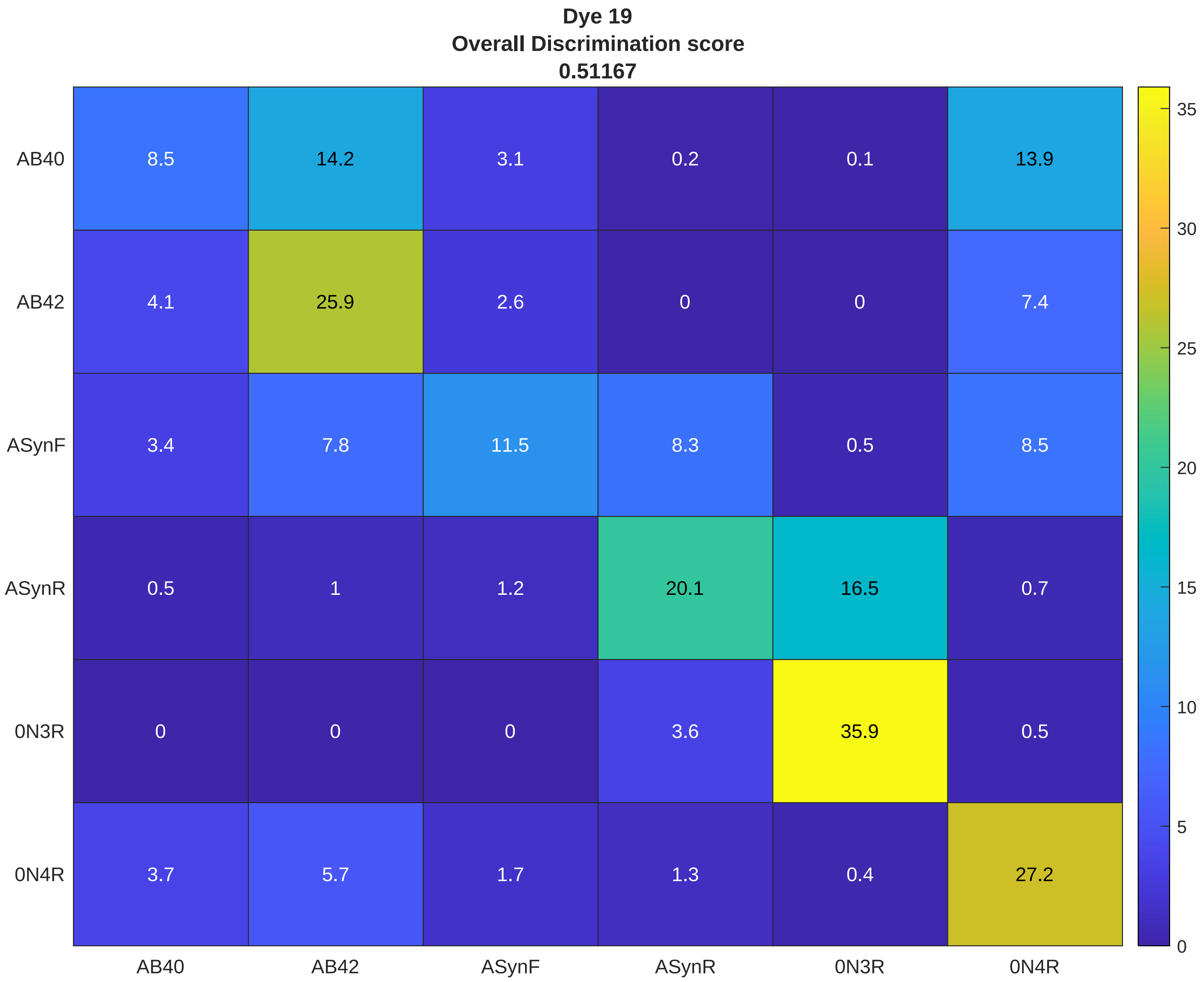
Dye 19
Overall Discrimination score
0.51167
AB40
AB42
ASynF
ASynR
0N3R
0N4R
8.5
14.2
3.1
0.2
0.1
13.9
4.1
25.9
2.6
0
0
7.4
3.4
7.8
11.5
8.3
0.5
8.5
0.5
1
1.2
20.1
16.5
0.7
0
0
0
3.6
35.9
0.5
3.7
5.7
1.7
1.3
0.4
27.2
AB40
AB42
ASynF
ASynR
0N3R
0N4R
35
30
25
20
15
10
5
0

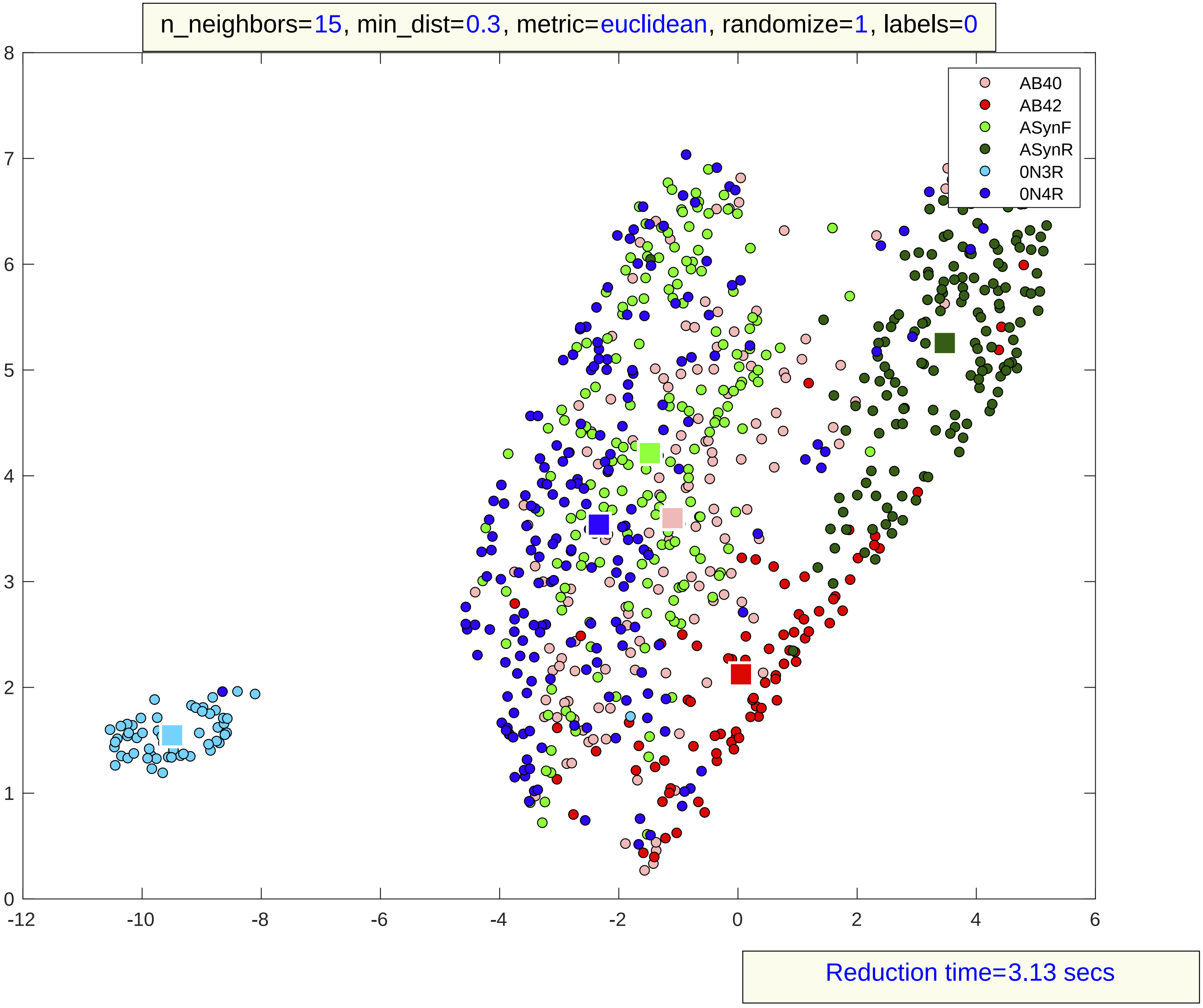

n_neighbors=15, min_dist=0.3, metric=euclidean, randomize=1, labels=0
AB40
AB42
ASynF
ASynR
0N3R
0N4R
8
7
6
5
4
3
2
1
0
-12
-10
-8
-6
-4
-2
0
2
4
6
Reduction time=3.13 secs

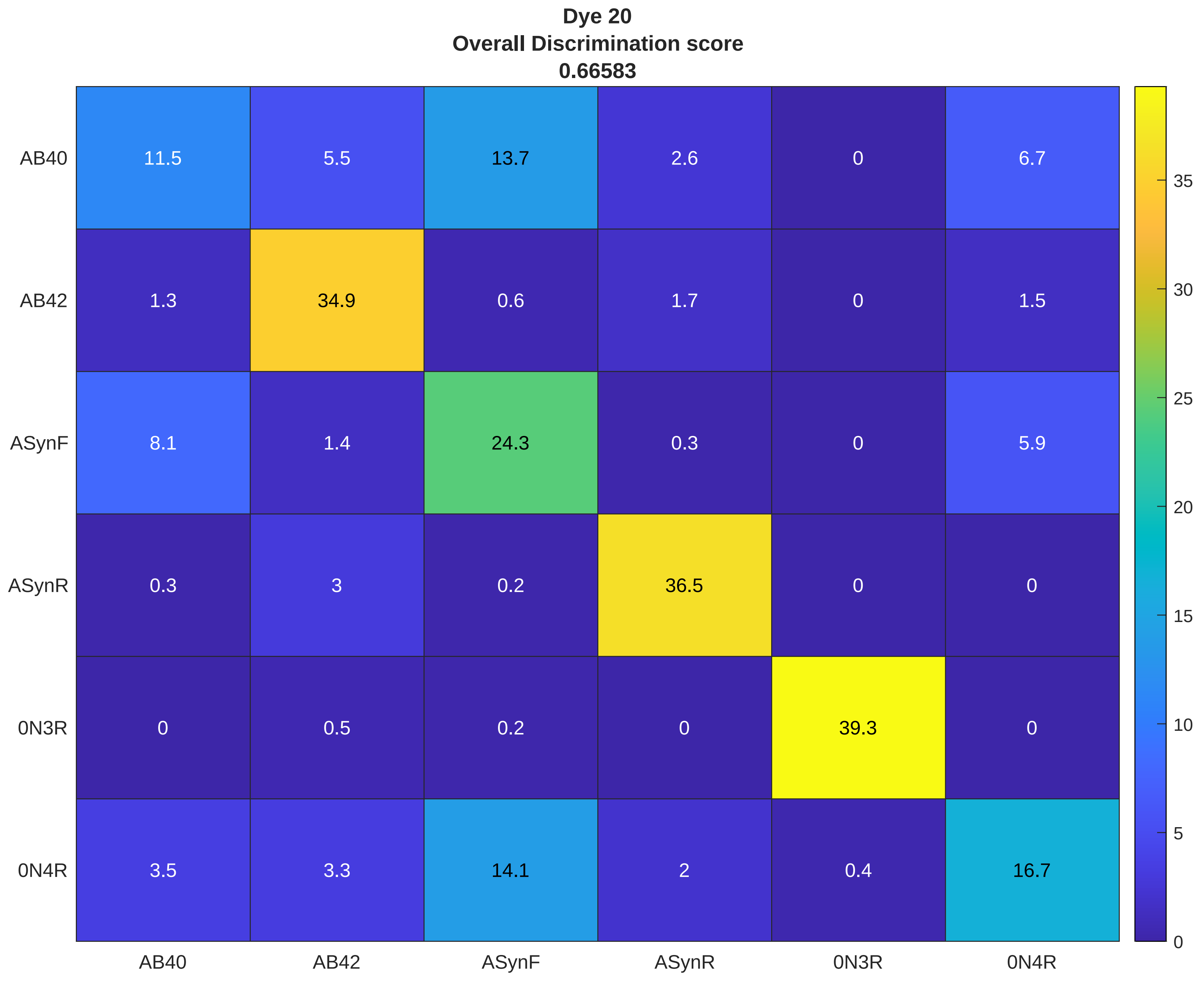
Dye 20
Overall Discrimination score
0.66583
AB40
AB42
ASynF
ASynR
0N3R
0N4R
11.5
5.5
13.7
2.6
0
6.7
1.3
34.9
0.6
1.7
0
1.5
8.1
1.4
24.3
0.3
0
5.9
0.3
3
0.2
36.5
0
0
0
0.5
0.2
0
39.3
0
3.5
3.3
14.1
2
0.4
16.7
AB40
AB42
ASynF
ASynR
0N3R
0N4R
35
30
25
20
15
10
5
0

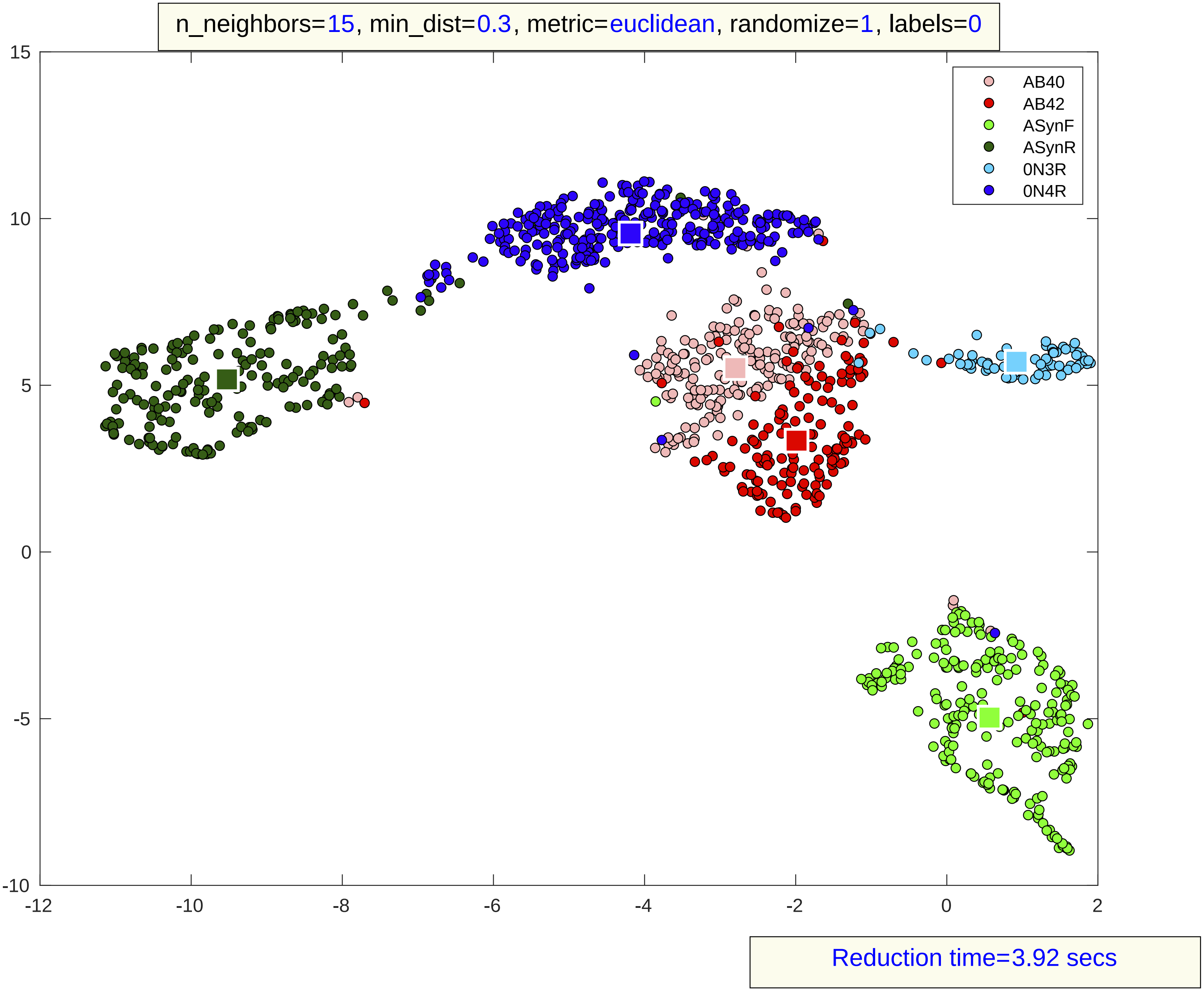

n_neighbors=15, min_dist=0.3, metric=euclidean, randomize=1, labels=0
AB40
AB42
ASynF
ASynR
0N3R
0N4R
15
10
5
0
-5
-10
-12
-10
-8
-6
-4
-2
0
2
Reduction time=3.92 secs

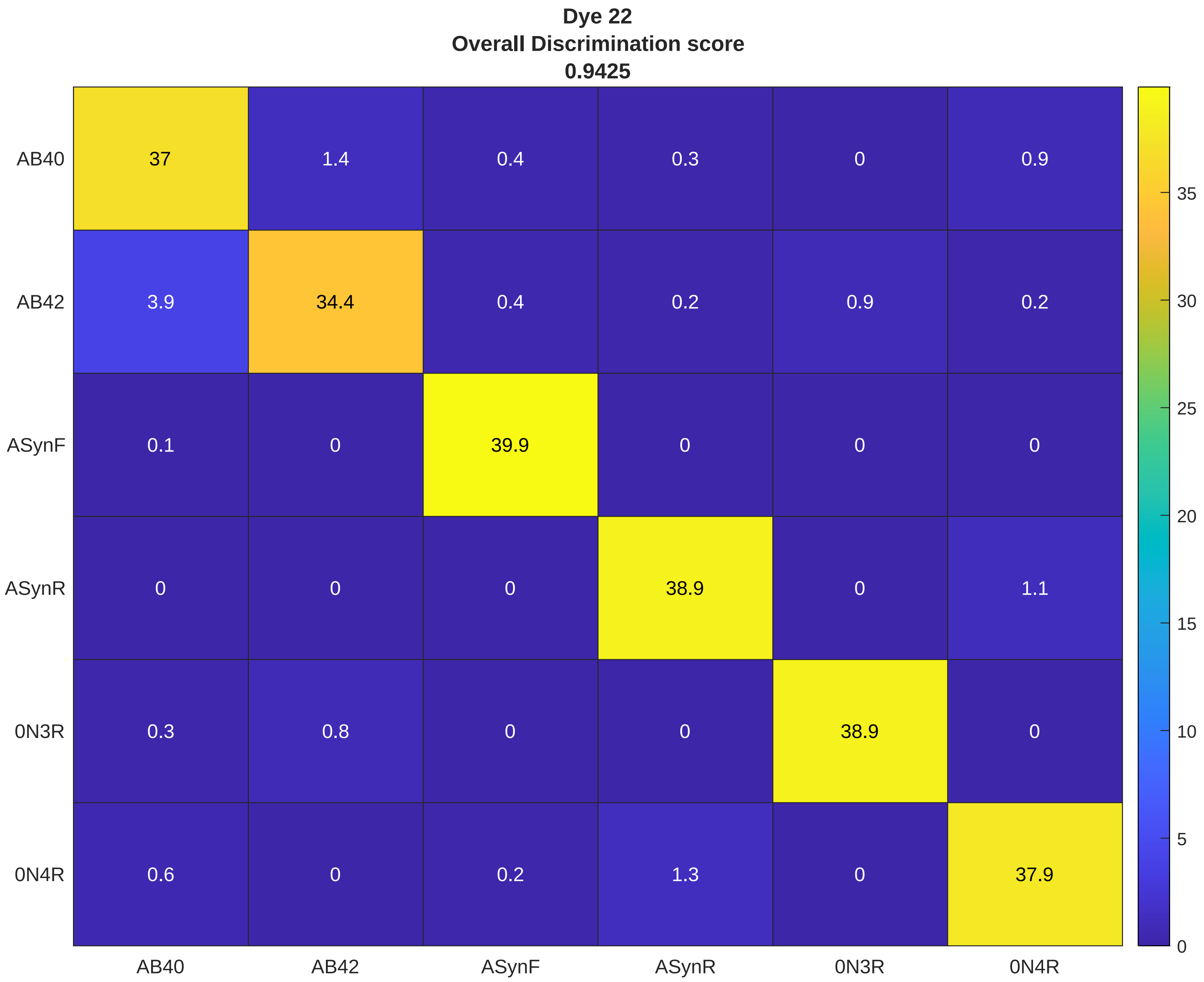
Dye 22
Overall Discrimination score
0.9425
AB40
AB42
ASynF
ASynR
0N3R
0N4R
37
1.4
0.4
0.3
0
0.9
3.9
34.4
0.4
0.2
0.9
0.2
0.1
0
39.9
0
0
0
0
0
0
38.9
0
1.1
0.3
0.8
0
0
38.9
0
0.6
0
0.2
1.3
0
37.9
AB40
AB42
ASynF
ASynR
0N3R
0N4R
35
30
25
20
15
10
5
0

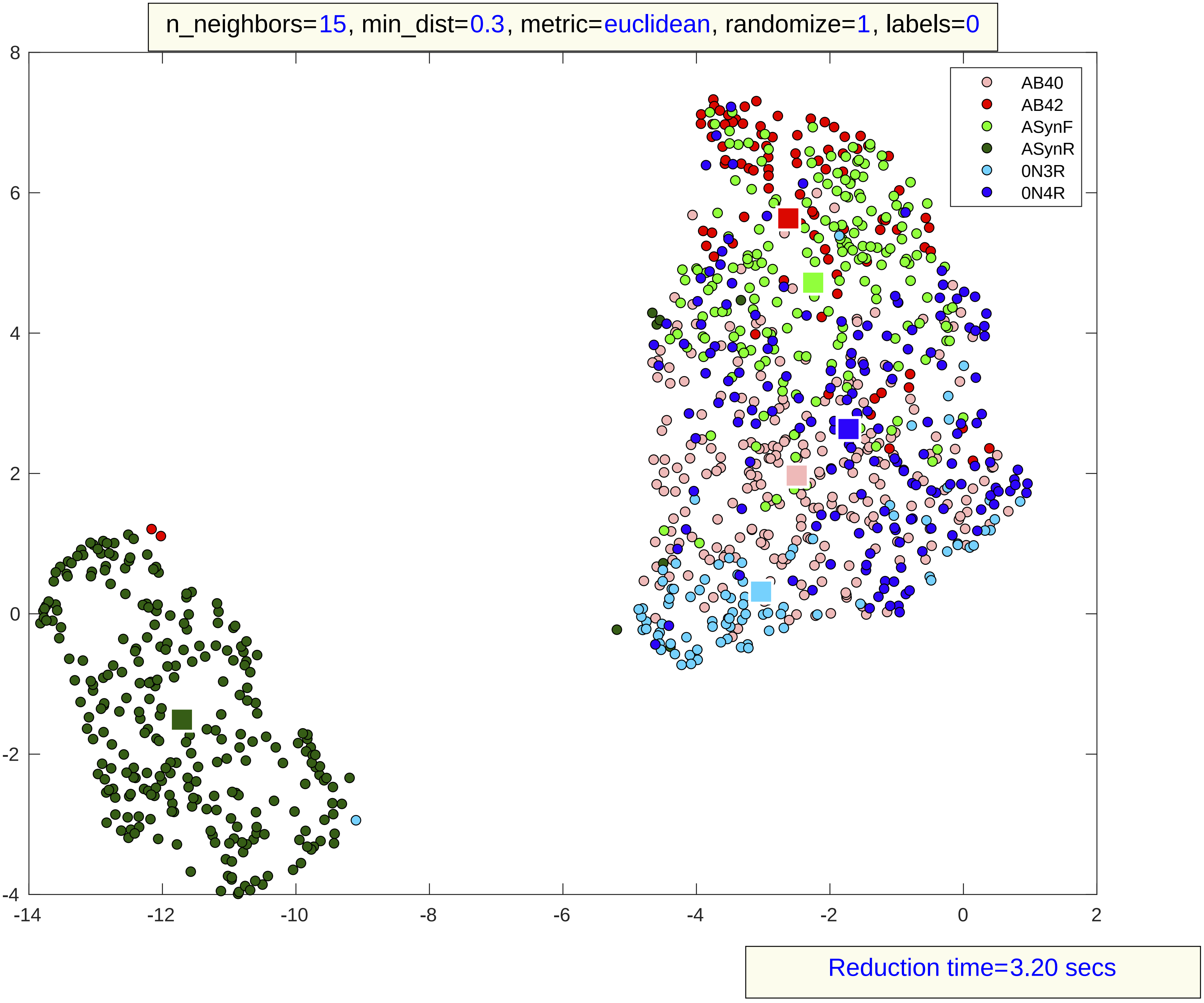
n_neighbors=15, min_dist=0.3, metric=euclidean, randomize=1, labels=0
AB40
AB42
ASynF
ASynR
0N3R
0N4R
8
6
4
2
0
-2
-4
-14
-12
-10
-8
-6
-4
-2
0
2
Reduction time=3.20 secs

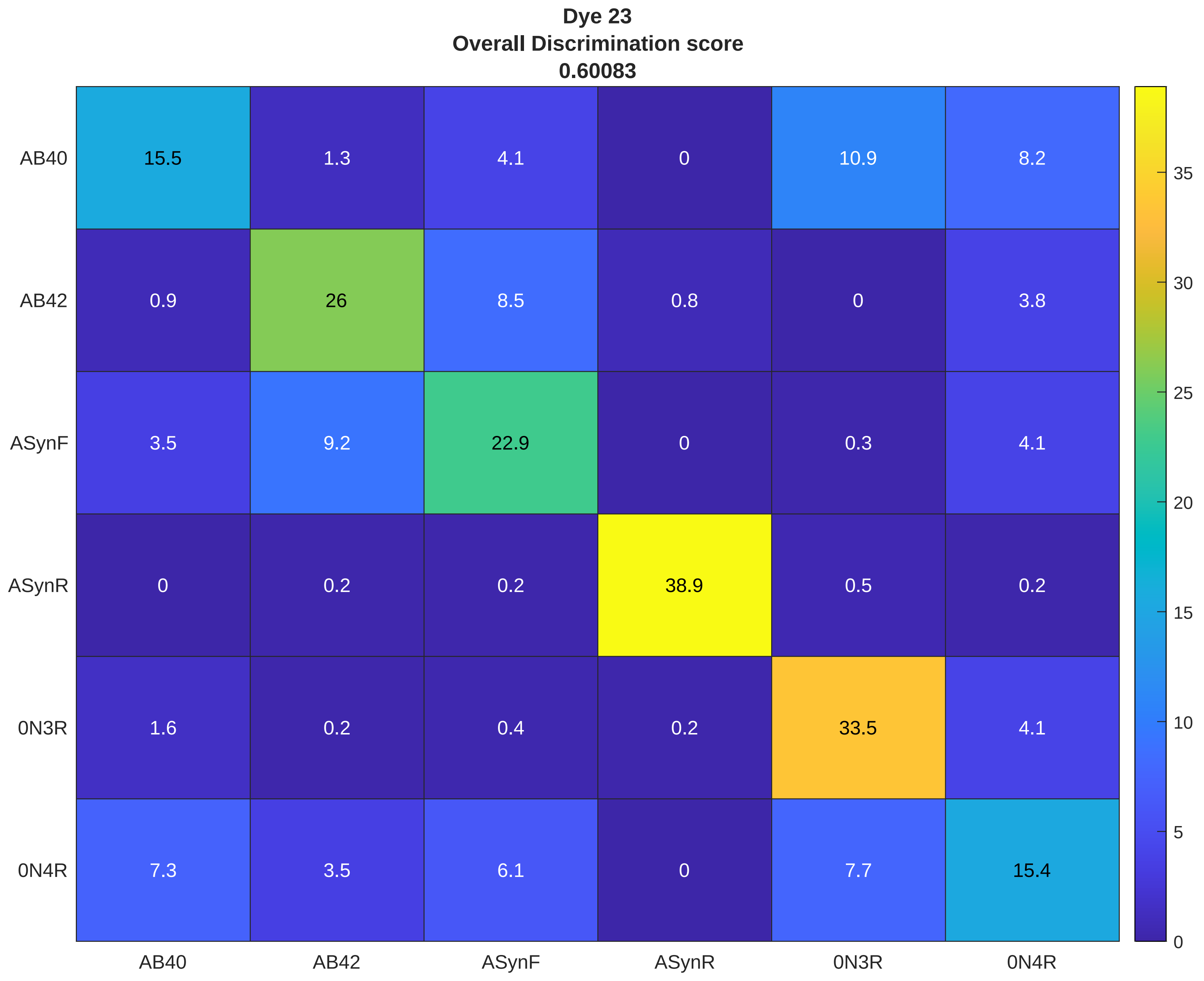
Dye 23
Overall Discrimination score
0.60083
AB40 15.5 1.3 4.1 0 10.9 8.2
AB42 0.9 26 8.5 0.8 0 3.8
ASynF 3.5 9.2 22.9 0 0.3 4.1
ASynR 0 0.2 0.2 38.9 0.5 0.2
0N3R 1.6 0.2 0.4 0.2 33.5 4.1
0N4R 7.3 3.5 6.1 0 7.7 15.4
AB40 AB42 ASynF ASynR 0N3R 0N4R
0 5 10 15 20 25 30 35

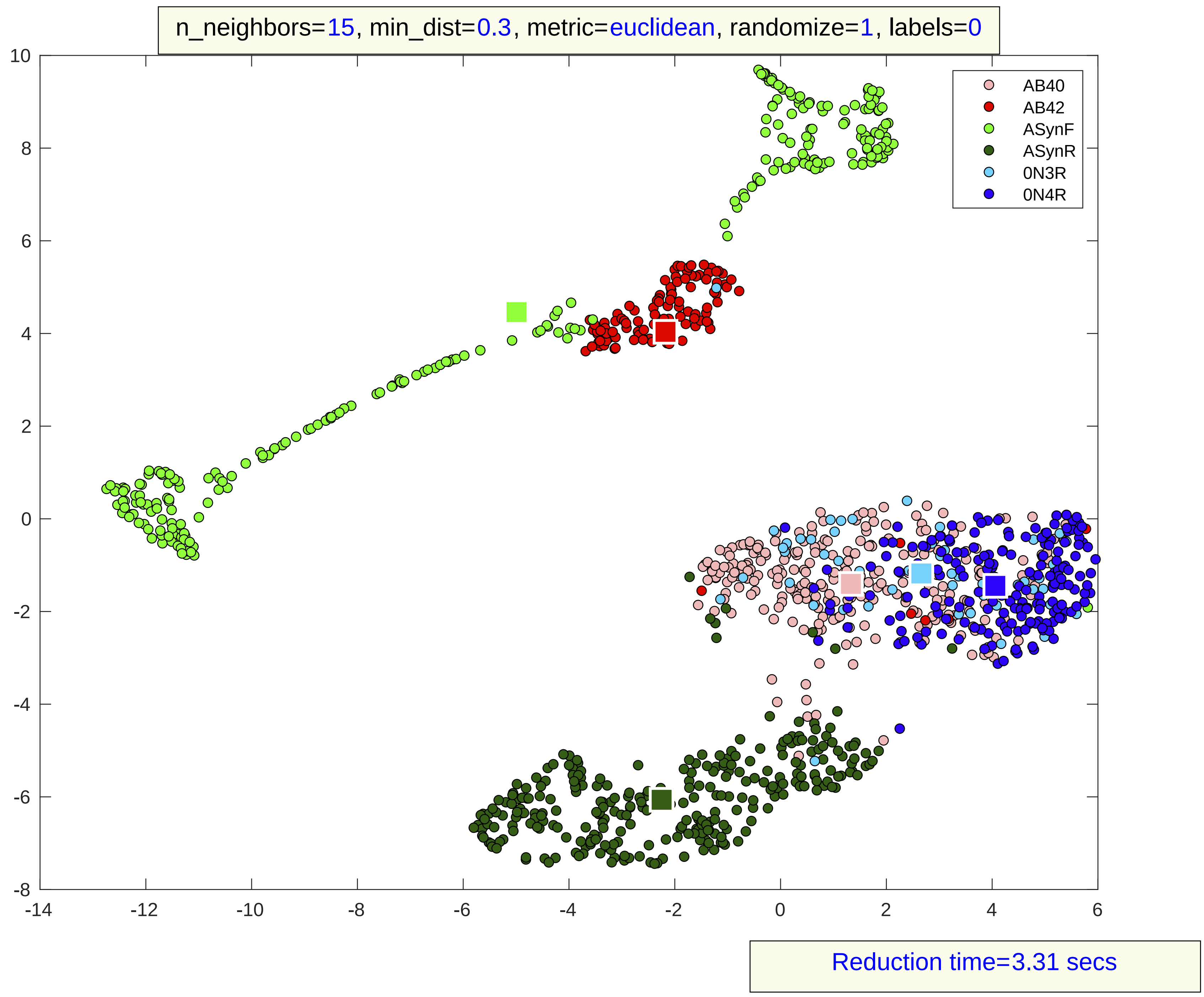

n_neighbors=15, min_dist=0.3, metric=euclidean, randomize=1, labels=0
AB40
AB42
ASynF
ASynR
0N3R
0N4R
10
8
6
4
2
0
-2
-4
-6
-8
-14
-12
-10
-8
-6
-4
-2
0
2
4
6
Reduction time=3.31 secs

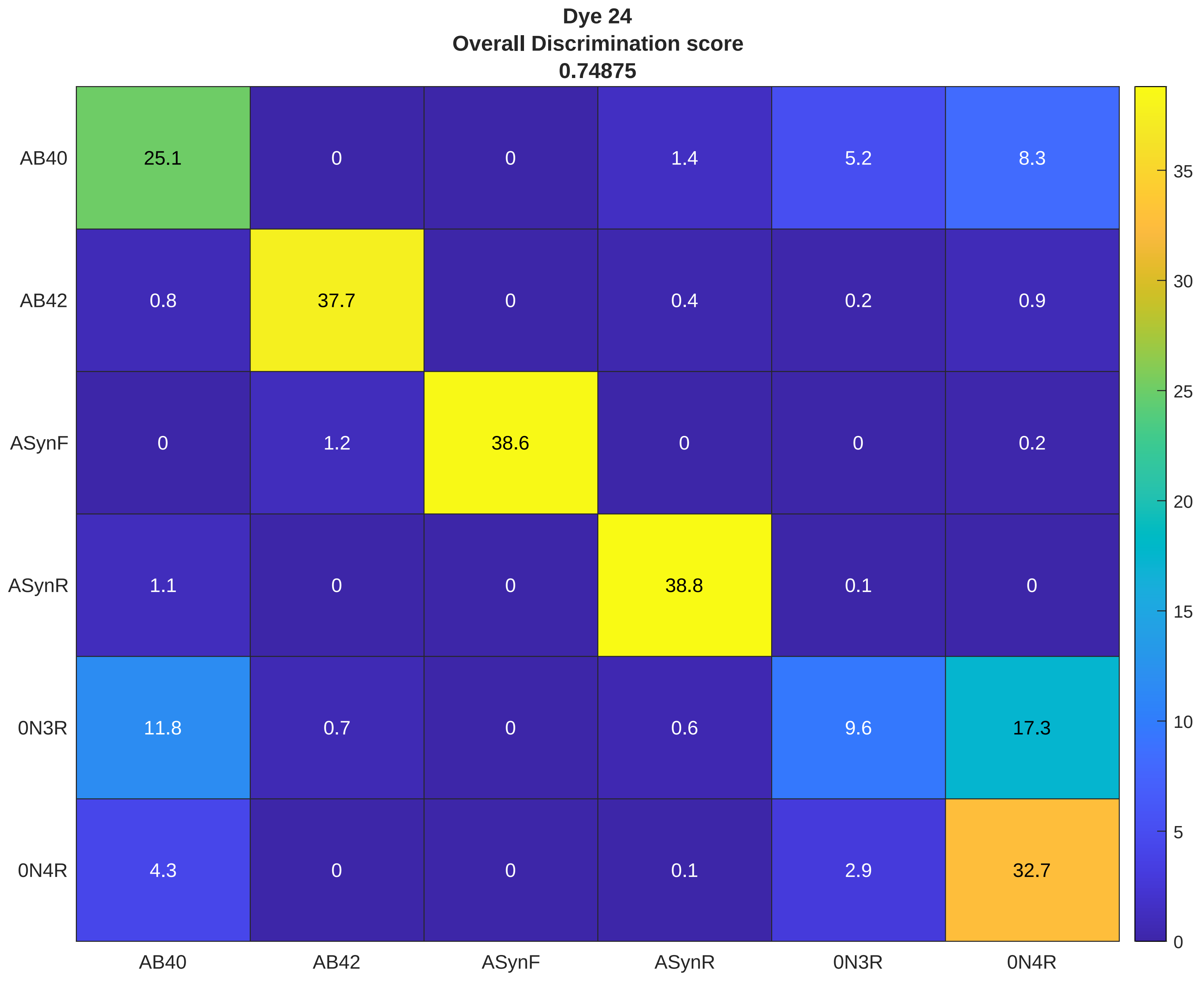
Dye 24
Overall Discrimination score
0.74875
AB40
AB42
ASynF
ASynR
0N3R
0N4R
25.1
0
0
1.4
5.2
8.3
0.8
37.7
0
0.4
0.2
0.9
0
1.2
38.6
0
0
0.2
1.1
0
0
38.8
0.1
0
11.8
0.7
0
0.6
9.6
17.3
4.3
0
0
0.1
2.9
32.7
AB40
AB42
ASynF
ASynR
0N3R
0N4R
35
30
25
20
15
10
5
0

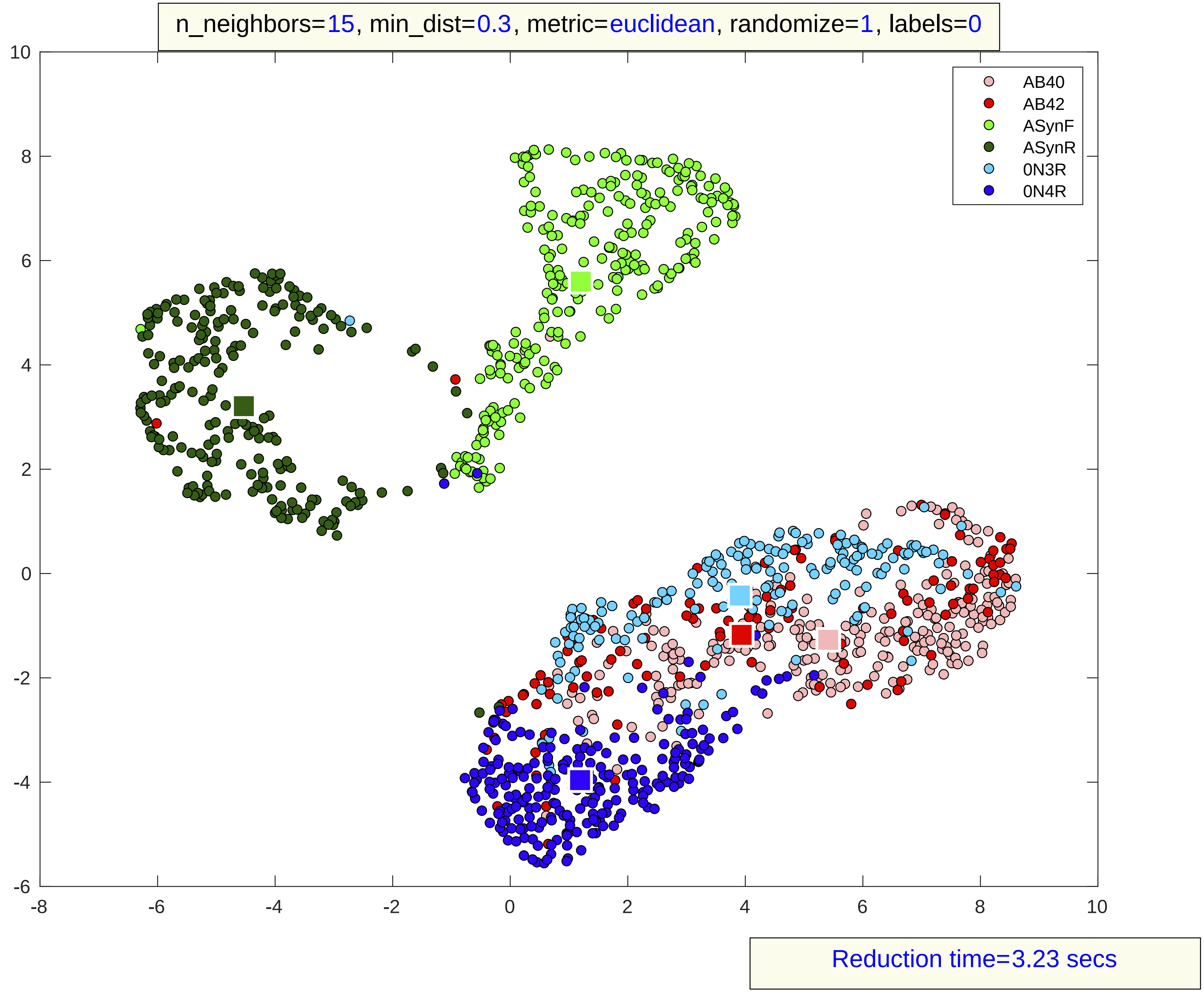
n_neighbors=15, min_dist=0.3, metric=euclidean, randomize=1, labels=0
AB40
AB42
ASynF
ASynR
0N3R
0N4R
10
8
6
4
2
0
-2
-4
-6
-8
-6
-4
-2
0
2
4
6
8
10
Reduction time=3.23 secs

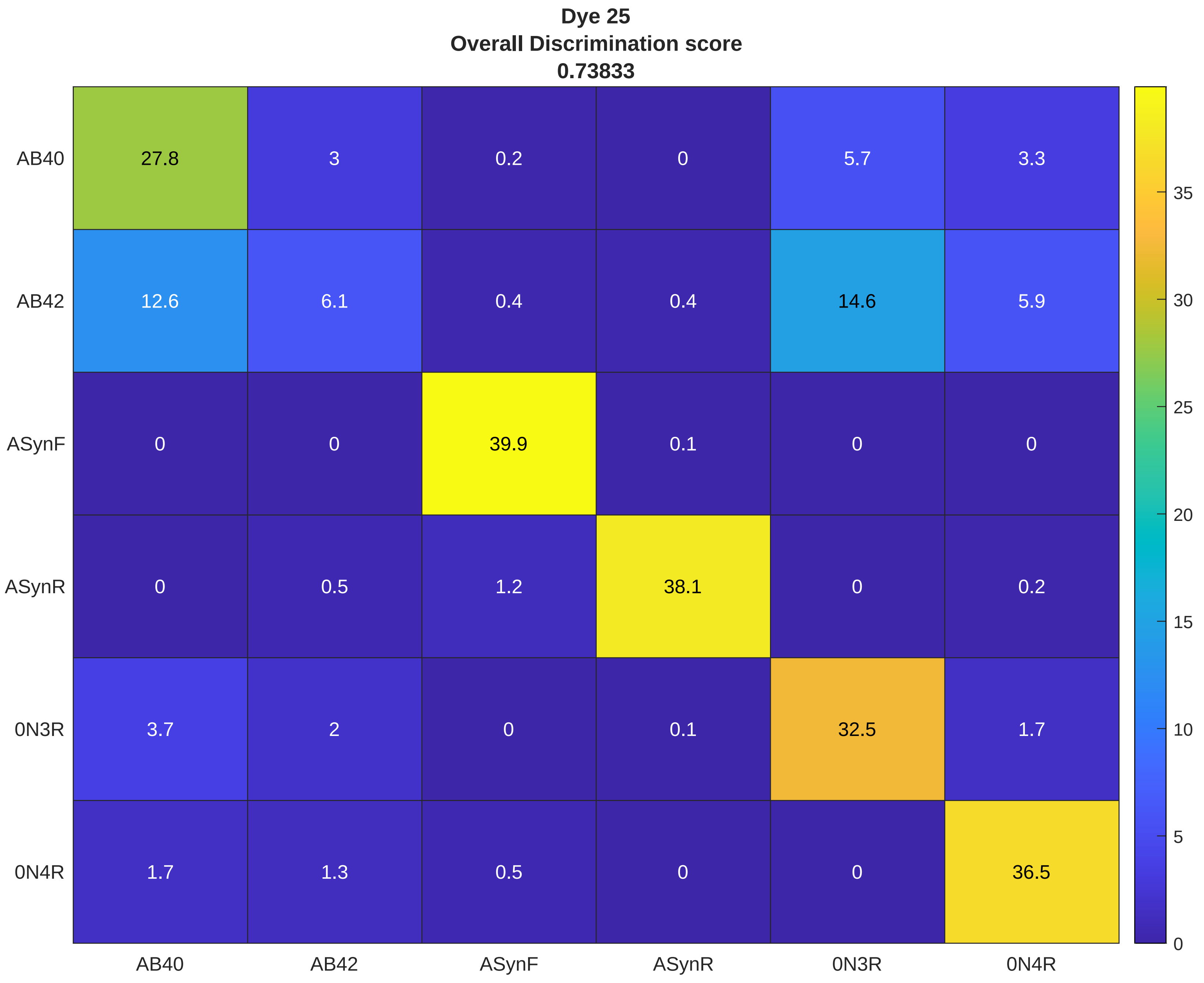

Dye 25
Overall Discrimination score
0.73833
AB40
AB42
ASynF
ASynR
0N3R
0N4R
27.8
3
0.2
0
5.7
3.3
12.6
6.1
0.4
0.4
14.6
5.9
0
0
39.9
0.1
0
0
0
0.5
1.2
38.1
0
0.2
3.7
2
0
0.1
32.5
1.7
1.7
1.3
0.5
0
0
36.5
AB40
AB42
ASynF
ASynR
0N3R
0N4R
35
30
25
20
15
10
5
0

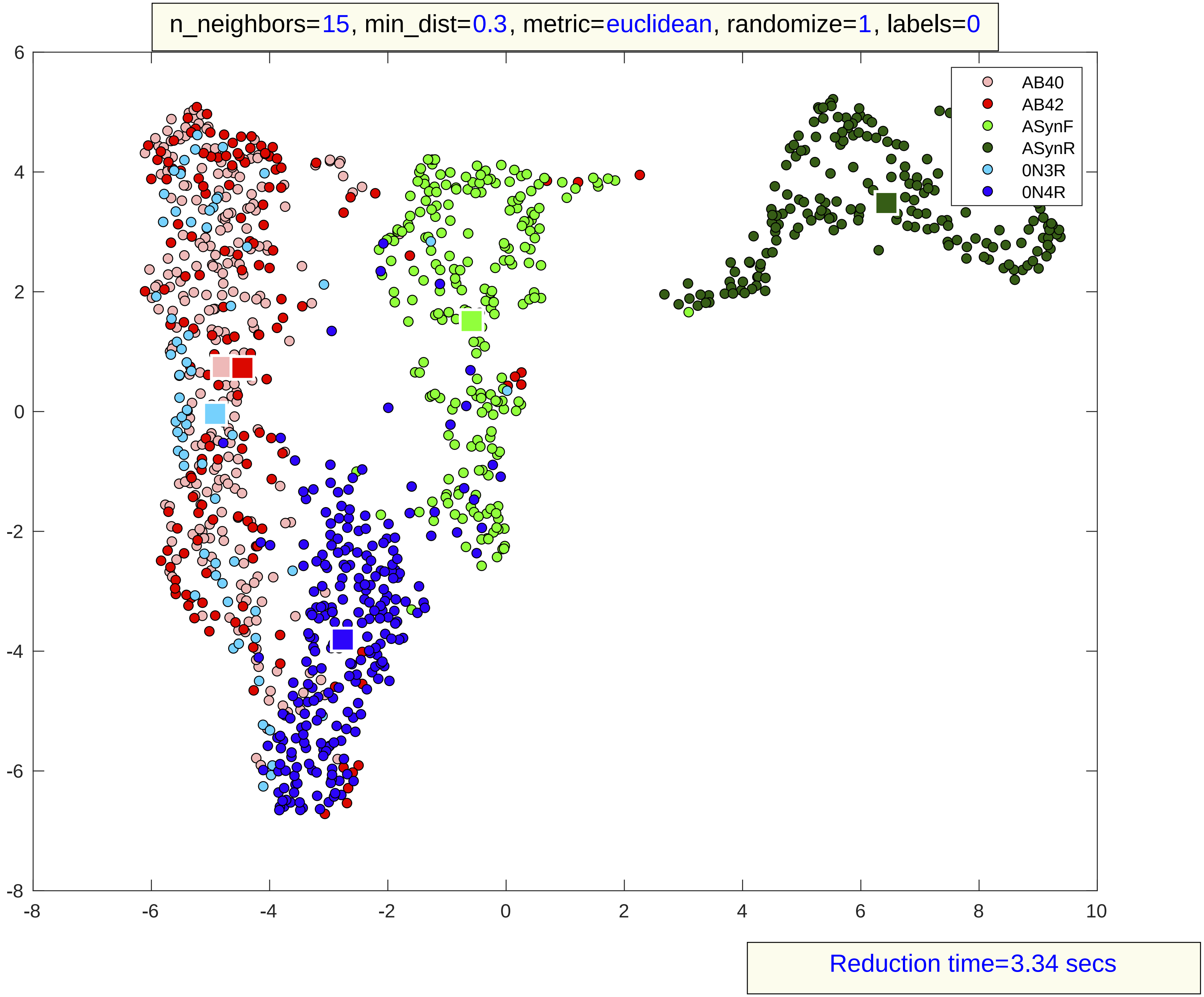

n_neighbors=15, min_dist=0.3, metric=euclidean, randomize=1, labels=0
AB40
AB42
ASynF
ASynR
0N3R
0N4R
6
4
2
0
-2
-4
-6
-8
-8
-6
-4
-2
0
2
4
6
8
10
Reduction time=3.34 secs

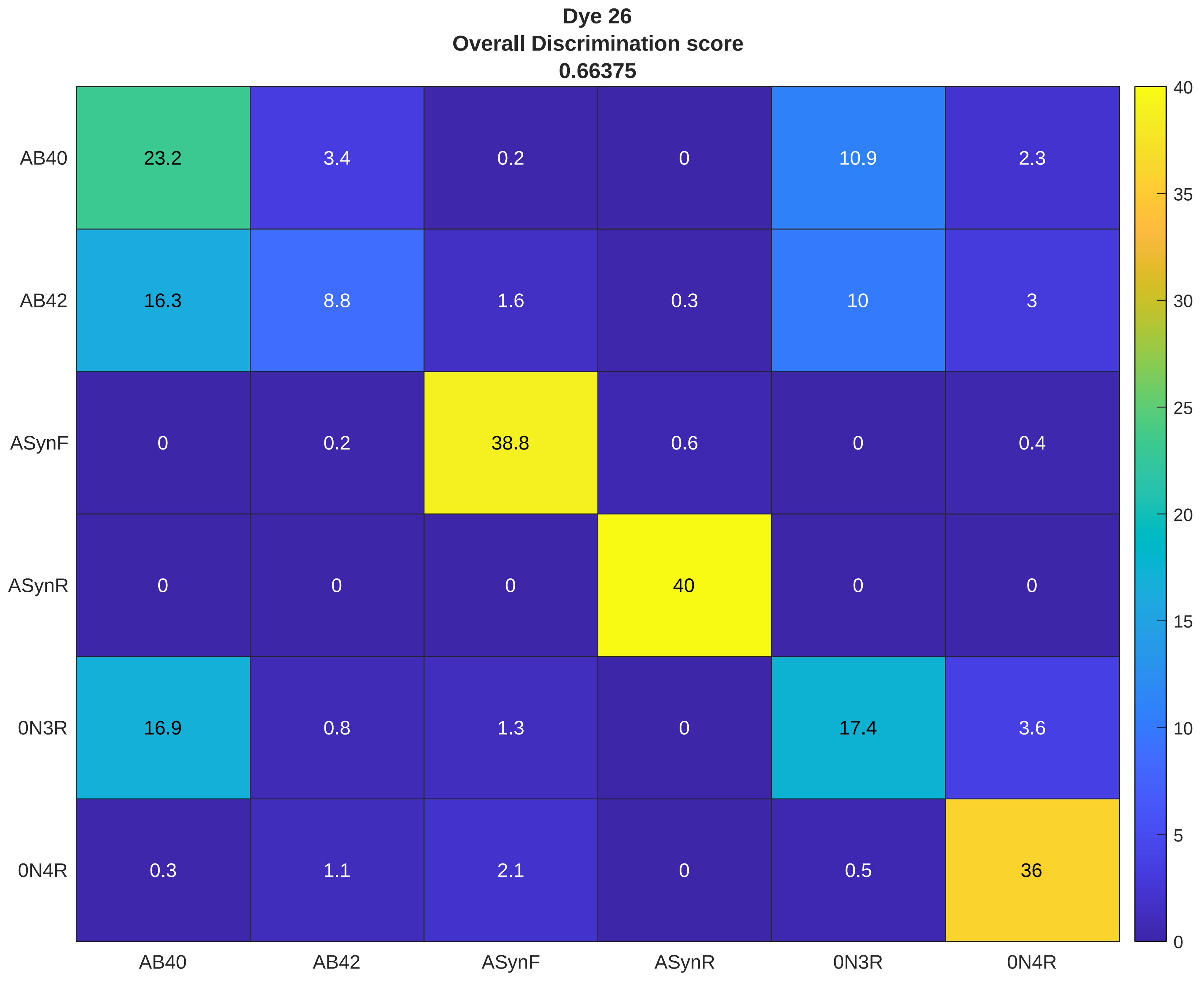
Dye 26
Overall Discrimination score
0.66375
AB40
AB42
ASynF
ASynR
0N3R
0N4R
23.2
3.4
0.2
0
10.9
2.3
16.3
8.8
1.6
0.3
10
3
0
0.2
38.8
0.6
0
0.4
0
0
0
40
0
0
16.9
0.8
1.3
0
17.4
3.6
0.3
1.1
2.1
0
0.5
36
AB40
AB42
ASynF
ASynR
0N3R
0N4R
40
35
30
25
20
15
10
5
0

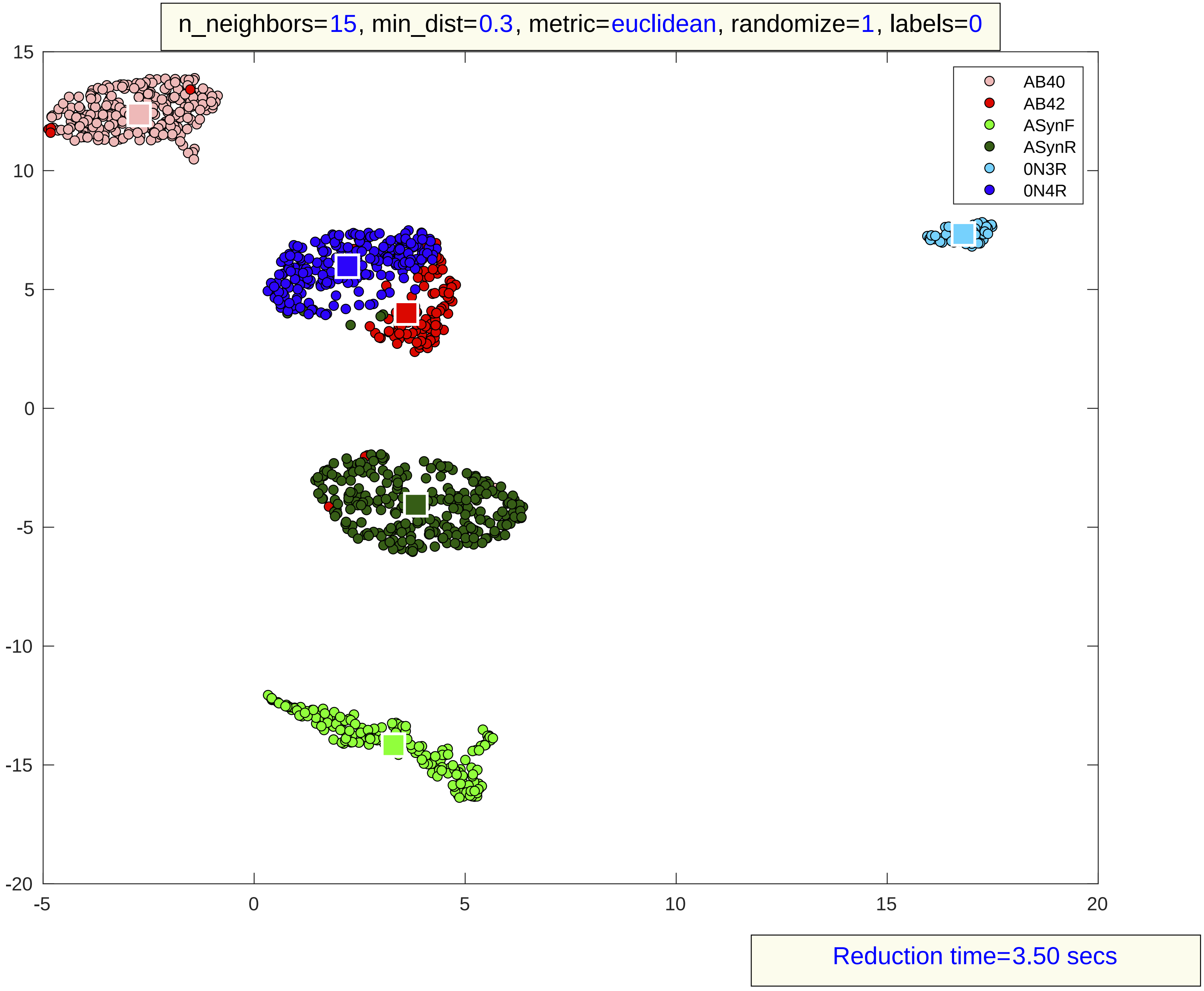

n_neighbors=15, min_dist=0.3, metric=euclidean, randomize=1, labels=0
15
10
5
0
-5
-10
-15
-20
-5
0
5
10
15
20
AB40
AB42
ASynF
ASynR
0N3R
0N4R
Reduction time=3.50 secs

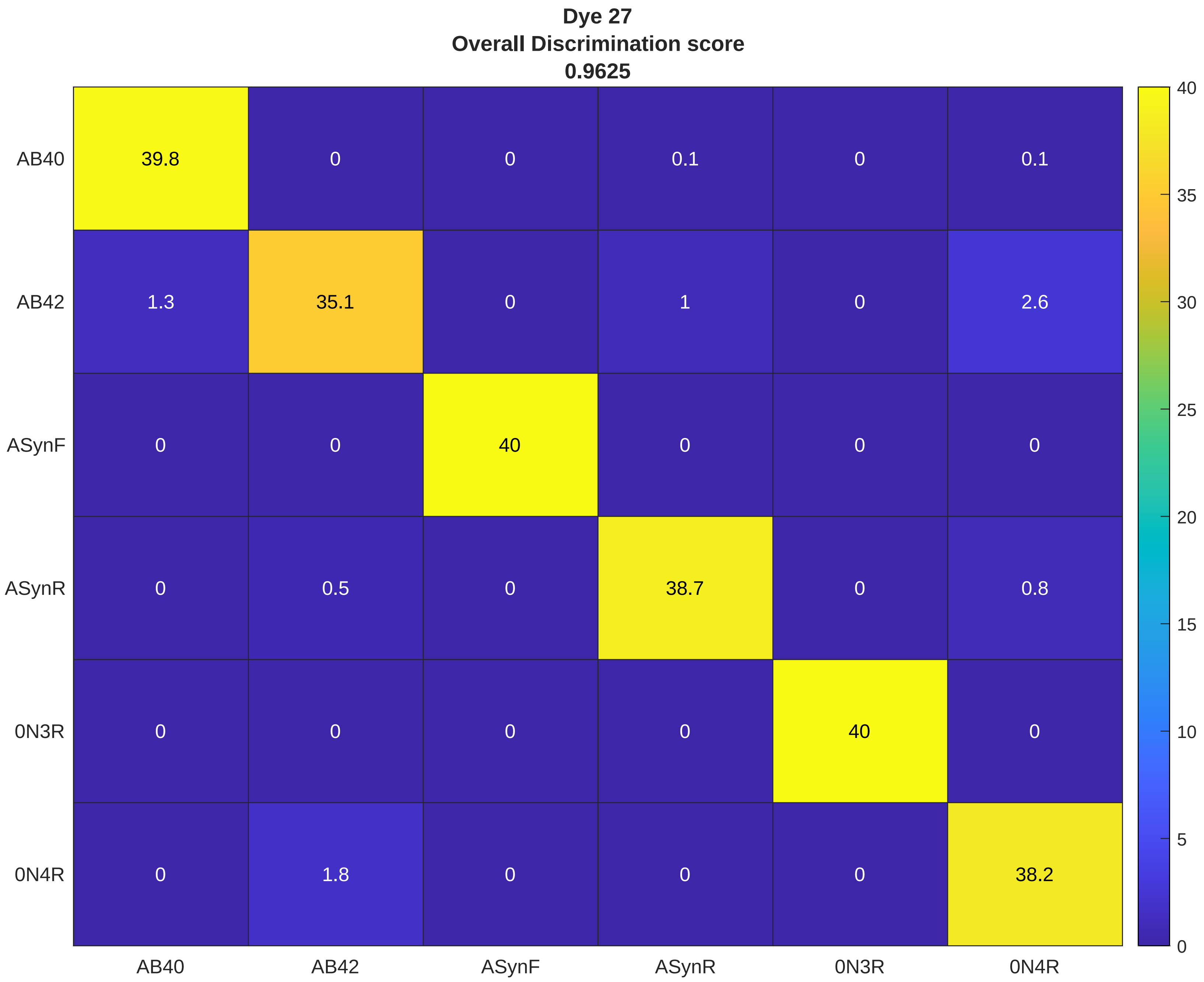

Dye 27
Overall Discrimination score
0.9625
AB40
AB42
ASynF
ASynR
0N3R
0N4R
39.8
0
0
0.1
0
0.1
1.3
35.1
0
1
0
2.6
0
0
40
0
0
0
0
0.5
0
38.7
0
0.8
0
0
0
0
40
0
0
1.8
0
0
0
38.2
AB40
AB42
ASynF
ASynR
0N3R
0N4R
40
35
30
25
20
15
10
5
0

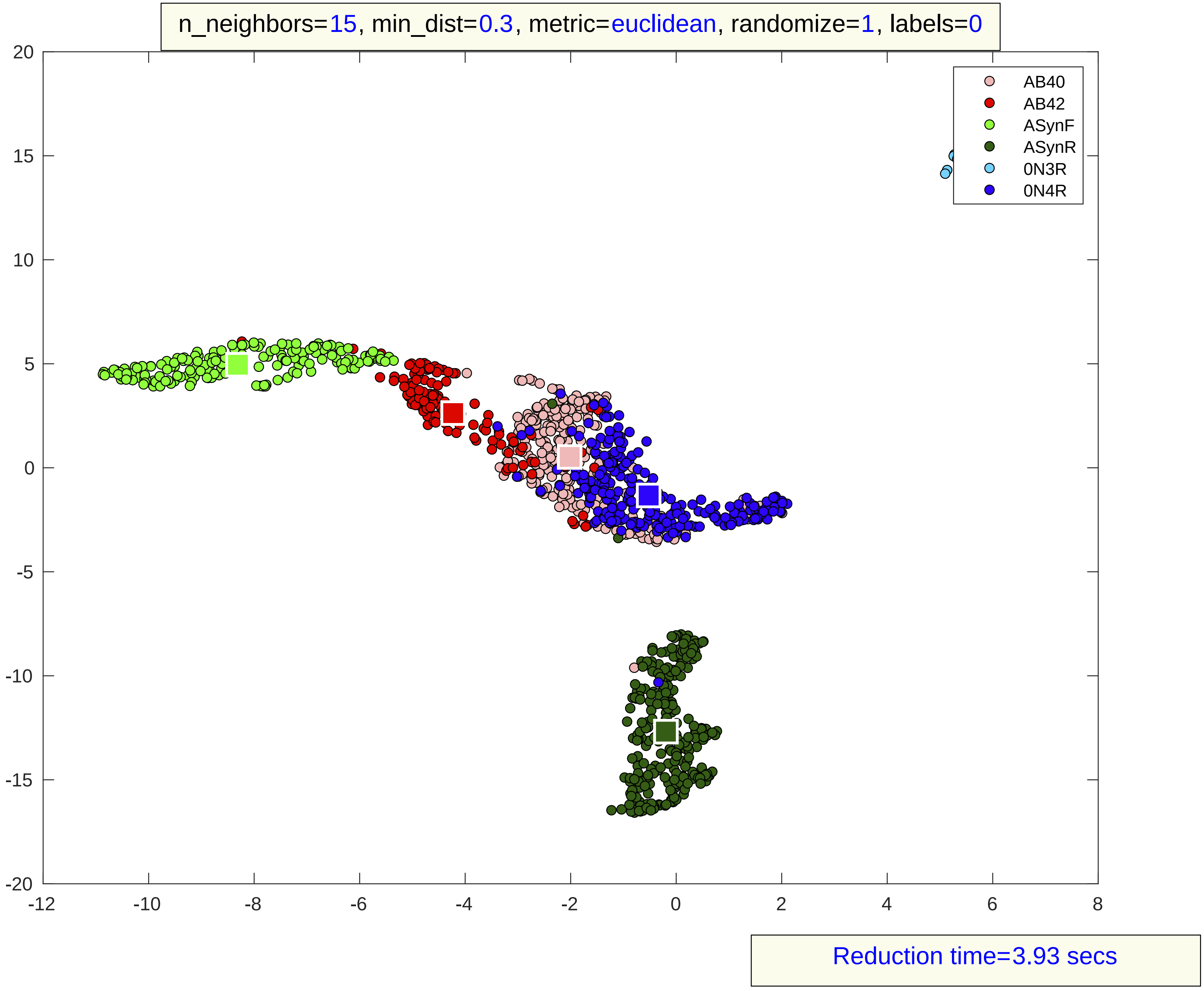

n_neighbors=15, min_dist=0.3, metric=euclidean, randomize=1, labels=0
AB40
AB42
ASynF
ASynR
0N3R
0N4R
20
15
10
5
0
-5
-10
-15
-20
-12
-10
-8
-6
-4
-2
0
2
4
6
8
Reduction time=3.93 secs

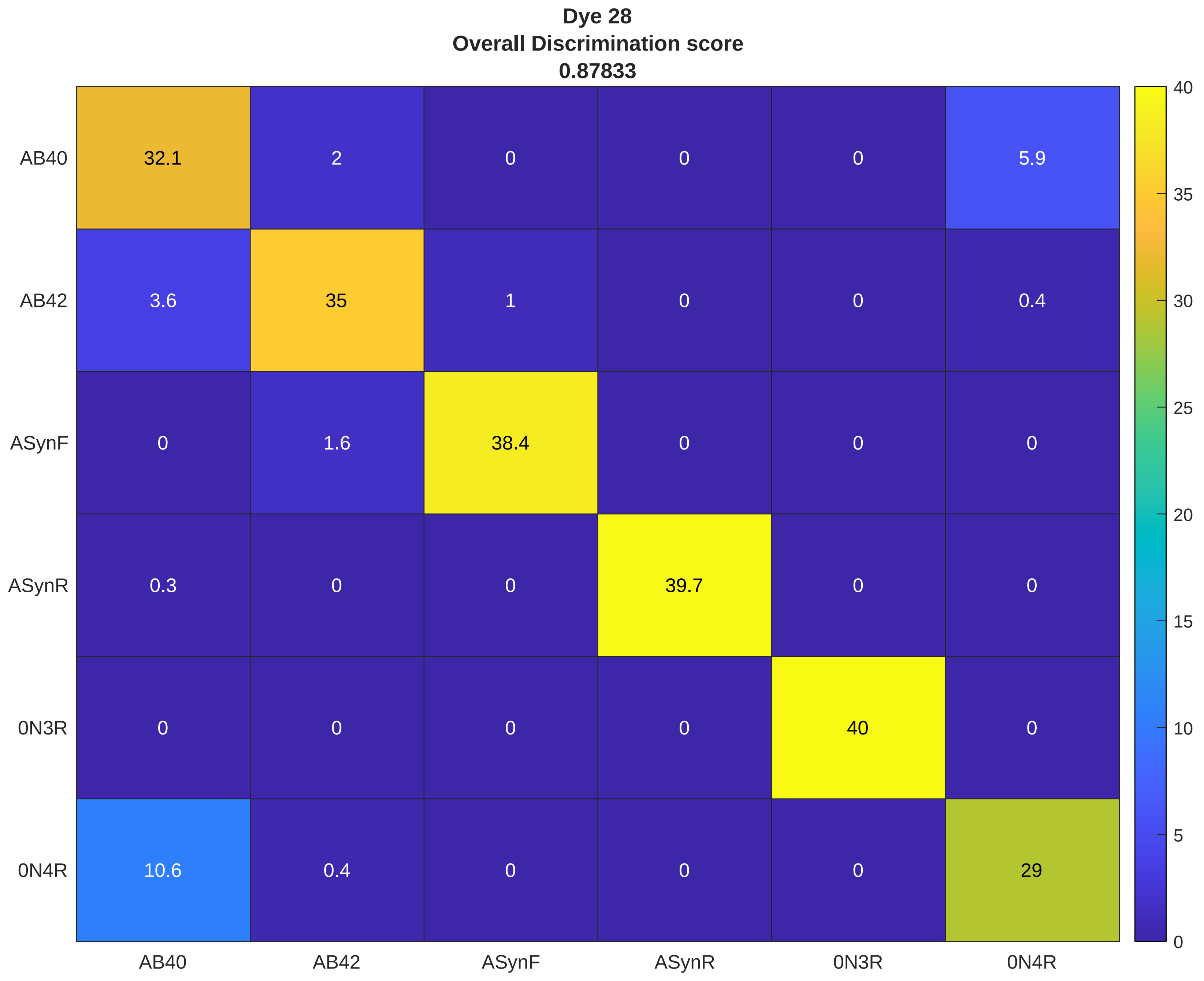
Dye 28
Overall Discrimination score
0.87833
AB40
AB42
ASynF
ASynR
0N3R
0N4R
32.1
2
0
0
0
5.9
3.6
35
1
0
0
0.4
0
1.6
38.4
0
0
0
0.3
0
0
39.7
0
0
0
0
0
0
40
0
10.6
0.4
0
0
0
29
AB40
AB42
ASynF
ASynR
0N3R
0N4R
40
35
30
25
20
15
10
5
0

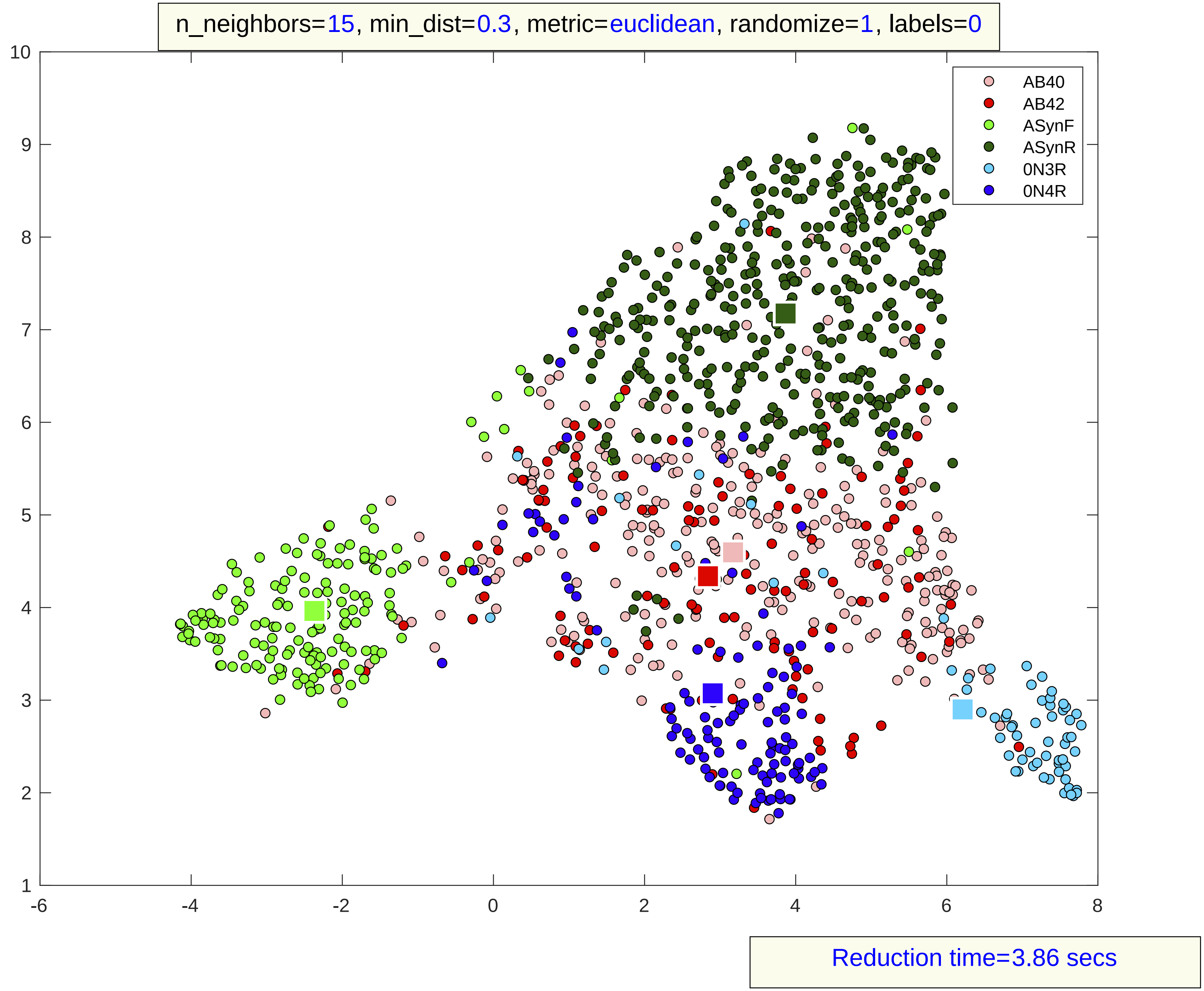

n_neighbors=15, min_dist=0.3, metric=euclidean, randomize=1, labels=0
AB40
AB42
ASynF
ASynR
0N3R
0N4R
10
9
8
7
6
5
4
3
2
1
-6
-4
-2
0
2
4
6
8
Reduction time=3.86 secs

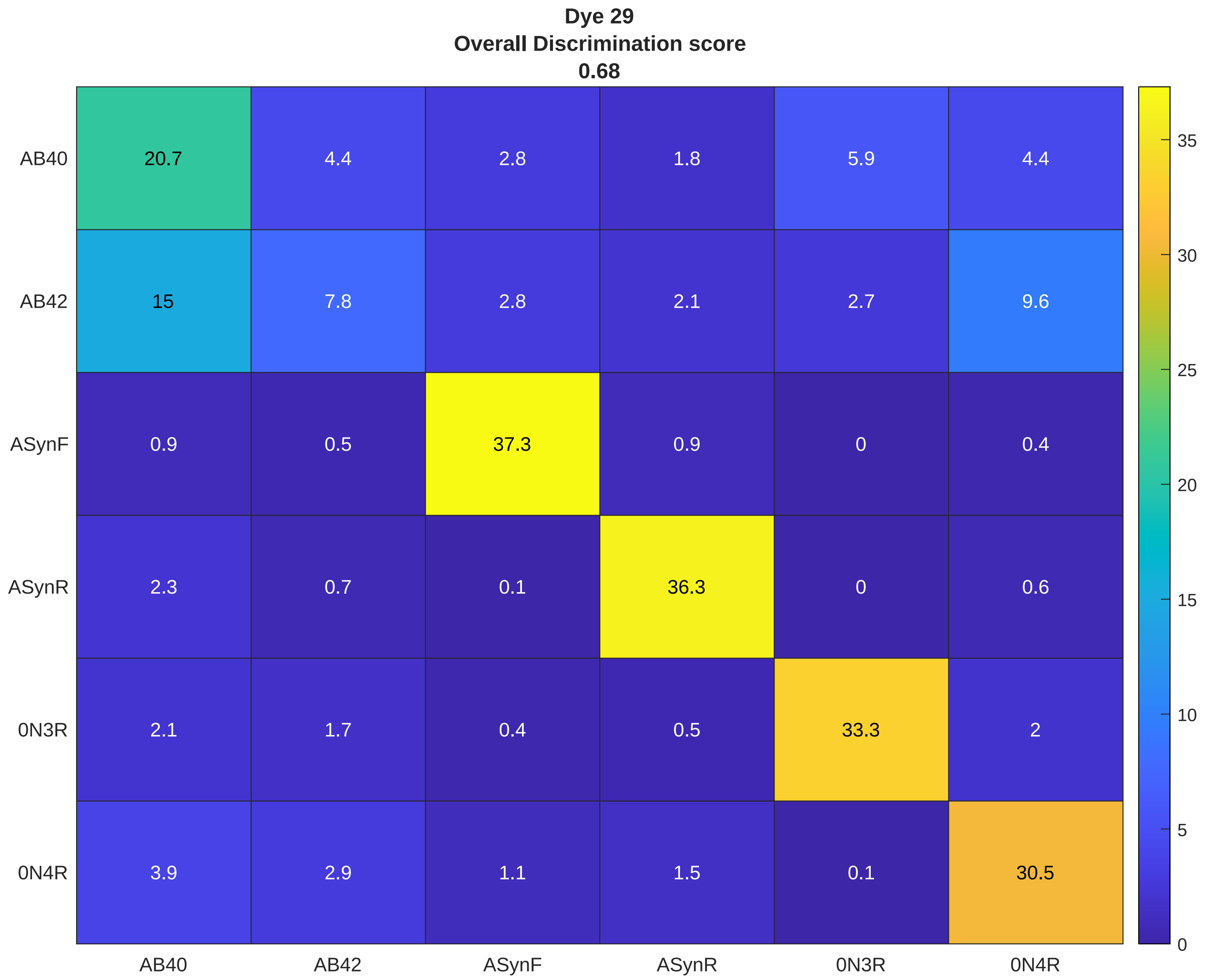
Dye 29
Overall Discrimination score
0.68
AB40
AB42
ASynF
ASynR
0N3R
0N4R
20.7
4.4
2.8
1.8
5.9
4.4
15
7.8
2.8
2.1
2.7
9.6
0.9
0.5
37.3
0.9
0
0.4
2.3
0.7
0.1
36.3
0
0.6
2.1
1.7
0.4
0.5
33.3
2
3.9
2.9
1.1
1.5
0.1
30.5
AB40
AB42
ASynF
ASynR
0N3R
0N4R
35
30
25
20
15
10
5
0

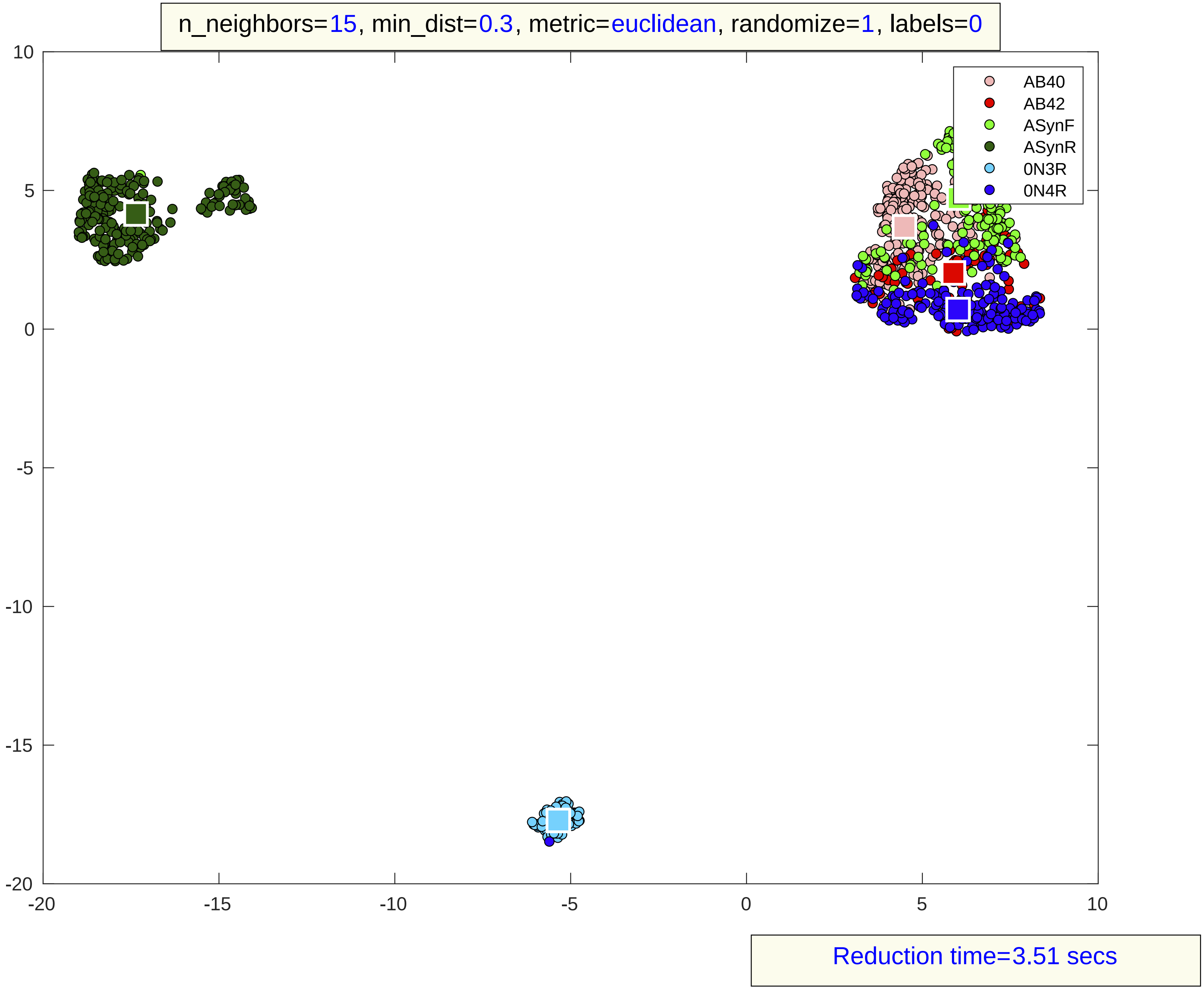
n_neighbors=15, min_dist=0.3, metric=euclidean, randomize=1, labels=0
10
5
0
-5
-10
-15
-20
-20
-15
-10
-5
0
5
10
AB40
AB42
ASynF
ASynR
0N3R
0N4R
Reduction time=3.51 secs

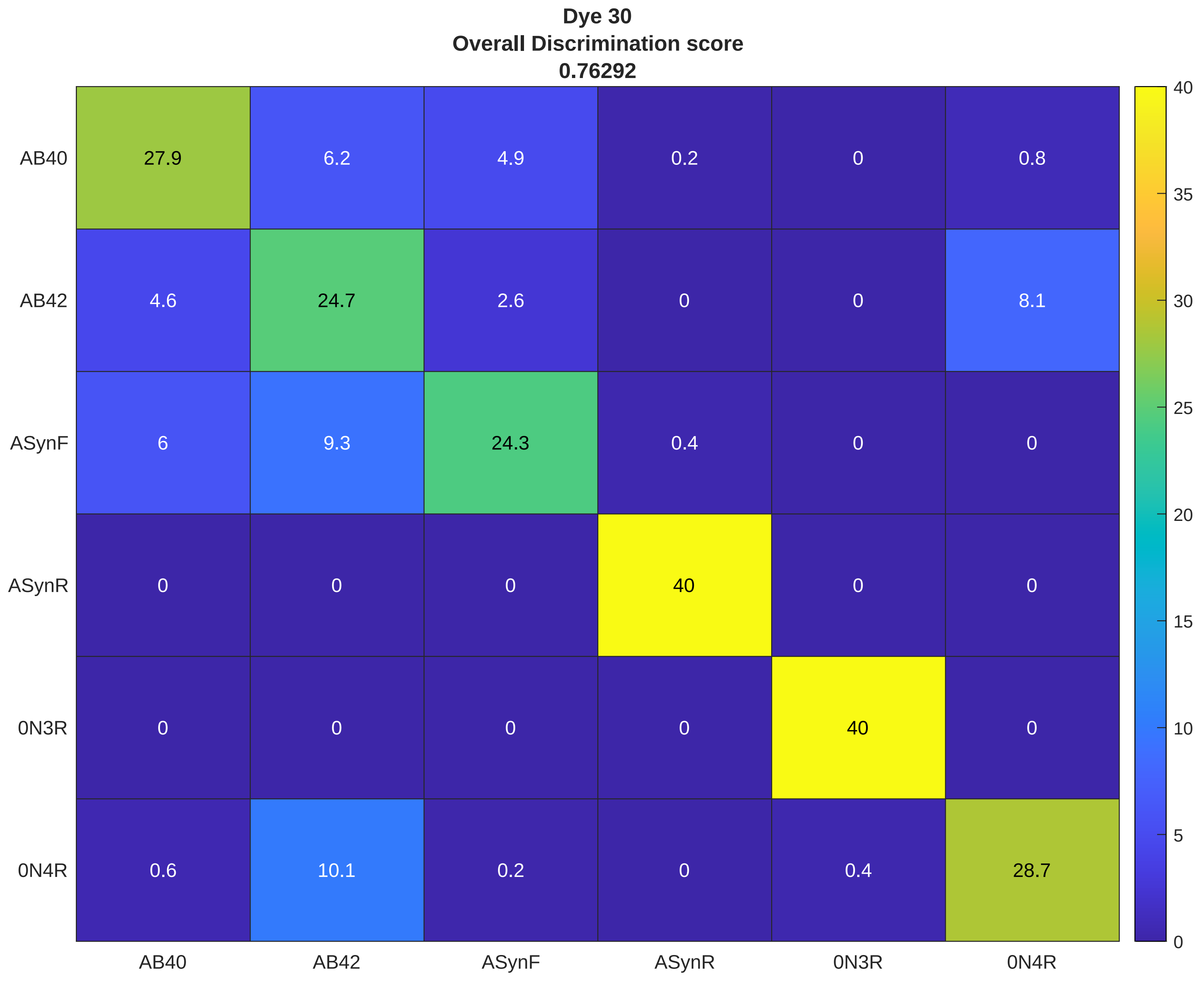
Dye 30
Overall Discrimination score
0.76292
AB40
AB42
ASynF
ASynR
0N3R
0N4R
27.9
6.2
4.9
0.2
0
0.8
4.6
24.7
2.6
0
0
8.1
6
9.3
24.3
0.4
0
0
0
0
0
40
0
0
0
0
0
0
40
0
0.6
10.1
0.2
0
0.4
28.7
AB40
AB42
ASynF
ASynR
0N3R
0N4R
40
35
30
25
20
15
10
5
0

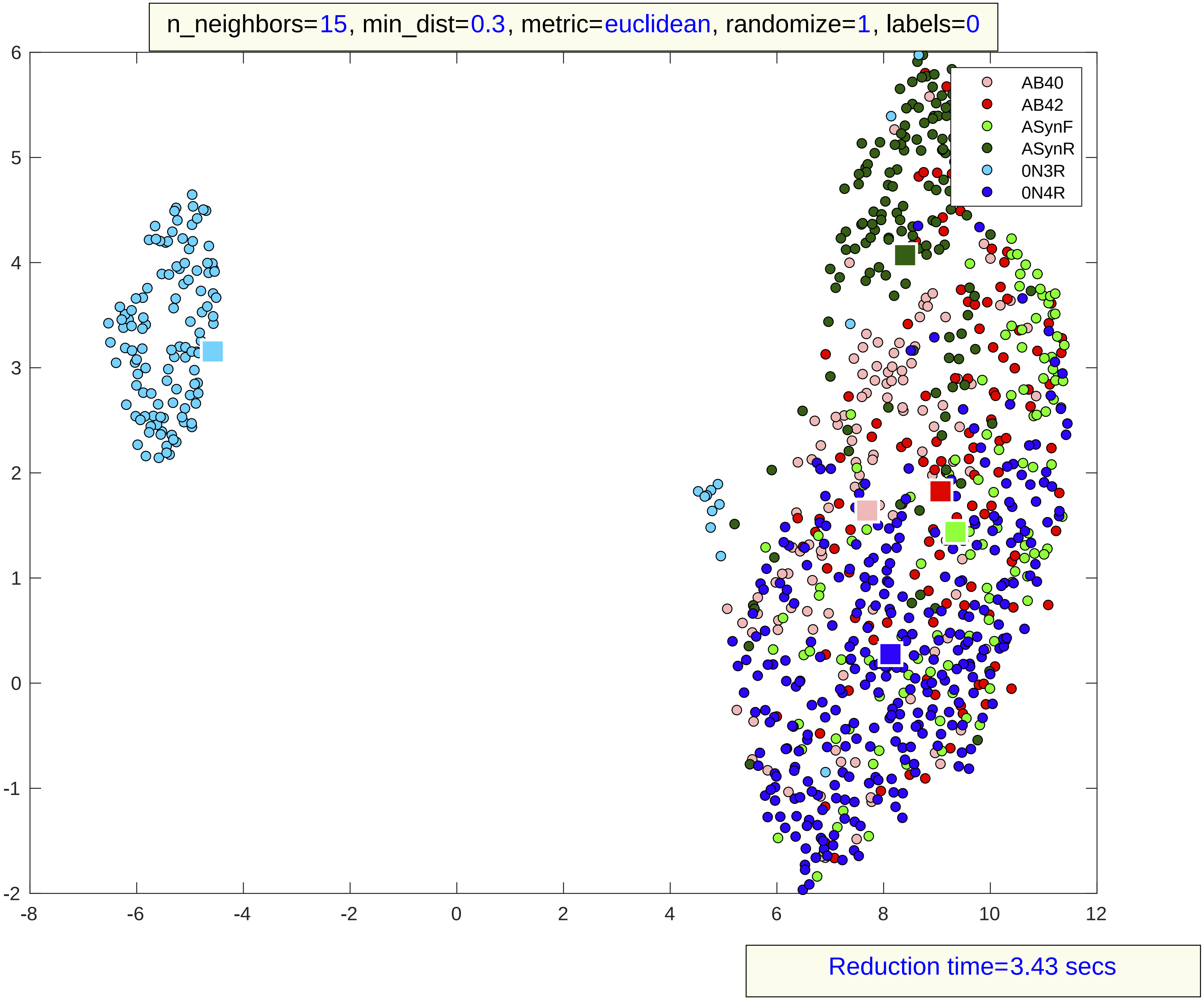

n_neighbors=15, min_dist=0.3, metric=euclidean, randomize=1, labels=0
AB40
AB42
ASynF
ASynR
0N3R
0N4R
6
5
4
3
2
1
0
-1
-2
-8
-6
-4
-2
0
2
4
6
8
10
12
Reduction time=3.43 secs

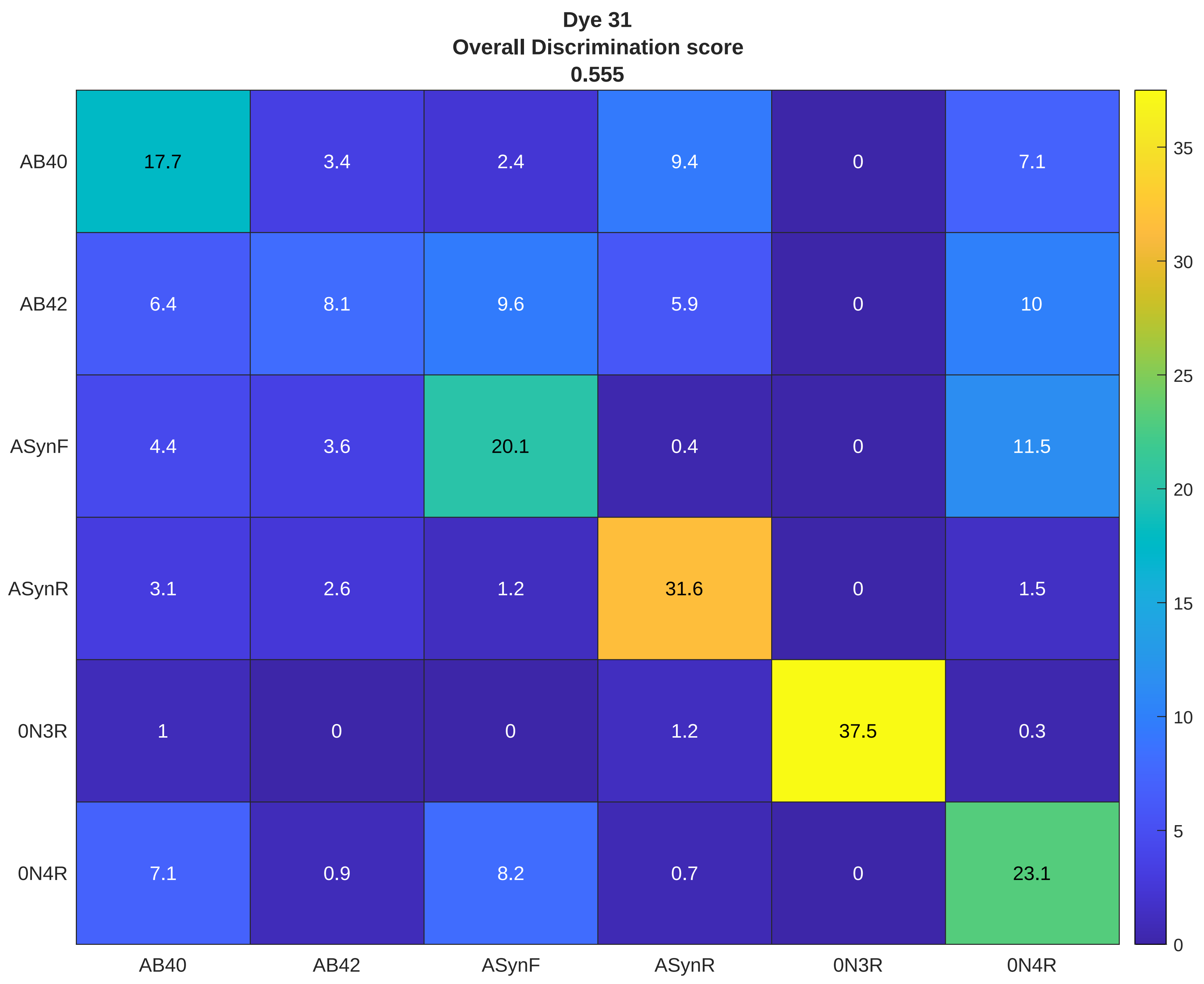

Dye 31
Overall Discrimination score
0.555
AB40
AB42
ASynF
ASynR
0N3R
0N4R
17.7
3.4
2.4
9.4
0
7.1
6.4
8.1
9.6
5.9
0
10
4.4
3.6
20.1
0.4
0
11.5
3.1
2.6
1.2
31.6
0
1.5
1
0
0
1.2
37.5
0.3
7.1
0.9
8.2
0.7
0
23.1
AB40
AB42
ASynF
ASynR
0N3R
0N4R
35
30
25
20
15
10
5
0

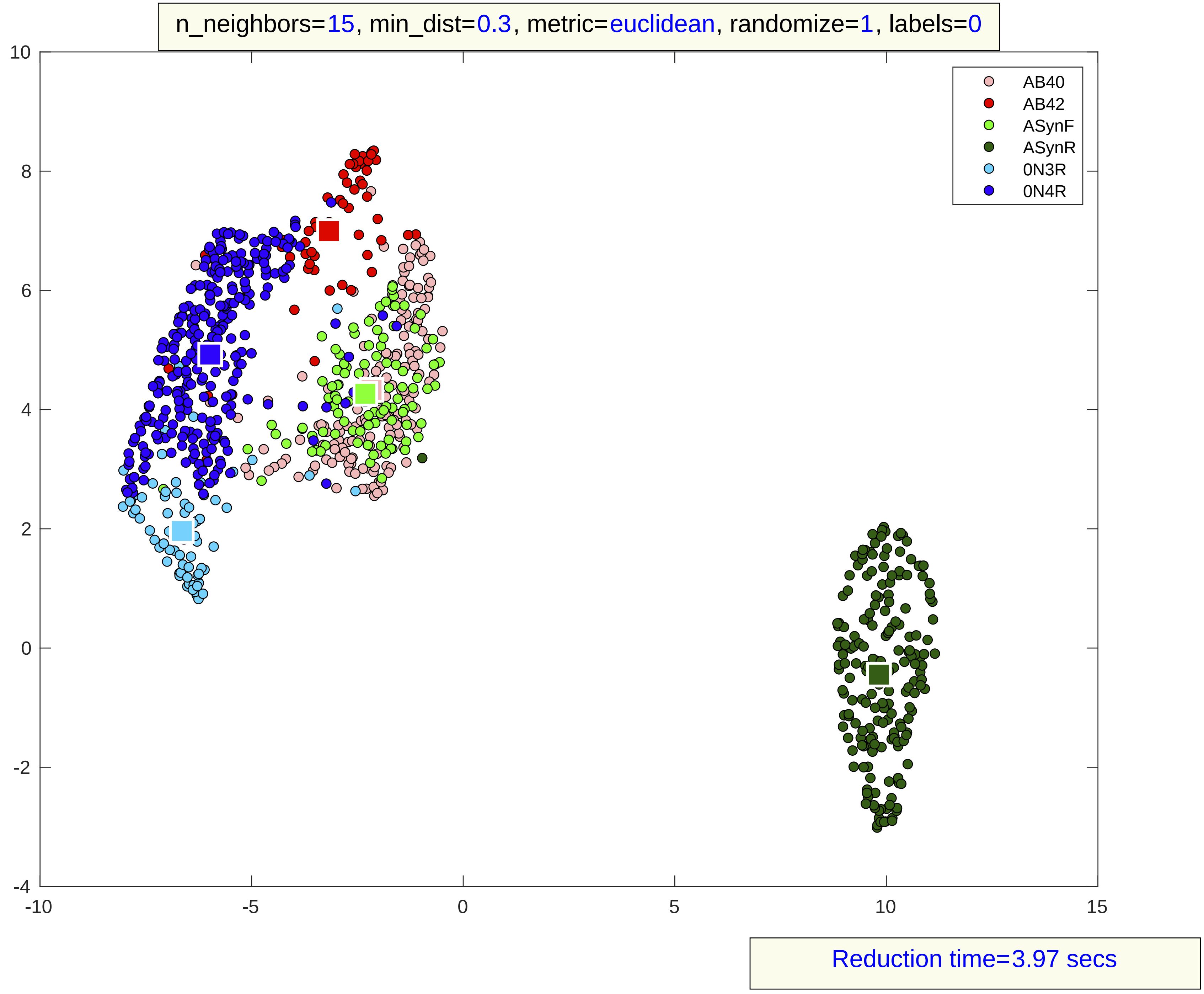
n_neighbors=15, min_dist=0.3, metric=euclidean, randomize=1, labels=0
AB40
AB42
ASynF
ASynR
0N3R
0N4R
10
8
6
4
2
0
-2
-4
-10
-5
0
5
10
15
Reduction time=3.97 secs

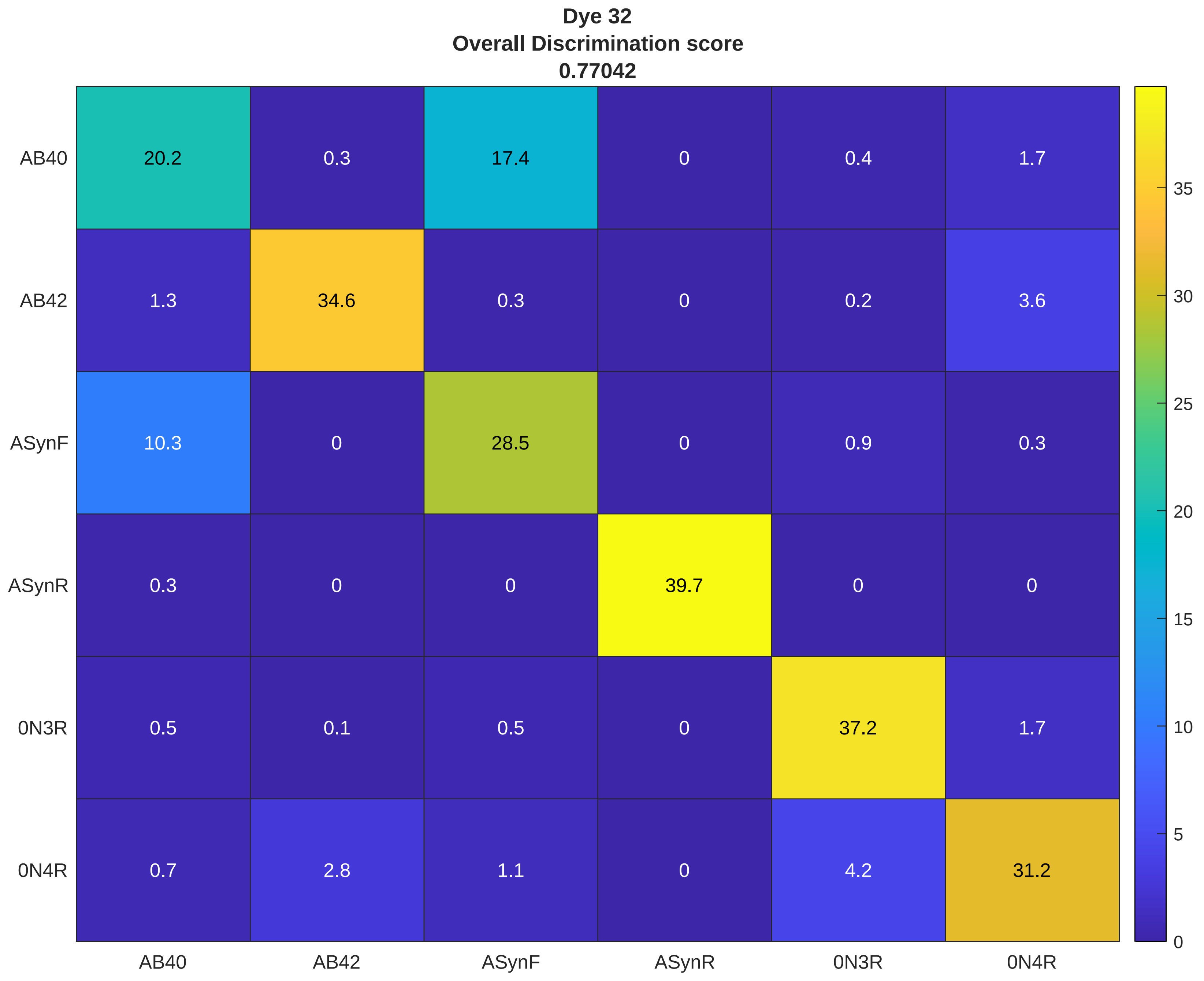
Dye 32
Overall Discrimination score
0.77042
AB40
AB42
ASynF
ASynR
0N3R
0N4R
20.2 0.3 17.4 0 0.4 1.7
1.3 34.6 0.3 0 0.2 3.6
10.3 0 28.5 0 0.9 0.3
0.3 0 0 39.7 0 0
0.5 0.1 0.5 0 37.2 1.7
0.7 2.8 1.1 0 4.2 31.2
AB40
AB42
ASynF
ASynR
0N3R
0N4R
35
30
25
20
15
10
5
0

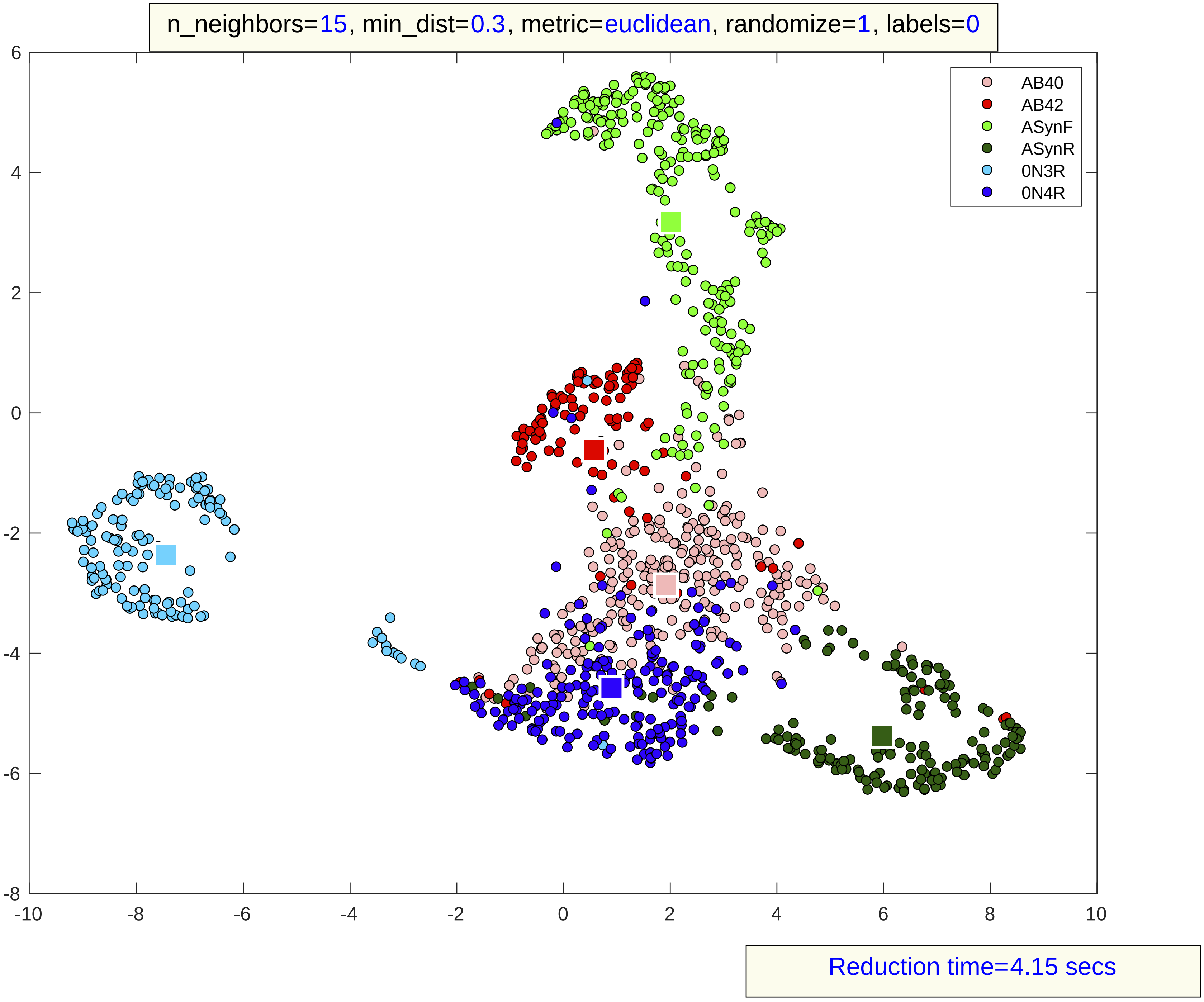
n_neighbors=15, min_dist=0.3, metric=euclidean, randomize=1, labels=0
AB40
AB42
ASynF
ASynR
0N3R
0N4R
6
4
2
0
-2
-4
-6
-8
-10
-8
-6
-4
-2
0
2
4
6
8
10
Reduction time=4.15 secs

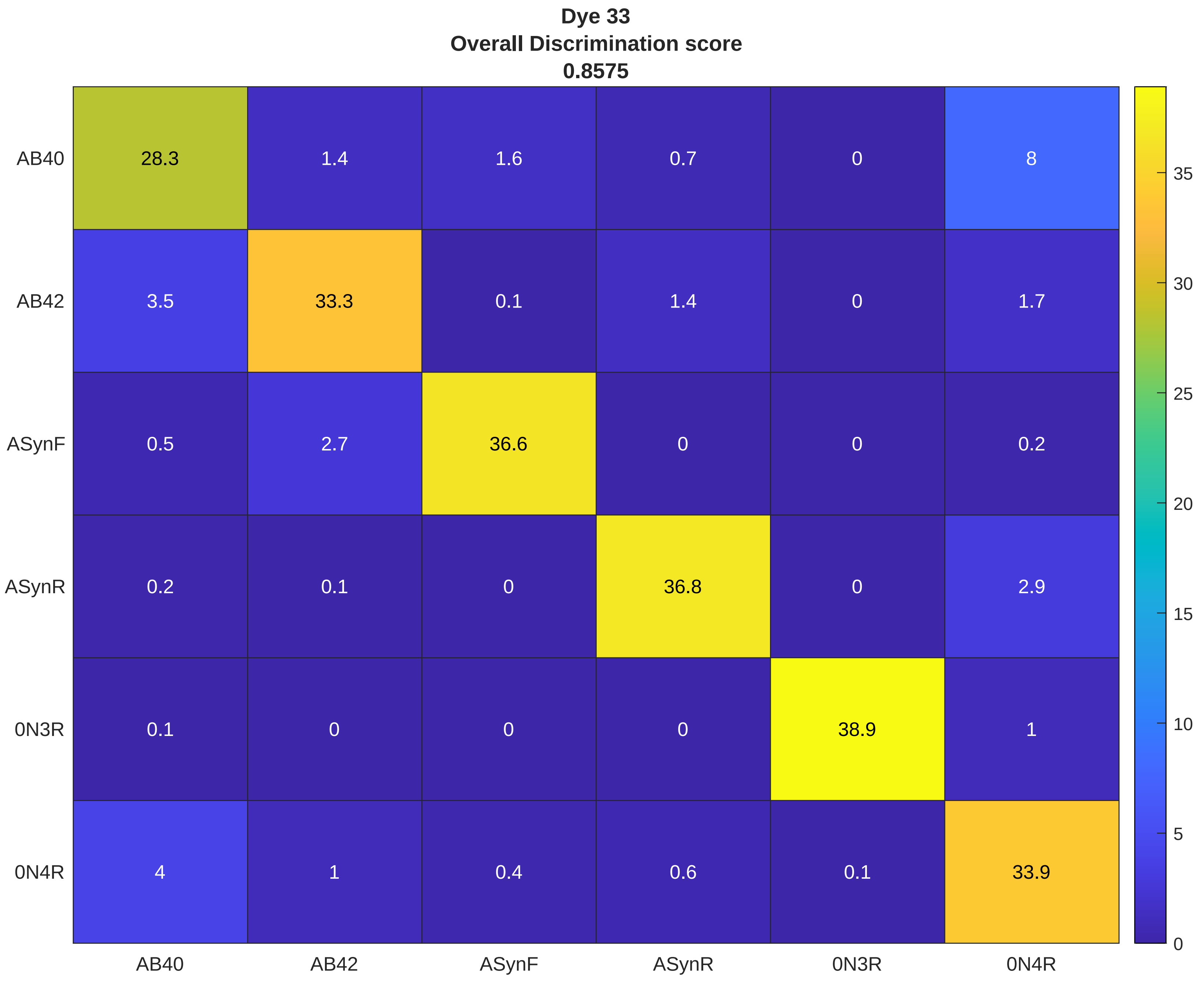

Dye 33
Overall Discrimination score
0.8575
AB40
AB42
ASynF
ASynR
0N3R
0N4R
28.3
1.4
1.6
0.7
0
8
3.5
33.3
0.1
1.4
0
1.7
0.5
2.7
36.6
0
0
0.2
0.2
0.1
0
36.8
0
2.9
0.1
0
0
0
38.9
1
4
1
0.4
0.6
0.1
33.9
AB40
AB42
ASynF
ASynR
0N3R
0N4R
35
30
25
20
15
10
5
0

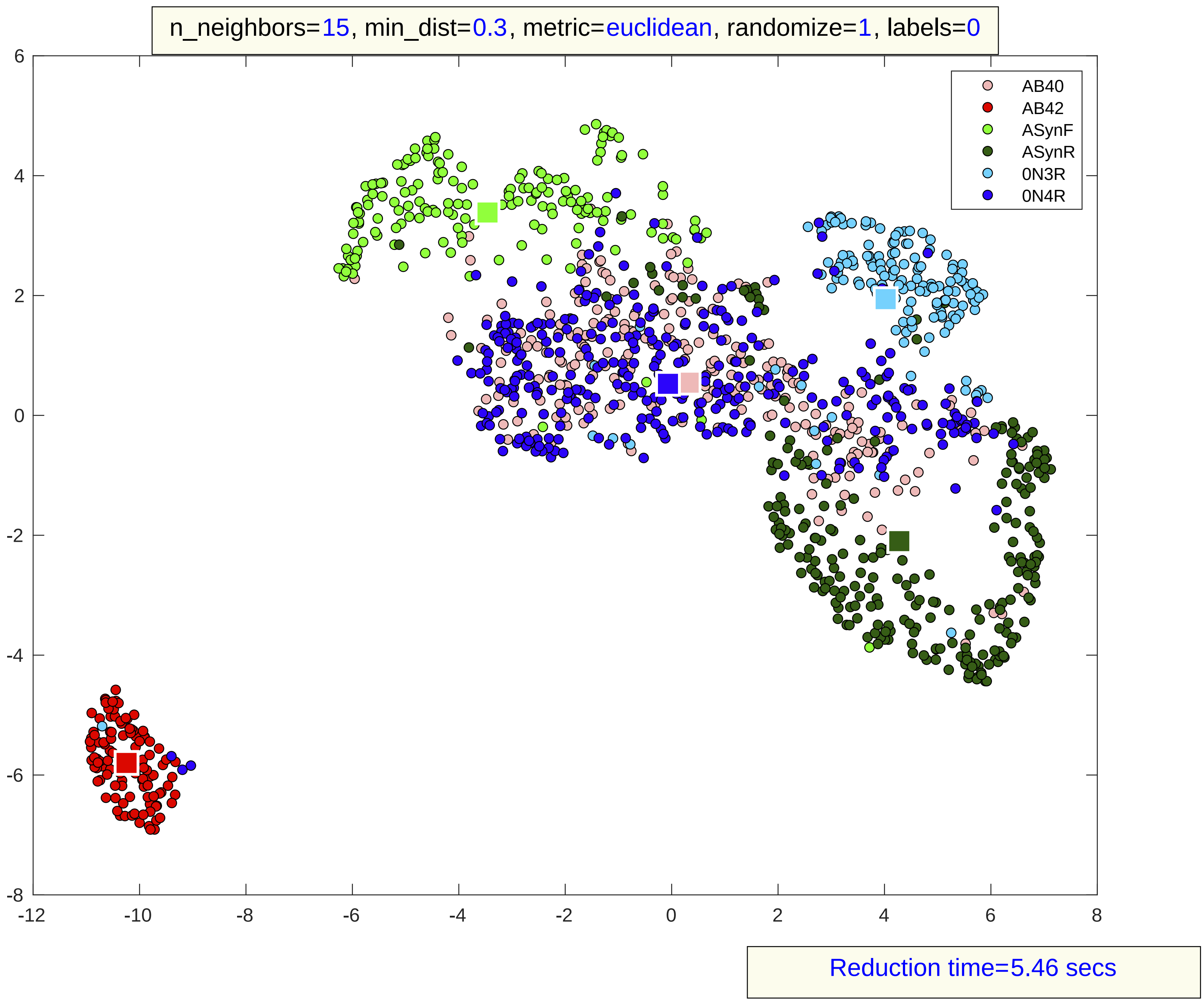

n_neighbors=15, min_dist=0.3, metric=euclidean, randomize=1, labels=0
AB40
AB42
ASynF
ASynR
0N3R
0N4R
6
4
2
0
-2
-4
-6
-8
-12
-10
-8
-6
-4
-2
0
2
4
6
8
Reduction time=5.46 secs

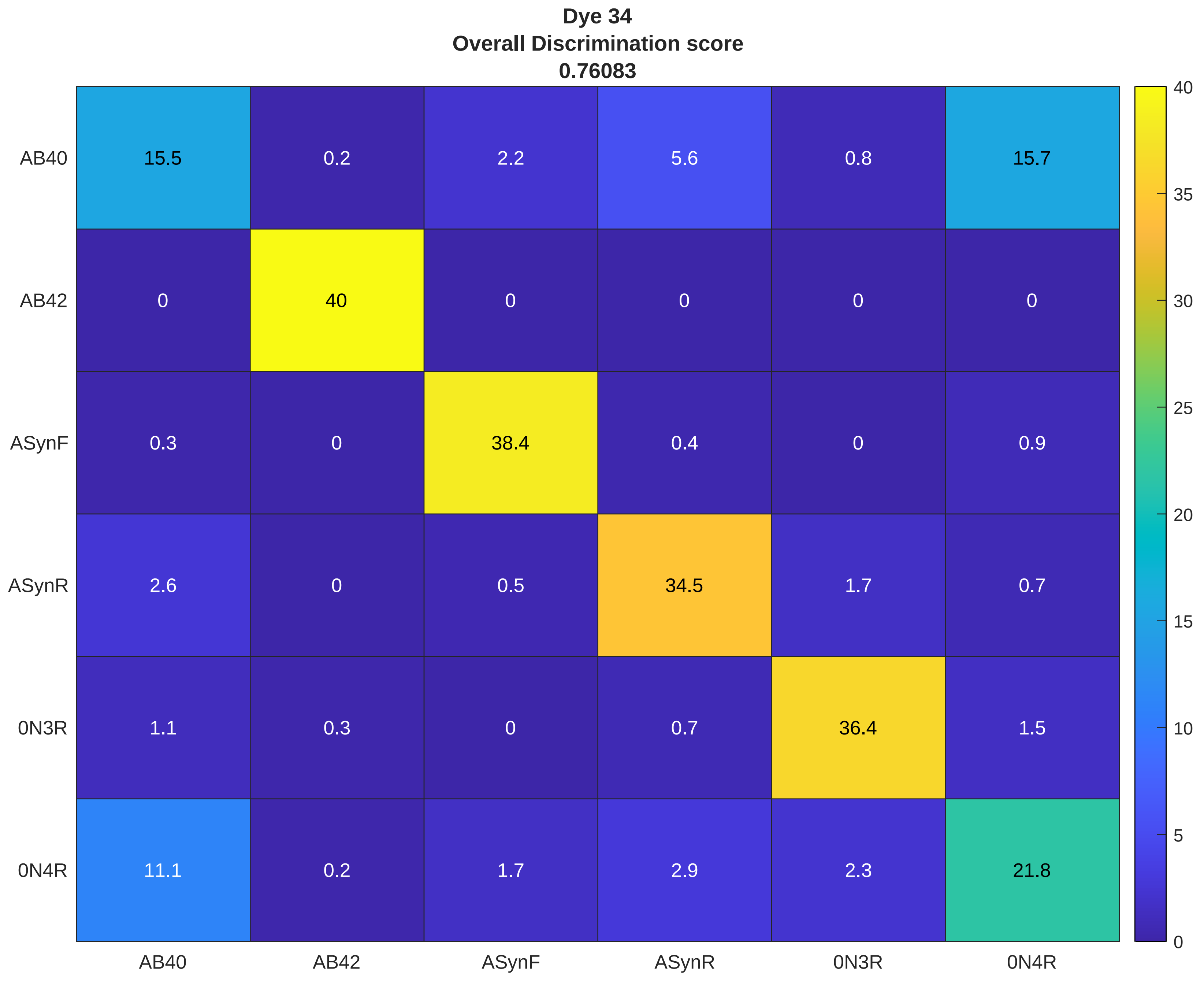
Dye 34
Overall Discrimination score
0.76083
AB40
AB42
ASynF
ASynR
0N3R
0N4R
15.5
0.2
2.2
5.6
0.8
15.7
0
40
0
0
0
0
0.3
0
38.4
0.4
0
0.9
2.6
0
0.5
34.5
1.7
0.7
1.1
0.3
0
0.7
36.4
1.5
11.1
0.2
1.7
2.9
2.3
21.8
AB40
AB42
ASynF
ASynR
0N3R
0N4R
40
35
30
25
20
15
10
5
0

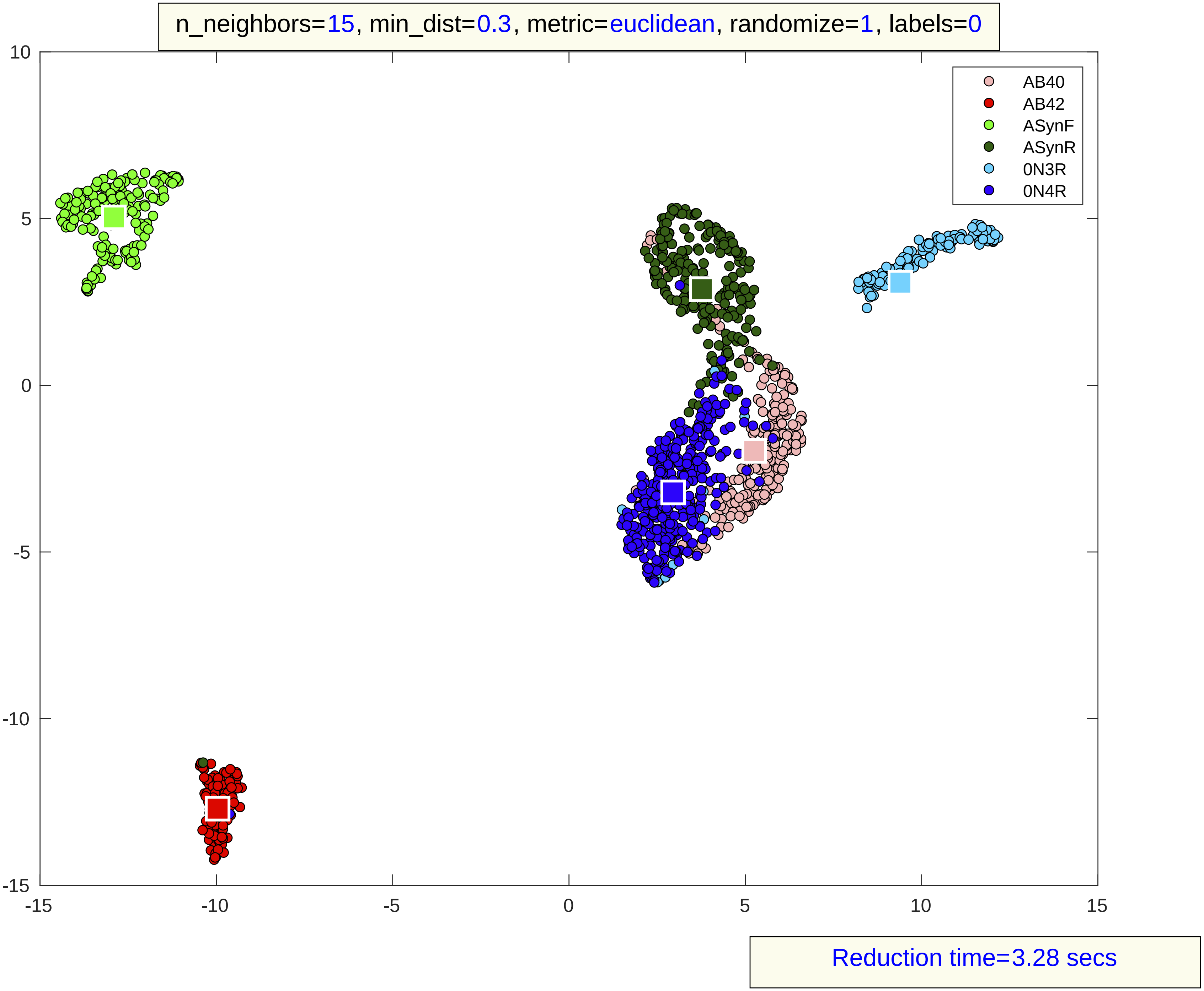
n_neighbors=15, min_dist=0.3, metric=euclidean, randomize=1, labels=0
AB40
AB42
ASynF
ASynR
0N3R
0N4R
10
5
0
-5
-10
-15
-15
-10
-5
0
5
10
15
Reduction time=3.28 secs

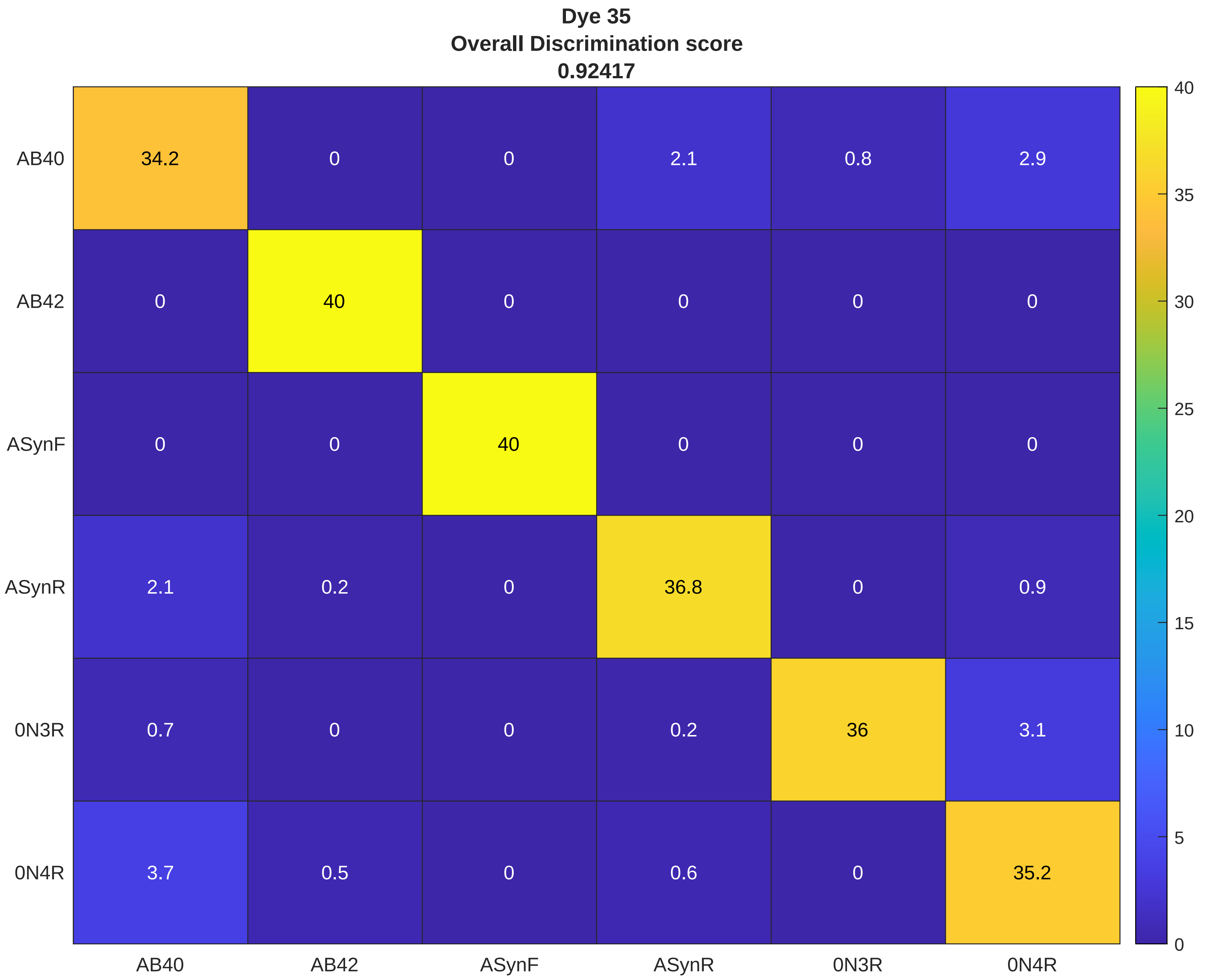
Dye 35
Overall Discrimination score
0.92417
AB40
AB42
ASynF
ASynR
0N3R
0N4R
34.2
0
0
2.1
0.8
2.9
0
40
0
0
0
0
0
0
40
0
0
0
2.1
0.2
0
36.8
0
0.9
0.7
0
0
0.2
36
3.1
3.7
0.5
0
0.6
0
35.2
AB40
AB42
ASynF
ASynR
0N3R
0N4R
40
35
30
25
20
15
10
5
0

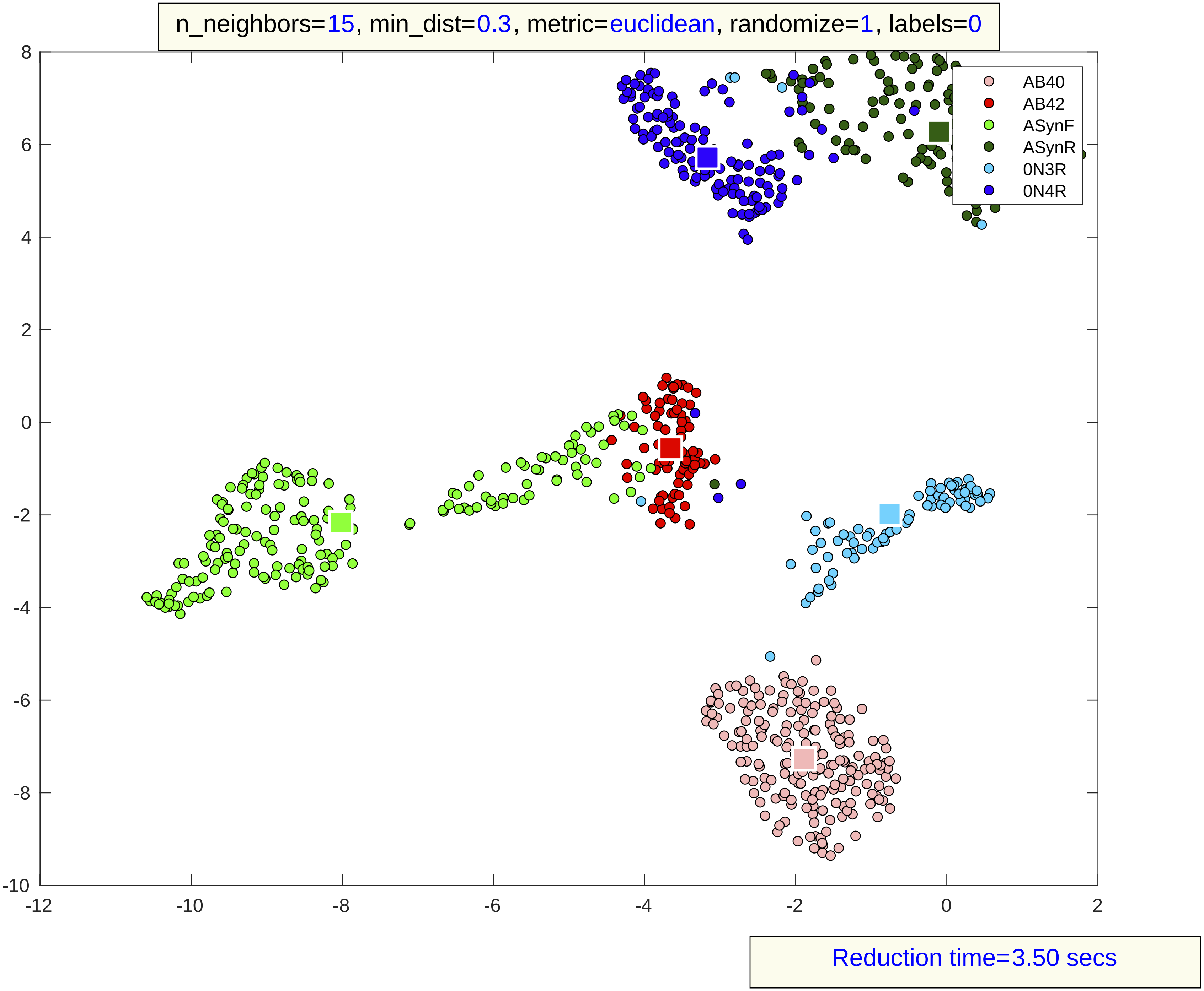
n_neighbors=15, min_dist=0.3, metric=euclidean, randomize=1, labels=0
AB40
AB42
ASynF
ASynR
0N3R
0N4R
8
6
4
2
0
-2
-4
-6
-8
-10
-12
-10
-8
-6
-4
-2
0
2
Reduction time=3.50 secs

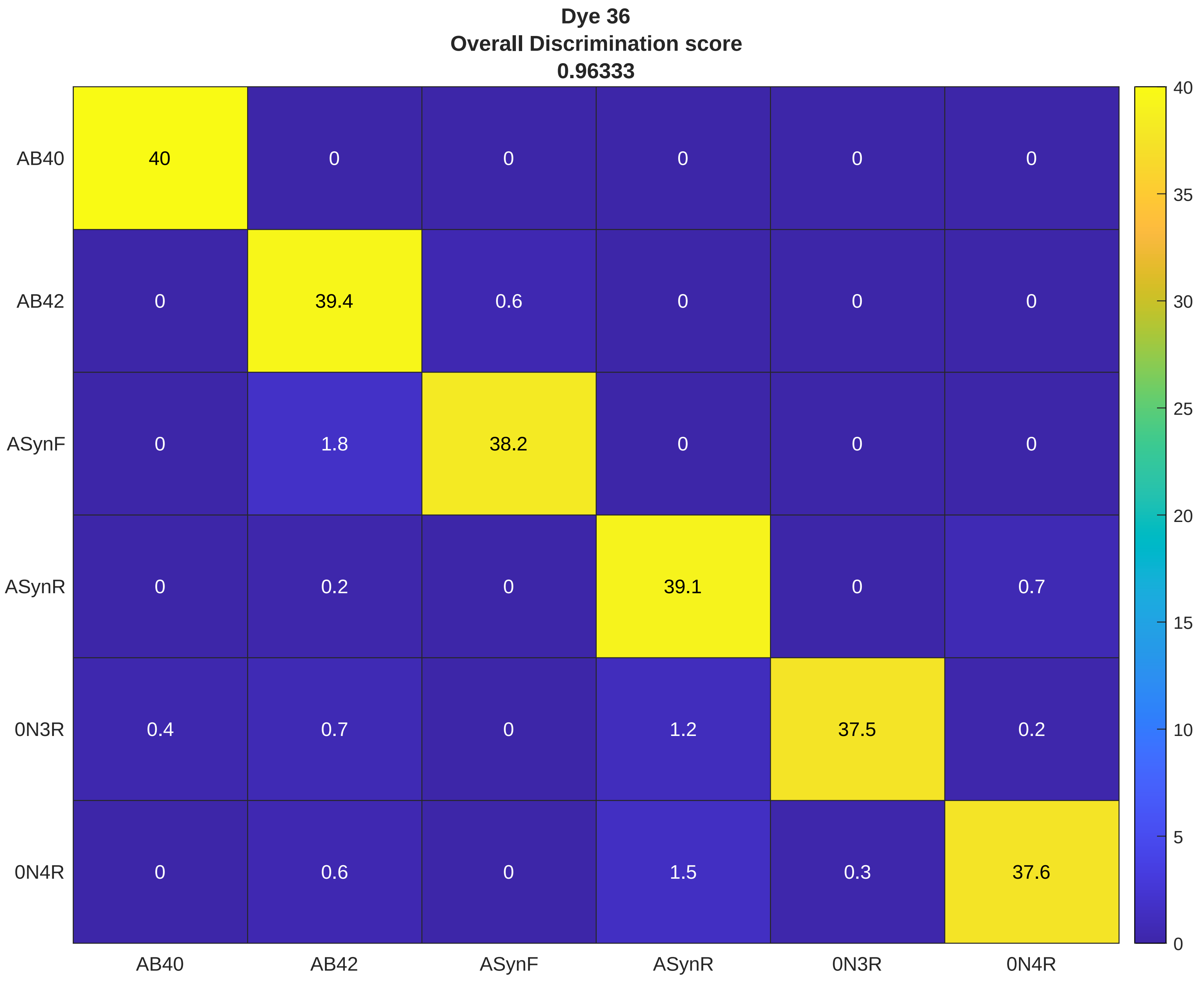
Dye 36
Overall Discrimination score
0.96333
AB40
AB42
ASynF
ASynR
0N3R
0N4R
40
0
0
0
0
0
0
39.4
0.6
0
0
0
0
1.8
38.2
0
0
0
0
0.2
0
39.1
0
0.7
0.4
0.7
0
1.2
37.5
0.2
0
0.6
0
1.5
0.3
37.6
AB40
AB42
ASynF
ASynR
0N3R
0N4R
40
35
30
25
20
15
10
5
0

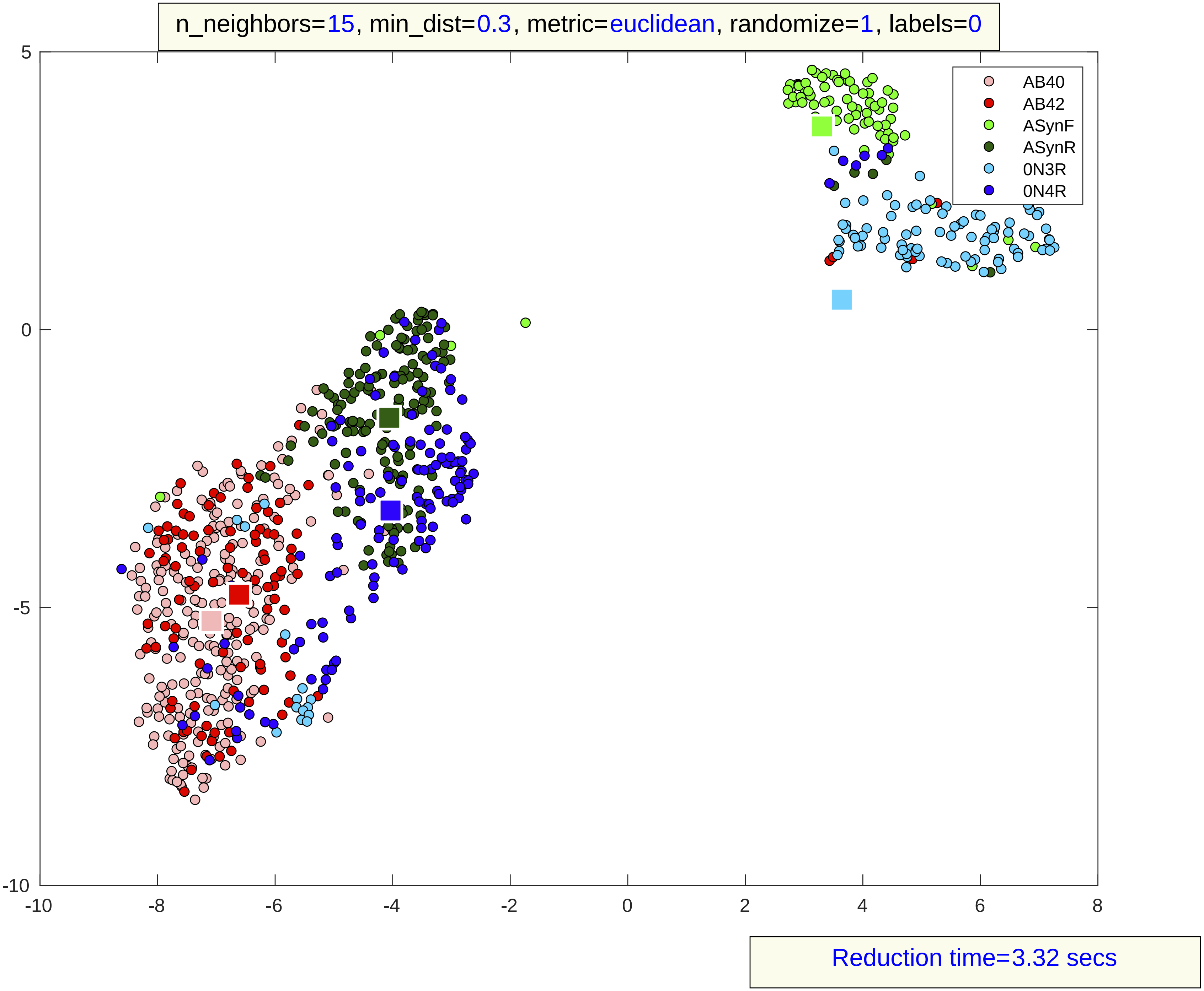

n_neighbors=15, min_dist=0.3, metric=euclidean, randomize=1, labels=0
AB40
AB42
ASynF
ASynR
0N3R
0N4R
5
0
-5
-10
-10
-8
-6
-4
-2
0
2
4
6
8
Reduction time=3.32 secs

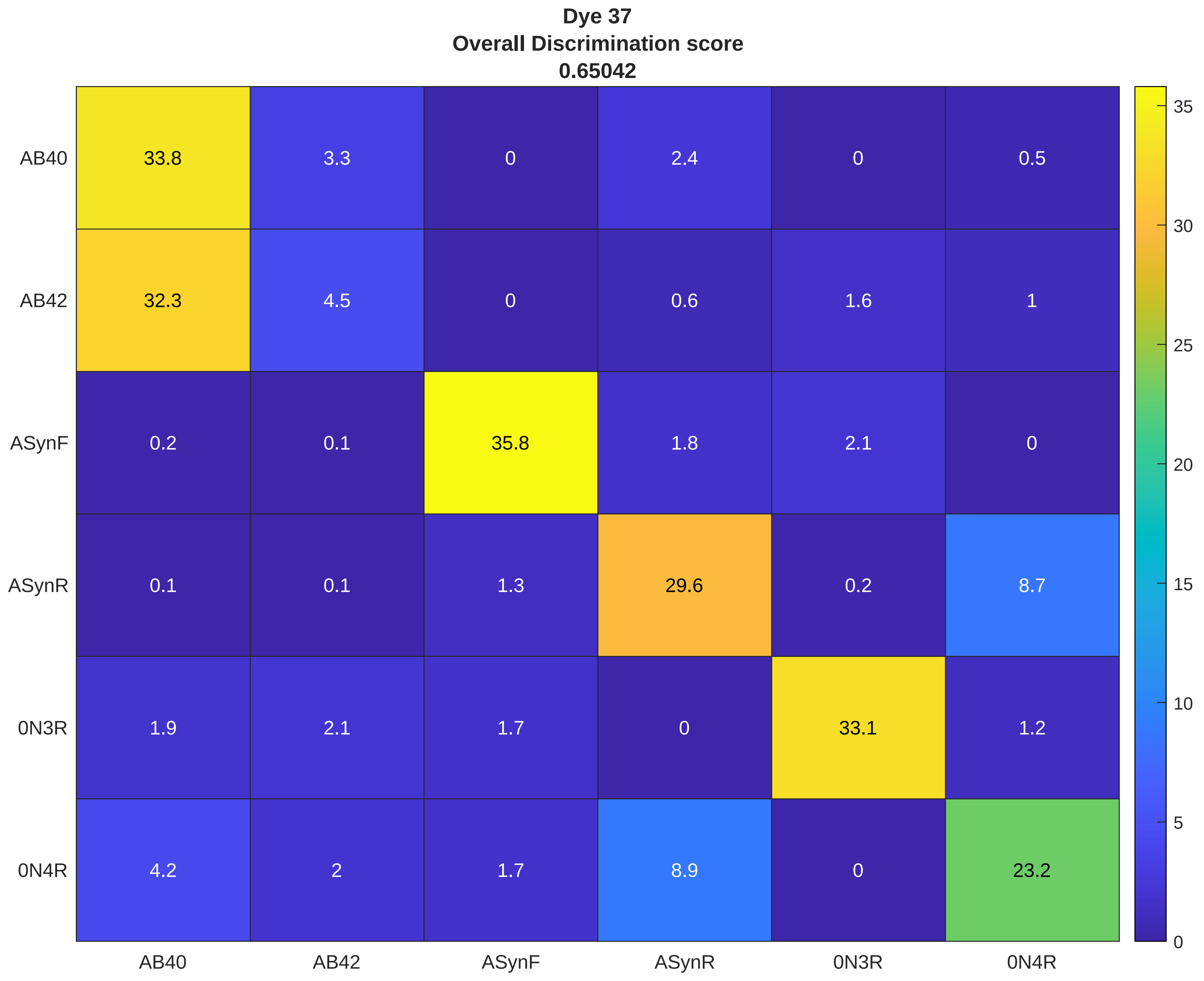

Dye 37
Overall Discrimination score
0.65042
AB40
AB42
ASynF
ASynR
0N3R
0N4R
33.8
3.3
0
2.4
0
0.5
32.3
4.5
0
0.6
1.6
1
0.2
0.1
35.8
1.8
2.1
0
0.1
0.1
1.3
29.6
0.2
8.7
1.9
2.1
1.7
0
33.1
1.2
4.2
2
1.7
8.9
0
23.2
AB40
AB42
ASynF
ASynR
0N3R
0N4R
35
30
25
20
15
10
5
0

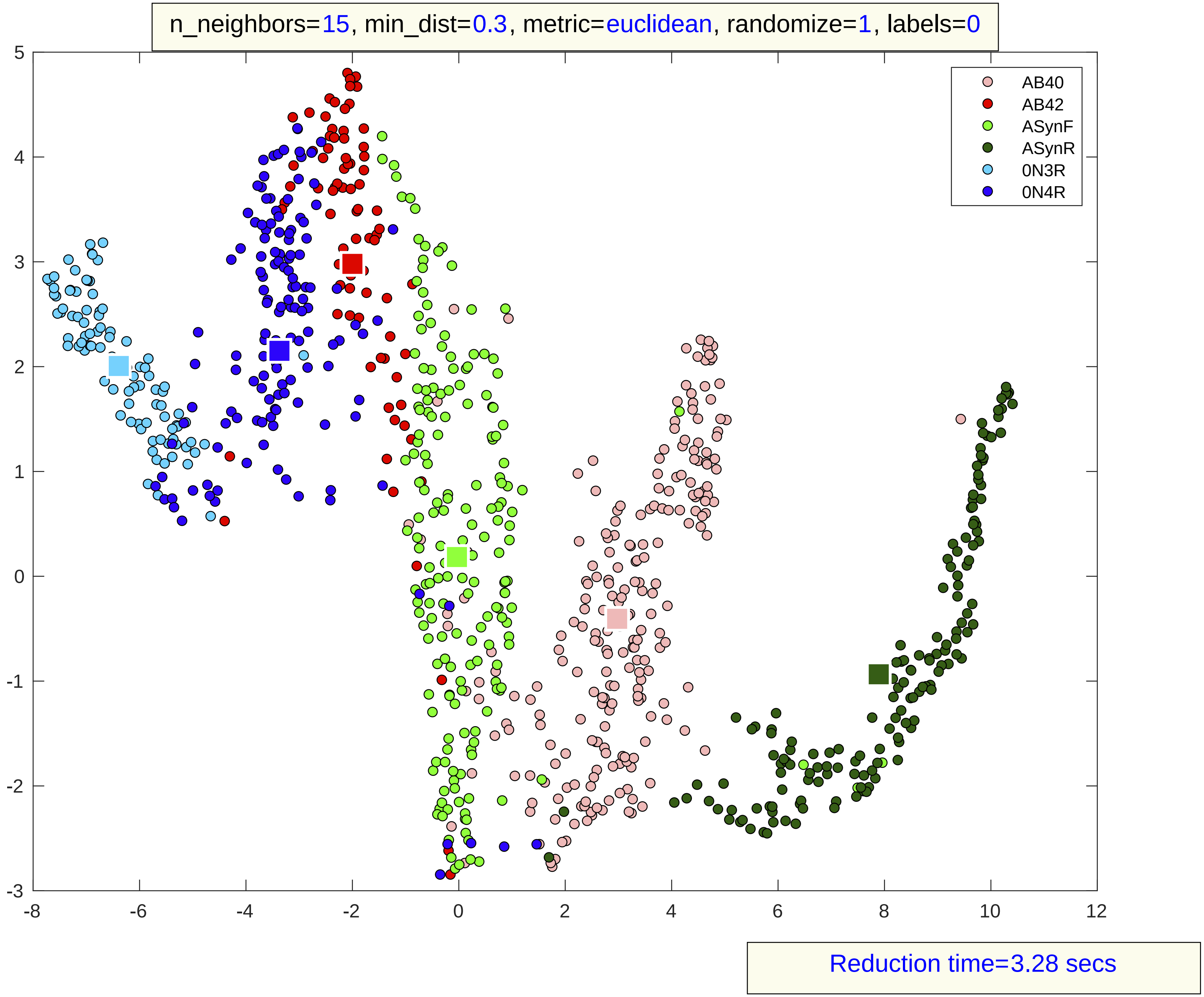

n_neighbors=15, min_dist=0.3, metric=euclidean, randomize=1, labels=0
AB40
AB42
ASynF
ASynR
0N3R
0N4R
5
4
3
2
1
0
-1
-2
-3
-8
-6
-4
-2
0
2
4
6
8
10
12
Reduction time=3.28 secs

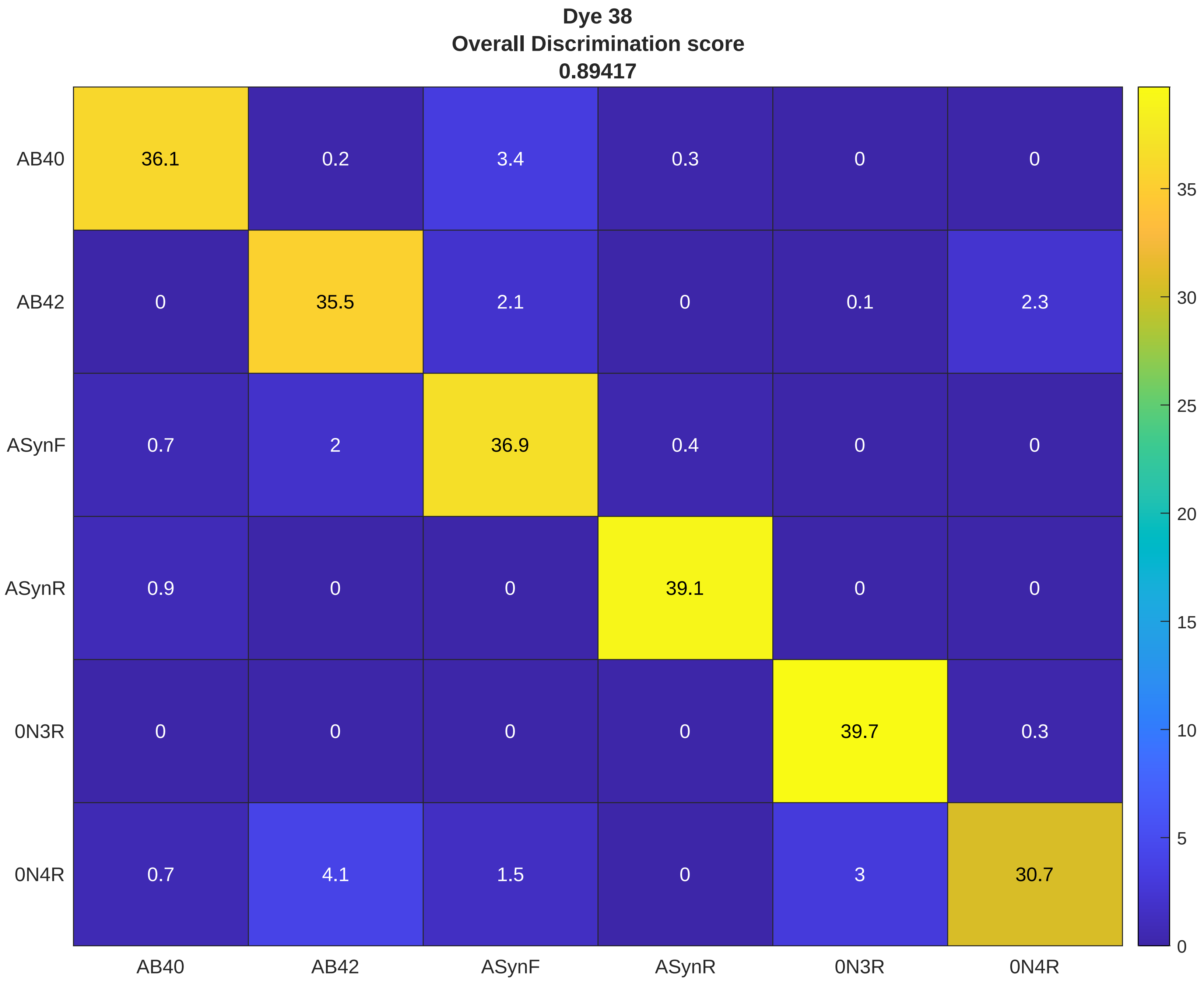
Dye 38
Overall Discrimination score
0.89417
AB40
AB42
ASynF
ASynR
0N3R
0N4R
36.1
0.2
3.4
0.3
0
0
0
35.5
2.1
0
0.1
2.3
0.7
2
36.9
0.4
0
0
0.9
0
0
39.1
0
0
0
0
0
0
39.7
0.3
0.7
4.1
1.5
0
3
30.7
AB40
AB42
ASynF
ASynR
0N3R
0N4R
35
30
25
20
15
10
5
0

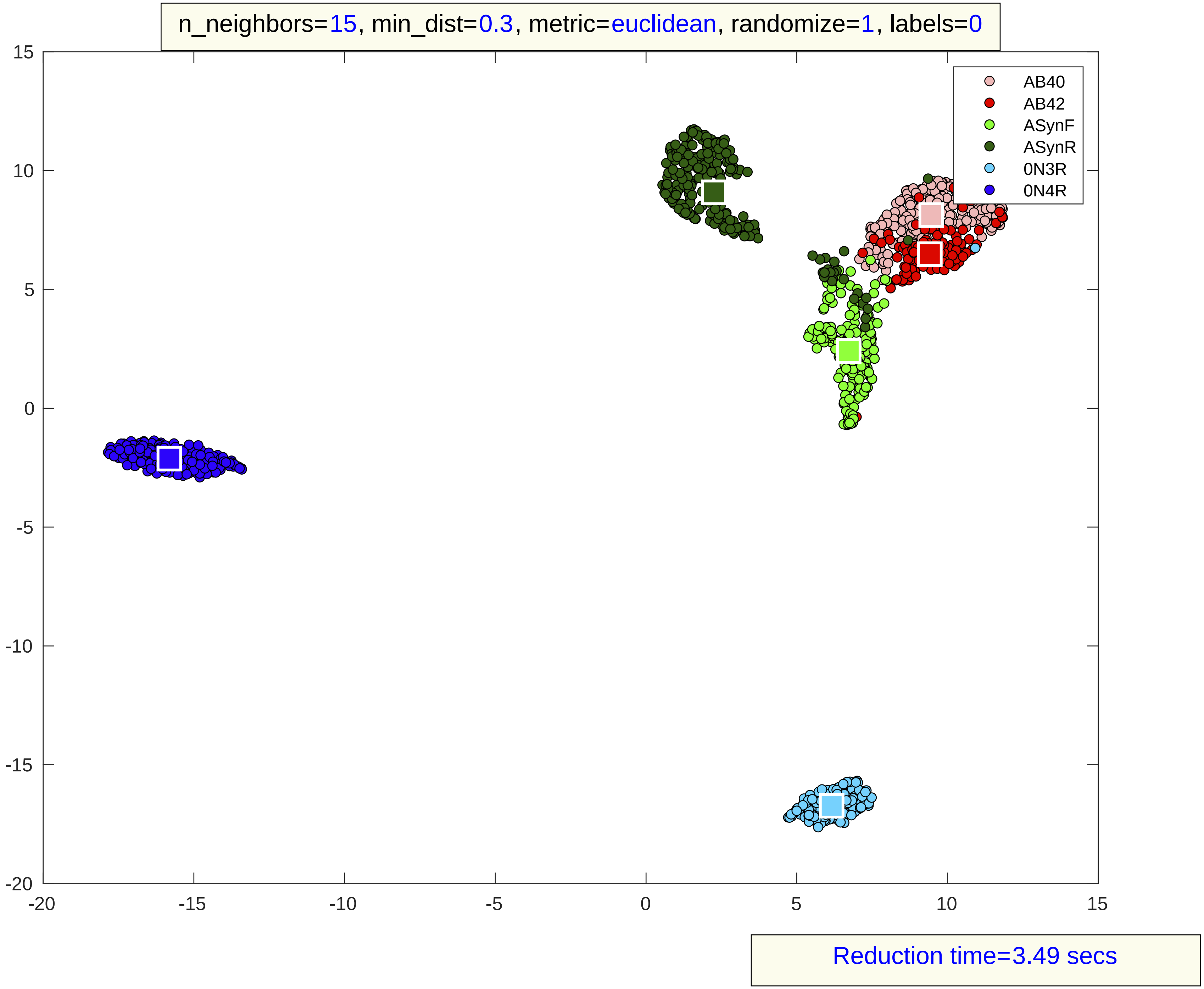

n_neighbors=15, min_dist=0.3, metric=euclidean, randomize=1, labels=0
AB40
AB42
ASynF
ASynR
0N3R
0N4R
15
10
5
0
-5
-10
-15
-20
-20
-15
-10
-5
0
5
10
15
Reduction time=3.49 secs

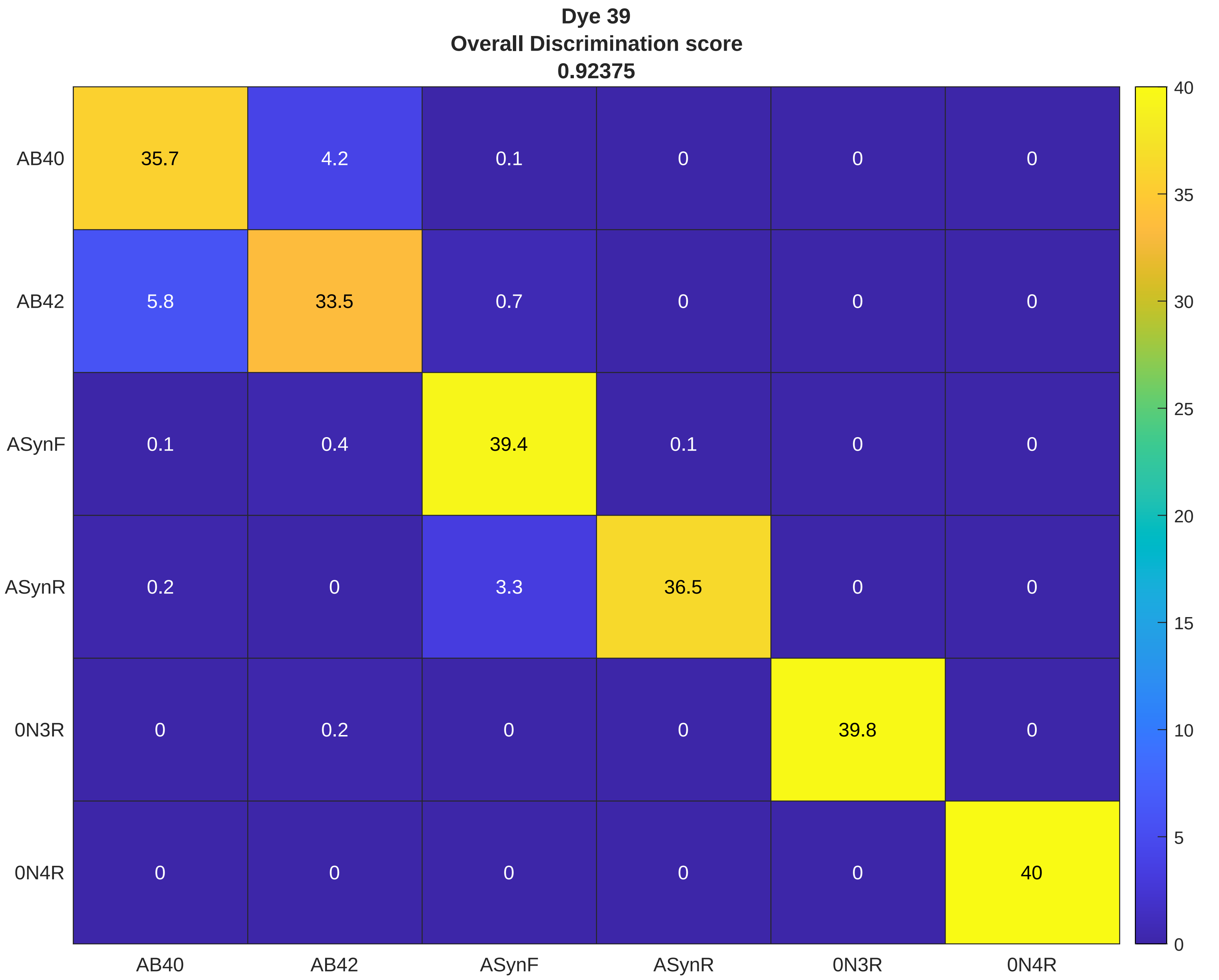
Dye 39
Overall Discrimination score
0.92375
AB40
AB42
ASynF
ASynR
0N3R
0N4R
35.7
4.2
0.1
0
0
0
5.8
33.5
0.7
0
0
0
0.1
0.4
39.4
0.1
0
0
0.2
0
3.3
36.5
0
0
0
0.2
0
0
39.8
0
0
0
0
0
0
40
AB40
AB42
ASynF
ASynR
0N3R
0N4R
40
35
30
25
20
15
10
5
0

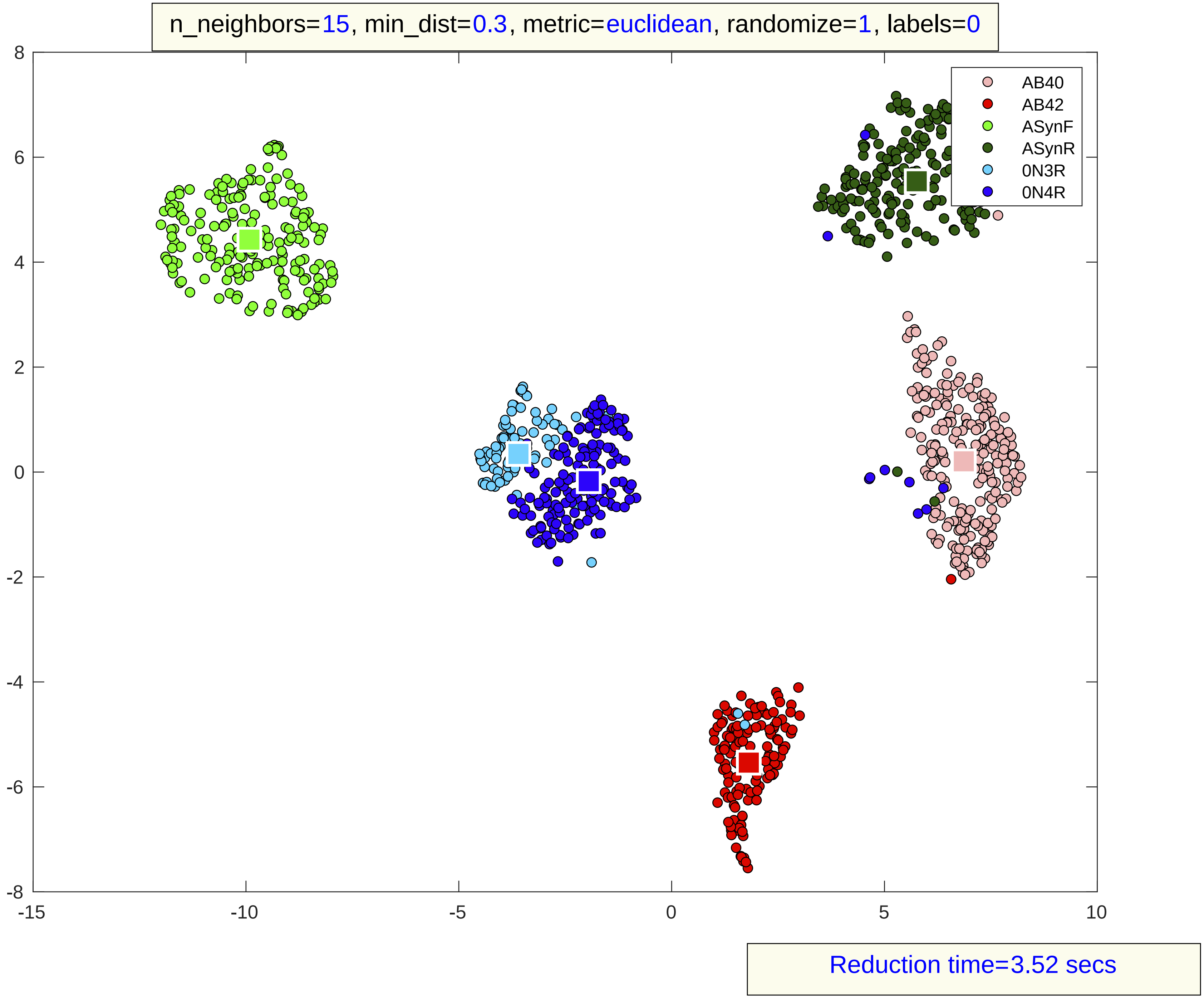
n_neighbors=15, min_dist=0.3, metric=euclidean, randomize=1, labels=0
AB40
AB42
ASynF
ASynR
0N3R
0N4R
8
6
4
2
0
-2
-4
-6
-8
-15
-10
-5
0
5
10
Reduction time=3.52 secs

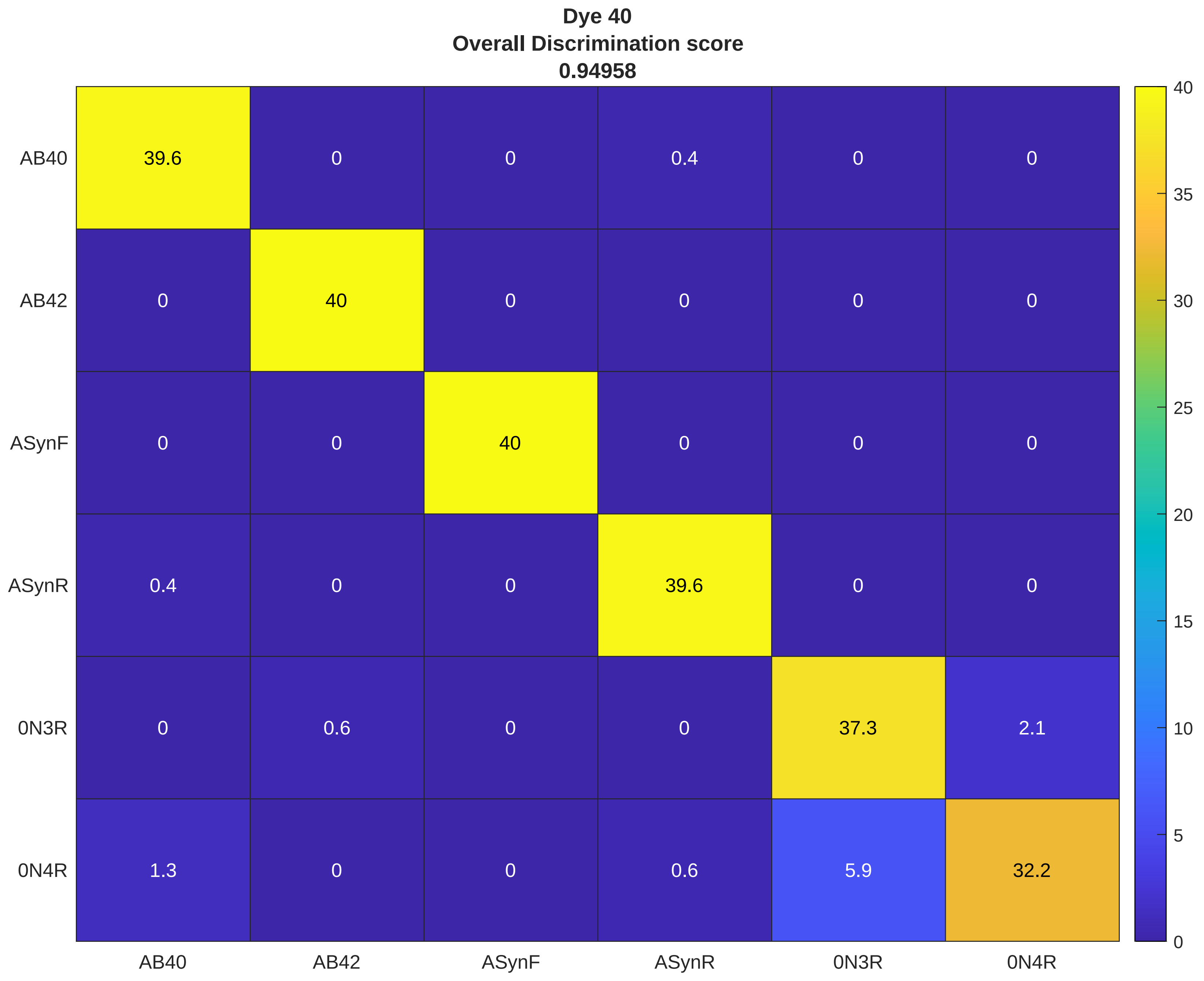

Dye 40
Overall Discrimination score
0.94958
AB40
AB42
ASynF
ASynR
0N3R
0N4R
39.6 0 0 0.4 0 0
0 40 0 0 0 0
0 0 40 0 0 0
0.4 0 0 39.6 0 0
0 0.6 0 0 37.3 2.1
1.3 0 0 0.6 5.9 32.2
AB40
AB42
ASynF
ASynR
0N3R
0N4R
40
35
30
25
20
15
10
5
0

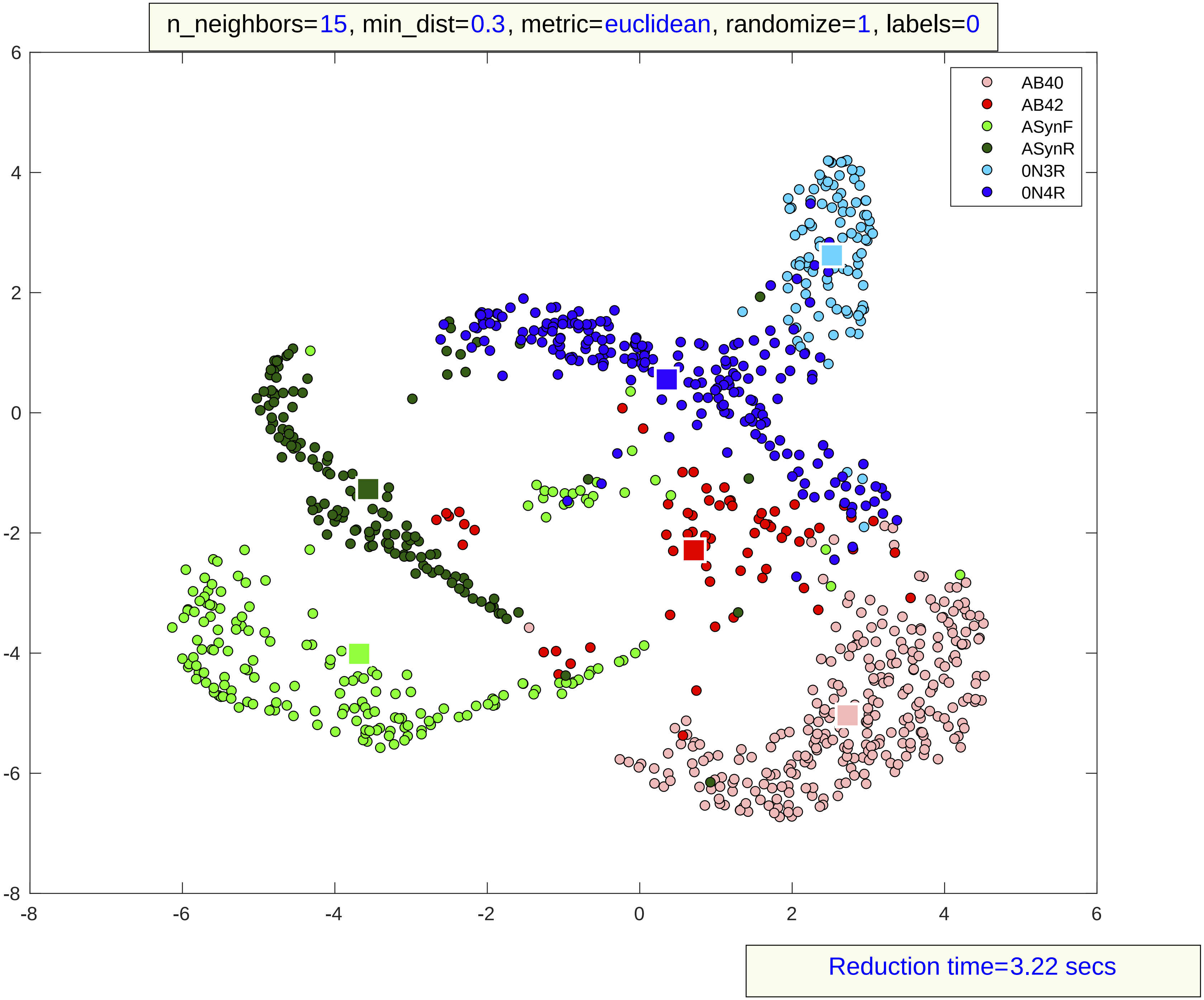

n_neighbors=15, min_dist=0.3, metric=euclidean, randomize=1, labels=0
AB40
AB42
ASynF
ASynR
0N3R
0N4R
6
4
2
0
-2
-4
-6
-8
-8
-6
-4
-2
0
2
4
6
Reduction time=3.22 secs

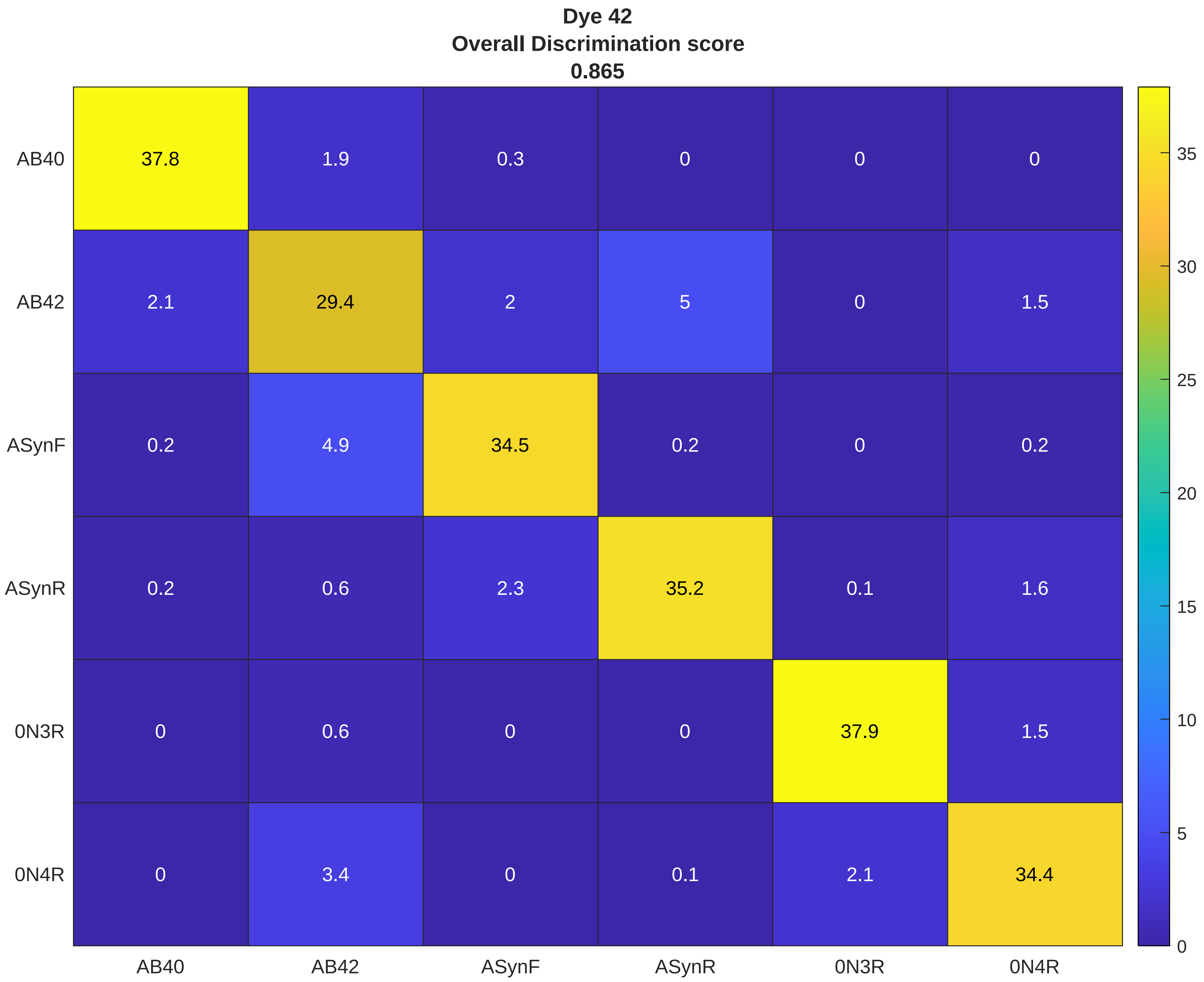
Dye 42
Overall Discrimination score
0.865
AB40
AB42
ASynF
ASynR
0N3R
0N4R
37.8 1.9 0.3 0 0 0
2.1 29.4 2 5 0 1.5
0.2 4.9 34.5 0.2 0 0.2
0.2 0.6 2.3 35.2 0.1 1.6
0 0.6 0 0 37.9 1.5
0 3.4 0 0.1 2.1 34.4
AB40
AB42
ASynF
ASynR
0N3R
0N4R
0
5
10
15
20
25
30
35

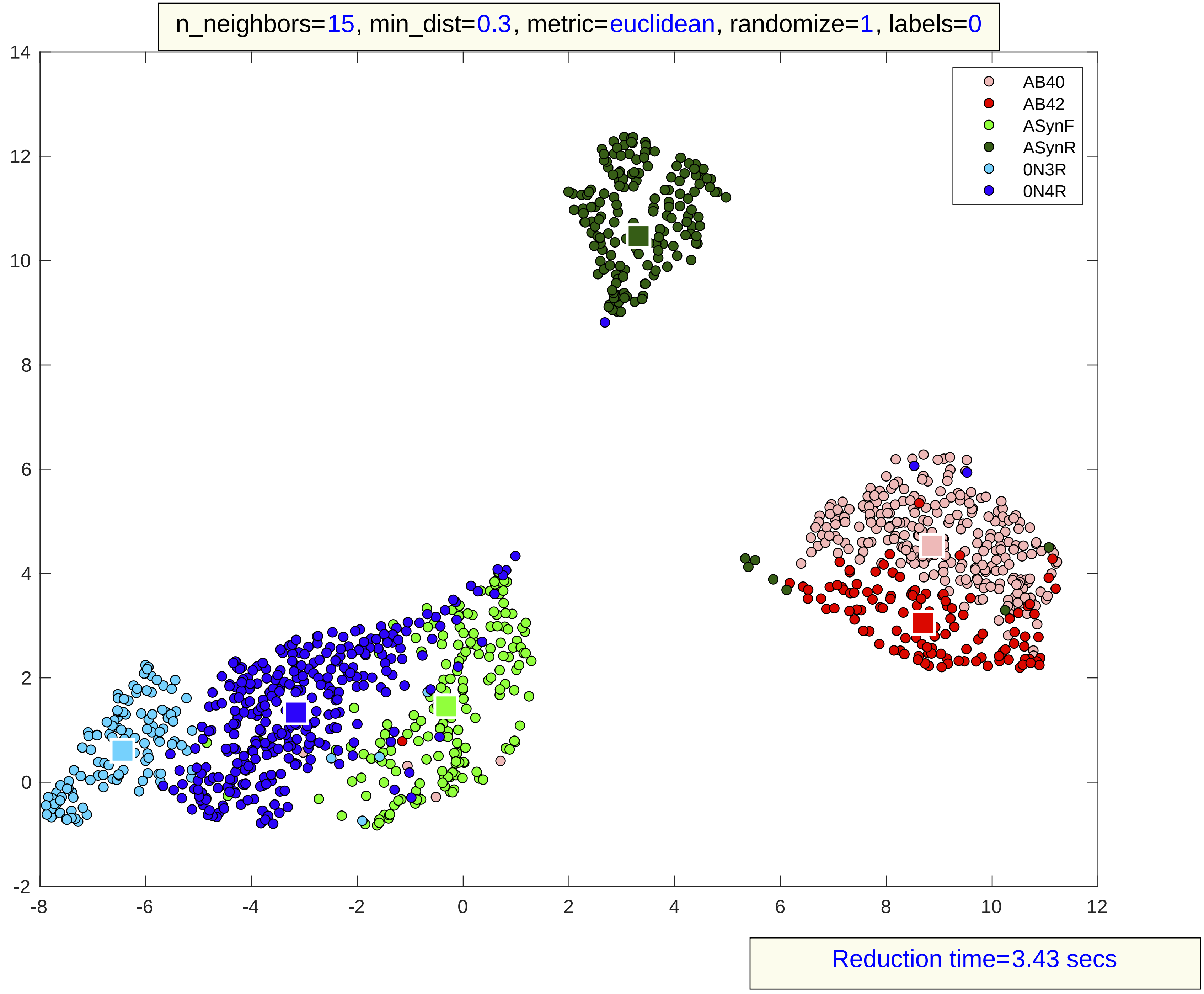
n_neighbors=15, min_dist=0.3, metric=euclidean, randomize=1, labels=0
AB40
AB42
ASynF
ASynR
0N3R
0N4R
14
12
10
8
6
4
2
0
-2
-8
-6
-4
-2
0
2
4
6
8
10
12
Reduction time=3.43 secs

Dye 43
Overall Discrimination score
0.90125

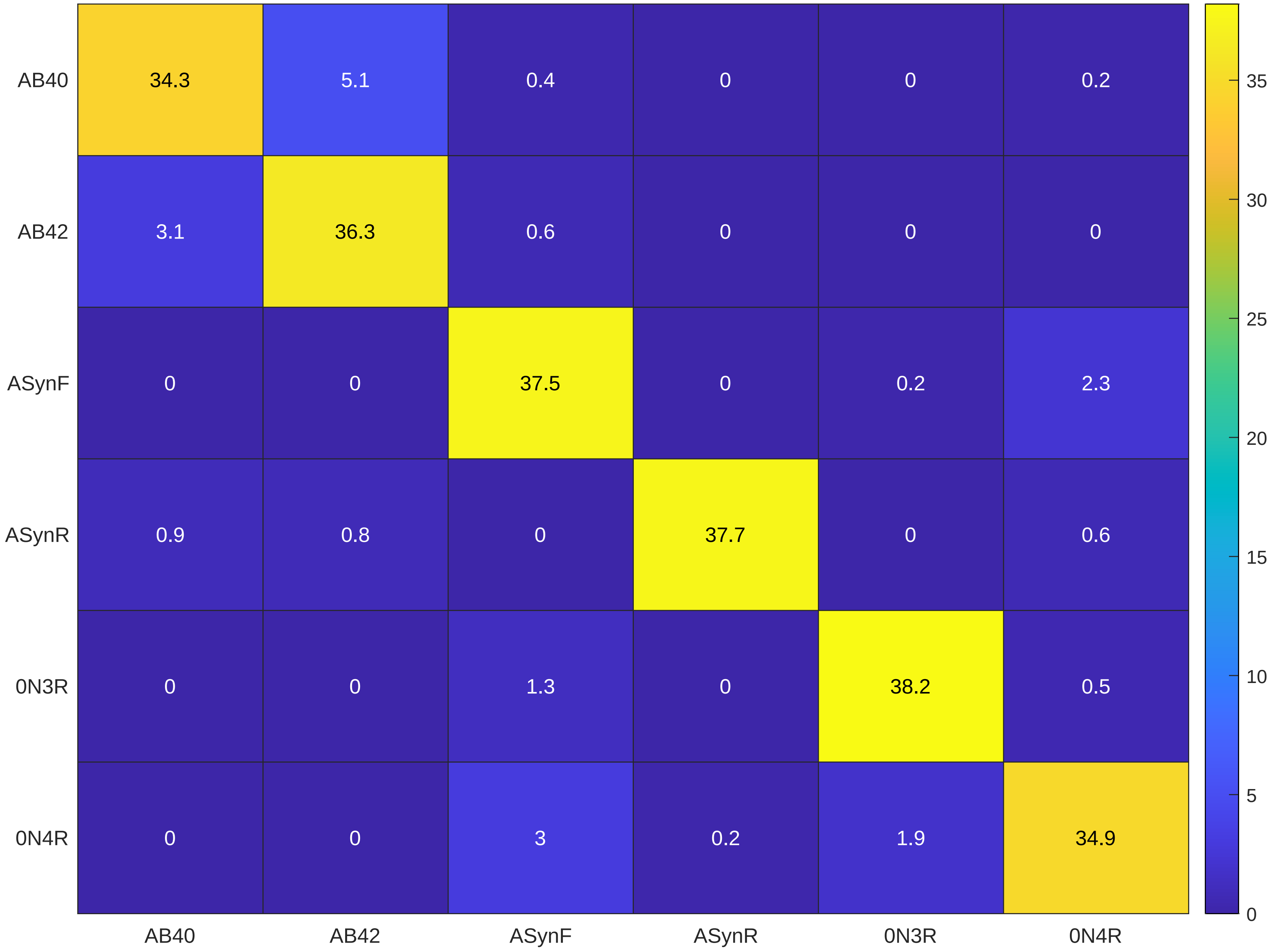

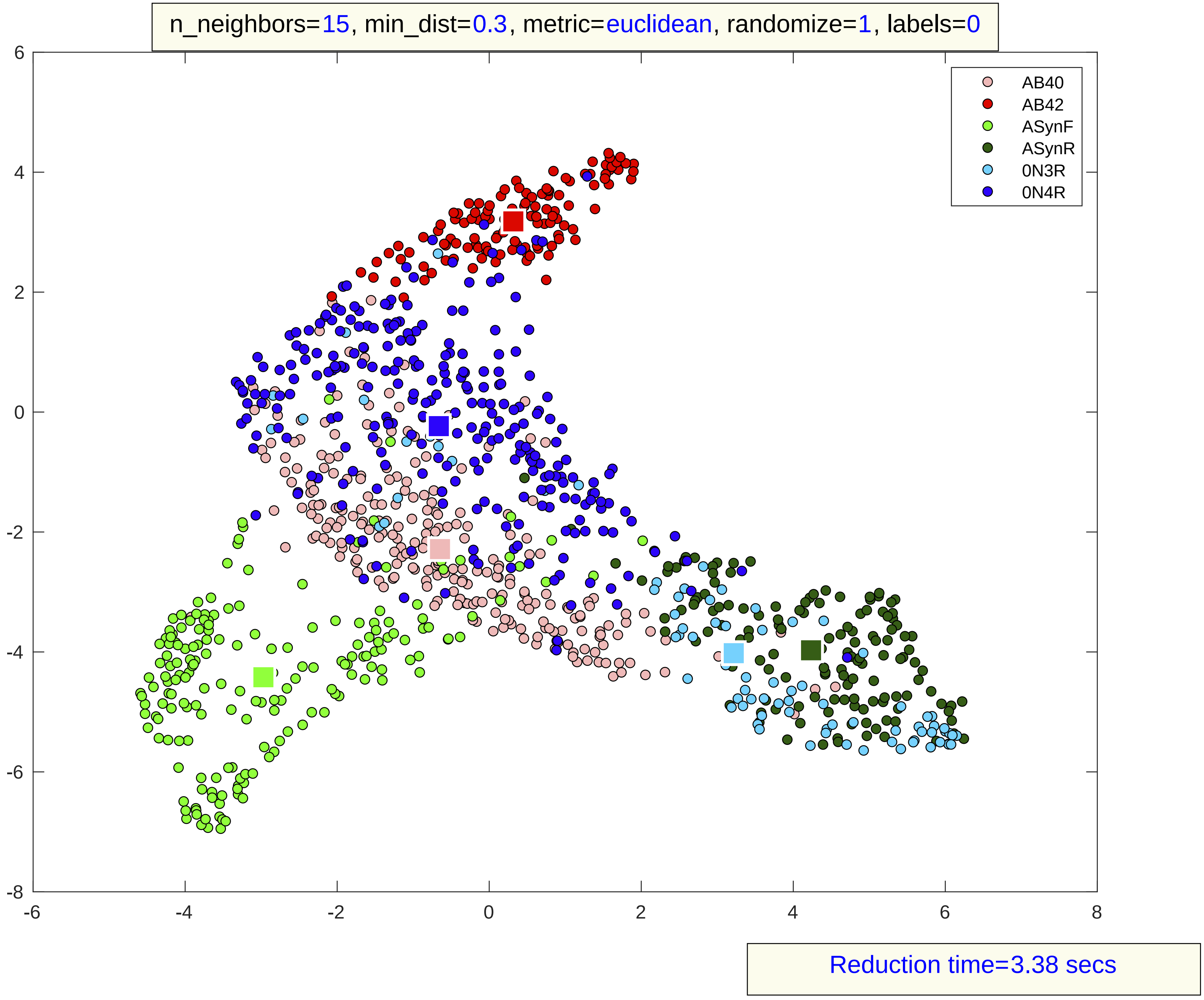
n_neighbors=15, min_dist=0.3, metric=euclidean, randomize=1, labels=0
AB40
AB42
ASynF
ASynR
0N3R
0N4R
6
4
2
0
-2
-4
-6
-8
-6
-4
-2
0
2
4
6
8
Reduction time=3.38 secs

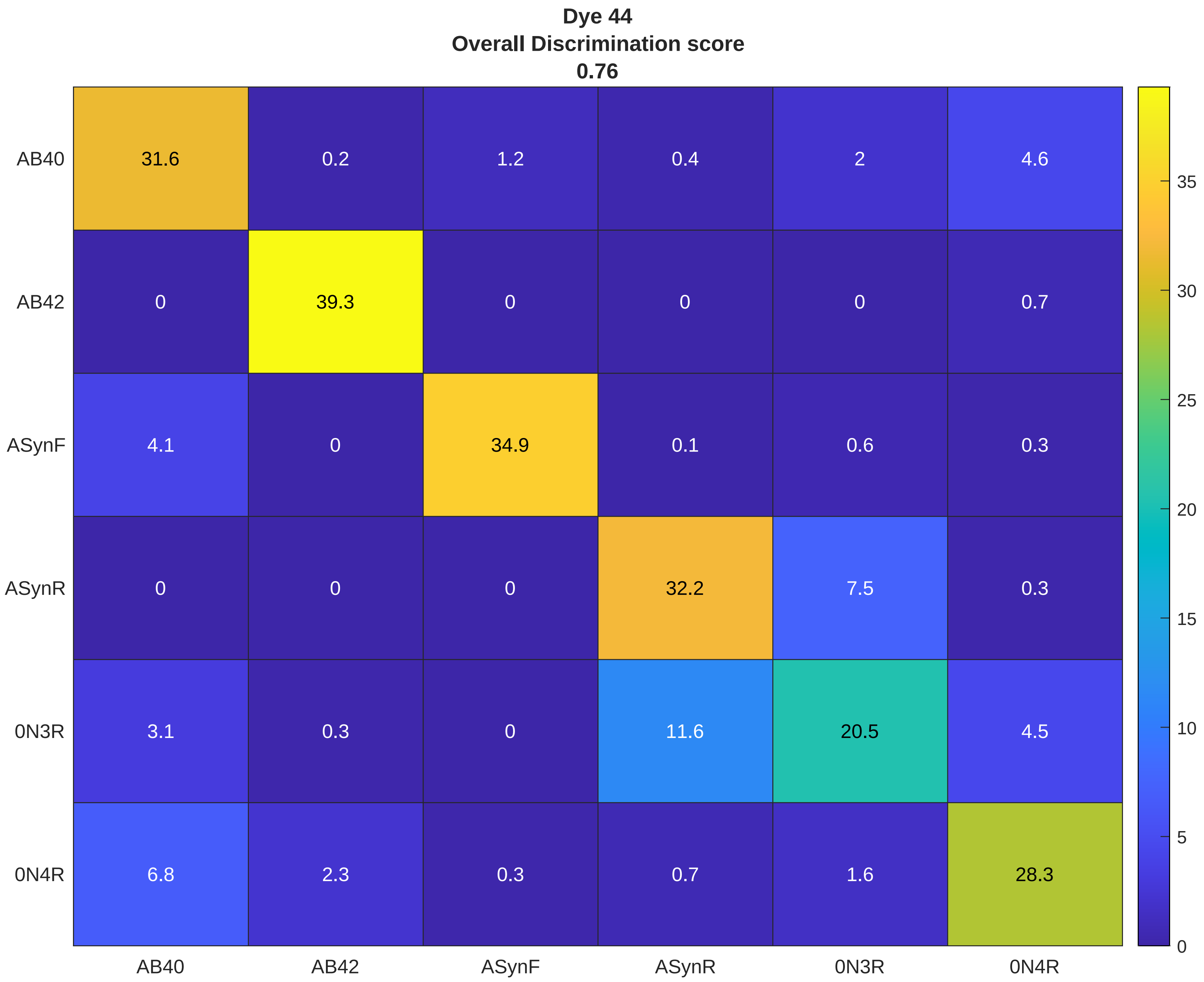
Dye 44
Overall Discrimination score
0.76
AB40
AB42
ASynF
ASynR
0N3R
0N4R
31.6
0.2
1.2
0.4
2
4.6
0
39.3
0
0
0
0.7
4.1
0
34.9
0.1
0.6
0.3
0
0
0
32.2
7.5
0.3
3.1
0.3
0
11.6
20.5
4.5
6.8
2.3
0.3
0.7
1.6
28.3
AB40
AB42
ASynF
ASynR
0N3R
0N4R
35
30
25
20
15
10
5
0

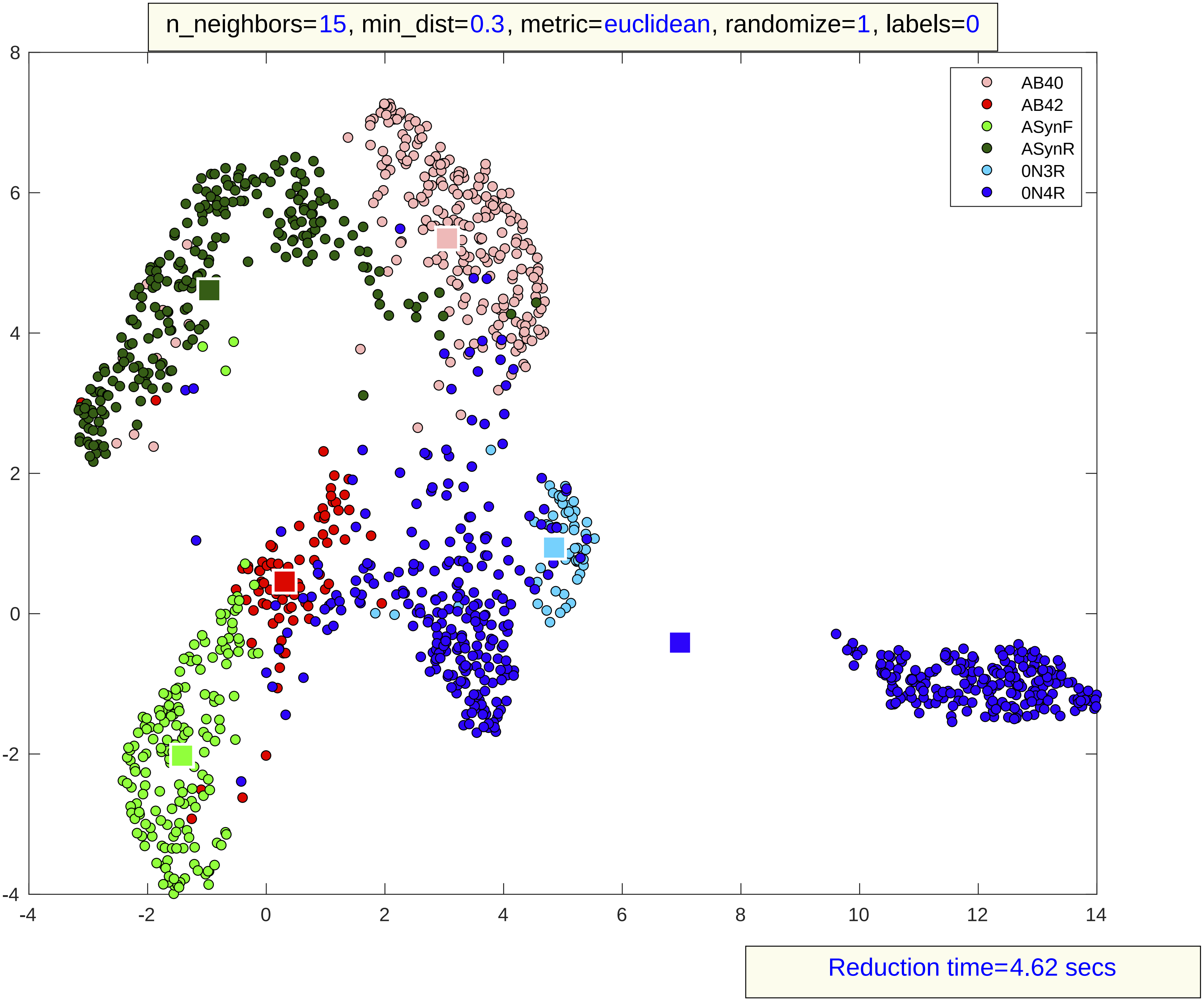
n_neighbors=15, min_dist=0.3, metric=euclidean, randomize=1, labels=0
AB40
AB42
ASynF
ASynR
0N3R
0N4R
8
6
4
2
0
-2
-4
-4
-2
0
2
4
6
8
10
12
14
Reduction time=4.62 secs

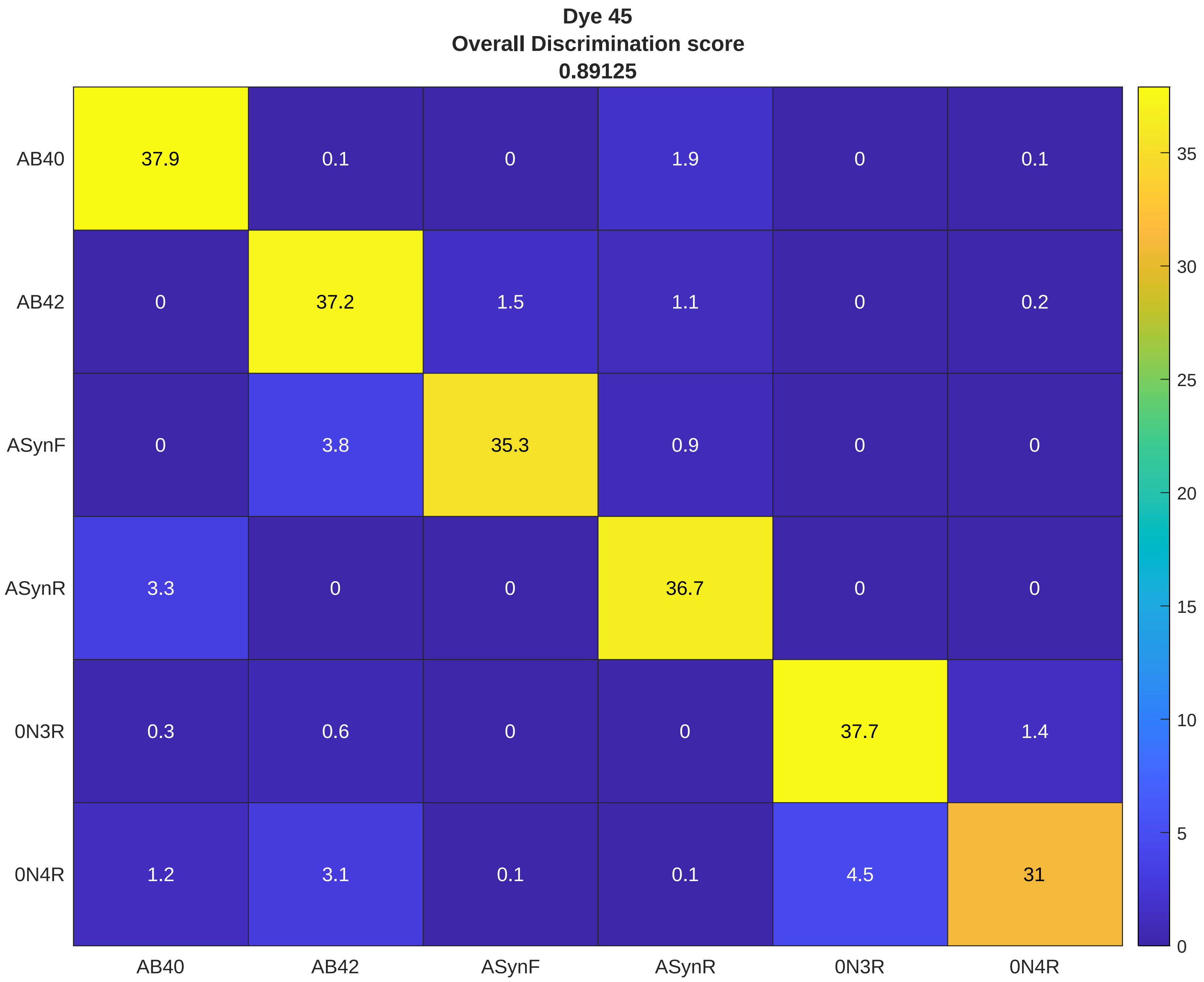
Dye 45
Overall Discrimination score
0.89125
AB40
AB42
ASynF
ASynR
0N3R
0N4R
37.9
0.1
0
1.9
0
0.1
0
37.2
1.5
1.1
0
0.2
0
3.8
35.3
0.9
0
0
3.3
0
0
36.7
0
0
0.3
0.6
0
0
37.7
1.4
1.2
3.1
0.1
0.1
4.5
31
AB40
AB42
ASynF
ASynR
0N3R
0N4R
35
30
25
20
15
10
5
0

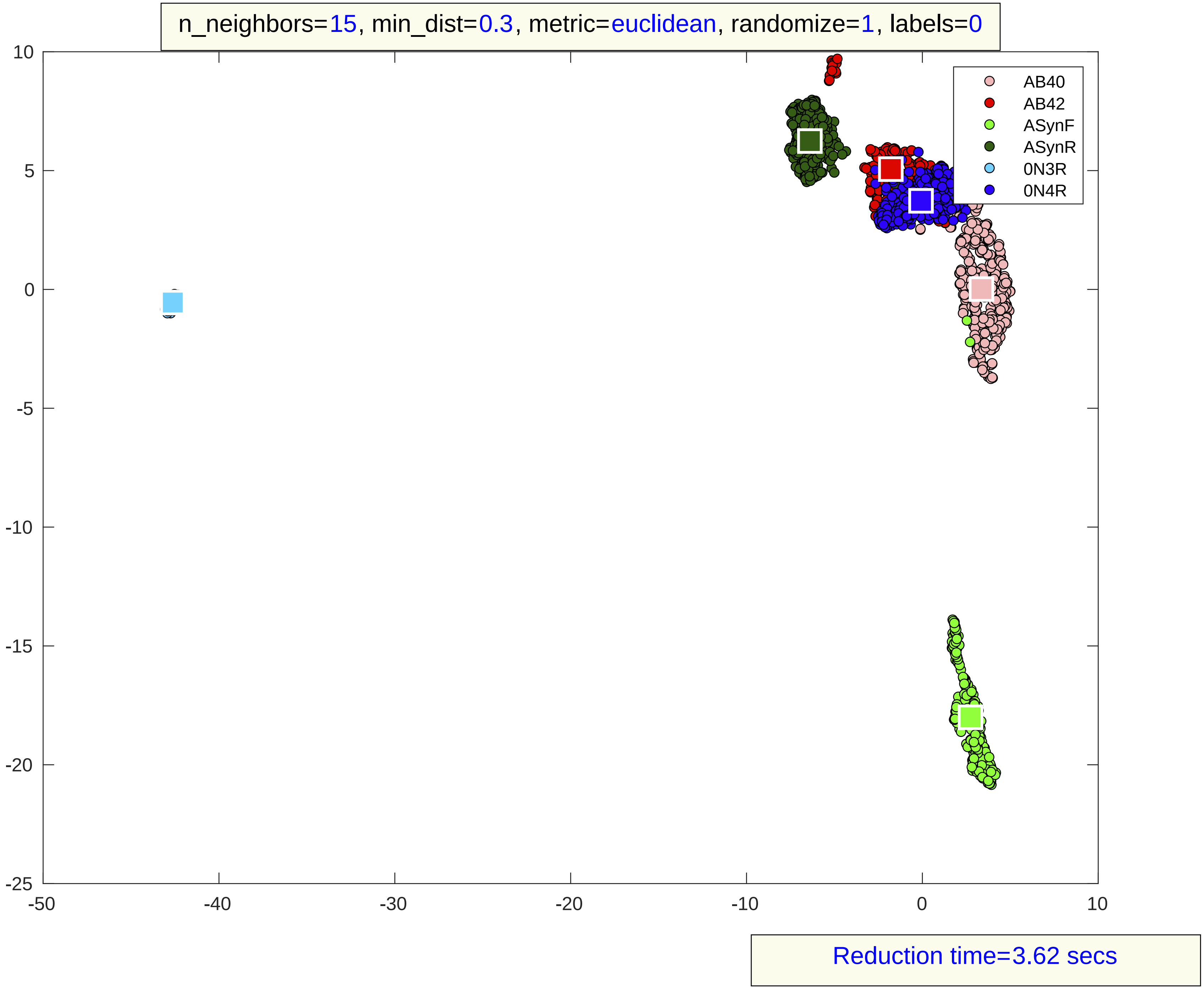

n_neighbors=15, min_dist=0.3, metric=euclidean, randomize=1, labels=0
AB40
AB42
ASynF
ASynR
0N3R
0N4R
10
5
0
-5
-10
-15
-20
-25
-50
-40
-30
-20
-10
0
10
Reduction time=3.62 secs

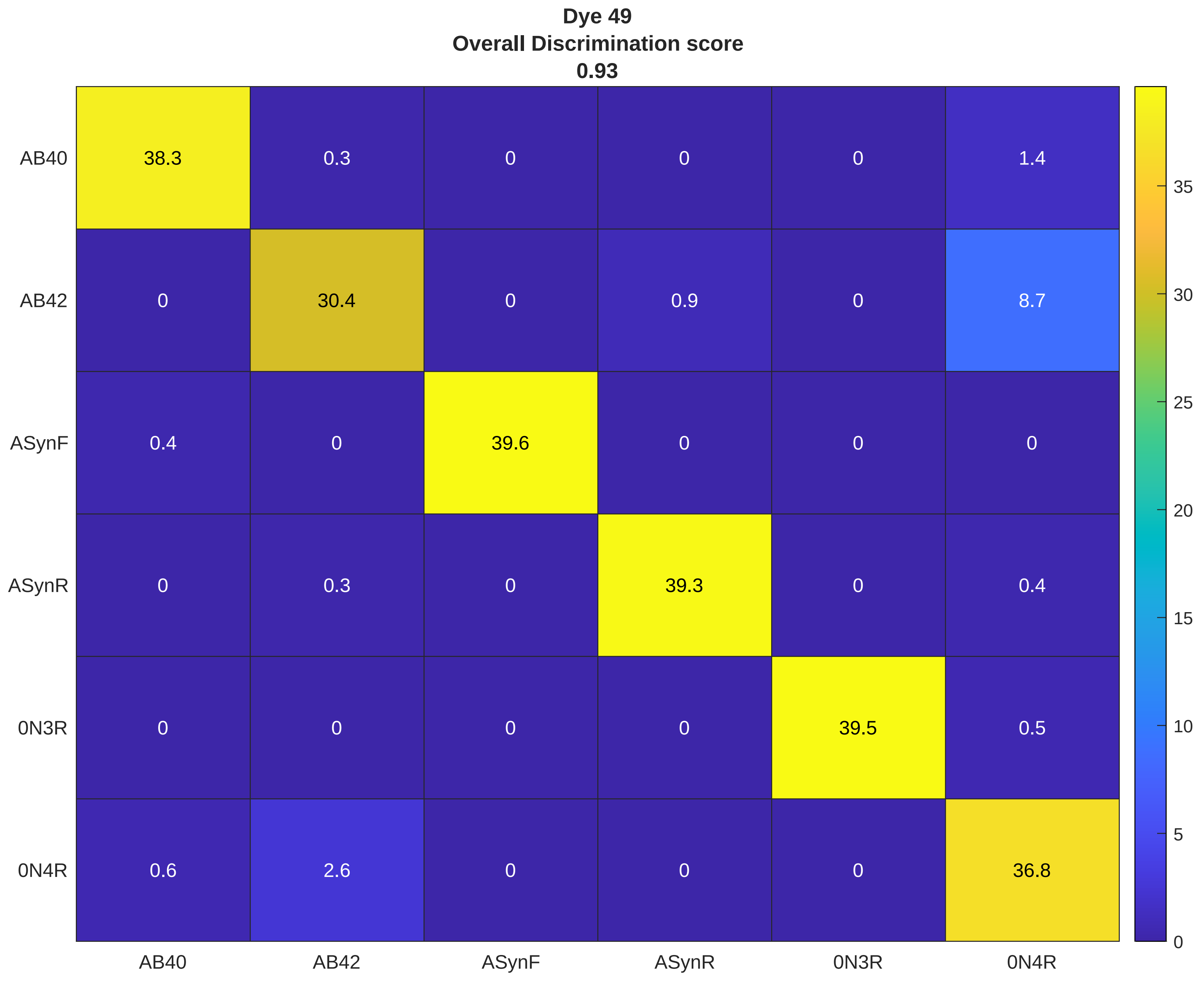
Dye 49
Overall Discrimination score
0.93
AB40
AB42
ASynF
ASynR
0N3R
0N4R
38.3
0.3
0
0
0
1.4
0
30.4
0
0.9
0
8.7
0.4
0
39.6
0
0
0
0
0.3
0
39.3
0
0.4
0
0
0
0
39.5
0.5
0.6
2.6
0
0
0
36.8
AB40
AB42
ASynF
ASynR
0N3R
0N4R
35
30
25
20
15
10
5
0

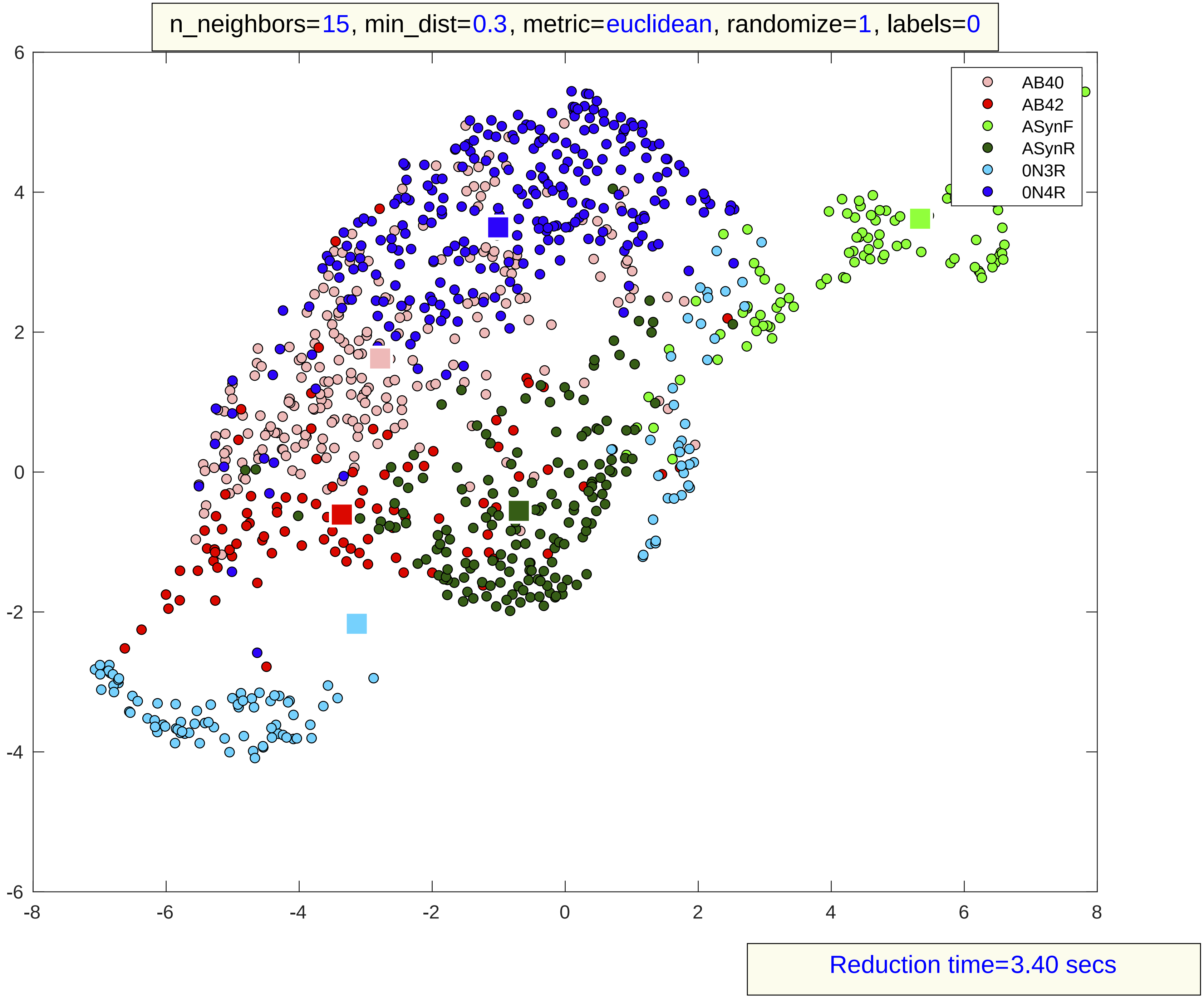
n_neighbors=15, min_dist=0.3, metric=euclidean, randomize=1, labels=0
AB40
AB42
ASynF
ASynR
0N3R
0N4R
6
4
2
0
-2
-4
-6
-8
-6
-4
-2
0
2
4
6
8
Reduction time=3.40 secs

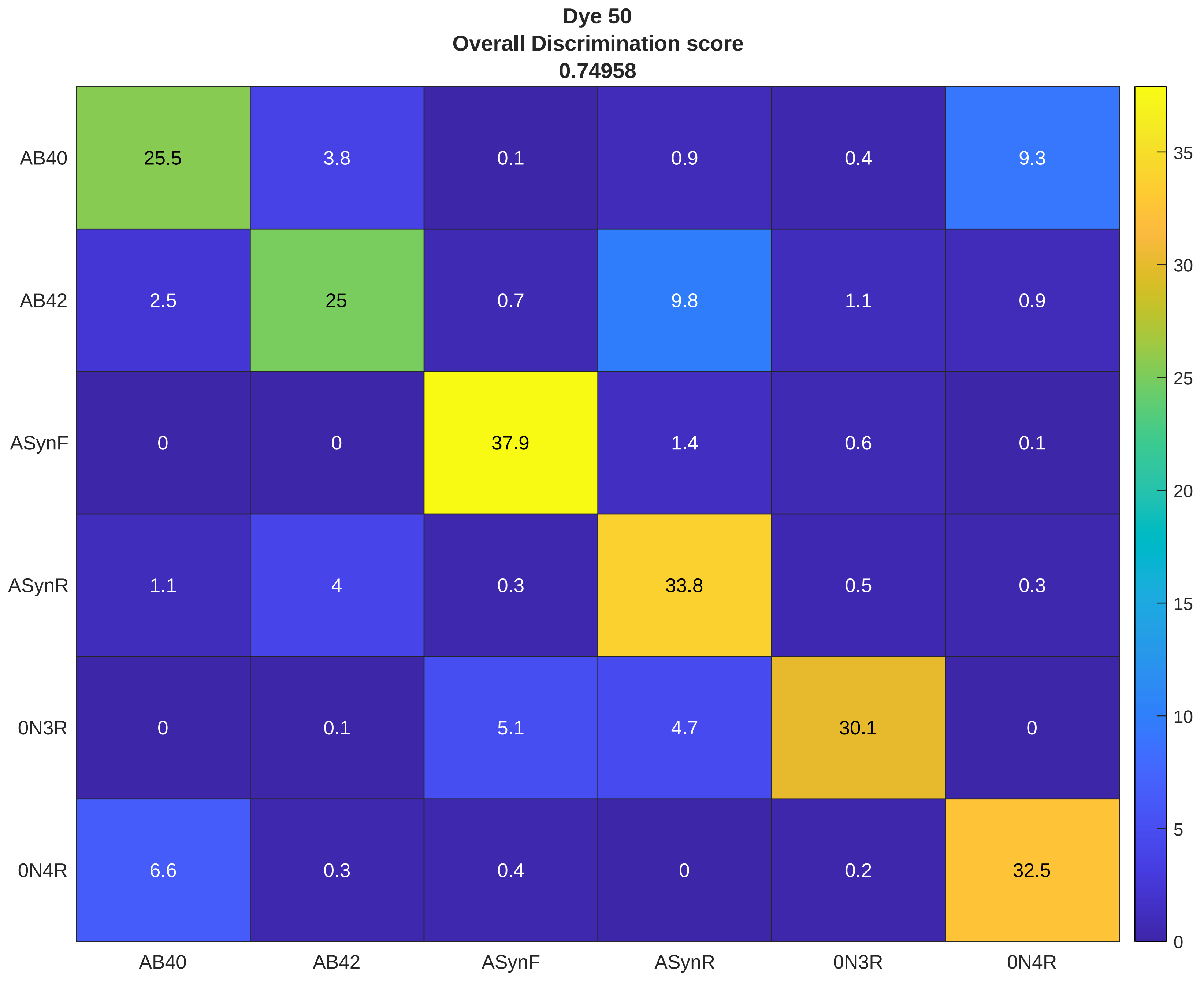
Dye 50
Overall Discrimination score
0.74958
AB40
AB42
ASynF
ASynR
0N3R
0N4R
25.5 3.8 0.1 0.9 0.4 9.3
2.5 25 0.7 9.8 1.1 0.9
0 0 37.9 1.4 0.6 0.1
1.1 4 0.3 33.8 0.5 0.3
0 0.1 5.1 4.7 30.1 0
6.6 0.3 0.4 0 0.2 32.5
AB40
AB42
ASynF
ASynR
0N3R
0N4R
35
30
25
20
15
10
5
0

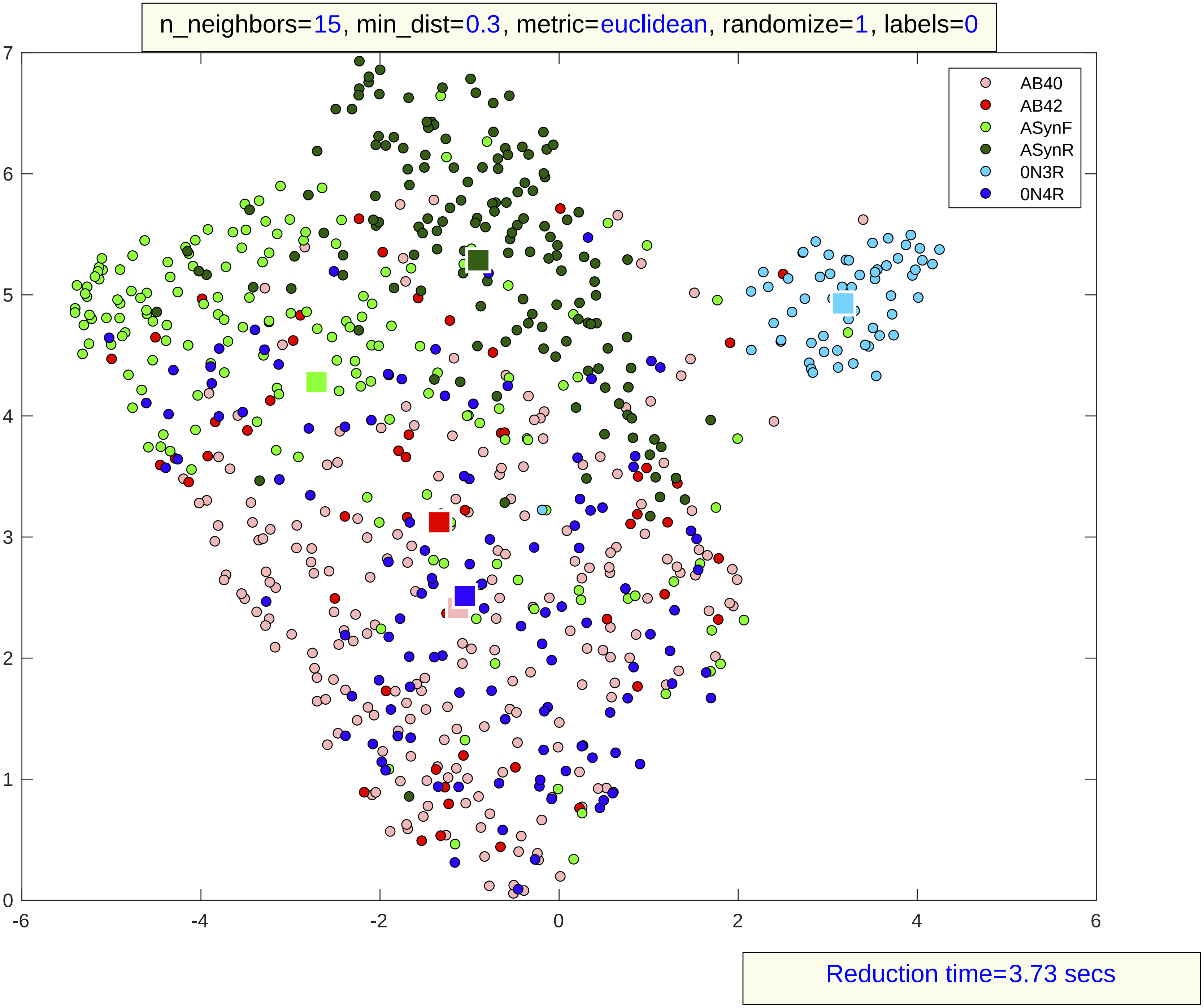
n_neighbors=15, min_dist=0.3, metric=euclidean, randomize=1, labels=0
AB40
AB42
ASynF
ASynR
0N3R
0N4R
7
6
5
4
3
2
1
0
-6
-4
-2
0
2
4
6
Reduction time=3.73 secs

Dye 53
Overall Discrimination score
0.55

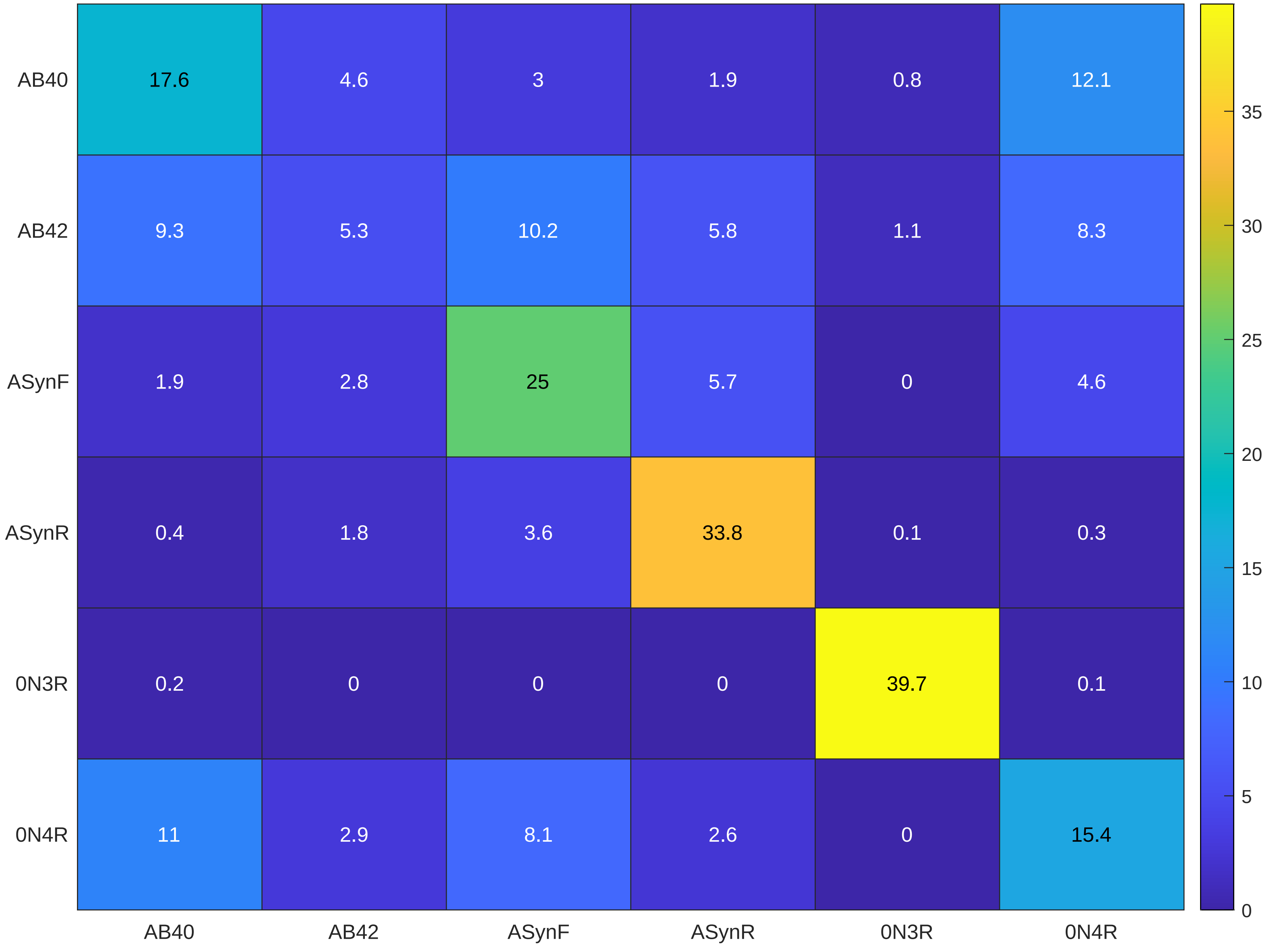

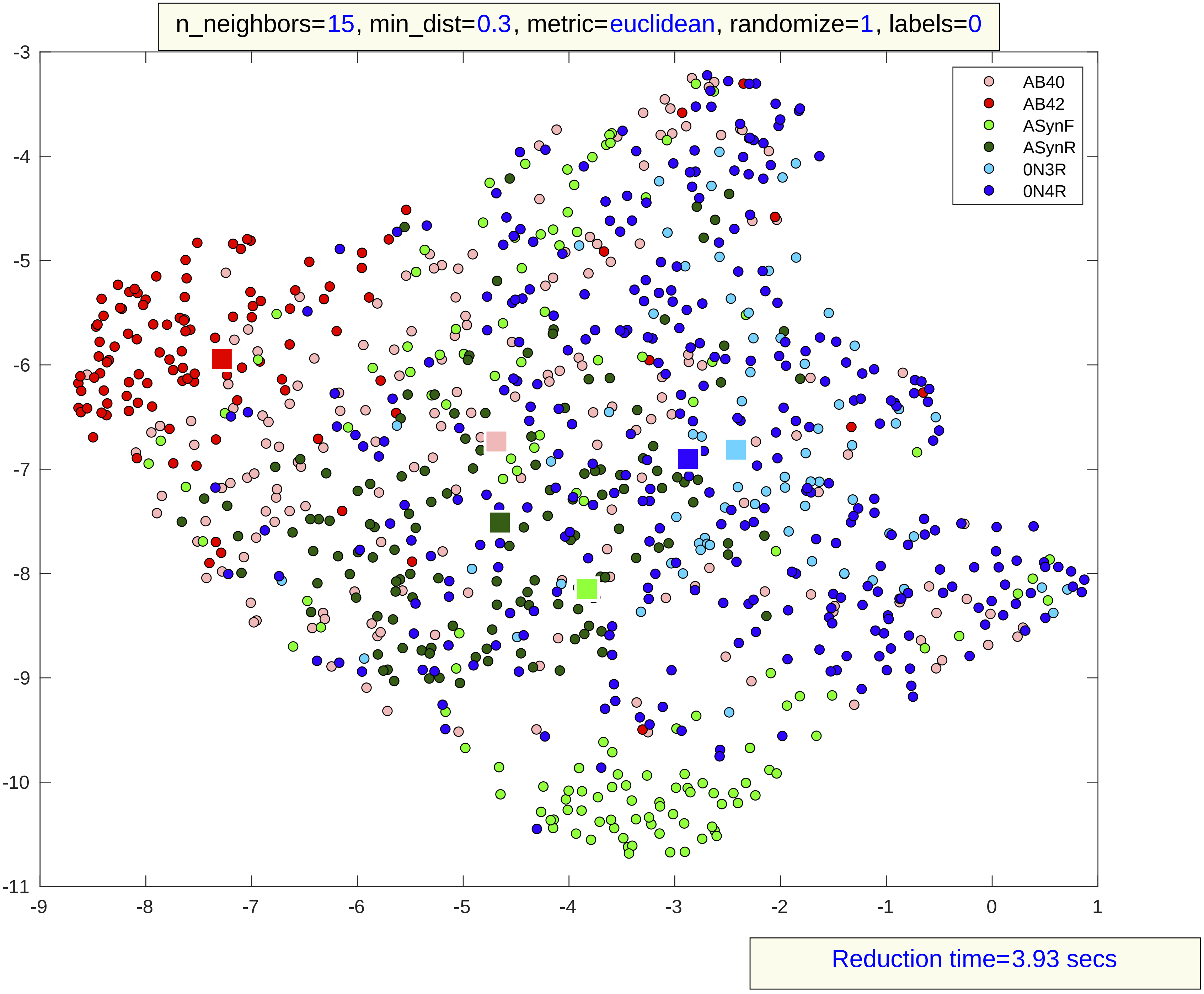
n_neighbors=15, min_dist=0.3, metric=euclidean, randomize=1, labels=0
AB40
AB42
ASynF
ASynR
0N3R
0N4R
-3
-4
-5
-6
-7
-8
-9
-10
-11
-9
-8
-7
-6
-5
-4
-3
-2
-1
0
1
Reduction time=3.93 secs

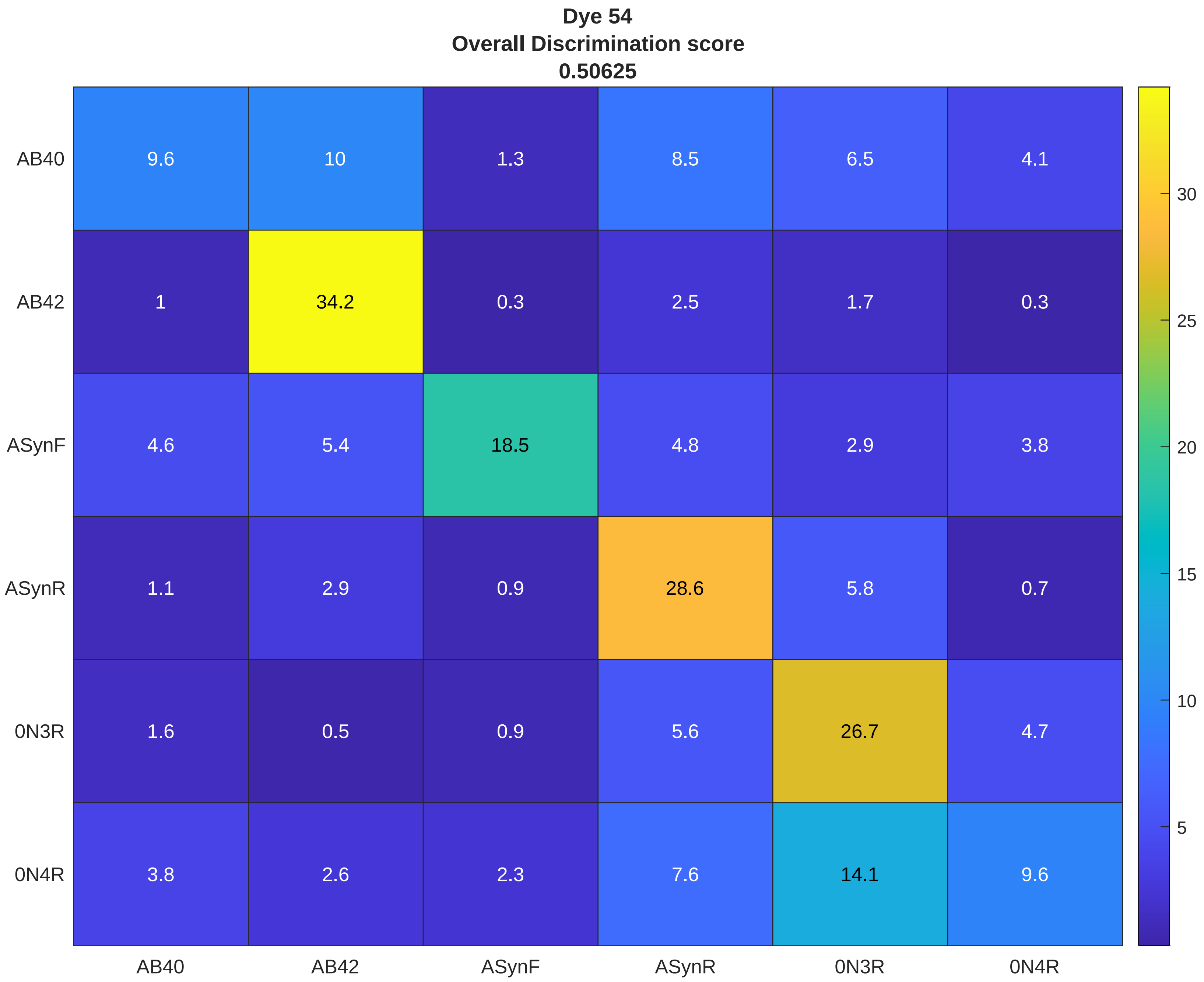
Dye 54
Overall Discrimination score
0.50625
AB40
AB42
ASynF
ASynR
0N3R
0N4R
9.6 10 1.3 8.5 6.5 4.1
1 34.2 0.3 2.5 1.7 0.3
4.6 5.4 18.5 4.8 2.9 3.8
1.1 2.9 0.9 28.6 5.8 0.7
1.6 0.5 0.9 5.6 26.7 4.7
3.8 2.6 2.3 7.6 14.1 9.6
AB40
AB42
ASynF
ASynR
0N3R
0N4R
30
25
20
15
10
5

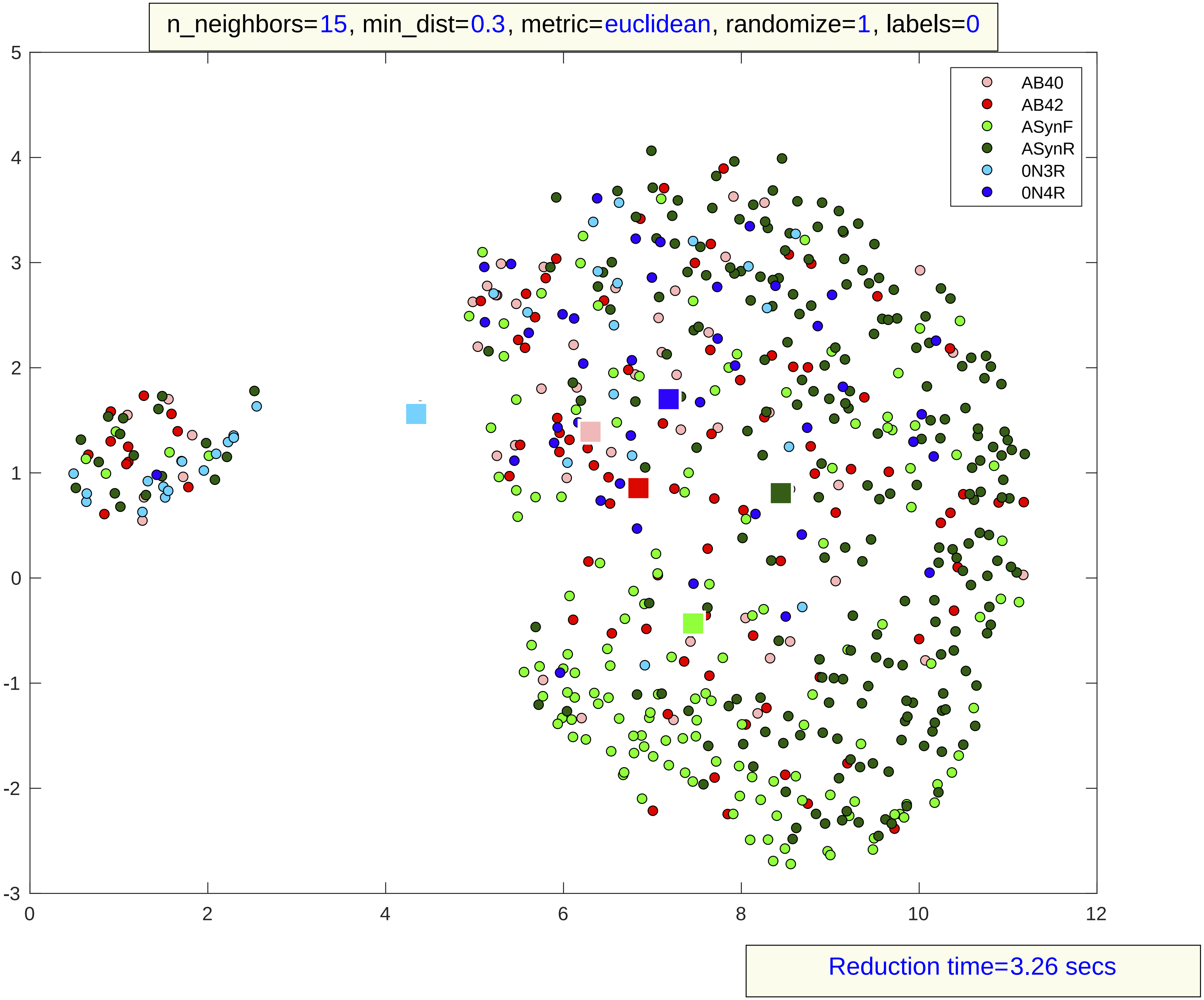

n_neighbors=15, min_dist=0.3, metric=euclidean, randomize=1, labels=0
AB40
AB42
ASynF
ASynR
0N3R
0N4R
5
4
3
2
1
0
-1
-2
-3
0
2
4
6
8
10
12
Reduction time=3.26 secs

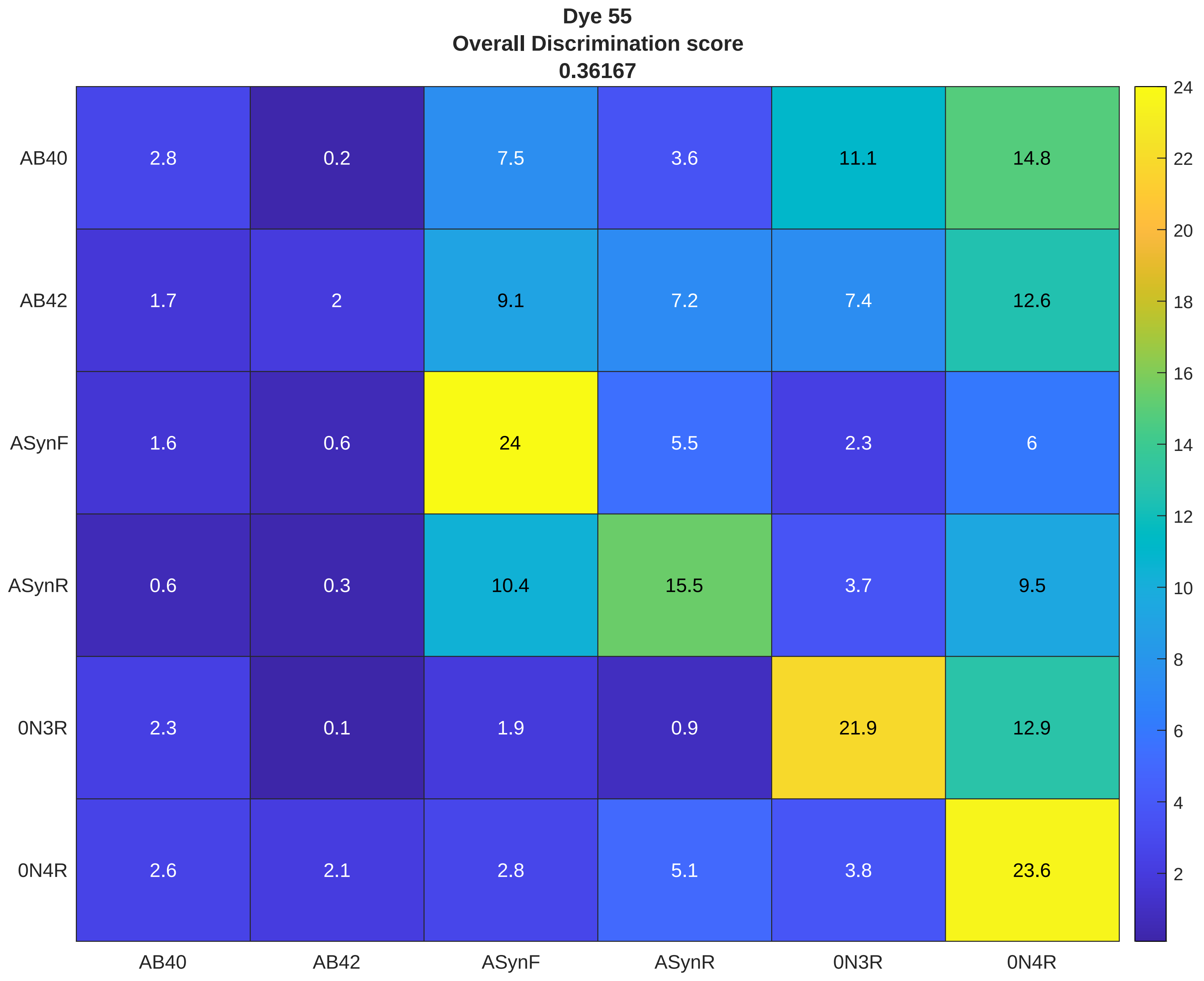
Dye 55
Overall Discrimination score
0.36167
AB40
AB42
ASynF
ASynR
0N3R
0N4R
2.8
0.2
7.5
3.6
11.1
14.8
1.7
2
9.1
7.2
7.4
12.6
1.6
0.6
24
5.5
2.3
6
0.6
0.3
10.4
15.5
3.7
9.5
2.3
0.1
1.9
0.9
21.9
12.9
2.6
2.1
2.8
5.1
3.8
23.6
AB40
AB42
ASynF
ASynR
0N3R
0N4R
24
22
20
18
16
14
12
10
8
6
4
2

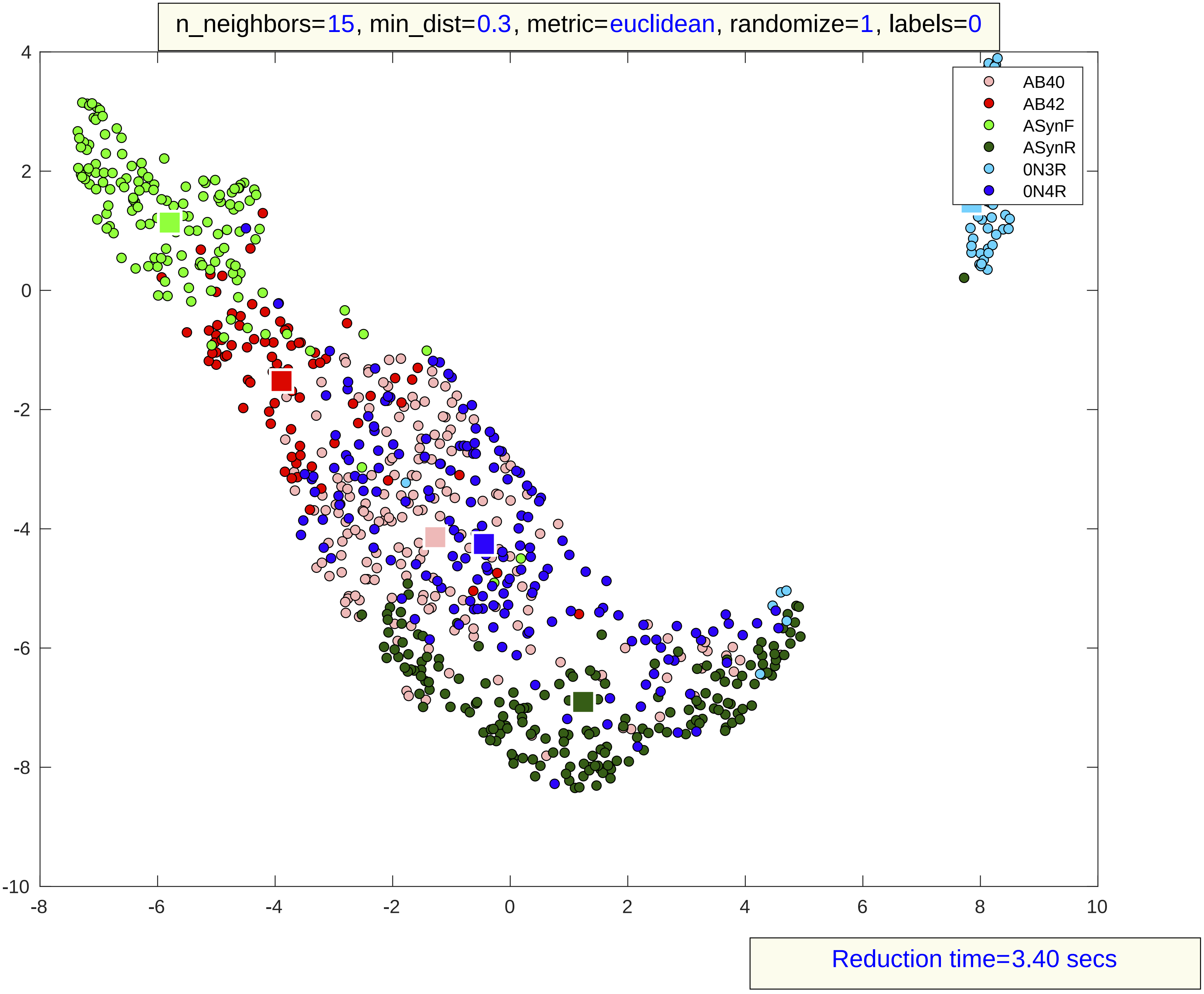
n_neighbors=15, min_dist=0.3, metric=euclidean, randomize=1, labels=0
AB40
AB42
ASynF
ASynR
0N3R
0N4R
4
2
0
-2
-4
-6
-8
-10
-8
-6
-4
-2
0
2
4
6
8
10
Reduction time=3.40 secs

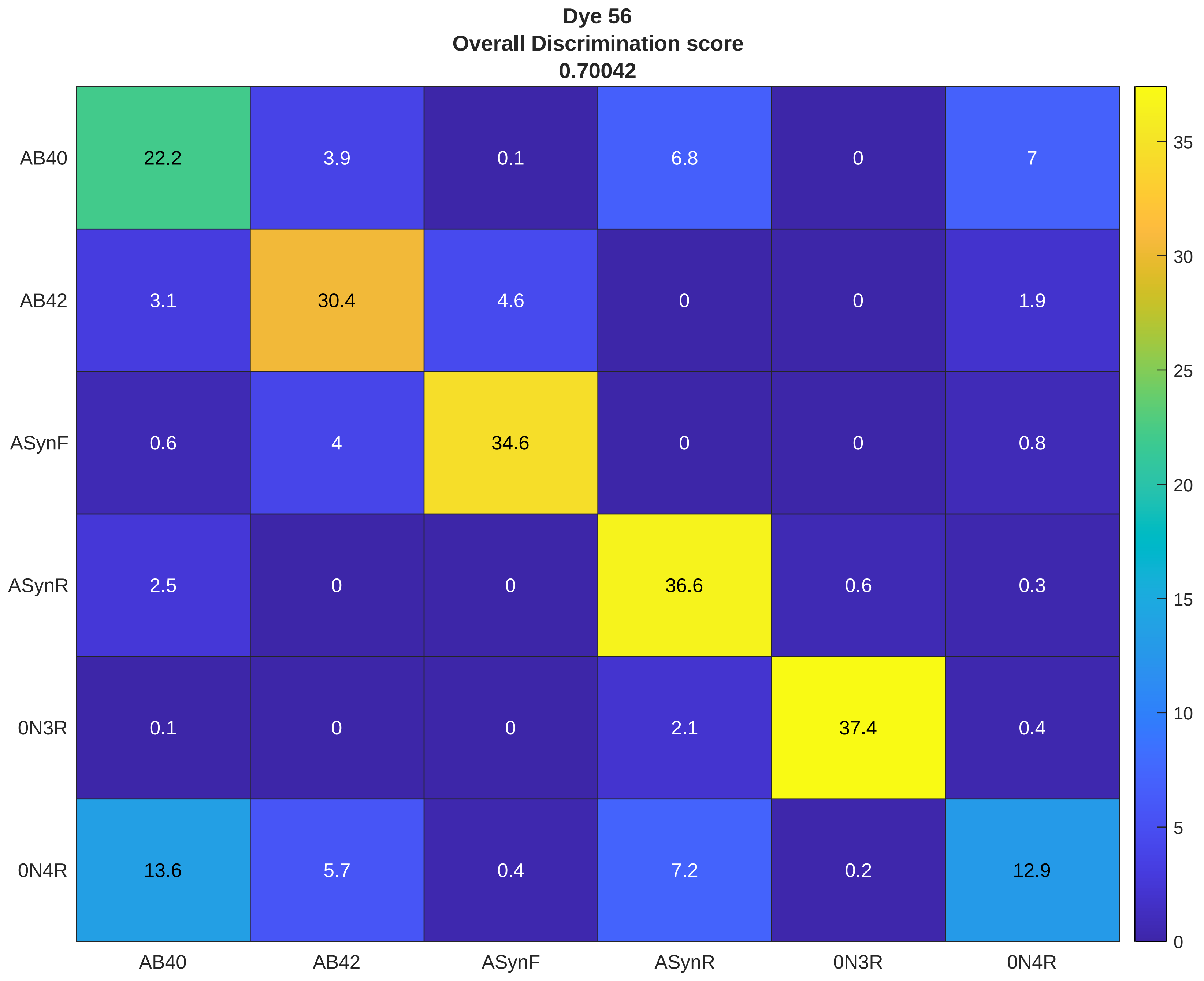
Dye 56
Overall Discrimination score
0.70042
AB40
AB42
ASynF
ASynR
0N3R
0N4R
22.2
3.9
0.1
6.8
0
7
3.1
30.4
4.6
0
0
1.9
0.6
4
34.6
0
0
0.8
2.5
0
0
36.6
0.6
0.3
0.1
0
0
2.1
37.4
0.4
13.6
5.7
0.4
7.2
0.2
12.9
AB40
AB42
ASynF
ASynR
0N3R
0N4R
35
30
25
20
15
10
5
0

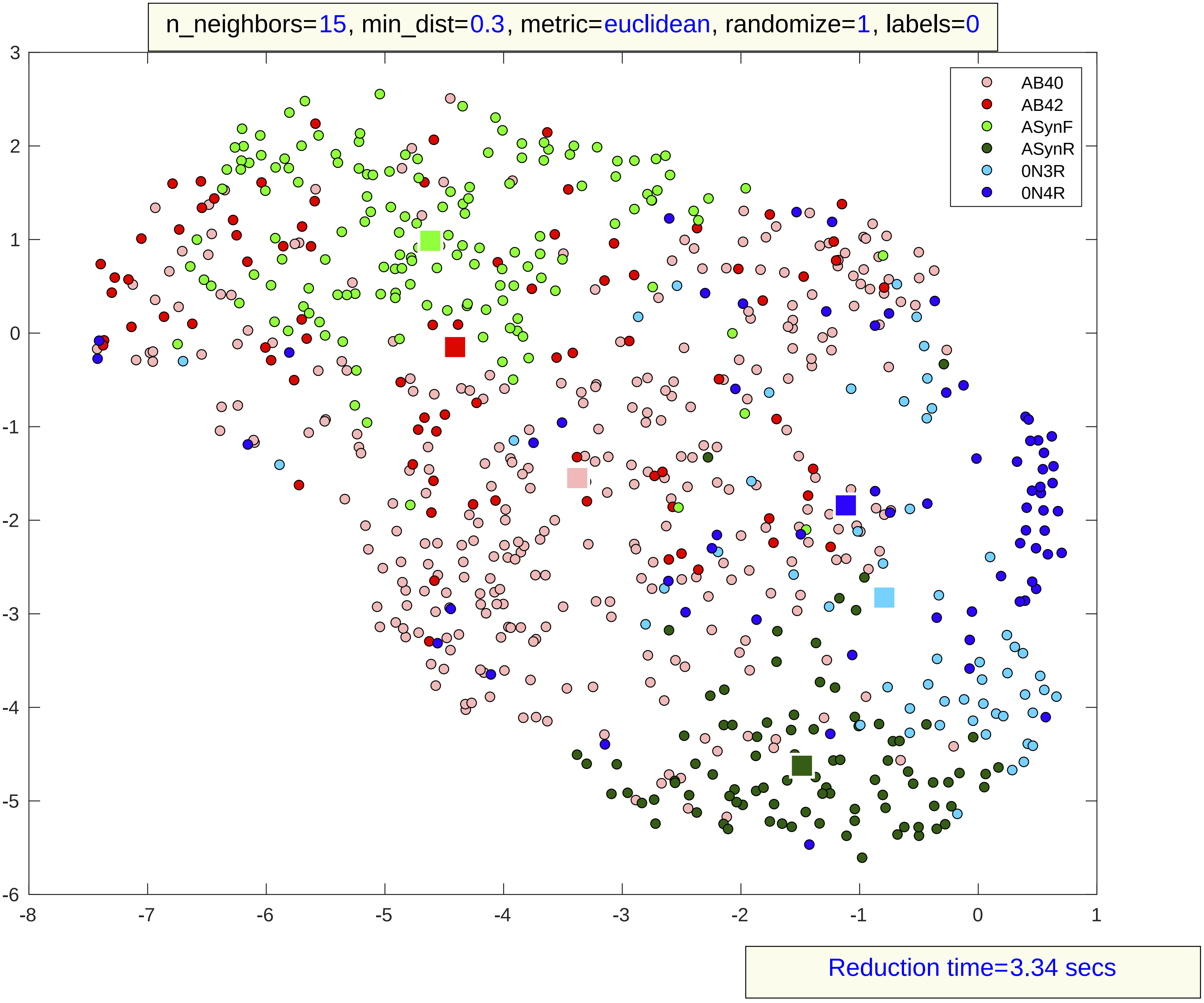
n_neighbors=15, min_dist=0.3, metric=euclidean, randomize=1, labels=0
AB40
AB42
ASynF
ASynR
0N3R
0N4R
3
2
1
0
-1
-2
-3
-4
-5
-6
-8
-7
-6
-5
-4
-3
-2
-1
0
1
Reduction time=3.34 secs

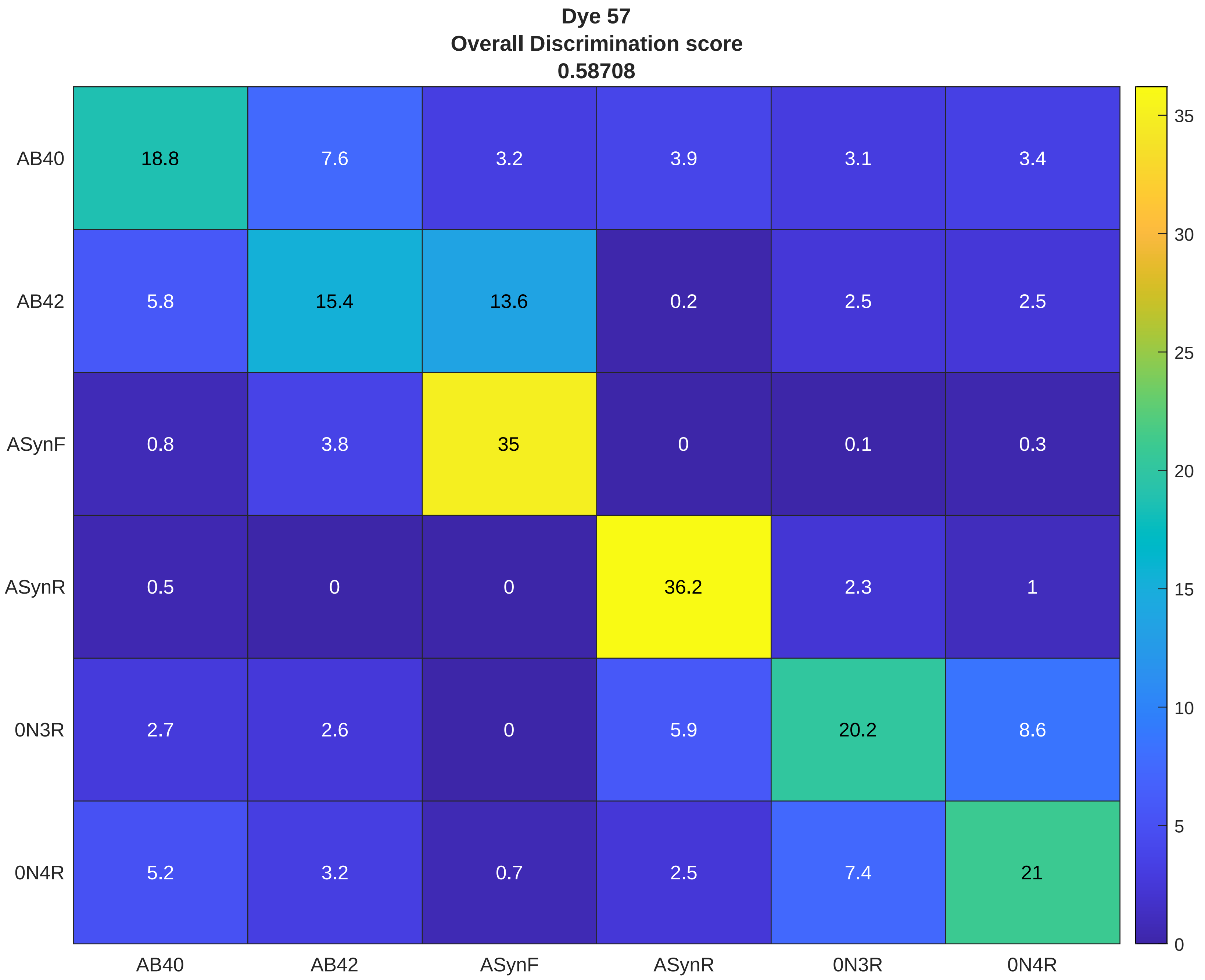
Dye 57
Overall Discrimination score
0.58708
AB40
18.8
7.6
3.2
3.9
3.1
3.4
AB42
5.8
15.4
13.6
0.2
2.5
2.5
ASynF
0.8
3.8
35
0
0.1
0.3
ASynR
0.5
0
0
36.2
2.3
1
0N3R
2.7
2.6
0
5.9
20.2
8.6
0N4R
5.2
3.2
0.7
2.5
7.4
21
AB40
AB42
ASynF
ASynR
0N3R
0N4R
35
30
25
20
15
10
5
0

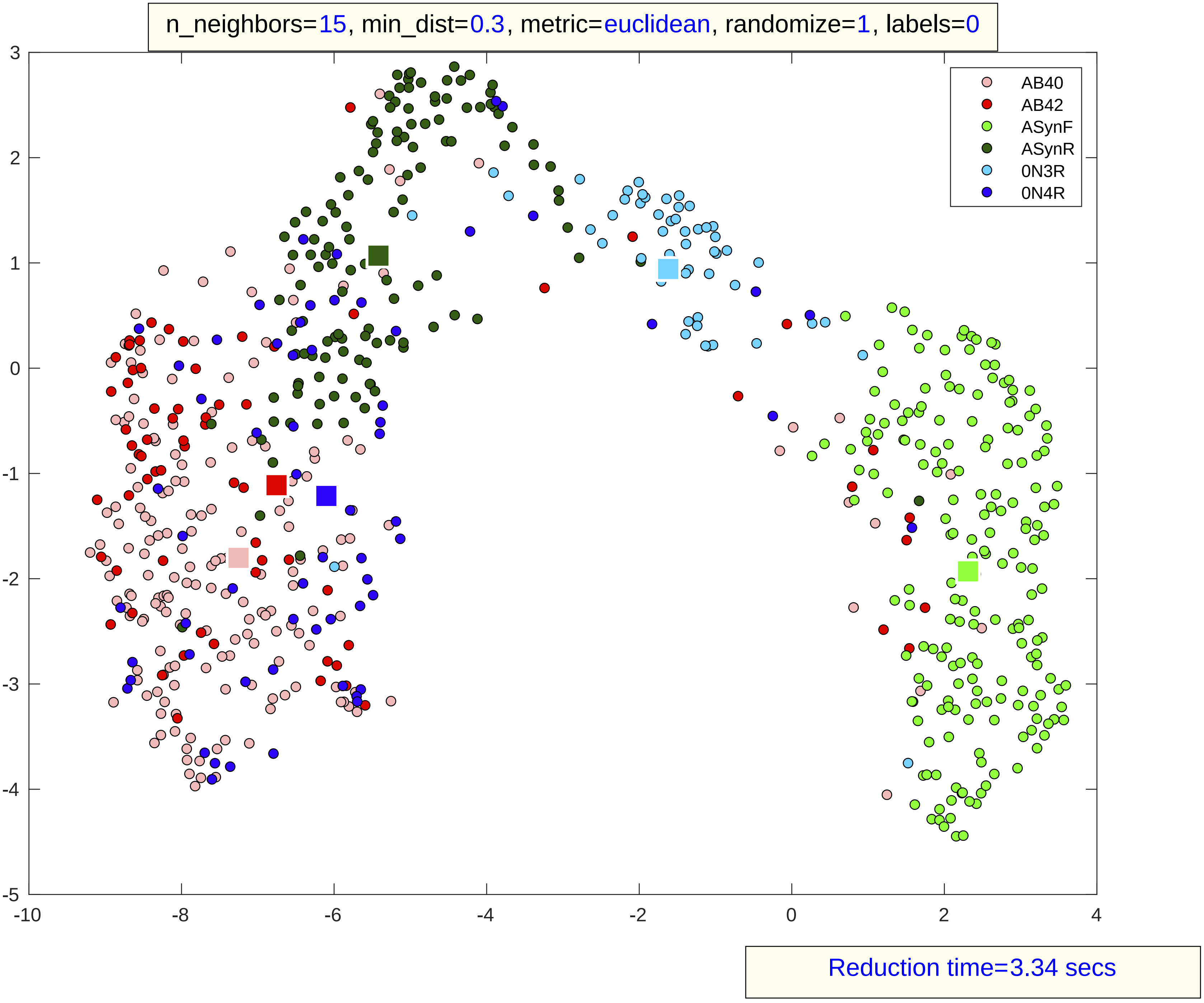
n_neighbors=15, min_dist=0.3, metric=euclidean, randomize=1, labels=0
AB40
AB42
ASynF
ASynR
0N3R
0N4R
3
2
1
0
-1
-2
-3
-4
-5
-10
-8
-6
-4
-2
0
2
4
Reduction time=3.34 secs

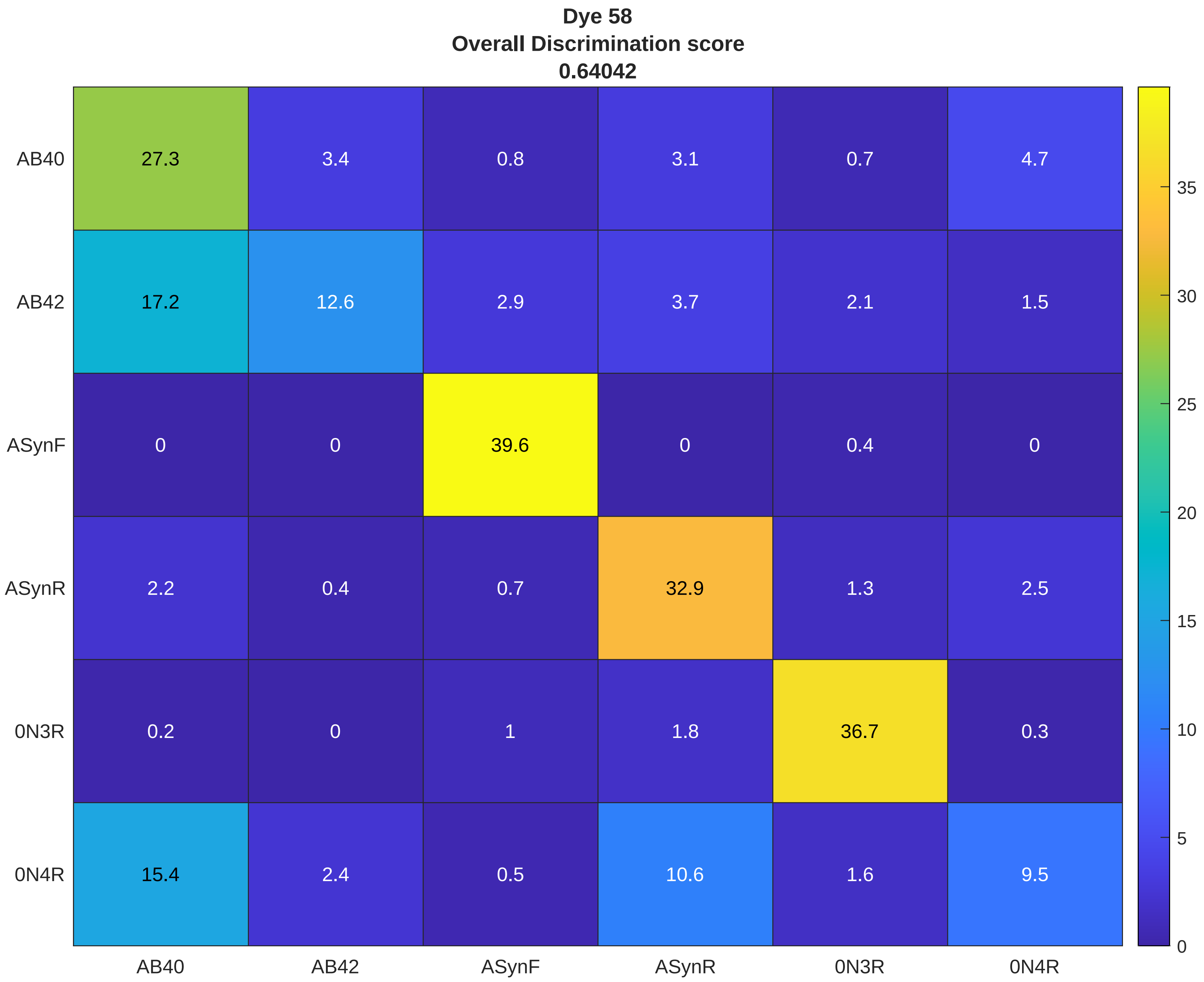
Dye 58
Overall Discrimination score
0.64042
AB40
AB42
ASynF
ASynR
0N3R
0N4R
27.3
3.4
0.8
3.1
0.7
4.7
17.2
12.6
2.9
3.7
2.1
1.5
0
0
39.6
0
0.4
0
2.2
0.4
0.7
32.9
1.3
2.5
0.2
0
1
1.8
36.7
0.3
15.4
2.4
0.5
10.6
1.6
9.5
35
30
25
20
15
10
5
0

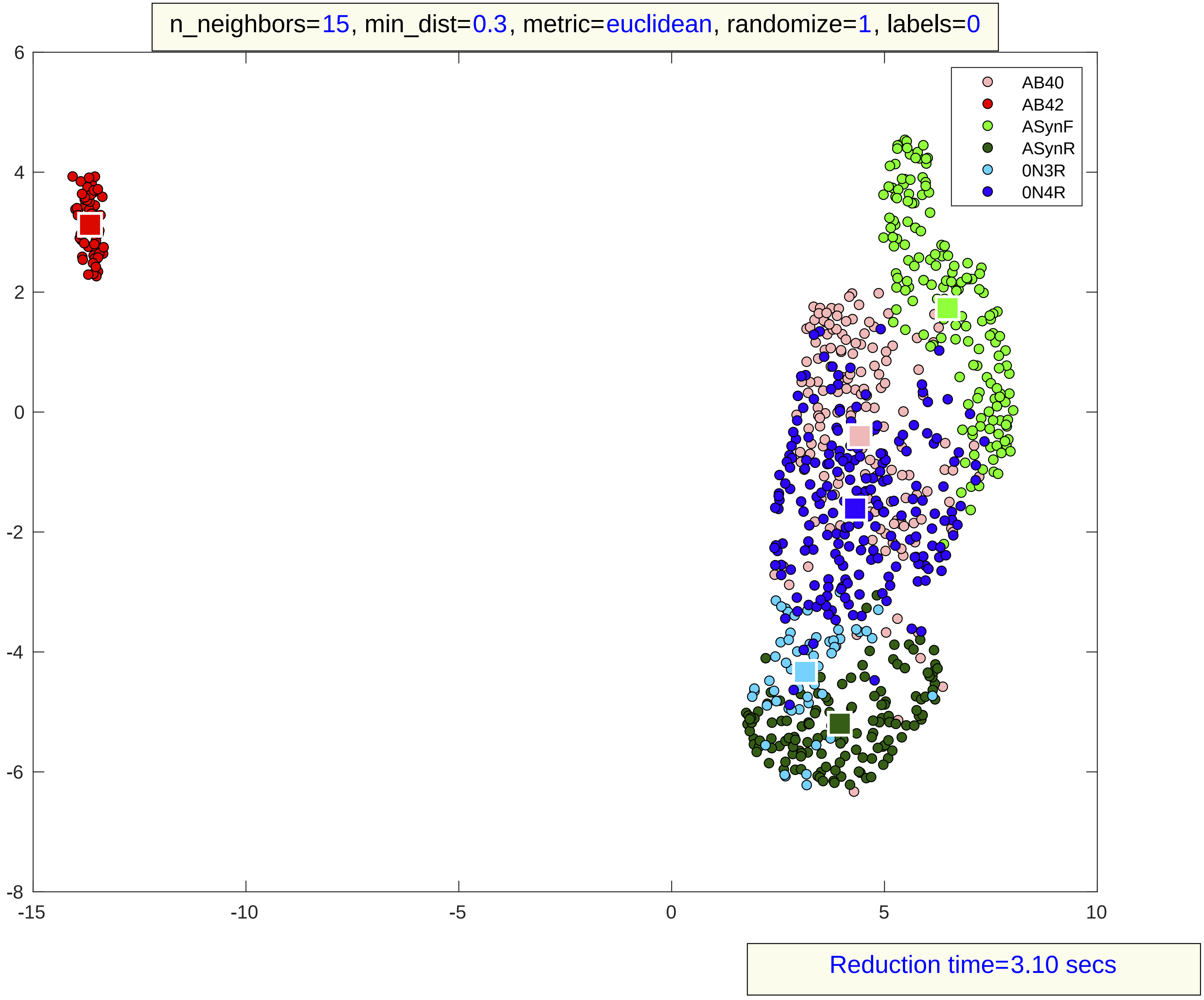

n_neighbors=15, min_dist=0.3, metric=euclidean, randomize=1, labels=0
AB40
AB42
ASynF
ASynR
0N3R
0N4R
6
4
2
0
-2
-4
-6
-8
-15
-10
-5
0
5
10
Reduction time=3.10 secs

Dye 59
Overall Discrimination score
0.79208

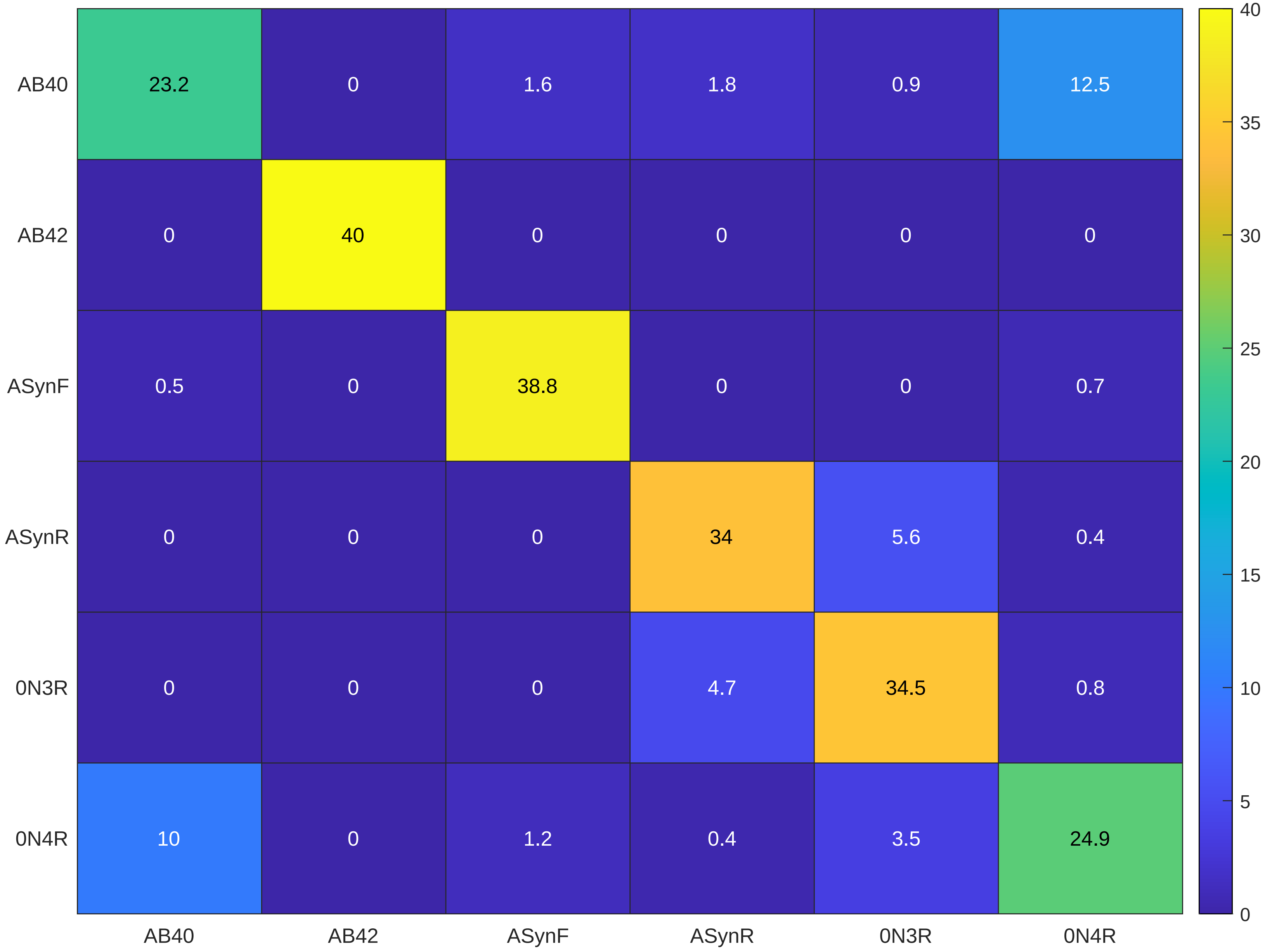

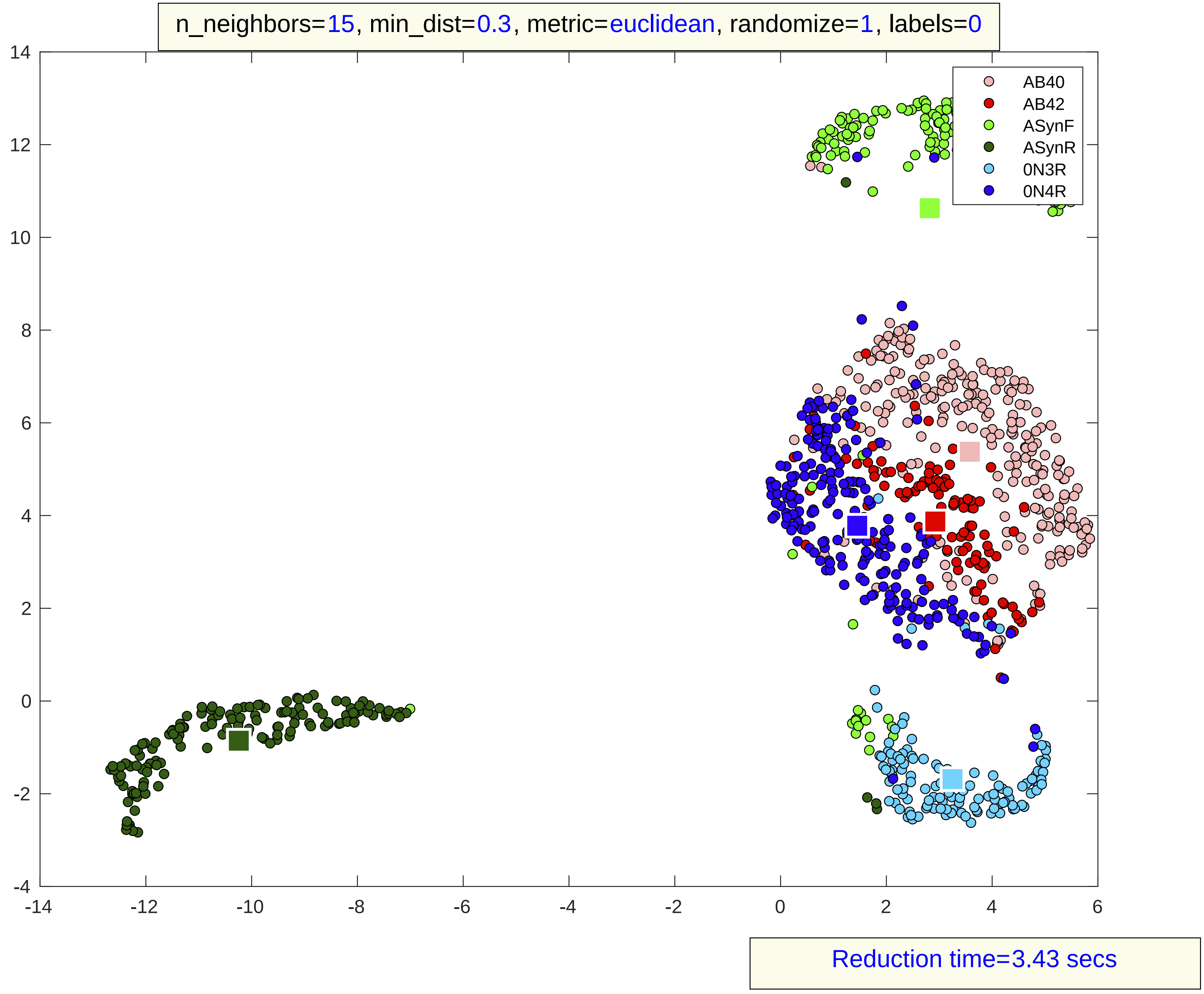

n_neighbors=15, min_dist=0.3, metric=euclidean, randomize=1, labels=0
AB40
AB42
ASynF
ASynR
0N3R
0N4R
14
12
10
8
6
4
2
0
-2
-4
-14
-12
-10
-8
-6
-4
-2
0
2
4
6
Reduction time=3.43 secs

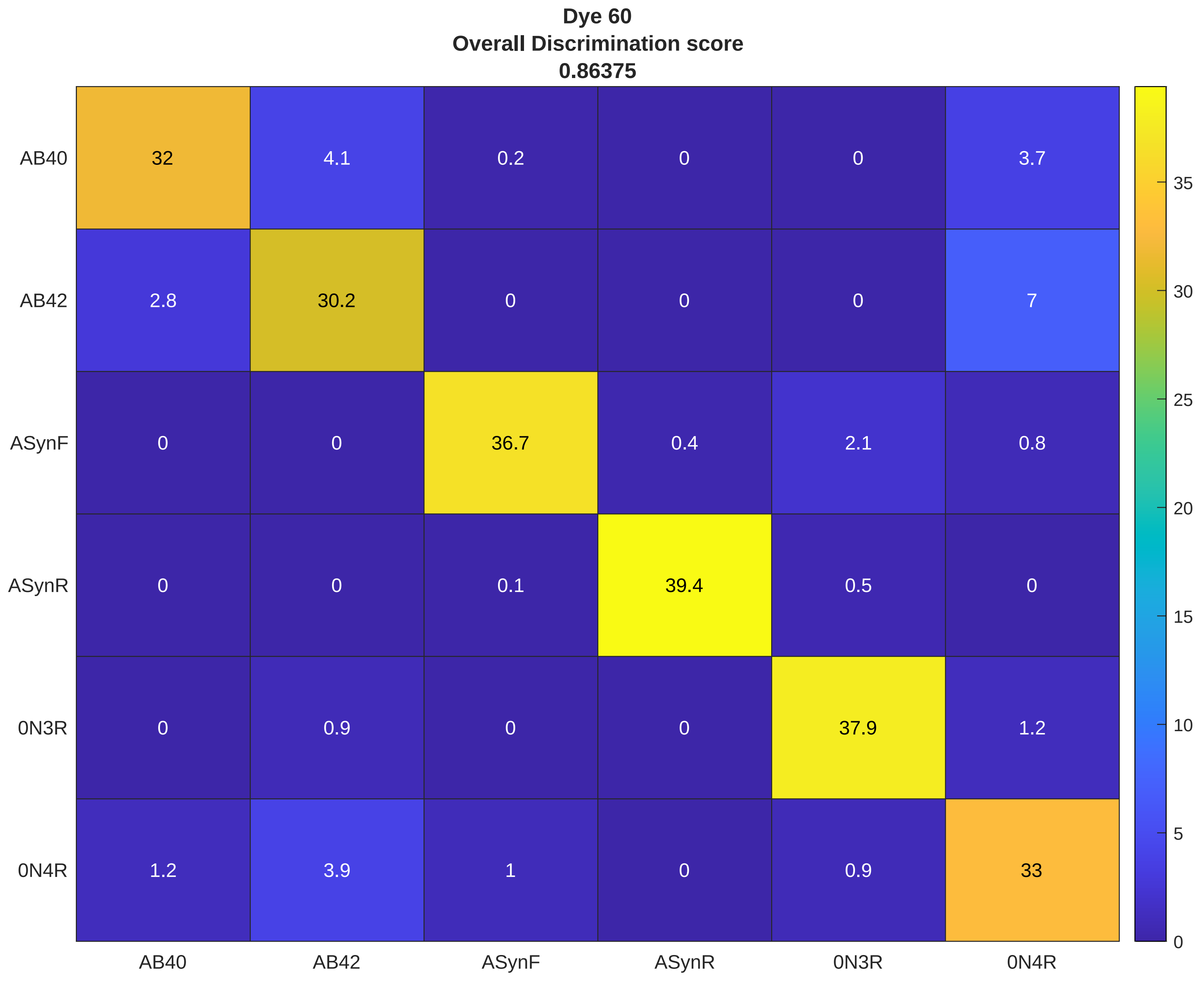
Dye 60
Overall Discrimination score
0.86375
AB40
AB42
ASynF
ASynR
0N3R
0N4R
32 4.1 0.2 0 0 3.7
2.8 30.2 0 0 0 7
0 0 36.7 0.4 2.1 0.8
0 0 0.1 39.4 0.5 0
0 0.9 0 0 37.9 1.2
1.2 3.9 1 0 0.9 33
AB40
AB42
ASynF
ASynR
0N3R
0N4R
35
30
25
20
15
10
5
0

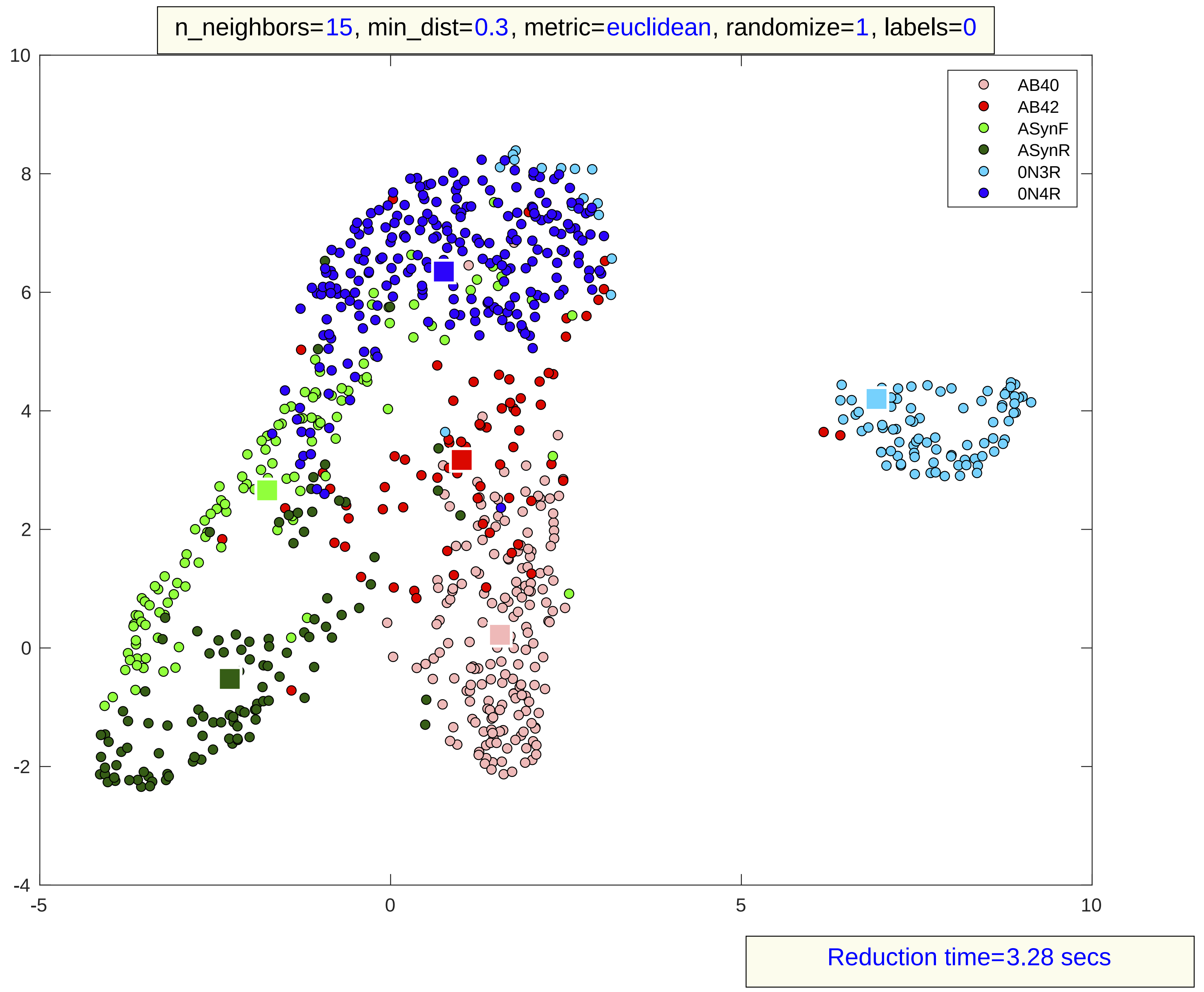

n_neighbors=15, min_dist=0.3, metric=euclidean, randomize=1, labels=0
AB40
AB42
ASynF
ASynR
0N3R
0N4R
10
8
6
4
2
0
-2
-4
-5
0
5
10
Reduction time=3.28 secs

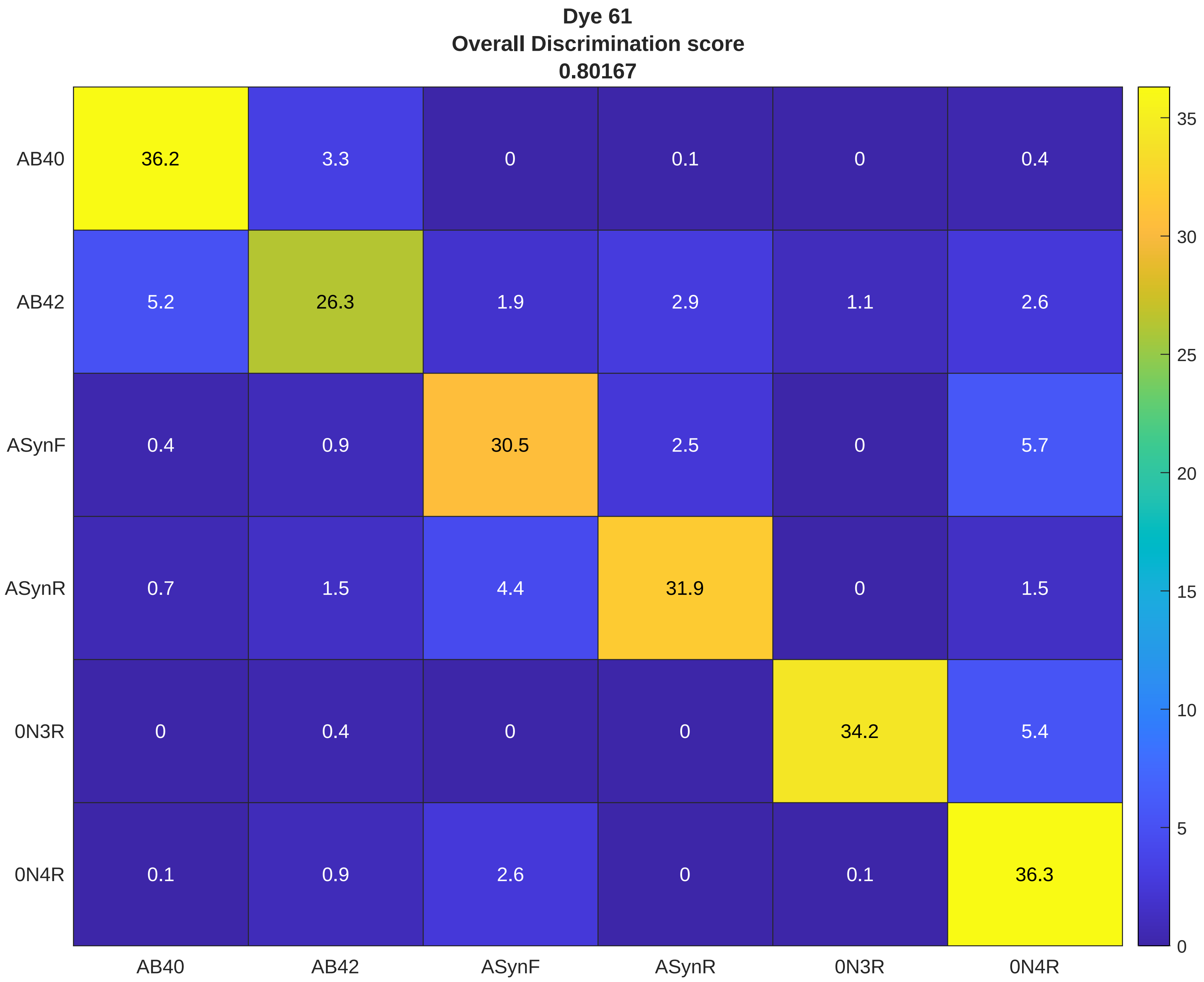
Dye 61
Overall Discrimination score
0.80167
AB40
AB42
ASynF
ASynR
0N3R
0N4R
36.2
3.3
0
0.1
0
0.4
5.2
26.3
1.9
2.9
1.1
2.6
0.4
0.9
30.5
2.5
0
5.7
0.7
1.5
4.4
31.9
0
1.5
0
0.4
0
0
34.2
5.4
0.1
0.9
2.6
0
0.1
36.3
AB40
AB42
ASynF
ASynR
0N3R
0N4R
35
30
25
20
15
10
5
0

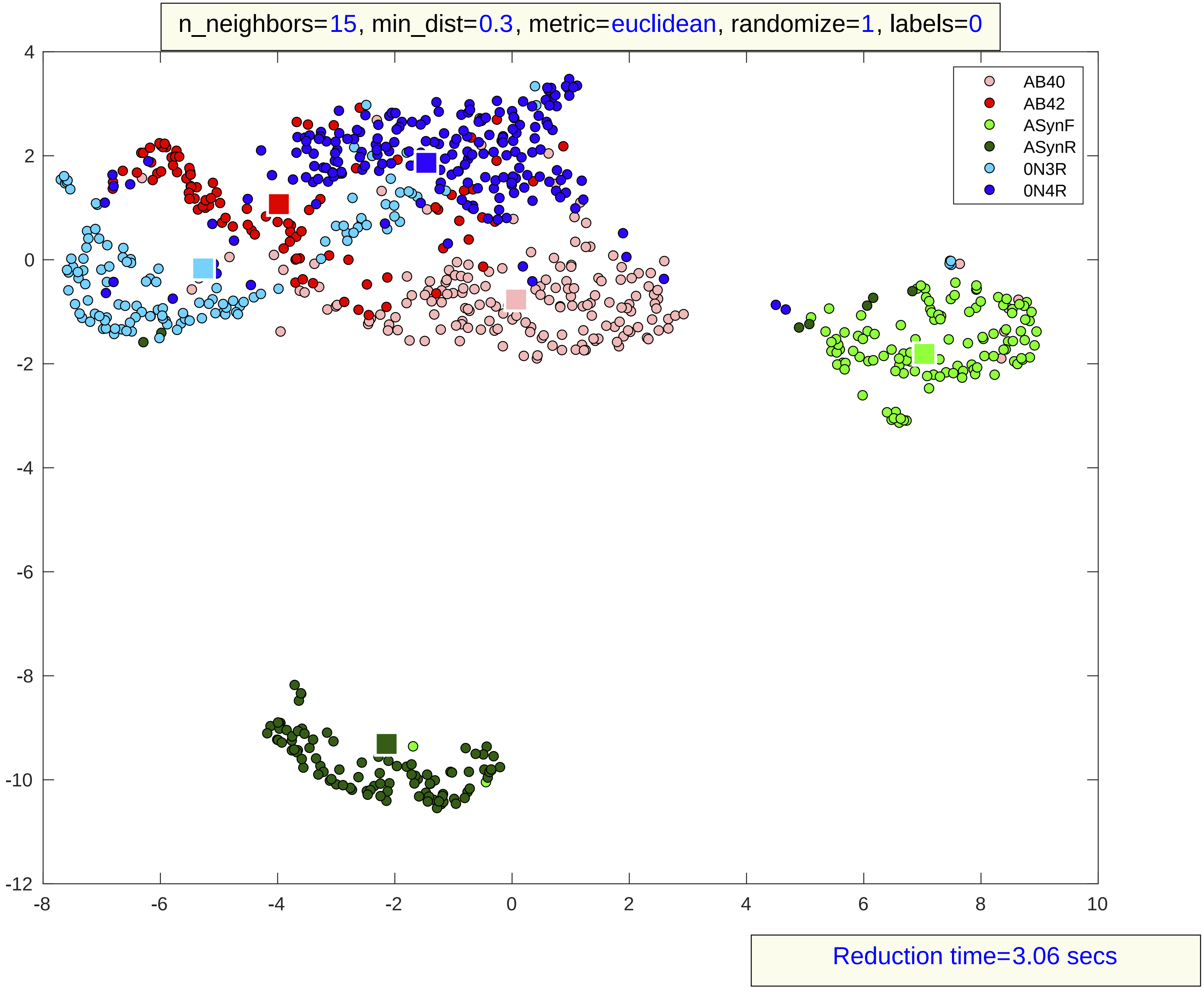

n_neighbors=15, min_dist=0.3, metric=euclidean, randomize=1, labels=0
AB40
AB42
ASynF
ASynR
0N3R
0N4R
4
2
0
-2
-4
-6
-8
-10
-12
-8
-6
-4
-2
0
2
4
6
8
10
Reduction time=3.06 secs

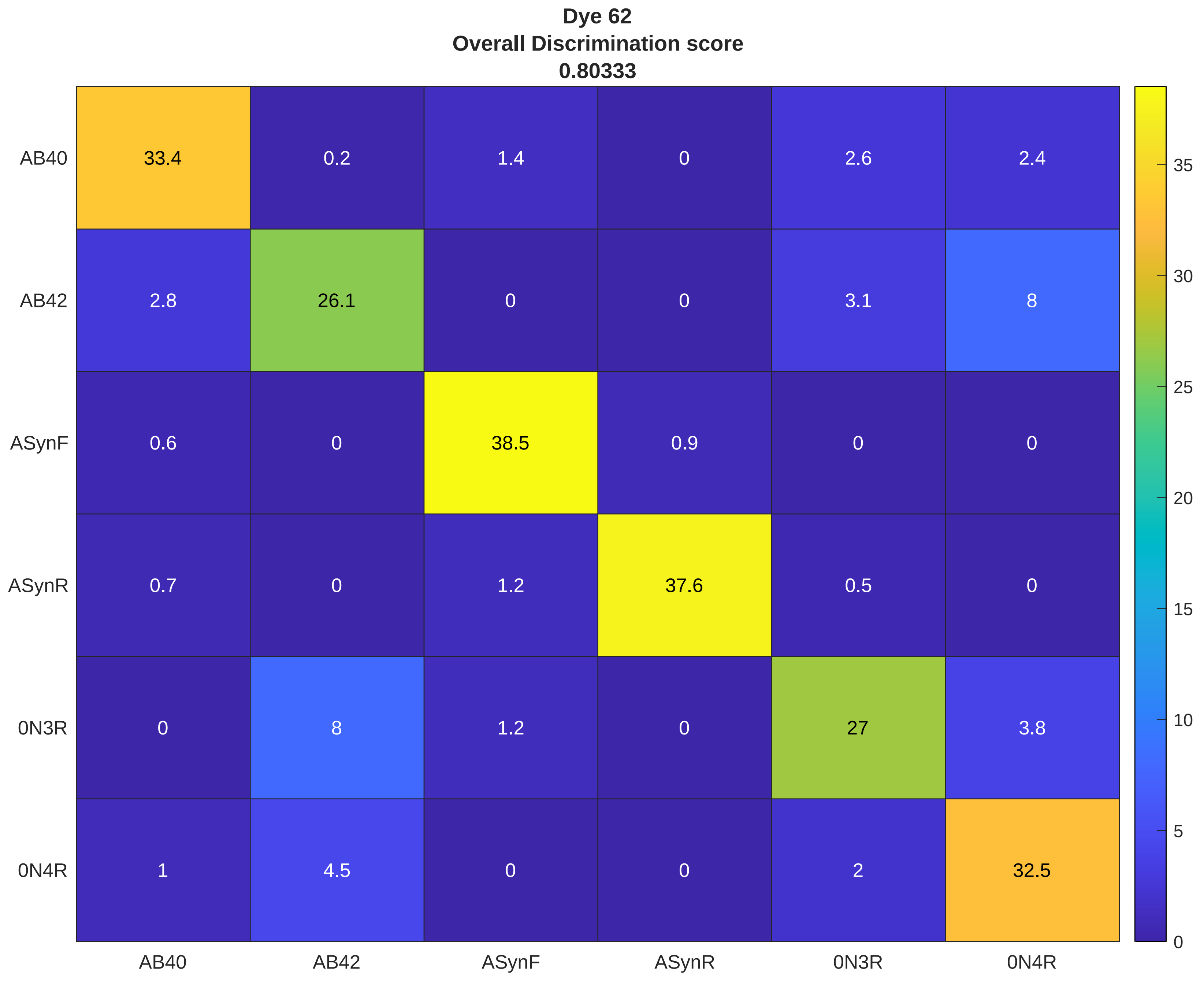
Dye 62
Overall Discrimination score
0.80333
AB40
AB42
ASynF
ASynR
0N3R
0N4R
33.4
0.2
1.4
0
2.6
2.4
2.8
26.1
0
0
3.1
8
0.6
0
38.5
0.9
0
0
0.7
0
1.2
37.6
0.5
0
0
8
1.2
0
27
3.8
1
4.5
0
0
2
32.5
35
30
25
20
15
10
5
0

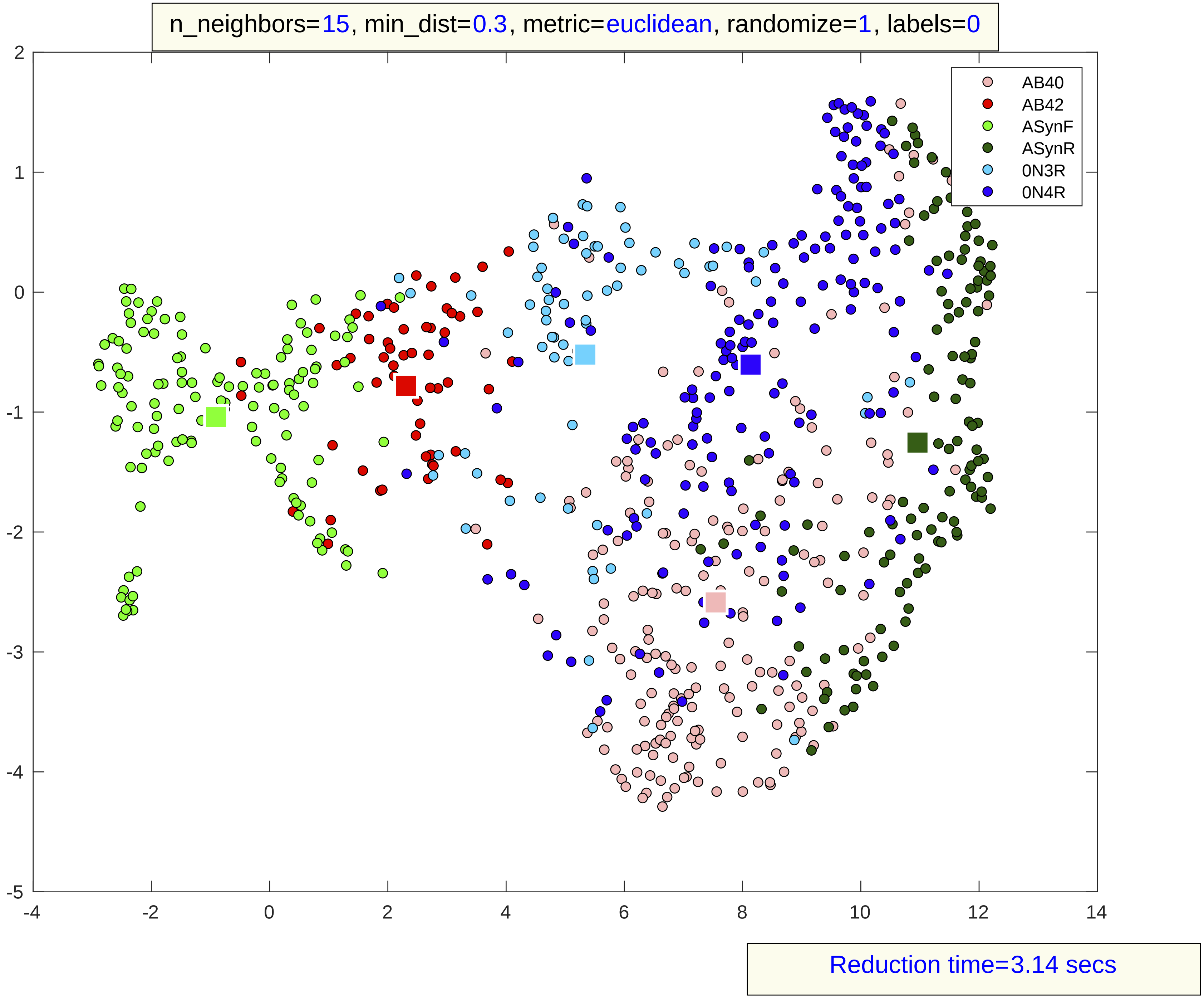

n_neighbors=15, min_dist=0.3, metric=euclidean, randomize=1, labels=0
AB40
AB42
ASynF
ASynR
0N3R
0N4R
2
1
0
-1
-2
-3
-4
-5
-4
-2
0
2
4
6
8
10
12
14
Reduction time=3.14 secs

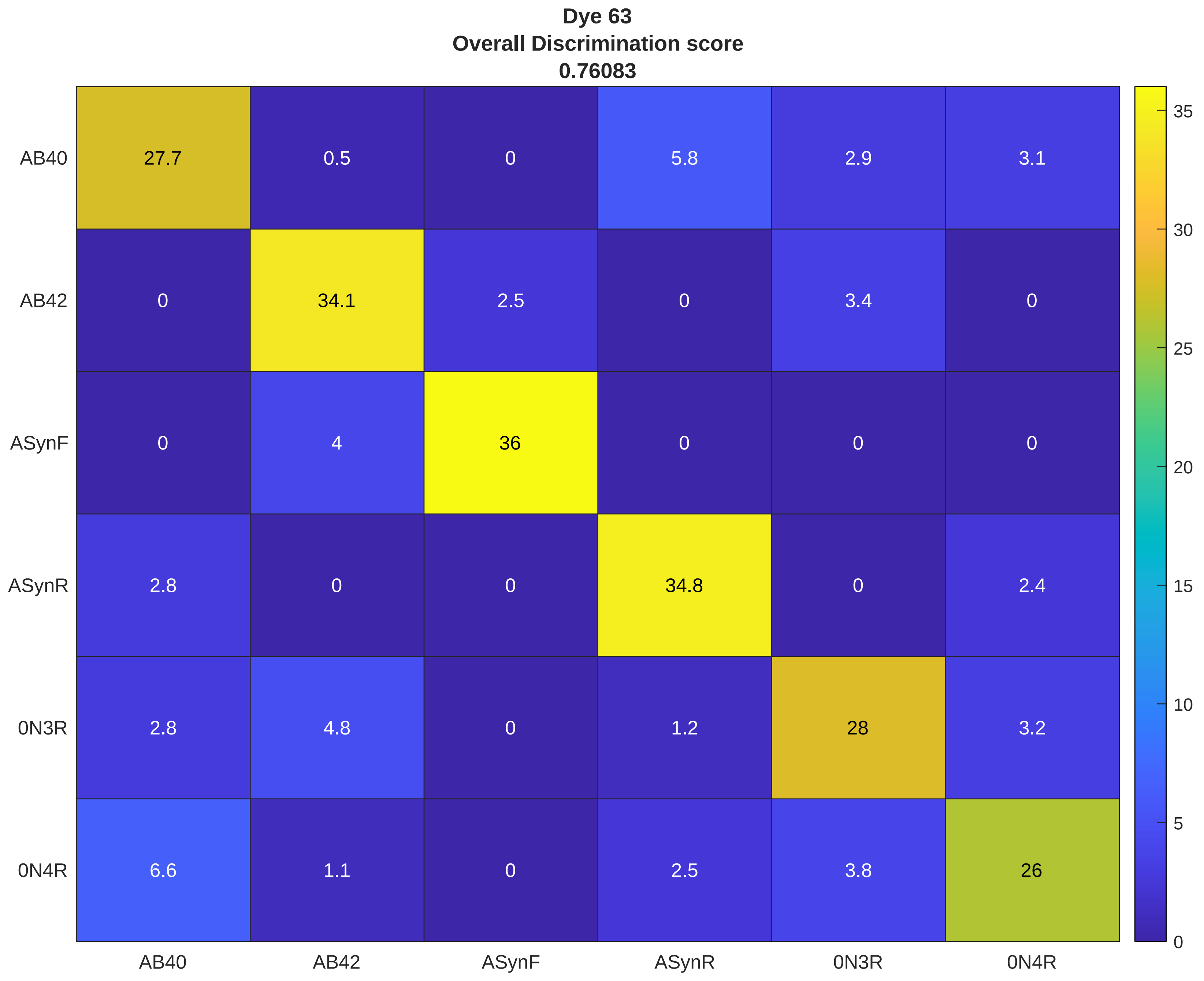
Dye 63
Overall Discrimination score
0.76083
AB40
27.7
0.5
0
5.8
2.9
3.1
AB42
0
34.1
2.5
0
3.4
0
ASynF
0
4
36
0
0
0
ASynR
2.8
0
0
34.8
0
2.4
0N3R
2.8
4.8
0
1.2
28
3.2
0N4R
6.6
1.1
0
2.5
3.8
26
AB40
AB42
ASynF
ASynR
0N3R
0N4R
35
30
25
20
15
10
5
0

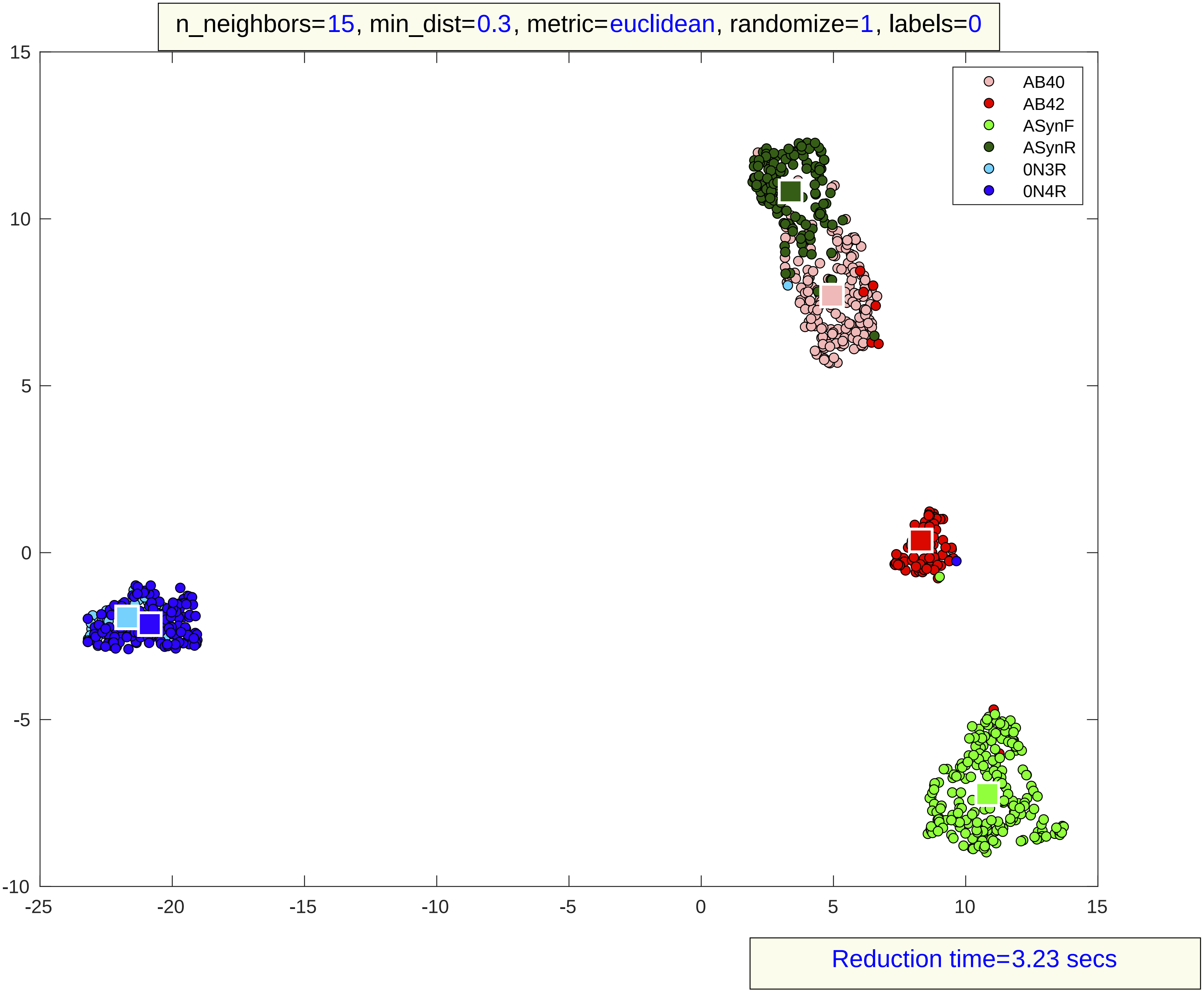
n_neighbors=15, min_dist=0.3, metric=euclidean, randomize=1, labels=0
AB40
AB42
ASynF
ASynR
0N3R
0N4R
15
10
5
0
-5
-10
-25
-20
-15
-10
-5
0
5
10
15
Reduction time=3.23 secs

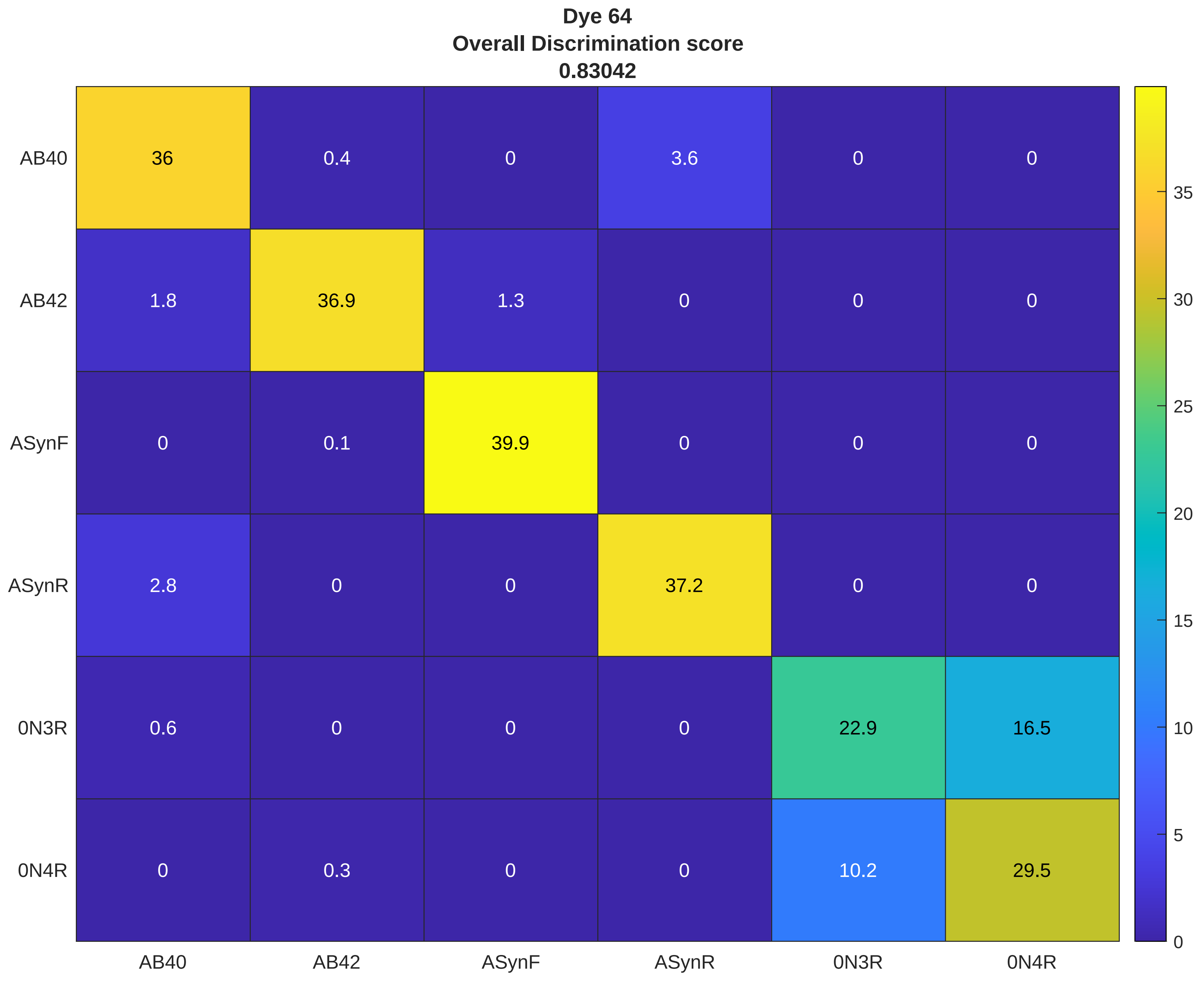
Dye 64
Overall Discrimination score
0.83042
AB40
AB42
ASynF
ASynR
0N3R
0N4R
36
0.4
0
3.6
0
0
1.8
36.9
1.3
0
0
0
0
0.1
39.9
0
0
0
2.8
0
0
37.2
0
0
0.6
0
0
0
22.9
16.5
0
0.3
0
0
10.2
29.5
AB40
AB42
ASynF
ASynR
0N3R
0N4R
35
30
25
20
15
10
5
0

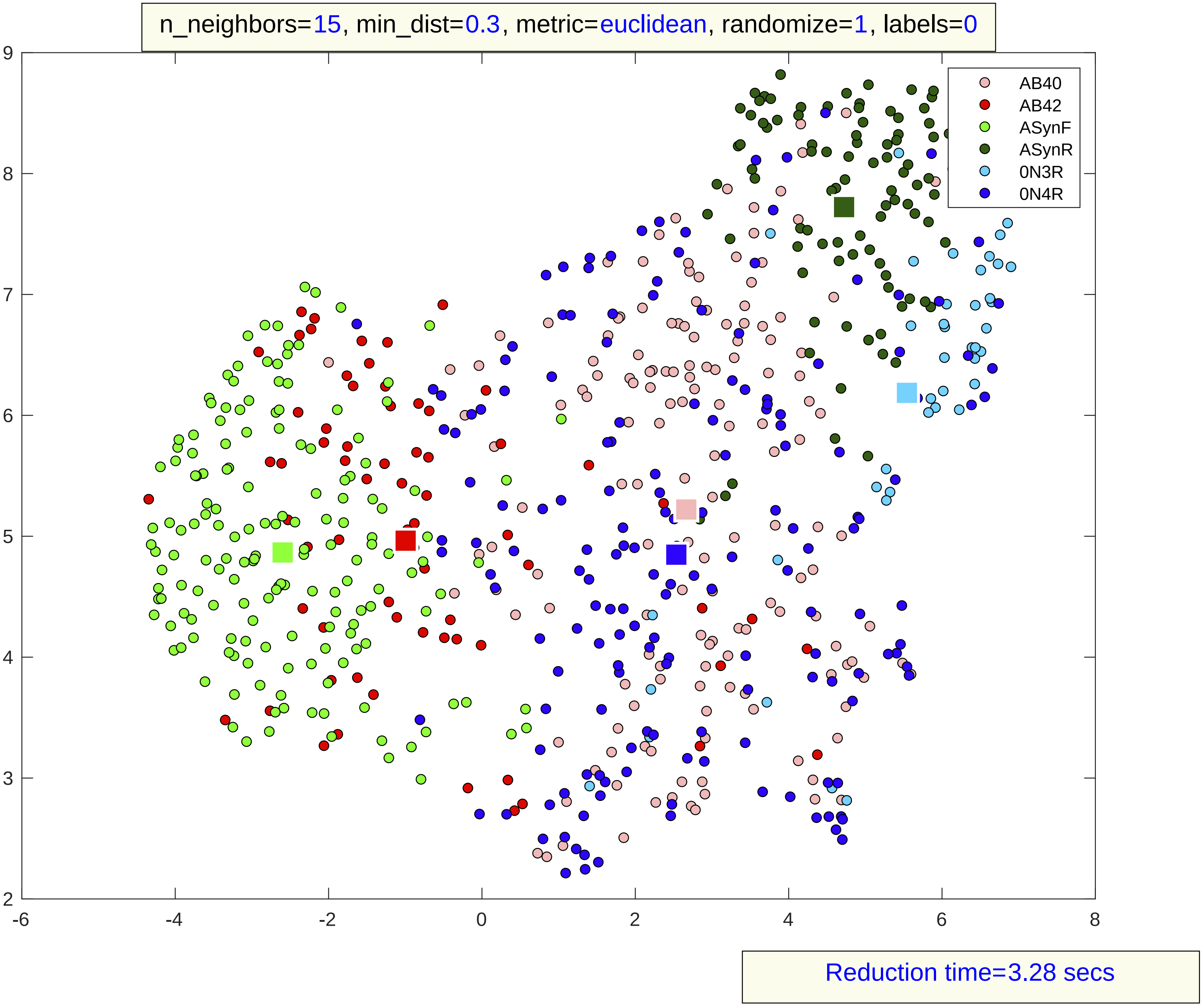
n_neighbors=15, min_dist=0.3, metric=euclidean, randomize=1, labels=0
AB40
AB42
ASynF
ASynR
0N3R
0N4R
9
8
7
6
5
4
3
2
-6
-4
-2
0
2
4
6
8
Reduction time=3.28 secs

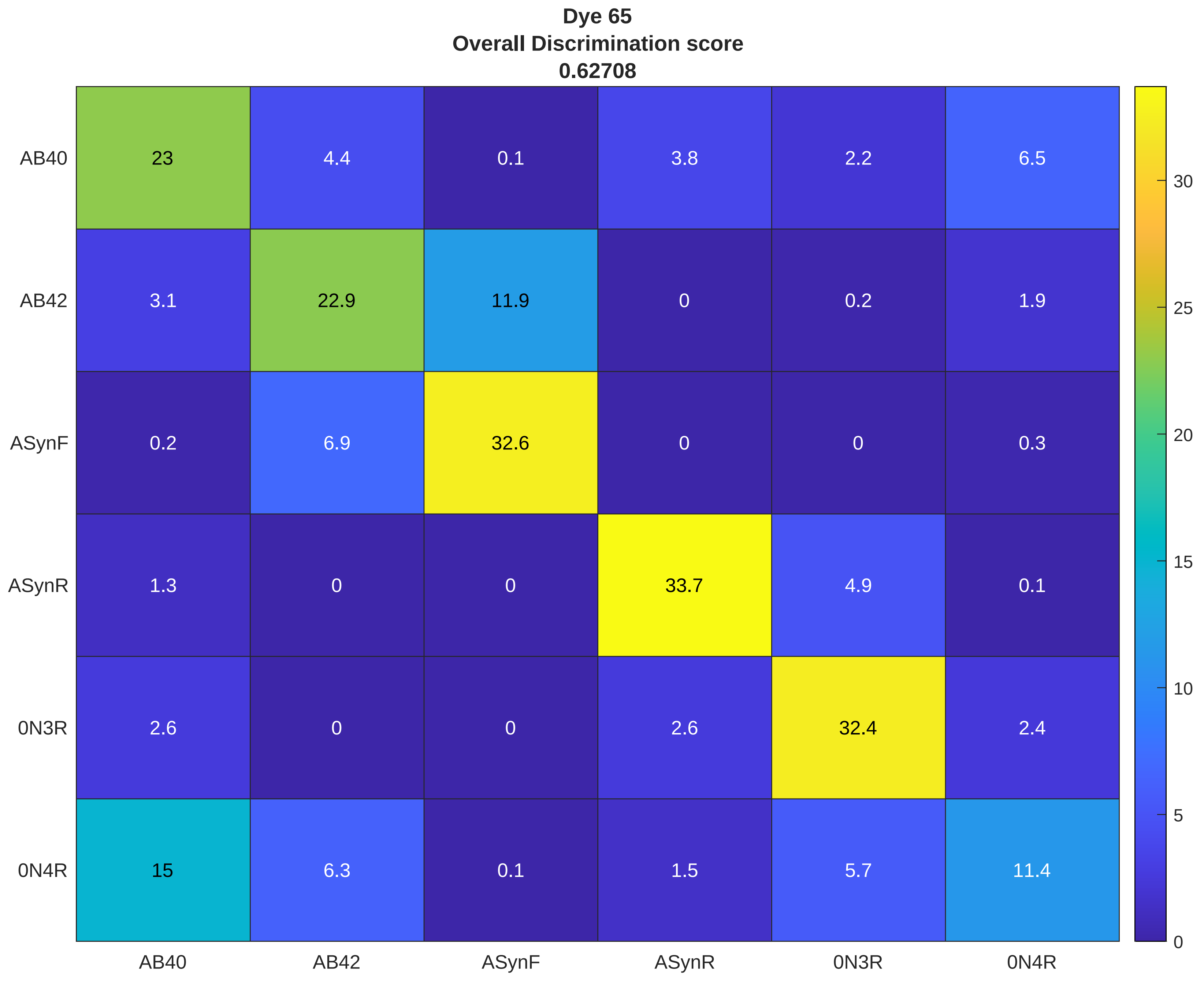
Dye 65
Overall Discrimination score
0.62708
AB40
AB42
ASynF
ASynR
0N3R
0N4R
23
4.4
0.1
3.8
2.2
6.5
3.1
22.9
11.9
0
0.2
1.9
0.2
6.9
32.6
0
0
0.3
1.3
0
0
33.7
4.9
0.1
2.6
0
0
2.6
32.4
2.4
15
6.3
0.1
1.5
5.7
11.4
AB40
AB42
ASynF
ASynR
0N3R
0N4R
30
25
20
15
10
5
0

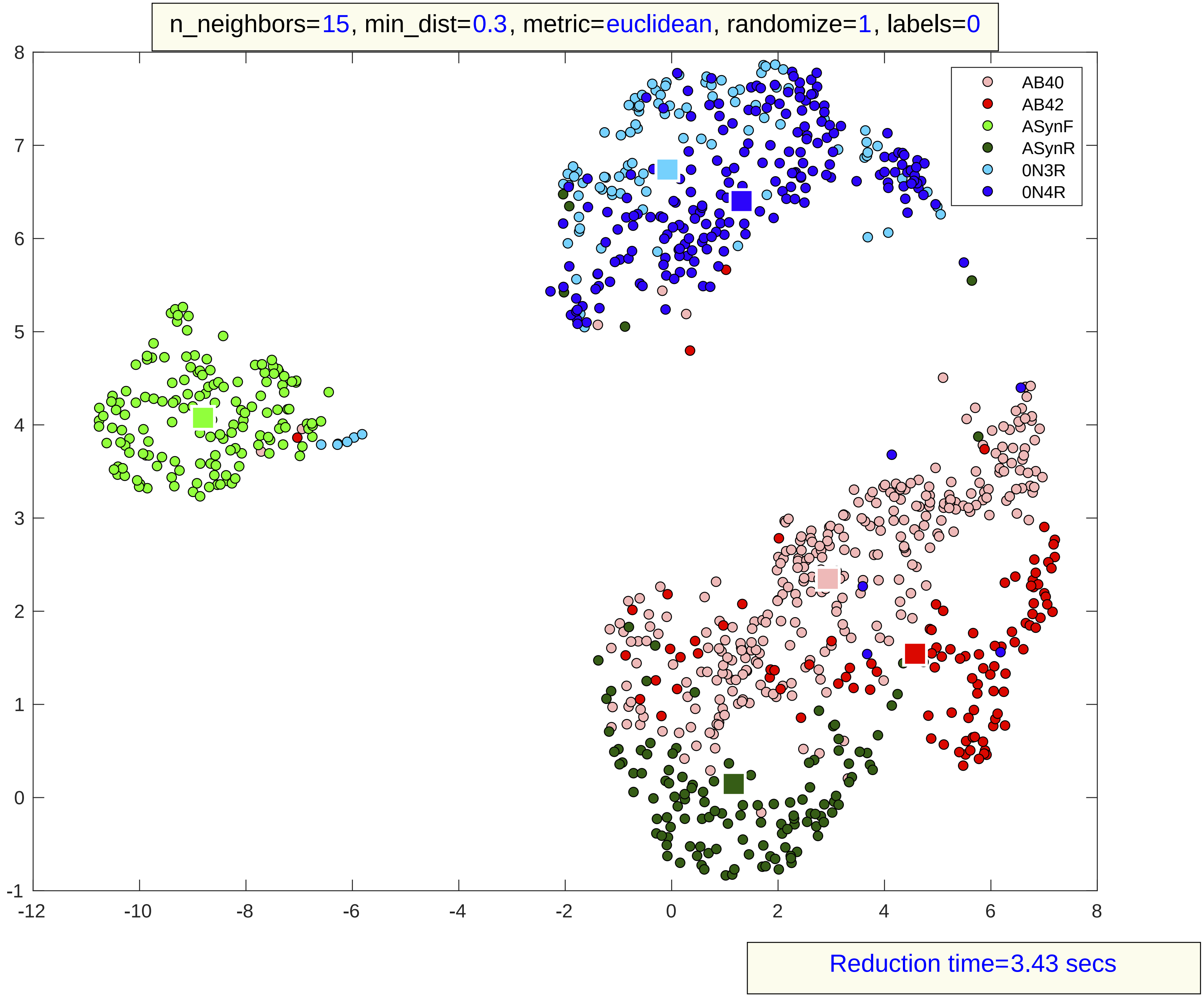
n_neighbors=15, min_dist=0.3, metric=euclidean, randomize=1, labels=0
AB40
AB42
ASynF
ASynR
0N3R
0N4R
8
7
6
5
4
3
2
1
0
-1
-12
-10
-8
-6
-4
-2
0
2
4
6
8
Reduction time=3.43 secs

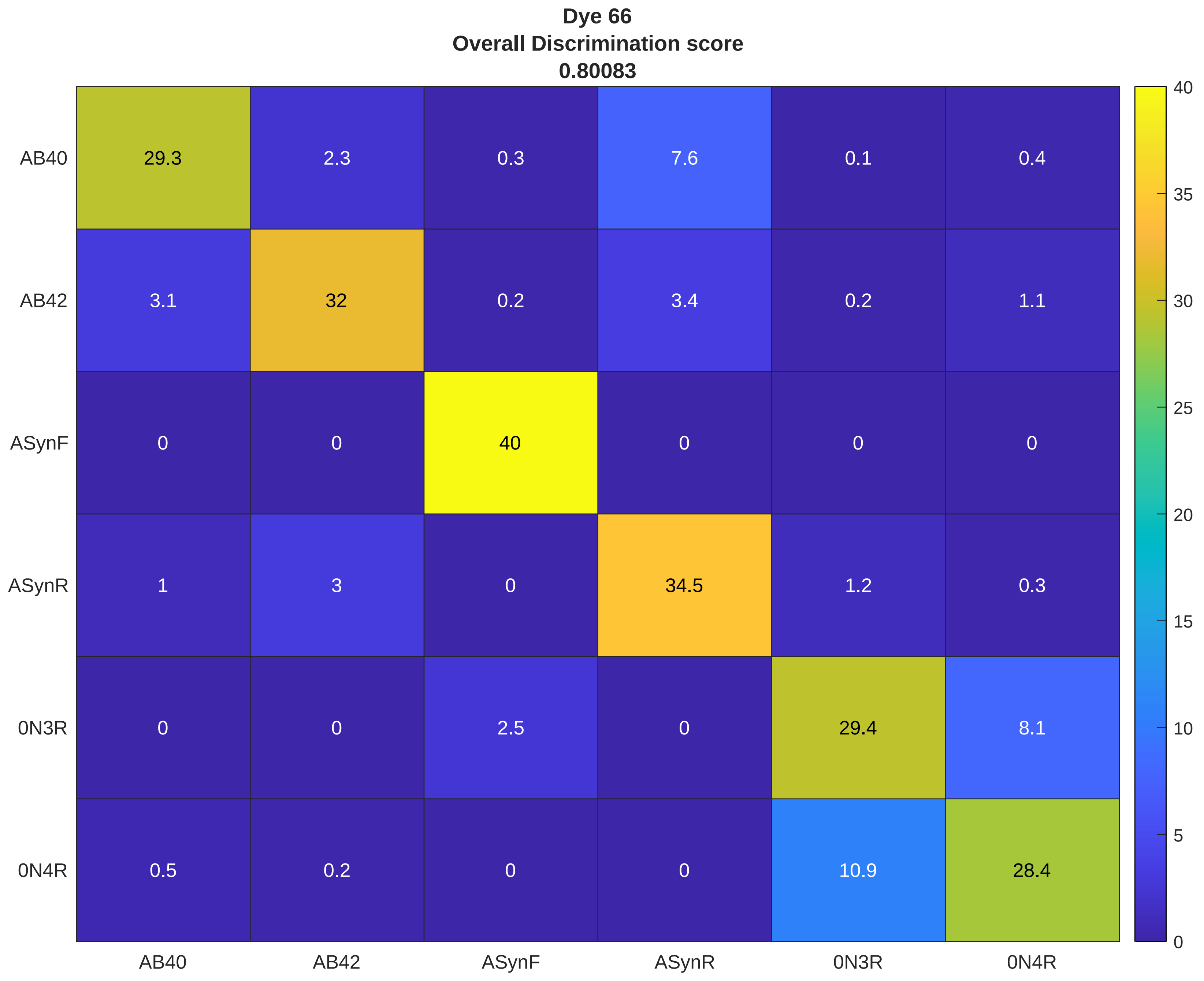
Dye 66
Overall Discrimination score
0.80083
AB40
AB42
ASynF
ASynR
0N3R
0N4R
29.3
2.3
0.3
7.6
0.1
0.4
3.1
32
0.2
3.4
0.2
1.1
0
0
40
0
0
0
1
3
0
34.5
1.2
0.3
0
0
2.5
0
29.4
8.1
0.5
0.2
0
0
10.9
28.4
AB40
AB42
ASynF
ASynR
0N3R
0N4R
40
35
30
25
20
15
10
5
0

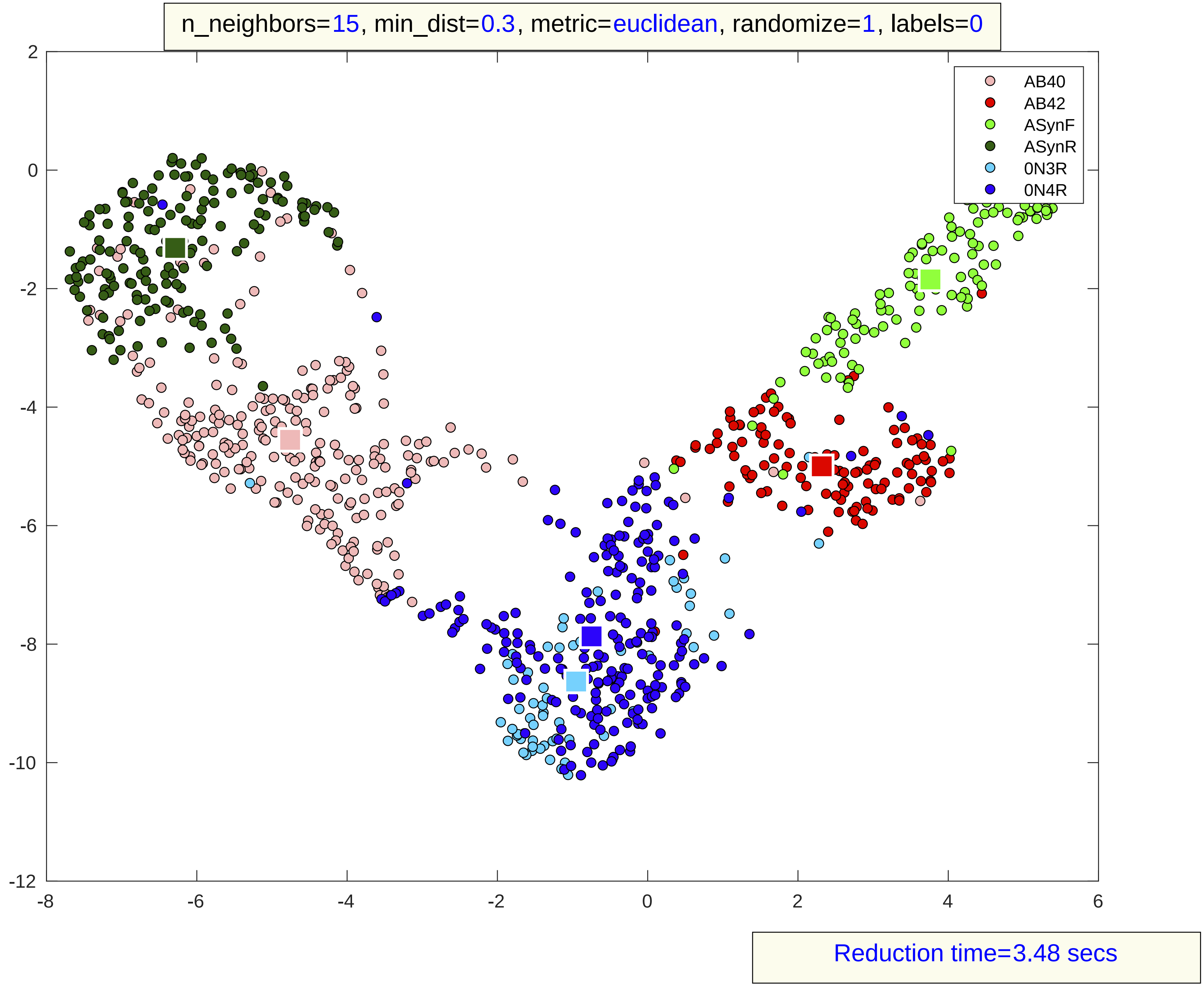

n_neighbors=15, min_dist=0.3, metric=euclidean, randomize=1, labels=0
AB40
AB42
ASynF
ASynR
0N3R
0N4R
2
0
-2
-4
-6
-8
-10
-12
-8
-6
-4
-2
0
2
4
6
Reduction time=3.48 secs

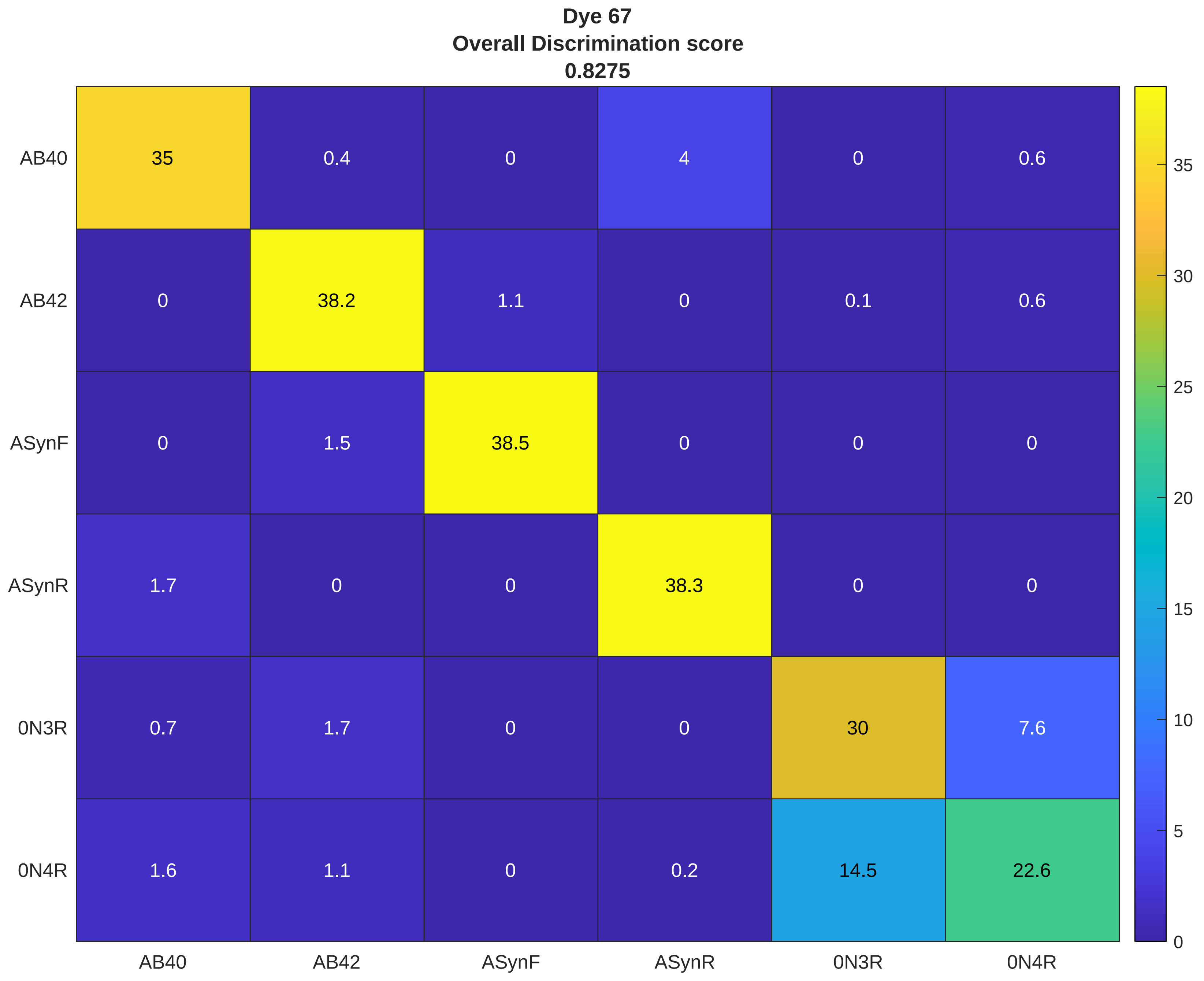
Dye 67
Overall Discrimination score
0.8275
AB40
AB42
ASynF
ASynR
0N3R
0N4R
35
0.4
0
4
0
0.6
0
38.2
1.1
0
0.1
0.6
0
1.5
38.5
0
0
0
1.7
0
0
38.3
0
0
0.7
1.7
0
0
30
7.6
1.6
1.1
0
0.2
14.5
22.6
AB40
AB42
ASynF
ASynR
0N3R
0N4R
35
30
25
20
15
10
5
0

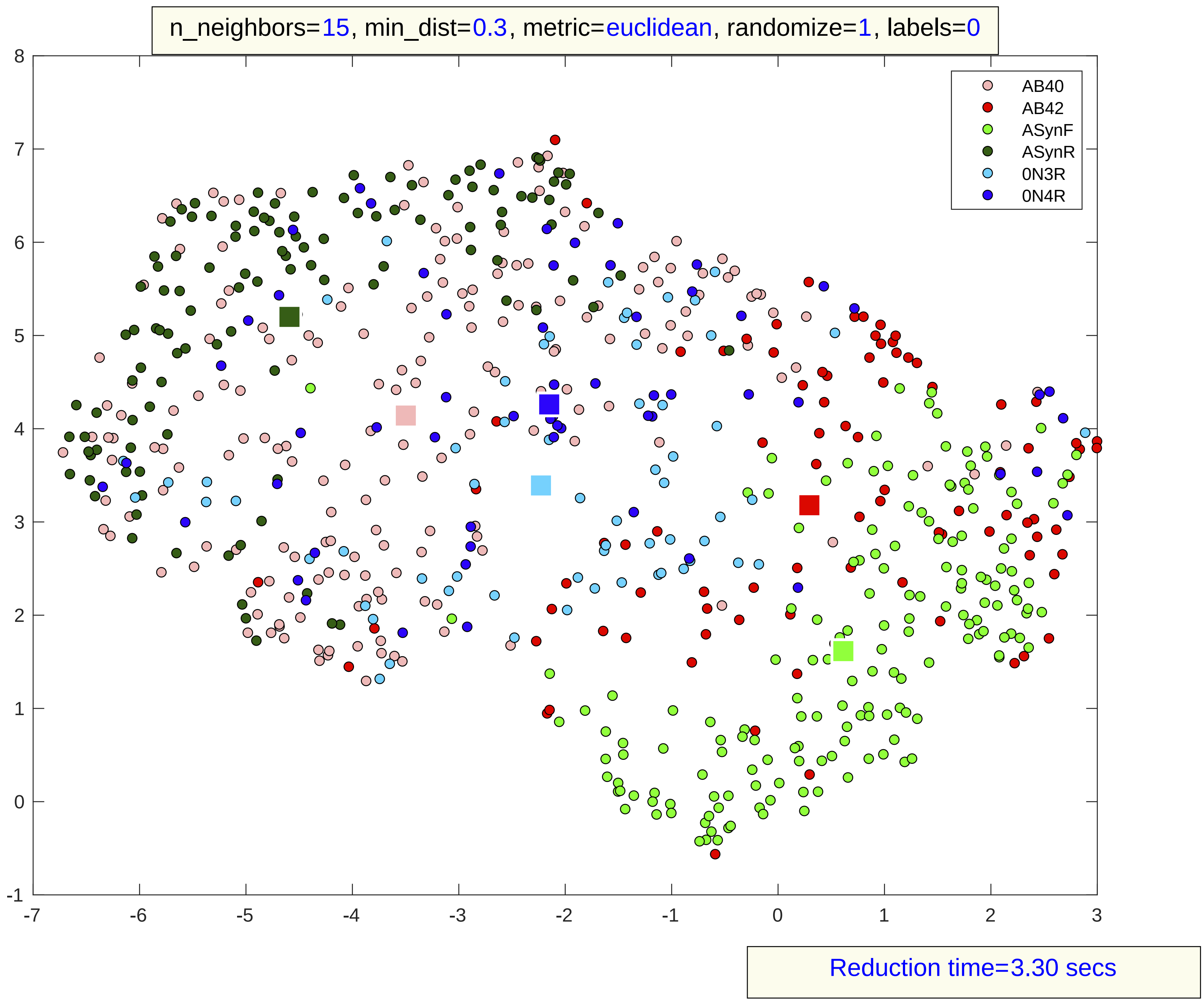

n_neighbors=15, min_dist=0.3, metric=euclidean, randomize=1, labels=0
8
7
6
5
4
3
2
1
0
-1
-7
-6
-5
-4
-3
-2
-1
0
1
2
3
AB40
AB42
ASynF
ASynR
0N3R
0N4R
Reduction time=3.30 secs

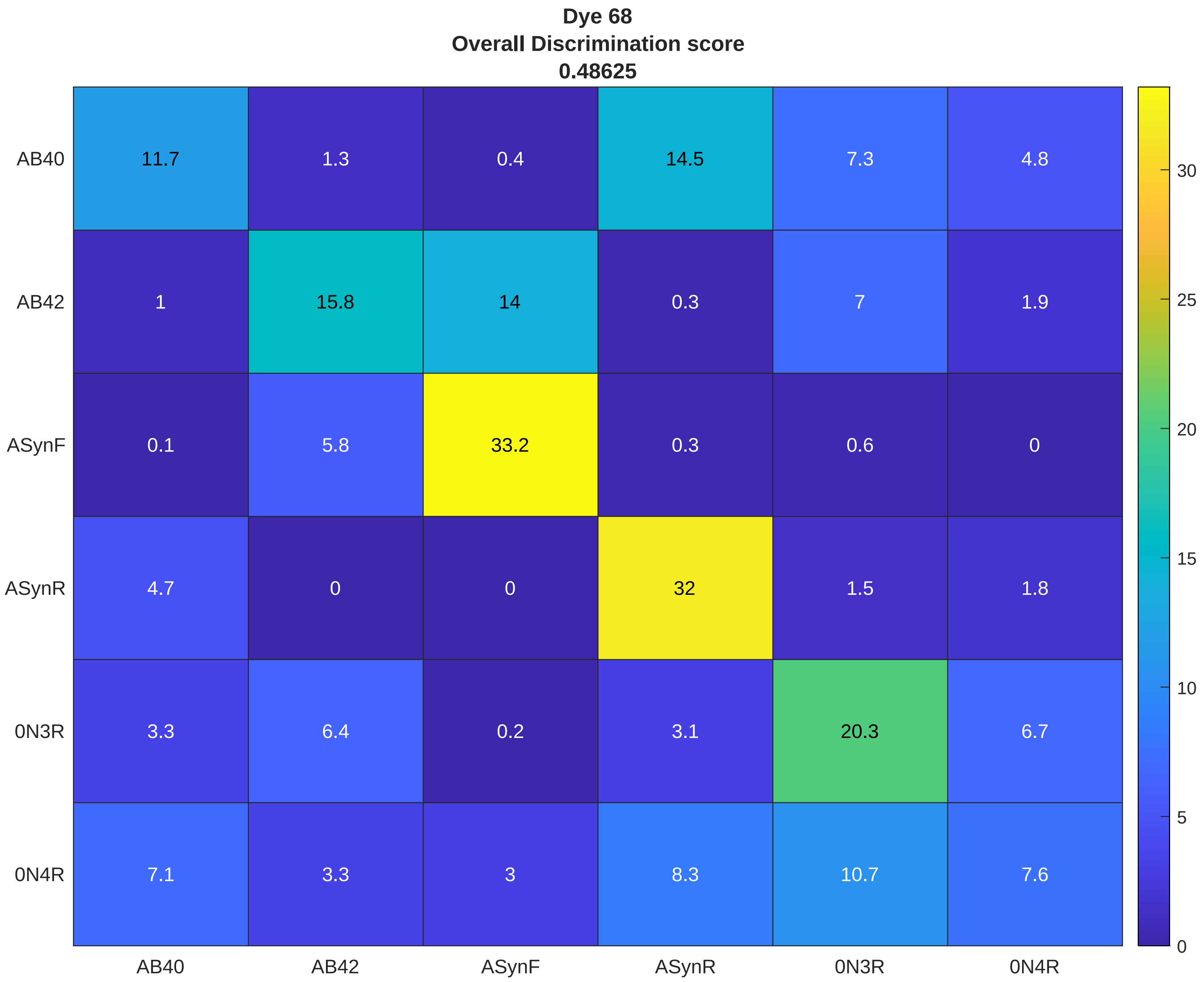

Dye 68
Overall Discrimination score
0.48625
AB40
AB42
ASynF
ASynR
0N3R
0N4R
11.7
1.3
0.4
14.5
7.3
4.8
1
15.8
14
0.3
7
1.9
0.1
5.8
33.2
0.3
0.6
0
4.7
0
0
32
1.5
1.8
3.3
6.4
0.2
3.1
20.3
6.7
7.1
3.3
3
8.3
10.7
7.6
AB40
AB42
ASynF
ASynR
0N3R
0N4R
30
25
20
15
10
5
0

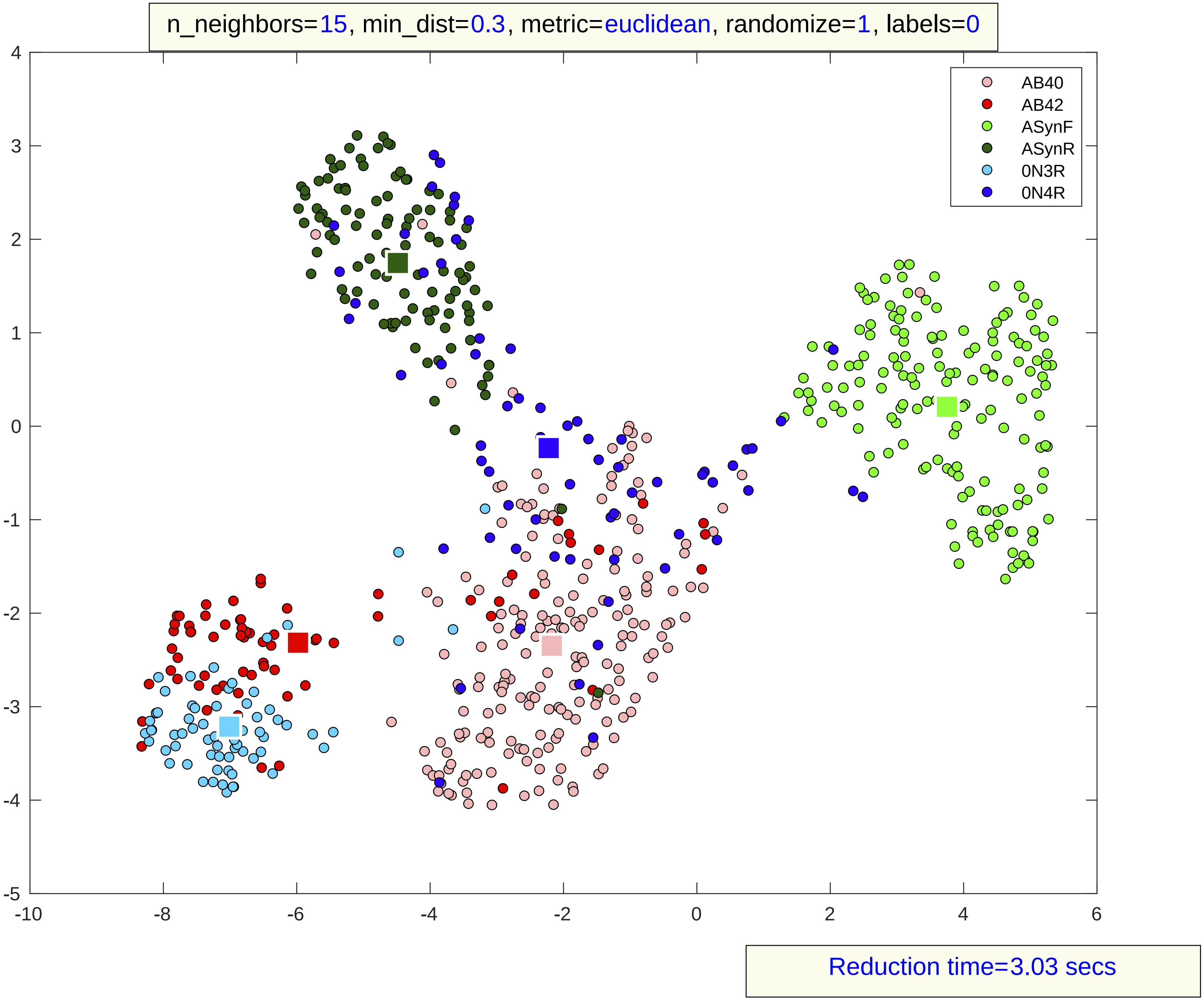

n_neighbors=15, min_dist=0.3, metric=euclidean, randomize=1, labels=0
AB40
AB42
ASynF
ASynR
0N3R
0N4R
4
3
2
1
0
-1
-2
-3
-4
-5
-10
-8
-6
-4
-2
0
2
4
6
Reduction time=3.03 secs

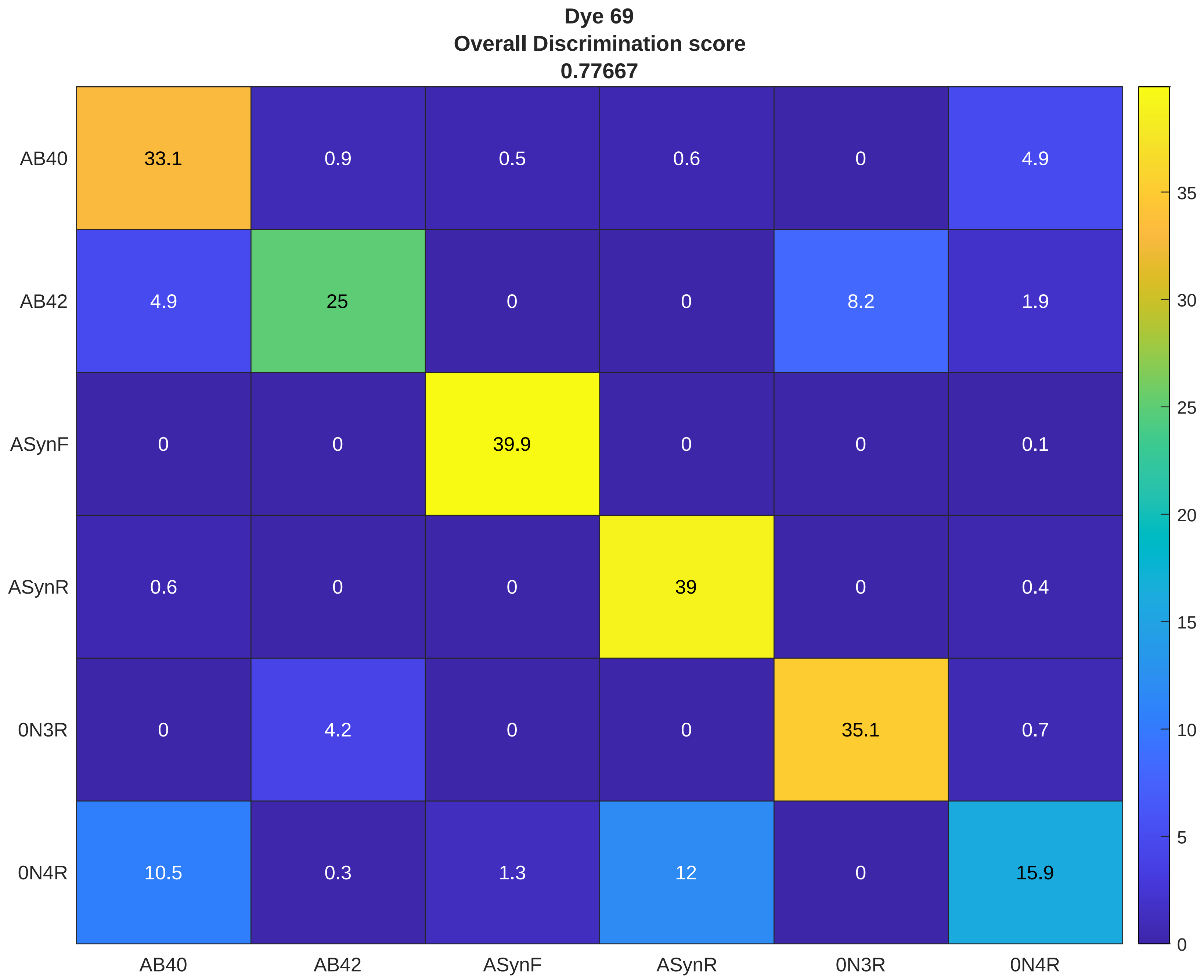
Dye 69
Overall Discrimination score
0.77667
AB40
AB42
ASynF
ASynR
0N3R
0N4R
33.1
0.9
0.5
0.6
0
4.9
4.9
25
0
0
8.2
1.9
0
0
39.9
0
0
0.1
0.6
0
0
39
0
0.4
0
4.2
0
0
35.1
0.7
10.5
0.3
1.3
12
0
15.9
AB40
AB42
ASynF
ASynR
0N3R
0N4R
35
30
25
20
15
10
5
0

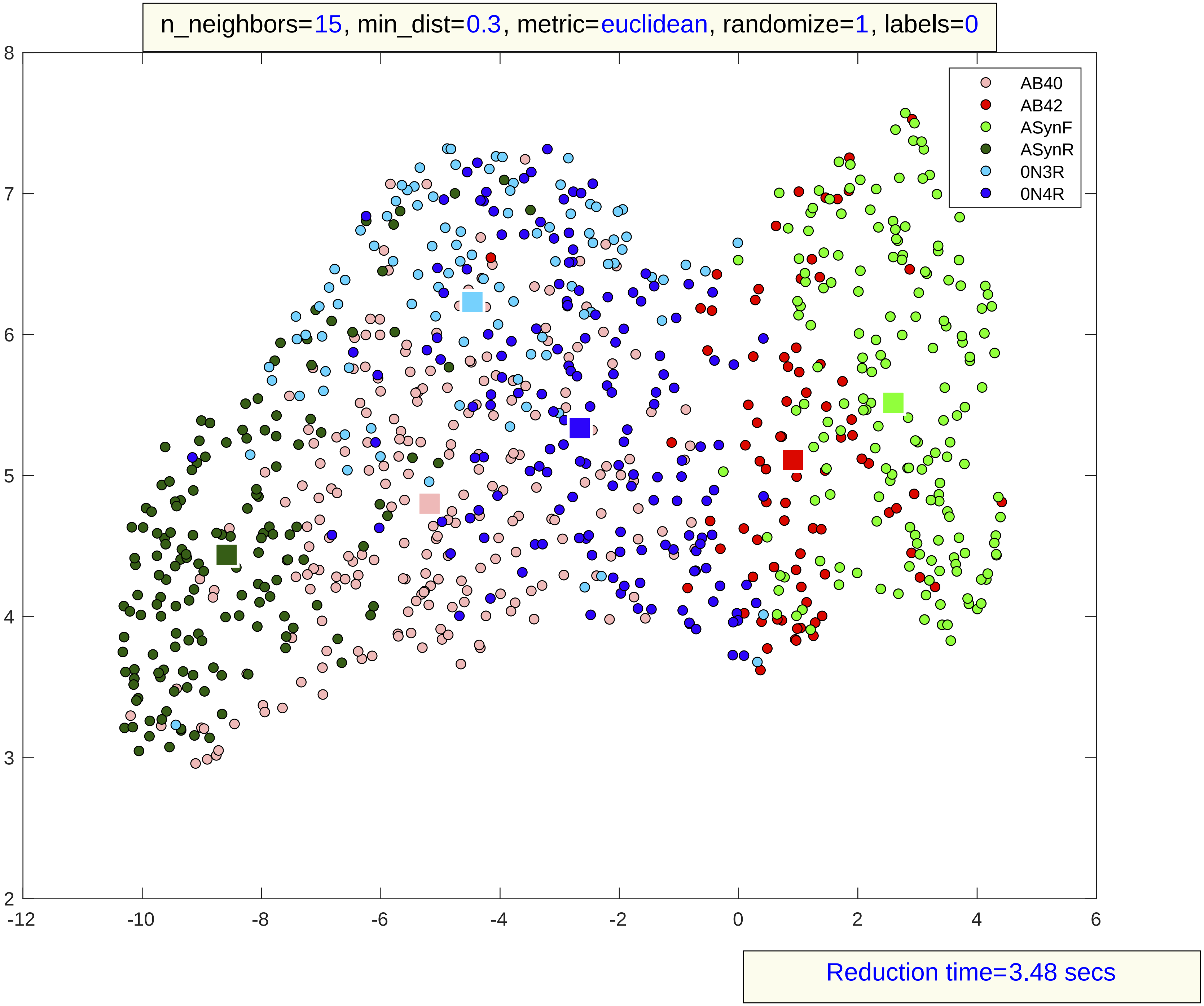

n_neighbors=15, min_dist=0.3, metric=euclidean, randomize=1, labels=0
AB40
AB42
ASynF
ASynR
0N3R
0N4R
8
7
6
5
4
3
2
-12
-10
-8
-6
-4
-2
0
2
4
6
Reduction time=3.48 secs

Dye 70
Overall Discrimination score
0.66

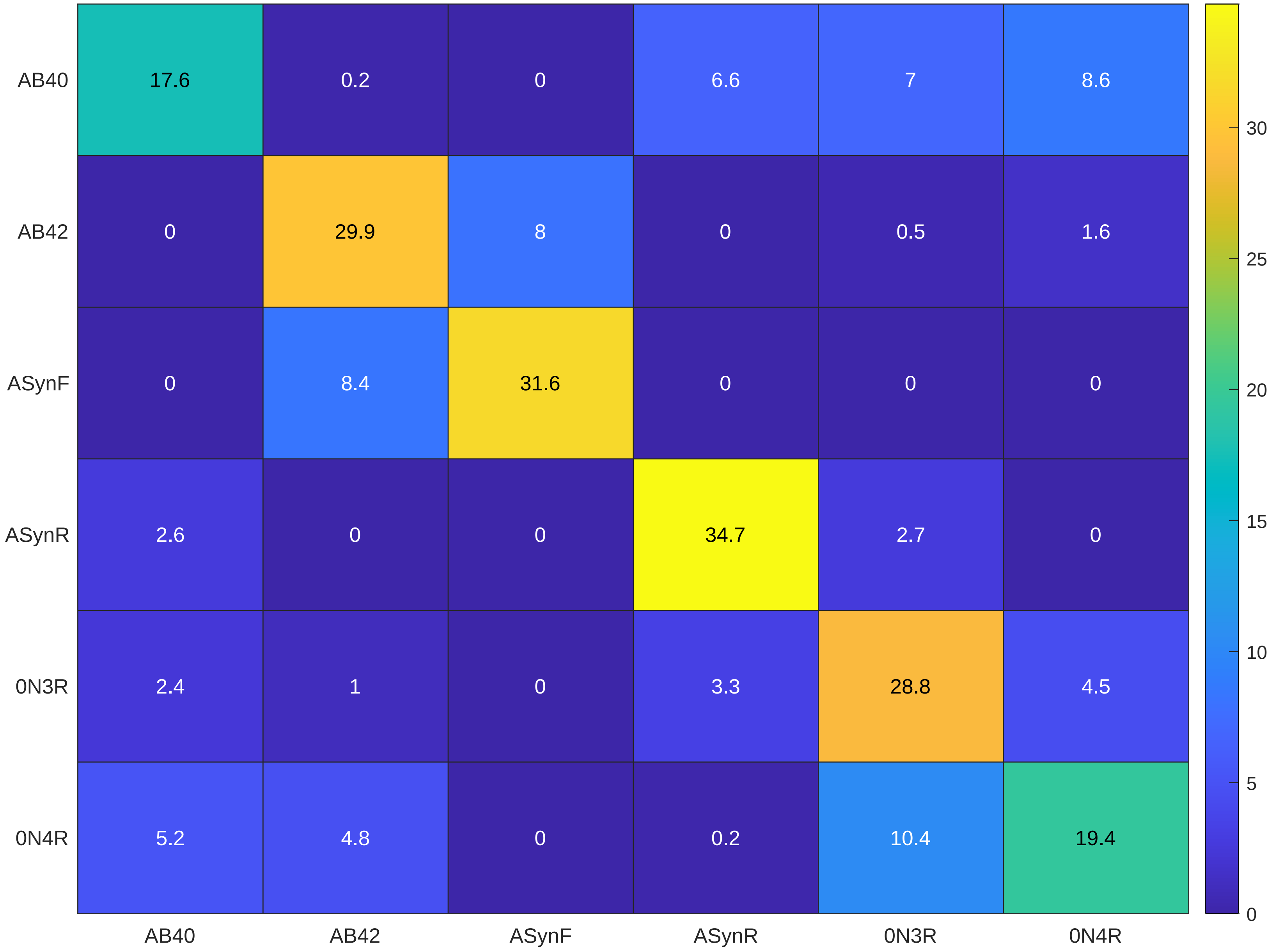

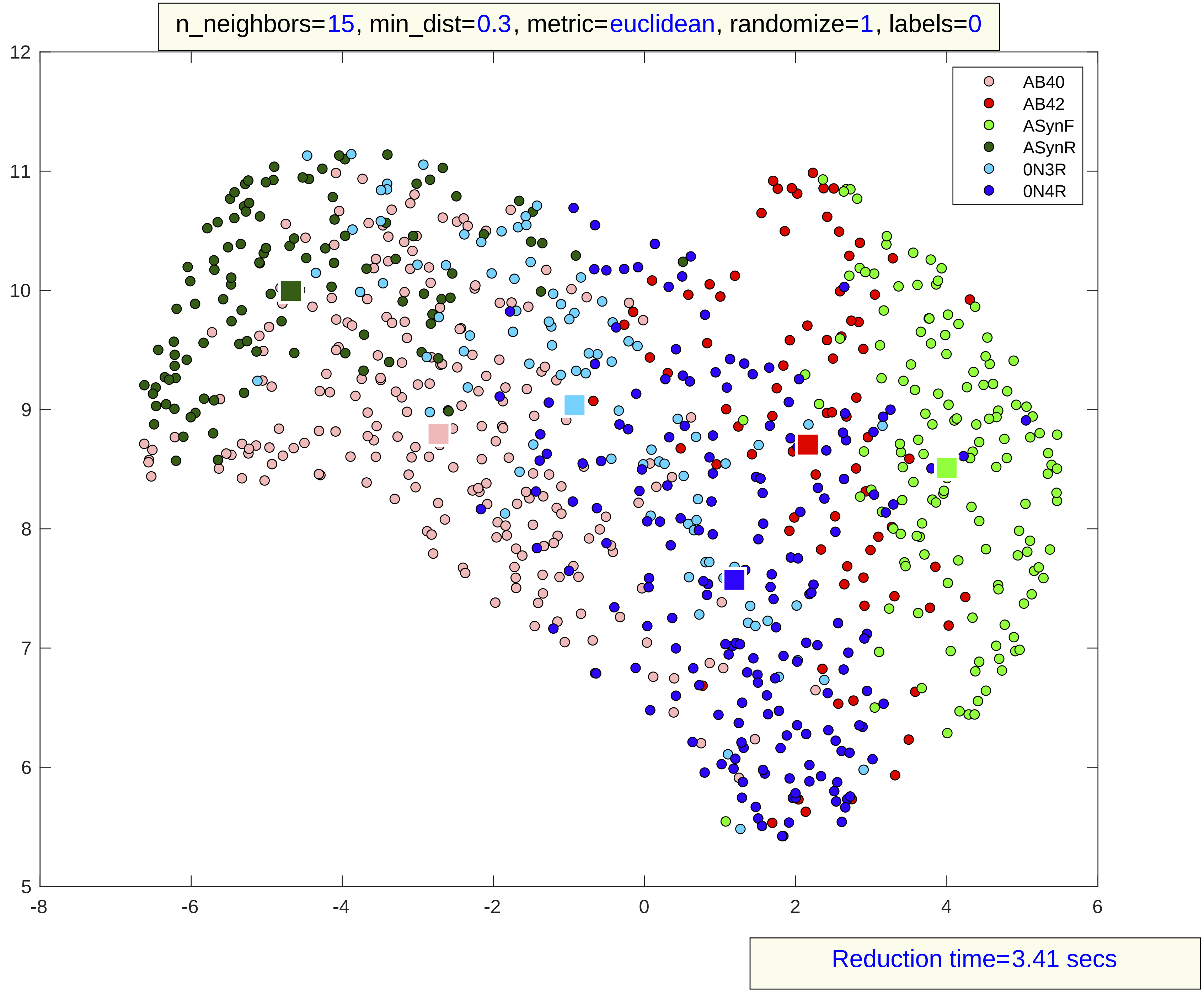
n_neighbors=15, min_dist=0.3, metric=euclidean, randomize=1, labels=0
AB40
AB42
ASynF
ASynR
0N3R
0N4R
12
11
10
9
8
7
6
5
-8
-6
-4
-2
0
2
4
6
Reduction time=3.41 secs

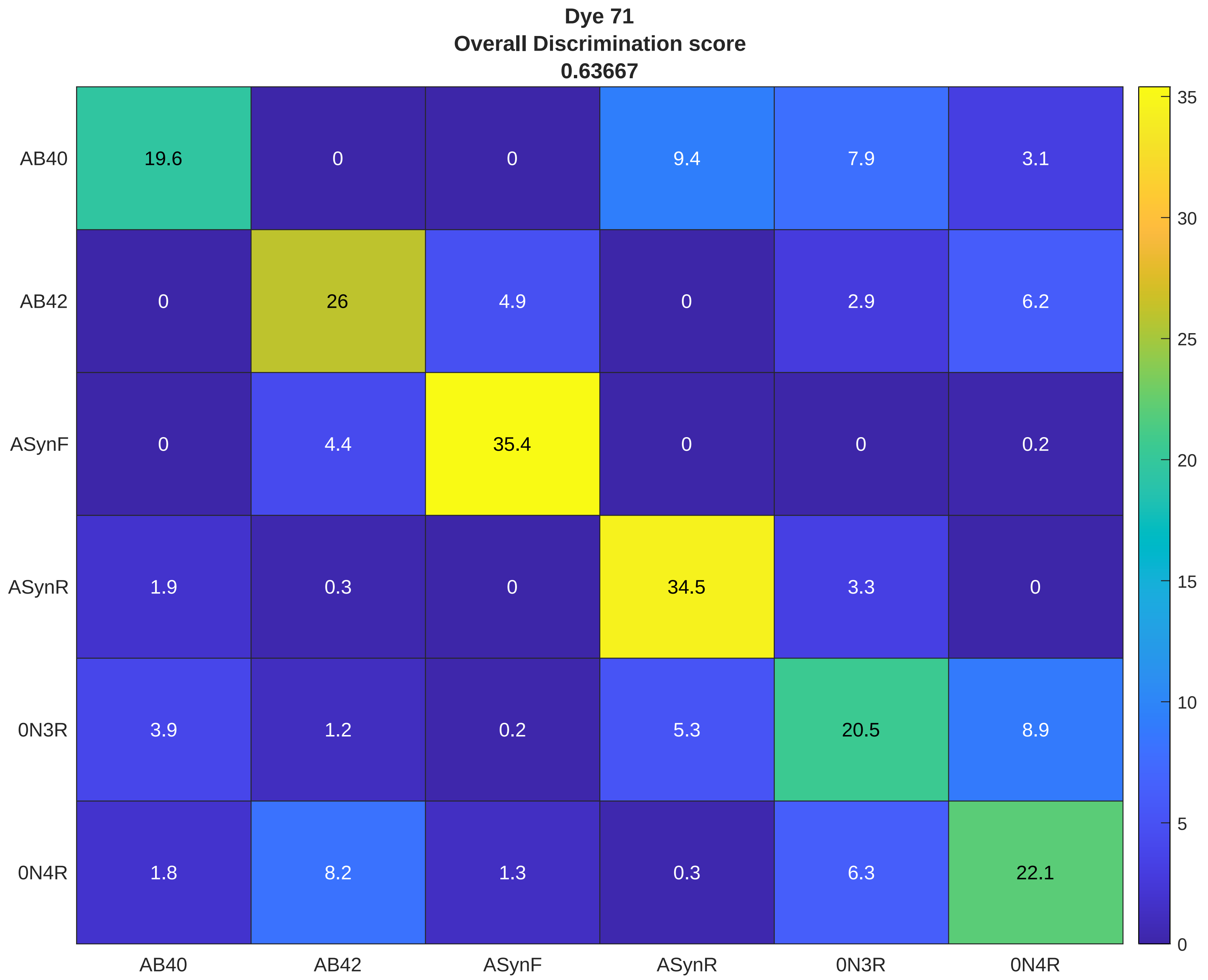
Dye 71
Overall Discrimination score
0.63667
AB40
AB42
ASynF
ASynR
0N3R
0N4R
19.6
0
0
9.4
7.9
3.1
0
26
4.9
0
2.9
6.2
0
4.4
35.4
0
0
0.2
1.9
0.3
0
34.5
3.3
0
3.9
1.2
0.2
5.3
20.5
8.9
1.8
8.2
1.3
0.3
6.3
22.1
AB40
AB42
ASynF
ASynR
0N3R
0N4R
35
30
25
20
15
10
5
0

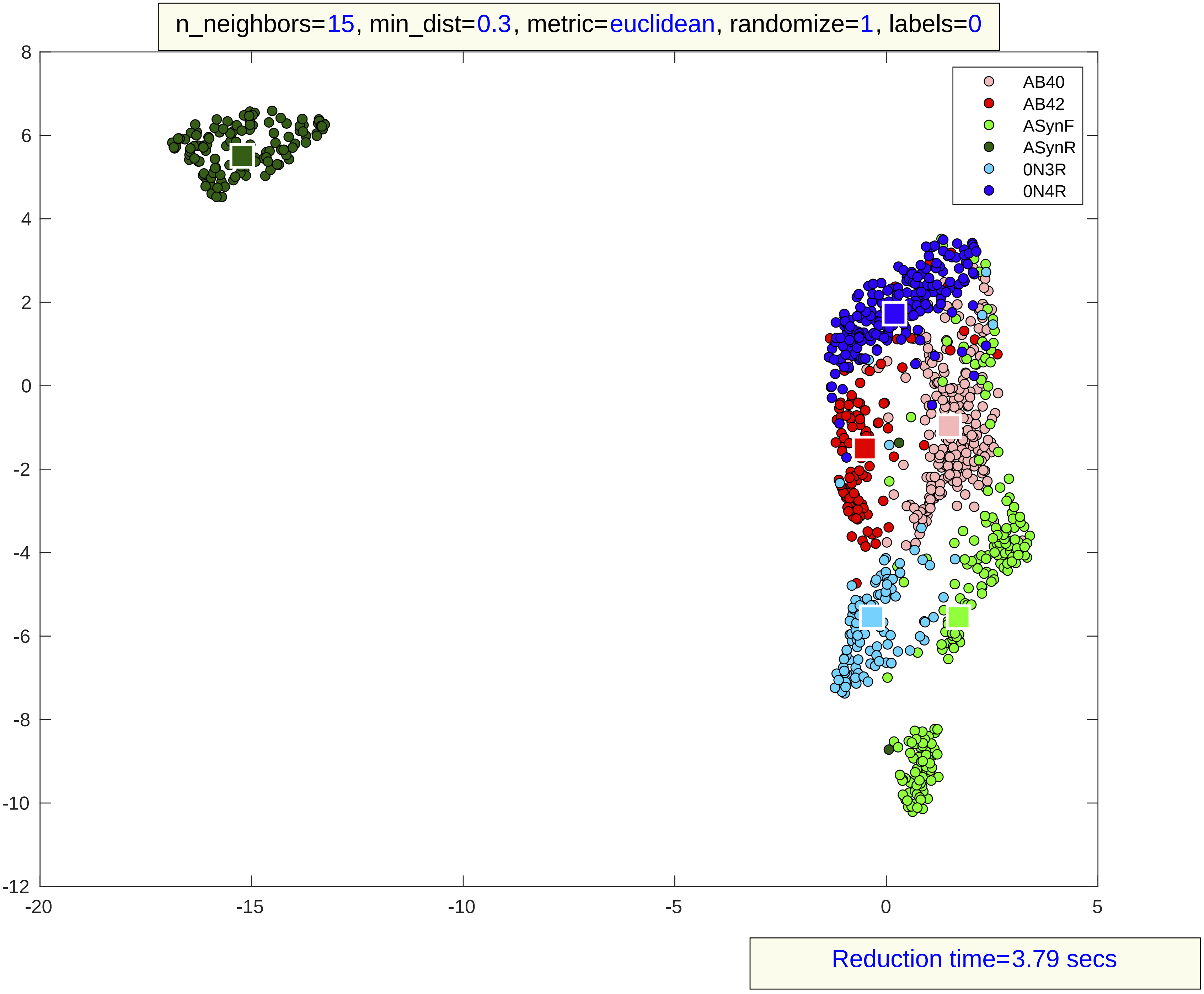

n_neighbors=15, min_dist=0.3, metric=euclidean, randomize=1, labels=0
AB40
AB42
ASynF
ASynR
0N3R
0N4R
8
6
4
2
0
-2
-4
-6
-8
-10
-12
-20
-15
-10
-5
0
5
Reduction time=3.79 secs

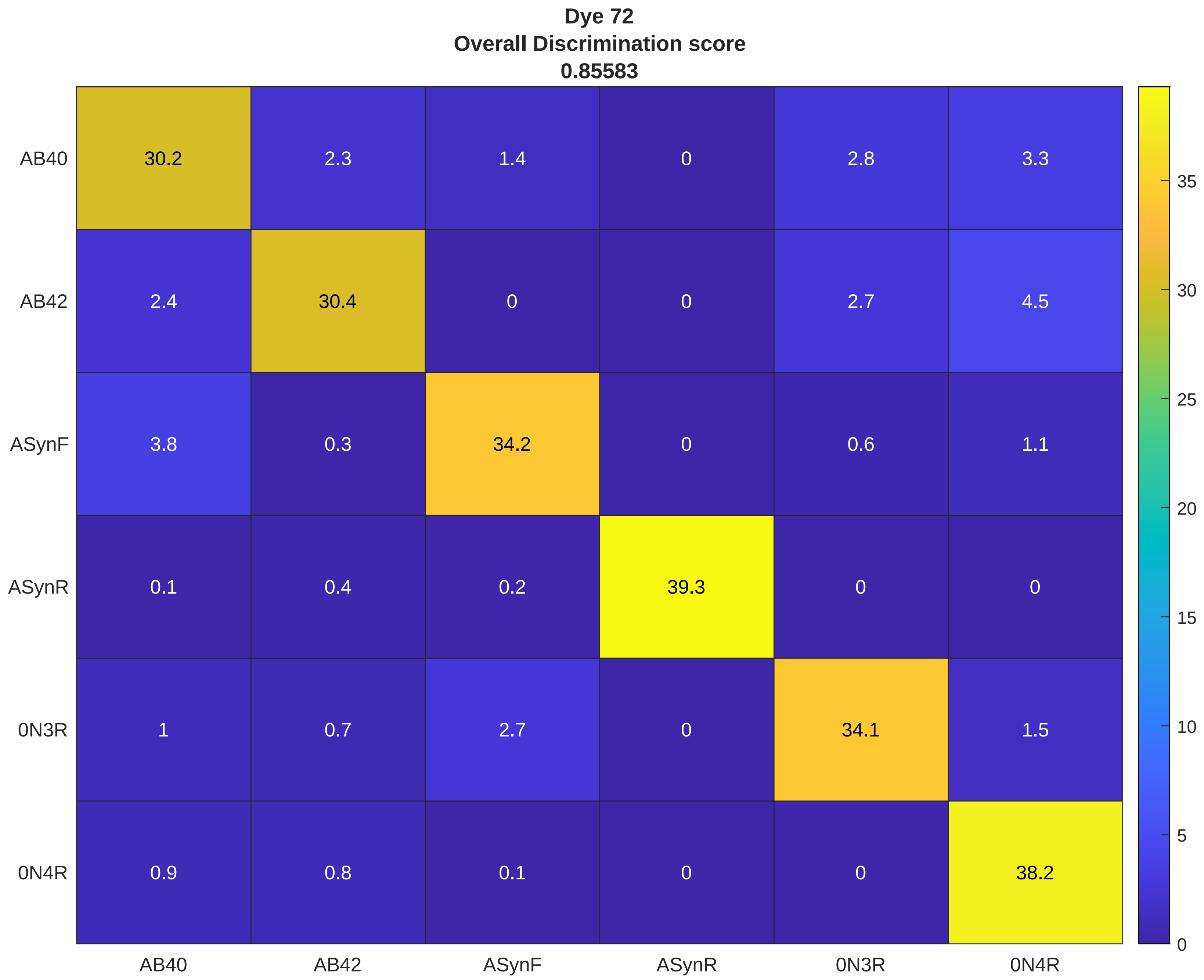
Dye 72
Overall Discrimination score
0.85583
AB40
AB42
ASynF
ASynR
0N3R
0N4R
30.2
2.3
1.4
0
2.8
3.3
2.4
30.4
0
0
2.7
4.5
3.8
0.3
34.2
0
0.6
1.1
0.1
0.4
0.2
39.3
0
0
1
0.7
2.7
0
34.1
1.5
0.9
0.8
0.1
0
0
38.2
AB40
AB42
ASynF
ASynR
0N3R
0N4R
35
30
25
20
15
10
5
0

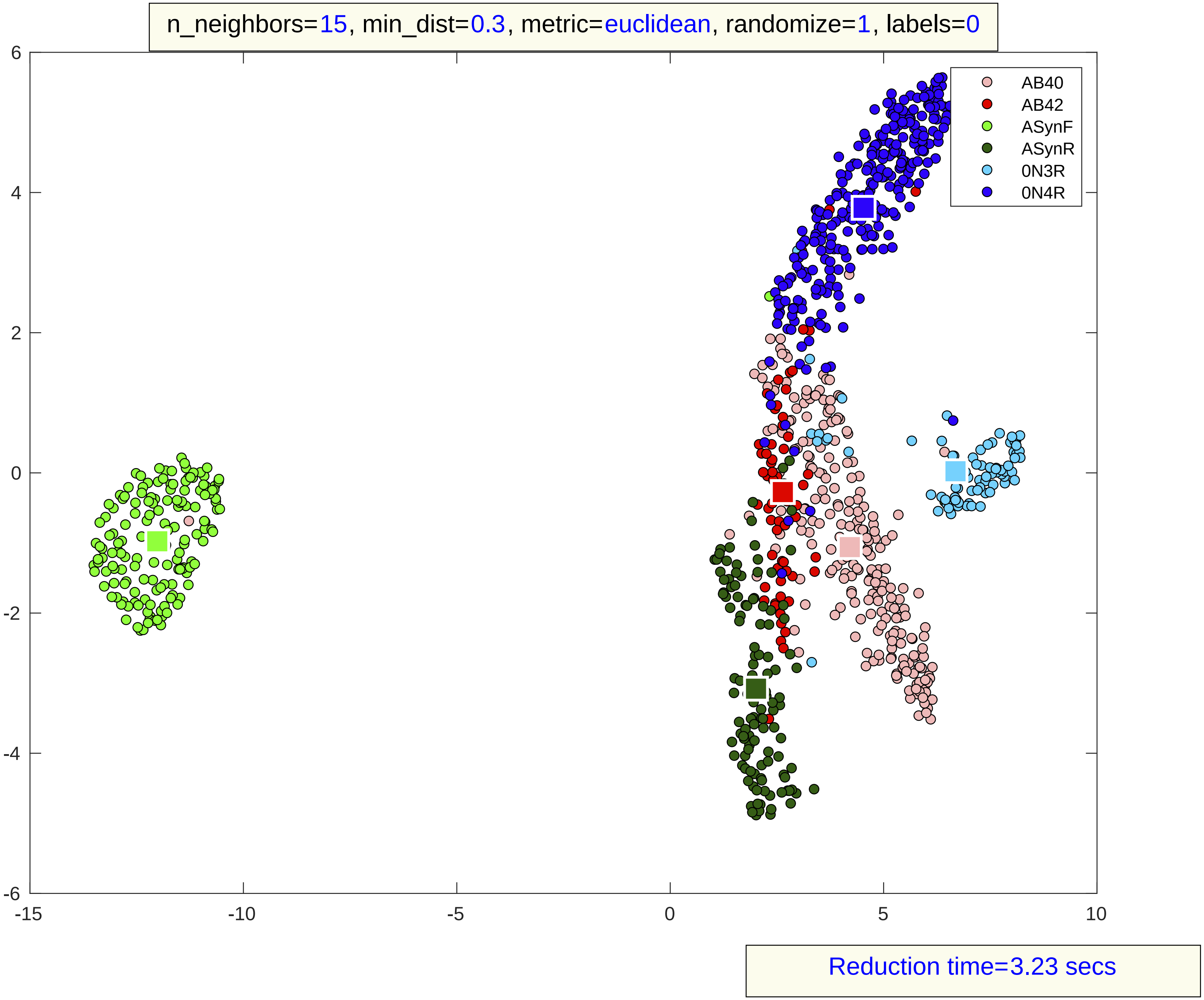
n_neighbors=15, min_dist=0.3, metric=euclidean, randomize=1, labels=0
AB40
AB42
ASynF
ASynR
0N3R
0N4R
6
4
2
0
-2
-4
-6
-15
-10
-5
0
5
10
Reduction time=3.23 secs

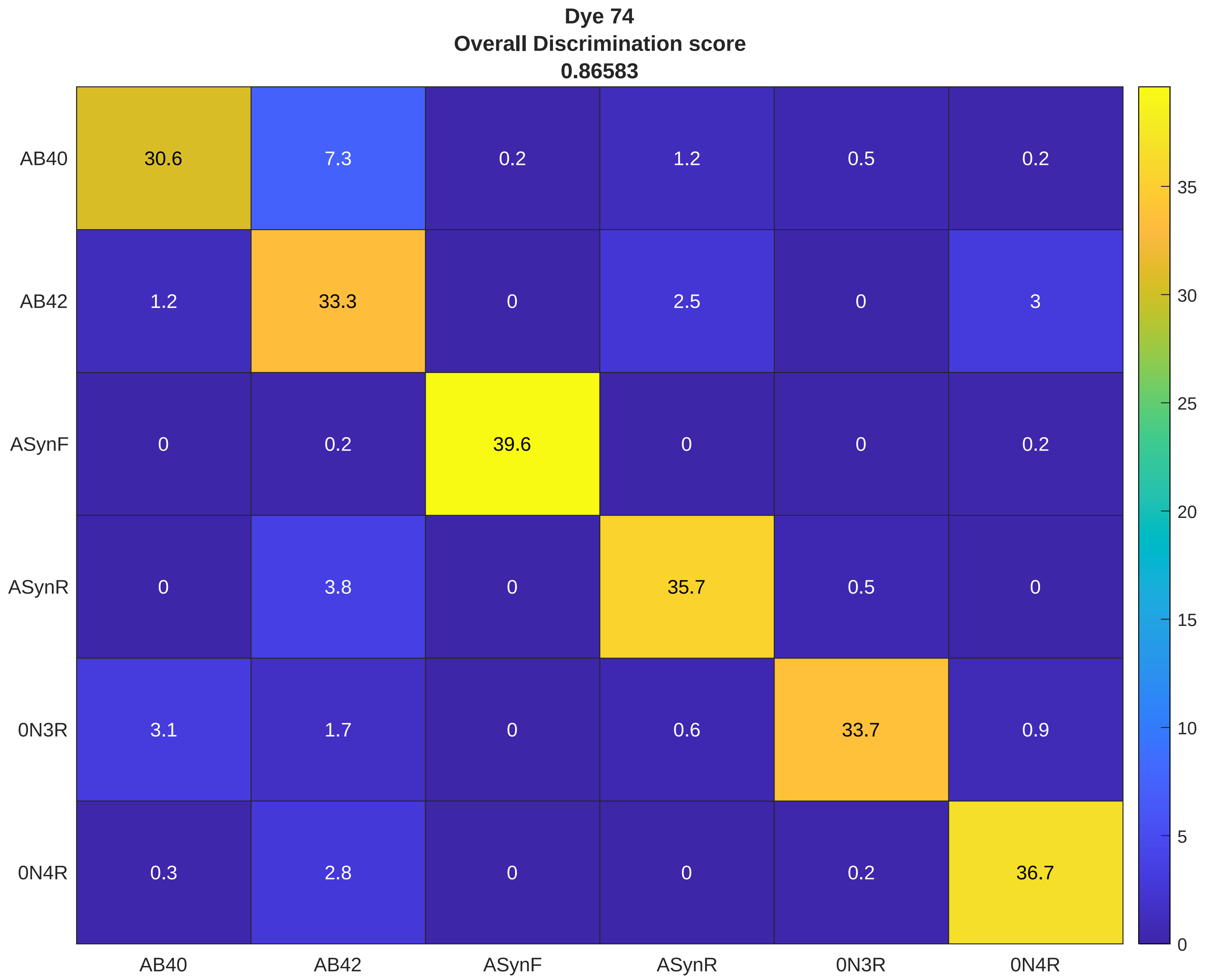
Dye 74
Overall Discrimination score
0.86583
AB40
AB42
ASynF
ASynR
0N3R
0N4R
30.6 7.3 0.2 1.2 0.5 0.2
1.2 33.3 0 2.5 0 3
0 0.2 39.6 0 0 0.2
0 3.8 0 35.7 0.5 0
3.1 1.7 0 0.6 33.7 0.9
0.3 2.8 0 0 0.2 36.7
AB40
AB42
ASynF
ASynR
0N3R
0N4R
35
30
25
20
15
10
5
0

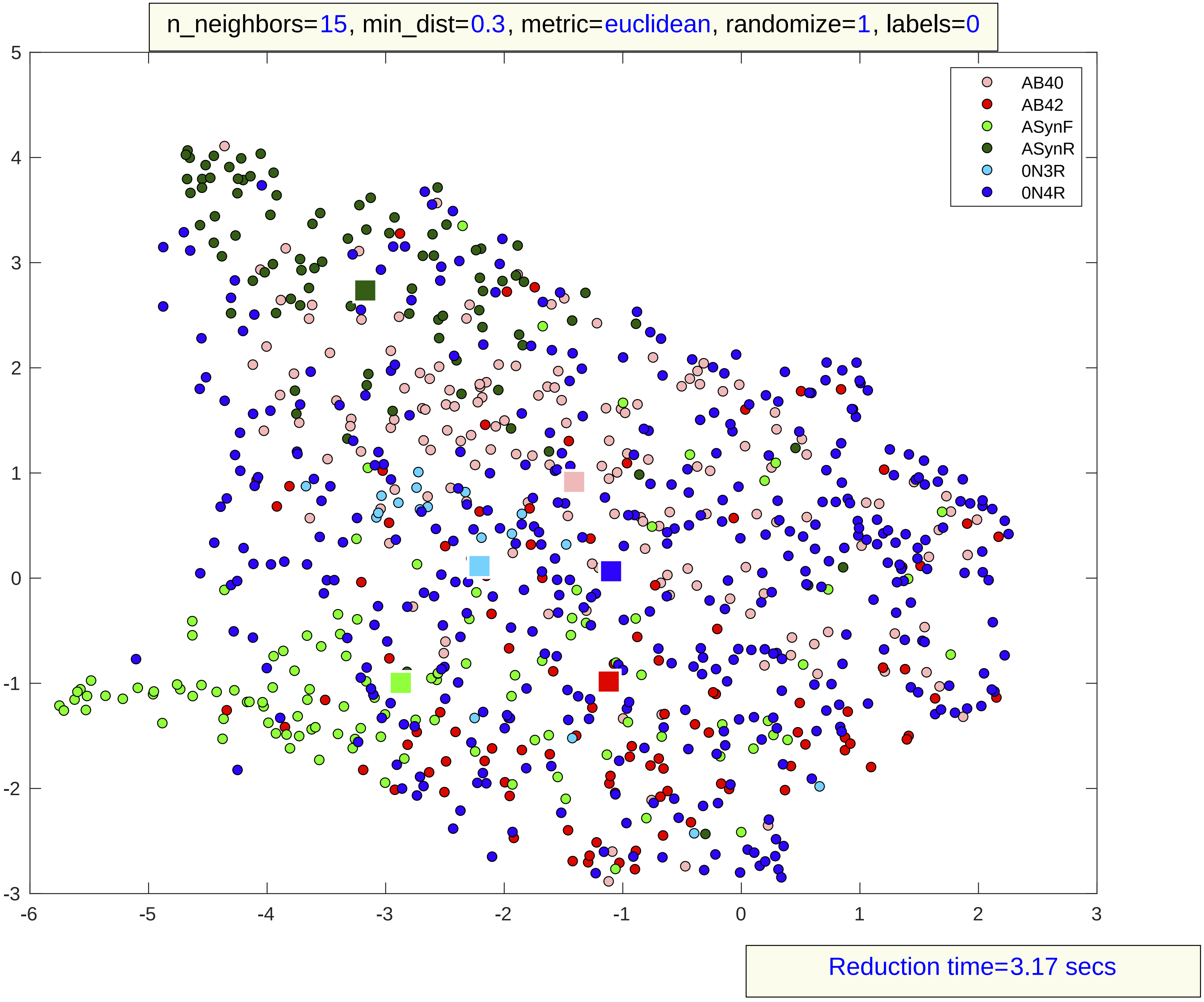

n_neighbors=15, min_dist=0.3, metric=euclidean, randomize=1, labels=0
AB40
AB42
ASynF
ASynR
0N3R
0N4R
5
4
3
2
1
0
-1
-2
-3
-6
-5
-4
-3
-2
-1
0
1
2
3
Reduction time=3.17 secs

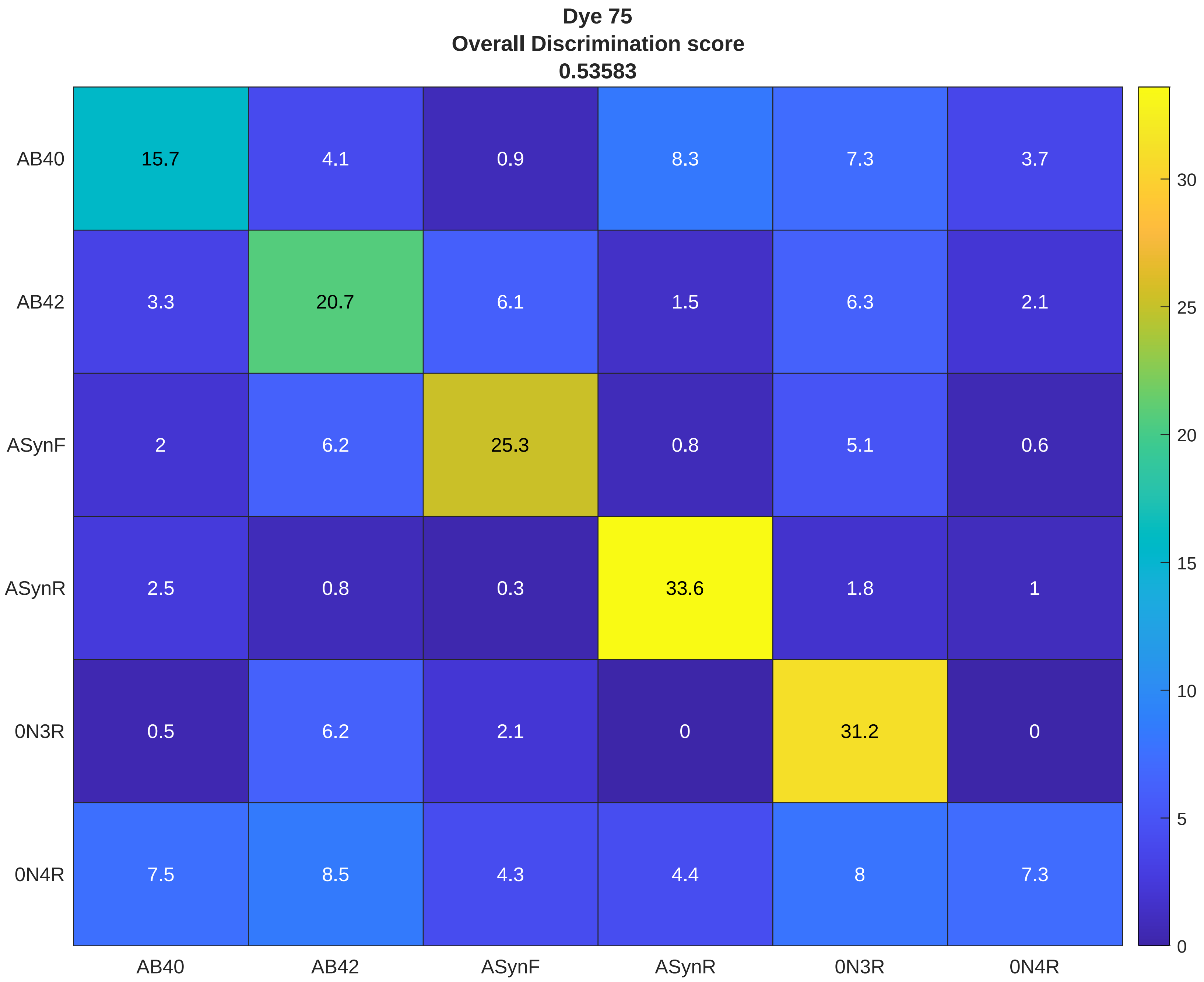
Dye 75
Overall Discrimination score
0.53583
AB40
AB42
ASynF
ASynR
0N3R
0N4R
15.7
4.1
0.9
8.3
7.3
3.7
3.3
20.7
6.1
1.5
6.3
2.1
2
6.2
25.3
0.8
5.1
0.6
2.5
0.8
0.3
33.6
1.8
1
0.5
6.2
2.1
0
31.2
0
7.5
8.5
4.3
4.4
8
7.3
AB40
AB42
ASynF
ASynR
0N3R
0N4R
30
25
20
15
10
5
0

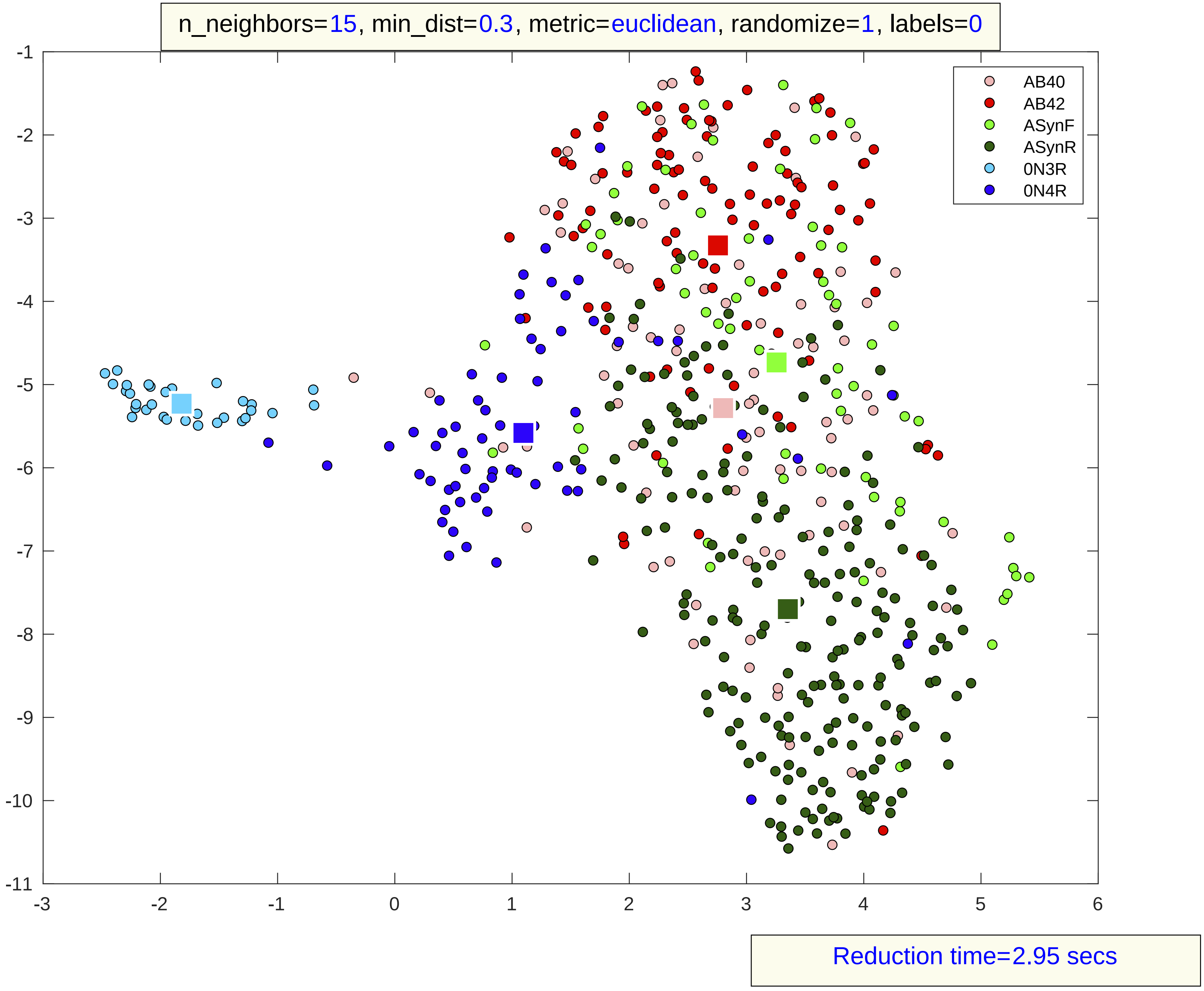

n_neighbors=15, min_dist=0.3, metric=euclidean, randomize=1, labels=0
AB40
AB42
ASynF
ASynR
0N3R
0N4R
-1
-2
-3
-4
-5
-6
-7
-8
-9
-10
-11
-3
-2
-1
0
1
2
3
4
5
6
Reduction time=2.95 secs

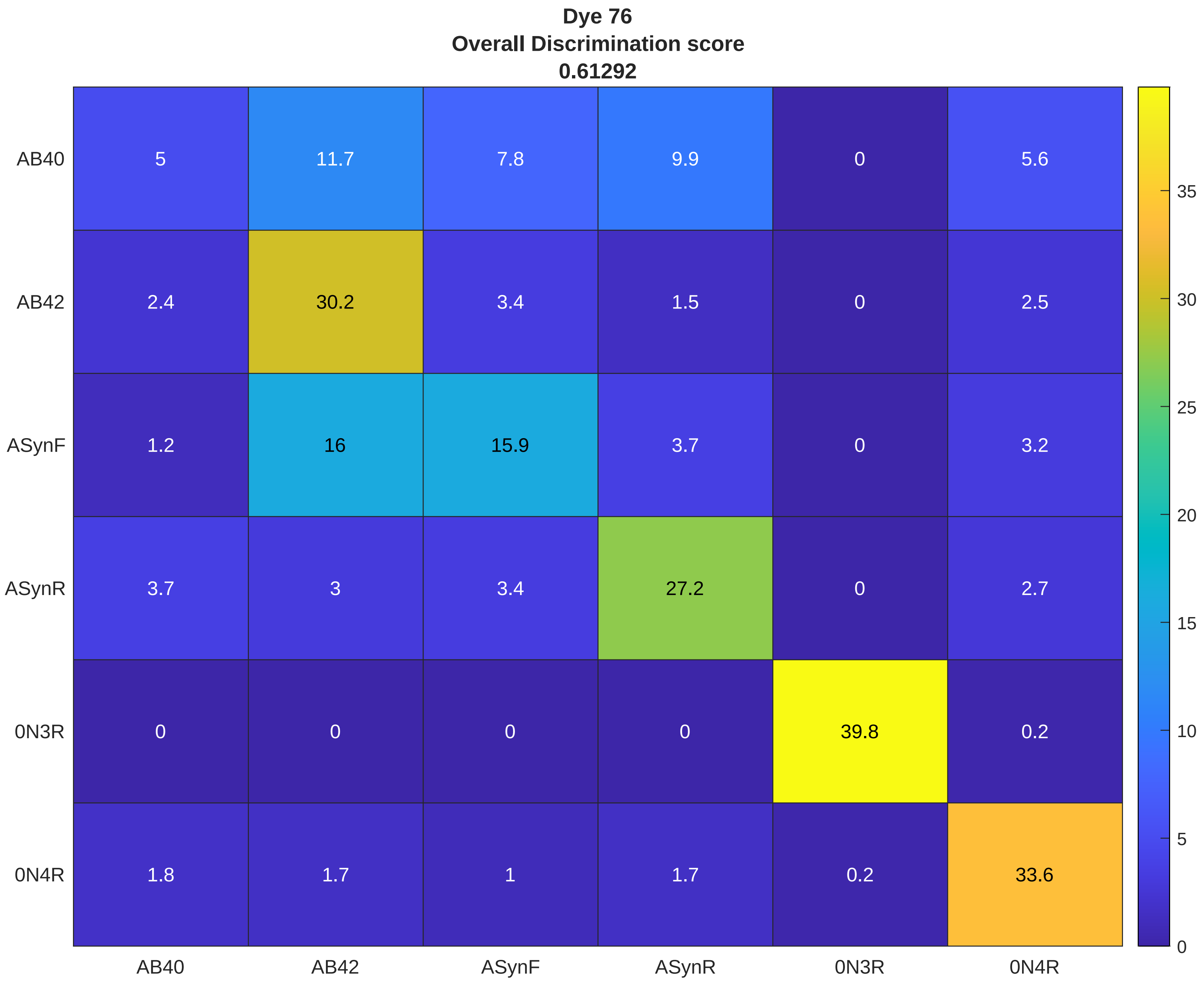
Dye 76
Overall Discrimination score
0.61292
AB40
AB42
ASynF
ASynR
0N3R
0N4R
5
11.7
7.8
9.9
0
5.6
2.4
30.2
3.4
1.5
0
2.5
1.2
16
15.9
3.7
0
3.2
3.7
3
3.4
27.2
0
2.7
0
0
0
0
39.8
0.2
1.8
1.7
1
1.7
0.2
33.6
AB40
AB42
ASynF
ASynR
0N3R
0N4R
35
30
25
20
15
10
5
0

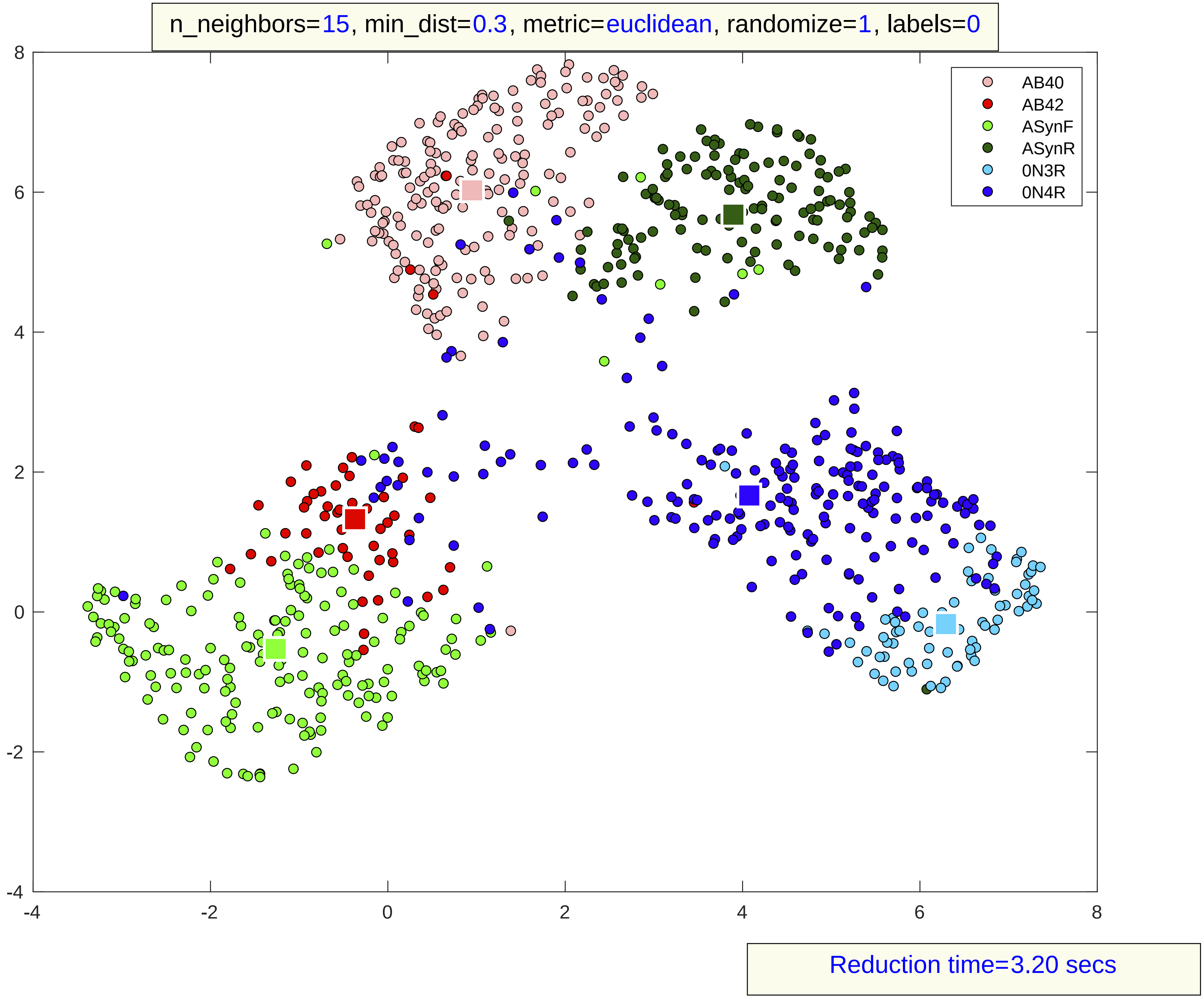

n_neighbors=15, min_dist=0.3, metric=euclidean, randomize=1, labels=0
AB40
AB42
ASynF
ASynR
0N3R
0N4R
8
6
4
2
0
-2
-4
-4
-2
0
2
4
6
8
Reduction time=3.20 secs

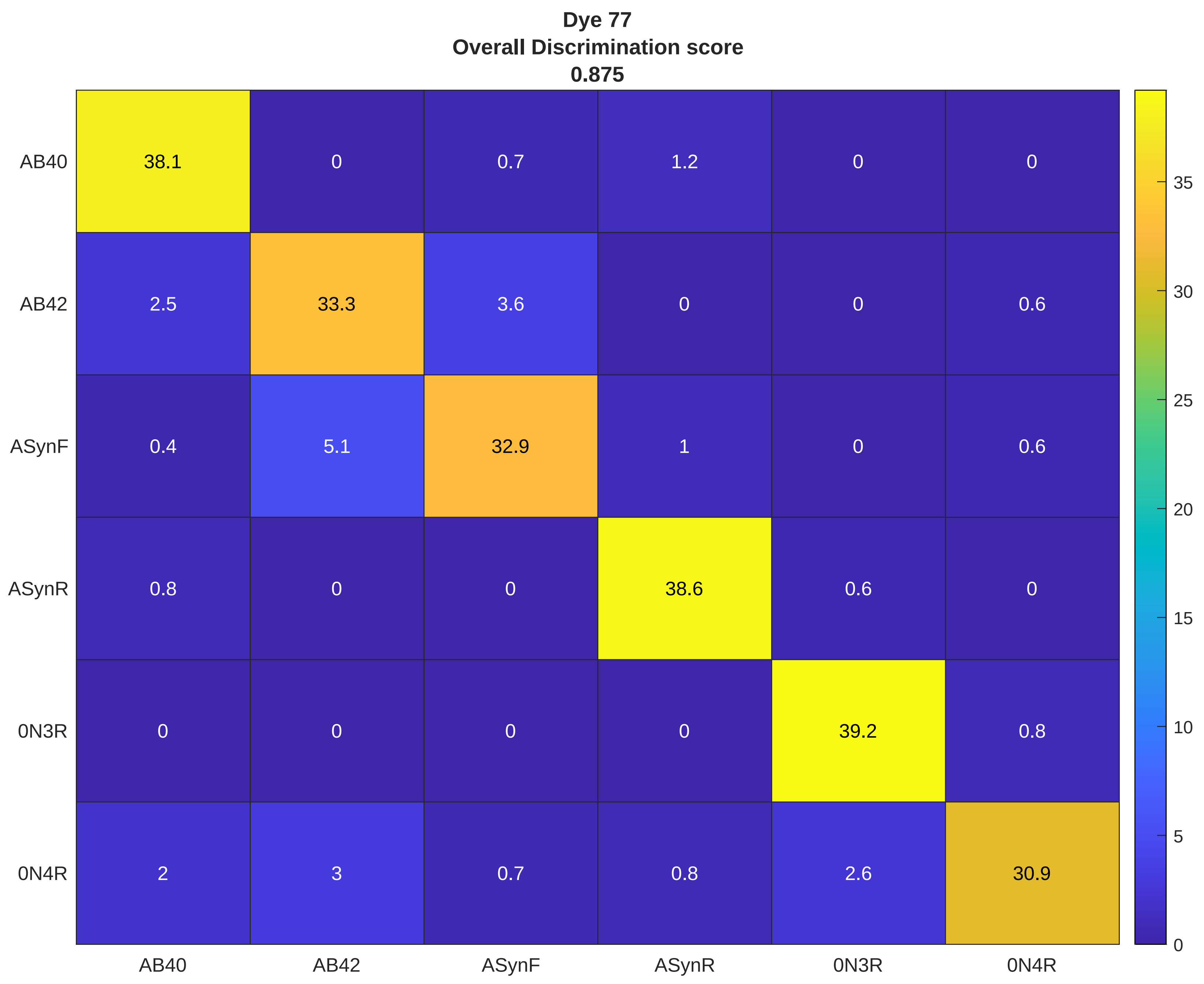

Dye 77
Overall Discrimination score
0.875
AB40
AB42
ASynF
ASynR
0N3R
0N4R
38.1
0
0.7
1.2
0
0
2.5
33.3
3.6
0
0
0.6
0.4
5.1
32.9
1
0
0.6
0.8
0
0
38.6
0.6
0
0
0
0
0
39.2
0.8
2
3
0.7
0.8
2.6
30.9
AB40
AB42
ASynF
ASynR
0N3R
0N4R
35
30
25
20
15
10
5
0

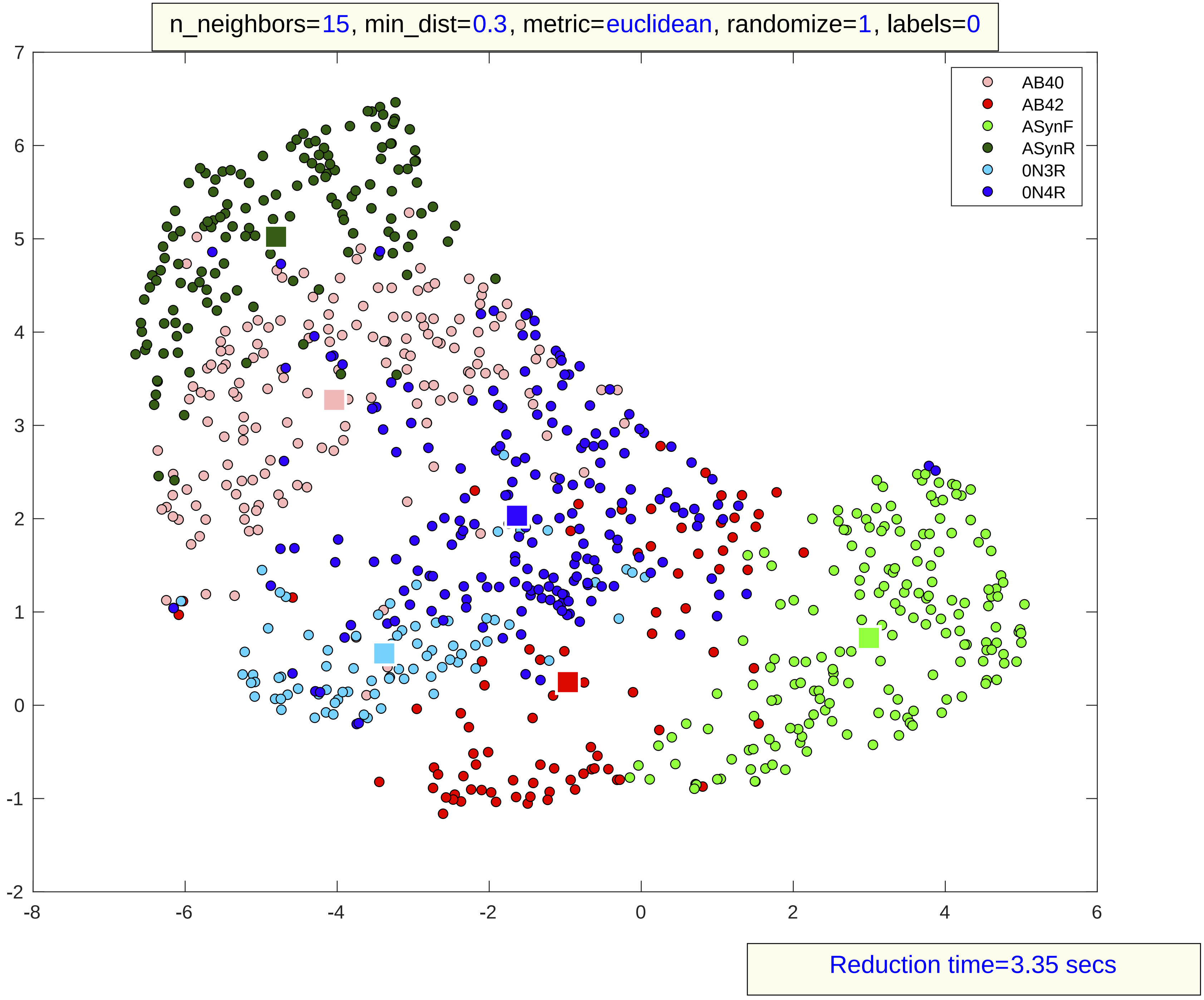
n_neighbors=15, min_dist=0.3, metric=euclidean, randomize=1, labels=0
AB40
AB42
ASynF
ASynR
0N3R
0N4R
7
6
5
4
3
2
1
0
-1
-2
-8
-6
-4
-2
0
2
4
6
Reduction time=3.35 secs

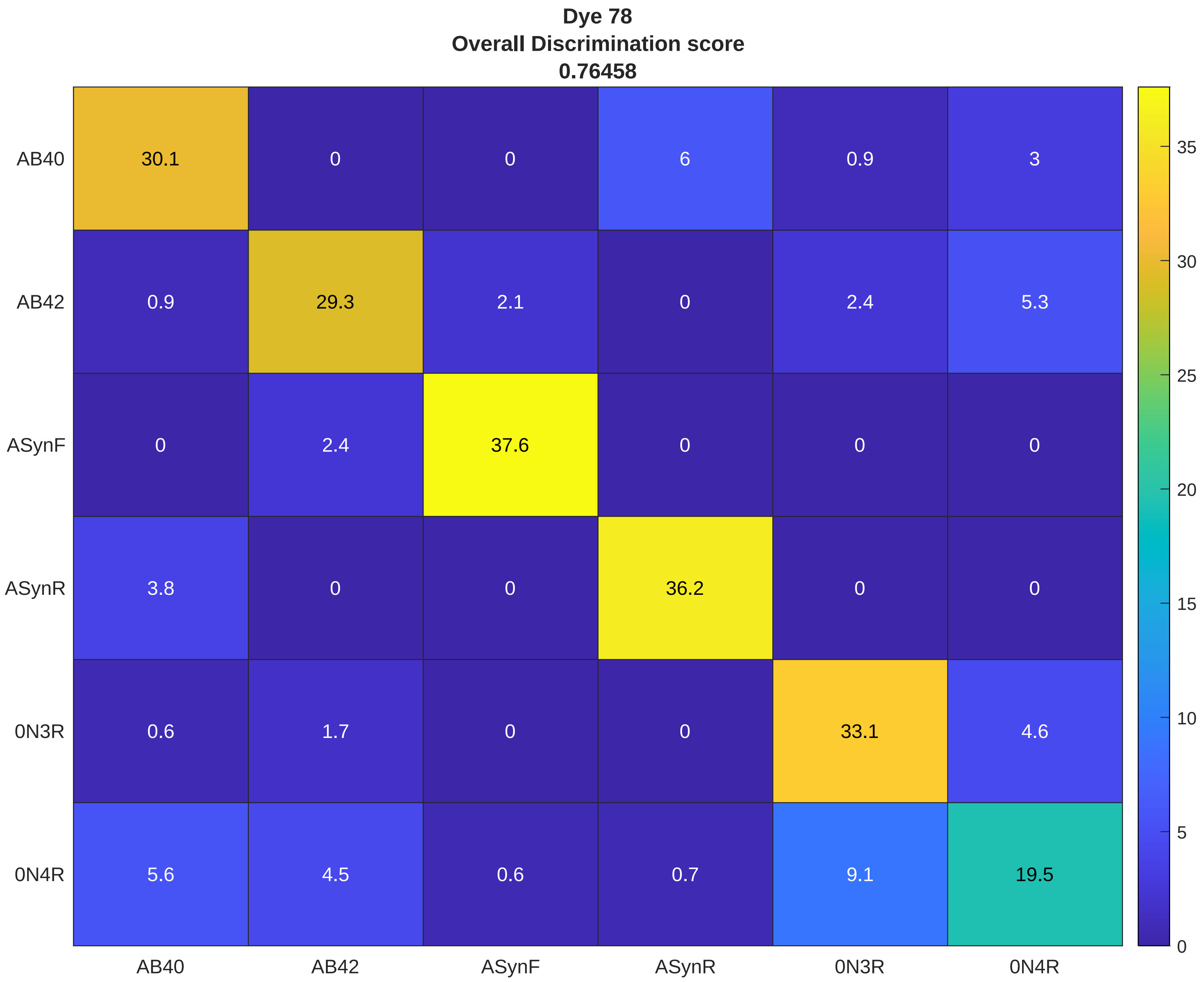
Dye 78
Overall Discrimination score
0.76458
AB40
AB42
ASynF
ASynR
0N3R
0N4R
30.1
0
0
6
0.9
3
0.9
29.3
2.1
0
2.4
5.3
0
2.4
37.6
0
0
0
3.8
0
0
36.2
0
0
0.6
1.7
0
0
33.1
4.6
5.6
4.5
0.6
0.7
9.1
19.5
AB40
AB42
ASynF
ASynR
0N3R
0N4R
35
30
25
20
15
10
5
0

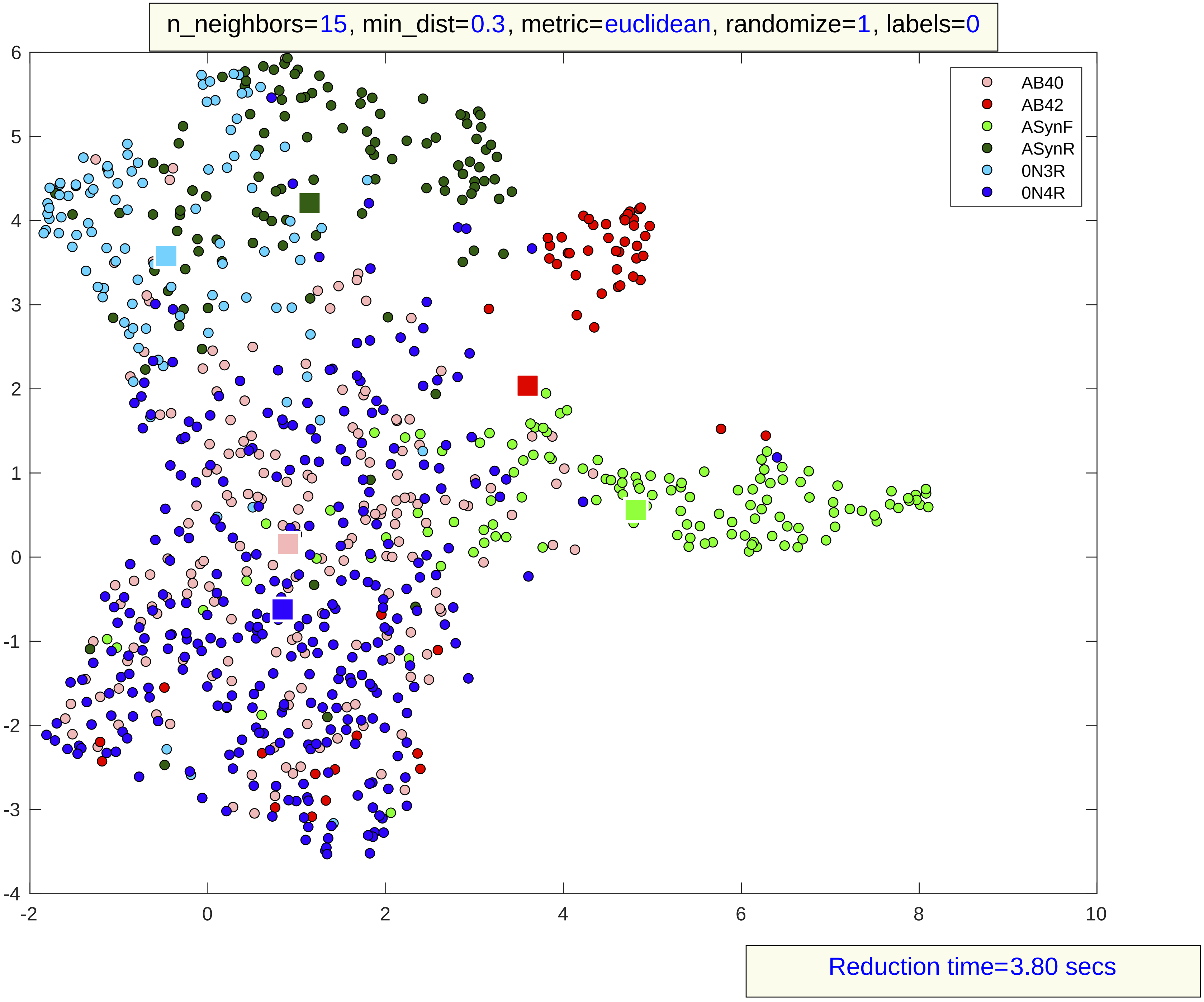
n_neighbors=15, min_dist=0.3, metric=euclidean, randomize=1, labels=0
AB40
AB42
ASynF
ASynR
0N3R
0N4R
6
5
4
3
2
1
0
-1
-2
-3
-4
-2
0
2
4
6
8
10
Reduction time=3.80 secs

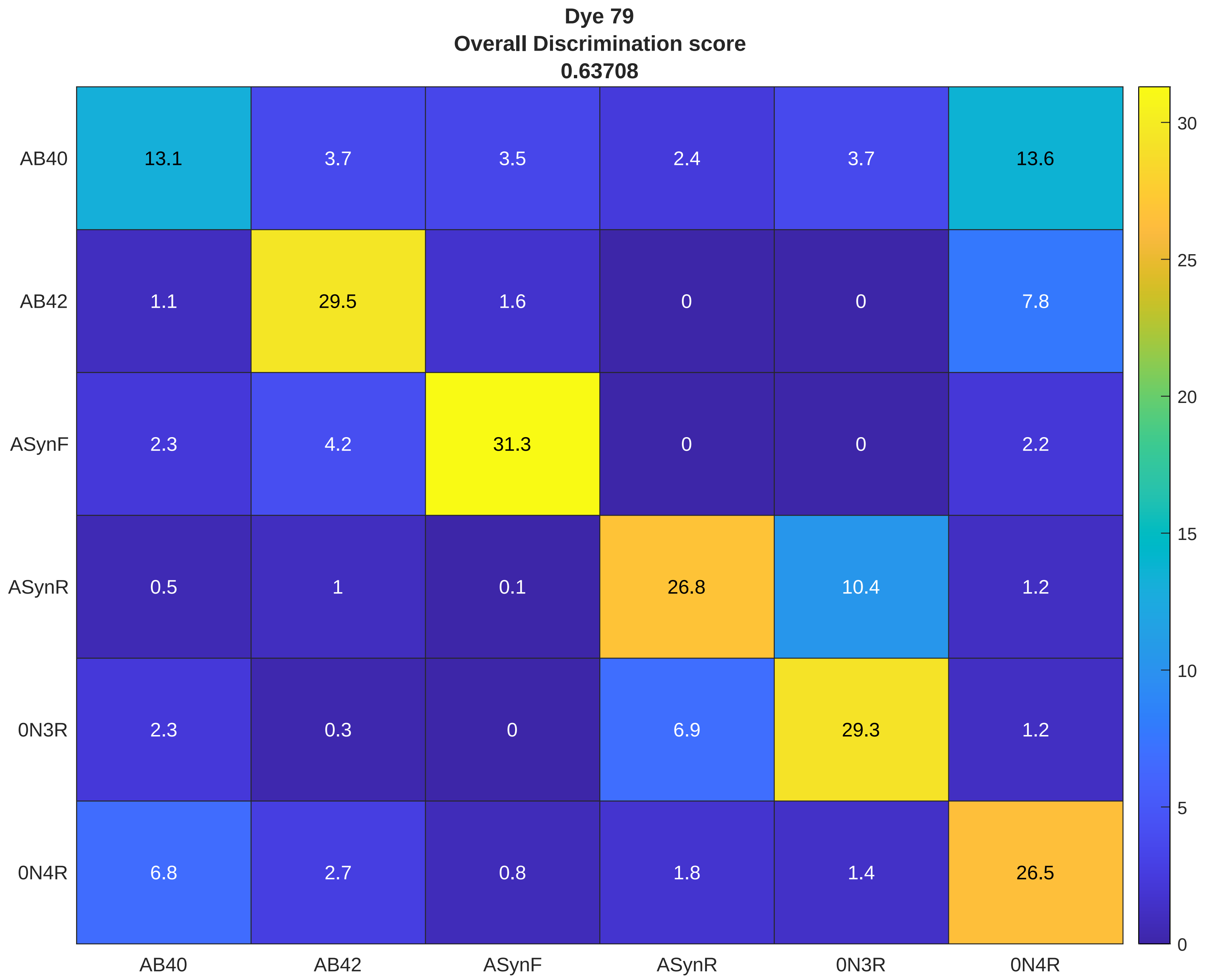
Dye 79
Overall Discrimination score
0.63708
AB40
AB42
ASynF
ASynR
0N3R
0N4R
13.1
3.7
3.5
2.4
3.7
13.6
1.1
29.5
1.6
0
0
7.8
2.3
4.2
31.3
0
0
2.2
0.5
1
0.1
26.8
10.4
1.2
2.3
0.3
0
6.9
29.3
1.2
6.8
2.7
0.8
1.8
1.4
26.5
AB40
AB42
ASynF
ASynR
0N3R
0N4R
30
25
20
15
10
5
0

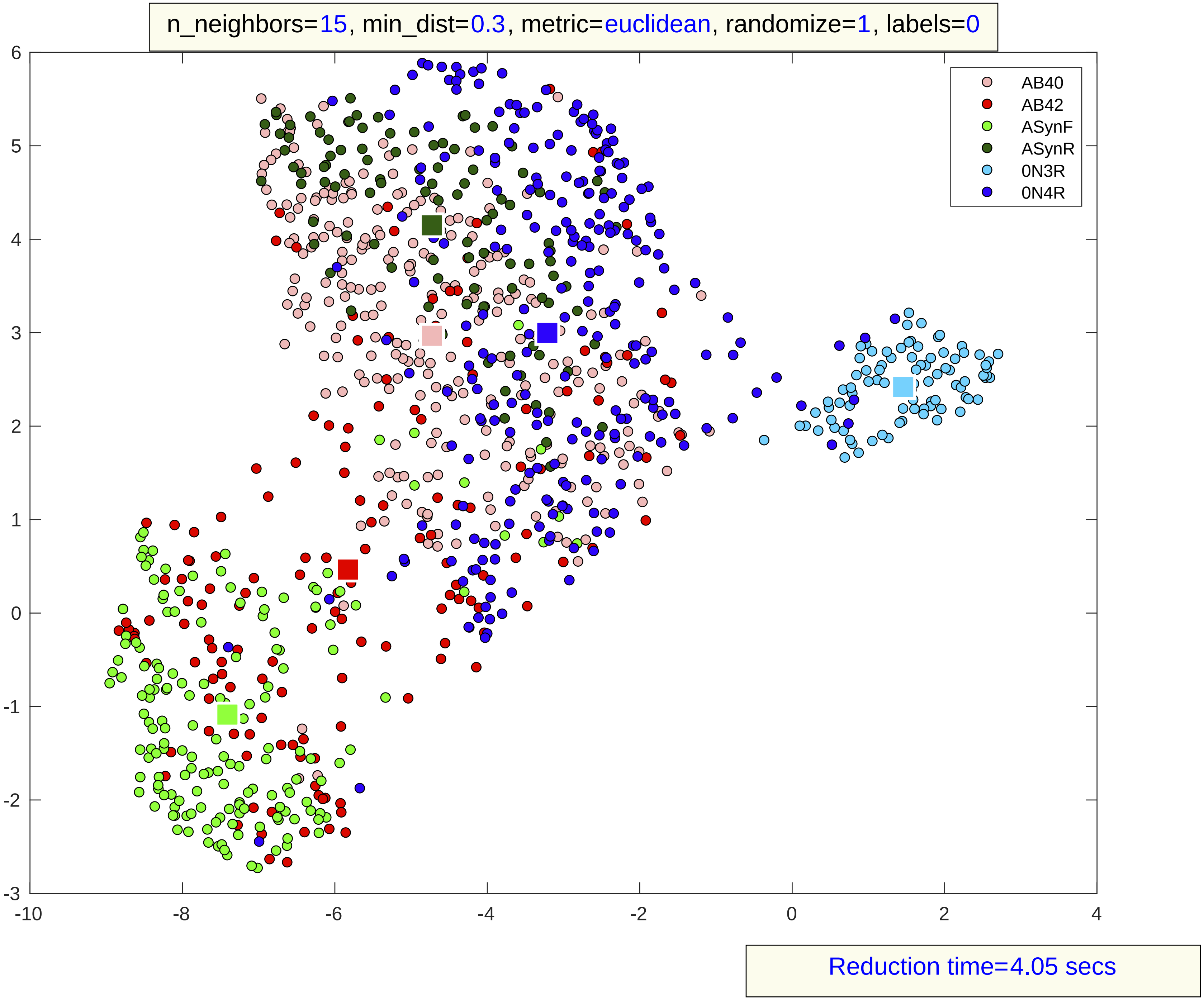

n_neighbors=15, min_dist=0.3, metric=euclidean, randomize=1, labels=0
AB40
AB42
ASynF
ASynR
0N3R
0N4R
6
5
4
3
2
1
0
-1
-2
-3
-10
-8
-6
-4
-2
0
2
4
Reduction time=4.05 secs

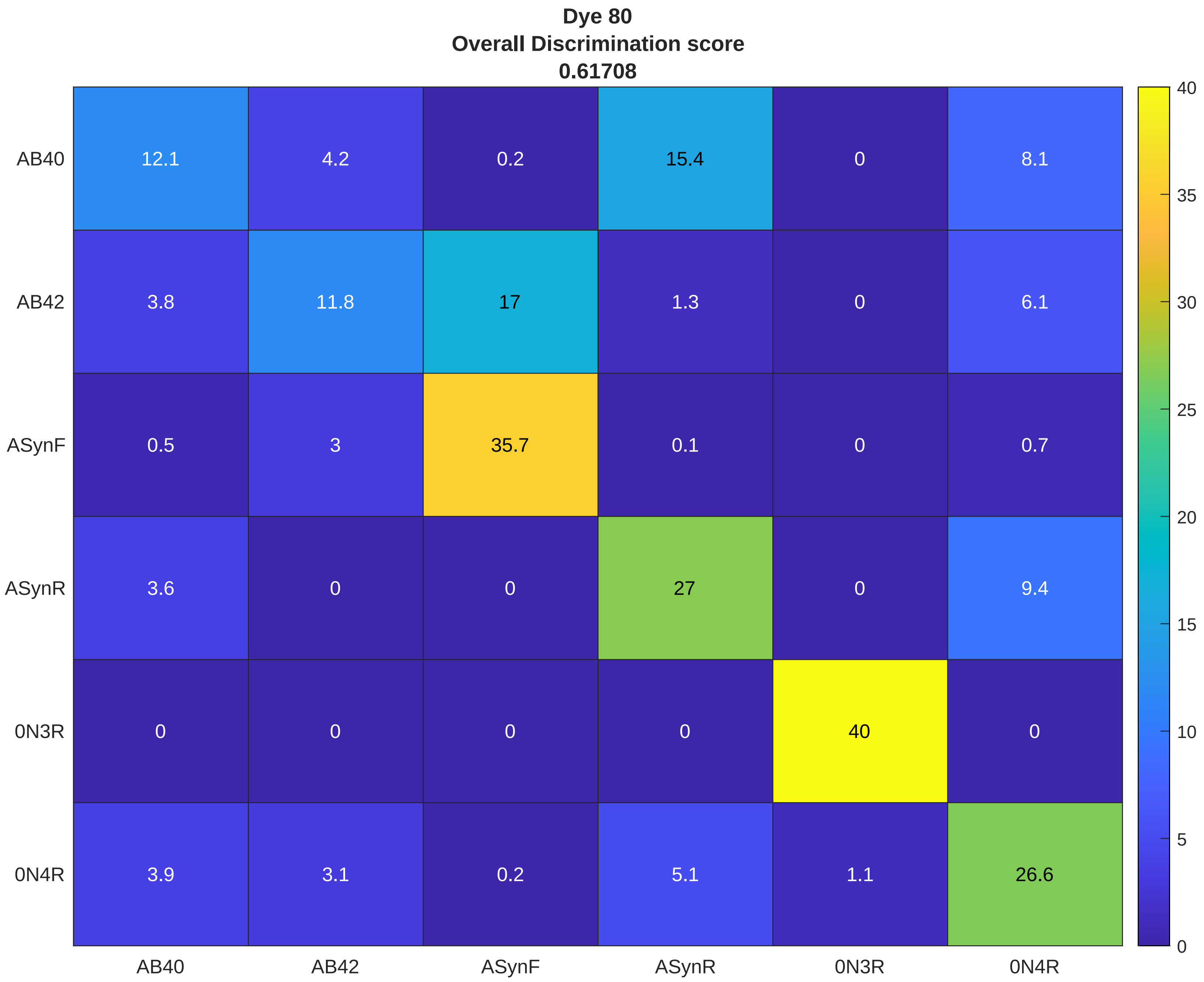
Dye 80
Overall Discrimination score
0.61708
AB40
AB42
ASynF
ASynR
0N3R
0N4R
12.1 4.2 0.2 15.4 0 8.1
3.8 11.8 17 1.3 0 6.1
0.5 3 35.7 0.1 0 0.7
3.6 0 0 27 0 9.4
0 0 0 0 40 0
3.9 3.1 0.2 5.1 1.1 26.6
AB40
AB42
ASynF
ASynR
0N3R
0N4R
40
35
30
25
20
15
10
5
0

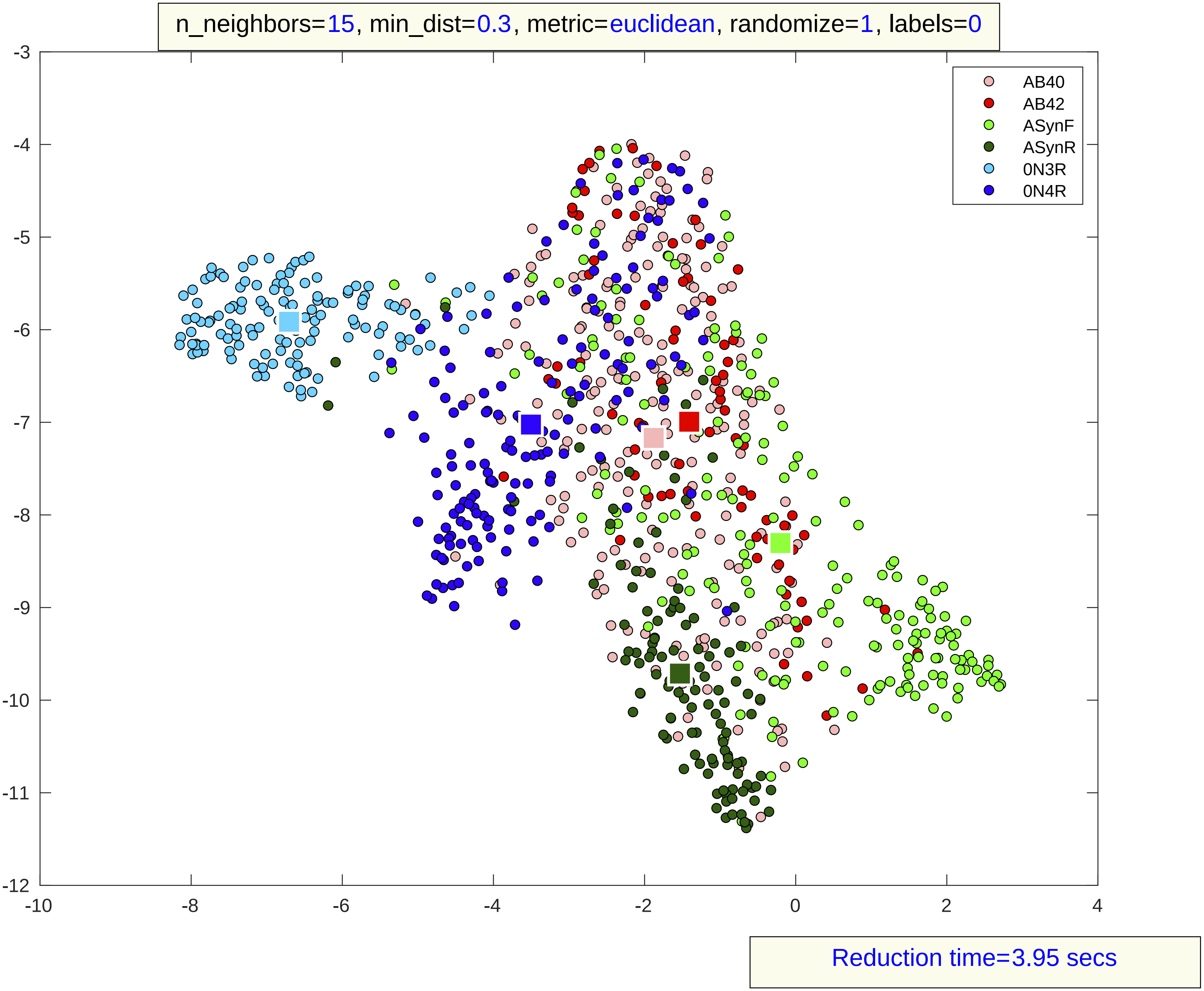
n_neighbors=15, min_dist=0.3, metric=euclidean, randomize=1, labels=0
AB40
AB42
ASynF
ASynR
0N3R
0N4R
-3
-4
-5
-6
-7
-8
-9
-10
-11
-12
-10
-8
-6
-4
-2
0
2
4
Reduction time=3.95 secs

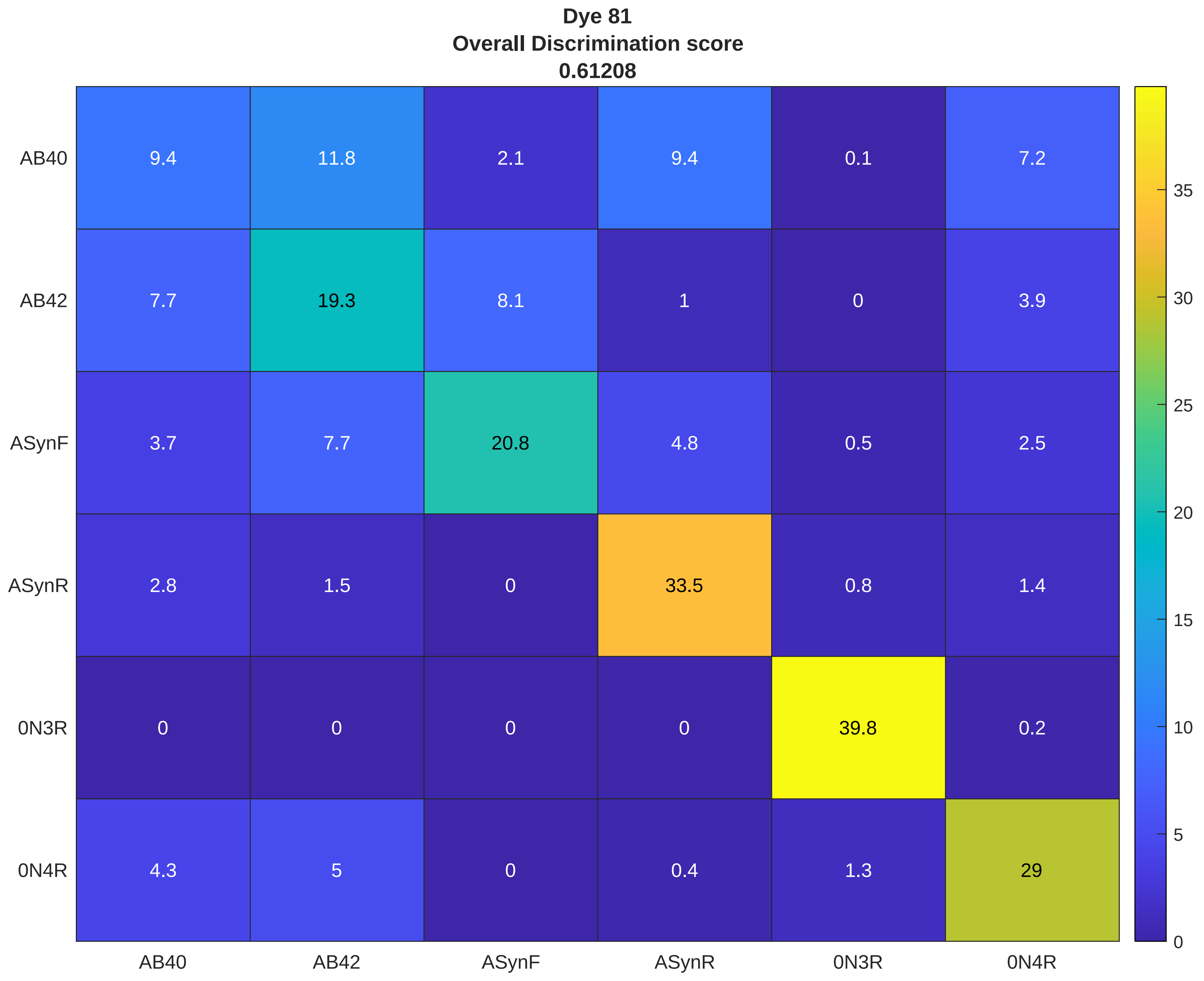
Dye 81
Overall Discrimination score
0.61208
AB40
AB42
ASynF
ASynR
0N3R
0N4R
9.4 11.8 2.1 9.4 0.1 7.2
7.7 19.3 8.1 1 0 3.9
3.7 7.7 20.8 4.8 0.5 2.5
2.8 1.5 0 33.5 0.8 1.4
0 0 0 0 39.8 0.2
4.3 5 0 0.4 1.3 29
AB40
AB42
ASynF
ASynR
0N3R
0N4R
35
30
25
20
15
10
5
0

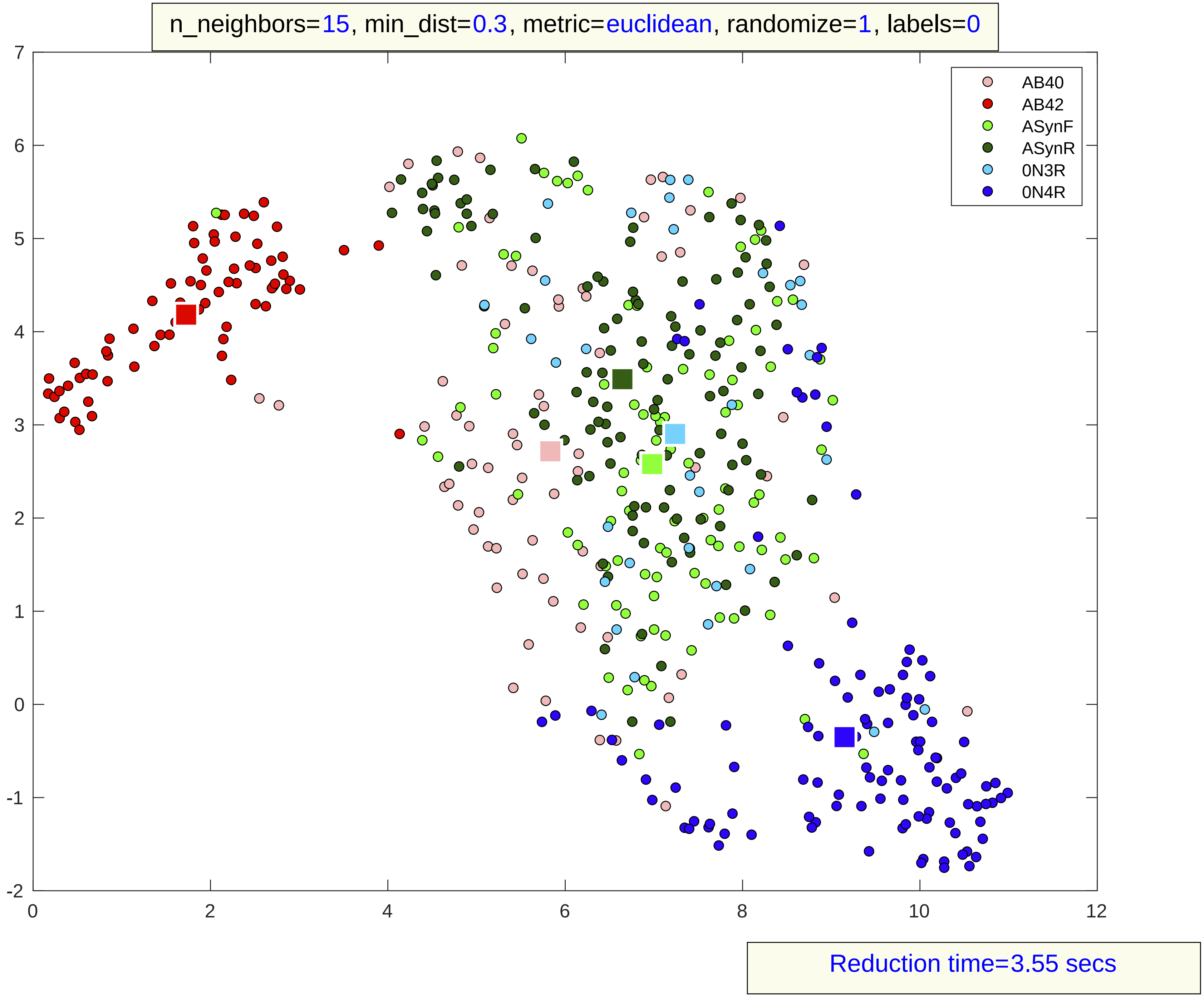

n_neighbors=15, min_dist=0.3, metric=euclidean, randomize=1, labels=0
AB40
AB42
ASynF
ASynR
0N3R
0N4R
7
6
5
4
3
2
1
0
-1
-2
0
2
4
6
8
10
12
Reduction time=3.55 secs

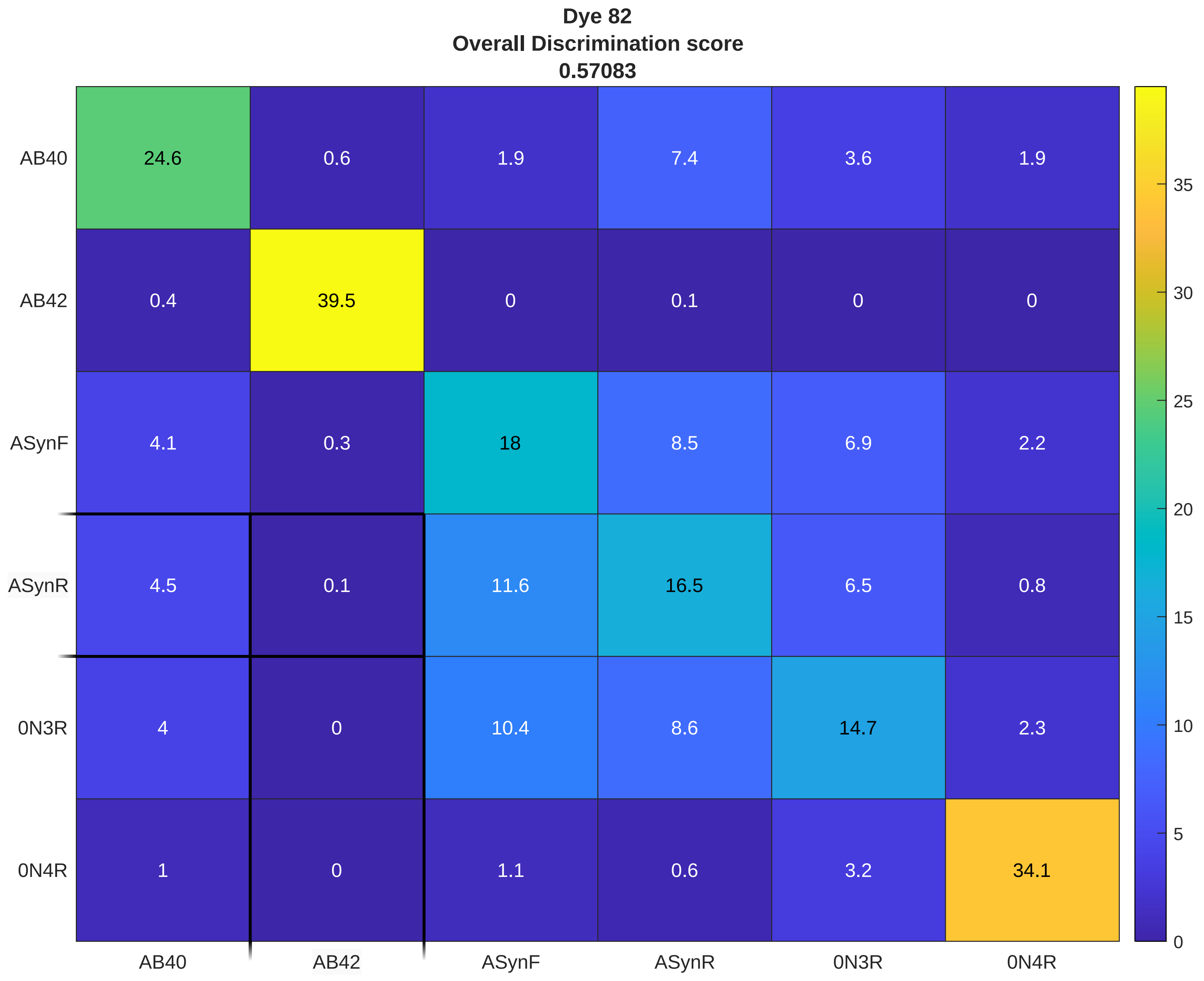
Dye 82
Overall Discrimination score
0.57083
AB40
AB42
ASynF
ASynR
0N3R
0N4R
24.6
0.6
1.9
7.4
3.6
1.9
0.4
39.5
0
0.1
0
0
4.1
0.3
18
8.5
6.9
2.2
4.5
0.1
11.6
16.5
6.5
0.8
4
0
10.4
8.6
14.7
2.3
1
0
1.1
0.6
3.2
34.1
AB40
AB42
ASynF
ASynR
0N3R
0N4R
35
30
25
20
15
10
5
0

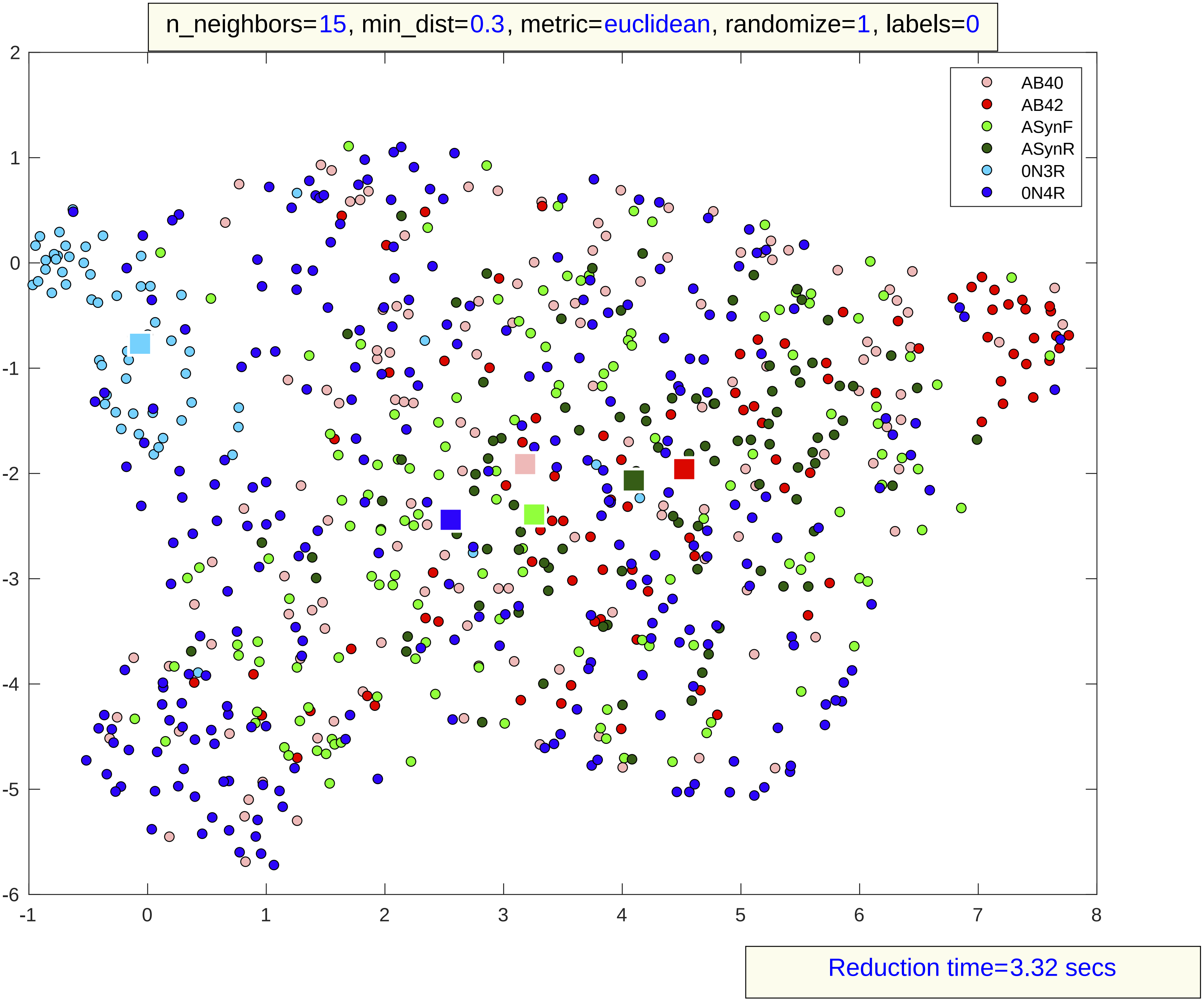
n_neighbors=15, min_dist=0.3, metric=euclidean, randomize=1, labels=0
AB40
AB42
ASynF
ASynR
0N3R
0N4R
2
1
0
-1
-2
-3
-4
-5
-6
-1
0
1
2
3
4
5
6
7
8
Reduction time=3.32 secs

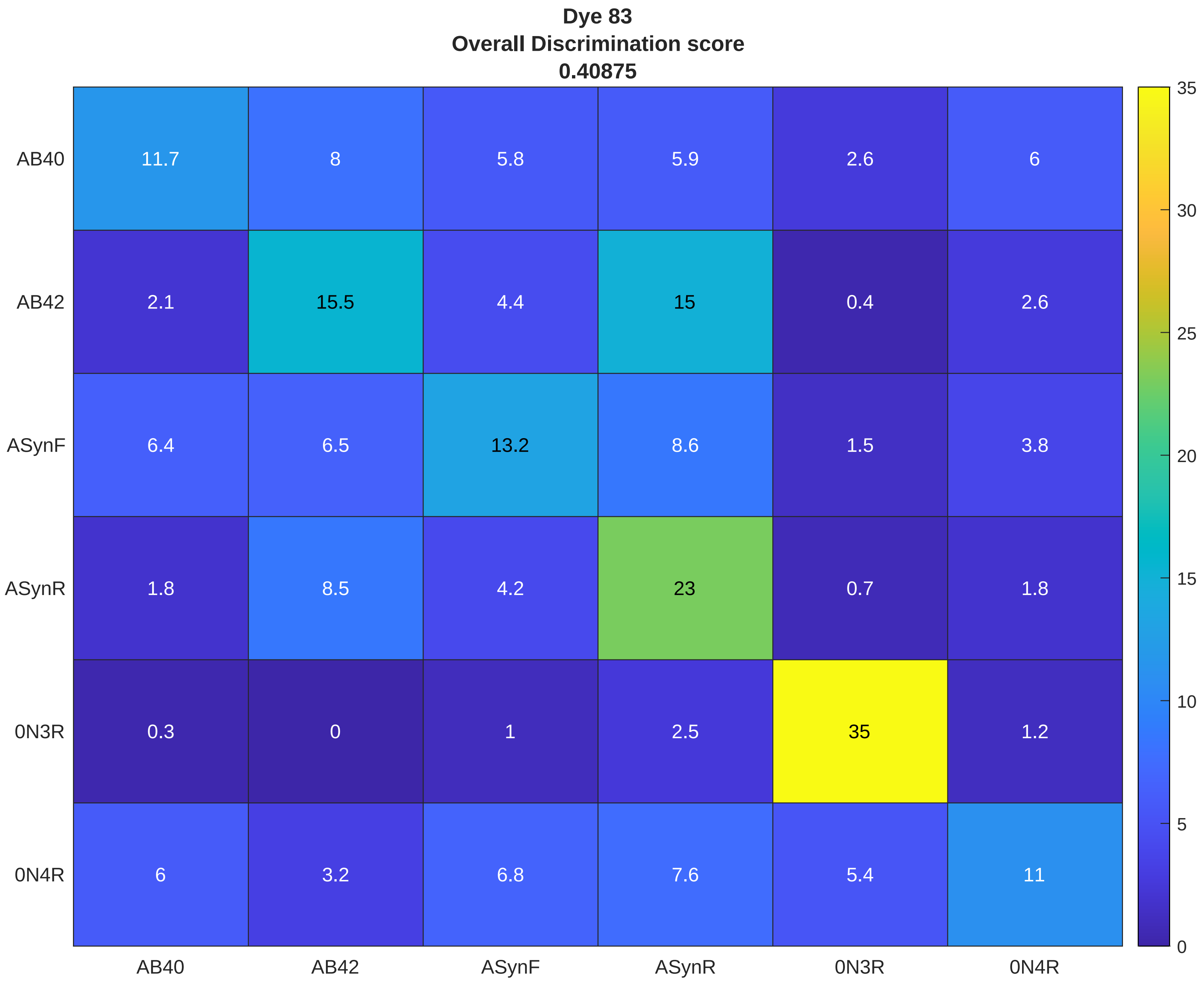
Dye 83
Overall Discrimination score
0.40875
AB40
AB42
ASynF
ASynR
0N3R
0N4R
11.7
8
5.8
5.9
2.6
6
2.1
15.5
4.4
15
0.4
2.6
6.4
6.5
13.2
8.6
1.5
3.8
1.8
8.5
4.2
23
0.7
1.8
0.3
0
1
2.5
35
1.2
6
3.2
6.8
7.6
5.4
11
AB40
AB42
ASynF
ASynR
0N3R
0N4R
35
30
25
20
15
10
5
0

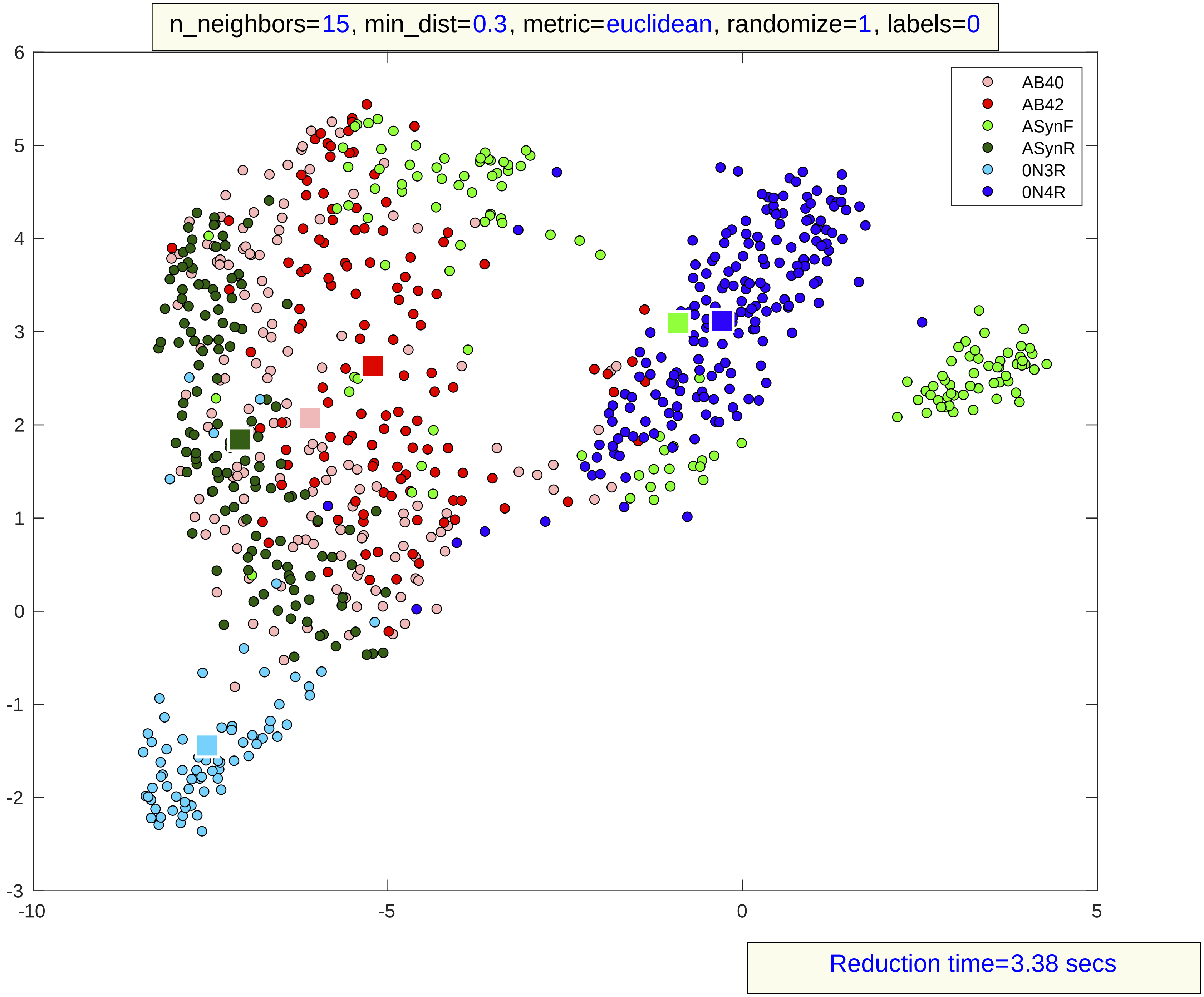

n_neighbors=15, min_dist=0.3, metric=euclidean, randomize=1, labels=0
AB40
AB42
ASynF
ASynR
0N3R
0N4R
6
5
4
3
2
1
0
-1
-2
-3
-10
-5
0
5
Reduction time=3.38 secs

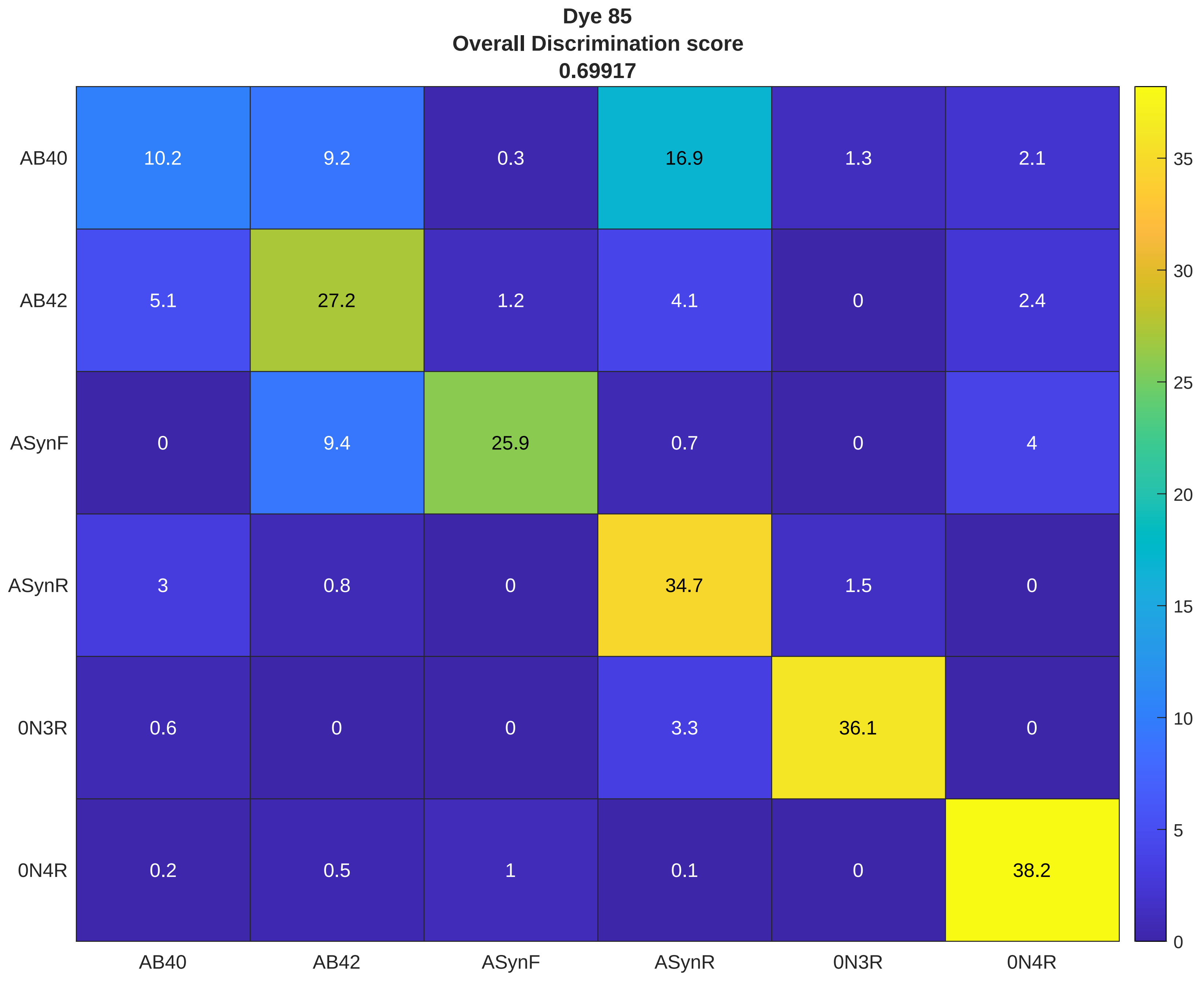

Dye 85
Overall Discrimination score
0.69917
AB40
AB42
ASynF
ASynR
0N3R
0N4R
10.2
9.2
0.3
16.9
1.3
2.1
5.1
27.2
1.2
4.1
0
2.4
0
9.4
25.9
0.7
0
4
3
0.8
0
34.7
1.5
0
0.6
0
0
3.3
36.1
0
0.2
0.5
1
0.1
0
38.2
AB40
AB42
ASynF
ASynR
0N3R
0N4R
35
30
25
20
15
10
5
0

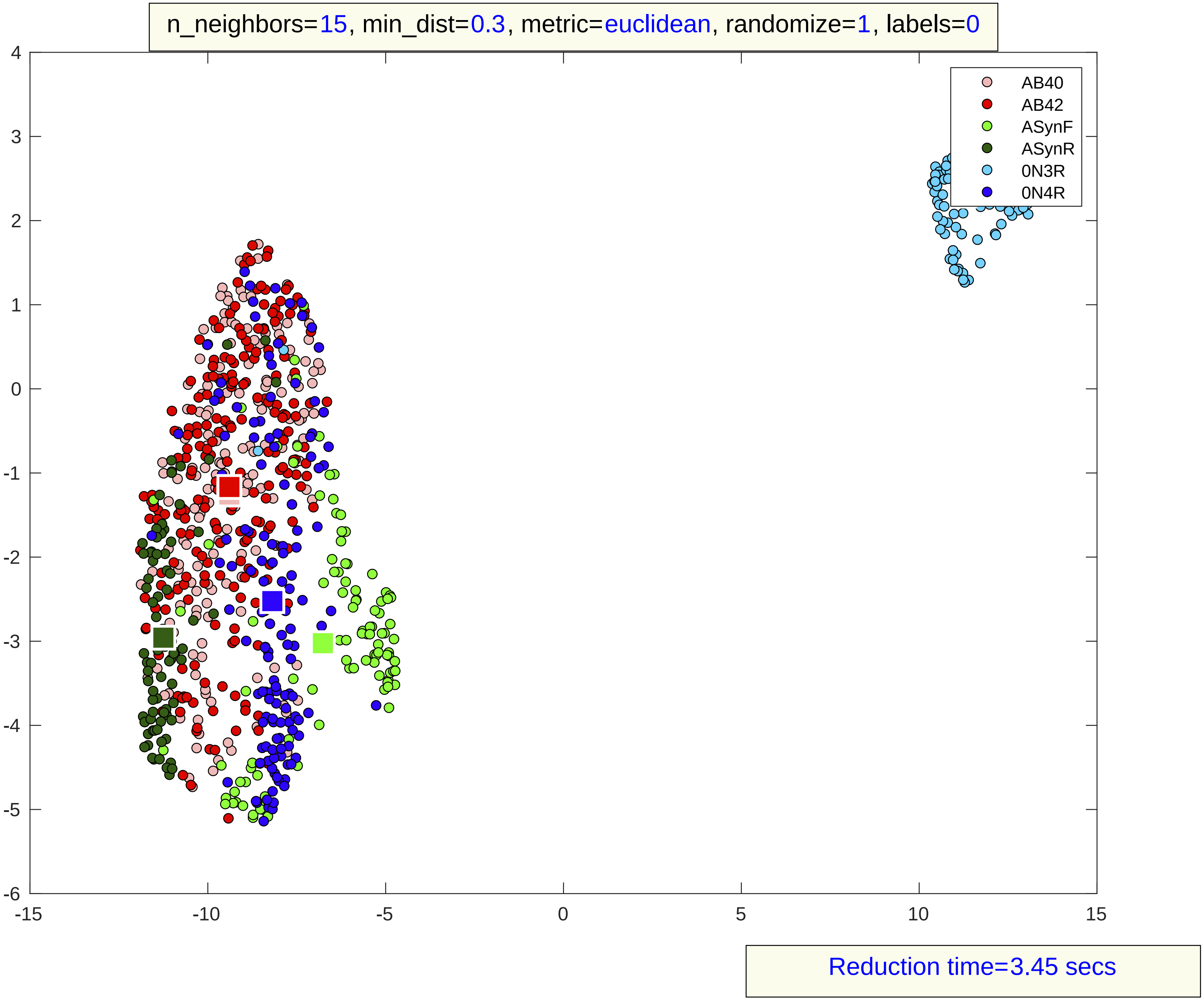

n_neighbors=15, min_dist=0.3, metric=euclidean, randomize=1, labels=0
AB40
AB42
ASynF
ASynR
0N3R
0N4R
4
3
2
1
0
-1
-2
-3
-4
-5
-6
-15
-10
-5
0
5
10
15
Reduction time=3.45 secs

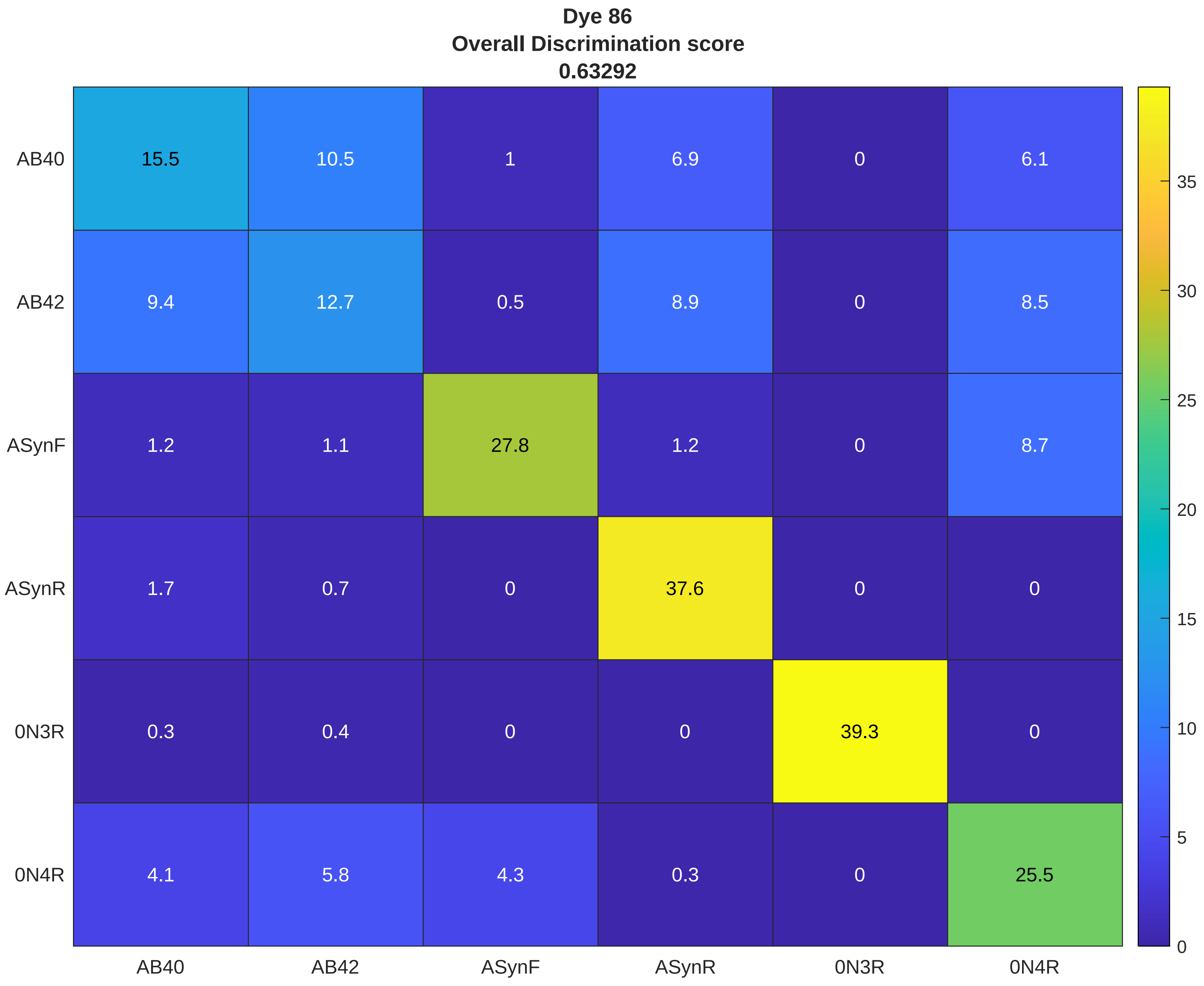
Dye 86
Overall Discrimination score
0.63292
AB40
AB42
ASynF
ASynR
0N3R
0N4R
15.5
10.5
1
6.9
0
6.1
9.4
12.7
0.5
8.9
0
8.5
1.2
1.1
27.8
1.2
0
8.7
1.7
0.7
0
37.6
0
0
0.3
0.4
0
0
39.3
0
4.1
5.8
4.3
0.3
0
25.5
AB40
AB42
ASynF
ASynR
0N3R
0N4R
35
30
25
20
15
10
5
0

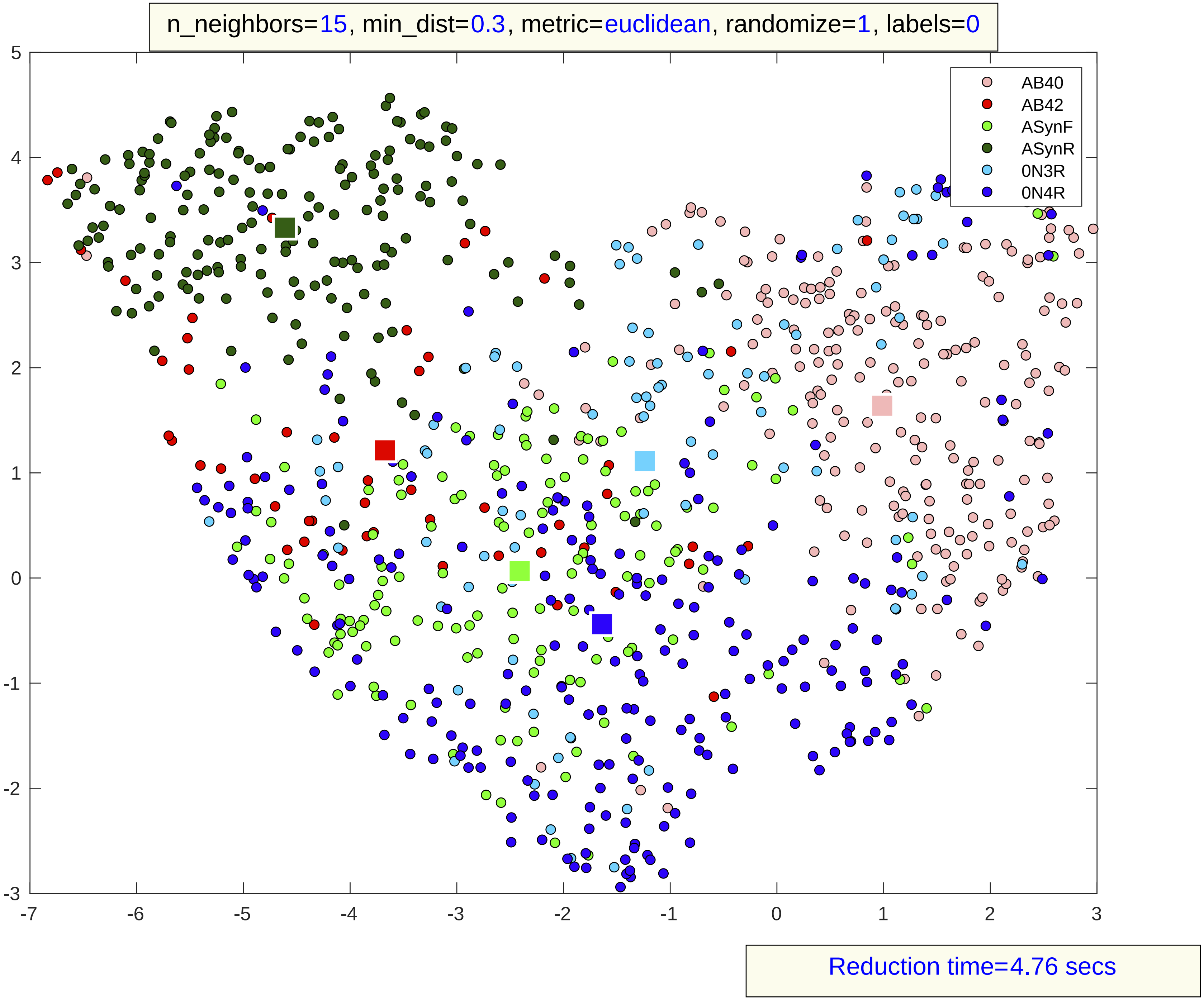
n_neighbors=15, min_dist=0.3, metric=euclidean, randomize=1, labels=0
AB40
AB42
ASynF
ASynR
0N3R
0N4R
5
4
3
2
1
0
-1
-2
-3
-7
-6
-5
-4
-3
-2
-1
0
1
2
3
Reduction time=4.76 secs

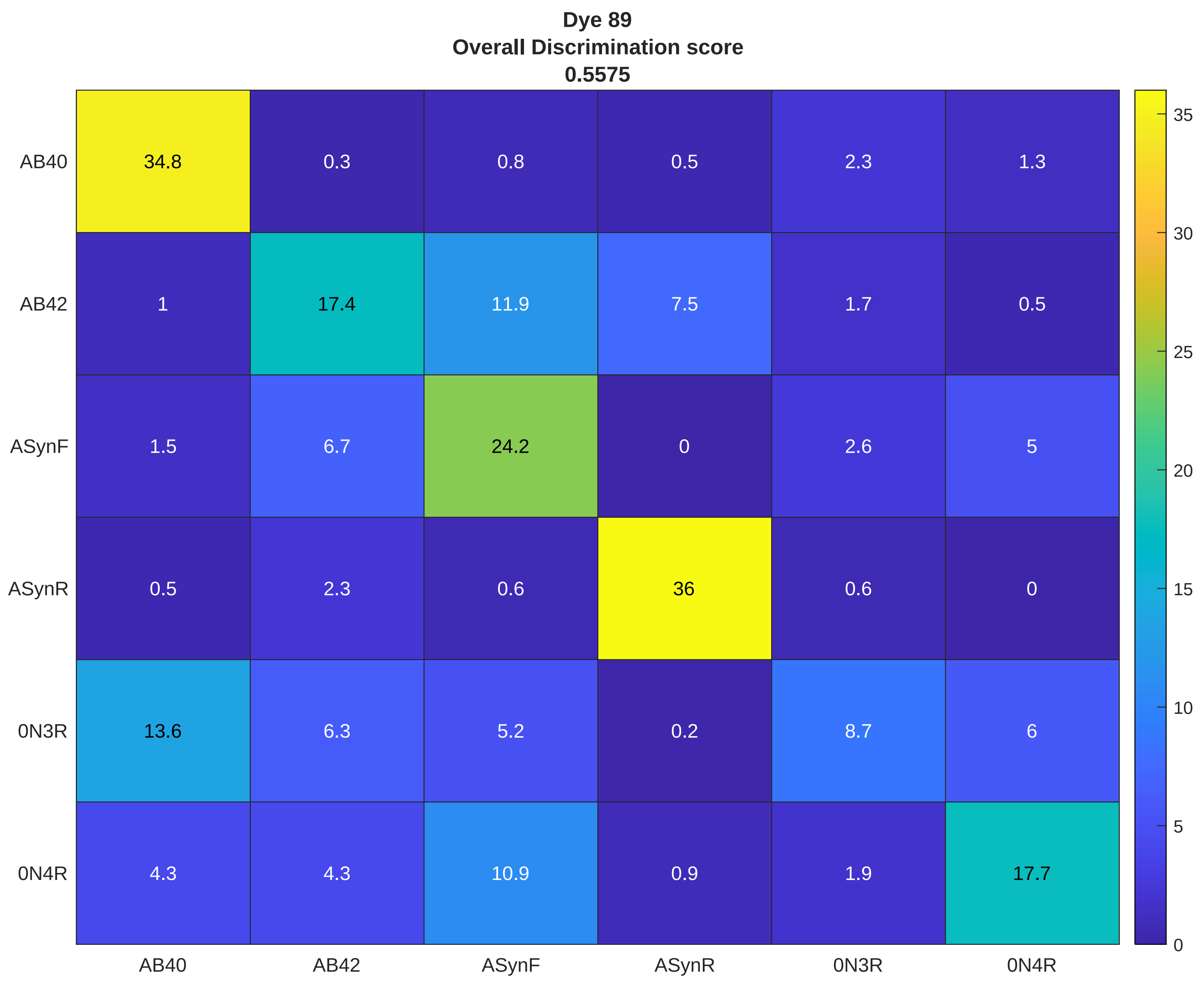
Dye 89
Overall Discrimination score
0.5575
AB40
AB42
ASynF
ASynR
0N3R
0N4R
34.8 0.3 0.8 0.5 2.3 1.3
1 17.4 11.9 7.5 1.7 0.5
1.5 6.7 24.2 0 2.6 5
0.5 2.3 0.6 36 0.6 0
13.6 6.3 5.2 0.2 8.7 6
4.3 4.3 10.9 0.9 1.9 17.7
AB40
AB42
ASynF
ASynR
0N3R
0N4R
35
30
25
20
15
10
5
0

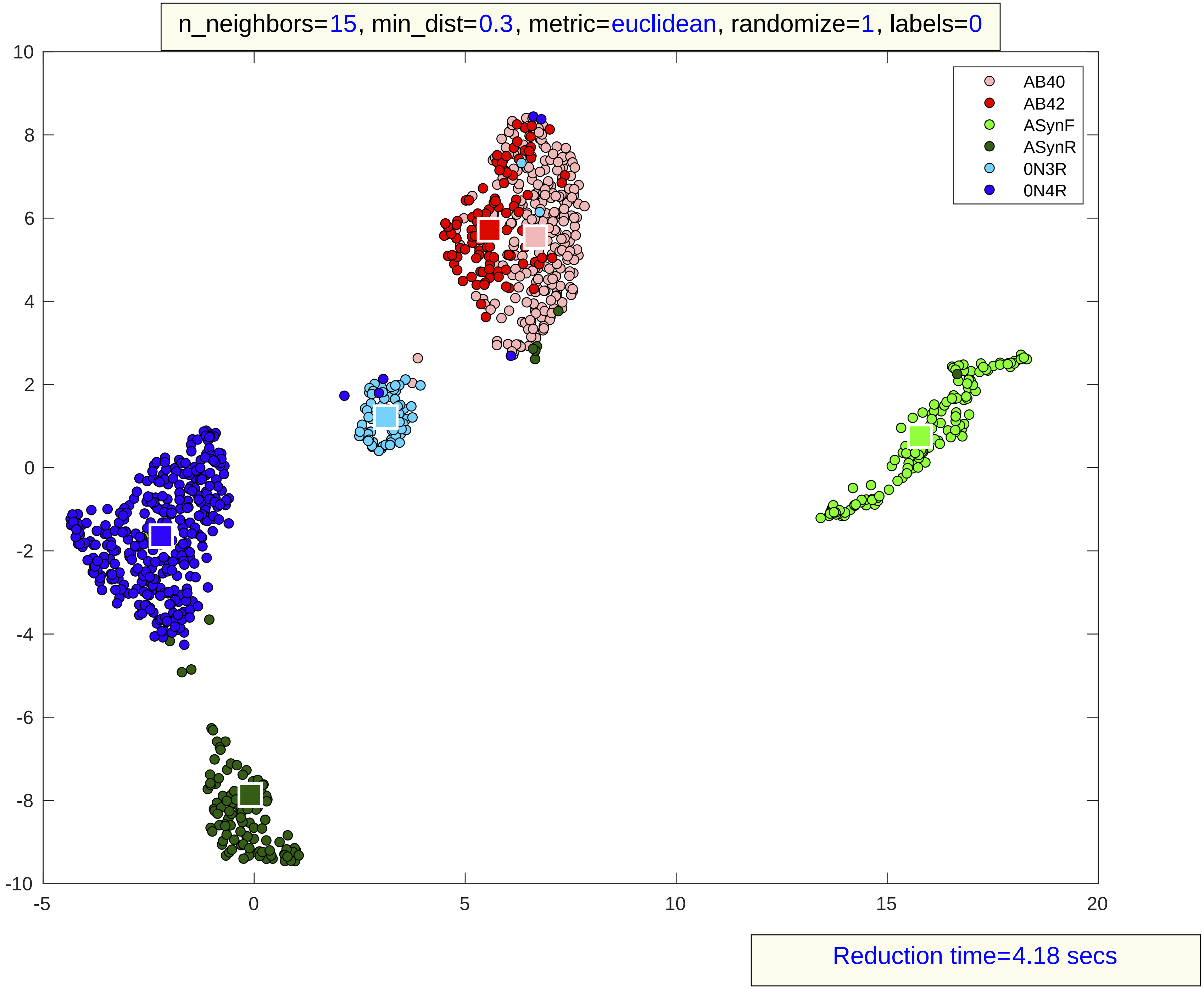

n_neighbors=15, min_dist=0.3, metric=euclidean, randomize=1, labels=0
AB40
AB42
ASynF
ASynR
0N3R
0N4R
10
8
6
4
2
0
-2
-4
-6
-8
-10
-5
0
5
10
15
20
Reduction time=4.18 secs

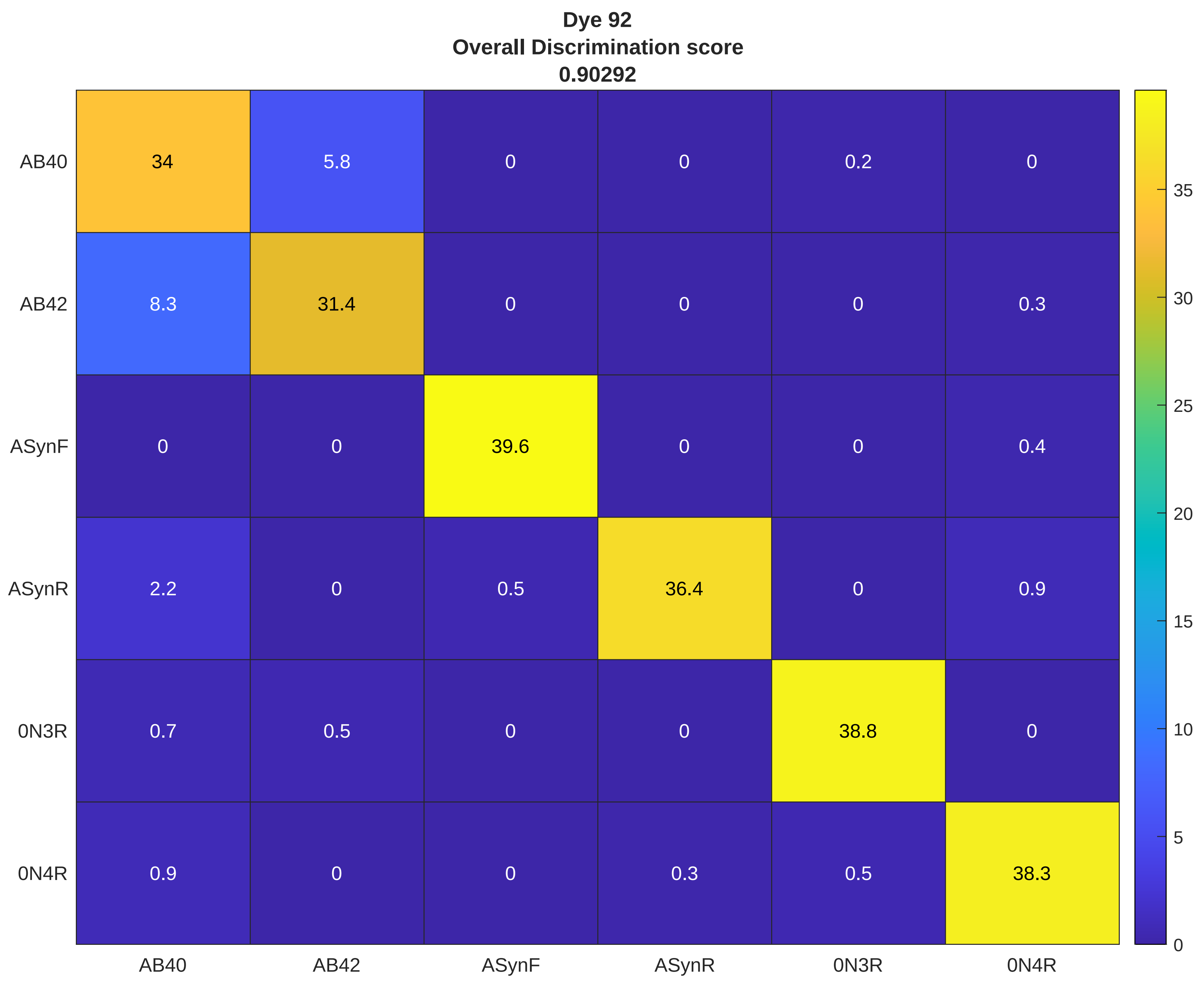
Dye 92
Overall Discrimination score
0.90292
AB40
AB42
ASynF
ASynR
0N3R
0N4R
34
5.8
0
0
0.2
0
8.3
31.4
0
0
0
0.3
0
0
39.6
0
0
0.4
2.2
0
0.5
36.4
0
0.9
0.7
0.5
0
0
38.8
0
0.9
0
0
0.3
0.5
38.3
AB40
AB42
ASynF
ASynR
0N3R
0N4R
35
30
25
20
15
10
5
0

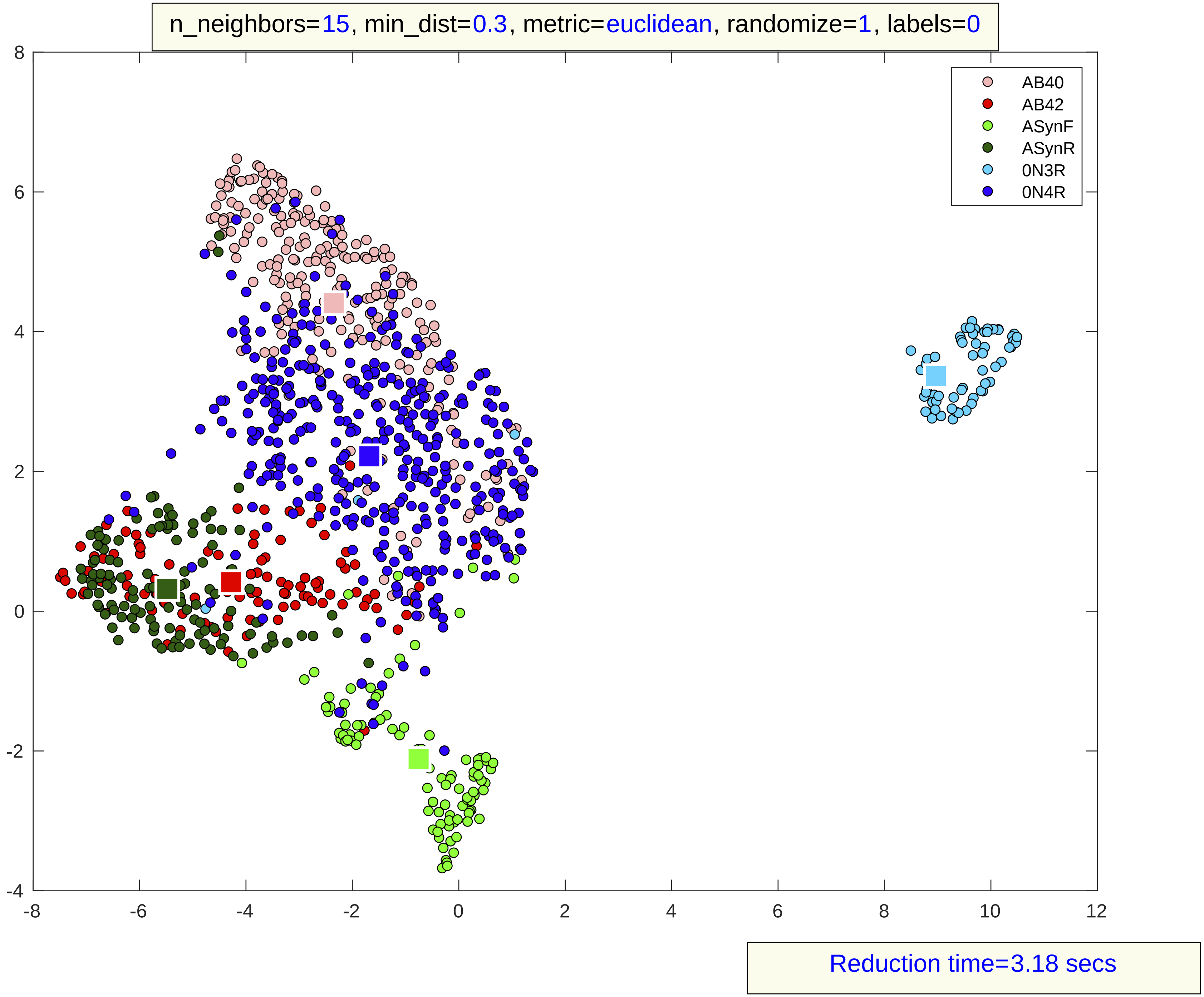

n_neighbors=15, min_dist=0.3, metric=euclidean, randomize=1, labels=0
AB40
AB42
ASynF
ASynR
0N3R
0N4R
8
6
4
2
0
-2
-4
-8
-6
-4
-2
0
2
4
6
8
10
12
Reduction time=3.18 secs

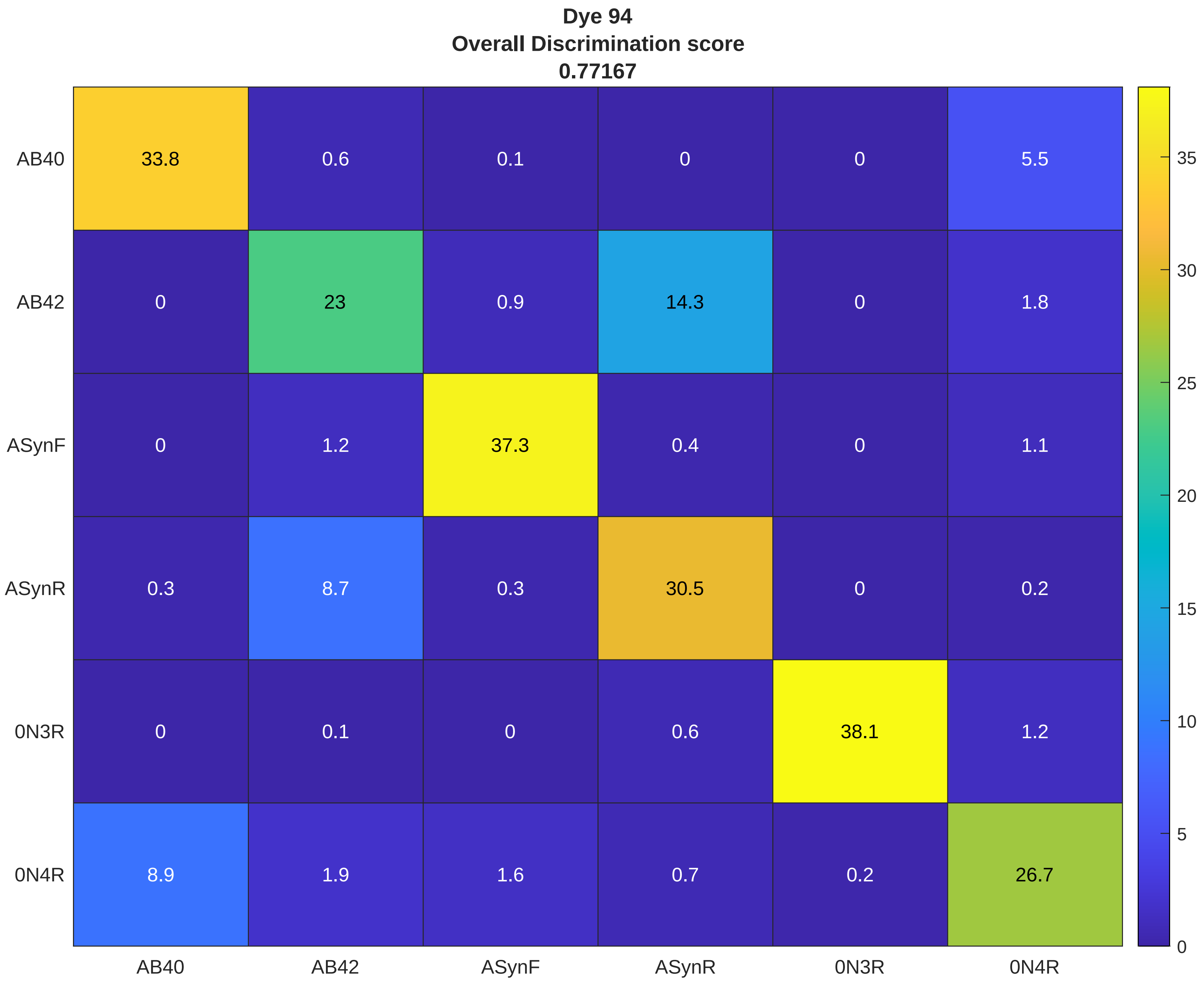
Dye 94
Overall Discrimination score
0.77167
AB40
AB42
ASynF
ASynR
0N3R
0N4R
33.8
0.6
0.1
0
0
5.5
0
23
0.9
14.3
0
1.8
0
1.2
37.3
0.4
0
1.1
0.3
8.7
0.3
30.5
0
0.2
0
0.1
0
0.6
38.1
1.2
8.9
1.9
1.6
0.7
0.2
26.7
AB40
AB42
ASynF
ASynR
0N3R
0N4R
35
30
25
20
15
10
5
0

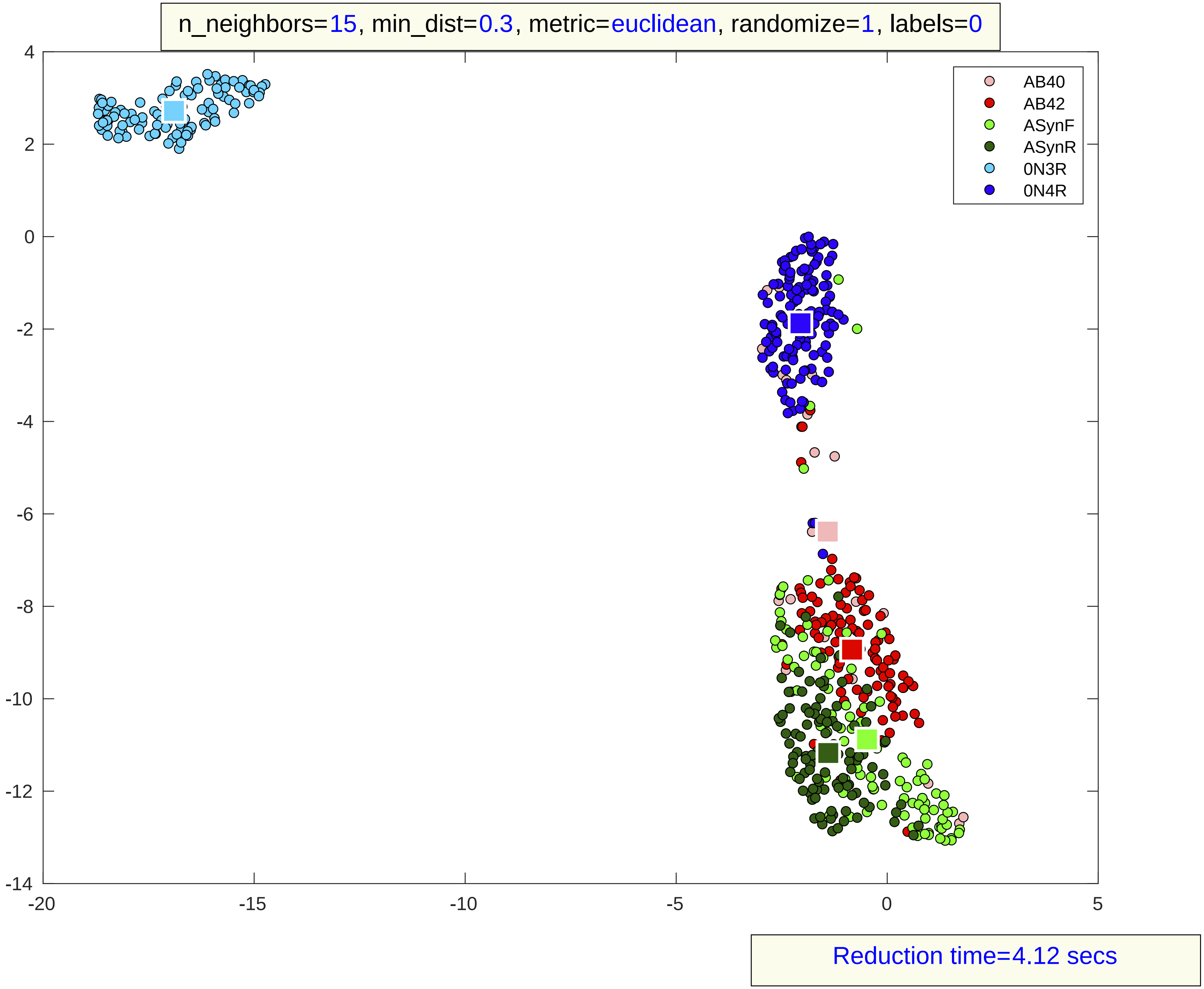

n_neighbors=15, min_dist=0.3, metric=euclidean, randomize=1, labels=0
AB40
AB42
ASynF
ASynR
0N3R
0N4R
4
2
0
-2
-4
-6
-8
-10
-12
-14
-20
-15
-10
-5
0
5
Reduction time=4.12 secs

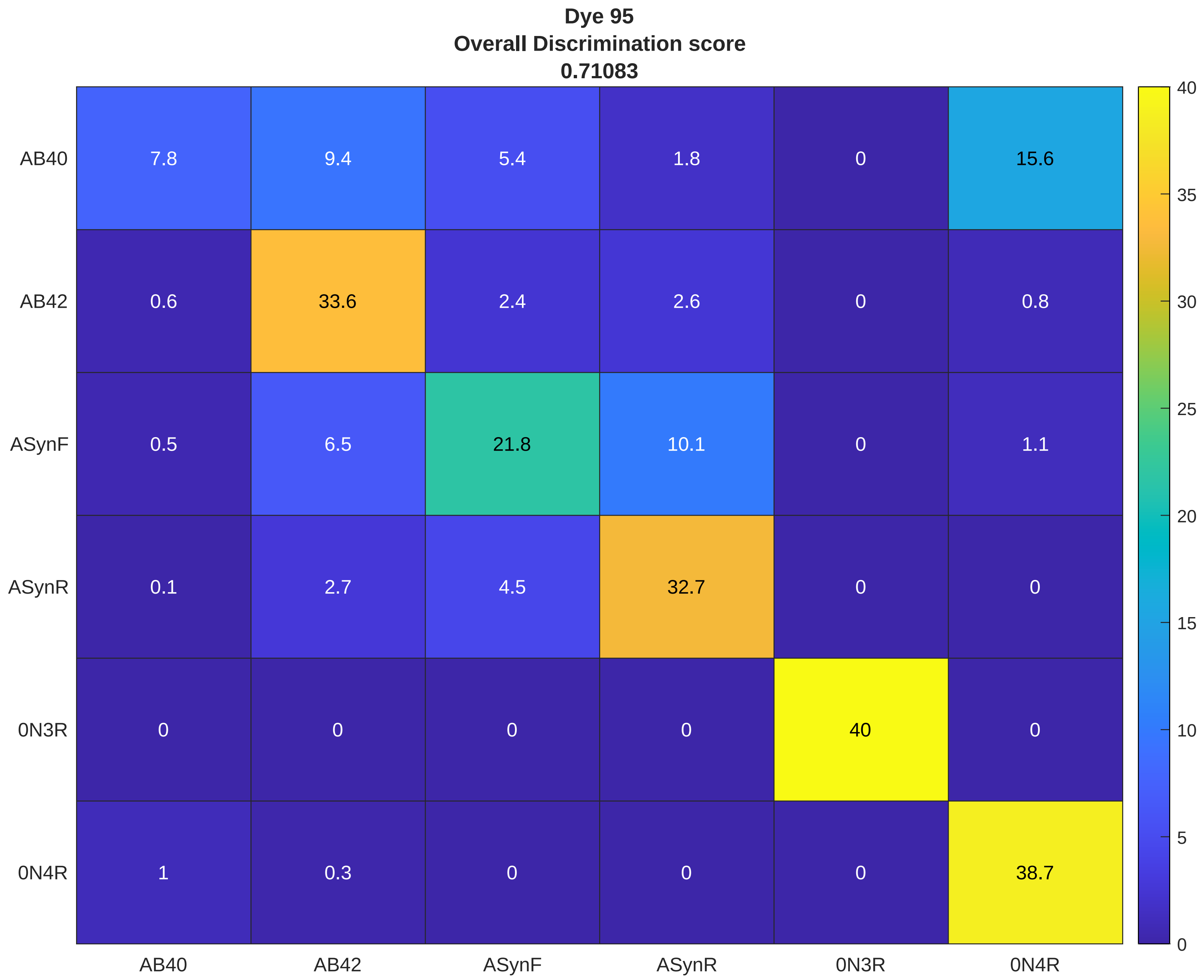
Dye 95
Overall Discrimination score
0.71083
AB40
AB42
ASynF
ASynR
0N3R
0N4R
7.8
9.4
5.4
1.8
0
15.6
0.6
33.6
2.4
2.6
0
0.8
0.5
6.5
21.8
10.1
0
1.1
0.1
2.7
4.5
32.7
0
0
0
0
0
0
40
0
1
0.3
0
0
0
38.7
AB40
AB42
ASynF
ASynR
0N3R
0N4R
40
35
30
25
20
15
10
5
0

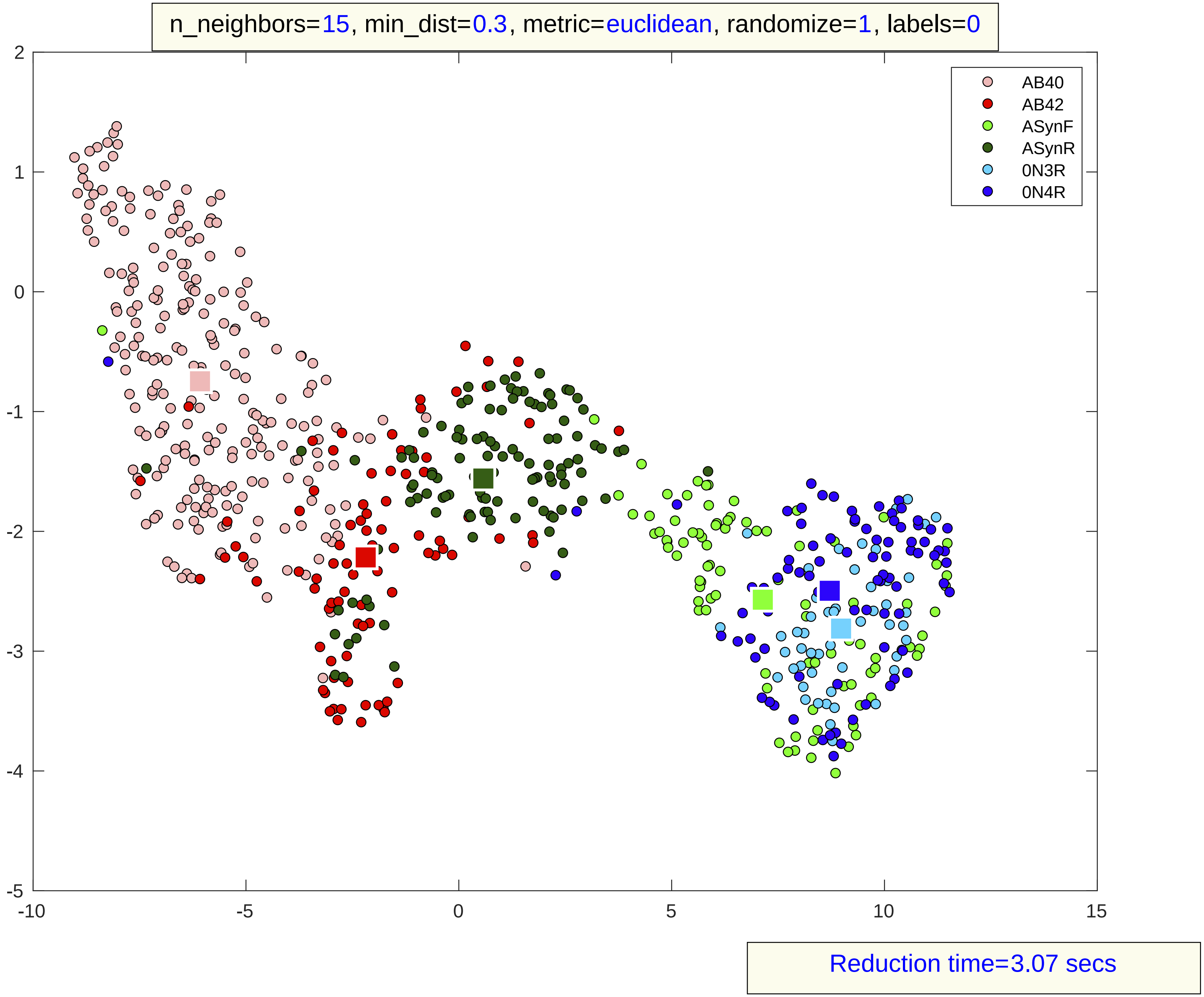
n_neighbors=15, min_dist=0.3, metric=euclidean, randomize=1, labels=0
AB40
AB42
ASynF
ASynR
0N3R
0N4R
2
1
0
-1
-2
-3
-4
-5
-10
-5
0
5
10
15
Reduction time=3.07 secs

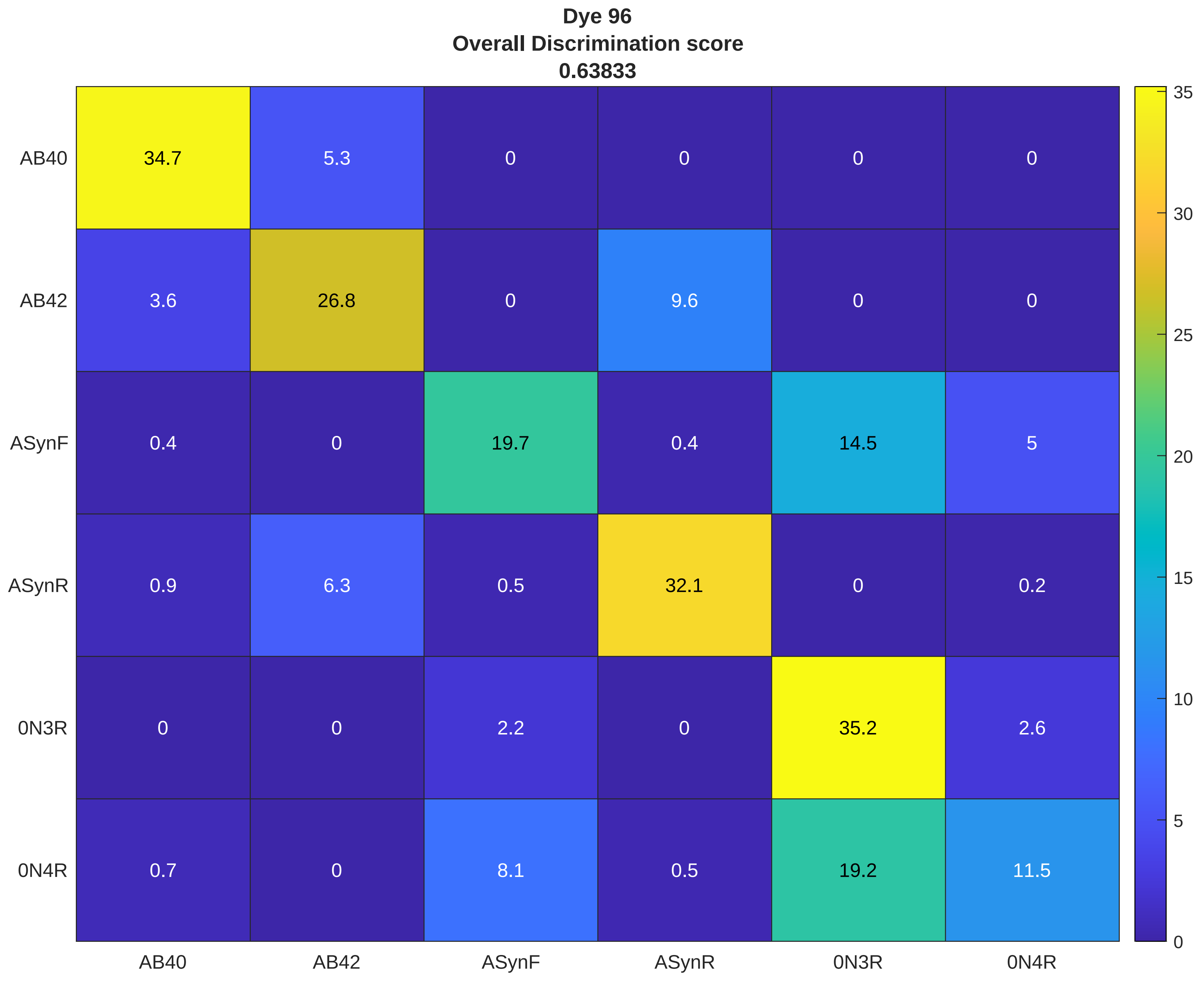

Dye 96
Overall Discrimination score
0.63833
AB40
AB42
ASynF
ASynR
0N3R
0N4R
34.7
5.3
0
0
0
0
3.6
26.8
0
9.6
0
0
0.4
0
19.7
0.4
14.5
5
0.9
6.3
0.5
32.1
0
0.2
0
0
2.2
0
35.2
2.6
0.7
0
8.1
0.5
19.2
11.5
AB40
AB42
ASynF
ASynR
0N3R
0N4R
35
30
25
20
15
10
5
0

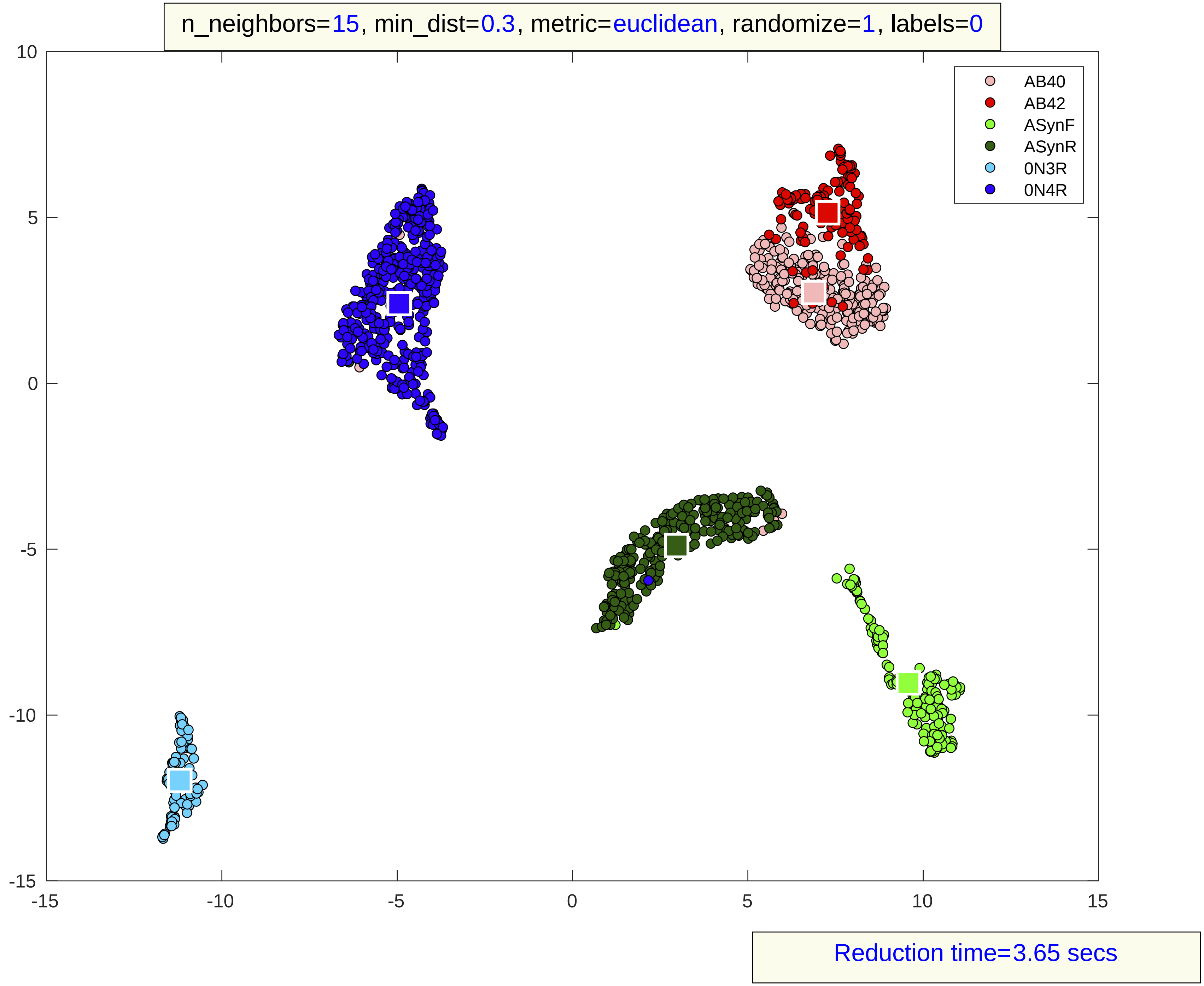
n_neighbors=15, min_dist=0.3, metric=euclidean, randomize=1, labels=0
AB40
AB42
ASynF
ASynR
0N3R
0N4R
10
5
0
-5
-10
-15
-15
-10
-5
0
5
10
15
Reduction time=3.65 secs

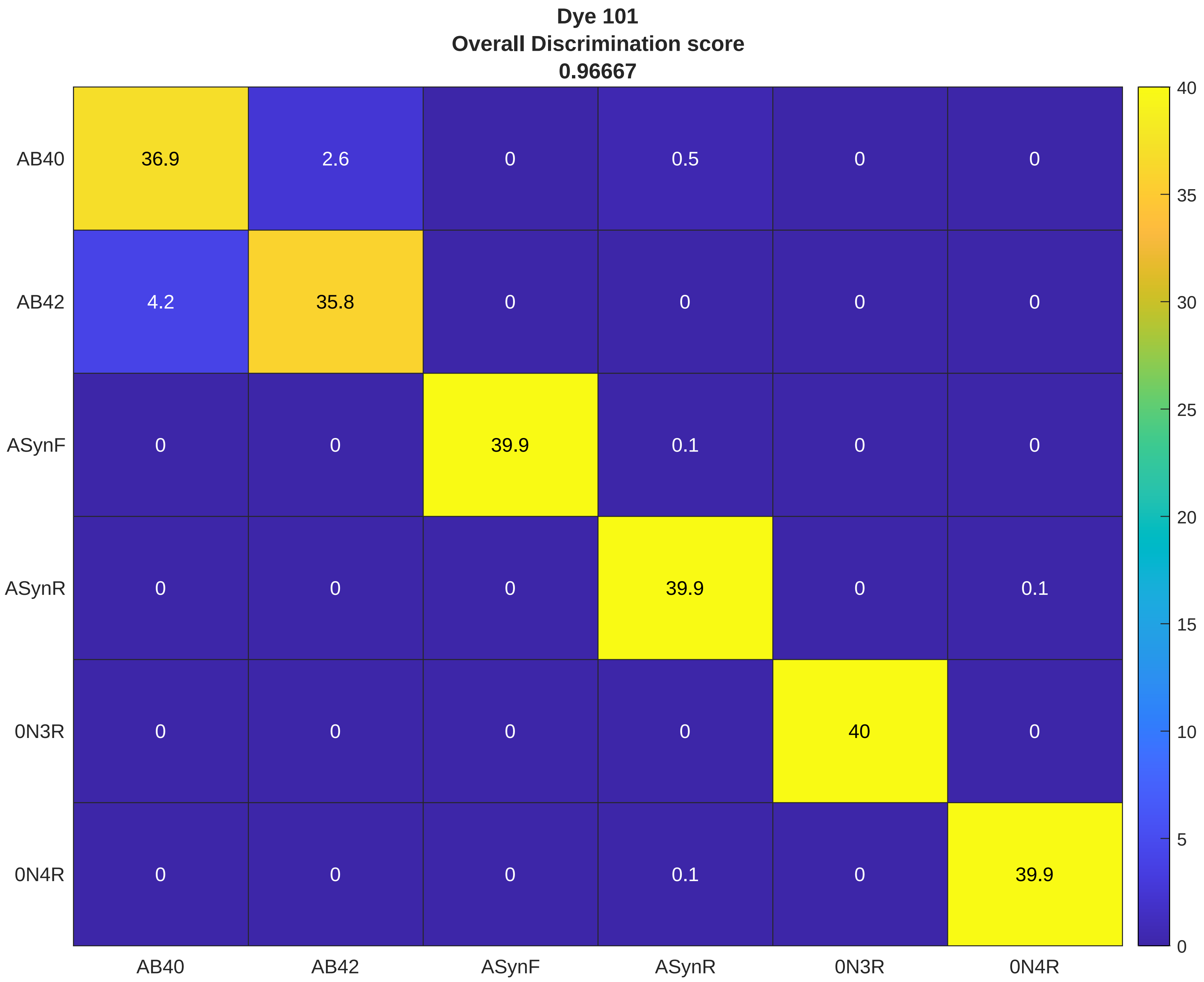
Dye 101
Overall Discrimination score
0.96667
AB40
AB42
ASynF
ASynR
0N3R
0N4R
36.9
2.6
0
0.5
0
0
4.2
35.8
0
0
0
0
0
0
39.9
0.1
0
0
0
0
0
39.9
0
0.1
0
0
0
0
40
0
0
0
0
0.1
0
39.9
AB40
AB42
ASynF
ASynR
0N3R
0N4R
40
35
30
25
20
15
10
5
0

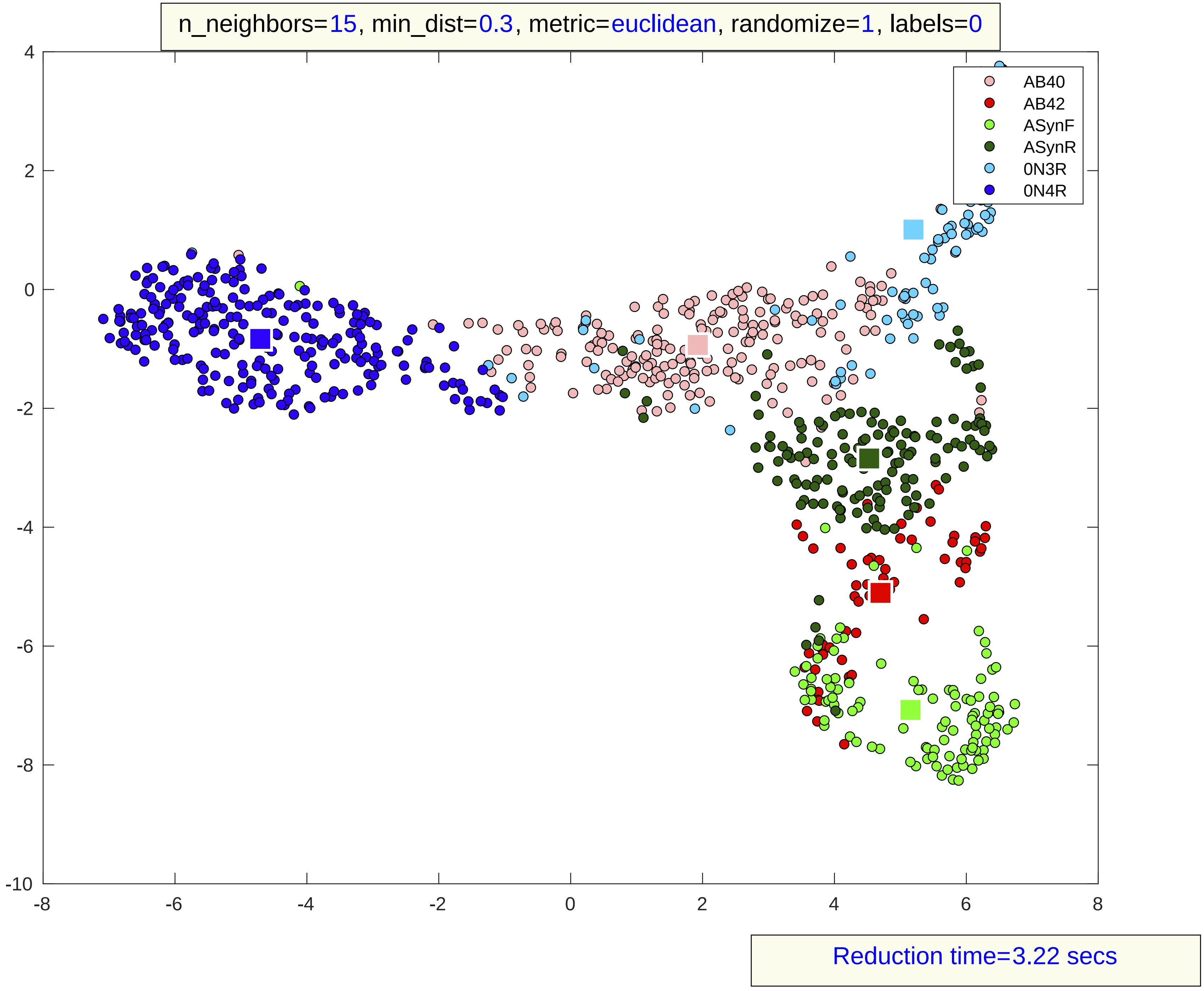
n_neighbors=15, min_dist=0.3, metric=euclidean, randomize=1, labels=0
AB40
AB42
ASynF
ASynR
0N3R
0N4R
4
2
0
-2
-4
-6
-8
-10
-8
-6
-4
-2
0
2
4
6
8
Reduction time=3.22 secs

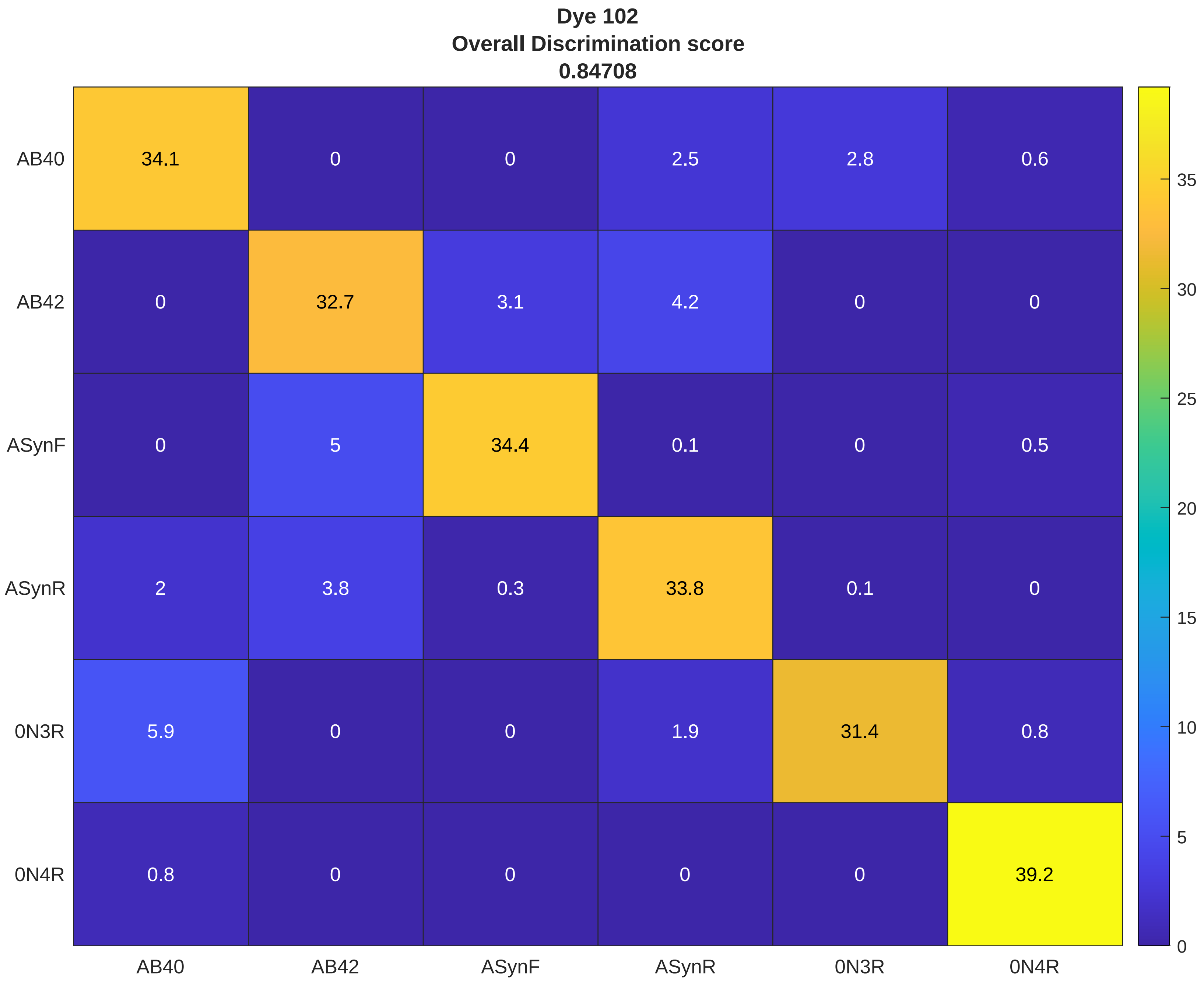
Dye 102
Overall Discrimination score
0.84708
AB40
AB42
ASynF
ASynR
0N3R
0N4R
34.1
0
0
2.5
2.8
0.6
0
32.7
3.1
4.2
0
0
0
5
34.4
0.1
0
0.5
2
3.8
0.3
33.8
0.1
0
5.9
0
0
1.9
31.4
0.8
0.8
0
0
0
0
39.2
AB40
AB42
ASynF
ASynR
0N3R
0N4R
35
30
25
20
15
10
5
0

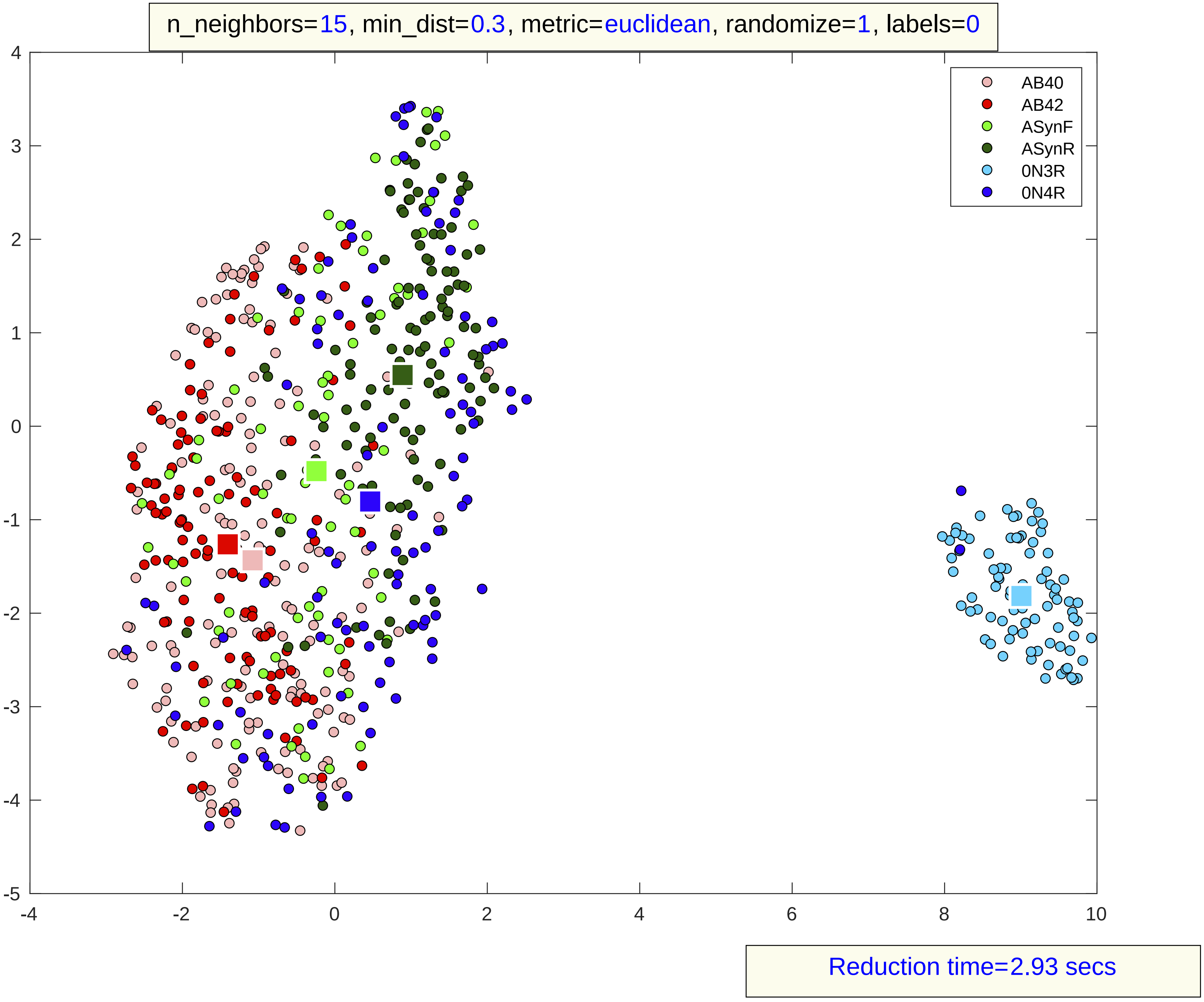
n_neighbors=15, min_dist=0.3, metric=euclidean, randomize=1, labels=0
AB40
AB42
ASynF
ASynR
0N3R
0N4R
Reduction time=2.93 secs

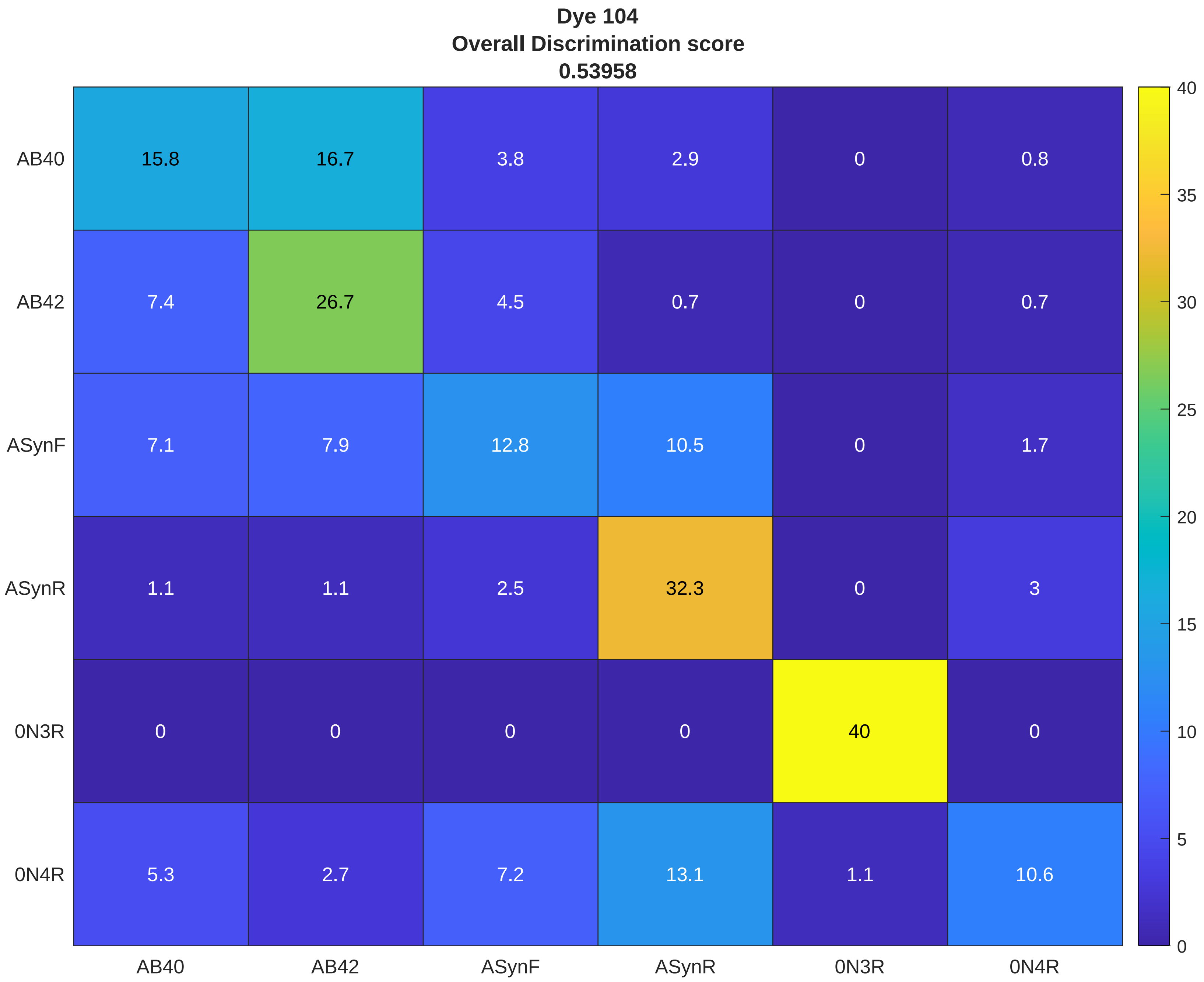
Dye 104
Overall Discrimination score
0.53958
AB40
AB42
ASynF
ASynR
0N3R
0N4R
15.8
16.7
3.8
2.9
0
0.8
7.4
26.7
4.5
0.7
0
0.7
7.1
7.9
12.8
10.5
0
1.7
1.1
1.1
2.5
32.3
0
3
0
0
0
0
40
0
5.3
2.7
7.2
13.1
1.1
10.6
AB40
AB42
ASynF
ASynR
0N3R
0N4R
40
35
30
25
20
15
10
5
0

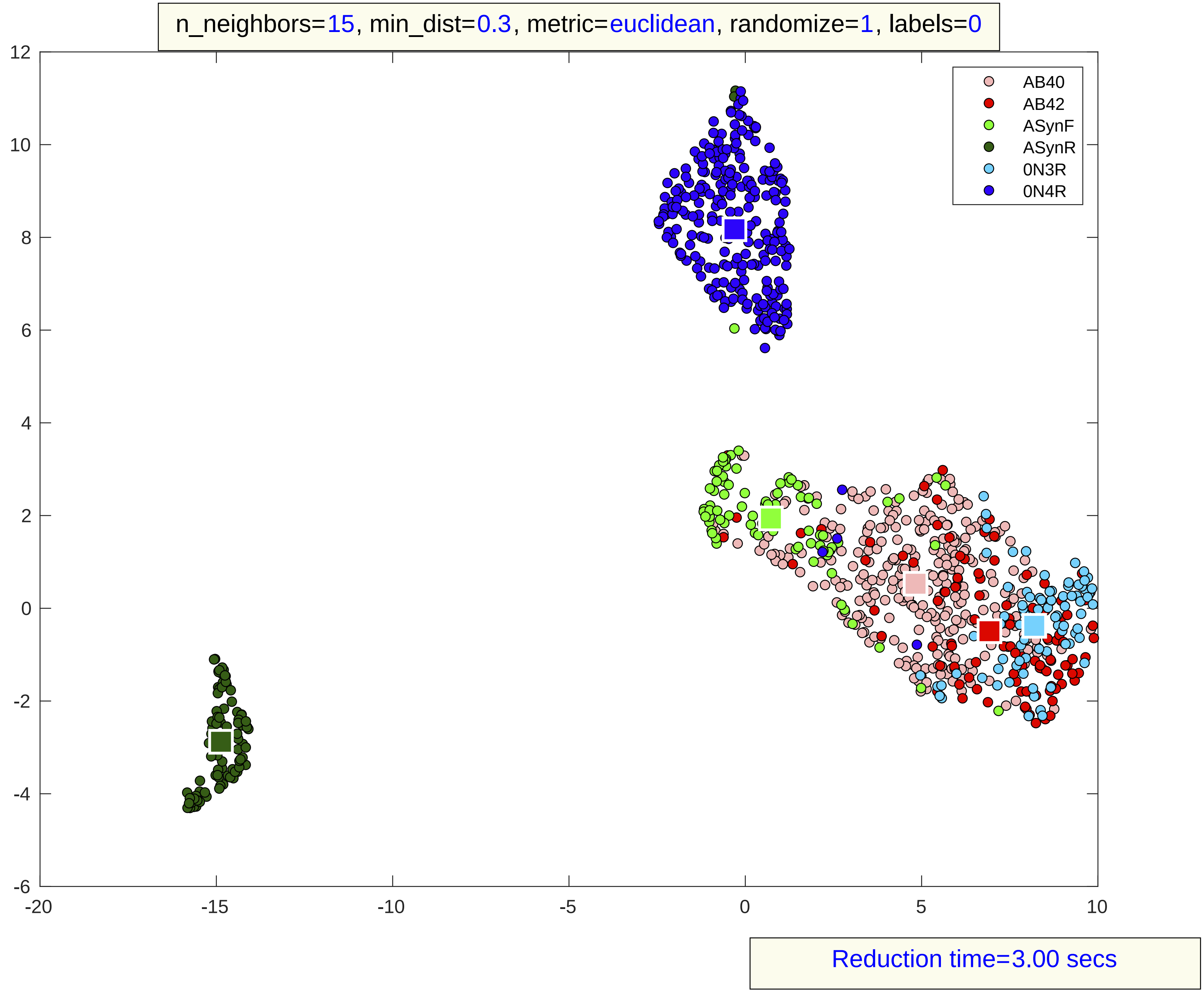

n_neighbors=15, min_dist=0.3, metric=euclidean, randomize=1, labels=0
AB40
AB42
ASynF
ASynR
0N3R
0N4R
12
10
8
6
4
2
0
-2
-4
-6
-20
-15
-10
-5
0
5
10
Reduction time=3.00 secs

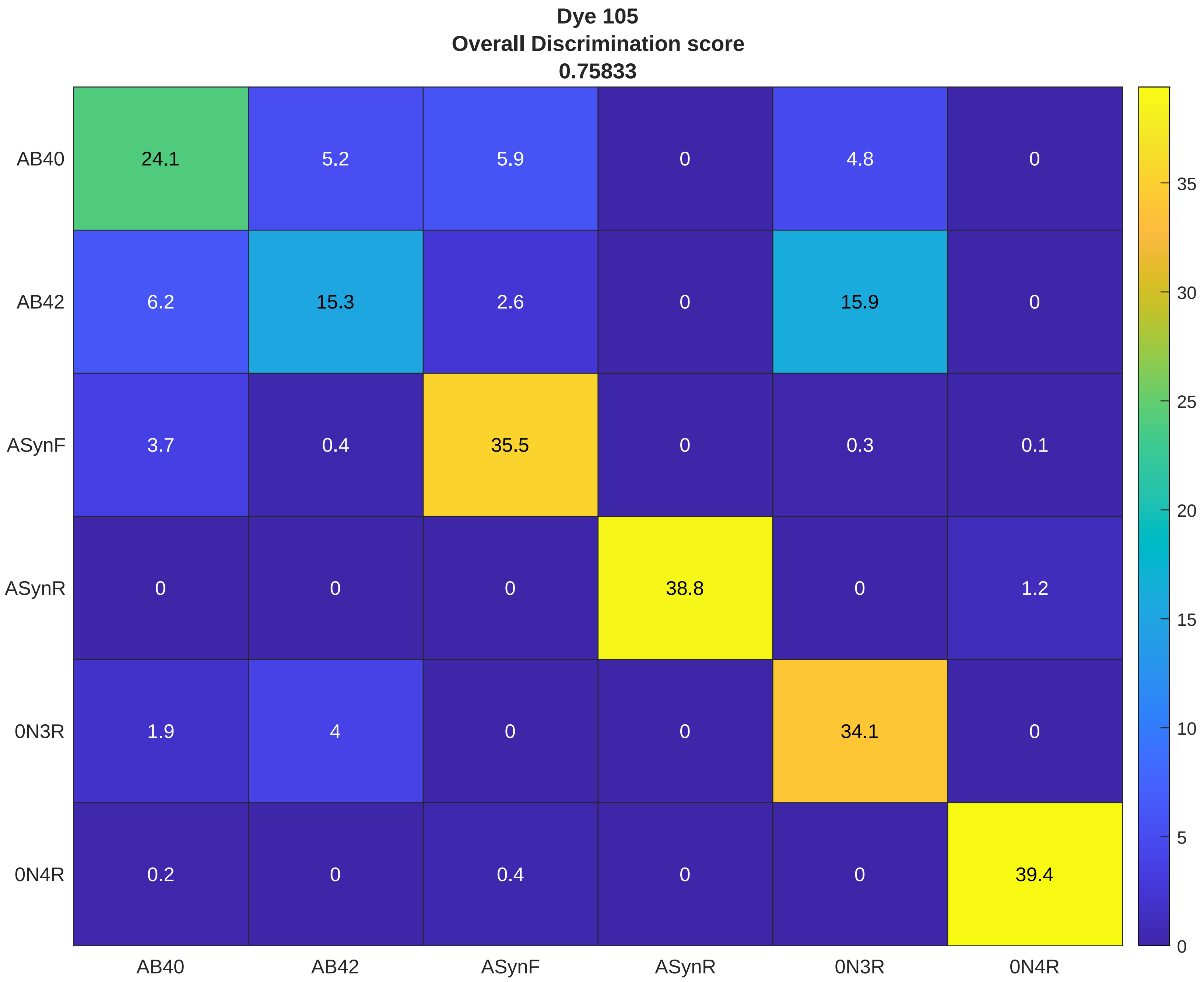
Dye 105
Overall Discrimination score
0.75833
AB40
AB42
ASynF
ASynR
0N3R
0N4R
24.1
5.2
5.9
0
4.8
0
6.2
15.3
2.6
0
15.9
0
3.7
0.4
35.5
0
0.3
0.1
0
0
0
38.8
0
1.2
1.9
4
0
0
34.1
0
0.2
0
0.4
0
0
39.4
AB40
AB42
ASynF
ASynR
0N3R
0N4R
35
30
25
20
15
10
5
0

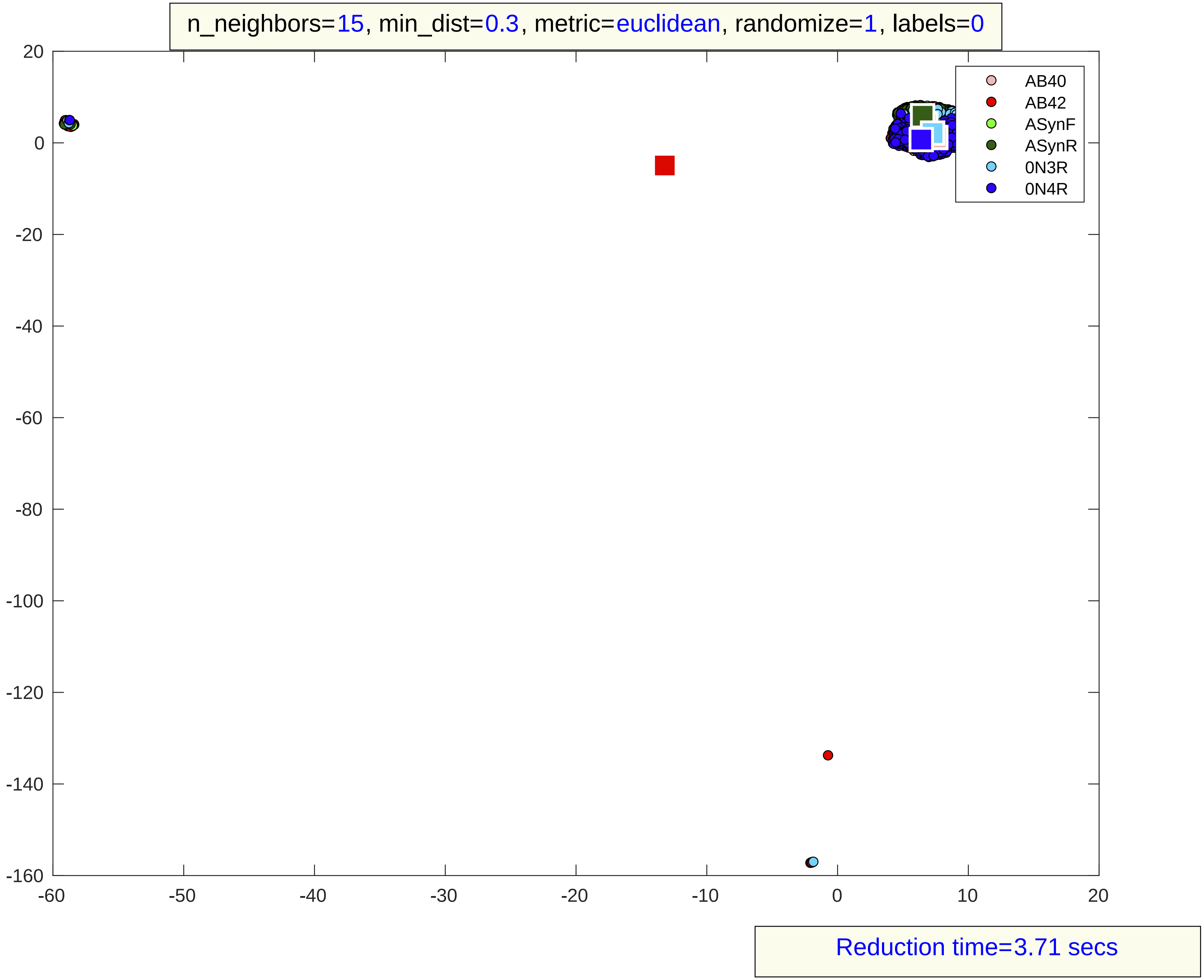

n_neighbors=15, min_dist=0.3, metric=euclidean, randomize=1, labels=0
20
0
-20
-40
-60
-80
-100
-120
-140
-160
-60
-50
-40
-30
-20
-10
0
10
20
AB40
AB42
ASynF
ASynR
0N3R
0N4R
Reduction time=3.71 secs

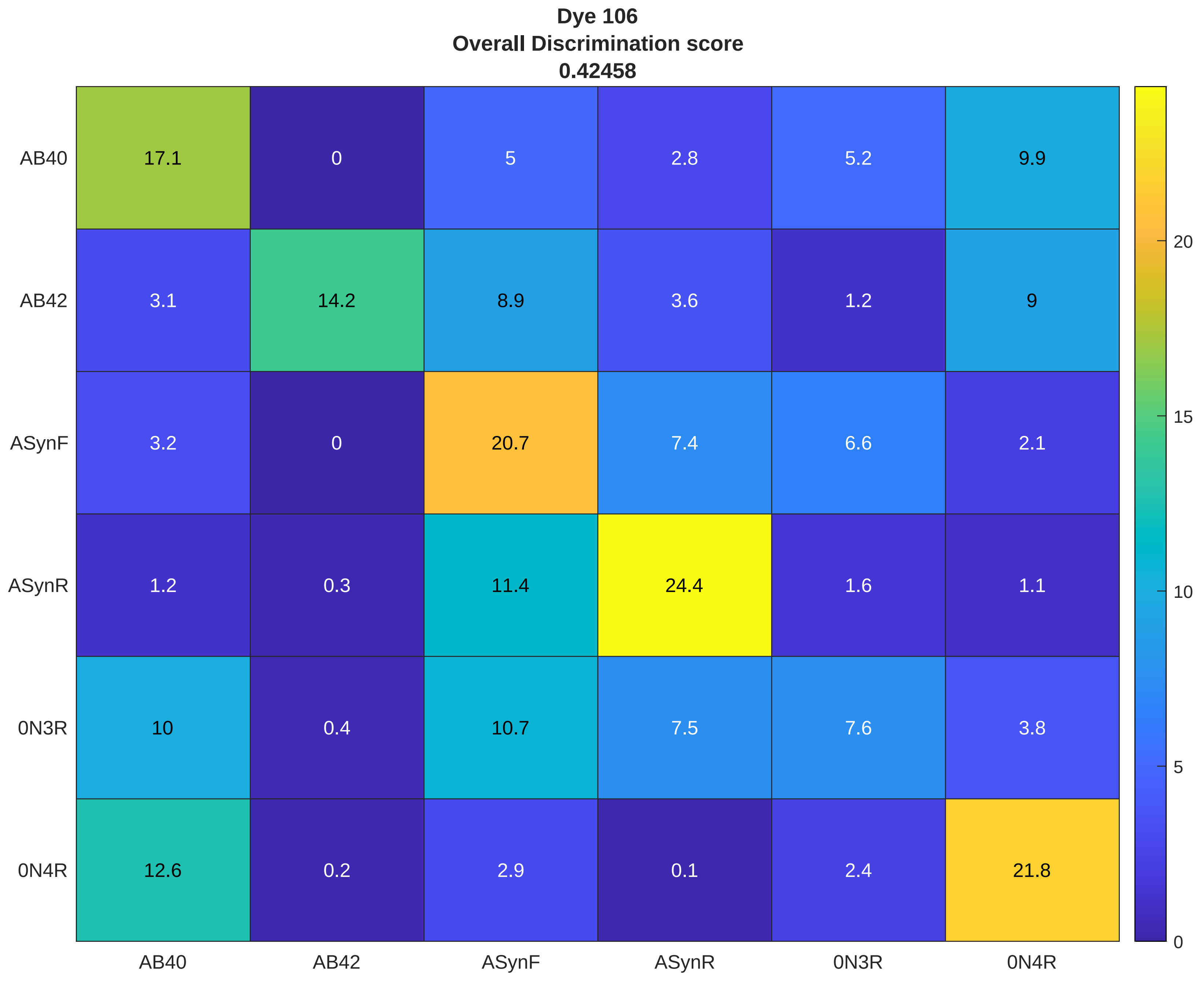
Dye 106
Overall Discrimination score
0.42458
AB40
AB42
ASynF
ASynR
0N3R
0N4R
17.1
0
5
2.8
5.2
9.9
3.1
14.2
8.9
3.6
1.2
9
3.2
0
20.7
7.4
6.6
2.1
1.2
0.3
11.4
24.4
1.6
1.1
10
0.4
10.7
7.5
7.6
3.8
12.6
0.2
2.9
0.1
2.4
21.8
AB40
AB42
ASynF
ASynR
0N3R
0N4R
0
5
10
15
20

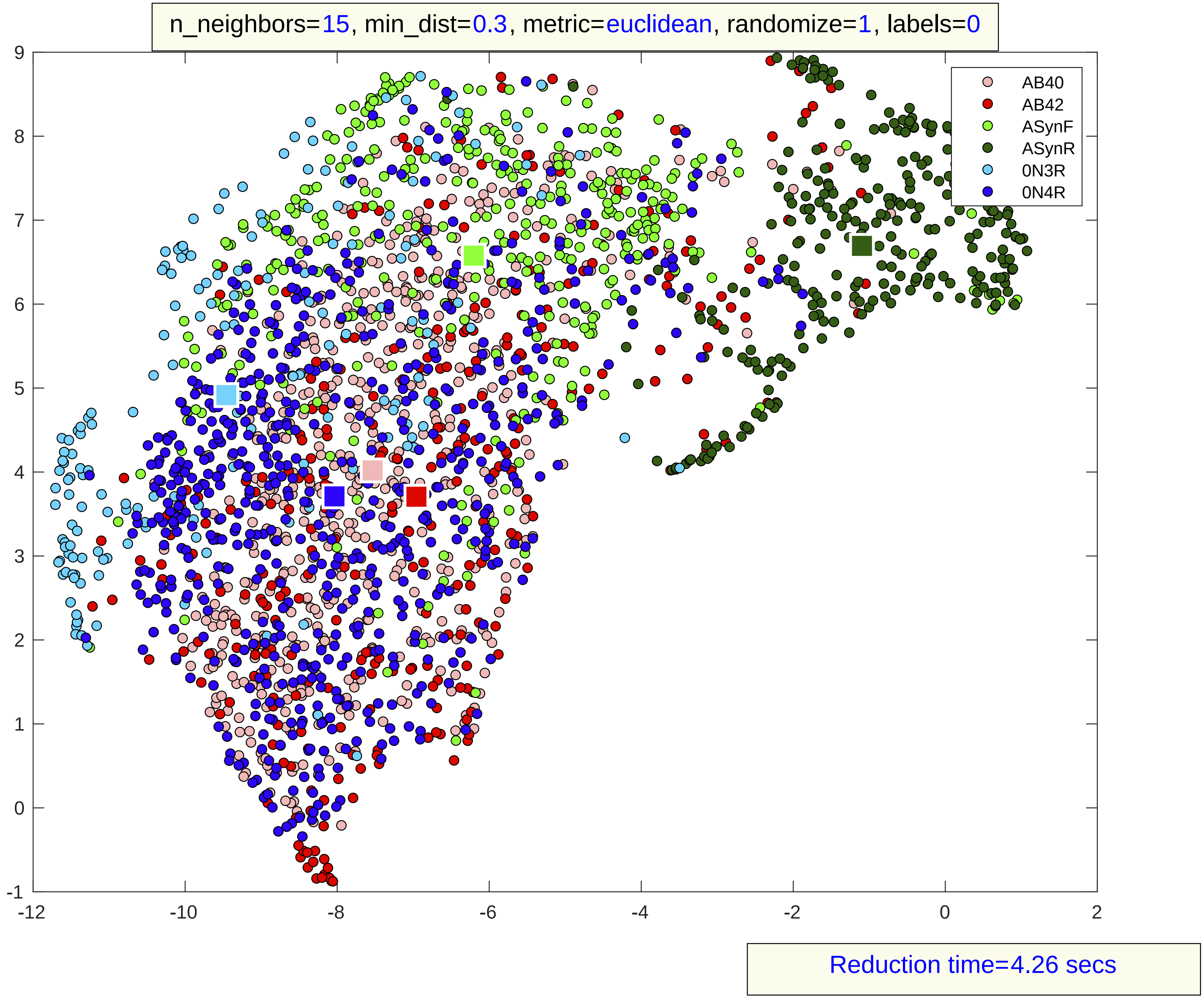
n_neighbors=15, min_dist=0.3, metric=euclidean, randomize=1, labels=0
9
8
7
6
5
4
3
2
1
0
-1
-12
-10
-8
-6
-4
-2
0
2
AB40
AB42
ASynF
ASynR
0N3R
0N4R
Reduction time=4.26 secs

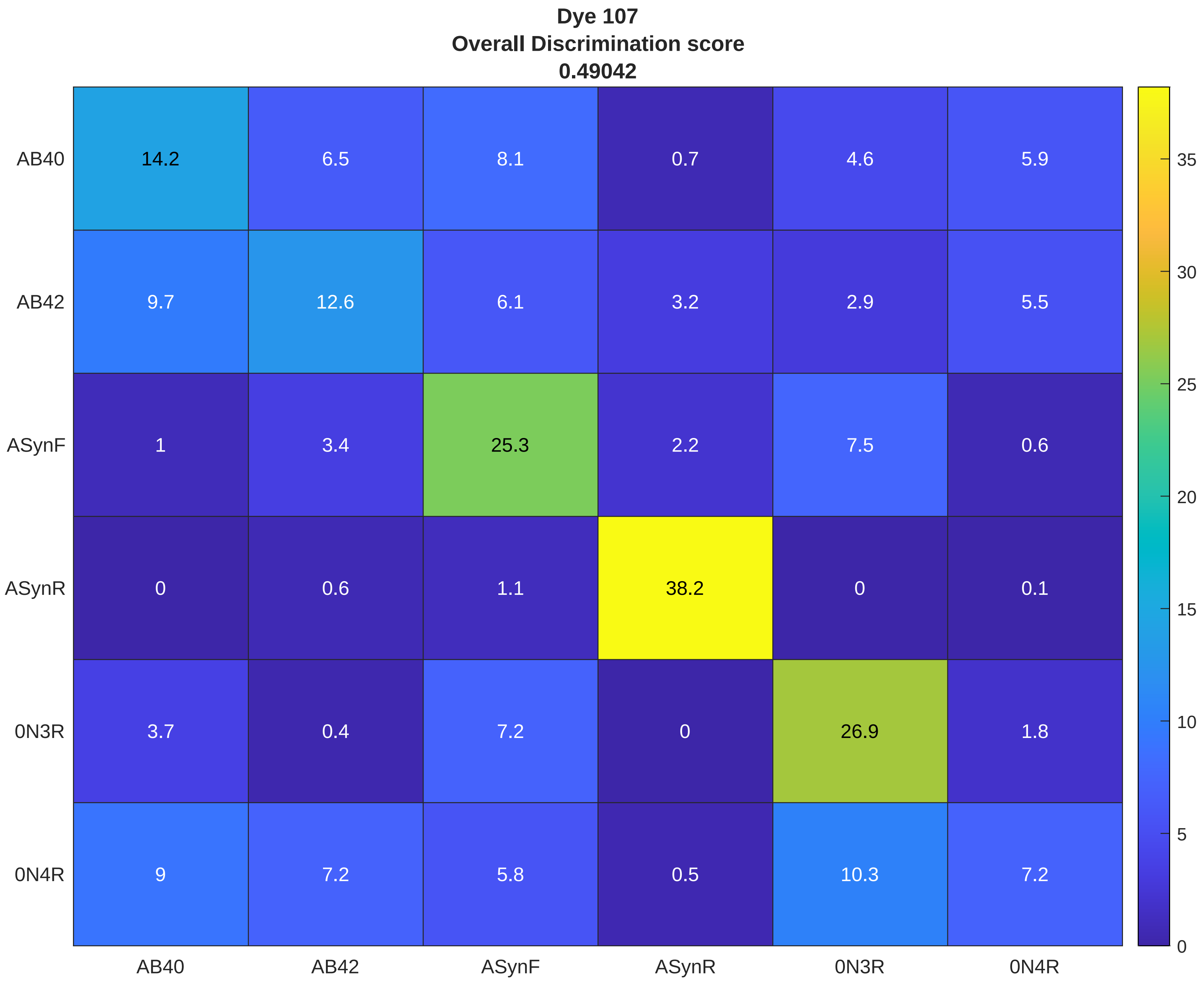

Dye 107
Overall Discrimination score
0.49042
AB40
AB42
ASynF
ASynR
0N3R
0N4R
14.2
6.5
8.1
0.7
4.6
5.9
9.7
12.6
6.1
3.2
2.9
5.5
1
3.4
25.3
2.2
7.5
0.6
0
0.6
1.1
38.2
0
0.1
3.7
0.4
7.2
0
26.9
1.8
9
7.2
5.8
0.5
10.3
7.2
AB40
AB42
ASynF
ASynR
0N3R
0N4R
35
30
25
20
15
10
5
0

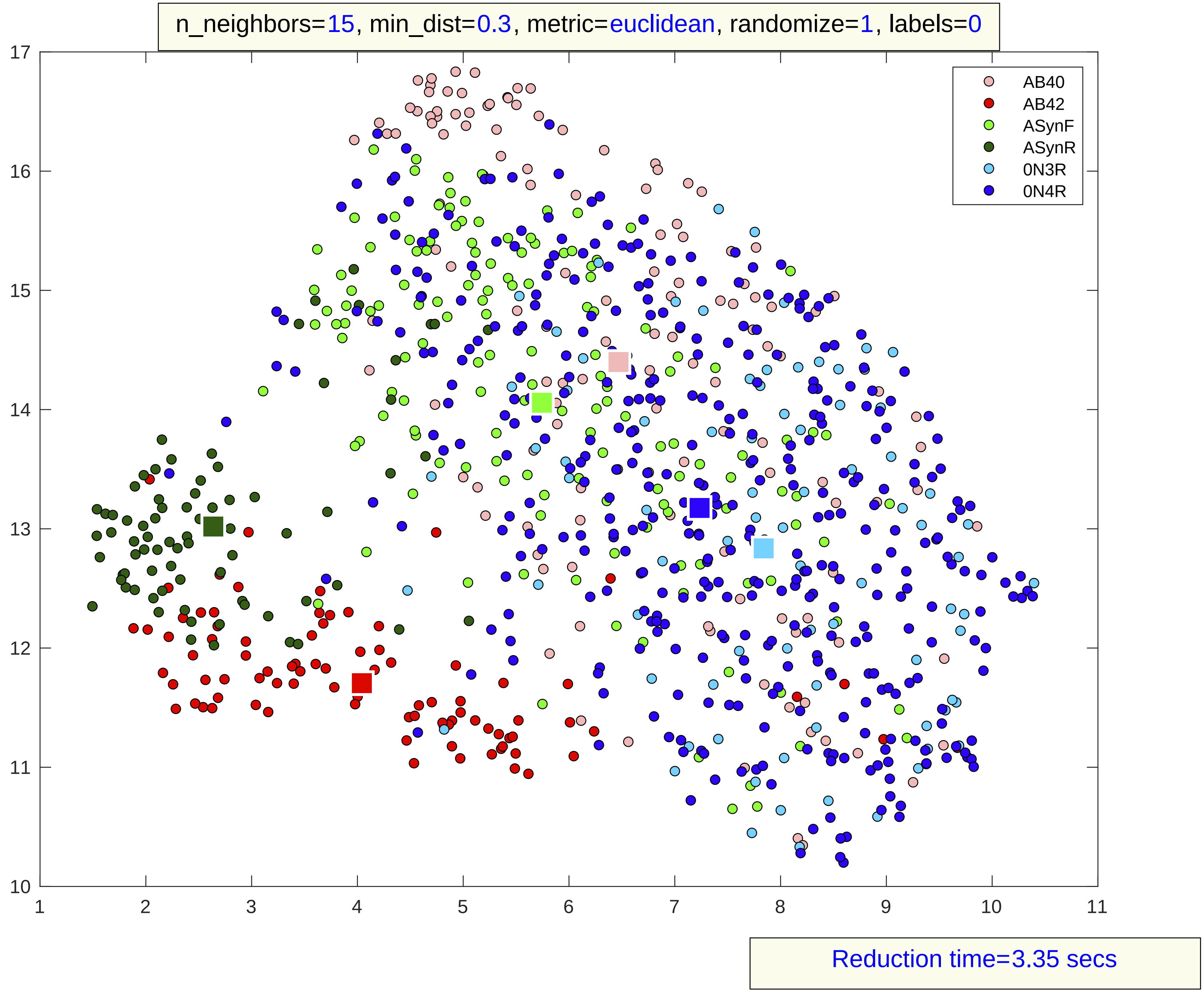

n_neighbors=15, min_dist=0.3, metric=euclidean, randomize=1, labels=0
AB40
AB42
ASynF
ASynR
0N3R
0N4R
17
16
15
14
13
12
11
10
1
2
3
4
5
6
7
8
9
10
11
Reduction time=3.35 secs

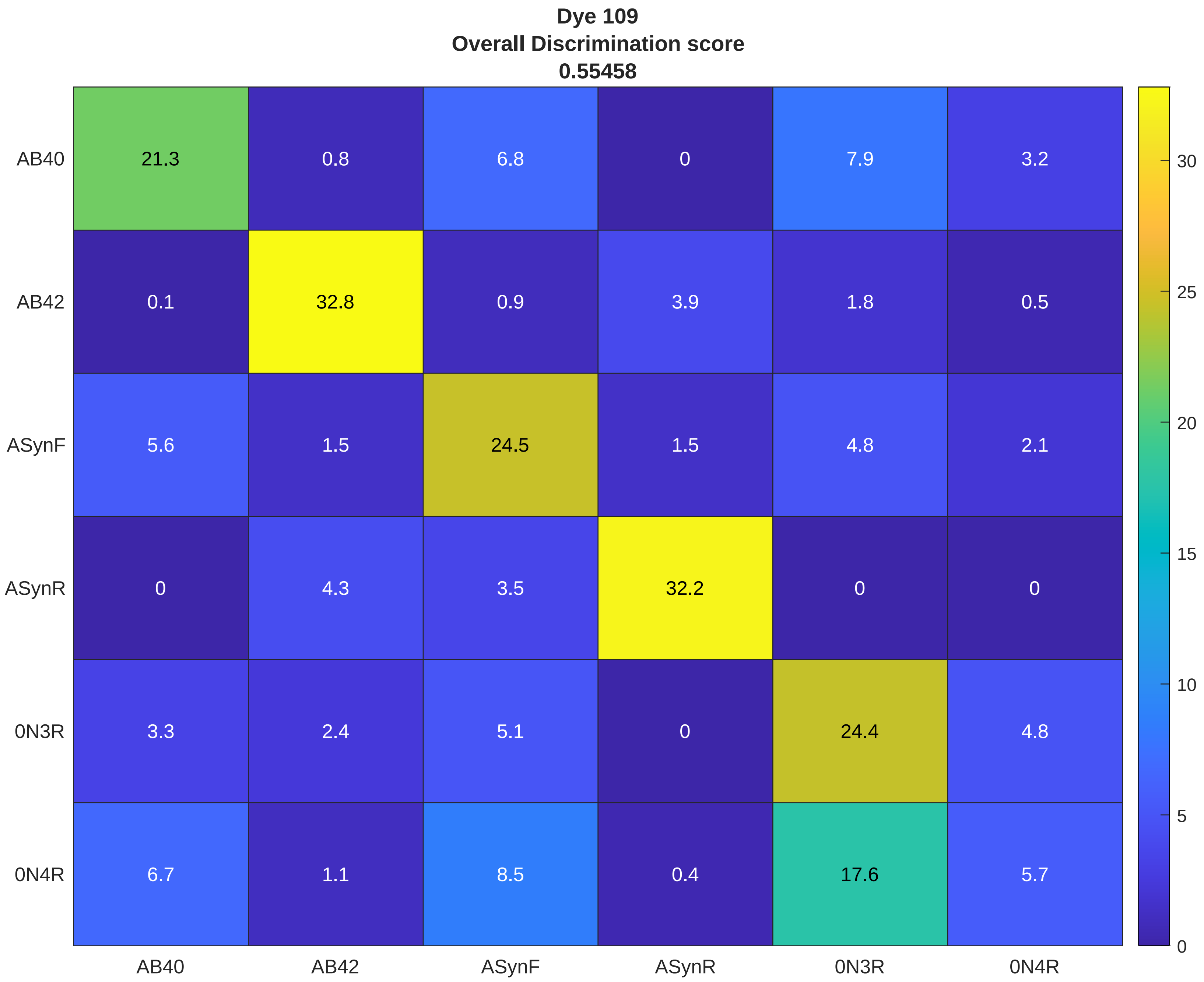
Dye 109
Overall Discrimination score
0.55458
AB40
AB42
ASynF
ASynR
0N3R
0N4R
21.3 0.8 6.8 0 7.9 3.2
0.1 32.8 0.9 3.9 1.8 0.5
5.6 1.5 24.5 1.5 4.8 2.1
0 4.3 3.5 32.2 0 0
3.3 2.4 5.1 0 24.4 4.8
6.7 1.1 8.5 0.4 17.6 5.7
AB40
AB42
ASynF
ASynR
0N3R
0N4R
0
5
10
15
20
25
30

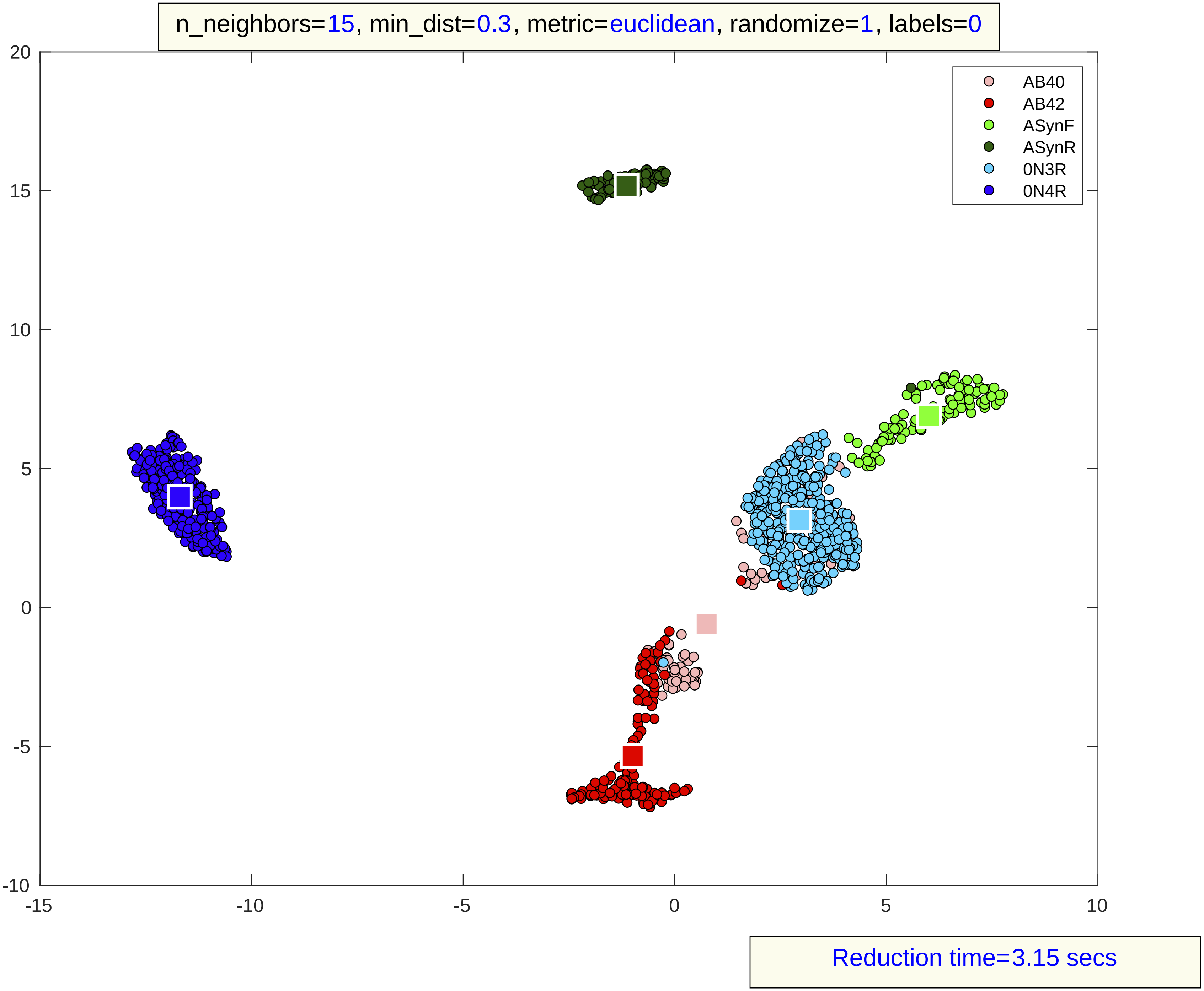

n_neighbors=15, min_dist=0.3, metric=euclidean, randomize=1, labels=0
AB40
AB42
ASynF
ASynR
0N3R
0N4R
20
15
10
5
0
-5
-10
-15
-10
-5
0
5
10
Reduction time=3.15 secs

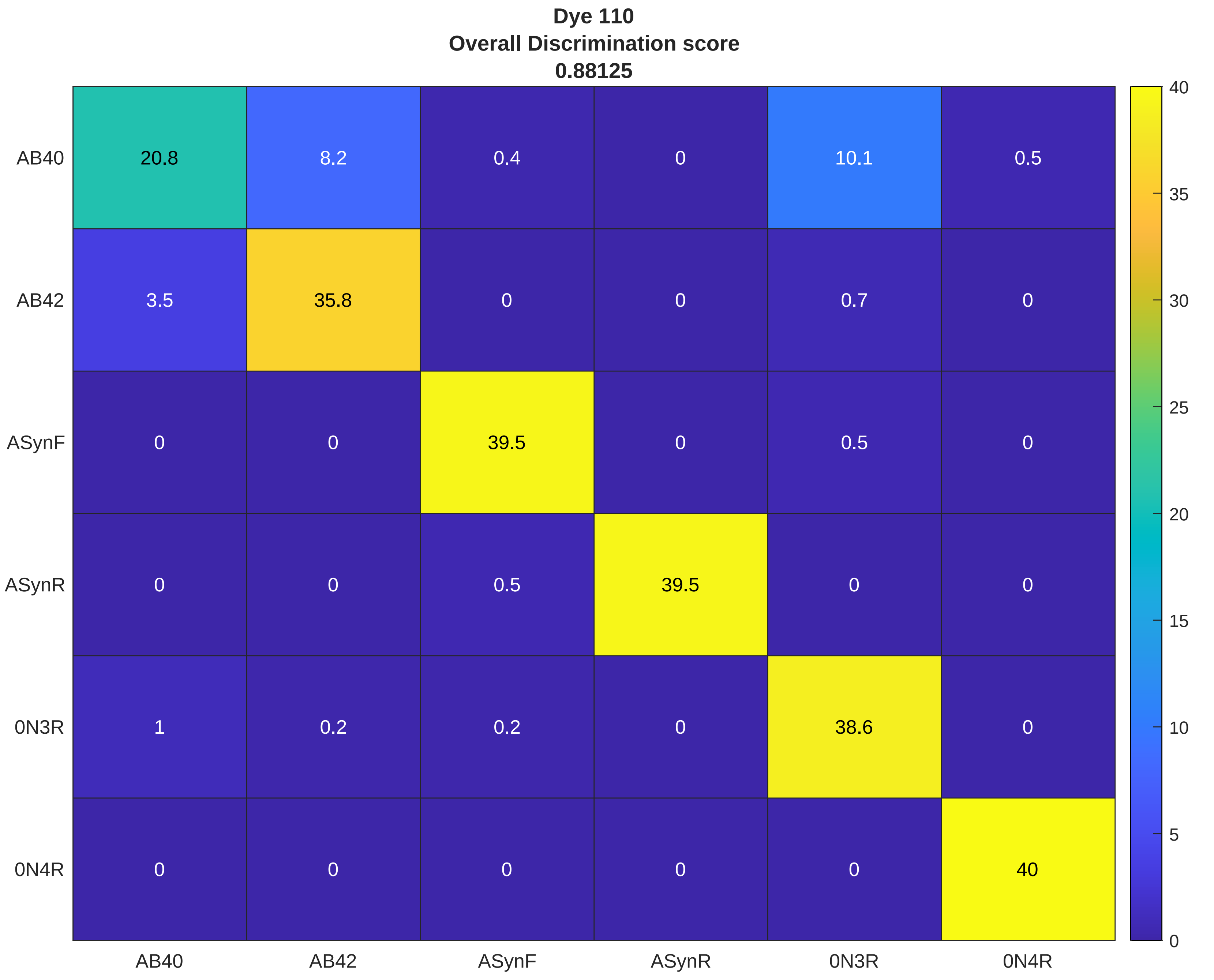
Dye 110
Overall Discrimination score
0.88125
AB40
AB42
ASynF
ASynR
0N3R
0N4R
20.8 8.2 0.4 0 10.1 0.5
3.5 35.8 0 0 0.7 0
0 0 39.5 0 0.5 0
0 0 0.5 39.5 0 0
1 0.2 0.2 0 38.6 0
0 0 0 0 0 40
AB40
AB42
ASynF
ASynR
0N3R
0N4R
40
35
30
25
20
15
10
5
0

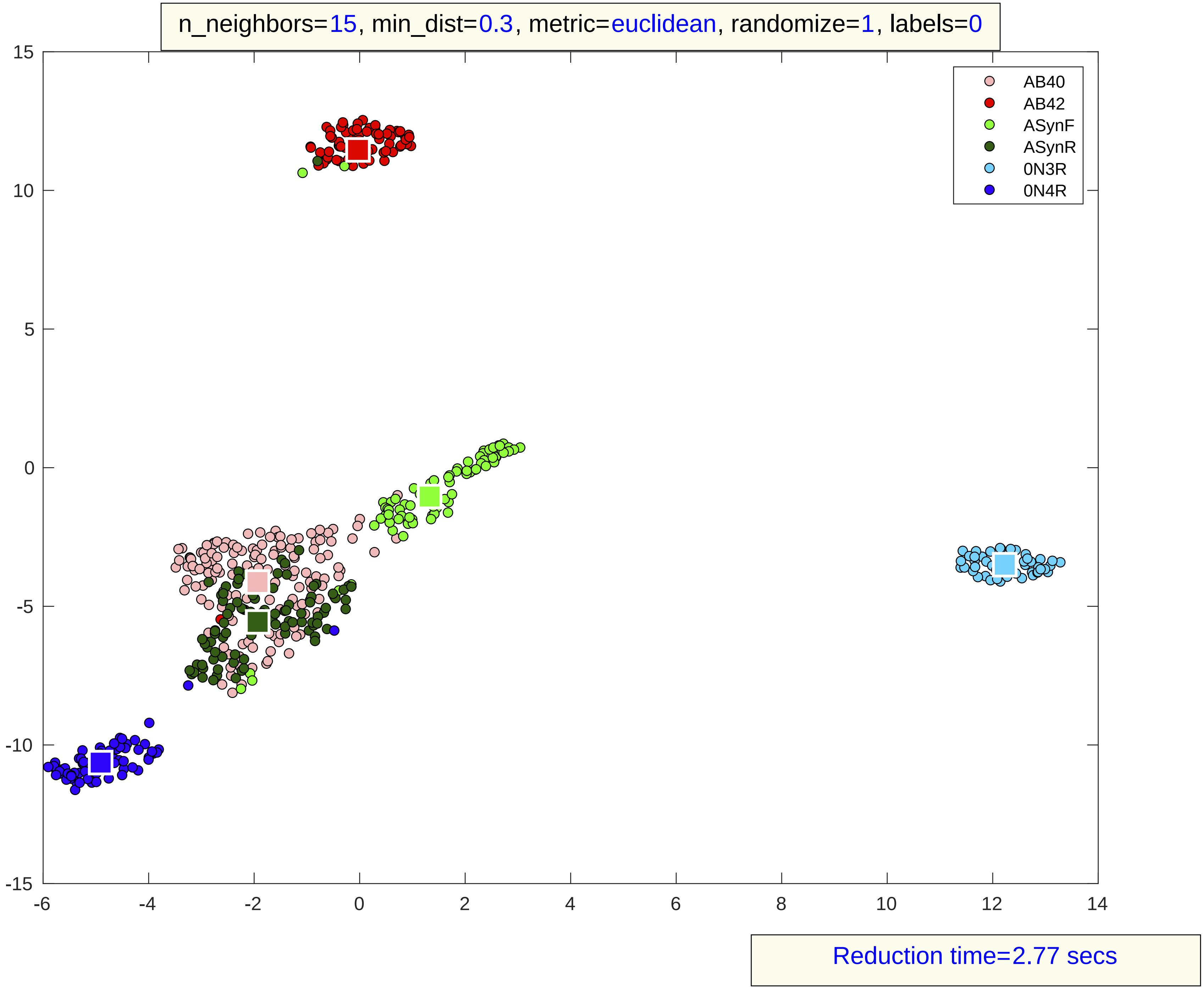
n_neighbors=15, min_dist=0.3, metric=euclidean, randomize=1, labels=0
AB40
AB42
ASynF
ASynR
0N3R
0N4R
15
10
5
0
-5
-10
-15
-6
-4
-2
0
2
4
6
8
10
12
14
Reduction time=2.77 secs

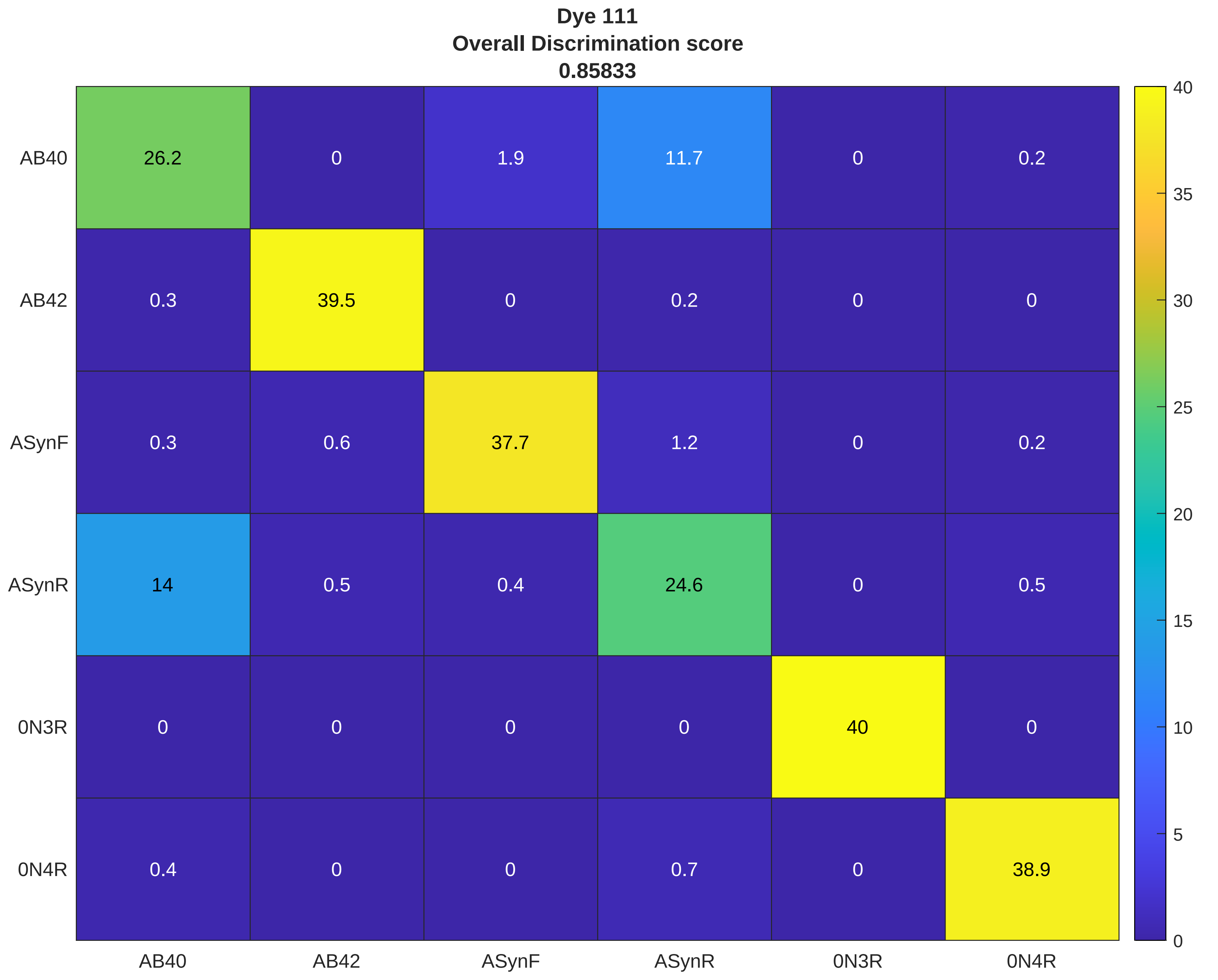

Dye 111
Overall Discrimination score
0.85833
AB40
AB42
ASynF
ASynR
0N3R
0N4R
26.2
0
1.9
11.7
0
0.2
0.3
39.5
0
0.2
0
0
0.3
0.6
37.7
1.2
0
0.2
14
0.5
0.4
24.6
0
0.5
0
0
0
0
40
0
0.4
0
0
0.7
0
38.9
AB40
AB42
ASynF
ASynR
0N3R
0N4R
40
35
30
25
20
15
10
5
0

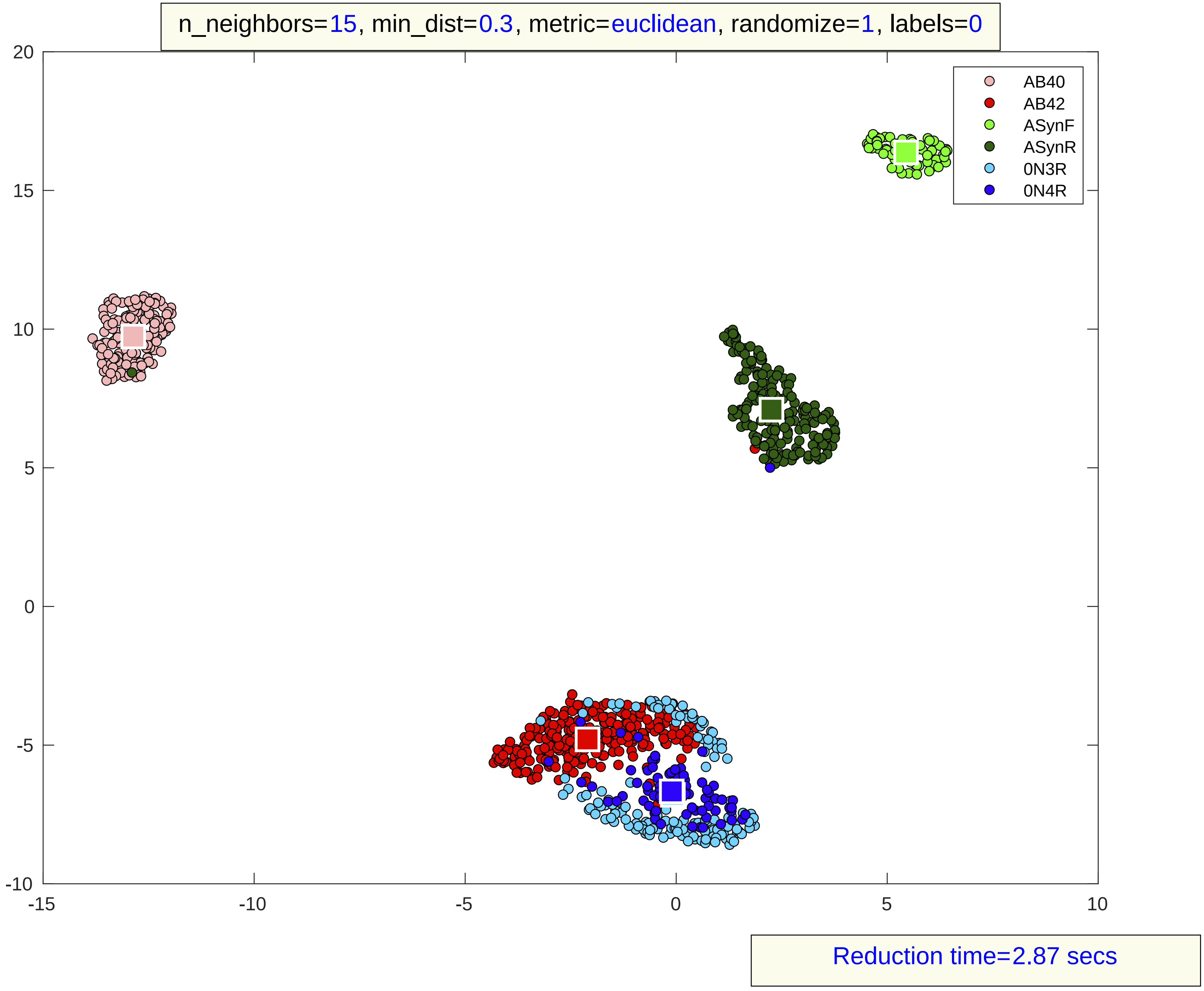
n_neighbors=15, min_dist=0.3, metric=euclidean, randomize=1, labels=0
AB40
AB42
ASynF
ASynR
0N3R
0N4R
20
15
10
5
0
-5
-10
-15
-10
-5
0
5
10
Reduction time=2.87 secs

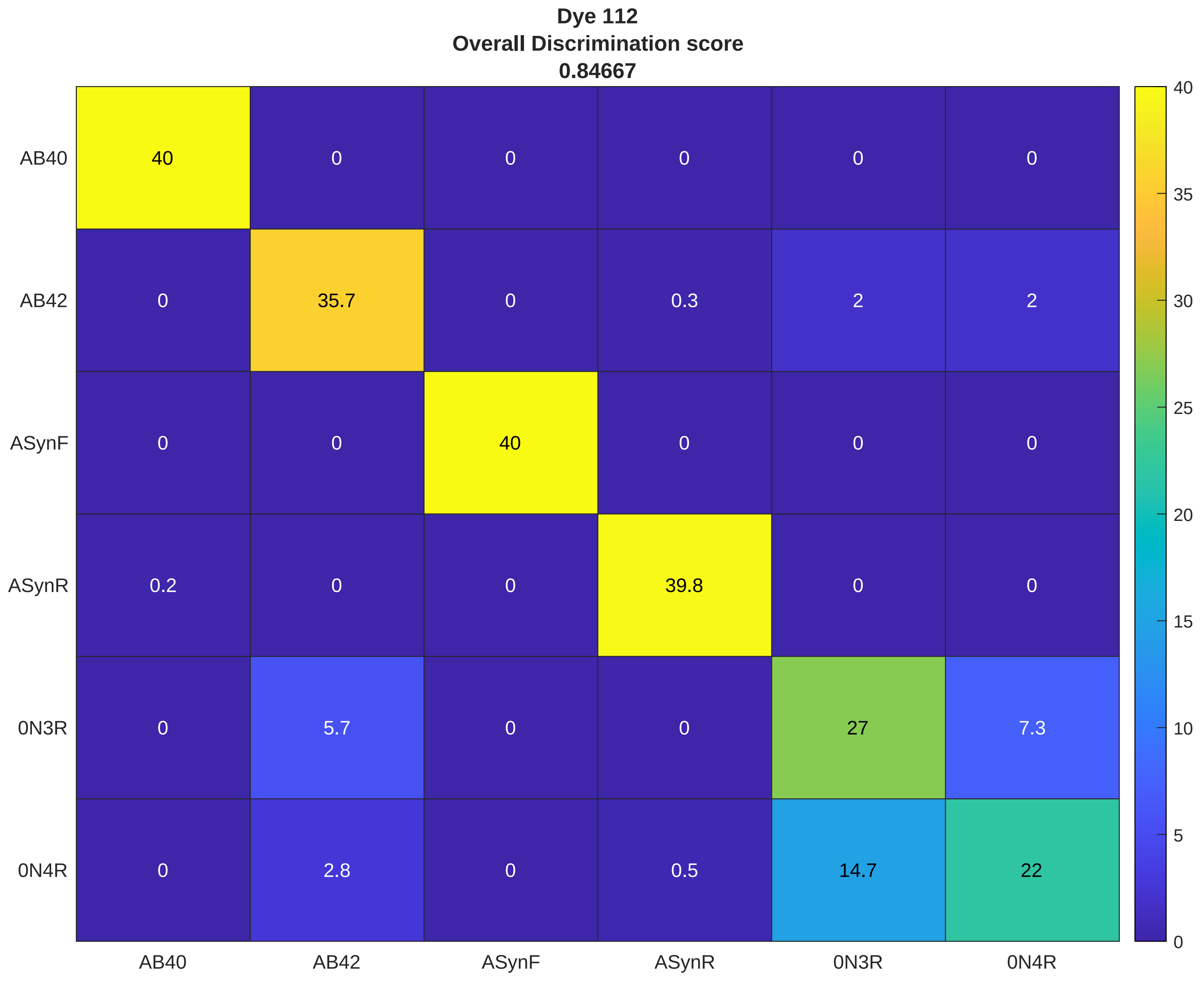
Dye 112
Overall Discrimination score
0.84667
AB40
AB42
ASynF
ASynR
0N3R
0N4R
40 0 0 0 0 0
0 35.7 0 0.3 2 2
0 0 40 0 0 0
0.2 0 0 39.8 0 0
0 5.7 0 0 27 7.3
0 2.8 0 0.5 14.7 22
AB40
AB42
ASynF
ASynR
0N3R
0N4R
40
35
30
25
20
15
10
5
0

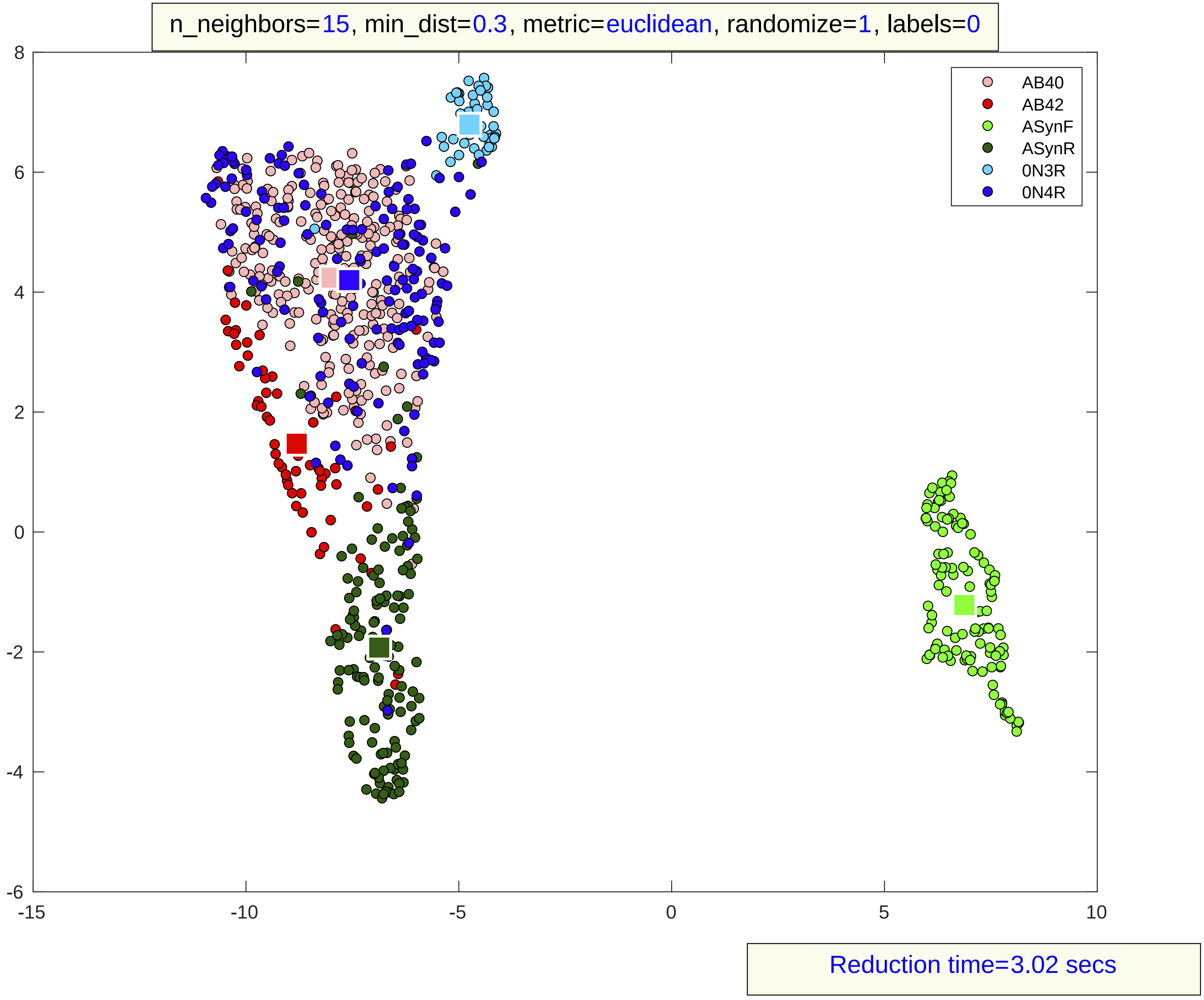
n_neighbors=15, min_dist=0.3, metric=euclidean, randomize=1, labels=0
AB40
AB42
ASynF
ASynR
0N3R
0N4R
8
6
4
2
0
-2
-4
-6
-15
-10
-5
0
5
10
Reduction time=3.02 secs

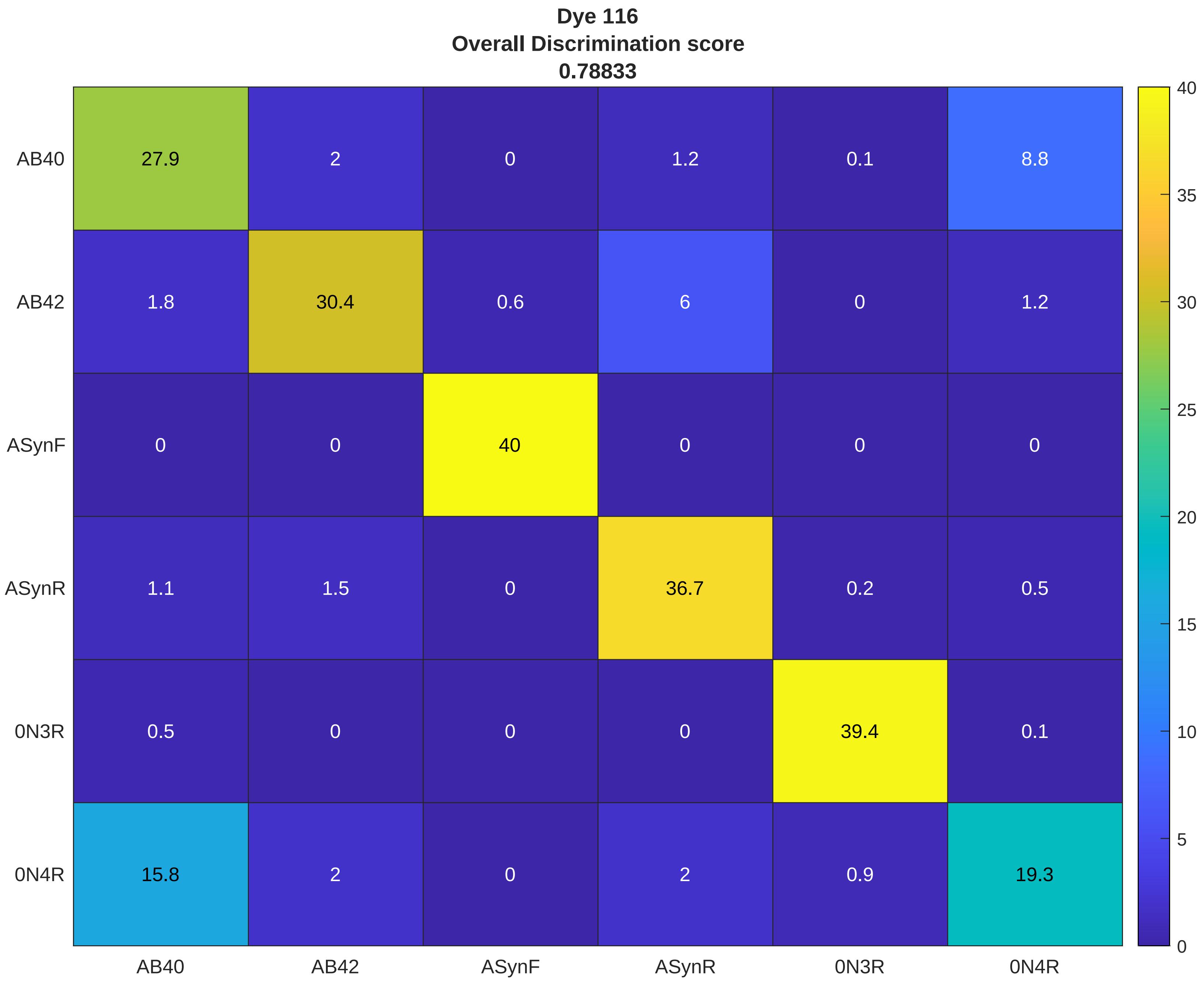
Dye 116
Overall Discrimination score
0.78833
AB40
AB42
ASynF
ASynR
0N3R
0N4R
27.9
2
0
1.2
0.1
8.8
1.8
30.4
0.6
6
0
1.2
0
0
40
0
0
0
1.1
1.5
0
36.7
0.2
0.5
0.5
0
0
0
39.4
0.1
15.8
2
0
2
0.9
19.3
AB40
AB42
ASynF
ASynR
0N3R
0N4R
40
35
30
25
20
15
10
5
0

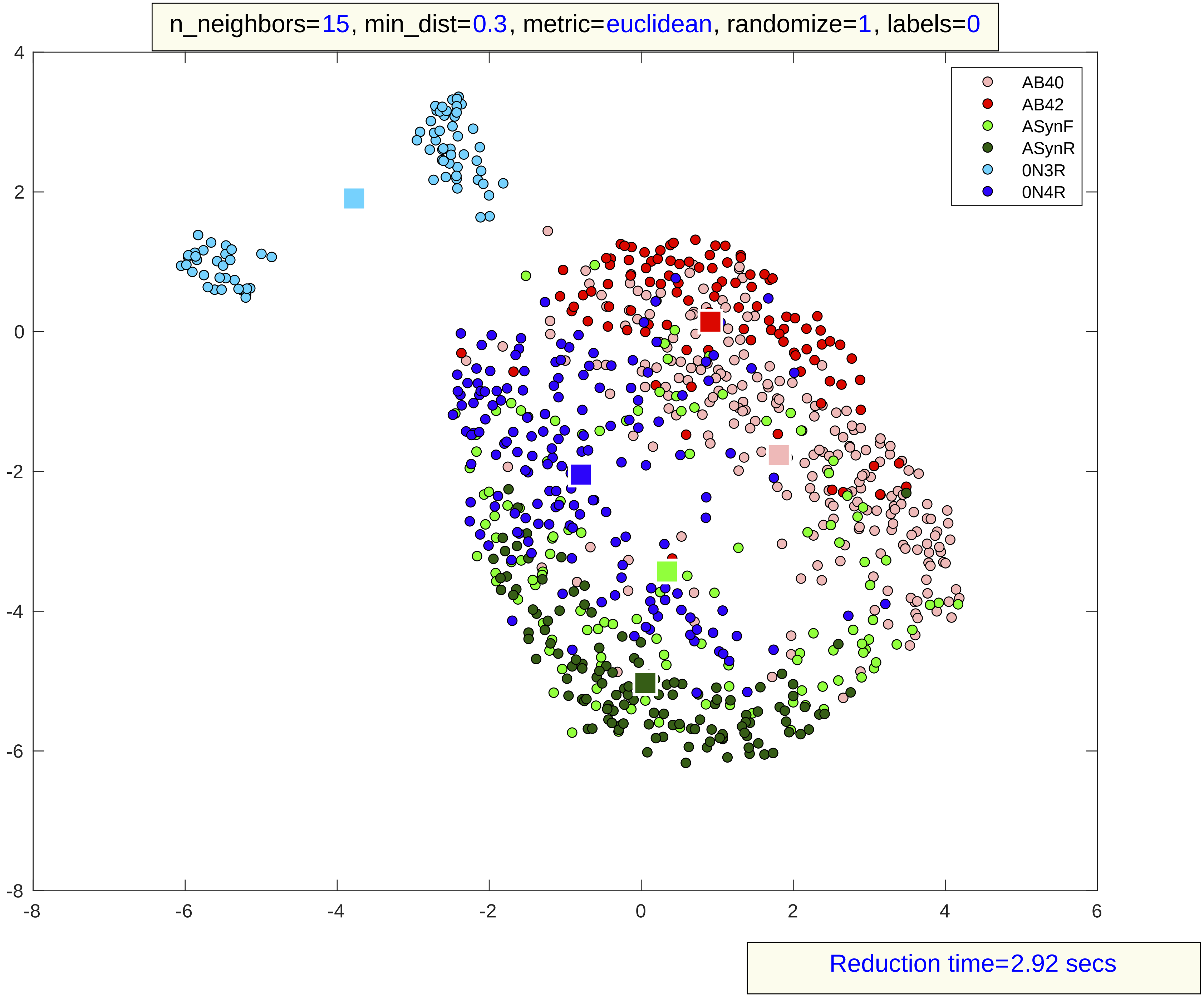

n_neighbors=15, min_dist=0.3, metric=euclidean, randomize=1, labels=0
AB40
AB42
ASynF
ASynR
0N3R
0N4R
4
2
0
-2
-4
-6
-8
-8
-6
-4
-2
0
2
4
6
Reduction time=2.92 secs

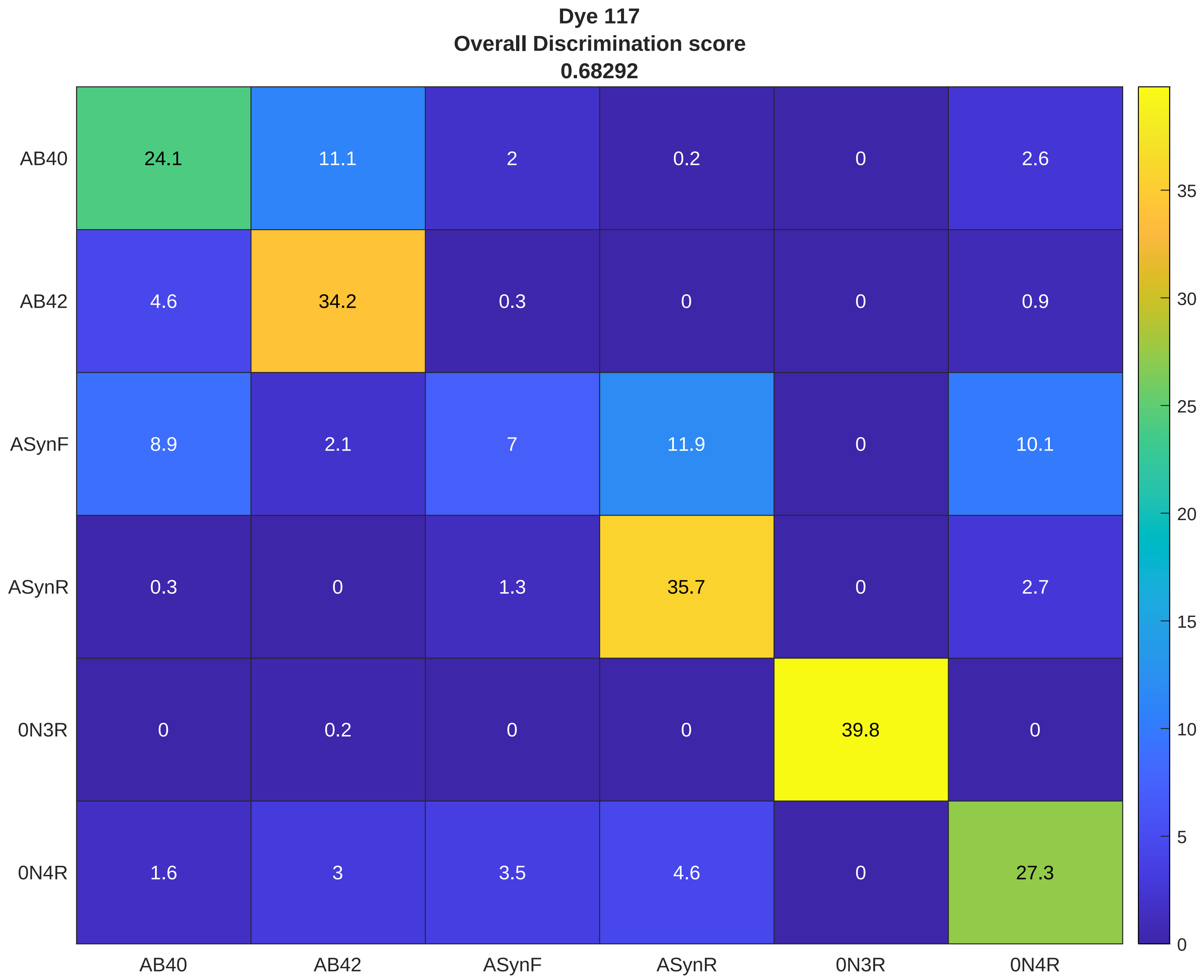
Dye 117
Overall Discrimination score
0.68292
AB40
AB42
ASynF
ASynR
0N3R
0N4R
24.1 11.1 2 0.2 0 2.6
4.6 34.2 0.3 0 0 0.9
8.9 2.1 7 11.9 0 10.1
0.3 0 1.3 35.7 0 2.7
0 0.2 0 0 39.8 0
1.6 3 3.5 4.6 0 27.3
AB40
AB42
ASynF
ASynR
0N3R
0N4R
35
30
25
20
15
10
5
0

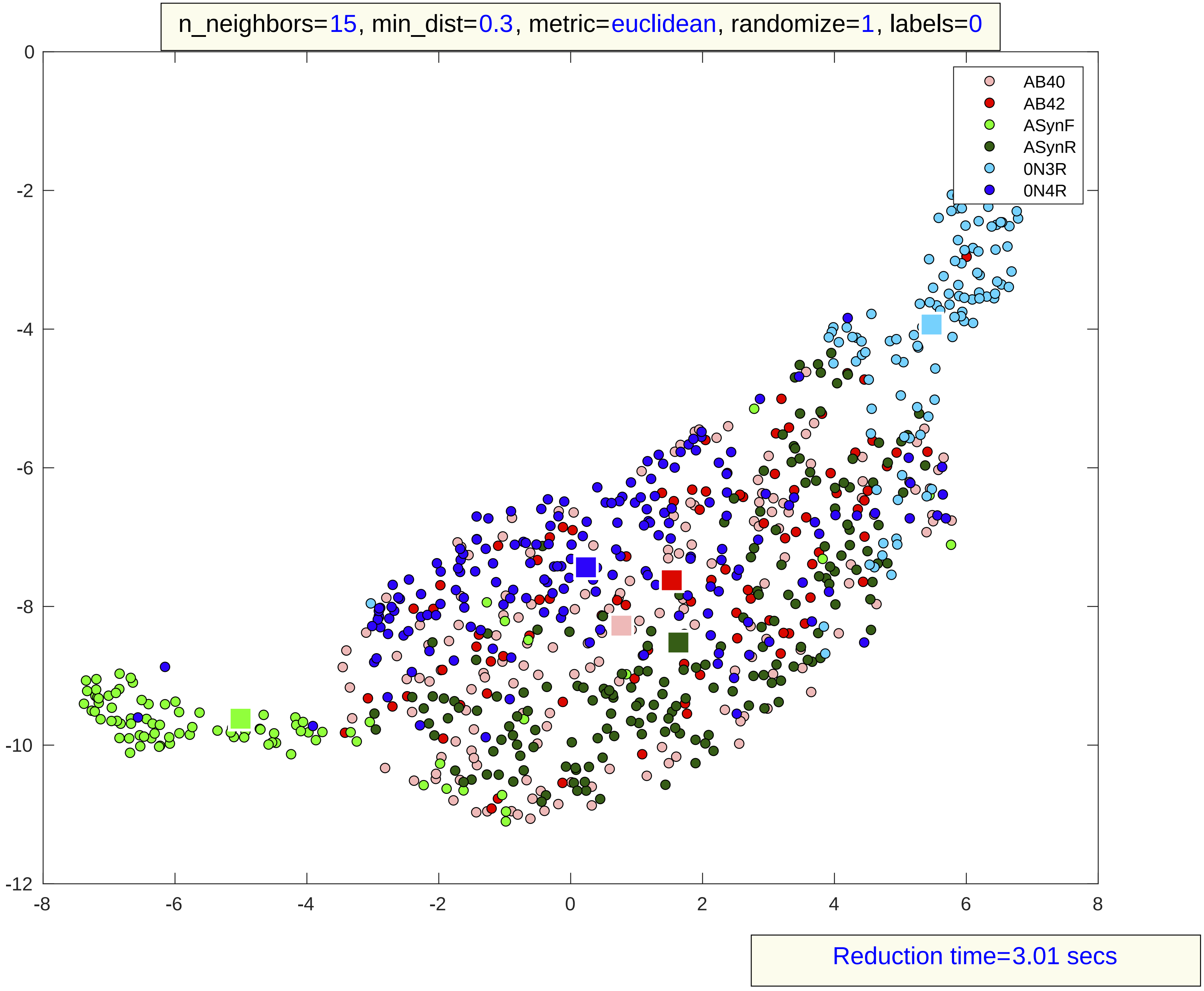
n_neighbors=15, min_dist=0.3, metric=euclidean, randomize=1, labels=0
AB40
AB42
ASynF
ASynR
0N3R
0N4R
0
-2
-4
-6
-8
-10
-12
-8
-6
-4
-2
0
2
4
6
8
Reduction time=3.01 secs

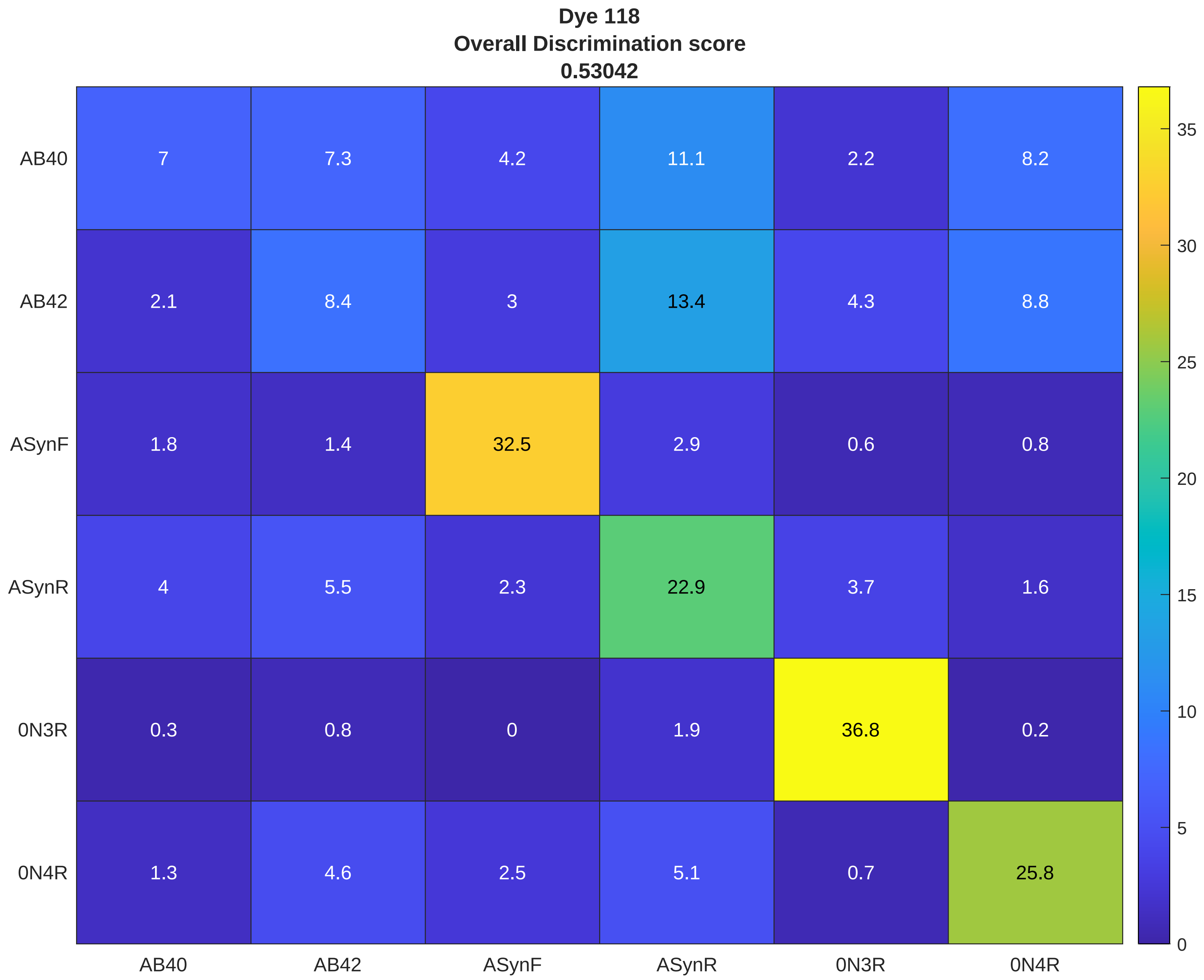
Dye 118
Overall Discrimination score
0.53042
AB40
AB42
ASynF
ASynR
0N3R
0N4R
7
7.3
4.2
11.1
2.2
8.2
2.1
8.4
3
13.4
4.3
8.8
1.8
1.4
32.5
2.9
0.6
0.8
4
5.5
2.3
22.9
3.7
1.6
0.3
0.8
0
1.9
36.8
0.2
1.3
4.6
2.5
5.1
0.7
25.8
AB40
AB42
ASynF
ASynR
0N3R
0N4R
35
30
25
20
15
10
5
0

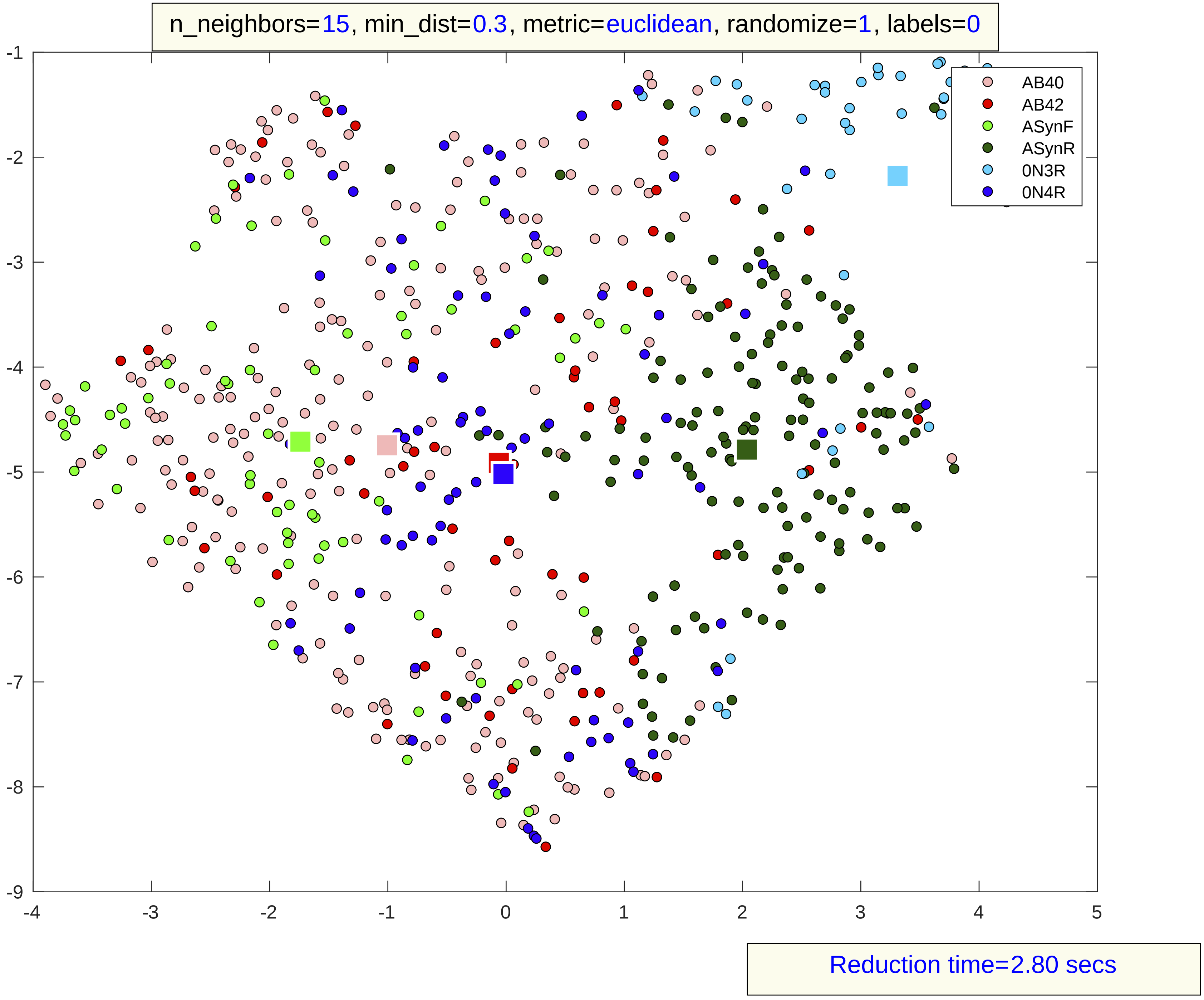
n_neighbors=15, min_dist=0.3, metric=euclidean, randomize=1, labels=0
AB40
AB42
ASynF
ASynR
0N3R
0N4R
-1
-2
-3
-4
-5
-6
-7
-8
-9
-4
-3
-2
-1
0
1
2
3
4
5
Reduction time=2.80 secs

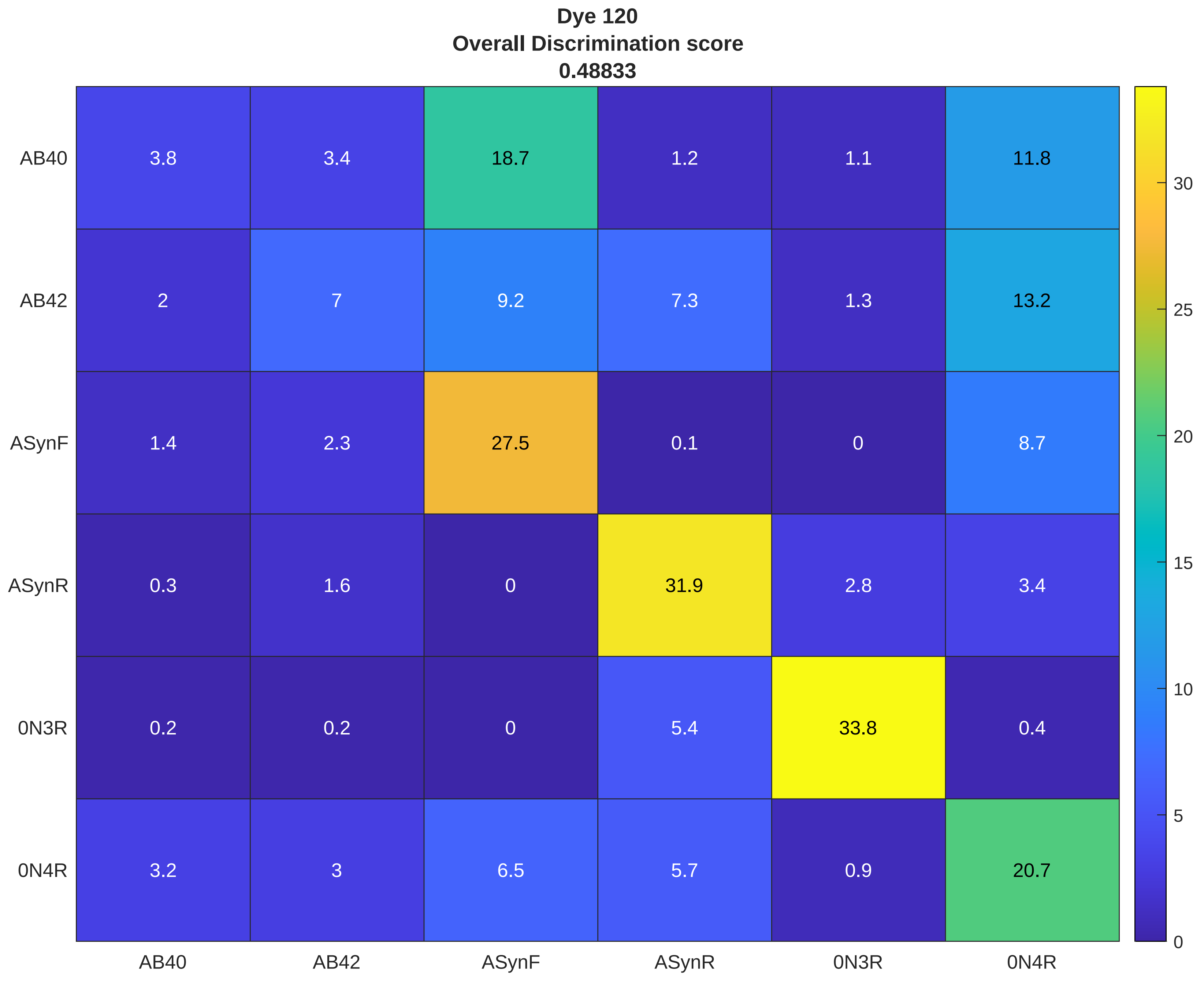

Dye 120
Overall Discrimination score
0.48833
AB40
AB42
ASynF
ASynR
0N3R
0N4R
3.8
3.4
18.7
1.2
1.1
11.8
2
7
9.2
7.3
1.3
13.2
1.4
2.3
27.5
0.1
0
8.7
0.3
1.6
0
31.9
2.8
3.4
0.2
0.2
0
5.4
33.8
0.4
3.2
3
6.5
5.7
0.9
20.7
AB40
AB42
ASynF
ASynR
0N3R
0N4R
30
25
20
15
10
5
0

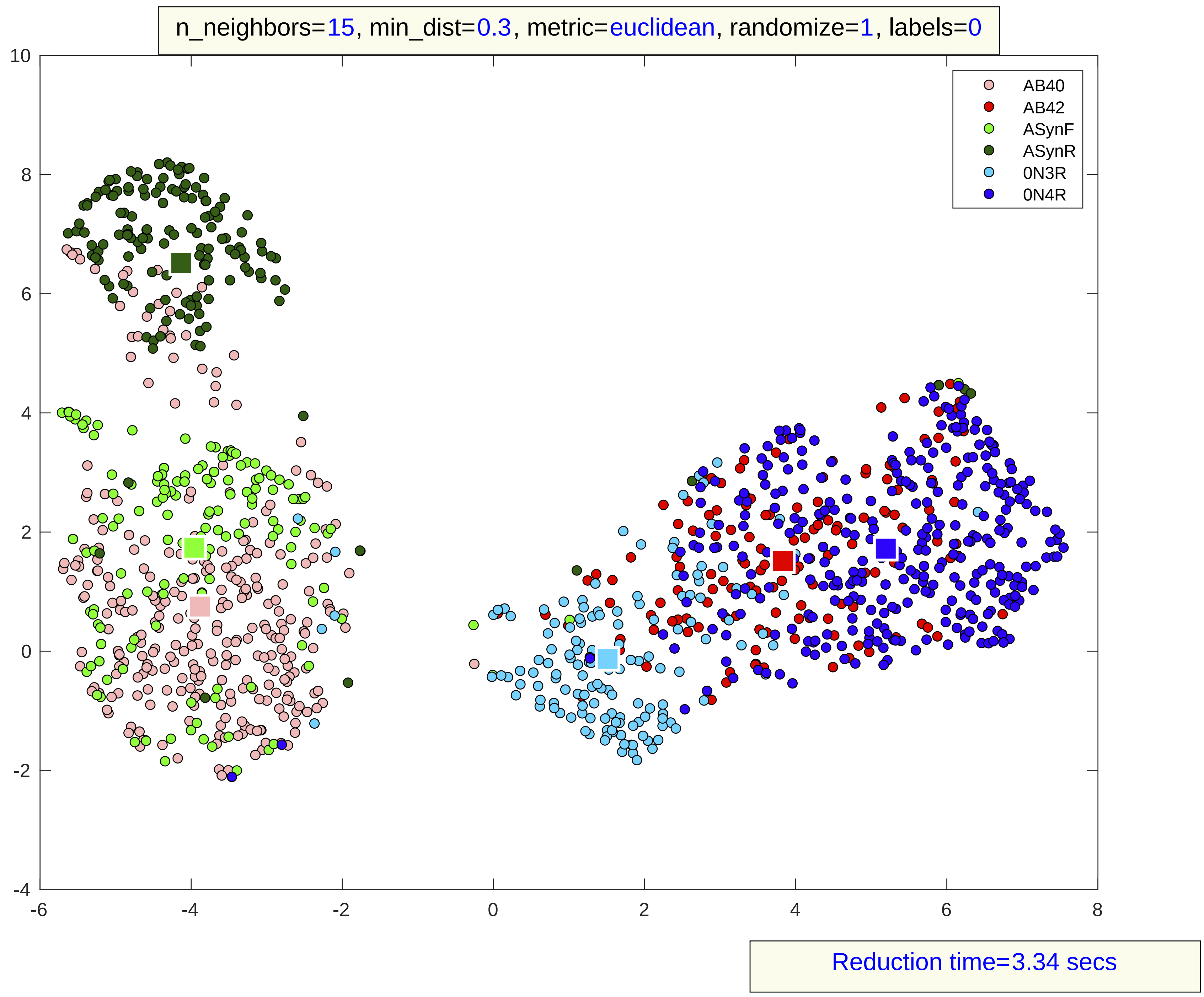

n_neighbors=15, min_dist=0.3, metric=euclidean, randomize=1, labels=0
AB40
AB42
ASynF
ASynR
0N3R
0N4R
10
8
6
4
2
0
-2
-4
-6
-4
-2
0
2
4
6
8
Reduction time=3.34 secs

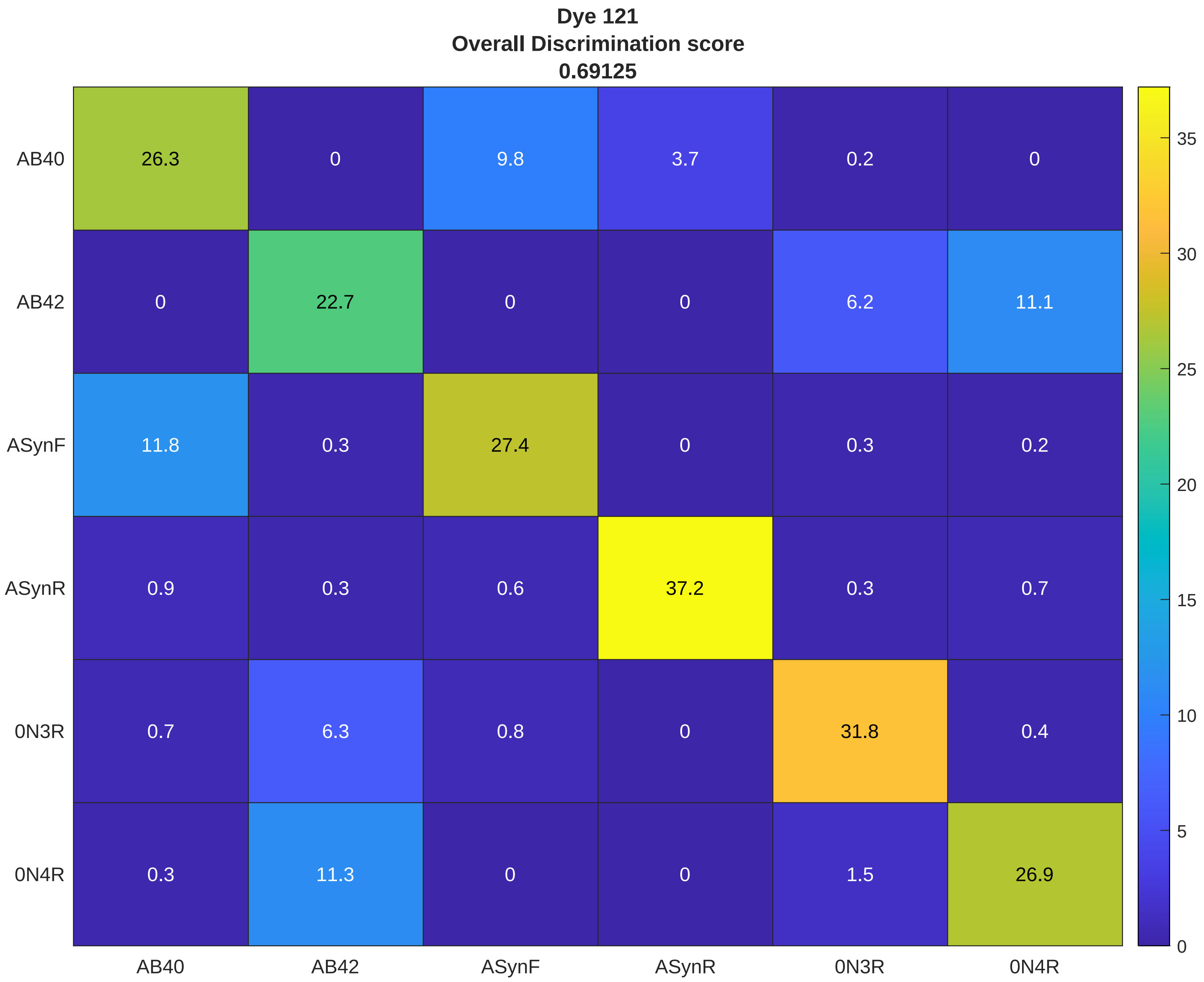

Dye 121
Overall Discrimination score
0.69125
AB40
AB42
ASynF
ASynR
0N3R
0N4R
26.3
0
9.8
3.7
0.2
0
0
22.7
0
0
6.2
11.1
11.8
0.3
27.4
0
0.3
0.2
0.9
0.3
0.6
37.2
0.3
0.7
0.7
6.3
0.8
0
31.8
0.4
0.3
11.3
0
0
1.5
26.9
AB40
AB42
ASynF
ASynR
0N3R
0N4R
35
30
25
20
15
10
5
0

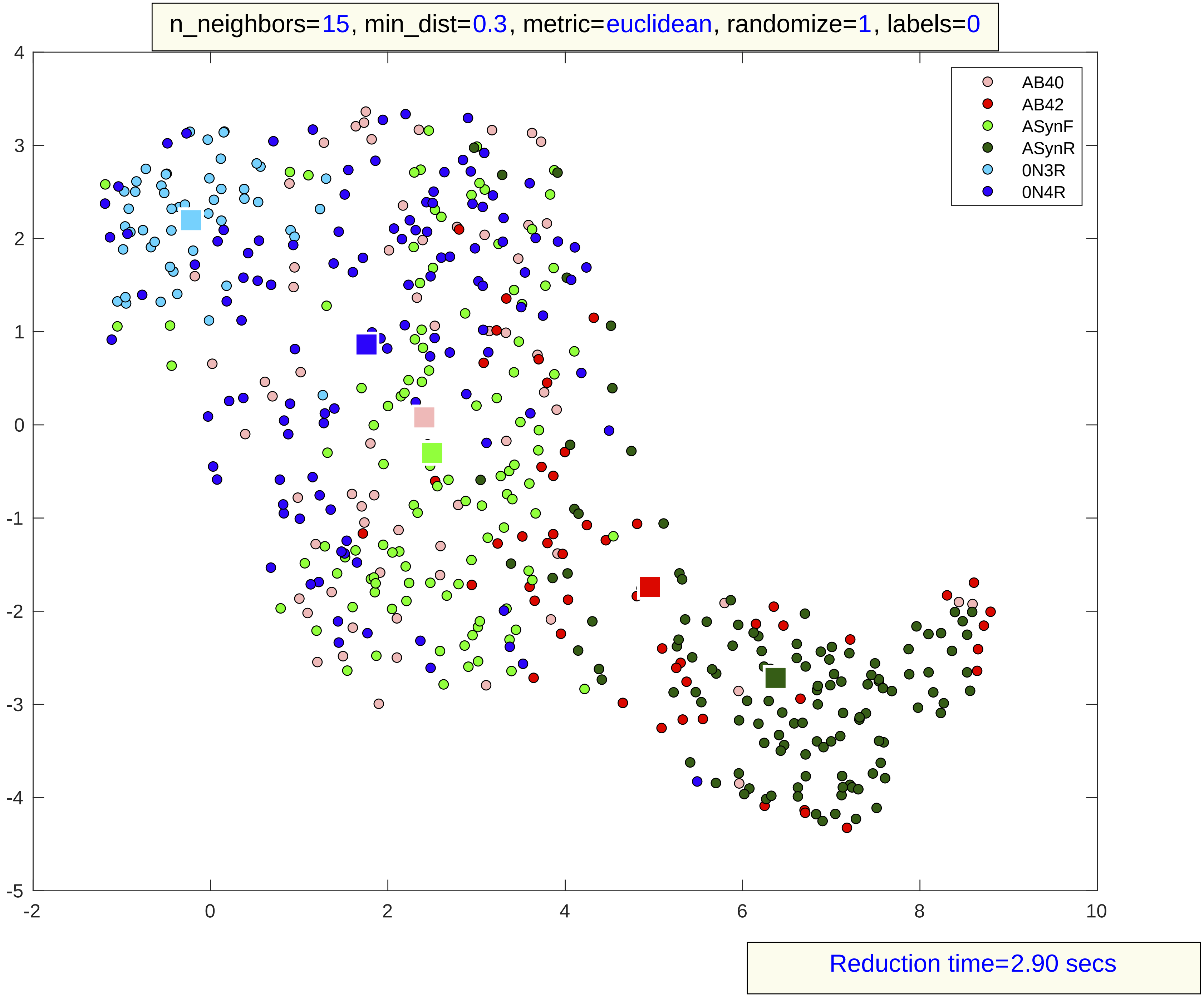

n_neighbors=15, min_dist=0.3, metric=euclidean, randomize=1, labels=0
AB40
AB42
ASynF
ASynR
0N3R
0N4R
4
3
2
1
0
-1
-2
-3
-4
-5
-2
0
2
4
6
8
10
Reduction time=2.90 secs

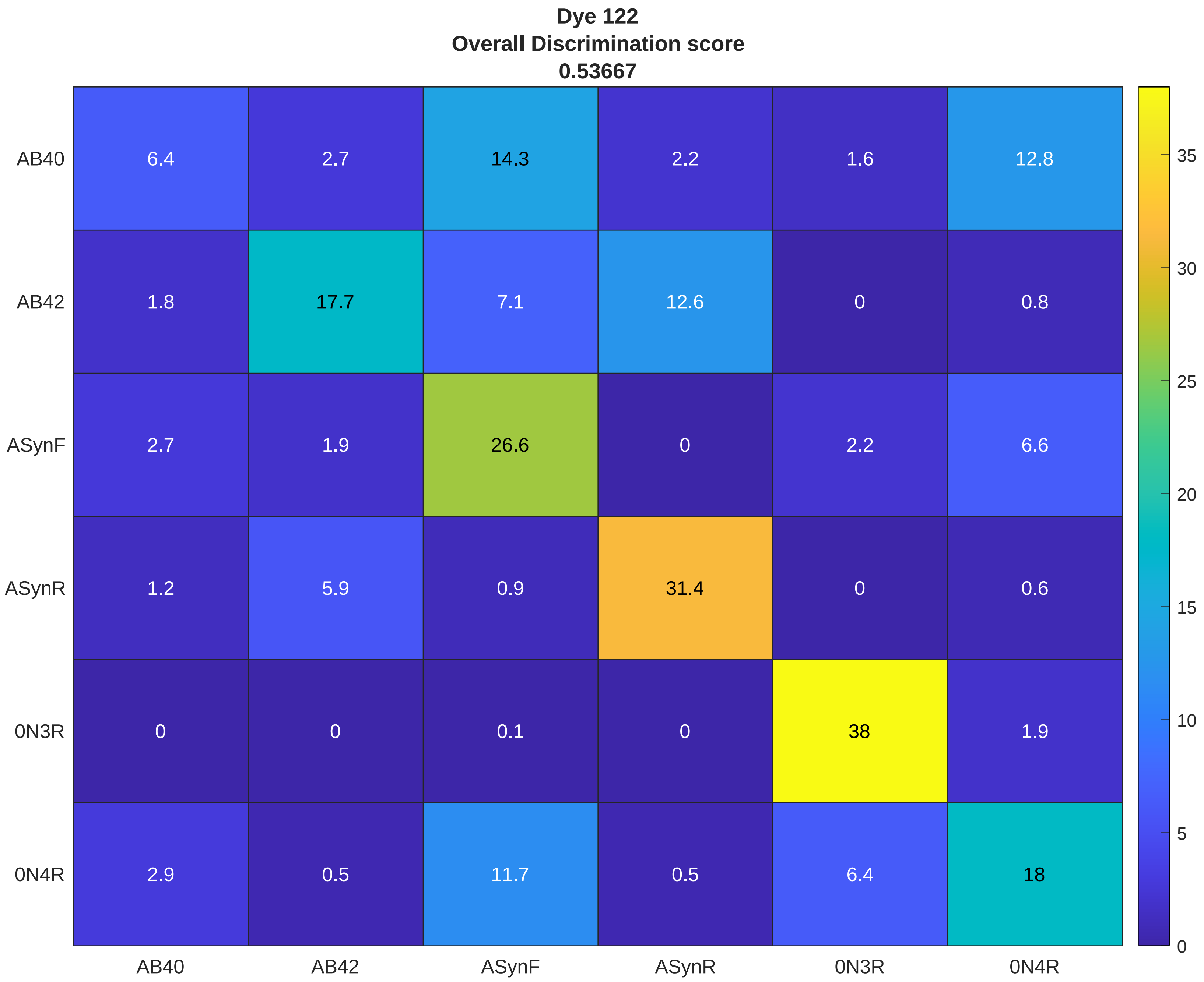
Dye 122
Overall Discrimination score
0.53667
AB40
AB42
ASynF
ASynR
0N3R
0N4R
6.4
2.7
14.3
2.2
1.6
12.8
1.8
17.7
7.1
12.6
0
0.8
2.7
1.9
26.6
0
2.2
6.6
1.2
5.9
0.9
31.4
0
0.6
0
0
0.1
0
38
1.9
2.9
0.5
11.7
0.5
6.4
18
AB40
AB42
ASynF
ASynR
0N3R
0N4R
35
30
25
20
15
10
5
0

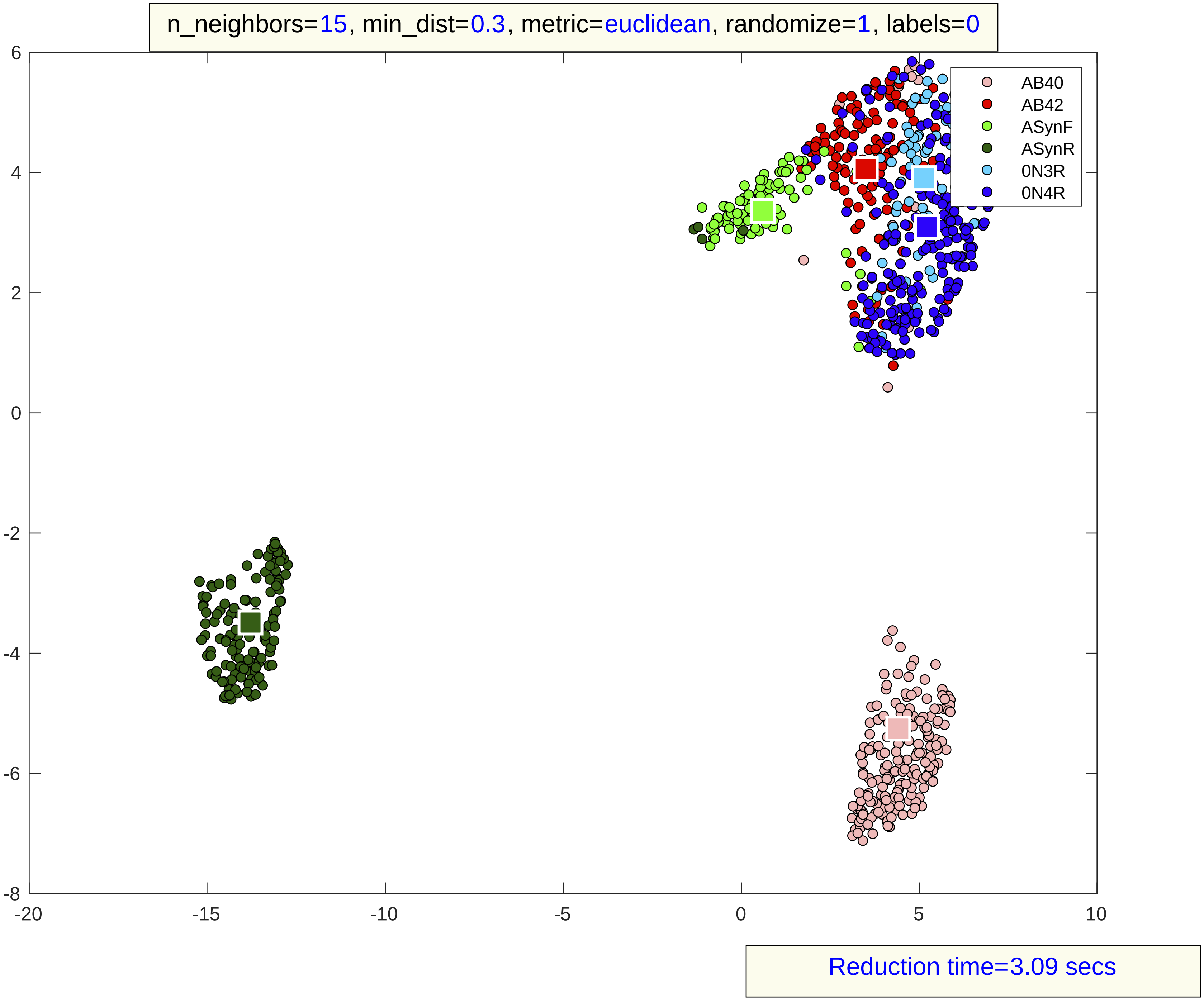

n_neighbors=15, min_dist=0.3, metric=euclidean, randomize=1, labels=0
AB40
AB42
ASynF
ASynR
0N3R
0N4R
6
4
2
0
-2
-4
-6
-8
-20
-15
-10
-5
0
5
10
Reduction time=3.09 secs

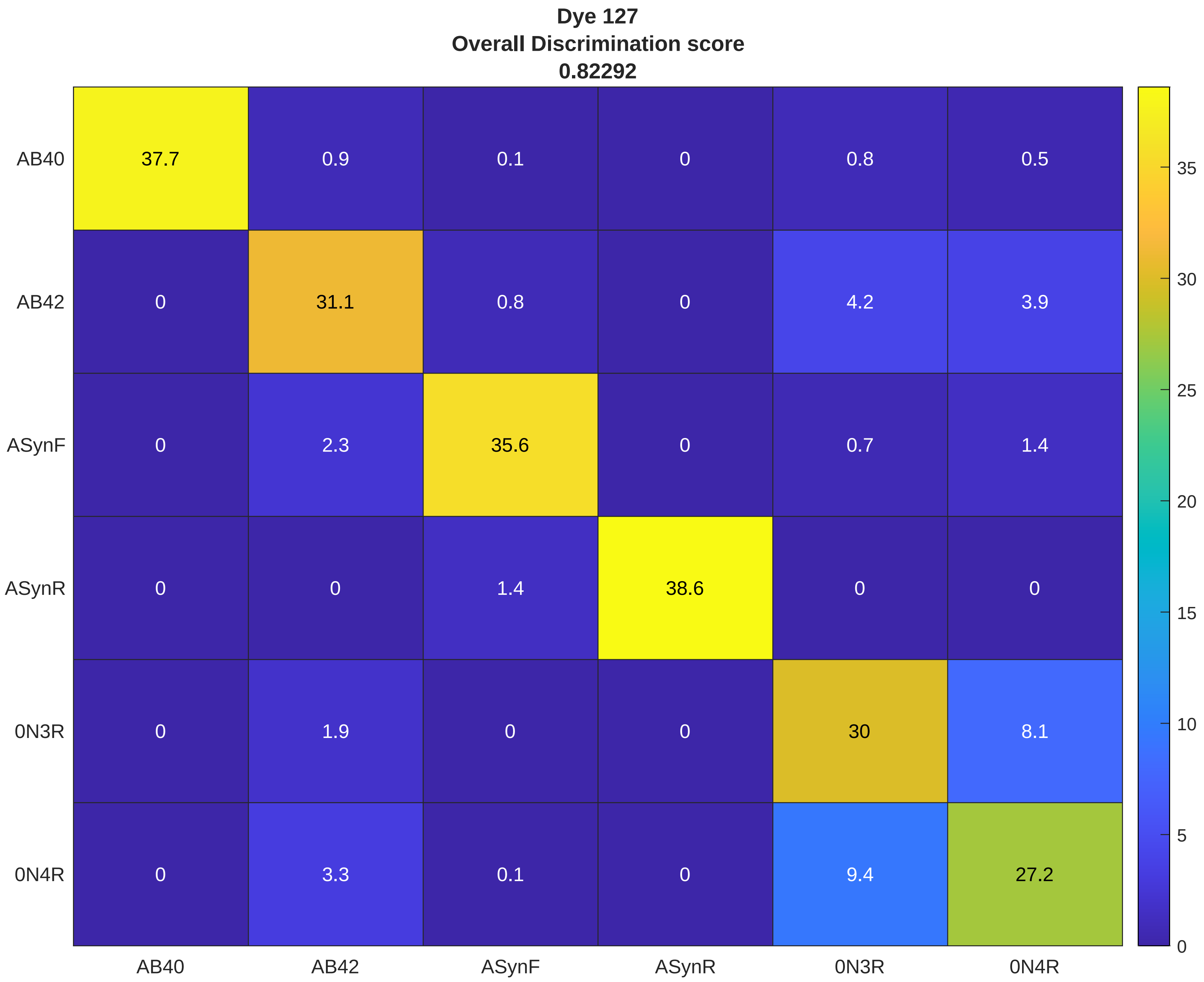
Dye 127
Overall Discrimination score
0.82292
AB40
AB42
ASynF
ASynR
0N3R
0N4R
37.7
0.9
0.1
0
0.8
0.5
0
31.1
0.8
0
4.2
3.9
0
2.3
35.6
0
0.7
1.4
0
0
1.4
38.6
0
0
0
1.9
0
0
30
8.1
0
3.3
0.1
0
9.4
27.2
AB40
AB42
ASynF
ASynR
0N3R
0N4R
35
30
25
20
15
10
5
0

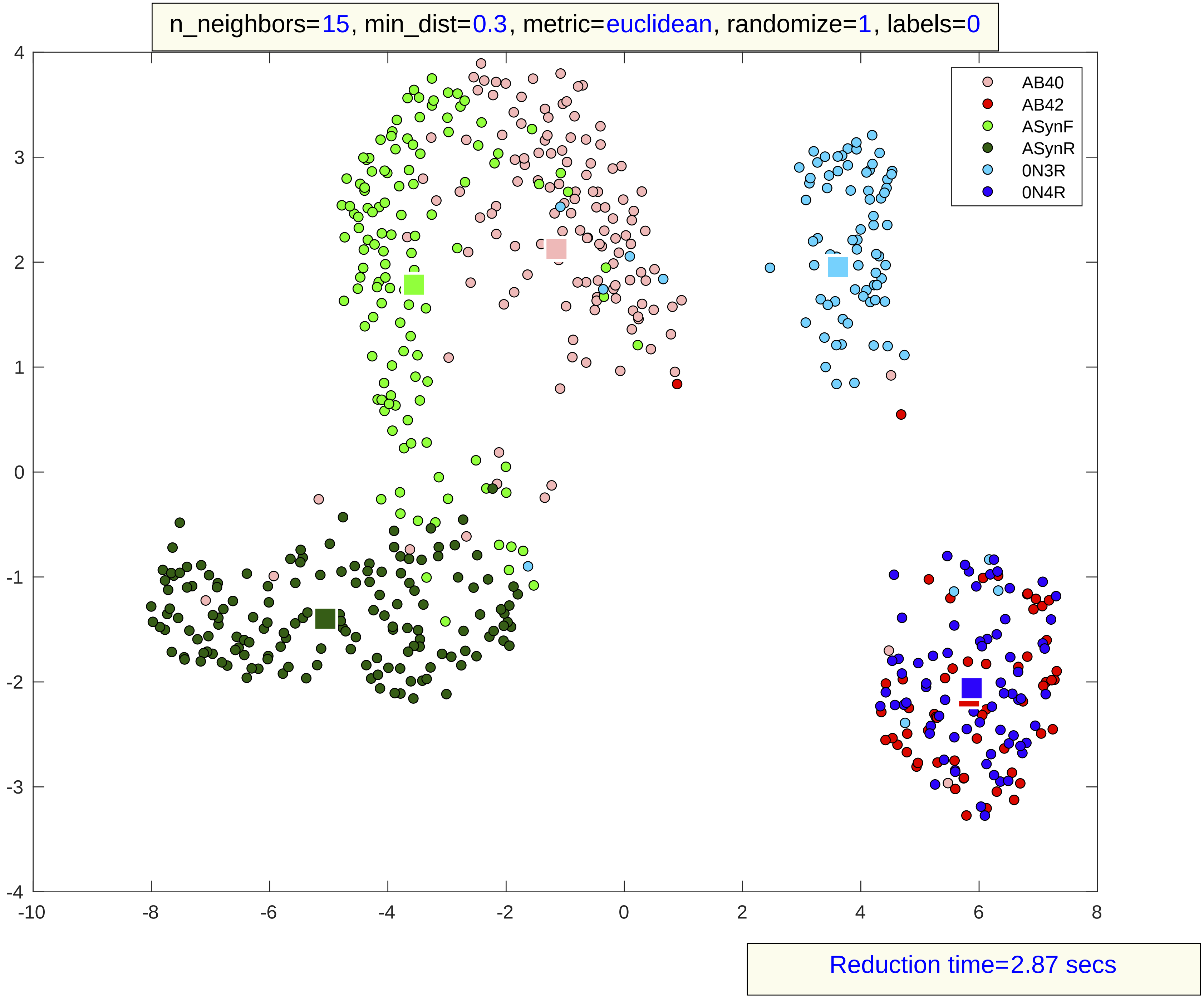

n_neighbors=15, min_dist=0.3, metric=euclidean, randomize=1, labels=0
AB40
AB42
ASynF
ASynR
0N3R
0N4R
4
3
2
1
0
-1
-2
-3
-4
-10
-8
-6
-4
-2
0
2
4
6
8
Reduction time=2.87 secs

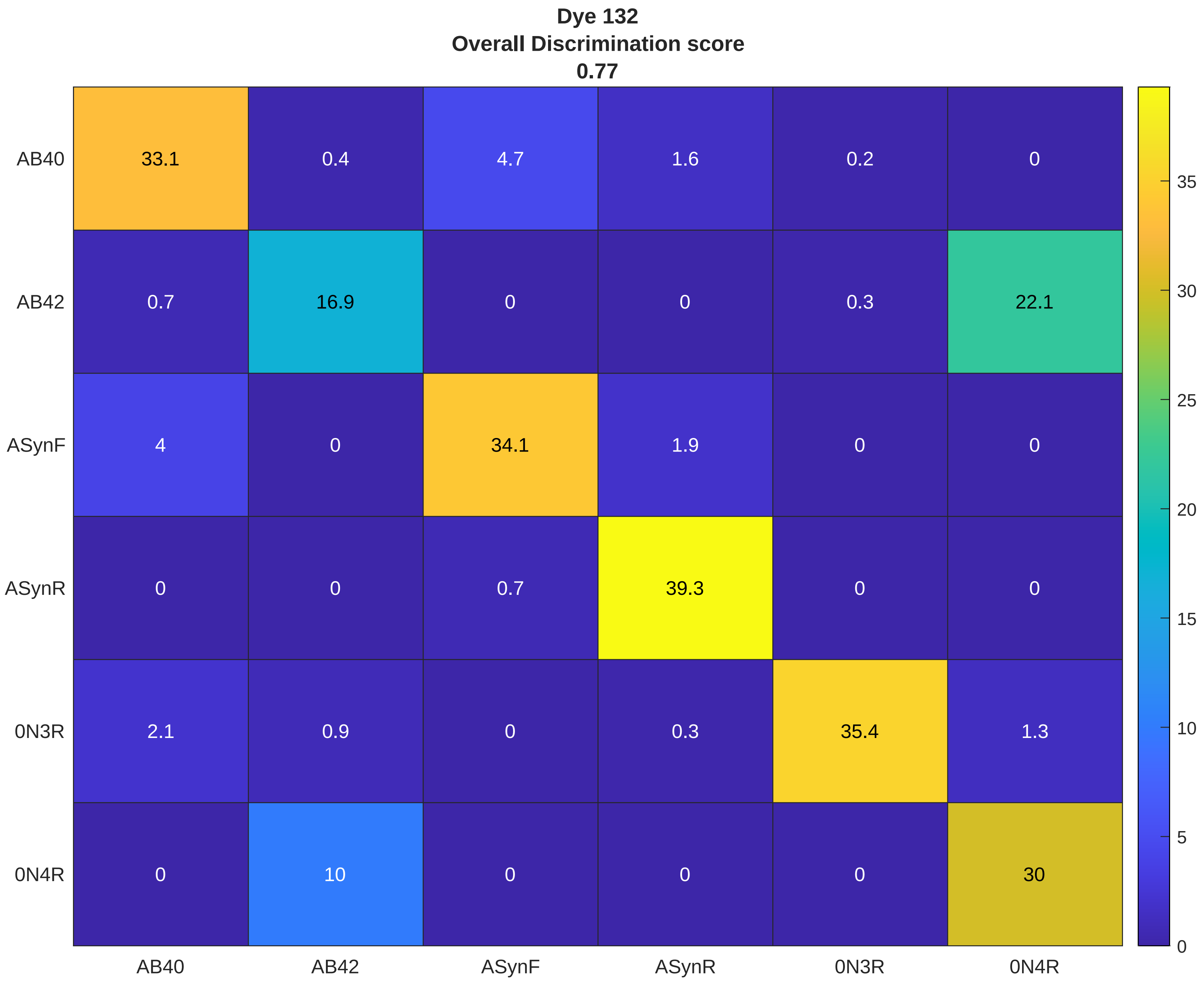
Dye 132
Overall Discrimination score
0.77
AB40
AB42
ASynF
ASynR
0N3R
0N4R
33.1
0.4
4.7
1.6
0.2
0
0.7
16.9
0
0
0.3
22.1
4
0
34.1
1.9
0
0
0
0
0.7
39.3
0
0
2.1
0.9
0
0.3
35.4
1.3
0
10
0
0
0
30
AB40
AB42
ASynF
ASynR
0N3R
0N4R
35
30
25
20
15
10
5
0

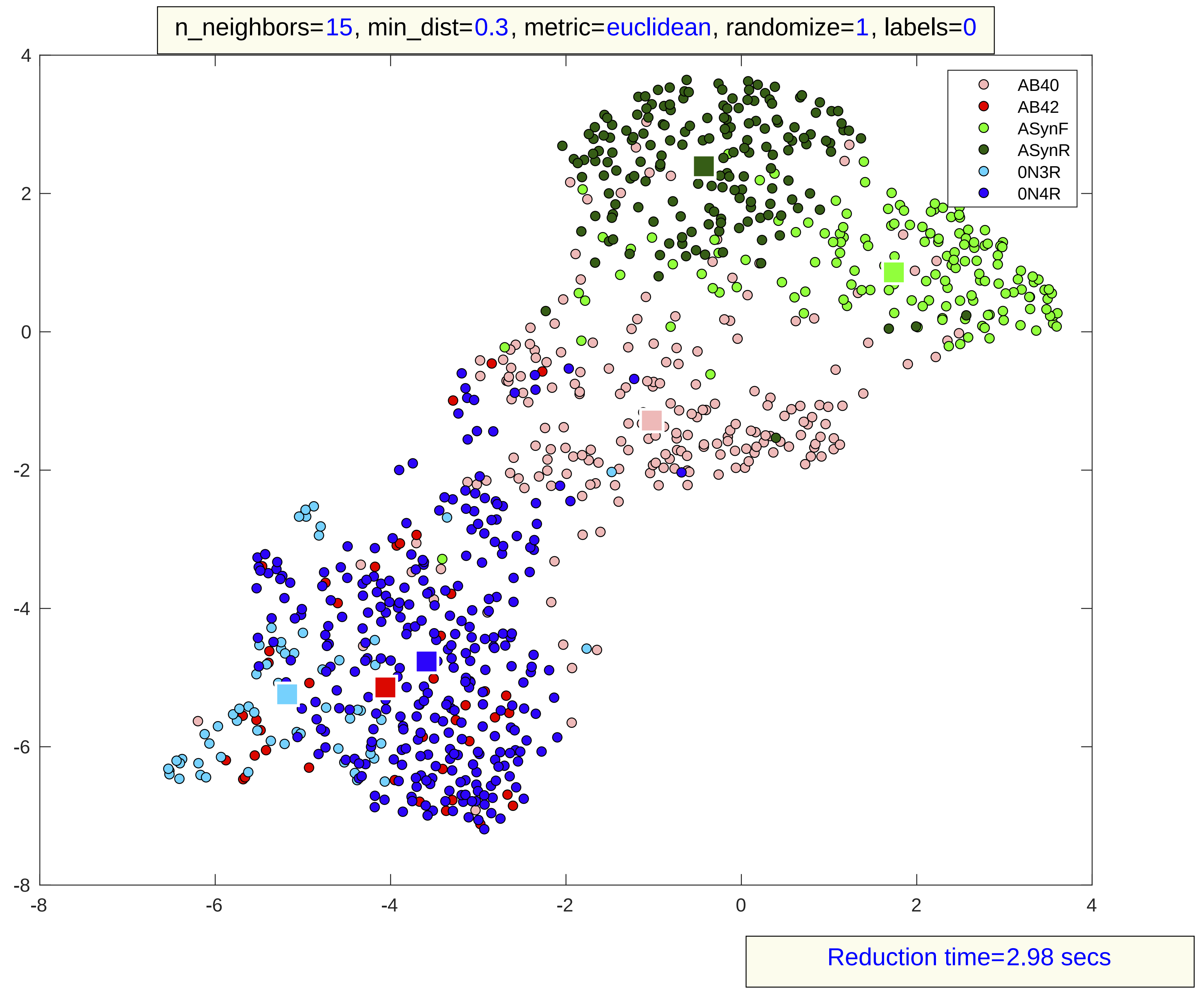

n_neighbors=15, min_dist=0.3, metric=euclidean, randomize=1, labels=0
AB40
AB42
ASynF
ASynR
0N3R
0N4R
4
2
0
-2
-4
-6
-8
-8
-6
-4
-2
0
2
4
Reduction time=2.98 secs

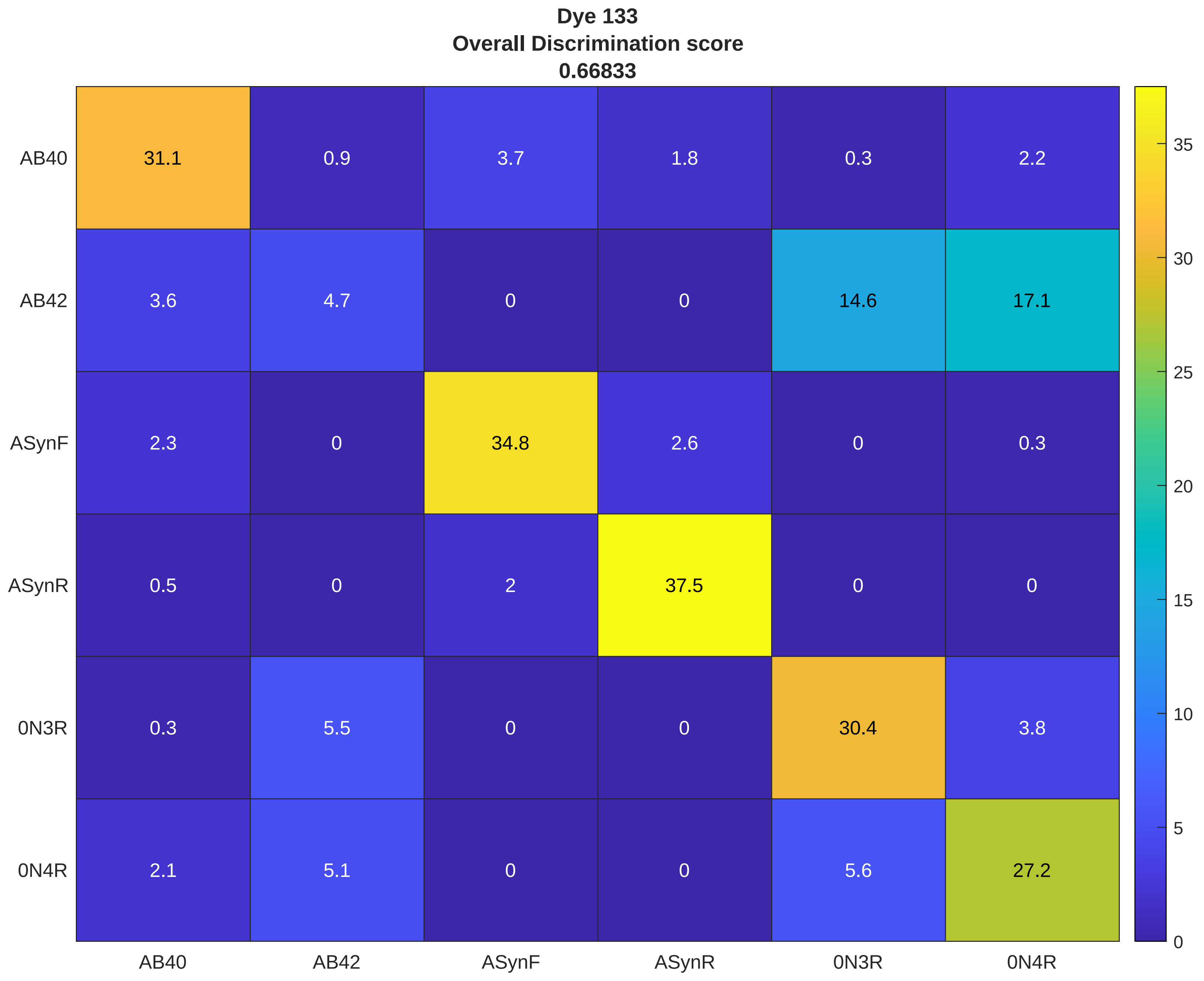

Dye 133
Overall Discrimination score
0.66833
AB40
AB42
ASynF
ASynR
0N3R
0N4R
31.1
0.9
3.7
1.8
0.3
2.2
3.6
4.7
0
0
14.6
17.1
2.3
0
34.8
2.6
0
0.3
0.5
0
2
37.5
0
0
0.3
5.5
0
0
30.4
3.8
2.1
5.1
0
0
5.6
27.2
0
5
10
15
20
25
30
35

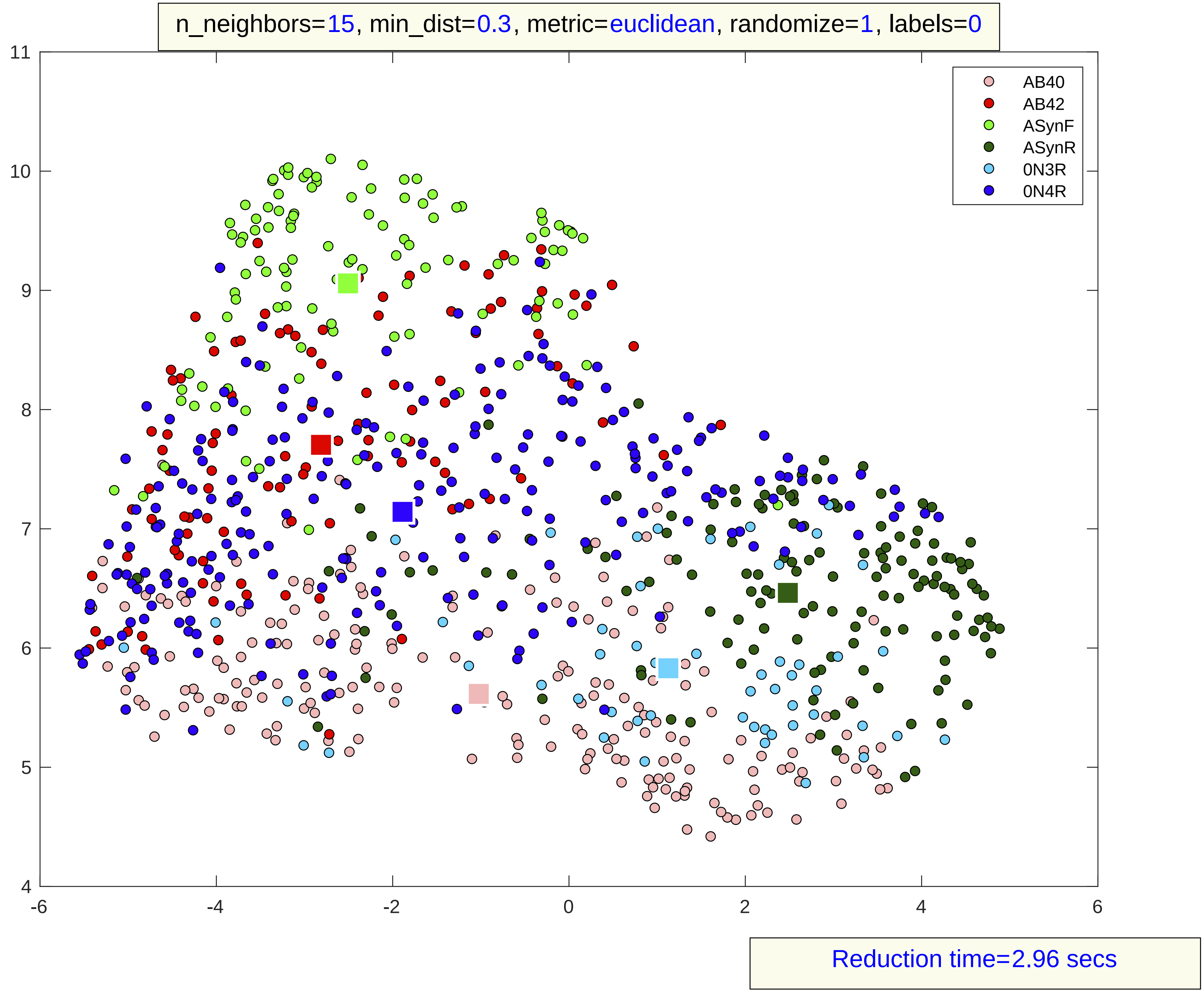
n_neighbors=15, min_dist=0.3, metric=euclidean, randomize=1, labels=0
11
10
9
8
7
6
5
4
-6
-4
-2
0
2
4
6
AB40
AB42
ASynF
ASynR
0N3R
0N4R
Reduction time=2.96 secs

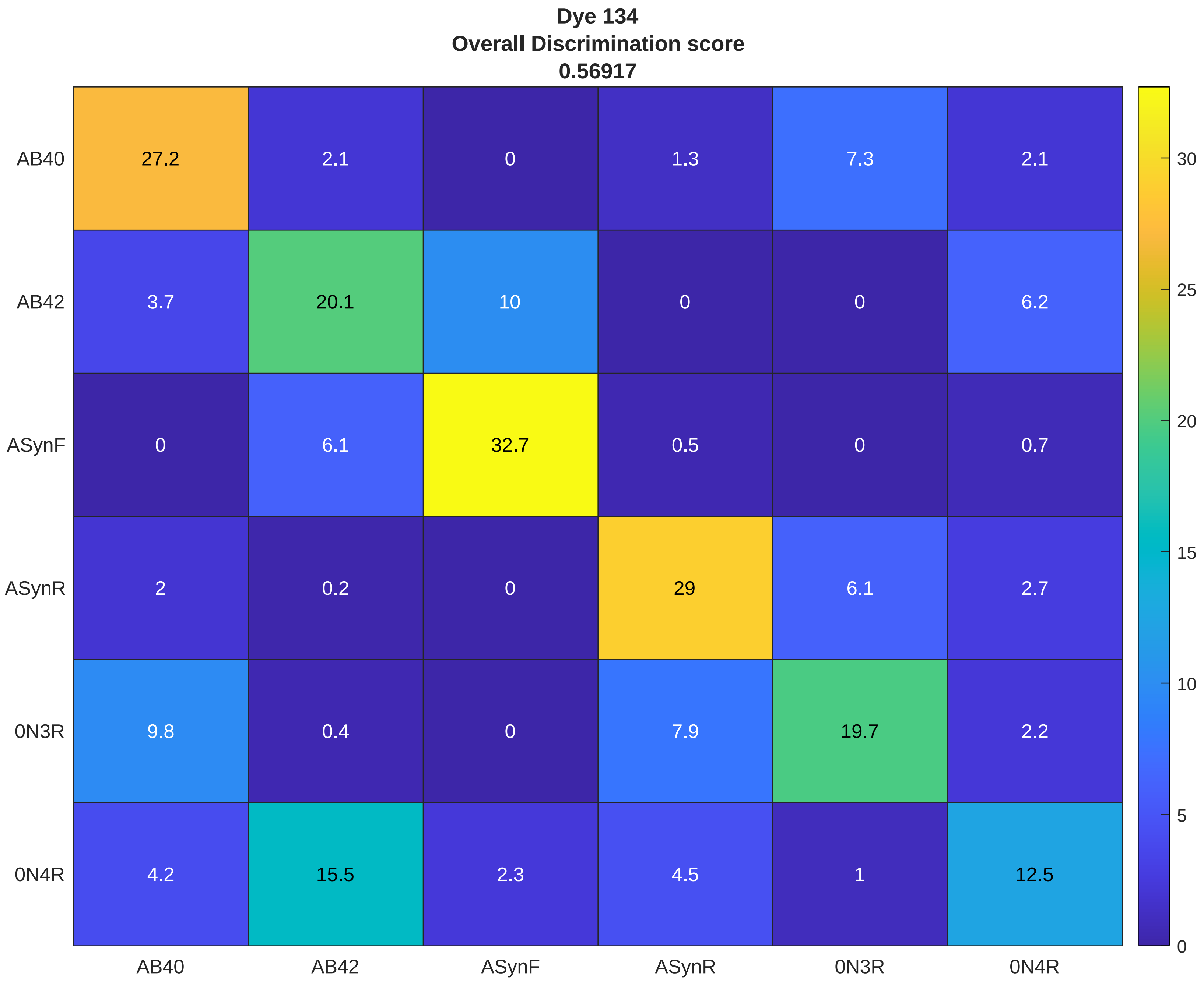
Dye 134
Overall Discrimination score
0.56917
AB40
AB42
ASynF
ASynR
0N3R
0N4R
27.2
2.1
0
1.3
7.3
2.1
3.7
20.1
10
0
0
6.2
0
6.1
32.7
0.5
0
0.7
2
0.2
0
29
6.1
2.7
9.8
0.4
0
7.9
19.7
2.2
4.2
15.5
2.3
4.5
1
12.5
AB40
AB42
ASynF
ASynR
0N3R
0N4R
30
25
20
15
10
5
0

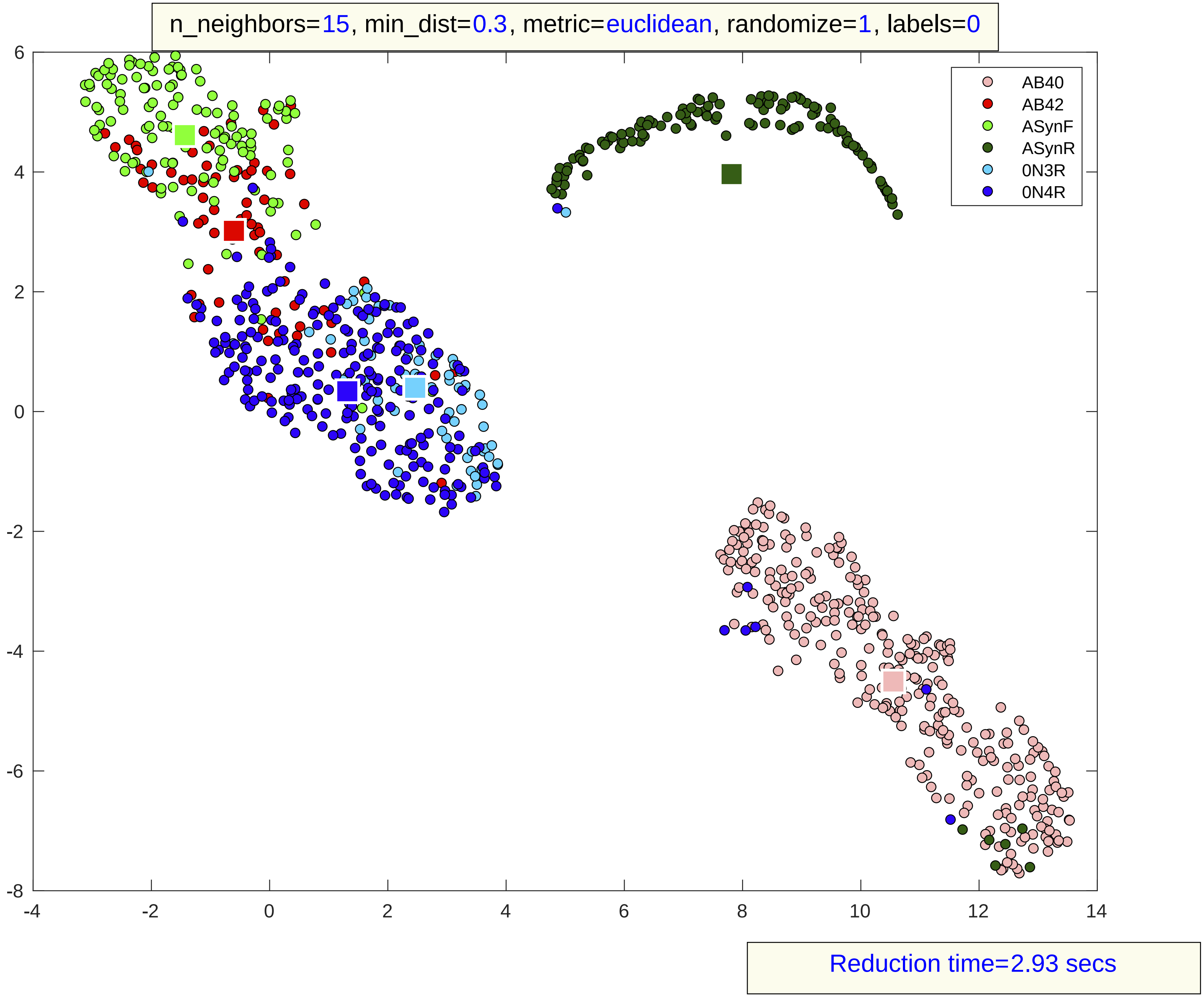
n_neighbors=15, min_dist=0.3, metric=euclidean, randomize=1, labels=0
AB40
AB42
ASynF
ASynR
0N3R
0N4R
6
4
2
0
-2
-4
-6
-8
-4
-2
0
2
4
6
8
10
12
14
Reduction time=2.93 secs

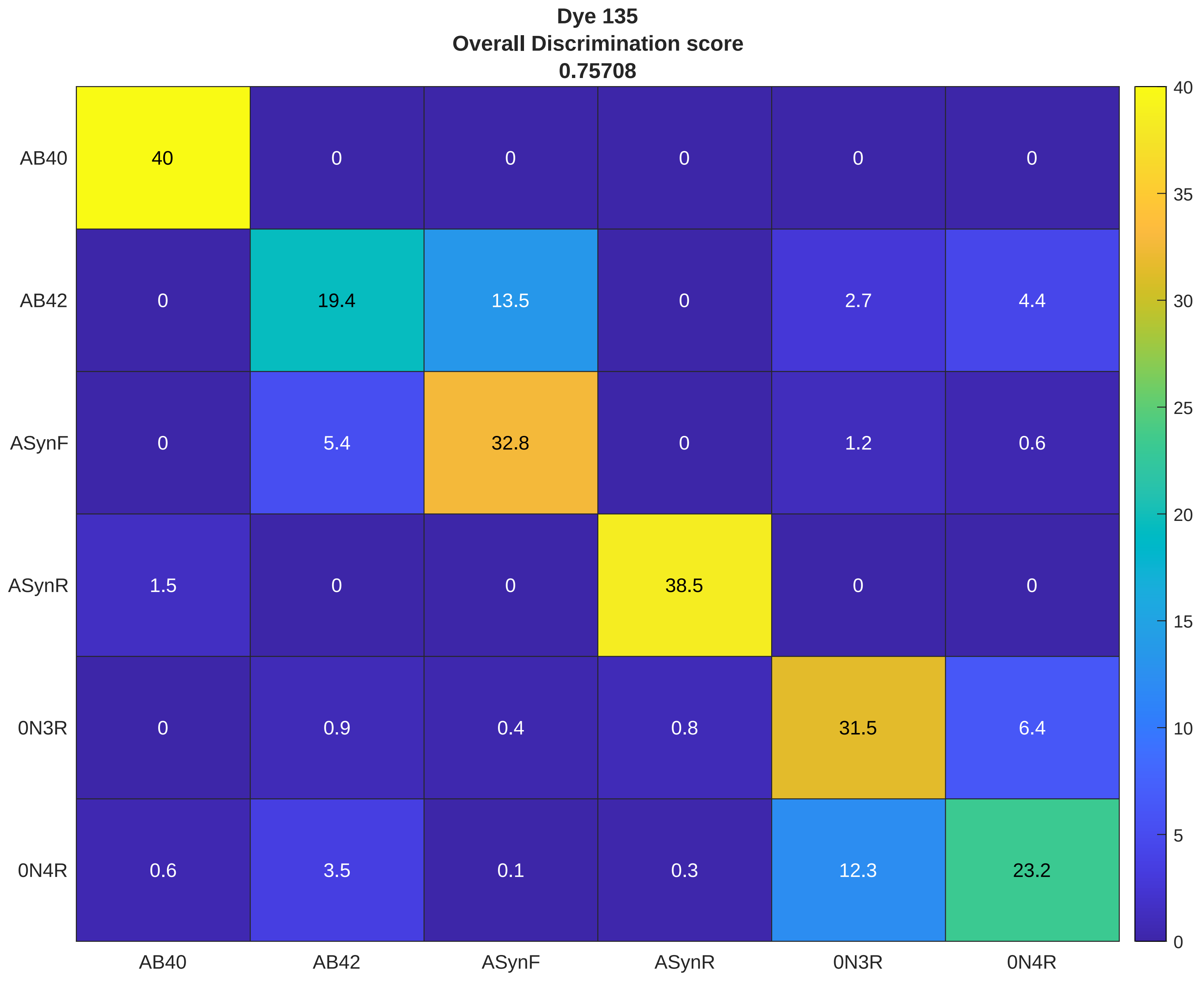
Dye 135
Overall Discrimination score
0.75708
AB40
AB42
ASynF
ASynR
0N3R
0N4R
40
0
0
0
0
0
0
19.4
13.5
0
2.7
4.4
0
5.4
32.8
0
1.2
0.6
1.5
0
0
38.5
0
0
0
0.9
0.4
0.8
31.5
6.4
0.6
3.5
0.1
0.3
12.3
23.2
AB40
AB42
ASynF
ASynR
0N3R
0N4R
40
35
30
25
20
15
10
5
0

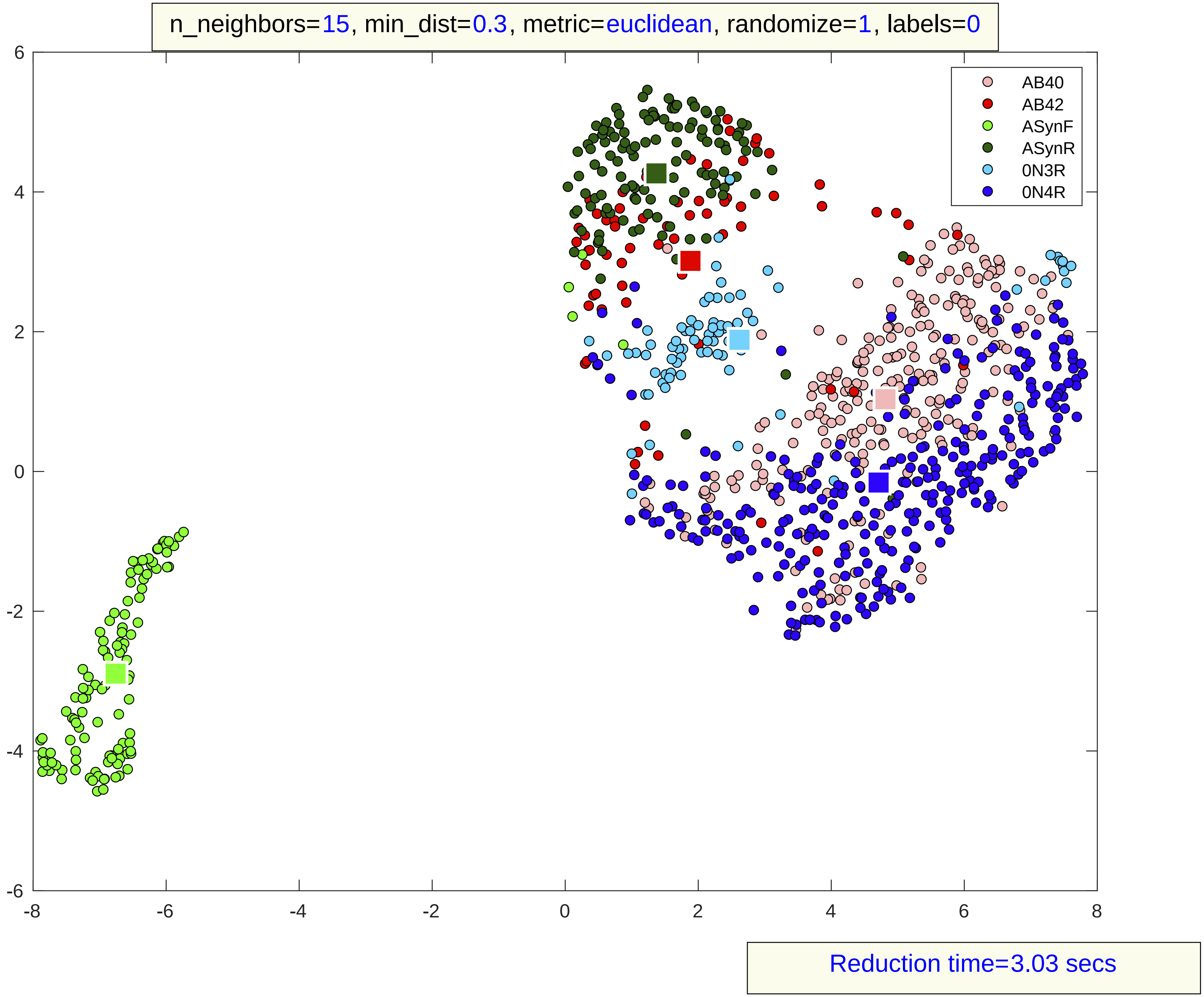
n_neighbors=15, min_dist=0.3, metric=euclidean, randomize=1, labels=0
AB40
AB42
ASynF
ASynR
0N3R
0N4R
6
4
2
0
-2
-4
-6
-8
-6
-4
-2
0
2
4
6
8
Reduction time=3.03 secs

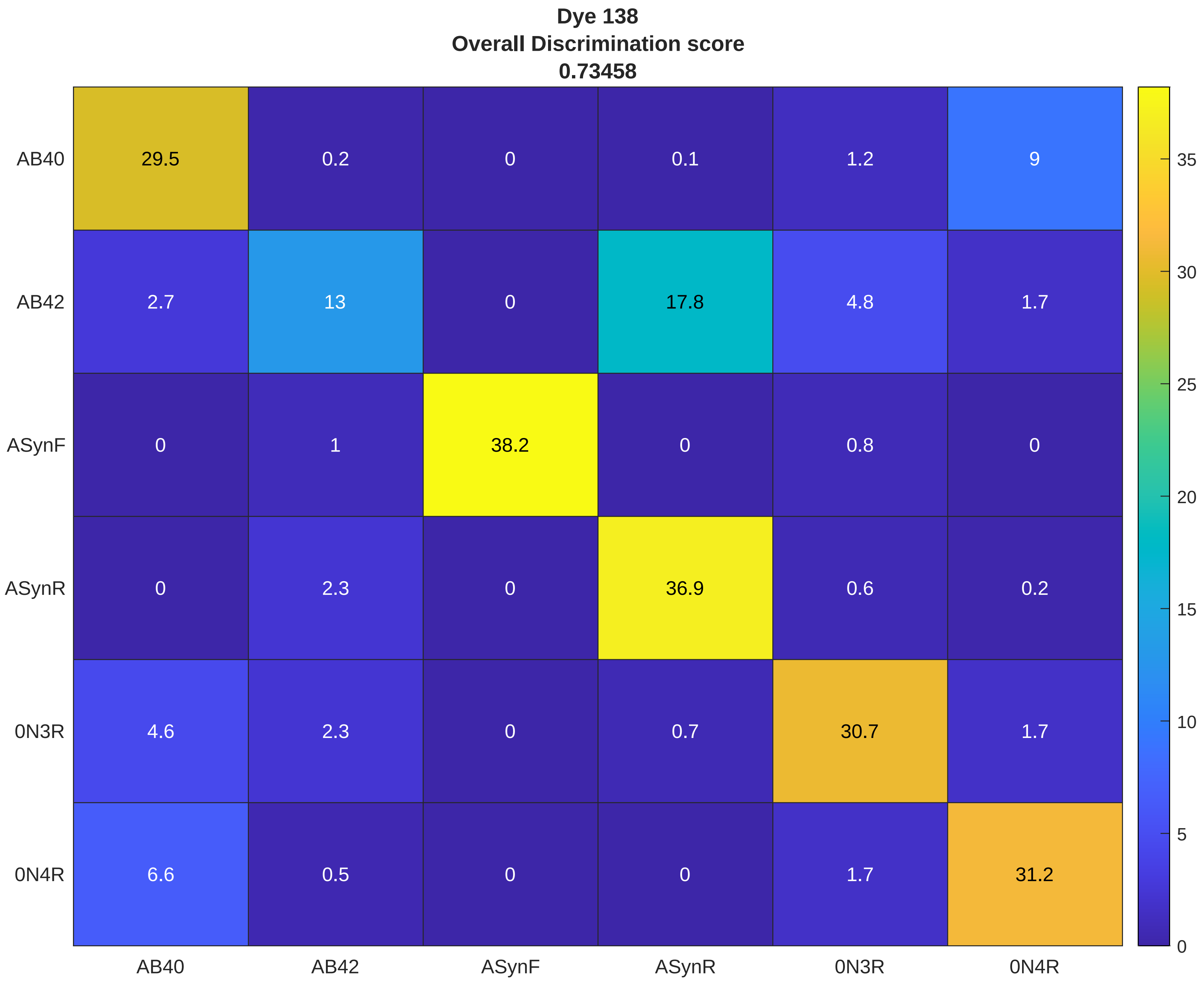
Dye 138
Overall Discrimination score
0.73458
AB40
AB42
ASynF
ASynR
0N3R
0N4R
29.5
0.2
0
0.1
1.2
9
2.7
13
0
17.8
4.8
1.7
0
1
38.2
0
0.8
0
0
2.3
0
36.9
0.6
0.2
4.6
2.3
0
0.7
30.7
1.7
6.6
0.5
0
0
1.7
31.2
AB40
AB42
ASynF
ASynR
0N3R
0N4R
35
30
25
20
15
10
5
0

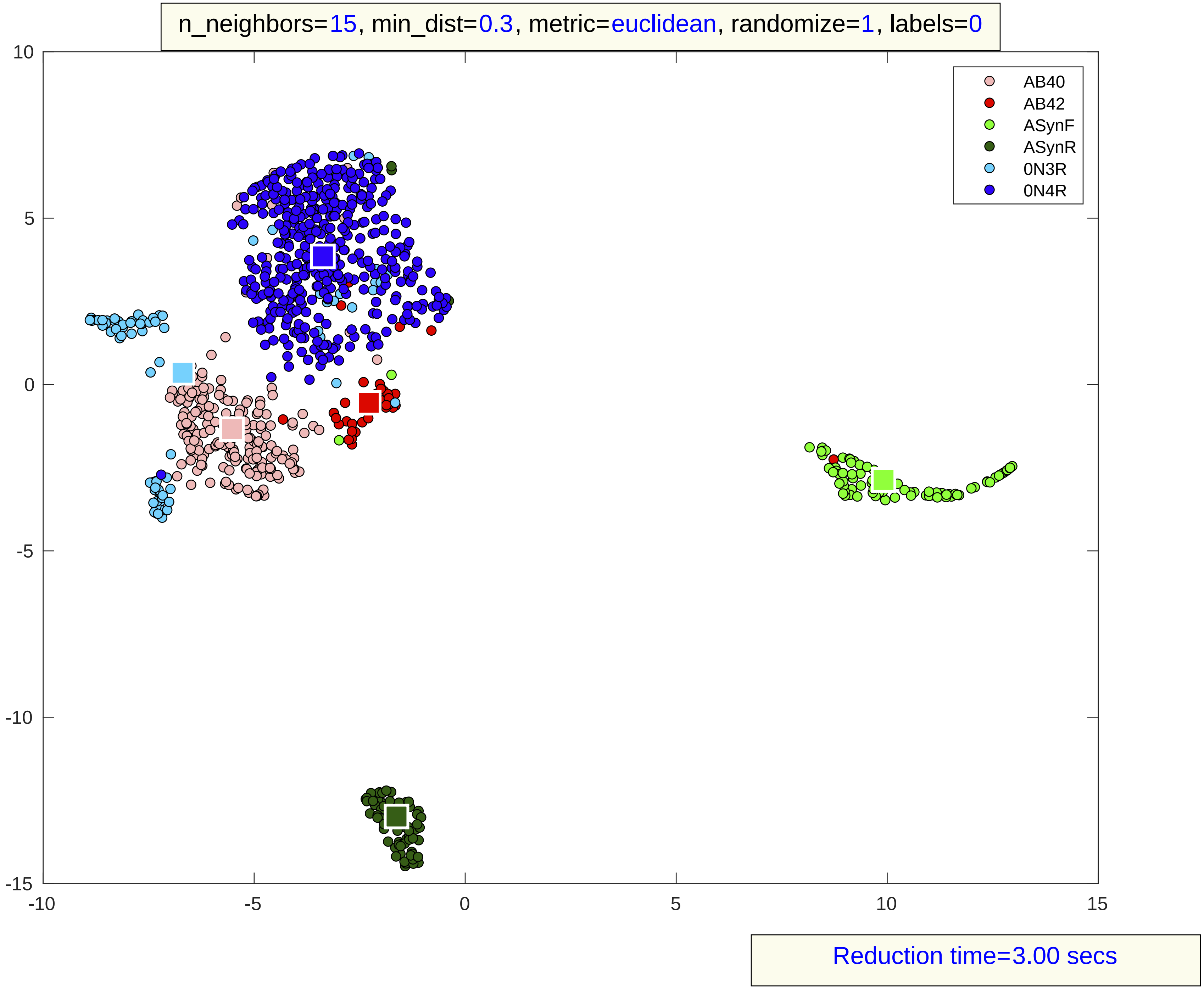
n_neighbors=15, min_dist=0.3, metric=euclidean, randomize=1, labels=0
AB40
AB42
ASynF
ASynR
0N3R
0N4R
10
5
0
-5
-10
-15
-10
-5
0
5
10
15
Reduction time=3.00 secs

Dye 141
Overall Discrimination score
0.8825

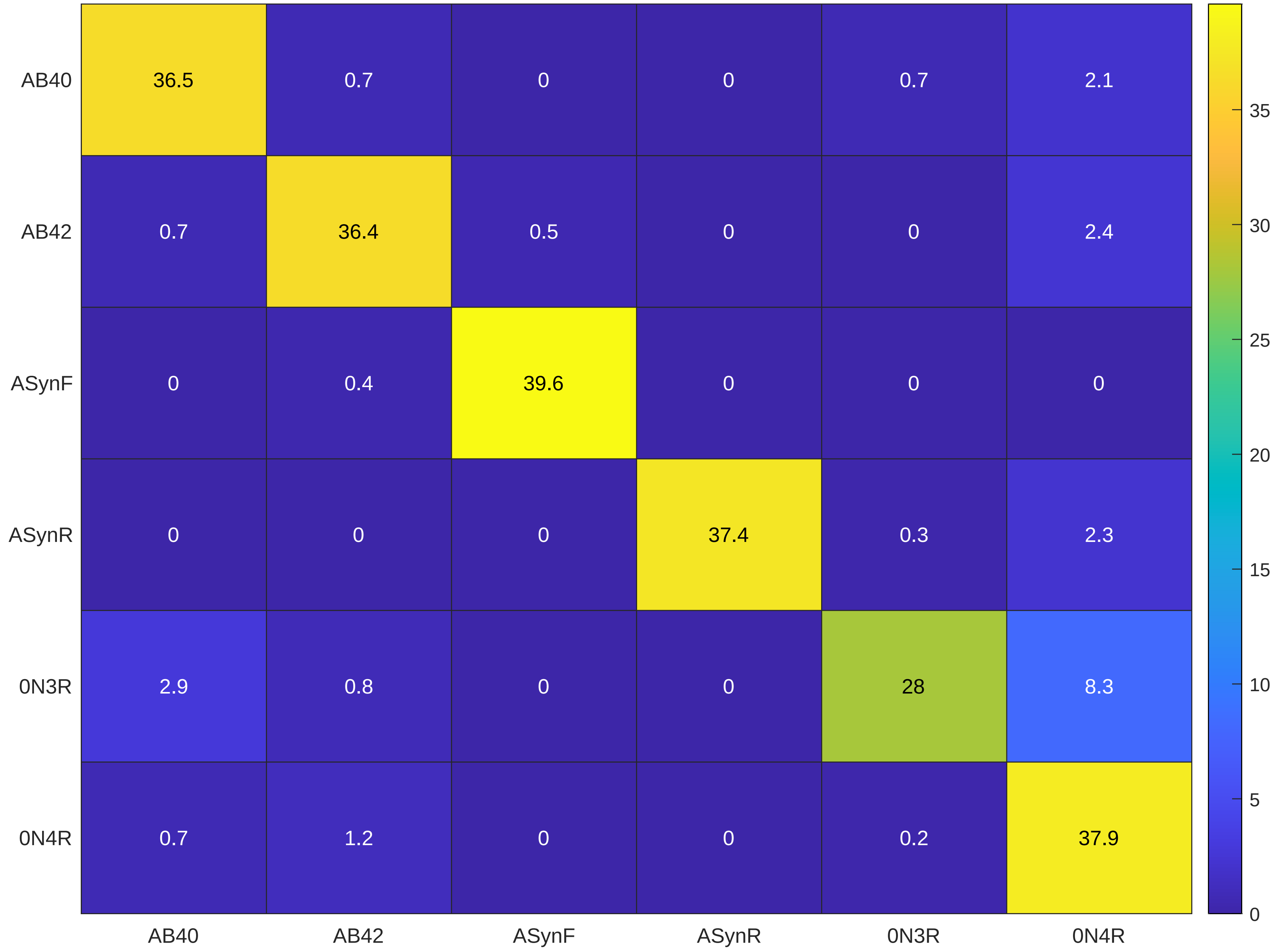

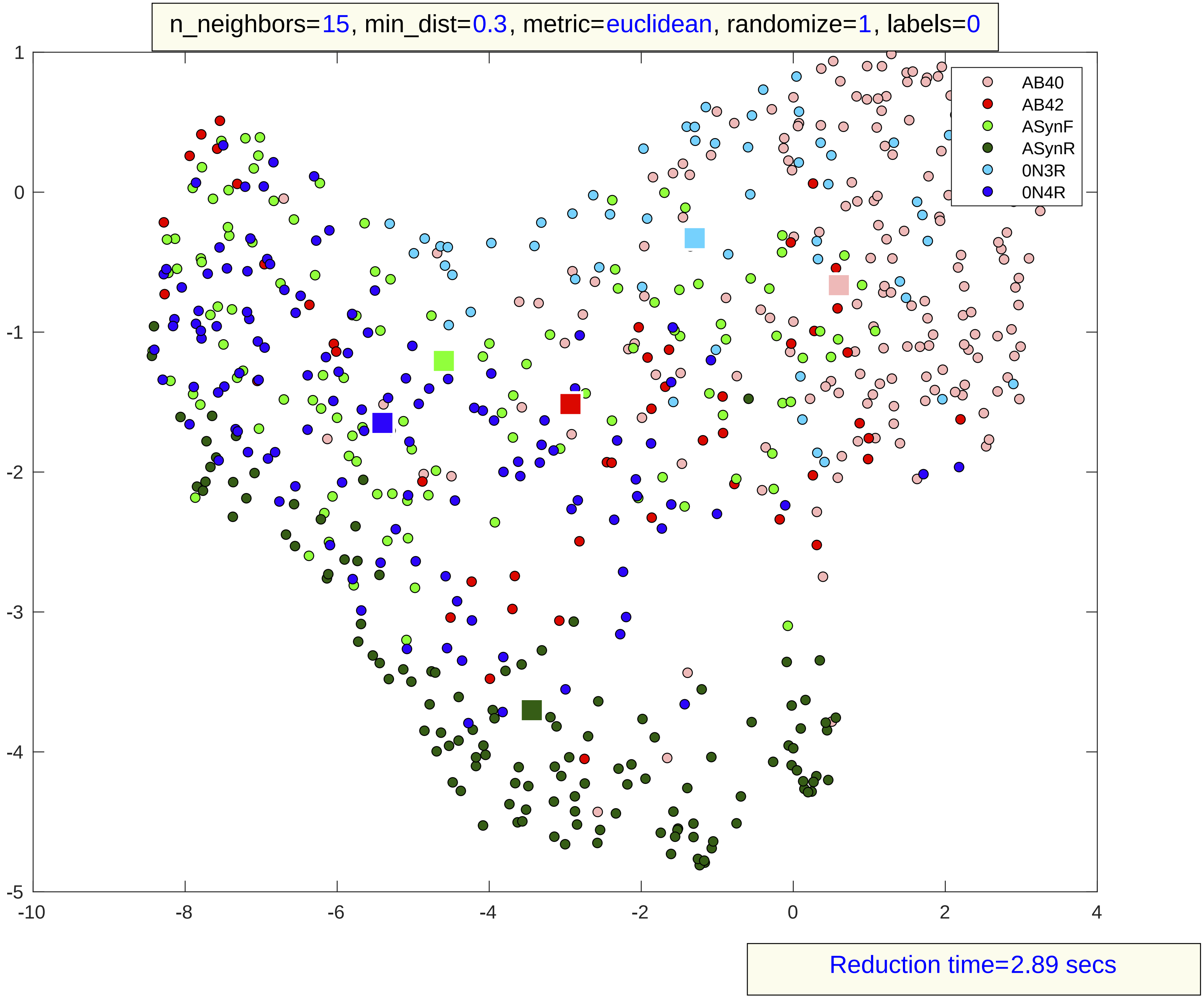

n_neighbors=15, min_dist=0.3, metric=euclidean, randomize=1, labels=0
AB40
AB42
ASynF
ASynR
0N3R
0N4R
1
0
-1
-2
-3
-4
-5
-10
-8
-6
-4
-2
0
2
4
Reduction time=2.89 secs

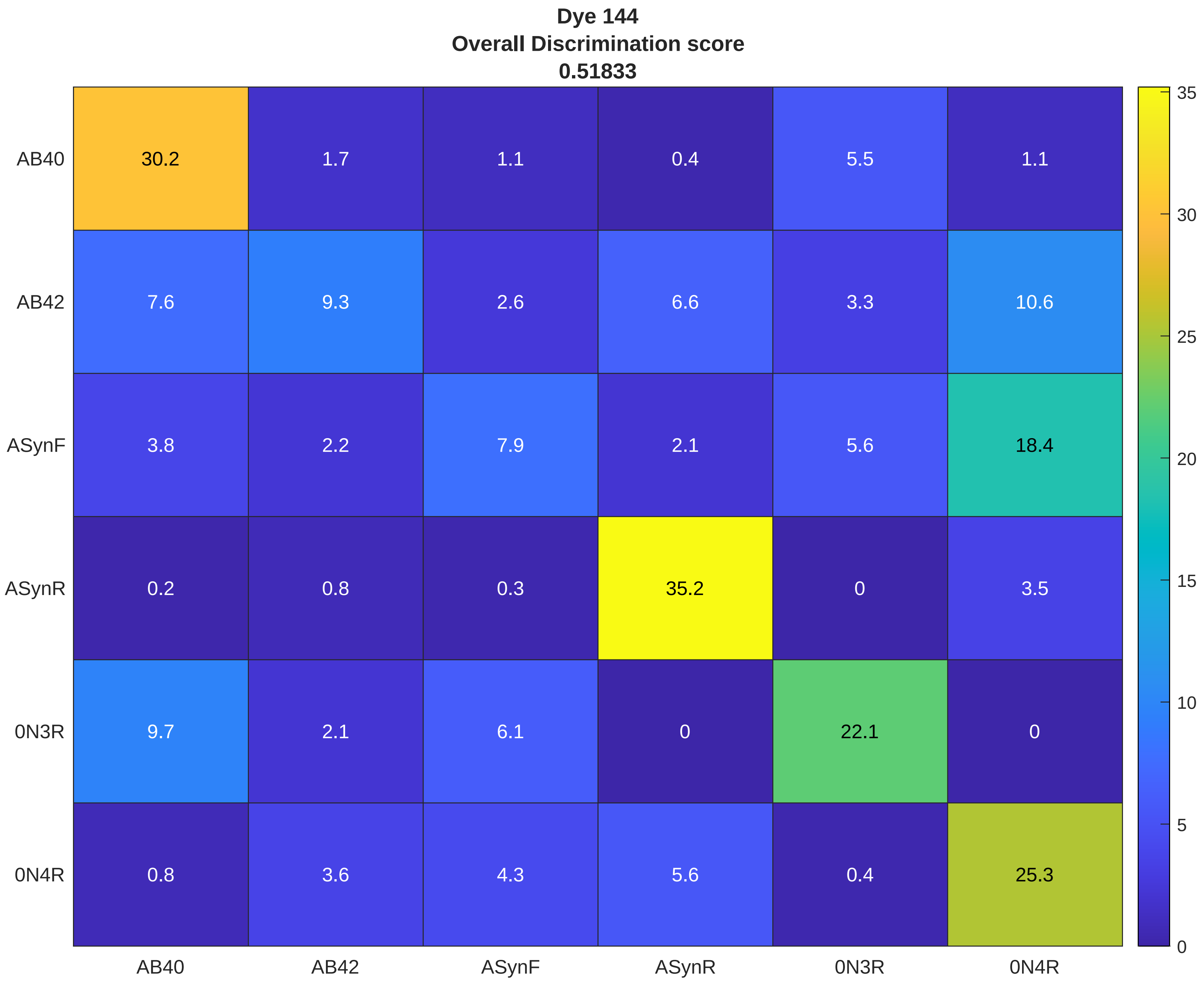
Dye 144
Overall Discrimination score
0.51833
AB40
AB42
ASynF
ASynR
0N3R
0N4R
30.2
1.7
1.1
0.4
5.5
1.1
7.6
9.3
2.6
6.6
3.3
10.6
3.8
2.2
7.9
2.1
5.6
18.4
0.2
0.8
0.3
35.2
0
3.5
9.7
2.1
6.1
0
22.1
0
0.8
3.6
4.3
5.6
0.4
25.3
AB40
AB42
ASynF
ASynR
0N3R
0N4R
35
30
25
20
15
10
5
0

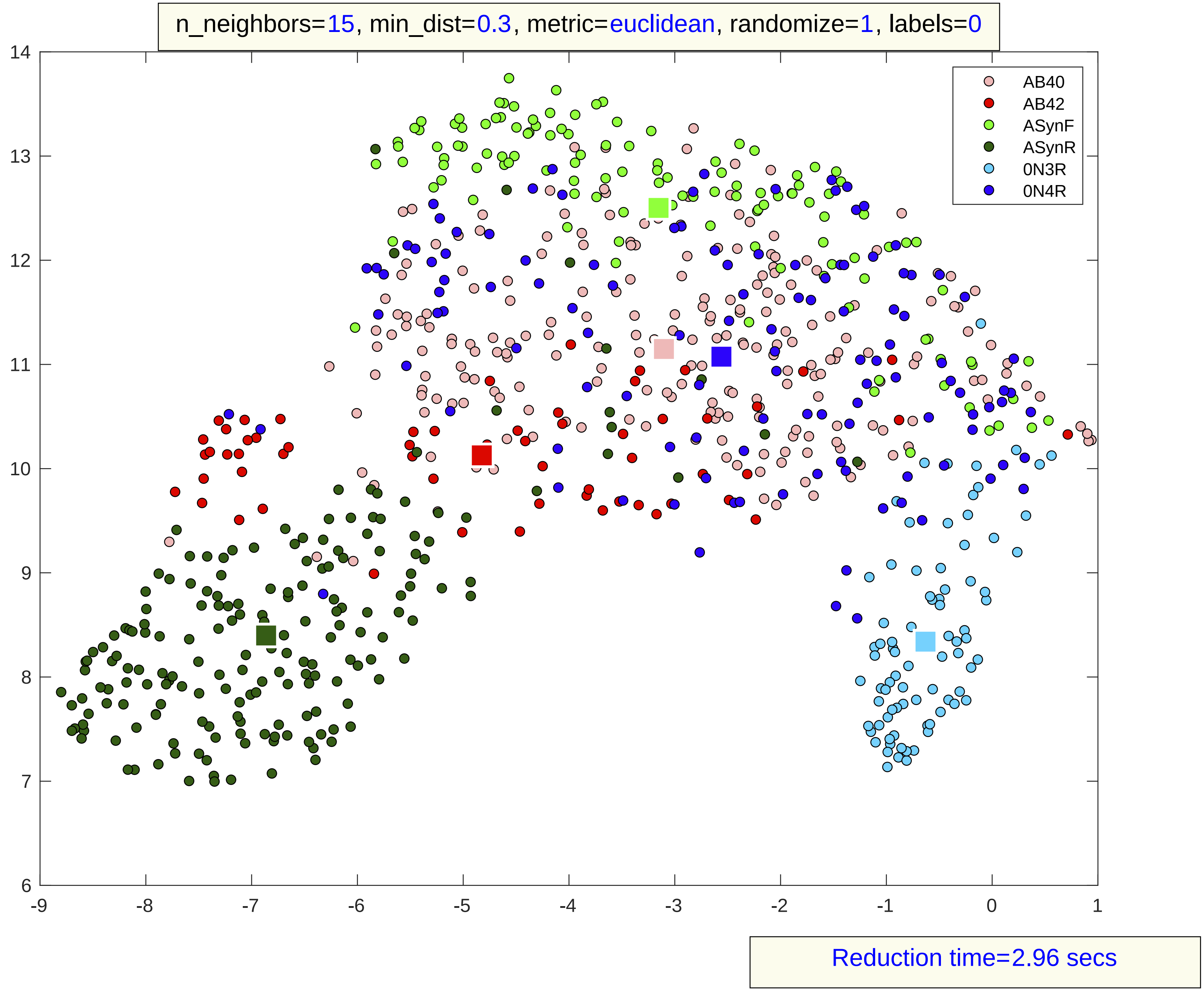

n_neighbors=15, min_dist=0.3, metric=euclidean, randomize=1, labels=0
AB40
AB42
ASynF
ASynR
0N3R
0N4R
14
13
12
11
10
9
8
7
6
-9
-8
-7
-6
-5
-4
-3
-2
-1
0
1
Reduction time=2.96 secs

Dye 145
Overall Discrimination score
0.68583

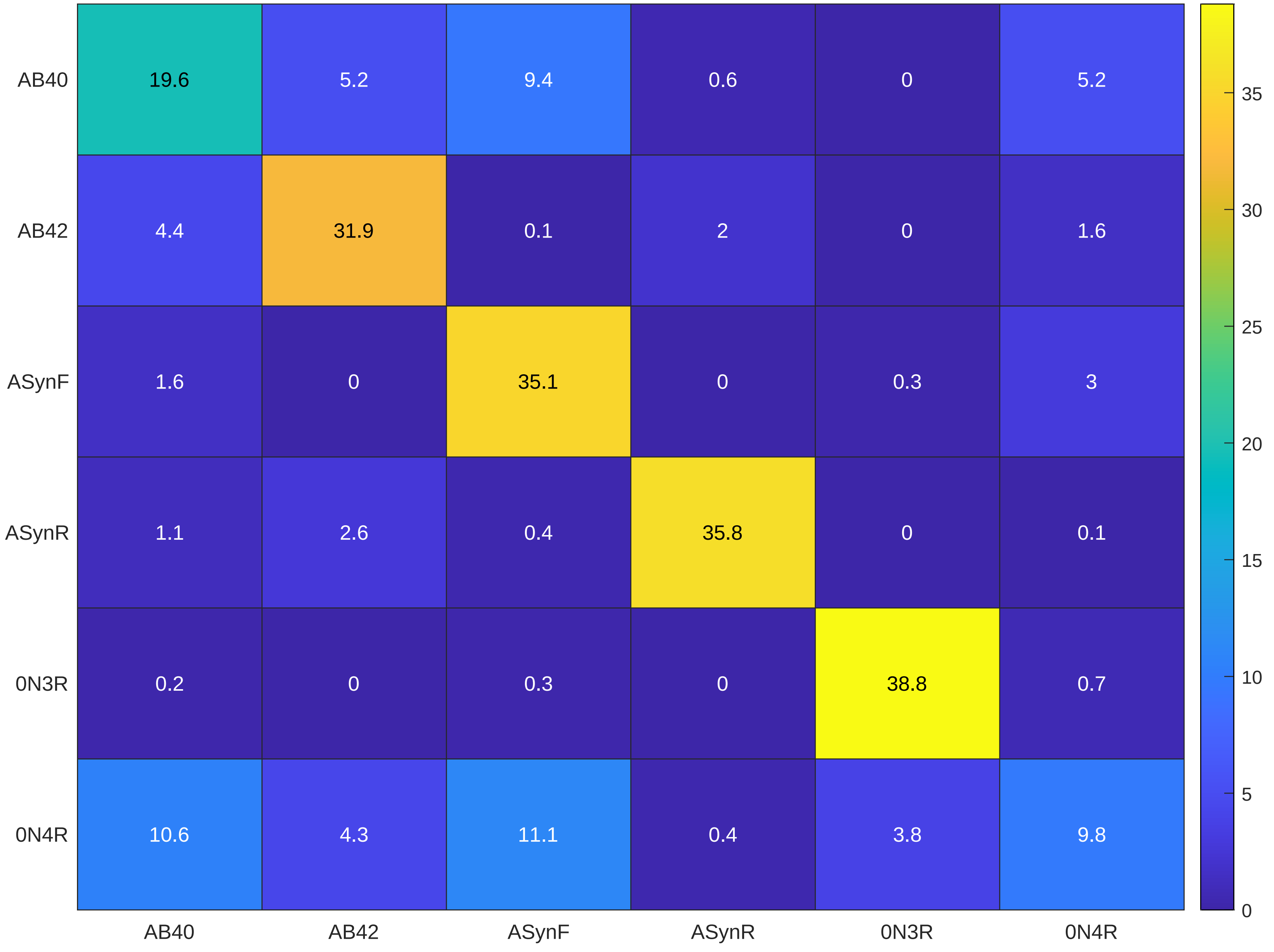

Supplement: Supplementary file 3 — Dataset S02 (PDF) [file pnas.2300769120.sd02.pdf]
